# Supplementary material for: Locations and structures of influenza A virus packaging-associated signals and other functional elements via an in silico pipeline for predicting constrained features in RNA viruses
Source: PLoS Comput Biol. 2024 Apr 22;20(4):e1012009. doi: 10.1371/journal.pcbi.1012009 (PMC11034665; doi:10.1371/journal.pcbi.1012009)
Supplement: S3 Code — The content of the notebook follows the same pattern as that in S1 Code. (ZIP) [file S3_Code.pdf › S3_Code.pdf]

H3N2 human hosts

PB2

Gene length histogram

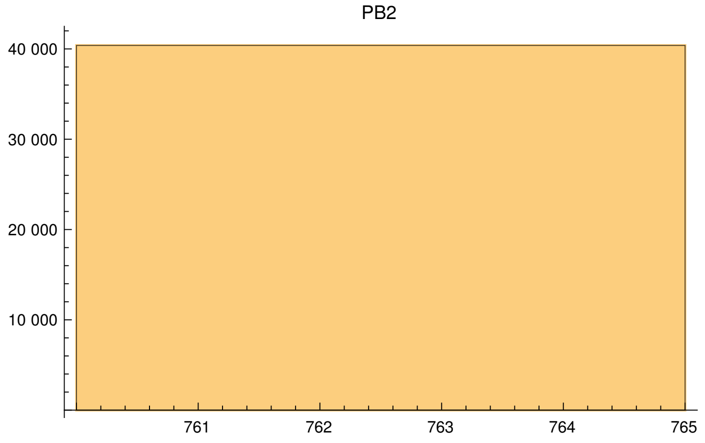

Information vs. nPD

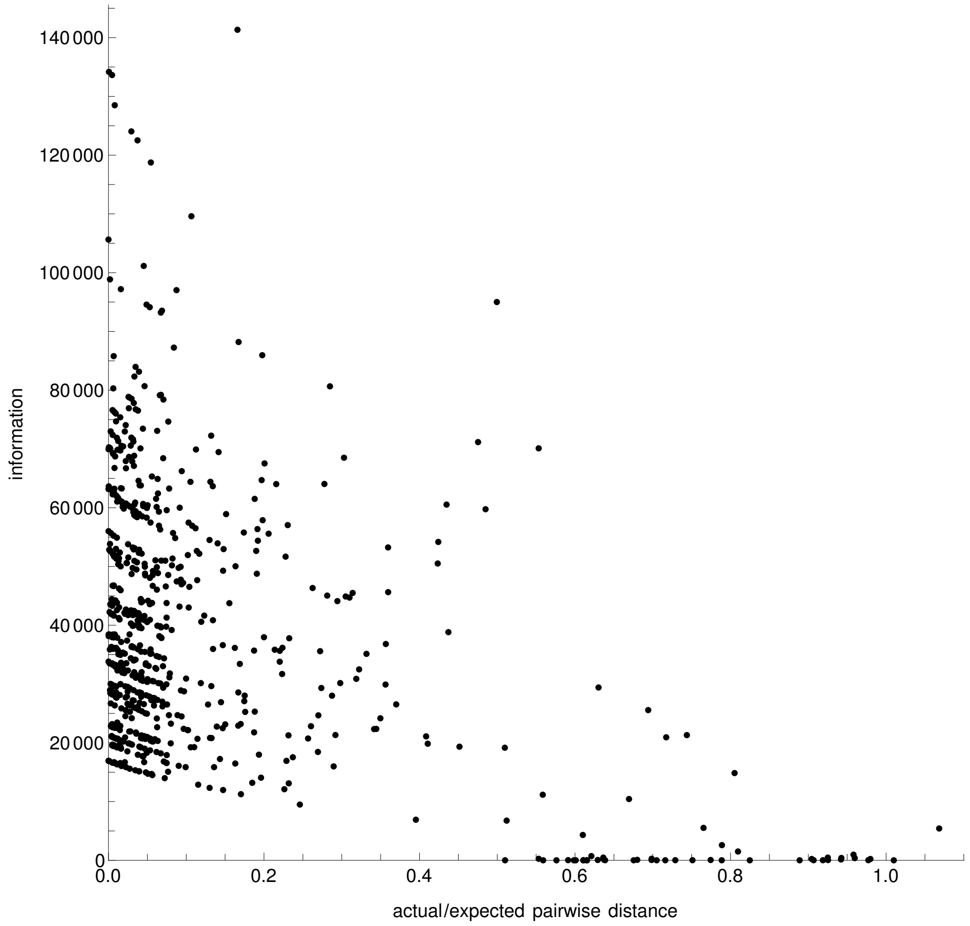

PB2

Example sequences highlighted by regions found in analyses to be conserved

```
Interesting points (by weighted raw PD) highlighted for gene PB2:
ATGGAAAGAATAAAAGAACTACGGAATCTAATGTCGCAGTCTCGCACTCGCGAGATACTGACGAAAACCACAGTGG
    ACCATATGGCCATAATTAAGAAGTACACATCGGGGAGACAGGAAAAGAACCCGTCA  ---CTTAGGATGAAATGGAT
    GATGGCAATGAAATATCCAATTACTGCTGACAAAAGGATAACAGAAATGGTTCCGGAGAGAAATGAACAAGGACA
    AACTCTGTGGAGCAAAATGAGTGATGCTGGATCAGACAGAGTGATGGTATCACCTTTGGCTGTAACATGGTGGAA
    TAGGAATGGACCCGTGACAAATACGGTCCATTACCCAAAAGTGTACAAGACTTATTTCGACAAAGTCGAAAGGTT
    AAAACATGGAACCTTTGGCCCTGTCCATTTTAGAAATCAAGTCAAGATACGAAGAAGAGTAGACATAAACCCTGG
    TCATGCAGACCTCAGTGCCAAAGAGGCACAAGATGTAATTATGGAAGTTGTTTTTCCCAATGAAGTGGGAGCCAG
    AATACTAACATCAGAATCACAGCTAACAATAACTAAAGAGAAAAAAGAAGAACTCCGAGATTGCAAAATTTCTCC
    CTTGATGGTCGCATACATGCTAGAGAGAGAACTTGTGCGGAAAACAAGATTTCTCCCAGTTGCTGGCGGAACAAG
    CAGTATATACATTGAAGTTTTACATTTGACTCAAGGAACGTGTTGGGAACAAATGTACACTCCAGGTGGAGGAGT
    GAGGAATGACGATGTTGACCAAAGCCTAATTATTGCGGCCAGGAACATAGTAAGAAGAGCCGCAGTGTCAGCAGA
    TCCACTCGCATCTTTATTGGAGATGTGTCACAGCACGCAAATTGGCGGAACAAGGATGGTGGACATTCTTAGACA
    GAACCCGACTGAAGAACAAGCTGTTGATATATGCAAAGCTGCAATGGGATTGAGAATCAGCTCATCTTTCAGCTT
    TGGTGGCTTTACATTTAAAAGAACGAGCGGGTCGTCAGTC  ---AAAAGAGAGGAAGAG ---GTTCTTACAGGCAAT
    CTCCAGACATTGAGAATAAGAGTACATGAGGGATATGAGGAGTTCACAATGGTGGGGAAAAGAGCAACAGCTATA
    CTAAGAAAAGCAACCAGAAGACTGGTTCAACTCATAGTGAGTGGGAGAGACGAACAGTCAATAGCCGAAGCAATA
    ATCGTGGCCATGGTGTTTTCACAAGAAGATTGCGTGATAAAAGCAGTTAGAGGTGACCTGAATTTTGTCAACAGA
    GCAAATCAGCGGTTGAACCCCATGCATCAGCTTTTAAGGCATTTTCAGAAAGATGCGAAAGTGCTCTTTCAAAAT
    TGGGGAGTTGAACACATCGACAGTGTGATGGGAATGGTTGGAGTATTACCAGATATGACTCCAAGCACAGAGATG
    TCAATGAGAGGAATAAGAGTCAGCAAAATGGGTGTAGATGAATACTCCAGTACAGAGAGGGTGGTGGTTAGCATT
    GATCGGTTTTTGAGAGTTCGAGATCAACGTGGGAATGTATTATTATCTCCTGAGGAGGTCAGTGAAACACAGGGA
    ACTGAAAGACTGACCATAACTTACTCATCATCGATGATGTGGGAGATTAACGGTCCTGAGTCGGTTTTGGTCAAT
    ACCTATCAATGGATCATCAGGAATTGGGAAGCTGTCAAAATTCAATGGTCTCAGAACCCTGCAATGTTGTACAAC
    AAAATGGAATTTGAACCATTTCAATCTTTAGTCCCCAAGGCCACTAGAAGCCAATACAGTGGGTTTGTCAGAACT
    CTATTCCAACAAATGAGAGACGTACTTGGGACATTTGACACTGCCCAGATAATAAAGCTTCTCCCTTTTGCAGCT
    GCTCCACCGAAGCAAAGCAGAATGCAGTTCTCTTCACTGACTGTGAATGTGAGGGGATCAGGGATGAGAATACTT
    GTAAGGGGCAATTCTCCTGTATTCAACTACAACAAGACCACTAAAAGGCTAACAATTCTTGGAAAAGATGCCGGC
    ACTTTAATTGAAGACCCAGATGAAAGCACATCCGGAGTGGAGTCCGCCGTCTTG  ---AGAGGGTTCCTCATTATAA
    GTAAAGAAGACAGAAGATACGGACCAGCATTAAGCATCAATGAACTGAGTAACCTTGCAAAAGGGGAAAAGGCTA
    ATGTGCTAATTGGGCAAGGAGACGTGGTGTTGGTAATGAAACGGAAACGGGACTCTAGTATACTTACTGACAGCC
    AGACAGCGACCAAAAGAATTCGGATGGCCATCAATTAA

Interesting points (by weighted ranked PD) highlighted for gene PB2:
ATGGAAAGAATAAAAGAACTACGGAATCTAATGTCGCAGTCTCGCACTCGCGAGATACTGACGAAAACCACAGTGGAC
    CATATGGCCATAATTAAGAAGTACACATCGGGGAGACAGGAAAAGAACCCGTCA  ---CTTAGGATGAAATGGATGATG
    GCAATGAAATATCCAATTACTGCTGACAAAAGGATAACAGAAATGGTTCCGGAGAGAAATGAACAAGGACAAACT
    CTGTGGAGCAAAATGAGTGATGCTGGATCAGACAGAGTGATGGTATCACCTTTGGCTGTAACATGGTGGAATAGG
    AATGGACCCGTGACAAATACGGTCCATTACCCAAAAGTGTACAAGACTTATTTCGACAAAGTCGAAAGGTTAAAA
    CATGGAACCTTTGGCCCTGTCCATTTTAGAAATCAAGTCAAGATACGAAGAAGAGTAGACATAAACCCTGGTCAT
    GCAGACCTCAGTGCCAAAGAGGCACAAGATGTAATTATGGAAGTTGTTTTTCCCAATGAAGTGGGAGCCAGAATA
    CTAACATCAGAATCACAGCTAACAATAACTAAAGAGAAAAAAGAAGAACTCCGAGATTGCAAAATTTCTCCCTTG
    ATGGTCGCATACATGCTAGAGAGAGAACTTGTGCGGAAAACAAGATTTCTCCCAGTTGCTGGCGGAACAAGCAGT
    ATATACATTGAAGTTTTACATTTGACTCAAGGAACGTGTTGGGAACAAATGTACACTCCAGGTGGAGGAGTGAGG
```

AATGACGATGTTGACCAAAGCCTAATTATTGCGGCCAGGAACATAGTAAGAAGAGCCGCAGTGTCAGCAGATCCA∵.
CTCGCATCTTTATTGGAGATGTGTCACAGCACGCAAATTGGCGGAACAAGGATGGTGGACATTCTTAGACAGAAC∵.
CCGACTGAAGAACAAGCTGTTGATATATGCAAAGCTGCAATGGGATTGAGAATCAGCTCATCTTTCAGCTTTGGT∵.
GGCTTTACATTTAAAAGAACGAGCGGGTCGTCAGTC ---AAAAGAGAGGAAGAG ---GTTCTTACAGGCAATCTCC∵.
AGACATTGAGAATAAGAGTACATGAGGGATATGAGGAGTTCACAATGGTGGGGAAAAGAGCAACAGCTATACTAA∵.
GAAAAGCAACCAGAAGACTGGTTCAACTCATAGTGAGTGGGAGAGACGAACAGTCAATAGCCGAAGCAATAATCG∵.
TGGCCATGGTGTTTTCACAAGAAGATTGCGTGATAAAAGCAGTTAGAGGTGACCTGAATTTTGTCAACAGAGCAA∵.
ATCAGCGGTTGAACCCCATGCATCAGCTTTTAAGGCATTTTCAGAAAGATGCGAAAGTGCTCTTTCAAAATTGGG∵.
GAGTTGAACACATCGACAGTGTGATGGGAATGGTTGGAGTATTACCAGATATGACTCCAAGCACAGAGATGTCAA∵.
TGAGAGGAATAAGAGTCAGCAAAATGGGTGTAGATGAATACTCCAGTACAGAGAGGGTGGTGGTTAGCATTGATC∵.
GGTTTTTGAGAGTTCGAGATCAACGTGGGAATGTATTATTATCTCCTGAGGAGGTCAGTGAAACACAGGGAACTG∵.
AAAGACTGACCATAACTTACTCATCATCGATGATGTGGGAGATTAACGGTCCTGAGTCGGTTTTGGTCAATACCT∵.
ATCAATGGATCATCAGGAATTGGGAAGCTGTCAAAATTCAATGGTCTCAGAACCCTGCAATGTTGTACAACAAAA∵.
TGGAATTTGAACCATTTCAATCTTTAGTCCCCAAGGCCACTAGAAGCCAATACAGTGGGTTTGTCAGAACTCTAT∵.
TCCAACAAATGAGAGACGTACTTGGGACATTTGACACTGCCCAGATAATAAAGCTTCTCCCTTTTGCAGCTGCTC∵.
CACCGAAGCAAAGCAGAATGCAGTTCTCTTCACTGACTGTGAATGTGAGGGGATCAGGGATGAGAATACTTGTAA∵.
GGGGCAATTCTCCTGTATTCAACTACAACAAGACCACTAAAAGGCTAACAATTCTTGGAAAAGATGCCGGCACTT∵.
TAATTGAAGACCCAGATGAAAGCACATCCGGAGTGGAGTCCGCCGTCTTG ---AGAGGGTTCCTCATTATAAGTAA∵.
AGAAGACAGAAGATACGGACCAGCATTAAGCATCAATGAACTGAGTAACCTTGCAAAAGGGGAAAAGGCTAATGT∵.
GCTAATTGGGCAAGGAGACGTGGTGTTGGTAATGAAACGGAAACGGGACTCTAGT
ATACTTACTGACAGCCAGACAGCGACCAAAAGAATTCGGATGGCCATCAATTAA

Per codon conservation report

PB2

Pos.	1	obs:	exp:
atg	M	40384	40384.00
---	--	------	------
mPD		0	0
		nPD:	1.
		N. weight:	0.
		Sc. PD:	0
		Sc. rank:	0

PB2

Pos.	2	obs:	exp:
gat	D	1	1.70
gac	D	2	1.30
gaa	E	39716	23806.00
gag	E	663	16573.00
ggt	G	0	0.27
ggc	G	0	0.28
gga	G	2	0.92
ggg	G	0	0.53
---	--	------	------
mPD		0.033	0.48
		nPD:	0.07
		N. weight:	0.48
		Sc. PD:	-0.0013
		Sc. rank:	6978.5

PB2

Pos.	3	obs:	exp:
cgt	R	0	800.70
cgc	R	0	1453.00
cga	R	4	3684.00
cgg	R	0	3468.00
aaa	K	2	1.31
aag	K	0	0.69
aga	R	40215	19807.00
agg	R	163	11170.00
---	--	------	------
mPD		0.0083	0.89
		nPD:	0.01
		N. weight:	0.72
		Sc. PD:	-0.043
		Sc. rank:	-13714.0

PB2

Pos.	4	obs:	exp:
tta	L	0	0.12
ttg	L	0	0.21
ctt	L	0	0.21
ctc	L	0	0.15
cta	L	1	0.15
ctg	L	0	0.17
att	I	2	14168.00
atc	I	0	10605.00
ata	I	40358	15587.00
atg	M	7	7.00
act	T	0	0.31
acc	T	0	0.18
aca	T	1	0.43
acg	T	0	0.08
gtt	V	0	3.26
gtc	V	0	2.65
gta	V	13	2.82
gtg	V	0	4.28
gat	D	2	1.13
gac	D	0	0.87
---	--	------	------
mPD		0.0015	0.66
		nPD:	0.
		N. weight:	1.
		Sc. PD:	-0.067
		Sc. rank:	-29611.0

PB2

Pos.	5	obs:	exp:
cgt	R	0	0.16
cgc	R	0	0.29
cga	R	0	0.73
cgg	R	0	0.69
act	T	0	0.62
acc	T	0	0.35
aca	T	2	0.87
acg	T	0	0.16
aat	N	1	0.56
aac	N	0	0.44
aaa	K	40317	26542.00
aag	K	55	13830.00
aga	R	8	3.92
agg	R	0	2.21
gaa	E	1	0.59
gag	E	0	0.41
---	--	------	------
mPD		0.0033	0.45
		nPD:	0.01
		N. weight:	0.43
		Sc. PD:	-0.027
		Sc. rank:	-9275.5

PB2

Pos.	6	obs:	exp:
aaa	K	1	0.66
aag	K	0	0.34
gtt	V	0	0.25
gtc	V	0	0.20
gta	V	1	0.22
gtg	V	0	0.33
gat	D	0	1.70
gac	D	3	1.30
gaa	E	40122	23804.00
gag	E	253	16571.00
ggt	G	0	0.54
ggc	G	0	0.56
gga	G	4	1.85
ggg	G	0	1.06
---	--	------	------
mPD		0.013	0.48
		nPD:	0.03
		N. weight:	0.52
		Sc. PD:	-0.022
		Sc. rank:	-3471.4

PB2

Pos.	7	obs:	exp:
ttt	F	2	0.97
ttc	F	0	1.03
tta	L	758	4686.00
ttg	L	2	8348.00
ctt	L	4290	8447.00
ctc	L	11	5921.00
cta	L	35069	6201.00
ctg	L	250	6777.00
cct	P	0	0.33
ccc	P	0	0.19
cca	P	1	0.34
ccg	P	0	0.14
att	I	1	0.35
atc	I	0	0.26
ata	I	0	0.39
---	--	------	------
mPD		0.24	1.2
		nPD:	0.21
		N. weight:	1.5
		Sc. PD:	0.19
		Sc. rank:	40427.0

PB2

Pos.	8	obs:	exp:
tta	L	0	0.93
ttg	L	0	1.65
tgg	W	2	2.00
ctt	L	0	1.67
ctc	L	0	1.17
cta	L	0	1.23
ctg	L	8	1.34
caa	Q	0	18.95
cag	Q	31	12.05
cgt	R	47	799.90
cgc	R	0	1452.00
cga	R	349	3681.00
cgg	R	39517	3464.00
aga	R	387	19788.00
agg	R	43	11159.00
---	--	------	------
mPD		0.061	0.89
		nPD:	0.07
		N. weight:	2.4
		Sc. PD:	-0.0029
		Sc. rank:	38257.0

PB2

Pos.	9	obs:	exp:
tct	S	0	1.61
tcc	S	0	1.49
tca	S	0	2.69
tcg	S	0	0.39
tat	Y	1	0.53
tac	Y	0	0.47
cat	H	2	1.69
cac	H	1	1.31
att	I	4	1.40
atc	I	0	1.05
ata	I	0	1.54
act	T	3	27.33
acc	T	85	15.56
aca	T	0	38.08
acg	T	0	7.03
aat	N	39264	22284.00
aac	N	567	17547.00
aaa	K	1	0.66
aag	K	0	0.34
agt	S	10	1.75
agc	S	0	2.06
gat	D	430	249.30
gac	D	11	191.70
gaa	E	0	0.59
gag	E	1	0.41
ggt	G	4	0.54
ggc	G	0	0.56
gga	G	0	1.85
ggg	G	0	1.06
---	--	------	------
mPD		0.060	0.52
		nPD:	0.11
		N. weight:	0.54
		Sc. PD:	0.024
		Sc. rank:	11999.0

PB2

Pos.	10	obs:	exp:
tta	L	45	4686.00
ttg	L	20	8349.00
ctt	L	9	8447.00
ctc	L	3	5922.00
cta	L	34023	6201.00
ctg	L	6281	6777.00
att	I	0	1.05
atc	I	0	0.79
ata	I	3	1.16
---	--	------	------
mPD		0.27	1.2
		nPD:	0.23
		N. weight:	1.5
		Sc. PD:	0.24
		Sc. rank:	42754.0

PB2			
Pos.	11	obs:	exp:
tta	L	0	0.12
ttg	L	1	0.21
ctt	L	0	0.21
ctc	L	0	0.15
cta	L	0	0.15
ctg	L	0	0.17
att	I	1	1.40
atc	I	0	1.05
ata	I	3	1.54
atg	M	40379	40379.00
---	--	------	------
mPD		0.00025	0.00028
		nPD:	0.89
		N. weight:	0.00011
		Sc. PD:	0.000086
		Sc. rank:	3.5

,

PB2			
Pos.	12	obs:	exp:
tta	L	0	1.74
ttg	L	15	3.10
tct	S	67	6514.00
tcc	S	0	6033.00
tca	S	185	10850.00
tcg	S	40117	1589.00
ctt	L	0	3.14
ctc	L	0	2.20
cta	L	0	2.30
ctg	L	0	2.52
agt	S	0	7055.00
agc	S	0	8328.00
---	--	------	------
mPD		0.013	1.6
		nPD:	0.01
		N. weight:	3.4
		Sc. PD:	-0.2
		Sc. rank:	-67925.0

,

PB2			
Pos.	13	obs:	exp:
tta	L	0	1.86
ttg	L	0	3.31
ctt	L	0	3.35
ctc	L	0	2.35
cta	L	0	2.46
ctg	L	16	2.69
cct	P	0	0.33
ccc	P	0	0.19
cca	P	0	0.34
ccg	P	1	0.14
cat	H	10	5.64
cac	H	0	4.36
caa	Q	511	24669.00
cag	Q	39846	15688.00
---	--	------	------
mPD		0.026	0.48
		nPD:	0.06
		N. weight:	0.92
		Sc. PD:	-0.013
		Sc. rank:	9760.2

,

PB2			
Pos.	14	obs:	exp:
tct	S	32536	6516.00
tcc	S	7842	6034.00
tca	S	0	10853.00
tcg	S	0	1589.00
tat	Y	1	0.53
tac	Y	0	0.47
cct	P	3	1.00
ccc	P	0	0.56
cca	P	0	1.01
ccg	P	0	0.42
act	T	1	0.31
acc	T	0	0.18
aca	T	0	0.43
acg	T	0	0.08
agt	S	0	7056.00
agc	S	0	8330.00
gct	A	1	0.27
gcc	A	0	0.25
gca	A	0	0.40
gcg	A	0	0.07
---	--	------	------
mPD		0.31	1.6
		nPD:	0.19
		N. weight:	1.4
		Sc. PD:	0.17
		Sc. rank:	38264.0

,

PB2			
Pos.	15	obs:	exp:
cat	H	0	1.13
cac	H	2	0.87
cgt	R	2	800.70
cgc	R	40373	1453.00
cga	R	7	3684.00
cgg	R	0	3468.00
aga	R	0	19807.00
agg	R	0	11170.00
---	--	------	------
mPD		0.00054	0.89
		nPD:	0.
		N. weight:	3.5
		Sc. PD:	-0.24
		Sc. rank:	-110460.0

,

PB2			
Pos.	16	obs:	exp:
att	I	4	1.40
atc	I	0	1.05
ata	I	0	1.54
act	T	39601	12540.00
acc	T	773	7139.00
aca	T	4	17475.00
acg	T	1	3225.00
aat	N	1	0.56
aac	N	0	0.44
---	--	------	------
mPD		0.038	0.68
		nPD:	0.06
		N. weight:	1.1
		Sc. PD:	-0.016
		Sc. rank:	12533.0

,

PB2			
Pos.	17	obs:	exp:
tct	S	0	0.32
tcc	S	0	0.30
tca	S	0	0.54
tcg	S	0	0.08
tgt	C	0	2.74
tgc	C	7	4.26
cat	H	0	0.56
cac	H	1	0.44
cgt	R	1	800.50
cgc	R	40301	1453.00
cga	R	71	3684.00
cgg	R	1	3467.00
agt	S	0	0.35
agc	S	2	0.41
aga	R	0	19803.00
agg	R	0	11167.00
---	--	------	------
mPD		0.0041	0.89
		nPD:	0.
		N. weight:	3.5
		Sc. PD:	-0.22
		Sc. rank:	-93095.0

,

PB2			
Pos.	18	obs:	exp:
caa	Q	0	0.61
cag	Q	1	0.39
gat	D	4	2.26
gac	D	0	1.74
gaa	E	8	23804.00
gag	E	40368	16572.00
ggt	G	0	0.40
ggc	G	0	0.42
gga	G	1	1.38
ggg	G	2	0.79
---	--	------	------
mPD		0.00084	0.48
		nPD:	0.
		N. weight:	0.94
		Sc. PD:	-0.063
		Sc. rank:	-28801.0

,

PB2			
Pos.	19	obs:	exp:
att	I	0	14174.00
atc	I	0	10610.00
ata	I	40378	15594.00
act	T	0	0.62
acc	T	0	0.35
aca	T	2	0.87
acg	T	0	0.16
gtt	V	0	0.75
gtc	V	0	0.61
gta	V	3	0.65
gtg	V	0	0.99
---	-	1	1.00
---	--	------	------
mPD		0.00025	0.66
		nPD:	0.
		N. weight:	1.
		Sc. PD:	-0.069
		Sc. rank:	-32096.0

,

PB2			
Pos.	20	obs:	exp:
tta	L	2	4686.00
ttg	L	23	8348.00
ctt	L	24	8447.00
ctc	L	376	5921.00
cta	L	908	6200.00
ctg	L	39046	6777.00
caa	Q	0	1.22
cag	Q	2	0.78
atg	M	3	3.00
---	--	------	------
mPD		0.065	1.2
		nPD:	0.06
		N. weight:	1.7
		Sc. PD:	-0.024
		Sc. rank:	18527.0

,

PB2			
Pos.	21	obs:	exp:
tct	S	0	0.32
tcc	S	0	0.30
tca	S	2	0.54
tcg	S	0	0.08
cgt	R	0	0.02
cgc	R	0	0.04
cga	R	0	0.09
cgg	R	0	0.09
att	I	0	8.42
atc	I	0	6.31
ata	I	24	9.27
act	T	377	12527.00
acc	T	6	7132.00
aca	T	37851	17458.00
acg	T	2105	3222.00
aaa	K	1	0.66
aag	K	0	0.34
agt	S	0	0.35
agc	S	0	0.41
aga	R	1	0.49
agg	R	0	0.28
gct	A	1	4.63
gcc	A	0	4.25
gca	A	16	6.88
gcg	A	0	1.24
---	--	------	------
mPD		0.12	0.68
		nPD:	0.17
		N. weight:	0.71
		Sc. PD:	0.073
		Sc. rank:	18376.0

,

PB2			
Pos.	22	obs:	exp:
cgt	R	0	0.54
cgc	R	0	0.97
cga	R	0	2.46
cgg	R	0	2.32
act	T	0	0.31
acc	T	0	0.18
aca	T	1	0.43
acg	T	0	0.08
aat	N	0	7.83
aac	N	14	6.17
aaa	K	39940	26522.00
aag	K	402	13820.00
aga	R	27	13.24
agg	R	0	7.47
---	--	------	------
mPD		0.022	0.45
		nPD:	0.05
		N. weight:	0.39
		Sc. PD:	-0.0084
		Sc. rank:	3082.9

,

PB2			
Pos.	23	obs:	exp:
att	I	0	0.35
atc	I	1	0.26
ata	I	0	0.39
act	T	291	12541.00
acc	T	40077	7140.00
aca	T	15	17477.00
acg	T	0	3225.00
---	--	------	------
mPD		0.015	0.68
		nPD:	0.02
		N. weight:	1.8
		Sc. PD:	-0.083
		Sc. rank:	-16301.0

,

PB2			
Pos.	24	obs:	exp:
act	T	375	12541.00
acc	T	25	7140.00
aca	T	39956	17477.00
acg	T	27	3225.00
gct	A	0	0.27
gcc	A	0	0.25
gca	A	1	0.40
gcg	A	0	0.07
---	--	------	------
mPD		0.021	0.68
		nPD:	0.03
		N. weight:	0.82
		Sc. PD:	-0.032
		Sc. rank:	-3325.0

,

PB2			
Pos.	25	obs:	exp:
gtt	V	55	10115.00
gtc	V	0	8229.00
gta	V	35	8753.00
gtg	V	40294	13287.00
---	--	------	------
mPD		0.0044	0.74
		nPD:	0.01
		N. weight:	1.2
		Sc. PD:	-0.073
		Sc. rank:	-27928.0

,

PB2			
Pos.	26	obs:	exp:
aat	N	0	0.56
aac	N	1	0.44
gat	D	13	22827.00
gac	D	40370	17556.00
---	--	------	------
mPD		0.00069	0.49
		nPD:	0.
		N. weight:	0.88
		Sc. PD:	-0.059
		Sc. rank:	-27164.0

,

PB2			
Pos.	27	obs:	exp:
cat	H	40329	22778.00
cac	H	55	17606.00
---	--	------	------
mPD		0.0027	0.49
		nPD:	0.01
		N. weight:	0.59
		Sc. PD:	-0.038
		Sc. rank:	-14813.0

,

PB2			
Pos.	28	obs:	exp:
att	I	4	7.72
atc	I	0	5.78
ata	I	18	8.50
atg	M	40358	40358.00
aaa	K	0	0.66
aag	K	1	0.34
gtt	V	0	0.75
gtc	V	0	0.61
gta	V	0	0.65
gtg	V	3	0.99
---	--	------	------
mPD		0.0013	0.0014
		nPD:	0.91
		N. weight:	0.00044
		Sc. PD:	0.00036
		Sc. rank:	14.5

,

PB2			
Pos.	29	obs:	exp:
tct	S	0	1.13
tcc	S	7	1.05
tca	S	0	1.88
tcg	S	0	0.28
act	T	0	0.62
acc	T	2	0.35
aca	T	0	0.87
acg	T	0	0.16
agt	S	0	1.22
agc	S	0	1.44
gtt	V	0	1.00
gtc	V	4	0.82
gta	V	0	0.87
gtg	V	0	1.32
gct	A	1018	11003.00
gcc	A	39332	10086.00
gca	A	21	16330.00
gcg	A	0	2951.00
---	--	------	------
mPD		0.051	0.70
		nPD:	0.07
		N. weight:	1.3
		Sc. PD:	0.004
		Sc. rank:	21999.0

,

PB2			
Pos.	30	obs:	exp:
att	I	8	14174.00
atc	I	203	10610.00
ata	I	40166	15593.00
gtt	V	0	1.75
gtc	V	0	1.43
gta	V	7	1.52
gtg	V	0	2.30
---	--	------	------
mPD		0.011	0.66
		nPD:	0.02
		N. weight:	0.97
		Sc. PD:	-0.051
		Sc. rank:	-11921.0

,

PB2			
Pos.	31	obs:	exp:
tta	L	0	0.58
ttg	L	0	1.03
ctt	L	5	1.05
ctc	L	0	0.73
cta	L	0	0.77
ctg	L	0	0.84
att	I	39948	14174.00
atc	I	409	10609.00
ata	I	19	15593.00
gtt	V	3	0.75
gtc	V	0	0.61
gta	V	0	0.65
gtg	V	0	0.99
---	--	------	------
mPD		0.021	0.66
		nPD:	0.03
		N. weight:	1.
		Sc. PD:	-0.039
		Sc. rank:	-2773.2

,

PB2			
Pos.	32	obs:	exp:
cgt	R	0	0.28
cgc	R	0	0.50
cga	R	0	1.28
cgg	R	0	1.20
aaa	K	570	26541.00
aag	K	39800	13829.00
aga	R	0	6.87
agg	R	14	3.87
---	--	------	------
mPD		0.029	0.45
		nPD:	0.06
		N. weight:	1.
		Sc. PD:	-0.007
		Sc. rank:	13944.0

,

PB2			
Pos.	33	obs:	exp:
cgt	R	0	0.12
cgc	R	0	0.22
cga	R	0	0.55
cgg	R	0	0.52
aat	N	0	0.56
aac	N	1	0.44
aaa	K	224	26545.00
aag	K	40153	13832.00
aga	R	0	2.94
agg	R	6	1.66
---	--	------	------
mPD		0.011	0.45
		nPD:	0.03
		N. weight:	1.1
		Sc. PD:	-0.048
		Sc. rank:	-8190.6

,

PB2			
Pos.	34	obs:	exp:
ttt	F	0	0.49
ttc	F	1	0.51
tat	Y	73	21286.00
tac	Y	40310	19097.00
---	--	------	------
mPD		0.0037	0.50
		nPD:	0.01
		N. weight:	0.78
		Sc. PD:	-0.048
		Sc. rank:	-16665.0

,

PB2			
Pos.	35	obs:	exp:
tct	S	0	0.48
tcc	S	0	0.45
tca	S	3	0.81
tcg	S	0	0.12
act	T	2	12540.00
acc	T	11	7139.00
aca	T	40206	17476.00
acg	T	161	3225.00
agt	S	0	0.52
agc	S	0	0.62
gct	A	0	0.27
gcc	A	0	0.25
gca	A	1	0.40
gcg	A	0	0.07
---	--	------	------
mPD		0.0088	0.68
		nPD:	0.01
		N. weight:	0.86
		Sc. PD:	-0.048
		Sc. rank:	-12949.0

,

PB2			
Pos.	36	obs:	exp:
tta	L	0	0.12
ttg	L	1	0.21
tct	S	421	6516.00
tcc	S	0	6034.00
tca	S	1438	10853.00
tcg	S	38521	1590.00
ctt	L	0	0.21
ctc	L	0	0.15
cta	L	0	0.15
ctg	L	0	0.17
act	T	0	0.93
acc	T	0	0.53
aca	T	2	1.30
acg	T	1	0.24
agt	S	0	7057.00
agc	S	0	8330.00
---	--	------	------
mPD		0.089	1.6
		nPD:	0.05
		N. weight:	3.1
		Sc. PD:	-0.047
		Sc. rank:	32487.0

,

PB2			
Pos.	37	obs:	exp:
gtt	V	0	0.25
gtc	V	0	0.20
gta	V	0	0.22
gtg	V	1	0.33
gaa	E	0	2.36
gag	E	4	1.64
ggt	G	57	5436.00
ggc	G	0	5636.00
gga	G	590	18640.00
ggg	G	39732	10668.00
---	--	------	------
mPD		0.032	0.68
		nPD:	0.05
		N. weight:	1.3
		Sc. PD:	-0.03
		Sc. rank:	9523.0

,

PB2			
Pos.	38	obs:	exp:
cgt	R	0	800.60
cgc	R	0	1453.00
cga	R	4	3684.00
cgg	R	0	3467.00
att	I	0	0.35
atc	I	0	0.26
ata	I	1	0.39
act	T	0	0.31
acc	T	0	0.18
aca	T	1	0.43
acg	T	0	0.08
aaa	K	3	1.97
aag	K	0	1.03
aga	R	39887	19805.00
agg	R	488	11169.00
---	--	------	------
mPD		0.024	0.89
		nPD:	0.03
		N. weight:	0.69
		Sc. PD:	-0.029
		Sc. rank:	-4162.2

,

PB2			
Pos.	39	obs:	exp:
tta	L	0	0.35
ttg	L	0	0.62
ctt	L	0	0.63
ctc	L	0	0.44
cta	L	0	0.46
ctg	L	3	0.50
cat	H	2	1.13
cac	H	0	0.87
caa	Q	307	24682.00
cag	Q	40070	15695.00
cgt	R	0	0.02
cgc	R	0	0.04
cga	R	0	0.09
cgg	R	1	0.09
aaa	K	0	0.66
aag	K	1	0.34
aga	R	0	0.49
agg	R	0	0.28
---	--	------	------
mPD		0.015	0.48
		nPD:	0.03
		N. weight:	0.95
		Sc. PD:	-0.035
		Sc. rank:	-2378.4

,

PB2			
Pos.	40	obs:	exp:
gat	D	2	1.13
gac	D	0	0.87
gaa	E	39924	23808.00
gag	E	458	16574.00
---	--	------	------
mPD		0.023	0.48
		nPD:	0.05
		N. weight:	0.5
		Sc. PD:	-0.011
		Sc. rank:	3565.7

,

PB2			
Pos.	41	obs:	exp:
cgt	R	0	0.14
cgc	R	0	0.25
cga	R	0	0.64
cgg	R	0	0.60
act	T	0	0.31
acc	T	0	0.18
aca	T	0	0.43
acg	T	1	0.08
aat	N	1	0.56
aac	N	0	0.44
aaa	K	90	26544.00
aag	K	40285	13831.00
aga	R	0	3.43
agg	R	7	1.94
---	--	------	------
mPD		0.0049	0.45
		nPD:	0.01
		N. weight:	1.1
		Sc. PD:	-0.065
		Sc. rank:	-19063.0

,

PB2			
Pos.	42	obs:	exp:
tct	S	0	0.48
tcc	S	0	0.45
tca	S	0	0.81
tcg	S	0	0.12
aat	N	176	22592.00
aac	N	40205	17789.00
agt	S	0	0.52
agc	S	3	0.62
---	--	------	------
mPD		0.0088	0.49
		nPD:	0.02
		N. weight:	0.83
		Sc. PD:	-0.043
		Sc. rank:	-9422.5

,

PB2			
Pos.	43	obs:	exp:
cct	P	123	13457.00
ccc	P	357	7571.00
cca	P	445	13644.00
ccg	P	39459	5712.00
---	--	------	------
mPD		0.045	0.72
		nPD:	0.06
		N. weight:	1.9
		Sc. PD:	-0.014
		Sc. rank:	24795.0

,

PB2			
Pos.	44	obs:	exp:
tct	S	7	6458.00
tcc	S	10	5980.00
tca	S	39944	10756.00
tcg	S	57	1575.00
act	T	0	0.31
acc	T	0	0.18
aca	T	1	0.43
acg	T	0	0.08
agt	S	0	6993.00
agc	S	0	8256.00
gct	A	0	99.48
gcc	A	0	91.19
gca	A	319	147.60
gcg	A	46	26.68
---	--	------	------
mPD		0.024	1.6
		nPD:	0.01
		N. weight:	1.4
		Sc. PD:	-0.075
		Sc. rank:	-19026.0

,

PB2			
Pos.	45	obs:	exp:
cgt	R	0	0.02
cgc	R	0	0.04
cga	R	0	0.09
cgg	R	0	0.09
aga	R	1	0.49
agg	R	0	0.28
---	-	40383	40383.00
---	--	------	------
mPD		0	5.4×10^{-10}
		nPD:	0.
		N. weight:	0.
		Sc. PD:	0
		Sc. rank:	0

,

PB2			
Pos.	46	obs:	exp:
tta	L	0	4686.00
ttg	L	0	8349.00
ctt	L	39938	8448.00
ctc	L	421	5922.00
cta	L	3	6201.00
ctg	L	20	6777.00
cct	P	0	0.33
ccc	P	1	0.19
cca	P	0	0.34
ccg	P	0	0.14
att	I	1	0.35
atc	I	0	0.26
ata	I	0	0.39
---	--	------	------
mPD		0.022	1.2
		nPD:	0.02
		N. weight:	1.6
		Sc. PD:	-0.08
		Sc. rank:	-17049.0

,

PB2			
Pos.	47	obs:	exp:
cgt	R	0	800.70
cgc	R	0	1453.00
cga	R	2	3685.00
cgg	R	3	3468.00
aga	R	8332	19808.00
agg	R	32047	11170.00
---	--	------	------
mPD		0.33	0.89
		nPD:	0.37
		N. weight:	0.69
		Sc. PD:	0.2
		Sc. rank:	21753.0

,

PB2			
Pos.	48	obs:	exp:
att	I	1	5.62
atc	I	0	4.20
ata	I	15	6.18
atg	M	40366	40366.00
act	T	0	0.31
acc	T	0	0.18
aca	T	0	0.43
acg	T	1	0.08
gtt	V	0	0.25
gtc	V	0	0.20
gta	V	0	0.22
gtg	V	1	0.33
---	--	------	------
mPD		0.00089	0.00097
		nPD:	0.92
		N. weight:	0.00042
		Sc. PD:	0.00035
		Sc. rank:	14.0

,

PB2			
Pos.	49	obs:	exp:
cgt	R	0	0.02
cgc	R	0	0.04
cga	R	0	0.09
cgg	R	0	0.09
aaa	K	39864	26549.00
aag	K	519	13834.00
aga	R	1	0.49
agg	R	0	0.28
---	--	------	------
mPD		0.025	0.45
		nPD:	0.06
		N. weight:	0.38
		Sc. PD:	-0.0051
		Sc. rank:	4190.0

,

PB2			
Pos.	50	obs:	exp:
tgg	W	40383	40383.00
cgt	R	0	0.02
cgc	R	0	0.04
cga	R	0	0.09
cgg	R	1	0.09
aga	R	0	0.49
agg	R	0	0.28
---	--	------	------
mPD		0.000050	0.000081
		nPD:	0.61
		N. weight:	0.000064
		Sc. PD:	0.000034
		Sc. rank:	2.1

,

PB2			
Pos.	51	obs:	exp:
tta	L	0	0.23
ttg	L	2	0.41
ctt	L	0	0.42
ctc	L	0	0.29
cta	L	0	0.31
ctg	L	0	0.34
att	I	1	3.16
atc	I	0	2.36
ata	I	8	3.48
atg	M	40365	40365.00
act	T	0	0.93
acc	T	0	0.53
aca	T	0	1.30
acg	T	3	0.24
gtt	V	0	1.00
gtc	V	0	0.82
gta	V	0	0.87
gtg	V	4	1.32
---	-	1	1.00
---	--	------	------
mPD		0.00089	0.0012
		nPD:	0.73
		N. weight:	0.00056
		Sc. PD:	0.00037
		Sc. rank:	18.6

,

PB2			
Pos.	52	obs:	exp:
atg	M	40383	40383.00
gtt	V	0	0.25
gtc	V	0	0.20
gta	V	0	0.22
gtg	V	1	0.33
---	--	------	------
mPD		0.000050	0.000083
		nPD:	0.6
		N. weight:	0.000029
		Sc. PD:	0.000015
		Sc. rank:	1.0

,

PB2			
Pos.	53	obs:	exp:
tct	S	0	0.32
tcc	S	0	0.30
tca	S	2	0.54
tcg	S	0	0.08
agt	S	0	0.35
agc	S	0	0.41
gtt	V	0	0.75
gtc	V	0	0.61
gta	V	3	0.65
gtg	V	0	0.99
gct	A	0	11005.00
gcc	A	43	10088.00
gca	A	40318	16333.00
gcg	A	16	2952.00
gaa	E	0	0.59
gag	E	1	0.41
ggt	G	0	0.13
ggc	G	0	0.14
gga	G	1	0.46
ggg	G	0	0.26
---	--	------	------
mPD		0.0033	0.69
		nPD:	0.
		N. weight:	0.94
		Sc. PD:	-0.061
		Sc. rank:	-24847.0

,

PB2			
Pos.	54	obs:	exp:
cgt	R	0	0.04
cgc	R	0	0.07
cga	R	0	0.18
cgg	R	0	0.17
att	I	0	2.11
atc	I	0	1.58
ata	I	6	2.32
atg	M	40375	40375.00
aga	R	0	0.98
agg	R	2	0.55
gtt	V	0	0.25
gtc	V	0	0.20
gta	V	0	0.22
gtg	V	1	0.33
---	--	------	------
mPD		0.00045	0.00057
		nPD:	0.79
		N. weight:	0.00025
		Sc. PD:	0.00017
		Sc. rank:	8.1

,

PB2			
Pos.	55	obs:	exp:
cgt	R	0	0.34
cgc	R	0	0.61
cga	R	0	1.55
cgg	R	0	1.46
aaa	K	40276	26538.00
aag	K	90	13828.00
aga	R	17	8.34
agg	R	0	4.70
---	-	1	1.00
---	--	------	------
mPD		0.0053	0.45
		nPD:	0.01
		N. weight:	0.43
		Sc. PD:	-0.025
		Sc. rank:	-7050.9

,

PB2			
Pos.	56	obs:	exp:
ttt	F	3	1.46
ttc	F	0	1.54
tat	Y	37929	21278.00
tac	Y	2438	19089.00
tgt	C	1	0.39
tgc	C	0	0.61
cat	H	12	6.77
cac	H	0	5.23
gat	D	1	0.57
gac	D	0	0.43
---	--	------	------
mPD		0.11	0.50
		nPD:	0.23
		N. weight:	0.44
		Sc. PD:	0.069
		Sc. rank:	12626.0

,

PB2			
Pos.	57	obs:	exp:
tct	S	0	0.32
tcc	S	0	0.30
tca	S	2	0.54
tcg	S	0	0.08
cct	P	21	13456.00
ccc	P	2	7570.00
cca	P	40322	13643.00
ccg	P	36	5711.00
caa	Q	1	0.61
cag	Q	0	0.39
agt	S	0	0.35
agc	S	0	0.41
---	--	------	------
mPD		0.0031	0.72
		nPD:	0.
		N. weight:	1.1
		Sc. PD:	-0.073
		Sc. rank:	-30900.0

,

PB2			
Pos.	58	obs:	exp:
tta	L	0	0.23
ttg	L	0	0.41
tct	S	0	0.16
tcc	S	0	0.15
tca	S	0	0.27
tcg	S	0	0.04
ctt	L	1	0.42
ctc	L	1	0.29
cta	L	0	0.31
ctg	L	0	0.34
att	I	17260	14175.00
atc	I	23107	10610.00
ata	I	13	15595.00
agt	S	0	0.17
agc	S	1	0.21
gtt	V	0	0.25
gtc	V	1	0.20
gta	V	0	0.22
gtg	V	0	0.33
---	--	------	------
mPD		0.49	0.66
		nPD:	0.74
		N. weight:	0.56
		Sc. PD:	0.37
		Sc. rank:	18372.0

,

PB2			
Pos.	59	obs:	exp:
tct	S	0	0.32
tcc	S	0	0.30
tca	S	0	0.54
tcg	S	0	0.08
att	I	2	0.70
atc	I	0	0.53
ata	I	0	0.77
act	T	39800	12537.00
acc	T	116	7137.00
aca	T	250	17471.00
acg	T	203	3224.00
aat	N	1	0.56
aac	N	0	0.44
aaa	K	1	0.66
aag	K	0	0.34
agt	S	2	0.35
agc	S	0	0.41
gct	A	9	2.45
gcc	A	0	2.25
gca	A	0	3.64
gcg	A	0	0.66
---	--	------	------
mPD		0.029	0.68
		nPD:	0.04
		N. weight:	1.1
		Sc. PD:	-0.031
		Sc. rank:	5421.9

,

PB2			
Pos.	60	obs:	exp:
tct	S	3	0.48
tcc	S	0	0.45
tca	S	0	0.81
tcg	S	0	0.12
act	T	1	0.31
acc	T	0	0.18
aca	T	0	0.43
acg	T	0	0.08
agt	S	0	0.52
agc	S	0	0.62
gct	A	39945	11005.00
gcc	A	48	10088.00
gca	A	297	16334.00
gcg	A	89	2952.00
gaa	E	1	0.59
gag	E	0	0.41
---	--	------	------
mPD		0.022	0.69
		nPD:	0.03
		N. weight:	1.3
		Sc. PD:	-0.05
		Sc. rank:	-4935.2

,

PB2			
Pos.	61	obs:	exp:
aat	N	0	10.63
aac	N	19	8.37
gtt	V	0	0.50
gtc	V	2	0.41
gta	V	0	0.43
gtg	V	0	0.66
gat	D	45	22804.00
gac	D	40298	17539.00
gaa	E	15	9.43
gag	E	1	6.57
ggt	G	0	0.54
ggc	G	4	0.56
gga	G	0	1.85
ggg	G	0	1.06
---	--	------	------
mPD		0.0043	0.49
		nPD:	0.01
		N. weight:	0.87
		Sc. PD:	-0.053
		Sc. rank:	-17032.0

,

PB2			
Pos.	62	obs:	exp:
cgt	R	0	3.83
cgc	R	0	6.94
cga	R	0	17.61
cgg	R	0	16.57
att	I	0	0.35
atc	I	0	0.26
ata	I	1	0.39
atg	M	1	1.00
aat	N	0	0.56
aac	N	1	0.44
aaa	K	39806	26420.00
aag	K	381	13767.00
aga	R	48	94.66
agg	R	145	53.38
gaa	E	1	0.59
gag	E	0	0.41
---	--	------	------
mPD		0.035	0.46
		nPD:	0.08
		N. weight:	0.39
		Sc. PD:	0.0026
		Sc. rank:	6816.8

,

PB2			
Pos.	63	obs:	exp:
tct	S	0	0.16
tcc	S	0	0.15
tca	S	0	0.27
tcg	S	0	0.04
cgt	R	0	799.00
cgc	R	0	1450.00
cga	R	0	3677.00
cgg	R	185	3460.00
atg	M	4	4.00
aaa	K	2	41.42
aag	K	61	21.58
agt	S	1	0.17
agc	S	0	0.21
aga	R	762	19765.00
agg	R	39349	11146.00
ggt	G	0	2.69
ggc	G	0	2.79
gga	G	0	9.23
ggg	G	20	5.28
---	--	------	------
mPD		0.051	0.89
		nPD:	0.06
		N. weight:	1.2
		Sc. PD:	-0.016
		Sc. rank:	13811.0

,

PB2			
Pos.	64	obs:	exp:
tta	L	2	0.35
ttg	L	0	0.62
ctt	L	0	0.63
ctc	L	0	0.44
cta	L	1	0.46
ctg	L	0	0.50
att	I	1	12961.00
atc	I	52	9702.00
ata	I	36870	14260.00
atg	M	6	6.00
act	T	0	1.86
acc	T	0	1.06
aca	T	6	2.60
acg	T	0	0.48
aaa	K	0	0.66
aag	K	1	0.34
gtt	V	0	862.40
gtc	V	480	701.50
gta	V	2740	746.20
gtg	V	223	1133.00
gct	A	0	0.27
gcc	A	0	0.25
gca	A	1	0.40
gcg	A	0	0.07
ggt	G	0	0.13
ggc	G	0	0.14
gga	G	1	0.46
ggg	G	0	0.26
---	--	------	------
mPD		0.19	0.83
		nPD:	0.23
		N. weight:	0.99
		Sc. PD:	0.16
		Sc. rank:	28482.0

,

PB2			
Pos.	65	obs:	exp:
att	I	2	730.50
atc	I	0	546.80
ata	I	2079	803.70
atg	M	356	356.00
act	T	5	11777.00
acc	T	2	6705.00
aca	T	37873	16413.00
acg	T	44	3029.00
aaa	K	1	0.66
aag	K	0	0.34
gtt	V	0	0.25
gtc	V	0	0.20
gta	V	0	0.22
gtg	V	1	0.33
gct	A	0	5.72
gcc	A	0	5.25
gca	A	21	8.49
gcg	A	0	1.54
---	--	------	------
mPD		0.13	0.80
		nPD:	0.17
		N. weight:	0.87
		Sc. PD:	0.085
		Sc. rank:	22443.0

,

PB2			
Pos.	66	obs:	exp:
caa	Q	1	0.61
cag	Q	0	0.39
aat	N	0	6.15
aac	N	11	4.85
aaa	K	17	11.18
aag	K	0	5.82
gat	D	0	196.10
gac	D	347	150.90
gaa	E	39942	23582.00
gag	E	58	16418.00
ggt	G	0	1.08
ggc	G	1	1.12
gga	G	7	3.69
ggg	G	0	2.11
---	--	------	------
mPD		0.022	0.49
		nPD:	0.05
		N. weight:	0.55
		Sc. PD:	-0.013
		Sc. rank:	3451.4

,

PB2			
Pos.	67	obs:	exp:
tta	L	0	0.46
ttg	L	0	0.83
ctt	L	0	0.84
ctc	L	0	0.59
cta	L	0	0.61
ctg	L	4	0.67
att	I	49	101.80
atc	I	0	76.20
ata	I	241	112.00
atg	M	40045	40045.00
act	T	0	3.73
acc	T	0	2.12
aca	T	0	5.19
acg	T	12	0.96
aaa	K	0	2.63
aag	K	4	1.37
gtt	V	0	7.26
gtc	V	0	5.91
gta	V	0	6.29
gtg	V	29	9.54
---	--	------	------
mPD		0.017	0.018
		nPD:	0.9
		N. weight:	0.0059
		Sc. PD:	0.0048
		Sc. rank:	194.9

,

PB2			
Pos.	68	obs:	exp:
ttt	F	1	0.49
ttc	F	0	0.51
tta	L	1	0.23
ttg	L	0	0.41
ctt	L	1	0.42
ctc	L	0	0.29
cta	L	0	0.31
ctg	L	0	0.34
att	I	837	400.90
atc	I	303	300.10
ata	I	2	441.00
gtt	V	38815	9826.00
gtc	V	388	7993.00
gta	V	25	8502.00
gtg	V	0	12907.00
gct	A	7	1.91
gcc	A	0	1.75
gca	A	0	2.83
gcg	A	0	0.51
gat	D	4	2.26
gac	D	0	1.74
---	--	------	------
mPD		0.091	0.80
		nPD:	0.11
		N. weight:	1.4
		Sc. PD:	0.059
		Sc. rank:	30337.0

,

PB2			
Pos.	69	obs:	exp:
cct	P	3600	13456.00
ccc	P	3	7570.00
cca	P	2272	13643.00
ccg	P	34506	5711.00
caa	Q	0	0.61
cag	Q	1	0.39
act	T	0	0.62
acc	T	0	0.35
aca	T	0	0.87
acg	T	2	0.16
---	--	------	------
mPD		0.26	0.72
		nPD:	0.36
		N. weight:	1.4
		Sc. PD:	0.4
		Sc. rank:	43422.0

,

PB2			
Pos.	70	obs:	exp:
aaa	K	0	3.94
aag	K	6	2.06
gtt	V	0	0.25
gtc	V	0	0.20
gta	V	0	0.22
gtg	V	1	0.33
gat	D	13	7.91
gac	D	1	6.09
gaa	E	578	23797.00
gag	E	39785	16566.00
---	--	------	------
mPD		0.029	0.48
		nPD:	0.06
		N. weight:	0.85
		Sc. PD:	-0.0081
		Sc. rank:	10184.0

,

PB2			
Pos.	71	obs:	exp:
tct	S	0	0.16
tcc	S	0	0.15
tca	S	0	0.27
tcg	S	0	0.04
cgt	R	0	799.60
cgc	R	0	1451.00
cga	R	2	3680.00
cgg	R	0	3463.00
aaa	K	53	34.84
aag	K	0	18.16
agt	S	0	0.17
agc	S	1	0.21
aga	R	39856	19781.00
agg	R	471	11155.00
ggt	G	0	0.13
ggc	G	0	0.14
gga	G	1	0.46
ggg	G	0	0.26
---	--	------	------
mPD		0.026	0.89
		nPD:	0.03
		N. weight:	0.69
		Sc. PD:	-0.028
		Sc. rank:	-3638.5

,

PB2			
Pos.	72	obs:	exp:
aat	N	40117	22593.00
aac	N	266	17790.00
gat	D	1	0.57
gac	D	0	0.43
---	--	------	------
mPD		0.013	0.49
		nPD:	0.03
		N. weight:	0.57
		Sc. PD:	-0.024
		Sc. rank:	-3795.2

,

PB2			
Pos.	73	obs:	exp:
aaa	K	5	3.29
aag	K	0	1.71
gtt	V	0	0.75
gtc	V	0	0.61
gta	V	3	0.65
gtg	V	0	0.99
gat	D	2	3.39
gac	D	4	2.61
gaa	E	40210	23799.00
gag	E	157	16568.00
ggt	G	0	0.40
ggc	G	0	0.42
gga	G	2	1.38
ggg	G	1	0.79
---	--	------	------
mPD		0.0086	0.48
		nPD:	0.02
		N. weight:	0.53
		Sc. PD:	-0.027
		Sc. rank:	-6048.7

,

PB2			
Pos.	74	obs:	exp:
cat	H	2	6.77
cac	H	10	5.23
caa	Q	40249	24625.00
cag	Q	36	15660.00
cgt	R	0	0.10
cgc	R	0	0.18
cga	R	5	0.46
cgg	R	0	0.43
aaa	K	27	17.75
aag	K	0	9.25
aga	R	0	2.45
agg	R	0	1.38
gaa	E	55	32.43
gag	E	0	22.57
---	--	------	------
mPD		0.0067	0.48
		nPD:	0.01
		N. weight:	0.51
		Sc. PD:	-0.028
		Sc. rank:	-7374.6

,

PB2			
Pos.	75	obs:	exp:
cgt	R	0	0.14
cgc	R	0	0.25
cga	R	0	0.64
cgg	R	0	0.60
aga	R	7	3.43
agg	R	0	1.94
gaa	E	11	6.49
gag	E	0	4.51
ggt	G	13	5434.00
ggc	G	5	5634.00
gga	G	39799	18634.00
ggg	G	549	10664.00
---	--	------	------
mPD		0.029	0.68
		nPD:	0.04
		N. weight:	0.74
		Sc. PD:	-0.02
		Sc. rank:	3360.2

,

PB2			
Pos.	76	obs:	exp:
cat	H	2	2.26
cac	H	2	1.74
caa	Q	40228	24678.00
cag	Q	144	15694.00
cgt	R	0	0.08
cgc	R	0	0.14
cga	R	4	0.36
cgg	R	0	0.34
aaa	K	2	1.31
aag	K	0	0.69
aga	R	0	1.96
agg	R	0	1.11
gaa	E	2	1.18
gag	E	0	0.82
---	--	------	------
mPD		0.0077	0.48
		nPD:	0.02
		N. weight:	0.5
		Sc. PD:	-0.026
		Sc. rank:	-6174.4

,

PB2			
Pos.	77	obs:	exp:
tct	S	0	0.32
tcc	S	0	0.30
tca	S	0	0.54
tcg	S	0	0.08
cct	P	5	1.67
ccc	P	0	0.94
cca	P	0	1.69
ccg	P	0	0.71
att	I	35	13.69
atc	I	4	10.25
ata	I	0	15.06
act	T	36240	12512.00
acc	T	3903	7123.00
aca	T	90	17436.00
acg	T	56	3218.00
aat	N	19	10.63
aac	N	0	8.37
agt	S	2	0.35
agc	S	0	0.41
gct	A	29	8.18
gcc	A	1	7.50
gca	A	0	12.14
gcg	A	0	2.19
---	--	------	------
mPD		0.19	0.68
		nPD:	0.27
		N. weight:	0.93
		Sc. PD:	0.18
		Sc. rank:	27237.0

,

PB2			
Pos.	78	obs:	exp:
tta	L	32	4686.00
ttg	L	15	8348.00
ctt	L	20	8447.00
ctc	L	385	5921.00
cta	L	35137	6201.00
ctg	L	4791	6777.00
att	I	0	0.35
atc	I	0	0.26
ata	I	1	0.39
atg	M	3	3.00
---	--	------	------
mPD		0.23	1.2
		nPD:	0.2
		N. weight:	1.5
		Sc. PD:	0.19
		Sc. rank:	41542.0

,

PB2			
Pos.	79	obs:	exp:
tgg	W	40383	40383.00
cgt	R	0	0.02
cgc	R	0	0.04
cga	R	0	0.09
cgg	R	0	0.09
aga	R	0	0.49
agg	R	1	0.28
---	--	------	------
mPD		0.000050	0.000081
		nPD:	0.61
		N. weight:	0.000034
		Sc. PD:	0.000018
		Sc. rank:	1.1

,

PB2			
Pos.	80	obs:	exp:
tct	S	0	6511.00
tcc	S	0	6030.00
tca	S	0	10845.00
tcg	S	0	1588.00
tgt	C	4	2.34
tgc	C	2	3.66
att	I	9	3.86
atc	I	1	2.89
ata	I	1	4.25
aat	N	7	5.04
aac	N	2	3.96
agt	S	36661	7051.00
agc	S	3687	8324.00
ggt	G	8	1.35
ggc	G	2	1.40
gga	G	0	4.62
ggg	G	0	2.64
---	--	------	------
mPD		0.17	1.6
		nPD:	0.1
		N. weight:	1.5
		Sc. PD:	0.049
		Sc. rank:	31290.0

,

PB2			
Pos.	81	obs:	exp:
tct	S	0	0.16
tcc	S	0	0.15
tca	S	0	0.27
tcg	S	0	0.04
cgt	R	0	2.86
cgc	R	0	5.18
cga	R	0	13.14
cgg	R	0	12.36
att	I	0	0.35
atc	I	0	0.26
ata	I	1	0.39
act	T	0	0.93
acc	T	0	0.53
aca	T	3	1.30
acg	T	0	0.24
aat	N	0	0.56
aac	N	1	0.44
aaa	K	40189	26448.00
aag	K	40	13781.00
agt	S	1	0.17
agc	S	0	0.21
aga	R	144	70.63
agg	R	0	39.83
gaa	E	5	2.95
gag	E	0	2.05
---	--	------	------
mPD		0.0097	0.46
		nPD:	0.02
		N. weight:	0.44
		Sc. PD:	-0.021
		Sc. rank:	-4349.0

,

PB2			
Pos.	82	obs:	exp:
tta	L	0	0.93
ttg	L	2	1.65
tct	S	0	0.16
tcc	S	0	0.15
tca	S	0	0.27
tcg	S	0	0.04
ctt	L	0	1.67
ctc	L	0	1.17
cta	L	0	1.23
ctg	L	6	1.34
cgt	R	0	0.06
cgc	R	0	0.11
cga	R	0	0.27
cgg	R	0	0.26
att	I	12	262.90
atc	I	0	196.80
ata	I	737	289.30
atg	M	39223	39223.00
act	T	0	110.20
acc	T	0	62.76
aca	T	308	153.60
acg	T	47	28.35
agt	S	1	0.17
agc	S	0	0.21
aga	R	0	1.47
agg	R	3	0.83
gtt	V	0	11.02
gtc	V	0	8.97
gta	V	0	9.54
gtg	V	44	14.48
gct	A	0	0.27
gcc	A	0	0.25
gca	A	1	0.40
gcg	A	0	0.07
---	--	------	------
mPD		0.071	0.075
		nPD:	0.96
		N. weight:	0.025
		Sc. PD:	0.022
		Sc. rank:	830.3

,

PB2

Pos.	83	obs :	exp :
tct	S	0	6405.00
tcc	S	0	5932.00
tca	S	0	10669.00
tcg	S	0	1563.00
tgt	C	1	0.39
tgc	C	0	0.61
cgt	R	5	0.12
cgc	R	0	0.22
cga	R	0	0.55
cgg	R	0	0.52
att	I	9	3.16
atc	I	0	2.36
ata	I	0	3.48
act	T	13	8.07
acc	T	13	4.60
aca	T	0	11.25
acg	T	0	2.08
aat	N	277	344.60
aac	N	339	271.40
agt	S	39623	6937.00
agc	S	71	8189.00
aga	R	1	2.94
agg	R	0	1.66
gat	D	0	0.57
gac	D	1	0.43
ggt	G	31	4.17
ggc	G	0	4.33
gga	G	0	14.31
ggg	G	0	8.19
---	--	------	------
mPD		0.055	1.7
		nPD :	0.03
		N. weight :	1.8
		Sc. PD :	-0.065
		Sc. rank :	-3155.1

,

PB2

Pos.	84	obs :	exp :
caa	Q	1	0.61
cag	Q	0	0.39
gat	D	40353	22827.00
gac	D	30	17556.00
---	--	------	------
mPD		0.0016	0.49
		nPD :	0.
		N. weight :	0.6
		Sc. PD :	-0.039
		Sc. rank :	-16959.0

,

PB2

Pos.	85	obs :	exp :
tct	S	0	0.16
tcc	S	0	0.15
tca	S	0	0.27
tcg	S	0	0.04
act	T	7	2.17
acc	T	0	1.24
aca	T	0	3.03
acg	T	0	0.56
agt	S	1	0.17
agc	S	0	0.21
gtt	V	3	0.75
gtc	V	0	0.61
gta	V	0	0.65
gtg	V	0	0.99
gct	A	40215	11004.00
gcc	A	139	10087.00
gca	A	11	16331.00
gcg	A	8	2951.00
---	--	------	------
mPD		0.0084	0.69
		nPD :	0.01
		N. weight :	1.3
		Sc. PD :	-0.077
		Sc. rank :	-21466.0

,

PB2

Pos.	86	obs :	exp :
act	T	0	0.31
acc	T	0	0.18
aca	T	1	0.43
acg	T	0	0.08
gaa	E	1	0.59
gag	E	0	0.41
ggt	G	1	5436.00
ggc	G	8	5636.00
gga	G	36911	18641.00
ggg	G	3462	10669.00
---	--	------	------
mPD		0.16	0.68
		nPD :	0.23
		N. weight :	0.56
		Sc. PD :	0.088
		Sc. rank :	15966.0

,

PB2

Pos.	87	obs :	exp :
tct	S	10	6515.00
tcc	S	0	6034.00
tca	S	40163	10852.00
tcg	S	202	1589.00
cct	P	0	0.33
ccc	P	0	0.19
cca	P	1	0.34
ccg	P	0	0.14
act	T	0	2.48
acc	T	0	1.41
aca	T	8	3.46
acg	T	0	0.64
agt	S	0	7056.00
agc	S	0	8329.00
---	--	------	------
mPD		0.011	1.6
		nPD :	0.01
		N. weight :	1.4
		Sc. PD :	-0.085
		Sc. rank :	-30708.0

,

PB2

Pos.	88	obs :	exp :
aat	N	11	10.63
aac	N	8	8.37
gat	D	29749	22808.00
gac	D	10600	17541.00
gaa	E	4	5.31
gag	E	5	3.69
ggt	G	5	0.94
ggc	G	2	0.98
gga	G	0	3.23
ggg	G	0	1.85
---	--	------	------
mPD		0.39	0.49
		nPD :	0.79
		N. weight :	0.067
		Sc. PD :	0.048
		Sc. rank :	2230.2

,

PB2

Pos.	89	obs :	exp :
cgt	R	146	799.60
cgc	R	0	1451.00
cga	R	986	3680.00
cgg	R	3	3463.00
aaa	K	53	34.84
aag	K	0	18.16
aga	R	39106	19780.00
agg	R	87	11155.00
ggt	G	0	0.40
ggc	G	0	0.42
gga	G	3	1.38
ggg	G	0	0.79
---	--	------	------
mPD		0.069	0.89
		nPD :	0.08
		N. weight :	0.64
		Sc. PD :	0.0049
		Sc. rank :	11389.0

,

PB2

Pos.	90	obs :	exp :
tta	L	0	0.35
ttg	L	2	0.62
ctt	L	0	0.63
ctc	L	0	0.44
cta	L	0	0.46
ctg	L	1	0.50
atg	M	38	38.00
gtt	V	52	10104.00
gtc	V	0	8220.00
gta	V	606	8743.00
gtg	V	39682	13273.00
gct	A	0	0.82
gcc	A	0	0.75
gca	A	1	1.21
gcg	A	2	0.22
---	--	------	------
mPD		0.034	0.74
		nPD :	0.05
		N. weight :	1.1
		Sc. PD :	-0.025
		Sc. rank :	7434.0

,

PB2

Pos.	91	obs :	exp :
tta	L	0	0.35
ttg	L	2	0.62
ctt	L	0	0.63
ctc	L	0	0.44
cta	L	0	0.46
ctg	L	1	0.50
att	I	5	8.07
atc	I	0	6.04
ata	I	18	8.88
atg	M	40323	40323.00
act	T	0	1.24
acc	T	0	0.71
aca	T	0	1.73
acg	T	4	0.32
gtt	V	0	7.76
gtc	V	0	6.32
gta	V	0	6.72
gtg	V	31	10.20
---	--	------	------
mPD		0.0030	0.0043
		nPD :	0.7
		N. weight :	0.0016
		Sc. PD :	0.00097
		Sc. rank :	51.9

,

PB2

Pos.	92	obs :	exp :
tat	Y	0	0.53
tac	Y	1	0.47
att	I	0	10.53
atc	I	0	7.88
ata	I	30	11.59
gtt	V	8	10106.00
gtc	V	2	8221.00
gta	V	40111	8745.00
gtg	V	227	13275.00
gct	A	0	1.36
gcc	A	0	1.25
gca	A	5	2.02
gcg	A	0	0.37
---	--	------	------
mPD		0.014	0.74
		nPD :	0.02
		N. weight :	1.6
		Sc. PD :	-0.08
		Sc. rank :	-17529.0

,

PB2

Pos.	93	obs :	exp :
tct	S	3	6516.00
tcc	S	15	6034.00
tca	S	39602	10853.00
tcg	S	760	1590.00
cct	P	0	0.33
ccc	P	0	0.19
cca	P	1	0.34
ccg	P	0	0.14
act	T	0	0.93
acc	T	0	0.53
aca	T	3	1.30
acg	T	0	0.24
agt	S	0	7057.00
agc	S	0	8330.00
---	--	------	------
mPD		0.038	1.6
		nPD :	0.02
		N. weight :	1.3
		Sc. PD :	-0.061
		Sc. rank :	-11150.0

,

PB2

Pos.	94	obs :	exp :
tta	L	0	0.58
ttg	L	0	1.03
tct	S	16	2.90
tcc	S	1	2.69
tca	S	1	4.84
tcg	S	0	0.71
ctt	L	5	1.05
ctc	L	0	0.73
cta	L	0	0.77
ctg	L	0	0.84
cct	P	40057	13446.00
ccc	P	255	7565.00
cca	P	14	13633.00
ccg	P	25	5707.00
cat	H	3	1.69
cac	H	0	1.31
act	T	6	1.86
acc	T	0	1.06
aca	T	0	2.60
acg	T	0	0.48
agt	S	0	3.15
agc	S	0	3.71
gct	A	1	0.27
gcc	A	0	0.25
gca	A	0	0.40
gcg	A	0	0.07
---	--	------	------
mPD		0.016	0.72
		nPD :	0.02
		N. weight :	1.1
		Sc. PD :	-0.052
		Sc. rank :	-10133.0

,

PB2

Pos.	95	obs :	exp :
tta	L	286	4685.00
ttg	L	39653	8347.00
tct	S	0	1.45
tcc	S	0	1.34
tca	S	0	2.42
tcg	S	9	0.35
ctt	L	0	8445.00
ctc	L	0	5920.00
cta	L	1	6199.00
ctg	L	431	6775.00
cct	P	0	0.33
ccc	P	0	0.19
cca	P	0	0.34
ccg	P	1	0.14
atg	M	2	2.00
agt	S	0	1.57
agc	S	0	1.86
gtt	V	0	0.25
gtc	V	0	0.20
gta	V	0	0.22
gtg	V	1	0.33
---	--	------	------
mPD		0.036	1.2
		nPD :	0.03
		N. weight :	1.6
		Sc. PD :	-0.06
		Sc. rank :	-6111.7

,

PB2

Pos.	96	obs :	exp :
tct	S	0	0.16
tcc	S	0	0.15
tca	S	0	0.27
tcg	S	0	0.04
act	T	3	0.93
acc	T	0	0.53
aca	T	0	1.30
acg	T	0	0.24
agt	S	1	0.17
agc	S	0	0.21
gtt	V	1	0.25
gtc	V	0	0.20
gta	V	0	0.22
gtg	V	0	0.33
gct	A	39627	11005.00
gcc	A	308	10088.00
gca	A	421	16334.00
gcg	A	23	2952.00
---	--	------	------
mPD		0.037	0.69
		nPD :	0.05
		N. weight :	1.3
		Sc. PD :	-0.021
		Sc. rank :	12158.0

,

PB2			
Pos.	97	obs:	exp:
cgt	R	0	0.02
cgc	R	0	0.04
cga	R	0	0.09
cgg	R	0	0.09
att	I	0	11.94
atc	I	0	8.93
ata	I	34	13.13
aga	R	1	0.49
agg	R	0	0.28
gtt	V	2	10106.00
gtc	V	6	8221.00
gta	V	39559	8745.00
gtg	V	781	13275.00
gct	A	0	0.27
gcc	A	0	0.25
gca	A	1	0.40
gcg	A	0	0.07
---	--	------	------
mPD		0.040	0.74
		nPD:	0.05
		N. weight:	1.5
		Sc. PD:	-0.024
		Sc. rank:	15128.0

,

PB2			
Pos.	98	obs:	exp:
tta	L	0	0.12
ttg	L	0	0.21
tct	S	0	0.16
tcc	S	0	0.15
tca	S	1	0.27
tcg	S	0	0.04
tgt	C	0	0.39
tgc	C	1	0.61
ctt	L	0	0.21
ctc	L	0	0.15
cta	L	0	0.15
ctg	L	1	0.17
att	I	0	0.35
atc	I	0	0.26
ata	I	1	0.39
act	T	7	12540.00
acc	T	5	7139.00
aca	T	40191	17476.00
acg	T	177	3225.00
agt	S	0	0.17
agc	S	0	0.21
---	--	------	------
mPD		0.0097	0.68
		nPD:	0.01
		N. weight:	0.86
		Sc. PD:	-0.047
		Sc. rank:	-12179.0

,

PB2			
Pos.	99	obs:	exp:
tat	Y	1	0.53
tac	Y	0	0.47
tgg	W	40382	40382.00
cgt	R	0	0.02
cgc	R	0	0.04
cga	R	0	0.09
cgg	R	1	0.09
aga	R	0	0.49
agg	R	0	0.28
---	--	------	------
mPD		0.00015	0.00018
		nPD:	0.82
		N. weight:	0.000081
		Sc. PD:	0.00006
		Sc. rank:	2.7

,

PB2			
Pos.	100	obs:	exp:
tct	S	0	0.16
tcc	S	0	0.15
tca	S	1	0.27
tcg	S	0	0.04
tat	Y	0	0.53
tac	Y	1	0.47
tgg	W	40382	40382.00
agt	S	0	0.17
agc	S	0	0.21
---	--	------	------
mPD		0.00020	0.00020
		nPD:	1.01
		N. weight:	0.000054
		Sc. PD:	0.00005
		Sc. rank:	1.8

,

PB2			
Pos.	101	obs:	exp:
ttt	F	1	0.49
ttc	F	0	0.51
tct	S	0	0.32
tcc	S	0	0.30
tca	S	0	0.54
tcg	S	0	0.08
act	T	1	0.31
acc	T	0	0.18
aca	T	0	0.43
acg	T	0	0.08
aat	N	40277	22590.00
aac	N	100	17787.00
aaa	K	2	1.31
aag	K	0	0.69
agt	S	2	0.35
agc	S	0	0.41
gat	D	1	0.57
gac	D	0	0.43
---	--	------	------
mPD		0.0053	0.49
		nPD:	0.01
		N. weight:	0.59
		Sc. PD:	-0.035
		Sc. rank:	-10260.0

,

PB2			
Pos.	102	obs:	exp:
tct	S	0	0.16
tcc	S	0	0.15
tca	S	0	0.27
tcg	S	0	0.04
tgg	W	1	1.00
cgt	R	0	800.60
cgc	R	0	1453.00
cga	R	0	3684.00
cgg	R	2	3467.00
act	T	0	0.31
acc	T	0	0.18
aca	T	1	0.43
acg	T	0	0.08
agt	S	1	0.17
agc	S	0	0.21
aga	R	2330	19805.00
agg	R	38047	11169.00
ggt	G	0	0.27
ggc	G	0	0.28
gga	G	0	0.92
ggg	G	2	0.53
---	--	------	------
mPD		0.11	0.89
		nPD:	0.12
		N. weight:	1.1
		Sc. PD:	0.057
		Sc. rank:	24605.0

,

PB2			
Pos.	103	obs:	exp:
tct	S	0	5.16
tcc	S	0	4.78
tca	S	0	8.60
tcg	S	0	1.26
tgt	C	1	0.39
tgc	C	0	0.61
act	T	2	0.93
acc	T	0	0.53
aca	T	1	1.30
acg	T	0	0.24
aat	N	40234	22561.00
aac	N	91	17764.00
aaa	K	11	7.23
aag	K	0	3.77
agt	S	31	5.59
agc	S	1	6.60
gat	D	12	6.78
gac	D	0	5.22
---	--	------	------
mPD		0.0076	0.50
		nPD:	0.02
		N. weight:	0.6
		Sc. PD:	-0.032
		Sc. rank:	-8096.9

,

PB2			
Pos.	104	obs:	exp:
cgt	R	0	0.02
cgc	R	0	0.04
cga	R	0	0.09
cgg	R	0	0.09
aga	R	1	0.49
agg	R	0	0.28
gtt	V	0	0.25
gtc	V	0	0.20
gta	V	1	0.22
gtg	V	0	0.33
ggt	G	51	5436.00
ggc	G	314	5636.00
gga	G	37651	18641.00
ggg	G	2366	10669.00
---	--	------	------
mPD		0.13	0.68
		nPD:	0.19
		N. weight:	0.57
		Sc. PD:	0.066
		Sc. rank:	14933.0

,

PB2			
Pos.	105	obs:	exp:
tta	L	0	0.35
ttg	L	0	0.62
tct	S	0	1.29
tcc	S	8	1.20
tca	S	0	2.15
tcg	S	0	0.31
ctt	L	0	0.63
ctc	L	3	0.44
cta	L	0	0.46
ctg	L	0	0.50
cct	P	497	13451.00
ccc	P	39017	7567.00
cca	P	845	13637.00
ccg	P	6	5709.00
act	T	0	2.48
acc	T	7	1.41
aca	T	1	3.46
acg	T	0	0.64
agt	S	0	1.40
agc	S	0	1.65
---	--	------	------
mPD		0.066	0.72
		nPD:	0.09
		N. weight:	1.6
		Sc. PD:	0.033
		Sc. rank:	30672.0

,

PB2			
Pos.	106	obs:	exp:
tta	L	0	2.32
ttg	L	18	4.13
ctt	L	0	4.18
ctc	L	0	2.93
cta	L	2	3.07
ctg	L	0	3.36
att	I	0	1.76
atc	I	0	1.31
ata	I	5	1.93
atg	M	90	90.00
act	T	0	109.60
acc	T	0	62.41
aca	T	353	152.80
acg	T	0	28.19
gtt	V	41	9992.00
gtc	V	1	8128.00
gta	V	213	8646.00
gtg	V	39638	13126.00
gct	A	0	5.45
gcc	A	0	5.00
gca	A	1	8.09
gcg	A	19	1.46
gaa	E	0	0.59
gag	E	1	0.41
ggt	G	0	0.27
ggc	G	0	0.28
gga	G	0	0.92
ggg	G	2	0.53
---	--	------	------
mPD		0.071	0.78
		nPD:	0.09
		N. weight:	1.1
		Sc. PD:	0.024
		Sc. rank:	21914.0

,

PB2			
Pos.	107	obs:	exp:
tct	S	0	1.45
tcc	S	0	1.34
tca	S	9	2.42
tcg	S	0	0.35
att	I	0	3.86
atc	I	0	2.89
ata	I	11	4.25
act	T	5	12341.00
acc	T	0	7026.00
aca	T	39688	17198.00
acg	T	45	3174.00
aaa	K	9	6.57
aag	K	1	3.43
agt	S	0	1.57
agc	S	0	1.86
gct	A	0	167.60
gcc	A	0	153.70
gca	A	612	248.80
gcg	A	3	44.96
---	-	1	1.00
---	--	------	------
mPD		0.034	0.71
		nPD:	0.05
		N. weight:	0.88
		Sc. PD:	-0.019
		Sc. rank:	6878.5

,

PB2			
Pos.	108	obs:	exp:
tct	S	0	3985.00
tcc	S	0	3691.00
tca	S	0	6638.00
tcg	S	0	972.20
tgt	C	4	1.56
tgc	C	0	2.44
att	I	2	0.70
atc	I	0	0.53
ata	I	0	0.77
act	T	1	0.31
acc	T	0	0.18
aca	T	0	0.43
acg	T	0	0.08
aat	N	7197	4028.00
aac	N	3	3172.00
aaa	K	0	0.66
aag	K	1	0.34
agt	S	24460	4316.00
agc	S	238	5095.00
gat	D	8416	4779.00
gac	D	38	3675.00
ggt	G	23	3.10
ggc	G	0	3.21
gga	G	0	10.62
ggg	G	0	6.08
---	-	1	1.00
---	--	------	------
mPD		0.82	1.9
		nPD:	0.42
		N. weight:	1.3
		Sc. PD:	0.46
		Sc. rank:	41704.0

,

PB2

Pos.	109	obs:	exp:
atg	M	13	13.00
act	T	251	12532.00
acc	T	0	7135.00
aca	T	2137	17465.00
acg	T	37967	3223.00
aaa	K	0	0.66
aag	K	1	0.34
gtt	V	0	2.50
gtc	V	0	2.04
gta	V	0	2.17
gtg	V	10	3.29
gct	A	0	1.09
gcc	A	0	1.00
gca	A	0	1.62
gcg	A	4	0.29
gaa	E	0	0.59
gag	E	1	0.41
---	--	------	------
mPD		0.11	0.68
		nPD:	0.17
		N. weight:	2.3
		Sc. PD:	0.22
		Sc. rank:	59059.0

,

PB2

Pos.	110	obs:	exp:
ttt	F	0	2.92
ttc	F	6	3.08
att	I	2	50.20
atc	I	141	37.57
ata	I	0	55.23
gtt	V	778	10074.00
gtc	V	39434	8195.00
gta	V	6	8717.00
gtg	V	1	13233.00
gct	A	0	4.36
gcc	A	16	4.00
gca	A	0	6.47
gcg	A	0	1.17
---	--	------	------
mPD		0.046	0.75
		nPD:	0.06
		N. weight:	1.6
		Sc. PD:	-0.013
		Sc. rank:	19630.0

,

PB2

Pos.	111	obs:	exp:
tta	L	0	0.35
ttg	L	0	0.62
tat	Y	4	2.11
tac	Y	0	1.89
ctt	L	3	0.63
ctc	L	0	0.44
cta	L	0	0.46
ctg	L	0	0.50
cat	H	38904	22766.00
cac	H	1459	17597.00
caa	Q	5	3.06
cag	Q	0	1.94
cgt	R	3	0.06
cgc	R	0	0.11
cga	R	0	0.27
cgg	R	0	0.26
aat	N	6	3.36
aac	N	0	2.64
aga	R	0	1.47
agg	R	0	0.83
---	--	------	------
mPD		0.071	0.49
		nPD:	0.14
		N. weight:	0.45
		Sc. PD:	0.033
		Sc. rank:	10821.0

,

PB2

Pos.	112	obs:	exp:
ttt	F	0	0.97
ttc	F	2	1.03
tat	Y	557	21284.00
tac	Y	39822	19095.00
cat	H	1	1.13
cac	H	1	0.87
aat	N	0	0.56
aac	N	1	0.44
---	--	------	------
mPD		0.028	0.50
		nPD:	0.06
		N. weight:	0.71
		Sc. PD:	-0.01
		Sc. rank:	7498.0

,

PB2

Pos.	113	obs:	exp:
tta	L	0	1.04
ttg	L	0	1.86
tct	S	0	2.90
tcc	S	1	2.69
tca	S	17	4.84
tcg	S	0	0.71
ctt	L	0	1.88
ctc	L	0	1.32
cta	L	9	1.38
ctg	L	0	1.51
cct	P	367	13443.00
ccc	P	106	7563.00
cca	P	39833	13629.00
ccg	P	35	5706.00
caa	Q	12	7.34
cag	Q	0	4.66
act	T	0	1.24
acc	T	0	0.71
aca	T	4	1.73
acg	T	0	0.32
agt	S	0	3.15
agc	S	0	3.71
---	--	------	------
mPD		0.027	0.72
		nPD:	0.04
		N. weight:	1.1
		Sc. PD:	-0.034
		Sc. rank:	1525.8

,

PB2

Pos.	114	obs:	exp:
tat	Y	0	0.53
tac	Y	1	0.47
cgt	R	0	1.05
cgc	R	0	1.91
cga	R	0	4.84
cgg	R	0	4.55
act	T	0	0.93
acc	T	0	0.53
aca	T	3	1.30
acg	T	0	0.24
aat	N	0	0.56
aac	N	1	0.44
aaa	K	38989	26512.00
aag	K	1337	13814.00
aga	R	53	26.00
agg	R	0	14.66
---	--	------	------
mPD		0.067	0.45
		nPD:	0.15
		N. weight:	0.31
		Sc. PD:	0.024
		Sc. rank:	7611.4

,

PB2

Pos.	115	obs:	exp:
tta	L	0	0.12
ttg	L	1	0.21
ctt	L	0	0.21
ctc	L	0	0.15
cta	L	0	0.15
ctg	L	0	0.17
att	I	0	1.40
atc	I	4	1.05
ata	I	0	1.54
atg	M	1	1.00
gtt	V	17	10114.00
gtc	V	258	8227.00
gta	V	961	8751.00
gtg	V	39141	13285.00
gct	A	0	0.27
gcc	A	0	0.25
gca	A	0	0.40
gcg	A	1	0.07
---	--	------	------
mPD		0.060	0.74
		nPD:	0.08
		N. weight:	1.
		Sc. PD:	0.011
		Sc. rank:	18553.0

,

PB2

Pos.	116	obs:	exp:
tat	Y	249	21286.00
tac	Y	40134	19097.00
tgt	C	0	0.39
tgc	C	1	0.61
---	--	------	------
mPD		0.012	0.50
		nPD:	0.02
		N. weight:	0.75
		Sc. PD:	-0.033
		Sc. rank:	-5953.1

,

PB2

Pos.	117	obs:	exp:
cgt	R	0	0.30
cgc	R	0	0.54
cga	R	0	1.37
cgg	R	0	1.29
aat	N	2	1.68
aac	N	1	1.32
aaa	K	38004	26538.00
aag	K	2362	13828.00
aga	R	13	7.36
agg	R	2	4.15
---	--	------	------
mPD		0.11	0.45
		nPD:	0.25
		N. weight:	0.25
		Sc. PD:	0.043
		Sc. rank:	7165.6

,

PB2

Pos.	118	obs:	exp:
cct	P	1	0.33
ccc	P	0	0.19
cca	P	0	0.34
ccg	P	0	0.14
att	I	14	4.91
atc	I	0	3.68
ata	I	0	5.41
act	T	40198	12531.00
acc	T	46	7134.00
aca	T	8	17463.00
acg	T	99	3223.00
aat	N	8	4.48
aac	N	0	3.52
gct	A	10	2.73
gcc	A	0	2.50
gca	A	0	4.05
gcg	A	0	0.73
---	--	------	------
mPD		0.0092	0.68
		nPD:	0.01
		N. weight:	1.2
		Sc. PD:	-0.067
		Sc. rank:	-17792.0

,

PB2

Pos.	119	obs:	exp:
ttt	F	1	0.49
ttc	F	0	0.51
tat	Y	40164	21286.00
tac	Y	218	19096.00
tgt	C	1	0.39
tgc	C	0	0.61
---	--	------	------
mPD		0.011	0.50
		nPD:	0.02
		N. weight:	0.64
		Sc. PD:	-0.03
		Sc. rank:	-6169.1

,

PB2

Pos.	120	obs:	exp:
ttt	F	3169	19643.00
ttc	F	37213	20739.00
tta	L	0	0.12
ttg	L	1	0.21
ctt	L	0	0.21
ctc	L	0	0.15
cta	L	0	0.15
ctg	L	0	0.17
att	I	0	0.35
atc	I	1	0.26
ata	I	0	0.39
---	--	------	------
mPD		0.14	0.50
		nPD:	0.29
		N. weight:	0.42
		Sc. PD:	0.09
		Sc. rank:	12481.0

,

PB2

Pos.	121	obs:	exp:
aat	N	0	3.92
aac	N	7	3.08
gct	A	0	0.55
gcc	A	2	0.50
gca	A	0	0.81
gcg	A	0	0.15
gat	D	392	22594.00
gac	D	39578	17376.00
gaa	E	379	238.20
gag	E	25	165.80
ggt	G	0	0.13
ggc	G	1	0.14
gga	G	0	0.46
ggg	G	0	0.26
mPD		0.039	0.50
		nPD:	0.08
		N. weight:	0.81
		Sc. PD:	0.0067
		Sc. rank:	14530.0

PB2

Pos.	122	obs:	exp:
cgt	R	0	0.22
cgc	R	0	0.40
cga	R	0	1.00
cgg	R	0	0.94
aaa	K	40023	26543.00
aag	K	350	13830.00
aga	R	11	5.40
agg	R	0	3.04
mPD		0.018	0.45
		nPD:	0.04
		N. weight:	0.4
		Sc. PD:	-0.012
		Sc. rank:	1072.4

PB2

Pos.	123	obs:	exp:
ttt	F	0	6.32
ttc	F	13	6.68
att	I	1	74.77
atc	I	212	55.97
ata	I	0	82.26
gtt	V	767	10039.00
gtc	V	39225	8166.00
gta	V	85	8687.00
gtg	V	1	13187.00
gct	A	1	21.80
gcc	A	79	19.99
gca	A	0	32.36
gcg	A	0	5.85
mPD		0.057	0.76
		nPD:	0.07
		N. weight:	1.6
		Sc. PD:	0.0075
		Sc. rank:	26742.0

PB2

Pos.	124	obs:	exp:
caa	Q	1	0.61
cag	Q	0	0.39
aaa	K	12	7.89
aag	K	0	4.11
gat	D	0	1.13
gac	D	2	0.87
gaa	E	40344	23799.00
gag	E	23	16568.00
ggt	G	0	0.27
ggc	G	0	0.28
gga	G	2	0.92
ggg	G	0	0.53
mPD		0.0020	0.48
		nPD:	0.
		N. weight:	0.55
		Sc. PD:	-0.036
		Sc. rank:	-15280.0

PB2

Pos.	125	obs:	exp:
cgt	R	0	800.70
cgc	R	0	1453.00
cga	R	0	3685.00
cgg	R	15	3468.00
aga	R	268	19808.00
agg	R	40101	11170.00
mPD		0.014	0.89
		nPD:	0.02
		N. weight:	1.3
		Sc. PD:	-0.07
		Sc. rank:	-16886.0

PB2

Pos.	126	obs:	exp:
tta	L	37124	4686.00
ttg	L	2567	8349.00
ctt	L	0	8448.00
ctc	L	0	5922.00
cta	L	687	6201.00
ctg	L	6	6778.00
mPD		0.15	1.2
		nPD:	0.13
		N. weight:	1.9
		Sc. PD:	0.12
		Sc. rank:	43769.0

PB2

Pos.	127	obs:	exp:
caa	Q	7	4.28
cag	Q	0	2.72
cgt	R	0	0.52
cgc	R	0	0.94
cga	R	0	2.37
cgg	R	0	2.23
att	I	0	0.35
atc	I	0	0.26
ata	I	1	0.39
act	T	0	0.31
acc	T	0	0.18
aca	T	1	0.43
acg	T	0	0.08
aat	N	0	5.59
aac	N	10	4.41
aaa	K	40245	26520.00
aag	K	94	13819.00
aga	R	26	12.75
agg	R	0	7.19
mPD		0.0069	0.45
		nPD:	0.02
		N. weight:	0.43
		Sc. PD:	-0.023
		Sc. rank:	-5828.3

PB2

Pos.	128	obs:	exp:
tta	L	0	0.70
ttg	L	0	1.24
tat	Y	47	24.77
tac	Y	0	22.23
ctt	L	6	1.26
ctc	L	0	0.88
cta	L	0	0.92
ctg	L	0	1.01
cat	H	40263	22740.00
cac	H	54	17577.00
caa	Q	3	4.28
cag	Q	4	2.72
cgt	R	1	0.02
cgc	R	0	0.04
cga	R	0	0.09
cgg	R	0	0.09
aat	N	6	3.36
aac	N	0	2.64
aga	R	0	0.49
agg	R	0	0.28
mPD		0.0060	0.50
		nPD:	0.01
		N. weight:	0.59
		Sc. PD:	-0.034
		Sc. rank:	-9463.5

PB2

Pos.	129	obs:	exp:
ggt	G	356	5437.00
ggc	G	17	5636.00
gga	G	39813	18642.00
ggg	G	198	10669.00
mPD		0.028	0.68
		nPD:	0.04
		N. weight:	0.74
		Sc. PD:	-0.021
		Sc. rank:	2811.5

PB2

Pos.	130	obs:	exp:
att	I	0	10.88
atc	I	31	8.15
ata	I	0	11.97
act	T	668	12529.00
acc	T	39501	7133.00
aca	T	169	17460.00
acg	T	5	3222.00
aat	N	0	0.56
aac	N	1	0.44
gct	A	0	2.45
gcc	A	9	2.25
gca	A	0	3.64
gcg	A	0	0.66
mPD		0.043	0.68
		nPD:	0.06
		N. weight:	1.7
		Sc. PD:	-0.011
		Sc. rank:	22526.0

PB2

Pos.	131	obs:	exp:
ttt	F	40160	19644.00
ttc	F	224	20740.00
mPD		0.011	0.50
		nPD:	0.02
		N. weight:	0.72
		Sc. PD:	-0.034
		Sc. rank:	-6880.6

PB2

Pos.	132	obs:	exp:
gct	A	1	0.27
gcc	A	0	0.25
gca	A	0	0.40
gcg	A	0	0.07
ggt	G	7239	5437.00
ggc	G	33116	5636.00
gga	G	14	18641.00
ggg	G	14	10669.00
mPD		0.30	0.68
		nPD:	0.43
		N. weight:	1.6
		Sc. PD:	0.57
		Sc. rank:	50392.0

PB2

Pos.	133	obs:	exp:
tct	S	2	0.32
tcc	S	0	0.30
tca	S	0	0.54
tcg	S	0	0.08
cct	P	40185	13457.00
ccc	P	44	7571.00
cca	P	148	13643.00
ccg	P	5	5711.00
agt	S	0	0.35
agc	S	0	0.41
mPD		0.0098	0.72
		nPD:	0.01
		N. weight:	1.1
		Sc. PD:	-0.062
		Sc. rank:	-16315.0

PB2

Pos.	134	obs:	exp:
att	I	0	2.81
atc	I	8	2.10
ata	I	0	3.09
gtt	V	3047	10113.00
gtc	V	37310	8227.00
gta	V	19	8751.00
gtg	V	0	13285.00
mPD		0.14	0.74
		nPD:	0.19
		N. weight:	1.4
		Sc. PD:	0.16
		Sc. rank:	36546.0

PB2

Pos.	135	obs:	exp:
tat	Y	9	4.74
tac	Y	0	4.26
cat	H	40022	22748.00
cac	H	308	17582.00
caa	Q	1	0.61
cag	Q	0	0.39
cgt	R	44	0.87
cgc	R	0	1.58
cga	R	0	4.01
cgg	R	0	3.78
aga	R	0	21.58
agg	R	0	12.17
mPD		0.018	0.50
		nPD:	0.04
		N. weight:	0.56
		Sc. PD:	-0.019
		Sc. rank:	318.3

PB2

Pos.	136	obs:	exp:
ttt	F	38843	19643.00
ttc	F	1539	20739.00
tta	L	0	0.12
ttg	L	0	0.21
tat	Y	1	0.53
tac	Y	0	0.47
ctt	L	1	0.21
ctc	L	0	0.15
cta	L	0	0.15
ctg	L	0	0.17
mPD		0.073	0.50
		nPD:	0.15
		N. weight:	0.59
		Sc. PD:	0.044
		Sc. rank:	14179.0

PB2

Pos.	137	obs:	exp:
cgt	R	0	800.40
cgc	R	0	1452.00
cga	R	1	3683.00
cgg	R	0	3466.00
aaa	K	13	8.55
aag	K	0	4.45
aga	R	40284	19799.00
agg	R	82	11166.00
ggt	G	0	0.54
ggc	G	0	0.56
gga	G	4	1.85
ggg	G	0	1.06
---	--	------	------
mPD		0.0049	0.89
		nPD:	0.01
		N. weight:	0.74
		Sc. PD:	-0.047
		Sc. rank:	-18050.0

,

PB2

Pos.	138	obs:	exp:
tct	S	0	4.36
tcc	S	0	4.03
tca	S	0	7.26
tcg	S	0	1.06
cat	H	0	0.56
cac	H	1	0.44
act	T	1	0.31
acc	T	0	0.18
aca	T	0	0.43
acg	T	0	0.08
aat	N	39782	22577.00
aac	N	573	17778.00
agt	S	25	4.72
agc	S	2	5.57
---	--	------	------
mPD		0.030	0.50
		nPD:	0.06
		N. weight:	0.54
		Sc. PD:	-0.0055
		Sc. rank:	6375.2

,

PB2

Pos.	139	obs:	exp:
caa	Q	40315	24671.00
cag	Q	45	15689.00
cgt	R	0	0.38
cgc	R	0	0.68
cga	R	19	1.73
cgg	R	0	1.63
aaa	K	5	3.29
aag	K	0	1.71
aga	R	0	9.32
agg	R	0	5.26
---	--	------	------
mPD		0.0034	0.48
		nPD:	0.01
		N. weight:	0.51
		Sc. PD:	-0.032
		Sc. rank:	-11102.0

,

PB2

Pos.	140	obs:	exp:
att	I	0	8.42
atc	I	24	6.31
ata	I	0	9.27
gtt	V	620	10109.00
gtc	V	39707	8224.00
gta	V	33	8748.00
gtg	V	0	13279.00
---	--	------	------
mPD		0.033	0.74
		nPD:	0.04
		N. weight:	1.6
		Sc. PD:	-0.04
		Sc. rank:	9532.6

,

PB2

Pos.	141	obs:	exp:
cgt	R	0	0.02
cgc	R	0	0.04
cga	R	0	0.09
cgg	R	0	0.09
aaa	K	9609	26549.00
aag	K	30774	13834.00
aga	R	1	0.49
agg	R	0	0.28
---	--	------	------
mPD		0.36	0.45
		nPD:	0.81
		N. weight:	0.39
		Sc. PD:	0.28
		Sc. rank:	12823.0

,

PB2

Pos.	142	obs:	exp:
tta	L	10	1.16
ttg	L	0	2.07
ctt	L	0	2.09
ctc	L	0	1.47
cta	L	0	1.54
ctg	L	0	1.68
cgt	R	0	0.02
cgc	R	0	0.04
cga	R	0	0.09
cgg	R	0	0.09
att	I	5	14167.00
atc	I	11	10604.00
ata	I	40341	15586.00
act	T	0	3.73
acc	T	0	2.12
aca	T	12	5.19
acg	T	0	0.96
aaa	K	1	0.66
aag	K	0	0.34
aga	R	1	0.49
agg	R	0	0.28
gtt	V	0	0.75
gtc	V	0	0.61
gta	V	3	0.65
gtg	V	0	0.99
---	--	------	------
mPD		0.0021	0.66
		nPD:	0.
		N. weight:	1.
		Sc. PD:	-0.066
		Sc. rank:	-28392.0

,

PB2

Pos.	143	obs:	exp:
tta	L	0	0.12
ttg	L	0	0.21
tgt	C	0	0.39
tgc	C	1	0.61
ctt	L	0	0.21
ctc	L	1	0.15
cta	L	0	0.15
ctg	L	0	0.17
cat	H	0	0.56
cac	H	1	0.44
cgt	R	858	800.70
cgc	R	28852	1453.00
cga	R	10203	3684.00
cgg	R	110	3467.00
aga	R	19	19806.00
agg	R	339	11169.00
---	--	------	------
mPD		0.44	0.89
		nPD:	0.5
		N. weight:	2.5
		Sc. PD:	1.
		Sc. rank:	80686.0

,

PB2

Pos.	144	obs:	exp:
cgt	R	0	800.40
cgc	R	0	1452.00
cga	R	526	3683.00
cgg	R	6	3466.00
aaa	K	17	11.18
aag	K	0	5.82
aga	R	39262	19799.00
agg	R	572	11165.00
ggt	G	0	0.13
ggc	G	0	0.14
gga	G	1	0.46
ggg	G	0	0.26
---	--	------	------
mPD		0.055	0.89
		nPD:	0.06
		N. weight:	0.63
		Sc. PD:	-0.0049
		Sc. rank:	7914.9

,

PB2

Pos.	145	obs:	exp:
tct	S	0	0.16
tcc	S	0	0.15
tca	S	0	0.27
tcg	S	0	0.04
caa	Q	2	1.22
cag	Q	0	0.78
cgt	R	0	800.40
cgc	R	0	1453.00
cga	R	4931	3683.00
cgg	R	2	3466.00
aaa	K	12	7.89
aag	K	0	4.11
agt	S	1	0.17
agc	S	0	0.21
aga	R	35188	19800.00
agg	R	248	11166.00
---	--	------	------
mPD		0.23	0.89
		nPD:	0.26
		N. weight:	0.54
		Sc. PD:	0.099
		Sc. rank:	15684.0

,

PB2

Pos.	146	obs:	exp:
att	I	0	0.35
atc	I	0	0.26
ata	I	1	0.39
gtt	V	424	10115.00
gtc	V	17	8228.00
gta	V	39864	8753.00
gtg	V	78	13287.00
---	--	------	------
mPD		0.025	0.74
		nPD:	0.03
		N. weight:	1.5
		Sc. PD:	-0.054
		Sc. rank:	-506.5

,

PB2

Pos.	147	obs:	exp:
tat	Y	0	1.05
tac	Y	2	0.95
aat	N	0	0.56
aac	N	1	0.44
gat	D	1957	22824.00
gac	D	38421	17554.00
gaa	E	0	1.18
gag	E	2	0.82
ggt	G	0	0.13
ggc	G	1	0.14
gga	G	0	0.46
ggg	G	0	0.26
---	--	------	------
mPD		0.093	0.49
		nPD:	0.19
		N. weight:	0.66
		Sc. PD:	0.077
		Sc. rank:	17482.0

,

PB2

Pos.	148	obs:	exp:
tta	L	5	0.93
ttg	L	0	1.65
ctt	L	0	1.67
ctc	L	0	1.17
cta	L	3	1.23
ctg	L	0	1.34
att	I	16	13851.00
atc	I	5	10368.00
ata	I	39436	15238.00
atg	M	15	15.00
act	T	0	273.00
acc	T	0	155.40
aca	T	879	380.40
acg	T	0	70.20
gtt	V	0	6.26
gtc	V	0	5.09
gta	V	25	5.42
gtg	V	0	8.23
---	--	------	------
mPD		0.046	0.70
		nPD:	0.07
		N. weight:	1.
		Sc. PD:	-0.0046
		Sc. rank:	14121.0

,

PB2

Pos.	149	obs:	exp:
tct	S	0	0.48
tcc	S	0	0.45
tca	S	0	0.81
tcg	S	0	0.12
aat	N	8417	22591.00
aac	N	31963	17789.00
agt	S	1	0.52
agc	S	2	0.62
gat	D	0	0.57
gac	D	1	0.43
---	--	------	------
mPD		0.33	0.49
		nPD:	0.67
		N. weight:	0.27
		Sc. PD:	0.16
		Sc. rank:	8953.0

,

PB2

Pos.	150	obs:	exp:
tct	S	1	0.16
tcc	S	0	0.15
tca	S	0	0.27
tcg	S	0	0.04
cct	P	40291	13457.00
ccc	P	78	7571.00
cca	P	11	13644.00
ccg	P	3	5712.00
agt	S	0	0.17
agc	S	0	0.21
---	--	------	------
mPD		0.0046	0.72
		nPD:	0.01
		N. weight:	1.1
		Sc. PD:	-0.072
		Sc. rank:	-26728.0

,

PB2

Pos.	151	obs:	exp:
tct	S	0	0.32
tcc	S	0	0.30
tca	S	0	0.54
tcg	S	0	0.08
agt	S	2	0.35
agc	S	0	0.41
ggt	G	40010	5436.00
ggc	G	353	5636.00
gga	G	13	18641.00
ggg	G	6	10669.00
---	--	------	------
mPD		0.018	0.68
		nPD:	0.03
		N. weight:	2.1
		Sc. PD:	-0.087
		Sc. rank:	-12937.0

,

PB2

Pos.	152	obs:	exp:
tat	Y	2	1.05
tac	Y	0	0.95
cat	H	40340	22775.00
cac	H	38	17603.00
caa	Q	0	0.61
cag	Q	1	0.39
cgt	R	1	0.02
cgc	R	0	0.04
cga	R	0	0.09
cgg	R	0	0.09
aat	N	2	1.12
aac	N	0	0.88
aga	R	0	0.49
agg	R	0	0.28
---	--	------	------
mPD		0.0022	0.49
		nPD:	0.
		N. weight:	0.6
		Sc. PD:	-0.039
		Sc. rank:	-16202.0

,

PB2

Pos.	153	obs:	exp:
tct	S	0	0.81
tcc	S	0	0.75
tca	S	5	1.34
tcg	S	0	0.20
act	T	0	3.11
acc	T	0	1.77
aca	T	10	4.33
acg	T	0	0.80
agt	S	0	0.87
agc	S	0	1.03
gtt	V	0	2.76
gtc	V	0	2.24
gta	V	11	2.38
gtg	V	0	3.62
gct	A	33	10999.00
gcc	A	0	10083.00
gca	A	40213	16325.00
gcg	A	111	2950.00
gaa	E	1	0.59
gag	E	0	0.41
---	--	------	------
mPD		0.0084	0.70
		nPD:	0.01
		N. weight:	0.93
		Sc. PD:	-0.053
		Sc. rank:	-14795.0

,

PB2

Pos.	154	obs:	exp:
tat	Y	0	0.53
tac	Y	1	0.47
aat	N	0	2.80
aac	N	5	2.20
gat	D	1481	22822.00
gac	D	38893	17552.00
gaa	E	2	1.18
gag	E	0	0.82
ggt	G	1	0.27
ggc	G	1	0.28
gga	G	0	0.92
ggg	G	0	0.53
---	--	------	------
mPD		0.071	0.49
		nPD:	0.14
		N. weight:	0.7
		Sc. PD:	0.052
		Sc. rank:	16930.0

,

PB2

Pos.	155	obs:	exp:
ttt	F	0	0.97
ttc	F	2	1.03
tta	L	0	4686.00
ttg	L	0	8348.00
ctt	L	56	8447.00
ctc	L	40261	5921.00
cta	L	63	6201.00
ctg	L	0	6777.00
att	I	0	0.70
atc	I	2	0.53
ata	I	0	0.77
---	--	------	------
mPD		0.0061	1.2
		nPD:	0.01
		N. weight:	2.
		Sc. PD:	-0.13
		Sc. rank:	-51126.0

,

PB2

Pos.	156	obs:	exp:
tct	S	0	6512.00
tcc	S	0	6030.00
tca	S	0	10846.00
tcg	S	0	1588.00
tgt	C	3	1.17
tgc	C	0	1.83
cgt	R	1	0.02
cgc	R	0	0.04
cga	R	0	0.09
cgg	R	0	0.09
act	T	1	0.31
acc	T	0	0.18
aca	T	0	0.43
acg	T	0	0.08
aat	N	26	14.55
aac	N	0	11.45
agt	S	40321	7052.00
agc	S	31	8324.00
aga	R	0	0.49
agg	R	0	0.28
ggt	G	1	0.13
ggc	G	0	0.14
gga	G	0	0.46
ggg	G	0	0.26
---	--	------	------
mPD		0.0031	1.6
		nPD:	0.
		N. weight:	1.8
		Sc. PD:	-0.12
		Sc. rank:	-56018.0

,

PB2

Pos.	157	obs:	exp:
tct	S	0	0.81
tcc	S	5	0.75
tca	S	0	1.34
tcg	S	0	0.20
act	T	0	2.48
acc	T	8	1.41
aca	T	0	3.46
acg	T	0	0.64
agt	S	0	0.87
agc	S	0	1.03
gtt	V	0	7.01
gtc	V	28	5.71
gta	V	0	6.07
gtg	V	0	9.21
gct	A	1262	10996.00
gcc	A	39038	10079.00
gca	A	43	16319.00
gcg	A	0	2949.00
---	--	------	------
mPD		0.065	0.70
		nPD:	0.09
		N. weight:	1.3
		Sc. PD:	0.029
		Sc. rank:	25678.0

,

PB2

Pos.	158	obs:	exp:
caa	Q	2	1.22
cag	Q	0	0.78
cgt	R	0	1.11
cgc	R	0	2.01
cga	R	0	5.11
cgg	R	0	4.81
att	I	0	0.35
atc	I	0	0.26
ata	I	1	0.39
aat	N	0	1.12
aac	N	2	0.88
aaa	K	39303	26509.00
aag	K	1019	13813.00
aga	R	55	27.47
agg	R	1	15.49
ggt	G	0	0.13
ggc	G	0	0.14
gga	G	0	0.46
ggg	G	1	0.26
---	--	------	------
mPD		0.052	0.45
		nPD:	0.12
		N. weight:	0.34
		Sc. PD:	0.015
		Sc. rank:	7477.4

,

PB2

Pos.	159	obs:	exp:
aaa	K	0	0.66
aag	K	1	0.34
gat	D	1	0.57
gac	D	0	0.43
gaa	E	124	23807.00
gag	E	40257	16574.00
ggt	G	0	0.13
ggc	G	0	0.14
gga	G	0	0.46
ggg	G	1	0.26
---	--	------	------
mPD		0.0063	0.48
		nPD:	0.01
		N. weight:	0.92
		Sc. PD:	-0.051
		Sc. rank:	-13717.0

,

PB2

Pos.	160	obs:	exp:
gct	A	19	11007.00
gcc	A	1	10090.00
gca	A	40326	16336.00
gcg	A	38	2952.00
---	--	------	------
mPD		0.0029	0.69
		nPD:	0.
		N. weight:	0.94
		Sc. PD:	-0.061
		Sc. rank:	-25944.0

,

PB2

Pos.	161	obs:	exp:
caa	Q	39950	24683.00
cag	Q	430	15697.00
cgt	R	0	0.02
cgc	R	0	0.04
cga	R	1	0.09
cgg	R	0	0.09
aaa	K	3	1.97
aag	K	0	1.03
aga	R	0	0.49
agg	R	0	0.28
---	--	------	------
mPD		0.021	0.48
		nPD:	0.04
		N. weight:	0.46
		Sc. PD:	-0.012
		Sc. rank:	2863.4

PB2

Pos.	162	obs:	exp:
aat	N	4	2.24
aac	N	0	1.76
gat	D	40302	22821.00
gac	D	71	17552.00
gaa	E	1	3.54
gag	E	5	2.46
ggt	G	1	0.13
ggc	G	0	0.14
gga	G	0	0.46
ggg	G	0	0.26
---	--	------	------
mPD		0.0041	0.49
		nPD:	0.01
		N. weight:	0.59
		Sc. PD:	-0.036
		Sc. rank:	-11675.0

PB2

Pos.	163	obs:	exp:
att	I	0	4.56
atc	I	0	3.42
ata	I	13	5.02
gtt	V	38	10112.00
gtc	V	4	8226.00
gta	V	39883	8750.00
gtg	V	446	13283.00
---	--	------	------
mPD		0.025	0.74
		nPD:	0.03
		N. weight:	1.5
		Sc. PD:	-0.056
		Sc. rank:	-2488.7

PB2

Pos.	164	obs:	exp:
att	I	39967	14173.00
atc	I	328	10609.00
ata	I	80	15593.00
act	T	0	0.31
acc	T	1	0.18
aca	T	0	0.43
acg	T	0	0.08
gtt	V	8	2.00
gtc	V	0	1.63
gta	V	0	1.73
gtg	V	0	2.63
---	--	------	------
mPD		0.021	0.66
		nPD:	0.03
		N. weight:	1.
		Sc. PD:	-0.04
		Sc. rank:	-3862.9

PB2

Pos.	165	obs:	exp:
atg	M	40382	40382.00
aaa	K	0	0.66
aag	K	1	0.34
gtt	V	0	0.25
gtc	V	0	0.20
gta	V	0	0.22
gtg	V	1	0.33
---	--	------	------
mPD		0.000099	0.00016
		nPD:	0.6
		N. weight:	0.000057
		Sc. PD:	0.00003
		Sc. rank:	1.9

PB2

Pos.	166	obs:	exp:
aaa	K	1	0.66
aag	K	0	0.34
gtt	V	0	0.25
gtc	V	0	0.20
gta	V	1	0.22
gtg	V	0	0.33
gaa	E	40216	23808.00
gag	E	166	16574.00
---	--	------	------
mPD		0.0083	0.48
		nPD:	0.02
		N. weight:	0.53
		Sc. PD:	-0.028
		Sc. rank:	-6098.8

PB2

Pos.	167	obs:	exp:
att	I	22	7.72
atc	I	0	5.78
ata	I	0	8.50
gtt	V	40337	10110.00
gtc	V	21	8224.00
gta	V	4	8748.00
gtg	V	0	13280.00
---	--	------	------
mPD		0.0023	0.74
		nPD:	0.
		N. weight:	1.5
		Sc. PD:	-0.096
		Sc. rank:	-41569.0

PB2

Pos.	168	obs:	exp:
gtt	V	40004	10115.00
gtc	V	371	8229.00
gta	V	6	8753.00
gtg	V	3	13287.00
---	--	------	------
mPD		0.019	0.74
		nPD:	0.03
		N. weight:	1.4
		Sc. PD:	-0.062
		Sc. rank:	-10690.0

PB2

Pos.	169	obs:	exp:
ttt	F	39412	19642.00
ttc	F	969	20739.00
tat	Y	3	1.58
tac	Y	0	1.42
---	--	------	------
mPD		0.047	0.50
		nPD:	0.09
		N. weight:	0.64
		Sc. PD:	0.015
		Sc. rank:	12802.0

PB2

Pos.	170	obs:	exp:
cct	P	213	13457.00
ccc	P	39796	7571.00
cca	P	374	13644.00
ccg	P	0	5712.00
gct	A	0	0.27
gcc	A	1	0.25
gca	A	0	0.40
gcg	A	0	0.07
---	--	------	------
mPD		0.029	0.72
		nPD:	0.04
		N. weight:	1.7
		Sc. PD:	-0.049
		Sc. rank:	5607.7

PB2

Pos.	171	obs:	exp:
tct	S	0	0.32
tcc	S	0	0.30
tca	S	0	0.54
tcg	S	0	0.08
aat	N	40290	22592.00
aac	N	91	17789.00
agt	S	2	0.35
agc	S	0	0.41
gat	D	1	0.57
gac	D	0	0.43
---	--	------	------
mPD		0.0046	0.49
		nPD:	0.01
		N. weight:	0.6
		Sc. PD:	-0.036
		Sc. rank:	-11246.0

PB2

Pos.	172	obs:	exp:
gaa	E	40287	23809.00
gag	E	97	16575.00
---	--	------	------
mPD		0.0048	0.48
		nPD:	0.01
		N. weight:	0.54
		Sc. PD:	-0.032
		Sc. rank:	-9848.0

PB2

Pos.	173	obs:	exp:
atg	M	2	2.00
gtt	V	23	10115.00
gtc	V	0	8228.00
gta	V	38	8752.00
gtg	V	40321	13287.00
---	--	------	------
mPD		0.0031	0.74
		nPD:	0.
		N. weight:	1.2
		Sc. PD:	-0.075
		Sc. rank:	-31760.0

PB2

Pos.	174	obs:	exp:
ggt	G	2	5437.00
ggc	G	0	5636.00
gga	G	39826	18642.00
ggg	G	556	10669.00
---	--	------	------
mPD		0.027	0.68
		nPD:	0.04
		N. weight:	0.75
		Sc. PD:	-0.022
		Sc. rank:	2559.4

PB2

Pos.	175	obs:	exp:
act	T	0	0.31
acc	T	1	0.18
aca	T	0	0.43
acg	T	0	0.08
gtt	V	0	0.25
gtc	V	1	0.20
gta	V	0	0.22
gtg	V	0	0.33
gct	A	59	11006.00
gcc	A	39943	10089.00
gca	A	380	16335.00
gcg	A	0	2952.00
---	--	------	------
mPD		0.022	0.69
		nPD:	0.03
		N. weight:	1.4
		Sc. PD:	-0.053
		Sc. rank:	-4987.2

PB2

Pos.	176	obs:	exp:
cgt	R	0	799.70
cgc	R	0	1451.00
cga	R	28	3680.00
cgg	R	2	3463.00
aaa	K	46	32.87
aag	K	4	17.13
aga	R	37906	19783.00
agg	R	2398	11156.00
---	--	------	------
mPD		0.12	0.89
		nPD:	0.13
		N. weight:	0.54
		Sc. PD:	0.032
		Sc. rank:	12449.0

PB2			
Pos.	177	obs :	exp :
tta	L	0	0.35
ttg	L	0	0.62
ctt	L	0	0.63
ctc	L	0	0.44
cta	L	3	0.46
ctg	L	0	0.50
att	I	13	14164.00
atc	I	5	10602.00
ata	I	40331	15583.00
atg	M	3	3.00
act	T	0	3.11
acc	T	0	1.77
aca	T	10	4.33
acg	T	0	0.80
gtt	V	0	4.76
gtc	V	0	3.87
gta	V	19	4.12
gtg	V	0	6.25
---	--	------	------
mPD		0.0026	0.66
		nPD :	0.
		N. weight :	1.
		Sc. PD :	-0.065
		Sc. rank :	-27693.0

PB2			
Pos.	178	obs :	exp :
tta	L	40	4685.00
ttg	L	2	8347.00
ctt	L	8	8446.00
ctc	L	0	5920.00
cta	L	39687	6200.00
ctg	L	637	6776.00
att	I	0	3.51
atc	I	0	2.63
ata	I	10	3.86
---	--	------	------
mPD		0.034	1.2
		nPD :	0.03
		N. weight :	1.9
		Sc. PD :	-0.075
		Sc. rank :	-9590.4

PB2			
Pos.	179	obs :	exp :
tct	S	0	0.16
tcc	S	0	0.15
tca	S	1	0.27
tcg	S	0	0.04
att	I	0	0.70
atc	I	0	0.53
ata	I	2	0.77
act	T	14	12539.00
acc	T	14	7138.00
aca	T	40261	17473.00
acg	T	86	3225.00
agt	S	0	0.17
agc	S	0	0.21
gct	A	0	1.36
gcc	A	0	1.25
gca	A	5	2.02
gcg	A	0	0.37
---	-	1	1.00
---	--	------	------
mPD		0.0060	0.68
		nPD :	0.01
		N. weight :	0.86
		Sc. PD :	-0.052
		Sc. rank :	-16571.0

PB2			
Pos.	180	obs :	exp :
tta	L	1	0.12
ttg	L	0	0.21
tct	S	7	6516.00
tcc	S	22	6034.00
tca	S	40201	10853.00
tcg	S	148	1589.00
ctt	L	0	0.21
ctc	L	0	0.15
cta	L	0	0.15
ctg	L	0	0.17
agt	S	0	7056.00
agc	S	0	8330.00
gct	A	0	1.36
gcc	A	0	1.25
gca	A	5	2.02
gcg	A	0	0.37
---	--	------	------
mPD		0.0090	1.6
		nPD :	0.01
		N. weight :	1.4
		Sc. PD :	-0.086
		Sc. rank :	-33437.0

PB2			
Pos.	181	obs :	exp :
gaa	E	39976	23807.00
gag	E	404	16573.00
ggt	G	0	0.54
ggc	G	0	0.56
gga	G	4	1.85
ggg	G	0	1.06
---	--	------	------
mPD		0.020	0.48
		nPD :	0.04
		N. weight :	0.5
		Sc. PD :	-0.014
		Sc. rank :	1927.9

PB2			
Pos.	182	obs :	exp :
tct	S	1	6516.00
tcc	S	5	6034.00
tca	S	40080	10854.00
tcg	S	295	1590.00
act	T	0	0.62
acc	T	0	0.35
aca	T	2	0.87
acg	T	0	0.16
agt	S	0	7057.00
agc	S	0	8330.00
gct	A	0	0.27
gcc	A	0	0.25
gca	A	1	0.40
gcg	A	0	0.07
---	--	------	------
mPD		0.015	1.6
		nPD :	0.01
		N. weight :	1.4
		Sc. PD :	-0.081
		Sc. rank :	-25678.0

PB2			
Pos.	183	obs :	exp :
cat	H	1	0.56
cac	H	0	0.44
caa	Q	5693	24685.00
cag	Q	34690	15698.00
---	--	------	------
mPD		0.24	0.48
		nPD :	0.51
		N. weight :	0.5
		Sc. PD :	0.22
		Sc. rank :	16295.0

PB2			
Pos.	184	obs :	exp :
tta	L	2920	4684.00
ttg	L	19	8345.00
tct	S	0	0.16
tcc	S	0	0.15
tca	S	1	0.27
tcg	S	0	0.04
ctt	L	0	8444.00
ctc	L	1	5919.00
cta	L	37045	6198.00
ctg	L	380	6774.00
caa	Q	3	1.83
cag	Q	0	1.17
att	I	0	4.91
atc	I	0	3.68
ata	I	14	5.41
atg	M	1	1.00
agt	S	0	0.17
agc	S	0	0.21
---	--	------	------
mPD		0.16	1.2
		nPD :	0.13
		N. weight :	1.7
		Sc. PD :	0.1
		Sc. rank :	38989.0

PB2			
Pos.	185	obs :	exp :
tct	S	0	0.32
tcc	S	0	0.30
tca	S	2	0.54
tcg	S	0	0.08
att	I	0	2.46
atc	I	0	1.84
ata	I	7	2.70
act	T	2	12456.00
acc	T	0	7091.00
aca	T	40061	17358.00
acg	T	46	3203.00
aaa	K	2	1.31
aag	K	0	0.69
agt	S	0	0.35
agc	S	0	0.41
gct	A	0	71.95
gcc	A	0	65.96
gca	A	264	106.80
gcg	A	0	19.30
---	--	------	------
mPD		0.016	0.69
		nPD :	0.02
		N. weight :	0.88
		Sc. PD :	-0.041
		Sc. rank :	-7492.5

PB2			
Pos.	186	obs :	exp :
tta	L	0	0.23
ttg	L	0	0.41
ctt	L	0	0.42
ctc	L	0	0.29
cta	L	2	0.31
ctg	L	0	0.34
att	I	0	14171.00
atc	I	73	10607.00
ata	I	40296	15590.00
atg	M	2	2.00
act	T	0	0.31
acc	T	0	0.18
aca	T	1	0.43
acg	T	0	0.08
gtt	V	0	2.50
gtc	V	1	2.04
gta	V	9	2.17
gtg	V	0	3.29
---	--	------	------
mPD		0.0044	0.66
		nPD :	0.01
		N. weight :	0.99
		Sc. PD :	-0.062
		Sc. rank :	-22473.0

PB2			
Pos.	187	obs :	exp :
tct	S	0	0.16
tcc	S	0	0.15
tca	S	0	0.27
tcg	S	0	0.04
att	I	2	0.70
atc	I	0	0.53
ata	I	0	0.77
act	T	37074	12540.00
acc	T	2941	7139.00
aca	T	363	17476.00
acg	T	3	3225.00
agt	S	0	0.17
agc	S	1	0.21
---	--	------	------
mPD		0.15	0.68
		nPD :	0.22
		N. weight :	0.94
		Sc. PD :	0.14
		Sc. rank :	26810.0

PB2			
Pos.	188	obs :	exp :
cgt	R	0	0.42
cgc	R	0	0.76
cga	R	0	1.92
cgg	R	0	1.80
aaa	K	40305	26535.00
aag	K	57	13827.00
aga	R	21	10.30
agg	R	0	5.81
gaa	E	1	0.59
gag	E	0	0.41
---	--	------	------
mPD		0.0039	0.45
		nPD :	0.01
		N. weight :	0.43
		Sc. PD :	-0.026
		Sc. rank :	-8355.6

PB2

Pos.	189	obs:	exp:
caa	Q	0	0.61
cag	Q	1	0.39
aaa	K	0	4.60
aag	K	7	2.40
gtt	V	0	0.25
gtc	V	0	0.20
gta	V	0	0.22
gtg	V	1	0.33
gat	D	30	16.96
gac	D	0	13.04
gaa	E	242	23785.00
gag	E	40101	16558.00
ggt	G	0	0.27
ggc	G	0	0.28
gga	G	0	0.92
ggg	G	2	0.53
---	--	------	------
mPD		0.014	0.49
		nPD:	0.03
		N. weight:	0.9
		Sc. PD:	-0.037
		Sc. rank:	-4994.5

,

PB2

Pos.	190	obs:	exp:
cgt	R	0	0.02
cgc	R	0	0.04
cga	R	0	0.09
cgg	R	0	0.09
att	I	0	0.35
atc	I	0	0.26
ata	I	1	0.39
aaa	K	39715	26549.00
aag	K	667	13833.00
aga	R	1	0.49
agg	R	0	0.28
---	--	------	------
mPD		0.033	0.45
		nPD:	0.07
		N. weight:	0.36
		Sc. PD:	0.00083
		Sc. rank:	5988.5

,

PB2

Pos.	191	obs:	exp:
cgt	R	0	0.42
cgc	R	0	0.76
cga	R	0	1.92
cgg	R	0	1.80
aaa	K	39163	26535.00
aag	K	1199	13827.00
aga	R	20	10.30
agg	R	1	5.81
gaa	E	1	0.59
gag	E	0	0.41
---	--	------	------
mPD		0.059	0.45
		nPD:	0.13
		N. weight:	0.32
		Sc. PD:	0.019
		Sc. rank:	7353.3

,

PB2

Pos.	192	obs:	exp:
aaa	K	9	5.92
aag	K	0	3.08
gct	A	0	0.27
gcc	A	0	0.25
gca	A	1	0.40
gcg	A	0	0.07
gat	D	1	2.26
gac	D	3	1.74
gaa	E	40268	23797.00
gag	E	95	16566.00
ggt	G	0	0.94
ggc	G	0	0.98
gga	G	7	3.23
ggg	G	0	1.85
---	--	------	------
mPD		0.0057	0.48
		nPD:	0.01
		N. weight:	0.54
		Sc. PD:	-0.031
		Sc. rank:	-8810.7

,

PB2

Pos.	193	obs:	exp:
aaa	K	3	1.97
aag	K	0	1.03
gat	D	2	1.13
gac	D	0	0.87
gaa	E	37974	23805.00
gag	E	2404	16573.00
ggt	G	0	0.13
ggc	G	0	0.14
gga	G	1	0.46
ggg	G	0	0.26
---	--	------	------
mPD		0.11	0.48
		nPD:	0.23
		N. weight:	0.34
		Sc. PD:	0.054
		Sc. rank:	9845.0

,

PB2

Pos.	194	obs:	exp:
ttt	F	0	1.46
ttc	F	3	1.54
tta	L	4	4685.00
ttg	L	0	8347.00
ctt	L	134	8446.00
ctc	L	35389	5921.00
cta	L	4847	6200.00
ctg	L	1	6776.00
att	I	0	2.11
atc	I	4	1.58
ata	I	2	2.32
---	--	------	------
mPD		0.22	1.2
		nPD:	0.19
		N. weight:	1.6
		Sc. PD:	0.19
		Sc. rank:	42407.0

,

PB2

Pos.	195	obs:	exp:
tta	L	0	0.58
ttg	L	0	1.03
ctt	L	0	1.05
ctc	L	0	0.73
cta	L	5	0.77
ctg	L	0	0.84
cat	H	3	1.69
cac	H	0	1.31
caa	Q	139	311.10
cag	Q	370	197.90
cgt	R	1	790.50
cgc	R	1	1434.00
cga	R	39487	3637.00
cgg	R	326	3423.00
aga	R	51	19554.00
agg	R	1	11027.00
---	--	------	------
mPD		0.062	0.92
		nPD:	0.07
		N. weight:	2.4
		Sc. PD:	-0.0064
		Sc. rank:	36255.0

,

PB2

Pos.	196	obs:	exp:
tct	S	0	0.16
tcc	S	0	0.15
tca	S	0	0.27
tcg	S	0	0.04
cat	H	1	0.56
cac	H	0	0.44
aat	N	258	144.30
aac	N	0	113.70
agt	S	1	0.17
agc	S	0	0.21
gat	D	39237	22652.00
gac	D	836	17421.00
gaa	E	2	5.31
gag	E	7	3.69
ggt	G	41	5.65
ggc	G	1	5.86
gga	G	0	19.39
ggg	G	0	11.10
---	--	------	------
mPD		0.056	0.51
		nPD:	0.11
		N. weight:	0.5
		Sc. PD:	0.02
		Sc. rank:	10895.0

,

PB2

Pos.	197	obs:	exp:
ttt	F	0	0.97
ttc	F	2	1.03
tct	S	0	0.16
tcc	S	0	0.15
tca	S	0	0.27
tcg	S	0	0.04
tgt	C	975	15781.00
tgc	C	39406	24600.00
agt	S	0	0.17
agc	S	1	0.21
---	--	------	------
mPD		0.047	0.48
		nPD:	0.1
		N. weight:	0.41
		Sc. PD:	0.012
		Sc. rank:	8461.6

,

PB2

Pos.	198	obs:	exp:
caa	Q	4	2.45
cag	Q	0	1.55
cgt	R	0	1.31
cgc	R	0	2.37
cga	R	0	6.02
cgg	R	0	5.67
act	T	0	1.24
acc	T	0	0.71
aca	T	4	1.73
acg	T	0	0.32
aat	N	0	2.24
aac	N	4	1.76
aaa	K	40244	26498.00
aag	K	61	13807.00
aga	R	66	32.37
agg	R	0	18.26
gaa	E	1	0.59
gag	E	0	0.41
---	--	------	------
mPD		0.0069	0.46
		nPD:	0.02
		N. weight:	0.43
		Sc. PD:	-0.023
		Sc. rank:	-5881.5

,

PB2

Pos.	199	obs:	exp:
att	I	40193	14175.00
atc	I	78	10610.00
ata	I	108	15594.00
atg	M	1	1.00
gtt	V	4	1.00
gtc	V	0	0.82
gta	V	0	0.87
gtg	V	0	1.32
---	--	------	------
mPD		0.0094	0.66
		nPD:	0.01
		N. weight:	1.1
		Sc. PD:	-0.059
		Sc. rank:	-15079.0

,

PB2

Pos.	200	obs:	exp:
ttt	F	1	0.49
ttc	F	0	0.51
tct	S	39425	6446.00
tcc	S	497	5970.00
tca	S	27	10737.00
tcg	S	0	1573.00
tat	Y	1	0.53
tac	Y	0	0.47
act	T	1	0.31
acc	T	0	0.18
aca	T	0	0.43
acg	T	0	0.08
agt	S	0	6981.00
agc	S	0	8241.00
gct	A	432	117.70
gcc	A	0	107.90
gca	A	0	174.70
gcg	A	0	31.58
---	--	------	------
mPD		0.047	1.6
		nPD:	0.03
		N. weight:	1.8
		Sc. PD:	-0.075
		Sc. rank:	-10391.0

,

PB2

Pos.	201	obs:	exp:
tta	L	0	0.12
ttg	L	0	0.21
ctt	L	0	0.21
ctc	L	1	0.15
cta	L	0	0.15
ctg	L	0	0.17
cct	P	497	13457.00
ccc	P	39771	7571.00
cca	P	115	13644.00
ccg	P	0	5712.00
---	--	------	------
mPD		0.030	0.72
		nPD:	0.04
		N. weight:	1.7
		Sc. PD:	-0.047
		Sc. rank:	6805.4

,

PB2

Pos.	202	obs:	exp:
tta	L	3767	4686.00
ttg	L	36571	8349.00
ctt	L	1	8448.00
ctc	L	0	5922.00
cta	L	3	6201.00
ctg	L	42	6778.00
---	--	------	------
mPD		0.17	1.2
		nPD:	0.15
		N. weight:	1.4
		Sc. PD:	0.11
		Sc. rank:	33866.0

,

PB2

Pos.	203	obs:	exp:
cgt	R	0	0.02
cgc	R	0	0.04
cga	R	0	0.09
cgg	R	0	0.09
att	I	1	0.70
atc	I	0	0.53
ata	I	1	0.77
atg	M	40378	40378.00
aga	R	0	0.49
agg	R	1	0.28
gtt	V	0	0.75
gtc	V	0	0.61
gta	V	0	0.65
gtg	V	3	0.99
---	--	------	------
mPD		0.00030	0.00044
		nPD:	0.68
		N. weight:	0.00015
		Sc. PD:	0.000092
		Sc. rank:	5.1

,

PB2

Pos.	204	obs:	exp:
att	I	2	38.26
atc	I	107	28.64
ata	I	0	42.10
gtt	V	4724	10088.00
gtc	V	35037	8206.00
gta	V	160	8729.00
gtg	V	354	13251.00
---	--	------	------
mPD		0.23	0.75
		nPD:	0.31
		N. weight:	1.2
		Sc. PD:	0.29
		Sc. rank:	36335.0

,

PB2

Pos.	205	obs:	exp:
gct	A	10	11007.00
gcc	A	18	10090.00
gca	A	40087	16336.00
gcg	A	269	2952.00
---	--	------	------
mPD		0.015	0.69
		nPD:	0.02
		N. weight:	0.92
		Sc. PD:	-0.044
		Sc. rank:	-9087.0

,

PB2

Pos.	206	obs:	exp:
ttt	F	0	0.97
ttc	F	2	1.03
tat	Y	96	21284.00
tac	Y	40283	19095.00
cat	H	0	1.69
cac	H	3	1.31
---	--	------	------
mPD		0.0050	0.50
		nPD:	0.01
		N. weight:	0.77
		Sc. PD:	-0.046
		Sc. rank:	-13812.0

,

PB2

Pos.	207	obs:	exp:
atg	M	40382	40382.00
act	T	0	0.31
acc	T	0	0.18
aca	T	0	0.43
acg	T	1	0.08
aaa	K	0	0.66
aag	K	1	0.34
---	--	------	------
mPD		0.000099	0.00018
		nPD:	0.56
		N. weight:	0.000094
		Sc. PD:	0.000045
		Sc. rank:	3.1

,

PB2

Pos.	208	obs:	exp:
tta	L	23202	4686.00
ttg	L	54	8349.00
ctt	L	1	8448.00
ctc	L	3	5922.00
cta	L	17120	6201.00
ctg	L	4	6778.00
---	--	------	------
mPD		0.49	1.2
		nPD:	0.42
		N. weight:	1.4
		Sc. PD:	0.49
		Sc. rank:	44908.0

,

PB2

Pos.	209	obs:	exp:
gat	D	1	0.57
gac	D	0	0.43
gaa	E	1195	23808.00
gag	E	39188	16575.00
---	--	------	------
mPD		0.057	0.48
		nPD:	0.12
		N. weight:	0.79
		Sc. PD:	0.038
		Sc. rank:	17668.0

,

PB2

Pos.	210	obs:	exp:
cgt	R	0	800.70
cgc	R	0	1453.00
cga	R	0	3684.00
cgg	R	0	3467.00
aaa	K	3	1.97
aag	K	0	1.03
aga	R	40301	19806.00
agg	R	80	11169.00
---	--	------	------
mPD		0.0041	0.89
		nPD:	0.
		N. weight:	0.74
		Sc. PD:	-0.047
		Sc. rank:	-19625.0

,

PB2

Pos.	211	obs:	exp:
aaa	K	1	0.66
aag	K	0	0.34
gat	D	0	0.57
gac	D	1	0.43
gaa	E	40316	23808.00
gag	E	66	16574.00
---	--	------	------
mPD		0.0034	0.48
		nPD:	0.01
		N. weight:	0.54
		Sc. PD:	-0.034
		Sc. rank:	-11943.0

,

PB2

Pos.	212	obs:	exp:
ttt	F	1	0.49
ttc	F	0	0.51
tta	L	2	4686.00
ttg	L	351	8349.00
ctt	L	39948	8448.00
ctc	L	68	5922.00
cta	L	2	6201.00
ctg	L	12	6777.00
---	--	------	------
mPD		0.039	1.2
		nPD:	0.03
		N. weight:	1.6
		Sc. PD:	-0.057
		Sc. rank:	-1755.4

,

PB2

Pos.	213	obs:	exp:
atg	M	4	4.00
gtt	V	220	10114.00
gtc	V	744	8227.00
gta	V	2620	8751.00
gtg	V	36793	13285.00
gct	A	0	0.82
gcc	A	0	0.75
gca	A	1	1.21
gcg	A	2	0.22
---	--	------	------
mPD		0.17	0.74
		nPD:	0.22
		N. weight:	0.83
		Sc. PD:	0.12
		Sc. rank:	23438.0

,

PB2

Pos.	214	obs:	exp:
cgt	R	358	800.70
cgc	R	14	1453.00
cga	R	10688	3685.00
cgg	R	28956	3468.00
aga	R	16	19808.00
agg	R	352	11170.00
---	--	------	------
mPD		0.42	0.89
		nPD:	0.48
		N. weight:	1.9
		Sc. PD:	0.74
		Sc. rank:	59791.0

,

PB2

Pos.	215	obs:	exp:
cgt	R	0	0.22
cgc	R	0	0.40
cga	R	0	1.00
cgg	R	0	0.94
aaa	K	40178	26540.00
aag	K	191	13829.00
aga	R	11	5.40
agg	R	0	3.04
gaa	E	4	2.36
gag	E	0	1.64
---	--	------	------
mPD		0.010	0.45
		nPD:	0.02
		N. weight:	0.41
		Sc. PD:	-0.019
		Sc. rank:	-3629.8

,

PB2

Pos.	216	obs:	exp:
tct	S	0	0.16
tcc	S	0	0.15
tca	S	1	0.27
tcg	S	0	0.04
act	T	9	12540.00
acc	T	5	7139.00
aca	T	39925	17476.00
acg	T	442	3225.00
agt	S	0	0.17
agc	S	0	0.21
gct	A	0	0.55
gcc	A	0	0.50
gca	A	2	0.81
gcg	A	0	0.15
---	--	------	------
mPD		0.022	0.68
		nPD:	0.03
		N. weight:	0.84
		Sc. PD:	-0.03
		Sc. rank:	-1295.3

,

PB2

Pos.	217	obs:	exp:
cgt	R	0	800.50
cgc	R	0	1453.00
cga	R	4	3684.00
cgg	R	0	3467.00
aaa	K	10	6.57
aag	K	0	3.43
aga	R	38894	19802.00
agg	R	1474	11167.00
ggt	G	0	0.27
ggc	G	0	0.28
gga	G	2	0.92
ggg	G	0	0.53
---	--	------	------
mPD		0.071	0.89
		nPD:	0.08
		N. weight:	0.61
		Sc. PD:	0.0061
		Sc. rank:	10956.0

,

PB2

Pos.	218	obs:	exp:
ttt	F	40237	19644.00
ttc	F	147	20740.00
---	--	------	------
mPD		0.0073	0.50
		nPD:	0.01
		N. weight:	0.73
		Sc. PD:	-0.04
		Sc. rank:	-10302.0

,

PB2

Pos.	219	obs:	exp:
tta	L	0	4686.00
ttg	L	0	8349.00
ctt	L	200	8448.00
ctc	L	40162	5922.00
cta	L	20	6201.00
ctg	L	0	6777.00
att	I	0	0.70
atc	I	2	0.53
ata	I	0	0.77
---	--	------	------
mPD		0.011	1.2
		nPD:	0.01
		N. weight:	2.
		Sc. PD:	-0.12
		Sc. rank:	-37099.0

,

PB2

Pos.	220	obs:	exp:
cct	P	19	13457.00
ccc	P	10	7571.00
cca	P	39992	13644.00
ccg	P	363	5712.00
---	--	------	------
mPD		0.019	0.72
		nPD:	0.03
		N. weight:	1.1
		Sc. PD:	-0.047
		Sc. rank:	-7137.9

,

PB2

Pos.	221	obs:	exp:
att	I	75	27.03
atc	I	0	20.23
ata	I	2	29.74
gtt	V	39880	10096.00
gtc	V	34	8213.00
gta	V	230	8736.00
gtg	V	163	13262.00
---	--	------	------
mPD		0.025	0.74
		nPD:	0.03
		N. weight:	1.4
		Sc. PD:	-0.05
		Sc. rank:	-1373.0

,

PB2

Pos.	222	obs:	exp:
tct	S	664	107.50
tcc	S	0	99.53
tca	S	1	179.00
tcg	S	1	26.22
act	T	56	18.01
acc	T	2	10.25
aca	T	0	25.10
acg	T	0	4.63
agt	S	0	116.40
agc	S	0	137.40
gtt	V	314	78.65
gtc	V	0	63.98
gta	V	0	68.06
gtg	V	0	103.30
gct	A	39110	10724.00
gcc	A	217	9830.00
gca	A	12	15916.00
gcg	A	7	2876.00
---	--	------	------
mPD		0.063	0.76
		nPD:	0.08
		N. weight:	1.3
		Sc. PD:	0.016
		Sc. rank:	24622.0

,

PB2

Pos.	223	obs:	exp:
ggt	G	1365	5437.00
ggc	G	38362	5636.00
gga	G	651	18642.00
ggg	G	6	10669.00
---	--	------	------
mPD		0.096	0.68
		nPD:	0.14
		N. weight:	1.8
		Sc. PD:	0.13
		Sc. rank:	43527.0

,

PB2

Pos.	224	obs:	exp:
ggt	G	16	5437.00
ggc	G	2	5636.00
gga	G	40011	18642.00
ggg	G	355	10669.00
---	--	------	------
mPD		0.018	0.68
		nPD:	0.03
		N. weight:	0.76
		Sc. PD:	-0.032
		Sc. rank:	-4941.1

,

PB2

Pos.	225	obs:	exp:
att	I	0	1.05
atc	I	0	0.79
ata	I	3	1.16
act	T	3	12535.00
acc	T	1	7137.00
aca	T	40317	17469.00
acg	T	44	3224.00
gct	A	0	4.36
gcc	A	0	4.00
gca	A	16	6.47
gcg	A	0	1.17
---	--	------	------
mPD		0.0033	0.68
		nPD:	0.
		N. weight:	0.87
		Sc. PD:	-0.056
		Sc. rank:	-22675.0

,

PB2

Pos.	226	obs:	exp:
tct	S	0	6490.00
tcc	S	0	6010.00
tca	S	0	10809.00
tcg	S	0	1583.00
agt	S	83	7028.00
agc	S	40133	8296.00
ggt	G	0	22.62
ggc	G	168	23.45
gga	G	0	77.55
ggg	G	0	44.38
---	--	------	------
mPD		0.012	1.6
		nPD:	0.01
		N. weight:	1.7
		Sc. PD:	-0.1
		Sc. rank:	-34748.0

,

PB2

Pos.	227	obs:	exp:
tct	S	0	6516.00
tcc	S	0	6034.00
tca	S	0	10853.00
tcg	S	0	1590.00
tgt	C	1	0.39
tgc	C	0	0.61
agt	S	36458	7057.00
agc	S	3922	8330.00
ggt	G	3	0.40
ggc	G	0	0.42
gga	G	0	1.38
ggg	G	0	0.79
---	--	------	------
mPD		0.18	1.6
		nPD:	0.11
		N. weight:	1.5
		Sc. PD:	0.055
		Sc. rank:	32141.0

,

PB2

Pos.	228	obs:	exp:
tta	L	0	0.12
ttg	L	0	0.21
ctt	L	0	0.21
ctc	L	0	0.15
cta	L	1	0.15
ctg	L	0	0.17
att	I	13	13996.00
atc	I	20	10476.00
ata	I	39837	15398.00
atg	M	21	21.00
act	T	0	1.55
acc	T	0	0.88
aca	T	5	2.16
acg	T	0	0.40
gtt	V	353	122.00
gtc	V	2	99.23
gta	V	91	105.60
gtg	V	41	160.20
---	--	------	------
mPD		0.046	0.69
		nPD:	0.07
		N. weight:	0.99
		Sc. PD:	-0.0026
		Sc. rank:	14853.0

,

PB2

Pos.	229	obs:	exp:
tat	Y	428	21287.00
tac	Y	39956	19097.00
---	--	------	------
mPD		0.021	0.50
		nPD:	0.04
		N. weight:	0.73
		Sc. PD:	-0.02
		Sc. rank:	3329.3

,

PB2

Pos.	230	obs:	exp:
att	I	40319	14170.00
atc	I	46	10607.00
ata	I	1	15589.00
act	T	2	0.62
acc	T	0	0.35
aca	T	0	0.87
acg	T	0	0.16
gtt	V	16	4.01
gtc	V	0	3.26
gta	V	0	3.47
gtg	V	0	5.26
---	--	------	------
mPD		0.0032	0.66
		nPD:	0.
		N. weight:	1.1
		Sc. PD:	-0.07
		Sc. rank:	-28448.0

,

PB2

Pos.	231	obs:	exp:
gaa	E	40131	23809.00
gag	E	253	16575.00
---	--	------	------
mPD		0.012	0.48
		nPD:	0.03
		N. weight:	0.52
		Sc. PD:	-0.023
		Sc. rank:	-3789.7

,

PB2

Pos.	232	obs:	exp:
att	I	13	4.56
atc	I	0	3.42
ata	I	0	5.02
gtt	V	39737	10112.00
gtc	V	172	8226.00
gta	V	0	8750.00
gtg	V	461	13283.00
gct	A	1	0.27
gcc	A	0	0.25
gca	A	0	0.40
gcg	A	0	0.07
---	--	------	------
mPD		0.032	0.74
		nPD:	0.04
		N. weight:	1.4
		Sc. PD:	-0.037
		Sc. rank:	7050.9

,

PB2			
Pos.	233	obs:	exp:
tta	L	35031	4686.00
ttg	L	5243	8349.00
ctt	L	0	8448.00
ctc	L	0	5922.00
cta	L	74	6201.00
ctg	L	34	6777.00
att	I	0	0.35
atc	I	0	0.26
ata	I	1	0.39
gtt	V	0	0.25
gtc	V	0	0.20
gta	V	1	0.22
gtg	V	0	0.33
---	--	------	------
mPD		0.23	1.2
		nPD:	0.2
		N. weight:	1.8
		Sc. PD:	0.23
		Sc. rank:	48898.0

,

PB2			
Pos.	234	obs:	exp:
cat	H	39905	22778.00
cac	H	479	17606.00
---	--	------	------
mPD		0.023	0.49
		nPD:	0.05
		N. weight:	0.54
		Sc. PD:	-0.012
		Sc. rank:	4154.6

,

PB2			
Pos.	235	obs:	exp:
ttt	F	4	1.95
ttc	F	0	2.05
tta	L	325	4686.00
ttg	L	39448	8348.00
ctt	L	0	8447.00
ctc	L	0	5921.00
cta	L	0	6200.00
ctg	L	606	6777.00
atg	M	1	1.00
---	--	------	------
mPD		0.046	1.2
		nPD:	0.04
		N. weight:	1.5
		Sc. PD:	-0.046
		Sc. rank:	4558.6

,

PB2			
Pos.	236	obs:	exp:
act	T	39804	12540.00
acc	T	533	7139.00
aca	T	38	17475.00
acg	T	4	3225.00
gct	A	5	1.36
gcc	A	0	1.25
gca	A	0	2.02
gcg	A	0	0.37
---	--	------	------
mPD		0.028	0.68
		nPD:	0.04
		N. weight:	1.2
		Sc. PD:	-0.032
		Sc. rank:	4929.6

,

PB2			
Pos.	237	obs:	exp:
cat	H	2	1.13
cac	H	0	0.87
caa	Q	38082	24685.00
cag	Q	2300	15697.00
---	--	------	------
mPD		0.11	0.48
		nPD:	0.23
		N. weight:	0.32
		Sc. PD:	0.049
		Sc. rank:	8978.4

,

PB2			
Pos.	238	obs:	exp:
ggt	G	1	5437.00
ggc	G	1	5636.00
gga	G	40004	18642.00
ggg	G	378	10669.00
---	--	------	------
mPD		0.019	0.68
		nPD:	0.03
		N. weight:	0.76
		Sc. PD:	-0.032
		Sc. rank:	-4664.3

,

PB2			
Pos.	239	obs:	exp:
cct	P	0	0.33
ccc	P	0	0.19
cca	P	0	0.34
ccg	P	1	0.14
atg	M	8	8.00
act	T	36	12537.00
acc	T	1	7138.00
aca	T	4324	17472.00
acg	T	36010	3224.00
gct	A	0	1.09
gcc	A	0	1.00
gca	A	0	1.62
gcg	A	4	0.29
---	--	------	------
mPD		0.19	0.68
		nPD:	0.28
		N. weight:	2.1
		Sc. PD:	0.45
		Sc. rank:	62775.0

,

PB2			
Pos.	240	obs:	exp:
tct	S	0	0.32
tcc	S	0	0.30
tca	S	0	0.54
tcg	S	0	0.08
tgt	C	39978	15781.00
tgc	C	404	24601.00
agt	S	0	0.35
agc	S	2	0.41
---	--	------	------
mPD		0.020	0.48
		nPD:	0.04
		N. weight:	0.93
		Sc. PD:	-0.026
		Sc. rank:	4129.6

,

PB2			
Pos.	241	obs:	exp:
tct	S	0	0.32
tcc	S	0	0.30
tca	S	0	0.54
tcg	S	2	0.08
tgg	W	40382	40382.00
agt	S	0	0.35
agc	S	0	0.41
---	--	------	------
mPD		0.000099	0.00019
		nPD:	0.51
		N. weight:	0.00017
		Sc. PD:	0.000073
		Sc. rank:	5.5

,

PB2			
Pos.	242	obs:	exp:
aaa	K	1	0.66
aag	K	0	0.34
gct	A	0	0.55
gcc	A	0	0.50
gca	A	2	0.81
gcg	A	0	0.15
gaa	E	39010	23807.00
gag	E	1371	16574.00
---	--	------	------
mPD		0.066	0.48
		nPD:	0.14
		N. weight:	0.41
		Sc. PD:	0.027
		Sc. rank:	9796.8

,

PB2			
Pos.	243	obs:	exp:
caa	Q	40018	24686.00
cag	Q	366	15698.00
---	--	------	------
mPD		0.018	0.48
		nPD:	0.04
		N. weight:	0.47
		Sc. PD:	-0.015
		Sc. rank:	756.3

,

PB2			
Pos.	244	obs:	exp:
tta	L	0	3.71
ttg	L	3	6.62
ctt	L	0	6.69
ctc	L	0	4.69
cta	L	0	4.91
ctg	L	29	5.37
att	I	0	1.05
atc	I	0	0.79
ata	I	3	1.16
atg	M	40344	40344.00
act	T	0	0.31
acc	T	0	0.18
aca	T	0	0.43
acg	T	1	0.08
aaa	K	0	2.63
aag	K	4	1.37
---	--	------	------
mPD		0.0020	0.0031
		nPD:	0.63
		N. weight:	0.0015
		Sc. PD:	0.00083
		Sc. rank:	49.3

,

PB2			
Pos.	245	obs:	exp:
tat	Y	39	21287.00
tac	Y	40345	19097.00
---	--	------	------
mPD		0.0019	0.50
		nPD:	0.
		N. weight:	0.78
		Sc. PD:	-0.051
		Sc. rank:	-21764.0

,

PB2			
Pos.	246	obs:	exp:
att	I	1	0.35
atc	I	0	0.26
ata	I	0	0.39
act	T	40168	12538.00
acc	T	186	7138.00
aca	T	18	17473.00
acg	T	2	3225.00
aat	N	9	5.04
aac	N	0	3.96
---	--	------	------
mPD		0.011	0.68
		nPD:	0.02
		N. weight:	1.2
		Sc. PD:	-0.064
		Sc. rank:	-15703.0

,

PB2			
Pos.	247	obs:	exp:
cct	P	4317	13457.00
ccc	P	18	7571.00
cca	P	35930	13644.00
ccg	P	119	5712.00
---	--	------	------
mPD		0.20	0.72
		nPD:	0.27
		N. weight:	0.77
		Sc. PD:	0.15
		Sc. rank:	22493.0

,

PB2			
Pos.	248	obs:	exp:
tct	S	0	0.48
tcc	S	0	0.45
tca	S	0	0.81
tcg	S	0	0.12
cgt	R	1	0.02
cgc	R	0	0.04
cga	R	0	0.09
cgg	R	0	0.09
agt	S	3	0.52
agc	S	0	0.62
aga	R	0	0.49
agg	R	0	0.28
gaa	E	1	0.59
gag	E	0	0.41
ggt	G	39934	5436.00
ggc	G	58	5636.00
gga	G	364	18640.00
ggg	G	23	10668.00
---	--	------	------
mPD		0.022	0.68
		nPD:	0.03
		N. weight:	2.
		Sc. PD:	-0.075
		Sc. rank:	-4507.5

,

PB2			
Pos.	249	obs:	exp:
gaa	E	7	4.13
gag	E	0	2.87
ggt	G	26	5436.00
ggc	G	2	5635.00
gga	G	39936	18639.00
ggg	G	413	10667.00
---	--	------	------
mPD		0.022	0.68
		nPD:	0.03
		N. weight:	0.76
		Sc. PD:	-0.028
		Sc. rank:	-2135.9

,

PB2			
Pos.	250	obs:	exp:
tct	S	0	0.48
tcc	S	0	0.45
tca	S	0	0.81
tcg	S	0	0.12
cgt	R	0	0.24
cgc	R	0	0.43
cga	R	0	1.09
cgg	R	0	1.03
aaa	K	3	1.97
aag	K	0	1.03
agt	S	3	0.52
agc	S	0	0.62
aga	R	9	5.89
agg	R	3	3.32
gtt	V	0	0.25
gtc	V	0	0.20
gta	V	1	0.22
gtg	V	0	0.33
gat	D	16	9.04
gac	D	0	6.96
gaa	E	1119	662.70
gag	E	5	461.30
ggt	G	5212	5281.00
ggc	G	3	5475.00
gga	G	33570	18107.00
ggg	G	440	10363.00
---	--	------	------
mPD		0.30	0.73
		nPD:	0.41
		N. weight:	0.52
		Sc. PD:	0.17
		Sc. rank:	16323.0

,

PB2			
Pos.	251	obs:	exp:
tta	L	0	0.23
ttg	L	2	0.41
ctt	L	0	0.42
ctc	L	0	0.29
cta	L	0	0.31
ctg	L	0	0.34
atg	M	6	6.00
gtt	V	47	10113.00
gtc	V	0	8227.00
gta	V	537	8751.00
gtg	V	39790	13284.00
gct	A	0	0.27
gcc	A	0	0.25
gca	A	0	0.40
gcg	A	1	0.07
gaa	E	0	0.59
gag	E	1	0.41
---	--	------	------
mPD		0.029	0.74
		nPD:	0.04
		N. weight:	1.1
		Sc. PD:	-0.033
		Sc. rank:	2720.7

,

PB2			
Pos.	252	obs:	exp:
tct	S	0	0.32
tcc	S	0	0.30
tca	S	0	0.54
tcg	S	0	0.08
tgg	W	1	1.00
cgt	R	0	793.60
cgc	R	0	1440.00
cga	R	0	3652.00
cgg	R	4	3437.00
atg	M	22	22.00
aaa	K	2	208.40
aag	K	315	108.60
agt	S	2	0.35
agc	S	0	0.41
aga	R	500	19632.00
agg	R	39522	11071.00
ggt	G	0	2.15
ggc	G	0	2.23
gga	G	0	7.39
ggg	G	16	4.23
---	--	------	------
mPD		0.042	0.90
		nPD:	0.05
		N. weight:	1.3
		Sc. PD:	-0.029
		Sc. rank:	9542.9

,

PB2			
Pos.	253	obs:	exp:
tct	S	0	1.45
tcc	S	0	1.34
tca	S	0	2.42
tcg	S	0	0.35
aat	N	40338	22589.00
aac	N	37	17786.00
agt	S	9	1.57
agc	S	0	1.86
---	--	------	------
mPD		0.0023	0.49
		nPD:	0.
		N. weight:	0.61
		Sc. PD:	-0.039
		Sc. rank:	-16331.0

,

PB2			
Pos.	254	obs:	exp:
aat	N	1	1.12
aac	N	1	0.88
gat	D	579	22825.00
gac	D	39801	17555.00
gaa	E	2	1.18
gag	E	0	0.82
---	--	------	------
mPD		0.029	0.49
		nPD:	0.06
		N. weight:	0.8
		Sc. PD:	-0.0094
		Sc. rank:	9236.5

,

PB2			
Pos.	255	obs:	exp:
gat	D	40339	22828.00
gac	D	45	17556.00
---	--	------	------
mPD		0.0022	0.49
		nPD:	0.
		N. weight:	0.59
		Sc. PD:	-0.038
		Sc. rank:	-16074.0

,

PB2			
Pos.	256	obs:	exp:
ttt	F	3	1.46
ttc	F	0	1.54
tta	L	0	1.16
ttg	L	0	2.07
ctt	L	10	2.09
ctc	L	0	1.47
cta	L	0	1.54
ctg	L	0	1.68
att	I	2255	792.70
atc	I	3	593.30
ata	I	0	872.00
gtt	V	38029	9546.00
gtc	V	49	7765.00
gta	V	31	8260.00
gtg	V	1	12539.00
gct	A	3	0.82
gcc	A	0	0.75
gca	A	0	1.21
gcg	A	0	0.22
---	--	------	------
mPD		0.11	0.85
		nPD:	0.13
		N. weight:	1.4
		Sc. PD:	0.084
		Sc. rank:	32467.0

,

PB2			
Pos.	257	obs:	exp:
gat	D	362	22827.00
gac	D	40021	17556.00
gaa	E	1	0.59
gag	E	0	0.41
---	--	------	------
mPD		0.018	0.49
		nPD:	0.04
		N. weight:	0.82
		Sc. PD:	-0.027
		Sc. rank:	615.9

,

PB2			
Pos.	258	obs:	exp:
cat	H	1	0.56
cac	H	0	0.44
caa	Q	40280	24684.00
cag	Q	101	15697.00
cgt	R	0	0.04
cgc	R	0	0.07
cga	R	2	0.18
cgg	R	0	0.17
aga	R	0	0.98
agg	R	0	0.55
---	--	------	------
mPD		0.0051	0.48
		nPD:	0.01
		N. weight:	0.5
		Sc. PD:	-0.029
		Sc. rank:	-8683.0

,

PB2			
Pos.	259	obs:	exp:
tct	S	0	6516.00
tcc	S	0	6034.00
tca	S	0	10854.00
tcg	S	0	1590.00
act	T	1	0.93
acc	T	2	0.53
aca	T	0	1.30
acg	T	0	0.24
agt	S	1246	7057.00
agc	S	39135	8330.00
---	--	------	------
mPD		0.060	1.6
		nPD:	0.04
		N. weight:	1.5
		Sc. PD:	-0.05
		Sc. rank:	1519.3

,

PB2			
Pos.	260	obs:	exp:
tta	L	1236	4686.00
ttg	L	363	8349.00
ctt	L	133	8448.00
ctc	L	1	5922.00
cta	L	38469	6201.00
ctg	L	181	6777.00
att	I	0	0.35
atc	I	0	0.26
ata	I	1	0.39
---	--	------	------
mPD		0.11	1.2
		nPD:	0.09
		N. weight:	1.7
		Sc. PD:	0.041
		Sc. rank:	34887.0

,

PB2

Pos.	261	obs:	exp:
att	I	39849	14148.00
atc	I	451	10590.00
ata	I	2	15565.00
gtt	V	82	20.54
gtc	V	0	16.71
gta	V	0	17.77
gtg	V	0	26.98
---	--	------	------
mPD		0.026	0.66
		nPD:	0.04
		N. weight:	1.
		Sc. PD:	-0.031
		Sc. rank:	3313.0

,

PB2

Pos.	262	obs:	exp:
att	I	39996	14162.00
atc	I	321	10600.00
ata	I	25	15580.00
act	T	3	0.93
acc	T	0	0.53
aca	T	0	1.30
acg	T	0	0.24
gtt	V	39	9.77
gtc	V	0	7.95
gta	V	0	8.45
gtg	V	0	12.83
---	--	------	------
mPD		0.019	0.66
		nPD:	0.03
		N. weight:	1.1
		Sc. PD:	-0.043
		Sc. rank:	-5710.0

,

PB2

Pos.	263	obs:	exp:
act	T	0	0.31
acc	T	0	0.18
aca	T	0	0.43
acg	T	1	0.08
gtt	V	0	0.25
gtc	V	0	0.20
gta	V	0	0.22
gtg	V	1	0.33
gct	A	276	11006.00
gcc	A	142	10089.00
gca	A	836	16335.00
gcg	A	39128	2952.00
---	--	------	------
mPD		0.061	0.69
		nPD:	0.09
		N. weight:	2.5
		Sc. PD:	0.044
		Sc. rank:	48212.0

,

PB2

Pos.	264	obs:	exp:
tct	S	0	0.65
tcc	S	4	0.60
tca	S	0	1.08
tcg	S	0	0.16
cct	P	0	0.33
ccc	P	1	0.19
cca	P	0	0.34
ccg	P	0	0.14
agt	S	0	0.70
agc	S	0	0.83
gct	A	510	11005.00
gcc	A	39823	10088.00
gca	A	46	16334.00
gcg	A	0	2952.00
---	--	------	------
mPD		0.027	0.69
		nPD:	0.04
		N. weight:	1.4
		Sc. PD:	-0.041
		Sc. rank:	3903.9

,

PB2

Pos.	265	obs:	exp:
cgt	R	0	800.70
cgc	R	0	1453.00
cga	R	0	3685.00
cgg	R	6	3468.00
aga	R	2559	19808.00
agg	R	37819	11170.00
---	--	------	------
mPD		0.12	0.89
		nPD:	0.13
		N. weight:	1.1
		Sc. PD:	0.068
		Sc. rank:	25120.0

,

PB2

Pos.	266	obs:	exp:
tct	S	0	5.33
tcc	S	0	4.93
tca	S	0	8.87
tcg	S	0	1.30
cat	H	0	0.56
cac	H	1	0.44
aat	N	126	22575.00
aac	N	40224	17775.00
agt	S	0	5.77
agc	S	33	6.81
---	--	------	------
mPD		0.0079	0.50
		nPD:	0.02
		N. weight:	0.84
		Sc. PD:	-0.045
		Sc. rank:	-10639.0

,

PB2

Pos.	267	obs:	exp:
att	I	15	14175.00
atc	I	44	10610.00
ata	I	40321	15595.00
atg	M	1	1.00
gtt	V	0	0.75
gtc	V	0	0.61
gta	V	3	0.65
gtg	V	0	0.99
---	--	------	------
mPD		0.0031	0.66
		nPD:	0.
		N. weight:	0.99
		Sc. PD:	-0.064
		Sc. rank:	-26194.0

,

PB2

Pos.	268	obs:	exp:
tta	L	1	0.12
ttg	L	0	0.21
ctt	L	0	0.21
ctc	L	0	0.15
cta	L	0	0.15
ctg	L	0	0.17
att	I	0	2.11
atc	I	0	1.58
ata	I	6	2.32
gtt	V	5	10114.00
gtc	V	5	8227.00
gta	V	35616	8751.00
gtg	V	4751	13285.00
---	--	------	------
mPD		0.21	0.74
		nPD:	0.28
		N. weight:	1.2
		Sc. PD:	0.24
		Sc. rank:	34844.0

,

PB2

Pos.	269	obs:	exp:
cgt	R	0	800.70
cgc	R	0	1453.00
cga	R	40	3685.00
cgg	R	0	3468.00
aga	R	40341	19808.00
agg	R	3	11170.00
---	--	------	------
mPD		0.0021	0.89
		nPD:	0.
		N. weight:	0.74
		Sc. PD:	-0.05
		Sc. rank:	-21849.0

,

PB2

Pos.	270	obs:	exp:
cgt	R	0	800.70
cgc	R	0	1453.00
cga	R	9	3685.00
cgg	R	0	3468.00
aga	R	40337	19808.00
agg	R	38	11170.00
---	--	------	------
mPD		0.0023	0.89
		nPD:	0.
		N. weight:	0.74
		Sc. PD:	-0.049
		Sc. rank:	-21698.0

,

PB2

Pos.	271	obs:	exp:
tct	S	1	1.13
tcc	S	6	1.05
tca	S	0	1.88
tcg	S	0	0.28
agt	S	0	1.22
agc	S	0	1.44
gct	A	1029	11005.00
gcc	A	38733	10088.00
gca	A	614	16333.00
gcg	A	1	2952.00
---	--	------	------
mPD		0.079	0.69
		nPD:	0.11
		N. weight:	1.2
		Sc. PD:	0.054
		Sc. rank:	27607.0

,

PB2

Pos.	272	obs:	exp:
tct	S	0	5.00
tcc	S	0	4.63
tca	S	31	8.33
tcg	S	0	1.22
act	T	0	1.55
acc	T	0	0.88
aca	T	5	2.16
acg	T	0	0.40
agt	S	0	5.42
agc	S	0	6.40
gtt	V	0	0.75
gtc	V	0	0.61
gta	V	3	0.65
gtg	V	0	0.99
gct	A	22	10996.00
gcc	A	7	10080.00
gca	A	40285	16320.00
gcg	A	31	2949.00
---	--	------	------
mPD		0.0049	0.70
		nPD:	0.01
		N. weight:	0.94
		Sc. PD:	-0.058
		Sc. rank:	-20521.0

,

PB2

Pos.	273	obs:	exp:
gtt	V	14	10115.00
gtc	V	18	8229.00
gta	V	5482	8753.00
gtg	V	34870	13287.00
---	--	------	------
mPD		0.24	0.74
		nPD:	0.32
		N. weight:	0.81
		Sc. PD:	0.2
		Sc. rank:	24721.0

,

PB2

Pos.	274	obs:	exp:
tct	S	8	6516.00
tcc	S	9	6034.00
tca	S	40313	10853.00
tcg	S	50	1590.00
agt	S	0	7057.00
agc	S	0	8330.00
gct	A	0	1.09
gcc	A	0	1.00
gca	A	4	1.62
gcg	A	0	0.29
---	--	------	------
mPD		0.0035	1.6
		nPD:	0.
		N. weight:	1.4
		Sc. PD:	-0.092
		Sc. rank:	-41091.0

,

PB2

Pos.	275	obs:	exp:
tct	S	0	2.58
tcc	S	0	2.39
tca	S	16	4.30
tcg	S	0	0.63
act	T	0	9.63
acc	T	0	5.48
aca	T	31	13.42
acg	T	0	2.48
agt	S	0	2.80
agc	S	0	3.30
gct	A	25	10994.00
gcc	A	6	10078.00
gca	A	40224	16317.00
gcg	A	82	2949.00
---	--	------	------
mPD		0.0079	0.70
		nPD:	0.01
		N. weight:	0.94
		Sc. PD:	-0.054
		Sc. rank:	-15633.0

,

PB2

Pos.	276	obs:	exp:
gat	D	38695	22828.00
gac	D	1689	17556.00
---	--	------	------
mPD		0.080	0.49
		nPD:	0.16
		N. weight:	0.43
		Sc. PD:	0.039
		Sc. rank:	10706.0

,

PB2			
Pos.	277	obs:	exp:
tta	L	0	0.12
ttg	L	0	0.21
ctt	L	0	0.21
ctc	L	0	0.15
cta	L	1	0.15
ctg	L	0	0.17
cct	P	2	13457.00
ccc	P	2	7571.00
cca	P	40329	13644.00
ccg	P	50	5712.00
---	--	------	------
mPD		0.0027	0.72
		nPD:	0.
		N. weight:	1.1
		Sc. PD:	-0.074
		Sc. rank:	-31666.0

,

PB2			
Pos.	278	obs:	exp:
tta	L	707	4686.00
ttg	L	5	8349.00
ctt	L	1415	8448.00
ctc	L	8287	5922.00
cta	L	29901	6201.00
ctg	L	67	6777.00
att	I	0	0.70
atc	I	0	0.53
ata	I	2	0.77
---	--	------	------
mPD		0.42	1.2
		nPD:	0.36
		N. weight:	1.2
		Sc. PD:	0.34
		Sc. rank:	37342.0

,

PB2			
Pos.	279	obs:	exp:
tct	S	0	0.81
tcc	S	0	0.75
tca	S	5	1.34
tcg	S	0	0.20
agt	S	0	0.87
agc	S	0	1.03
gtt	V	0	1.00
gtc	V	0	0.82
gta	V	4	0.87
gtg	V	0	1.32
gct	A	19	11004.00
gcc	A	4	10087.00
gca	A	40123	16332.00
gcg	A	229	2951.00
---	--	------	------
mPD		0.013	0.69
		nPD:	0.02
		N. weight:	0.92
		Sc. PD:	-0.047
		Sc. rank:	-10229.0

,

PB2			
Pos.	280	obs:	exp:
ttt	F	2	0.97
ttc	F	0	1.03
tct	S	40026	6516.00
tcc	S	37	6034.00
tca	S	13	10853.00
tcg	S	301	1589.00
agt	S	0	7056.00
agc	S	0	8330.00
gct	A	5	1.36
gcc	A	0	1.25
gca	A	0	2.02
gcg	A	0	0.37
---	--	------	------
mPD		0.018	1.6
		nPD:	0.01
		N. weight:	1.9
		Sc. PD:	-0.11
		Sc. rank:	-32791.0

,

PB2			
Pos.	281	obs:	exp:
tta	L	39916	4686.00
ttg	L	61	8349.00
ctt	L	0	8448.00
ctc	L	355	5922.00
cta	L	52	6201.00
ctg	L	0	6778.00
---	--	------	------
mPD		0.040	1.2
		nPD:	0.03
		N. weight:	2.2
		Sc. PD:	-0.076
		Sc. rank:	-62.8

,

PB2			
Pos.	282	obs:	exp:
tta	L	109	4686.00
ttg	L	39727	8349.00
tgg	W	1	1.00
ctt	L	0	8448.00
ctc	L	0	5922.00
cta	L	3	6201.00
ctg	L	544	6777.00
---	--	------	------
mPD		0.032	1.2
		nPD:	0.03
		N. weight:	1.6
		Sc. PD:	-0.065
		Sc. rank:	-9190.3

,

PB2			
Pos.	283	obs:	exp:
gat	D	6	3.39
gac	D	0	2.61
gaa	E	1328	23804.00
gag	E	39048	16572.00
ggt	G	0	0.27
ggc	G	0	0.28
gga	G	1	0.92
ggg	G	1	0.53
---	--	------	------
mPD		0.064	0.48
		nPD:	0.13
		N. weight:	0.77
		Sc. PD:	0.047
		Sc. rank:	18007.0

,

PB2			
Pos.	284	obs:	exp:
tta	L	0	0.23
ttg	L	1	0.41
ctt	L	0	0.42
ctc	L	0	0.29
cta	L	0	0.31
ctg	L	1	0.34
att	I	1	16.15
atc	I	0	12.09
ata	I	45	17.77
atg	M	40332	40332.00
act	T	0	0.31
acc	T	0	0.18
aca	T	0	0.43
acg	T	1	0.08
gtt	V	0	0.75
gtc	V	0	0.61
gta	V	0	0.65
gtg	V	3	0.99
---	--	------	------
mPD		0.0026	0.0028
		nPD:	0.93
		N. weight:	0.0013
		Sc. PD:	0.0011
		Sc. rank:	42.0

,

PB2			
Pos.	285	obs:	exp:
tgt	C	696	15782.00
tgc	C	39688	24602.00
---	--	------	------
mPD		0.034	0.48
		nPD:	0.07
		N. weight:	0.44
		Sc. PD:	0.00049
		Sc. rank:	7153.7

,

PB2			
Pos.	286	obs:	exp:
tat	Y	0	1.58
tac	Y	3	1.42
cat	H	75	22772.00
cac	H	40298	17601.00
caa	Q	4	2.45
cag	Q	0	1.55
aat	N	0	2.24
aac	N	4	1.76
---	--	------	------
mPD		0.0043	0.49
		nPD:	0.01
		N. weight:	0.86
		Sc. PD:	-0.052
		Sc. rank:	-16671.0

,

PB2			
Pos.	287	obs:	exp:
tct	S	0	6515.00
tcc	S	0	6033.00
tca	S	0	10852.00
tcg	S	0	1589.00
tgt	C	0	0.39
tgc	C	1	0.61
aat	N	0	4.48
aac	N	8	3.52
agt	S	62	7055.00
agc	S	40312	8329.00
ggt	G	0	0.13
ggc	G	1	0.14
gga	G	0	0.46
ggg	G	0	0.26
---	--	------	------
mPD		0.0036	1.6
		nPD:	0.
		N. weight:	1.7
		Sc. PD:	-0.11
		Sc. rank:	-49180.0

,

PB2			
Pos.	288	obs:	exp:
act	T	180	12541.00
acc	T	36	7140.00
aca	T	2688	17477.00
acg	T	37480	3225.00
---	--	------	------
mPD		0.13	0.68
		nPD:	0.2
		N. weight:	2.2
		Sc. PD:	0.28
		Sc. rank:	61400.0

,

PB2

Pos.	289	obs:	exp:
tta	L	0	14.85
ttg	L	0	26.46
ctt	L	0	26.78
ctc	L	0	18.77
cta	L	128	19.66
ctg	L	0	21.48
cat	H	0	0.56
cac	H	1	0.44
caa	Q	39897	24590.00
cag	Q	331	15638.00
cgt	R	0	0.06
cgc	R	0	0.11
cga	R	3	0.27
cgg	R	0	0.26
aaa	K	23	15.12
aag	K	0	7.88
aga	R	0	1.47
agg	R	0	0.83
gaa	E	1	0.59
gag	E	0	0.41
---	--	------	------
mPD		0.024	0.49
		nPD:	0.05
		N. weight:	0.48
		Sc. PD:	-0.0098
		Sc. rank:	4007.0

,

PB2

Pos.	290	obs:	exp:
att	I	40315	14175.00
atc	I	60	10610.00
ata	I	4	15594.00
gtt	V	5	1.25
gtc	V	0	1.02
gta	V	0	1.08
gtg	V	0	1.65
---	--	------	------
mPD		0.0034	0.66
		nPD:	0.01
		N. weight:	1.1
		Sc. PD:	-0.07
		Sc. rank:	-28089.0

,

PB2

Pos.	291	obs:	exp:
tct	S	0	2.10
tcc	S	0	1.94
tca	S	0	3.49
tcg	S	0	0.51
agt	S	5	2.27
agc	S	8	2.68
ggt	G	3682	5435.00
ggc	G	36184	5634.00
gga	G	502	18636.00
ggg	G	3	10666.00
---	--	------	------
mPD		0.19	0.68
		nPD:	0.28
		N. weight:	1.7
		Sc. PD:	0.34
		Sc. rank:	49368.0

,

PB2

Pos.	292	obs:	exp:
cgt	R	0	0.48
cgc	R	0	0.86
cga	R	0	2.19
cgg	R	0	2.06
aga	R	18	11.77
agg	R	6	6.64
gaa	E	2	1.18
gag	E	0	0.82
ggt	G	203	5433.00
ggc	G	0	5633.00
gga	G	37965	18630.00
ggg	G	2190	10662.00
---	--	------	------
mPD		0.11	0.68
		nPD:	0.17
		N. weight:	0.6
		Sc. PD:	0.057
		Sc. rank:	15183.0

,

PB2

Pos.	293	obs:	exp:
cgt	R	0	0.02
cgc	R	0	0.04
cga	R	0	0.09
cgg	R	0	0.09
att	I	5	60.03
atc	I	0	44.93
ata	I	166	66.04
atg	M	3	3.00
act	T	8	12399.00
acc	T	4	7059.00
aca	T	39774	17279.00
acg	T	140	3189.00
aaa	K	4	2.63
aag	K	0	1.37
aga	R	1	0.49
agg	R	0	0.28
gtt	V	0	51.85
gtc	V	0	42.18
gta	V	15	44.87
gtg	V	192	68.11
gct	A	0	19.62
gcc	A	0	17.99
gca	A	72	29.12
gcg	A	0	5.26
---	--	------	------
mPD		0.050	0.71
		nPD:	0.07
		N. weight:	0.86
		Sc. PD:	0.000093
		Sc. rank:	13681.0

,

PB2

Pos.	294	obs:	exp:
tta	L	0	0.12
ttg	L	0	0.21
tct	S	0	0.16
tcc	S	0	0.15
tca	S	0	0.27
tcg	S	0	0.04
tgg	W	7	7.00
ctt	L	0	0.21
ctc	L	0	0.15
cta	L	0	0.15
ctg	L	1	0.17
cgt	R	0	779.90
cgc	R	0	1415.00
cga	R	1	3589.00
cgg	R	128	3378.00
atg	M	68	68.00
aaa	K	62	623.20
aag	K	886	324.80
agt	S	1	0.17
agc	S	0	0.21
aga	R	593	19293.00
agg	R	38613	10880.00
ggt	G	0	3.23
ggc	G	0	3.35
gga	G	0	11.08
ggg	G	24	6.34
---	--	------	------
mPD		0.089	0.93
		nPD:	0.1
		N. weight:	1.2
		Sc. PD:	0.032
		Sc. rank:	25002.0

,

PB2

Pos.	295	obs:	exp:
tta	L	0	5.80
ttg	L	50	10.34
ctt	L	0	10.46
ctc	L	0	7.33
cta	L	0	7.68
ctg	L	0	8.39
att	I	0	1.76
atc	I	0	1.31
ata	I	5	1.93
atg	M	40329	40329.00
---	--	------	------
mPD		0.0027	0.0043
		nPD:	0.64
		N. weight:	0.0022
		Sc. PD:	0.0012
		Sc. rank:	71.7

,

PB2

Pos.	296	obs:	exp:
tta	L	0	0.12
ttg	L	1	0.21
ctt	L	0	0.21
ctc	L	0	0.15
cta	L	0	0.15
ctg	L	0	0.17
att	I	0	29.14
atc	I	0	21.81
ata	I	83	32.05
atg	M	57	57.00
act	T	0	0.31
acc	T	0	0.18
aca	T	1	0.43
acg	T	0	0.08
gtt	V	24	10079.00
gtc	V	0	8199.00
gta	V	2778	8721.00
gtg	V	37437	13240.00
gct	A	0	0.82
gcc	A	0	0.75
gca	A	0	1.21
gcg	A	3	0.22
---	--	------	------
mPD		0.14	0.75
		nPD:	0.19
		N. weight:	0.93
		Sc. PD:	0.11
		Sc. rank:	24430.0

,

PB2

Pos.	297	obs:	exp:
aat	N	0	3.92
aac	N	7	3.08
gtt	V	0	0.25
gtc	V	1	0.20
gta	V	0	0.22
gtg	V	0	0.33
gat	D	51	22820.00
gac	D	40320	17551.00
gaa	E	4	2.36
gag	E	0	1.64
ggt	G	0	0.13
ggc	G	1	0.14
gga	G	0	0.46
ggg	G	0	0.26
---	--	------	------
mPD		0.0032	0.49
		nPD:	0.01
		N. weight:	0.87
		Sc. PD:	-0.054
		Sc. rank:	-20248.0

,

PB2

Pos.	298	obs:	exp:
ttt	F	1	0.49
ttc	F	0	0.51
att	I	39812	14170.00
atc	I	550	10606.00
ata	I	3	15589.00
act	T	0	0.31
acc	T	1	0.18
aca	T	0	0.43
acg	T	0	0.08
gtt	V	17	4.26
gtc	V	0	3.46
gta	V	0	3.68
gtg	V	0	5.59
---	--	------	------
mPD		0.028	0.66
		nPD:	0.04
		N. weight:	1.
		Sc. PD:	-0.028
		Sc. rank:	5140.4

,

PB2

Pos.	299	obs:	exp:
tta	L	0	4683.00
ttg	L	0	8343.00
ctt	L	40231	8442.00
ctc	L	115	5918.00
cta	L	5	6197.00
ctg	L	3	6773.00
att	I	30	10.53
atc	I	0	7.88
ata	I	0	11.59
---	--	------	------
mPD		0.0076	1.2
		nPD:	0.01
		N. weight:	1.6
		Sc. PD:	-0.1
		Sc. rank:	-37749.0

,

PB2

Pos.	300	obs:	exp:
cgt	R	0	532.70
cgc	R	0	966.70
cga	R	2	2451.00
cgg	R	0	2307.00
att	I	0	2.81
atc	I	0	2.10
ata	I	8	3.09
atg	M	1	1.00
act	T	0	0.31
acc	T	0	0.18
aca	T	1	0.43
acg	T	0	0.08
aat	N	0	24.06
aac	N	43	18.94
aaa	K	13437	8846.00
aag	K	19	4610.00
aga	R	25904	13178.00
agg	R	962	7432.00
gaa	E	1	0.59
gag	E	0	0.41
ggt	G	0	0.81
ggc	G	0	0.84
gga	G	4	2.77
ggg	G	2	1.59
---	--	------	------
mPD		0.50	1.2
		nPD:	0.41
		N. weight:	0.55
		Sc. PD:	0.18
		Sc. rank:	17328.0

,

PB2			
Pos.	301	obs:	exp:
cat	H	10	5.64
cac	H	0	4.36
caa	Q	687	24679.00
cag	Q	39686	15694.00
aaa	K	0	0.66
aag	K	1	0.34
---	--	------	------
mPD		0.034	0.48
		nPD:	0.07
		N. weight:	0.9
		Sc. PD:	0.0013
		Sc. rank:	14695.0

PB2			
Pos.	302	obs:	exp:
tct	S	0	0.81
tcc	S	0	0.75
tca	S	0	1.34
tcg	S	0	0.20
act	T	0	1.55
acc	T	5	0.88
aca	T	0	2.16
acg	T	0	0.40
aat	N	342	22587.00
aac	N	40031	17786.00
agt	S	0	0.87
agc	S	5	1.03
gat	D	0	0.57
gac	D	1	0.43
---	--	------	------
mPD		0.017	0.49
		nPD:	0.04
		N. weight:	0.81
		Sc. PD:	-0.028
		Sc. rank:	331.7

PB2			
Pos.	303	obs:	exp:
tta	L	0	0.81
ttg	L	0	1.45
tct	S	0	1.61
tcc	S	0	1.49
tca	S	1	2.69
tcg	S	9	0.39
ctt	L	0	1.46
ctc	L	0	1.03
cta	L	0	1.07
ctg	L	7	1.17
cct	P	219	13450.00
ccc	P	1	7567.00
cca	P	1456	13636.00
ccg	P	38685	5708.00
caa	Q	0	3.67
cag	Q	6	2.33
agt	S	0	1.75
agc	S	0	2.06
---	--	------	------
mPD		0.081	0.72
		nPD:	0.11
		N. weight:	1.8
		Sc. PD:	0.076
		Sc. rank:	40073.0

PB2			
Pos.	304	obs:	exp:
cct	P	1	0.33
ccc	P	0	0.19
cca	P	0	0.34
ccg	P	0	0.14
att	I	21	7.37
atc	I	0	5.52
ata	I	0	8.11
act	T	39645	12531.00
acc	T	79	7134.00
aca	T	209	17463.00
acg	T	417	3223.00
gct	A	12	3.27
gcc	A	0	3.00
gca	A	0	4.85
gcg	A	0	0.88
---	--	------	------
mPD		0.036	0.68
		nPD:	0.05
		N. weight:	1.1
		Sc. PD:	-0.019
		Sc. rank:	10901.0

PB2			
Pos.	305	obs:	exp:
gat	D	16	10.74
gac	D	3	8.26
gaa	E	39992	23798.00
gag	E	373	16567.00
---	--	------	------
mPD		0.019	0.48
		nPD:	0.04
		N. weight:	0.51
		Sc. PD:	-0.015
		Sc. rank:	1627.4

PB2			
Pos.	306	obs:	exp:
gat	D	0	1.13
gac	D	2	0.87
gaa	E	40101	23808.00
gag	E	281	16574.00
---	--	------	------
mPD		0.014	0.48
		nPD:	0.03
		N. weight:	0.52
		Sc. PD:	-0.021
		Sc. rank:	-2847.6

PB2			
Pos.	307	obs:	exp:
caa	Q	33281	24686.00
cag	Q	7103	15698.00
---	--	------	------
mPD		0.29	0.48
		nPD:	0.61
		N. weight:	0.11
		Sc. PD:	0.06
		Sc. rank:	3693.5

PB2			
Pos.	308	obs:	exp:
tct	S	1	0.16
tcc	S	0	0.15
tca	S	0	0.27
tcg	S	0	0.04
agt	S	0	0.17
agc	S	0	0.21
gct	A	39875	11006.00
gcc	A	395	10089.00
gca	A	94	16335.00
gcg	A	18	2952.00
ggt	G	0	0.13
ggc	G	1	0.14
gga	G	0	0.46
ggg	G	0	0.26
---	--	------	------
mPD		0.025	0.69
		nPD:	0.04
		N. weight:	1.3
		Sc. PD:	-0.043
		Sc. rank:	876.0

PB2			
Pos.	309	obs:	exp:
caa	Q	0	0.61
cag	Q	1	0.39
att	I	1	1.40
atc	I	0	1.05
ata	I	3	1.54
gtt	V	1171	10114.00
gtc	V	0	8228.00
gta	V	912	8752.00
gtg	V	38296	13286.00
---	--	------	------
mPD		0.10	0.74
		nPD:	0.13
		N. weight:	0.94
		Sc. PD:	0.06
		Sc. rank:	22192.0

PB2			
Pos.	310	obs:	exp:
tat	Y	1	0.53
tac	Y	0	0.47
aat	N	6	3.36
aac	N	0	2.64
gct	A	1	0.27
gcc	A	0	0.25
gca	A	0	0.40
gcg	A	0	0.07
gat	D	39837	22820.00
gac	D	534	17551.00
ggt	G	5	0.67
ggc	G	0	0.70
gga	G	0	2.31
ggg	G	0	1.32
---	--	------	------
mPD		0.027	0.49
		nPD:	0.05
		N. weight:	0.53
		Sc. PD:	-0.0082
		Sc. rank:	5424.1

PB2			
Pos.	311	obs:	exp:
caa	Q	1	0.61
cag	Q	0	0.39
att	I	6	14176.00
atc	I	9	10611.00
ata	I	40367	15595.00
atg	M	1	1.00
---	--	------	------
mPD		0.00089	0.66
		nPD:	0.
		N. weight:	1.
		Sc. PD:	-0.068
		Sc. rank:	-31100.0

PB2			
Pos.	312	obs:	exp:
tgt	C	46	15781.00
tgc	C	40337	24602.00
att	I	0	0.35
atc	I	0	0.26
ata	I	1	0.39
---	--	------	------
mPD		0.0024	0.48
		nPD:	0.01
		N. weight:	0.51
		Sc. PD:	-0.033
		Sc. rank:	-13271.0

PB2			
Pos.	313	obs:	exp:
tat	Y	1	0.53
tac	Y	0	0.47
cgt	R	0	0.20
cgc	R	0	0.36
cga	R	0	0.91
cgg	R	0	0.86
aaa	K	16291	26543.00
aag	K	24082	13830.00
aga	R	2	4.90
agg	R	8	2.77
---	--	------	------
mPD		0.48	0.45
		nPD:	1.07
		N. weight:	0.14
		Sc. PD:	0.14
		Sc. rank:	4682.5

PB2			
Pos.	314	obs:	exp:
gct	A	39713	11007.00
gcc	A	304	10090.00
gca	A	360	16336.00
gcg	A	7	2952.00
---	--	------	------
mPD		0.033	0.69
		nPD:	0.05
		N. weight:	1.3
		Sc. PD:	-0.028
		Sc. rank:	9623.2

PB2			
Pos.	315	obs:	exp:
tct	S	0	0.97
tcc	S	0	0.90
tca	S	6	1.61
tcg	S	0	0.24
agt	S	0	1.05
agc	S	0	1.24
gct	A	5	11005.00
gcc	A	655	10088.00
gca	A	39646	16333.00
gcg	A	72	2952.00
---	--	------	------
mPD		0.036	0.69
		nPD:	0.05
		N. weight:	0.86
		Sc. PD:	-0.016
		Sc. rank:	7950.0

PB2			
Pos.	316	obs:	exp:
tta	L	0	0.70
ttg	L	5	1.24
ctt	L	0	1.26
ctc	L	0	0.88
cta	L	0	0.92
ctg	L	1	1.01
att	I	0	80.04
atc	I	0	59.91
ata	I	228	88.05
atg	M	40149	40149.00
gtt	V	0	0.25
gtc	V	0	0.20
gta	V	0	0.22
gtg	V	1	0.33
---	--	------	------
mPD		0.012	0.012
		nPD:	0.98
		N. weight:	0.0059
		Sc. PD:	0.0053
		Sc. rank:	195.2

PB2			
Pos.	317	obs:	exp:
cgt	R	0	0.02
cgc	R	0	0.04
cga	R	1	0.09
cgg	R	0	0.09
aga	R	0	0.49
agg	R	0	0.28
ggt	G	6	5437.00
ggc	G	5	5636.00
gga	G	39559	18641.00
ggg	G	813	10669.00
---	--	------	------
mPD		0.040	0.68
		nPD:	0.06
		N. weight:	0.72
		Sc. PD:	-0.0079
		Sc. rank:	8503.8

,

PB2			
Pos.	318	obs:	exp:
ttt	F	1	0.49
ttc	F	0	0.51
tta	L	130	4686.00
ttg	L	39854	8348.00
ctt	L	0	8447.00
ctc	L	0	5921.00
cta	L	0	6201.00
ctg	L	396	6777.00
atg	M	3	3.00
---	--	------	------
mPD		0.026	1.2
		nPD:	0.02
		N. weight:	1.6
		Sc. PD:	-0.074
		Sc. rank:	-14196.0

,

PB2			
Pos.	319	obs:	exp:
cgt	R	0	800.30
cgc	R	0	1452.00
cga	R	0	3683.00
cgg	R	0	3466.00
aaa	K	19	12.49
aag	K	0	6.51
aga	R	37854	19798.00
agg	R	2510	11165.00
ggt	G	0	0.13
ggc	G	0	0.14
gga	G	1	0.46
ggg	G	0	0.26
---	--	------	------
mPD		0.12	0.89
		nPD:	0.13
		N. weight:	0.54
		Sc. PD:	0.033
		Sc. rank:	12662.0

,

PB2			
Pos.	320	obs:	exp:
att	I	429	14159.00
atc	I	39757	10598.00
ata	I	147	15576.00
act	T	0	0.31
acc	T	1	0.18
aca	T	0	0.43
acg	T	0	0.08
gtt	V	1	12.52
gtc	V	49	10.19
gta	V	0	10.84
gtg	V	0	16.45
---	--	------	------
mPD		0.031	0.66
		nPD:	0.05
		N. weight:	1.3
		Sc. PD:	-0.03
		Sc. rank:	9413.0

,

PB2			
Pos.	321	obs:	exp:
tct	S	0	6515.00
tcc	S	0	6034.00
tca	S	0	10852.00
tcg	S	0	1589.00
tgt	C	0	0.39
tgc	C	1	0.61
aat	N	0	3.92
aac	N	7	3.08
agt	S	82	7056.00
agc	S	40293	8329.00
ggt	G	0	0.13
ggc	G	1	0.14
gga	G	0	0.46
ggg	G	0	0.26
---	--	------	------
mPD		0.0045	1.6
		nPD:	0.
		N. weight:	1.6
		Sc. PD:	-0.11
		Sc. rank:	-47873.0

,

PB2			
Pos.	322	obs:	exp:
tta	L	2	0.23
ttg	L	0	0.41
tct	S	8	6515.00
tcc	S	152	6033.00
tca	S	40055	10851.00
tcg	S	157	1589.00
ctt	L	0	0.42
ctc	L	0	0.29
cta	L	0	0.31
ctg	L	0	0.34
cct	P	0	3.33
ccc	P	1	1.87
cca	P	9	3.38
ccg	P	0	1.41
agt	S	0	7055.00
agc	S	0	8329.00
---	--	------	------
mPD		0.016	1.6
		nPD:	0.01
		N. weight:	1.3
		Sc. PD:	-0.079
		Sc. rank:	-24087.0

,

PB2			
Pos.	323	obs:	exp:
tct	S	17048	6517.00
tcc	S	22711	6035.00
tca	S	623	10854.00
tcg	S	2	1590.00
agt	S	0	7057.00
agc	S	0	8331.00
---	--	------	------
mPD		0.51	1.6
		nPD:	0.31
		N. weight:	1.2
		Sc. PD:	0.28
		Sc. rank:	35585.0

,

PB2			
Pos.	324	obs:	exp:
ttt	F	164	19642.00
ttc	F	40216	20738.00
tta	L	1	0.35
ttg	L	0	0.62
tat	Y	0	0.53
tac	Y	1	0.47
ctt	L	0	0.63
ctc	L	2	0.44
cta	L	0	0.46
ctg	L	0	0.50
---	--	------	------
mPD		0.0083	0.50
		nPD:	0.02
		N. weight:	0.68
		Sc. PD:	-0.036
		Sc. rank:	-8163.5

,

PB2			
Pos.	325	obs:	exp:
tct	S	0	6516.00
tcc	S	0	6034.00
tca	S	0	10853.00
tcg	S	0	1590.00
tgt	C	0	0.39
tgc	C	1	0.61
cgt	R	0	0.04
cgc	R	2	0.07
cga	R	0	0.18
cgg	R	0	0.17
att	I	0	0.35
atc	I	1	0.26
ata	I	0	0.39
agt	S	466	7057.00
agc	S	39914	8330.00
aga	R	0	0.98
agg	R	0	0.55
---	--	------	------
mPD		0.023	1.6
		nPD:	0.01
		N. weight:	1.6
		Sc. PD:	-0.088
		Sc. rank:	-22817.0

,

PB2			
Pos.	326	obs:	exp:
ttt	F	40294	19643.00
ttc	F	89	20740.00
tat	Y	1	0.53
tac	Y	0	0.47
---	--	------	------
mPD		0.0044	0.50
		nPD:	0.01
		N. weight:	0.74
		Sc. PD:	-0.045
		Sc. rank:	-14192.0

,

PB2			
Pos.	327	obs:	exp:
tct	S	0	0.16
tcc	S	0	0.15
tca	S	0	0.27
tcg	S	0	0.04
agt	S	1	0.17
agc	S	0	0.21
ggt	G	40299	5437.00
ggc	G	67	5636.00
gga	G	10	18641.00
ggg	G	7	10669.00
---	--	------	------
mPD		0.0042	0.68
		nPD:	0.01
		N. weight:	2.1
		Sc. PD:	-0.13
		Sc. rank:	-50150.0

,

PB2			
Pos.	328	obs:	exp:
tct	S	0	5.81
tcc	S	0	5.38
tca	S	0	9.68
tcg	S	0	1.42
tgt	C	0	0.78
tgc	C	2	1.22
agt	S	0	6.29
agc	S	36	7.43
gtt	V	0	0.50
gtc	V	1	0.41
gta	V	1	0.43
gtg	V	0	0.66
gat	D	0	0.57
gac	D	1	0.43
gaa	E	1	0.59
gag	E	0	0.41
ggt	G	115	5431.00
ggc	G	37524	5630.00
gga	G	1058	18623.00
ggg	G	1645	10658.00
---	--	------	------
mPD		0.13	0.68
		nPD:	0.2
		N. weight:	1.7
		Sc. PD:	0.21
		Sc. rank:	45887.0

,

PB2			
Pos.	329	obs:	exp:
ttt	F	29988	19641.00
ttc	F	10390	20737.00
tta	L	0	0.23
ttg	L	0	0.41
tat	Y	0	2.11
tac	Y	4	1.89
ctt	L	2	0.42
ctc	L	0	0.29
cta	L	0	0.31
ctg	L	0	0.34
---	--	------	------
mPD		0.38	0.50
		nPD:	0.77
		N. weight:	0.14
		Sc. PD:	0.099
		Sc. rank:	4763.5

PB2			
Pos.	330	obs:	exp:
tct	S	0	0.16
tcc	S	0	0.15
tca	S	1	0.27
tcg	S	0	0.04
att	I	0	1.05
atc	I	0	0.79
ata	I	3	1.16
act	T	323	12539.00
acc	T	223	7139.00
aca	T	39771	17474.00
acg	T	59	3225.00
agt	S	0	0.17
agc	S	0	0.21
gct	A	0	1.09
gcc	A	0	1.00
gca	A	4	1.62
gcg	A	0	0.29
---	--	------	------
mPD		0.030	0.68
		nPD:	0.04
		N. weight:	0.8
		Sc. PD:	-0.02
		Sc. rank:	4557.8

PB2			
Pos.	331	obs:	exp:
ttt	F	39467	19641.00
ttc	F	912	20738.00
tta	L	1	0.46
ttg	L	0	0.83
tct	S	1	0.16
tcc	S	0	0.15
tca	S	0	0.27
tcg	S	0	0.04
ctt	L	3	0.84
ctc	L	0	0.59
cta	L	0	0.61
ctg	L	0	0.67
agt	S	0	0.17
agc	S	0	0.21
---	--	------	------
mPD		0.044	0.50
		nPD:	0.09
		N. weight:	0.64
		Sc. PD:	0.012
		Sc. rank:	12380.0

PB2			
Pos.	332	obs:	exp:
cgt	R	0	0.04
cgc	R	0	0.07
cga	R	0	0.18
cgg	R	0	0.17
aaa	K	40284	26547.00
aag	K	96	13833.00
aga	R	2	0.98
agg	R	0	0.55
gaa	E	1	0.59
gag	E	0	0.41
ggt	G	0	0.13
ggc	G	0	0.14
gga	G	1	0.46
ggg	G	0	0.26
---	--	------	------
mPD		0.0050	0.45
		nPD:	0.01
		N. weight:	0.43
		Sc. PD:	-0.025
		Sc. rank:	-7253.8

PB2			
Pos.	333	obs:	exp:
cgt	R	0	800.40
cgc	R	0	1453.00
cga	R	6	3683.00
cgg	R	0	3466.00
aaa	K	11	7.89
aag	K	1	4.11
aga	R	38465	19801.00
agg	R	1899	11166.00
ggt	G	0	0.27
ggc	G	1	0.28
gga	G	1	0.92
ggg	G	0	0.53
---	--	------	------
mPD		0.091	0.89
		nPD:	0.1
		N. weight:	0.58
		Sc. PD:	0.018
		Sc. rank:	11985.0

PB2			
Pos.	334	obs:	exp:
act	T	66	12541.00
acc	T	2	7140.00
aca	T	27926	17477.00
acg	T	12389	3225.00
gct	A	0	0.27
gcc	A	0	0.25
gca	A	1	0.40
gcg	A	0	0.07
---	--	------	------
mPD		0.43	0.68
		nPD:	0.63
		N. weight:	0.77
		Sc. PD:	0.42
		Sc. rank:	25224.0

PB2			
Pos.	335	obs:	exp:
tct	S	0	6515.00
tcc	S	0	6034.00
tca	S	0	10852.00
tcg	S	0	1589.00
att	I	0	0.35
atc	I	1	0.26
ata	I	0	0.39
aat	N	0	1.12
aac	N	2	0.88
agt	S	247	7056.00
agc	S	40129	8329.00
gct	A	0	0.27
gcc	A	1	0.25
gca	A	0	0.40
gcg	A	0	0.07
ggt	G	0	0.54
ggc	G	4	0.56
gga	G	0	1.85
ggg	G	0	1.06
---	--	------	------
mPD		0.013	1.6
		nPD:	0.01
		N. weight:	1.6
		Sc. PD:	-0.1
		Sc. rank:	-33835.0

PB2			
Pos.	336	obs:	exp:
cgt	R	0	0.02
cgc	R	0	0.04
cga	R	0	0.09
cgg	R	0	0.09
aga	R	0	0.49
agg	R	1	0.28
ggt	G	92	5437.00
ggc	G	0	5636.00
gga	G	705	18641.00
ggg	G	39586	10669.00
---	--	------	------
mPD		0.039	0.68
		nPD:	0.06
		N. weight:	1.3
		Sc. PD:	-0.016
		Sc. rank:	14781.0

PB2			
Pos.	337	obs:	exp:
tta	L	0	1.51
ttg	L	13	2.69
tct	S	132	6509.00
tcc	S	200	6027.00
tca	S	3455	10841.00
tcg	S	36547	1588.00
ctt	L	0	2.72
ctc	L	0	1.91
cta	L	0	2.00
ctg	L	0	2.18
act	T	0	11.49
acc	T	0	6.54
aca	T	0	16.01
acg	T	37	2.96
agt	S	0	7048.00
agc	S	0	8321.00
---	--	------	------
mPD		0.17	1.6
		nPD:	0.11
		N. weight:	2.9
		Sc. PD:	0.1
		Sc. rank:	61509.0

PB2			
Pos.	338	obs:	exp:
tct	S	157	6517.00
tcc	S	1	6035.00
tca	S	40144	10854.00
tcg	S	82	1590.00
agt	S	0	7057.00
agc	S	0	8331.00
---	--	------	------
mPD		0.012	1.6
		nPD:	0.01
		N. weight:	1.3
		Sc. PD:	-0.083
		Sc. rank:	-29082.0

PB2			
Pos.	339	obs:	exp:
ttt	F	0	7.30
ttc	F	15	7.70
att	I	0	166.00
atc	I	473	124.30
ata	I	0	182.70
act	T	0	0.31
acc	T	1	0.18
aca	T	0	0.43
acg	T	0	0.08
gtt	V	418	9986.00
gtc	V	39427	8123.00
gta	V	21	8641.00
gtg	V	0	13117.00
gct	A	0	7.63
gcc	A	28	7.00
gca	A	0	11.33
gcg	A	0	2.05
gat	D	0	0.57
gac	D	1	0.43
---	--	------	------
mPD		0.047	0.77
		nPD:	0.06
		N. weight:	1.6
		Sc. PD:	-0.014
		Sc. rank:	19556.0

PB2			
Pos.	340	obs:	exp:
aaa	K	5	3.29
aag	K	0	1.71
---	-	40379	40379.00
---	--	------	------
mPD		0	6.9×10^{-9}
		nPD:	0.
		N. weight:	0.
		Sc. PD:	0
		Sc. rank:	0

PB2

Pos.	341	obs:	exp:
cgt	R	0	0.40
cgc	R	0	0.72
cga	R	0	1.82
cgg	R	0	1.72
att	I	0	3.16
atc	I	0	2.36
ata	I	9	3.48
act	T	0	0.62
acc	T	0	0.35
aca	T	2	0.87
acg	T	0	0.16
aat	N	1	3.36
aac	N	5	2.64
aaa	K	39871	26522.00
aag	K	471	13820.00
aga	R	20	9.81
agg	R	0	5.53
gaa	E	5	2.95
gag	E	0	2.05
---	--	------	------
mPD		0.025	0.45
		nPD:	0.06
		N. weight:	0.38
		Sc. PD:	-0.0055
		Sc. rank:	4087.6

,

PB2

Pos.	342	obs:	exp:
caa	Q	1	0.61
cag	Q	0	0.39
cgt	R	0	337.40
cgc	R	0	612.40
cga	R	3	1553.00
cgg	R	0	1461.00
att	I	0	1.05
atc	I	0	0.79
ata	I	3	1.16
act	T	0	0.93
acc	T	0	0.53
aca	T	3	1.30
acg	T	0	0.24
aat	N	2	2.24
aac	N	2	1.76
aaa	K	22735	15350.00
aag	K	614	7999.00
aga	R	16997	8348.00
agg	R	19	4707.00
gaa	E	3	1.77
gag	E	0	1.23
ggt	G	0	0.27
ggc	G	0	0.28
gga	G	2	0.92
ggg	G	0	0.53
---	--	------	------
mPD		0.52	1.2
		nPD:	0.45
		N. weight:	0.5
		Sc. PD:	0.19
		Sc. rank:	16161.0

,

PB2

Pos.	343	obs:	exp:
aaa	K	1	0.66
aag	K	0	0.34
gtt	V	0	0.25
gtc	V	0	0.20
gta	V	1	0.22
gtg	V	0	0.33
gat	D	20	16.96
gac	D	10	13.04
gaa	E	26703	23787.00
gag	E	13644	16560.00
ggt	G	0	0.67
ggc	G	0	0.70
gga	G	5	2.31
ggg	G	0	1.32
---	--	------	------
mPD		0.45	0.49
		nPD:	0.93
		N. weight:	0.012
		Sc. PD:	0.01
		Sc. rank:	396.4

,

PB2

Pos.	344	obs:	exp:
aaa	K	2	1.31
aag	K	0	0.69
gat	D	0	1.13
gac	D	2	0.87
gaa	E	40332	23805.00
gag	E	46	16573.00
ggt	G	0	0.27
ggc	G	0	0.28
gga	G	2	0.92
ggg	G	0	0.53
---	--	------	------
mPD		0.0026	0.48
		nPD:	0.01
		N. weight:	0.55
		Sc. PD:	-0.035
		Sc. rank:	-13988.0

,

PB2

Pos.	345	obs:	exp:
aaa	K	0	1.31
aag	K	2	0.69
gat	D	7	3.96
gac	D	0	3.04
gaa	E	1780	23802.00
gag	E	38592	16570.00
ggt	G	0	0.40
ggc	G	0	0.42
gga	G	0	1.38
ggg	G	3	0.79
---	--	------	------
mPD		0.085	0.48
		nPD:	0.18
		N. weight:	0.73
		Sc. PD:	0.076
		Sc. rank:	19062.0

,

PB2

Pos.	346	obs:	exp:
gtt	V	0	0.25
gtc	V	0	0.20
gta	V	0	0.22
gtg	V	1	0.33
---	-	40383	40383.00
---	--	------	------
mPD		0	4.5×10^{-10}
		nPD:	0.
		N. weight:	0.
		Sc. PD:	0
		Sc. rank:	0

,

PB2

Pos.	347	obs:	exp:
ttt	F	5	2.43
ttc	F	0	2.57
tta	L	0	3.02
ttg	L	25	5.38
ctt	L	1	5.44
ctc	L	0	3.81
cta	L	0	3.99
ctg	L	0	4.36
att	I	70	24.57
atc	I	0	18.39
ata	I	0	27.03
atg	M	222	222.00
gtt	V	34324	10034.00
gtc	V	149	8163.00
gta	V	23	8683.00
gtg	V	5564	13181.00
gct	A	1	0.27
gcc	A	0	0.25
gca	A	0	0.40
gcg	A	0	0.07
---	--	------	------
mPD		0.27	0.76
		nPD:	0.36
		N. weight:	0.96
		Sc. PD:	0.27
		Sc. rank:	29905.0

,

PB2

Pos.	348	obs:	exp:
ttt	F	1	0.49
ttc	F	0	0.51
tta	L	0	4686.00
ttg	L	0	8349.00
ctt	L	35309	8448.00
ctc	L	4851	5922.00
cta	L	213	6201.00
ctg	L	9	6777.00
att	I	1	0.35
atc	I	0	0.26
ata	I	0	0.39
---	--	------	------
mPD		0.22	1.2
		nPD:	0.19
		N. weight:	1.3
		Sc. PD:	0.15
		Sc. rank:	33991.0

,

PB2

Pos.	349	obs:	exp:
act	T	91	12541.00
acc	T	10	7140.00
aca	T	39805	17477.00
acg	T	477	3225.00
gct	A	0	0.27
gcc	A	0	0.25
gca	A	1	0.40
gcg	A	0	0.07
---	--	------	------
mPD		0.028	0.68
		nPD:	0.04
		N. weight:	0.82
		Sc. PD:	-0.023
		Sc. rank:	3402.3

,

PB2

Pos.	350	obs:	exp:
ggt	G	452	5437.00
ggc	G	39898	5636.00
gga	G	32	18642.00
ggg	G	2	10669.00
---	--	------	------
mPD		0.024	0.68
		nPD:	0.04
		N. weight:	2.
		Sc. PD:	-0.069
		Sc. rank:	560.8

,

PB2

Pos.	351	obs:	exp:
tct	S	0	0.65
tcc	S	0	0.60
tca	S	0	1.08
tcg	S	0	0.16
aat	N	39896	22591.00
aac	N	484	17789.00
agt	S	4	0.70
agc	S	0	0.83
---	--	------	------
mPD		0.024	0.49
		nPD:	0.05
		N. weight:	0.55
		Sc. PD:	-0.012
		Sc. rank:	4343.1

,

PB2

Pos.	352	obs:	exp:
tta	L	0	4686.00
ttg	L	0	8349.00
ctt	L	151	8448.00
ctc	L	40221	5922.00
cta	L	12	6201.00
ctg	L	0	6778.00
---	--	------	------
mPD		0.0080	1.2
		nPD:	0.01
		N. weight:	2.
		Sc. PD:	-0.12
		Sc. rank:	-43788.0

,

PB2			
Pos.	353	obs:	exp:
caa	Q	29885	24685.00
cag	Q	10498	15698.00
cgt	R	0	0.02
cgc	R	0	0.04
cga	R	1	0.09
cgg	R	0	0.09
aga	R	0	0.49
agg	R	0	0.28
---	--	------	------
mPD		0.38	0.48
		nPD:	0.81
		N. weight:	0.039
		Sc. PD:	0.028
		Sc. rank:	1292.9

,

PB2			
Pos.	354	obs:	exp:
tct	S	0	0.65
tcc	S	0	0.60
tca	S	4	1.08
tcg	S	0	0.16
cct	P	0	0.33
ccc	P	0	0.19
cca	P	1	0.34
ccg	P	0	0.14
att	I	0	0.70
atc	I	0	0.53
ata	I	2	0.77
act	T	11	12536.00
acc	T	2	7137.00
aca	T	40250	17469.00
acg	T	103	3224.00
aaa	K	2	1.31
aag	K	0	0.69
agt	S	0	0.70
agc	S	0	0.83
gct	A	0	2.45
gcc	A	0	2.25
gca	A	9	3.64
gcg	A	0	0.66
---	--	------	------
mPD		0.0066	0.68
		nPD:	0.01
		N. weight:	0.87
		Sc. PD:	-0.051
		Sc. rank:	-15972.0

,

PB2			
Pos.	355	obs:	exp:
tta	L	166	4686.00
ttg	L	39793	8349.00
ctt	L	0	8448.00
ctc	L	0	5922.00
cta	L	0	6201.00
ctg	L	424	6777.00
atg	M	1	1.00
---	--	------	------
mPD		0.029	1.2
		nPD:	0.03
		N. weight:	1.6
		Sc. PD:	-0.07
		Sc. rank:	-12198.0

,

PB2			
Pos.	356	obs:	exp:
cgt	R	0	759.10
cgc	R	0	1378.00
cga	R	1	3493.00
cgg	R	1	3288.00
att	I	0	1.05
atc	I	0	0.79
ata	I	3	1.16
aat	N	1	0.56
aac	N	0	0.44
aaa	K	1730	1376.00
aag	K	363	717.00
aga	R	38132	18779.00
agg	R	152	10590.00
ggt	G	0	0.13
ggc	G	0	0.14
gga	G	1	0.46
ggg	G	0	0.26
---	--	------	------
mPD		0.12	0.97
		nPD:	0.13
		N. weight:	0.69
		Sc. PD:	0.04
		Sc. rank:	15706.0

,

PB2			
Pos.	357	obs:	exp:
tta	L	198	24.60
ttg	L	6	43.83
ctt	L	0	44.35
ctc	L	0	31.09
cta	L	8	32.55
ctg	L	0	35.58
att	I	3	14050.00
atc	I	2	10517.00
ata	I	40020	15458.00
atg	M	132	132.00
gtt	V	0	3.76
gtc	V	0	3.06
gta	V	15	3.25
gtg	V	0	4.94
---	--	------	------
mPD		0.018	0.67
		nPD:	0.03
		N. weight:	1.
		Sc. PD:	-0.042
		Sc. rank:	-6228.4

,

PB2			
Pos.	358	obs:	exp:
tct	S	0	5.81
tcc	S	0	5.38
tca	S	0	9.68
tcg	S	0	1.42
cgt	R	0	794.80
cgc	R	0	1442.00
cga	R	7	3658.00
cgg	R	0	3442.00
att	I	0	1.40
atc	I	0	1.05
ata	I	4	1.54
aaa	K	250	164.40
aag	K	0	85.64
agt	S	34	6.29
agc	S	2	7.43
aga	R	39951	19663.00
agg	R	130	11088.00
ggt	G	0	0.81
ggc	G	0	0.84
gga	G	5	2.77
ggg	G	1	1.59
---	--	------	------
mPD		0.021	0.90
		nPD:	0.02
		N. weight:	0.73
		Sc. PD:	-0.033
		Sc. rank:	-6013.1

,

PB2			
Pos.	359	obs:	exp:
tta	L	1	0.12
ttg	L	0	0.21
ctt	L	0	0.21
ctc	L	0	0.15
cta	L	0	0.15
ctg	L	0	0.17
att	I	0	38.61
atc	I	0	28.90
ata	I	110	42.48
atg	M	1	1.00
gtt	V	4	10087.00
gtc	V	1	8206.00
gta	V	39722	8729.00
gtg	V	545	13250.00
---	--	------	------
mPD		0.032	0.75
		nPD:	0.04
		N. weight:	1.5
		Sc. PD:	-0.04
		Sc. rank:	8343.7

,

PB2			
Pos.	360	obs:	exp:
tat	Y	3	1.58
tac	Y	0	1.42
cat	H	40352	22775.00
cac	H	26	17603.00
aat	N	3	1.68
aac	N	0	1.32
---	--	------	------
mPD		0.0016	0.49
		nPD:	0.
		N. weight:	0.6
		Sc. PD:	-0.039
		Sc. rank:	-17076.0

,

PB2			
Pos.	361	obs:	exp:
aaa	K	0	5.26
aag	K	8	2.74
gtt	V	0	0.25
gtc	V	0	0.20
gta	V	0	0.22
gtg	V	1	0.33
gat	D	2	1.13
gac	D	0	0.87
gaa	E	459	23801.00
gag	E	39911	16569.00
ggt	G	0	0.40
ggc	G	0	0.42
gga	G	1	1.38
ggg	G	2	0.79
---	--	------	------
mPD		0.023	0.48
		nPD:	0.05
		N. weight:	0.87
		Sc. PD:	-0.019
		Sc. rank:	6762.6

,

PB2			
Pos.	362	obs:	exp:
ggt	G	5	5437.00
ggc	G	1	5636.00
gga	G	2253	18642.00
ggg	G	38125	10669.00
---	--	------	------
mPD		0.11	0.68
		nPD:	0.16
		N. weight:	1.1
		Sc. PD:	0.096
		Sc. rank:	28305.0

,

PB2			
Pos.	363	obs:	exp:
ttt	F	8	3.89
ttc	F	0	4.11
tat	Y	40081	21279.00
tac	Y	288	19090.00
tgt	C	6	2.34
tgc	C	0	3.66
cat	H	0	0.56
cac	H	1	0.44
---	--	------	------
mPD		0.015	0.50
		nPD:	0.03
		N. weight:	0.63
		Sc. PD:	-0.025
		Sc. rank:	-2778.5

,

PB2			
Pos.	364	obs:	exp:
gaa	E	318	23808.00
gag	E	40065	16575.00
ggt	G	0	0.13
ggc	G	0	0.14
gga	G	0	0.46
ggg	G	1	0.26
---	--	------	------
mPD		0.016	0.48
		nPD:	0.03
		N. weight:	0.89
		Sc. PD:	-0.033
		Sc. rank:	-2440.0

,

PB2

Pos.	365	obs:	exp:
gaa	E	2837	23809.00
gag	E	37547	16575.00
---	--	------	------
mPD		0.13	0.48
		nPD:	0.27
		N. weight:	0.64
		Sc. PD:	0.13
		Sc. rank:	18830.0

,

PB2

Pos.	366	obs:	exp:
ttt	F	27	19642.00
ttc	F	40354	20739.00
tta	L	0	0.35
ttg	L	0	0.62
ctt	L	0	0.63
ctc	L	3	0.44
cta	L	0	0.46
ctg	L	0	0.50
---	--	------	------
mPD		0.0015	0.50
		nPD:	0.
		N. weight:	0.7
		Sc. PD:	-0.046
		Sc. rank:	-20074.0

,

PB2

Pos.	367	obs:	exp:
act	T	57	12539.00
acc	T	11	7139.00
aca	T	40288	17474.00
acg	T	21	3225.00
aaa	K	1	0.66
aag	K	0	0.34
gct	A	0	1.64
gcc	A	0	1.50
gca	A	6	2.43
gcg	A	0	0.44
---	--	------	------
mPD		0.0047	0.68
		nPD:	0.01
		N. weight:	0.87
		Sc. PD:	-0.054
		Sc. rank:	-18919.0

,

PB2

Pos.	368	obs:	exp:
tta	L	0	1.74
ttg	L	6	3.10
ctt	L	0	3.14
ctc	L	0	2.20
cta	L	0	2.30
ctg	L	9	2.52
att	I	8	13.34
atc	I	0	9.98
ata	I	30	14.68
atg	M	40315	40315.00
gtt	V	0	4.01
gtc	V	0	3.26
gta	V	0	3.47
gtg	V	16	5.26
---	--	------	------
mPD		0.0034	0.0044
		nPD:	0.77
		N. weight:	0.0014
		Sc. PD:	0.00098
		Sc. rank:	46.6

,

PB2

Pos.	369	obs:	exp:
atg	M	6	6.00
gtt	V	322	10114.00
gtc	V	147	8227.00
gta	V	163	8751.00
gtg	V	39745	13285.00
gct	A	0	0.27
gcc	A	0	0.25
gca	A	0	0.40
gcg	A	1	0.07
---	--	------	------
mPD		0.031	0.74
		nPD:	0.04
		N. weight:	1.1
		Sc. PD:	-0.029
		Sc. rank:	5190.4

,

PB2

Pos.	370	obs:	exp:
gtt	V	0	0.25
gtc	V	0	0.20
gta	V	0	0.22
gtg	V	1	0.33
ggt	G	4	5437.00
ggc	G	0	5636.00
gga	G	457	18641.00
ggg	G	39922	10669.00
---	--	------	------
mPD		0.023	0.68
		nPD:	0.03
		N. weight:	1.3
		Sc. PD:	-0.048
		Sc. rank:	-1908.2

,

PB2

Pos.	371	obs:	exp:
cgt	R	0	7.67
cgc	R	0	13.92
cga	R	0	35.31
cgg	R	0	33.23
act	T	1	0.93
acc	T	0	0.53
aca	T	2	1.30
acg	T	0	0.24
aat	N	1	1.12
aac	N	1	0.88
aaa	K	39942	26291.00
aag	K	48	13699.00
aga	R	386	189.80
agg	R	1	107.00
gaa	E	2	1.18
gag	E	0	0.82
---	--	------	------
mPD		0.022	0.48
		nPD:	0.05
		N. weight:	0.44
		Sc. PD:	-0.01
		Sc. rank:	2925.2

,

PB2

Pos.	372	obs:	exp:
tct	S	0	0.16
tcc	S	0	0.15
tca	S	0	0.27
tcg	S	0	0.04
cgt	R	0	799.80
cgc	R	0	1451.00
cga	R	46	3681.00
cgg	R	0	3464.00
aaa	K	39	25.64
aag	K	0	13.36
agt	S	1	0.17
agc	S	0	0.21
aga	R	39744	19786.00
agg	R	549	11158.00
ggt	G	0	0.67
ggc	G	0	0.70
gga	G	5	2.31
ggg	G	0	1.32
---	--	------	------
mPD		0.031	0.89
		nPD:	0.04
		N. weight:	0.68
		Sc. PD:	-0.023
		Sc. rank:	316.2

,

PB2

Pos.	373	obs:	exp:
tct	S	0	1.78
tcc	S	0	1.64
tca	S	11	2.96
tcg	S	0	0.43
act	T	0	0.62
acc	T	0	0.35
aca	T	2	0.87
acg	T	0	0.16
agt	S	0	1.92
agc	S	0	2.27
gct	A	31	11003.00
gcc	A	3	10086.00
gca	A	40302	16330.00
gcg	A	35	2951.00
---	--	------	------
mPD		0.0041	0.70
		nPD:	0.01
		N. weight:	0.94
		Sc. PD:	-0.06
		Sc. rank:	-22948.0

,

PB2

Pos.	374	obs:	exp:
act	T	37	12541.00
acc	T	5	7140.00
aca	T	40318	17477.00
acg	T	24	3225.00
---	--	------	------
mPD		0.0033	0.68
		nPD:	0.
		N. weight:	0.87
		Sc. PD:	-0.056
		Sc. rank:	-22778.0

,

PB2

Pos.	375	obs:	exp:
tct	S	2	0.32
tcc	S	0	0.30
tca	S	0	0.54
tcg	S	0	0.08
act	T	5	1.55
acc	T	0	0.88
aca	T	0	2.16
acg	T	0	0.40
agt	S	0	0.35
agc	S	0	0.41
gct	A	40212	11005.00
gcc	A	129	10088.00
gca	A	0	16333.00
gcg	A	36	2952.00
---	--	------	------
mPD		0.0085	0.69
		nPD:	0.01
		N. weight:	1.3
		Sc. PD:	-0.076
		Sc. rank:	-20938.0

,

PB2

Pos.	376	obs:	exp:
tta	L	1	0.12
ttg	L	0	0.21
tat	Y	0	0.53
tac	Y	1	0.47
ctt	L	0	0.21
ctc	L	0	0.15
cta	L	0	0.15
ctg	L	0	0.17
att	I	357	14167.00
atc	I	11	10604.00
ata	I	39988	15585.00
atg	M	16	16.00
act	T	0	1.55
acc	T	0	0.88
aca	T	5	2.16
acg	T	0	0.40
gtt	V	0	1.25
gtc	V	0	1.02
gta	V	5	1.08
gtg	V	0	1.65
---	--	------	------
mPD		0.020	0.66
		nPD:	0.03
		N. weight:	0.95
		Sc. PD:	-0.038
		Sc. rank:	-4496.6

,

PB2			
Pos.	377	obs:	exp:
ttt	F	0	0.49
ttc	F	1	0.51
tta	L	1316	4685.00
ttg	L	0	8347.00
ctt	L	20	8446.00
ctc	L	1094	5921.00
cta	L	37842	6200.00
ctg	L	103	6776.00
att	I	0	2.81
atc	I	1	2.10
ata	I	7	3.09
---	--	------	------
mPD		0.12	1.2
		nPD:	0.11
		N. weight:	1.7
		Sc. PD:	0.059
		Sc. rank:	35652.0

,

PB2			
Pos.	378	obs:	exp:
cgt	R	0	800.30
cgc	R	0	1452.00
cga	R	8	3683.00
cgg	R	0	3466.00
aaa	K	21	13.81
aag	K	0	7.19
aga	R	39941	19797.00
agg	R	413	11164.00
ggt	G	0	0.13
ggc	G	0	0.14
gga	G	1	0.46
ggg	G	0	0.26
---	--	------	------
mPD		0.022	0.89
		nPD:	0.02
		N. weight:	0.7
		Sc. PD:	-0.031
		Sc. rank:	-5616.4

,

PB2			
Pos.	379	obs:	exp:
cgt	R	0	0.14
cgc	R	0	0.25
cga	R	0	0.64
cgg	R	0	0.60
aaa	K	39917	26545.00
aag	K	460	13832.00
aga	R	7	3.43
agg	R	0	1.94
---	--	------	------
mPD		0.023	0.45
		nPD:	0.05
		N. weight:	0.38
		Sc. PD:	-0.0073
		Sc. rank:	3472.3

,

PB2			
Pos.	380	obs:	exp:
tct	S	0	1.94
tcc	S	0	1.79
tca	S	12	3.23
tcg	S	0	0.47
act	T	0	2.17
acc	T	0	1.24
aca	T	7	3.03
acg	T	0	0.56
agt	S	0	2.10
agc	S	0	2.48
gtt	V	0	0.25
gtc	V	0	0.20
gta	V	1	0.22
gtg	V	0	0.33
gct	A	14	11001.00
gcc	A	3	10085.00
gca	A	40309	16327.00
gcg	A	38	2951.00
---	--	------	------
mPD		0.0037	0.70
		nPD:	0.01
		N. weight:	0.94
		Sc. PD:	-0.06
		Sc. rank:	-23918.0

,

PB2			
Pos.	381	obs:	exp:
act	T	44	12541.00
acc	T	40308	7140.00
aca	T	31	17477.00
acg	T	0	3225.00
aat	N	0	0.56
aac	N	1	0.44
---	--	------	------
mPD		0.0038	0.68
		nPD:	0.01
		N. weight:	1.8
		Sc. PD:	-0.11
		Sc. rank:	-44816.0

,

PB2			
Pos.	382	obs:	exp:
cgt	R	0	800.10
cgc	R	0	1452.00
cga	R	30	3682.00
cgg	R	0	3465.00
aaa	K	30	19.72
aag	K	0	10.28
aga	R	39603	19793.00
agg	R	721	11162.00
---	--	------	------
mPD		0.038	0.89
		nPD:	0.04
		N. weight:	0.66
		Sc. PD:	-0.018
		Sc. rank:	3465.2

,

PB2			
Pos.	383	obs:	exp:
cgt	R	0	800.70
cgc	R	0	1453.00
cga	R	19	3684.00
cgg	R	0	3468.00
aaa	K	2	1.31
aag	K	0	0.69
aga	R	40202	19807.00
agg	R	161	11170.00
---	--	------	------
mPD		0.0090	0.89
		nPD:	0.01
		N. weight:	0.72
		Sc. PD:	-0.043
		Sc. rank:	-12890.0

,

PB2			
Pos.	384	obs:	exp:
tta	L	73	4686.00
ttg	L	20788	8349.00
ctt	L	8	8448.00
ctc	L	0	5922.00
cta	L	64	6201.00
ctg	L	19450	6777.00
atg	M	1	1.00
---	--	------	------
mPD		0.51	1.2
		nPD:	0.44
		N. weight:	1.
		Sc. PD:	0.37
		Sc. rank:	32414.0

,

PB2			
Pos.	385	obs:	exp:
att	I	110	161.80
atc	I	351	121.10
ata	I	0	178.00
gtt	V	38367	10000.00
gtc	V	1541	8134.00
gta	V	14	8653.00
gtg	V	0	13135.00
gct	A	1	0.27
gcc	A	0	0.25
gca	A	0	0.40
gcg	A	0	0.07
---	--	------	------
mPD		0.11	0.76
		nPD:	0.15
		N. weight:	1.3
		Sc. PD:	0.098
		Sc. rank:	31322.0

,

PB2			
Pos.	386	obs:	exp:
cat	H	2	1.69
cac	H	1	1.31
caa	Q	38524	24684.00
cag	Q	1857	15697.00
---	--	------	------
mPD		0.088	0.48
		nPD:	0.18
		N. weight:	0.34
		Sc. PD:	0.039
		Sc. rank:	9001.3

,

PB2			
Pos.	387	obs:	exp:
ttt	F	0	27.73
ttc	F	57	29.27
tta	L	339	4679.00
ttg	L	14	8337.00
ctt	L	139	8435.00
ctc	L	39688	5913.00
cta	L	115	6192.00
ctg	L	29	6767.00
att	I	0	1.05
atc	I	3	0.79
ata	I	0	1.16
---	--	------	------
mPD		0.051	1.2
		nPD:	0.04
		N. weight:	1.9
		Sc. PD:	-0.049
		Sc. rank:	11282.0

,

PB2			
Pos.	388	obs:	exp:
tta	L	1	0.23
ttg	L	0	0.41
ctt	L	0	0.42
ctc	L	0	0.29
cta	L	1	0.31
ctg	L	0	0.34
att	I	10	14175.00
atc	I	26	10610.00
ata	I	40343	15594.00
atg	M	2	2.00
gtt	V	0	0.25
gtc	V	0	0.20
gta	V	1	0.22
gtg	V	0	0.33
---	--	------	------
mPD		0.0020	0.66
		nPD:	0.
		N. weight:	1.
		Sc. PD:	-0.066
		Sc. rank:	-28586.0

,

PB2			
Pos.	389	obs:	exp:
tta	L	0	0.35
ttg	L	3	0.62
ctt	L	0	0.63
ctc	L	0	0.44
cta	L	0	0.46
ctg	L	0	0.50
att	I	1	0.35
atc	I	0	0.26
ata	I	0	0.39
atg	M	2	2.00
gtt	V	11	10113.00
gtc	V	0	8227.00
gta	V	565	8751.00
gtg	V	39800	13285.00
gct	A	0	0.55
gcc	A	0	0.50
gca	A	0	0.81
gcg	A	2	0.15
---	--	------	------
mPD		0.029	0.74
		nPD:	0.04
		N. weight:	1.1
		Sc. PD:	-0.034
		Sc. rank:	2271.4

,

PB2			
Pos.	390	obs:	exp:
tct	S	0	6516.00
tcc	S	0	6034.00
tca	S	0	10853.00
tcg	S	0	1589.00
tgt	C	4	1.56
tgc	C	0	2.44
aat	N	1	0.56
aac	N	0	0.44
agt	S	40098	7056.00
agc	S	280	8330.00
ggt	G	1	0.13
ggc	G	0	0.14
gga	G	0	0.46
ggg	G	0	0.26
---	--	------	------
mPD		0.014	1.6
		nPD:	0.01
		N. weight:	1.8
		Sc. PD:	-0.11
		Sc. rank:	-34894.0

,

PB2			
Pos.	391	obs:	exp:
ggt	G	13	5437.00
ggc	G	1	5636.00
gga	G	37899	18642.00
ggg	G	2471	10669.00
---	--	------	------
mPD		0.12	0.68
		nPD:	0.17
		N. weight:	0.61
		Sc. PD:	0.06
		Sc. rank:	15610.0

,

PB2			
Pos.	392	obs:	exp:
tct	S	0	0.16
tcc	S	0	0.15
tca	S	0	0.27
tcg	S	0	0.04
cgt	R	0	785.60
cgc	R	0	1426.00
cga	R	14	3615.00
cgg	R	0	3402.00
aaa	K	619	494.40
aag	K	133	257.60
agt	S	1	0.17
agc	S	0	0.21
aga	R	39211	19434.00
agg	R	397	10959.00
ggt	G	0	1.21
ggc	G	0	1.26
gga	G	9	4.15
ggg	G	0	2.38
---	--	------	------
mPD		0.064	0.92
		nPD:	0.07
		N. weight:	0.68
		Sc. PD:	-0.0004
		Sc. rank:	10798.0

,

PB2			
Pos.	393	obs:	exp:
aat	N	0	19.58
aac	N	35	15.42
gat	D	206	22803.00
gac	D	40134	17537.00
gaa	E	4	2.36
gag	E	0	1.64
ggt	G	0	0.67
ggc	G	5	0.70
gga	G	0	2.31
ggg	G	0	1.32
---	--	------	------
mPD		0.012	0.49
		nPD:	0.02
		N. weight:	0.84
		Sc. PD:	-0.037
		Sc. rank:	-6637.3

,

PB2			
Pos.	394	obs:	exp:
aat	N	3	1.68
aac	N	0	1.32
gct	A	0	0.27
gcc	A	0	0.25
gca	A	1	0.40
gcg	A	0	0.07
gat	D	152	407.00
gac	D	568	313.00
gaa	E	39293	23378.00
gag	E	360	16275.00
ggt	G	0	0.94
ggc	G	0	0.98
gga	G	7	3.23
ggg	G	0	1.85
---	--	------	------
mPD		0.053	0.50
		nPD:	0.11
		N. weight:	0.5
		Sc. PD:	0.018
		Sc. rank:	10670.0

,

PB2			
Pos.	395	obs:	exp:
tta	L	0	0.35
ttg	L	0	0.62
ctt	L	0	0.63
ctc	L	0	0.44
cta	L	0	0.46
ctg	L	3	0.50
cct	P	0	0.67
ccc	P	0	0.37
cca	P	0	0.68
ccg	P	2	0.28
cat	H	17	9.59
cac	H	0	7.41
caa	Q	266	24671.00
cag	Q	40094	15689.00
cgt	R	0	0.02
cgc	R	0	0.04
cga	R	0	0.09
cgg	R	1	0.09
aaa	K	0	0.66
aag	K	1	0.34
aga	R	0	0.49
agg	R	0	0.28
---	--	------	------
mPD		0.014	0.48
		nPD:	0.03
		N. weight:	0.95
		Sc. PD:	-0.038
		Sc. rank:	-4107.7

,

PB2			
Pos.	396	obs:	exp:
tct	S	4	6516.00
tcc	S	3	6035.00
tca	S	40353	10854.00
tcg	S	22	1590.00
agt	S	0	7057.00
agc	S	0	8331.00
gct	A	0	0.55
gcc	A	0	0.50
gca	A	2	0.81
gcg	A	0	0.15
---	--	------	------
mPD		0.0015	1.6
		nPD:	0.
		N. weight:	1.4
		Sc. PD:	-0.094
		Sc. rank:	-43344.0

,

PB2			
Pos.	397	obs:	exp:
tta	L	1	0.12
ttg	L	0	0.21
ctt	L	0	0.21
ctc	L	0	0.15
cta	L	0	0.15
ctg	L	0	0.17
att	I	173	14124.00
atc	I	1	10572.00
ata	I	40061	15539.00
atg	M	7	7.00
act	T	0	3.73
acc	T	0	2.12
aca	T	12	5.19
acg	T	0	0.96
gtt	V	0	32.31
gtc	V	0	26.28
gta	V	129	27.96
gtg	V	0	42.44
---	--	------	------
mPD		0.016	0.67
		nPD:	0.02
		N. weight:	0.98
		Sc. PD:	-0.044
		Sc. rank:	-7981.8

,

PB2			
Pos.	398	obs:	exp:
tct	S	0	2.90
tcc	S	18	2.69
tca	S	0	4.84
tcg	S	0	0.71
act	T	0	5.28
acc	T	17	3.01
aca	T	0	7.36
acg	T	0	1.36
agt	S	0	3.15
agc	S	0	3.71
gtt	V	0	0.25
gtc	V	1	0.20
gta	V	0	0.22
gtg	V	0	0.33
gct	A	782	10997.00
gcc	A	39410	10081.00
gca	A	152	16321.00
gcg	A	4	2950.00
---	--	------	------
mPD		0.047	0.70
		nPD:	0.07
		N. weight:	1.3
		Sc. PD:	-0.0027
		Sc. rank:	20464.0

,

PB2			
Pos.	399	obs:	exp:
caa	Q	2	1.22
cag	Q	0	0.78
aaa	K	4	2.63
aag	K	0	1.37
gaa	E	39771	23803.00
gag	E	603	16571.00
ggt	G	0	0.54
ggc	G	0	0.56
gga	G	4	1.85
ggg	G	0	1.06
---	--	------	------
mPD		0.030	0.48
		nPD:	0.06
		N. weight:	0.48
		Sc. PD:	-0.0039
		Sc. rank:	5913.0

,

PB2			
Pos.	400	obs:	exp:
tct	S	0	1.61
tcc	S	0	1.49
tca	S	10	2.69
tcg	S	0	0.39
act	T	0	3.11
acc	T	0	1.77
aca	T	10	4.33
acg	T	0	0.80
agt	S	0	1.75
agc	S	0	2.06
gtt	V	0	12.52
gtc	V	0	10.19
gta	V	50	10.84
gtg	V	0	16.45
gct	A	9	10988.00
gcc	A	1	10072.00
gca	A	40244	16307.00
gcg	A	60	2947.00
---	--	------	------
mPD		0.0069	0.70
		nPD:	0.01
		N. weight:	0.94
		Sc. PD:	-0.056
		Sc. rank:	-17127.0

,

PB2			
Pos.	401	obs:	exp:
tta	L	4	0.46
ttg	L	0	0.83
ctt	L	0	0.84
ctc	L	0	0.59
cta	L	0	0.61
ctg	L	0	0.67
att	I	1	14148.00
atc	I	0	10590.00
ata	I	40303	15565.00
atg	M	16	16.00
act	T	0	3.42
acc	T	0	1.94
aca	T	11	4.76
acg	T	0	0.88
gtt	V	0	12.27
gtc	V	0	9.98
gta	V	49	10.62
gtg	V	0	16.12
---	--	------	------
mPD		0.0040	0.66
		nPD:	0.01
		N. weight:	1.
		Sc. PD:	-0.063
		Sc. rank:	-24178.0

,

PB2			
Pos.	402	obs:	exp:
att	I	954	14174.00
atc	I	39337	10609.00
ata	I	85	15593.00
act	T	0	2.17
acc	T	7	1.24
aca	T	0	3.03
acg	T	0	0.56
gtt	V	0	0.25
gtc	V	1	0.20
gta	V	0	0.22
gtg	V	0	0.33
---	--	------	------
mPD		0.051	0.66
		nPD:	0.08
		N. weight:	1.3
		Sc. PD:	0.0085
		Sc. rank:	22102.0

,

PB2			
Pos.	403	obs:	exp:
tta	L	0	0.12
ttg	L	0	0.21
ctt	L	0	0.21
ctc	L	0	0.15
cta	L	0	0.15
ctg	L	1	0.17
att	I	0	0.35
atc	I	0	0.26
ata	I	1	0.39
gtt	V	19	10114.00
gtc	V	6	8227.00
gta	V	483	8752.00
gtg	V	39870	13285.00
gct	A	0	1.09
gcc	A	0	1.00
gca	A	0	1.62
gcg	A	4	0.29
---	--	------	------
mPD		0.025	0.74
		nPD:	0.03
		N. weight:	1.1
		Sc. PD:	-0.039
		Sc. rank:	-664.3

,

PB2			
Pos.	404	obs:	exp:
tct	S	0	0.32
tcc	S	2	0.30
tca	S	0	0.54
tcg	S	0	0.08
agt	S	0	0.35
agc	S	0	0.41
gtt	V	0	0.25
gtc	V	1	0.20
gta	V	0	0.22
gtg	V	0	0.33
gct	A	30	11006.00
gcc	A	40232	10089.00
gca	A	118	16334.00
gcg	A	0	2952.00
gat	D	0	0.57
gac	D	1	0.43
---	--	------	------
mPD		0.0075	0.69
		nPD:	0.01
		N. weight:	1.4
		Sc. PD:	-0.084
		Sc. rank:	-24861.0

,

PB2			
Pos.	405	obs:	exp:
att	I	2	9.83
atc	I	0	7.36
ata	I	26	10.81
atg	M	40355	40355.00
gtt	V	0	0.25
gtc	V	0	0.20
gta	V	0	0.22
gtg	V	1	0.33
---	--	------	------
mPD		0.0014	0.0015
		nPD:	0.98
		N. weight:	0.00057
		Sc. PD:	0.00051
		Sc. rank:	19.0

,

PB2			
Pos.	406	obs:	exp:
att	I	0	0.35
atc	I	0	0.26
ata	I	1	0.39
gtt	V	441	10115.00
gtc	V	1	8228.00
gta	V	570	8753.00
gtg	V	39371	13287.00
---	--	------	------
mPD		0.049	0.74
		nPD:	0.07
		N. weight:	1.
		Sc. PD:	-0.0036
		Sc. rank:	15158.0

,

PB2			
Pos.	407	obs:	exp:
ttt	F	40067	19644.00
ttc	F	317	20740.00
---	--	------	------
mPD		0.016	0.50
		nPD:	0.03
		N. weight:	0.71
		Sc. PD:	-0.027
		Sc. rank:	-2507.9

,

PB2			
Pos.	408	obs:	exp:
tta	L	1	0.12
ttg	L	0	0.21
tct	S	0	6515.00
tcc	S	411	6034.00
tca	S	39955	10852.00
tcg	S	9	1589.00
ctt	L	0	0.21
ctc	L	0	0.15
cta	L	0	0.15
ctg	L	0	0.17
act	T	0	2.17
acc	T	0	1.24
aca	T	7	3.03
acg	T	0	0.56
agt	S	0	7056.00
agc	S	0	8329.00
gct	A	0	0.27
gcc	A	0	0.25
gca	A	1	0.40
gcg	A	0	0.07
---	--	------	------
mPD		0.021	1.6
		nPD:	0.01
		N. weight:	1.3
		Sc. PD:	-0.075
		Sc. rank:	-20124.0

,

PB2			
Pos.	409	obs:	exp:
caa	Q	40324	24686.00
cag	Q	60	15698.00
---	--	------	------
mPD		0.0030	0.48
		nPD:	0.01
		N. weight:	0.51
		Sc. PD:	-0.032
		Sc. rank:	-12023.0

,

PB2			
Pos.	410	obs:	exp:
gaa	E	38366	23809.00
gag	E	2018	16575.00
---	--	------	------
mPD		0.095	0.48
		nPD:	0.2
		N. weight:	0.37
		Sc. PD:	0.046
		Sc. rank:	9918.4

,

PB2			
Pos.	411	obs:	exp:
aat	N	3	1.68
aac	N	0	1.32
gtt	V	1	0.25
gtc	V	0	0.20
gta	V	0	0.22
gtg	V	0	0.33
gat	D	40238	22822.00
gac	D	137	17553.00
gaa	E	1	1.77
gag	E	2	1.23
ggt	G	2	0.27
ggc	G	0	0.28
gga	G	0	0.92
ggg	G	0	0.53
---	--	------	------
mPD		0.0072	0.49
		nPD:	0.01
		N. weight:	0.58
		Sc. PD:	-0.032
		Sc. rank:	-8020.7

,

PB2			
Pos.	412	obs:	exp:
tct	S	0	1.78
tcc	S	0	1.64
tca	S	0	2.96
tcg	S	0	0.43
tgt	C	128	15772.00
tgc	C	40232	24588.00
cgt	R	0	0.22
cgc	R	11	0.40
cga	R	0	1.00
cgg	R	0	0.94
agt	S	0	1.92
agc	S	11	2.27
aga	R	0	5.40
agg	R	0	3.04
ggt	G	0	0.27
ggc	G	2	0.28
gga	G	0	0.92
ggg	G	0	0.53
---	--	------	------
mPD		0.0075	0.48
		nPD:	0.02
		N. weight:	0.5
		Sc. PD:	-0.027
		Sc. rank:	-6556.9

,

PB2			
Pos.	413	obs:	exp:
atg	M	30207	30207.00
gtt	V	0	2549.00
gtc	V	5	2074.00
gta	V	17	2206.00
gtg	V	10155	3348.00
---	--	------	------
mPD		0.38	0.68
		nPD:	0.56
		N. weight:	0.29
		Sc. PD:	0.14
		Sc. rank:	9547.1

,

PB2			
Pos.	414	obs:	exp:
tta	L	89	10.79
ttg	L	0	19.23
ctt	L	0	19.45
ctc	L	0	13.64
cta	L	4	14.28
ctg	L	0	15.61
att	I	4	14110.00
atc	I	358	10562.00
ata	I	39833	15523.00
atg	M	30	30.00
act	T	0	0.62
acc	T	0	0.35
aca	T	2	0.87
acg	T	0	0.16
gtt	V	0	16.03
gtc	V	0	13.04
gta	V	64	13.87
gtg	V	0	21.06
---	--	------	------
mPD		0.027	0.67
		nPD:	0.04
		N. weight:	0.95
		Sc. PD:	-0.028
		Sc. rank:	3413.4

,

PB2			
Pos.	415	obs:	exp:
cgt	R	0	0.10
cgc	R	0	0.18
cga	R	0	0.46
cgg	R	0	0.43
aaa	K	40068	26547.00
aag	K	311	13832.00
aga	R	5	2.45
agg	R	0	1.38
---	--	------	------
mPD		0.016	0.45
		nPD:	0.03
		N. weight:	0.4
		Sc. PD:	-0.014
		Sc. rank:	-118.5

,

PB2			
Pos.	416	obs:	exp:
tct	S	0	0.65
tcc	S	0	0.60
tca	S	4	1.08
tcg	S	0	0.16
act	T	0	0.62
acc	T	0	0.35
aca	T	2	0.87
acg	T	0	0.16
agt	S	0	0.70
agc	S	0	0.83
gct	A	24	11005.00
gcc	A	4	10088.00
gca	A	40320	16333.00
gcg	A	30	2952.00
---	--	------	------
mPD		0.0032	0.69
		nPD:	0.
		N. weight:	0.94
		Sc. PD:	-0.061
		Sc. rank:	-25507.0

,

PB2			
Pos.	417	obs:	exp:
att	I	4	1.40
atc	I	0	1.05
ata	I	0	1.54
gtt	V	40284	10114.00
gtc	V	91	8228.00
gta	V	2	8752.00
gtg	V	2	13286.00
gct	A	1	0.27
gcc	A	0	0.25
gca	A	0	0.40
gcg	A	0	0.07
---	--	------	------
mPD		0.0049	0.74
		nPD:	0.01
		N. weight:	1.4
		Sc. PD:	-0.09
		Sc. rank:	-33040.0

,

PB2			
Pos.	418	obs:	exp:
cgt	R	0	800.70
cgc	R	0	1453.00
cga	R	2	3685.00
cgg	R	0	3468.00
aga	R	38476	19808.00
agg	R	1906	11170.00
---	--	------	------
mPD		0.090	0.89
		nPD:	0.1
		N. weight:	0.58
		Sc. PD:	0.018
		Sc. rank:	11897.0

,

PB2			
Pos.	419	obs:	exp:
tct	S	0	0.65
tcc	S	0	0.60
tca	S	0	1.08
tcg	S	0	0.16
tgt	C	1	0.39
tgc	C	0	0.61
agt	S	4	0.70
agc	S	0	0.83
gtt	V	1	0.25
gtc	V	0	0.20
gta	V	0	0.22
gtg	V	0	0.33
ggt	G	39973	5436.00
ggc	G	376	5635.00
gga	G	23	18639.00
ggg	G	6	10667.00
---	--	------	------
mPD		0.020	0.68
		nPD:	0.03
		N. weight:	2.1
		Sc. PD:	-0.081
		Sc. rank:	-9363.0

,

PB2			
Pos.	420	obs:	exp:
gat	D	646	22828.00
gac	D	39738	17556.00
---	--	------	------
mPD		0.031	0.49
		nPD:	0.06
		N. weight:	0.79
		Sc. PD:	-0.0046
		Sc. rank:	10829.0

,

PB2			
Pos.	421	obs:	exp:
tta	L	0	4686.00
ttg	L	65	8349.00
ctt	L	48	8448.00
ctc	L	0	5922.00
cta	L	245	6201.00
ctg	L	40025	6777.00
atg	M	1	1.00
---	--	------	------
mPD		0.018	1.2
		nPD:	0.02
		N. weight:	1.8
		Sc. PD:	-0.098
		Sc. rank:	-24283.0

,

PB2			
Pos.	422	obs:	exp:
aat	N	39952	22594.00
aac	N	432	17790.00
---	--	------	------
mPD		0.021	0.49
		nPD:	0.04
		N. weight:	0.55
		Sc. PD:	-0.015
		Sc. rank:	2918.4

,

PB2			
Pos.	423	obs:	exp:
ttt	F	37454	19644.00
ttc	F	2930	20740.00
---	--	------	------
mPD		0.13	0.50
		nPD:	0.27
		N. weight:	0.48
		Sc. PD:	0.095
		Sc. rank:	14048.0

,

PB2			
Pos.	424	obs:	exp:
att	I	1	1.76
atc	I	4	1.31
ata	I	0	1.93
gtt	V	327	10114.00
gtc	V	39708	8227.00
gta	V	343	8752.00
gtg	V	0	13285.00
gat	D	0	0.57
gac	D	1	0.43
---	--	------	------
mPD		0.033	0.74
		nPD:	0.04
		N. weight:	1.6
		Sc. PD:	-0.039
		Sc. rank:	9691.1

,

PB2			
Pos.	425	obs:	exp:
act	T	1	0.31
acc	T	0	0.18
aca	T	0	0.43
acg	T	0	0.08
aat	N	418	22593.00
aac	N	39965	17790.00
---	--	------	------
mPD		0.021	0.49
		nPD:	0.04
		N. weight:	0.8
		Sc. PD:	-0.022
		Sc. rank:	3466.4

,

PB2			
Pos.	426	obs:	exp:
tct	S	0	0.16
tcc	S	0	0.15
tca	S	0	0.27
tcg	S	0	0.04
cgt	R	0	800.70
cgc	R	0	1453.00
cga	R	4	3685.00
cgg	R	1	3468.00
agt	S	1	0.17
agc	S	0	0.21
aga	R	39481	19807.00
agg	R	897	11170.00
---	--	------	------
mPD		0.044	0.89
		nPD:	0.05
		N. weight:	0.65
		Sc. PD:	-0.013
		Sc. rank:	5589.1

,

PB2			
Pos.	427	obs:	exp:
gtt	V	0	0.25
gtc	V	0	0.20
gta	V	1	0.22
gtg	V	0	0.33
gct	A	40	11007.00
gcc	A	2	10089.00
gca	A	40300	16335.00
gcg	A	41	2952.00
---	--	------	------
mPD		0.0042	0.69
		nPD:	0.01
		N. weight:	0.94
		Sc. PD:	-0.059
		Sc. rank:	-22816.0

,

PB2			
Pos.	428	obs:	exp:
aat	N	39743	22594.00
aac	N	641	17790.00
---	--	------	------
mPD		0.031	0.49
		nPD:	0.06
		N. weight:	0.53
		Sc. PD:	-0.0035
		Sc. rank:	7124.9

,

PB2			
Pos.	429	obs:	exp:
caa	Q	455	24686.00
cag	Q	39929	15698.00
---	--	------	------
mPD		0.022	0.48
		nPD:	0.05
		N. weight:	0.93
		Sc. PD:	-0.021
		Sc. rank:	6834.1

,

PB2			
Pos.	430	obs:	exp:
cgt	R	7	800.70
cgc	R	1	1453.00
cga	R	422	3685.00
cgg	R	39692	3468.00
aga	R	195	19808.00
agg	R	67	11170.00
---	--	------	------
mPD		0.043	0.89
		nPD:	0.05
		N. weight:	2.5
		Sc. PD:	-0.051
		Sc. rank:	20286.0

,

PB2			
Pos.	431	obs:	exp:
tta	L	1624	4686.00
ttg	L	38374	8349.00
ctt	L	0	8448.00
ctc	L	0	5922.00
cta	L	2	6201.00
ctg	L	384	6778.00
---	--	------	------
mPD		0.096	1.2
		nPD:	0.08
		N. weight:	1.5
		Sc. PD:	0.019
		Sc. rank:	26888.0

,

PB2			
Pos.	432	obs:	exp:
tct	S	0	0.16
tcc	S	0	0.15
tca	S	0	0.27
tcg	S	0	0.04
act	T	0	0.31
acc	T	1	0.18
aca	T	0	0.43
acg	T	0	0.08
aat	N	710	22592.00
aac	N	39671	17789.00
agt	S	0	0.17
agc	S	1	0.21
gat	D	0	0.57
gac	D	1	0.43
---	--	------	------
mPD		0.035	0.49
		nPD:	0.07
		N. weight:	0.77
		Sc. PD:	0.00026
		Sc. rank:	12213.0

,

PB2

Pos.	433	obs:	exp:
tct	S	0	1.13
tcc	S	7	1.05
tca	S	0	1.88
tcg	S	0	0.28
cct	P	59	13454.00
ccc	P	40271	7569.00
cca	P	44	13640.00
ccg	P	0	5710.00
act	T	0	0.93
acc	T	3	0.53
aca	T	0	1.30
acg	T	0	0.24
agt	S	0	1.22
agc	S	0	1.44
---	--	------	------
mPD		0.0056	0.72
		nPD:	0.01
		N. weight:	1.7
		Sc. PD:	-0.11
		Sc. rank:	-35802.0

,

PB2

Pos.	434	obs:	exp:
atg	M	40384	40384.00
---	--	------	------
mPD		0	0
		nPD:	1.
		N. weight:	0.
		Sc. PD:	0
		Sc. rank:	0

,

PB2

Pos.	435	obs:	exp:
tat	Y	2	1.05
tac	Y	0	0.95
cat	H	39803	22777.00
cac	H	579	17605.00
---	--	------	------
mPD		0.028	0.49
		nPD:	0.06
		N. weight:	0.53
		Sc. PD:	-0.0064
		Sc. rank:	6103.1

,

PB2

Pos.	436	obs:	exp:
caa	Q	635	24686.00
cag	Q	39749	15698.00
---	--	------	------
mPD		0.031	0.48
		nPD:	0.07
		N. weight:	0.9
		Sc. PD:	-0.0043
		Sc. rank:	12734.0

,

PB2

Pos.	437	obs:	exp:
ttt	F	2	0.97
ttc	F	0	1.03
tta	L	0	4686.00
ttg	L	0	8349.00
ctt	L	39848	8448.00
ctc	L	517	5922.00
cta	L	1	6201.00
ctg	L	16	6777.00
---	--	------	------
mPD		0.026	1.2
		nPD:	0.02
		N. weight:	1.6
		Sc. PD:	-0.074
		Sc. rank:	-13623.0

,

PB2

Pos.	438	obs:	exp:
tta	L	38779	4686.00
ttg	L	1568	8349.00
ctt	L	0	8448.00
ctc	L	0	5922.00
cta	L	36	6201.00
ctg	L	1	6778.00
---	--	------	------
mPD		0.077	1.2
		nPD:	0.07
		N. weight:	2.1
		Sc. PD:	-0.0081
		Sc. rank:	29669.0

,

PB2

Pos.	439	obs:	exp:
cgt	R	0	800.70
cgc	R	0	1453.00
cga	R	0	3685.00
cgg	R	6	3468.00
aga	R	220	19808.00
agg	R	40158	11170.00
---	--	------	------
mPD		0.011	0.89
		nPD:	0.01
		N. weight:	1.3
		Sc. PD:	-0.074
		Sc. rank:	-20152.0

,

PB2

Pos.	440	obs:	exp:
cat	H	40319	22778.00
cac	H	65	17606.00
---	--	------	------
mPD		0.0032	0.49
		nPD:	0.01
		N. weight:	0.59
		Sc. PD:	-0.037
		Sc. rank:	-13691.0

,

PB2

Pos.	441	obs:	exp:
ttt	F	38926	19644.00
ttc	F	1458	20740.00
---	--	------	------
mPD		0.070	0.50
		nPD:	0.14
		N. weight:	0.59
		Sc. PD:	0.041
		Sc. rank:	14092.0

,

PB2

Pos.	442	obs:	exp:
caa	Q	603	24686.00
cag	Q	39781	15698.00
---	--	------	------
mPD		0.029	0.48
		nPD:	0.06
		N. weight:	0.91
		Sc. PD:	-0.0072
		Sc. rank:	11224.0

,

PB2

Pos.	443	obs:	exp:
aaa	K	40134	26550.00
aag	K	250	13834.00
---	--	------	------
mPD		0.012	0.45
		nPD:	0.03
		N. weight:	0.41
		Sc. PD:	-0.017
		Sc. rank:	-2510.2

,

PB2

Pos.	444	obs:	exp:
aat	N	23	12.87
aac	N	0	10.13
gat	D	40266	22806.00
gac	D	80	17540.00
gaa	E	1	1.18
gag	E	1	0.82
ggt	G	13	1.75
ggc	G	0	1.81
gga	G	0	6.00
ggg	G	0	3.43
---	--	------	------
mPD		0.0058	0.49
		nPD:	0.01
		N. weight:	0.59
		Sc. PD:	-0.034
		Sc. rank:	-9545.1

,

PB2

Pos.	445	obs:	exp:
tct	S	0	0.65
tcc	S	0	0.60
tca	S	0	1.08
tcg	S	4	0.16
act	T	0	0.93
acc	T	0	0.53
aca	T	0	1.30
acg	T	3	0.24
agt	S	0	0.70
agc	S	0	0.83
gtt	V	0	0.50
gtc	V	0	0.41
gta	V	0	0.43
gtg	V	2	0.66
gct	A	61	11002.00
gcc	A	0	10085.00
gca	A	567	16329.00
gcg	A	39739	2951.00
gaa	E	1	4.72
gag	E	7	3.28
---	--	------	------
mPD		0.032	0.70
		nPD:	0.05
		N. weight:	2.6
		Sc. PD:	-0.064
		Sc. rank:	17203.0

,

PB2

Pos.	446	obs:	exp:
cgt	R	0	0.02
cgc	R	0	0.04
cga	R	0	0.09
cgg	R	0	0.09
aat	N	0	0.56
aac	N	1	0.44
aaa	K	40231	26549.00
aag	K	151	13833.00
aga	R	1	0.49
agg	R	0	0.28
---	--	------	------
mPD		0.0075	0.45
		nPD:	0.02
		N. weight:	0.42
		Sc. PD:	-0.022
		Sc. rank:	-4983.2

,

PB2

Pos.	447	obs:	exp:
tta	L	5	0.58
ttg	L	0	1.03
ctt	L	0	1.05
ctc	L	0	0.73
cta	L	0	0.77
ctg	L	0	0.84
att	I	0	351.40
atc	I	0	263.00
ata	I	1001	386.60
atg	M	10	10.00
gtt	V	13	9860.00
gtc	V	0	8021.00
gta	V	19692	8532.00
gtg	V	19661	12952.00
gct	A	0	0.27
gcc	A	0	0.25
gca	A	1	0.40
gcg	A	0	0.07
ggt	G	0	0.13
ggc	G	1	0.14
gga	G	0	0.46
ggg	G	0	0.26
---	--	------	------
mPD		0.55	0.79
		nPD:	0.69
		N. weight:	0.67
		Sc. PD:	0.41
		Sc. rank:	21980.0

,

PB2

Pos.	448	obs:	exp:
tta	L	0	4686.00
ttg	L	0	8349.00
ctt	L	3253	8448.00
ctc	L	37048	5922.00
cta	L	34	6201.00
ctg	L	49	6778.00
---	--	------	------
mPD		0.15	1.2
		nPD:	0.13
		N. weight:	1.7
		Sc. PD:	0.1
		Sc. rank:	38741.0

,

PB2			
Pos.	449	obs:	exp:
ttt	F	39919	19641.00
ttc	F	459	20737.00
tta	L	1	0.70
ttg	L	0	1.24
ctt	L	5	1.26
ctc	L	0	0.88
cta	L	0	0.92
ctg	L	0	1.01
---	--	------	------
mPD		0.023	0.50
		nPD:	0.05
		N. weight:	0.69
		Sc. PD:	-0.017
		Sc. rank:	4561.8

PB2			
Pos.	450	obs:	exp:
cat	H	1	2.26
cac	H	3	1.74
caa	Q	39497	24675.00
cag	Q	869	15691.00
cgt	R	0	0.02
cgc	R	0	0.04
cga	R	1	0.09
cgg	R	0	0.09
aaa	K	13	8.55
aag	K	0	4.45
aga	R	0	0.49
agg	R	0	0.28
---	--	------	------
mPD		0.043	0.48
		nPD:	0.09
		N. weight:	0.42
		Sc. PD:	0.0084
		Sc. rank:	8121.3

PB2			
Pos.	451	obs:	exp:
tct	S	0	1.13
tcc	S	0	1.05
tca	S	0	1.88
tcg	S	0	0.28
cat	H	1	0.56
cac	H	0	0.44
att	I	1	0.35
atc	I	0	0.26
ata	I	0	0.39
act	T	1	0.31
acc	T	0	0.18
aca	T	0	0.43
acg	T	0	0.08
aat	N	39960	22587.00
aac	N	413	17786.00
aaa	K	1	0.66
aag	K	0	0.34
agt	S	7	1.22
agc	S	0	1.44
---	--	------	------
mPD		0.021	0.49
		nPD:	0.04
		N. weight:	0.56
		Sc. PD:	-0.015
		Sc. rank:	2570.4

PB2			
Pos.	452	obs:	exp:
tgg	W	40383	40383.00
ggt	G	0	0.13
ggc	G	0	0.14
gga	G	0	0.46
ggg	G	1	0.26
---	--	------	------
mPD		0.000050	0.000086
		nPD:	0.58
		N. weight:	0.000035
		Sc. PD:	0.000017
		Sc. rank:	1.1

PB2			
Pos.	453	obs:	exp:
gaa	E	3	1.77
gag	E	0	1.23
ggt	G	8	5436.00
ggc	G	0	5636.00
gga	G	40170	18641.00
ggg	G	203	10668.00
---	--	------	------
mPD		0.011	0.68
		nPD:	0.02
		N. weight:	0.78
		Sc. PD:	-0.042
		Sc. rank:	-10328.0

PB2			
Pos.	454	obs:	exp:
ttt	F	3	1.46
ttc	F	0	1.54
tta	L	0	0.23
ttg	L	0	0.41
ctt	L	2	0.42
ctc	L	0	0.29
cta	L	0	0.31
ctg	L	0	0.34
att	I	1196	451.40
atc	I	89	337.90
ata	I	1	496.60
act	T	9	2.79
acc	T	0	1.59
aca	T	0	3.89
acg	T	0	0.72
gtt	V	39046	9790.00
gtc	V	21	7964.00
gta	V	9	8471.00
gtg	V	8	12860.00
---	--	------	------
mPD		0.069	0.81
		nPD:	0.09
		N. weight:	1.4
		Sc. PD:	0.022
		Sc. rank:	26933.0

PB2			
Pos.	455	obs:	exp:
gaa	E	40086	23809.00
gag	E	298	16575.00
---	--	------	------
mPD		0.015	0.48
		nPD:	0.03
		N. weight:	0.51
		Sc. PD:	-0.02
		Sc. rank:	-2196.5

PB2			
Pos.	456	obs:	exp:
tct	S	0	2.26
tcc	S	14	2.09
tca	S	0	3.76
tcg	S	0	0.55
tat	Y	0	19.50
tac	Y	37	17.50
cct	P	4	52.98
ccc	P	155	29.81
cca	P	0	53.72
ccg	P	0	22.49
cat	H	1098	22487.00
cac	H	38770	17381.00
caa	Q	35	23.23
cag	Q	3	14.77
cgt	R	6	0.56
cgc	R	22	1.01
cga	R	0	2.55
cgg	R	0	2.40
act	T	0	60.56
acc	T	195	34.48
aca	T	0	84.39
acg	T	0	15.57
aat	N	0	25.18
aac	N	45	19.82
agt	S	0	2.45
agc	S	0	2.89
aga	R	0	13.73
agg	R	0	7.74
---	--	------	------
mPD		0.089	0.53
		nPD:	0.17
		N. weight:	0.75
		Sc. PD:	0.071
		Sc. rank:	18977.0

PB2			
Pos.	457	obs:	exp:
ttt	F	0	0.49
ttc	F	1	0.51
att	I	1218	14174.00
atc	I	39108	10610.00
ata	I	51	15593.00
act	T	0	0.31
acc	T	1	0.18
aca	T	0	0.43
acg	T	0	0.08
gtt	V	0	1.25
gtc	V	5	1.02
gta	V	0	1.08
gtg	V	0	1.65
---	--	------	------
mPD		0.061	0.66
		nPD:	0.09
		N. weight:	1.2
		Sc. PD:	0.028
		Sc. rank:	24687.0

PB2			
Pos.	458	obs:	exp:
gat	D	134	22826.00
gac	D	40247	17555.00
gaa	E	3	1.77
gag	E	0	1.23
---	--	------	------
mPD		0.0068	0.49
		nPD:	0.01
		N. weight:	0.85
		Sc. PD:	-0.047
		Sc. rank:	-12335.0

PB2			
Pos.	459	obs:	exp:
tct	S	0	6456.00
tcc	S	0	5978.00
tca	S	0	10753.00
tcg	S	0	1575.00
cgt	R	1	0.08
cgc	R	0	0.14
cga	R	0	0.36
cgg	R	0	0.34
att	I	4	1.40
atc	I	0	1.05
ata	I	0	1.54
aat	N	368	205.90
aac	N	0	162.10
agt	S	39989	6991.00
agc	S	17	8253.00
aga	R	3	1.96
agg	R	0	1.11
ggt	G	2	0.27
ggc	G	0	0.28
gga	G	0	0.92
ggg	G	0	0.53
---	--	------	------
mPD		0.019	1.6
		nPD:	0.01
		N. weight:	1.8
		Sc. PD:	-0.1
		Sc. rank:	-29813.0

PB2			
Pos.	460	obs:	exp:
att	I	0	0.70
atc	I	0	0.53
ata	I	2	0.77
atg	M	1	1.00
gtt	V	95	10115.00
gtc	V	2	8228.00
gta	V	3505	8752.00
gtg	V	36779	13286.00
---	--	------	------
mPD		0.16	0.74
		nPD:	0.22
		N. weight:	0.88
		Sc. PD:	0.13
		Sc. rank:	24844.0

PB2

Pos.	461	obs:	exp:
tta	L	0	0.23
ttg	L	1	0.41
ctt	L	0	0.42
ctc	L	0	0.29
cta	L	0	0.31
ctg	L	1	0.34
atg	M	40382	40382.00
---	--	------	------
mPD		0.000099	0.00016
		nPD:	0.62
		N. weight:	0.00007
		Sc. PD:	0.000037
		Sc. rank:	2.3

PB2

Pos.	462	obs:	exp:
ggt	G	14	5437.00
ggc	G	6	5636.00
gga	G	40174	18642.00
ggg	G	190	10669.00
---	--	------	------
mPD		0.010	0.68
		nPD:	0.02
		N. weight:	0.78
		Sc. PD:	-0.042
		Sc. rank:	-10557.0

PB2

Pos.	463	obs:	exp:
tta	L	0	0.35
ttg	L	0	0.62
ctt	L	0	0.63
ctc	L	0	0.44
cta	L	0	0.46
ctg	L	3	0.50
atg	M	40380	40380.00
gtt	V	0	0.25
gtc	V	0	0.20
gta	V	0	0.22
gtg	V	1	0.33
---	--	------	------
mPD		0.00020	0.00032
		nPD:	0.61
		N. weight:	0.00017
		Sc. PD:	0.00009
		Sc. rank:	5.5

PB2

Pos.	464	obs:	exp:
tta	L	0	0.58
ttg	L	0	1.03
ctt	L	5	1.05
ctc	L	0	0.73
cta	L	0	0.77
ctg	L	0	0.84
att	I	1732	731.60
atc	I	350	547.60
ata	I	2	804.80
gtt	V	38238	9591.00
gtc	V	45	7802.00
gta	V	7	8299.00
gtg	V	1	12599.00
gct	A	4	1.09
gcc	A	0	1.00
gca	A	0	1.62
gcg	A	0	0.29
---	--	------	------
mPD		0.12	0.84
		nPD:	0.14
		N. weight:	1.4
		Sc. PD:	0.098
		Sc. rank:	33576.0

PB2

Pos.	465	obs:	exp:
ggt	G	48	5437.00
ggc	G	4	5636.00
gga	G	39503	18642.00
ggg	G	829	10669.00
---	--	------	------
mPD		0.043	0.68
		nPD:	0.06
		N. weight:	0.71
		Sc. PD:	-0.005
		Sc. rank:	9370.5

PB2

Pos.	466	obs:	exp:
tta	L	7	1.04
ttg	L	0	1.86
ctt	L	0	1.88
ctc	L	0	1.32
cta	L	2	1.38
ctg	L	0	1.51
att	I	0	190.30
atc	I	0	142.40
ata	I	542	209.30
atg	M	8	8.00
gtt	V	3	9975.00
gtc	V	3	8115.00
gta	V	35095	8632.00
gtg	V	4724	13103.00
---	--	------	------
mPD		0.23	0.77
		nPD:	0.31
		N. weight:	1.2
		Sc. PD:	0.27
		Sc. rank:	35614.0

PB2

Pos.	467	obs:	exp:
ttt	F	0	0.49
ttc	F	1	0.51
tta	L	38974	4686.00
ttg	L	232	8349.00
ctt	L	1	8448.00
ctc	L	0	5922.00
cta	L	969	6201.00
ctg	L	207	6777.00
---	--	------	------
mPD		0.078	1.2
		nPD:	0.07
		N. weight:	2.1
		Sc. PD:	-0.0052
		Sc. rank:	31358.0

PB2

Pos.	468	obs:	exp:
tct	S	0	1.45
tcc	S	0	1.34
tca	S	9	2.42
tcg	S	0	0.35
cct	P	161	13454.00
ccc	P	213	7569.00
cca	P	39820	13640.00
ccg	P	179	5710.00
caa	Q	1	0.61
cag	Q	0	0.39
agt	S	0	1.57
agc	S	0	1.86
gct	A	0	0.27
gcc	A	0	0.25
gca	A	1	0.40
gcg	A	0	0.07
---	--	------	------
mPD		0.028	0.72
		nPD:	0.04
		N. weight:	1.1
		Sc. PD:	-0.033
		Sc. rank:	2090.8

PB2

Pos.	469	obs:	exp:
aat	N	1	0.56
aac	N	0	0.44
gtt	V	1	0.25
gtc	V	0	0.20
gta	V	0	0.22
gtg	V	0	0.33
gat	D	39814	22826.00
gac	D	567	17555.00
ggt	G	1	0.13
ggc	G	0	0.14
gga	G	0	0.46
ggg	G	0	0.26
---	--	------	------
mPD		0.028	0.49
		nPD:	0.06
		N. weight:	0.53
		Sc. PD:	-0.007
		Sc. rank:	5862.6

PB2

Pos.	470	obs:	exp:
tta	L	0	1.04
ttg	L	7	1.86
ctt	L	0	1.88
ctc	L	0	1.32
cta	L	0	1.38
ctg	L	2	1.51
att	I	1	3.16
atc	I	0	2.36
ata	I	8	3.48
atg	M	40365	40365.00
gtt	V	0	0.25
gtc	V	0	0.20
gta	V	0	0.22
gtg	V	1	0.33
---	--	------	------
mPD		0.00094	0.0013
		nPD:	0.75
		N. weight:	0.00049
		Sc. PD:	0.00033
		Sc. rank:	16.0

PB2

Pos.	471	obs:	exp:
tct	S	3	1.13
tcc	S	0	1.05
tca	S	0	1.88
tcg	S	0	0.28
att	I	3	1.05
atc	I	0	0.79
ata	I	0	1.16
act	T	39674	12519.00
acc	T	606	7127.00
aca	T	30	17446.00
acg	T	1	3220.00
aat	N	1	0.56
aac	N	0	0.44
agt	S	4	1.22
agc	S	0	1.44
gct	A	61	16.90
gcc	A	1	15.49
gca	A	0	25.08
gcg	A	0	4.53
---	--	------	------
mPD		0.035	0.68
		nPD:	0.05
		N. weight:	1.2
		Sc. PD:	-0.022
		Sc. rank:	10529.0

PB2

Pos.	472	obs:	exp:
tta	L	0	0.23
ttg	L	0	0.41
ctt	L	0	0.42
ctc	L	0	0.29
cta	L	2	0.31
ctg	L	0	0.34
cct	P	9	13457.00
ccc	P	12	7571.00
cca	P	40314	13643.00
ccg	P	47	5711.00
---	--	------	------
mPD		0.0035	0.72
		nPD:	0.
		N. weight:	1.1
		Sc. PD:	-0.073
		Sc. rank:	-29683.0

PB2

Pos.	473	obs:	exp:
tct	S	0	6515.00
tcc	S	0	6033.00
tca	S	0	10851.00
tcg	S	0	1589.00
tgt	C	0	1.17
tgc	C	3	1.83
act	T	0	0.31
acc	T	1	0.18
aca	T	0	0.43
acg	T	0	0.08
aat	N	1	5.04
aac	N	8	3.96
agt	S	532	7055.00
agc	S	39839	8328.00
---	--	------	------
mPD		0.027	1.6
		nPD:	0.02
		N. weight:	1.6
		Sc. PD:	-0.084
		Sc. rank:	-19348.0

PB2

Pos.	474	obs:	exp:
tta	L	2	0.23
ttg	L	0	0.41
tct	S	0	15.49
tcc	S	0	14.35
tca	S	96	25.80
tcg	S	0	3.78
ctt	L	0	0.42
ctc	L	0	0.29
cta	L	0	0.31
ctg	L	0	0.34
cct	P	0	0.33
ccc	P	0	0.19
cca	P	1	0.34
ccg	P	0	0.14
att	I	0	8.07
atc	I	4	6.04
ata	I	19	8.88
atg	M	1	1.00
act	T	15	12499.00
acc	T	8	7116.00
aca	T	39796	17418.00
acg	T	429	3215.00
aaa	K	5	3.29
aag	K	0	1.71
agt	S	0	16.78
agc	S	0	19.80
gct	A	0	2.18
gcc	A	0	2.00
gca	A	8	3.24
gcg	A	0	0.58
---	--	------	------
mPD		0.029	0.69
		nPD:	0.04
		N. weight:	0.84
		Sc. PD:	-0.023
		Sc. rank:	4227.6

PB2

Pos.	475	obs:	exp:
aaa	K	0	7.23
aag	K	11	3.77
gat	D	32	18.09
gac	D	0	13.91
gaa	E	289	23776.00
gag	E	40040	16553.00
ggt	G	0	1.62
ggc	G	0	1.67
gga	G	0	5.54
ggg	G	12	3.17
---	--	------	------
mPD		0.017	0.49
		nPD:	0.03
		N. weight:	0.89
		Sc. PD:	-0.031
		Sc. rank:	-195.2

PB2

Pos.	476	obs:	exp:
tta	L	0	0.70
ttg	L	2	1.24
ctt	L	0	1.26
ctc	L	0	0.88
cta	L	0	0.92
ctg	L	4	1.01
cgt	R	0	0.02
cgc	R	0	0.04
cga	R	0	0.09
cgg	R	0	0.09
att	I	5	31.24
atc	I	2	23.39
ata	I	82	34.37
atg	M	40098	40098.00
act	T	0	39.75
acc	T	0	22.63
aca	T	0	55.40
acg	T	128	10.22
aaa	K	0	2.63
aag	K	4	1.37
aga	R	0	0.49
agg	R	1	0.28
gtt	V	0	14.53
gtc	V	0	11.82
gta	V	0	12.57
gtg	V	58	19.08
---	--	------	------
mPD		0.014	0.022
		nPD:	0.64
		N. weight:	0.012
		Sc. PD:	0.0067
		Sc. rank:	395.7

PB2

Pos.	477	obs:	exp:
tct	S	5	6517.00
tcc	S	35	6035.00
tca	S	40128	10854.00
tcg	S	216	1590.00
agt	S	0	7057.00
agc	S	0	8331.00
---	--	------	------
mPD		0.013	1.6
		nPD:	0.01
		N. weight:	1.4
		Sc. PD:	-0.083
		Sc. rank:	-27996.0

PB2

Pos.	478	obs:	exp:
tta	L	0	52.91
ttg	L	22	94.28
ctt	L	0	95.39
ctc	L	0	66.87
cta	L	2	70.02
ctg	L	432	76.53
att	I	1	3.51
atc	I	0	2.63
ata	I	9	3.86
atg	M	39918	39918.00
---	--	------	------
mPD		0.023	0.037
		nPD:	0.62
		N. weight:	0.019
		Sc. PD:	0.01
		Sc. rank:	615.9

PB2

Pos.	479	obs:	exp:
cgt	R	0	800.60
cgc	R	0	1453.00
cga	R	4	3684.00
cgg	R	0	3467.00
aaa	K	6	3.94
aag	K	0	2.06
aga	R	40196	19805.00
agg	R	178	11169.00
---	--	------	------
mPD		0.0093	0.89
		nPD:	0.01
		N. weight:	0.72
		Sc. PD:	-0.042
		Sc. rank:	-12749.0

PB2

Pos.	480	obs:	exp:
cgt	R	0	0.14
cgc	R	0	0.25
cga	R	0	0.64
cgg	R	0	0.60
aga	R	7	3.43
agg	R	0	1.94
ggt	G	153	5436.00
ggc	G	2	5635.00
gga	G	39867	18639.00
ggg	G	355	10667.00
---	--	------	------
mPD		0.025	0.68
		nPD:	0.04
		N. weight:	0.75
		Sc. PD:	-0.024
		Sc. rank:	791.2

PB2

Pos.	481	obs:	exp:
att	I	2	14140.00
atc	I	1	10584.00
ata	I	40278	15556.00
atg	M	1	1.00
gtt	V	0	25.55
gtc	V	0	20.78
gta	V	98	22.11
gtg	V	4	33.56
---	--	------	------
mPD		0.0054	0.66
		nPD:	0.01
		N. weight:	1.
		Sc. PD:	-0.061
		Sc. rank:	-20131.0

PB2

Pos.	482	obs:	exp:
cgt	R	0	800.60
cgc	R	0	1453.00
cga	R	5	3684.00
cgg	R	0	3467.00
aaa	K	6	3.94
aag	K	0	2.06
aga	R	40301	19805.00
agg	R	72	11169.00
---	--	------	------
mPD		0.0041	0.89
		nPD:	0.
		N. weight:	0.74
		Sc. PD:	-0.047
		Sc. rank:	-19581.0

PB2

Pos.	483	obs:	exp:
ttt	F	0	0.97
ttc	F	2	1.03
tta	L	0	0.12
ttg	L	0	0.21
ctt	L	0	0.21
ctc	L	1	0.15
cta	L	0	0.15
ctg	L	0	0.17
att	I	0	609.40
atc	I	1728	456.20
ata	I	8	670.40
gtt	V	523	9679.00
gtc	V	37781	7874.00
gta	V	338	8376.00
gtg	V	1	12714.00
gct	A	0	0.55
gcc	A	2	0.50
gca	A	0	0.81
gcg	A	0	0.15
---	--	------	------
mPD		0.12	0.83
		nPD:	0.15
		N. weight:	1.5
		Sc. PD:	0.12
		Sc. rank:	37958.0

PB2

Pos.	484	obs:	exp:
tct	S	0	6517.00
tcc	S	0	6035.00
tca	S	0	10854.00
tcg	S	0	1590.00
tgt	C	0	0.39
tgc	C	1	0.61
agt	S	72	7057.00
agc	S	40311	8331.00
---	--	------	------
mPD		0.0036	1.6
		nPD:	0.
		N. weight:	1.7
		Sc. PD:	-0.11
		Sc. rank:	-48899.0

PB2			
Pos.	485	obs:	exp:
cgt	R	0	0.26
cgc	R	0	0.47
cga	R	0	1.19
cgg	R	0	1.12
act	T	0	0.31
acc	T	0	0.18
aca	T	1	0.43
acg	T	0	0.08
aat	N	0	0.56
aac	N	1	0.44
aaa	K	38768	26539.00
aag	K	1600	13829.00
aga	R	13	6.38
agg	R	0	3.60
gaa	E	1	0.59
gag	E	0	0.41
---	--	------	------
mPD		0.077	0.45
		nPD:	0.17
		N. weight:	0.29
		Sc. PD:	0.029
		Sc. rank:	7582.8

,

PB2			
Pos.	486	obs:	exp:
tta	L	0	0.12
ttg	L	0	0.21
ctt	L	0	0.21
ctc	L	0	0.15
cta	L	0	0.15
ctg	L	1	0.17
cgt	R	0	0.04
cgc	R	0	0.07
cga	R	0	0.18
cgg	R	0	0.17
att	I	0	4.21
atc	I	0	3.15
ata	I	12	4.63
atg	M	40269	40269.00
act	T	0	29.19
acc	T	0	16.62
aca	T	0	40.68
acg	T	94	7.51
aaa	K	0	1.31
aag	K	2	0.69
aga	R	0	0.98
agg	R	2	0.55
gtt	V	0	1.00
gtc	V	0	0.82
gta	V	0	0.87
gtg	V	4	1.32
---	--	------	------
mPD		0.0057	0.010
		nPD:	0.55
		N. weight:	0.0068
		Sc. PD:	0.0032
		Sc. rank:	221.2

,

PB2			
Pos.	487	obs:	exp:
gat	D	1	0.57
gac	D	0	0.43
ggt	G	39307	5437.00
ggc	G	665	5636.00
gga	G	360	18641.00
ggg	G	51	10669.00
---	--	------	------
mPD		0.052	0.68
		nPD:	0.08
		N. weight:	1.9
		Sc. PD:	0.013
		Sc. rank:	34314.0

,

PB2			
Pos.	488	obs:	exp:
att	I	0	0.35
atc	I	0	0.26
ata	I	1	0.39
gtt	V	40	10115.00
gtc	V	0	8228.00
gta	V	2551	8752.00
gtg	V	37791	13287.00
gct	A	0	0.27
gcc	A	0	0.25
gca	A	0	0.40
gcg	A	1	0.07
---	--	------	------
mPD		0.12	0.74
		nPD:	0.16
		N. weight:	0.94
		Sc. PD:	0.086
		Sc. rank:	23461.0

,

PB2			
Pos.	489	obs:	exp:
gat	D	40329	22828.00
gac	D	55	17556.00
---	--	------	------
mPD		0.0027	0.49
		nPD:	0.01
		N. weight:	0.59
		Sc. PD:	-0.038
		Sc. rank:	-14726.0

,

PB2			
Pos.	490	obs:	exp:
gaa	E	40301	23806.00
gag	E	78	16573.00
ggt	G	0	0.67
ggc	G	0	0.70
gga	G	5	2.31
ggg	G	0	1.32
---	--	------	------
mPD		0.0041	0.48
		nPD:	0.01
		N. weight:	0.54
		Sc. PD:	-0.033
		Sc. rank:	-10689.0

,

PB2			
Pos.	491	obs:	exp:
tat	Y	548	21275.00
tac	Y	39814	19087.00
tgt	C	0	0.39
tgc	C	1	0.61
cat	H	0	11.84
cac	H	21	9.16
---	--	------	------
mPD		0.028	0.50
		nPD:	0.06
		N. weight:	0.71
		Sc. PD:	-0.01
		Sc. rank:	7634.2

,

PB2			
Pos.	492	obs:	exp:
ttt	F	0	7.30
ttc	F	15	7.70
tct	S	45	6513.00
tcc	S	40061	6031.00
tca	S	255	10848.00
tcg	S	0	1589.00
tat	Y	0	2.11
tac	Y	4	1.89
cct	P	0	0.67
ccc	P	2	0.37
cca	P	0	0.68
ccg	P	0	0.28
act	T	0	0.62
acc	T	2	0.35
aca	T	0	0.87
acg	T	0	0.16
agt	S	0	7053.00
agc	S	0	8326.00
---	--	------	------
mPD		0.016	1.6
		nPD:	0.01
		N. weight:	1.9
		Sc. PD:	-0.12
		Sc. rank:	-35639.0

,

PB2			
Pos.	493	obs:	exp:
tct	S	0	6510.00
tcc	S	0	6029.00
tca	S	0	10844.00
tcg	S	0	1588.00
tgt	C	6	2.34
tgc	C	0	3.66
cgt	R	1	0.02
cgc	R	0	0.04
cga	R	0	0.09
cgg	R	0	0.09
aat	N	25	13.99
aac	N	0	11.01
agt	S	39631	7050.00
agc	S	714	8323.00
aga	R	0	0.49
agg	R	0	0.28
ggt	G	7	0.94
ggc	G	0	0.98
gga	G	0	3.23
ggg	G	0	1.85
---	--	------	------
mPD		0.037	1.6
		nPD:	0.02
		N. weight:	1.7
		Sc. PD:	-0.082
		Sc. rank:	-15334.0

,

PB2			
Pos.	494	obs:	exp:
tct	S	0	0.32
tcc	S	0	0.30
tca	S	2	0.54
tcg	S	0	0.08
cct	P	0	0.33
ccc	P	0	0.19
cca	P	1	0.34
ccg	P	0	0.14
att	I	0	0.70
atc	I	0	0.53
ata	I	2	0.77
act	T	159	12536.00
acc	T	2	7137.00
aca	T	39796	17470.00
acg	T	411	3224.00
agt	S	0	0.35
agc	S	0	0.41
gct	A	0	3.00
gcc	A	0	2.75
gca	A	11	4.45
gcg	A	0	0.80
---	--	------	------
mPD		0.029	0.68
		nPD:	0.04
		N. weight:	0.82
		Sc. PD:	-0.022
		Sc. rank:	3990.4

,

PB2			
Pos.	495	obs:	exp:
gat	D	7	3.96
gac	D	0	3.04
gaa	E	144	23805.00
gag	E	40233	16572.00
---	--	------	------
mPD		0.0075	0.48
		nPD:	0.02
		N. weight:	0.91
		Sc. PD:	-0.049
		Sc. rank:	-12100.0

,

PB2			
Pos.	496	obs:	exp:
cgt	R	0	800.50
cgc	R	0	1453.00
cga	R	0	3683.00
cgg	R	10	3467.00
aaa	K	2	7.23
aag	K	9	3.77
aga	R	1120	19801.00
agg	R	39241	11167.00
ggt	G	0	0.27
ggc	G	0	0.28
gga	G	0	0.92
ggg	G	2	0.53
---	--	------	------
mPD		0.055	0.89
		nPD:	0.06
		N. weight:	1.2
		Sc. PD:	-0.0092
		Sc. rank:	15365.0

,

PB2			
Pos.	497	obs:	exp:
att	I	3	1.05
atc	I	0	0.79
ata	I	0	1.16
atg	M	3	3.00
gtt	V	29	10114.00
gtc	V	6	8227.00
gta	V	119	8751.00
gtg	V	40223	13285.00
gct	A	0	0.27
gcc	A	0	0.25
gca	A	0	0.40
gcg	A	1	0.07
---	--	------	------
mPD		0.0081	0.74
		nPD:	0.01
		N. weight:	1.1
		Sc. PD:	-0.067
		Sc. rank:	-19522.0

PB2			
Pos.	498	obs:	exp:
tta	L	1	0.12
ttg	L	0	0.21
ctt	L	0	0.21
ctc	L	0	0.15
cta	L	0	0.15
ctg	L	0	0.17
atg	M	12	12.00
gtt	V	20	10109.00
gtc	V	0	8224.00
gta	V	450	8748.00
gtg	V	39890	13279.00
gct	A	0	3.00
gcc	A	0	2.75
gca	A	0	4.45
gcg	A	11	0.80
---	--	------	------
mPD		0.024	0.74
		nPD:	0.03
		N. weight:	1.1
		Sc. PD:	-0.041
		Sc. rank:	-2352.5

PB2			
Pos.	499	obs:	exp:
att	I	1	0.35
atc	I	0	0.26
ata	I	0	0.39
gtt	V	39894	10114.00
gtc	V	93	8228.00
gta	V	16	8752.00
gtg	V	376	13286.00
gct	A	3	1.09
gcc	A	0	1.00
gca	A	1	1.62
gcg	A	0	0.29
---	--	------	------
mPD		0.024	0.74
		nPD:	0.03
		N. weight:	1.4
		Sc. PD:	-0.051
		Sc. rank:	-3308.0

PB2			
Pos.	500	obs:	exp:
tct	S	0	6509.00
tcc	S	0	6028.00
tca	S	0	10842.00
tcg	S	0	1588.00
tgt	C	0	0.78
tgc	C	2	1.22
cgt	R	0	0.10
cgc	R	1	0.18
cga	R	0	0.46
cgg	R	0	0.43
aat	N	1	21.26
aac	N	37	16.74
agt	S	290	7049.00
agc	S	40046	8321.00
aga	R	4	2.45
agg	R	0	1.38
ggt	G	0	0.40
ggc	G	3	0.42
gga	G	0	1.38
ggg	G	0	0.79
---	--	------	------
mPD		0.017	1.6
		nPD:	0.01
		N. weight:	1.6
		Sc. PD:	-0.095
		Sc. rank:	-28659.0

PB2			
Pos.	501	obs:	exp:
att	I	40340	14176.00
atc	I	39	10611.00
ata	I	5	15596.00
---	--	------	------
mPD		0.0022	0.66
		nPD:	0.
		N. weight:	1.1
		Sc. PD:	-0.072
		Sc. rank:	-30988.0

PB2			
Pos.	502	obs:	exp:
gat	D	39945	22828.00
gac	D	439	17556.00
---	--	------	------
mPD		0.022	0.49
		nPD:	0.04
		N. weight:	0.54
		Sc. PD:	-0.014
		Sc. rank:	3035.7

PB2			
Pos.	503	obs:	exp:
caa	Q	0	1.22
cag	Q	2	0.78
cgt	R	10	800.70
cgc	R	0	1453.00
cga	R	874	3684.00
cgg	R	39420	3468.00
aga	R	5	19807.00
agg	R	73	11170.00
---	--	------	------
mPD		0.047	0.89
		nPD:	0.05
		N. weight:	2.5
		Sc. PD:	-0.041
		Sc. rank:	23302.0

PB2			
Pos.	504	obs:	exp:
ttt	F	40283	19630.00
ttc	F	72	20725.00
tct	S	1	0.16
tcc	S	0	0.15
tca	S	0	0.27
tcg	S	0	0.04
tat	Y	27	14.23
tac	Y	0	12.77
agt	S	0	0.17
agc	S	0	0.21
gtt	V	1	0.25
gtc	V	0	0.20
gta	V	0	0.22
gtg	V	0	0.33
---	--	------	------
mPD		0.0050	0.50
		nPD:	0.01
		N. weight:	0.75
		Sc. PD:	-0.044
		Sc. rank:	-13489.0

PB2			
Pos.	505	obs:	exp:
tta	L	130	4686.00
ttg	L	40211	8349.00
ctt	L	0	8448.00
ctc	L	0	5922.00
cta	L	1	6201.00
ctg	L	42	6778.00
---	--	------	------
mPD		0.0086	1.2
		nPD:	0.01
		N. weight:	1.6
		Sc. PD:	-0.1
		Sc. rank:	-34789.0

PB2			
Pos.	506	obs:	exp:
tct	S	0	0.16
tcc	S	0	0.15
tca	S	0	0.27
tcg	S	0	0.04
cgt	R	0	800.60
cgc	R	0	1453.00
cga	R	25	3684.00
cgg	R	2	3467.00
aaa	K	4	2.63
aag	K	0	1.37
agt	S	1	0.17
agc	S	0	0.21
aga	R	39925	19805.00
agg	R	427	11169.00
---	--	------	------
mPD		0.023	0.89
		nPD:	0.03
		N. weight:	0.69
		Sc. PD:	-0.03
		Sc. rank:	-5072.2

PB2			
Pos.	507	obs:	exp:
att	I	3	2.46
atc	I	4	1.84
ata	I	0	2.70
gtt	V	40288	10114.00
gtc	V	74	8227.00
gta	V	14	8751.00
gtg	V	1	13285.00
---	--	------	------
mPD		0.0049	0.74
		nPD:	0.01
		N. weight:	1.4
		Sc. PD:	-0.09
		Sc. rank:	-32864.0

PB2			
Pos.	508	obs:	exp:
tta	L	0	0.12
ttg	L	0	0.21
tct	S	0	0.16
tcc	S	0	0.15
tca	S	0	0.27
tcg	S	0	0.04
ctt	L	0	0.21
ctc	L	0	0.15
cta	L	1	0.15
ctg	L	0	0.17
caa	Q	3	1.83
cag	Q	0	1.17
cgt	R	33	800.60
cgc	R	3	1453.00
cga	R	38839	3684.00
cgg	R	206	3467.00
aaa	K	1	0.66
aag	K	0	0.34
agt	S	0	0.17
agc	S	1	0.21
aga	R	1296	19805.00
agg	R	1	11169.00
---	--	------	------
mPD		0.075	0.89
		nPD:	0.08
		N. weight:	2.3
		Sc. PD:	0.032
		Sc. rank:	42570.0

PB2

Pos.	509	obs:	exp:
aat	N	0	1.12
aac	N	2	0.88
gat	D	2772	22825.00
gac	D	37608	17555.00
ggt	G	0	0.27
ggc	G	2	0.28
gga	G	0	0.92
ggg	G	0	0.53
---	--	------	------
mPD		0.13	0.49
		nPD:	0.26
		N. weight:	0.6
		Sc. PD:	0.11
		Sc. rank:	17296.0

,

PB2

Pos.	510	obs:	exp:
cct	P	0	0.33
ccc	P	0	0.19
cca	P	1	0.34
ccg	P	0	0.14
cat	H	1	0.56
cac	H	0	0.44
caa	Q	40323	24685.00
cag	Q	59	15697.00
---	--	------	------
mPD		0.0030	0.48
		nPD:	0.01
		N. weight:	0.51
		Sc. PD:	-0.032
		Sc. rank:	-11945.0

,

PB2

Pos.	511	obs:	exp:
tta	L	0	1.16
ttg	L	0	2.07
tct	S	0	4.36
tcc	S	0	4.03
tca	S	0	7.26
tcg	S	0	1.06
tgt	C	54	21.10
tgc	C	0	32.90
ctt	L	10	2.09
ctc	L	0	1.47
cta	L	0	1.54
ctg	L	0	1.68
cat	H	19	10.72
cac	H	0	8.28
caa	Q	1	1.83
cag	Q	2	1.17
cgt	R	37443	798.50
cgc	R	135	1449.00
cga	R	214	3674.00
cgg	R	2264	3458.00
agt	S	27	4.72
agc	S	0	5.57
aga	R	209	19752.00
agg	R	6	11139.00
---	--	------	------
mPD		0.15	0.89
		nPD:	0.17
		N. weight:	3.7
		Sc. PD:	0.35
		Sc. rank:	93468.0

,

PB2

Pos.	512	obs:	exp:
tgg	W	1	1.00
gaa	E	0	1.18
gag	E	2	0.82
ggt	G	20	5436.00
ggc	G	0	5636.00
gga	G	1219	18641.00
ggg	G	39142	10668.00
---	--	------	------
mPD		0.060	0.68
		nPD:	0.09
		N. weight:	1.2
		Sc. PD:	0.022
		Sc. rank:	23712.0

,

PB2

Pos.	513	obs:	exp:
tct	S	0	1.29
tcc	S	0	1.20
tca	S	0	2.15
tcg	S	0	0.31
aat	N	39880	22580.00
aac	N	479	17779.00
agt	S	8	1.40
agc	S	0	1.65
gat	D	17	9.61
gac	D	0	7.39
---	--	------	------
mPD		0.025	0.49
		nPD:	0.05
		N. weight:	0.55
		Sc. PD:	-0.011
		Sc. rank:	4815.2

,

PB2

Pos.	514	obs:	exp:
tta	L	1	0.12
ttg	L	0	0.21
ctt	L	0	0.21
ctc	L	0	0.15
cta	L	0	0.15
ctg	L	0	0.17
att	I	0	48.44
atc	I	0	36.26
ata	I	138	53.30
gtt	V	8	10079.00
gtc	V	2	8199.00
gta	V	40096	8721.00
gtg	V	133	13240.00
gct	A	0	1.64
gcc	A	0	1.50
gca	A	6	2.43
gcg	A	0	0.44
---	--	------	------
mPD		0.014	0.75
		nPD:	0.02
		N. weight:	1.6
		Sc. PD:	-0.079
		Sc. rank:	-16783.0

,

PB2

Pos.	515	obs:	exp:
tta	L	39285	4686.00
ttg	L	684	8349.00
ctt	L	0	8448.00
ctc	L	0	5922.00
cta	L	412	6201.00
ctg	L	3	6778.00
---	--	------	------
mPD		0.054	1.2
		nPD:	0.05
		N. weight:	2.1
		Sc. PD:	-0.049
		Sc. rank:	14810.0

,

PB2

Pos.	516	obs:	exp:
ttt	F	0	0.49
ttc	F	1	0.51
tta	L	39600	4683.00
ttg	L	525	8344.00
tct	S	0	0.32
tcc	S	0	0.30
tca	S	2	0.54
tcg	S	0	0.08
ctt	L	0	8443.00
ctc	L	1	5918.00
cta	L	230	6198.00
ctg	L	4	6774.00
att	I	0	5.97
atc	I	0	4.47
ata	I	17	6.57
agt	S	0	0.35
agc	S	0	0.41
gtt	V	0	1.00
gtc	V	0	0.82
gta	V	4	0.87
gtg	V	0	1.32
---	--	------	------
mPD		0.039	1.2
		nPD:	0.03
		N. weight:	2.1
		Sc. PD:	-0.078
		Sc. rank:	-2681.5

PB2

Pos.	517	obs:	exp:
tct	S	39969	6516.00
tcc	S	403	6034.00
tca	S	8	10854.00
tcg	S	1	1590.00
tgt	C	1	0.39
tgc	C	0	0.61
act	T	1	0.62
acc	T	1	0.35
aca	T	0	0.87
acg	T	0	0.16
agt	S	0	7057.00
agc	S	0	8330.00
---	--	------	------
mPD		0.020	1.6
		nPD:	0.01
		N. weight:	1.9
		Sc. PD:	-0.11
		Sc. rank:	-28762.0

,

PB2

Pos.	518	obs:	exp:
tct	S	2	0.32
tcc	S	0	0.30
tca	S	0	0.54
tcg	S	0	0.08
cct	P	39652	13457.00
ccc	P	383	7571.00
cca	P	55	13643.00
ccg	P	292	5711.00
agt	S	0	0.35
agc	S	0	0.41
---	--	------	------
mPD		0.036	0.72
		nPD:	0.05
		N. weight:	1.1
		Sc. PD:	-0.021
		Sc. rank:	9183.1

,

PB2

Pos.	519	obs:	exp:
gaa	E	610	23809.00
gag	E	39774	16575.00
---	--	------	------
mPD		0.030	0.48
		nPD:	0.06
		N. weight:	0.85
		Sc. PD:	-0.0071
		Sc. rank:	10420.0

,

PB2

Pos.	520	obs:	exp:
gaa	E	3672	23809.00
gag	E	36712	16575.00
---	--	------	------
mPD		0.17	0.48
		nPD:	0.34
		N. weight:	0.58
		Sc. PD:	0.16
		Sc. rank:	17996.0

,

PB2			
Pos.	521	obs:	exp:
att	I	0	2.46
atc	I	7	1.84
ata	I	0	2.70
gtt	V	971	10114.00
gtc	V	39390	8227.00
gta	V	16	8751.00
gtg	V	0	13285.00
---	--	------	------
mPD		0.048	0.74
		nPD:	0.06
		N. weight:	1.5
		Sc. PD:	-0.0079
		Sc. rank:	21508.0

,

PB2			
Pos.	522	obs:	exp:
tct	S	0	6517.00
tcc	S	0	6035.00
tca	S	0	10854.00
tcg	S	0	1590.00
aat	N	1	0.56
aac	N	0	0.44
agt	S	40179	7057.00
agc	S	204	8331.00
---	--	------	------
mPD		0.010	1.6
		nPD:	0.01
		N. weight:	1.8
		Sc. PD:	-0.11
		Sc. rank:	-42885.0

,

PB2			
Pos.	523	obs:	exp:
gct	A	0	0.27
gcc	A	0	0.25
gca	A	1	0.40
gcg	A	0	0.07
gaa	E	40194	23808.00
gag	E	189	16575.00
---	--	------	------
mPD		0.0094	0.48
		nPD:	0.02
		N. weight:	0.53
		Sc. PD:	-0.026
		Sc. rank:	-5531.0

,

PB2			
Pos.	524	obs:	exp:
act	T	3	12539.00
acc	T	4	7139.00
aca	T	40151	17474.00
acg	T	218	3225.00
gct	A	0	2.18
gcc	A	0	2.00
gca	A	8	3.24
gcg	A	0	0.58
---	--	------	------
mPD		0.011	0.68
		nPD:	0.02
		N. weight:	0.86
		Sc. PD:	-0.045
		Sc. rank:	-9997.0

,

PB2			
Pos.	525	obs:	exp:
tta	L	0	0.12
ttg	L	0	0.21
ctt	L	0	0.21
ctc	L	0	0.15
cta	L	0	0.15
ctg	L	1	0.17
cat	H	11	6.20
cac	H	0	4.80
caa	Q	3682	24677.00
cag	Q	36688	15693.00
cgt	R	0	0.04
cgc	R	0	0.07
cga	R	0	0.18
cgg	R	2	0.17
aga	R	0	0.98
agg	R	0	0.55
---	--	------	------
mPD		0.17	0.48
		nPD:	0.35
		N. weight:	0.63
		Sc. PD:	0.17
		Sc. rank:	19542.0

,

PB2			
Pos.	526	obs:	exp:
ggt	G	56	5437.00
ggc	G	2	5636.00
gga	G	39412	18642.00
ggg	G	914	10669.00
---	--	------	------
mPD		0.047	0.68
		nPD:	0.07
		N. weight:	0.7
		Sc. PD:	-0.00054
		Sc. rank:	11085.0

,

PB2			
Pos.	527	obs:	exp:
tct	S	0	0.16
tcc	S	0	0.15
tca	S	0	0.27
tcg	S	0	0.04
cct	P	1	0.33
ccc	P	0	0.19
cca	P	0	0.34
ccg	P	0	0.14
att	I	29	10.18
atc	I	0	7.62
ata	I	0	11.20
act	T	39312	12375.00
acc	T	56	7046.00
aca	T	479	17246.00
acg	T	3	3183.00
aat	N	443	249.50
aac	N	3	196.50
aaa	K	1	0.66
aag	K	0	0.34
agt	S	1	0.17
agc	S	0	0.21
gtt	V	1	0.25
gtc	V	0	0.20
gta	V	0	0.22
gtg	V	0	0.33
gct	A	54	14.99
gcc	A	0	13.74
gca	A	1	22.25
gcg	A	0	4.02
---	--	------	------
mPD		0.053	0.71
		nPD:	0.07
		N. weight:	1.1
		Sc. PD:	0.0053
		Sc. rank:	19453.0

,

PB2			
Pos.	528	obs:	exp:
gct	A	0	0.27
gcc	A	0	0.25
gca	A	1	0.40
gcg	A	0	0.07
gaa	E	34532	23808.00
gag	E	5851	16575.00
---	--	------	------
mPD		0.25	0.48
		nPD:	0.51
		N. weight:	0.18
		Sc. PD:	0.077
		Sc. rank:	5746.8

,

PB2			
Pos.	529	obs:	exp:
cgt	R	0	791.70
cgc	R	0	1437.00
cga	R	42	3643.00
cgg	R	0	3429.00
aaa	K	236	299.10
aag	K	219	155.90
aga	R	39520	19585.00
agg	R	367	11044.00
---	--	------	------
mPD		0.053	0.90
		nPD:	0.06
		N. weight:	0.69
		Sc. PD:	-0.0078
		Sc. rank:	8070.1

,

PB2			
Pos.	530	obs:	exp:
tta	L	3	4686.00
ttg	L	554	8349.00
ctt	L	16	8448.00
ctc	L	0	5922.00
cta	L	1298	6201.00
ctg	L	38511	6777.00
atg	M	2	2.00
---	--	------	------
mPD		0.090	1.2
		nPD:	0.08
		N. weight:	1.7
		Sc. PD:	0.013
		Sc. rank:	29408.0

,

PB2			
Pos.	531	obs:	exp:
tct	S	0	0.48
tcc	S	2	0.45
tca	S	1	0.81
tcg	S	0	0.12
act	T	52	12539.00
acc	T	16659	7139.00
aca	T	23652	17474.00
acg	T	14	3225.00
agt	S	0	0.52
agc	S	0	0.62
gct	A	0	1.09
gcc	A	1	1.00
gca	A	3	1.62
gcg	A	0	0.29
---	--	------	------
mPD		0.49	0.68
		nPD:	0.72
		N. weight:	0.55
		Sc. PD:	0.35
		Sc. rank:	18026.0

,

PB2			
Pos.	532	obs:	exp:
att	I	8	14175.00
atc	I	0	10610.00
ata	I	40371	15594.00
act	T	0	0.31
acc	T	0	0.18
aca	T	1	0.43
acg	T	0	0.08
gtt	V	0	1.00
gtc	V	0	0.82
gta	V	4	0.87
gtg	V	0	1.32
---	--	------	------
mPD		0.00064	0.66
		nPD:	0.
		N. weight:	1.
		Sc. PD:	-0.068
		Sc. rank:	-31365.0

,

PB2			
Pos.	533	obs:	exp:
tct	S	2	0.32
tcc	S	0	0.30
tca	S	0	0.54
tcg	S	0	0.08
att	I	11	3.86
atc	I	0	2.89
ata	I	0	4.25
act	T	40294	12536.00
acc	T	53	7137.00
aca	T	15	17470.00
acg	T	6	3224.00
agt	S	0	0.35
agc	S	0	0.41
gct	A	3	0.82
gcc	A	0	0.75
gca	A	0	1.21
gcg	A	0	0.22
---	--	------	------
mPD		0.0045	0.68
		nPD:	0.01
		N. weight:	1.2
		Sc. PD:	-0.076
		Sc. rank:	-28066.0

,

PB2			
Pos.	534	obs:	exp:
tat	Y	38332	21287.00
tac	Y	2052	19097.00
---	--	------	------
mPD		0.096	0.50
		nPD:	0.19
		N. weight:	0.47
		Sc. PD:	0.057
		Sc. rank:	12664.0

,

PB2			
Pos.	535	obs:	exp:
ttt	F	1	0.49
ttc	F	0	0.51
tta	L	5	0.58
ttg	L	0	1.03
tct	S	13	6515.00
tcc	S	1	6034.00
tca	S	39934	10852.00
tcg	S	428	1589.00
ctt	L	0	1.05
ctc	L	0	0.73
cta	L	0	0.77
ctg	L	0	0.84
act	T	0	0.62
acc	T	0	0.35
aca	T	2	0.87
acg	T	0	0.16
agt	S	0	7056.00
agc	S	0	8329.00
---	--	------	------
mPD		0.022	1.6
		nPD:	0.01
		N. weight:	1.3
		Sc. PD:	-0.075
		Sc. rank:	-19613.0

,

PB2			
Pos.	536	obs:	exp:
tta	L	1	0.12
ttg	L	0	0.21
tct	S	28	6517.00
tcc	S	0	6035.00
tca	S	17790	10854.00
tcg	S	22565	1590.00
ctt	L	0	0.21
ctc	L	0	0.15
cta	L	0	0.15
ctg	L	0	0.17
agt	S	0	7057.00
agc	S	0	8331.00
---	--	------	------
mPD		0.49	1.6
		nPD:	0.3
		N. weight:	1.8
		Sc. PD:	0.41
		Sc. rank:	54164.0

,

PB2			
Pos.	537	obs:	exp:
tta	L	0	0.12
ttg	L	1	0.21
tct	S	23	6515.00
tcc	S	0	6033.00
tca	S	1234	10852.00
tcg	S	39117	1589.00
ctt	L	0	0.21
ctc	L	0	0.15
cta	L	0	0.15
ctg	L	0	0.17
cct	P	0	0.67
ccc	P	0	0.37
cca	P	0	0.68
ccg	P	2	0.28
agt	S	0	7055.00
agc	S	0	8329.00
gct	A	0	1.91
gcc	A	0	1.75
gca	A	1	2.83
gcg	A	6	0.51
---	--	------	------
mPD		0.061	1.6
		nPD:	0.04
		N. weight:	3.2
		Sc. PD:	-0.1
		Sc. rank:	4282.9

,

PB2			
Pos.	538	obs:	exp:
tta	L	0	0.46
ttg	L	2	0.83
ctt	L	0	0.84
ctc	L	0	0.59
cta	L	0	0.61
ctg	L	2	0.67
att	I	0	1.05
atc	I	0	0.79
ata	I	3	1.16
atg	M	40376	40376.00
gtt	V	0	0.25
gtc	V	0	0.20
gta	V	0	0.22
gtg	V	1	0.33
---	--	------	------
mPD		0.00040	0.00055
		nPD:	0.72
		N. weight:	0.00023
		Sc. PD:	0.00015
		Sc. rank:	7.6

,

PB2			
Pos.	539	obs:	exp:
tta	L	0	0.23
ttg	L	1	0.41
ctt	L	0	0.42
ctc	L	0	0.29
cta	L	0	0.31
ctg	L	1	0.34
atg	M	40382	40382.00
---	--	------	------
mPD		0.000099	0.00016
		nPD:	0.62
		N. weight:	0.00007
		Sc. PD:	0.000037
		Sc. rank:	2.3

,

PB2			
Pos.	540	obs:	exp:
tgg	W	40384	40384.00
---	--	------	------
mPD		0	0
		nPD:	1.
		N. weight:	0.
		Sc. PD:	0
		Sc. rank:	0

,

PB2			
Pos.	541	obs:	exp:
gaa	E	125	23809.00
gag	E	40259	16575.00
---	--	------	------
mPD		0.0062	0.48
		nPD:	0.01
		N. weight:	0.92
		Sc. PD:	-0.052
		Sc. rank:	-13962.0

,

PB2			
Pos.	542	obs:	exp:
tta	L	0	0.35
ttg	L	0	0.62
ctt	L	3	0.63
ctc	L	0	0.44
cta	L	0	0.46
ctg	L	0	0.50
att	I	39851	14168.00
atc	I	489	10605.00
ata	I	21	15587.00
atg	M	1	1.00
act	T	2	0.62
acc	T	0	0.35
aca	T	0	0.87
acg	T	0	0.16
gtt	V	17	4.26
gtc	V	0	3.46
gta	V	0	3.68
gtg	V	0	5.59
---	--	------	------
mPD		0.026	0.66
		nPD:	0.04
		N. weight:	1.
		Sc. PD:	-0.031
		Sc. rank:	3191.4

,

PB2			
Pos.	543	obs:	exp:
tct	S	0	0.32
tcc	S	0	0.30
tca	S	0	0.54
tcg	S	0	0.08
cat	H	0	1.13
cac	H	2	0.87
act	T	0	0.62
acc	T	2	0.35
aca	T	0	0.87
acg	T	0	0.16
aat	N	3144	22589.00
aac	N	37231	17786.00
agt	S	0	0.35
agc	S	2	0.41
gat	D	0	1.70
gac	D	3	1.30
---	--	------	------
mPD		0.14	0.49
		nPD:	0.29
		N. weight:	0.56
		Sc. PD:	0.12
		Sc. rank:	16674.0

,

PB2			
Pos.	544	obs:	exp:
ggt	G	31997	5437.00
ggc	G	8348	5636.00
gga	G	28	18642.00
ggg	G	11	10669.00
---	--	------	------
mPD		0.33	0.68
		nPD:	0.48
		N. weight:	1.6
		Sc. PD:	0.64
		Sc. rank:	50405.0

,

PB2

Pos.	545	obs:	exp:
cct	P	38126	13457.00
ccc	P	2183	7571.00
cca	P	73	13643.00
ccg	P	0	5711.00
cat	H	1	0.56
cac	H	0	0.44
caa	Q	1	0.61
cag	Q	0	0.39
---	--	------	------
mPD		0.11	0.72
		nPD:	0.15
		N. weight:	0.96
		Sc. PD:	0.072
		Sc. rank:	23167.0

,

PB2

Pos.	546	obs:	exp:
gat	D	1	0.57
gac	D	0	0.43
gaa	E	999	23808.00
gag	E	39384	16575.00
---	--	------	------
mPD		0.048	0.48
		nPD:	0.1
		N. weight:	0.81
		Sc. PD:	0.024
		Sc. rank:	16556.0

,

PB2

Pos.	547	obs:	exp:
tct	S	9	6516.00
tcc	S	0	6035.00
tca	S	981	10854.00
tcg	S	39392	1590.00
cct	P	0	0.67
ccc	P	0	0.37
cca	P	1	0.68
ccg	P	1	0.28
agt	S	0	7057.00
agc	S	0	8331.00
---	--	------	------
mPD		0.048	1.6
		nPD:	0.03
		N. weight:	3.2
		Sc. PD:	-0.13
		Sc. rank:	-15628.0

,

PB2

Pos.	548	obs:	exp:
att	I	295	103.90
atc	I	1	77.78
ata	I	0	114.30
gtt	V	38985	10041.00
gtc	V	550	8168.00
gta	V	85	8689.00
gtg	V	467	13190.00
gat	D	1	0.57
gac	D	0	0.43
---	--	------	------
mPD		0.068	0.76
		nPD:	0.09
		N. weight:	1.3
		Sc. PD:	0.026
		Sc. rank:	25085.0

,

PB2

Pos.	549	obs:	exp:
tta	L	308	4686.00
ttg	L	39038	8349.00
ctt	L	0	8448.00
ctc	L	0	5922.00
cta	L	207	6201.00
ctg	L	829	6777.00
atg	M	2	2.00
---	--	------	------
mPD		0.075	1.2
		nPD:	0.06
		N. weight:	1.5
		Sc. PD:	-0.0074
		Sc. rank:	20834.0

,

PB2

Pos.	550	obs:	exp:
att	I	0	43.53
atc	I	124	32.58
ata	I	0	47.89
gtt	V	38	10084.00
gtc	V	40142	8203.00
gta	V	78	8726.00
gtg	V	1	13246.00
gct	A	0	0.27
gcc	A	1	0.25
gca	A	0	0.40
gcg	A	0	0.07
---	--	------	------
mPD		0.012	0.75
		nPD:	0.02
		N. weight:	1.7
		Sc. PD:	-0.088
		Sc. rank:	-20654.0

,

PB2

Pos.	551	obs:	exp:
tct	S	0	0.16
tcc	S	0	0.15
tca	S	0	0.27
tcg	S	0	0.04
aat	N	39842	22593.00
aac	N	541	17790.00
agt	S	1	0.17
agc	S	0	0.21
---	--	------	------
mPD		0.026	0.49
		nPD:	0.05
		N. weight:	0.54
		Sc. PD:	-0.0087
		Sc. rank:	5339.7

,

PB2

Pos.	552	obs:	exp:
act	T	423	12541.00
acc	T	39931	7140.00
aca	T	30	17477.00
acg	T	0	3225.00
---	--	------	------
mPD		0.022	0.68
		nPD:	0.03
		N. weight:	1.8
		Sc. PD:	-0.064
		Sc. rank:	-3452.8

,

PB2

Pos.	553	obs:	exp:
ttt	F	1	0.49
ttc	F	0	0.51
tat	Y	39744	21286.00
tac	Y	639	19097.00
---	--	------	------
mPD		0.031	0.50
		nPD:	0.06
		N. weight:	0.59
		Sc. PD:	-0.0043
		Sc. rank:	7586.3

,

PB2

Pos.	554	obs:	exp:
caa	Q	39656	24683.00
cag	Q	723	15696.00
cgt	R	0	0.02
cgc	R	0	0.04
cga	R	1	0.09
cgg	R	0	0.09
aaa	K	4	2.63
aag	K	0	1.37
aga	R	0	0.49
agg	R	0	0.28
---	--	------	------
mPD		0.035	0.48
		nPD:	0.07
		N. weight:	0.43
		Sc. PD:	0.0019
		Sc. rank:	7256.2

,

PB2

Pos.	555	obs:	exp:
tgg	W	40384	40384.00
---	--	------	------
mPD		0	0
		nPD:	1.
		N. weight:	0.
		Sc. PD:	0
		Sc. rank:	0

,

PB2

Pos.	556	obs:	exp:
tta	L	0	0.46
ttg	L	0	0.83
ctt	L	0	0.84
ctc	L	4	0.59
cta	L	0	0.61
ctg	L	0	0.67
att	I	227	14119.00
atc	I	39537	10568.00
ata	I	455	15532.00
act	T	0	2.48
acc	T	8	1.41
aca	T	0	3.46
acg	T	0	0.64
aat	N	0	0.56
aac	N	1	0.44
gtt	V	1	38.07
gtc	V	151	30.97
gta	V	0	32.94
gtg	V	0	50.01
---	--	------	------
mPD		0.042	0.67
		nPD:	0.06
		N. weight:	1.3
		Sc. PD:	-0.01
		Sc. rank:	16507.0

,

PB2

Pos.	557	obs:	exp:
tta	L	0	0.23
ttg	L	0	0.41
tct	S	0	0.16
tcc	S	0	0.15
tca	S	0	0.27
tcg	S	0	0.04
ctt	L	0	0.42
ctc	L	2	0.29
cta	L	0	0.31
ctg	L	0	0.34
att	I	67	14076.00
atc	I	39852	10536.00
ata	I	178	15485.00
act	T	0	4.97
acc	T	16	2.83
aca	T	0	6.92
acg	T	0	1.28
aat	N	0	0.56
aac	N	1	0.44
agt	S	0	0.17
agc	S	1	0.21
gtt	V	1	66.63
gtc	V	265	54.20
gta	V	0	57.65
gtg	V	0	87.52
gct	A	0	0.27
gcc	A	1	0.25
gca	A	0	0.40
gcg	A	0	0.07
---	--	------	------
mPD		0.026	0.67
		nPD:	0.04
		N. weight:	1.4
		Sc. PD:	-0.042
		Sc. rank:	3266.6

,

PB2

Pos.	558	obs:	exp:
cgt	R	0	800.10
cgc	R	0	1452.00
cga	R	0	3682.00
cgg	R	14	3465.00
aaa	K	8	20.38
aag	K	23	10.62
aga	R	4219	19792.00
agg	R	36120	11162.00
---	--	------	------
mPD		0.19	0.89
		nPD:	0.21
		N. weight:	0.94
		Sc. PD:	0.13
		Sc. rank:	26142.0

,

PB2

Pos.	559	obs:	exp:
tct	S	0	1.78
tcc	S	0	1.64
tca	S	0	2.96
tcg	S	0	0.43
cat	H	2	1.13
cac	H	0	0.87
aat	N	39921	22582.00
aac	N	443	17782.00
agt	S	11	1.92
agc	S	0	2.27
gat	D	7	3.96
gac	D	0	3.04
---	--	------	------
mPD		0.023	0.49
		nPD:	0.05
		N. weight:	0.55
		Sc. PD:	-0.013
		Sc. rank:	3726.7

,

PB2

Pos.	560	obs:	exp:
tgg	W	40384	40384.00
---	--	------	------
mPD		0	0
		nPD:	1.
		N. weight:	0.
		Sc. PD:	0
		Sc. rank:	0

,

PB2

Pos.	561	obs:	exp:
gaa	E	40303	23809.00
gag	E	81	16575.00
---	--	------	------
mPD		0.0040	0.48
		nPD:	0.01
		N. weight:	0.54
		Sc. PD:	-0.033
		Sc. rank:	-10741.0

,

PB2

Pos.	562	obs:	exp:
ttt	F	7	3.40
ttc	F	0	3.60
tct	S	28	27.92
tcc	S	0	25.85
tca	S	0	46.50
tcg	S	0	6.81
att	I	204	71.61
atc	I	0	53.60
ata	I	0	78.78
act	T	386	120.50
acc	T	0	68.60
aca	T	0	167.90
acg	T	2	30.99
aat	N	5	3.36
aac	N	1	2.64
agt	S	0	30.23
agc	S	145	35.69
gtt	V	246	61.62
gtc	V	0	50.12
gta	V	0	53.32
gtg	V	0	80.94
gct	A	39307	10727.00
gcc	A	21	9833.00
gca	A	27	15921.00
gcg	A	3	2877.00
gat	D	1	0.57
gac	D	0	0.43
ggt	G	1	0.13
ggc	G	0	0.14
gga	G	0	0.46
ggg	G	0	0.26
---	--	------	------
mPD		0.077	0.76
		nPD:	0.1
		N. weight:	1.4
		Sc. PD:	0.043
		Sc. rank:	28038.0

,

PB2

Pos.	563	obs:	exp:
ttt	F	0	0.49
ttc	F	1	0.51
tta	L	1	16.94
ttg	L	145	30.18
ctt	L	0	30.54
ctc	L	0	21.41
cta	L	0	22.42
ctg	L	0	24.50
att	I	594	388.60
atc	I	508	290.90
ata	I	5	427.50
gtt	V	871	9800.00
gtc	V	37913	7972.00
gta	V	127	8480.00
gtg	V	214	12873.00
gct	A	1	1.09
gcc	A	3	1.00
gca	A	0	1.62
gcg	A	0	0.29
gat	D	0	0.57
gac	D	1	0.43
---	--	------	------
mPD		0.15	0.80
		nPD:	0.19
		N. weight:	1.5
		Sc. PD:	0.18
		Sc. rank:	39471.0

,

PB2

Pos.	564	obs:	exp:
aaa	K	40338	26550.00
aag	K	46	13834.00
---	--	------	------
mPD		0.0023	0.45
		nPD:	0.01
		N. weight:	0.43
		Sc. PD:	-0.028
		Sc. rank:	-11222.0

,

PB2

Pos.	565	obs:	exp:
ttt	F	1	0.49
ttc	F	0	0.51
att	I	40209	14169.00
atc	I	136	10606.00
ata	I	18	15588.00
atg	M	13	13.00
aat	N	0	0.56
aac	N	1	0.44
gtt	V	6	1.50
gtc	V	0	1.22
gta	V	0	1.30
gtg	V	0	1.97
---	--	------	------
mPD		0.0087	0.66
		nPD:	0.01
		N. weight:	1.1
		Sc. PD:	-0.06
		Sc. rank:	-15975.0

PB2

Pos.	566	obs:	exp:
caa	Q	40277	24683.00
cag	Q	103	15697.00
cgt	R	0	0.02
cgc	R	0	0.04
cga	R	1	0.09
cgg	R	0	0.09
aaa	K	3	1.97
aag	K	0	1.03
aga	R	0	0.49
agg	R	0	0.28
---	--	------	------
mPD		0.0053	0.48
		nPD:	0.01
		N. weight:	0.5
		Sc. PD:	-0.029
		Sc. rank:	-8512.5

PB2

Pos.	567	obs:	exp:
tgg	W	40384	40384.00
---	--	------	------
mPD		0	0
		nPD:	1.
		N. weight:	0.
		Sc. PD:	0
		Sc. rank:	0

PB2

Pos.	568	obs:	exp:
ttt	F	2	0.97
ttc	F	0	1.03
tct	S	39501	6516.00
tcc	S	476	6034.00
tca	S	395	10853.00
tcg	S	6	1589.00
tat	Y	3	1.58
tac	Y	0	1.42
act	T	1	0.31
acc	T	0	0.18
aca	T	0	0.43
acg	T	0	0.08
agt	S	0	7056.00
agc	S	0	8330.00
---	--	------	------
mPD		0.043	1.6
		nPD:	0.03
		N. weight:	1.8
		Sc. PD:	-0.077
		Sc. rank:	-12056.0

PB2

Pos.	569	obs:	exp:
cat	H	8	4.51
cac	H	0	3.49
caa	Q	303	24680.00
cag	Q	40072	15695.00
cgt	R	0	0.02
cgc	R	0	0.04
cga	R	0	0.09
cgg	R	1	0.09
aga	R	0	0.49
agg	R	0	0.28
---	--	------	------
mPD		0.015	0.48
		nPD:	0.03
		N. weight:	0.95
		Sc. PD:	-0.035
		Sc. rank:	-2736.1

PB2

Pos.	570	obs:	exp:
tct	S	0	7.10
tcc	S	0	6.58
tca	S	0	11.83
tcg	S	0	1.73
act	T	0	0.31
acc	T	1	0.18
aca	T	0	0.43
acg	T	0	0.08
aat	N	24655	22349.00
aac	N	15292	17598.00
aaa	K	2	3.94
aag	K	4	2.06
agt	S	18	7.69
agc	S	26	9.08
gat	D	131	212.00
gac	D	244	163.00
gaa	E	11	6.49
gag	E	0	4.51
---	--	------	------
mPD		0.50	0.52
		nPD:	0.96
		N. weight:	0.0097
		Sc. PD:	0.0085
		Sc. rank:	321.3

PB2

Pos.	571	obs:	exp:
cct	P	39892	13457.00
ccc	P	459	7571.00
cca	P	28	13644.00
ccg	P	5	5712.00
---	--	------	------
mPD		0.024	0.72
		nPD:	0.03
		N. weight:	1.1
		Sc. PD:	-0.039
		Sc. rank:	-978.2

PB2

Pos.	572	obs:	exp:
tct	S	0	3.23
tcc	S	0	2.99
tca	S	20	5.38
tcg	S	0	0.79
act	T	0	188.80
acc	T	9	107.50
aca	T	506	263.10
acg	T	93	48.56
agt	S	0	3.50
agc	S	0	4.13
gtt	V	0	0.25
gtc	V	0	0.20
gta	V	1	0.22
gtg	V	0	0.33
gct	A	16	10835.00
gcc	A	9	9932.00
gca	A	39683	16081.00
gcg	A	46	2906.00
gaa	E	1	0.59
gag	E	0	0.41
---	--	------	------
mPD		0.039	0.73
		nPD:	0.05
		N. weight:	0.94
		Sc. PD:	-0.015
		Sc. rank:	9525.1

PB2

Pos.	573	obs:	exp:
tta	L	0	0.46
ttg	L	2	0.83
ctt	L	0	0.84
ctc	L	0	0.59
cta	L	0	0.61
ctg	L	2	0.67
cgt	R	0	0.02
cgc	R	0	0.04
cga	R	0	0.09
cgg	R	0	0.09
att	I	1	8.42
atc	I	1	6.31
ata	I	22	9.27
atg	M	40327	40327.00
act	T	0	5.90
acc	T	0	3.36
aca	T	0	8.22
acg	T	19	1.52
aaa	K	0	2.63
aag	K	4	1.37
aga	R	0	0.49
agg	R	1	0.28
gtt	V	0	1.25
gtc	V	0	1.02
gta	V	0	1.08
gtg	V	5	1.65
---	--	------	------
mPD		0.0028	0.0041
		nPD:	0.68
		N. weight:	0.0021
		Sc. PD:	0.0013
		Sc. rank:	69.6

PB2

Pos.	574	obs:	exp:
tta	L	428	4686.00
ttg	L	39566	8349.00
ctt	L	0	8448.00
ctc	L	0	5922.00
cta	L	7	6201.00
ctg	L	382	6777.00
aaa	K	1	0.66
aag	K	0	0.34
---	--	------	------
mPD		0.041	1.2
		nPD:	0.03
		N. weight:	1.6
		Sc. PD:	-0.054
		Sc. rank:	164.3

PB2

Pos.	575	obs:	exp:
tat	Y	27	21287.00
tac	Y	40357	19097.00
---	--	------	------
mPD		0.0013	0.50
		nPD:	0.
		N. weight:	0.78
		Sc. PD:	-0.052
		Sc. rank:	-22864.0

PB2

Pos.	576	obs:	exp:
tct	S	0	0.16
tcc	S	0	0.15
tca	S	0	0.27
tcg	S	0	0.04
aat	N	113	22593.00
aac	N	40269	17789.00
aaa	K	0	0.66
aag	K	1	0.34
agt	S	0	0.17
agc	S	1	0.21
---	--	------	------
mPD		0.0057	0.49
		nPD:	0.01
		N. weight:	0.84
		Sc. PD:	-0.049
		Sc. rank:	-13951.0

PB2

Pos.	577	obs:	exp:
cgt	R	0	0.38
cgc	R	0	0.68
cga	R	0	1.73
cgg	R	0	1.63
aaa	K	40310	26537.00
aag	K	55	13828.00
aga	R	19	9.32
agg	R	0	5.26
---	--	------	------
mPD		0.0037	0.45
		nPD:	0.01
		N. weight:	0.43
		Sc. PD:	-0.026
		Sc. rank:	-8739.8

,

PB2

Pos.	578	obs:	exp:
tta	L	0	18.45
ttg	L	135	32.87
tgg	W	1	1.00
ctt	L	0	33.26
ctc	L	0	23.32
cta	L	0	24.42
ctg	L	24	26.68
att	I	5	27.03
atc	I	0	20.23
ata	I	72	29.74
atg	M	40133	40133.00
gtt	V	0	3.51
gtc	V	0	2.85
gta	V	0	3.03
gtg	V	14	4.61
---	--	------	------
mPD		0.012	0.018
		nPD:	0.7
		N. weight:	0.0069
		Sc. PD:	0.0043
		Sc. rank:	226.2

,

PB2

Pos.	579	obs:	exp:
gaa	E	40323	23809.00
gag	E	61	16575.00
---	--	------	------
mPD		0.0030	0.48
		nPD:	0.01
		N. weight:	0.55
		Sc. PD:	-0.034
		Sc. rank:	-12943.0

,

PB2

Pos.	580	obs:	exp:
ttt	F	40340	19644.00
ttc	F	44	20740.00
---	--	------	------
mPD		0.0022	0.50
		nPD:	0.
		N. weight:	0.75
		Sc. PD:	-0.049
		Sc. rank:	-20441.0

,

PB2

Pos.	581	obs:	exp:
gaa	E	40201	23804.00
gag	E	175	16572.00
ggt	G	0	1.08
ggc	G	0	1.12
gga	G	8	3.69
ggg	G	0	2.11
---	--	------	------
mPD		0.0090	0.48
		nPD:	0.02
		N. weight:	0.53
		Sc. PD:	-0.027
		Sc. rank:	-5844.3

,

PB2

Pos.	582	obs:	exp:
tct	S	0	2.10
tcc	S	0	1.94
tca	S	11	3.49
tcg	S	2	0.51
cct	P	17	13453.00
ccc	P	5	7568.00
cca	P	34979	13639.00
ccg	P	5370	5710.00
agt	S	0	2.27
agc	S	0	2.68
---	--	------	------
mPD		0.23	0.72
		nPD:	0.32
		N. weight:	0.85
		Sc. PD:	0.21
		Sc. rank:	26067.0

,

PB2

Pos.	583	obs:	exp:
ttt	F	40054	19643.00
ttc	F	328	20739.00
tta	L	1	0.23
ttg	L	0	0.41
ctt	L	1	0.42
ctc	L	0	0.29
cta	L	0	0.31
ctg	L	0	0.34
---	--	------	------
mPD		0.016	0.50
		nPD:	0.03
		N. weight:	0.71
		Sc. PD:	-0.026
		Sc. rank:	-1843.3

,

PB2

Pos.	584	obs:	exp:
caa	Q	39653	24686.00
cag	Q	731	15698.00
---	--	------	------
mPD		0.036	0.48
		nPD:	0.07
		N. weight:	0.43
		Sc. PD:	0.002
		Sc. rank:	7372.0

,

PB2

Pos.	585	obs:	exp:
ttt	F	1	0.49
ttc	F	0	0.51
tct	S	39753	6516.00
tcc	S	618	6034.00
tca	S	3	10853.00
tcg	S	6	1590.00
cct	P	1	0.33
ccc	P	0	0.19
cca	P	0	0.34
ccg	P	0	0.14
agt	S	0	7057.00
agc	S	0	8330.00
gct	A	2	0.55
gcc	A	0	0.50
gca	A	0	0.81
gcg	A	0	0.15
---	--	------	------
mPD		0.031	1.6
		nPD:	0.02
		N. weight:	1.8
		Sc. PD:	-0.093
		Sc. rank:	-20005.0

,

PB2

Pos.	586	obs:	exp:
tta	L	39812	4686.00
ttg	L	147	8349.00
ctt	L	354	8448.00
ctc	L	1	5922.00
cta	L	69	6201.00
ctg	L	1	6778.00
---	--	------	------
mPD		0.045	1.2
		nPD:	0.04
		N. weight:	2.2
		Sc. PD:	-0.066
		Sc. rank:	5806.2

,

PB2

Pos.	587	obs:	exp:
att	I	3	23.17
atc	I	63	17.34
ata	I	0	25.49
gtt	V	664	10099.00
gtc	V	39619	8215.00
gta	V	35	8739.00
gtg	V	0	13266.00
---	--	------	------
mPD		0.037	0.74
		nPD:	0.05
		N. weight:	1.6
		Sc. PD:	-0.031
		Sc. rank:	14192.0

,

PB2

Pos.	588	obs:	exp:
cct	P	910	13457.00
ccc	P	39438	7571.00
cca	P	36	13644.00
ccg	P	0	5712.00
---	--	------	------
mPD		0.046	0.72
		nPD:	0.06
		N. weight:	1.6
		Sc. PD:	-0.01
		Sc. rank:	22122.0

,

PB2			
Pos.	589	obs:	exp:
caa	Q	0	1.22
cag	Q	2	0.78
cgt	R	0	0.46
cgc	R	0	0.83
cga	R	0	2.10
cgg	R	0	1.97
act	T	0	0.31
acc	T	0	0.18
aca	T	0	0.43
acg	T	1	0.08
aat	N	4	2.24
aac	N	0	1.76
aaa	K	158	26530.00
aag	K	40196	13824.00
aga	R	0	11.28
agg	R	23	6.36
---	--	------	------
mPD		0.0093	0.45
		nPD:	0.02
		N. weight:	1.1
		Sc. PD:	-0.054
		Sc. rank:	-11199.0

PB2			
Pos.	590	obs:	exp:
act	T	0	0.31
acc	T	1	0.18
aca	T	0	0.43
acg	T	0	0.08
gct	A	62	11006.00
gcc	A	39905	10089.00
gca	A	366	16335.00
gcg	A	49	2952.00
ggt	G	0	0.13
ggc	G	1	0.14
gga	G	0	0.46
ggg	G	0	0.26
---	--	------	------
mPD		0.023	0.69
		nPD:	0.03
		N. weight:	1.4
		Sc. PD:	-0.049
		Sc. rank:	-964.9

PB2			
Pos.	591	obs:	exp:
ttt	F	5	2.43
ttc	F	0	2.57
tta	L	0	0.46
ttg	L	0	0.83
tct	S	7	2.10
tcc	S	0	1.94
tca	S	0	3.49
tcg	S	0	0.51
ctt	L	4	0.84
ctc	L	0	0.59
cta	L	0	0.61
ctg	L	0	0.67
att	I	5294	1865.00
atc	I	18	1396.00
ata	I	2	2052.00
act	T	34309	10802.00
acc	T	443	6150.00
aca	T	28	15053.00
acg	T	2	2778.00
aat	N	14	7.83
aac	N	0	6.17
agt	S	6	2.27
agc	S	0	2.68
gtt	V	166	41.58
gtc	V	0	33.82
gta	V	0	35.98
gtg	V	0	54.62
gct	A	85	23.44
gcc	A	1	21.49
gca	A	0	34.79
gcg	A	0	6.29
---	--	------	------
mPD		0.27	0.93
		nPD:	0.29
		N. weight:	1.2
		Sc. PD:	0.25
		Sc. rank:	34614.0

PB2			
Pos.	592	obs:	exp:
tct	S	0	0.16
tcc	S	0	0.15
tca	S	0	0.27
tcg	S	0	0.04
cgt	R	0	800.60
cgc	R	0	1453.00
cga	R	1	3684.00
cgg	R	0	3467.00
aaa	K	3	1.97
aag	K	0	1.03
agt	S	0	0.17
agc	S	1	0.21
aga	R	40323	19806.00
agg	R	56	11169.00
---	--	------	------
mPD		0.0030	0.89
		nPD:	0.
		N. weight:	0.74
		Sc. PD:	-0.049
		Sc. rank:	-20918.0

PB2			
Pos.	593	obs:	exp:
tct	S	0	6432.00
tcc	S	0	5956.00
tca	S	0	10713.00
tcg	S	0	1569.00
tgt	C	0	0.78
tgc	C	2	1.22
cgt	R	0	0.08
cgc	R	0	0.14
cga	R	0	0.36
cgg	R	0	0.34
act	T	0	0.62
acc	T	2	0.35
aca	T	0	0.87
acg	T	0	0.16
aat	N	7	17.34
aac	N	24	13.66
agt	S	3699	6965.00
agc	S	36158	8222.00
aga	R	2	1.96
agg	R	2	1.11
ggt	G	2	65.70
ggc	G	485	68.11
gga	G	1	225.30
ggg	G	0	128.90
---	--	------	------
mPD		0.19	1.6
		nPD:	0.12
		N. weight:	1.4
		Sc. PD:	0.063
		Sc. rank:	30480.0

PB2			
Pos.	594	obs:	exp:
tta	L	0	0.23
ttg	L	0	0.41
ctt	L	0	0.42
ctc	L	0	0.29
cta	L	2	0.31
ctg	L	0	0.34
cct	P	0	0.67
ccc	P	0	0.37
cca	P	2	0.68
ccg	P	0	0.28
cat	H	0	3.38
cac	H	6	2.62
caa	Q	39913	24458.00
cag	Q	98	15553.00
cgt	R	147	7.18
cgc	R	0	13.03
cga	R	5	33.03
cgg	R	210	31.08
aaa	K	1	0.66
aag	K	0	0.34
aga	R	0	177.60
agg	R	0	100.10
---	--	------	------
mPD		0.041	0.51
		nPD:	0.08
		N. weight:	0.52
		Sc. PD:	0.0051
		Sc. rank:	9350.5

PB2			
Pos.	595	obs:	exp:
tat	Y	596	21287.00
tac	Y	39788	19097.00
---	--	------	------
mPD		0.029	0.50
		nPD:	0.06
		N. weight:	0.71
		Sc. PD:	-0.0081
		Sc. rank:	8255.0

PB2			
Pos.	596	obs:	exp:
tct	S	0	6517.00
tcc	S	0	6035.00
tca	S	0	10854.00
tcg	S	0	1590.00
agt	S	40365	7057.00
agc	S	19	8331.00
---	--	------	------
mPD		0.00094	1.6
		nPD:	0.
		N. weight:	1.8
		Sc. PD:	-0.13
		Sc. rank:	-58417.0

PB2			
Pos.	597	obs:	exp:
ggt	G	33	5437.00
ggc	G	0	5636.00
gga	G	860	18642.00
ggg	G	39491	10669.00
---	--	------	------
mPD		0.043	0.68
		nPD:	0.06
		N. weight:	1.3
		Sc. PD:	-0.0079
		Sc. rank:	17460.0

PB2			
Pos.	598	obs:	exp:
ttt	F	39977	19643.00
ttc	F	405	20739.00
tta	L	0	0.23
ttg	L	0	0.41
ctt	L	0	0.42
ctc	L	2	0.29
cta	L	0	0.31
ctg	L	0	0.34
---	--	------	------
mPD		0.020	0.50
		nPD:	0.04
		N. weight:	0.7
		Sc. PD:	-0.021
		Sc. rank:	2444.6

PB2			
Pos.	599	obs:	exp:
att	I	0	1.40
atc	I	4	1.05
ata	I	0	1.54
gtt	V	92	10114.00
gtc	V	39866	8228.00
gta	V	276	8752.00
gtg	V	146	13286.00
---	--	------	------
mPD		0.025	0.74
		nPD:	0.03
		N. weight:	1.6
		Sc. PD:	-0.056
		Sc. rank:	-737.3

PB2			
Pos.	600	obs:	exp:
cgt	R	0	800.70
cgc	R	0	1453.00
cga	R	16	3685.00
cgg	R	0	3468.00
aga	R	39518	19808.00
agg	R	850	11170.00
---	--	------	------
mPD		0.042	0.89
		nPD:	0.05
		N. weight:	0.65
		Sc. PD:	-0.015
		Sc. rank:	5003.7

PB2

Pos.	601	obs:	exp:
tct	S	0	0.16
tcc	S	0	0.15
tca	S	0	0.27
tcg	S	0	0.04
cct	P	1	0.33
ccc	P	0	0.19
cca	P	0	0.34
ccg	P	0	0.14
att	I	63	22.12
atc	I	0	16.55
ata	I	0	24.33
act	T	39777	12516.00
acc	T	154	7126.00
aca	T	372	17443.00
acg	T	1	3219.00
agt	S	1	0.17
agc	S	0	0.21
gct	A	15	4.09
gcc	A	0	3.75
gca	A	0	6.07
gcg	A	0	1.10
---	--	------	------
mPD		0.030	0.68
		nPD:	0.04
		N. weight:	1.2
		Sc. PD:	-0.03
		Sc. rank:	6396.2

,

PB2

Pos.	602	obs:	exp:
tta	L	48	4686.00
ttg	L	0	8349.00
ctt	L	11	8448.00
ctc	L	3	5922.00
cta	L	39634	6201.00
ctg	L	688	6778.00
---	--	------	------
mPD		0.037	1.2
		nPD:	0.03
		N. weight:	1.9
		Sc. PD:	-0.071
		Sc. rank:	-6115.6

,

PB2

Pos.	603	obs:	exp:
ttt	F	223	19643.00
ttc	F	40160	20740.00
tta	L	0	0.12
ttg	L	0	0.21
ctt	L	0	0.21
ctc	L	1	0.15
cta	L	0	0.15
ctg	L	0	0.17
---	--	------	------
mPD		0.011	0.50
		nPD:	0.02
		N. weight:	0.67
		Sc. PD:	-0.031
		Sc. rank:	-6385.5

,

PB2

Pos.	604	obs:	exp:
caa	Q	39959	24686.00
cag	Q	425	15698.00
---	--	------	------
mPD		0.021	0.48
		nPD:	0.04
		N. weight:	0.46
		Sc. PD:	-0.012
		Sc. rank:	2612.1

,

PB2

Pos.	605	obs:	exp:
cct	P	0	0.33
ccc	P	0	0.19
cca	P	1	0.34
ccg	P	0	0.14
caa	Q	40339	24685.00
cag	Q	44	15698.00
---	--	------	------
mPD		0.0022	0.48
		nPD:	0.
		N. weight:	0.51
		Sc. PD:	-0.033
		Sc. rank:	-13536.0

,

PB2

Pos.	606	obs:	exp:
att	I	0	1.05
atc	I	1	0.79
ata	I	2	1.16
atg	M	40378	40378.00
act	T	0	0.62
acc	T	0	0.35
aca	T	0	0.87
acg	T	2	0.16
gtt	V	0	0.25
gtc	V	0	0.20
gta	V	0	0.22
gtg	V	1	0.33
---	--	------	------
mPD		0.00030	0.00042
		nPD:	0.7
		N. weight:	0.00022
		Sc. PD:	0.00014
		Sc. rank:	7.1

,

PB2

Pos.	607	obs:	exp:
cgt	R	0	800.70
cgc	R	0	1453.00
cga	R	1	3685.00
cgg	R	155	3468.00
aga	R	39857	19808.00
agg	R	371	11170.00
---	--	------	------
mPD		0.033	0.89
		nPD:	0.04
		N. weight:	0.68
		Sc. PD:	-0.022
		Sc. rank:	1016.7

,

PB2

Pos.	608	obs:	exp:
tat	Y	0	0.53
tac	Y	1	0.47
gat	D	986	22826.00
gac	D	39395	17555.00
gaa	E	1	0.59
gag	E	0	0.41
ggt	G	0	0.13
ggc	G	1	0.14
gga	G	0	0.46
ggg	G	0	0.26
---	--	------	------
mPD		0.048	0.49
		nPD:	0.1
		N. weight:	0.75
		Sc. PD:	0.02
		Sc. rank:	15314.0

,

PB2

Pos.	609	obs:	exp:
tta	L	1	0.12
ttg	L	0	0.21
ctt	L	0	0.21
ctc	L	0	0.15
cta	L	0	0.15
ctg	L	0	0.17
att	I	0	25.63
atc	I	0	19.18
ata	I	73	28.19
gtt	V	243	10097.00
gtc	V	20	8213.00
gta	V	39367	8737.00
gtg	V	680	13263.00
---	--	------	------
mPD		0.049	0.74
		nPD:	0.07
		N. weight:	1.5
		Sc. PD:	-0.0051
		Sc. rank:	21293.0

,

PB2

Pos.	610	obs:	exp:
ttt	F	7	3.40
ttc	F	0	3.60
tta	L	0	4681.00
ttg	L	0	8340.00
ctt	L	39202	8439.00
ctc	L	1124	5915.00
cta	L	8	6194.00
ctg	L	5	6770.00
cct	P	1	0.33
ccc	P	0	0.19
cca	P	0	0.34
ccg	P	0	0.14
att	I	37	12.99
atc	I	0	9.72
ata	I	0	14.29
---	--	------	------
mPD		0.057	1.2
		nPD:	0.05
		N. weight:	1.5
		Sc. PD:	-0.031
		Sc. rank:	12709.0

,

PB2

Pos.	611	obs:	exp:
gaa	E	0	0.59
gag	E	1	0.41
ggt	G	33	5437.00
ggc	G	0	5636.00
gga	G	5190	18641.00
ggg	G	35160	10669.00
---	--	------	------
mPD		0.23	0.68
		nPD:	0.33
		N. weight:	0.92
		Sc. PD:	0.24
		Sc. rank:	28264.0

,

PB2

Pos.	612	obs:	exp:
act	T	37	12541.00
acc	T	4	7140.00
aca	T	40308	17477.00
acg	T	35	3225.00
---	--	------	------
mPD		0.0038	0.68
		nPD:	0.01
		N. weight:	0.87
		Sc. PD:	-0.055
		Sc. rank:	-21452.0

,

PB2			
Pos.	613	obs:	exp:
ttt	F	40359	19643.00
ttc	F	23	20739.00
tta	L	0	0.12
ttg	L	0	0.21
tat	Y	1	0.53
tac	Y	0	0.47
ctt	L	1	0.21
ctc	L	0	0.15
cta	L	0	0.15
ctg	L	0	0.17
---	--	------	------
mPD		0.0012	0.50
		nPD:	0.
		N. weight:	0.75
		Sc. PD:	-0.05
		Sc. rank:	-22120.0

PB2			
Pos.	614	obs:	exp:
tat	Y	0	0.53
tac	Y	1	0.47
aat	N	0	2.24
aac	N	4	1.76
gtt	V	0	0.25
gtc	V	1	0.20
gta	V	0	0.22
gtg	V	0	0.33
gat	D	660	22820.00
gac	D	39711	17551.00
gaa	E	5	2.95
gag	E	0	2.05
ggt	G	0	0.27
ggc	G	2	0.28
gga	G	0	0.92
ggg	G	0	0.53
---	--	------	------
mPD		0.033	0.49
		nPD:	0.07
		N. weight:	0.79
		Sc. PD:	-0.0026
		Sc. rank:	11510.0

PB2			
Pos.	615	obs:	exp:
tct	S	1	0.48
tcc	S	2	0.45
tca	S	0	0.81
tcg	S	0	0.12
att	I	1	0.35
atc	I	0	0.26
ata	I	0	0.39
act	T	37434	12536.00
acc	T	2863	7137.00
aca	T	64	17470.00
acg	T	7	3224.00
agt	S	0	0.52
agc	S	0	0.62
gct	A	12	3.27
gcc	A	0	3.00
gca	A	0	4.85
gcg	A	0	0.88
---	--	------	------
mPD		0.14	0.68
		nPD:	0.2
		N. weight:	0.99
		Sc. PD:	0.13
		Sc. rank:	27335.0

PB2			
Pos.	616	obs:	exp:
tct	S	0	2.26
tcc	S	12	2.09
tca	S	0	3.76
tcg	S	0	0.55
att	I	0	10.53
atc	I	30	7.88
ata	I	0	11.59
act	T	26	754.30
acc	T	2403	429.50
aca	T	0	1051.00
acg	T	0	194.00
aat	N	0	0.56
aac	N	1	0.44
agt	S	0	2.45
agc	S	2	2.89
gtt	V	3	101.20
gtc	V	401	82.32
gta	V	0	87.56
gtg	V	0	132.90
gct	A	126	10222.00
gcc	A	37366	9371.00
gca	A	14	15171.00
gcg	A	0	2742.00
---	--	------	------
mPD		0.14	0.83
		nPD:	0.17
		N. weight:	1.5
		Sc. PD:	0.15
		Sc. rank:	37757.0

PB2			
Pos.	617	obs:	exp:
cat	H	1	0.56
cac	H	0	0.44
caa	Q	523	24685.00
cag	Q	39859	15697.00
cgt	R	0	0.02
cgc	R	0	0.04
cga	R	0	0.09
cgg	R	1	0.09
aga	R	0	0.49
agg	R	0	0.28
---	--	------	------
mPD		0.026	0.48
		nPD:	0.05
		N. weight:	0.92
		Sc. PD:	-0.014
		Sc. rank:	9128.4

PB2			
Pos.	618	obs:	exp:
att	I	4	14152.00
atc	I	78	10593.00
ata	I	40232	15569.00
atg	M	7	7.00
act	T	0	2.17
acc	T	0	1.24
aca	T	7	3.03
acg	T	0	0.56
gtt	V	0	14.03
gtc	V	0	11.41
gta	V	56	12.14
gtg	V	0	18.43
---	--	------	------
mPD		0.0075	0.66
		nPD:	0.01
		N. weight:	0.99
		Sc. PD:	-0.057
		Sc. rank:	-16428.0

PB2			
Pos.	619	obs:	exp:
att	I	3	14173.00
atc	I	0	10608.00
ata	I	40370	15592.00
atg	M	2	2.00
act	T	0	0.31
acc	T	0	0.18
aca	T	1	0.43
acg	T	0	0.08
gtt	V	0	2.00
gtc	V	0	1.63
gta	V	8	1.73
gtg	V	0	2.63
---	--	------	------
mPD		0.00069	0.66
		nPD:	0.
		N. weight:	1.
		Sc. PD:	-0.068
		Sc. rank:	-31314.0

PB2			
Pos.	620	obs:	exp:
cgt	R	0	0.02
cgc	R	0	0.04
cga	R	0	0.09
cgg	R	0	0.09
aaa	K	624	26549.00
aag	K	39759	13834.00
aga	R	0	0.49
agg	R	1	0.28
---	--	------	------
mPD		0.030	0.45
		nPD:	0.07
		N. weight:	1.
		Sc. PD:	-0.0024
		Sc. rank:	15785.0

PB2			
Pos.	621	obs:	exp:
tta	L	0	4686.00
ttg	L	0	8349.00
ctt	L	40258	8448.00
ctc	L	77	5922.00
cta	L	45	6201.00
ctg	L	4	6778.00
---	--	------	------
mPD		0.0062	1.2
		nPD:	0.01
		N. weight:	1.6
		Sc. PD:	-0.1
		Sc. rank:	-40949.0

PB2			
Pos.	622	obs:	exp:
ttt	F	0	0.49
ttc	F	1	0.51
tta	L	0	4683.00
ttg	L	0	8343.00
ctt	L	280	8441.00
ctc	L	40027	5917.00
cta	L	46	6196.00
ctg	L	0	6772.00
att	I	0	10.53
atc	I	30	7.88
ata	I	0	11.59
---	--	------	------
mPD		0.018	1.2
		nPD:	0.02
		N. weight:	2.
		Sc. PD:	-0.11
		Sc. rank:	-26944.0

PB2			
Pos.	623	obs:	exp:
cct	P	39594	13457.00
ccc	P	787	7571.00
cca	P	3	13644.00
ccg	P	0	5712.00
---	--	------	------
mPD		0.038	0.72
		nPD:	0.05
		N. weight:	1.1
		Sc. PD:	-0.018
		Sc. rank:	10453.0

PB2			
Pos.	624	obs:	exp:
ttt	F	40364	19642.00
ttc	F	16	20738.00
tta	L	0	0.12
ttg	L	1	0.21
tct	S	1	0.16
tcc	S	0	0.15
tca	S	0	0.27
tcg	S	0	0.04
tat	Y	2	1.05
tac	Y	0	0.95
ctt	L	0	0.21
ctc	L	0	0.15
cta	L	0	0.15
ctg	L	0	0.17
agt	S	0	0.17
agc	S	0	0.21
---	--	------	------
mPD		0.00099	0.50
		nPD:	0.
		N. weight:	0.76
		Sc. PD:	-0.051
		Sc. rank:	-22816.0

PB2

Pos.	625	obs:	exp:
tct	S	0	0.81
tcc	S	0	0.75
tca	S	5	1.34
tcg	S	0	0.20
act	T	0	2.79
acc	T	0	1.59
aca	T	9	3.89
acg	T	0	0.72
agt	S	0	0.87
agc	S	0	1.03
gtt	V	3	1.00
gtc	V	0	0.82
gta	V	1	0.87
gtg	V	0	1.32
gct	A	406	11002.00
gcc	A	10	10085.00
gca	A	39911	16328.00
gcg	A	39	2951.00
---	--	------	------
mPD		0.023	0.70
		nPD:	0.03
		N. weight:	0.89
		Sc. PD:	-0.032
		Sc. rank:	-728.4

PB2

Pos.	626	obs:	exp:
tct	S	5	0.81
tcc	S	0	0.75
tca	S	0	1.34
tcg	S	0	0.20
act	T	1	0.62
acc	T	1	0.35
aca	T	0	0.87
acg	T	0	0.16
agt	S	0	0.87
agc	S	0	1.03
gct	A	39572	11005.00
gcc	A	788	10088.00
gca	A	14	16333.00
gcg	A	3	2952.00
---	--	------	------
mPD		0.039	0.69
		nPD:	0.06
		N. weight:	1.3
		Sc. PD:	-0.016
		Sc. rank:	14218.0

PB2

Pos.	627	obs:	exp:
tct	S	11	1.78
tcc	S	0	1.64
tca	S	0	2.96
tcg	S	0	0.43
act	T	1	0.31
acc	T	0	0.18
aca	T	0	0.43
acg	T	0	0.08
agt	S	0	1.92
agc	S	0	2.27
gct	A	40049	11004.00
gcc	A	303	10087.00
gca	A	11	16331.00
gcg	A	9	2951.00
---	--	------	------
mPD		0.016	0.70
		nPD:	0.02
		N. weight:	1.3
		Sc. PD:	-0.06
		Sc. rank:	-10993.0

PB2

Pos.	628	obs:	exp:
tta	L	0	0.12
ttg	L	0	0.21
tct	S	0	0.16
tcc	S	0	0.15
tca	S	1	0.27
tcg	S	0	0.04
ctt	L	0	0.21
ctc	L	0	0.15
cta	L	1	0.15
ctg	L	0	0.17
cct	P	6	13457.00
ccc	P	4	7571.00
cca	P	40350	13643.00
ccg	P	22	5711.00
agt	S	0	0.17
agc	S	0	0.21
---	--	------	------
mPD		0.0017	0.72
		nPD:	0.
		N. weight:	1.1
		Sc. PD:	-0.076
		Sc. rank:	-33479.0

PB2

Pos.	629	obs:	exp:
cct	P	52	13457.00
ccc	P	2	7571.00
cca	P	3366	13644.00
ccg	P	36964	5712.00
---	--	------	------
mPD		0.16	0.72
		nPD:	0.22
		N. weight:	1.7
		Sc. PD:	0.24
		Sc. rank:	46940.0

PB2

Pos.	630	obs:	exp:
cgt	R	0	0.26
cgc	R	0	0.47
cga	R	0	1.19
cgg	R	0	1.12
act	T	0	0.31
acc	T	0	0.18
aca	T	0	0.43
acg	T	1	0.08
aaa	K	394	26296.00
aag	K	39604	13702.00
aga	R	0	6.38
agg	R	13	3.60
gaa	E	370	219.30
gag	E	2	152.70
---	--	------	------
mPD		0.056	0.47
		nPD:	0.12
		N. weight:	1.1
		Sc. PD:	0.051
		Sc. rank:	23882.0

PB2

Pos.	631	obs:	exp:
tta	L	0	0.23
ttg	L	0	0.41
ctt	L	0	0.42
ctc	L	0	0.29
cta	L	2	0.31
ctg	L	0	0.34
cat	H	1	0.56
cac	H	0	0.44
caa	Q	39949	24681.00
cag	Q	427	15695.00
cgt	R	0	0.02
cgc	R	0	0.04
cga	R	1	0.09
cgg	R	0	0.09
aaa	K	4	2.63
aag	K	0	1.37
aga	R	0	0.49
agg	R	0	0.28
---	--	------	------
mPD		0.021	0.48
		nPD:	0.04
		N. weight:	0.46
		Sc. PD:	-0.011
		Sc. rank:	2884.4

PB2

Pos.	632	obs:	exp:
tct	S	0	6510.00
tcc	S	0	6028.00
tca	S	0	10843.00
tcg	S	0	1588.00
aat	N	0	20.70
aac	N	37	16.30
agt	S	362	7050.00
agc	S	39979	8322.00
ggt	G	0	0.81
ggc	G	6	0.84
gga	G	0	2.77
ggg	G	0	1.59
---	--	------	------
mPD		0.020	1.6
		nPD:	0.01
		N. weight:	1.6
		Sc. PD:	-0.092
		Sc. rank:	-25327.0

PB2

Pos.	633	obs:	exp:
cgt	R	0	800.20
cgc	R	0	1452.00
cga	R	5	3682.00
cgg	R	0	3466.00
aaa	K	24	15.78
aag	K	0	8.22
aga	R	39746	19796.00
agg	R	609	11164.00
---	--	------	------
mPD		0.031	0.89
		nPD:	0.04
		N. weight:	0.68
		Sc. PD:	-0.023
		Sc. rank:	229.1

PB2

Pos.	634	obs:	exp:
tta	L	0	0.35
ttg	L	0	0.62
ctt	L	0	0.63
ctc	L	0	0.44
cta	L	0	0.46
ctg	L	3	0.50
caa	Q	0	0.61
cag	Q	1	0.39
att	I	1	0.70
atc	I	0	0.53
ata	I	1	0.77
atg	M	40364	40364.00
gtt	V	0	3.51
gtc	V	0	2.85
gta	V	0	3.03
gtg	V	14	4.61
---	--	------	------
mPD		0.0010	0.0016
		nPD:	0.64
		N. weight:	0.0006
		Sc. PD:	0.00034
		Sc. rank:	19.9

PB2

Pos.	635	obs:	exp:
tgg	W	1	1.00
caa	Q	364	24685.00
cag	Q	40019	15698.00
---	--	------	------
mPD		0.018	0.48
		nPD:	0.04
		N. weight:	0.94
		Sc. PD:	-0.03
		Sc. rank:	1474.3

PB2

Pos.	636	obs:	exp:
ttt	F	276	19642.00
ttc	F	40105	20739.00
tta	L	0	0.12
ttg	L	0	0.21
tgt	C	0	0.39
tgc	C	1	0.61
ctt	L	0	0.21
ctc	L	1	0.15
cta	L	0	0.15
ctg	L	0	0.17
gtt	V	1	0.25
gtc	V	0	0.20
gta	V	0	0.22
gtg	V	0	0.33
---	--	------	------
mPD		0.014	0.50
		nPD:	0.03
		N. weight:	0.66
		Sc. PD:	-0.028
		Sc. rank:	-3950.9

PB2

Pos.	637	obs:	exp:
tct	S	39829	6516.00
tcc	S	383	6035.00
tca	S	32	10854.00
tcg	S	138	1590.00
tgt	C	1	0.39
tgc	C	0	0.61
act	T	1	0.31
acc	T	0	0.18
aca	T	0	0.43
acg	T	0	0.08
agt	S	0	7057.00
agc	S	0	8331.00
---	--	------	------
mPD		0.027	1.6
		nPD:	0.02
		N. weight:	1.8
		Sc. PD:	-0.097
		Sc. rank:	-21973.0

,

PB2

Pos.	638	obs:	exp:
tct	S	19	6517.00
tcc	S	8	6035.00
tca	S	40137	10854.00
tcg	S	219	1590.00
caa	Q	0	0.61
cag	Q	1	0.39
agt	S	0	7057.00
agc	S	0	8331.00
---	--	------	------
mPD		0.012	1.6
		nPD:	0.01
		N. weight:	1.4
		Sc. PD:	-0.083
		Sc. rank:	-28715.0

,

PB2

Pos.	639	obs:	exp:
ttt	F	4	1.95
ttc	F	0	2.05
tta	L	11	4685.00
ttg	L	5598	8346.00
ctt	L	325	8445.00
ctc	L	0	5920.00
cta	L	153	6199.00
ctg	L	34283	6775.00
atg	M	9	9.00
aat	N	0	0.56
aac	N	1	0.44
---	--	------	------
mPD		0.26	1.2
		nPD:	0.23
		N. weight:	1.3
		Sc. PD:	0.21
		Sc. rank:	38504.0

,

PB2

Pos.	640	obs:	exp:
tct	S	2	0.48
tcc	S	0	0.45
tca	S	0	0.81
tcg	S	0	0.12
cgt	R	0	0.02
cgc	R	0	0.04
cga	R	0	0.09
cgg	R	0	0.09
att	I	17	5.97
atc	I	0	4.47
ata	I	0	6.57
act	T	40290	12530.00
acc	T	37	7134.00
aca	T	11	17462.00
acg	T	10	3223.00
aat	N	1	0.56
aac	N	0	0.44
agt	S	1	0.52
agc	S	0	0.62
aga	R	1	0.49
agg	R	0	0.28
gct	A	14	3.82
gcc	A	0	3.50
gca	A	0	5.66
gcg	A	0	1.02
---	--	------	------
mPD		0.0047	0.68
		nPD:	0.01
		N. weight:	1.2
		Sc. PD:	-0.076
		Sc. rank:	-27091.0

,

PB2

Pos.	641	obs:	exp:
tta	L	1	0.23
ttg	L	1	0.41
ctt	L	0	0.42
ctc	L	0	0.29
cta	L	0	0.31
ctg	L	0	0.34
att	I	0	0.70
atc	I	0	0.53
ata	I	2	0.77
gtt	V	17	10114.00
gtc	V	1	8228.00
gta	V	6286	8752.00
gtg	V	34076	13286.00
---	--	------	------
mPD		0.26	0.74
		nPD:	0.36
		N. weight:	0.78
		Sc. PD:	0.22
		Sc. rank:	24236.0

,

PB2

Pos.	642	obs:	exp:
tta	L	0	0.12
ttg	L	0	0.21
tct	S	0	0.81
tcc	S	0	0.75
tca	S	0	1.34
tcg	S	0	0.20
ctt	L	1	0.21
ctc	L	0	0.15
cta	L	0	0.15
ctg	L	0	0.17
act	T	3	0.93
acc	T	0	0.53
aca	T	0	1.30
acg	T	0	0.24
aat	N	40316	22587.00
aac	N	57	17786.00
aaa	K	1	0.66
aag	K	0	0.34
agt	S	5	0.87
agc	S	0	1.03
gat	D	1	0.57
gac	D	0	0.43
---	--	------	------
mPD		0.0034	0.49
		nPD:	0.01
		N. weight:	0.6
		Sc. PD:	-0.037
		Sc. rank:	-13318.0

,

PB2

Pos.	643	obs:	exp:
atg	M	2	2.00
gtt	V	31	10115.00
gtc	V	1	8228.00
gta	V	405	8752.00
gtg	V	39945	13287.00
---	--	------	------
mPD		0.022	0.74
		nPD:	0.03
		N. weight:	1.1
		Sc. PD:	-0.045
		Sc. rank:	-5919.9

,

PB2

Pos.	644	obs:	exp:
cgt	R	0	800.70
cgc	R	0	1453.00
cga	R	1	3685.00
cgg	R	0	3468.00
aaa	K	0	0.66
aag	K	1	0.34
aga	R	1936	19807.00
agg	R	38446	11170.00
---	--	------	------
mPD		0.091	0.89
		nPD:	0.1
		N. weight:	1.1
		Sc. PD:	0.037
		Sc. rank:	23531.0

,

PB2

Pos.	645	obs:	exp:
ggt	G	27	5437.00
ggc	G	3	5636.00
gga	G	39585	18642.00
ggg	G	769	10669.00
---	--	------	------
mPD		0.039	0.68
		nPD:	0.06
		N. weight:	0.72
		Sc. PD:	-0.0092
		Sc. rank:	8215.1

,

PB2

Pos.	646	obs:	exp:
tta	L	9	1.04
ttg	L	0	1.86
tct	S	6	6513.00
tcc	S	1	6032.00
tca	S	40331	10849.00
tcg	S	25	1589.00
ctt	L	0	1.88
ctc	L	0	1.32
cta	L	0	1.38
ctg	L	0	1.51
act	T	0	3.11
acc	T	0	1.77
aca	T	10	4.33
acg	T	0	0.80
agt	S	0	7054.00
agc	S	0	8327.00
gtt	V	0	0.25
gtc	V	0	0.20
gta	V	1	0.22
gtg	V	0	0.33
gct	A	0	0.27
gcc	A	0	0.25
gca	A	1	0.40
gcg	A	0	0.07
---	--	------	------
mPD		0.0027	1.6
		nPD:	0.
		N. weight:	1.4
		Sc. PD:	-0.093
		Sc. rank:	-42520.0

,

PB2

Pos.	647	obs:	exp:
tgg	W	1	1.00
gct	A	0	0.27
gcc	A	0	0.25
gca	A	0	0.40
gcg	A	1	0.07
ggt	G	2	5436.00
ggc	G	0	5636.00
gga	G	349	18641.00
ggg	G	40031	10669.00
---	--	------	------
mPD		0.017	0.68
		nPD:	0.03
		N. weight:	1.3
		Sc. PD:	-0.059
		Sc. rank:	-9957.5

,

PB2

Pos.	648	obs:	exp:
tta	L	194	42.12
ttg	L	24	75.05
ctt	L	0	75.94
ctc	L	0	53.23
cta	L	1	55.74
ctg	L	144	60.92
atg	M	40018	40018.00
gtt	V	0	0.75
gtc	V	0	0.61
gta	V	0	0.65
gtg	V	3	0.99
---	--	------	------
mPD		0.028	0.029
		nPD:	0.94
		N. weight:	0.01
		Sc. PD:	0.0089
		Sc. rank:	344.5

,

PB2			
Pos.	649	obs:	exp:
cgt	R	0	800.70
cgc	R	0	1453.00
cga	R	7	3685.00
cgg	R	0	3468.00
aga	R	39917	19808.00
agg	R	460	11170.00
---	--	------	------
mPD		0.023	0.89
		nPD:	0.03
		N. weight:	0.69
		Sc. PD:	-0.03
		Sc. rank:	-4875.8

,

PB2			
Pos.	650	obs:	exp:
cgt	R	0	0.02
cgc	R	0	0.04
cga	R	0	0.09
cgg	R	0	0.09
att	I	3	14164.00
atc	I	26	10602.00
ata	I	40320	15583.00
atg	M	5	5.00
aga	R	1	0.49
agg	R	0	0.28
gtt	V	0	7.26
gtc	V	0	5.91
gta	V	28	6.29
gtg	V	1	9.54
---	--	------	------
mPD		0.0032	0.66
		nPD:	0.
		N. weight:	1.
		Sc. PD:	-0.064
		Sc. rank:	-26009.0

,

PB2			
Pos.	651	obs:	exp:
tta	L	0	4684.00
ttg	L	1	8345.00
ctt	L	39990	8444.00
ctc	L	16	5919.00
cta	L	2	6198.00
ctg	L	354	6774.00
cat	H	1	0.56
cac	H	0	0.44
att	I	16	5.62
atc	I	0	4.20
ata	I	0	6.18
atg	M	4	4.00
---	--	------	------
mPD		0.020	1.2
		nPD:	0.02
		N. weight:	1.6
		Sc. PD:	-0.083
		Sc. rank:	-18741.0

,

PB2			
Pos.	652	obs:	exp:
att	I	0	29.49
atc	I	0	22.07
ata	I	84	32.44
gtt	V	3	10094.00
gtc	V	3	8211.00
gta	V	40216	8734.00
gtg	V	77	13259.00
gaa	E	1	0.59
gag	E	0	0.41
---	--	------	------
mPD		0.0083	0.74
		nPD:	0.01
		N. weight:	1.6
		Sc. PD:	-0.092
		Sc. rank:	-26832.0

,

PB2			
Pos.	653	obs:	exp:
cgt	R	0	800.70
cgc	R	0	1453.00
cga	R	0	3684.00
cgg	R	12	3468.00
act	T	0	0.31
acc	T	0	0.18
aca	T	1	0.43
acg	T	0	0.08
aaa	K	0	0.66
aag	K	1	0.34
aga	R	524	19807.00
agg	R	39846	11170.00
---	--	------	------
mPD		0.026	0.89
		nPD:	0.03
		N. weight:	1.3
		Sc. PD:	-0.05
		Sc. rank:	-5665.9

,

PB2			
Pos.	654	obs:	exp:
ggt	G	58	5437.00
ggc	G	40022	5636.00
gga	G	302	18642.00
ggg	G	2	10669.00
---	--	------	------
mPD		0.018	0.68
		nPD:	0.03
		N. weight:	2.
		Sc. PD:	-0.087
		Sc. rank:	-13823.0

,

PB2			
Pos.	655	obs:	exp:
tct	S	0	0.32
tcc	S	0	0.30
tca	S	0	0.54
tcg	S	0	0.08
aat	N	39950	22592.00
aac	N	431	17789.00
aaa	K	0	0.66
aag	K	1	0.34
agt	S	2	0.35
agc	S	0	0.41
---	--	------	------
mPD		0.021	0.49
		nPD:	0.04
		N. weight:	0.55
		Sc. PD:	-0.015
		Sc. rank:	2946.0

,

PB2			
Pos.	656	obs:	exp:
tct	S	40204	6517.00
tcc	S	125	6035.00
tca	S	51	10854.00
tcg	S	3	1590.00
act	T	1	0.31
acc	T	0	0.18
aca	T	0	0.43
acg	T	0	0.08
agt	S	0	7057.00
agc	S	0	8331.00
---	--	------	------
mPD		0.0089	1.6
		nPD:	0.01
		N. weight:	1.9
		Sc. PD:	-0.12
		Sc. rank:	-47285.0

,

PB2			
Pos.	657	obs:	exp:
tct	S	4	0.65
tcc	S	0	0.60
tca	S	0	1.08
tcg	S	0	0.16
cct	P	39924	13456.00
ccc	P	59	7570.00
cca	P	392	13642.00
ccg	P	4	5711.00
act	T	1	0.31
acc	T	0	0.18
aca	T	0	0.43
acg	T	0	0.08
agt	S	0	0.70
agc	S	0	0.83
---	--	------	------
mPD		0.023	0.72
		nPD:	0.03
		N. weight:	1.1
		Sc. PD:	-0.041
		Sc. rank:	-3739.2

,

PB2			
Pos.	658	obs:	exp:
tta	L	8	0.93
ttg	L	0	1.65
ctt	L	0	1.67
ctc	L	0	1.17
cta	L	0	1.23
ctg	L	0	1.34
att	I	0	6.67
atc	I	0	4.99
ata	I	19	7.34
gtt	V	9	10089.00
gtc	V	46	8207.00
gta	V	39898	8730.00
gtg	V	325	13252.00
gct	A	0	21.26
gcc	A	0	19.49
gca	A	78	31.55
gcg	A	0	5.70
ggt	G	0	0.13
ggc	G	0	0.14
gga	G	1	0.46
ggg	G	0	0.26
---	--	------	------
mPD		0.024	0.75
		nPD:	0.03
		N. weight:	1.5
		Sc. PD:	-0.058
		Sc. rank:	-4599.3

,

PB2			
Pos.	659	obs:	exp:
ttt	F	78	19642.00
ttc	F	40303	20739.00
tct	S	0	0.16
tcc	S	1	0.15
tca	S	0	0.27
tcg	S	0	0.04
tat	Y	0	0.53
tac	Y	1	0.47
atg	M	1	1.00
agt	S	0	0.17
agc	S	0	0.21
---	--	------	------
mPD		0.0041	0.50
		nPD:	0.01
		N. weight:	0.69
		Sc. PD:	-0.042
		Sc. rank:	-13864.0

,

PB2			
Pos.	660	obs:	exp:
tct	S	0	0.81
tcc	S	0	0.75
tca	S	0	1.34
tcg	S	0	0.20
cat	H	0	2.82
cac	H	5	2.18
att	I	0	0.35
atc	I	0	0.26
ata	I	1	0.39
aat	N	1821	22586.00
aac	N	38550	17785.00
aaa	K	2	1.31
aag	K	0	0.69
agt	S	0	0.87
agc	S	5	1.03
---	--	------	------
mPD		0.087	0.49
		nPD:	0.18
		N. weight:	0.66
		Sc. PD:	0.069
		Sc. rank:	17216.0

,

PB2

Pos.	661	obs:	exp:
tat	Y	79	21284.00
tac	Y	40300	19095.00
tgt	C	0	0.78
tgc	C	2	1.22
cat	H	0	1.69
cac	H	3	1.31
---	--	------	------
mPD		0.0042	0.50
		nPD:	0.01
		N. weight:	0.77
		Sc. PD:	-0.047
		Sc. rank:	-15277.0

,

PB2

Pos.	662	obs:	exp:
tct	S	0	2.10
tcc	S	0	1.94
tca	S	0	3.49
tcg	S	0	0.51
aat	N	192	22586.00
aac	N	40179	17785.00
agt	S	0	2.27
agc	S	13	2.68
---	--	------	------
mPD		0.010	0.49
		nPD:	0.02
		N. weight:	0.83
		Sc. PD:	-0.041
		Sc. rank:	-8546.0

,

PB2

Pos.	663	obs:	exp:
cgt	R	0	1.78
cgc	R	0	3.24
cga	R	0	8.21
cgg	R	0	7.73
aaa	K	602	26491.00
aag	K	39692	13803.00
aga	R	1	44.14
agg	R	89	24.89
---	--	------	------
mPD		0.034	0.46
		nPD:	0.07
		N. weight:	1.
		Sc. PD:	0.0043
		Sc. rank:	17357.0

,

PB2

Pos.	664	obs:	exp:
tct	S	0	0.32
tcc	S	2	0.30
tca	S	0	0.54
tcg	S	0	0.08
att	I	1	35.10
atc	I	99	26.28
ata	I	0	38.62
act	T	36	12375.00
acc	T	39728	7045.00
aca	T	84	17245.00
acg	T	0	3183.00
aat	N	0	3.36
aac	N	6	2.64
agt	S	0	0.35
agc	S	0	0.41
gct	A	1	116.70
gcc	A	73	106.90
gca	A	353	173.10
gcg	A	1	31.29
---	--	------	------
mPD		0.050	0.71
		nPD:	0.07
		N. weight:	1.8
		Sc. PD:	0.00062
		Sc. rank:	28731.0

,

PB2

Pos.	665	obs:	exp:
tct	S	1	0.48
tcc	S	0	0.45
tca	S	0	0.81
tcg	S	0	0.12
att	I	2	0.70
atc	I	0	0.53
ata	I	0	0.77
act	T	39807	12535.00
acc	T	514	7136.00
aca	T	6	17468.00
acg	T	36	3224.00
aat	N	10	5.59
aac	N	0	4.41
agt	S	2	0.52
agc	S	0	0.62
gct	A	6	1.64
gcc	A	0	1.50
gca	A	0	2.43
gcg	A	0	0.44
---	--	------	------
mPD		0.028	0.68
		nPD:	0.04
		N. weight:	1.2
		Sc. PD:	-0.033
		Sc. rank:	4575.6

,

PB2

Pos.	666	obs:	exp:
cgt	R	0	0.57
cgc	R	0	1.04
cga	R	0	2.65
cgg	R	0	2.49
aat	N	0	1.12
aac	N	2	0.88
aaa	K	40320	26530.00
aag	K	33	13823.00
aga	R	27	14.22
agg	R	2	8.02
---	--	------	------
mPD		0.0033	0.45
		nPD:	0.01
		N. weight:	0.44
		Sc. PD:	-0.027
		Sc. rank:	-9448.8

,

PB2

Pos.	667	obs:	exp:
cgt	R	0	800.00
cgc	R	0	1452.00
cga	R	209	3682.00
cgg	R	28	3465.00
att	I	0	0.35
atc	I	0	0.26
ata	I	1	0.39
atg	M	2	2.00
aaa	K	1	18.41
aag	K	27	9.59
aga	R	3881	19791.00
agg	R	36232	11161.00
ggt	G	0	0.40
ggc	G	0	0.42
gga	G	0	1.38
ggg	G	3	0.79
---	--	------	------
mPD		0.20	0.89
		nPD:	0.22
		N. weight:	0.93
		Sc. PD:	0.14
		Sc. rank:	26285.0

,

PB2

Pos.	668	obs:	exp:
tta	L	442	4685.00
ttg	L	0	8348.00
ctt	L	360	8446.00
ctc	L	6	5921.00
cta	L	39411	6200.00
ctg	L	158	6776.00
att	I	0	1.76
atc	I	0	1.31
ata	I	5	1.93
gtt	V	0	0.50
gtc	V	0	0.41
gta	V	2	0.43
gtg	V	0	0.66
---	--	------	------
mPD		0.048	1.2
		nPD:	0.04
		N. weight:	1.8
		Sc. PD:	-0.052
		Sc. rank:	6831.2

,

PB2

Pos.	669	obs:	exp:
tct	S	0	0.16
tcc	S	0	0.15
tca	S	1	0.27
tcg	S	0	0.04
att	I	0	5.97
atc	I	0	4.47
ata	I	17	6.57
atg	M	4	4.00
act	T	3	12531.00
acc	T	2	7134.00
aca	T	40143	17463.00
acg	T	202	3223.00
agt	S	0	0.17
agc	S	0	0.21
gct	A	0	3.27
gcc	A	0	3.00
gca	A	12	4.85
gcg	A	0	0.88
---	--	------	------
mPD		0.012	0.68
		nPD:	0.02
		N. weight:	0.86
		Sc. PD:	-0.044
		Sc. rank:	-9793.0

,

PB2

Pos.	670	obs:	exp:
att	I	39759	14044.00
atc	I	238	10512.00
ata	I	10	15451.00
act	T	1	0.31
acc	T	0	0.18
aca	T	0	0.43
acg	T	0	0.08
gtt	V	367	94.18
gtc	V	9	76.61
gta	V	0	81.50
gtg	V	0	123.70
---	--	------	------
mPD		0.031	0.68
		nPD:	0.05
		N. weight:	1.1
		Sc. PD:	-0.025
		Sc. rank:	7117.6

,

PB2

Pos.	671	obs:	exp:
tta	L	0	4685.00
ttg	L	0	8347.00
ctt	L	32835	8445.00
ctc	L	7526	5920.00
cta	L	10	6199.00
ctg	L	1	6776.00
att	I	11	4.21
atc	I	1	3.15
ata	I	0	4.63
---	--	------	------
mPD		0.30	1.2
		nPD:	0.26
		N. weight:	1.2
		Sc. PD:	0.23
		Sc. rank:	35248.0

,

PB2

Pos.	672	obs:	exp:
ggt	G	19	5437.00
ggc	G	0	5636.00
gga	G	39708	18642.00
ggg	G	657	10669.00
---	--	------	------
mPD		0.033	0.68
		nPD:	0.05
		N. weight:	0.73
		Sc. PD:	-0.016
		Sc. rank:	5868.9

,

PB2			
Pos.	673	obs:	exp:
cgt	R	0	0.06
cgc	R	0	0.11
cga	R	0	0.27
cgg	R	0	0.26
act	T	0	0.31
acc	T	0	0.18
aca	T	1	0.43
acg	T	0	0.08
aaa	K	40021	26547.00
aag	K	359	13833.00
aga	R	3	1.47
agg	R	0	0.83
---	--	------	------
mPD		0.018	0.45
		nPD:	0.04
		N. weight:	0.39
		Sc. PD:	-0.012
		Sc. rank:	1183.2

,

PB2			
Pos.	674	obs:	exp:
tat	Y	2	1.05
tac	Y	0	0.95
aat	N	30	16.78
aac	N	0	13.22
gtt	V	3	0.75
gtc	V	0	0.61
gta	V	0	0.65
gtg	V	0	0.99
gat	D	40287	22801.00
gac	D	50	17536.00
gaa	E	7	4.13
gag	E	0	2.87
ggt	G	5	0.67
ggc	G	0	0.70
gga	G	0	2.31
ggg	G	0	1.32
---	--	------	------
mPD		0.0048	0.49
		nPD:	0.01
		N. weight:	0.59
		Sc. PD:	-0.035
		Sc. rank:	-10965.0

,

PB2			
Pos.	675	obs:	exp:
tct	S	0	0.16
tcc	S	1	0.15
tca	S	0	0.27
tcg	S	0	0.04
cct	P	0	1.00
ccc	P	3	0.56
cca	P	0	1.01
ccg	P	0	0.42
act	T	0	0.93
acc	T	3	0.53
aca	T	0	1.30
acg	T	0	0.24
agt	S	0	0.17
agc	S	0	0.21
gct	A	419	11005.00
gcc	A	39524	10088.00
gca	A	433	16332.00
gcg	A	0	2952.00
ggt	G	0	0.13
ggc	G	1	0.14
gga	G	0	0.46
ggg	G	0	0.26
---	--	------	------
mPD		0.042	0.69
		nPD:	0.06
		N. weight:	1.3
		Sc. PD:	-0.013
		Sc. rank:	16043.0

,

PB2			
Pos.	676	obs:	exp:
gtt	V	0	0.75
gtc	V	3	0.61
gta	V	0	0.65
gtg	V	0	0.99
ggt	G	476	5436.00
ggc	G	39857	5636.00
gga	G	47	18641.00
ggg	G	1	10668.00
---	--	------	------
mPD		0.026	0.68
		nPD:	0.04
		N. weight:	2.
		Sc. PD:	-0.063
		Sc. rank:	3758.1

,

PB2			
Pos.	677	obs:	exp:
tct	S	155	25.01
tcc	S	0	23.16
tca	S	0	41.66
tcg	S	0	6.10
att	I	69	24.22
atc	I	0	18.13
ata	I	0	26.65
act	T	39675	12340.00
acc	T	49	7026.00
aca	T	9	17197.00
acg	T	4	3174.00
aat	N	9	5.04
aac	N	0	3.96
agt	S	0	27.09
agc	S	0	31.98
gct	A	55	112.80
gcc	A	0	103.40
gca	A	359	167.50
gcg	A	0	30.26
---	--	------	------
mPD		0.052	0.71
		nPD:	0.07
		N. weight:	1.2
		Sc. PD:	0.0044
		Sc. rank:	20232.0

,

PB2			
Pos.	678	obs:	exp:
tta	L	38671	4686.00
ttg	L	1544	8349.00
ctt	L	0	8448.00
ctc	L	0	5922.00
cta	L	168	6201.00
ctg	L	0	6777.00
att	I	0	0.35
atc	I	0	0.26
ata	I	1	0.39
---	--	------	------
mPD		0.082	1.2
		nPD:	0.07
		N. weight:	2.
		Sc. PD:	0.0013
		Sc. rank:	33289.0

,

PB2			
Pos.	679	obs:	exp:
tta	L	0	0.35
ttg	L	0	0.62
tct	S	1	0.97
tcc	S	0	0.90
tca	S	0	1.61
tcg	S	0	0.24
ctt	L	3	0.63
ctc	L	0	0.44
cta	L	0	0.46
ctg	L	0	0.50
att	I	39868	14020.00
atc	I	47	10494.00
ata	I	24	15424.00
atg	M	1	1.00
act	T	396	124.80
acc	T	6	71.07
aca	T	0	174.00
acg	T	0	32.11
aat	N	5	2.80
aac	N	0	2.20
agt	S	5	1.05
agc	S	0	1.24
gtt	V	26	6.51
gtc	V	0	5.30
gta	V	0	5.64
gtg	V	0	8.55
gct	A	2	0.55
gcc	A	0	0.50
gca	A	0	0.81
gcg	A	0	0.15
---	--	------	------
mPD		0.026	0.68
		nPD:	0.04
		N. weight:	1.1
		Sc. PD:	-0.035
		Sc. rank:	1861.6

,

PB2			
Pos.	680	obs:	exp:
aaa	K	4	2.63
aag	K	0	1.37
gtt	V	0	0.25
gtc	V	0	0.20
gta	V	1	0.22
gtg	V	0	0.33
gct	A	0	0.55
gcc	A	0	0.50
gca	A	2	0.81
gcg	A	0	0.15
gat	D	3	77.44
gac	D	134	59.56
gaa	E	40173	23723.00
gag	E	65	16515.00
ggt	G	0	0.27
ggc	G	0	0.28
gga	G	2	0.92
ggg	G	0	0.53
---	--	------	------
mPD		0.010	0.49
		nPD:	0.02
		N. weight:	0.55
		Sc. PD:	-0.026
		Sc. rank:	-5325.7

,

PB2

Pos.	681	obs:	exp:
tat	Y	1	1.58
tac	Y	2	1.42
gat	D	498	22824.00
gac	D	39880	17554.00
gaa	E	2	1.18
gag	E	0	0.82
---	-	1	1.00
---	--	------	------
mPD		0.025	0.49
		nPD:	0.05
		N. weight:	0.8
		Sc. PD:	-0.016
		Sc. rank:	7131.0

PB2

Pos.	682	obs:	exp:
tta	L	0	0.12
ttg	L	0	0.21
tct	S	0	0.32
tcc	S	0	0.30
tca	S	2	0.54
tcg	S	0	0.08
ctt	L	0	0.21
ctc	L	0	0.15
cta	L	1	0.15
ctg	L	0	0.17
cct	P	4	13456.00
ccc	P	1	7570.00
cca	P	40324	13642.00
ccg	P	50	5711.00
caa	Q	1	0.61
cag	Q	0	0.39
act	T	0	0.31
acc	T	0	0.18
aca	T	1	0.43
acg	T	0	0.08
agt	S	0	0.35
agc	S	0	0.41
---	--	------	------
mPD		0.0030	0.72
		nPD:	0.
		N. weight:	1.1
		Sc. PD:	-0.074
		Sc. rank:	-31264.0

PB2

Pos.	683	obs:	exp:
att	I	1	0.35
atc	I	0	0.26
ata	I	0	0.39
aat	N	21	11.75
aac	N	0	9.25
gct	A	1	0.27
gcc	A	0	0.25
gca	A	0	0.40
gcg	A	0	0.07
gat	D	40102	22772.00
gac	D	184	17514.00
gaa	E	3	40.68
gag	E	66	28.32
ggt	G	6	0.81
ggc	G	0	0.84
gga	G	0	2.77
ggg	G	0	1.59
---	--	------	------
mPD		0.014	0.49
		nPD:	0.03
		N. weight:	0.57
		Sc. PD:	-0.024
		Sc. rank:	-3321.6

PB2

Pos.	684	obs:	exp:
aaa	K	1	0.66
aag	K	0	0.34
gct	A	0	0.27
gcc	A	0	0.25
gca	A	1	0.40
gcg	A	0	0.07
gaa	E	40256	23807.00
gag	E	124	16573.00
ggt	G	0	0.27
ggc	G	0	0.28
gga	G	2	0.92
ggg	G	0	0.53
---	--	------	------
mPD		0.0063	0.48
		nPD:	0.01
		N. weight:	0.54
		Sc. PD:	-0.03
		Sc. rank:	-8008.8

PB2

Pos.	685	obs:	exp:
tct	S	0	6439.00
tcc	S	0	5963.00
tca	S	0	10725.00
tcg	S	0	1571.00
tgt	C	0	0.78
tgc	C	2	1.22
cgt	R	0	0.02
cgc	R	0	0.04
cga	R	0	0.09
cgg	R	0	0.09
att	I	0	1.05
atc	I	3	0.79
ata	I	0	1.16
aat	N	0	41.96
aac	N	75	33.04
agt	S	66	6973.00
agc	S	39837	8232.00
aga	R	1	0.49
agg	R	0	0.28
ggt	G	25	53.85
ggc	G	375	55.83
gga	G	0	184.60
ggg	G	0	105.70
---	--	------	------
mPD		0.028	1.6
		nPD:	0.02
		N. weight:	1.7
		Sc. PD:	-0.086
		Sc. rank:	-19063.0

PB2

Pos.	686	obs:	exp:
tct	S	0	0.32
tcc	S	0	0.30
tca	S	2	0.54
tcg	S	0	0.08
att	I	0	3.16
atc	I	0	2.36
ata	I	9	3.48
act	T	8	12520.00
acc	T	949	7128.00
aca	T	39336	17448.00
acg	T	23	3220.00
aat	N	0	0.56
aac	N	1	0.44
aaa	K	27	17.75
aag	K	0	9.25
agt	S	0	0.35
agc	S	0	0.41
gct	A	0	7.90
gcc	A	0	7.25
gca	A	29	11.73
gcg	A	0	2.12
---	--	------	------
mPD		0.051	0.68
		nPD:	0.07
		N. weight:	0.78
		Sc. PD:	0.0034
		Sc. rank:	13156.0

PB2

Pos.	687	obs:	exp:
tct	S	358	6490.00
tcc	S	39795	6010.00
tca	S	66	10810.00
tcg	S	0	1583.00
tat	Y	0	1.05
tac	Y	2	0.95
cct	P	0	0.33
ccc	P	1	0.19
cca	P	0	0.34
ccg	P	0	0.14
act	T	0	2.48
acc	T	8	1.41
aca	T	0	3.46
acg	T	0	0.64
agt	S	0	7028.00
agc	S	0	8297.00
gct	A	150	41.97
gcc	A	4	38.48
gca	A	0	62.29
gcg	A	0	11.26
---	--	------	------
mPD		0.036	1.6
		nPD:	0.02
		N. weight:	1.9
		Sc. PD:	-0.091
		Sc. rank:	-18066.0

PB2

Pos.	688	obs:	exp:
cgt	R	0	0.20
cgc	R	0	0.36
cga	R	0	0.91
cgg	R	0	0.86
aga	R	10	4.90
agg	R	0	2.77
gtt	V	0	1.75
gtc	V	0	1.43
gta	V	7	1.52
gtg	V	0	2.30
gaa	E	12	7.07
gag	E	0	4.93
ggt	G	17	5433.00
ggc	G	0	5632.00
gga	G	40044	18629.00
ggg	G	294	10661.00
---	--	------	------
mPD		0.017	0.68
		nPD:	0.02
		N. weight:	0.77
		Sc. PD:	-0.034
		Sc. rank:	-6172.7

PB2

Pos.	689	obs:	exp:
tta	L	0	2.90
ttg	L	25	5.17
ctt	L	0	5.23
ctc	L	0	3.67
cta	L	0	3.84
ctg	L	0	4.20
atg	M	10	10.00
gtt	V	9	10106.00
gtc	V	0	8221.00
gta	V	87	8745.00
gtg	V	40251	13275.00
gct	A	0	0.27
gcc	A	0	0.25
gca	A	0	0.40
gcg	A	1	0.07
gaa	E	0	0.59
gag	E	1	0.41
---	--	------	------
mPD		0.0066	0.74
		nPD:	0.01
		N. weight:	1.2
		Sc. PD:	-0.07
		Sc. rank:	-22212.0

PB2

Pos.	690	obs:	exp:
gaa	E	787	23809.00
gag	E	39597	16575.00
---	--	------	------
mPD		0.038	0.48
		nPD:	0.08
		N. weight:	0.83
		Sc. PD:	0.0073
		Sc. rank:	14902.0

PB2

Pos.	691	obs:	exp:
tct	S	953	6517.00
tcc	S	39304	6035.00
tca	S	122	10854.00
tcg	S	4	1590.00
agt	S	0	7057.00
agc	S	0	8331.00
gct	A	0	0.27
gcc	A	1	0.25
gca	A	0	0.40
gcg	A	0	0.07
---	--	------	------
mPD		0.052	1.6
		nPD:	0.03
		N. weight:	1.9
		Sc. PD:	-0.07
		Sc. rank:	-5782.2

PB2

Pos.	692	obs:	exp:
gct	A	1067	11007.00
gcc	A	39208	10090.00
gca	A	109	16336.00
gcg	A	0	2952.00
---	--	------	------
mPD		0.057	0.69
		nPD:	0.08
		N. weight:	1.3
		Sc. PD:	0.015
		Sc. rank:	23911.0

PB2			
Pos.	693	obs:	exp:
att	I	0	2.11
atc	I	6	1.58
ata	I	0	2.32
gtt	V	505	10114.00
gtc	V	39635	8227.00
gta	V	237	8751.00
gtg	V	0	13285.00
gct	A	0	0.27
gcc	A	1	0.25
gca	A	0	0.40
gcg	A	0	0.07
---	--	------	------
mPD		0.037	0.74
		nPD:	0.05
		N. weight:	1.6
		Sc. PD:	-0.032
		Sc. rank:	13348.0

,

PB2			
Pos.	694	obs:	exp:
tta	L	312	4686.00
ttg	L	36190	8349.00
ctt	L	0	8448.00
ctc	L	0	5922.00
cta	L	5	6201.00
ctg	L	3877	6778.00
---	--	------	------
mPD		0.19	1.2
		nPD:	0.16
		N. weight:	1.3
		Sc. PD:	0.12
		Sc. rank:	32675.0

,

PB2			
Pos.	695	obs:	exp:
cgt	R	0	0.02
cgc	R	0	0.04
cga	R	0	0.09
cgg	R	0	0.09
aga	R	1	0.49
agg	R	0	0.28
---	-	40383	40383.00
---	--	------	------
mPD		0	5.4×10^{-10}
		nPD:	0.
		N. weight:	0.
		Sc. PD:	0
		Sc. rank:	0

,

PB2			
Pos.	696	obs:	exp:
cgt	R	0	800.70
cgc	R	0	1453.00
cga	R	1	3684.00
cgg	R	0	3467.00
att	I	0	0.35
atc	I	0	0.26
ata	I	1	0.39
aaa	K	2	1.31
aag	K	0	0.69
aga	R	39787	19806.00
agg	R	593	11169.00
---	--	------	------
mPD		0.029	0.89
		nPD:	0.03
		N. weight:	0.68
		Sc. PD:	-0.025
		Sc. rank:	-1298.0

,

PB2			
Pos.	697	obs:	exp:
act	T	0	0.31
acc	T	0	0.18
aca	T	0	0.43
acg	T	1	0.08
ggt	G	55	5437.00
ggc	G	10	5636.00
gga	G	1264	18641.00
ggg	G	39054	10669.00
---	--	------	------
mPD		0.064	0.68
		nPD:	0.09
		N. weight:	1.2
		Sc. PD:	0.029
		Sc. rank:	24492.0

,

PB2			
Pos.	698	obs:	exp:
ttt	F	2551	19642.00
ttc	F	37830	20739.00
tat	Y	0	1.58
tac	Y	3	1.42
---	--	------	------
mPD		0.12	0.50
		nPD:	0.24
		N. weight:	0.46
		Sc. PD:	0.075
		Sc. rank:	13239.0

,

PB2			
Pos.	699	obs:	exp:
tta	L	0	4686.00
ttg	L	0	8349.00
ctt	L	179	8448.00
ctc	L	40191	5922.00
cta	L	12	6201.00
ctg	L	0	6777.00
cat	H	0	1.13
cac	H	2	0.87
---	--	------	------
mPD		0.0095	1.2
		nPD:	0.01
		N. weight:	2.
		Sc. PD:	-0.12
		Sc. rank:	-39576.0

,

PB2			
Pos.	700	obs:	exp:
att	I	40292	14176.00
atc	I	85	10611.00
ata	I	6	15596.00
gtt	V	1	0.25
gtc	V	0	0.20
gta	V	0	0.22
gtg	V	0	0.33
---	--	------	------
mPD		0.0045	0.66
		nPD:	0.01
		N. weight:	1.1
		Sc. PD:	-0.068
		Sc. rank:	-24232.0

,

PB2			
Pos.	701	obs:	exp:
tta	L	238	74.96
ttg	L	157	133.60
ctt	L	0	135.10
ctc	L	83	94.73
cta	L	138	99.20
ctg	L	30	108.40
att	I	15	13943.00
atc	I	18	10437.00
ata	I	39687	15340.00
atg	M	12	12.00
gtt	V	0	1.25
gtc	V	0	1.02
gta	V	5	1.08
gtg	V	0	1.65
---	-	1	1.00
---	--	------	------
mPD		0.047	0.69
		nPD:	0.07
		N. weight:	0.99
		Sc. PD:	-0.0018
		Sc. rank:	15263.0

,

PB2			
Pos.	702	obs:	exp:
tct	S	0	0.97
tcc	S	0	0.90
tca	S	0	1.61
tcg	S	0	0.24
agt	S	6	1.05
agc	S	0	1.24
gat	D	2	1.13
gac	D	0	0.87
ggt	G	40026	5435.00
ggc	G	330	5635.00
gga	G	11	18638.00
ggg	G	8	10667.00
---	-	1	1.00
---	--	------	------
mPD		0.018	0.68
		nPD:	0.03
		N. weight:	2.1
		Sc. PD:	-0.09
		Sc. rank:	-14652.0

,

PB2			
Pos.	703	obs:	exp:
caa	Q	1	0.61
cag	Q	0	0.39
cgt	R	0	0.85
cgc	R	0	1.55
cga	R	0	3.92
cgg	R	0	3.69
atg	M	1	1.00
aat	N	1	0.56
aac	N	0	0.44
aaa	K	27988	26520.00
aag	K	12350	13818.00
aga	R	36	21.09
agg	R	7	11.89
---	--	------	------
mPD		0.43	0.45
		nPD:	0.94
		N. weight:	0.0038
		Sc. PD:	0.0032
		Sc. rank:	125.0

,

PB2			
Pos.	704	obs:	exp:
aaa	K	2	1.31
aag	K	0	0.69
gat	D	1	0.57
gac	D	0	0.43
gaa	E	39638	23807.00
gag	E	743	16574.00
---	--	------	------
mPD		0.036	0.48
		nPD:	0.07
		N. weight:	0.47
		Sc. PD:	0.0023
		Sc. rank:	8055.6

,

PB2			
Pos.	705	obs:	exp:
aat	N	7	16.78
aac	N	23	13.22
gat	D	149	22808.00
gac	D	40201	17542.00
gaa	E	2	2.36
gag	E	2	1.64
---	--	------	------
mPD		0.0094	0.49
		nPD:	0.02
		N. weight:	0.85
		Sc. PD:	-0.043
		Sc. rank:	-8962.4

,

PB2			
Pos.	706	obs:	exp:
tct	S	0	1.29
tcc	S	0	1.20
tca	S	0	2.15
tcg	S	0	0.31
cgt	R	0	791.60
cgc	R	0	1437.00
cga	R	1	3643.00
cgg	R	0	3428.00
att	I	0	0.35
atc	I	0	0.26
ata	I	1	0.39
aaa	K	68	293.90
aag	K	379	153.10
agt	S	2	1.40
agc	S	6	1.65
aga	R	39791	19583.00
agg	R	133	11043.00
ggt	G	0	0.40
ggc	G	0	0.42
gga	G	3	1.38
ggg	G	0	0.79
---	--	------	------
mPD		0.048	0.90
		nPD:	0.05
		N. weight:	0.73
		Sc. PD:	-0.013
		Sc. rank:	6746.8

,

PB2			
Pos.	707	obs:	exp:
cgt	R	0	800.50
cgc	R	0	1453.00
cga	R	17	3684.00
cgg	R	0	3467.00
aaa	K	11	7.23
aag	K	0	3.77
aga	R	38575	19802.00
agg	R	1781	11167.00
---	--	------	------
mPD		0.086	0.89
		nPD:	0.1
		N. weight:	0.58
		Sc. PD:	0.015
		Sc. rank:	11878.0

,

PB2			
Pos.	708	obs:	exp:
ttt	F	0	19.46
ttc	F	40	20.54
tct	S	0	0.16
tcc	S	1	0.15
tca	S	0	0.27
tcg	S	0	0.04
tat	Y	730	21263.00
tac	Y	39609	19076.00
tgt	C	0	0.39
tgc	C	1	0.61
cat	H	0	1.13
cac	H	2	0.87
aat	N	0	0.56
aac	N	1	0.44
agt	S	0	0.17
agc	S	0	0.21
---	--	------	------
mPD		0.038	0.50
		nPD:	0.08
		N. weight:	0.69
		Sc. PD:	0.0036
		Sc. rank:	11970.0

,

PB2

Pos.	709	obs:	exp:
ggt	G	30	5437.00
ggc	G	363	5636.00
gga	G	38226	18642.00
ggg	G	1765	10669.00
---	--	------	------
mPD		0.10	0.68
		nPD:	0.15
		N. weight:	0.6
		Sc. PD:	0.048
		Sc. rank:	14825.0

,

PB2

Pos.	710	obs:	exp:
tct	S	0	0.48
tcc	S	0	0.45
tca	S	3	0.81
tcg	S	0	0.12
cct	P	272	13456.00
ccc	P	2	7570.00
cca	P	40074	13643.00
ccg	P	33	5711.00
agt	S	0	0.52
agc	S	0	0.62
---	--	------	------
mPD		0.015	0.72
		nPD:	0.02
		N. weight:	1.1
		Sc. PD:	-0.053
		Sc. rank:	-10734.0

,

PB2

Pos.	711	obs:	exp:
tct	S	0	2.58
tcc	S	0	2.39
tca	S	16	4.30
tcg	S	0	0.63
act	T	0	0.62
acc	T	0	0.35
aca	T	2	0.87
acg	T	0	0.16
agt	S	0	2.80
agc	S	0	3.30
gtt	V	0	0.25
gtc	V	0	0.20
gta	V	1	0.22
gtg	V	0	0.33
gct	A	2	11002.00
gcc	A	10	10085.00
gca	A	40333	16328.00
gcg	A	20	2951.00
---	--	------	------
mPD		0.0025	0.70
		nPD:	0.
		N. weight:	0.95
		Sc. PD:	-0.062
		Sc. rank:	-26564.0

,

PB2

Pos.	712	obs:	exp:
tta	L	40226	4686.00
ttg	L	59	8349.00
ctt	L	0	8448.00
ctc	L	0	5922.00
cta	L	97	6201.00
ctg	L	0	6777.00
att	I	0	0.70
atc	I	0	0.53
ata	I	2	0.77
---	--	------	------
mPD		0.0078	1.2
		nPD:	0.01
		N. weight:	2.2
		Sc. PD:	-0.14
		Sc. rank:	-50143.0

,

PB2

Pos.	713	obs:	exp:
tct	S	0	6515.00
tcc	S	0	6034.00
tca	S	0	10852.00
tcg	S	0	1589.00
tgt	C	0	1.95
tgc	C	5	3.05
aat	N	0	2.24
aac	N	4	1.76
agt	S	106	7056.00
agc	S	40269	8329.00
---	--	------	------
mPD		0.0057	1.6
		nPD:	0.
		N. weight:	1.6
		Sc. PD:	-0.11
		Sc. rank:	-46232.0

,

PB2

Pos.	714	obs:	exp:
att	I	41	14176.00
atc	I	40256	10611.00
ata	I	85	15595.00
gtt	V	0	0.50
gtc	V	2	0.41
gta	V	0	0.43
gtg	V	0	0.66
---	--	------	------
mPD		0.0063	0.66
		nPD:	0.01
		N. weight:	1.4
		Sc. PD:	-0.082
		Sc. rank:	-25697.0

,

PB2

Pos.	715	obs:	exp:
tct	S	0	2.90
tcc	S	0	2.69
tca	S	0	4.84
tcg	S	0	0.71
att	I	1	0.35
atc	I	0	0.26
ata	I	0	0.39
act	T	14	4.35
acc	T	0	2.48
aca	T	0	6.06
acg	T	0	1.12
aat	N	40027	22566.00
aac	N	308	17769.00
agt	S	18	3.15
agc	S	0	3.71
gat	D	15	8.48
gac	D	0	6.52
---	-	1	1.00
---	--	------	------
mPD		0.018	0.50
		nPD:	0.04
		N. weight:	0.57
		Sc. PD:	-0.019
		Sc. rank:	293.6

,

PB2

Pos.	716	obs:	exp:
gaa	E	40346	23809.00
gag	E	38	16575.00
---	--	------	------
mPD		0.0019	0.48
		nPD:	0.
		N. weight:	0.55
		Sc. PD:	-0.036
		Sc. rank:	-15281.0

,

PB2

Pos.	717	obs:	exp:
tta	L	0	4685.00
ttg	L	450	8348.00
ctt	L	15	8447.00
ctc	L	0	5921.00
cta	L	251	6200.00
ctg	L	39662	6777.00
atg	M	6	6.00
---	--	------	------
mPD		0.035	1.2
		nPD:	0.03
		N. weight:	1.8
		Sc. PD:	-0.069
		Sc. rank:	-7299.4

,

PB2

Pos.	718	obs:	exp:
tct	S	0	6510.00
tcc	S	0	6029.00
tca	S	0	10843.00
tcg	S	0	1588.00
tgt	C	1	0.39
tgc	C	0	0.61
cgt	R	0	0.02
cgc	R	1	0.04
cga	R	0	0.09
cgg	R	0	0.09
att	I	1	0.35
atc	I	0	0.26
ata	I	0	0.39
act	T	1	0.31
acc	T	0	0.18
aca	T	0	0.43
acg	T	0	0.08
aat	N	14	7.83
aac	N	0	6.17
agt	S	39074	7050.00
agc	S	1268	8322.00
aga	R	0	0.49
agg	R	0	0.28
ggt	G	21	3.23
ggc	G	3	3.35
gga	G	0	11.08
ggg	G	0	6.34
---	--	------	------
mPD		0.063	1.6
		nPD:	0.04
		N. weight:	1.7
		Sc. PD:	-0.052
		Sc. rank:	3734.3

,

PB2

Pos.	719	obs:	exp:
tct	S	0	1.29
tcc	S	0	1.20
tca	S	0	2.15
tcg	S	0	0.31
cat	H	0	1.13
cac	H	2	0.87
aat	N	487	22586.00
aac	N	39883	17784.00
aaa	K	4	2.63
aag	K	0	1.37
agt	S	2	1.40
agc	S	6	1.65
---	--	------	------
mPD		0.025	0.49
		nPD:	0.05
		N. weight:	0.79
		Sc. PD:	-0.016
		Sc. rank:	6929.9

,

PB2

Pos.	720	obs:	exp:
tta	L	0	4686.00
ttg	L	0	8349.00
ctt	L	40102	8448.00
ctc	L	281	5922.00
cta	L	0	6201.00
ctg	L	0	6777.00
att	I	1	0.35
atc	I	0	0.26
ata	I	0	0.39
---	--	------	------
mPD		0.014	1.2
		nPD:	0.01
		N. weight:	1.6
		Sc. PD:	-0.092
		Sc. rank:	-25908.0

,

PB2			
Pos.	721	obs:	exp:
tct	S	0	5.65
tcc	S	0	5.23
tca	S	35	9.41
tcg	S	0	1.38
cct	P	0	0.33
ccc	P	0	0.19
cca	P	1	0.34
ccg	P	0	0.14
act	T	16	36.33
acc	T	0	20.69
aca	T	101	50.63
acg	T	0	9.34
agt	S	0	6.12
agc	S	0	7.22
gtt	V	5	3.26
gtc	V	0	2.65
gta	V	8	2.82
gtg	V	0	4.28
gct	A	5341	10961.00
gcc	A	19	10048.00
gca	A	34790	16268.00
gcg	A	67	2940.00
---	-	1	1.00
---	--	------	------
mPD		0.24	0.70
		nPD:	0.34
		N. weight:	0.58
		Sc. PD:	0.16
		Sc. rank:	18047.0

,

PB2			
Pos.	722	obs:	exp:
cgt	R	0	2.70
cgc	R	0	4.89
cga	R	0	12.41
cgg	R	0	11.68
aaa	K	36459	26460.00
aag	K	3789	13788.00
aga	R	19	66.71
agg	R	117	37.62
---	--	------	------
mPD		0.18	0.46
		nPD:	0.4
		N. weight:	0.18
		Sc. PD:	0.058
		Sc. rank:	5666.9

,

PB2			
Pos.	723	obs:	exp:
tgg	W	1	1.00
cgt	R	0	0.02
cgc	R	0	0.04
cga	R	0	0.09
cgg	R	0	0.09
aga	R	0	0.49
agg	R	1	0.28
ggt	G	10	5436.00
ggc	G	1	5636.00
gga	G	1468	18641.00
ggg	G	38903	10669.00
---	--	------	------
mPD		0.071	0.68
		nPD:	0.1
		N. weight:	1.2
		Sc. PD:	0.041
		Sc. rank:	25585.0

,

PB2			
Pos.	724	obs:	exp:
gaa	E	39855	23809.00
gag	E	529	16575.00
---	--	------	------
mPD		0.026	0.48
		nPD:	0.05
		N. weight:	0.49
		Sc. PD:	-0.008
		Sc. rank:	4800.5

,

PB2			
Pos.	725	obs:	exp:
cgt	R	0	0.28
cgc	R	0	0.50
cga	R	0	1.28
cgg	R	0	1.20
aaa	K	567	26541.00
aag	K	39803	13829.00
aga	R	0	6.87
agg	R	14	3.87
---	--	------	------
mPD		0.028	0.45
		nPD:	0.06
		N. weight:	1.
		Sc. PD:	-0.0073
		Sc. rank:	13637.0

,

PB2			
Pos.	726	obs:	exp:
act	T	19	5.90
acc	T	0	3.36
aca	T	0	8.22
acg	T	0	1.52
gtt	V	6	1.50
gtc	V	0	1.22
gta	V	0	1.30
gtg	V	0	1.97
gct	A	40295	11000.00
gcc	A	37	10083.00
gca	A	22	16325.00
gcg	A	4	2950.00
ggt	G	1	0.13
ggc	G	0	0.14
gga	G	0	0.46
ggg	G	0	0.26
---	--	------	------
mPD		0.0044	0.70
		nPD:	0.01
		N. weight:	1.4
		Sc. PD:	-0.085
		Sc. rank:	-31960.0

,

PB2			
Pos.	727	obs:	exp:
aat	N	40325	22592.00
aac	N	56	17789.00
aaa	K	1	0.66
aag	K	0	0.34
---	-	2	2.00
---	--	------	------
mPD		0.0028	0.49
		nPD:	0.01
		N. weight:	0.6
		Sc. PD:	-0.038
		Sc. rank:	-14710.0

,

PB2			
Pos.	728	obs:	exp:
gtt	V	4848	10115.00
gtc	V	1	8229.00
gta	V	217	8753.00
gtg	V	35318	13287.00
---	--	------	------
mPD		0.22	0.74
		nPD:	0.3
		N. weight:	0.79
		Sc. PD:	0.18
		Sc. rank:	23722.0

,

PB2			
Pos.	729	obs:	exp:
tta	L	449	4686.00
ttg	L	0	8348.00
ctt	L	2	8447.00
ctc	L	4	5921.00
cta	L	39887	6200.00
ctg	L	37	6777.00
cct	P	0	0.33
ccc	P	0	0.19
cca	P	1	0.34
ccg	P	0	0.14
caa	Q	2	1.22
cag	Q	0	0.78
att	I	0	0.35
atc	I	0	0.26
ata	I	1	0.39
gtt	V	0	0.25
gtc	V	0	0.20
gta	V	1	0.22
gtg	V	0	0.33
---	--	------	------
mPD		0.024	1.2
		nPD:	0.02
		N. weight:	1.9
		Sc. PD:	-0.092
		Sc. rank:	-19079.0

,

PB2			
Pos.	730	obs:	exp:
att	I	36178	14176.00
atc	I	3585	10611.00
ata	I	620	15596.00
atg	M	1	1.00
---	--	------	------
mPD		0.19	0.66
		nPD:	0.29
		N. weight:	0.73
		Sc. PD:	0.16
		Sc. rank:	21850.0

,

PB2			
Pos.	731	obs:	exp:
ggt	G	7	5437.00
ggc	G	3	5636.00
gga	G	499	18642.00
ggg	G	39875	10669.00
---	--	------	------
mPD		0.025	0.68
		nPD:	0.04
		N. weight:	1.3
		Sc. PD:	-0.044
		Sc. rank:	1144.1

,

PB2			
Pos.	732	obs:	exp:
caa	Q	40149	24686.00
cag	Q	235	15698.00
---	--	------	------
mPD		0.012	0.48
		nPD:	0.02
		N. weight:	0.48
		Sc. PD:	-0.022
		Sc. rank:	-3938.9

,

PB2			
Pos.	733	obs:	exp:
ggt	G	4	5437.00
ggc	G	1	5636.00
gga	G	40088	18642.00
ggg	G	291	10669.00
---	--	------	------
mPD		0.015	0.68
		nPD:	0.02
		N. weight:	0.77
		Sc. PD:	-0.037
		Sc. rank:	-7490.4

,

PB2			
Pos.	734	obs:	exp:
aat	N	0	0.56
aac	N	1	0.44
gct	A	0	0.27
gcc	A	1	0.25
gca	A	0	0.40
gcg	A	0	0.07
gat	D	565	22826.00
gac	D	39816	17555.00
ggt	G	0	0.13
ggc	G	1	0.14
gga	G	0	0.46
ggg	G	0	0.26
---	--	------	------
mPD		0.028	0.49
		nPD:	0.06
		N. weight:	0.8
		Sc. PD:	-0.011
		Sc. rank:	8791.5

,

PB2			
Pos.	735	obs:	exp:
tta	L	0	0.12
ttg	L	1	0.21
ctt	L	0	0.21
ctc	L	0	0.15
cta	L	0	0.15
ctg	L	0	0.17
att	I	0	79.69
atc	I	0	59.65
ata	I	227	87.67
atg	M	3	3.00
gtt	V	115	10057.00
gtc	V	1	8181.00
gta	V	581	8703.00
gtg	V	39456	13211.00
---	--	------	------
mPD		0.056	0.75
		nPD:	0.07
		N. weight:	1.1
		Sc. PD:	0.005
		Sc. rank:	18243.0

,

PB2			
Pos.	736	obs:	exp:
atg	M	3	3.00
gtt	V	2	10114.00
gtc	V	1	8228.00
gta	V	108	8752.00
gtg	V	40269	13286.00
gct	A	0	0.27
gcc	A	0	0.25
gca	A	0	0.40
gcg	A	1	0.07
---	--	------	------
mPD		0.0057	0.74
		nPD:	0.01
		N. weight:	1.2
		Sc. PD:	-0.071
		Sc. rank:	-24153.0

,

PB2			
Pos.	737	obs:	exp:
tta	L	33	4686.00
ttg	L	40334	8348.00
tct	S	0	0.32
tcc	S	0	0.30
tca	S	0	0.54
tcg	S	2	0.08
ctt	L	0	8447.00
ctc	L	0	5921.00
cta	L	1	6201.00
ctg	L	12	6777.00
agt	S	0	0.35
agc	S	0	0.41
gtt	V	0	0.25
gtc	V	0	0.20
gta	V	0	0.22
gtg	V	1	0.33
---	-	1	1.00
---	--	------	------
mPD		0.0025	1.2
		nPD:	0.
		N. weight:	1.7
		Sc. PD:	-0.11
		Sc. rank:	-49621.0

,

PB2			
Pos.	738	obs:	exp:
att	I	0	3.51
atc	I	0	2.63
ata	I	10	3.86
gtt	V	7	10113.00
gtc	V	15	8226.00
gta	V	40088	8751.00
gtg	V	263	13284.00
gct	A	0	0.27
gcc	A	0	0.25
gca	A	1	0.40
gcg	A	0	0.07
---	--	------	------
mPD		0.015	0.74
		nPD:	0.02
		N. weight:	1.6
		Sc. PD:	-0.077
		Sc. rank:	-16170.0

,

PB2			
Pos.	739	obs:	exp:
atg	M	40384	40384.00
---	--	------	------
mPD		0	0
		nPD:	1.
		N. weight:	0.
		Sc. PD:	0
		Sc. rank:	0

,

PB2			
Pos.	740	obs:	exp:
aaa	K	40383	26550.00
aag	K	1	13834.00
---	--	------	------
mPD		0.000050	0.45
		nPD:	0.
		N. weight:	0.44
		Sc. PD:	-0.03
		Sc. rank:	-14336.0

,

PB2			
Pos.	741	obs:	exp:
caa	Q	1	0.61
cag	Q	0	0.39
cgt	R	6	800.70
cgc	R	0	1453.00
cga	R	23058	3685.00
cgg	R	17318	3468.00
aga	R	1	19807.00
agg	R	0	11170.00
---	--	------	------
mPD		0.49	0.89
		nPD:	0.55
		N. weight:	1.8
		Sc. PD:	0.87
		Sc. rank:	59940.0

,

PB2			
Pos.	742	obs:	exp:
cgt	R	0	0.06
cgc	R	0	0.11
cga	R	0	0.27
cgg	R	0	0.26
aaa	K	40374	26548.00
aag	K	7	13833.00
aga	R	3	1.47
agg	R	0	0.83
---	--	------	------
mPD		0.00050	0.45
		nPD:	0.
		N. weight:	0.44
		Sc. PD:	-0.03
		Sc. rank:	-13748.0

,

PB2			
Pos.	743	obs:	exp:
cgt	R	0	800.70
cgc	R	0	1453.00
cga	R	216	3685.00
cgg	R	40099	3468.00
aga	R	1	19808.00
agg	R	68	11170.00
---	--	------	------
mPD		0.014	0.89
		nPD:	0.02
		N. weight:	2.5
		Sc. PD:	-0.14
		Sc. rank:	-32249.0

,

PB2			
Pos.	744	obs:	exp:
tat	Y	0	0.53
tac	Y	1	0.47
aat	N	0	88.96
aac	N	159	70.04
gat	D	1	22735.00
gac	D	40220	17486.00
gaa	E	2	1.18
gag	E	0	0.82
ggt	G	0	0.13
ggc	G	1	0.14
gga	G	0	0.46
ggg	G	0	0.26
---	--	------	------
mPD		0.0081	0.50
		nPD:	0.02
		N. weight:	0.88
		Sc. PD:	-0.047
		Sc. rank:	-10863.0

,

PB2			
Pos.	745	obs:	exp:
ttt	F	1	0.49
ttc	F	0	0.51
tct	S	40290	6517.00
tcc	S	87	6035.00
tca	S	5	10854.00
tcg	S	1	1590.00
agt	S	0	7057.00
agc	S	0	8331.00
---	--	------	------
mPD		0.0046	1.6
		nPD:	0.
		N. weight:	1.9
		Sc. PD:	-0.13
		Sc. rank:	-55005.0

,

PB2			
Pos.	746	obs:	exp:
tct	S	0	6517.00
tcc	S	0	6035.00
tca	S	0	10854.00
tcg	S	0	1590.00
agt	S	36281	7057.00
agc	S	4102	8331.00
ggt	G	1	0.13
ggc	G	0	0.14
gga	G	0	0.46
ggg	G	0	0.26
---	--	------	------
mPD		0.18	1.6
		nPD:	0.11
		N. weight:	1.5
		Sc. PD:	0.061
		Sc. rank:	32153.0

,

PB2			
Pos.	747	obs:	exp:
att	I	0	14175.00
atc	I	0	10611.00
ata	I	40381	15595.00
act	T	0	0.31
acc	T	0	0.18
aca	T	1	0.43
acg	T	0	0.08
aaa	K	1	0.66
aag	K	0	0.34
gtt	V	0	0.25
gtc	V	0	0.20
gta	V	1	0.22
gtg	V	0	0.33
---	--	------	------
mPD		0.00015	0.66
		nPD:	0.
		N. weight:	1.
		Sc. PD:	-0.069
		Sc. rank:	-32328.0

,

PB2			
Pos.	748	obs:	exp:
tta	L	0	4686.00
ttg	L	0	8349.00
ctt	L	40383	8448.00
ctc	L	1	5922.00
cta	L	0	6201.00
ctg	L	0	6778.00
---	--	------	------
mPD		0.000050	1.2
		nPD:	0.
		N. weight:	1.6
		Sc. PD:	-0.11
		Sc. rank:	-54162.0

,

PB2			
Pos.	749	obs:	exp:
act	T	40311	12541.00
acc	T	73	7140.00
aca	T	0	17477.00
acg	T	0	3225.00
---	--	------	------
mPD		0.0036	0.68
		nPD:	0.01
		N. weight:	1.2
		Sc. PD:	-0.078
		Sc. rank:	-31001.0

PB2			
Pos.	750	obs:	exp:
gat	D	947	22828.00
gac	D	39437	17556.00
---	--	------	------
mPD		0.046	0.49
		nPD:	0.09
		N. weight:	0.75
		Sc. PD:	0.017
		Sc. rank:	14992.0

PB2			
Pos.	751	obs:	exp:
tct	S	0	6517.00
tcc	S	0	6035.00
tca	S	0	10854.00
tcg	S	0	1590.00
agt	S	12	7057.00
agc	S	40372	8331.00
---	--	------	------
mPD		0.00059	1.6
		nPD:	0.
		N. weight:	1.7
		Sc. PD:	-0.11
		Sc. rank:	-53306.0

PB2			
Pos.	752	obs:	exp:
cct	P	0	0.33
ccc	P	0	0.19
cca	P	0	0.34
ccg	P	1	0.14
caa	Q	0	24685.00
cag	Q	40382	15697.00
cgt	R	0	0.02
cgc	R	0	0.04
cga	R	0	0.09
cgg	R	1	0.09
aga	R	0	0.49
agg	R	0	0.28
---	--	------	------
mPD		0.000099	0.48
		nPD:	0.
		N. weight:	1.
		Sc. PD:	-0.068
		Sc. rank:	-32195.0

PB2			
Pos.	753	obs:	exp:
act	T	0	12541.00
acc	T	1	7140.00
aca	T	40383	17477.00
acg	T	0	3225.00
---	--	------	------
mPD		0.000050	0.68
		nPD:	0.
		N. weight:	0.88
		Sc. PD:	-0.061
		Sc. rank:	-28775.0

PB2			
Pos.	754	obs:	exp:
gct	A	0	11007.00
gcc	A	0	10090.00
gca	A	0	16336.00
gcg	A	40384	2952.00
---	--	------	------
mPD		0	0.69
		nPD:	0.
		N. weight:	2.8
		Sc. PD:	-0.19
		Sc. rank:	-90970.0

PB2			
Pos.	755	obs:	exp:
act	T	0	12541.00
acc	T	40383	7140.00
aca	T	1	17477.00
acg	T	0	3225.00
---	--	------	------
mPD		0.000050	0.68
		nPD:	0.
		N. weight:	1.8
		Sc. PD:	-0.13
		Sc. rank:	-59539.0

PB2			
Pos.	756	obs:	exp:
aaa	K	40383	26550.00
aag	K	1	13834.00
---	--	------	------
mPD		0.000050	0.45
		nPD:	0.
		N. weight:	0.44
		Sc. PD:	-0.03
		Sc. rank:	-14336.0

PB2			
Pos.	757	obs:	exp:
cgt	R	0	800.70
cgc	R	0	1453.00
cga	R	404	3685.00
cgg	R	1	3468.00
att	I	0	0.35
atc	I	0	0.26
ata	I	1	0.39
aga	R	39684	19807.00
agg	R	294	11170.00
---	--	------	------
mPD		0.034	0.89
		nPD:	0.04
		N. weight:	0.67
		Sc. PD:	-0.021
		Sc. rank:	1519.9

PB2			
Pos.	758	obs:	exp:
ttt	F	3	1.46
ttc	F	0	1.54
tta	L	0	0.58
ttg	L	0	1.03
ctt	L	5	1.05
ctc	L	0	0.73
cta	L	0	0.77
ctg	L	0	0.84
att	I	40365	14171.00
atc	I	4	10607.00
ata	I	0	15590.00
gtt	V	7	1.75
gtc	V	0	1.43
gta	V	0	1.52
gtg	V	0	2.30
---	--	------	------
mPD		0.00094	0.66
		nPD:	0.
		N. weight:	1.1
		Sc. PD:	-0.074
		Sc. rank:	-34125.0

PB2			
Pos.	759	obs:	exp:
caa	Q	0	0.61
cag	Q	1	0.39
cgt	R	0	800.70
cgc	R	0	1453.00
cga	R	34	3685.00
cgg	R	40349	3468.00
aga	R	0	19807.00
agg	R	0	11170.00
---	--	------	------
mPD		0.0017	0.89
		nPD:	0.
		N. weight:	2.6
		Sc. PD:	-0.17
		Sc. rank:	-78045.0

PB2			
Pos.	760	obs:	exp:
tta	L	0	0.12
ttg	L	1	0.21
ctt	L	0	0.21
ctc	L	0	0.15
cta	L	0	0.15
ctg	L	0	0.17
atg	M	40362	40362.00
act	T	0	0.62
acc	T	0	0.35
aca	T	0	0.87
acg	T	2	0.16
gtt	V	0	4.76
gtc	V	0	3.87
gta	V	0	4.12
gtg	V	19	6.25
---	--	------	------
mPD		0.0011	0.0018
		nPD:	0.59
		N. weight:	0.00072
		Sc. PD:	0.00037
		Sc. rank:	23.7

PB2			
Pos.	761	obs:	exp:
cct	P	0	0.33
ccc	P	1	0.19
cca	P	0	0.34
ccg	P	0	0.14
gct	A	0	11007.00
gcc	A	40383	10089.00
gca	A	0	16335.00
gcg	A	0	2952.00
---	--	------	------
mPD		0.000050	0.69
		nPD:	0.
		N. weight:	1.5
		Sc. PD:	-0.1
		Sc. rank:	-47837.0

PB2			
Pos.	762	obs:	exp:
att	I	16	14173.00
atc	I	40358	10609.00
ata	I	0	15592.00
act	T	0	3.11
acc	T	10	1.77
aca	T	0	4.33
acg	T	0	0.80
---	--	------	------
mPD		0.0013	0.66
		nPD:	0.
		N. weight:	1.4
		Sc. PD:	-0.094
		Sc. rank:	-42812.0

PB2			
Pos.	763	obs:	exp:
act	T	1	0.31
acc	T	0	0.18
aca	T	0	0.43
acg	T	0	0.08
aat	N	40271	22534.00
aac	N	6	17743.00
aaa	K	2	69.03
aag	K	103	35.97
gat	D	1	0.57
gac	D	0	0.43
---	--	------	------
mPD		0.0056	0.50
		nPD:	0.01
		N. weight:	0.61
		Sc. PD:	-0.035
		Sc. rank:	-10269.0

PB2			
Pos.	764	obs:	exp:
taa	.	39844	22941.00
tag	.	528	4423.00
tga	.	12	13020.00
---	--	------	------
mPD		0.026	0.63
		nPD:	0.04
		N. weight:	0.54
		Sc. PD:	-0.015
		Sc. rank:	2374.9

}

Amino acid level conservation per codon

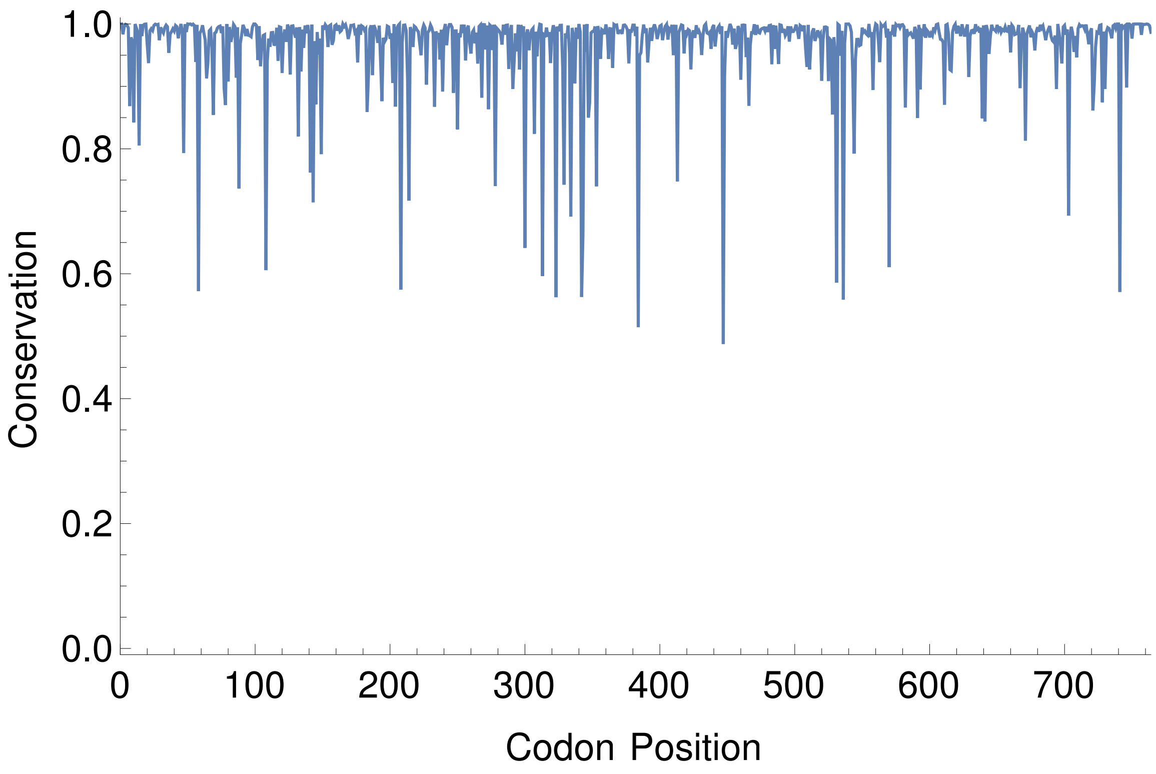

Conservation analysis plots

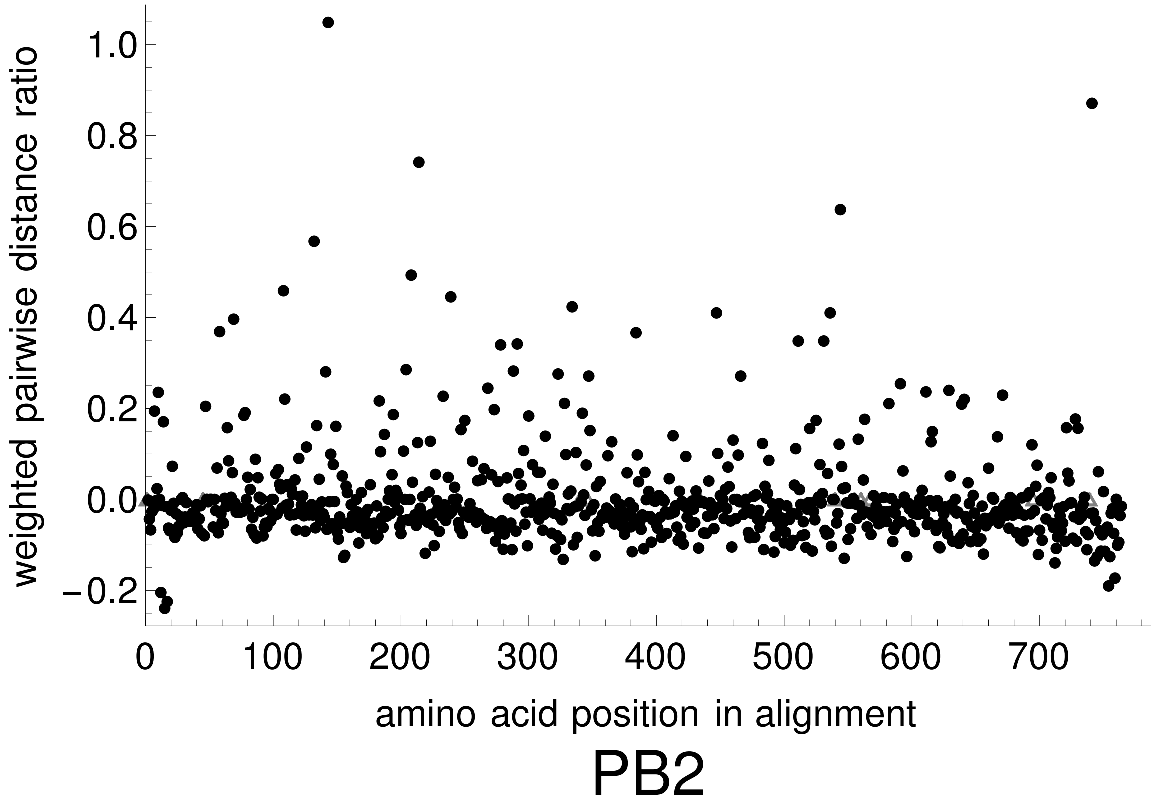

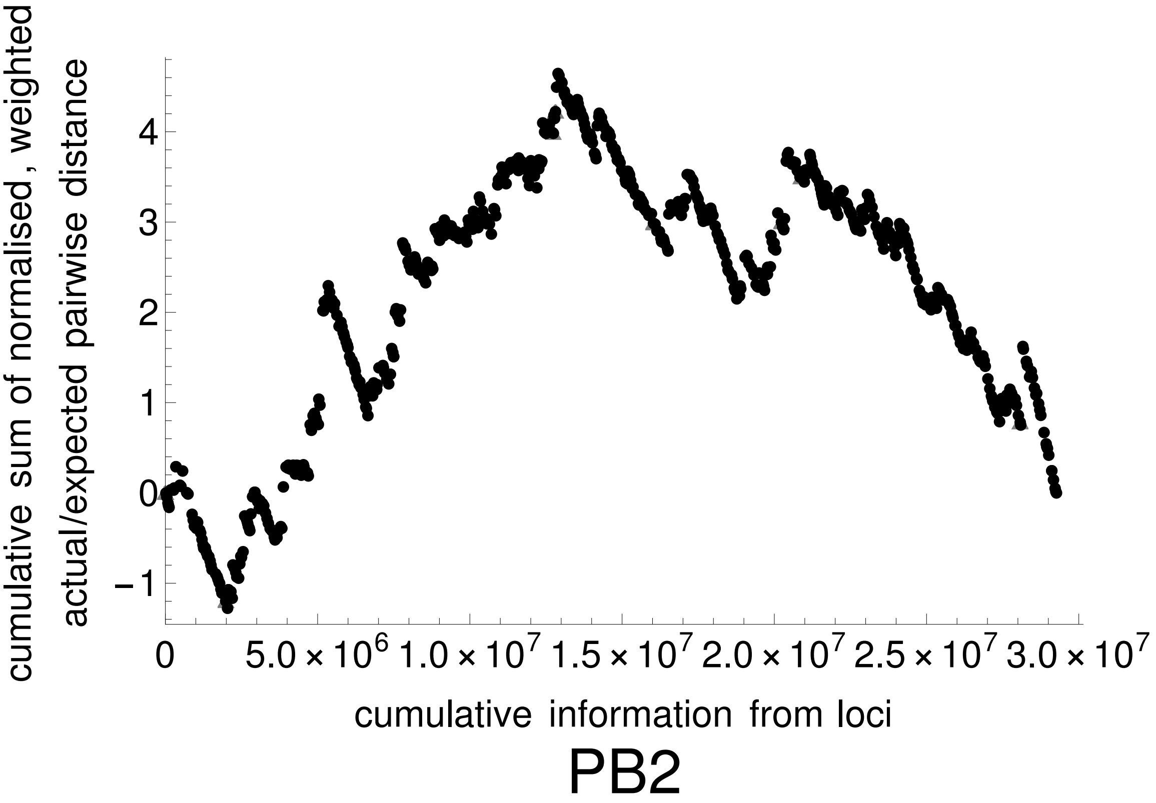

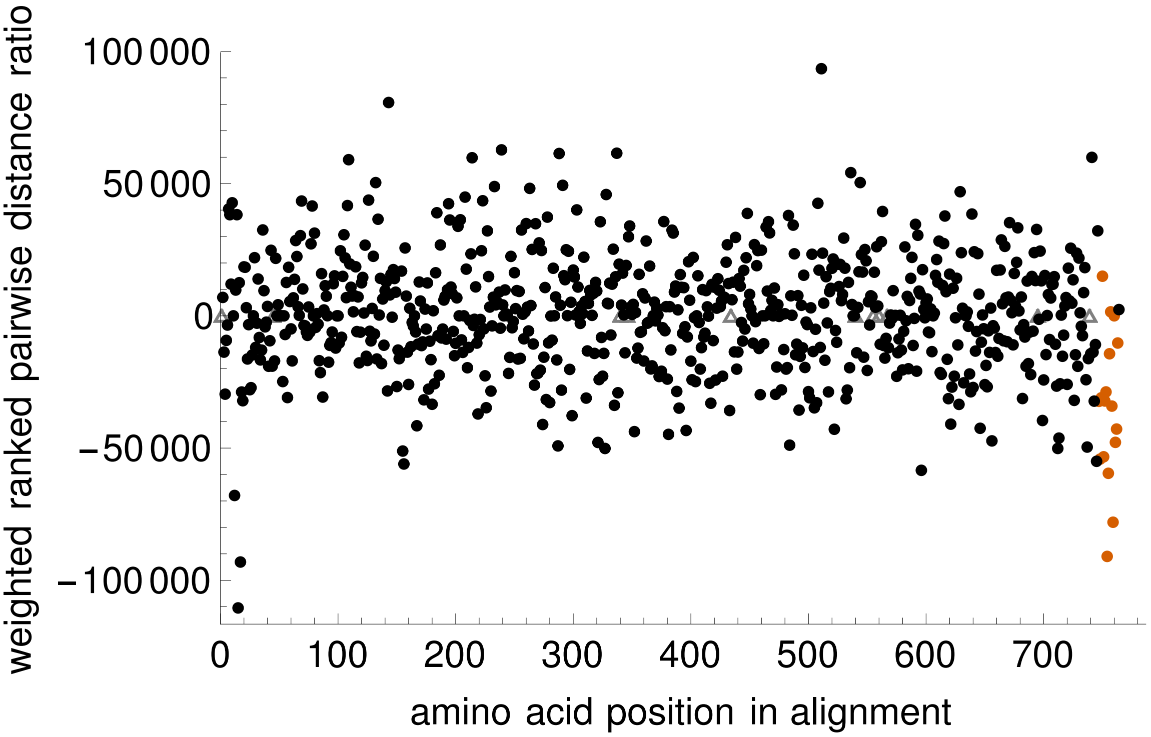
weighted ranked pairwise distance ratio
100 000
50 000
0
−50 000
−100 000
0
100
200
300
400
500
600
700
amino acid position in alignment

PB2

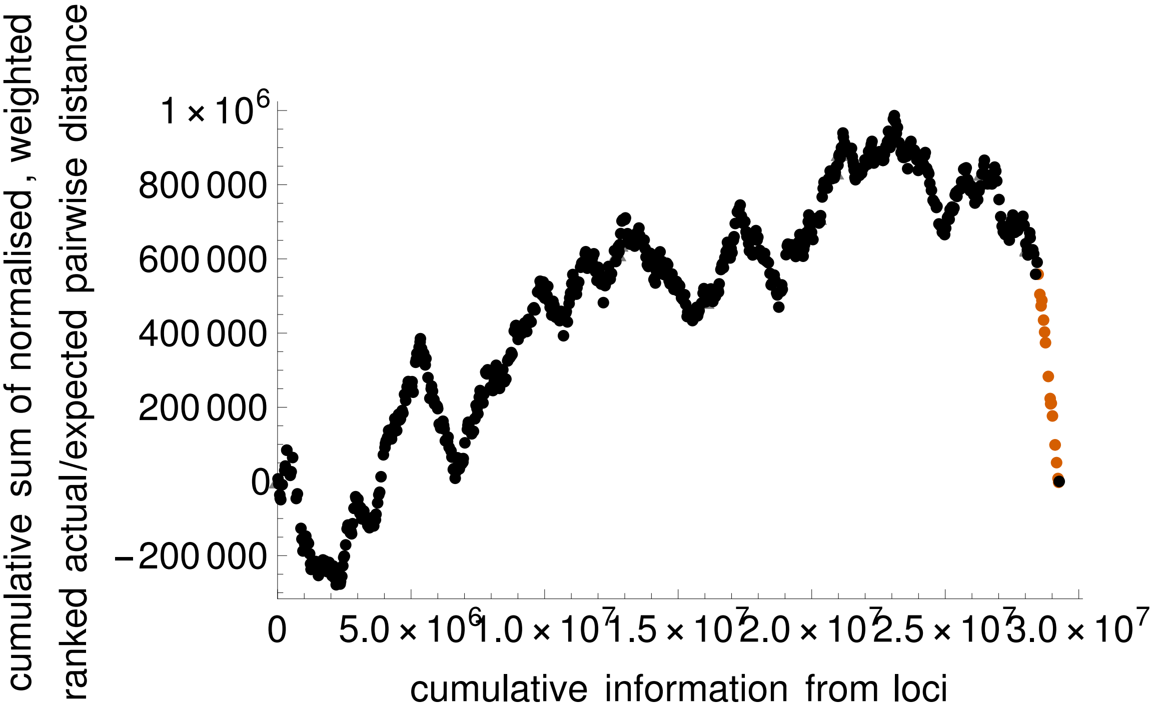
cumulative sum of normalised, weighted ranked actual/expected pairwise distance
1 × 10^6
800 000
600 000
400 000
200 000
0
−200 000
0
5.0 × 10^6
1.0 × 10^7
1.5 × 10^7
2.0 × 10^7
2.5 × 10^7
3.0 × 10^7
cumulative information from loci

PB2

PB1

Gene length histogram

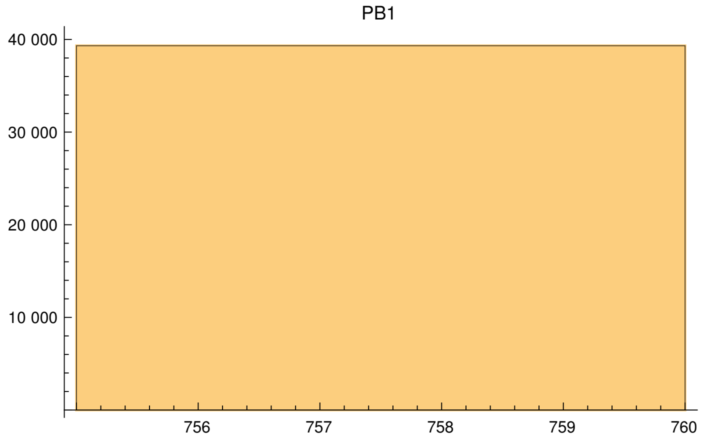

Information vs. nPD

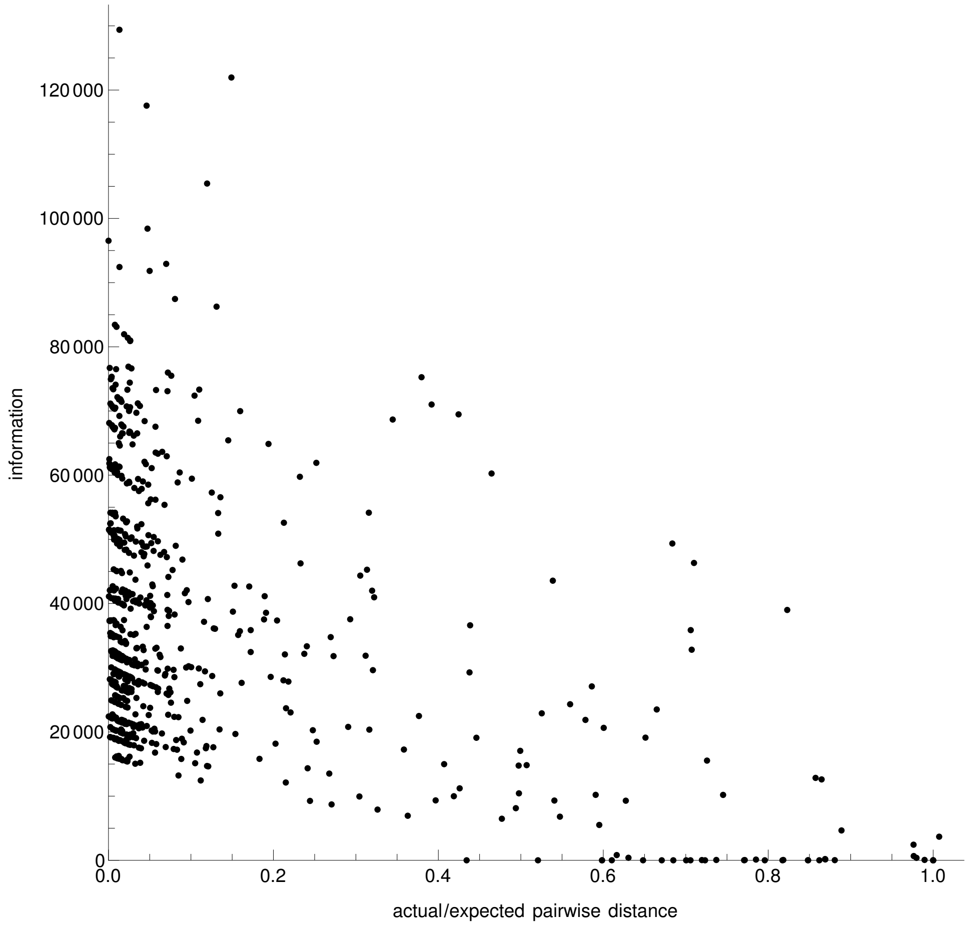

PB1

Example sequences highlighted by regions found in analyses to be conserved

```
Interesting points (by weighted raw PD) highlighted for gene PB1:
ATGGATGTCAATCCGACTCTACTGTTCTTAAAAGTTCCAGCGCAAAATGCCATAAGCACAACATTCCCTTATACTG⋱
    GAGATCCTCCATACAGCCATGGAACAGGGACAGGGTACACTATGGACACAGTCAACAGAACACACCAATACTCAG⋱
    AGAGGGGGAAGTGGACGACAAATACAGAAACTGGGGCTCCCCAGCTCAACCCAATTGATGGACCACTACCTGAGG⋱
    ATAATGAACCAAGTGGATATGCACAAACAGACTGTGTCCTGGAGGCTATGGCCTTCCTTGAAGAATCCCATCCAG⋱
    GTATCTTTGAAAACTCCTGCCTTGAAACAATGGAAGCCGTTCAACAGACAAGGGTAGACAAACTAACCCAAGGTC⋱
    GCCAGACTTATGATTGGACATTAAACAGGAACCAACCGGCAGCAACTGCATTAGCCAACACCATAGAAGTCTTTA⋱
    GATCGAACGGATTAACAGCTAATGAATCAGGAAGGCTAATAGATTTCCTCAAAGATGTGATGGAATCAATGGATA⋱
    AGGAGGAAATGGAGATAACAACACACTTTCAAAGAAAAAGGAGAGTAAGGGACAACATGACCAAGAAAATGGTCA⋱
    CACAAAGAACAATAGGGAAGAAAAAACAAAGAGTGAATAAGAGAGGCTACCTAATAAGAGCTCTGACATTAAACA⋱
    CGATGACCAAGGATGCAGAGAGAGGCAAATTAAAAAGAAGGGCTATTGCAACACCCGGGATGCAAATTAGAGGGT⋱
    TCGTGTACTTCGTTGAAACTTTAGCTAGAAGCATTTGCGAAAAGCTTGAACAATCTGGACTTCCGGTTGGGGGTA⋱
    ATGAAAAGAAGGCCAAACTGGCAAATGTTGTGAGAAAAATGATGACTAATTCACAAGACACAGAGCTTTCTTTCA⋱
    CAATCACTGGAGACAACACTAAGTGGAATGAAAATCAAAACCCCCGAATGTTTTTGGCAATGATTACATACATCA⋱
    CAAAGAACCAACCTGAATGGTTCAGAAACATACTGAGCATAGCACCAATAATGTTCTCAAATAAAATGGCAAAAC⋱
    TGGGAAAAGGATACATGTTCGAGAGTAAGAGAATGAAGCTCCGGACACAAATACCTGCAGAAATGCTAGCAAGCA⋱
    TTGACCTGAAGTATTTCAACGAATCAACAAGGAAGAAAATTGAGAAAATAAGGCCTCTTCTAATAGATGGAACAG⋱
    CATCATTGAGCCCTGGAATGATGATGGGCATGTTCAACATGCTAAGTACAGTTTTAGGAGTCTCGATACTGAATC⋱
    TTGGACAAAAGAAATACACCAAGACAACGTACTGGTGGGATGGGCTCCAATCCTCAGACGATTTTGCCCTCATAG⋱
    TGAATGCACCAAATCATGAGGGGATACAAGCAGGAGTGGATAGATTCTATAGGACCTGCAAGTTAGTGGGAATCA⋱
    ACATGAGCAAAAAGAAGTCCTATATAAATAAAACAGGGACATTTGAATTCACTAGCTTCTTTTATCGATATGGAT⋱
    TTGTGGCTAATTTTAGCATGGAGCTGCCAAGTTTTGGAGTGTCAGGAATAAACGAGTCAGCTGACATGAGCATTG⋱
    GAGTAACAGTGATAAAGAACAACATGATAAATAATGAC ------CTTGGACCAGCAACAGCCCAAATGGCTCTCCA⋱
    ATTGTTCATCAAAGATTACAGATACACGTATCGGTGCCATAGAGGAGACACGCAAATCCAAACGAGAAGATCATT⋱
    CGAGATAAAGAAGCTATGGGATCAAACCCAATCAAGGACAGGATTATTGGTATCAGATGGGGGACCAAACTTATA⋱
    CAATATCCGGAATCTTCACATCCCTGAAGTCTGCTTAAAGTGGGAGCTGATGGATGATAATTATCGGGGAAGACT⋱
    TTGTAATCCCCTGAATCCCTTTGTCAGCCATAAAGAAATTGAATCTGTAAACAATGCTGTAGTAATGCCAGCCCA⋱
    TGGCCCTGCCAAAAGTATGGAATATGATGCCGTTGCAACTACACACTCCTGGATTCCCAAAAGGAACCGCTCTAT⋱
    TCTAAACACAAGCCAAAGGGGAATTCTTGAGGATGAACAAATGTACCAGAAGTGCTGCACCTTGTTCGAGAAATT⋱
    TTTCCCTAGTAGTTCATATAGGAGACCGATTGGAATTTCTAGCATGGTGGAGGCCATGGTATCTAGGGCCCGGAT⋱
    TGATGCCAGAATTGACTTCGAGTCTGGAAGGATTAAGAAAGAAGAGTTCTCTGAGATCATGAAGATCTGTTCCAC⋱
    CATTGAAGAACTCAGACGGCAA ---AAA---TAA
```

```
Interesting points (by weighted ranked PD) highlighted for gene PB1:
ATGGATGTCAATCCGACTCTACTGTTCTTAAAAGTTCCAGCGCAAAATGCCATAAGCACAACATTCCCTTATACTGGA⋱
    GATCCTCCATACAGCCATGGAACAGGGACAGGGTACACTATGGACACAGTCAACAGAACACACCAATACTCAGAGAG⋱
    GGGGAAGTGGACGACAAATACAGAAACTGGGGCTCCCCAGCTCAACCCAATTGATGGACCACTACCTGAGGATAATG⋱
    AACCAAGTGGATATGCACAAACAGACTGTGTCCTGGAGGCTATGGCCTTCCTTGAAGAATCCCATCCAGGTATCTTT⋱
    GAAAACTCCTGCCTTGAAACAATGGAAGCCGTTCAACAGACAAGGGTAGACAAACTAACCCAAGGTCGCCAGACTTA⋱
    TGATTGGACATTAAACAGGAACCAACCGGCAGCAACTGCATTAGCCAACACCATAGAAGTCTTTAGATCGAACGGAT⋱
    TAACAGCTAATGAATCAGGAAGGCTAATAGATTTCCTCAAAGATGTGATGGAATCAATGGATAAGGAGGAAATGGAG⋱
    ATAACAACACACTTTCAAAGAAAAAGGAGAGTAAGGGACAACATGACCAAGAAAATGGTCACACAAAGAACAATAGG⋱
    GAAGAAAAAACAAAGAGTGAATAAGAGAGGCTACCTAATAAGAGCTCTGACATTAAACACGATGACCAAGGATGCAG⋱
    AGAGAGGCAAATTAAAAAGAAGGGCTATTGCAACACCCGGGATGCAAATTAGAGGGTTCGTGTACTTCGTTGAAACT⋱
```

TTAGCTAGAAGCATTTGCGAAAAGCTTGAACAATCTGGACTTCCGGTTGGGGGTAATGAAAAGAAGGCCAAACTGGC⋱
AAATGTTGTGAGAAAAATGATGACTAATTCACAAGACACAGAGCTTTCTTTCACAATCACTGGAGACAACACTAAGT⋱
GGAATGAAAATCAAAACCCCCGAATGTTTTTGGCAATGATTACATACATCACAAAGAACCAACCTGAATGGTTCAGA⋱
AACATACTGAGCATAGCACCAATAATGTTCTCAAATAAAATGGCAAAACTGGGAAAAGGATACATGTTCGAGAGTAA⋱
GAGAATGAAGCTCCGGACACAAATACCTGCAGAAATGCTAGCAAGCATTGACCTGAAGTATTTCAACGAATCAACAA⋱
GGAAGAAAATTGAGAAAATAAGGCCTCTTCTAATAGATGGAACAGCATCATTGAGCCCTGGAATGATGATGGGCATG⋱
TTCAACATGCTAAGTACAGTTTTAGGAGTCTCGATACTGAATCTTGGACAAAAGAAATACACCAAGACAACGTACTG⋱
GTGGGATGGGCTCCAATCCTCAGACGATTTTGCCCTCATAGTGAATGCACCAAATCATGAGGGGATACAAGCAGGAG⋱
TGGATAGATTCTATAGGACCTGCAAGTTAGTGGGAATCAACATGAGCAAAAAGAAGTCCTATATAAATAAAACAGGG⋱
ACATTTGAATTCACTAGCTTCTTTTATCGATATGGATTTGTGGCTAATTTTAGCATGGAGCTGCCAAGTTTTGGAGT⋱
GTCAGGAATAAACGAGTCAGCTGACATGAGCATTGGAGTAACAGTGATAAAGAACAACATGATAAATAATGAC ----·
TTGGACCAGCAACAGCCCAAATGGCTCTCCAATTGTTCATCAAAGATTACAGATACACGTATCGGTGCCATAGAG⋱
GAGACACGCAAATCCAAACGAGAAGATCATTCGAGATAAAGAAGCTATGGGATCAAACCCAATCAAGGACAGGAT⋱
TATTGGTATCAGATGGGGGACCAAACTTATACAATATCCGGAATCTTCACATCCCTGAAGTCTGCTTAAAGTGGG⋱
AGCTGATGGATGATAATTATCGGGGAAGACTTTGTAATCCCCTGAATCCCTTTGTCAGCCATAAAGAAATTGAAT⋱
CTGTAAACAATGCTGTAGTAATGCCAGCCCATGGCCCTGCCAAAAGTATGGAATATGATGCCGTTGCAACTACAC⋱
ACTCCTGGATTCCCAAAAGGAACCGCTCTATTCTAAACACAAGCCAAAGGGGAATTCTTGAGGATGAACAAATGT⋱
ACCAGAAGTGCTGCACCTTGTTCGAGAAATTTTTCCCTAGTAGTTCATATAGGAGACCGATTGGAATTTCTAGCA⋱
TGGTGGAGGCCATGGTATCTAGGGCCCGGATTGATGCCAGAATTGACTTCGAGTCTGGAAGGATTAAGAAAGAAG⋱
AGTTC
TCTGAGATCATGAAGATCTGTTCCACCATTGAAGAACTCAGACGGCAA---AAA---TAA

Per codon conservation report

PB1

Pos.	1	obs:	exp:
atg	M	39322	39322.00
---	--	------	------
mPD		0	0
		nPD:	1.
		N. weight:	0.
		Sc. PD:	0
		Sc. rank:	0

PB1

Pos.	2	obs:	exp:
aat	N	1	0.56
aac	N	0	0.44
gat	D	39319	22227.00
gac	D	2	17094.00
---	--	------	------
mPD		0.00015	0.49
		nPD:	0.
		N. weight:	0.63
		Sc. PD:	-0.038
		Sc. rank:	-19271.0

PB1

Pos.	3	obs:	exp:
att	I	0	2.46
atc	I	7	1.84
ata	I	0	2.70
gtt	V	5	9847.00
gtc	V	39308	8010.00
gta	V	0	8521.00
gtg	V	0	12935.00
gct	A	0	0.55
gcc	A	1	0.50
gca	A	1	0.81
gcg	A	0	0.15
---	--	------	------
mPD		0.00076	0.74
		nPD:	0.
		N. weight:	1.7
		Sc. PD:	-0.1
		Sc. rank:	-52205.0

PB1

Pos.	4	obs:	exp:
tct	S	0	0.16
tcc	S	0	0.15
tca	S	0	0.27
tcg	S	0	0.04
att	I	1	0.35
atc	I	0	0.26
ata	I	0	0.39
aat	N	39133	21996.00
aac	N	183	17320.00
aaa	K	2	1.97
aag	K	1	1.03
agt	S	1	0.17
agc	S	0	0.21
gat	D	1	0.57
gac	D	0	0.43
---	--	------	------
mPD		0.0096	0.49
		nPD:	0.02
		N. weight:	0.61
		Sc. PD:	-0.025
		Sc. rank:	-1781.1

PB1

Pos.	5	obs:	exp:
cct	P	17	13103.00
ccc	P	2	7372.00
cca	P	2	13285.00
ccg	P	39300	5561.00
cgt	R	0	0.02
cgc	R	0	0.04
cga	R	0	0.09
cgg	R	1	0.09
aga	R	0	0.49
agg	R	0	0.28
---	--	------	------
mPD		0.0011	0.72
		nPD:	0.
		N. weight:	2.1
		Sc. PD:	-0.13
		Sc. rank:	-62721.0

PB1

Pos.	6	obs:	exp:
att	I	62	21.76
atc	I	0	16.29
ata	I	0	23.94
act	T	39119	12192.00
acc	T	128	6941.00
aca	T	8	16990.00
acg	T	3	3136.00
aat	N	1	0.56
aac	N	0	0.44
gct	A	1	0.27
gcc	A	0	0.25
gca	A	0	0.40
gcg	A	0	0.07
---	--	------	------
mPD		0.010	0.68
		nPD:	0.02
		N. weight:	1.3
		Sc. PD:	-0.058
		Sc. rank:	-8807.6

PB1

Pos.	7	obs:	exp:
tta	L	694	4562.00
ttg	L	0	8128.00
ctt	L	3	8225.00
ctc	L	2	5765.00
cta	L	38533	6037.00
ctg	L	84	6598.00
---	-	6	6.00
---	--	------	------
mPD		0.039	1.2
		nPD:	0.03
		N. weight:	1.9
		Sc. PD:	-0.054
		Sc. rank:	14530.0

PB1

Pos.	8	obs:	exp:
tta	L	16	4563.00
ttg	L	7208	8129.00
ctt	L	755	8226.00
ctc	L	2	5766.00
cta	L	310	6038.00
ctg	L	31030	6599.00
---	-	1	1.00
---	--	------	------
mPD		0.35	1.2
		nPD:	0.31
		N. weight:	1.2
		Sc. PD:	0.29
		Sc. rank:	34463.0

PB1

Pos.	9	obs:	exp:
ttt	F	1357	19121.00
ttc	F	37952	20188.00
tta	L	2	1.16
ttg	L	0	2.07
tct	S	0	0.48
tcc	S	2	0.45
tca	S	0	0.81
tcg	S	1	0.12
ctt	L	0	2.09
ctc	L	7	1.47
cta	L	1	1.54
ctg	L	0	1.68
agt	S	0	0.52
agc	S	0	0.62
---	--	------	------
mPD		0.067	0.50
		nPD:	0.13
		N. weight:	0.57
		Sc. PD:	0.04
		Sc. rank:	13737.0

PB1

Pos.	10	obs:	exp:
tta	L	35303	4562.00
ttg	L	126	8128.00
ctt	L	2	8224.00
ctc	L	0	5765.00
cta	L	3880	6037.00
ctg	L	3	6598.00
caa	Q	1	0.61
cag	Q	0	0.39
att	I	0	2.46
atc	I	0	1.84
ata	I	7	2.70
---	--	------	------
mPD		0.19	1.2
		nPD:	0.16
		N. weight:	2.
		Sc. PD:	0.19
		Sc. rank:	49414.0

PB1

Pos.	11	obs:	exp:
cgt	R	0	0.08
cgc	R	0	0.14
cga	R	0	0.36
cgg	R	0	0.34
aat	N	1	0.56
aac	N	0	0.44
aaa	K	36759	25846.00
aag	K	2555	13468.00
aga	R	2	1.96
agg	R	2	1.11
gaa	E	0	0.59
gag	E	1	0.41
---	-	2	2.00
---	--	------	------
mPD		0.12	0.45
		nPD:	0.27
		N. weight:	0.24
		Sc. PD:	0.049
		Sc. rank:	6686.6

PB1

Pos.	12	obs:	exp:
att	I	91	32.65
atc	I	2	24.44
ata	I	0	35.92
atg	M	1	1.00
gtt	V	38811	9826.00
gtc	V	369	7993.00
gta	V	15	8502.00
gtg	V	33	12907.00
---	--	------	------
mPD		0.026	0.75
		nPD:	0.03
		N. weight:	1.5
		Sc. PD:	-0.039
		Sc. rank:	11416.0

PB1

Pos.	13	obs:	exp:
tct	S	0	2.42
tcc	S	0	2.24
tca	S	15	4.03
tcg	S	0	0.59
cct	P	15	13099.00
ccc	P	2	7369.00
cca	P	39228	13280.00
ccg	P	62	5559.00
agt	S	0	2.62
agc	S	0	3.09
---	--	------	------
mPD		0.0048	0.72
		nPD:	0.01
		N. weight:	1.2
		Sc. PD:	-0.064
		Sc. rank:	-23033.0

PB1

Pos.	14	obs:	exp:
tct	S	0	0.48
tcc	S	0	0.45
tca	S	0	0.81
tcg	S	3	0.12
act	T	0	1.55
acc	T	0	0.88
aca	T	0	2.16
acg	T	5	0.40
agt	S	0	0.52
agc	S	0	0.62
gtt	V	1	5.51
gtc	V	0	4.48
gta	V	0	4.77
gtg	V	21	7.24
gct	A	39	10709.00
gcc	A	3	9817.00
gca	A	584	15894.00
gcg	A	38666	2872.00
---	--	------	------
mPD		0.033	0.70
		nPD:	0.05
		N. weight:	2.7
		Sc. PD:	-0.041
		Sc. rank:	35393.0

PB1

Pos.	15	obs:	exp:
cct	P	0	0.33
ccc	P	0	0.19
cca	P	1	0.34
ccg	P	0	0.14
cat	H	0	0.56
cac	H	1	0.44
caa	Q	38787	24035.00
cag	Q	533	15285.00
---	--	------	------
mPD		0.027	0.48
		nPD:	0.06
		N. weight:	0.47
		Sc. PD:	-0.0028
		Sc. rank:	7260.9

PB1			
Pos.	16	obs:	exp:
tct	S	0	3.55
tcc	S	0	3.29
tca	S	0	5.91
tcg	S	0	0.87
tat	Y	8	4.22
tac	Y	0	3.78
act	T	1	0.31
acc	T	0	0.18
aca	T	0	0.43
acg	T	0	0.08
aat	N	39275	21982.00
aac	N	15	17308.00
aaa	K	0	0.66
aag	K	1	0.34
agt	S	22	3.84
agc	S	0	4.54
---	--	------	------
mPD		0.0024	0.50
		nPD:	0.
		N. weight:	0.63
		Sc. PD:	-0.036
		Sc. rank:	-14763.0

PB1			
Pos.	17	obs:	exp:
gtt	V	0	0.25
gtc	V	1	0.20
gta	V	0	0.22
gtg	V	0	0.33
gct	A	280	10717.00
gcc	A	39031	9824.00
gca	A	10	15906.00
gcg	A	0	2874.00
---	--	------	------
mPD		0.015	0.69
		nPD:	0.02
		N. weight:	1.5
		Sc. PD:	-0.059
		Sc. rank:	-1970.2

PB1			
Pos.	18	obs:	exp:
att	I	7	13802.00
atc	I	5	10331.00
ata	I	39305	15184.00
gtt	V	0	1.25
gtc	V	0	1.02
gta	V	5	1.08
gtg	V	0	1.65
---	--	------	------
mPD		0.00086	0.66
		nPD:	0.
		N. weight:	1.
		Sc. PD:	-0.062
		Sc. rank:	-30948.0

PB1			
Pos.	19	obs:	exp:
tct	S	0	6344.00
tcc	S	0	5875.00
tca	S	0	10568.00
tcg	S	0	1548.00
cgt	R	0	0.02
cgc	R	0	0.04
cga	R	0	0.09
cgg	R	0	0.09
aat	N	0	1.12
aac	N	2	0.88
agt	S	53	6871.00
agc	S	39264	8111.00
aga	R	0	0.49
agg	R	1	0.28
ggt	G	0	0.27
ggc	G	2	0.28
gga	G	0	0.92
ggg	G	0	0.53
---	--	------	------
mPD		0.0029	1.6
		nPD:	0.
		N. weight:	1.7
		Sc. PD:	-0.1
		Sc. rank:	-49675.0

PB1			
Pos.	20	obs:	exp:
tct	S	0	0.16
tcc	S	0	0.15
tca	S	1	0.27
tcg	S	0	0.04
att	I	0	3.16
atc	I	1	2.36
ata	I	8	3.48
act	T	15	12208.00
acc	T	893	6950.00
aca	T	38230	17012.00
acg	T	172	3140.00
agt	S	0	0.17
agc	S	0	0.21
gct	A	0	0.55
gcc	A	0	0.50
gca	A	2	0.81
gcg	A	0	0.15
---	--	------	------
mPD		0.054	0.68
		nPD:	0.08
		N. weight:	0.8
		Sc. PD:	0.014
		Sc. rank:	15935.0

PB1			
Pos.	21	obs:	exp:
act	T	7	12212.00
acc	T	19	6952.00
aca	T	39279	17018.00
acg	T	17	3141.00
---	--	------	------
mPD		0.0022	0.68
		nPD:	0.
		N. weight:	0.91
		Sc. PD:	-0.053
		Sc. rank:	-23449.0

PB1			
Pos.	22	obs:	exp:
ttt	F	100	19127.00
ttc	F	39221	20194.00
tta	L	0	0.12
ttg	L	0	0.21
ctt	L	0	0.21
ctc	L	1	0.15
cta	L	0	0.15
ctg	L	0	0.17
---	--	------	------
mPD		0.0051	0.50
		nPD:	0.01
		N. weight:	0.71
		Sc. PD:	-0.036
		Sc. rank:	-9275.2

PB1			
Pos.	23	obs:	exp:
tct	S	1	0.16
tcc	S	0	0.15
tca	S	0	0.27
tcg	S	0	0.04
cct	P	39192	13103.00
ccc	P	97	7372.00
cca	P	19	13285.00
ccg	P	13	5561.00
agt	S	0	0.17
agc	S	0	0.21
---	--	------	------
mPD		0.0066	0.72
		nPD:	0.01
		N. weight:	1.2
		Sc. PD:	-0.061
		Sc. rank:	-17381.0

PB1			
Pos.	24	obs:	exp:
tat	Y	39094	20727.00
tac	Y	228	18595.00
---	--	------	------
mPD		0.012	0.50
		nPD:	0.02
		N. weight:	0.66
		Sc. PD:	-0.026
		Sc. rank:	403.0

PB1			
Pos.	25	obs:	exp:
act	T	39237	12212.00
acc	T	32	6952.00
aca	T	53	17018.00
acg	T	0	3141.00
---	--	------	------
mPD		0.0043	0.68
		nPD:	0.01
		N. weight:	1.3
		Sc. PD:	-0.069
		Sc. rank:	-25471.0

PB1			
Pos.	26	obs:	exp:
ggt	G	1	5294.00
ggc	G	2	5488.00
gga	G	39276	18152.00
ggg	G	43	10388.00
---	--	------	------
mPD		0.0023	0.68
		nPD:	0.
		N. weight:	0.84
		Sc. PD:	-0.048
		Sc. rank:	-21415.0

PB1			
Pos.	27	obs:	exp:
tat	Y	2	1.05
tac	Y	0	0.95
aat	N	2	1.12
aac	N	0	0.88
gat	D	38512	22222.00
gac	D	801	17091.00
gaa	E	4	2.36
gag	E	0	1.64
ggt	G	1	0.13
ggc	G	0	0.14
gga	G	0	0.46
ggg	G	0	0.26
---	--	------	------
mPD		0.040	0.49
		nPD:	0.08
		N. weight:	0.52
		Sc. PD:	0.01
		Sc. rank:	10691.0

PB1			
Pos.	28	obs:	exp:
cct	P	39249	13103.00
ccc	P	62	7372.00
cca	P	7	13285.00
ccg	P	3	5561.00
cat	H	1	0.56
cac	H	0	0.44
---	--	------	------
mPD		0.0037	0.72
		nPD:	0.01
		N. weight:	1.2
		Sc. PD:	-0.067
		Sc. rank:	-26382.0

PB1			
Pos.	29	obs:	exp:
tct	S	0	0.16
tcc	S	0	0.15
tca	S	1	0.27
tcg	S	0	0.04
cct	P	11	13103.00
ccc	P	11	7371.00
cca	P	38856	13284.00
ccg	P	442	5561.00
act	T	0	0.31
acc	T	0	0.18
aca	T	1	0.43
acg	T	0	0.08
agt	S	0	0.17
agc	S	0	0.21
---	--	------	------
mPD		0.023	0.72
		nPD:	0.03
		N. weight:	1.1
		Sc. PD:	-0.033
		Sc. rank:	7656.1

PB1			
Pos.	30	obs:	exp:
tat	Y	48	20722.00
tac	Y	39264	18590.00
cat	H	0	5.08
cac	H	9	3.92
gat	D	1	0.57
gac	D	0	0.43
---	--	------	------
mPD		0.0030	0.50
		nPD:	0.01
		N. weight:	0.81
		Sc. PD:	-0.045
		Sc. rank:	-16895.0

PB1			
Pos.	31	obs:	exp:
tct	S	0	6345.00
tcc	S	0	5876.00
tca	S	0	10568.00
tcg	S	0	1548.00
cgt	R	0	0.02
cgc	R	0	0.04
cga	R	0	0.09
cgg	R	0	0.09
act	T	0	0.31
acc	T	1	0.18
aca	T	0	0.43
acg	T	0	0.08
agt	S	94	6871.00
agc	S	39225	8111.00
aga	R	0	0.49
agg	R	1	0.28
ggt	G	0	0.13
ggc	G	1	0.14
gga	G	0	0.46
ggg	G	0	0.26
---	--	------	------
mPD		0.0049	1.6
		nPD:	0.
		N. weight:	1.7
		Sc. PD:	-0.099
		Sc. rank:	-44519.0

PB1			
Pos.	32	obs:	exp:
cct	P	1	0.33
ccc	P	0	0.19
cca	P	0	0.34
ccg	P	0	0.14
cat	H	39280	22179.00
cac	H	41	17142.00
---	--	------	------
mPD		0.0021	0.49
		nPD:	0.
		N. weight:	0.62
		Sc. PD:	-0.035
		Sc. rank:	-14871.0

PB1			
Pos.	33	obs:	exp:
gtt	V	0	0.50
gtc	V	0	0.41
gta	V	2	0.43
gtg	V	0	0.66
ggt	G	1	5293.00
ggc	G	3	5488.00
gga	G	39222	18151.00
ggg	G	94	10388.00
---	--	------	------
mPD		0.0051	0.68
		nPD:	0.01
		N. weight:	0.83
		Sc. PD:	-0.045
		Sc. rank:	-15126.0

PB1			
Pos.	34	obs:	exp:
cct	P	0	0.33
ccc	P	0	0.19
cca	P	1	0.34
ccg	P	0	0.14
act	T	21	12211.00
acc	T	1	6952.00
aca	T	38240	17017.00
acg	T	1059	3141.00
---	--	------	------
mPD		0.054	0.68
		nPD:	0.08
		N. weight:	0.83
		Sc. PD:	0.013
		Sc. rank:	16441.0

PB1			
Pos.	35	obs:	exp:
cgt	R	0	0.06
cgc	R	0	0.11
cga	R	0	0.27
cgg	R	0	0.26
aga	R	3	1.47
agg	R	0	0.83
ggt	G	3	5293.00
ggc	G	0	5488.00
gga	G	2151	18150.00
ggg	G	37165	10388.00
---	--	------	------
mPD		0.10	0.68
		nPD:	0.15
		N. weight:	1.2
		Sc. PD:	0.11
		Sc. rank:	29770.0

PB1			
Pos.	36	obs:	exp:
act	T	27	12211.00
acc	T	6	6952.00
aca	T	39267	17017.00
acg	T	20	3140.00
gct	A	0	0.55
gcc	A	0	0.50
gca	A	2	0.81
gcg	A	0	0.15
---	--	------	------
mPD		0.0028	0.68
		nPD:	0.
		N. weight:	0.91
		Sc. PD:	-0.052
		Sc. rank:	-22469.0

,

PB1			
Pos.	37	obs:	exp:
gtt	V	0	0.25
gtc	V	0	0.20
gta	V	0	0.22
gtg	V	1	0.33
gaa	E	0	0.59
gag	E	1	0.41
ggt	G	51	5293.00
ggc	G	4	5488.00
gga	G	802	18151.00
ggg	G	38463	10388.00
---	--	------	------
mPD		0.043	0.68
		nPD:	0.06
		N. weight:	1.3
		Sc. PD:	0.00058
		Sc. rank:	22697.0

,

PB1			
Pos.	38	obs:	exp:
tat	Y	112	20718.00
tac	Y	39193	18587.00
cat	H	0	9.59
cac	H	17	7.41
---	--	------	------
mPD		0.0065	0.50
		nPD:	0.01
		N. weight:	0.8
		Sc. PD:	-0.039
		Sc. rank:	-7759.6

,

PB1			
Pos.	39	obs:	exp:
act	T	36621	12210.00
acc	T	2674	6951.00
aca	T	22	17016.00
acg	T	1	3140.00
gct	A	4	1.09
gcc	A	0	1.00
gca	A	0	1.62
gcg	A	0	0.29
---	--	------	------
mPD		0.13	0.68
		nPD:	0.19
		N. weight:	1.
		Sc. PD:	0.13
		Sc. rank:	26957.0

,

PB1			
Pos.	40	obs:	exp:
tta	L	0	0.58
ttg	L	0	1.03
ctt	L	0	1.05
ctc	L	0	0.73
cta	L	0	0.77
ctg	L	5	0.84
att	I	4	9.13
atc	I	0	6.83
ata	I	22	10.04
atg	M	39289	39289.00
gtt	V	0	0.50
gtc	V	0	0.41
gta	V	0	0.43
gtg	V	2	0.66
---	--	------	------
mPD		0.0017	0.0019
		nPD:	0.88
		N. weight:	0.00074
		Sc. PD:	0.00059
		Sc. rank:	23.0

,

PB1			
Pos.	41	obs:	exp:
aat	N	0	2.24
aac	N	4	1.76
gct	A	0	0.27
gcc	A	1	0.25
gca	A	0	0.40
gcg	A	0	0.07
gat	D	99	22219.00
gac	D	39209	17089.00
gaa	E	7	4.72
gag	E	1	3.28
ggt	G	0	0.13
ggc	G	1	0.14
gga	G	0	0.46
ggg	G	0	0.26
---	--	------	------
mPD		0.0057	0.49
		nPD:	0.01
		N. weight:	0.89
		Sc. PD:	-0.044
		Sc. rank:	-10182.0

,

PB1			
Pos.	42	obs:	exp:
att	I	0	0.35
atc	I	0	0.26
ata	I	1	0.39
act	T	37	12211.00
acc	T	1	6952.00
aca	T	39224	17017.00
acg	T	58	3140.00
gaa	E	1	0.59
gag	E	0	0.41
---	--	------	------
mPD		0.0050	0.68
		nPD:	0.01
		N. weight:	0.9
		Sc. PD:	-0.048
		Sc. rank:	-16485.0

,

PB1			
Pos.	43	obs:	exp:
att	I	0	0.35
atc	I	1	0.26
ata	I	0	0.39
gtt	V	418	9849.00
gtc	V	38794	8012.00
gta	V	108	8522.00
gtg	V	0	12937.00
gct	A	0	0.27
gcc	A	1	0.25
gca	A	0	0.40
gcg	A	0	0.07
---	--	------	------
mPD		0.027	0.74
		nPD:	0.04
		N. weight:	1.7
		Sc. PD:	-0.043
		Sc. rank:	14365.0

,

PB1			
Pos.	44	obs:	exp:
tct	S	0	6.78
tcc	S	0	6.28
tca	S	0	11.29
tcg	S	0	1.65
cat	H	0	0.56
cac	H	1	0.44
att	I	0	0.35
atc	I	1	0.26
ata	I	0	0.39
act	T	0	0.31
acc	T	1	0.18
aca	T	0	0.43
acg	T	0	0.08
aat	N	171	21974.00
aac	N	39105	17302.00
aaa	K	1	0.66
aag	K	0	0.34
agt	S	0	7.34
agc	S	42	8.66
---	--	------	------
mPD		0.011	0.50
		nPD:	0.02
		N. weight:	0.87
		Sc. PD:	-0.034
		Sc. rank:	-94.0

,

PB1			
Pos.	45	obs:	exp:
cgt	R	0	779.60
cgc	R	0	1415.00
cga	R	5	3588.00
cgg	R	0	3376.00
aaa	K	1	0.66
aag	K	0	0.34
aga	R	39206	19286.00
agg	R	110	10876.00
---	--	------	------
mPD		0.0059	0.89
		nPD:	0.01
		N. weight:	0.76
		Sc. PD:	-0.041
		Sc. rank:	-14872.0

,

PB1			
Pos.	46	obs:	exp:
att	I	0	0.35
atc	I	0	0.26
ata	I	1	0.39
act	T	2	12211.00
acc	T	39	6952.00
aca	T	39235	17017.00
acg	T	45	3141.00
---	--	------	------
mPD		0.0044	0.68
		nPD:	0.01
		N. weight:	0.9
		Sc. PD:	-0.049
		Sc. rank:	-17999.0

,

PB1			
Pos.	47	obs:	exp:
cat	H	5741	22179.00
cac	H	33581	17143.00
---	--	------	------
mPD		0.25	0.49
		nPD:	0.51
		N. weight:	0.41
		Sc. PD:	0.18
		Sc. rank:	12333.0

,

PB1			
Pos.	48	obs:	exp:
tta	L	0	0.12
ttg	L	0	0.21
ctt	L	0	0.21
ctc	L	0	0.15
cta	L	1	0.15
ctg	L	0	0.17
cat	H	0	1.13
cac	H	2	0.87
caa	Q	39226	24031.00
cag	Q	86	15281.00
cgt	R	0	0.08
cgc	R	0	0.14
cga	R	4	0.36
cgg	R	0	0.34
aaa	K	3	1.97
aag	K	0	1.03
aga	R	0	1.96
agg	R	0	1.11
---	--	------	------
mPD		0.0049	0.48
		nPD:	0.01
		N. weight:	0.52
		Sc. PD:	-0.027
		Sc. rank:	-6862.1

,

PB1			
Pos.	49	obs:	exp:
tat	Y	4497	20724.00
tac	Y	34819	18592.00
tgt	C	4	2.34
tgc	C	2	3.66
---	--	------	------
mPD		0.20	0.50
		nPD:	0.41
		N. weight:	0.42
		Sc. PD:	0.14
		Sc. rank:	12171.0

,

PB1			
Pos.	50	obs:	exp:
tct	S	28	6345.00
tcc	S	1	5876.00
tca	S	39249	10568.00
tcg	S	42	1548.00
act	T	0	0.31
acc	T	0	0.18
aca	T	1	0.43
acg	T	0	0.08
agt	S	0	6871.00
agc	S	0	8112.00
gct	A	0	0.27
gcc	A	0	0.25
gca	A	1	0.40
gcg	A	0	0.07
---	--	------	------
mPD		0.0037	1.6
		nPD:	0.
		N. weight:	1.4
		Sc. PD:	-0.084
		Sc. rank:	-40190.0

,

PB1

Pos.	51	obs:	exp:
aaa	K	2	16.44
aag	K	23	8.56
gtt	V	0	1.00
gtc	V	0	0.82
gta	V	0	0.87
gtg	V	4	1.32
gat	D	6	3.39
gac	D	0	2.61
gaa	E	1029	23157.00
gag	E	38250	16122.00
ggt	G	0	1.08
ggc	G	0	1.12
gga	G	0	3.69
ggg	G	8	2.11
---	--	------	------
mPD		0.053	0.49
		nPD:	0.11
		N. weight:	0.83
		Sc. PD:	0.038
		Sc. rank:	18619.0

PB1

Pos.	52	obs:	exp:
tgg	W	7	7.00
cgt	R	0	752.60
cgc	R	0	1366.00
cga	R	0	3463.00
cgg	R	3	3259.00
atg	M	10	10.00
aat	N	0	0.56
aac	N	1	0.44
aaa	K	265	882.30
aag	K	1077	459.70
aga	R	3827	18617.00
agg	R	34126	10499.00
ggt	G	0	0.81
ggc	G	0	0.84
gga	G	0	2.77
ggg	G	6	1.59
---	--	------	------
mPD		0.25	0.94
		nPD:	0.27
		N. weight:	0.97
		Sc. PD:	0.2
		Sc. rank:	26678.0

PB1

Pos.	53	obs:	exp:
cct	P	0	0.33
ccc	P	1	0.19
cca	P	0	0.34
ccg	P	0	0.14
cgt	R	0	0.02
cgc	R	0	0.04
cga	R	0	0.09
cgg	R	0	0.09
aga	R	0	0.49
agg	R	1	0.28
ggt	G	2	5293.00
ggc	G	0	5488.00
gga	G	442	18150.00
ggg	G	38875	10388.00
---	-	1	1.00
---	--	------	------
mPD		0.023	0.68
		nPD:	0.03
		N. weight:	1.4
		Sc. PD:	-0.04
		Sc. rank:	9876.1

PB1

Pos.	54	obs:	exp:
cgt	R	0	1.84
cgc	R	0	3.35
cga	R	0	8.49
cgg	R	0	7.99
atg	M	3	3.00
act	T	0	0.31
acc	T	0	0.18
aca	T	0	0.43
acg	T	1	0.08
aat	N	8	4.48
aac	N	0	3.52
aaa	K	553	25779.00
aag	K	38658	13432.00
aga	R	4	45.61
agg	R	89	25.72
gaa	E	1	3.54
gag	E	5	2.46
---	--	------	------
mPD		0.034	0.46
		nPD:	0.07
		N. weight:	1.1
		Sc. PD:	0.012
		Sc. rank:	20966.0

PB1

Pos.	55	obs:	exp:
tgg	W	39320	39320.00
cct	P	0	0.33
ccc	P	0	0.19
cca	P	0	0.34
ccg	P	1	0.14
gtt	V	0	0.25
gtc	V	0	0.20
gta	V	0	0.22
gtg	V	1	0.33
---	--	------	------
mPD		0.00020	0.00028
		nPD:	0.72
		N. weight:	0.000086
		Sc. PD:	0.000055
		Sc. rank:	2.6

PB1

Pos.	56	obs:	exp:
tct	S	0	0.16
tcc	S	0	0.15
tca	S	1	0.27
tcg	S	0	0.04
att	I	0	1.40
atc	I	0	1.05
ata	I	4	1.54
atg	M	75	75.00
act	T	39	12144.00
acc	T	14	6914.00
aca	T	14033	16924.00
acg	T	25020	3123.00
aaa	K	6	17.75
aag	K	21	9.25
agt	S	0	0.17
agc	S	0	0.21
gct	A	0	29.16
gcc	A	0	26.73
gca	A	17	43.28
gcg	A	90	7.82
gat	D	0	0.57
gac	D	1	0.43
---	-	1	1.00
---	--	------	------
mPD		0.47	0.69
		nPD:	0.68
		N. weight:	1.4
		Sc. PD:	0.83
		Sc. rank:	41980.0

PB1

Pos.	57	obs:	exp:
cct	P	0	0.33
ccc	P	0	0.19
cca	P	1	0.34
ccg	P	0	0.14
cgt	R	0	0.02
cgc	R	0	0.04
cga	R	0	0.09
cgg	R	0	0.09
att	I	0	62.84
atc	I	0	47.03
ata	I	179	69.13
atg	M	6	6.00
act	T	57	12148.00
acc	T	1	6916.00
aca	T	37565	16929.00
acg	T	1493	3124.00
aaa	K	11	7.89
aag	K	1	4.11
aga	R	1	0.49
agg	R	0	0.28
gct	A	0	1.64
gcc	A	0	1.50
gca	A	6	2.43
gcg	A	0	0.44
gaa	E	1	0.59
gag	E	0	0.41
---	--	------	------
mPD		0.087	0.69
		nPD:	0.13
		N. weight:	0.8
		Sc. PD:	0.049
		Sc. rank:	18797.0

PB1

Pos.	58	obs:	exp:
tct	S	0	3.87
tcc	S	0	3.59
tca	S	0	6.45
tcg	S	0	0.94
aat	N	38437	21985.00
aac	N	859	17311.00
aaa	K	2	1.31
aag	K	0	0.69
agt	S	24	4.19
agc	S	0	4.95
---	--	------	------
mPD		0.044	0.50
		nPD:	0.09
		N. weight:	0.53
		Sc. PD:	0.014
		Sc. rank:	11095.0

PB1

Pos.	59	obs:	exp:
tct	S	0	0.32
tcc	S	0	0.30
tca	S	2	0.54
tcg	S	0	0.08
att	I	0	4.91
atc	I	0	3.68
ata	I	14	5.41
act	T	25	12203.00
acc	T	11	6947.00
aca	T	38342	17005.00
acg	T	915	3138.00
aaa	K	1	0.66
aag	K	0	0.34
agt	S	0	0.35
agc	S	0	0.41
gct	A	0	3.27
gcc	A	0	3.00
gca	A	10	4.85
gcg	A	2	0.88
---	--	------	------
mPD		0.049	0.68
		nPD:	0.07
		N. weight:	0.83
		Sc. PD:	0.0075
		Sc. rank:	15644.0

PB1

Pos.	60	obs:	exp:
caa	Q	1	0.61
cag	Q	0	0.39
gaa	E	39001	23182.00
gag	E	320	16139.00
---	--	------	------
mPD		0.016	0.48
		nPD:	0.03
		N. weight:	0.53
		Sc. PD:	-0.015
		Sc. rank:	3834.9

PB1

Pos.	61	obs:	exp:
att	I	6	2.11
atc	I	0	1.58
ata	I	0	2.32
act	T	39196	12209.00
acc	T	32	6951.00
aca	T	82	17015.00
acg	T	5	3140.00
aat	N	1	0.56
aac	N	0	0.44
---	--	------	------
mPD		0.0064	0.68
		nPD:	0.01
		N. weight:	1.3
		Sc. PD:	-0.065
		Sc. rank:	-17660.0

PB1

Pos.	62	obs:	exp:
tgg	W	2	2.00
cgt	R	0	0.12
cgc	R	0	0.22
cga	R	0	0.55
cgg	R	0	0.52
aga	R	0	2.94
agg	R	6	1.66
gaa	E	0	27.71
gag	E	47	19.29
ggt	G	33	5286.00
ggc	G	2	5480.00
gga	G	180	18126.00
ggg	G	39052	10374.00
---	--	------	------
mPD		0.014	0.68
		nPD:	0.02
		N. weight:	1.4
		Sc. PD:	-0.059
		Sc. rank:	-3349.5

PB1

Pos.	63	obs:	exp:
tct	S	0	0.16
tcc	S	0	0.15
tca	S	0	0.27
tcg	S	1	0.04
act	T	0	2.17
acc	T	0	1.24
aca	T	1	3.03
acg	T	6	0.56
agt	S	0	0.17
agc	S	0	0.21
gtt	V	5	2.00
gtc	V	0	1.63
gta	V	0	1.73
gtg	V	3	2.63
gct	A	16474	10713.00
gcc	A	50	9820.00
gca	A	3288	15899.00
gcg	A	19494	2873.00
---	--	------	------
mPD		0.57	0.70
		nPD:	0.82
		N. weight:	1.1
		Sc. PD:	0.81
		Sc. rank:	33620.0

PB1

Pos.	64	obs:	exp:
tta	L	0	1.28
ttg	L	0	2.27
tct	S	0	6.94
tcc	S	43	6.43
tca	S	0	11.56
tcg	S	0	1.69
ctt	L	0	2.30
ctc	L	11	1.61
cta	L	0	1.69
ctg	L	0	1.85
cct	P	104	13081.00
ccc	P	39144	7359.00
cca	P	3	13262.00
ccg	P	3	5552.00
cat	H	0	4.51
cac	H	8	3.49
act	T	0	0.62
acc	T	2	0.35
aca	T	0	0.87
acg	T	0	0.16
agt	S	0	7.51
agc	S	0	8.87
gct	A	0	1.09
gcc	A	4	1.00
gca	A	0	1.62
gcg	A	0	0.29
---	--	------	------
mPD		0.0090	0.72
		nPD:	0.01
		N. weight:	1.8
		Sc. PD:	-0.089
		Sc. rank:	-19181.0

PB1

Pos.	65	obs:	exp:
tta	L	0	0.12
ttg	L	0	0.21
ctt	L	0	0.21
ctc	L	0	0.15
cta	L	0	0.15
ctg	L	1	0.17
caa	Q	2093	24036.00
cag	Q	37228	15285.00
---	--	------	------
mPD		0.10	0.48
		nPD:	0.21
		N. weight:	0.78
		Sc. PD:	0.11
		Sc. rank:	20597.0

PB1			
Pos.	66	obs:	exp:
tta	L	14	4562.00
ttg	L	0	8129.00
ctt	L	145	8225.00
ctc	L	38450	5766.00
cta	L	709	6038.00
ctg	L	0	6599.00
att	I	0	1.40
atc	I	4	1.05
ata	I	0	1.54
---	--	------	------
mPD		0.044	1.2
		nPD:	0.04
		N. weight:	2.
		Sc. PD:	-0.047
		Sc. rank:	18385.0

,

PB1			
Pos.	67	obs:	exp:
ttt	F	0	0.49
ttc	F	1	0.51
tct	S	0	0.16
tcc	S	0	0.15
tca	S	0	0.27
tcg	S	0	0.04
aat	N	439	21998.00
aac	N	38881	17322.00
agt	S	0	0.17
agc	S	1	0.21
---	--	------	------
mPD		0.022	0.49
		nPD:	0.05
		N. weight:	0.83
		Sc. PD:	-0.014
		Sc. rank:	9758.1

,

PB1			
Pos.	68	obs:	exp:
cct	P	16	13104.00
ccc	P	3	7372.00
cca	P	38865	13285.00
ccg	P	438	5562.00
---	--	------	------
mPD		0.023	0.72
		nPD:	0.03
		N. weight:	1.1
		Sc. PD:	-0.034
		Sc. rank:	7441.9

,

PB1			
Pos.	69	obs:	exp:
att	I	39244	13793.00
atc	I	45	10324.00
ata	I	3	15174.00
act	T	12	3.73
acc	T	0	2.12
aca	T	0	5.19
acg	T	0	0.96
gtt	V	18	4.51
gtc	V	0	3.67
gta	V	0	3.90
gtg	V	0	5.92
---	--	------	------
mPD		0.0040	0.66
		nPD:	0.01
		N. weight:	1.1
		Sc. PD:	-0.063
		Sc. rank:	-23787.0

,

PB1			
Pos.	70	obs:	exp:
aat	N	3	1.68
aac	N	0	1.32
gat	D	39211	22225.00
gac	D	107	17093.00
---	-	1	1.00
---	--	------	------
mPD		0.0056	0.49
		nPD:	0.01
		N. weight:	0.61
		Sc. PD:	-0.03
		Sc. rank:	-7071.8

,

PB1			
Pos.	71	obs:	exp:
ggt	G	1	5294.00
ggc	G	10	5488.00
gga	G	39192	18152.00
ggg	G	119	10388.00
---	--	------	------
mPD		0.0066	0.68
		nPD:	0.01
		N. weight:	0.82
		Sc. PD:	-0.043
		Sc. rank:	-11349.0

,

PB1			
Pos.	72	obs:	exp:
tct	S	0	0.32
tcc	S	0	0.30
tca	S	2	0.54
tcg	S	0	0.08
cct	P	0	13103.00
ccc	P	28	7371.00
cca	P	39246	13284.00
ccg	P	46	5561.00
agt	S	0	0.35
agc	S	0	0.41
---	--	------	------
mPD		0.0039	0.72
		nPD:	0.01
		N. weight:	1.2
		Sc. PD:	-0.066
		Sc. rank:	-25202.0

,

PB1			
Pos.	73	obs:	exp:
tta	L	172	4562.00
ttg	L	0	8128.00
ctt	L	0	8224.00
ctc	L	18	5765.00
cta	L	39032	6037.00
ctg	L	93	6598.00
att	I	0	2.46
atc	I	0	1.84
ata	I	7	2.70
---	--	------	------
mPD		0.015	1.2
		nPD:	0.01
		N. weight:	2.
		Sc. PD:	-0.097
		Sc. rank:	-20498.0

,

PB1			
Pos.	74	obs:	exp:
tta	L	0	0.12
ttg	L	0	0.21
ctt	L	1	0.21
ctc	L	0	0.15
cta	L	0	0.15
ctg	L	0	0.17
cct	P	39250	13103.00
ccc	P	46	7371.00
cca	P	17	13284.00
ccg	P	7	5561.00
cat	H	1	0.56
cac	H	0	0.44
---	--	------	------
mPD		0.0037	0.72
		nPD:	0.01
		N. weight:	1.2
		Sc. PD:	-0.067
		Sc. rank:	-26657.0

,

PB1			
Pos.	75	obs:	exp:
aaa	K	0	17.09
aag	K	26	8.91
gtt	V	0	0.75
gtc	V	0	0.61
gta	V	0	0.65
gtg	V	3	0.99
gat	D	215	121.50
gac	D	0	93.47
gaa	E	4617	23027.00
gag	E	34441	16031.00
ggt	G	4	2.69
ggc	G	0	2.79
gga	G	2	9.23
ggg	G	14	5.28
---	--	------	------
mPD		0.22	0.49
		nPD:	0.45
		N. weight:	0.53
		Sc. PD:	0.2
		Sc. rank:	15723.0

,

PB1			
Pos.	76	obs:	exp:
aat	N	177	99.03
aac	N	0	77.97
gat	D	38861	22118.00
gac	D	268	17011.00
ggt	G	16	2.15
ggc	G	0	2.23
gga	G	0	7.39
ggg	G	0	4.23
---	--	------	------
mPD		0.023	0.50
		nPD:	0.05
		N. weight:	0.58
		Sc. PD:	-0.0091
		Sc. rank:	7278.8

,

PB1			
Pos.	77	obs:	exp:
aat	N	39261	21992.00
aac	N	48	17317.00
aaa	K	0	0.66
aag	K	1	0.34
gat	D	12	6.78
gac	D	0	5.22
---	--	------	------
mPD		0.0031	0.49
		nPD:	0.01
		N. weight:	0.63
		Sc. PD:	-0.034
		Sc. rank:	-12830.0

,

PB1			
Pos.	78	obs:	exp:
caa	Q	1	0.61
cag	Q	0	0.39
aaa	K	5	3.29
aag	K	0	1.71
gat	D	2	4.52
gac	D	6	3.48
gaa	E	38554	23172.00
gag	E	749	16131.00
ggt	G	0	0.67
ggc	G	0	0.70
gga	G	5	2.31
ggg	G	0	1.32
---	--	------	------
mPD		0.038	0.48
		nPD:	0.08
		N. weight:	0.48
		Sc. PD:	0.0078
		Sc. rank:	9627.3

,

PB1			
Pos.	79	obs:	exp:
cct	P	3	13103.00
ccc	P	4	7372.00
cca	P	39247	13285.00
ccg	P	67	5561.00
gct	A	0	0.27
gcc	A	0	0.25
gca	A	0	0.40
gcg	A	1	0.07
---	--	------	------
mPD		0.0039	0.72
		nPD:	0.01
		N. weight:	1.2
		Sc. PD:	-0.066
		Sc. rank:	-25331.0

,

PB1			
Pos.	80	obs:	exp:
tct	S	0	6343.00
tcc	S	0	5874.00
tca	S	0	10565.00
tcg	S	0	1547.00
tgt	C	2	0.78
tgc	C	0	1.22
att	I	5	1.76
atc	I	0	1.31
ata	I	0	1.93
aat	N	8	4.48
aac	N	0	3.52
agt	S	39160	6869.00
agc	S	146	8109.00
ggt	G	1	0.13
ggc	G	0	0.14
gga	G	0	0.46
ggg	G	0	0.26
---	--	------	------
mPD		0.0082	1.6
		nPD:	0.01
		N. weight:	1.9
		Sc. PD:	-0.11
		Sc. rank:	-42540.0

,

PB1			
Pos.	81	obs:	exp:
ggt	G	7	5294.00
ggc	G	7	5488.00
gga	G	39010	18152.00
ggg	G	298	10388.00
---	--	------	------
mPD		0.016	0.68
		nPD:	0.02
		N. weight:	0.8
		Sc. PD:	-0.031
		Sc. rank:	538.1

,

PB1			
Pos.	82	obs:	exp:
tat	Y	39247	20727.00
tac	Y	74	18594.00
cat	H	1	0.56
cac	H	0	0.44
---	--	------	------
mPD		0.0038	0.50
		nPD:	0.01
		N. weight:	0.69
		Sc. PD:	-0.037
		Sc. rank:	-12003.0

,

PB1			
Pos.	83	obs:	exp:
tct	S	0	0.32
tcc	S	0	0.30
tca	S	2	0.54
tcg	S	0	0.08
agt	S	0	0.35
agc	S	0	0.41
gtt	V	0	0.25
gtc	V	0	0.20
gta	V	1	0.22
gtg	V	0	0.33
gct	A	0	10716.00
gcc	A	8	9823.00
gca	A	39290	15904.00
gcg	A	20	2874.00
gaa	E	1	0.59
gag	E	0	0.41
---	--	------	------
mPD		0.0016	0.69
		nPD:	0.
		N. weight:	0.99
		Sc. PD:	-0.058
		Sc. rank:	-27098.0

,

PB1			
Pos.	84	obs:	exp:
caa	Q	39297	24037.00
cag	Q	25	15285.00
---	--	------	------
mPD		0.0013	0.48
		nPD:	0.
		N. weight:	0.53
		Sc. PD:	-0.031
		Sc. rank:	-14574.0

,

PB1			
Pos.	85	obs:	exp:
act	T	5	12212.00
acc	T	1	6952.00
aca	T	39236	17018.00
acg	T	80	3141.00
---	--	------	------
mPD		0.0044	0.68
		nPD:	0.01
		N. weight:	0.9
		Sc. PD:	-0.05
		Sc. rank:	-18103.0

,

PB1

Pos.	86	obs :	exp :
aat	N	0	0.56
aac	N	1	0.44
gat	D	575	22223.00
gac	D	38740	17092.00
gaa	E	4	3.54
gag	E	2	2.46
---	--	------	------
mPD		0.029	0.49
		nPD :	0.06
		N. weight :	0.83
		Sc. PD :	-0.0026
		Sc. rank :	13602.0

PB1

Pos.	87	obs :	exp :
tat	Y	2	1.05
tac	Y	0	0.95
tgt	C	39033	15366.00
tgc	C	287	23954.00
---	--	------	------
mPD		0.015	0.48
		nPD :	0.03
		N. weight :	0.98
		Sc. PD :	-0.03
		Sc. rank :	5892.7

PB1

Pos.	88	obs :	exp :
att	I	0	0.35
atc	I	1	0.26
ata	I	0	0.39
gtt	V	576	9849.00
gtc	V	38609	8012.00
gta	V	136	8523.00
gtg	V	0	12938.00
---	--	------	------
mPD		0.036	0.74
		nPD :	0.05
		N. weight :	1.6
		Sc. PD :	-0.023
		Sc. rank :	21644.0

PB1

Pos.	89	obs :	exp :
tta	L	1	4563.00
ttg	L	1244	8130.00
ctt	L	8	8226.00
ctc	L	0	5766.00
cta	L	152	6038.00
ctg	L	37917	6599.00
---	--	------	------
mPD		0.069	1.2
		nPD :	0.06
		N. weight :	1.8
		Sc. PD :	-0.0045
		Sc. rank :	29664.0

PB1

Pos.	90	obs :	exp :
aaa	K	0	0.66
aag	K	1	0.34
gaa	E	155	23182.00
gag	E	39165	16138.00
ggt	G	0	0.13
ggc	G	0	0.14
gga	G	0	0.46
ggg	G	1	0.26
---	--	------	------
mPD		0.0080	0.48
		nPD :	0.02
		N. weight :	0.95
		Sc. PD :	-0.043
		Sc. rank :	-5183.4

PB1

Pos.	91	obs :	exp :
act	T	1	0.31
acc	T	0	0.18
aca	T	0	0.43
acg	T	0	0.08
gtt	V	2	0.50
gtc	V	0	0.41
gta	V	0	0.43
gtg	V	0	0.66
gct	A	38770	10717.00
gcc	A	465	9823.00
gca	A	76	15905.00
gcg	A	8	2874.00
---	--	------	------
mPD		0.028	0.69
		nPD :	0.04
		N. weight :	1.3
		Sc. PD :	-0.03
		Sc. rank :	12963.0

PB1

Pos.	92	obs :	exp :
atg	M	39322	39322.00
---	--	------	------
mPD		0	0
		nPD :	1.
		N. weight :	0.
		Sc. PD :	0
		Sc. rank :	0

PB1

Pos.	93	obs :	exp :
tct	S	0	0.16
tcc	S	1	0.15
tca	S	0	0.27
tcg	S	0	0.04
agt	S	0	0.17
agc	S	0	0.21
gtt	V	0	0.50
gtc	V	2	0.41
gta	V	0	0.43
gtg	V	0	0.66
gct	A	646	10717.00
gcc	A	38560	9823.00
gca	A	113	15905.00
gcg	A	0	2874.00
---	--	------	------
mPD		0.038	0.69
		nPD :	0.05
		N. weight :	1.4
		Sc. PD :	-0.011
		Sc. rank :	21553.0

PB1

Pos.	94	obs :	exp :
ttt	F	6296	19095.00
ttc	F	32960	20161.00
tta	L	11	4.53
ttg	L	1	8.06
tct	S	1	3.87
tcc	S	23	3.59
tca	S	0	6.45
tcg	S	0	0.94
tat	Y	0	1.05
tac	Y	2	0.95
tgt	C	0	0.39
tgc	C	1	0.61
ctt	L	2	8.16
ctc	L	25	5.72
cta	L	0	5.99
ctg	L	0	6.55
agt	S	0	4.19
agc	S	0	4.95
---	--	------	------
mPD		0.27	0.50
		nPD :	0.54
		N. weight :	0.26
		Sc. PD :	0.12
		Sc. rank :	7790.7

PB1

Pos.	95	obs :	exp :
tta	L	0	4563.00
ttg	L	0	8130.00
ctt	L	39274	8226.00
ctc	L	32	5766.00
cta	L	13	6038.00
ctg	L	3	6599.00
---	--	------	------
mPD		0.0024	1.2
		nPD :	0.
		N. weight :	1.7
		Sc. PD :	-0.1
		Sc. rank :	-48330.0

PB1

Pos.	96	obs :	exp :
gat	D	0	0.57
gac	D	1	0.43
gaa	E	39104	23181.00
gag	E	215	16138.00
ggt	G	0	0.27
ggc	G	0	0.28
gga	G	2	0.92
ggg	G	0	0.53
---	--	------	------
mPD		0.011	0.48
		nPD :	0.02
		N. weight :	0.54
		Sc. PD :	-0.021
		Sc. rank :	172.2

PB1

Pos.	97	obs :	exp :
aaa	K	39	25.64
aag	K	0	13.36
gct	A	0	0.27
gcc	A	0	0.25
gca	A	1	0.40
gcg	A	0	0.07
gat	D	4	14.70
gac	D	22	11.30
gaa	E	39163	23142.00
gag	E	90	16111.00
ggt	G	0	0.40
ggc	G	0	0.42
gga	G	3	1.38
ggg	G	0	0.79
---	--	------	------
mPD		0.0081	0.49
		nPD :	0.02
		N. weight :	0.56
		Sc. PD :	-0.025
		Sc. rank :	-2928.5

PB1

Pos.	98	obs :	exp :
ttt	F	0	0.49
ttc	F	1	0.51
tct	S	78	6345.00
tcc	S	39134	5876.00
tca	S	107	10568.00
tcg	S	0	1548.00
act	T	0	0.31
acc	T	1	0.18
aca	T	0	0.43
acg	T	0	0.08
agt	S	0	6871.00
agc	S	0	8111.00
gct	A	0	0.27
gcc	A	1	0.25
gca	A	0	0.40
gcg	A	0	0.07
---	--	------	------
mPD		0.0095	1.6
		nPD :	0.01
		N. weight :	2.
		Sc. PD :	-0.11
		Sc. rank :	-43288.0

PB1

Pos.	99	obs :	exp :
cat	H	3341	22178.00
cac	H	35979	17142.00
caa	Q	1	0.61
cag	Q	0	0.39
aat	N	0	0.56
aac	N	1	0.44
---	--	------	------
mPD		0.16	0.49
		nPD :	0.32
		N. weight :	0.57
		Sc. PD :	0.14
		Sc. rank :	16013.0

PB1

Pos.	100	obs :	exp :
tct	S	0	0.16
tcc	S	0	0.15
tca	S	1	0.27
tcg	S	0	0.04
cct	P	7	13103.00
ccc	P	6	7372.00
cca	P	39226	13285.00
ccg	P	82	5561.00
agt	S	0	0.17
agc	S	0	0.21
---	--	------	------
mPD		0.0049	0.72
		nPD :	0.01
		N. weight :	1.2
		Sc. PD :	-0.064
		Sc. rank :	-22671.0

PB1

Pos.	101	obs :	exp :
tct	S	0	0.65
tcc	S	0	0.60
tca	S	0	1.08
tcg	S	0	0.16
tgt	C	3	1.17
tgc	C	0	1.83
agt	S	4	0.70
agc	S	0	0.83
gat	D	3	1.70
gac	D	0	1.30
ggt	G	38347	5292.00
ggc	G	277	5487.00
gga	G	43	18147.00
ggg	G	645	10386.00
---	--	------	------
mPD		0.049	0.68
		nPD :	0.07
		N. weight :	2.
		Sc. PD :	0.018
		Sc. rank :	37808.0

PB1

Pos.	102	obs :	exp :
att	I	134	13803.00
atc	I	38712	10332.00
ata	I	473	15185.00
gtt	V	0	0.75
gtc	V	3	0.61
gta	V	0	0.65
gtg	V	0	0.99
---	--	------	------
mPD		0.031	0.66
		nPD :	0.05
		N. weight :	1.4
		Sc. PD :	-0.021
		Sc. rank :	17154.0

PB1			
Pos.	103	obs:	exp:
ttt	F	39254	19126.00
ttc	F	66	20194.00
tta	L	0	0.12
ttg	L	0	0.21
tat	Y	1	0.53
tac	Y	0	0.47
ctt	L	1	0.21
ctc	L	0	0.15
cta	L	0	0.15
ctg	L	0	0.17
---	--	------	------
mPD		0.0035	0.50
		nPD:	0.01
		N. weight:	0.78
		Sc. PD:	-0.042
		Sc. rank:	-14655.0

PB1			
Pos.	104	obs:	exp:
aaa	K	0	2.63
aag	K	4	1.37
gtt	V	0	0.50
gtc	V	0	0.41
gta	V	1	0.43
gtg	V	1	0.66
gat	D	10	5.65
gac	D	0	4.35
gaa	E	9272	23173.00
gag	E	30034	16133.00
---	--	------	------
mPD		0.36	0.48
		nPD:	0.75
		N. weight:	0.28
		Sc. PD:	0.19
		Sc. rank:	8758.0

PB1			
Pos.	105	obs:	exp:
tct	S	0	5.16
tcc	S	0	4.78
tca	S	0	8.60
tcg	S	0	1.26
cat	H	0	1.13
cac	H	2	0.87
act	T	0	6.83
acc	T	22	3.89
aca	T	0	9.52
acg	T	0	1.76
aat	N	308	21950.00
aac	N	38926	17284.00
aaa	K	3	1.97
aag	K	0	1.03
agt	S	0	5.59
agc	S	32	6.60
gat	D	0	16.39
gac	D	29	12.61
---	--	------	------
mPD		0.020	0.50
		nPD:	0.04
		N. weight:	0.85
		Sc. PD:	-0.019
		Sc. rank:	8373.2

PB1			
Pos.	106	obs:	exp:
tct	S	35	6344.00
tcc	S	29952	5875.00
tca	S	7726	10568.00
tcg	S	1604	1548.00
act	T	0	0.31
acc	T	0	0.18
aca	T	1	0.43
acg	T	0	0.08
agt	S	0	6871.00
agc	S	0	8111.00
gct	A	0	1.09
gcc	A	2	1.00
gca	A	2	1.62
gcg	A	0	0.29
---	--	------	------
mPD		0.38	1.6
		nPD:	0.23
		N. weight:	1.3
		Sc. PD:	0.21
		Sc. rank:	34795.0

PB1			
Pos.	107	obs:	exp:
tct	S	0	0.16
tcc	S	0	0.15
tca	S	0	0.27
tcg	S	0	0.04
tgt	C	155	15366.00
tgc	C	39165	23954.00
tgg	W	1	1.00
agt	S	0	0.17
agc	S	1	0.21
---	--	------	------
mPD		0.0080	0.48
		nPD:	0.02
		N. weight:	0.52
		Sc. PD:	-0.023
		Sc. rank:	-2545.5

PB1			
Pos.	108	obs:	exp:
ttt	F	12	5.84
ttc	F	0	6.16
tta	L	0	4558.00
ttg	L	0	8121.00
ctt	L	39261	8217.00
ctc	L	17	5760.00
cta	L	2	6032.00
ctg	L	2	6593.00
cct	P	1	0.33
ccc	P	0	0.19
cca	P	0	0.34
ccg	P	0	0.14
cat	H	2	1.13
cac	H	0	0.87
cgt	R	1	0.02
cgc	R	0	0.04
cga	R	0	0.09
cgg	R	0	0.09
att	I	24	8.42
atc	I	0	6.31
ata	I	0	9.27
aga	R	0	0.49
agg	R	0	0.28
---	--	------	------
mPD		0.0031	1.2
		nPD:	0.
		N. weight:	1.7
		Sc. PD:	-0.1
		Sc. rank:	-46717.0

PB1			
Pos.	109	obs:	exp:
gat	D	0	0.57
gac	D	1	0.43
gaa	E	39181	23182.00
gag	E	139	16138.00
ggt	G	0	0.13
ggc	G	0	0.14
gga	G	1	0.46
ggg	G	0	0.26
---	--	------	------
mPD		0.0071	0.48
		nPD:	0.01
		N. weight:	0.56
		Sc. PD:	-0.026
		Sc. rank:	-4086.9

PB1			
Pos.	110	obs:	exp:
att	I	0	0.35
atc	I	0	0.26
ata	I	1	0.39
act	T	26	12209.00
acc	T	1	6951.00
aca	T	39201	17015.00
acg	T	87	3140.00
gct	A	0	1.64
gcc	A	0	1.50
gca	A	2	2.43
gcg	A	4	0.44
---	--	------	------
mPD		0.0063	0.68
		nPD:	0.01
		N. weight:	0.9
		Sc. PD:	-0.047
		Sc. rank:	-12764.0

PB1			
Pos.	111	obs:	exp:
tta	L	0	0.23
ttg	L	2	0.41
ctt	L	0	0.42
ctc	L	0	0.29
cta	L	0	0.31
ctg	L	0	0.34
cgt	R	0	0.02
cgc	R	0	0.04
cga	R	0	0.09
cgg	R	0	0.09
att	I	8	916.90
atc	I	1	686.30
ata	I	2603	1009.00
atg	M	36704	36704.00
act	T	0	0.31
acc	T	0	0.18
aca	T	0	0.43
acg	T	1	0.08
aaa	K	0	0.66
aag	K	1	0.34
aga	R	0	0.49
agg	R	1	0.28
gtt	V	0	0.25
gtc	V	0	0.20
gta	V	1	0.22
gtg	V	0	0.33
---	--	------	------
mPD		0.12	0.13
		nPD:	0.98
		N. weight:	0.068
		Sc. PD:	0.061
		Sc. rank:	2104.5

PB1			
Pos.	112	obs:	exp:
aaa	K	4	2.63
aag	K	0	1.37
gat	D	3	2.83
gac	D	2	2.17
gaa	E	39174	23176.00
gag	E	137	16135.00
ggt	G	0	0.27
ggc	G	0	0.28
gga	G	2	0.92
ggg	G	0	0.53
---	--	------	------
mPD		0.0075	0.48
		nPD:	0.02
		N. weight:	0.56
		Sc. PD:	-0.025
		Sc. rank:	-3678.4

PB1			
Pos.	113	obs:	exp:
ttt	F	1	0.49
ttc	F	0	0.51
tct	S	0	3.23
tcc	S	19	2.99
tca	S	1	5.38
tcg	S	0	0.79
att	I	174	66.70
atc	I	16	49.92
ata	I	0	73.38
act	T	0	9.01
acc	T	29	5.13
aca	T	0	12.55
acg	T	0	2.32
agt	S	0	3.50
agc	S	0	4.13
gtt	V	122	185.60
gtc	V	616	151.00
gta	V	3	160.60
gtg	V	0	243.80
gct	A	108	10450.00
gcc	A	37799	9579.00
gca	A	431	15509.00
gcg	A	2	2803.00
gat	D	0	0.57
gac	D	1	0.43
---	--	------	------
mPD		0.10	0.76
		nPD:	0.13
		N. weight:	1.4
		Sc. PD:	0.098
		Sc. rank:	34218.0

PB1			
Pos.	114	obs:	exp:
ttt	F	1	0.49
ttc	F	0	0.51
att	I	221	80.04
atc	I	7	59.91
ata	I	0	88.05
gtt	V	38733	9790.00
gtc	V	344	7964.00
gta	V	3	8471.00
gtg	V	4	12860.00
gct	A	9	2.45
gcc	A	0	2.25
gca	A	0	3.64
gcg	A	0	0.66
---	--	------	------
mPD		0.030	0.75
		nPD:	0.04
		N. weight:	1.5
		Sc. PD:	-0.032
		Sc. rank:	14318.0

PB1

Pos.	115	obs:	exp:
caa	Q	38157	24037.00
cag	Q	1165	15285.00
---	--	------	------
mPD		0.058	0.48
		nPD:	0.12
		N. weight:	0.41
		Sc. PD:	0.023
		Sc. rank:	9502.7

PB1

Pos.	116	obs:	exp:
tta	L	0	0.12
ttg	L	0	0.21
ctt	L	0	0.21
ctc	L	0	0.15
cta	L	0	0.15
ctg	L	1	0.17
cat	H	0	0.56
cac	H	1	0.44
caa	Q	2152	24033.00
cag	Q	37164	15283.00
cgt	R	0	0.02
cgc	R	0	0.04
cga	R	0	0.09
cgg	R	1	0.09
aaa	K	0	1.31
aag	K	2	0.69
aga	R	0	0.49
agg	R	0	0.28
gaa	E	0	0.59
gag	E	1	0.41
---	--	------	------
mPD		0.10	0.48
		nPD:	0.22
		N. weight:	0.78
		Sc. PD:	0.12
		Sc. rank:	20716.0

PB1

Pos.	117	obs:	exp:
act	T	31	12210.00
acc	T	3	6951.00
aca	T	39227	17016.00
acg	T	57	3140.00
gct	A	0	1.09
gcc	A	0	1.00
gca	A	4	1.62
gcg	A	0	0.29
---	--	------	------
mPD		0.0048	0.68
		nPD:	0.01
		N. weight:	0.9
		Sc. PD:	-0.049
		Sc. rank:	-16806.0

PB1

Pos.	118	obs:	exp:
cgt	R	0	779.60
cgc	R	0	1415.00
cga	R	3	3587.00
cgg	R	5	3376.00
aaa	K	0	2.63
aag	K	4	1.37
aga	R	422	19285.00
agg	R	38888	10875.00
---	--	------	------
mPD		0.022	0.89
		nPD:	0.02
		N. weight:	1.3
		Sc. PD:	-0.049
		Sc. rank:	2242.9

PB1

Pos.	119	obs:	exp:
att	I	0	2.46
atc	I	0	1.84
ata	I	7	2.70
atg	M	11	11.00
gtt	V	72	9845.00
gtc	V	1	8008.00
gta	V	11381	8519.00
gtg	V	27850	12932.00
---	--	------	------
mPD		0.42	0.74
		nPD:	0.56
		N. weight:	0.68
		Sc. PD:	0.33
		Sc. rank:	20391.0

PB1

Pos.	120	obs:	exp:
gat	D	315	22227.00
gac	D	39006	17094.00
gaa	E	0	0.59
gag	E	1	0.41
---	--	------	------
mPD		0.016	0.49
		nPD:	0.03
		N. weight:	0.86
		Sc. PD:	-0.025
		Sc. rank:	5729.9

PB1

Pos.	121	obs:	exp:
cgt	R	0	1.49
cgc	R	0	2.70
cga	R	0	6.84
cgg	R	0	6.44
aat	N	0	1.12
aac	N	2	0.88
aaa	K	39210	25800.00
aag	K	34	13444.00
aga	R	75	36.79
agg	R	0	20.75
gaa	E	1	0.59
gag	E	0	0.41
---	--	------	------
mPD		0.0057	0.46
		nPD:	0.01
		N. weight:	0.45
		Sc. PD:	-0.022
		Sc. rank:	-4764.2

PB1

Pos.	122	obs:	exp:
tta	L	58	4562.00
ttg	L	0	8129.00
ctt	L	3	8225.00
ctc	L	6	5766.00
cta	L	38959	6038.00
ctg	L	292	6599.00
att	I	0	1.05
atc	I	0	0.79
ata	I	3	1.16
ggt	G	0	0.13
ggc	G	0	0.14
gga	G	1	0.46
ggg	G	0	0.26
---	--	------	------
mPD		0.018	1.2
		nPD:	0.02
		N. weight:	2.
		Sc. PD:	-0.091
		Sc. rank:	-11840.0

PB1

Pos.	123	obs:	exp:
att	I	0	0.70
atc	I	2	0.53
ata	I	0	0.77
act	T	628	12208.00
acc	T	38546	6950.00
aca	T	138	17013.00
acg	T	0	3140.00
gct	A	0	2.18
gcc	A	8	2.00
gca	A	0	3.24
gcg	A	0	0.58
---	--	------	------
mPD		0.039	0.68
		nPD:	0.06
		N. weight:	1.8
		Sc. PD:	-0.0093
		Sc. rank:	28457.0

PB1

Pos.	124	obs:	exp:
tta	L	0	0.12
ttg	L	0	0.21
ctt	L	0	0.21
ctc	L	0	0.15
cta	L	1	0.15
ctg	L	0	0.17
cat	H	0	0.56
cac	H	1	0.44
caa	Q	38950	24033.00
cag	Q	366	15283.00
cgt	R	0	0.02
cgc	R	0	0.04
cga	R	1	0.09
cgg	R	0	0.09
aaa	K	3	1.97
aag	K	0	1.03
aga	R	0	0.49
agg	R	0	0.28
---	--	------	------
mPD		0.019	0.48
		nPD:	0.04
		N. weight:	0.49
		Sc. PD:	-0.011
		Sc. rank:	4637.1

PB1

Pos.	125	obs:	exp:
cgt	R	1	0.02
cgc	R	0	0.04
cga	R	0	0.09
cgg	R	0	0.09
aga	R	0	0.49
agg	R	0	0.28
ggt	G	36550	5294.00
ggc	G	240	5488.00
gga	G	2527	18151.00
ggg	G	4	10388.00
---	--	------	------
mPD		0.13	0.68
		nPD:	0.19
		N. weight:	1.8
		Sc. PD:	0.23
		Sc. rank:	47193.0

PB1

Pos.	126	obs:	exp:
cat	H	0	6.77
cac	H	12	5.23
cgt	R	205	779.40
cgc	R	39089	1414.00
cga	R	13	3587.00
cgg	R	3	3375.00
aga	R	0	19281.00
agg	R	0	10873.00
---	--	------	------
mPD		0.012	0.89
		nPD:	0.01
		N. weight:	3.6
		Sc. PD:	-0.17
		Sc. rank:	-32926.0

PB1

Pos.	127	obs:	exp:
caa	Q	1933	24037.00
cag	Q	37389	15285.00
---	--	------	------
mPD		0.093	0.48
		nPD:	0.2
		N. weight:	0.8
		Sc. PD:	0.1
		Sc. rank:	20841.0

PB1

Pos.	128	obs:	exp:
tct	S	1	0.16
tcc	S	0	0.15
tca	S	0	0.27
tcg	S	0	0.04
act	T	38350	12211.00
acc	T	789	6952.00
aca	T	173	17017.00
acg	T	9	3141.00
agt	S	0	0.17
agc	S	0	0.21
---	--	------	------
mPD		0.048	0.68
		nPD:	0.07
		N. weight:	1.2
		Sc. PD:	0.0099
		Sc. rank:	21192.0

PB1

Pos.	129	obs:	exp:
ttt	F	1	0.49
ttc	F	0	0.51
tat	Y	39280	20725.00
tac	Y	39	18594.00
cat	H	2	1.13
cac	H	0	0.87
---	--	------	------
mPD		0.0021	0.50
		nPD:	0.
		N. weight:	0.69
		Sc. PD:	-0.039
		Sc. rank:	-16792.0

PB1

Pos.	130	obs:	exp:
gat	D	39165	22227.00
gac	D	156	17094.00
ggt	G	0	0.13
ggc	G	0	0.14
gga	G	0	0.46
ggg	G	1	0.26
---	--	------	------
mPD		0.0080	0.49
		nPD:	0.02
		N. weight:	0.6
		Sc. PD:	-0.027
		Sc. rank:	-3437.4

PB1			
Pos.	131	obs:	exp:
tgg	W	39321	39321.00
---	-	1	1.00
---	--	------	------
mPD		0	0
		nPD:	1.
		N. weight:	0.
		Sc. PD:	0
		Sc. rank:	0

,

PB1			
Pos.	132	obs:	exp:
act	T	13	12212.00
acc	T	26	6952.00
aca	T	39264	17018.00
acg	T	19	3141.00
---	--	------	------
mPD		0.0029	0.68
		nPD:	0.
		N. weight:	0.91
		Sc. PD:	-0.051
		Sc. rank:	-21697.0

,

PB1			
Pos.	133	obs:	exp:
tta	L	39101	4562.00
ttg	L	138	8128.00
tct	S	0	0.16
tcc	S	0	0.15
tca	S	1	0.27
tcg	S	0	0.04
ctt	L	0	8225.00
ctc	L	0	5765.00
cta	L	76	6037.00
ctg	L	1	6598.00
cct	P	0	0.33
ccc	P	0	0.19
cca	P	1	0.34
ccg	P	0	0.14
att	I	0	0.35
atc	I	0	0.26
ata	I	1	0.39
agt	S	0	0.17
agc	S	0	0.21
gtt	V	0	0.75
gtc	V	0	0.61
gta	V	3	0.65
gtg	V	0	0.99
---	--	------	------
mPD		0.011	1.2
		nPD:	0.01
		N. weight:	2.3
		Sc. PD:	-0.12
		Sc. rank:	-31268.0

,

PB1			
Pos.	134	obs:	exp:
tct	S	0	0.65
tcc	S	0	0.60
tca	S	0	1.08
tcg	S	0	0.16
cat	H	0	0.56
cac	H	1	0.44
act	T	0	1.55
acc	T	5	0.88
aca	T	0	2.16
acg	T	0	0.40
aat	N	332	21992.00
aac	N	38976	17316.00
aaa	K	1	0.66
aag	K	0	0.34
agt	S	0	0.70
agc	S	4	0.83
gat	D	0	1.70
gac	D	3	1.30
---	--	------	------
mPD		0.017	0.49
		nPD:	0.04
		N. weight:	0.84
		Sc. PD:	-0.022
		Sc. rank:	6986.4

,

PB1			
Pos.	135	obs:	exp:
cgt	R	0	779.10
cgc	R	0	1414.00
cga	R	1	3585.00
cgg	R	1	3374.00
aaa	K	2	17.09
aag	K	24	8.91
aga	R	4734	19274.00
agg	R	34560	10869.00
---	--	------	------
mPD		0.21	0.89
		nPD:	0.24
		N. weight:	0.93
		Sc. PD:	0.16
		Sc. rank:	25218.0

,

PB1			
Pos.	136	obs:	exp:
aat	N	5624	21999.00
aac	N	33697	17322.00
aaa	K	1	0.66
aag	K	0	0.34
---	--	------	------
mPD		0.25	0.49
		nPD:	0.5
		N. weight:	0.41
		Sc. PD:	0.17
		Sc. rank:	12237.0

,

PB1			
Pos.	137	obs:	exp:
tta	L	0	0.12
ttg	L	0	0.21
ctt	L	0	0.21
ctc	L	0	0.15
cta	L	1	0.15
ctg	L	0	0.17
cat	H	0	0.56
cac	H	1	0.44
caa	Q	39128	24035.00
cag	Q	192	15285.00
---	--	------	------
mPD		0.0098	0.48
		nPD:	0.02
		N. weight:	0.51
		Sc. PD:	-0.021
		Sc. rank:	-937.3

,

PB1			
Pos.	138	obs:	exp:
tta	L	0	0.23
ttg	L	0	0.41
tct	S	0	0.81
tcc	S	0	0.75
tca	S	0	1.34
tcg	S	5	0.20
ctt	L	0	0.42
ctc	L	0	0.29
cta	L	0	0.31
ctg	L	2	0.34
cct	P	49	13100.00
ccc	P	1	7370.00
cca	P	309	13282.00
ccg	P	38953	5560.00
caa	Q	0	1.22
cag	Q	2	0.78
act	T	0	0.31
acc	T	0	0.18
aca	T	0	0.43
acg	T	1	0.08
agt	S	0	0.87
agc	S	0	1.03
---	--	------	------
mPD		0.019	0.72
		nPD:	0.03
		N. weight:	2.1
		Sc. PD:	-0.074
		Sc. rank:	6844.8

,

PB1			
Pos.	139	obs:	exp:
gtt	V	0	0.25
gtc	V	0	0.20
gta	V	1	0.22
gtg	V	0	0.33
gct	A	27	10717.00
gcc	A	65	9824.00
gca	A	39046	15906.00
gcg	A	183	2874.00
---	--	------	------
mPD		0.014	0.69
		nPD:	0.02
		N. weight:	0.95
		Sc. PD:	-0.039
		Sc. rank:	-2127.3

,

PB1			
Pos.	140	obs:	exp:
tct	S	0	0.97
tcc	S	0	0.90
tca	S	6	1.61
tcg	S	0	0.24
agt	S	0	1.05
agc	S	0	1.24
gct	A	5	10716.00
gcc	A	2	9823.00
gca	A	39292	15904.00
gcg	A	17	2874.00
---	--	------	------
mPD		0.0015	0.69
		nPD:	0.
		N. weight:	0.99
		Sc. PD:	-0.058
		Sc. rank:	-27914.0

,

PB1			
Pos.	141	obs:	exp:
act	T	37993	12208.00
acc	T	23	6950.00
aca	T	468	17013.00
acg	T	828	3140.00
aat	N	8	4.48
aac	N	0	3.52
gct	A	2	0.55
gcc	A	0	0.50
gca	A	0	0.81
gcg	A	0	0.15
---	--	------	------
mPD		0.066	0.68
		nPD:	0.1
		N. weight:	1.1
		Sc. PD:	0.038
		Sc. rank:	24191.0

,

PB1			
Pos.	142	obs:	exp:
tct	S	0	0.48
tcc	S	0	0.45
tca	S	3	0.81
tcg	S	0	0.12
cct	P	0	0.33
ccc	P	0	0.19
cca	P	1	0.34
ccg	P	0	0.14
act	T	0	0.31
acc	T	0	0.18
aca	T	1	0.43
acg	T	0	0.08
agt	S	0	0.52
agc	S	0	0.62
gct	A	15	10716.00
gcc	A	3	9823.00
gca	A	39209	15904.00
gcg	A	89	2874.00
gaa	E	1	0.59
gag	E	0	0.41
---	--	------	------
mPD		0.0057	0.69
		nPD:	0.01
		N. weight:	0.98
		Sc. PD:	-0.052
		Sc. rank:	-15840.0

,

PB1

Pos. 143		obs:	exp:
tta	L	38715	4563.00
ttg	L	516	8129.00
tct	S	1	0.16
tcc	S	0	0.15
tca	S	0	0.27
tcg	S	0	0.04
ctt	L	0	8226.00
ctc	L	0	5766.00
cta	L	89	6038.00
ctg	L	1	6599.00
agt	S	0	0.17
agc	S	0	0.21
mPD		0.031	1.2
		nPD:	0.03
		N. weight:	2.3
		Sc. PD:	-0.08
		Sc. rank:	9408.3

PB1

Pos. 144		obs:	exp:
tct	S	0	0.16
tcc	S	1	0.15
tca	S	0	0.27
tcg	S	0	0.04
act	T	0	2.17
acc	T	7	1.24
aca	T	0	3.03
acg	T	0	0.56
agt	S	0	0.17
agc	S	0	0.21
gtt	V	0	0.75
gtc	V	2	0.61
gta	V	1	0.65
gtg	V	0	0.99
gct	A	80	10714.00
gcc	A	39211	9821.00
gca	A	20	15901.00
gcg	A	0	2874.00
mPD		0.0057	0.69
		nPD:	0.01
		N. weight:	1.5
		Sc. PD:	-0.08
		Sc. rank:	-24574.0

PB1

Pos. 145		obs:	exp:
tct	S	0	0.16
tcc	S	0	0.15
tca	S	0	0.27
tcg	S	0	0.04
aat	N	48	21999.00
aac	N	39273	17322.00
agt	S	0	0.17
agc	S	1	0.21
mPD		0.0025	0.49
		nPD:	0.01
		N. weight:	0.89
		Sc. PD:	-0.05
		Sc. rank:	-19984.0

PB1

Pos. 146		obs:	exp:
tct	S	0	0.16
tcc	S	0	0.15
tca	S	0	0.27
tcg	S	0	0.04
att	I	0	1.05
atc	I	3	0.79
ata	I	0	1.16
act	T	191	12210.00
acc	T	39090	6951.00
aca	T	36	17015.00
acg	T	0	3140.00
agt	S	0	0.17
agc	S	1	0.21
gct	A	0	0.27
gcc	A	1	0.25
gca	A	0	0.40
gcg	A	0	0.07
mPD		0.012	0.68
		nPD:	0.02
		N. weight:	1.9
		Sc. PD:	-0.082
		Sc. rank:	-8028.5

PB1

Pos. 147		obs:	exp:
tta	L	0	0.12
ttg	L	0	0.21
ctt	L	0	0.21
ctc	L	0	0.15
cta	L	1	0.15
ctg	L	0	0.17
att	I	5	13801.00
atc	I	6	10330.00
ata	I	39304	15183.00
atg	M	2	2.00
gtt	V	0	1.00
gtc	V	0	0.82
gta	V	4	0.87
gtg	V	0	1.32
mPD		0.00092	0.66
		nPD:	0.
		N. weight:	1.
		Sc. PD:	-0.062
		Sc. rank:	-30777.0

PB1

Pos. 148		obs:	exp:
gaa	E	39233	23183.00
gag	E	89	16139.00
mPD		0.0045	0.48
		nPD:	0.01
		N. weight:	0.56
		Sc. PD:	-0.029
		Sc. rank:	-8027.3

PB1

Pos. 149		obs:	exp:
att	I	11	34.05
atc	I	86	25.49
ata	I	0	37.46
gtt	V	1870	9825.00
gtc	V	37290	7992.00
gta	V	60	8502.00
gtg	V	5	12906.00
mPD		0.099	0.75
		nPD:	0.13
		N. weight:	1.5
		Sc. PD:	0.1
		Sc. rank:	36203.0

PB1

Pos. 150		obs:	exp:
ttt	F	39072	19125.00
ttc	F	246	20193.00
tta	L	1	0.35
ttg	L	0	0.62
tat	Y	1	0.53
tac	Y	0	0.47
ctt	L	2	0.63
ctc	L	0	0.44
cta	L	0	0.46
ctg	L	0	0.50
mPD		0.013	0.50
		nPD:	0.03
		N. weight:	0.75
		Sc. PD:	-0.027
		Sc. rank:	2081.3

PB1

Pos. 151		obs:	exp:
cgt	R	0	778.40
cgc	R	0	1413.00
cga	R	1	3582.00
cgg	R	0	3371.00
att	I	0	0.35
atc	I	0	0.26
ata	I	1	0.39
aaa	K	56	36.82
aag	K	0	19.18
aga	R	38898	19257.00
agg	R	362	10860.00
ggt	G	0	0.54
ggc	G	0	0.56
gga	G	4	1.85
ggg	G	0	1.06
mPD		0.021	0.89
		nPD:	0.02
		N. weight:	0.73
		Sc. PD:	-0.027
		Sc. rank:	882.7

PB1

Pos. 152		obs:	exp:
tta	L	0	4.06
ttg	L	35	7.24
tct	S	26	6336.00
tcc	S	2	5867.00
tca	S	4197	10553.00
tcg	S	35037	1545.00
ctt	L	0	7.32
ctc	L	0	5.13
cta	L	0	5.37
ctg	L	0	5.87
act	T	0	7.45
acc	T	0	4.24
aca	T	4	10.39
acg	T	20	1.92
agt	S	0	6861.00
agc	S	0	8100.00
gct	A	0	0.27
gcc	A	0	0.25
gca	A	0	0.40
gcg	A	1	0.07
mPD		0.20	1.6
		nPD:	0.12
		N. weight:	2.9
		Sc. PD:	0.16
		Sc. rank:	68072.0

PB1

Pos. 153		obs:	exp:
tat	Y	0	0.53
tac	Y	1	0.47
aat	N	1360	21999.00
aac	N	37961	17322.00
mPD		0.067	0.49
		nPD:	0.14
		N. weight:	0.73
		Sc. PD:	0.052
		Sc. rank:	17561.0

PB1

Pos. 154		obs:	exp:
cgt	R	0	2.16
cgc	R	0	3.92
cga	R	0	9.95
cgg	R	0	9.36
aaa	K	1	0.66
aag	K	0	0.34
aga	R	109	53.46
agg	R	0	30.15
gat	D	0	9.61
gac	D	17	7.39
gaa	E	33	19.46
gag	E	0	13.54
ggt	G	639	5272.00
ggc	G	21	5466.00
gga	G	38328	18078.00
ggg	G	174	10346.00
mPD		0.051	0.69
		nPD:	0.07
		N. weight:	0.75
		Sc. PD:	0.0082
		Sc. rank:	14477.0

PB1			
Pos.	155	obs:	exp:
ttt	F	2	0.97
ttc	F	0	1.03
tta	L	37657	4561.00
ttg	L	666	8127.00
tct	S	0	0.16
tcc	S	0	0.15
tca	S	1	0.27
tcg	S	0	0.04
ctt	L	0	8223.00
ctc	L	11	5764.00
cta	L	971	6036.00
ctg	L	3	6597.00
att	I	0	3.86
atc	I	0	2.89
ata	I	11	4.25
agt	S	0	0.17
agc	S	0	0.21
---	--	------	------
mPD		0.084	1.2
		nPD:	0.07
		N. weight:	2.1
		Sc. PD:	0.02
		Sc. rank:	40181.0

PB1			
Pos.	156	obs:	exp:
cct	P	0	0.33
ccc	P	0	0.19
cca	P	1	0.34
ccg	P	0	0.14
cgt	R	0	0.02
cgc	R	0	0.04
cga	R	0	0.09
cgg	R	0	0.09
att	I	0	5.97
atc	I	0	4.47
ata	I	17	6.57
act	T	4	12198.00
acc	T	2	6944.00
aca	T	39220	16998.00
acg	T	51	3137.00
aaa	K	7	4.60
aag	K	0	2.40
aga	R	1	0.49
agg	R	0	0.28
gct	A	0	5.18
gcc	A	0	4.75
gca	A	19	7.69
gcg	A	0	1.39
---	--	------	------
mPD		0.0052	0.68
		nPD:	0.01
		N. weight:	0.91
		Sc. PD:	-0.049
		Sc. rank:	-15969.0

PB1			
Pos.	157	obs:	exp:
tct	S	5	0.97
tcc	S	1	0.90
tca	S	0	1.61
tcg	S	0	0.24
act	T	73	25.47
acc	T	9	14.50
aca	T	0	35.49
acg	T	0	6.55
aat	N	1	0.56
aac	N	0	0.44
agt	S	0	1.05
agc	S	0	1.24
gtt	V	7	2.25
gtc	V	2	1.83
gta	V	0	1.95
gtg	V	0	2.96
gct	A	34806	10688.00
gcc	A	4390	9797.00
gca	A	17	15862.00
gcg	A	1	2867.00
gat	D	10	5.65
gac	D	0	4.35
---	--	------	------
mPD		0.21	0.70
		nPD:	0.29
		N. weight:	1.
		Sc. PD:	0.24
		Sc. rank:	29050.0

PB1			
Pos.	158	obs:	exp:
tct	S	0	10.81
tcc	S	0	10.01
tca	S	0	18.01
tcg	S	0	2.64
act	T	6	1.86
acc	T	0	1.06
aca	T	0	2.60
acg	T	0	0.48
aat	N	36799	21958.00
aac	N	2449	17290.00
aaa	K	1	0.66
aag	K	0	0.34
agt	S	66	11.71
agc	S	1	13.82
---	--	------	------
mPD		0.12	0.50
		nPD:	0.24
		N. weight:	0.4
		Sc. PD:	0.07
		Sc. rank:	10858.0

PB1			
Pos.	159	obs:	exp:
gtt	V	0	0.25
gtc	V	0	0.20
gta	V	1	0.22
gtg	V	0	0.33
gat	D	0	0.57
gac	D	1	0.43
gaa	E	38762	23174.00
gag	E	545	16133.00
ggt	G	0	1.75
ggc	G	0	1.81
gga	G	13	6.00
ggg	G	0	3.43
---	--	------	------
mPD		0.028	0.48
		nPD:	0.06
		N. weight:	0.51
		Sc. PD:	-0.0023
		Sc. rank:	8248.5

PB1			
Pos.	160	obs:	exp:
tct	S	14	6345.00
tcc	S	3	5876.00
tca	S	39089	10569.00
tcg	S	215	1548.00
act	T	0	0.31
acc	T	0	0.18
aca	T	1	0.43
acg	T	0	0.08
agt	S	0	6871.00
agc	S	0	8112.00
---	--	------	------
mPD		0.012	1.6
		nPD:	0.01
		N. weight:	1.4
		Sc. PD:	-0.076
		Sc. rank:	-26137.0

PB1			
Pos.	161	obs:	exp:
ggt	G	1	5294.00
ggc	G	0	5488.00
gga	G	39154	18152.00
ggg	G	167	10388.00
---	--	------	------
mPD		0.0085	0.68
		nPD:	0.01
		N. weight:	0.82
		Sc. PD:	-0.04
		Sc. rank:	-8528.3

PB1			
Pos.	162	obs:	exp:
cgt	R	0	779.70
cgc	R	0	1415.00
cga	R	2	3588.00
cgg	R	19	3376.00
aga	R	819	19287.00
agg	R	38482	10876.00
---	--	------	------
mPD		0.042	0.89
		nPD:	0.05
		N. weight:	1.3
		Sc. PD:	-0.019
		Sc. rank:	16654.0

PB1			
Pos.	163	obs:	exp:
tta	L	259	4563.00
ttg	L	0	8129.00
ctt	L	7	8225.00
ctc	L	5	5766.00
cta	L	38742	6038.00
ctg	L	307	6599.00
att	I	0	0.70
atc	I	0	0.53
ata	I	2	0.77
---	--	------	------
mPD		0.029	1.2
		nPD:	0.03
		N. weight:	2.
		Sc. PD:	-0.071
		Sc. rank:	5067.3

PB1			
Pos.	164	obs:	exp:
tta	L	1	0.12
ttg	L	0	0.21
ctt	L	0	0.21
ctc	L	0	0.15
cta	L	0	0.15
ctg	L	0	0.17
att	I	75	13786.00
atc	I	2	10319.00
ata	I	39195	15167.00
atg	M	6	6.00
gtt	V	0	10.77
gtc	V	0	8.76
gta	V	43	9.32
gtg	V	0	14.15
---	--	------	------
mPD		0.0064	0.66
		nPD:	0.01
		N. weight:	1.
		Sc. PD:	-0.053
		Sc. rank:	-14068.0

PB1			
Pos.	165	obs:	exp:
gat	D	39026	22227.00
gac	D	295	17094.00
gaa	E	1	0.59
gag	E	0	0.41
---	--	------	------
mPD		0.015	0.49
		nPD:	0.03
		N. weight:	0.58
		Sc. PD:	-0.018
		Sc. rank:	3352.1

PB1			
Pos.	166	obs:	exp:
ttt	F	2088	19119.00
ttc	F	37218	20187.00
tta	L	1	1.28
ttg	L	1	2.27
tat	Y	0	2.64
tac	Y	5	2.36
ctt	L	0	2.30
ctc	L	9	1.61
cta	L	0	1.69
ctg	L	0	1.85
---	--	------	------
mPD		0.10	0.50
		nPD:	0.2
		N. weight:	0.51
		Sc. PD:	0.069
		Sc. rank:	13272.0

PB1			
Pos.	167	obs:	exp:
tta	L	197	4563.00
ttg	L	0	8129.00
ctt	L	55	8226.00
ctc	L	38993	5766.00
cta	L	75	6038.00
ctg	L	1	6599.00
cct	P	0	0.33
ccc	P	1	0.19
cca	P	0	0.34
ccg	P	0	0.14
---	--	------	------
mPD		0.027	1.2
		nPD:	0.02
		N. weight:	2.
		Sc. PD:	-0.079
		Sc. rank:	1130.4

PB1			
Pos.	168	obs:	exp:
cgt	R	0	0.34
cgc	R	0	0.61
cga	R	0	1.55
cgg	R	0	1.46
aaa	K	2213	25840.00
aag	K	37091	13464.00
aga	R	2	8.34
agg	R	15	4.70
gaa	E	0	0.59
gag	E	1	0.41
---	--	------	------
mPD		0.11	0.45
		nPD:	0.24
		N. weight:	0.9
		Sc. PD:	0.15
		Sc. rank:	24263.0

PB1			
Pos.	169	obs:	exp:
gat	D	39282	22227.00
gac	D	40	17095.00
---	--	------	------
mPD		0.0020	0.49
		nPD:	0.
		N. weight:	0.62
		Sc. PD:	-0.035
		Sc. rank:	-15252.0

PB1			
Pos.	170	obs:	exp:
att	I	0	0.35
atc	I	0	0.26
ata	I	1	0.39
atg	M	2	2.00
gtt	V	17	9849.00
gtc	V	0	8012.00
gta	V	409	8522.00
gtg	V	38893	12937.00
---	--	------	------
mPD		0.022	0.74
		nPD:	0.03
		N. weight:	1.2
		Sc. PD:	-0.038
		Sc. rank:	6291.7

PB1

Pos.	171	obs:	exp:
tta	L	0	1.16
ttg	L	8	2.07
ctt	L	0	2.09
ctc	L	0	1.47
cta	L	0	1.54
ctg	L	2	1.68
att	I	13	17.55
atc	I	0	13.14
ata	I	37	19.31
atg	M	38946	38946.00
act	T	0	2.17
acc	T	0	1.24
aca	T	0	3.03
acg	T	7	0.56
gtt	V	0	77.40
gtc	V	0	62.96
gta	V	0	66.97
gtg	V	309	101.70
---	--	------	------
mPD		0.019	0.030
		nPD:	0.63
		N. weight:	0.011
		Sc. PD:	0.0061
		Sc. rank:	334.3

,

PB1

Pos.	172	obs:	exp:
aaa	K	25	16.44
aag	K	0	8.56
gtt	V	0	2.00
gtc	V	0	1.63
gta	V	8	1.73
gtg	V	0	2.63
gct	A	0	0.55
gcc	A	0	0.50
gca	A	2	0.81
gcg	A	0	0.15
gat	D	8	8.48
gac	D	7	6.52
gaa	E	39175	23129.00
gag	E	56	16102.00
ggt	G	0	5.52
ggc	G	0	5.72
gga	G	41	18.93
ggg	G	0	10.83
---	--	------	------
mPD		0.0075	0.49
		nPD:	0.02
		N. weight:	0.57
		Sc. PD:	-0.026
		Sc. rank:	-3863.5

,

PB1

Pos.	173	obs:	exp:
tct	S	5	6345.00
tcc	S	1	5876.00
tca	S	39301	10569.00
tcg	S	15	1548.00
agt	S	0	6872.00
agc	S	0	8112.00
---	--	------	------
mPD		0.0011	1.6
		nPD:	0.
		N. weight:	1.4
		Sc. PD:	-0.087
		Sc. rank:	-43986.0

,

PB1

Pos.	174	obs:	exp:
tta	L	0	1.04
ttg	L	3	1.86
ctt	L	0	1.88
ctc	L	0	1.32
cta	L	0	1.38
ctg	L	6	1.51
att	I	3	12.99
atc	I	0	9.72
ata	I	34	14.29
atg	M	39268	39268.00
act	T	0	1.24
acc	T	0	0.71
aca	T	0	1.73
acg	T	4	0.32
gtt	V	0	1.00
gtc	V	0	0.82
gta	V	0	0.87
gtg	V	4	1.32
---	--	------	------
mPD		0.0027	0.0034
		nPD:	0.82
		N. weight:	0.0015
		Sc. PD:	0.0011
		Sc. rank:	44.8

,

PB1

Pos.	175	obs:	exp:
aat	N	294	193.60
aac	N	52	152.40
gat	D	34703	22023.00
gac	D	4258	16938.00
gaa	E	9	6.49
gag	E	2	4.51
ggt	G	3	0.54
ggc	G	1	0.56
gga	G	0	1.85
ggg	G	0	1.06
---	--	------	------
mPD		0.21	0.51
		nPD:	0.42
		N. weight:	0.28
		Sc. PD:	0.097
		Sc. rank:	8118.1

,

PB1

Pos.	176	obs:	exp:
caa	Q	1	1.22
cag	Q	1	0.78
cgt	R	0	0.42
cgc	R	0	0.76
cga	R	0	1.92
cgg	R	0	1.80
atg	M	1	1.00
act	T	0	0.31
acc	T	0	0.18
aca	T	0	0.43
acg	T	1	0.08
aat	N	0	1.68
aac	N	3	1.32
aaa	K	10277	25833.00
aag	K	29016	13460.00
aga	R	5	10.30
agg	R	16	5.81
gaa	E	0	0.59
gag	E	1	0.41
---	--	------	------
mPD		0.39	0.45
		nPD:	0.86
		N. weight:	0.36
		Sc. PD:	0.28
		Sc. rank:	11079.0

,

PB1

Pos.	177	obs:	exp:
aaa	K	1	50.62
aag	K	76	26.38
gtt	V	0	0.25
gtc	V	0	0.20
gta	V	0	0.22
gtg	V	1	0.33
gat	D	14	7.91
gac	D	0	6.09
gaa	E	279	23110.00
gag	E	38920	16089.00
ggt	G	0	4.17
ggc	G	0	4.33
gga	G	0	14.31
ggg	G	31	8.19
---	--	------	------
mPD		0.020	0.49
		nPD:	0.04
		N. weight:	0.93
		Sc. PD:	-0.019
		Sc. rank:	9670.7

,

PB1

Pos.	178	obs:	exp:
caa	Q	1	0.61
cag	Q	0	0.39
aaa	K	19	12.49
aag	K	0	6.51
gtt	V	0	0.25
gtc	V	0	0.20
gta	V	1	0.22
gtg	V	0	0.33
gat	D	1	42.96
gac	D	75	33.04
gaa	E	39032	23109.00
gag	E	165	16088.00
ggt	G	0	3.77
ggc	G	0	3.91
gga	G	28	12.93
ggg	G	0	7.40
---	--	------	------
mPD		0.015	0.49
		nPD:	0.03
		N. weight:	0.55
		Sc. PD:	-0.017
		Sc. rank:	3141.5

,

PB1

Pos.	179	obs:	exp:
tta	L	0	0.70
ttg	L	2	1.24
ctt	L	0	1.26
ctc	L	0	0.88
cta	L	0	0.92
ctg	L	4	1.01
att	I	95	227.80
atc	I	0	170.50
ata	I	554	250.60
atg	M	38656	38656.00
act	T	0	0.62
acc	T	0	0.35
aca	T	0	0.87
acg	T	2	0.16
gtt	V	0	2.25
gtc	V	0	1.83
gta	V	0	1.95
gtg	V	9	2.96
---	--	------	------
mPD		0.033	0.034
		nPD:	0.98
		N. weight:	0.011
		Sc. PD:	0.0095
		Sc. rank:	330.2

,

PB1

Pos.	180	obs:	exp:
aaa	K	1	10.52
aag	K	15	5.48
gtt	V	0	1.00
gtc	V	0	0.82
gta	V	0	0.87
gtg	V	4	1.32
gat	D	3	1.70
gac	D	0	1.30
gaa	E	174	23165.00
gag	E	39118	16127.00
ggt	G	0	0.94
ggc	G	0	0.98
gga	G	0	3.23
ggg	G	7	1.85
---	--	------	------
mPD		0.010	0.49
		nPD:	0.02
		N. weight:	0.94
		Sc. PD:	-0.038
		Sc. rank:	-1034.5

,

PB1

Pos.	181	obs:	exp:
tta	L	1	0.23
ttg	L	0	0.41
ctt	L	0	0.42
ctc	L	0	0.29
cta	L	1	0.31
ctg	L	0	0.34
att	I	0	13720.00
atc	I	11	10270.00
ata	I	39072	15094.00
atg	M	17	17.00
gtt	V	0	55.11
gtc	V	0	44.83
gta	V	220	47.68
gtg	V	0	72.39
---	--	------	------
mPD		0.013	0.67
		nPD:	0.02
		N. weight:	1.
		Sc. PD:	-0.044
		Sc. rank:	-3551.2

,

PB1

Pos.	182	obs:	exp:
tct	S	0	0.16
tcc	S	0	0.15
tca	S	1	0.27
tcg	S	0	0.04
att	I	0	14.04
atc	I	0	10.51
ata	I	40	15.45
act	T	3	12199.00
acc	T	10	6945.00
aca	T	39251	17000.00
acg	T	17	3137.00
agt	S	0	0.17
agc	S	0	0.21
---	--	------	------
mPD		0.0036	0.68
		nPD:	0.01
		N. weight:	0.91
		Sc. PD:	-0.051
		Sc. rank:	-19949.0

,

PB1

Pos.	183	obs:	exp:
cgt	R	0	0.02
cgc	R	0	0.04
cga	R	0	0.09
cgg	R	0	0.09
act	T	13	12210.00
acc	T	53	6951.00
aca	T	39228	17016.00
acg	T	24	3140.00
aga	R	1	0.49
agg	R	0	0.28
gct	A	0	0.82
gcc	A	0	0.75
gca	A	3	1.21
gcg	A	0	0.22
---	--	------	------
mPD		0.0048	0.68
		nPD:	0.01
		N. weight:	0.9
		Sc. PD:	-0.049
		Sc. rank:	-16936.0

,

PB1

Pos.	184	obs:	exp:
tat	Y	0	2.11
tac	Y	4	1.89
cat	H	196	22177.00
cac	H	39122	17141.00
---	--	------	------
mPD		0.010	0.49
		nPD:	0.02
		N. weight:	0.87
		Sc. PD:	-0.036
		Sc. rank:	-1648.5

,

PB1

Pos.	185	obs:	exp:
ttt	F	39045	19127.00
ttc	F	277	20195.00
---	--	------	------
mPD		0.014	0.50
		nPD:	0.03
		N. weight:	0.74
		Sc. PD:	-0.025
		Sc. rank:	3683.3

,

PB1

Pos.	186	obs:	exp:
caa	Q	39304	24037.00
cag	Q	18	15285.00
---	--	------	------
mPD		0.00092	0.48
		nPD:	0.
		N. weight:	0.54
		Sc. PD:	-0.032
		Sc. rank:	-15369.0

,

PB1

Pos.	187	obs:	exp:
cgt	R	0	779.60
cgc	R	0	1415.00
cga	R	8	3588.00
cgg	R	0	3376.00
att	I	0	0.35
atc	I	0	0.26
ata	I	1	0.39
act	T	0	0.31
acc	T	0	0.18
aca	T	1	0.43
acg	T	0	0.08
aga	R	39141	19286.00
agg	R	171	10876.00
---	--	------	------
mPD		0.0092	0.89
		nPD:	0.01
		N. weight:	0.75
		Sc. PD:	-0.038
		Sc. rank:	-9670.6

,

PB1

Pos.	188	obs:	exp:
cgt	R	0	0.20
cgc	R	0	0.36
cga	R	0	0.91
cgg	R	0	0.86
aaa	K	39118	25845.00
aag	K	194	13467.00
aga	R	10	4.90
agg	R	0	2.77
---	--	------	------
mPD		0.010	0.45
		nPD:	0.02
		N. weight:	0.43
		Sc. PD:	-0.017
		Sc. rank:	165.9

,

PB1

Pos.	189	obs:	exp:
cgt	R	0	779.50
cgc	R	0	1415.00
cga	R	0	3587.00
cgg	R	4	3376.00
aaa	K	0	3.94
aag	K	6	2.06
aga	R	2821	19284.00
agg	R	36491	10875.00
---	--	------	------
mPD		0.13	0.89
		nPD:	0.15
		N. weight:	1.1
		Sc. PD:	0.093
		Sc. rank:	26858.0

,

PB1

Pos.	190	obs:	exp:
tct	S	0	3.39
tcc	S	0	3.14
tca	S	0	5.64
tcg	S	0	0.83
cgt	R	0	779.10
cgc	R	0	1414.00
cga	R	3	3585.00
cgg	R	3	3374.00
att	I	0	0.35
atc	I	0	0.26
ata	I	1	0.39
aaa	K	6	3.94
aag	K	0	2.06
agt	S	21	3.67
agc	S	0	4.33
aga	R	39156	19273.00
agg	R	132	10869.00
---	--	------	------
mPD		0.0086	0.89
		nPD:	0.01
		N. weight:	0.76
		Sc. PD:	-0.039
		Sc. rank:	-10482.0

,

PB1

Pos.	191	obs:	exp:
tta	L	6	0.70
ttg	L	0	1.24
ctt	L	0	1.26
ctc	L	0	0.88
cta	L	0	0.92
ctg	L	0	1.01
att	I	0	43.88
atc	I	0	32.85
ata	I	125	48.27
gtt	V	13	9816.00
gtc	V	1	7985.00
gta	V	38775	8494.00
gtg	V	401	12894.00
gct	A	0	0.27
gcc	A	0	0.25
gca	A	1	0.40
gcg	A	0	0.07
---	--	------	------
mPD		0.028	0.75
		nPD:	0.04
		N. weight:	1.6
		Sc. PD:	-0.04
		Sc. rank:	14461.0

,

PB1

Pos.	192	obs:	exp:
cgt	R	0	779.20
cgc	R	0	1414.00
cga	R	36	3586.00
cgg	R	15	3374.00
aaa	K	0	15.78
aag	K	24	8.22
aga	R	1194	19275.00
agg	R	38053	10870.00
---	--	------	------
mPD		0.064	0.89
		nPD:	0.07
		N. weight:	1.2
		Sc. PD:	0.012
		Sc. rank:	23532.0

,

PB1

Pos.	193	obs:	exp:
aat	N	0	1.12
aac	N	2	0.88
gat	D	243	22226.00
gac	D	39077	17094.00
---	--	------	------
mPD		0.012	0.49
		nPD:	0.03
		N. weight:	0.87
		Sc. PD:	-0.032
		Sc. rank:	2112.4

,

PB1

Pos.	194	obs:	exp:
tct	S	0	3.23
tcc	S	0	2.99
tca	S	0	5.38
tcg	S	0	0.79
att	I	0	0.35
atc	I	1	0.26
ata	I	0	0.39
aat	N	68	21988.00
aac	N	39233	17313.00
agt	S	0	3.50
agc	S	20	4.13
---	--	------	------
mPD		0.0045	0.49
		nPD:	0.01
		N. weight:	0.89
		Sc. PD:	-0.046
		Sc. rank:	-13118.0

,

PB1			
Pos.	195	obs:	exp:
tta	L	0	48.04
ttg	L	8	85.59
ctt	L	0	86.60
ctc	L	0	60.71
cta	L	0	63.57
ctg	L	406	69.48
cgt	R	0	0.02
cgc	R	0	0.04
cga	R	0	0.09
cgg	R	0	0.09
att	I	5	7.37
atc	I	0	5.52
ata	I	16	8.11
atg	M	38824	38824.00
act	T	0	8.38
acc	T	0	4.77
aca	T	0	11.68
acg	T	27	2.16
aga	R	0	0.49
agg	R	1	0.28
gtt	V	0	8.77
gtc	V	0	7.13
gta	V	0	7.59
gtg	V	35	11.52
---	--	------	------
mPD		0.025	0.041
		nPD:	0.62
		N. weight:	0.023
		Sc. PD:	0.012
		Sc. rank:	691.1

,

PB1			
Pos.	196	obs:	exp:
tct	S	0	0.16
tcc	S	1	0.15
tca	S	0	0.27
tcg	S	0	0.04
att	I	0	0.35
atc	I	1	0.26
ata	I	0	0.39
act	T	157	12210.00
acc	T	39101	6951.00
aca	T	58	17015.00
acg	T	0	3140.00
aat	N	0	0.56
aac	N	1	0.44
agt	S	0	0.17
agc	S	0	0.21
gct	A	0	0.82
gcc	A	3	0.75
gca	A	0	1.21
gcg	A	0	0.22
---	--	------	------
mPD		0.011	0.68
		nPD:	0.02
		N. weight:	1.9
		Sc. PD:	-0.083
		Sc. rank:	-9877.5

,

PB1			
Pos.	197	obs:	exp:
cgt	R	0	0.85
cgc	R	0	1.55
cga	R	0	3.92
cgg	R	0	3.69
atg	M	2	2.00
aat	N	10	5.59
aac	N	0	4.41
aaa	K	671	25816.00
aag	K	38596	13451.00
aga	R	1	21.09
agg	R	42	11.89
---	--	------	------
mPD		0.036	0.45
		nPD:	0.08
		N. weight:	1.1
		Sc. PD:	0.018
		Sc. rank:	21474.0

,

PB1			
Pos.	198	obs:	exp:
ttt	F	0	0.49
ttc	F	1	0.51
cgt	R	0	0.20
cgc	R	0	0.36
cga	R	0	0.91
cgg	R	0	0.86
aat	N	0	1.12
aac	N	2	0.88
aaa	K	39034	25842.00
aag	K	274	13466.00
aga	R	10	4.90
agg	R	0	2.77
gaa	E	1	0.59
gag	E	0	0.41
---	--	------	------
mPD		0.015	0.45
		nPD:	0.03
		N. weight:	0.42
		Sc. PD:	-0.012
		Sc. rank:	2809.3

,

PB1			
Pos.	199	obs:	exp:
tta	L	0	0.23
ttg	L	2	0.41
ctt	L	0	0.42
ctc	L	0	0.29
cta	L	0	0.31
ctg	L	0	0.34
att	I	0	0.35
atc	I	0	0.26
ata	I	1	0.39
atg	M	39318	39318.00
act	T	0	0.31
acc	T	0	0.18
aca	T	0	0.43
acg	T	1	0.08
---	--	------	------
mPD		0.00020	0.00031
		nPD:	0.65
		N. weight:	0.00018
		Sc. PD:	0.00011
		Sc. rank:	5.6

,

PB1			
Pos.	200	obs:	exp:
ttt	F	0	0.49
ttc	F	1	0.51
tta	L	0	0.12
ttg	L	0	0.21
ctt	L	0	0.21
ctc	L	1	0.15
cta	L	0	0.15
ctg	L	0	0.17
att	I	1	215.50
atc	I	592	161.30
ata	I	21	237.10
gtt	V	130	9695.00
gtc	V	38538	7887.00
gta	V	38	8389.00
gtg	V	0	12735.00
---	--	------	------
mPD		0.040	0.77
		nPD:	0.05
		N. weight:	1.7
		Sc. PD:	-0.017
		Sc. rank:	25024.0

,

PB1			
Pos.	201	obs:	exp:
att	I	0	6.67
atc	I	0	4.99
ata	I	19	7.34
act	T	48	12206.00
acc	T	7	6949.00
aca	T	39117	17009.00
acg	T	131	3139.00
---	--	------	------
mPD		0.010	0.68
		nPD:	0.02
		N. weight:	0.89
		Sc. PD:	-0.041
		Sc. rank:	-5975.7

,

PB1			
Pos.	202	obs:	exp:
tta	L	0	0.23
ttg	L	0	0.41
ctt	L	0	0.42
ctc	L	0	0.29
cta	L	2	0.31
ctg	L	0	0.34
cat	H	0	1.13
cac	H	2	0.87
caa	Q	39142	24033.00
cag	Q	174	15283.00
cgt	R	0	0.02
cgc	R	0	0.04
cga	R	1	0.09
cgg	R	0	0.09
aaa	K	1	0.66
aag	K	0	0.34
aga	R	0	0.49
agg	R	0	0.28
---	--	------	------
mPD		0.0091	0.48
		nPD:	0.02
		N. weight:	0.51
		Sc. PD:	-0.022
		Sc. rank:	-1528.0

,

PB1			
Pos.	203	obs:	exp:
cgt	R	0	779.70
cgc	R	0	1415.00
cga	R	6	3588.00
cgg	R	0	3376.00
aga	R	39159	19287.00
agg	R	157	10876.00
---	--	------	------
mPD		0.0083	0.89
		nPD:	0.01
		N. weight:	0.75
		Sc. PD:	-0.039
		Sc. rank:	-10823.0

,

PB1			
Pos.	204	obs:	exp:
att	I	0	0.70
atc	I	0	0.53
ata	I	2	0.77
act	T	5	12211.00
acc	T	7	6952.00
aca	T	39193	17017.00
acg	T	115	3140.00
---	--	------	------
mPD		0.0065	0.68
		nPD:	0.01
		N. weight:	0.9
		Sc. PD:	-0.046
		Sc. rank:	-12490.0

,

PB1			
Pos.	205	obs:	exp:
att	I	13	13799.00
atc	I	0	10329.00
ata	I	39296	15181.00
atg	M	9	9.00
gtt	V	0	1.00
gtc	V	0	0.82
gta	V	4	0.87
gtg	V	0	1.32
---	--	------	------
mPD		0.0013	0.66
		nPD:	0.
		N. weight:	1.
		Sc. PD:	-0.061
		Sc. rank:	-29750.0

,

PB1			
Pos.	206	obs:	exp:
ggt	G	9	5294.00
ggc	G	0	5488.00
gga	G	736	18152.00
ggg	G	38577	10388.00
---	--	------	------
mPD		0.037	0.68
		nPD:	0.05
		N. weight:	1.3
		Sc. PD:	-0.01
		Sc. rank:	20472.0

,

PB1			
Pos.	207	obs:	exp:
cgt	R	0	0.28
cgc	R	0	0.50
cga	R	0	1.28
cgg	R	0	1.20
aaa	K	3	25842.00
aag	K	39305	13466.00
aga	R	0	6.87
agg	R	14	3.87
---	--	------	------
mPD		0.00086	0.45
		nPD:	0.
		N. weight:	1.2
		Sc. PD:	-0.069
		Sc. rank:	-33734.0

,

PB1			
Pos.	208	obs:	exp:
cgt	R	0	0.08
cgc	R	0	0.14
cga	R	0	0.36
cgg	R	0	0.34
act	T	0	0.31
acc	T	0	0.18
aca	T	1	0.43
acg	T	0	0.08
aaa	K	39211	25848.00
aag	K	106	13469.00
aga	R	4	1.96
agg	R	0	1.11
---	--	------	------
mPD		0.0056	0.45
		nPD:	0.01
		N. weight:	0.44
		Sc. PD:	-0.022
		Sc. rank:	-4621.5

,

PB1			
Pos.	209	obs:	exp:
cgt	R	0	0.30
cgc	R	0	0.54
cga	R	0	1.37
cgg	R	0	1.29
aaa	K	35787	25842.00
aag	K	3520	13465.00
aga	R	14	7.36
agg	R	1	4.15
---	--	------	------
mPD		0.16	0.45
		nPD:	0.36
		N. weight:	0.19
		Sc. PD:	0.057
		Sc. rank:	5555.2

,

PB1			
Pos.	210	obs:	exp:
cat	H	0	0.56
cac	H	1	0.44
caa	Q	39074	24034.00
cag	Q	244	15284.00
cgt	R	0	0.06
cgc	R	0	0.11
cga	R	3	0.27
cgg	R	0	0.26
aga	R	0	1.47
agg	R	0	0.83
---	--	------	------
mPD		0.013	0.48
		nPD:	0.03
		N. weight:	0.5
		Sc. PD:	-0.018
		Sc. rank:	1997.7

,

PB1			
Pos.	211	obs:	exp:
cgt	R	0	775.70
cgc	R	0	1408.00
cga	R	8	3570.00
cgg	R	6	3359.00
att	I	0	0.35
atc	I	0	0.26
ata	I	1	0.39
aaa	K	143	127.50
aag	K	51	66.46
aga	R	26631	19190.00
agg	R	12479	10822.00
ggt	G	0	0.40
ggc	G	0	0.42
gga	G	2	1.38
ggg	G	1	0.79
---	--	------	------
mPD		0.45	0.89
		nPD:	0.5
		N. weight:	0.29
		Sc. PD:	0.12
		Sc. rank:	8660.4

,

PB1			
Pos.	212	obs:	exp:
tta	L	0	7.54
ttg	L	33	13.44
ctt	L	0	13.60
ctc	L	0	9.53
cta	L	0	9.98
ctg	L	32	10.91
att	I	2	1.05
atc	I	0	0.79
ata	I	1	1.16
atg	M	3245	3245.00
gtt	V	383	9019.00
gtc	V	0	7337.00
gta	V	115	7805.00
gtg	V	35511	11848.00
---	--	------	------
mPD		0.18	0.88
		nPD:	0.2
		N. weight:	1.
		Sc. PD:	0.14
		Sc. rank:	27379.0

,

PB1			
Pos.	213	obs:	exp:
tct	S	0	7.10
tcc	S	0	6.58
tca	S	0	11.83
tcg	S	0	1.73
tat	Y	2	1.05
tac	Y	0	0.95
cat	H	3	1.69
cac	H	0	1.31
att	I	1	0.35
atc	I	0	0.26
ata	I	0	0.39
act	T	7	2.17
acc	T	0	1.24
aca	T	0	3.03
acg	T	0	0.56
aat	N	38931	21947.00
aac	N	298	17282.00
aaa	K	7	5.92
aag	K	2	3.08
agt	S	43	7.69
agc	S	1	9.08
gat	D	26	14.70
gac	D	0	11.30
ggt	G	1	0.13
ggc	G	0	0.14
gga	G	0	0.46
ggg	G	0	0.26
---	--	------	------
mPD		0.020	0.50
		nPD:	0.04
		N. weight:	0.59
		Sc. PD:	-0.013
		Sc. rank:	5664.3

,

PB1			
Pos.	214	obs:	exp:
cgt	R	0	1.13
cgc	R	0	2.05
cga	R	0	5.20
cgg	R	0	4.89
aat	N	4	2.24
aac	N	0	1.76
aaa	K	126	25812.00
aag	K	39135	13449.00
aga	R	0	27.96
agg	R	57	15.77
---	--	------	------
mPD		0.0095	0.45
		nPD:	0.02
		N. weight:	1.1
		Sc. PD:	-0.047
		Sc. rank:	-1808.6

,

PB1			
Pos.	215	obs:	exp:
cgt	R	0	763.60
cgc	R	0	1386.00
cga	R	4	3514.00
cgg	R	0	3307.00
att	I	0	0.70
atc	I	0	0.53
ata	I	2	0.77
aaa	K	796	529.90
aag	K	10	276.10
aga	R	38232	18889.00
agg	R	275	10652.00
ggt	G	0	0.40
ggc	G	0	0.42
gga	G	3	1.38
ggg	G	0	0.79
---	--	------	------
mPD		0.055	0.92
		nPD:	0.06
		N. weight:	0.73
		Sc. PD:	-0.0019
		Sc. rank:	12094.0

,

PB1			
Pos.	216	obs:	exp:
tct	S	0	112.60
tcc	S	0	104.30
tca	S	0	187.60
tcg	S	0	27.48
tgt	C	0	17.98
tgc	C	46	28.02
aat	N	1	2.80
aac	N	4	2.20
agt	S	190	122.00
agc	S	508	144.00
gat	D	2	17.52
gac	D	29	13.48
ggt	G	87	5189.00
ggc	G	38389	5379.00
gga	G	65	17792.00
ggg	G	1	10182.00
---	--	------	------
mPD		0.057	0.74
		nPD:	0.08
		N. weight:	2.1
		Sc. PD:	0.028
		Sc. rank:	41465.0

,

PB1			
Pos.	217	obs:	exp:
tat	Y	959	20727.00
tac	Y	38363	18595.00
---	--	------	------
mPD		0.048	0.50
		nPD:	0.1
		N. weight:	0.69
		Sc. PD:	0.022
		Sc. rank:	14872.0

,

PB1			
Pos.	218	obs:	exp:
tta	L	269	4560.00
ttg	L	2	8125.00
ctt	L	0	8221.00
ctc	L	0	5763.00
cta	L	38995	6035.00
ctg	L	34	6596.00
att	I	0	7.72
atc	I	0	5.78
ata	I	22	8.50
---	--	------	------
mPD		0.017	1.2
		nPD:	0.01
		N. weight:	2.
		Sc. PD:	-0.094
		Sc. rank:	-15597.0

,

PB1			
Pos.	219	obs:	exp:
tta	L	0	0.58
ttg	L	0	1.03
ctt	L	0	1.05
ctc	L	0	0.73
cta	L	5	0.77
ctg	L	0	0.84
cgt	R	0	0.06
cgc	R	0	0.11
cga	R	0	0.27
cgg	R	0	0.26
att	I	4	13789.00
atc	I	1	10321.00
ata	I	39275	15170.00
act	T	0	5.28
acc	T	0	3.01
aca	T	17	7.36
acg	T	0	1.36
aga	R	3	1.47
agg	R	0	0.83
gtt	V	0	4.26
gtc	V	0	3.46
gta	V	17	3.68
gtg	V	0	5.59
---	--	------	------
mPD		0.0024	0.66
		nPD:	0.
		N. weight:	1.
		Sc. PD:	-0.06
		Sc. rank:	-26400.0

,

PB1			
Pos.	220	obs:	exp:
cgt	R	0	779.00
cgc	R	0	1414.00
cga	R	4	3585.00
cgg	R	0	3374.00
aaa	K	32	21.04
aag	K	0	10.96
aga	R	39173	19271.00
agg	R	113	10868.00
---	--	------	------
mPD		0.0076	0.89
		nPD:	0.01
		N. weight:	0.76
		Sc. PD:	-0.04
		Sc. rank:	-12044.0

,

PB1			
Pos.	221	obs:	exp:
tct	S	5	0.97
tcc	S	0	0.90
tca	S	1	1.61
tcg	S	0	0.24
act	T	7	3.42
acc	T	3	1.94
aca	T	1	4.76
acg	T	0	0.88
agt	S	0	1.05
agc	S	0	1.24
gtt	V	16	4.01
gtc	V	0	3.26
gta	V	0	3.47
gtg	V	0	5.26
gct	A	38725	10708.00
gcc	A	69	9816.00
gca	A	477	15892.00
gcg	A	17	2872.00
ggt	G	1	0.13
ggc	G	0	0.14
gga	G	0	0.46
ggg	G	0	0.26
---	--	------	------
mPD		0.030	0.70
		nPD:	0.04
		N. weight:	1.3
		Sc. PD:	-0.025
		Sc. rank:	14711.0

,

PB1			
Pos.	222	obs:	exp:
tta	L	125	4563.00
ttg	L	22429	8130.00
ctt	L	13	8226.00
ctc	L	1	5766.00
cta	L	239	6038.00
ctg	L	16515	6599.00
---	--	------	------
mPD		0.51	1.2
		nPD:	0.44
		N. weight:	1.
		Sc. PD:	0.37
		Sc. rank:	30107.0

,

PB1			
Pos.	223	obs:	exp:
act	T	10	12211.00
acc	T	8	6952.00
aca	T	39251	17016.00
acg	T	50	3140.00
gct	A	0	0.82
gcc	A	0	0.75
gca	A	3	1.21
gcg	A	0	0.22
---	--	------	------
mPD		0.0036	0.68
		nPD:	0.01
		N. weight:	0.91
		Sc. PD:	-0.051
		Sc. rank:	-19744.0

PB1			
Pos.	224	obs:	exp:
tta	L	262	4563.00
ttg	L	38964	8130.00
ctt	L	1	8226.00
ctc	L	0	5766.00
cta	L	2	6038.00
ctg	L	93	6599.00
---	--	------	------
mPD		0.018	1.2
		nPD:	0.02
		N. weight:	1.7
		Sc. PD:	-0.076
		Sc. rank:	-10502.0

PB1			
Pos.	225	obs:	exp:
aat	N	279	21999.00
aac	N	39042	17322.00
aaa	K	1	0.66
aag	K	0	0.34
---	--	------	------
mPD		0.014	0.49
		nPD:	0.03
		N. weight:	0.85
		Sc. PD:	-0.028
		Sc. rank:	4424.0

PB1			
Pos.	226	obs:	exp:
act	T	151	12212.00
acc	T	0	6952.00
aca	T	803	17018.00
acg	T	38368	3141.00
---	--	------	------
mPD		0.048	0.68
		nPD:	0.07
		N. weight:	2.6
		Sc. PD:	0.019
		Sc. rank:	46592.0

PB1			
Pos.	227	obs:	exp:
att	I	0	0.35
atc	I	0	0.26
ata	I	1	0.39
atg	M	39321	39321.00
---	--	------	------
mPD		0.000051	0.000051
		nPD:	1.
		N. weight:	0.000027
		Sc. PD:	0.000024
		Sc. rank:	0.8

PB1			
Pos.	228	obs:	exp:
act	T	36	12211.00
acc	T	39230	6952.00
aca	T	53	17016.00
acg	T	0	3140.00
gct	A	0	0.82
gcc	A	3	0.75
gca	A	0	1.21
gcg	A	0	0.22
---	--	------	------
mPD		0.0047	0.68
		nPD:	0.01
		N. weight:	1.9
		Sc. PD:	-0.1
		Sc. rank:	-35834.0

PB1			
Pos.	229	obs:	exp:
aaa	K	10418	25852.00
aag	K	28904	13470.00
---	--	------	------
mPD		0.39	0.45
		nPD:	0.86
		N. weight:	0.35
		Sc. PD:	0.28
		Sc. rank:	10886.0

PB1			
Pos.	230	obs:	exp:
gat	D	39285	22227.00
gac	D	37	17095.00
---	--	------	------
mPD		0.0019	0.49
		nPD:	0.
		N. weight:	0.62
		Sc. PD:	-0.035
		Sc. rank:	-15543.0

PB1			
Pos.	231	obs:	exp:
gct	A	1219	10717.00
gcc	A	13	9824.00
gca	A	37995	15906.00
gcg	A	95	2875.00
---	--	------	------
mPD		0.065	0.69
		nPD:	0.09
		N. weight:	0.84
		Sc. PD:	0.026
		Sc. rank:	17855.0

PB1			
Pos.	232	obs:	exp:
gaa	E	5528	23183.00
gag	E	33794	16139.00
---	--	------	------
mPD		0.24	0.48
		nPD:	0.5
		N. weight:	0.48
		Sc. PD:	0.2
		Sc. rank:	14172.0

PB1			
Pos.	233	obs:	exp:
cgt	R	0	779.70
cgc	R	0	1415.00
cga	R	4	3588.00
cgg	R	0	3376.00
aga	R	39247	19287.00
agg	R	71	10876.00
---	--	------	------
mPD		0.0038	0.89
		nPD:	0.
		N. weight:	0.77
		Sc. PD:	-0.044
		Sc. rank:	-18516.0

PB1			
Pos.	234	obs:	exp:
ggt	G	709	5294.00
ggc	G	38537	5488.00
gga	G	73	18152.00
ggg	G	3	10388.00
---	--	------	------
mPD		0.039	0.68
		nPD:	0.06
		N. weight:	2.
		Sc. PD:	-0.0097
		Sc. rank:	33287.0

PB1			
Pos.	235	obs:	exp:
aaa	K	39174	25852.00
aag	K	148	13470.00
---	--	------	------
mPD		0.0075	0.45
		nPD:	0.02
		N. weight:	0.44
		Sc. PD:	-0.019
		Sc. rank:	-2220.6

PB1			
Pos.	236	obs:	exp:
tta	L	36677	4563.00
ttg	L	229	8130.00
ctt	L	2	8226.00
ctc	L	0	5766.00
cta	L	2403	6038.00
ctg	L	11	6599.00
---	--	------	------
mPD		0.13	1.2
		nPD:	0.11
		N. weight:	2.
		Sc. PD:	0.095
		Sc. rank:	46028.0

PB1			
Pos.	237	obs:	exp:
cgt	R	0	0.46
cgc	R	0	0.83
cga	R	0	2.10
cgg	R	0	1.97
aaa	K	39181	25837.00
aag	K	118	13462.00
aga	R	23	11.28
agg	R	0	6.36
---	--	------	------
mPD		0.0072	0.45
		nPD:	0.02
		N. weight:	0.44
		Sc. PD:	-0.02
		Sc. rank:	-2686.5

PB1			
Pos.	238	obs:	exp:
cgt	R	0	779.60
cgc	R	0	1415.00
cga	R	8	3588.00
cgg	R	3	3376.00
aga	R	39134	19286.00
agg	R	176	10876.00
ggt	G	0	0.13
ggc	G	0	0.14
gga	G	1	0.46
ggg	G	0	0.26
---	--	------	------
mPD		0.0097	0.89
		nPD:	0.01
		N. weight:	0.75
		Sc. PD:	-0.038
		Sc. rank:	-9038.7

PB1			
Pos.	239	obs:	exp:
tgg	W	1	1.00
cgt	R	0	779.60
cgc	R	0	1415.00
cga	R	0	3588.00
cgg	R	5	3376.00
aaa	K	1	0.66
aag	K	0	0.34
aga	R	2216	19286.00
agg	R	37099	10876.00
---	--	------	------
mPD		0.11	0.89
		nPD:	0.12
		N. weight:	1.1
		Sc. PD:	0.064
		Sc. rank:	26383.0

PB1			
Pos.	240	obs:	exp:
act	T	1	0.31
acc	T	0	0.18
aca	T	0	0.43
acg	T	0	0.08
gct	A	39183	10717.00
gcc	A	116	9824.00
gca	A	21	15906.00
gcg	A	1	2874.00
---	--	------	------
mPD		0.0070	0.69
		nPD:	0.01
		N. weight:	1.4
		Sc. PD:	-0.071
		Sc. rank:	-18369.0

PB1			
Pos.	241	obs:	exp:
att	I	38971	13804.00
atc	I	337	10332.00
ata	I	14	15186.00
---	--	------	------
mPD		0.018	0.66
		nPD:	0.03
		N. weight:	1.1
		Sc. PD:	-0.038
		Sc. rank:	4863.6

PB1			
Pos.	242	obs:	exp:
cct	P	0	0.33
ccc	P	0	0.19
cca	P	1	0.34
ccg	P	0	0.14
gct	A	3	10717.00
gcc	A	6	9824.00
gca	A	39142	15906.00
gcg	A	170	2874.00
---	--	------	------
mPD		0.0091	0.69
		nPD:	0.01
		N. weight:	0.97
		Sc. PD:	-0.047
		Sc. rank:	-9316.4

PB1

Pos.	243	obs:	exp:
cct	P	0	0.33
ccc	P	0	0.19
cca	P	1	0.34
ccg	P	0	0.14
act	T	2	12211.00
acc	T	57	6952.00
aca	T	39255	17017.00
acg	T	7	3141.00
mPD		0.0034	0.68
		nPD:	0.01
		N. weight :	0.91
		Sc. PD:	-0.051
		Sc. rank :	-20583.0

,

PB1

Pos.	244	obs:	exp:
cct	P	461	13104.00
ccc	P	38672	7372.00
cca	P	171	13285.00
ccg	P	18	5562.00
mPD		0.033	0.72
		nPD:	0.05
		N. weight :	1.7
		Sc. PD:	-0.029
		Sc. rank :	20622.0

,

PB1

Pos.	245	obs:	exp:
ggt	G	10	5294.00
ggc	G	0	5488.00
gga	G	348	18152.00
ggg	G	38964	10388.00
mPD		0.018	0.68
		nPD:	0.03
		N. weight :	1.4
		Sc. PD:	-0.049
		Sc. rank :	5976.3

,

PB1

Pos.	246	obs:	exp:
att	I	2	2.11
atc	I	0	1.58
ata	I	4	2.32
atg	M	39313	39313.00
gtt	V	0	0.75
gtc	V	0	0.61
gta	V	0	0.65
gtg	V	3	0.99
mPD		0.00046	0.00056
		nPD:	0.82
		N. weight :	0.00018
		Sc. PD:	0.00013
		Sc. rank :	5.6

,

PB1

Pos.	247	obs:	exp:
cat	H	0	1.13
cac	H	2	0.87
caa	Q	39249	24035.00
cag	Q	71	15285.00
mPD		0.0037	0.48
		nPD:	0.01
		N. weight :	0.53
		Sc. PD:	-0.028
		Sc. rank :	-8994.5

,

PB1

Pos.	248	obs:	exp:
att	I	38643	13803.00
atc	I	669	10332.00
ata	I	9	15186.00
atg	M	1	1.00
mPD		0.034	0.66
		nPD:	0.05
		N. weight :	1.1
		Sc. PD:	-0.011
		Sc. rank :	15139.0

,

PB1

Pos.	249	obs:	exp:
cgt	R	0	779.70
cgc	R	0	1415.00
cga	R	0	3588.00
cgg	R	0	3376.00
aga	R	39027	19287.00
agg	R	295	10876.00
mPD		0.015	0.89
		nPD:	0.02
		N. weight :	0.74
		Sc. PD:	-0.033
		Sc. rank :	-3585.2

,

PB1

Pos.	250	obs:	exp:
gtt	V	0	0.50
gtc	V	0	0.41
gta	V	0	0.43
gtg	V	2	0.66
ggt	G	129	5293.00
ggc	G	6	5488.00
gga	G	6974	18151.00
ggg	G	32211	10388.00
mPD		0.30	0.68
		nPD:	0.44
		N. weight :	0.82
		Sc. PD:	0.3
		Sc. rank :	24004.0

,

PB1

Pos.	251	obs:	exp:
ttt	F	146	19112.00
ttc	F	39144	20178.00
tta	L	1	0.35
ttg	L	0	0.62
ctt	L	0	0.63
ctc	L	2	0.44
cta	L	0	0.46
ctg	L	0	0.50
gtt	V	0	7.26
gtc	V	29	5.91
gta	V	0	6.29
gtg	V	0	9.54
mPD		0.0090	0.50
		nPD:	0.02
		N. weight :	0.71
		Sc. PD:	-0.031
		Sc. rank :	-2835.1

,

PB1

Pos.	252	obs:	exp:
atg	M	2	2.00
gtt	V	33	9849.00
gtc	V	1	8012.00
gta	V	216	8522.00
gtg	V	39070	12937.00
mPD		0.013	0.74
		nPD:	0.02
		N. weight :	1.2
		Sc. PD:	-0.052
		Sc. rank :	-5272.2

,

PB1

Pos.	253	obs:	exp:
ttt	F	0	0.97
ttc	F	2	1.03
tat	Y	7046	20721.00
tac	Y	32264	18589.00
cat	H	3	5.64
cac	H	7	4.36
mPD		0.29	0.50
		nPD:	0.59
		N. weight :	0.28
		Sc. PD:	0.15
		Sc. rank :	8591.1

,

PB1

Pos.	254	obs:	exp:
ttt	F	716	19123.00
ttc	F	38597	20190.00
tta	L	2	0.35
ttg	L	0	0.62
tct	S	0	0.81
tcc	S	5	0.75
tca	S	0	1.34
tcg	S	0	0.20
ctt	L	0	0.63
ctc	L	1	0.44
cta	L	0	0.46
ctg	L	0	0.50
agt	S	0	0.87
agc	S	0	1.03
gtt	V	0	0.25
gtc	V	1	0.20
gta	V	0	0.22
gtg	V	0	0.33
mPD		0.036	0.50
		nPD:	0.07
		N. weight :	0.63
		Sc. PD:	0.0061
		Sc. rank :	12025.0

,

PB1

Pos.	255	obs:	exp:
att	I	1	0.35
atc	I	0	0.26
ata	I	0	0.39
gtt	V	38805	9848.00
gtc	V	490	8011.00
gta	V	4	8522.00
gtg	V	19	12937.00
gct	A	3	0.82
gcc	A	0	0.75
gca	A	0	1.21
gcg	A	0	0.22
mPD		0.026	0.74
		nPD:	0.04
		N. weight :	1.4
		Sc. PD:	-0.039
		Sc. rank :	11740.0

,

PB1

Pos.	256	obs:	exp:
gaa	E	39036	23183.00
gag	E	286	16139.00
mPD		0.014	0.48
		nPD:	0.03
		N. weight :	0.54
		Sc. PD:	-0.017
		Sc. rank :	3021.3

,

PB1

Pos.	257	obs:	exp:
tct	S	0	0.16
tcc	S	0	0.15
tca	S	0	0.27
tcg	S	0	0.04
att	I	145	50.90
atc	I	0	38.10
ata	I	0	56.00
act	T	38972	12152.00
acc	T	82	6918.00
aca	T	52	16935.00
acg	T	24	3125.00
aat	N	8	5.04
aac	N	1	3.96
agt	S	1	0.17
agc	S	0	0.21
gct	A	37	10.08
gcc	A	0	9.24
gca	A	0	14.97
gcg	A	0	2.70
mPD		0.018	0.69
		nPD:	0.03
		N. weight :	1.3
		Sc. PD:	-0.045
		Sc. rank :	4252.6

,

PB1

Pos.	258	obs:	exp:
tta	L	38891	4563.00
ttg	L	78	8130.00
ctt	L	0	8226.00
ctc	L	0	5766.00
cta	L	349	6038.00
ctg	L	4	6599.00
mPD		0.022	1.2
		nPD:	0.02
		N. weight :	2.3
		Sc. PD:	-0.097
		Sc. rank :	-7350.0

,

PB1			
Pos.	259	obs:	exp:
tct	S	1	0.16
tcc	S	0	0.15
tca	S	0	0.27
tcg	S	0	0.04
agt	S	0	0.17
agc	S	0	0.21
gct	A	39138	10717.00
gcc	A	171	9824.00
gca	A	8	15906.00
gcg	A	4	2874.00
---	--	------	------
mPD		0.0093	0.69
		nPD:	0.01
		N. weight:	1.4
		Sc. PD:	-0.067
		Sc. rank:	-12253.0

,

PB1			
Pos.	260	obs:	exp:
cgt	R	0	776.50
cgc	R	0	1409.00
cga	R	4	3573.00
cgg	R	0	3363.00
aaa	K	159	105.80
aag	K	2	55.15
aga	R	38832	19208.00
agg	R	325	10832.00
---	--	------	------
mPD		0.025	0.89
		nPD:	0.03
		N. weight:	0.73
		Sc. PD:	-0.025
		Sc. rank:	3450.0

,

PB1			
Pos.	261	obs:	exp:
tct	S	0	6317.00
tcc	S	0	5850.00
tca	S	0	10521.00
tcg	S	0	1541.00
tgt	C	0	1.17
tgc	C	3	1.83
cgt	R	0	0.04
cgc	R	1	0.07
cga	R	0	0.18
cgg	R	0	0.17
att	I	0	1.05
atc	I	3	0.79
ata	I	0	1.16
aat	N	0	94.55
aac	N	169	74.45
agt	S	65	6841.00
agc	S	39080	8075.00
aga	R	1	0.98
agg	R	0	0.55
---	--	------	------
mPD		0.012	1.6
		nPD:	0.01
		N. weight:	1.7
		Sc. PD:	-0.092
		Sc. rank:	-31007.0

,

PB1			
Pos.	262	obs:	exp:
att	I	39167	13804.00
atc	I	144	10332.00
ata	I	11	15186.00
---	--	------	------
mPD		0.0079	0.66
		nPD:	0.01
		N. weight:	1.1
		Sc. PD:	-0.055
		Sc. rank:	-12432.0

,

PB1			
Pos.	263	obs:	exp:
tct	S	0	0.16
tcc	S	0	0.15
tca	S	0	0.27
tcg	S	0	0.04
tgt	C	844	15366.00
tgc	C	38477	23955.00
agt	S	0	0.17
agc	S	1	0.21
---	--	------	------
mPD		0.042	0.48
		nPD:	0.09
		N. weight:	0.44
		Sc. PD:	0.011
		Sc. rank:	9231.3

,

PB1			
Pos.	264	obs:	exp:
aaa	K	1	0.66
aag	K	0	0.34
gct	A	0	0.27
gcc	A	0	0.25
gca	A	1	0.40
gcg	A	0	0.07
gaa	E	38814	23176.00
gag	E	497	16135.00
ggt	G	0	1.21
ggc	G	0	1.26
gga	G	9	4.15
ggg	G	0	2.38
---	--	------	------
mPD		0.026	0.48
		nPD:	0.05
		N. weight:	0.51
		Sc. PD:	-0.0049
		Sc. rank:	7515.1

,

PB1			
Pos.	265	obs:	exp:
caa	Q	0	0.61
cag	Q	1	0.39
cgt	R	0	0.04
cgc	R	0	0.07
cga	R	0	0.18
cgg	R	0	0.17
aat	N	1	1.12
aac	N	1	0.88
aaa	K	7017	25848.00
aag	K	32300	13469.00
aga	R	0	0.98
agg	R	2	0.55
---	--	------	------
mPD		0.29	0.45
		nPD:	0.65
		N. weight:	0.53
		Sc. PD:	0.31
		Sc. rank:	16168.0

,

PB1			
Pos.	266	obs:	exp:
tta	L	0	4563.00
ttg	L	0	8129.00
ctt	L	39260	8225.00
ctc	L	46	5766.00
cta	L	9	6038.00
ctg	L	4	6599.00
att	I	3	1.05
atc	I	0	0.79
ata	I	0	1.16
---	--	------	------
mPD		0.0031	1.2
		nPD:	0.
		N. weight:	1.7
		Sc. PD:	-0.099
		Sc. rank:	-45772.0

,

PB1			
Pos.	267	obs:	exp:
gaa	E	39262	23183.00
gag	E	60	16139.00
---	--	------	------
mPD		0.0030	0.48
		nPD:	0.01
		N. weight:	0.57
		Sc. PD:	-0.031
		Sc. rank:	-11495.0

,

PB1			
Pos.	268	obs:	exp:
caa	Q	36237	24037.00
cag	Q	3085	15285.00
---	--	------	------
mPD		0.14	0.48
		nPD:	0.3
		N. weight:	0.28
		Sc. PD:	0.065
		Sc. rank:	7697.8

,

PB1			
Pos.	269	obs:	exp:
tct	S	38895	6345.00
tcc	S	325	5876.00
tca	S	17	10569.00
tcg	S	84	1548.00
agt	S	1	6872.00
agc	S	0	8112.00
---	--	------	------
mPD		0.022	1.6
		nPD:	0.01
		N. weight:	1.9
		Sc. PD:	-0.093
		Sc. rank:	-18185.0

,

PB1			
Pos.	270	obs:	exp:
caa	Q	1	0.61
cag	Q	0	0.39
ggt	G	3	5294.00
ggc	G	0	5488.00
gga	G	38952	18151.00
ggg	G	366	10388.00
---	--	------	------
mPD		0.019	0.68
		nPD:	0.03
		N. weight:	0.8
		Sc. PD:	-0.027
		Sc. rank:	3626.1

,

PB1			
Pos.	271	obs:	exp:
tta	L	0	4562.00
ttg	L	4	8128.00
ctt	L	38942	8225.00
ctc	L	361	5765.00
cta	L	9	6037.00
ctg	L	0	6598.00
att	I	6	2.11
atc	I	0	1.58
ata	I	0	2.32
---	--	------	------
mPD		0.019	1.2
		nPD:	0.02
		N. weight:	1.7
		Sc. PD:	-0.074
		Sc. rank:	-8232.6

,

PB1			
Pos.	272	obs:	exp:
cct	P	85	13104.00
ccc	P	4	7372.00
cca	P	1511	13285.00
ccg	P	37722	5562.00
---	--	------	------
mPD		0.078	0.72
		nPD:	0.11
		N. weight:	1.9
		Sc. PD:	0.086
		Sc. rank:	42533.0

,

PB1			
Pos.	273	obs:	exp:
att	I	38	13.34
atc	I	0	9.98
ata	I	0	14.68
gtt	V	39005	9840.00
gtc	V	33	8004.00
gta	V	230	8514.00
gtg	V	16	12925.00
---	--	------	------
mPD		0.016	0.74
		nPD:	0.02
		N. weight:	1.5
		Sc. PD:	-0.059
		Sc. rank:	-1033.2

,

PB1			
Pos.	274	obs:	exp:
ggt	G	16	5294.00
ggc	G	0	5488.00
gga	G	267	18152.00
ggg	G	39039	10388.00
---	--	------	------
mPD		0.014	0.68
		nPD:	0.02
		N. weight:	1.4
		Sc. PD:	-0.057
		Sc. rank:	-2053.6

,

PB1			
Pos.	275	obs:	exp:
ggt	G	38946	5294.00
ggc	G	361	5488.00
gga	G	8	18152.00
ggg	G	7	10388.00
---	--	------	------
mPD		0.019	0.68
		nPD:	0.03
		N. weight:	2.1
		Sc. PD:	-0.072
		Sc. rank:	10451.0

,

PB1			
Pos.	276	obs:	exp:
aat	N	39232	21999.00
aac	N	90	17323.00
---	--	------	------
mPD		0.0046	0.49
		nPD:	0.01
		N. weight:	0.62
		Sc. PD:	-0.032
		Sc. rank:	-8987.1

,

PB1			
Pos.	277	obs:	exp:
gaa	E	38906	23183.00
gag	E	416	16139.00
---	--	------	------
mPD		0.021	0.48
		nPD:	0.04
		N. weight:	0.52
		Sc. PD:	-0.0098
		Sc. rank:	5684.6

,

PB1			
Pos.	278	obs:	exp:
aat	N	1	0.56
aac	N	0	0.44
aaa	K	297	25850.00
aag	K	39023	13470.00
gaa	E	0	0.59
gag	E	1	0.41
---	--	------	------
mPD		0.015	0.45
		nPD:	0.03
		N. weight:	1.1
		Sc. PD:	-0.032
		Sc. rank:	8197.6

,

PB1

Pos.	279	obs:	exp:
aaa	K	451	25852.00
aag	K	38871	13470.00
---	--	------	------
mPD		0.023	0.45
		nPD:	0.05
		N. weight:	1.1
		Sc. PD:	-0.013
		Sc. rank:	15071.0

,

PB1

Pos.	280	obs:	exp:
cct	P	0	0.33
ccc	P	1	0.19
cca	P	0	0.34
ccg	P	0	0.14
act	T	0	0.31
acc	T	1	0.18
aca	T	0	0.43
acg	T	0	0.08
gct	A	29	10717.00
gcc	A	39261	9824.00
gca	A	30	15905.00
gcg	A	0	2874.00
---	--	------	------
mPD		0.0031	0.69
		nPD:	0.
		N. weight:	1.5
		Sc. PD:	-0.085
		Sc. rank:	-35680.0

,

PB1

Pos.	281	obs:	exp:
aaa	K	39250	25852.00
aag	K	72	13470.00
---	--	------	------
mPD		0.0037	0.45
		nPD:	0.01
		N. weight:	0.45
		Sc. PD:	-0.024
		Sc. rank:	-7372.7

,

PB1

Pos.	282	obs:	exp:
tta	L	11	4563.00
ttg	L	496	8130.00
ctt	L	14	8226.00
ctc	L	0	5766.00
cta	L	54	6038.00
ctg	L	38747	6599.00
---	--	------	------
mPD		0.029	1.2
		nPD:	0.03
		N. weight:	1.9
		Sc. PD:	-0.067
		Sc. rank:	5463.3

,

PB1

Pos.	283	obs:	exp:
tct	S	0	0.16
tcc	S	0	0.15
tca	S	1	0.27
tcg	S	0	0.04
act	T	0	0.93
acc	T	0	0.53
aca	T	3	1.30
acg	T	0	0.24
agt	S	0	0.17
agc	S	0	0.21
gct	A	26	10716.00
gcc	A	6	9823.00
gca	A	39275	15904.00
gcg	A	11	2874.00
---	--	------	------
mPD		0.0024	0.69
		nPD:	0.
		N. weight:	0.98
		Sc. PD:	-0.057
		Sc. rank:	-25181.0

,

PB1

Pos.	284	obs:	exp:
aat	N	39054	21999.00
aac	N	267	17322.00
gat	D	1	0.57
gac	D	0	0.43
---	--	------	------
mPD		0.014	0.49
		nPD:	0.03
		N. weight:	0.59
		Sc. PD:	-0.02
		Sc. rank:	2672.0

,

PB1

Pos.	285	obs:	exp:
act	T	1	0.31
acc	T	0	0.18
aca	T	0	0.43
acg	T	0	0.08
gtt	V	39289	9849.00
gtc	V	18	8012.00
gta	V	5	8523.00
gtg	V	9	12938.00
---	--	------	------
mPD		0.0017	0.74
		nPD:	0.
		N. weight:	1.5
		Sc. PD:	-0.089
		Sc. rank:	-41836.0

,

PB1

Pos.	286	obs:	exp:
gtt	V	47	9849.00
gtc	V	6	8012.00
gta	V	1016	8523.00
gtg	V	38252	12938.00
ggt	G	0	0.13
ggc	G	0	0.14
gga	G	0	0.46
ggg	G	1	0.26
---	--	------	------
mPD		0.053	0.74
		nPD:	0.07
		N. weight:	1.1
		Sc. PD:	0.0096
		Sc. rank:	20283.0

,

PB1

Pos.	287	obs:	exp:
cgt	R	0	779.70
cgc	R	0	1415.00
cga	R	0	3588.00
cgg	R	0	3376.00
aga	R	39236	19287.00
agg	R	86	10876.00
---	--	------	------
mPD		0.0044	0.89
		nPD:	0.
		N. weight:	0.77
		Sc. PD:	-0.043
		Sc. rank:	-17578.0

,

PB1

Pos.	288	obs:	exp:
aaa	K	38555	25852.00
aag	K	767	13470.00
---	--	------	------
mPD		0.038	0.45
		nPD:	0.08
		N. weight:	0.37
		Sc. PD:	0.0081
		Sc. rank:	7635.1

,

PB1

Pos.	289	obs:	exp:
atg	M	39322	39322.00
---	--	------	------
mPD		0	0
		nPD:	1.
		N. weight:	0.
		Sc. PD:	0
		Sc. rank:	0

,

PB1

Pos.	290	obs:	exp:
att	I	2	2.11
atc	I	0	1.58
ata	I	4	2.32
atg	M	39316	39316.00
---	--	------	------
mPD		0.00031	0.00031
		nPD:	1.
		N. weight:	0.000089
		Sc. PD:	0.000081
		Sc. rank:	2.8

,

PB1

Pos.	291	obs:	exp:
tct	S	0	0.16
tcc	S	0	0.15
tca	S	0	0.27
tcg	S	0	0.04
att	I	8	2.81
atc	I	0	2.10
ata	I	0	3.09
act	T	39102	12207.00
acc	T	185	6950.00
aca	T	6	17011.00
acg	T	14	3139.00
aat	N	3	1.68
aac	N	0	1.32
agt	S	1	0.17
agc	S	0	0.21
gct	A	3	0.82
gcc	A	0	0.75
gca	A	0	1.21
gcg	A	0	0.22
---	--	------	------
mPD		0.011	0.68
		nPD:	0.02
		N. weight:	1.2
		Sc. PD:	-0.056
		Sc. rank:	-6928.2

,

PB1

Pos.	292	obs:	exp:
tct	S	0	0.81
tcc	S	0	0.75
tca	S	0	1.34
tcg	S	0	0.20
tat	Y	1	0.53
tac	Y	0	0.47
cat	H	14	7.90
cac	H	0	6.10
act	T	2	0.62
acc	T	0	0.35
aca	T	0	0.87
acg	T	0	0.16
aat	N	38046	21983.00
aac	N	1247	17310.00
agt	S	5	0.87
agc	S	0	1.03
gat	D	7	3.96
gac	D	0	3.04
---	--	------	------
mPD		0.063	0.49
		nPD:	0.13
		N. weight:	0.49
		Sc. PD:	0.031
		Sc. rank:	11551.0

,

PB1

Pos.	293	obs:	exp:
tta	L	1	0.12
ttg	L	0	0.21
tct	S	10	6345.00
tcc	S	5	5876.00
tca	S	39201	10568.00
tcg	S	102	1548.00
tat	Y	0	0.53
tac	Y	1	0.47
ctt	L	0	0.21
ctc	L	0	0.15
cta	L	0	0.15
ctg	L	0	0.17
cct	P	0	0.33
ccc	P	0	0.19
cca	P	1	0.34
ccg	P	0	0.14
agt	S	0	6871.00
agc	S	0	8111.00
gct	A	0	0.27
gcc	A	0	0.25
gca	A	1	0.40
gcg	A	0	0.07
---	--	------	------
mPD		0.0062	1.6
		nPD:	0.
		N. weight:	1.4
		Sc. PD:	-0.082
		Sc. rank:	-35876.0

,

PB1

Pos.	294	obs:	exp:
cat	H	9	14.10
cac	H	16	10.90
caa	Q	39231	24015.00
cag	Q	56	15272.00
cgt	R	0	0.12
cgc	R	0	0.22
cga	R	5	0.55
cgg	R	0	0.52
aaa	K	3	1.97
aag	K	0	1.03
aga	R	1	2.94
agg	R	0	1.66
gaa	E	1	0.59
gag	E	0	0.41
---	--	------	------
mPD		0.0047	0.48
		nPD:	0.01
		N. weight:	0.53
		Sc. PD:	-0.027
		Sc. rank:	-7090.3

,

PB1			
Pos.	295	obs:	exp:
aat	N	0	0.56
aac	N	1	0.44
gat	D	210	22226.00
gac	D	39110	17094.00
gaa	E	1	0.59
gag	E	0	0.41
---	--	------	------
mPD		0.011	0.49
		nPD:	0.02
		N. weight:	0.88
		Sc. PD:	-0.035
		Sc. rank:	-415.2

,

PB1			
Pos.	296	obs:	exp:
tct	S	0	0.16
tcc	S	0	0.15
tca	S	1	0.27
tcg	S	0	0.04
att	I	0	2.46
atc	I	0	1.84
ata	I	7	2.70
act	T	195	12208.00
acc	T	5	6950.00
aca	T	39057	17012.00
acg	T	53	3140.00
agt	S	0	0.17
agc	S	0	0.21
gct	A	2	1.09
gcc	A	0	1.00
gca	A	2	1.62
gcg	A	0	0.29
---	--	------	------
mPD		0.014	0.68
		nPD:	0.02
		N. weight:	0.88
		Sc. PD:	-0.036
		Sc. rank:	-2240.4

,

PB1			
Pos.	297	obs:	exp:
aaa	K	0	0.66
aag	K	1	0.34
gat	D	2	1.70
gac	D	1	1.30
gaa	E	1143	23180.00
gag	E	38175	16138.00
---	--	------	------
mPD		0.057	0.48
		nPD:	0.12
		N. weight:	0.82
		Sc. PD:	0.044
		Sc. rank:	18715.0

,

PB1			
Pos.	298	obs:	exp:
ttt	F	1	0.49
ttc	F	0	0.51
tta	L	0	4554.00
ttg	L	0	8115.00
ctt	L	39165	8210.00
ctc	L	73	5756.00
cta	L	1	6027.00
ctg	L	10	6587.00
cct	P	1	0.33
ccc	P	0	0.19
cca	P	0	0.34
ccg	P	0	0.14
cat	H	1	0.56
cac	H	0	0.44
att	I	69	24.57
atc	I	1	18.39
ata	I	0	27.03
---	--	------	------
mPD		0.0080	1.2
		nPD:	0.01
		N. weight:	1.7
		Sc. PD:	-0.092
		Sc. rank:	-32140.0

,

PB1			
Pos.	299	obs:	exp:
tct	S	38173	6345.00
tcc	S	1126	5876.00
tca	S	11	10569.00
tcg	S	12	1548.00
agt	S	0	6872.00
agc	S	0	8112.00
---	--	------	------
mPD		0.057	1.6
		nPD:	0.03
		N. weight:	1.9
		Sc. PD:	-0.05
		Sc. rank:	14886.0

,

PB1			
Pos.	300	obs:	exp:
ttt	F	101	19127.00
ttc	F	39221	20195.00
---	--	------	------
mPD		0.0051	0.50
		nPD:	0.01
		N. weight:	0.71
		Sc. PD:	-0.036
		Sc. rank:	-9231.3

,

PB1			
Pos.	301	obs:	exp:
att	I	0	0.70
atc	I	0	0.53
ata	I	2	0.77
act	T	11	12211.00
acc	T	28	6952.00
aca	T	39148	17017.00
acg	T	133	3140.00
---	--	------	------
mPD		0.0088	0.68
		nPD:	0.01
		N. weight:	0.89
		Sc. PD:	-0.043
		Sc. rank:	-8776.4

,

PB1			
Pos.	302	obs:	exp:
tta	L	0	0.12
ttg	L	0	0.21
ctt	L	0	0.21
ctc	L	1	0.15
cta	L	0	0.15
ctg	L	0	0.17
att	I	308	13799.00
atc	I	38956	10329.00
ata	I	45	15181.00
gtt	V	0	3.01
gtc	V	12	2.45
gta	V	0	2.60
gtg	V	0	3.95
---	--	------	------
mPD		0.018	0.66
		nPD:	0.03
		N. weight:	1.4
		Sc. PD:	-0.047
		Sc. rank:	7106.3

,

PB1			
Pos.	303	obs:	exp:
act	T	39188	12212.00
acc	T	83	6952.00
aca	T	27	17018.00
acg	T	24	3141.00
---	--	------	------
mPD		0.0068	0.68
		nPD:	0.01
		N. weight:	1.3
		Sc. PD:	-0.064
		Sc. rank:	-16643.0

,

PB1			
Pos.	304	obs:	exp:
ggt	G	51	5294.00
ggc	G	0	5488.00
gga	G	17128	18152.00
ggg	G	22143	10388.00
---	--	------	------
mPD		0.49	0.68
		nPD:	0.73
		N. weight:	0.43
		Sc. PD:	0.28
		Sc. rank:	13343.0

,

PB1			
Pos.	305	obs:	exp:
gat	D	183	22226.00
gac	D	39137	17094.00
gaa	E	1	0.59
gag	E	0	0.41
ggt	G	0	0.13
ggc	G	1	0.14
gga	G	0	0.46
ggg	G	0	0.26
---	--	------	------
mPD		0.0094	0.49
		nPD:	0.02
		N. weight:	0.88
		Sc. PD:	-0.037
		Sc. rank:	-2766.1

,

PB1			
Pos.	306	obs:	exp:
cat	H	0	0.56
cac	H	1	0.44
aat	N	287	21999.00
aac	N	39034	17322.00
---	--	------	------
mPD		0.015	0.49
		nPD:	0.03
		N. weight:	0.85
		Sc. PD:	-0.027
		Sc. rank:	4682.3

,

PB1			
Pos.	307	obs:	exp:
act	T	38882	12211.00
acc	T	417	6952.00
aca	T	16	17017.00
acg	T	6	3141.00
gct	A	0	0.27
gcc	A	1	0.25
gca	A	0	0.40
gcg	A	0	0.07
---	--	------	------
mPD		0.022	0.68
		nPD:	0.03
		N. weight:	1.2
		Sc. PD:	-0.035
		Sc. rank:	8397.1

,

PB1			
Pos.	308	obs:	exp:
atg	M	1	1.00
aaa	K	5391	25850.00
aag	K	33929	13470.00
gaa	E	0	0.59
gag	E	1	0.41
---	--	------	------
mPD		0.24	0.45
		nPD:	0.53
		N. weight:	0.64
		Sc. PD:	0.29
		Sc. rank:	19083.0

,

PB1			
Pos.	309	obs:	exp:
tgt	C	0	0.39
tgc	C	1	0.61
tgg	W	39321	39321.00
---	--	------	------
mPD		0.000051	0.000051
		nPD:	1.
		N. weight:	0.000014
		Sc. PD:	0.000013
		Sc. rank:	0.4

,

PB1			
Pos.	310	obs:	exp:
aat	N	38923	21998.00
aac	N	397	17322.00
aaa	K	0	0.66
aag	K	1	0.34
gat	D	1	0.57
gac	D	0	0.43
---	--	------	------
mPD		0.020	0.49
		nPD:	0.04
		N. weight:	0.58
		Sc. PD:	-0.012
		Sc. rank:	5912.6

,

PB1

Pos.	311	obs:	exp:
aaa	K	1	0.66
aag	K	0	0.34
gat	D	3	1.70
gac	D	0	1.30
gaa	E	39273	23180.00
gag	E	45	16138.00
---	--	------	------
mPD		0.0025	0.48
		nPD:	0.01
		N. weight:	0.57
		Sc. PD:	-0.032
		Sc. rank:	-12744.0

PB1

Pos.	312	obs:	exp:
tct	S	0	0.32
tcc	S	0	0.30
tca	S	0	0.54
tcg	S	0	0.08
aat	N	38917	21998.00
aac	N	403	17322.00
agt	S	2	0.35
agc	S	0	0.41
---	--	------	------
mPD		0.020	0.49
		nPD:	0.04
		N. weight:	0.58
		Sc. PD:	-0.012
		Sc. rank:	5969.2

PB1

Pos.	313	obs:	exp:
caa	Q	39205	24037.00
cag	Q	117	15285.00
---	--	------	------
mPD		0.0059	0.48
		nPD:	0.01
		N. weight:	0.52
		Sc. PD:	-0.025
		Sc. rank:	-5470.9

PB1

Pos.	314	obs:	exp:
tct	S	0	3.71
tcc	S	0	3.44
tca	S	0	6.18
tcg	S	0	0.91
tat	Y	0	2.11
tac	Y	4	1.89
aat	N	650	21984.00
aac	N	38645	17311.00
agt	S	0	4.02
agc	S	23	4.74
---	--	------	------
mPD		0.034	0.50
		nPD:	0.07
		N. weight:	0.8
		Sc. PD:	0.0046
		Sc. rank:	14181.0

PB1

Pos.	315	obs:	exp:
cct	P	3274	13104.00
ccc	P	36039	7372.00
cca	P	8	13285.00
ccg	P	1	5562.00
---	--	------	------
mPD		0.15	0.72
		nPD:	0.21
		N. weight:	1.5
		Sc. PD:	0.21
		Sc. rank:	38809.0

PB1

Pos.	316	obs:	exp:
tta	L	0	0.12
ttg	L	0	0.21
ctt	L	0	0.21
ctc	L	0	0.15
cta	L	1	0.15
ctg	L	0	0.17
cgt	R	6	779.60
cgc	R	1	1415.00
cga	R	39089	3588.00
cgg	R	142	3376.00
aga	R	83	19286.00
agg	R	0	10876.00
---	--	------	------
mPD		0.012	0.89
		nPD:	0.01
		N. weight:	2.6
		Sc. PD:	-0.12
		Sc. rank:	-22979.0

PB1

Pos.	317	obs:	exp:
att	I	7	44.23
atc	I	1	33.11
ata	I	118	48.66
atg	M	39164	39164.00
act	T	0	2.79
acc	T	0	1.59
aca	T	0	3.89
acg	T	9	0.72
gtt	V	0	5.76
gtc	V	0	4.69
gta	V	0	4.99
gtg	V	23	7.57
---	--	------	------
mPD		0.0080	0.0092
		nPD:	0.87
		N. weight:	0.0039
		Sc. PD:	0.0031
		Sc. rank:	120.3

PB1

Pos.	318	obs:	exp:
ttt	F	38563	19127.00
ttc	F	758	20194.00
att	I	1	0.35
atc	I	0	0.26
ata	I	0	0.39
---	--	------	------
mPD		0.038	0.50
		nPD:	0.08
		N. weight:	0.69
		Sc. PD:	0.0088
		Sc. rank:	13371.0

PB1

Pos.	319	obs:	exp:
tta	L	57	4563.00
ttg	L	38043	8130.00
ctt	L	0	8226.00
ctc	L	0	5766.00
cta	L	58	6038.00
ctg	L	1164	6599.00
---	--	------	------
mPD		0.066	1.2
		nPD:	0.06
		N. weight:	1.6
		Sc. PD:	-0.0085
		Sc. rank:	24941.0

PB1

Pos.	320	obs:	exp:
act	T	0	0.93
acc	T	0	0.53
aca	T	1	1.30
acg	T	2	0.24
gtt	V	0	0.25
gtc	V	0	0.20
gta	V	1	0.22
gtg	V	0	0.33
gct	A	86	10716.00
gcc	A	4	9823.00
gca	A	16922	15904.00
gcg	A	22306	2874.00
---	--	------	------
mPD		0.49	0.69
		nPD:	0.71
		N. weight:	1.3
		Sc. PD:	0.82
		Sc. rank:	39741.0

PB1

Pos.	321	obs:	exp:
atg	M	39322	39322.00
---	--	------	------
mPD		0	0
		nPD:	1.
		N. weight:	0.
		Sc. PD:	0
		Sc. rank:	0

PB1

Pos.	322	obs:	exp:
att	I	39083	13797.00
atc	I	205	10327.00
ata	I	14	15178.00
gtt	V	20	5.01
gtc	V	0	4.08
gta	V	0	4.33
gtg	V	0	6.58
---	--	------	------
mPD		0.012	0.66
		nPD:	0.02
		N. weight:	1.1
		Sc. PD:	-0.048
		Sc. rank:	-4115.4

PB1

Pos.	323	obs:	exp:
act	T	5	12211.00
acc	T	21	6952.00
aca	T	39099	17017.00
acg	T	196	3141.00
gct	A	0	0.27
gcc	A	0	0.25
gca	A	1	0.40
gcg	A	0	0.07
---	--	------	------
mPD		0.011	0.68
		nPD:	0.02
		N. weight:	0.89
		Sc. PD:	-0.04
		Sc. rank:	-4557.2

PB1

Pos.	324	obs:	exp:
tct	S	1	0.32
tcc	S	1	0.30
tca	S	0	0.54
tcg	S	0	0.08
tat	Y	7625	20725.00
tac	Y	31693	18593.00
tgt	C	0	0.39
tgc	C	1	0.61
cat	H	1	0.56
cac	H	0	0.44
agt	S	0	0.35
agc	S	0	0.41
---	--	------	------
mPD		0.31	0.50
		nPD:	0.63
		N. weight:	0.26
		Sc. PD:	0.14
		Sc. rank:	7845.0

PB1

Pos.	325	obs:	exp:
ttt	F	0	0.49
ttc	F	1	0.51
att	I	78	13802.00
atc	I	39175	10331.00
ata	I	65	15185.00
atg	M	3	3.00
---	--	------	------
mPD		0.0075	0.66
		nPD:	0.01
		N. weight:	1.4
		Sc. PD:	-0.072
		Sc. rank:	-16824.0

PB1

Pos.	326	obs:	exp:
cct	P	0	0.33
ccc	P	0	0.19
cca	P	1	0.34
ccg	P	0	0.14
act	T	2	12211.00
acc	T	234	6952.00
aca	T	39078	17017.00
acg	T	7	3141.00
---	--	------	------
mPD		0.012	0.68
		nPD:	0.02
		N. weight:	0.88
		Sc. PD:	-0.038
		Sc. rank:	-3484.9

PB1			
Pos.	327	obs:	exp:
cgt	R	0	5.57
cgc	R	0	10.11
cga	R	0	25.64
cgg	R	0	24.13
aaa	K	5739	25667.00
aag	K	33302	13374.00
aga	R	210	137.80
agg	R	71	77.72
---	--	------	------
mPD		0.27	0.47
		nPD:	0.58
		N. weight:	0.61
		Sc. PD:	0.31
		Sc. rank:	18376.0

,

PB1			
Pos.	328	obs:	exp:
tct	S	0	2.10
tcc	S	0	1.94
tca	S	0	3.49
tcg	S	0	0.51
cat	H	3	2.26
cac	H	1	1.74
cgt	R	0	0.02
cgc	R	0	0.04
cga	R	0	0.09
cgg	R	0	0.09
act	T	2	0.93
acc	T	1	0.53
aca	T	0	1.30
acg	T	0	0.24
aat	N	22638	21964.00
aac	N	16620	17294.00
aaa	K	17	11.18
aag	K	0	5.82
agt	S	7	2.27
agc	S	6	2.68
aga	R	1	0.49
agg	R	0	0.28
gat	D	7	14.70
gac	D	19	11.30
---	--	------	------
mPD		0.49	0.50
		nPD:	0.99
		N. weight:	0.0017
		Sc. PD:	0.0016
		Sc. rank:	53.3

,

PB1			
Pos.	329	obs:	exp:
cct	P	0	0.33
ccc	P	0	0.19
cca	P	1	0.34
ccg	P	0	0.14
caa	Q	38171	24035.00
cag	Q	1148	15284.00
cgt	R	0	0.04
cgc	R	0	0.07
cga	R	2	0.18
cgg	R	0	0.17
aga	R	0	0.98
agg	R	0	0.55
---	--	------	------
mPD		0.057	0.48
		nPD:	0.12
		N. weight:	0.41
		Sc. PD:	0.023
		Sc. rank:	9389.5

,

PB1			
Pos.	330	obs:	exp:
tta	L	0	0.12
ttg	L	0	0.21
tct	S	2	0.32
tcc	S	0	0.30
tca	S	0	0.54
tcg	S	0	0.08
ctt	L	1	0.21
ctc	L	0	0.15
cta	L	0	0.15
ctg	L	0	0.17
cct	P	39039	13103.00
ccc	P	243	7371.00
cca	P	16	13284.00
ccg	P	21	5561.00
agt	S	0	0.35
agc	S	0	0.41
---	--	------	------
mPD		0.014	0.72
		nPD:	0.02
		N. weight:	1.2
		Sc. PD:	-0.048
		Sc. rank:	-3047.9

,

PB1			
Pos.	331	obs:	exp:
aat	N	1	0.56
aac	N	0	0.44
aaa	K	21	14.46
aag	K	1	7.54
gtt	V	0	1.50
gtc	V	0	1.22
gta	V	5	1.30
gtg	V	1	1.97
gct	A	0	0.27
gcc	A	0	0.25
gca	A	1	0.40
gcg	A	0	0.07
gat	D	375	223.30
gac	D	20	171.70
gaa	E	32364	22922.00
gag	E	6516	15958.00
ggt	G	0	2.29
ggc	G	0	2.37
gga	G	14	7.85
ggg	G	3	4.49
---	--	------	------
mPD		0.30	0.50
		nPD:	0.6
		N. weight:	0.15
		Sc. PD:	0.08
		Sc. rank:	4643.5

,

PB1			
Pos.	332	obs:	exp:
tgg	W	39322	39322.00
---	--	------	------
mPD		0	0
		nPD:	1.
		N. weight:	0.
		Sc. PD:	0
		Sc. rank:	0

,

PB1			
Pos.	333	obs:	exp:
ttt	F	69	19121.00
ttc	F	39241	20189.00
tta	L	7	1.39
ttg	L	0	2.48
ctt	L	0	2.51
ctc	L	5	1.76
cta	L	0	1.84
ctg	L	0	2.01
---	--	------	------
mPD		0.0041	0.50
		nPD:	0.01
		N. weight:	0.72
		Sc. PD:	-0.038
		Sc. rank:	-11707.0

,

PB1			
Pos.	334	obs:	exp:
cgt	R	0	779.60
cgc	R	0	1415.00
cga	R	6	3588.00
cgg	R	0	3376.00
aaa	K	2	1.31
aag	K	0	0.69
aga	R	39242	19286.00
agg	R	72	10876.00
---	--	------	------
mPD		0.0041	0.89
		nPD:	0.
		N. weight:	0.77
		Sc. PD:	-0.043
		Sc. rank:	-18097.0

,

PB1			
Pos.	335	obs:	exp:
cat	H	0	1.13
cac	H	2	0.87
aat	N	48	21998.00
aac	N	39272	17322.00
---	--	------	------
mPD		0.0025	0.49
		nPD:	0.01
		N. weight:	0.89
		Sc. PD:	-0.05
		Sc. rank:	-19621.0

,

PB1			
Pos.	336	obs:	exp:
att	I	106	13778.00
atc	I	37009	10313.00
ata	I	2134	15158.00
act	T	0	0.31
acc	T	1	0.18
aca	T	0	0.43
acg	T	0	0.08
gtt	V	32	17.53
gtc	V	37	14.26
gta	V	1	15.17
gtg	V	0	23.03
ggt	G	0	0.27
ggc	G	2	0.28
gga	G	0	0.92
ggg	G	0	0.53
---	--	------	------
mPD		0.11	0.66
		nPD:	0.17
		N. weight:	1.2
		Sc. PD:	0.13
		Sc. rank:	30313.0

,

PB1			
Pos.	337	obs:	exp:
tta	L	2	4562.00
ttg	L	71	8128.00
ctt	L	111	8224.00
ctc	L	0	5765.00
cta	L	512	6037.00
ctg	L	38617	6598.00
caa	Q	0	2.45
cag	Q	4	1.55
cgt	R	0	0.02
cgc	R	0	0.04
cga	R	0	0.09
cgg	R	1	0.09
atg	M	4	4.00
aga	R	0	0.49
agg	R	0	0.28
---	--	------	------
mPD		0.036	1.2
		nPD:	0.03
		N. weight:	1.8
		Sc. PD:	-0.057
		Sc. rank:	10952.0

,

PB1			
Pos.	338	obs:	exp:
tct	S	0	6345.00
tcc	S	0	5876.00
tca	S	0	10568.00
tcg	S	0	1548.00
act	T	0	0.93
acc	T	3	0.53
aca	T	0	1.30
acg	T	0	0.24
agt	S	342	6871.00
agc	S	38977	8111.00
---	--	------	------
mPD		0.017	1.6
		nPD:	0.01
		N. weight:	1.7
		Sc. PD:	-0.085
		Sc. rank:	-20806.0

,

PB1

Pos.	339	obs:	exp:
tta	L	0	0.12
ttg	L	0	0.21
ctt	L	0	0.21
ctc	L	1	0.15
cta	L	0	0.15
ctg	L	0	0.17
att	I	325	13721.00
atc	I	38637	10270.00
ata	I	124	15095.00
atg	M	220	220.00
act	T	0	0.62
acc	T	2	0.35
aca	T	0	0.87
acg	T	0	0.16
gtt	V	0	3.26
gtc	V	13	2.65
gta	V	0	2.82
gtg	V	0	4.28
---	--	------	------
mPD		0.034	0.66
		nPD:	0.05
		N. weight:	1.4
		Sc. PD:	-0.014
		Sc. rank:	19898.0

,

PB1

Pos.	340	obs:	exp:
tta	L	1	0.12
ttg	L	0	0.21
ctt	L	0	0.21
ctc	L	0	0.15
cta	L	0	0.15
ctg	L	0	0.17
gct	A	25	10717.00
gcc	A	7	9824.00
gca	A	39194	15905.00
gcg	A	94	2874.00
ggt	G	0	0.13
ggc	G	0	0.14
gga	G	1	0.46
ggg	G	0	0.26
---	--	------	------
mPD		0.0065	0.69
		nPD:	0.01
		N. weight:	0.97
		Sc. PD:	-0.05
		Sc. rank:	-13567.0

,

PB1

Pos.	341	obs:	exp:
cct	P	4	13104.00
ccc	P	486	7372.00
cca	P	38787	13285.00
ccg	P	45	5562.00
---	--	------	------
mPD		0.027	0.72
		nPD:	0.04
		N. weight:	1.1
		Sc. PD:	-0.027
		Sc. rank:	10155.0

,

PB1

Pos.	342	obs:	exp:
tta	L	1	0.12
ttg	L	0	0.21
ctt	L	0	0.21
ctc	L	0	0.15
cta	L	0	0.15
ctg	L	0	0.17
att	I	2	13793.00
atc	I	3	10324.00
ata	I	39286	15174.00
atg	M	2	2.00
act	T	0	0.31
acc	T	0	0.18
aca	T	1	0.43
acg	T	0	0.08
gtt	V	0	6.76
gtc	V	0	5.50
gta	V	27	5.85
gtg	V	0	8.88
---	--	------	------
mPD		0.0018	0.66
		nPD:	0.
		N. weight:	1.
		Sc. PD:	-0.061
		Sc. rank:	-27725.0

,

PB1

Pos.	343	obs:	exp:
tta	L	0	0.23
ttg	L	0	0.41
ctt	L	0	0.42
ctc	L	0	0.29
cta	L	0	0.31
ctg	L	2	0.34
att	I	0	1.76
atc	I	0	1.31
ata	I	5	1.93
atg	M	39315	39315.00
---	--	------	------
mPD		0.00036	0.00042
		nPD:	0.85
		N. weight:	0.00023
		Sc. PD:	0.00018
		Sc. rank:	7.2

,

PB1

Pos.	344	obs:	exp:
ttt	F	112	19127.00
ttc	F	39210	20195.00
---	--	------	------
mPD		0.0057	0.50
		nPD:	0.01
		N. weight:	0.71
		Sc. PD:	-0.035
		Sc. rank:	-8241.7

,

PB1

Pos.	345	obs:	exp:
tct	S	2	6345.00
tcc	S	2	5876.00
tca	S	39296	10569.00
tcg	S	21	1548.00
act	T	0	0.31
acc	T	0	0.18
aca	T	1	0.43
acg	T	0	0.08
agt	S	0	6871.00
agc	S	0	8112.00
---	--	------	------
mPD		0.0013	1.6
		nPD:	0.
		N. weight:	1.4
		Sc. PD:	-0.086
		Sc. rank:	-43634.0

,

PB1

Pos.	346	obs:	exp:
tct	S	0	1.13
tcc	S	0	1.05
tca	S	0	1.88
tcg	S	0	0.28
aat	N	2266	21996.00
aac	N	37049	17319.00
agt	S	0	1.22
agc	S	7	1.44
---	--	------	------
mPD		0.11	0.49
		nPD:	0.22
		N. weight:	0.64
		Sc. PD:	0.099
		Sc. rank:	17174.0

,

PB1

Pos.	347	obs:	exp:
aaa	K	39227	25852.00
aag	K	95	13470.00
---	--	------	------
mPD		0.0048	0.45
		nPD:	0.01
		N. weight:	0.44
		Sc. PD:	-0.022
		Sc. rank:	-5438.3

,

PB1

Pos.	348	obs:	exp:
tta	L	0	0.12
ttg	L	0	0.21
ctt	L	0	0.21
ctc	L	0	0.15
cta	L	0	0.15
ctg	L	1	0.17
att	I	0	0.35
atc	I	0	0.26
ata	I	1	0.39
atg	M	39319	39319.00
aaa	K	0	0.66
aag	K	1	0.34
---	--	------	------
mPD		0.00015	0.00022
		nPD:	0.7
		N. weight:	0.00011
		Sc. PD:	0.000066
		Sc. rank:	3.2

,

PB1

Pos.	349	obs:	exp:
gct	A	13	10717.00
gcc	A	1	9824.00
gca	A	39178	15906.00
gcg	A	130	2875.00
---	--	------	------
mPD		0.0073	0.69
		nPD:	0.01
		N. weight:	0.97
		Sc. PD:	-0.049
		Sc. rank:	-12175.0

,

PB1

Pos.	350	obs:	exp:
cgt	R	0	778.30
cgc	R	0	1412.00
cga	R	28	3581.00
cgg	R	0	3371.00
aaa	K	68	44.71
aag	K	0	23.29
aga	R	38957	19253.00
agg	R	268	10857.00
ggt	G	0	0.13
ggc	G	0	0.14
gga	G	1	0.46
ggg	G	0	0.26
---	--	------	------
mPD		0.018	0.89
		nPD:	0.02
		N. weight:	0.74
		Sc. PD:	-0.03
		Sc. rank:	-1266.0

,

PB1

Pos.	351	obs:	exp:
tta	L	23	4561.00
ttg	L	958	8127.00
ctt	L	13	8223.00
ctc	L	0	5764.00
cta	L	1345	6036.00
ctg	L	36969	6597.00
att	I	0	0.70
atc	I	0	0.53
ata	I	2	0.77
atg	M	12	12.00
---	--	------	------
mPD		0.12	1.2
		nPD:	0.1
		N. weight:	1.7
		Sc. PD:	0.062
		Sc. rank:	36277.0

,

PB1

Pos.	352	obs:	exp:
cgt	R	0	0.04
cgc	R	0	0.07
cga	R	2	0.18
cgg	R	0	0.17
aga	R	0	0.98
agg	R	0	0.55
ggt	G	7	5293.00
ggc	G	0	5488.00
gga	G	38290	18151.00
ggg	G	1023	10388.00
---	--	------	------
mPD		0.051	0.68
		nPD:	0.08
		N. weight:	0.73
		Sc. PD:	0.009
		Sc. rank:	14211.0

,

PB1

Pos.	353	obs:	exp:
caa	Q	1	0.61
cag	Q	0	0.39
cgt	R	0	3.41
cgc	R	0	6.19
cga	R	0	15.69
cgg	R	0	14.77
aat	N	0	0.56
aac	N	1	0.44
aaa	K	39089	25737.00
aag	K	59	13411.00
aga	R	172	84.36
agg	R	0	47.58
---	--	------	------
mPD		0.012	0.46
		nPD:	0.03
		N. weight:	0.45
		Sc. PD:	-0.016
		Sc. rank:	1336.7

,

PB1

Pos.	354	obs:	exp:
ggt	G	14	5294.00
ggc	G	5	5488.00
gga	G	38826	18152.00
ggg	G	477	10388.00
---	--	------	------
mPD		0.025	0.68
		nPD:	0.04
		N. weight:	0.78
		Sc. PD:	-0.02
		Sc. rank:	6853.9

,

PB1			
Pos.	355	obs:	exp:
ttt	F	0	1.46
ttc	F	3	1.54
tct	S	0	0.32
tcc	S	2	0.30
tca	S	0	0.54
tcg	S	0	0.08
tat	Y	114	20723.00
tac	Y	39201	18592.00
cat	H	0	1.13
cac	H	2	0.87
agt	S	0	0.35
agc	S	0	0.41
---	--	------	------
mPD		0.0061	0.50
		nPD:	0.01
		N. weight:	0.8
		Sc. PD:	-0.039
		Sc. rank:	-8654.6

,

PB1			
Pos.	356	obs:	exp:
atg	M	39322	39322.00
---	--	------	------
mPD		0	0
		nPD:	1.
		N. weight:	0.
		Sc. PD:	0
		Sc. rank:	0

,

PB1			
Pos.	357	obs:	exp:
ttt	F	1575	19127.00
ttc	F	37747	20195.00
---	--	------	------
mPD		0.077	0.50
		nPD:	0.15
		N. weight:	0.55
		Sc. PD:	0.049
		Sc. rank:	13723.0

,

PB1			
Pos.	358	obs:	exp:
aaa	K	0	1.97
aag	K	3	1.03
gaa	E	318	23174.00
gag	E	38989	16133.00
ggt	G	0	1.62
ggc	G	0	1.67
gga	G	0	5.54
ggg	G	12	3.17
---	--	------	------
mPD		0.017	0.48
		nPD:	0.03
		N. weight:	0.92
		Sc. PD:	-0.025
		Sc. rank:	7151.4

,

PB1			
Pos.	359	obs:	exp:
tct	S	0	6345.00
tcc	S	0	5876.00
tca	S	0	10569.00
tcg	S	0	1548.00
agt	S	39167	6872.00
agc	S	155	8112.00
---	--	------	------
mPD		0.0079	1.6
		nPD:	0.
		N. weight:	1.9
		Sc. PD:	-0.11
		Sc. rank:	-43963.0

,

PB1			
Pos.	360	obs:	exp:
caa	Q	0	1.22
cag	Q	2	0.78
cgt	R	0	0.46
cgc	R	0	0.83
cga	R	0	2.10
cgg	R	0	1.97
atg	M	1	1.00
aat	N	4	2.24
aac	N	0	1.76
aaa	K	1417	25830.00
aag	K	37872	13459.00
aga	R	0	11.28
agg	R	23	6.36
gaa	E	0	1.77
gag	E	3	1.23
---	--	------	------
mPD		0.071	0.45
		nPD:	0.16
		N. weight:	0.98
		Sc. PD:	0.091
		Sc. rank:	24557.0

,

PB1			
Pos.	361	obs:	exp:
tct	S	0	5.33
tcc	S	0	4.93
tca	S	0	8.87
tcg	S	0	1.30
cgt	R	0	760.60
cgc	R	0	1380.00
cga	R	3	3500.00
cgg	R	0	3294.00
att	I	0	1.40
atc	I	0	1.05
ata	I	4	1.54
aaa	K	907	604.80
aag	K	13	315.20
agt	S	4	5.77
agc	S	29	6.81
aga	R	38027	18815.00
agg	R	330	10610.00
ggt	G	0	0.67
ggc	G	0	0.70
gga	G	5	2.31
ggg	G	0	1.32
---	--	------	------
mPD		0.065	0.93
		nPD:	0.07
		N. weight:	0.72
		Sc. PD:	0.0056
		Sc. rank:	13067.0

,

PB1			
Pos.	362	obs:	exp:
tta	L	0	0.12
ttg	L	1	0.21
ctt	L	0	0.21
ctc	L	0	0.15
cta	L	0	0.15
ctg	L	0	0.17
att	I	1	1.05
atc	I	0	0.79
ata	I	2	1.16
atg	M	39317	39317.00
aaa	K	0	0.66
aag	K	1	0.34
---	--	------	------
mPD		0.00025	0.00032
		nPD:	0.8
		N. weight:	0.00013
		Sc. PD:	0.00009
		Sc. rank:	3.9

,

PB1			
Pos.	363	obs:	exp:
cgt	R	0	0.26
cgc	R	0	0.47
cga	R	0	1.19
cgg	R	0	1.12
aaa	K	387	25843.00
aag	K	38922	13466.00
aga	R	0	6.38
agg	R	13	3.60
---	--	------	------
mPD		0.020	0.45
		nPD:	0.04
		N. weight:	1.1
		Sc. PD:	-0.019
		Sc. rank:	12960.0

,

PB1			
Pos.	364	obs:	exp:
ttt	F	0	4.38
ttc	F	9	4.62
tta	L	0	4283.00
ttg	L	0	7631.00
ctt	L	195	7722.00
ctc	L	36690	5413.00
cta	L	27	5668.00
ctg	L	0	6195.00
att	I	30	839.70
atc	I	2360	628.50
ata	I	2	923.80
atg	M	1	1.00
act	T	0	1.55
acc	T	5	0.88
aca	T	0	2.16
acg	T	0	0.40
gtt	V	0	0.75
gtc	V	3	0.61
gta	V	0	0.65
gtg	V	0	0.99
---	--	------	------
mPD		0.13	1.2
		nPD:	0.1
		N. weight:	2.
		Sc. PD:	0.082
		Sc. rank:	44502.0

,

PB1			
Pos.	365	obs:	exp:
cgt	R	2	779.70
cgc	R	0	1415.00
cga	R	2146	3588.00
cgg	R	36925	3376.00
aga	R	5	19287.00
agg	R	244	10876.00
---	--	------	------
mPD		0.12	0.89
		nPD:	0.13
		N. weight:	2.4
		Sc. PD:	0.16
		Sc. rank:	57425.0

,

PB1			
Pos.	366	obs:	exp:
tct	S	0	0.16
tcc	S	0	0.15
tca	S	1	0.27
tcg	S	0	0.04
act	T	5	12205.00
acc	T	4	6949.00
aca	T	39169	17009.00
acg	T	124	3139.00
agt	S	0	0.17
agc	S	0	0.21
gct	A	0	5.18
gcc	A	0	4.75
gca	A	19	7.69
gcg	A	0	1.39
---	--	------	------
mPD		0.0078	0.68
		nPD:	0.01
		N. weight:	0.9
		Sc. PD:	-0.045
		Sc. rank:	-10374.0

,

PB1

Pos.	367	obs:	exp:
cct	P	0	0.33
ccc	P	0	0.19
cca	P	1	0.34
ccg	P	0	0.14
caa	Q	39097	24034.00
cag	Q	220	15283.00
cgt	R	0	0.04
cgc	R	0	0.07
cga	R	2	0.18
cgg	R	0	0.17
aaa	K	2	1.31
aag	K	0	0.69
aga	R	0	0.98
agg	R	0	0.55
---	--	------	------
mPD		0.011	0.48
		nPD:	0.02
		N. weight:	0.5
		Sc. PD:	-0.019
		Sc. rank:	581.2

,

PB1

Pos.	368	obs:	exp:
tta	L	0	0.58
ttg	L	0	1.03
ctt	L	0	1.05
ctc	L	0	0.73
cta	L	5	0.77
ctg	L	0	0.84
att	I	0	13779.00
atc	I	8	10314.00
ata	I	39245	15159.00
atg	M	3	3.00
act	T	0	0.31
acc	T	0	0.18
aca	T	1	0.43
acg	T	0	0.08
aaa	K	1	0.66
aag	K	0	0.34
gtt	V	0	14.78
gtc	V	0	12.02
gta	V	59	12.79
gtg	V	0	19.41
---	--	------	------
mPD		0.0039	0.66
		nPD:	0.01
		N. weight:	1.
		Sc. PD:	-0.058
		Sc. rank:	-21960.0

,

PB1

Pos.	369	obs:	exp:
tta	L	0	0.12
ttg	L	0	0.21
tct	S	41	6.78
tcc	S	0	6.28
tca	S	1	11.29
tcg	S	0	1.65
ctt	L	1	0.21
ctc	L	0	0.15
cta	L	0	0.15
ctg	L	0	0.17
cct	P	38265	13089.00
ccc	P	450	7363.00
cca	P	554	13270.00
ccg	P	8	5555.00
act	T	2	0.62
acc	T	0	0.35
aca	T	0	0.87
acg	T	0	0.16
agt	S	0	7.34
agc	S	0	8.66
---	--	------	------
mPD		0.053	0.72
		nPD:	0.07
		N. weight:	1.1
		Sc. PD:	0.011
		Sc. rank:	20446.0

,

PB1

Pos.	370	obs:	exp:
tct	S	0	0.97
tcc	S	0	0.90
tca	S	6	1.61
tcg	S	0	0.24
act	T	0	1.55
acc	T	0	0.88
aca	T	5	2.16
acg	T	0	0.40
agt	S	0	1.05
agc	S	0	1.24
gtt	V	0	0.50
gtc	V	0	0.41
gta	V	2	0.43
gtg	V	0	0.66
gct	A	22	10714.00
gcc	A	4	9821.00
gca	A	39263	15901.00
gcg	A	20	2874.00
---	--	------	------
mPD		0.0030	0.70
		nPD:	0.
		N. weight:	0.98
		Sc. PD:	-0.056
		Sc. rank:	-23650.0

,

PB1

Pos.	371	obs:	exp:
gat	D	0	4.52
gac	D	8	3.48
gaa	E	39233	23177.00
gag	E	79	16135.00
ggt	G	0	0.27
ggc	G	0	0.28
gga	G	2	0.92
ggg	G	0	0.53
---	--	------	------
mPD		0.0045	0.48
		nPD:	0.01
		N. weight:	0.56
		Sc. PD:	-0.029
		Sc. rank:	-8077.4

,

PB1

Pos.	372	obs:	exp:
tta	L	0	1.16
ttg	L	6	2.07
ctt	L	0	2.09
ctc	L	0	1.47
cta	L	0	1.54
ctg	L	4	1.68
cgt	R	0	0.04
cgc	R	0	0.07
cga	R	0	0.18
cgg	R	0	0.17
att	I	6	26.33
atc	I	0	19.71
ata	I	69	28.96
atg	M	39195	39195.00
act	T	0	1.55
acc	T	0	0.88
aca	T	1	2.16
acg	T	4	0.40
aaa	K	0	1.31
aag	K	2	0.69
aga	R	0	0.98
agg	R	2	0.55
gtt	V	0	8.27
gtc	V	0	6.72
gta	V	0	7.15
gtg	V	33	10.86
---	--	------	------
mPD		0.0065	0.0083
		nPD:	0.79
		N. weight:	0.0032
		Sc. PD:	0.0023
		Sc. rank:	98.7

,

PB1

Pos.	373	obs:	exp:
tta	L	49	4560.00
ttg	L	0	8124.00
ctt	L	3	8220.00
ctc	L	2	5762.00
cta	L	39074	6034.00
ctg	L	166	6595.00
cct	P	0	0.33
ccc	P	0	0.19
cca	P	1	0.34
ccg	P	0	0.14
att	I	0	9.13
atc	I	0	6.83
ata	I	26	10.04
gtt	V	0	0.25
gtc	V	0	0.20
gta	V	1	0.22
gtg	V	0	0.33
---	--	------	------
mPD		0.013	1.2
		nPD:	0.01
		N. weight:	2.
		Sc. PD:	-0.1
		Sc. rank:	-24366.0

,

PB1

Pos.	374	obs:	exp:
tct	S	0	32.43
tcc	S	0	30.04
tca	S	201	54.02
tcg	S	0	7.91
act	T	0	17.08
acc	T	0	9.72
aca	T	55	23.80
acg	T	0	4.39
agt	S	0	35.13
agc	S	0	41.47
gtt	V	0	5.76
gtc	V	0	4.69
gta	V	23	4.99
gtg	V	0	7.57
gct	A	8	10634.00
gcc	A	8	9748.00
gca	A	38946	15782.00
gcg	A	53	2852.00
gaa	E	27	15.92
gag	E	0	11.08
ggt	G	0	0.13
ggc	G	0	0.14
gga	G	1	0.46
ggg	G	0	0.26
---	--	------	------
mPD		0.019	0.71
		nPD:	0.03
		N. weight:	0.98
		Sc. PD:	-0.034
		Sc. rank:	4282.3

,

PB1

Pos.	375	obs:	exp:
tct	S	0	6289.00
tcc	S	0	5824.00
tca	S	0	10476.00
tcg	S	0	1534.00
tgt	C	0	0.78
tgc	C	2	1.22
cgt	R	0	0.12
cgc	R	3	0.22
cga	R	0	0.55
cgg	R	0	0.52
att	I	0	8.07
atc	I	23	6.04
ata	I	0	8.88
act	T	0	0.31
acc	T	1	0.18
aca	T	0	0.43
acg	T	0	0.08
aat	N	0	142.70
aac	N	255	112.30
agt	S	86	6811.00
agc	S	38889	8040.00
aga	R	3	2.94
agg	R	0	1.66
gat	D	0	1.70
gac	D	3	1.30
ggt	G	0	7.67
ggc	G	57	7.96
gga	G	0	26.31
ggg	G	0	15.06
---	--	------	------
mPD		0.022	1.6
		nPD:	0.01
		N. weight:	1.7
		Sc. PD:	-0.082
		Sc. rank:	-14805.0

,

PB1

Pos.	376	obs:	exp:
att	I	39113	13797.00
atc	I	185	10328.00
ata	I	6	15179.00
atg	M	2	2.00
act	T	3	0.93
acc	T	0	0.53
aca	T	0	1.30
acg	T	0	0.24
gtt	V	13	3.26
gtc	V	0	2.65
gta	V	0	2.82
gtg	V	0	4.28
---	--	------	------
mPD		0.011	0.66
		nPD:	0.02
		N. weight:	1.1
		Sc. PD:	-0.051
		Sc. rank:	-6460.4

,

PB1

Pos.	377	obs:	exp:
aat	N	0	12.87
aac	N	23	10.13
gat	D	223	22177.00
gac	D	39010	17056.00
gaa	E	60	35.37
gag	E	0	24.63
ggt	G	0	0.81
ggc	G	6	0.84
gga	G	0	2.77
ggg	G	0	1.59
---	--	------	------
mPD		0.016	0.49
		nPD:	0.03
		N. weight:	0.87
		Sc. PD:	-0.026
		Sc. rank:	5675.8

,

PB1

Pos.	378	obs:	exp:
tta	L	0	4561.00
ttg	L	125	8127.00
ctt	L	16	8223.00
ctc	L	1	5764.00
cta	L	202	6036.00
ctg	L	38965	6597.00
cct	P	0	0.33
ccc	P	0	0.19
cca	P	0	0.34
ccg	P	1	0.14
atg	M	12	12.00
---	--	------	------
mPD		0.018	1.2
		nPD:	0.02
		N. weight:	1.9
		Sc. PD:	-0.087
		Sc. rank:	-12248.0

,

PB1

Pos.	379	obs:	exp:
cgt	R	0	3.33
cgc	R	0	6.04
cga	R	0	15.33
cgg	R	0	14.43
act	T	0	0.31
acc	T	0	0.18
aca	T	0	0.43
acg	T	1	0.08
aat	N	12	6.71
aac	N	0	5.29
aaa	K	172	25733.00
aag	K	38969	13408.00
aga	R	0	82.40
agg	R	168	46.47
---	--	------	------
mPD		0.018	0.46
		nPD:	0.04
		N. weight:	1.1
		Sc. PD:	-0.027
		Sc. rank:	10834.0

,

PB1

Pos.	380	obs:	exp:
tat	Y	39207	20725.00
tac	Y	112	18594.00
cat	H	3	1.69
cac	H	0	1.31
---	--	------	------
mPD		0.0058	0.50
		nPD:	0.01
		N. weight:	0.68
		Sc. PD:	-0.034
		Sc. rank:	-7725.2

,

PB1

Pos.	381	obs:	exp:
ttt	F	799	19127.00
ttc	F	38522	20194.00
tta	L	1	0.12
ttg	L	0	0.21
ctt	L	0	0.21
ctc	L	0	0.15
cta	L	0	0.15
ctg	L	0	0.17
---	--	------	------
mPD		0.040	0.50
		nPD:	0.08
		N. weight:	0.62
		Sc. PD:	0.01
		Sc. rank:	12437.0

,

PB1

Pos.	382	obs:	exp:
tct	S	0	0.65
tcc	S	0	0.60
tca	S	0	1.08
tcg	S	0	0.16
aat	N	37458	21996.00
aac	N	1858	17320.00
aaa	K	2	1.31
aag	K	0	0.69
agt	S	4	0.70
agc	S	0	0.83
---	--	------	------
mPD		0.090	0.49
		nPD:	0.18
		N. weight:	0.44
		Sc. PD:	0.052
		Sc. rank:	11316.0

,

PB1

Pos.	383	obs:	exp:
aat	N	0	0.56
aac	N	1	0.44
aaa	K	23	15.12
aag	K	0	7.88
gtt	V	0	0.75
gtc	V	0	0.61
gta	V	3	0.65
gtg	V	0	0.99
gct	A	0	0.82
gcc	A	0	0.75
gca	A	3	1.21
gcg	A	0	0.22
gat	D	12	478.20
gac	D	834	367.80
gaa	E	38308	22656.00
gag	E	120	15772.00
ggt	G	0	2.42
ggc	G	0	2.51
gga	G	18	8.31
ggg	G	0	4.76
---	--	------	------
mPD		0.051	0.51
		nPD:	0.1
		N. weight:	0.56
		Sc. PD:	0.02
		Sc. rank:	12217.0

,

PB1

Pos.	384	obs:	exp:
tta	L	39	4.53
ttg	L	0	8.06
tct	S	3	6317.00
tcc	S	0	5850.00
tca	S	39111	10522.00
tcg	S	34	1541.00
ctt	L	0	8.16
ctc	L	0	5.72
cta	L	0	5.99
ctg	L	0	6.55
cct	P	0	34.32
ccc	P	0	19.31
cca	P	103	34.80
ccg	P	0	14.57
act	T	0	8.70
acc	T	0	4.95
aca	T	28	12.12
acg	T	0	2.24
agt	S	0	6841.00
agc	S	0	8076.00
gct	A	0	0.55
gcc	A	0	0.50
gca	A	2	0.81
gcg	A	0	0.15
gaa	E	2	1.18
gag	E	0	0.82
---	--	------	------
mPD		0.011	1.6
		nPD:	0.01
		N. weight:	1.4
		Sc. PD:	-0.078
		Sc. rank:	-28259.0

,

PB1

Pos.	385	obs:	exp:
cct	P	0	0.67
ccc	P	0	0.37
cca	P	2	0.68
ccg	P	0	0.28
act	T	6	12211.00
acc	T	0	6952.00
aca	T	39274	17017.00
acg	T	40	3140.00
---	--	------	------
mPD		0.0024	0.68
		nPD:	0.
		N. weight:	0.91
		Sc. PD:	-0.052
		Sc. rank:	-23129.0

,

PB1

Pos.	386	obs:	exp:
cgt	R	0	775.10
cgc	R	0	1407.00
cga	R	0	3567.00
cgg	R	2	3357.00
aaa	K	6	149.90
aag	K	222	78.10
aga	R	524	19174.00
agg	R	38567	10813.00
ggt	G	0	0.13
ggc	G	0	0.14
gga	G	0	0.46
ggg	G	1	0.26
---	--	------	------
mPD		0.038	0.90
		nPD:	0.04
		N. weight:	1.3
		Sc. PD:	-0.026
		Sc. rank:	14400.0

,

PB1

Pos.	387	obs:	exp:
caa	Q	0	0.61
cag	Q	1	0.39
cgt	R	0	1.45
cgc	R	0	2.63
cga	R	0	6.66
cgg	R	0	6.27
atg	M	4	4.00
act	T	0	1.86
acc	T	0	1.06
aca	T	0	2.60
acg	T	6	0.48
aat	N	18	10.07
aac	N	0	7.93
aaa	K	117	25781.00
aag	K	39097	13433.00
aga	R	1	35.81
agg	R	72	20.19
gaa	E	0	3.54
gag	E	6	2.46
---	--	------	------
mPD		0.011	0.46
		nPD:	0.03
		N. weight:	1.2
		Sc. PD:	-0.042
		Sc. rank:	2486.2

,

PB1

Pos.	388	obs:	exp:
cgt	R	0	0.28
cgc	R	0	0.50
cga	R	0	1.28
cgg	R	0	1.20
aaa	K	39193	25842.00
aag	K	115	13466.00
aga	R	14	6.87
agg	R	0	3.87
---	--	------	------
mPD		0.0065	0.45
		nPD:	0.01
		N. weight:	0.44
		Sc. PD:	-0.021
		Sc. rank:	-3412.5

,

PB1

Pos.	389	obs:	exp:
att	I	39222	13800.00
atc	I	55	10330.00
ata	I	36	15183.00
gtt	V	9	2.25
gtc	V	0	1.83
gta	V	0	1.95
gtg	V	0	2.96
---	--	------	------
mPD		0.0051	0.66
		nPD:	0.01
		N. weight:	1.1
		Sc. PD:	-0.06
		Sc. rank:	-19557.0

,

PB1

Pos.	390	obs:	exp:
aat	N	1	0.56
aac	N	0	0.44
aaa	K	3	17.75
aag	K	24	9.25
gtt	V	0	0.50
gtc	V	0	0.41
gta	V	0	0.43
gtg	V	2	0.66
gct	A	0	1.91
gcc	A	0	1.75
gca	A	0	2.83
gcg	A	7	0.51
gat	D	22	14.70
gac	D	4	11.30
gaa	E	528	23142.00
gag	E	38725	16111.00
ggt	G	0	0.81
ggc	G	0	0.84
gga	G	0	2.77
ggg	G	6	1.59
---	--	------	------
mPD		0.030	0.49
		nPD:	0.06
		N. weight:	0.89
		Sc. PD:	-0.00048
		Sc. rank:	15175.0

,

PB1

Pos.	391	obs:	exp:
cgt	R	0	1.51
cgc	R	0	2.73
cga	R	0	6.93
cgg	R	0	6.53
act	T	0	0.31
acc	T	0	0.18
aca	T	1	0.43
acg	T	0	0.08
aat	N	3	1.68
aac	N	0	1.32
aaa	K	39113	25799.00
aag	K	129	13443.00
aga	R	76	37.28
agg	R	0	21.02
---	--	------	------
mPD		0.011	0.46
		nPD:	0.02
		N. weight:	0.44
		Sc. PD:	-0.017
		Sc. rank:	310.7

,

PB1

Pos.	392	obs:	exp:
tta	L	1	0.23
ttg	L	0	0.41
ctt	L	0	0.42
ctc	L	0	0.29
cta	L	1	0.31
ctg	L	0	0.34
att	I	3	13759.00
atc	I	1047	10299.00
ata	I	38146	15137.00
atg	M	20	20.00
act	T	0	0.31
acc	T	0	0.18
aca	T	1	0.43
acg	T	0	0.08
gtt	V	0	25.80
gtc	V	0	20.99
gta	V	103	22.32
gtg	V	0	33.89
---	--	------	------
mPD		0.058	0.66
		nPD:	0.09
		N. weight:	0.92
		Sc. PD:	0.023
		Sc. rank:	19262.0

,

PB1

Pos.	393	obs:	exp:
cgt	R	0	779.40
cgc	R	0	1414.00
cga	R	3	3587.00
cgg	R	5	3375.00
act	T	0	0.31
acc	T	0	0.18
aca	T	0	0.43
acg	T	1	0.08
aaa	K	0	6.57
aag	K	10	3.43
aga	R	158	19280.00
agg	R	39143	10873.00
ggt	G	0	0.27
ggc	G	0	0.28
gga	G	0	0.92
ggg	G	2	0.53
---	--	------	------
mPD		0.0092	0.89
		nPD:	0.01
		N. weight:	1.4
		Sc. PD:	-0.07
		Sc. rank:	-17553.0

,

PB1

Pos.	394	obs:	exp:
tta	L	0	0.12
ttg	L	0	0.21
tct	S	10	1.61
tcc	S	0	1.49
tca	S	0	2.69
tcg	S	0	0.39
ctt	L	1	0.21
ctc	L	0	0.15
cta	L	0	0.15
ctg	L	0	0.17
cct	P	37607	13098.00
ccc	P	1693	7369.00
cca	P	3	13280.00
ccg	P	3	5559.00
cat	H	3	1.69
cac	H	0	1.31
act	T	2	0.62
acc	T	0	0.35
aca	T	0	0.87
acg	T	0	0.16
agt	S	0	1.75
agc	S	0	2.06
---	--	------	------
mPD		0.084	0.72
		nPD:	0.12
		N. weight:	1.
		Sc. PD:	0.054
		Sc. rank:	23555.0

,

PB1

Pos.	395	obs:	exp:
ttt	F	2	0.97
ttc	F	0	1.03
tta	L	0	4562.00
ttg	L	0	8127.00
ctt	L	39063	8223.00
ctc	L	226	5764.00
cta	L	15	6036.00
ctg	L	6	6597.00
att	I	10	3.51
atc	I	0	2.63
ata	I	0	3.86
---	--	------	------
mPD		0.013	1.2
		nPD:	0.01
		N. weight:	1.7
		Sc. PD:	-0.084
		Sc. rank:	-19818.0

,

PB1

Pos.	396	obs:	exp:
tta	L	258	4561.00
ttg	L	0	8126.00
ctt	L	2	8222.00
ctc	L	1	5763.00
cta	L	38975	6035.00
ctg	L	67	6596.00
att	I	0	6.67
atc	I	0	4.99
ata	I	19	7.34
---	--	------	------
mPD		0.018	1.2
		nPD:	0.02
		N. weight:	2.
		Sc. PD:	-0.092
		Sc. rank:	-13672.0

,

PB1

Pos.	397	obs:	exp:
tta	L	2	0.35
ttg	L	0	0.62
ctt	L	0	0.63
ctc	L	0	0.44
cta	L	1	0.46
ctg	L	0	0.50
cgt	R	0	0.02
cgc	R	0	0.04
cga	R	0	0.09
cgg	R	0	0.09
att	I	6	13774.00
atc	I	2	10310.00
ata	I	39230	15154.00
atg	M	40	40.00
act	T	0	9.94
acc	T	0	5.66
aca	T	32	13.85
acg	T	0	2.56
aga	R	1	0.49
agg	R	0	0.28
gtt	V	0	2.00
gtc	V	0	1.63
gta	V	8	1.73
gtg	V	0	2.63
---	--	------	------
mPD		0.0047	0.66
		nPD:	0.01
		N. weight:	1.
		Sc. PD:	-0.056
		Sc. rank:	-19463.0

,

PB1

Pos.	398	obs:	exp:
aat	N	21	11.75
aac	N	0	9.25
gtt	V	2	0.50
gtc	V	0	0.41
gta	V	0	0.43
gtg	V	0	0.66
gat	D	38793	22188.00
gac	D	459	17064.00
gaa	E	24	19.46
gag	E	9	13.54
ggt	G	14	1.88
ggc	G	0	1.95
gga	G	0	6.46
ggg	G	0	3.70
---	--	------	------
mPD		0.027	0.49
		nPD:	0.05
		N. weight:	0.56
		Sc. PD:	-0.0048
		Sc. rank:	8426.0

,

PB1			
Pos.	399	obs:	exp:
tct	S	0	1.13
tcc	S	0	1.05
tca	S	0	1.88
tcg	S	0	0.28
tgt	C	0	0.78
tgc	C	2	1.22
agt	S	0	1.22
agc	S	7	1.44
gat	D	0	6.22
gac	D	11	4.78
gaa	E	15	8.84
gag	E	0	6.16
ggt	G	180	5289.00
ggc	G	29964	5483.00
gga	G	9043	18136.00
ggg	G	100	10379.00
---	--	------	------
mPD		0.37	0.68
		nPD:	0.54
		N. weight:	1.2
		Sc. PD:	0.56
		Sc. rank:	36423.0

,

PB1			
Pos.	400	obs:	exp:
tct	S	0	0.16
tcc	S	0	0.15
tca	S	1	0.27
tcg	S	0	0.04
act	T	23	12205.00
acc	T	3	6948.00
aca	T	39203	17008.00
acg	T	71	3139.00
agt	S	0	0.17
agc	S	0	0.21
gct	A	0	5.72
gcc	A	0	5.25
gca	A	21	8.49
gcg	A	0	1.54
---	--	------	------
mPD		0.0060	0.68
		nPD:	0.01
		N. weight:	0.9
		Sc. PD:	-0.047
		Sc. rank:	-13616.0

,

PB1			
Pos.	401	obs:	exp:
tct	S	0	0.32
tcc	S	0	0.30
tca	S	2	0.54
tcg	S	0	0.08
agt	S	0	0.35
agc	S	0	0.41
gtt	V	3	7.26
gtc	V	0	5.91
gta	V	26	6.29
gtg	V	0	9.54
gct	A	463	10709.00
gcc	A	5	9816.00
gca	A	38735	15893.00
gcg	A	88	2872.00
---	--	------	------
mPD		0.030	0.70
		nPD:	0.04
		N. weight:	0.91
		Sc. PD:	-0.018
		Sc. rank:	9860.1

,

PB1			
Pos.	402	obs:	exp:
tct	S	2	6344.00
tcc	S	2	5875.00
tca	S	39297	10567.00
tcg	S	15	1548.00
cct	P	0	0.33
ccc	P	0	0.19
cca	P	1	0.34
ccg	P	0	0.14
agt	S	0	6871.00
agc	S	0	8111.00
gct	A	0	1.36
gcc	A	0	1.25
gca	A	5	2.02
gcg	A	0	0.37
---	--	------	------
mPD		0.0013	1.6
		nPD:	0.
		N. weight:	1.4
		Sc. PD:	-0.087
		Sc. rank:	-43830.0

,

PB1			
Pos.	403	obs:	exp:
tta	L	24	4563.00
ttg	L	39085	8130.00
ctt	L	1	8226.00
ctc	L	0	5766.00
cta	L	1	6038.00
ctg	L	211	6599.00
---	--	------	------
mPD		0.012	1.2
		nPD:	0.01
		N. weight:	1.7
		Sc. PD:	-0.086
		Sc. rank:	-21290.0

,

PB1			
Pos.	404	obs:	exp:
tct	S	0	6345.00
tcc	S	0	5876.00
tca	S	0	10568.00
tcg	S	0	1548.00
cgt	R	0	0.04
cgc	R	0	0.07
cga	R	0	0.18
cgg	R	0	0.17
agt	S	139	6871.00
agc	S	39181	8112.00
aga	R	1	0.98
agg	R	1	0.55
---	--	------	------
mPD		0.0071	1.6
		nPD:	0.
		N. weight:	1.7
		Sc. PD:	-0.097
		Sc. rank:	-40595.0

,

PB1			
Pos.	405	obs:	exp:
cct	P	34987	13104.00
ccc	P	4296	7372.00
cca	P	34	13285.00
ccg	P	5	5562.00
---	--	------	------
mPD		0.20	0.72
		nPD:	0.27
		N. weight:	0.89
		Sc. PD:	0.18
		Sc. rank:	24491.0

,

PB1			
Pos.	406	obs:	exp:
ggt	G	1	5294.00
ggc	G	1	5488.00
gga	G	38613	18152.00
ggg	G	707	10388.00
---	--	------	------
mPD		0.035	0.68
		nPD:	0.05
		N. weight:	0.76
		Sc. PD:	-0.0077
		Sc. rank:	11014.0

,

PB1			
Pos.	407	obs:	exp:
atg	M	39322	39322.00
---	--	------	------
mPD		0	0
		nPD:	1.
		N. weight:	0.
		Sc. PD:	0
		Sc. rank:	0

,

PB1			
Pos.	408	obs:	exp:
atg	M	39322	39322.00
---	--	------	------
mPD		0	0
		nPD:	1.
		N. weight:	0.
		Sc. PD:	0
		Sc. rank:	0

,

PB1			
Pos.	409	obs:	exp:
atg	M	39322	39322.00
---	--	------	------
mPD		0	0
		nPD:	1.
		N. weight:	0.
		Sc. PD:	0
		Sc. rank:	0

,

PB1			
Pos.	410	obs:	exp:
ggt	G	100	5294.00
ggc	G	39198	5488.00
gga	G	24	18152.00
ggg	G	0	10388.00
---	--	------	------
mPD		0.0063	0.68
		nPD:	0.01
		N. weight:	2.1
		Sc. PD:	-0.11
		Sc. rank:	-31049.0

,

PB1			
Pos.	411	obs:	exp:
atg	M	39321	39321.00
gtt	V	0	0.25
gtc	V	0	0.20
gta	V	0	0.22
gtg	V	1	0.33
---	--	------	------
mPD		0.000051	0.000085
		nPD:	0.6
		N. weight:	0.000031
		Sc. PD:	0.000016
		Sc. rank:	0.9

,

PB1			
Pos.	412	obs:	exp:
ttt	F	224	19127.00
ttc	F	39098	20195.00
---	--	------	------
mPD		0.011	0.50
		nPD:	0.02
		N. weight:	0.69
		Sc. PD:	-0.027
		Sc. rank:	188.3

,

PB1			
Pos.	413	obs:	exp:
aat	N	109	21999.00
aac	N	39213	17323.00
---	--	------	------
mPD		0.0055	0.49
		nPD:	0.01
		N. weight:	0.88
		Sc. PD:	-0.044
		Sc. rank:	-10508.0

,

PB1			
Pos.	414	obs:	exp:
atg	M	39321	39321.00
act	T	0	0.31
acc	T	0	0.18
aca	T	0	0.43
acg	T	1	0.08
---	--	------	------
mPD		0.000051	0.000098
		nPD:	0.52
		N. weight:	0.000071
		Sc. PD:	0.000032
		Sc. rank:	2.1

,

PB1			
Pos.	415	obs:	exp:
tta	L	1157	4563.00
ttg	L	3	8130.00
ctt	L	5	8226.00
ctc	L	116	5766.00
cta	L	37992	6038.00
ctg	L	49	6599.00
---	--	------	------
mPD		0.066	1.2
		nPD:	0.06
		N. weight:	1.9
		Sc. PD:	-0.01
		Sc. rank:	29748.0

,

PB1			
Pos.	416	obs:	exp:
tct	S	0	6345.00
tcc	S	0	5876.00
tca	S	0	10569.00
tcg	S	0	1548.00
aaa	K	0	0.66
aag	K	1	0.34
agt	S	38864	6871.00
agc	S	457	8112.00
---	--	------	------
mPD		0.023	1.6
		nPD:	0.01
		N. weight:	1.8
		Sc. PD:	-0.087
		Sc. rank:	-14822.0

,

PB1			
Pos.	417	obs:	exp:
tat	Y	0	0.53
tac	Y	1	0.47
act	T	41	12211.00
acc	T	30	6952.00
aca	T	37041	17017.00
acg	T	2209	3141.00
---	--	------	------
mPD		0.11	0.68
		nPD:	0.16
		N. weight:	0.77
		Sc. PD:	0.074
		Sc. rank:	19572.0

,

PB1			
Pos.	418	obs:	exp:
att	I	7	2.46
atc	I	0	1.84
ata	I	0	2.70
gtt	V	39194	9847.00
gtc	V	103	8011.00
gta	V	4	8521.00
gtg	V	13	12935.00
ggt	G	1	0.13
ggc	G	0	0.14
gga	G	0	0.46
ggg	G	0	0.26
---	--	------	------
mPD		0.0065	0.74
		nPD:	0.01
		N. weight:	1.5
		Sc. PD:	-0.078
		Sc. rank:	-22679.0

,

PB1

Pos.	419	obs:	exp:
tta	L	38782	4563.00
ttg	L	495	8130.00
ctt	L	0	8226.00
ctc	L	0	5766.00
cta	L	45	6038.00
ctg	L	0	6599.00
---	--	------	------
mPD		0.027	1.2
		nPD:	0.02
		N. weight:	2.3
		Sc. PD:	-0.087
		Sc. rank:	2306.2

PB1

Pos.	420	obs:	exp:
ggt	G	25	5294.00
ggc	G	6	5488.00
gga	G	38500	18152.00
ggg	G	791	10388.00
---	--	------	------
mPD		0.041	0.68
		nPD:	0.06
		N. weight:	0.75
		Sc. PD:	-0.0016
		Sc. rank:	12620.0

PB1

Pos.	421	obs:	exp:
gtt	V	69	9848.00
gtc	V	39204	8011.00
gta	V	45	8522.00
gtg	V	0	12937.00
gct	A	0	1.09
gcc	A	4	1.00
gca	A	0	1.62
gcg	A	0	0.29
---	--	------	------
mPD		0.0060	0.74
		nPD:	0.01
		N. weight:	1.7
		Sc. PD:	-0.091
		Sc. rank:	-28458.0

PB1

Pos.	422	obs:	exp:
tct	S	40	6345.00
tcc	S	3	5876.00
tca	S	1497	10569.00
tcg	S	37782	1548.00
agt	S	0	6872.00
agc	S	0	8112.00
---	--	------	------
mPD		0.075	1.6
		nPD:	0.05
		N. weight:	3.3
		Sc. PD:	-0.052
		Sc. rank:	40464.0

PB1

Pos.	423	obs:	exp:
att	I	1	13789.00
atc	I	76	10321.00
ata	I	39202	15169.00
atg	M	3	3.00
gtt	V	0	10.02
gtc	V	1	8.15
gta	V	39	8.67
gtg	V	0	13.16
---	--	------	------
mPD		0.0061	0.66
		nPD:	0.01
		N. weight:	1.
		Sc. PD:	-0.053
		Sc. rank:	-14835.0

PB1

Pos.	424	obs:	exp:
tta	L	1	4563.00
ttg	L	206	8129.00
ctt	L	9	8225.00
ctc	L	0	5766.00
cta	L	169	6038.00
ctg	L	38935	6599.00
atg	M	2	2.00
---	--	------	------
mPD		0.020	1.2
		nPD:	0.02
		N. weight:	1.9
		Sc. PD:	-0.084
		Sc. rank:	-8885.4

PB1

Pos.	425	obs:	exp:
tct	S	0	0.81
tcc	S	0	0.75
tca	S	0	1.34
tcg	S	0	0.20
aat	N	39155	21997.00
aac	N	162	17320.00
agt	S	5	0.87
agc	S	0	1.03
---	--	------	------
mPD		0.0085	0.49
		nPD:	0.02
		N. weight:	0.61
		Sc. PD:	-0.027
		Sc. rank:	-2786.3

PB1

Pos.	426	obs:	exp:
tta	L	0	4563.00
ttg	L	0	8129.00
ctt	L	39149	8226.00
ctc	L	150	5766.00
cta	L	5	6038.00
ctg	L	17	6599.00
att	I	1	0.35
atc	I	0	0.26
ata	I	0	0.39
---	--	------	------
mPD		0.0088	1.2
		nPD:	0.01
		N. weight:	1.7
		Sc. PD:	-0.09
		Sc. rank:	-29968.0

PB1

Pos.	427	obs:	exp:
ggt	G	2	5294.00
ggc	G	3	5488.00
gga	G	38536	18152.00
ggg	G	781	10388.00
---	--	------	------
mPD		0.039	0.68
		nPD:	0.06
		N. weight:	0.75
		Sc. PD:	-0.0036
		Sc. rank:	12123.0

PB1

Pos.	428	obs:	exp:
tta	L	0	1.51
ttg	L	0	2.69
ctt	L	0	2.72
ctc	L	0	1.91
cta	L	13	2.00
ctg	L	0	2.18
cat	H	1	0.56
cac	H	0	0.44
caa	Q	39189	24018.00
cag	Q	103	15274.00
cgt	R	0	0.04
cgc	R	0	0.07
cga	R	2	0.18
cgg	R	0	0.17
aaa	K	13	9.20
aag	K	1	4.80
aga	R	0	0.98
agg	R	0	0.55
---	--	------	------
mPD		0.0068	0.48
		nPD:	0.01
		N. weight:	0.52
		Sc. PD:	-0.025
		Sc. rank:	-4177.4

PB1

Pos.	429	obs:	exp:
cgt	R	0	0.12
cgc	R	0	0.22
cga	R	0	0.55
cgg	R	0	0.52
aaa	K	8	25847.00
aag	K	39307	13468.00
aga	R	0	2.94
agg	R	6	1.66
gaa	E	0	0.59
gag	E	1	0.41
---	--	------	------
mPD		0.00076	0.45
		nPD:	0.
		N. weight:	1.2
		Sc. PD:	-0.07
		Sc. rank:	-34031.0

PB1

Pos.	430	obs:	exp:
cgt	R	0	1.31
cgc	R	0	2.37
cga	R	0	6.02
cgg	R	0	5.67
aat	N	0	1.68
aac	N	3	1.32
aaa	K	39213	25805.00
aag	K	38	13446.00
aga	R	66	32.37
agg	R	0	18.26
gaa	E	2	1.18
gag	E	0	0.82
---	--	------	------
mPD		0.0055	0.45
		nPD:	0.01
		N. weight:	0.45
		Sc. PD:	-0.022
		Sc. rank:	-4959.0

PB1

Pos.	431	obs:	exp:
tat	Y	294	20716.00
tac	Y	39007	18585.00
tgt	C	0	0.78
tgc	C	2	1.22
cat	H	0	10.72
cac	H	19	8.28
---	--	------	------
mPD		0.016	0.50
		nPD:	0.03
		N. weight:	0.77
		Sc. PD:	-0.023
		Sc. rank:	4963.8

PB1

Pos.	432	obs:	exp:
att	I	0	0.35
atc	I	1	0.26
ata	I	0	0.39
act	T	941	12211.00
acc	T	38356	6952.00
aca	T	24	17017.00
acg	T	0	3141.00
---	--	------	------
mPD		0.048	0.68
		nPD:	0.07
		N. weight:	1.8
		Sc. PD:	0.014
		Sc. rank:	31918.0

PB1

Pos.	433	obs:	exp:
cgt	R	0	98.07
cgc	R	0	178.00
cga	R	0	451.30
cgg	R	0	424.70
aaa	K	348	22600.00
aag	K	34028	11776.00
aga	R	5	2426.00
agg	R	4941	1368.00
---	--	------	------
mPD		0.24	0.74
		nPD:	0.32
		N. weight:	1.1
		Sc. PD:	0.29
		Sc. rank:	32524.0

PB1

Pos.	434	obs:	exp:
act	T	8	12212.00
acc	T	3	6952.00
aca	T	39257	17018.00
acg	T	54	3141.00
---	--	------	------
mPD		0.0033	0.68
		nPD:	0.
		N. weight:	0.91
		Sc. PD:	-0.051
		Sc. rank:	-20936.0

PB1			
Pos.	435	obs:	exp:
att	I	0	3.16
atc	I	0	2.36
ata	I	9	3.48
atg	M	9	9.00
act	T	14	12203.00
acc	T	2	6948.00
aca	T	23648	17006.00
acg	T	15632	3139.00
gtt	V	0	0.50
gtc	V	0	0.41
gta	V	2	0.43
gtg	V	0	0.66
gct	A	0	1.64
gcc	A	0	1.50
gca	A	6	2.43
gcg	A	0	0.44
---	--	------	------
mPD		0.48	0.68
		nPD:	0.71
		N. weight:	0.92
		Sc. PD:	0.58
		Sc. rank:	28049.0

,

PB1			
Pos.	436	obs:	exp:
tat	Y	199	20726.00
tac	Y	39121	18594.00
cat	H	0	1.13
cac	H	2	0.87
---	--	------	------
mPD		0.010	0.50
		nPD:	0.02
		N. weight:	0.79
		Sc. PD:	-0.032
		Sc. rank:	-1656.8

,

PB1			
Pos.	437	obs:	exp:
tta	L	0	0.23
ttg	L	2	0.41
tgg	W	39320	39320.00
ctt	L	0	0.42
ctc	L	0	0.29
cta	L	0	0.31
ctg	L	0	0.34
---	--	------	------
mPD		0.00010	0.00023
		nPD:	0.43
		N. weight:	0.000088
		Sc. PD:	0.000032
		Sc. rank:	2.6

,

PB1			
Pos.	438	obs:	exp:
tgg	W	39322	39322.00
---	--	------	------
mPD		0	0
		nPD:	1.
		N. weight:	0.
		Sc. PD:	0
		Sc. rank:	0

,

PB1			
Pos.	439	obs:	exp:
aat	N	1	0.56
aac	N	0	0.44
gat	D	39127	22227.00
gac	D	194	17094.00
---	--	------	------
mPD		0.0099	0.49
		nPD:	0.02
		N. weight:	0.59
		Sc. PD:	-0.025
		Sc. rank:	-1373.0

,

PB1			
Pos.	440	obs:	exp:
gaa	E	1	0.59
gag	E	0	0.41
ggt	G	19	5294.00
ggc	G	3	5488.00
gga	G	548	18151.00
ggg	G	38751	10388.00
---	--	------	------
mPD		0.029	0.68
		nPD:	0.04
		N. weight:	1.4
		Sc. PD:	-0.027
		Sc. rank:	14567.0

,

PB1			
Pos.	441	obs:	exp:
ttt	F	1	0.97
ttc	F	1	1.03
tta	L	0	4562.00
ttg	L	0	8129.00
ctt	L	88	8225.00
ctc	L	39125	5766.00
cta	L	104	6038.00
ctg	L	1	6599.00
att	I	0	0.70
atc	I	2	0.53
ata	I	0	0.77
---	--	------	------
mPD		0.010	1.2
		nPD:	0.01
		N. weight:	2.1
		Sc. PD:	-0.11
		Sc. rank:	-31892.0

,

PB1			
Pos.	442	obs:	exp:
caa	Q	39250	24037.00
cag	Q	72	15285.00
---	--	------	------
mPD		0.0037	0.48
		nPD:	0.01
		N. weight:	0.53
		Sc. PD:	-0.028
		Sc. rank:	-9162.2

,

PB1			
Pos.	443	obs:	exp:
tct	S	596	6345.00
tcc	S	38604	5876.00
tca	S	120	10569.00
tcg	S	2	1548.00
agt	S	0	6872.00
agc	S	0	8112.00
---	--	------	------
mPD		0.036	1.6
		nPD:	0.02
		N. weight:	2.
		Sc. PD:	-0.078
		Sc. rank:	-530.3

,

PB1			
Pos.	444	obs:	exp:
tct	S	189	6345.00
tcc	S	3009	5876.00
tca	S	36106	10569.00
tcg	S	18	1548.00
agt	S	0	6872.00
agc	S	0	8112.00
---	--	------	------
mPD		0.15	1.6
		nPD:	0.09
		N. weight:	1.2
		Sc. PD:	0.034
		Sc. rank:	24657.0

,

PB1			
Pos.	445	obs:	exp:
gat	D	589	22227.00
gac	D	38733	17095.00
---	--	------	------
mPD		0.030	0.49
		nPD:	0.06
		N. weight:	0.82
		Sc. PD:	-0.002
		Sc. rank:	13891.0

,

PB1			
Pos.	446	obs:	exp:
gat	D	38146	22227.00
gac	D	1176	17095.00
---	--	------	------
mPD		0.058	0.49
		nPD:	0.12
		N. weight:	0.49
		Sc. PD:	0.026
		Sc. rank:	11120.0

,

PB1			
Pos.	447	obs:	exp:
ttt	F	39247	19127.00
ttc	F	75	20195.00
---	--	------	------
mPD		0.0038	0.50
		nPD:	0.01
		N. weight:	0.78
		Sc. PD:	-0.042
		Sc. rank:	-13575.0

,

PB1			
Pos.	448	obs:	exp:
act	T	0	1.86
acc	T	5	1.06
aca	T	1	2.60
acg	T	0	0.48
gtt	V	0	0.50
gtc	V	2	0.41
gta	V	0	0.43
gtg	V	0	0.66
gct	A	1060	10715.00
gcc	A	38178	9822.00
gca	A	75	15902.00
gcg	A	0	2874.00
ggt	G	0	0.13
ggc	G	1	0.14
gga	G	0	0.46
ggg	G	0	0.26
---	--	------	------
mPD		0.057	0.69
		nPD:	0.08
		N. weight:	1.4
		Sc. PD:	0.025
		Sc. rank:	27900.0

,

PB1			
Pos.	449	obs:	exp:
tta	L	0	4563.00
ttg	L	0	8130.00
ctt	L	30	8226.00
ctc	L	39257	5766.00
cta	L	32	6038.00
ctg	L	3	6599.00
---	--	------	------
mPD		0.0033	1.2
		nPD:	0.
		N. weight:	2.1
		Sc. PD:	-0.12
		Sc. rank:	-55146.0

,

PB1			
Pos.	450	obs:	exp:
tta	L	0	0.12
ttg	L	0	0.21
ctt	L	0	0.21
ctc	L	0	0.15
cta	L	1	0.15
ctg	L	0	0.17
att	I	105	13803.00
atc	I	6	10332.00
ata	I	39210	15186.00
---	--	------	------
mPD		0.0057	0.66
		nPD:	0.01
		N. weight:	1.
		Sc. PD:	-0.054
		Sc. rank:	-16008.0

PB1			
Pos.	451	obs:	exp:
att	I	0	0.35
atc	I	0	0.26
ata	I	1	0.39
atg	M	9	9.00
gtt	V	202	9847.00
gtc	V	0	8010.00
gta	V	582	8520.00
gtg	V	38527	12934.00
ggt	G	0	0.13
ggc	G	0	0.14
gga	G	0	0.46
ggg	G	1	0.26
---	--	------	------
mPD		0.040	0.74
		nPD:	0.05
		N. weight:	1.1
		Sc. PD:	-0.0095
		Sc. rank:	16627.0

PB1			
Pos.	452	obs:	exp:
aat	N	39084	21999.00
aac	N	238	17323.00
---	--	------	------
mPD		0.012	0.49
		nPD:	0.02
		N. weight:	0.6
		Sc. PD:	-0.022
		Sc. rank:	914.6

PB1			
Pos.	453	obs:	exp:
tct	S	0	0.32
tcc	S	0	0.30
tca	S	2	0.54
tcg	S	0	0.08
agt	S	0	0.35
agc	S	0	0.41
gct	A	12	10717.00
gcc	A	9	9824.00
gca	A	39251	15905.00
gcg	A	48	2874.00
---	--	------	------
mPD		0.0036	0.69
		nPD:	0.01
		N. weight:	0.98
		Sc. PD:	-0.055
		Sc. rank:	-21495.0

PB1			
Pos.	454	obs:	exp:
tta	L	0	0.70
ttg	L	0	1.24
tct	S	0	7.26
tcc	S	0	6.72
tca	S	44	12.10
tcg	S	1	1.77
ctt	L	0	1.26
ctc	L	0	0.88
cta	L	5	0.92
ctg	L	1	1.01
cct	P	3	13084.00
ccc	P	21	7361.00
cca	P	36918	13265.00
ccg	P	2321	5553.00
caa	Q	5	3.06
cag	Q	0	1.94
act	T	0	0.93
acc	T	0	0.53
aca	T	3	1.30
acg	T	0	0.24
agt	S	0	7.86
agc	S	0	9.28
---	--	------	------
mPD		0.12	0.72
		nPD:	0.16
		N. weight:	1.
		Sc. PD:	0.094
		Sc. rank:	25048.0

PB1			
Pos.	455	obs:	exp:
tct	S	0	0.81
tcc	S	0	0.75
tca	S	0	1.34
tcg	S	0	0.20
act	T	4	1.24
acc	T	0	0.71
aca	T	0	1.73
acg	T	0	0.32
aat	N	36522	21979.00
aac	N	2763	17306.00
agt	S	5	0.87
agc	S	0	1.03
gat	D	28	15.83
gac	D	0	12.17
---	--	------	------
mPD		0.13	0.50
		nPD:	0.27
		N. weight:	0.38
		Sc. PD:	0.075
		Sc. rank:	10338.0

PB1			
Pos.	456	obs:	exp:
tat	Y	36	18.98
tac	Y	0	17.02
cct	P	1	0.33
ccc	P	0	0.19
cca	P	0	0.34
ccg	P	0	0.14
cat	H	38995	22148.00
cac	H	272	17119.00
caa	Q	1	0.61
cag	Q	0	0.39
cgt	R	8	0.16
cgc	R	0	0.29
cga	R	0	0.73
cgg	R	0	0.69
aat	N	9	5.04
aac	N	0	3.96
aga	R	0	3.92
agg	R	0	2.21
---	--	------	------
mPD		0.017	0.50
		nPD:	0.03
		N. weight:	0.59
		Sc. PD:	-0.017
		Sc. rank:	4204.1

PB1			
Pos.	457	obs:	exp:
cgt	R	0	0.02
cgc	R	0	0.04
cga	R	0	0.09
cgg	R	0	0.09
aat	N	1	0.56
aac	N	0	0.44
aaa	K	0	9.20
aag	K	14	4.80
aga	R	0	0.49
agg	R	1	0.28
gtt	V	0	1.75
gtc	V	0	1.43
gta	V	0	1.52
gtg	V	7	2.30
gct	A	0	0.55
gcc	A	0	0.50
gca	A	0	0.81
gcg	A	2	0.15
gat	D	197	111.40
gac	D	0	85.64
gaa	E	296	23027.00
gag	E	38761	16030.00
ggt	G	0	5.79
ggc	G	0	6.00
gga	G	7	19.85
ggg	G	36	11.36
---	--	------	------
mPD		0.029	0.49
		nPD:	0.06
		N. weight:	0.92
		Sc. PD:	-0.0038
		Sc. rank:	15153.0

PB1			
Pos.	458	obs:	exp:
gct	A	0	0.27
gcc	A	0	0.25
gca	A	1	0.40
gcg	A	0	0.07
gaa	E	10	5.90
gag	E	0	4.10
ggt	G	6	5292.00
ggc	G	0	5487.00
gga	G	38630	18147.00
ggg	G	675	10386.00
---	--	------	------
mPD		0.035	0.68
		nPD:	0.05
		N. weight:	0.76
		Sc. PD:	-0.0087
		Sc. rank:	10615.0

PB1			
Pos.	459	obs:	exp:
att	I	3	13797.00
atc	I	167	10327.00
ata	I	39132	15178.00
atg	M	1	1.00
gtt	V	0	4.26
gtc	V	0	3.46
gta	V	17	3.68
gtg	V	0	5.59
gct	A	0	0.55
gcc	A	0	0.50
gca	A	2	0.81
gcg	A	0	0.15
---	--	------	------
mPD		0.0097	0.66
		nPD:	0.01
		N. weight:	1.
		Sc. PD:	-0.047
		Sc. rank:	-7711.5

PB1			
Pos.	460	obs:	exp:
caa	Q	38974	24034.00
cag	Q	343	15283.00
cgt	R	0	0.04
cgc	R	0	0.07
cga	R	2	0.18
cgg	R	0	0.17
aaa	K	2	1.31
aag	K	0	0.69
aga	R	0	0.98
agg	R	0	0.55
gaa	E	1	0.59
gag	E	0	0.41
---	--	------	------
mPD		0.018	0.48
		nPD:	0.04
		N. weight:	0.49
		Sc. PD:	-0.012
		Sc. rank:	4347.3

PB1			
Pos.	461	obs:	exp:
tct	S	0	0.48
tcc	S	0	0.45
tca	S	3	0.81
tcg	S	0	0.12
agt	S	0	0.52
agc	S	0	0.62
gct	A	15	10717.00
gcc	A	6	9823.00
gca	A	39269	15905.00
gcg	A	29	2874.00
---	--	------	------
mPD		0.0027	0.69
		nPD:	0.
		N. weight:	0.98
		Sc. PD:	-0.056
		Sc. rank:	-24641.0

PB1			
Pos.	462	obs:	exp:
ggt	G	3	5294.00
ggc	G	0	5488.00
gga	G	39056	18152.00
ggg	G	263	10388.00
---	--	------	------
mPD		0.013	0.68
		nPD:	0.02
		N. weight:	0.81
		Sc. PD:	-0.034
		Sc. rank:	-2197.7

PB1			
Pos.	463	obs:	exp:
atg	M	2	2.00
gtt	V	14	9849.00
gtc	V	3	8012.00
gta	V	282	8522.00
gtg	V	39021	12937.00
---	--	------	------
mPD		0.015	0.74
		nPD:	0.02
		N. weight:	1.2
		Sc. PD:	-0.048
		Sc. rank:	-2358.0

PB1			
Pos.	464	obs:	exp:
tct	S	0	0.32
tcc	S	0	0.30
tca	S	0	0.54
tcg	S	0	0.08
aat	N	189	105.70
aac	N	0	83.26
agt	S	2	0.35
agc	S	0	0.41
gat	D	38771	22118.00
gac	D	358	17011.00
gaa	E	1	0.59
gag	E	0	0.41
ggt	G	1	0.13
ggc	G	0	0.14
gga	G	0	0.46
ggg	G	0	0.26
---	--	------	------
mPD		0.028	0.50
		nPD:	0.06
		N. weight:	0.57
		Sc. PD:	-0.0038
		Sc. rank:	8848.7

PB1			
Pos.	465	obs:	exp:
cgt	R	0	779.20
cgc	R	0	1414.00
cga	R	9	3586.00
cgg	R	1	3375.00
aaa	K	22	14.46
aag	K	0	7.54
aga	R	38724	19276.00
agg	R	566	10870.00
---	--	------	------
mPD		0.030	0.89
		nPD:	0.03
		N. weight:	0.71
		Sc. PD:	-0.02
		Sc. rank:	5365.1

PB1			
Pos.	466	obs:	exp:
ttt	F	97	19127.00
ttc	F	39224	20194.00
tat	Y	0	0.53
tac	Y	1	0.47
---	--	------	------
mPD		0.0050	0.50
		nPD:	0.01
		N. weight:	0.71
		Sc. PD:	-0.037
		Sc. rank:	-9507.0

PB1			
Pos.	467	obs:	exp:
tat	Y	34575	20727.00
tac	Y	4747	18595.00
---	--	------	------
mPD		0.21	0.50
		nPD:	0.43
		N. weight:	0.31
		Sc. PD:	0.11
		Sc. rank:	9175.5

PB1			
Pos.	468	obs:	exp:
cgt	R	0	779.60
cgc	R	0	1415.00
cga	R	3	3588.00
cgg	R	5	3376.00
aga	R	333	19286.00
agg	R	38979	10876.00
ggt	G	0	0.27
ggc	G	0	0.28
gga	G	0	0.92
ggg	G	2	0.53
---	--	------	------
mPD		0.017	0.89
		nPD:	0.02
		N. weight:	1.3
		Sc. PD:	-0.056
		Sc. rank:	-3761.0

PB1			
Pos.	469	obs:	exp:
att	I	0	49.15
atc	I	136	36.79
ata	I	4	54.07
act	T	640	12166.00
acc	T	38434	6926.00
aca	T	102	16954.00
acg	T	0	3129.00
gct	A	0	1.64
gcc	A	6	1.50
gca	A	0	2.43
gcg	A	0	0.44
---	--	------	------
mPD		0.045	0.69
		nPD:	0.07
		N. weight:	1.8
		Sc. PD:	0.0046
		Sc. rank:	30814.0

PB1			
Pos.	470	obs:	exp:
tgt	C	57	15367.00
tgc	C	39265	23955.00
---	--	------	------
mPD		0.0029	0.48
		nPD:	0.01
		N. weight:	0.53
		Sc. PD:	-0.029
		Sc. rank:	-11001.0

PB1			
Pos.	471	obs:	exp:
cgt	R	0	0.04
cgc	R	0	0.07
cga	R	0	0.18
cgg	R	0	0.17
aaa	K	1163	25850.00
aag	K	38157	13470.00
aga	R	0	0.98
agg	R	2	0.55
---	--	------	------
mPD		0.058	0.45
		nPD:	0.13
		N. weight:	1.
		Sc. PD:	0.064
		Sc. rank:	23775.0

PB1			
Pos.	472	obs:	exp:
tta	L	38718	4563.00
ttg	L	376	8130.00
ctt	L	0	8226.00
ctc	L	0	5766.00
cta	L	228	6038.00
ctg	L	0	6599.00
---	--	------	------
mPD		0.030	1.2
		nPD:	0.03
		N. weight:	2.3
		Sc. PD:	-0.08
		Sc. rank:	8789.8

PB1			
Pos.	473	obs:	exp:
tta	L	0	0.35
ttg	L	0	0.62
ctt	L	3	0.63
ctc	L	0	0.44
cta	L	0	0.46
ctg	L	0	0.50
atg	M	36	36.00
gtt	V	26	9840.00
gtc	V	64	8004.00
gta	V	626	8514.00
gtg	V	38567	12925.00
---	--	------	------
mPD		0.038	0.74
		nPD:	0.05
		N. weight:	1.1
		Sc. PD:	-0.012
		Sc. rank:	15678.0

PB1			
Pos.	474	obs:	exp:
ggt	G	38	5294.00
ggc	G	3	5488.00
gga	G	38732	18152.00
ggg	G	549	10388.00
---	--	------	------
mPD		0.030	0.68
		nPD:	0.04
		N. weight:	0.77
		Sc. PD:	-0.014
		Sc. rank:	8536.6

PB1			
Pos.	475	obs:	exp:
att	I	18	13802.00
atc	I	39157	10331.00
ata	I	143	15185.00
gtt	V	0	1.00
gtc	V	4	0.82
gta	V	0	0.87
gtg	V	0	1.32
---	--	------	------
mPD		0.0084	0.66
		nPD:	0.01
		N. weight:	1.4
		Sc. PD:	-0.07
		Sc. rank:	-14505.0

PB1			
Pos.	476	obs:	exp:
tct	S	0	0.16
tcc	S	0	0.15
tca	S	0	0.27
tcg	S	0	0.04
aat	N	160	21999.00
aac	N	39161	17322.00
agt	S	0	0.17
agc	S	1	0.21
---	--	------	------
mPD		0.0082	0.49
		nPD:	0.02
		N. weight:	0.87
		Sc. PD:	-0.039
		Sc. rank:	-4564.2

PB1			
Pos.	477	obs:	exp:
atg	M	39319	39319.00
gtt	V	0	0.75
gtc	V	0	0.61
gta	V	0	0.65
gtg	V	3	0.99
---	--	------	------
mPD		0.00015	0.00025
		nPD:	0.6
		N. weight:	0.000093
		Sc. PD:	0.000049
		Sc. rank:	2.8

PB1			
Pos.	478	obs:	exp:
tct	S	0	6345.00
tcc	S	0	5876.00
tca	S	0	10569.00
tcg	S	0	1548.00
aat	N	0	0.56
aac	N	1	0.44
agt	S	93	6871.00
agc	S	39228	8112.00
---	--	------	------
mPD		0.0048	1.6
		nPD:	0.
		N. weight:	1.7
		Sc. PD:	-0.1
		Sc. rank:	-44961.0

PB1			
Pos.	479	obs:	exp:
caa	Q	1	0.61
cag	Q	0	0.39
aaa	K	38305	25851.00
aag	K	1016	13470.00
---	--	------	------
mPD		0.050	0.45
		nPD:	0.11
		N. weight:	0.35
		Sc. PD:	0.017
		Sc. rank:	7840.3

PB1			
Pos.	480	obs:	exp:
cgt	R	0	2.87
cgc	R	0	5.22
cga	R	0	13.23
cgg	R	0	12.45
aaa	K	80	25756.00
aag	K	39097	13421.00
aga	R	1	71.12
agg	R	144	40.11
---	--	------	------
mPD		0.011	0.46
		nPD:	0.02
		N. weight:	1.2
		Sc. PD:	-0.042
		Sc. rank:	2054.0

PB1			
Pos.	481	obs:	exp:
aaa	K	266	25852.00
aag	K	39056	13470.00
---	--	------	------
mPD		0.013	0.45
		nPD:	0.03
		N. weight:	1.1
		Sc. PD:	-0.036
		Sc. rank:	6305.1

PB1			
Pos.	482	obs:	exp:
tct	S	89	6345.00
tcc	S	39163	5876.00
tca	S	70	10569.00
tcg	S	0	1548.00
agt	S	0	6872.00
agc	S	0	8112.00
---	--	------	------
mPD		0.0081	1.6
		nPD:	0.
		N. weight:	2.1
		Sc. PD:	-0.12
		Sc. rank:	-46760.0

PB1			
Pos.	483	obs:	exp:
tat	Y	39200	20727.00
tac	Y	121	18594.00
cat	H	1	0.56
cac	H	0	0.44
---	--	------	------
mPD		0.0062	0.50
		nPD:	0.01
		N. weight:	0.68
		Sc. PD:	-0.033
		Sc. rank:	-7293.7

,

PB1			
Pos.	484	obs:	exp:
tta	L	1	0.12
ttg	L	0	0.21
ctt	L	0	0.21
ctc	L	0	0.15
cta	L	0	0.15
ctg	L	0	0.17
att	I	6	13799.00
atc	I	1	10329.00
ata	I	39302	15181.00
atg	M	6	6.00
gtt	V	0	1.50
gtc	V	0	1.22
gta	V	6	1.30
gtg	V	0	1.97
---	--	------	------
mPD		0.0010	0.66
		nPD:	0.
		N. weight:	1.
		Sc. PD:	-0.062
		Sc. rank:	-30717.0

,

PB1			
Pos.	485	obs:	exp:
aat	N	38135	21999.00
aac	N	1187	17323.00
---	--	------	------
mPD		0.059	0.49
		nPD:	0.12
		N. weight:	0.5
		Sc. PD:	0.027
		Sc. rank:	11357.0

,

PB1			
Pos.	486	obs:	exp:
cgt	R	0	2.36
cgc	R	0	4.28
cga	R	0	10.86
cgg	R	0	10.22
aat	N	1	0.56
aac	N	0	0.44
aaa	K	36223	25773.00
aag	K	2979	13429.00
aga	R	41	58.37
agg	R	78	32.92
---	--	------	------
mPD		0.15	0.46
		nPD:	0.33
		N. weight:	0.22
		Sc. PD:	0.057
		Sc. rank:	6278.0

,

PB1			
Pos.	487	obs:	exp:
act	T	155	12211.00
acc	T	1	6952.00
aca	T	38847	17017.00
acg	T	318	3141.00
gct	A	0	0.27
gcc	A	0	0.25
gca	A	1	0.40
gcg	A	0	0.07
---	--	------	------
mPD		0.024	0.68
		nPD:	0.04
		N. weight:	0.86
		Sc. PD:	-0.023
		Sc. rank:	7021.9

,

PB1			
Pos.	488	obs:	exp:
ggt	G	14	5294.00
ggc	G	0	5488.00
gga	G	588	18152.00
ggg	G	38720	10388.00
---	--	------	------
mPD		0.030	0.68
		nPD:	0.04
		N. weight:	1.4
		Sc. PD:	-0.024
		Sc. rank:	15821.0

,

PB1			
Pos.	489	obs:	exp:
act	T	25	12212.00
acc	T	2	6952.00
aca	T	39270	17018.00
acg	T	25	3141.00
---	--	------	------
mPD		0.0026	0.68
		nPD:	0.
		N. weight:	0.91
		Sc. PD:	-0.052
		Sc. rank:	-22704.0

,

PB1			
Pos.	490	obs:	exp:
ttt	F	39291	19126.00
ttc	F	29	20194.00
tta	L	1	0.12
ttg	L	0	0.21
tct	S	1	0.16
tcc	S	0	0.15
tca	S	0	0.27
tcg	S	0	0.04
ctt	L	0	0.21
ctc	L	0	0.15
cta	L	0	0.15
ctg	L	0	0.17
agt	S	0	0.17
agc	S	0	0.21
---	--	------	------
mPD		0.0016	0.50
		nPD:	0.
		N. weight:	0.78
		Sc. PD:	-0.045
		Sc. rank:	-20318.0

,

PB1			
Pos.	491	obs:	exp:
gaa	E	39243	23183.00
gag	E	79	16139.00
---	--	------	------
mPD		0.0040	0.48
		nPD:	0.01
		N. weight:	0.56
		Sc. PD:	-0.03
		Sc. rank:	-9066.3

,

PB1			
Pos.	492	obs:	exp:
ttt	F	120	19127.00
ttc	F	39202	20195.00
---	--	------	------
mPD		0.0061	0.50
		nPD:	0.01
		N. weight:	0.71
		Sc. PD:	-0.035
		Sc. rank:	-7715.2

,

PB1			
Pos.	493	obs:	exp:
act	T	36907	12211.00
acc	T	1516	6952.00
aca	T	889	17016.00
acg	T	7	3140.00
gct	A	3	0.82
gcc	A	0	0.75
gca	A	0	1.21
gcg	A	0	0.22
---	--	------	------
mPD		0.12	0.68
		nPD:	0.17
		N. weight:	1.
		Sc. PD:	0.11
		Sc. rank:	25639.0

,

PB1			
Pos.	494	obs:	exp:
tct	S	0	6345.00
tcc	S	0	5876.00
tca	S	0	10568.00
tcg	S	0	1548.00
att	I	0	0.35
atc	I	1	0.26
ata	I	0	0.39
aat	N	0	0.56
aac	N	1	0.44
agt	S	30	6871.00
agc	S	39289	8111.00
ggt	G	0	0.13
ggc	G	1	0.14
gga	G	0	0.46
ggg	G	0	0.26
---	--	------	------
mPD		0.0017	1.6
		nPD:	0.
		N. weight:	1.7
		Sc. PD:	-0.1
		Sc. rank:	-51888.0

,

PB1			
Pos.	495	obs:	exp:
ttt	F	22726	19127.00
ttc	F	16596	20195.00
---	--	------	------
mPD		0.49	0.50
		nPD:	0.98
		N. weight:	0.019
		Sc. PD:	0.017
		Sc. rank:	575.7

,

PB1			
Pos.	496	obs:	exp:
ttt	F	39139	19127.00
ttc	F	182	20194.00
tat	Y	1	0.53
tac	Y	0	0.47
---	--	------	------
mPD		0.0093	0.50
		nPD:	0.02
		N. weight:	0.76
		Sc. PD:	-0.033
		Sc. rank:	-2760.8

,

PB1			
Pos.	497	obs:	exp:
tat	Y	39170	20727.00
tac	Y	152	18595.00
---	--	------	------
mPD		0.0077	0.50
		nPD:	0.02
		N. weight:	0.68
		Sc. PD:	-0.031
		Sc. rank:	-4500.7

,

PB1			
Pos.	498	obs:	exp:
cgt	R	6	779.70
cgc	R	665	1415.00
cga	R	37881	3588.00
cgg	R	742	3376.00
aga	R	26	19287.00
agg	R	2	10876.00
---	--	------	------
mPD		0.071	0.89
		nPD:	0.08
		N. weight:	2.4
		Sc. PD:	0.043
		Sc. rank:	49524.0

,

PB1			
Pos.	499	obs:	exp:
tat	Y	39288	20726.00
tac	Y	32	18594.00
cat	H	2	1.13
cac	H	0	0.87
---	--	------	------
mPD		0.0017	0.50
		nPD:	0.
		N. weight:	0.7
		Sc. PD:	-0.04
		Sc. rank:	-17718.0

,

PB1			
Pos.	500	obs:	exp:
ggt	G	1	5294.00
ggc	G	0	5488.00
gga	G	39029	18152.00
ggg	G	292	10388.00
---	--	------	------
mPD		0.015	0.68
		nPD:	0.02
		N. weight:	0.8
		Sc. PD:	-0.032
		Sc. rank:	-513.1

,

PB1			
Pos.	501	obs:	exp:
ttt	F	39306	19127.00
ttc	F	15	20194.00
tta	L	1	0.12
ttg	L	0	0.21
ctt	L	0	0.21
ctc	L	0	0.15
cta	L	0	0.15
ctg	L	0	0.17
---	--	------	------
mPD		0.00081	0.50
		nPD:	0.
		N. weight:	0.79
		Sc. PD:	-0.047
		Sc. rank:	-22905.0

,

PB1			
Pos.	502	obs:	exp:
tta	L	1	0.23
ttg	L	1	0.41
ctt	L	0	0.42
ctc	L	0	0.29
cta	L	0	0.31
ctg	L	0	0.34
atg	M	8	8.00
gtt	V	59	9846.00
gtc	V	2	8010.00
gta	V	331	8520.00
gtg	V	38918	12934.00
gct	A	0	0.55
gcc	A	0	0.50
gca	A	0	0.81
gcg	A	2	0.15
---	--	------	------
mPD		0.020	0.74
		nPD:	0.03
		N. weight:	1.2
		Sc. PD:	-0.039
		Sc. rank:	5388.2

,

PB1			
Pos.	503	obs:	exp:
tct	S	3	0.48
tcc	S	0	0.45
tca	S	0	0.81
tcg	S	0	0.12
agt	S	0	0.52
agc	S	0	0.62
gtt	V	1	0.25
gtc	V	0	0.20
gta	V	0	0.22
gtg	V	0	0.33
gct	A	39059	10716.00
gcc	A	234	9823.00
gca	A	25	15904.00
gcg	A	0	2874.00
---	--	------	------
mPD		0.013	0.69
		nPD:	0.02
		N. weight:	1.4
		Sc. PD:	-0.058
		Sc. rank:	-4184.3

,

PB1			
Pos.	504	obs:	exp:
aat	N	38948	21999.00
aac	N	374	17323.00
---	--	------	------
mPD		0.019	0.49
		nPD:	0.04
		N. weight:	0.58
		Sc. PD:	-0.014
		Sc. rank:	5434.2

,

PB1			
Pos.	505	obs:	exp:
ttt	F	39237	19127.00
ttc	F	84	20194.00
att	I	1	0.35
atc	I	0	0.26
ata	I	0	0.39
---	--	------	------
mPD		0.0043	0.50
		nPD:	0.01
		N. weight:	0.77
		Sc. PD:	-0.041
		Sc. rank:	-12068.0

,

PB1			
Pos.	506	obs:	exp:
tct	S	0	6345.00
tcc	S	0	5876.00
tca	S	0	10569.00
tcg	S	0	1548.00
agt	S	86	6871.00
agc	S	39235	8112.00
ggt	G	0	0.13
ggc	G	1	0.14
gga	G	0	0.46
ggg	G	0	0.26
---	--	------	------
mPD		0.0044	1.6
		nPD:	0.
		N. weight:	1.7
		Sc. PD:	-0.1
		Sc. rank:	-46317.0

,

PB1			
Pos.	507	obs:	exp:
att	I	0	8.07
atc	I	0	6.04
ata	I	23	8.88
atg	M	39280	39280.00
act	T	0	2.79
acc	T	0	1.59
aca	T	0	3.89
acg	T	9	0.72
gtt	V	0	2.50
gtc	V	0	2.04
gta	V	0	2.17
gtg	V	10	3.29
---	--	------	------
mPD		0.0021	0.0029
		nPD:	0.74
		N. weight:	0.0016
		Sc. PD:	0.001
		Sc. rank:	47.9

,

PB1			
Pos.	508	obs:	exp:
gat	D	6	3.39
gac	D	0	2.61
gaa	E	197	23179.00
gag	E	39119	16137.00
---	--	------	------
mPD		0.010	0.48
		nPD:	0.02
		N. weight:	0.94
		Sc. PD:	-0.038
		Sc. rank:	-1186.8

,

PB1			
Pos.	509	obs:	exp:
tta	L	0	4563.00
ttg	L	74	8130.00
ctt	L	198	8226.00
ctc	L	0	5766.00
cta	L	307	6038.00
ctg	L	38743	6599.00
---	--	------	------
mPD		0.029	1.2
		nPD:	0.03
		N. weight:	1.9
		Sc. PD:	-0.068
		Sc. rank:	4373.8

,

PB1			
Pos.	510	obs:	exp:
tct	S	0	0.32
tcc	S	0	0.30
tca	S	2	0.54
tcg	S	0	0.08
cct	P	9	13103.00
ccc	P	2780	7371.00
cca	P	36058	13284.00
ccg	P	473	5561.00
agt	S	0	0.35
agc	S	0	0.41
---	--	------	------
mPD		0.15	0.72
		nPD:	0.21
		N. weight:	0.89
		Sc. PD:	0.13
		Sc. rank:	23736.0

,

PB1			
Pos.	511	obs:	exp:
tct	S	0	6342.00
tcc	S	0	5873.00
tca	S	0	10563.00
tcg	S	0	1547.00
aat	N	17	9.51
aac	N	0	7.49
agt	S	39189	6868.00
agc	S	112	8108.00
ggt	G	4	0.54
ggc	G	0	0.56
gga	G	0	1.85
ggg	G	0	1.06
---	--	------	------
mPD		0.0067	1.6
		nPD:	0.
		N. weight:	1.9
		Sc. PD:	-0.11
		Sc. rank:	-46126.0

,

PB1			
Pos.	512	obs:	exp:
ttt	F	36662	19126.00
ttc	F	2657	20193.00
tta	L	0	0.35
ttg	L	2	0.62
ctt	L	1	0.63
ctc	L	0	0.44
cta	L	0	0.46
ctg	L	0	0.50
---	--	------	------
mPD		0.13	0.50
		nPD:	0.25
		N. weight:	0.52
		Sc. PD:	0.095
		Sc. rank:	14120.0

,

PB1			
Pos.	513	obs:	exp:
cgt	R	0	0.02
cgc	R	0	0.04
cga	R	0	0.09
cgg	R	0	0.09
aga	R	1	0.49
agg	R	0	0.28
gaa	E	3	1.77
gag	E	0	1.23
ggt	G	12	5293.00
ggc	G	8	5488.00
gga	G	39051	18150.00
ggg	G	247	10387.00
---	--	------	------
mPD		0.014	0.68
		nPD:	0.02
		N. weight:	0.81
		Sc. PD:	-0.033
		Sc. rank:	-1749.7

,

PB1			
Pos.	514	obs:	exp:
gtt	V	72	9849.00
gtc	V	1	8012.00
gta	V	303	8523.00
gtg	V	38946	12938.00
---	--	------	------
mPD		0.019	0.74
		nPD:	0.03
		N. weight:	1.2
		Sc. PD:	-0.042
		Sc. rank:	3550.5

,

PB1

Pos.	515	obs:	exp:
tct	S	39079	6341.00
tcc	S	22	5872.00
tca	S	65	10561.00
tcg	S	128	1547.00
cct	P	26	8.66
ccc	P	0	4.87
cca	P	0	8.78
ccg	P	0	3.68
agt	S	0	6867.00
agc	S	0	8106.00
gct	A	2	0.55
gcc	A	0	0.50
gca	A	0	0.81
gcg	A	0	0.15
---	--	------	------
mPD		0.012	1.6
		nPD:	0.01
		N. weight:	2.
		Sc. PD:	-0.11
		Sc. rank:	-35166.0

,

PB1

Pos.	516	obs:	exp:
cgt	R	0	0.02
cgc	R	0	0.04
cga	R	0	0.09
cgg	R	0	0.09
aga	R	1	0.49
agg	R	0	0.28
ggt	G	10	5294.00
ggc	G	1	5488.00
gga	G	39133	18151.00
ggg	G	177	10388.00
---	--	------	------
mPD		0.0096	0.68
		nPD:	0.01
		N. weight:	0.82
		Sc. PD:	-0.039
		Sc. rank:	-6749.1

,

PB1

Pos.	517	obs:	exp:
tta	L	1	0.35
ttg	L	0	0.62
ctt	L	0	0.63
ctc	L	0	0.44
cta	L	2	0.46
ctg	L	0	0.50
att	I	194	13656.00
atc	I	6	10221.00
ata	I	38700	15023.00
atg	M	2	2.00
act	T	0	3.73
acc	T	0	2.12
aca	T	12	5.19
acg	T	0	0.96
gtt	V	4	101.40
gtc	V	0	82.52
gta	V	401	87.78
gtg	V	0	133.30
---	--	------	------
mPD		0.032	0.68
		nPD:	0.05
		N. weight:	1.
		Sc. PD:	-0.016
		Sc. rank:	12601.0

,

PB1

Pos.	518	obs:	exp:
tct	S	0	0.65
tcc	S	0	0.60
tca	S	0	1.08
tcg	S	0	0.16
aat	N	3051	21997.00
aac	N	36266	17320.00
agt	S	0	0.70
agc	S	4	0.83
gat	D	0	0.57
gac	D	1	0.43
---	--	------	------
mPD		0.14	0.49
		nPD:	0.29
		N. weight:	0.58
		Sc. PD:	0.13
		Sc. rank:	16029.0

,

PB1

Pos.	519	obs:	exp:
gct	A	0	0.27
gcc	A	0	0.25
gca	A	0	0.40
gcg	A	1	0.07
gaa	E	978	23182.00
gag	E	38343	16139.00
---	--	------	------
mPD		0.049	0.48
		nPD:	0.1
		N. weight:	0.84
		Sc. PD:	0.031
		Sc. rank:	18246.0

,

PB1

Pos.	520	obs:	exp:
tct	S	19	6345.00
tcc	S	8	5876.00
tca	S	39234	10569.00
tcg	S	60	1548.00
act	T	0	0.31
acc	T	0	0.18
aca	T	1	0.43
acg	T	0	0.08
agt	S	0	6871.00
agc	S	0	8112.00
---	--	------	------
mPD		0.0045	1.6
		nPD:	0.
		N. weight:	1.4
		Sc. PD:	-0.083
		Sc. rank:	-38034.0

,

PB1

Pos.	521	obs:	exp:
tct	S	1	0.16
tcc	S	0	0.15
tca	S	0	0.27
tcg	S	0	0.04
act	T	1	0.31
acc	T	0	0.18
aca	T	0	0.43
acg	T	0	0.08
agt	S	0	0.17
agc	S	0	0.21
gtt	V	0	0.25
gtc	V	0	0.20
gta	V	0	0.22
gtg	V	1	0.33
gct	A	39233	10717.00
gcc	A	70	9823.00
gca	A	6	15905.00
gcg	A	10	2874.00
---	--	------	------
mPD		0.0046	0.69
		nPD:	0.01
		N. weight:	1.4
		Sc. PD:	-0.077
		Sc. rank:	-27903.0

,

PB1

Pos.	522	obs:	exp:
gat	D	2204	22227.00
gac	D	37118	17095.00
---	--	------	------
mPD		0.11	0.49
		nPD:	0.22
		N. weight:	0.66
		Sc. PD:	0.098
		Sc. rank:	17584.0

,

PB1

Pos.	523	obs:	exp:
tta	L	0	0.23
ttg	L	1	0.41
ctt	L	0	0.42
ctc	L	0	0.29
cta	L	0	0.31
ctg	L	1	0.34
att	I	2	3.16
atc	I	0	2.36
ata	I	7	3.48
atg	M	39310	39310.00
gtt	V	0	0.25
gtc	V	0	0.20
gta	V	0	0.22
gtg	V	1	0.33
---	--	------	------
mPD		0.00061	0.00071
		nPD:	0.86
		N. weight:	0.00025
		Sc. PD:	0.00019
		Sc. rank:	7.7

,

PB1

Pos.	524	obs:	exp:
tct	S	0	6344.00
tcc	S	0	5875.00
tca	S	0	10567.00
tcg	S	0	1548.00
aat	N	0	1.68
aac	N	3	1.32
agt	S	283	6870.00
agc	S	39031	8110.00
ggt	G	0	0.67
ggc	G	5	0.70
gga	G	0	2.31
ggg	G	0	1.32
---	--	------	------
mPD		0.015	1.6
		nPD:	0.01
		N. weight:	1.7
		Sc. PD:	-0.088
		Sc. rank:	-25224.0

,

PB1

Pos.	525	obs:	exp:
att	I	39109	13800.00
atc	I	200	10330.00
ata	I	4	15183.00
gtt	V	9	2.25
gtc	V	0	1.83
gta	V	0	1.95
gtg	V	0	2.96
---	--	------	------
mPD		0.011	0.66
		nPD:	0.02
		N. weight:	1.1
		Sc. PD:	-0.05
		Sc. rank:	-6295.8

,

PB1

Pos.	526	obs:	exp:
ggt	G	6	5294.00
ggc	G	0	5488.00
gga	G	39072	18152.00
ggg	G	244	10388.00
---	--	------	------
mPD		0.013	0.68
		nPD:	0.02
		N. weight:	0.81
		Sc. PD:	-0.035
		Sc. rank:	-2893.6

,

PB1

Pos.	527	obs:	exp:
tta	L	0	0.12
ttg	L	0	0.21
ctt	L	0	0.21
ctc	L	0	0.15
cta	L	1	0.15
ctg	L	0	0.17
att	I	0	40.72
atc	I	0	30.48
ata	I	116	44.80
atg	M	2	2.00
act	T	0	0.31
acc	T	0	0.18
aca	T	1	0.43
acg	T	0	0.08
gtt	V	8	9814.00
gtc	V	2	7984.00
gta	V	38861	8492.00
gtg	V	311	12892.00
gct	A	0	5.18
gcc	A	0	4.75
gca	A	19	7.69
gcg	A	0	1.39
ggt	G	0	0.13
ggc	G	0	0.14
gga	G	1	0.46
ggg	G	0	0.26
---	--	------	------
mPD		0.023	0.75
		nPD:	0.03
		N. weight:	1.6
		Sc. PD:	-0.049
		Sc. rank:	10185.0

PB1

Pos.	528	obs:	exp:
tct	S	0	0.48
tcc	S	0	0.45
tca	S	1	0.81
tcg	S	0	0.12
cct	P	0	0.67
ccc	P	0	0.37
cca	P	2	0.68
ccg	P	0	0.28
att	I	0	0.35
atc	I	0	0.26
ata	I	1	0.39
act	T	8	12201.00
acc	T	6	6946.00
aca	T	39208	17003.00
acg	T	65	3138.00
agt	S	2	0.52
agc	S	0	0.62
gct	A	0	7.90
gcc	A	0	7.25
gca	A	29	11.73
gcg	A	0	2.12
---	--	------	------
mPD		0.0059	0.68
		nPD:	0.01
		N. weight:	0.91
		Sc. PD:	-0.048
		Sc. rank:	-13898.0

PB1

Pos.	529	obs:	exp:
tta	L	0	0.12
ttg	L	1	0.21
ctt	L	0	0.21
ctc	L	0	0.15
cta	L	0	0.15
ctg	L	0	0.17
atg	M	6	6.00
gtt	V	23	9847.00
gtc	V	3	8011.00
gta	V	323	8521.00
gtg	V	38965	12935.00
ggt	G	0	0.13
ggc	G	0	0.14
gga	G	0	0.46
ggg	G	1	0.26
---	--	------	------
mPD		0.018	0.74
		nPD:	0.02
		N. weight:	1.2
		Sc. PD:	-0.043
		Sc. rank:	1724.1

PB1

Pos.	530	obs:	exp:
att	I	5	13801.00
atc	I	4	10330.00
ata	I	39305	15183.00
aaa	K	0	0.66
aag	K	1	0.34
gtt	V	0	1.75
gtc	V	0	1.43
gta	V	7	1.52
gtg	V	0	2.30
---	--	------	------
mPD		0.00092	0.66
		nPD:	0.
		N. weight:	1.
		Sc. PD:	-0.062
		Sc. rank:	-30882.0

PB1

Pos.	531	obs:	exp:
cgt	R	0	0.59
cgc	R	0	1.08
cga	R	0	2.74
cgg	R	0	2.58
aaa	K	364	25831.00
aag	K	38927	13460.00
aga	R	3	14.71
agg	R	27	8.30
gaa	E	0	0.59
gag	E	1	0.41
---	--	------	------
mPD		0.020	0.45
		nPD:	0.04
		N. weight:	1.1
		Sc. PD:	-0.02
		Sc. rank:	12774.0

PB1

Pos.	532	obs:	exp:
tct	S	0	0.32
tcc	S	0	0.30
tca	S	0	0.54
tcg	S	0	0.08
cat	H	0	0.56
cac	H	1	0.44
act	T	0	1.24
acc	T	4	0.71
aca	T	0	1.73
acg	T	0	0.32
aat	N	78	21994.00
aac	N	39234	17318.00
aaa	K	1	0.66
aag	K	0	0.34
agt	S	0	0.35
agc	S	2	0.41
gat	D	0	1.13
gac	D	2	0.87
---	--	------	------
mPD		0.0045	0.49
		nPD:	0.01
		N. weight:	0.88
		Sc. PD:	-0.046
		Sc. rank:	-13150.0

PB1

Pos.	533	obs:	exp:
tct	S	0	0.97
tcc	S	0	0.90
tca	S	0	1.61
tcg	S	0	0.24
caa	Q	1	0.61
cag	Q	0	0.39
aat	N	138	21996.00
aac	N	39177	17319.00
agt	S	0	1.05
agc	S	6	1.24
---	--	------	------
mPD		0.0074	0.49
		nPD:	0.01
		N. weight:	0.87
		Sc. PD:	-0.04
		Sc. rank:	-6264.2

PB1

Pos.	534	obs:	exp:
cgt	R	0	0.02
cgc	R	0	0.04
cga	R	0	0.09
cgg	R	0	0.09
att	I	1	2.81
atc	I	0	2.10
ata	I	7	3.09
atg	M	39312	39312.00
act	T	0	0.31
acc	T	0	0.18
aca	T	0	0.43
acg	T	1	0.08
aga	R	0	0.49
agg	R	1	0.28
---	--	------	------
mPD		0.00051	0.00060
		nPD:	0.85
		N. weight:	0.00026
		Sc. PD:	0.0002
		Sc. rank:	8.1

PB1

Pos.	535	obs:	exp:
tta	L	1	0.12
ttg	L	0	0.21
ctt	L	0	0.21
ctc	L	0	0.15
cta	L	0	0.15
ctg	L	0	0.17
att	I	2	13776.00
atc	I	1	10312.00
ata	I	39241	15156.00
atg	M	1	1.00
act	T	0	1.24
acc	T	0	0.71
aca	T	4	1.73
acg	T	0	0.32
gtt	V	0	18.03
gtc	V	0	14.67
gta	V	71	15.61
gtg	V	1	23.69
---	--	------	------
mPD		0.0042	0.66
		nPD:	0.01
		N. weight:	1.
		Sc. PD:	-0.057
		Sc. rank:	-21265.0

PB1

Pos.	536	obs:	exp:
act	T	0	0.31
acc	T	1	0.18
aca	T	0	0.43
acg	T	0	0.08
aat	N	33744	21999.00
aac	N	5577	17322.00
---	--	------	------
mPD		0.24	0.49
		nPD:	0.49
		N. weight:	0.23
		Sc. PD:	0.095
		Sc. rank:	6727.4

PB1

Pos.	537	obs:	exp:
tct	S	0	0.81
tcc	S	0	0.75
tca	S	0	1.34
tcg	S	0	0.20
cat	H	1	0.56
cac	H	0	0.44
aat	N	38683	21996.00
aac	N	632	17319.00
aaa	K	0	0.66
aag	K	1	0.34
agt	S	5	0.87
agc	S	0	1.03
---	--	------	------
mPD		0.032	0.49
		nPD:	0.06
		N. weight:	0.55
		Sc. PD:	0.0012
		Sc. rank:	9488.4

PB1

Pos.	538	obs:	exp:
cgt	R	0	0.02
cgc	R	0	0.04
cga	R	0	0.09
cgg	R	0	0.09
aga	R	1	0.49
agg	R	0	0.28
gat	D	391	22227.00
gac	D	38930	17094.00
---	--	------	------
mPD		0.020	0.49
		nPD:	0.04
		N. weight:	0.85
		Sc. PD:	-0.018
		Sc. rank:	8654.7

PB1			
Pos.	539	obs:	exp:
ggt	G	0	0.13
ggc	G	1	0.14
gga	G	0	0.46
ggg	G	0	0.26
---	-	39321	39321.00
---	--	------	------
mPD		0	4.4×10^{-10}
		nPD:	0.
		N. weight:	0.
		Sc. PD:	0
		Sc. rank:	0

,

PB1			
Pos.	540	obs:	exp:
tat	Y	0	0.53
tac	Y	1	0.47
---	-	39321	39321.00
---	--	------	------
mPD		0	3.2×10^{-10}
		nPD:	0.
		N. weight:	0.
		Sc. PD:	0
		Sc. rank:	0

,

PB1			
Pos.	541	obs:	exp:
tta	L	0	4563.00
ttg	L	0	8130.00
ctt	L	39270	8226.00
ctc	L	28	5766.00
cta	L	22	6038.00
ctg	L	2	6599.00
---	--	------	------
mPD		0.0026	1.2
		nPD:	0.
		N. weight:	1.7
		Sc. PD:	-0.1
		Sc. rank:	-47734.0

,

PB1			
Pos.	542	obs:	exp:
att	I	0	0.35
atc	I	0	0.26
ata	I	1	0.39
ggt	G	6	5294.00
ggc	G	0	5488.00
gga	G	39127	18151.00
ggg	G	188	10388.00
---	--	------	------
mPD		0.0099	0.68
		nPD:	0.01
		N. weight:	0.82
		Sc. PD:	-0.038
		Sc. rank:	-6270.6

,

PB1			
Pos.	543	obs:	exp:
cct	P	218	13103.00
ccc	P	64	7372.00
cca	P	39013	13285.00
ccg	P	26	5561.00
cgt	R	0	0.02
cgc	R	0	0.04
cga	R	0	0.09
cgg	R	0	0.09
aga	R	1	0.49
agg	R	0	0.28
---	--	------	------
mPD		0.016	0.72
		nPD:	0.02
		N. weight:	1.1
		Sc. PD:	-0.045
		Sc. rank:	-619.3

,

PB1			
Pos.	544	obs:	exp:
gct	A	14	10717.00
gcc	A	2	9824.00
gca	A	38531	15906.00
gcg	A	775	2875.00
---	--	------	------
mPD		0.039	0.69
		nPD:	0.06
		N. weight:	0.92
		Sc. PD:	-0.0051
		Sc. rank:	14380.0

,

PB1			
Pos.	545	obs:	exp:
tta	L	0	0.12
ttg	L	1	0.21
ctt	L	0	0.21
ctc	L	0	0.15
cta	L	0	0.15
ctg	L	0	0.17
act	T	8	12211.00
acc	T	18	6952.00
aca	T	39244	17017.00
acg	T	51	3141.00
---	--	------	------
mPD		0.0041	0.68
		nPD:	0.01
		N. weight:	0.9
		Sc. PD:	-0.05
		Sc. rank:	-18984.0

,

PB1			
Pos.	546	obs:	exp:
tct	S	0	0.16
tcc	S	1	0.15
tca	S	0	0.27
tcg	S	0	0.04
act	T	0	0.31
acc	T	0	0.18
aca	T	1	0.43
acg	T	0	0.08
agt	S	0	0.17
agc	S	0	0.21
gct	A	290	10717.00
gcc	A	39027	9824.00
gca	A	3	15905.00
gcg	A	0	2874.00
---	--	------	------
mPD		0.015	0.69
		nPD:	0.02
		N. weight:	1.5
		Sc. PD:	-0.059
		Sc. rank:	-1373.6

,

PB1			
Pos.	547	obs:	exp:
caa	Q	38314	24037.00
cag	Q	1008	15285.00
---	--	------	------
mPD		0.050	0.48
		nPD:	0.11
		N. weight:	0.42
		Sc. PD:	0.018
		Sc. rank:	9313.6

,

PB1			
Pos.	548	obs:	exp:
tta	L	0	0.58
ttg	L	3	1.03
ctt	L	0	1.05
ctc	L	0	0.73
cta	L	0	0.77
ctg	L	2	0.84
atg	M	39315	39315.00
gtt	V	0	0.50
gtc	V	0	0.41
gta	V	0	0.43
gtg	V	2	0.66
---	--	------	------
mPD		0.00036	0.00058
		nPD:	0.61
		N. weight:	0.00023
		Sc. PD:	0.00012
		Sc. rank:	6.9

,

PB1			
Pos.	549	obs:	exp:
gct	A	36542	10717.00
gcc	A	2751	9824.00
gca	A	17	15906.00
gcg	A	12	2875.00
---	--	------	------
mPD		0.13	0.69
		nPD:	0.19
		N. weight:	1.1
		Sc. PD:	0.14
		Sc. rank:	29665.0

,

PB1			
Pos.	550	obs:	exp:
tta	L	0	4562.00
ttg	L	0	8127.00
ctt	L	806	8223.00
ctc	L	38495	5764.00
cta	L	9	6036.00
ctg	L	0	6597.00
att	I	0	4.21
atc	I	12	3.15
ata	I	0	4.63
---	--	------	------
mPD		0.041	1.2
		nPD:	0.04
		N. weight:	2.
		Sc. PD:	-0.052
		Sc. rank:	16944.0

,

PB1			
Pos.	551	obs:	exp:
cct	P	0	0.33
ccc	P	0	0.19
cca	P	1	0.34
ccg	P	0	0.14
caa	Q	39091	24036.00
cag	Q	230	15285.00
---	--	------	------
mPD		0.012	0.48
		nPD:	0.02
		N. weight:	0.5
		Sc. PD:	-0.019
		Sc. rank:	790.5

,

PB1			
Pos.	552	obs:	exp:
ttt	F	2	0.97
ttc	F	0	1.03
tta	L	443	4563.00
ttg	L	38748	8129.00
ctt	L	0	8225.00
ctc	L	0	5766.00
cta	L	0	6038.00
ctg	L	129	6599.00
---	--	------	------
mPD		0.029	1.2
		nPD:	0.02
		N. weight:	1.6
		Sc. PD:	-0.06
		Sc. rank:	3137.7

,

PB1			
Pos.	553	obs:	exp:
ttt	F	71	19124.00
ttc	F	39245	20192.00
tta	L	1	0.58
ttg	L	0	1.03
ctt	L	0	1.05
ctc	L	4	0.73
cta	L	0	0.77
ctg	L	0	0.84
aaa	K	0	0.66
aag	K	1	0.34
---	--	------	------
mPD		0.0040	0.50
		nPD:	0.01
		N. weight:	0.72
		Sc. PD:	-0.038
		Sc. rank:	-12007.0

,

PB1			
Pos.	554	obs:	exp:
tta	L	0	0.23
ttg	L	0	0.41
ctt	L	0	0.42
ctc	L	2	0.29
cta	L	0	0.31
ctg	L	0	0.34
att	I	167	13798.00
atc	I	39130	10328.00
ata	I	8	15179.00
act	T	0	0.62
acc	T	2	0.35
aca	T	0	0.87
acg	T	0	0.16
aat	N	0	0.56
aac	N	1	0.44
gtt	V	0	3.01
gtc	V	12	2.45
gta	V	0	2.60
gtg	V	0	3.95
---	--	------	------
mPD		0.0097	0.66
		nPD:	0.01
		N. weight:	1.4
		Sc. PD:	-0.067
		Sc. rank:	-10719.0

,

PB1			
Pos.	555	obs:	exp:
aaa	K	39168	25852.00
aag	K	154	13470.00
---	--	------	------
mPD		0.0078	0.45
		nPD:	0.02
		N. weight:	0.43
		Sc. PD:	-0.019
		Sc. rank:	-1866.5

PB1			
Pos.	556	obs:	exp:
aat	N	1	0.56
aac	N	0	0.44
gat	D	38419	22227.00
gac	D	902	17094.00
---	--	------	------
mPD		0.045	0.49
		nPD:	0.09
		N. weight:	0.51
		Sc. PD:	0.014
		Sc. rank:	10841.0

PB1			
Pos.	557	obs:	exp:
ttt	F	0	0.49
ttc	F	1	0.51
tat	Y	61	20726.00
tac	Y	39259	18594.00
tgt	C	0	0.39
tgc	C	1	0.61
---	--	------	------
mPD		0.0032	0.50
		nPD:	0.01
		N. weight:	0.81
		Sc. PD:	-0.044
		Sc. rank:	-16237.0

PB1			
Pos.	558	obs:	exp:
cgt	R	0	779.70
cgc	R	0	1415.00
cga	R	7	3588.00
cgg	R	0	3376.00
aga	R	39232	19287.00
agg	R	83	10876.00
---	--	------	------
mPD		0.0046	0.89
		nPD:	0.01
		N. weight:	0.76
		Sc. PD:	-0.043
		Sc. rank:	-16837.0

PB1			
Pos.	559	obs:	exp:
tat	Y	724	20727.00
tac	Y	38597	18594.00
cat	H	1	0.56
cac	H	0	0.44
---	--	------	------
mPD		0.036	0.50
		nPD:	0.07
		N. weight:	0.72
		Sc. PD:	0.0071
		Sc. rank:	13775.0

PB1			
Pos.	560	obs:	exp:
act	T	56	12212.00
acc	T	163	6952.00
aca	T	5940	17018.00
acg	T	33163	3141.00
---	--	------	------
mPD		0.27	0.68
		nPD:	0.39
		N. weight:	2.
		Sc. PD:	0.64
		Sc. rank:	57579.0

PB1			
Pos.	561	obs:	exp:
tct	S	1	0.16
tcc	S	0	0.15
tca	S	0	0.27
tcg	S	0	0.04
tat	Y	39112	20727.00
tac	Y	209	18594.00
agt	S	0	0.17
agc	S	0	0.21
---	--	------	------
mPD		0.011	0.50
		nPD:	0.02
		N. weight:	0.67
		Sc. PD:	-0.027
		Sc. rank:	-805.4

PB1			
Pos.	562	obs:	exp:
cgt	R	4	779.60
cgc	R	1	1415.00
cga	R	2096	3588.00
cgg	R	30130	3376.00
aga	R	327	19286.00
agg	R	6763	10876.00
ggt	G	0	0.13
ggc	G	0	0.14
gga	G	0	0.46
ggg	G	1	0.26
---	--	------	------
mPD		0.41	0.89
		nPD:	0.46
		N. weight:	1.7
		Sc. PD:	0.66
		Sc. rank:	49857.0

PB1			
Pos.	563	obs:	exp:
tgt	C	208	15367.00
tgc	C	39114	23955.00
---	--	------	------
mPD		0.011	0.48
		nPD:	0.02
		N. weight:	0.51
		Sc. PD:	-0.02
		Sc. rank:	-115.9

PB1			
Pos.	564	obs:	exp:
cat	H	38963	22179.00
cac	H	358	17142.00
caa	Q	1	0.61
cag	Q	0	0.39
---	--	------	------
mPD		0.018	0.49
		nPD:	0.04
		N. weight:	0.57
		Sc. PD:	-0.014
		Sc. rank:	5073.7

PB1			
Pos.	565	obs:	exp:
tct	S	0	0.32
tcc	S	0	0.30
tca	S	0	0.54
tcg	S	0	0.08
cgt	R	0	779.30
cgc	R	0	1414.00
cga	R	3	3586.00
cgg	R	0	3375.00
aaa	K	10	6.57
aag	K	0	3.43
agt	S	2	0.35
agc	S	0	0.41
aga	R	39217	19278.00
agg	R	85	10872.00
ggt	G	0	0.67
ggc	G	0	0.70
gga	G	5	2.31
ggg	G	0	1.32
---	--	------	------
mPD		0.0053	0.89
		nPD:	0.01
		N. weight:	0.77
		Sc. PD:	-0.042
		Sc. rank:	-15892.0

PB1			
Pos.	566	obs:	exp:
tct	S	0	0.16
tcc	S	0	0.15
tca	S	0	0.27
tcg	S	0	0.04
cgt	R	0	0.02
cgc	R	0	0.04
cga	R	0	0.09
cgg	R	0	0.09
agt	S	1	0.17
agc	S	0	0.21
aga	R	1	0.49
agg	R	0	0.28
ggt	G	17	5293.00
ggc	G	12	5488.00
gga	G	38937	18151.00
ggg	G	354	10388.00
---	--	------	------
mPD		0.019	0.68
		nPD:	0.03
		N. weight:	0.79
		Sc. PD:	-0.026
		Sc. rank:	4057.2

PB1			
Pos.	567	obs:	exp:
aat	N	0	1.68
aac	N	3	1.32
gat	D	1103	22225.00
gac	D	38215	17093.00
ggt	G	0	0.13
ggc	G	1	0.14
gga	G	0	0.46
ggg	G	0	0.26
---	--	------	------
mPD		0.055	0.49
		nPD:	0.11
		N. weight:	0.77
		Sc. PD:	0.036
		Sc. rank:	17272.0

PB1			
Pos.	568	obs:	exp:
tct	S	0	0.65
tcc	S	0	0.60
tca	S	4	1.08
tcg	S	0	0.16
att	I	0	1.40
atc	I	1	1.05
ata	I	3	1.54
act	T	1	12206.00
acc	T	8	6949.00
aca	T	38711	17010.00
acg	T	584	3139.00
aaa	K	6	3.94
aag	K	0	2.06
agt	S	0	0.70
agc	S	0	0.83
gct	A	0	1.09
gcc	A	0	1.00
gca	A	4	1.62
gcg	A	0	0.29
---	--	------	------
mPD		0.031	0.68
		nPD:	0.05
		N. weight:	0.86
		Sc. PD:	-0.015
		Sc. rank:	10174.0

PB1			
Pos.	569	obs:	exp:
cat	H	2	5.08
cac	H	7	3.92
caa	Q	39234	24027.00
cag	Q	72	15279.00
cgt	R	0	0.06
cgc	R	0	0.11
cga	R	3	0.27
cgg	R	0	0.26
aaa	K	2	1.31
aag	K	0	0.69
aga	R	0	1.47
agg	R	0	0.83
gaa	E	2	1.18
gag	E	0	0.82
---	--	------	------
mPD		0.0045	0.48
		nPD:	0.01
		N. weight:	0.53
		Sc. PD:	-0.027
		Sc. rank:	-7444.2

PB1			
Pos.	570	obs:	exp:
tta	L	0	0.12
ttg	L	0	0.21
ctt	L	0	0.21
ctc	L	1	0.15
cta	L	0	0.15
ctg	L	0	0.17
att	I	1152	13795.00
atc	I	38125	10326.00
ata	I	20	15176.00
act	T	0	0.31
acc	T	1	0.18
aca	T	0	0.43
acg	T	0	0.08
gtt	V	1	5.76
gtc	V	22	4.69
gta	V	0	4.99
gtg	V	0	7.57
---	--	------	------
mPD		0.059	0.66
		nPD:	0.09
		N. weight:	1.3
		Sc. PD:	0.035
		Sc. rank:	27584.0

PB1			
Pos.	571	obs:	exp:
tta	L	0	0.12
ttg	L	0	0.21
ctt	L	0	0.21
ctc	L	0	0.15
cta	L	0	0.15
ctg	L	1	0.17
cat	H	1	5.64
cac	H	9	4.36
caa	Q	37199	24024.00
cag	Q	2102	15277.00
cgt	R	0	0.12
cgc	R	0	0.22
cga	R	4	0.55
cgg	R	2	0.52
aaa	K	3	1.97
aag	K	0	1.03
aga	R	0	2.94
agg	R	0	1.66
gaa	E	0	0.59
gag	E	1	0.41
---	--	------	------
mPD		0.10	0.48
		nPD:	0.22
		N. weight:	0.34
		Sc. PD:	0.05
		Sc. rank:	8979.0

,

PB1			
Pos.	572	obs:	exp:
tct	S	0	0.32
tcc	S	0	0.30
tca	S	0	0.54
tcg	S	2	0.08
atg	M	1	1.00
act	T	15	12211.00
acc	T	6	6952.00
aca	T	6832	17016.00
acg	T	32466	3140.00
agt	S	0	0.35
agc	S	0	0.41
---	--	------	------
mPD		0.29	0.68
		nPD:	0.42
		N. weight:	1.9
		Sc. PD:	0.68
		Sc. rank:	56789.0

,

PB1			
Pos.	573	obs:	exp:
tct	S	0	0.16
tcc	S	0	0.15
tca	S	0	0.27
tcg	S	0	0.04
cgt	R	0	778.50
cgc	R	0	1413.00
cga	R	4	3582.00
cgg	R	0	3371.00
att	I	0	0.70
atc	I	0	0.53
ata	I	2	0.77
aaa	K	50	33.53
aag	K	1	17.47
agt	S	1	0.17
agc	S	0	0.21
aga	R	38911	19258.00
agg	R	348	10860.00
ggt	G	0	0.67
ggc	G	0	0.70
gga	G	5	2.31
ggg	G	0	1.32
---	--	------	------
mPD		0.021	0.89
		nPD:	0.02
		N. weight:	0.73
		Sc. PD:	-0.028
		Sc. rank:	606.6

,

PB1			
Pos.	574	obs:	exp:
cgt	R	0	779.70
cgc	R	0	1415.00
cga	R	12	3588.00
cgg	R	0	3376.00
aga	R	39237	19287.00
agg	R	73	10876.00
---	--	------	------
mPD		0.0043	0.89
		nPD:	0.
		N. weight:	0.77
		Sc. PD:	-0.043
		Sc. rank:	-17617.0

,

PB1			
Pos.	575	obs:	exp:
tct	S	11	6343.00
tcc	S	1	5874.00
tca	S	39228	10564.00
tcg	S	65	1547.00
act	T	0	5.28
acc	T	0	3.01
aca	T	17	7.36
acg	T	0	1.36
agt	S	0	6869.00
agc	S	0	8108.00
---	--	------	------
mPD		0.0048	1.6
		nPD:	0.
		N. weight:	1.4
		Sc. PD:	-0.083
		Sc. rank:	-37307.0

,

PB1			
Pos.	576	obs:	exp:
ttt	F	461	19106.00
ttc	F	38818	20173.00
tta	L	10	3.25
ttg	L	0	5.79
tct	S	0	1.13
tcc	S	7	1.05
tca	S	0	1.88
tcg	S	0	0.28
tat	Y	0	2.64
tac	Y	5	2.36
tgt	C	0	0.39
tgc	C	1	0.61
ctt	L	0	5.86
ctc	L	18	4.11
cta	L	0	4.30
ctg	L	0	4.70
att	I	0	0.35
atc	I	1	0.26
ata	I	0	0.39
agt	S	0	1.22
agc	S	0	1.44
gtt	V	0	0.25
gtc	V	1	0.20
gta	V	0	0.22
gtg	V	0	0.33
---	--	------	------
mPD		0.025	0.50
		nPD:	0.05
		N. weight:	0.66
		Sc. PD:	-0.0078
		Sc. rank:	9129.0

,

PB1			
Pos.	577	obs:	exp:
gat	D	2	1.13
gac	D	0	0.87
gaa	E	607	23182.00
gag	E	38713	16138.00
---	--	------	------
mPD		0.030	0.48
		nPD:	0.06
		N. weight:	0.88
		Sc. PD:	0.00041
		Sc. rank:	15166.0

,

PB1			
Pos.	578	obs:	exp:
tta	L	2159	380.40
ttg	L	15	677.70
ctt	L	1	685.70
ctc	L	0	480.70
cta	L	1100	503.40
ctg	L	3	550.10
att	I	28	12646.00
atc	I	4	9466.00
ata	I	35993	13913.00
atg	M	9	9.00
act	T	0	1.55
acc	T	0	0.88
aca	T	5	2.16
acg	T	0	0.40
gtt	V	0	1.25
gtc	V	0	1.02
gta	V	5	1.08
gtg	V	0	1.65
---	--	------	------
mPD		0.16	0.83
		nPD:	0.19
		N. weight:	1.1
		Sc. PD:	0.13
		Sc. rank:	27896.0

,

PB1			
Pos.	579	obs:	exp:
caa	Q	0	4.89
cag	Q	8	3.11
cgt	R	0	1.07
cgc	R	0	1.94
cga	R	0	4.93
cgg	R	0	4.64
atg	M	1	1.00
act	T	0	1.24
acc	T	0	0.71
aca	T	0	1.73
acg	T	4	0.32
aat	N	6	3.36
aac	N	0	2.64
aaa	K	343	25800.00
aag	K	38900	13443.00
aga	R	1	26.49
agg	R	53	14.94
gaa	E	0	3.54
gag	E	6	2.46
---	--	------	------
mPD		0.021	0.46
		nPD:	0.05
		N. weight:	1.1
		Sc. PD:	-0.017
		Sc. rank:	14118.0

,

PB1			
Pos.	580	obs:	exp:
tct	S	0	0.48
tcc	S	0	0.45
tca	S	0	0.81
tcg	S	0	0.12
cgt	R	0	2.91
cgc	R	0	5.29
cga	R	0	13.41
cgg	R	0	12.62
atg	M	1	1.00
act	T	0	1.24
acc	T	0	0.71
aca	T	0	1.73
acg	T	4	0.32
aat	N	5	2.80
aac	N	0	2.20
aaa	K	244	25739.00
aag	K	38906	13411.00
agt	S	0	0.52
agc	S	3	0.62
aga	R	1	72.10
agg	R	146	40.66
gaa	E	0	7.07
gag	E	12	4.93
---	--	------	------
mPD		0.021	0.46
		nPD:	0.05
		N. weight:	1.1
		Sc. PD:	-0.018
		Sc. rank:	13642.0

,

PB1			
Pos.	581	obs:	exp:
tta	L	0	4563.00
ttg	L	162	8129.00
ctt	L	5	8225.00
ctc	L	2	5766.00
cta	L	245	6038.00
ctg	L	38905	6599.00
atg	M	3	3.00
---	--	------	------
mPD		0.021	1.2
		nPD:	0.02
		N. weight:	1.9
		Sc. PD:	-0.082
		Sc. rank:	-7151.9

,

PB1			
Pos.	582	obs:	exp:
tgg	W	39322	39322.00
---	--	------	------
mPD		0	0
		nPD:	1.
		N. weight:	0.
		Sc. PD:	0
		Sc. rank:	0

,

PB1			
Pos.	583	obs:	exp:
tat	Y	1	0.53
tac	Y	0	0.47
aat	N	29	16.22
aac	N	0	12.78
gtt	V	1	0.25
gtc	V	0	0.20
gta	V	0	0.22
gtg	V	0	0.33
gat	D	38952	22141.00
gac	D	217	17028.00
gaa	E	20	63.67
gag	E	88	44.33
ggt	G	14	1.88
ggc	G	0	1.95
gga	G	0	6.46
ggg	G	0	3.70
---	--	------	------
mPD		0.019	0.50
		nPD:	0.04
		N. weight:	0.59
		Sc. PD:	-0.014
		Sc. rank:	5397.8

,

PB1			
Pos.	584	obs:	exp:
cat	H	2	1.69
cac	H	1	1.31
caa	Q	34194	24030.00
cag	Q	5117	15281.00
cgt	R	0	0.06
cgc	R	0	0.11
cga	R	2	0.27
cgg	R	1	0.26
aaa	K	5	3.29
aag	K	0	1.71
aga	R	0	1.47
agg	R	0	0.83
---	--	------	------
mPD		0.23	0.48
		nPD:	0.48
		N. weight:	0.18
		Sc. PD:	0.073
		Sc. rank:	5362.1

,

PB1			
Pos.	585	obs:	exp:
tct	S	0	0.16
tcc	S	0	0.15
tca	S	0	0.27
tcg	S	0	0.04
att	I	0	1.76
atc	I	5	1.31
ata	I	0	1.93
act	T	89	12209.00
acc	T	39216	6951.00
aca	T	7	17014.00
acg	T	1	3140.00
agt	S	0	0.17
agc	S	1	0.21
gct	A	0	0.82
gcc	A	3	0.75
gca	A	0	1.21
gcg	A	0	0.22
---	--	------	------
mPD		0.0054	0.68
		nPD:	0.01
		N. weight:	1.9
		Sc. PD:	-0.1
		Sc. rank:	-31824.0

,

PB1			
Pos.	586	obs:	exp:
tta	L	0	0.46
ttg	L	0	0.83
ctt	L	0	0.84
ctc	L	0	0.59
cta	L	4	0.61
ctg	L	0	0.67
cat	H	6	20.31
cac	H	30	15.69
caa	Q	39143	23962.00
cag	Q	57	15238.00
cgt	R	3	1.53
cgc	R	58	2.77
cga	R	16	7.03
cgg	R	0	6.61
aaa	K	5	3.29
aag	K	0	1.71
aga	R	0	37.77
agg	R	0	21.30
---	--	------	------
mPD		0.012	0.48
		nPD:	0.03
		N. weight:	0.53
		Sc. PD:	-0.019
		Sc. rank:	1174.8

,

PB1			
Pos.	587	obs:	exp:
tct	S	3	6345.00
tcc	S	3	5876.00
tca	S	39285	10569.00
tcg	S	30	1548.00
cct	P	0	0.33
ccc	P	0	0.19
cca	P	1	0.34
ccg	P	0	0.14
agt	S	0	6871.00
agc	S	0	8112.00
---	--	------	------
mPD		0.0019	1.6
		nPD:	0.
		N. weight:	1.4
		Sc. PD:	-0.086
		Sc. rank:	-42783.0

,

PB1			
Pos.	588	obs:	exp:
cgt	R	0	619.60
cgc	R	0	1124.00
cga	R	0	2851.00
cgg	R	2	2683.00
aaa	K	36	5306.00
aag	K	8034	2764.00
aga	R	592	15327.00
agg	R	30655	8643.00
gaa	E	0	1.18
gag	E	2	0.82
ggt	G	0	0.13
ggc	G	0	0.14
gga	G	0	0.46
ggg	G	1	0.26
---	--	------	------
mPD		0.36	1.1
		nPD:	0.31
		N. weight:	1.3
		Sc. PD:	0.31
		Sc. rank:	35396.0

,

PB1			
Pos.	589	obs:	exp:
tct	S	0	0.16
tcc	S	0	0.15
tca	S	1	0.27
tcg	S	0	0.04
cct	P	0	0.67
ccc	P	0	0.37
cca	P	2	0.68
ccg	P	0	0.28
att	I	0	9.48
atc	I	0	7.09
ata	I	27	10.43
act	T	51	11336.00
acc	T	1	6454.00
aca	T	36408	15798.00
acg	T	44	2916.00
agt	S	0	0.17
agc	S	0	0.21
gtt	V	0	10.02
gtc	V	0	8.15
gta	V	40	8.67
gtg	V	0	13.16
gct	A	4	749.00
gcc	A	2	686.60
gca	A	2738	1112.00
gcg	A	4	200.90
---	--	------	------
mPD		0.14	0.82
		nPD:	0.17
		N. weight:	0.91
		Sc. PD:	0.097
		Sc. rank:	23126.0

,

PB1			
Pos.	590	obs:	exp:
ggt	G	6	5294.00
ggc	G	1	5488.00
gga	G	38589	18152.00
ggg	G	726	10388.00
---	--	------	------
mPD		0.037	0.68
		nPD:	0.05
		N. weight:	0.76
		Sc. PD:	-0.0064
		Sc. rank:	11440.0

,

PB1			
Pos.	591	obs:	exp:
tta	L	9292	4563.00
ttg	L	28	8129.00
ctt	L	0	8226.00
ctc	L	1	5766.00
cta	L	29947	6038.00
ctg	L	53	6599.00
att	I	0	0.35
atc	I	0	0.26
ata	I	1	0.39
---	--	------	------
mPD		0.37	1.2
		nPD:	0.32
		N. weight:	1.5
		Sc. PD:	0.37
		Sc. rank:	42528.0

,

PB1			
Pos.	592	obs:	exp:
tta	L	501	4563.00
ttg	L	38550	8130.00
ctt	L	0	8226.00
ctc	L	0	5766.00
cta	L	159	6038.00
ctg	L	112	6599.00
---	--	------	------
mPD		0.047	1.2
		nPD:	0.04
		N. weight:	1.6
		Sc. PD:	-0.035
		Sc. rank:	16328.0

,

PB1			
Pos.	593	obs:	exp:
tta	L	1	0.23
ttg	L	1	0.41
ctt	L	0	0.42
ctc	L	0	0.29
cta	L	0	0.31
ctg	L	0	0.34
att	I	7	64.59
atc	I	0	48.35
ata	I	177	71.06
aaa	K	0	0.66
aag	K	1	0.34
gtt	V	98	9801.00
gtc	V	8	7973.00
gta	V	33942	8481.00
gtg	V	5083	12875.00
gct	A	0	1.09
gcc	A	0	1.00
gca	A	3	1.62
gcg	A	1	0.29
---	--	------	------
mPD		0.24	0.75
		nPD:	0.32
		N. weight:	1.2
		Sc. PD:	0.29
		Sc. rank:	33154.0

,

PB1			
Pos.	594	obs:	exp:
tct	S	212	6344.00
tcc	S	20	5875.00
tca	S	38843	10567.00
tcg	S	238	1548.00
agt	S	0	6870.00
agc	S	0	8110.00
gct	A	0	2.45
gcc	A	0	2.25
gca	A	9	3.64
gcg	A	0	0.66
---	--	------	------
mPD		0.024	1.6
		nPD:	0.01
		N. weight:	1.4
		Sc. PD:	-0.064
		Sc. rank:	-9968.0

,

PB1			
Pos.	595	obs:	exp:
gat	D	39277	22227.00
gac	D	45	17095.00
---	--	------	------
mPD		0.0023	0.49
		nPD:	0.
		N. weight:	0.62
		Sc. PD:	-0.035
		Sc. rank:	-14474.0

,

PB1			
Pos.	596	obs:	exp:
cgt	R	0	0.02
cgc	R	0	0.04
cga	R	0	0.09
cgg	R	0	0.09
aga	R	0	0.49
agg	R	1	0.28
ggt	G	3	5294.00
ggc	G	3	5488.00
gga	G	962	18151.00
ggg	G	38353	10388.00
---	--	------	------
mPD		0.048	0.68
		nPD:	0.07
		N. weight:	1.3
		Sc. PD:	0.011
		Sc. rank:	24091.0

,

PB1			
Pos.	597	obs:	exp:
cgt	R	0	0.02
cgc	R	0	0.04
cga	R	0	0.09
cgg	R	0	0.09
aga	R	1	0.49
agg	R	0	0.28
ggt	G	14	5294.00
ggc	G	22	5488.00
gga	G	33752	18151.00
ggg	G	5533	10388.00
---	--	------	------
mPD		0.24	0.68
		nPD:	0.36
		N. weight:	0.48
		Sc. PD:	0.14
		Sc. rank:	13800.0

,

PB1			
Pos.	598	obs:	exp:
tct	S	0	0.65
tcc	S	0	0.60
tca	S	4	1.08
tcg	S	0	0.16
cct	P	9	13102.00
ccc	P	6	7371.00
cca	P	34260	13283.00
ccg	P	5042	5561.00
agt	S	0	0.70
agc	S	0	0.83
gct	A	0	0.27
gcc	A	0	0.25
gca	A	1	0.40
gcg	A	0	0.07
---	--	------	------
mPD		0.22	0.72
		nPD:	0.31
		N. weight:	0.89
		Sc. PD:	0.22
		Sc. rank:	24830.0

,

PB1			
Pos.	599	obs:	exp:
att	I	0	0.35
atc	I	1	0.26
ata	I	0	0.39
aat	N	306	21999.00
aac	N	39015	17322.00
---	--	------	------
mPD		0.015	0.49
		nPD:	0.03
		N. weight:	0.85
		Sc. PD:	-0.026
		Sc. rank:	5392.2

,

PB1			
Pos.	600	obs:	exp:
ttt	F	1	0.49
ttc	F	0	0.51
tta	L	39145	4562.00
ttg	L	130	8128.00
tct	S	0	0.65
tcc	S	0	0.60
tca	S	4	1.08
tcg	S	0	0.16
ctt	L	0	8224.00
ctc	L	0	5765.00
cta	L	38	6037.00
ctg	L	0	6598.00
cct	P	0	0.33
ccc	P	0	0.19
cca	P	1	0.34
ccg	P	0	0.14
att	I	0	0.70
atc	I	0	0.53
ata	I	2	0.77
agt	S	0	0.70
agc	S	0	0.83
gtt	V	0	0.25
gtc	V	0	0.20
gta	V	1	0.22
gtg	V	0	0.33
---	--	------	------
mPD		0.0090	1.2
		nPD:	0.01
		N. weight:	2.3
		Sc. PD:	-0.12
		Sc. rank:	-39865.0

,

PB1			
Pos.	601	obs:	exp:
ttt	F	0	1.46
ttc	F	3	1.54
tat	Y	121	20724.00
tac	Y	39195	18592.00
tgt	C	0	0.78
tgc	C	2	1.22
cat	H	0	0.56
cac	H	1	0.44
---	--	------	------
mPD		0.0064	0.50
		nPD:	0.01
		N. weight:	0.8
		Sc. PD:	-0.039
		Sc. rank:	-7912.9

,

PB1			
Pos.	602	obs:	exp:
aat	N	38808	21999.00
aac	N	514	17323.00
---	--	------	------
mPD		0.026	0.49
		nPD:	0.05
		N. weight:	0.56
		Sc. PD:	-0.0056
		Sc. rank:	8204.6

,

PB1			
Pos.	603	obs:	exp:
att	I	471	13800.00
atc	I	38795	10330.00
ata	I	46	15182.00
gtt	V	0	2.50
gtc	V	10	2.04
gta	V	0	2.17
gtg	V	0	3.29
---	--	------	------
mPD		0.026	0.66
		nPD:	0.04
		N. weight:	1.4
		Sc. PD:	-0.03
		Sc. rank:	13786.0

,

PB1			
Pos.	604	obs:	exp:
cgt	R	5	779.70
cgc	R	1	1415.00
cga	R	8305	3588.00
cgg	R	30962	3376.00
aga	R	12	19287.00
agg	R	37	10876.00
---	--	------	------
mPD		0.34	0.89
		nPD:	0.38
		N. weight:	2.1
		Sc. PD:	0.65
		Sc. rank:	60686.0

,

PB1			
Pos.	605	obs:	exp:
aat	N	39028	21999.00
aac	N	294	17323.00
---	--	------	------
mPD		0.015	0.49
		nPD:	0.03
		N. weight:	0.59
		Sc. PD:	-0.019
		Sc. rank:	3389.2

,

PB1			
Pos.	606	obs:	exp:
ttt	F	11	5.35
ttc	F	0	5.65
tta	L	0	4561.00
ttg	L	0	8127.00
ctt	L	39133	8223.00
ctc	L	141	5764.00
cta	L	33	6036.00
ctg	L	0	6597.00
cct	P	1	0.33
ccc	P	0	0.19
cca	P	0	0.34
ccg	P	0	0.14
att	I	3	1.05
atc	I	0	0.79
ata	I	0	1.16
---	--	------	------
mPD		0.0096	1.2
		nPD:	0.01
		N. weight:	1.7
		Sc. PD:	-0.089
		Sc. rank:	-27123.0

,

PB1

Pos.	607	obs:	exp:
cat	H	44	22179.00
cac	H	39277	17142.00
caa	Q	1	0.61
cag	Q	0	0.39
---	--	------	------
mPD		0.0023	0.49
		nPD:	0.
		N. weight:	0.9
		Sc. PD:	-0.051
		Sc. rank:	-21199.0

,

PB1

Pos.	608	obs:	exp:
tta	L	0	0.23
ttg	L	0	0.41
ctt	L	0	0.42
ctc	L	2	0.29
cta	L	0	0.31
ctg	L	0	0.34
att	I	871	13799.00
atc	I	38428	10329.00
ata	I	10	15181.00
act	T	0	0.31
acc	T	1	0.18
aca	T	0	0.43
acg	T	0	0.08
gtt	V	0	2.50
gtc	V	10	2.04
gta	V	0	2.17
gtg	V	0	3.29
---	--	------	------
mPD		0.044	0.66
		nPD:	0.07
		N. weight:	1.3
		Sc. PD:	0.0064
		Sc. rank:	23405.0

,

PB1

Pos.	609	obs:	exp:
tct	S	3	0.48
tcc	S	0	0.45
tca	S	0	0.81
tcg	S	0	0.12
cct	P	39098	13103.00
ccc	P	161	7371.00
cca	P	24	13284.00
ccg	P	36	5561.00
agt	S	0	0.52
agc	S	0	0.62
---	--	------	------
mPD		0.011	0.72
		nPD:	0.02
		N. weight:	1.2
		Sc. PD:	-0.053
		Sc. rank:	-7219.7

,

PB1

Pos.	610	obs:	exp:
gaa	E	39153	23183.00
gag	E	169	16139.00
---	--	------	------
mPD		0.0086	0.48
		nPD:	0.02
		N. weight:	0.55
		Sc. PD:	-0.024
		Sc. rank:	-2340.2

,

PB1

Pos.	611	obs:	exp:
att	I	0	0.70
atc	I	2	0.53
ata	I	0	0.77
gtt	V	79	9844.00
gtc	V	39135	8008.00
gta	V	87	8518.00
gtg	V	0	12931.00
gct	A	0	5.18
gcc	A	19	4.75
gca	A	0	7.69
gcg	A	0	1.39
---	--	------	------
mPD		0.0095	0.74
		nPD:	0.01
		N. weight:	1.7
		Sc. PD:	-0.083
		Sc. rank:	-17074.0

,

PB1

Pos.	612	obs:	exp:
tgt	C	116	15367.00
tgc	C	39206	23955.00
---	--	------	------
mPD		0.0059	0.48
		nPD:	0.01
		N. weight:	0.52
		Sc. PD:	-0.026
		Sc. rank:	-5639.8

,

PB1

Pos.	613	obs:	exp:
ttt	F	1	0.49
ttc	F	0	0.51
tta	L	32399	4562.00
ttg	L	6821	8128.00
tct	S	0	0.16
tcc	S	0	0.15
tca	S	1	0.27
tcg	S	0	0.04
ctt	L	1	8224.00
ctc	L	0	5765.00
cta	L	86	6037.00
ctg	L	6	6598.00
att	I	0	2.11
atc	I	0	1.58
ata	I	6	2.32
agt	S	0	0.17
agc	S	0	0.21
gtt	V	0	0.25
gtc	V	0	0.20
gta	V	0	0.22
gtg	V	1	0.33
---	--	------	------
mPD		0.29	1.2
		nPD:	0.25
		N. weight:	1.7
		Sc. PD:	0.32
		Sc. rank:	47250.0

,

PB1

Pos.	614	obs:	exp:
cgt	R	0	0.02
cgc	R	0	0.04
cga	R	0	0.09
cgg	R	0	0.09
atg	M	1	1.00
aat	N	1	0.56
aac	N	0	0.44
aaa	K	1177	25850.00
aag	K	38142	13469.00
aga	R	0	0.49
agg	R	1	0.28
---	--	------	------
mPD		0.058	0.45
		nPD:	0.13
		N. weight:	1.
		Sc. PD:	0.065
		Sc. rank:	23818.0

,

PB1

Pos.	615	obs:	exp:
tgg	W	39322	39322.00
---	--	------	------
mPD		0	0
		nPD:	1.
		N. weight:	0.
		Sc. PD:	0
		Sc. rank:	0

,

PB1

Pos.	616	obs:	exp:
aat	N	2	1.12
aac	N	0	0.88
aaa	K	0	1.97
aag	K	3	1.03
gat	D	59	33.92
gac	D	1	26.08
gaa	E	115	23139.00
gag	E	39133	16109.00
ggt	G	0	1.21
ggc	G	0	1.26
gga	G	0	4.15
ggg	G	9	2.38
---	--	------	------
mPD		0.0097	0.49
		nPD:	0.02
		N. weight:	0.95
		Sc. PD:	-0.04
		Sc. rank:	-2362.9

,

PB1

Pos.	617	obs:	exp:
tta	L	0	4562.00
ttg	L	62	8128.00
ctt	L	894	8225.00
ctc	L	1	5765.00
cta	L	1078	6037.00
ctg	L	37281	6598.00
cct	P	0	0.33
ccc	P	0	0.19
cca	P	1	0.34
ccg	P	0	0.14
att	I	0	0.35
atc	I	0	0.26
ata	I	1	0.39
atg	M	3	3.00
gtt	V	0	0.25
gtc	V	0	0.20
gta	V	0	0.22
gtg	V	1	0.33
---	--	------	------
mPD		0.10	1.2
		nPD:	0.09
		N. weight:	1.7
		Sc. PD:	0.039
		Sc. rank:	35136.0

,

PB1

Pos.	618	obs:	exp:
atg	M	39322	39322.00
---	--	------	------
mPD		0	0
		nPD:	1.
		N. weight:	0.
		Sc. PD:	0
		Sc. rank:	0

,

PB1			
Pos.	619	obs:	exp:
gat	D	33023	22225.00
gac	D	6295	17093.00
gaa	E	1	1.18
gag	E	1	0.82
ggt	G	1	0.27
ggc	G	1	0.28
gga	G	0	0.92
ggg	G	0	0.53
---	--	------	------
mPD		0.27	0.49
		nPD:	0.55
		N. weight:	0.19
		Sc. PD:	0.09
		Sc. rank:	5690.5

,

PB1			
Pos.	620	obs:	exp:
tat	Y	0	0.53
tac	Y	1	0.47
aat	N	0	4.48
aac	N	8	3.52
aaa	K	0	15.12
aag	K	23	7.88
gtt	V	5	2.50
gtc	V	5	2.04
gta	V	0	2.17
gtg	V	0	3.29
gct	A	1	1.64
gcc	A	2	1.50
gca	A	0	2.43
gcg	A	3	0.44
gat	D	3260	5308.00
gac	D	6130	4082.00
gaa	E	64	17609.00
gag	E	29804	12259.00
ggt	G	0	2.15
ggc	G	3	2.23
gga	G	0	7.39
ggg	G	13	4.23
---	--	------	------
mPD		0.40	0.68
		nPD:	0.59
		N. weight:	0.76
		Sc. PD:	0.39
		Sc. rank:	22807.0

,

PB1			
Pos.	621	obs:	exp:
tct	S	0	19.20
tcc	S	0	17.78
tca	S	0	31.98
tcg	S	0	4.68
att	I	1	0.35
atc	I	0	0.26
ata	I	0	0.39
act	T	7	2.17
acc	T	0	1.24
aca	T	0	3.03
acg	T	0	0.56
aat	N	38158	21421.00
aac	N	130	16867.00
aaa	K	38	25.64
aag	K	1	13.36
agt	S	119	20.80
agc	S	0	24.55
gct	A	1	0.27
gcc	A	0	0.25
gca	A	0	0.40
gcg	A	0	0.07
gat	D	781	488.40
gac	D	83	375.60
gaa	E	3	1.77
gag	E	0	1.23
---	--	------	------
mPD		0.063	0.55
		nPD:	0.11
		N. weight:	0.61
		Sc. PD:	0.031
		Sc. rank:	13818.0

,

PB1			
Pos.	622	obs:	exp:
tat	Y	39117	20727.00
tac	Y	205	18595.00
---	--	------	------
mPD		0.010	0.50
		nPD:	0.02
		N. weight:	0.67
		Sc. PD:	-0.027
		Sc. rank:	-1112.5

,

PB1			
Pos.	623	obs:	exp:
caa	Q	1	148.50
cag	Q	242	94.46
cgt	R	3	774.00
cgc	R	0	1405.00
cga	R	1162	3562.00
cgg	R	32790	3352.00
aaa	K	1	26.30
aag	K	39	13.70
aga	R	278	19148.00
agg	R	4806	10798.00
---	--	------	------
mPD		0.31	0.90
		nPD:	0.34
		N. weight:	1.9
		Sc. PD:	0.53
		Sc. rank:	54839.0

,

PB1			
Pos.	624	obs:	exp:
ggt	G	41	5294.00
ggc	G	7	5488.00
gga	G	39133	18152.00
ggg	G	141	10388.00
---	--	------	------
mPD		0.0096	0.68
		nPD:	0.01
		N. weight:	0.82
		Sc. PD:	-0.039
		Sc. rank:	-6681.1

,

PB1			
Pos.	625	obs:	exp:
cgt	R	3	779.60
cgc	R	0	1415.00
cga	R	5	3587.00
cgg	R	1	3376.00
act	T	0	0.31
acc	T	0	0.18
aca	T	1	0.43
acg	T	0	0.08
aaa	K	2	1.31
aag	K	0	0.69
aga	R	39221	19285.00
agg	R	89	10876.00
---	--	------	------
mPD		0.0053	0.89
		nPD:	0.01
		N. weight:	0.76
		Sc. PD:	-0.042
		Sc. rank:	-15806.0

,

PB1			
Pos.	626	obs:	exp:
tta	L	3	4562.00
ttg	L	0	8127.00
ctt	L	39230	8223.00
ctc	L	69	5765.00
cta	L	3	6036.00
ctg	L	6	6597.00
att	I	11	3.86
atc	I	0	2.89
ata	I	0	4.25
---	--	------	------
mPD		0.0048	1.2
		nPD:	0.
		N. weight:	1.7
		Sc. PD:	-0.097
		Sc. rank:	-41217.0

,

PB1			
Pos.	627	obs:	exp:
ttt	F	2	0.97
ttc	F	0	1.03
tct	S	1	0.16
tcc	S	0	0.15
tca	S	0	0.27
tcg	S	0	0.04
tgt	C	35408	15365.00
tgc	C	3909	23952.00
tgg	W	1	1.00
agt	S	0	0.17
agc	S	0	0.21
ggt	G	0	0.13
ggc	G	0	0.14
gga	G	0	0.46
ggg	G	1	0.26
---	--	------	------
mPD		0.18	0.48
		nPD:	0.38
		N. weight:	0.63
		Sc. PD:	0.19
		Sc. rank:	18022.0

,

PB1			
Pos.	628	obs:	exp:
tat	Y	2	1.05
tac	Y	0	0.95
cat	H	2	1.13
cac	H	0	0.87
aat	N	38766	21997.00
aac	N	552	17321.00
---	--	------	------
mPD		0.028	0.49
		nPD:	0.06
		N. weight:	0.56
		Sc. PD:	-0.0033
		Sc. rank:	8710.6

,

PB1			
Pos.	629	obs:	exp:
tta	L	0	0.12
ttg	L	0	0.21
tct	S	0	1.94
tcc	S	12	1.79
tca	S	0	3.23
tcg	S	0	0.47
ctt	L	0	0.21
ctc	L	1	0.15
cta	L	0	0.15
ctg	L	0	0.17
cct	P	530	13099.00
ccc	P	38695	7369.00
cca	P	82	13280.00
ccg	P	0	5559.00
cat	H	0	0.56
cac	H	1	0.44
act	T	0	0.31
acc	T	1	0.18
aca	T	0	0.43
acg	T	0	0.08
agt	S	0	2.10
agc	S	0	2.48
---	--	------	------
mPD		0.031	0.72
		nPD:	0.04
		N. weight:	1.7
		Sc. PD:	-0.032
		Sc. rank:	19491.0

,

PB1			
Pos.	630	obs:	exp:
tta	L	1	4559.00
ttg	L	198	8122.00
ctt	L	21	8218.00
ctc	L	2	5761.00
cta	L	342	6032.00
ctg	L	38720	6593.00
cct	P	1	0.33
ccc	P	0	0.19
cca	P	0	0.34
ccg	P	0	0.14
caa	Q	0	5.50
cag	Q	9	3.50
cgt	R	0	0.06
cgc	R	0	0.11
cga	R	0	0.27
cgg	R	3	0.26
atg	M	25	25.00
aga	R	0	1.47
agg	R	0	0.83
---	--	------	------
mPD		0.030	1.2
		nPD:	0.03
		N. weight:	1.9
		Sc. PD:	-0.066
		Sc. rank:	6906.3

,

PB1

Pos.	631	obs:	exp:
tct	S	0	0.16
tcc	S	0	0.15
tca	S	0	0.27
tcg	S	0	0.04
cat	H	1	0.56
cac	H	0	0.44
aat	N	39168	21998.00
aac	N	151	17321.00
agt	S	1	0.17
agc	S	0	0.21
gat	D	1	0.57
gac	D	0	0.43
---	--	------	------
mPD		0.0078	0.49
		nPD:	0.02
		N. weight:	0.61
		Sc. PD:	-0.028
		Sc. rank:	-3749.0

,

PB1

Pos.	632	obs:	exp:
cct	P	588	13104.00
ccc	P	38107	7372.00
cca	P	627	13285.00
ccg	P	0	5562.00
---	--	------	------
mPD		0.060	0.72
		nPD:	0.08
		N. weight:	1.6
		Sc. PD:	0.034
		Sc. rank:	33874.0

,

PB1

Pos.	633	obs:	exp:
ttt	F	39291	19126.00
ttc	F	29	20194.00
tat	Y	1	0.53
tac	Y	0	0.47
tgt	C	1	0.39
tgc	C	0	0.61
---	--	------	------
mPD		0.0016	0.50
		nPD:	0.
		N. weight:	0.78
		Sc. PD:	-0.045
		Sc. rank:	-20267.0

,

PB1

Pos.	634	obs:	exp:
ttt	F	0	0.49
ttc	F	1	0.51
att	I	0	6.67
atc	I	19	4.99
ata	I	0	7.34
gtt	V	154	9843.00
gtc	V	38706	8007.00
gta	V	420	8517.00
gtg	V	16	12929.00
gct	A	0	1.64
gcc	A	6	1.50
gca	A	0	2.43
gcg	A	0	0.44
---	--	------	------
mPD		0.031	0.74
		nPD:	0.04
		N. weight:	1.6
		Sc. PD:	-0.033
		Sc. rank:	17371.0

,

PB1

Pos.	635	obs:	exp:
tct	S	0	6328.00
tcc	S	0	5860.00
tca	S	0	10540.00
tcg	S	0	1544.00
cgt	R	0	0.02
cgc	R	0	0.04
cga	R	0	0.09
cgg	R	0	0.09
att	I	0	1.76
atc	I	5	1.31
ata	I	0	1.93
act	T	0	0.31
acc	T	1	0.18
aca	T	0	0.43
acg	T	0	0.08
aat	N	0	52.03
aac	N	93	40.97
agt	S	136	6853.00
agc	S	39078	8090.00
aga	R	1	0.49
agg	R	0	0.28
ggt	G	0	1.08
ggc	G	8	1.12
gga	G	0	3.69
ggg	G	0	2.11
---	--	------	------
mPD		0.012	1.6
		nPD:	0.01
		N. weight:	1.7
		Sc. PD:	-0.091
		Sc. rank:	-30087.0

,

PB1

Pos.	636	obs:	exp:
tat	Y	4	2.11
tac	Y	0	1.89
cat	H	39222	22175.00
cac	H	93	17140.00
caa	Q	1	0.61
cag	Q	0	0.39
aat	N	2	1.12
aac	N	0	0.88
---	--	------	------
mPD		0.0051	0.49
		nPD:	0.01
		N. weight:	0.61
		Sc. PD:	-0.031
		Sc. rank:	-7891.3

,

PB1

Pos.	637	obs:	exp:
cgt	R	0	0.24
cgc	R	0	0.43
cga	R	0	1.09
cgg	R	0	1.03
aaa	K	39132	25844.00
aag	K	178	13466.00
aga	R	12	5.89
agg	R	0	3.32
---	--	------	------
mPD		0.0096	0.45
		nPD:	0.02
		N. weight:	0.43
		Sc. PD:	-0.017
		Sc. rank:	-506.3

,

PB1

Pos.	638	obs:	exp:
gat	D	3	1.70
gac	D	0	1.30
gaa	E	38650	23179.00
gag	E	666	16137.00
ggt	G	0	0.40
ggc	G	0	0.42
gga	G	3	1.38
ggg	G	0	0.79
---	--	------	------
mPD		0.034	0.48
		nPD:	0.07
		N. weight:	0.49
		Sc. PD:	0.0033
		Sc. rank:	8744.5

,

PB1

Pos.	639	obs:	exp:
ttt	F	3	1.46
ttc	F	0	1.54
tta	L	0	0.23
ttg	L	0	0.41
ctt	L	2	0.42
ctc	L	0	0.29
cta	L	0	0.31
ctg	L	0	0.34
att	I	39210	13782.00
atc	I	15	10316.00
ata	I	35	15162.00
act	T	2	0.62
acc	T	0	0.35
aca	T	0	0.87
acg	T	0	0.16
gtt	V	55	13.78
gtc	V	0	11.21
gta	V	0	11.92
gtg	V	0	18.10
---	--	------	------
mPD		0.0057	0.66
		nPD:	0.01
		N. weight:	1.1
		Sc. PD:	-0.06
		Sc. rank:	-17908.0

,

PB1

Pos.	640	obs:	exp:
aaa	K	7	4.60
aag	K	0	2.40
gat	D	262	158.30
gac	D	18	121.70
gaa	E	35052	23006.00
gag	E	3971	16017.00
ggt	G	1	1.62
ggc	G	0	1.67
gga	G	11	5.54
ggg	G	0	3.17
---	--	------	------
mPD		0.20	0.49
		nPD:	0.4
		N. weight:	0.26
		Sc. PD:	0.085
		Sc. rank:	7576.1

,

PB1

Pos.	641	obs:	exp:
ttt	F	7	3.40
ttc	F	0	3.60
tct	S	39247	6344.00
tcc	S	20	5875.00
tca	S	43	10567.00
tcg	S	5	1548.00
agt	S	0	6870.00
agc	S	0	8111.00
---	--	------	------
mPD		0.0038	1.6
		nPD:	0.
		N. weight:	2.
		Sc. PD:	-0.12
		Sc. rank:	-54649.0

,

PB1

Pos.	642	obs:	exp:
tta	L	5	0.58
ttg	L	0	1.03
ctt	L	0	1.05
ctc	L	0	0.73
cta	L	0	0.77
ctg	L	0	0.84
att	I	0	41.77
atc	I	0	31.27
ata	I	119	45.96
atg	M	3	3.00
gtt	V	2	9817.00
gtc	V	2	7986.00
gta	V	38997	8495.00
gtg	V	193	12896.00
ggt	G	1	0.13
ggc	G	0	0.14
gga	G	0	0.46
ggg	G	0	0.26
---	--	------	------
mPD		0.017	0.75
		nPD:	0.02
		N. weight:	1.6
		Sc. PD:	-0.064
		Sc. rank:	41.3

,

PB1			
Pos.	643	obs:	exp:
tct	S	0	0.32
tcc	S	0	0.30
tca	S	0	0.54
tcg	S	0	0.08
aat	N	131	21998.00
aac	N	39189	17322.00
agt	S	0	0.35
agc	S	2	0.41
---	--	------	------
mPD		0.0067	0.49
		nPD:	0.01
		N. weight:	0.87
		Sc. PD:	-0.042
		Sc. rank:	-7506.0

,

PB1			
Pos.	644	obs:	exp:
tct	S	0	44.05
tcc	S	0	40.80
tca	S	0	73.38
tcg	S	0	10.75
tat	Y	3	1.58
tac	Y	0	1.42
cat	H	1	0.56
cac	H	0	0.44
act	T	1	0.31
acc	T	0	0.18
aca	T	0	0.43
acg	T	0	0.08
aat	N	38987	21844.00
aac	N	57	17200.00
agt	S	273	47.71
agc	S	0	56.32
---	--	------	------
mPD		0.017	0.52
		nPD:	0.03
		N. weight:	0.63
		Sc. PD:	-0.018
		Sc. rank:	4401.0

,

PB1			
Pos.	645	obs:	exp:
act	T	8	2.48
acc	T	0	1.41
aca	T	0	3.46
acg	T	0	0.64
gtt	V	3	0.75
gtc	V	0	0.61
gta	V	0	0.65
gtg	V	0	0.99
gct	A	39157	10714.00
gcc	A	46	9821.00
gca	A	42	15901.00
gcg	A	65	2874.00
gat	D	1	0.57
gac	D	0	0.43
---	--	------	------
mPD		0.0084	0.69
		nPD:	0.01
		N. weight:	1.4
		Sc. PD:	-0.069
		Sc. rank:	-15330.0

,

PB1			
Pos.	646	obs:	exp:
att	I	0	17.90
atc	I	0	13.40
ata	I	51	19.70
atg	M	1	1.00
gtt	V	5	9835.00
gtc	V	6	8001.00
gta	V	38563	8511.00
gtg	V	692	12919.00
gct	A	0	0.82
gcc	A	0	0.75
gca	A	3	1.21
gcg	A	0	0.22
ggt	G	1	0.13
ggc	G	0	0.14
gga	G	0	0.46
ggg	G	0	0.26
---	--	------	------
mPD		0.038	0.74
		nPD:	0.05
		N. weight:	1.6
		Sc. PD:	-0.017
		Sc. rank:	22329.0

,

PB1			
Pos.	647	obs:	exp:
tta	L	1	0.12
ttg	L	0	0.21
ctt	L	0	0.21
ctc	L	0	0.15
cta	L	0	0.15
ctg	L	0	0.17
att	I	0	34.40
atc	I	0	25.75
ata	I	98	37.85
atg	M	6	6.00
gtt	V	1	9822.00
gtc	V	0	7990.00
gta	V	38311	8499.00
gtg	V	902	12902.00
gct	A	0	0.27
gcc	A	0	0.25
gca	A	1	0.40
gcg	A	0	0.07
ggt	G	1	0.27
ggc	G	0	0.28
gga	G	1	0.92
ggg	G	0	0.53
---	--	------	------
mPD		0.051	0.75
		nPD:	0.07
		N. weight:	1.5
		Sc. PD:	0.0082
		Sc. rank:	27178.0

,

PB1			
Pos.	648	obs:	exp:
cgt	R	0	0.06
cgc	R	0	0.11
cga	R	0	0.27
cgg	R	0	0.26
att	I	3	10.18
atc	I	1	7.62
ata	I	25	11.20
atg	M	39273	39273.00
act	T	0	0.62
acc	T	0	0.35
aca	T	0	0.87
acg	T	2	0.16
aga	R	0	1.47
agg	R	3	0.83
gtt	V	0	3.76
gtc	V	0	3.06
gta	V	0	3.25
gtg	V	15	4.94
---	--	------	------
mPD		0.0025	0.0032
		nPD:	0.77
		N. weight:	0.0012
		Sc. PD:	0.00082
		Sc. rank:	36.4

,

PB1			
Pos.	649	obs:	exp:
tct	S	0	0.65
tcc	S	0	0.60
tca	S	4	1.08
tcg	S	0	0.16
cct	P	12	13098.00
ccc	P	4	7369.00
cca	P	39249	13280.00
ccg	P	41	5559.00
caa	Q	10	6.11
cag	Q	0	3.89
act	T	0	0.62
acc	T	0	0.35
aca	T	2	0.87
acg	T	0	0.16
agt	S	0	0.70
agc	S	0	0.83
---	--	------	------
mPD		0.0037	0.72
		nPD:	0.01
		N. weight:	1.2
		Sc. PD:	-0.066
		Sc. rank:	-26214.0

,

PB1			
Pos.	650	obs:	exp:
tct	S	0	3.07
tcc	S	19	2.84
tca	S	0	5.11
tcg	S	0	0.75
act	T	0	4.35
acc	T	14	2.48
aca	T	0	6.06
acg	T	0	1.12
agt	S	0	3.32
agc	S	0	3.92
gtt	V	0	0.50
gtc	V	2	0.41
gta	V	0	0.43
gtg	V	0	0.66
gct	A	251	10708.00
gcc	A	39012	9815.00
gca	A	22	15892.00
gcg	A	2	2872.00
---	--	------	------
mPD		0.016	0.70
		nPD:	0.02
		N. weight:	1.5
		Sc. PD:	-0.058
		Sc. rank:	317.1

,

PB1			
Pos.	651	obs:	exp:
tat	Y	1	0.53
tac	Y	0	0.47
cat	H	39150	22178.00
cac	H	169	17141.00
caa	Q	0	0.61
cag	Q	1	0.39
aat	N	1	0.56
aac	N	0	0.44
---	--	------	------
mPD		0.0087	0.49
		nPD:	0.02
		N. weight:	0.6
		Sc. PD:	-0.026
		Sc. rank:	-2515.1

,

PB1			
Pos.	652	obs:	exp:
ggt	G	38996	5294.00
ggc	G	317	5488.00
gga	G	5	18152.00
ggg	G	4	10388.00
---	--	------	------
mPD		0.016	0.68
		nPD:	0.02
		N. weight:	2.1
		Sc. PD:	-0.08
		Sc. rank:	2971.6

,

PB1			
Pos.	653	obs:	exp:
cct	P	2370	13104.00
ccc	P	2	7372.00
cca	P	1159	13285.00
ccg	P	35791	5562.00
---	--	------	------
mPD		0.17	0.72
		nPD:	0.23
		N. weight:	1.7
		Sc. PD:	0.28
		Sc. rank:	44769.0

,

PB1			
Pos.	654	obs:	exp:
tct	S	0	1.13
tcc	S	7	1.05
tca	S	0	1.88
tcg	S	0	0.28
act	T	0	7.45
acc	T	24	4.24
aca	T	0	10.39
acg	T	0	1.92
agt	S	0	1.22
agc	S	0	1.44
gtt	V	0	7.76
gtc	V	31	6.32
gta	V	0	6.72
gtg	V	0	10.20
gct	A	46	10700.00
gcc	A	39078	9809.00
gca	A	136	15881.00
gcg	A	0	2870.00
---	--	------	------
mPD		0.012	0.70
		nPD:	0.02
		N. weight:	1.5
		Sc. PD:	-0.065
		Sc. rank:	-6054.6

,

PB1

Pos.	655	obs:	exp:
caa	Q	1	0.61
cag	Q	0	0.39
cgt	R	0	0.20
cgc	R	0	0.36
cga	R	0	0.91
cgg	R	0	0.86
aaa	K	39120	25844.00
aag	K	191	13467.00
aga	R	9	4.90
agg	R	1	2.77
---	--	------	------
mPD		0.010	0.45
		nPD:	0.02
		N. weight:	0.43
		Sc. PD:	-0.017
		Sc. rank:	151.0

,

PB1

Pos.	656	obs:	exp:
tct	S	0	6306.00
tcc	S	0	5840.00
tca	S	0	10503.00
tcg	S	0	1538.00
tgt	C	23	8.99
tgc	C	0	14.01
att	I	4	1.40
atc	I	0	1.05
ata	I	0	1.54
act	T	1	0.31
acc	T	0	0.18
aca	T	0	0.43
acg	T	0	0.08
aat	N	157	119.70
aac	N	57	94.27
agt	S	38426	6829.00
agc	S	651	8061.00
ggt	G	3	0.40
ggc	G	0	0.42
gga	G	0	1.38
ggg	G	0	0.79
---	--	------	------
mPD		0.048	1.6
		nPD:	0.03
		N. weight:	1.8
		Sc. PD:	-0.059
		Sc. rank:	9661.4

,

PB1

Pos.	657	obs:	exp:
tta	L	0	0.58
ttg	L	4	1.03
ctt	L	0	1.05
ctc	L	0	0.73
cta	L	0	0.77
ctg	L	1	0.84
att	I	2	5.62
atc	I	0	4.20
ata	I	14	6.18
atg	M	39283	39283.00
gtt	V	0	4.51
gtc	V	0	3.67
gta	V	0	3.90
gtg	V	18	5.92
---	--	------	------
mPD		0.0020	0.0028
		nPD:	0.72
		N. weight:	0.001
		Sc. PD:	0.00066
		Sc. rank:	31.8

,

PB1

Pos.	658	obs:	exp:
caa	Q	1	0.61
cag	Q	0	0.39
gtt	V	0	0.25
gtc	V	0	0.20
gta	V	1	0.22
gtg	V	0	0.33
gat	D	0	3.39
gac	D	6	2.61
gaa	E	39113	23177.00
gag	E	199	16135.00
ggt	G	0	0.27
ggc	G	0	0.28
gga	G	2	0.92
ggg	G	0	0.53
---	--	------	------
mPD		0.011	0.48
		nPD:	0.02
		N. weight:	0.55
		Sc. PD:	-0.022
		Sc. rank:	-234.9

,

PB1

Pos.	659	obs:	exp:
tat	Y	39206	20725.00
tac	Y	113	18594.00
cat	H	1	0.56
cac	H	0	0.44
gat	D	1	0.57
gac	D	0	0.43
ggt	G	1	0.13
ggc	G	0	0.14
gga	G	0	0.46
ggg	G	0	0.26
---	--	------	------
mPD		0.0059	0.50
		nPD:	0.01
		N. weight:	0.68
		Sc. PD:	-0.034
		Sc. rank:	-7623.0

,

PB1

Pos.	660	obs:	exp:
cat	H	2	1.13
cac	H	0	0.87
gat	D	39107	22219.00
gac	D	200	17088.00
gaa	E	9	7.66
gag	E	4	5.34
---	--	------	------
mPD		0.011	0.49
		nPD:	0.02
		N. weight:	0.59
		Sc. PD:	-0.023
		Sc. rank:	-106.0

,

PB1

Pos.	661	obs:	exp:
tct	S	0	0.32
tcc	S	2	0.30
tca	S	0	0.54
tcg	S	0	0.08
agt	S	0	0.35
agc	S	0	0.41
gct	A	334	10717.00
gcc	A	38491	9824.00
gca	A	495	15905.00
gcg	A	0	2874.00
---	--	------	------
mPD		0.042	0.69
		nPD:	0.06
		N. weight:	1.4
		Sc. PD:	-0.0036
		Sc. rank:	23074.0

,

PB1

Pos.	662	obs:	exp:
att	I	16	5.62
atc	I	0	4.20
ata	I	0	6.18
gtt	V	39239	9845.00
gtc	V	52	8009.00
gta	V	11	8519.00
gtg	V	4	12933.00
---	--	------	------
mPD		0.0042	0.74
		nPD:	0.01
		N. weight:	1.5
		Sc. PD:	-0.083
		Sc. rank:	-32054.0

,

PB1

Pos.	663	obs:	exp:
tct	S	0	0.48
tcc	S	0	0.45
tca	S	1	0.81
tcg	S	2	0.12
act	T	0	4.66
acc	T	0	2.65
aca	T	9	6.49
acg	T	6	1.20
agt	S	0	0.52
agc	S	0	0.62
gtt	V	0	0.75
gtc	V	0	0.61
gta	V	0	0.65
gtg	V	3	0.99
gct	A	160	10712.00
gcc	A	2	9819.00
gca	A	22877	15897.00
gcg	A	16262	2873.00
---	--	------	------
mPD		0.49	0.70
		nPD:	0.71
		N. weight:	1.
		Sc. PD:	0.63
		Sc. rank:	30584.0

,

PB1

Pos.	664	obs:	exp:
att	I	1	0.35
atc	I	0	0.26
ata	I	0	0.39
act	T	38592	12208.00
acc	T	702	6950.00
aca	T	13	17013.00
acg	T	4	3140.00
aat	N	10	5.59
aac	N	0	4.41
---	--	------	------
mPD		0.036	0.68
		nPD:	0.05
		N. weight:	1.2
		Sc. PD:	-0.01
		Sc. rank:	17771.0

,

PB1

Pos.	665	obs:	exp:
tct	S	0	0.16
tcc	S	0	0.15
tca	S	1	0.27
tcg	S	0	0.04
cct	P	0	0.33
ccc	P	1	0.19
cca	P	0	0.34
ccg	P	0	0.14
act	T	5	12211.00
acc	T	33	6952.00
aca	T	39169	17017.00
acg	T	113	3140.00
agt	S	0	0.17
agc	S	0	0.21
---	--	------	------
mPD		0.0078	0.68
		nPD:	0.01
		N. weight:	0.89
		Sc. PD:	-0.045
		Sc. rank:	-10259.0

,

PB1

Pos.	666	obs:	exp:
cat	H	12688	22178.00
cac	H	26632	17142.00
caa	Q	0	0.61
cag	Q	1	0.39
gat	D	0	0.57
gac	D	1	0.43
---	--	------	------
mPD		0.44	0.49
		nPD:	0.89
		N. weight:	0.13
		Sc. PD:	0.1
		Sc. rank:	4021.8

,

PB1

Pos.	667	obs:	exp:
tct	S	154	6345.00
tcc	S	38508	5876.00
tca	S	656	10568.00
tcg	S	1	1548.00
act	T	0	0.62
acc	T	2	0.35
aca	T	0	0.87
acg	T	0	0.16
agt	S	0	6871.00
agc	S	0	8111.00
gct	A	0	0.27
gcc	A	1	0.25
gca	A	0	0.40
gcg	A	0	0.07
---	--	------	------
mPD		0.041	1.6
		nPD:	0.02
		N. weight:	2.
		Sc. PD:	-0.072
		Sc. rank:	4035.1

,

PB1

Pos.	668	obs:	exp:
tgt	C	0	0.39
tgc	C	1	0.61
tgg	W	39319	39319.00
ggt	G	0	0.27
ggc	G	0	0.28
gga	G	0	0.92
ggg	G	2	0.53
---	--	------	------
mPD		0.00015	0.00023
		nPD:	0.67
		N. weight:	0.000088
		Sc. PD:	0.000052
		Sc. rank:	2.7

,

PB1

Pos.	669	obs:	exp:
ttt	F	2	0.97
ttc	F	0	1.03
tta	L	0	0.23
ttg	L	0	0.41
tct	S	0	0.48
tcc	S	0	0.45
tca	S	0	0.81
tcg	S	0	0.12
ctt	L	2	0.42
ctc	L	0	0.29
cta	L	0	0.31
ctg	L	0	0.34
att	I	38586	13704.00
atc	I	438	10258.00
ata	I	15	15077.00
atg	M	1	1.00
act	T	107	33.23
acc	T	0	18.92
aca	T	0	46.31
acg	T	0	8.55
aat	N	19	10.63
aac	N	0	8.37
agt	S	3	0.52
agc	S	0	0.62
gtt	V	149	37.32
gtc	V	0	30.36
gta	V	0	32.29
gtg	V	0	49.02
---	--	------	------
mPD		0.037	0.67
		nPD:	0.06
		N. weight:	1.1
		Sc. PD:	-0.0079
		Sc. rank:	16706.0

,

PB1

Pos.	670	obs:	exp:
tta	L	0	0.93
ttg	L	0	1.65
tct	S	0	2.90
tcc	S	18	2.69
tca	S	0	4.84
tcg	S	0	0.71
ctt	L	0	1.67
ctc	L	8	1.17
cta	L	0	1.23
ctg	L	0	1.34
cct	P	154	13095.00
ccc	P	39127	7367.00
cca	P	14	13276.00
ccg	P	0	5558.00
cat	H	0	0.56
cac	H	1	0.44
agt	S	0	3.15
agc	S	0	3.71
---	--	------	------
mPD		0.0099	0.72
		nPD:	0.01
		N. weight:	1.8
		Sc. PD:	-0.086
		Sc. rank:	-15214.0

,

PB1

Pos.	671	obs:	exp:
cgt	R	0	0.14
cgc	R	0	0.25
cga	R	0	0.64
cgg	R	0	0.60
aat	N	1	0.56
aac	N	0	0.44
aaa	K	37023	25846.00
aag	K	2291	13468.00
aga	R	6	3.43
agg	R	1	1.94
---	--	------	------
mPD		0.11	0.45
		nPD:	0.24
		N. weight:	0.26
		Sc. PD:	0.046
		Sc. rank:	7014.4

,

PB1

Pos.	672	obs:	exp:
cgt	R	0	779.60
cgc	R	0	1415.00
cga	R	0	3587.00
cgg	R	3	3376.00
aaa	K	0	1.97
aag	K	3	1.03
aga	R	236	19285.00
agg	R	39080	10876.00
---	--	------	------
mPD		0.012	0.89
		nPD:	0.01
		N. weight:	1.4
		Sc. PD:	-0.065
		Sc. rank:	-11371.0

,

PB1

Pos.	673	obs:	exp:
aat	N	145	21999.00
aac	N	39176	17322.00
gat	D	0	0.57
gac	D	1	0.43
---	--	------	------
mPD		0.0074	0.49
		nPD:	0.02
		N. weight:	0.87
		Sc. PD:	-0.04
		Sc. rank:	-6193.3

,

PB1

Pos.	674	obs:	exp:
cat	H	0	0.56
cac	H	1	0.44
cgt	R	2729	779.60
cgc	R	36532	1415.00
cga	R	60	3588.00
cgg	R	0	3376.00
aga	R	0	19286.00
agg	R	0	10876.00
---	--	------	------
mPD		0.13	0.89
		nPD:	0.15
		N. weight:	3.4
		Sc. PD:	0.29
		Sc. rank:	84249.0

,

PB1

Pos.	675	obs:	exp:
tct	S	34361	6345.00
tcc	S	4838	5876.00
tca	S	119	10569.00
tcg	S	3	1548.00
cct	P	1	0.33
ccc	P	0	0.19
cca	P	0	0.34
ccg	P	0	0.14
agt	S	0	6871.00
agc	S	0	8112.00
---	--	------	------
mPD		0.22	1.6
		nPD:	0.14
		N. weight:	1.6
		Sc. PD:	0.11
		Sc. rank:	38402.0

,

PB1

Pos.	676	obs:	exp:
att	I	39078	13797.00
atc	I	174	10328.00
ata	I	52	15179.00
act	T	2	0.62
acc	T	0	0.35
aca	T	0	0.87
acg	T	0	0.16
gtt	V	16	4.01
gtc	V	0	3.26
gta	V	0	3.47
gtg	V	0	5.26
---	--	------	------
mPD		0.012	0.66
		nPD:	0.02
		N. weight:	1.1
		Sc. PD:	-0.047
		Sc. rank:	-3861.7

,

PB1

Pos.	677	obs:	exp:
tta	L	54	4563.00
ttg	L	3	8130.00
ctt	L	4	8226.00
ctc	L	850	5766.00
cta	L	38305	6038.00
ctg	L	106	6599.00
---	--	------	------
mPD		0.051	1.2
		nPD:	0.04
		N. weight:	1.9
		Sc. PD:	-0.035
		Sc. rank:	21776.0

,

PB1

Pos.	678	obs:	exp:
aat	N	217	21999.00
aac	N	39104	17322.00
gat	D	0	0.57
gac	D	1	0.43
---	--	------	------
mPD		0.011	0.49
		nPD:	0.02
		N. weight:	0.86
		Sc. PD:	-0.034
		Sc. rank:	82.0

,

PB1

Pos.	679	obs:	exp:
act	T	15	12212.00
acc	T	5	6952.00
aca	T	39214	17018.00
acg	T	88	3141.00
---	--	------	------
mPD		0.0055	0.68
		nPD:	0.01
		N. weight:	0.9
		Sc. PD:	-0.048
		Sc. rank:	-15024.0

,

PB1

Pos.	680	obs:	exp:
tct	S	0	6344.00
tcc	S	0	5875.00
tca	S	0	10568.00
tcg	S	0	1548.00
tgt	C	0	0.39
tgc	C	1	0.61
att	I	0	0.35
atc	I	1	0.26
ata	I	0	0.39
aat	N	0	1.68
aac	N	3	1.32
agt	S	1606	6871.00
agc	S	37711	8111.00
---	--	------	------
mPD		0.079	1.6
		nPD:	0.05
		N. weight:	1.6
		Sc. PD:	-0.022
		Sc. rank:	20365.0

,

PB1

Pos.	681	obs:	exp:
caa	Q	39296	24037.00
cag	Q	26	15285.00
---	--	------	------
mPD		0.0013	0.48
		nPD:	0.
		N. weight:	0.53
		Sc. PD:	-0.031
		Sc. rank:	-14183.0

,

PB1

Pos.	682	obs:	exp:
cgt	R	0	778.70
cgc	R	0	1413.00
cga	R	0	3584.00
cgg	R	7	3373.00
aaa	K	0	30.24
aag	K	46	15.76
aga	R	326	19264.00
agg	R	38943	10864.00
---	--	------	------
mPD		0.019	0.89
		nPD:	0.02
		N. weight:	1.4
		Sc. PD:	-0.054
		Sc. rank:	-1111.3

,

PB1			
Pos.	683	obs:	exp:
ggt	G	2	5294.00
ggc	G	1	5488.00
gga	G	35673	18152.00
ggg	G	3646	10388.00
---	--	------	------
mPD		0.17	0.68
		nPD:	0.25
		N. weight:	0.57
		Sc. PD:	0.1
		Sc. rank:	15385.0

,

PB1			
Pos.	684	obs:	exp:
att	I	38649	13792.00
atc	I	615	10323.00
ata	I	24	15173.00
act	T	1	0.31
acc	T	0	0.18
aca	T	0	0.43
acg	T	0	0.08
gtt	V	31	8.27
gtc	V	0	6.72
gta	V	2	7.15
gtg	V	0	10.86
---	--	------	------
mPD		0.034	0.66
		nPD:	0.05
		N. weight:	1.1
		Sc. PD:	-0.012
		Sc. rank:	14950.0

,

PB1			
Pos.	685	obs:	exp:
tta	L	0	4563.00
ttg	L	0	8129.00
ctt	L	39285	8226.00
ctc	L	32	5766.00
cta	L	1	6038.00
ctg	L	3	6599.00
att	I	1	0.35
atc	I	0	0.26
ata	I	0	0.39
---	--	------	------
mPD		0.0019	1.2
		nPD:	0.
		N. weight:	1.7
		Sc. PD:	-0.1
		Sc. rank:	-49832.0

,

PB1			
Pos.	686	obs:	exp:
gaa	E	325	23183.00
gag	E	38997	16139.00
---	--	------	------
mPD		0.016	0.48
		nPD:	0.03
		N. weight:	0.92
		Sc. PD:	-0.026
		Sc. rank:	6950.8

,

PB1			
Pos.	687	obs:	exp:
gat	D	39194	22227.00
gac	D	127	17094.00
gaa	E	1	0.59
gag	E	0	0.41
---	--	------	------
mPD		0.0065	0.49
		nPD:	0.01
		N. weight:	0.6
		Sc. PD:	-0.029
		Sc. rank:	-5772.1

,

PB1			
Pos.	688	obs:	exp:
gtt	V	0	0.50
gtc	V	1	0.41
gta	V	1	0.43
gtg	V	0	0.66
gat	D	2	1.13
gac	D	0	0.87
gaa	E	39174	23180.00
gag	E	143	16137.00
ggt	G	0	0.13
ggc	G	0	0.14
gga	G	1	0.46
ggg	G	0	0.26
---	--	------	------
mPD		0.0076	0.48
		nPD:	0.02
		N. weight:	0.55
		Sc. PD:	-0.025
		Sc. rank:	-3562.4

,

PB1			
Pos.	689	obs:	exp:
tta	L	0	0.81
ttg	L	0	1.45
ctt	L	0	1.46
ctc	L	0	1.03
cta	L	0	1.07
ctg	L	7	1.17
cct	P	0	0.33
ccc	P	1	0.19
cca	P	0	0.34
ccg	P	0	0.14
cat	H	6	3.95
cac	H	1	3.05
caa	Q	15627	24025.00
cag	Q	23676	15278.00
cgt	R	0	0.06
cgc	R	0	0.11
cga	R	1	0.27
cgg	R	2	0.26
aaa	K	0	0.66
aag	K	1	0.34
aga	R	0	1.47
agg	R	0	0.83
---	--	------	------
mPD		0.48	0.48
		nPD:	1.01
		N. weight:	0.1
		Sc. PD:	0.095
		Sc. rank:	3180.4

,

PB1			
Pos.	690	obs:	exp:
cct	P	0	0.33
ccc	P	0	0.19
cca	P	0	0.34
ccg	P	1	0.14
cat	H	0	0.56
cac	H	1	0.44
att	I	0	1.76
atc	I	0	1.31
ata	I	5	1.93
atg	M	39310	39310.00
act	T	0	0.31
acc	T	0	0.18
aca	T	0	0.43
acg	T	1	0.08
gtt	V	0	1.00
gtc	V	0	0.82
gta	V	0	0.87
gtg	V	4	1.32
---	--	------	------
mPD		0.00076	0.00099
		nPD:	0.77
		N. weight:	0.0004
		Sc. PD:	0.00028
		Sc. rank:	12.5

,

PB1			
Pos.	691	obs:	exp:
tat	Y	353	20725.00
tac	Y	38966	18594.00
cct	P	0	0.33
ccc	P	1	0.19
cca	P	0	0.34
ccg	P	0	0.14
cat	H	0	0.56
cac	H	1	0.44
gat	D	0	0.57
gac	D	1	0.43
---	--	------	------
mPD		0.018	0.50
		nPD:	0.04
		N. weight:	0.76
		Sc. PD:	-0.02
		Sc. rank:	6672.6

,

PB1			
Pos.	692	obs:	exp:
cct	P	0	0.33
ccc	P	0	0.19
cca	P	0	0.34
ccg	P	1	0.14
cat	H	5	2.82
cac	H	0	2.18
caa	Q	295	24030.00
cag	Q	39016	15281.00
cgt	R	0	0.02
cgc	R	1	0.04
cga	R	0	0.09
cgg	R	0	0.09
aaa	K	3	2.63
aag	K	1	1.37
aga	R	0	0.49
agg	R	0	0.28
---	--	------	------
mPD		0.016	0.48
		nPD:	0.03
		N. weight:	0.98
		Sc. PD:	-0.028
		Sc. rank:	6907.3

,

PB1			
Pos.	693	obs:	exp:
cgt	R	0	0.14
cgc	R	0	0.25
cga	R	0	0.64
cgg	R	0	0.60
aat	N	1	0.56
aac	N	0	0.44
aaa	K	6335	25846.00
aag	K	32978	13467.00
aga	R	2	3.43
agg	R	5	1.94
---	-	1	1.00
---	--	------	------
mPD		0.27	0.45
		nPD:	0.6
		N. weight:	0.58
		Sc. PD:	0.3
		Sc. rank:	17419.0

,

PB1			
Pos.	694	obs:	exp:
tct	S	0	0.32
tcc	S	0	0.30
tca	S	0	0.54
tcg	S	0	0.08
tgt	C	289	15366.00
tgc	C	39030	23953.00
agt	S	0	0.35
agc	S	2	0.41
---	-	1	1.00
---	--	------	------
mPD		0.015	0.48
		nPD:	0.03
		N. weight:	0.5
		Sc. PD:	-0.015
		Sc. rank:	3025.7

,

PB1

Pos.	695	obs:	exp:
tat	Y	0	0.53
tac	Y	1	0.47
tgt	C	183	15366.00
tgc	C	39137	23954.00
cgt	R	0	0.02
cgc	R	1	0.04
cga	R	0	0.09
cgg	R	0	0.09
aga	R	0	0.49
agg	R	0	0.28
---	--	------	------
mPD		0.0094	0.48
		nPD:	0.02
		N. weight:	0.51
		Sc. PD:	-0.021
		Sc. rank:	-1488.3

,

PB1

Pos.	696	obs:	exp:
tct	S	0	5.33
tcc	S	0	4.93
tca	S	0	8.87
tcg	S	0	1.30
tat	Y	0	0.53
tac	Y	1	0.47
cat	H	0	0.56
cac	H	1	0.44
act	T	0	12.11
acc	T	39	6.90
aca	T	0	16.88
acg	T	0	3.11
aat	N	605	21954.00
aac	N	38636	17287.00
aaa	K	1	0.66
aag	K	0	0.34
agt	S	0	5.77
agc	S	33	6.81
gat	D	0	3.39
gac	D	6	2.61
---	--	------	------
mPD		0.034	0.50
		nPD:	0.07
		N. weight:	0.81
		Sc. PD:	0.0051
		Sc. rank:	14353.0

,

PB1

Pos.	697	obs:	exp:
tta	L	166	4562.00
ttg	L	38753	8128.00
tct	S	0	0.16
tcc	S	0	0.15
tca	S	0	0.27
tcg	S	1	0.04
ctt	L	1	8225.00
ctc	L	0	5765.00
cta	L	11	6037.00
ctg	L	385	6598.00
atg	M	4	4.00
agt	S	0	0.17
agc	S	0	0.21
gtt	V	0	0.25
gtc	V	0	0.20
gta	V	0	0.22
gtg	V	1	0.33
---	--	------	------
mPD		0.029	1.2
		nPD:	0.03
		N. weight:	1.6
		Sc. PD:	-0.06
		Sc. rank:	4461.4

,

PB1

Pos.	698	obs:	exp:
ttt	F	203	19127.00
ttc	F	39119	20195.00
---	--	------	------
mPD		0.010	0.50
		nPD:	0.02
		N. weight:	0.7
		Sc. PD:	-0.028
		Sc. rank:	-1360.8

,

PB1

Pos.	699	obs:	exp:
aaa	K	0	1.31
aag	K	2	0.69
gat	D	2	1.13
gac	D	0	0.87
gaa	E	134	23179.00
gag	E	39181	16136.00
ggt	G	0	0.40
ggc	G	0	0.42
gga	G	0	1.38
ggg	G	3	0.79
---	--	------	------
mPD		0.0071	0.48
		nPD:	0.01
		N. weight:	0.95
		Sc. PD:	-0.044
		Sc. rank:	-7039.1

,

PB1

Pos.	700	obs:	exp:
cgt	R	0	0.56
cgc	R	0	1.01
cga	R	0	2.55
cgg	R	0	2.40
aat	N	10	7.27
aac	N	3	5.73
aaa	K	39235	25823.00
aag	K	44	13456.00
aga	R	28	13.73
agg	R	0	7.74
gaa	E	2	1.18
gag	E	0	0.82
---	--	------	------
mPD		0.0044	0.45
		nPD:	0.01
		N. weight:	0.45
		Sc. PD:	-0.023
		Sc. rank:	-6072.7

,

PB1

Pos.	701	obs:	exp:
ttt	F	39117	19127.00
ttc	F	205	20195.00
---	--	------	------
mPD		0.010	0.50
		nPD:	0.02
		N. weight:	0.75
		Sc. PD:	-0.031
		Sc. rank:	-1344.1

,

PB1

Pos.	702	obs:	exp:
ttt	F	141	19127.00
ttc	F	39181	20195.00
---	--	------	------
mPD		0.0071	0.50
		nPD:	0.01
		N. weight:	0.71
		Sc. PD:	-0.033
		Sc. rank:	-5604.1

,

PB1

Pos.	703	obs:	exp:
tct	S	1	0.16
tcc	S	0	0.15
tca	S	0	0.27
tcg	S	0	0.04
cct	P	38982	13103.00
ccc	P	332	7371.00
cca	P	2	13284.00
ccg	P	3	5561.00
cat	H	2	1.13
cac	H	0	0.87
agt	S	0	0.17
agc	S	0	0.21
---	--	------	------
mPD		0.017	0.72
		nPD:	0.02
		N. weight:	1.2
		Sc. PD:	-0.044
		Sc. rank:	1283.1

,

PB1

Pos.	704	obs:	exp:
tct	S	0	6345.00
tcc	S	0	5876.00
tca	S	0	10568.00
tcg	S	0	1548.00
tgt	C	1	0.39
tgc	C	0	0.61
act	T	0	0.31
acc	T	0	0.18
aca	T	1	0.43
acg	T	0	0.08
agt	S	39088	6871.00
agc	S	232	8112.00
---	--	------	------
mPD		0.012	1.6
		nPD:	0.01
		N. weight:	1.9
		Sc. PD:	-0.1
		Sc. rank:	-34391.0

,

PB1

Pos.	705	obs:	exp:
tct	S	0	6345.00
tcc	S	0	5876.00
tca	S	0	10568.00
tcg	S	0	1548.00
tgt	C	1	0.39
tgc	C	0	0.61
att	I	2	0.70
atc	I	0	0.53
ata	I	0	0.77
agt	S	39123	6871.00
agc	S	196	8111.00
---	--	------	------
mPD		0.010	1.6
		nPD:	0.01
		N. weight:	1.9
		Sc. PD:	-0.1
		Sc. rank:	-38518.0

,

PB1

Pos.	706	obs:	exp:
tta	L	7	0.93
ttg	L	0	1.65
tct	S	15	6343.00
tcc	S	4	5874.00
tca	S	39102	10565.00
tcg	S	186	1547.00
ctt	L	0	1.67
ctc	L	0	1.17
cta	L	1	1.23
ctg	L	0	1.34
cct	P	0	0.33
ccc	P	0	0.19
cca	P	1	0.34
ccg	P	0	0.14
act	T	0	1.86
acc	T	0	1.06
aca	T	6	2.60
acg	T	0	0.48
agt	S	0	6869.00
agc	S	0	8109.00
---	--	------	------
mPD		0.011	1.6
		nPD:	0.01
		N. weight:	1.4
		Sc. PD:	-0.077
		Sc. rank:	-27172.0

,

PB1

Pos.	707	obs:	exp:
tat	Y	38838	20727.00
tac	Y	483	18594.00
gat	D	1	0.57
gac	D	0	0.43
---	--	------	------
mPD		0.024	0.50
		nPD:	0.05
		N. weight:	0.63
		Sc. PD:	-0.0085
		Sc. rank:	8474.5

,

PB1

Pos.	708	obs:	exp:
cgt	R	0	779.10
cgc	R	0	1414.00
cga	R	0	3585.00
cgg	R	3	3374.00
atg	M	3	3.00
aaa	K	0	17.09
aag	K	26	8.91
aga	R	514	19272.00
agg	R	38775	10868.00
---	-	1	1.00
---	--	------	------
mPD		0.027	0.89
		nPD:	0.03
		N. weight:	1.3
		Sc. PD:	-0.041
		Sc. rank:	8160.5

,

PB1

Pos.	709	obs:	exp:
cgt	R	0	779.50
cgc	R	0	1415.00
cga	R	6	3587.00
cgg	R	0	3376.00
att	I	0	0.35
atc	I	0	0.26
ata	I	1	0.39
aaa	K	4	2.63
aag	K	0	1.37
aga	R	39169	19283.00
agg	R	139	10874.00
ggt	G	0	0.40
ggc	G	0	0.42
gga	G	3	1.38
ggg	G	0	0.79
---	--	------	------
mPD		0.0078	0.89
		nPD:	0.01
		N. weight:	0.76
		Sc. PD:	-0.04
		Sc. rank:	-11572.0

,

PB1

Pos.	710	obs:	exp:
tct	S	0	0.32
tcc	S	0	0.30
tca	S	0	0.54
tcg	S	2	0.08
cct	P	630	13103.00
ccc	P	1	7371.00
cca	P	1515	13284.00
ccg	P	37174	5561.00
agt	S	0	0.35
agc	S	0	0.41
---	--	------	------
mPD		0.10	0.72
		nPD:	0.15
		N. weight:	1.8
		Sc. PD:	0.15
		Sc. rank:	44693.0

,

PB1

Pos.	711	obs:	exp:
att	I	38621	13565.00
atc	I	21	10154.00
ata	I	0	14923.00
gtt	V	677	170.30
gtc	V	3	138.60
gta	V	0	147.40
gtg	V	0	223.70
---	--	------	------
mPD		0.035	0.70
		nPD:	0.05
		N. weight:	1.1
		Sc. PD:	-0.013
		Sc. rank:	15943.0

,

PB1

Pos.	712	obs:	exp:
ggt	G	6	5294.00
ggc	G	13	5488.00
gga	G	38766	18152.00
ggg	G	537	10388.00
---	--	------	------
mPD		0.028	0.68
		nPD:	0.04
		N. weight:	0.77
		Sc. PD:	-0.016
		Sc. rank:	7958.0

,

PB1

Pos.	713	obs:	exp:
att	I	39183	13801.00
atc	I	128	10330.00
ata	I	4	15183.00
atg	M	2	2.00
act	T	1	0.31
acc	T	0	0.18
aca	T	0	0.43
acg	T	0	0.08
gtt	V	4	1.00
gtc	V	0	0.82
gta	V	0	0.87
gtg	V	0	1.32
---	--	------	------
mPD		0.0070	0.66
		nPD:	0.01
		N. weight:	1.1
		Sc. PD:	-0.057
		Sc. rank:	-13835.0

,

PB1

Pos.	714	obs:	exp:
tct	S	39150	6345.00
tcc	S	142	5876.00
tca	S	15	10569.00
tcg	S	15	1548.00
agt	S	0	6872.00
agc	S	0	8112.00
---	--	------	------
mPD		0.0087	1.6
		nPD:	0.01
		N. weight:	2.
		Sc. PD:	-0.11
		Sc. rank:	-42566.0

,

PB1

Pos.	715	obs:	exp:
tct	S	0	6344.00
tcc	S	0	5875.00
tca	S	0	10566.00
tcg	S	0	1547.00
tgt	C	0	0.78
tgc	C	2	1.22
cgt	R	0	0.02
cgc	R	0	0.04
cga	R	0	0.09
cgg	R	0	0.09
act	T	0	1.24
acc	T	4	0.71
aca	T	0	1.73
acg	T	0	0.32
aat	N	0	2.24
aac	N	4	1.76
agt	S	63	6870.00
agc	S	39248	8110.00
aga	R	1	0.49
agg	R	0	0.28
---	--	------	------
mPD		0.0038	1.6
		nPD:	0.
		N. weight:	1.7
		Sc. PD:	-0.1
		Sc. rank:	-47887.0

,

PB1

Pos.	716	obs:	exp:
tta	L	0	0.23
ttg	L	1	0.41
ctt	L	0	0.42
ctc	L	0	0.29
cta	L	0	0.31
ctg	L	1	0.34
att	I	0	0.35
atc	I	0	0.26
ata	I	1	0.39
atg	M	39319	39319.00
---	--	------	------
mPD		0.00015	0.00022
		nPD:	0.71
		N. weight:	0.0001
		Sc. PD:	0.000063
		Sc. rank:	3.1

,

PB1

Pos.	717	obs:	exp:
tta	L	0	0.12
ttg	L	1	0.21
ctt	L	0	0.21
ctc	L	0	0.15
cta	L	0	0.15
ctg	L	0	0.17
atg	M	47	47.00
gtt	V	9	9709.00
gtc	V	0	7898.00
gta	V	238	8401.00
gtg	V	38514	12753.00
gct	A	0	138.70
gcc	A	0	127.20
gca	A	4	205.90
gcg	A	505	37.21
gaa	E	0	0.59
gag	E	1	0.41
ggt	G	0	0.40
ggc	G	0	0.42
gga	G	0	1.38
ggg	G	3	0.79
---	--	------	------
mPD		0.041	0.77
		nPD:	0.05
		N. weight:	1.2
		Sc. PD:	-0.011
		Sc. rank:	17766.0

,

PB1

Pos.	718	obs:	exp:
gat	D	24	13.57
gac	D	0	10.43
gaa	E	31	23166.00
gag	E	39262	16127.00
ggt	G	0	0.67
ggc	G	0	0.70
gga	G	0	2.31
ggg	G	5	1.32
---	--	------	------
mPD		0.0030	0.48
		nPD:	0.01
		N. weight:	0.97
		Sc. PD:	-0.053
		Sc. rank:	-19673.0

,

PB1			
Pos.	719	obs:	exp:
gct	A	53	10717.00
gcc	A	38650	9824.00
gca	A	619	15906.00
gcg	A	0	2875.00
---	--	------	------
mPD		0.034	0.69
		nPD:	0.05
		N. weight:	1.4
		Sc. PD:	-0.019
		Sc. rank:	18896.0

,

PB1			
Pos.	720	obs:	exp:
att	I	0	0.35
atc	I	0	0.26
ata	I	1	0.39
atg	M	39320	39320.00
act	T	0	0.31
acc	T	0	0.18
aca	T	0	0.43
acg	T	1	0.08
---	--	------	------
mPD		0.00010	0.00015
		nPD:	0.68
		N. weight:	0.000097
		Sc. PD:	0.000059
		Sc. rank:	3.0

,

PB1			
Pos.	721	obs:	exp:
tta	L	3	0.58
ttg	L	2	1.03
ctt	L	0	1.05
ctc	L	0	0.73
cta	L	0	0.77
ctg	L	0	0.84
att	I	0	12.29
atc	I	0	9.20
ata	I	35	13.52
atg	M	27	27.00
gtt	V	59	9831.00
gtc	V	0	7997.00
gta	V	16747	8507.00
gtg	V	22444	12914.00
gct	A	0	1.36
gcc	A	0	1.25
gca	A	5	2.02
gcg	A	0	0.37
---	--	------	------
mPD		0.49	0.74
		nPD:	0.66
		N. weight:	0.66
		Sc. PD:	0.39
		Sc. rank:	19914.0

,

PB1			
Pos.	722	obs:	exp:
tct	S	39189	6345.00
tcc	S	124	5876.00
tca	S	5	10569.00
tcg	S	3	1548.00
tat	Y	1	0.53
tac	Y	0	0.47
agt	S	0	6871.00
agc	S	0	8112.00
---	--	------	------
mPD		0.0067	1.6
		nPD:	0.
		N. weight:	2.
		Sc. PD:	-0.11
		Sc. rank:	-48494.0

,

PB1			
Pos.	723	obs:	exp:
cgt	R	0	778.70
cgc	R	0	1413.00
cga	R	0	3584.00
cgg	R	1	3373.00
act	T	0	0.31
acc	T	0	0.18
aca	T	0	0.43
acg	T	1	0.08
aaa	K	0	29.58
aag	K	45	15.42
aga	R	71	19264.00
agg	R	39204	10864.00
---	--	------	------
mPD		0.0060	0.89
		nPD:	0.01
		N. weight:	1.4
		Sc. PD:	-0.076
		Sc. rank:	-27078.0

,

PB1			
Pos.	724	obs:	exp:
tct	S	0	7.26
tcc	S	45	6.72
tca	S	0	12.10
tcg	S	0	1.77
act	T	0	0.62
acc	T	2	0.35
aca	T	0	0.87
acg	T	0	0.16
agt	S	0	7.86
agc	S	0	9.28
gct	A	52	10702.00
gcc	A	39213	9810.00
gca	A	2	15884.00
gcg	A	0	2870.00
ggt	G	0	1.08
ggc	G	8	1.12
gga	G	0	3.69
ggg	G	0	2.11
---	--	------	------
mPD		0.0055	0.70
		nPD:	0.01
		N. weight:	1.5
		Sc. PD:	-0.08
		Sc. rank:	-25391.0

,

PB1			
Pos.	725	obs:	exp:
tta	L	0	0.35
ttg	L	0	0.62
tgg	W	1	1.00
ctt	L	0	0.63
ctc	L	0	0.44
cta	L	0	0.46
ctg	L	3	0.50
caa	Q	1	29.95
cag	Q	48	19.05
cgt	R	62	778.60
cgc	R	0	1413.00
cga	R	662	3583.00
cgg	R	38437	3372.00
aga	R	0	19261.00
agg	R	108	10862.00
---	--	------	------
mPD		0.044	0.89
		nPD:	0.05
		N. weight:	2.6
		Sc. PD:	-0.031
		Sc. rank:	34919.0

,

PB1			
Pos.	726	obs:	exp:
att	I	39279	13803.00
atc	I	39	10332.00
ata	I	2	15185.00
act	T	2	0.62
acc	T	0	0.35
aca	T	0	0.87
acg	T	0	0.16
---	--	------	------
mPD		0.0022	0.66
		nPD:	0.
		N. weight:	1.1
		Sc. PD:	-0.066
		Sc. rank:	-29270.0

,

PB1			
Pos.	727	obs:	exp:
aat	N	1	0.56
aac	N	0	0.44
gct	A	1	0.27
gcc	A	0	0.25
gca	A	0	0.40
gcg	A	0	0.07
gat	D	39304	22226.00
gac	D	16	17094.00
---	--	------	------
mPD		0.00092	0.49
		nPD:	0.
		N. weight:	0.62
		Sc. PD:	-0.037
		Sc. rank:	-17928.0

,

PB1			
Pos.	728	obs:	exp:
tct	S	0	0.97
tcc	S	6	0.90
tca	S	0	1.61
tcg	S	0	0.24
act	T	0	2.17
acc	T	7	1.24
aca	T	0	3.03
acg	T	0	0.56
agt	S	0	1.05
agc	S	0	1.24
gtt	V	0	1.50
gtc	V	6	1.22
gta	V	0	1.30
gtg	V	0	1.97
gct	A	35	10712.00
gcc	A	39256	9819.00
gca	A	12	15898.00
gcg	A	0	2873.00
---	--	------	------
mPD		0.0034	0.70
		nPD:	0.
		N. weight:	1.5
		Sc. PD:	-0.085
		Sc. rank:	-34974.0

,

PB1			
Pos.	729	obs:	exp:
tct	S	0	0.16
tcc	S	0	0.15
tca	S	0	0.27
tcg	S	0	0.04
cgt	R	0	779.60
cgc	R	0	1415.00
cga	R	8	3588.00
cgg	R	3	3376.00
aaa	K	1	0.66
aag	K	0	0.34
agt	S	1	0.17
agc	S	0	0.21
aga	R	39044	19286.00
agg	R	265	10876.00
---	--	------	------
mPD		0.014	0.89
		nPD:	0.02
		N. weight:	0.74
		Sc. PD:	-0.034
		Sc. rank:	-4347.1

,

PB1			
Pos.	730	obs:	exp:
tct	S	0	0.16
tcc	S	0	0.15
tca	S	0	0.27
tcg	S	0	0.04
att	I	39205	13782.00
atc	I	22	10316.00
ata	I	33	15162.00
act	T	2	0.93
acc	T	1	0.53
aca	T	0	1.30
acg	T	0	0.24
agt	S	1	0.17
agc	S	0	0.21
gtt	V	23	14.53
gtc	V	35	11.82
gta	V	0	12.57
gtg	V	0	19.08
---	--	------	------
mPD		0.0078	0.66
		nPD:	0.01
		N. weight:	1.1
		Sc. PD:	-0.056
		Sc. rank:	-12770.0

,

PB1

Pos.	731	obs:	exp:
tat	Y	0	0.53
tac	Y	1	0.47
aat	N	0	6.15
aac	N	11	4.85
gat	D	90	22206.00
gac	D	39195	17079.00
gaa	E	13	7.66
gag	E	0	5.34
ggt	G	0	1.62
ggc	G	12	1.67
gga	G	0	5.54
ggg	G	0	3.17
---	--	------	------
mPD		0.0064	0.49
		nPD:	0.01
		N. weight:	0.9
		Sc. PD:	-0.043
		Sc. rank:	-8747.7

,

PB1

Pos.	732	obs:	exp:
ttt	F	401	19118.00
ttc	F	38902	20185.00
tta	L	3	1.04
ttg	L	0	1.86
tct	S	0	1.45
tcc	S	9	1.34
tca	S	0	2.42
tcg	S	0	0.35
tat	Y	0	0.53
tac	Y	1	0.47
ctt	L	0	1.88
ctc	L	6	1.32
cta	L	0	1.38
ctg	L	0	1.51
agt	S	0	1.57
agc	S	0	1.86
---	--	------	------
mPD		0.021	0.50
		nPD:	0.04
		N. weight:	0.67
		Sc. PD:	-0.013
		Sc. rank:	7177.6

,

PB1

Pos.	733	obs:	exp:
gat	D	6	3.39
gac	D	0	2.61
gaa	E	941	23179.00
gag	E	38374	16136.00
ggt	G	0	0.13
ggc	G	0	0.14
gga	G	0	0.46
ggg	G	1	0.26
---	--	------	------
mPD		0.047	0.48
		nPD:	0.1
		N. weight:	0.84
		Sc. PD:	0.029
		Sc. rank:	18239.0

,

PB1

Pos.	734	obs:	exp:
ttt	F	1	0.97
ttc	F	1	1.03
tct	S	34809	6344.00
tcc	S	4349	5875.00
tca	S	156	10567.00
tcg	S	2	1548.00
act	T	1	0.31
acc	T	0	0.18
aca	T	0	0.43
acg	T	0	0.08
agt	S	0	6871.00
agc	S	0	8111.00
gct	A	3	0.82
gcc	A	0	0.75
gca	A	0	1.21
gcg	A	0	0.22
---	--	------	------
mPD		0.20	1.6
		nPD:	0.13
		N. weight:	1.6
		Sc. PD:	0.098
		Sc. rank:	37399.0

,

PB1

Pos.	735	obs:	exp:
cgt	R	0	0.24
cgc	R	0	0.43
cga	R	0	1.09
cgg	R	0	1.03
aga	R	11	5.89
agg	R	1	3.32
gtt	V	0	0.25
gtc	V	0	0.20
gta	V	1	0.22
gtg	V	0	0.33
gaa	E	10	5.90
gag	E	0	4.10
ggt	G	192	5291.00
ggc	G	0	5485.00
gga	G	38978	18141.00
ggg	G	129	10382.00
---	--	------	------
mPD		0.017	0.68
		nPD:	0.03
		N. weight:	0.8
		Sc. PD:	-0.029
		Sc. rank:	2498.1

,

PB1

Pos.	736	obs:	exp:
cgt	R	0	778.20
cgc	R	0	1412.00
cga	R	3	3581.00
cgg	R	1062	3370.00
aaa	K	1	46.02
aag	K	69	23.98
aga	R	247	19251.00
agg	R	37937	10856.00
ggt	G	0	0.40
ggc	G	0	0.42
gga	G	0	1.38
ggg	G	3	0.79
---	--	------	------
mPD		0.069	0.89
		nPD:	0.08
		N. weight:	1.3
		Sc. PD:	0.019
		Sc. rank:	24970.0

,

PB1

Pos.	737	obs:	exp:
tta	L	0	0.35
ttg	L	0	0.62
tct	S	0	0.32
tcc	S	0	0.30
tca	S	0	0.54
tcg	S	0	0.08
ctt	L	3	0.63
ctc	L	0	0.44
cta	L	0	0.46
ctg	L	0	0.50
att	I	38374	13785.00
atc	I	117	10318.00
ata	I	778	15166.00
atg	M	13	13.00
act	T	5	1.86
acc	T	1	1.06
aca	T	0	2.60
acg	T	0	0.48
agt	S	2	0.35
agc	S	0	0.41
gtt	V	29	7.26
gtc	V	0	5.91
gta	V	0	6.29
gtg	V	0	9.54
---	--	------	------
mPD		0.047	0.66
		nPD:	0.07
		N. weight:	1.
		Sc. PD:	0.009
		Sc. rank:	19064.0

,

PB1

Pos.	738	obs:	exp:
caa	Q	0	4.89
cag	Q	8	3.11
cgt	R	0	0.63
cgc	R	0	1.15
cga	R	0	2.92
cgg	R	0	2.75
atg	M	1	1.00
aat	N	20	11.19
aac	N	0	8.81
aaa	K	239	25801.00
aag	K	39006	13444.00
aga	R	0	15.70
agg	R	32	8.85
gaa	E	0	8.25
gag	E	14	5.75
ggt	G	0	0.27
ggc	G	0	0.28
gga	G	0	0.92
ggg	G	2	0.53
---	--	------	------
mPD		0.016	0.45
		nPD:	0.04
		N. weight:	1.1
		Sc. PD:	-0.03
		Sc. rank:	9459.3

,

PB1

Pos.	739	obs:	exp:
cgt	R	0	1.57
cgc	R	0	2.84
cga	R	0	7.21
cgg	R	0	6.78
act	T	0	0.62
acc	T	0	0.35
aca	T	1	0.87
acg	T	1	0.16
aat	N	5	2.80
aac	N	0	2.20
aaa	K	2966	25795.00
aag	K	36270	13441.00
aga	R	53	38.75
agg	R	26	21.85
---	--	------	------
mPD		0.15	0.46
		nPD:	0.32
		N. weight:	0.83
		Sc. PD:	0.21
		Sc. rank:	23437.0

,

PB1

Pos.	740	obs:	exp:
gtt	V	0	0.25
gtc	V	0	0.20
gta	V	1	0.22
gtg	V	0	0.33
gat	D	5	3.39
gac	D	1	2.61
gaa	E	38513	23167.00
gag	E	782	16128.00
ggt	G	0	2.69
ggc	G	0	2.79
gga	G	20	9.23
ggg	G	0	5.28
---	--	------	------
mPD		0.040	0.49
		nPD:	0.08
		N. weight:	0.48
		Sc. PD:	0.0097
		Sc. rank:	9851.1

,

PB1

Pos.	741	obs:	exp:
aaa	K	0	4.60
aag	K	7	2.40
gtt	V	0	0.25
gtc	V	0	0.20
gta	V	0	0.22
gtg	V	1	0.33
gct	A	0	0.27
gcc	A	0	0.25
gca	A	0	0.40
gcg	A	1	0.07
gat	D	7	3.96
gac	D	0	3.04
gaa	E	313	23173.00
gag	E	38992	16132.00
ggt	G	0	0.13
ggc	G	0	0.14
gga	G	0	0.46
ggg	G	1	0.26
---	--	------	------
mPD		0.017	0.48
		nPD:	0.03
		N. weight:	0.92
		Sc. PD:	-0.025
		Sc. rank:	7086.7

,

PB1

Pos.	742	obs:	exp:
ttt	F	818	19110.00
ttc	F	38468	20176.00
tta	L	7	3.48
ttg	L	0	6.20
tct	S	0	0.16
tcc	S	1	0.15
tca	S	0	0.27
tcg	S	0	0.04
tat	Y	0	0.53
tac	Y	1	0.47
tgt	C	0	0.39
tgc	C	1	0.61
ctt	L	0	6.28
ctc	L	23	4.40
cta	L	0	4.61
ctg	L	0	5.03
att	I	0	0.35
atc	I	1	0.26
ata	I	0	0.39
agt	S	0	0.17
agc	S	0	0.21
gtt	V	0	0.50
gtc	V	2	0.41
gta	V	0	0.43
gtg	V	0	0.66
---	--	------	------
mPD		0.043	0.50
		nPD:	0.08
		N. weight:	0.62
		Sc. PD:	0.014
		Sc. rank:	12865.0

,

PB1			
Pos.	743	obs:	exp:
ttt	F	4	1.95
ttc	F	0	2.05
tct	S	39091	6335.00
tcc	S	146	5867.00
tca	S	18	10553.00
tcg	S	6	1545.00
tat	Y	7	3.69
tac	Y	0	3.31
act	T	9	3.42
acc	T	2	1.94
aca	T	0	4.76
acg	T	0	0.88
agt	S	0	6861.00
agc	S	0	8099.00
gct	A	5	10.63
gcc	A	34	9.74
gca	A	0	15.78
gcg	A	0	2.85
---	--	------	------
mPD		0.014	1.6
		nPD:	0.01
		N. weight:	2.
		Sc. PD:	-0.1
		Sc. rank:	-31272.0

,

PB1			
Pos.	744	obs:	exp:
gat	D	11	6.22
gac	D	0	4.78
gaa	E	11	23173.00
gag	E	39295	16133.00
ggt	G	0	0.54
ggc	G	0	0.56
gga	G	0	1.85
ggg	G	4	1.06
---	-	1	1.00
---	--	------	------
mPD		0.0013	0.48
		nPD:	0.
		N. weight:	0.97
		Sc. PD:	-0.057
		Sc. rank:	-26092.0

,

PB1			
Pos.	745	obs:	exp:
att	I	9	13798.00
atc	I	39295	10328.00
ata	I	1	15179.00
act	T	0	3.42
acc	T	11	1.94
aca	T	0	4.76
acg	T	0	0.88
gtt	V	0	1.50
gtc	V	6	1.22
gta	V	0	1.30
gtg	V	0	1.97
---	--	------	------
mPD		0.0014	0.66
		nPD:	0.
		N. weight:	1.5
		Sc. PD:	-0.086
		Sc. rank:	-41679.0

,

PB1			
Pos.	746	obs:	exp:
tta	L	0	0.93
ttg	L	7	1.65
ctt	L	0	1.67
ctc	L	0	1.17
cta	L	0	1.23
ctg	L	1	1.34
att	I	5	55.11
atc	I	0	41.25
ata	I	152	60.63
atg	M	39125	39125.00
act	T	0	4.97
acc	T	0	2.83
aca	T	0	6.92
acg	T	16	1.28
aat	N	1	0.56
aac	N	0	0.44
aaa	K	0	1.31
aag	K	2	0.69
gtt	V	0	3.26
gtc	V	0	2.65
gta	V	0	2.82
gtg	V	13	4.28
---	--	------	------
mPD		0.010	0.012
		nPD:	0.87
		N. weight:	0.0055
		Sc. PD:	0.0043
		Sc. rank:	170.8

,

PB1			
Pos.	747	obs:	exp:
cgt	R	0	18.74
cgc	R	0	34.00
cga	R	0	86.22
cgg	R	0	81.15
act	T	0	0.31
acc	T	0	0.18
aca	T	0	0.43
acg	T	1	0.08
aat	N	1	0.56
aac	N	0	0.44
aaa	K	27	25228.00
aag	K	38347	13146.00
aga	R	1	463.50
agg	R	944	261.40
gaa	E	0	0.59
gag	E	1	0.41
---	--	------	------
mPD		0.048	0.51
		nPD:	0.09
		N. weight:	1.2
		Sc. PD:	0.037
		Sc. rank:	25130.0

,

PB1			
Pos.	748	obs:	exp:
att	I	0	13793.00
atc	I	39287	10325.00
ata	I	6	15175.00
act	T	0	7.45
acc	T	24	4.24
aca	T	0	10.39
acg	T	0	1.92
gtt	V	0	1.25
gtc	V	5	1.02
gta	V	0	1.08
gtg	V	0	1.65
---	--	------	------
mPD		0.0018	0.66
		nPD:	0.
		N. weight:	1.5
		Sc. PD:	-0.085
		Sc. rank:	-39766.0

,

PB1			
Pos.	749	obs:	exp:
tgt	C	39162	15367.00
tgc	C	160	23955.00
---	--	------	------
mPD		0.0081	0.48
		nPD:	0.02
		N. weight:	1.
		Sc. PD:	-0.044
		Sc. rank:	-4623.1

,

PB1			
Pos.	750	obs:	exp:
ttt	F	0	15.08
ttc	F	31	15.92
tct	S	793	6338.00
tcc	S	38465	5869.00
tca	S	18	10557.00
tcg	S	0	1546.00
tat	Y	0	6.33
tac	Y	12	5.67
act	T	0	0.62
acc	T	2	0.35
aca	T	0	0.87
acg	T	0	0.16
agt	S	0	6864.00
agc	S	0	8103.00
gct	A	0	0.27
gcc	A	1	0.25
gca	A	0	0.40
gcg	A	0	0.07
---	--	------	------
mPD		0.043	1.6
		nPD:	0.03
		N. weight:	2.
		Sc. PD:	-0.07
		Sc. rank:	7009.6

,

PB1			
Pos.	751	obs:	exp:
act	T	0	12208.00
acc	T	39310	6950.00
aca	T	0	17012.00
acg	T	0	3140.00
gct	A	0	3.27
gcc	A	12	3.00
gca	A	0	4.85
gcg	A	0	0.88
---	--	------	------
mPD		0.00061	0.68
		nPD:	0.
		N. weight:	1.9
		Sc. PD:	-0.11
		Sc. rank:	-57443.0

,

PB1			
Pos.	752	obs:	exp:
att	I	39315	13803.00
atc	I	2	10332.00
ata	I	3	15185.00
act	T	2	0.62
acc	T	0	0.35
aca	T	0	0.87
acg	T	0	0.16
---	--	------	------
mPD		0.00036	0.66
		nPD:	0.
		N. weight:	1.1
		Sc. PD:	-0.069
		Sc. rank:	-35246.0

,

PB1			
Pos.	753	obs:	exp:
aaa	K	16	10.52
aag	K	0	5.48
gaa	E	39300	23171.00
gag	E	2	16131.00
ggt	G	0	0.54
ggc	G	0	0.56
gga	G	4	1.85
ggg	G	0	1.06
---	--	------	------
mPD		0.0011	0.48
		nPD:	0.
		N. weight:	0.58
		Sc. PD:	-0.034
		Sc. rank:	-16106.0

,

PB1			
Pos.	754	obs:	exp:
cgt	R	0	0.04
cgc	R	0	0.07
cga	R	0	0.18
cgg	R	0	0.17
aaa	K	19	12.49
aag	K	0	6.51
aga	R	2	0.98
agg	R	0	0.55
gtt	V	0	1.25
gtc	V	0	1.02
gta	V	5	1.08
gtg	V	0	1.65
gct	A	0	0.27
gcc	A	0	0.25
gca	A	1	0.40
gcg	A	0	0.07
gat	D	1	1.70
gac	D	2	1.30
gaa	E	39071	23155.00
gag	E	204	16120.00
ggt	G	0	2.29
ggc	G	0	2.37
gga	G	17	7.85
ggg	G	0	4.49
---	--	------	------
mPD		0.013	0.49
		nPD:	0.03
		N. weight:	0.55
		Sc. PD:	-0.019
		Sc. rank:	2152.3

,

PB1

Pos.	755	obs:	exp:
ttt	F	0	16.54
ttc	F	34	17.46
tta	L	0	4554.00
ttg	L	0	8113.00
ctt	L	6	8209.00
ctc	L	39233	5754.00
cta	L	3	6026.00
ctg	L	0	6586.00
cct	P	0	0.33
ccc	P	1	0.19
cca	P	0	0.34
ccg	P	0	0.14
cat	H	0	0.56
cac	H	1	0.44
cgt	R	0	0.02
cgc	R	1	0.04
cga	R	0	0.09
cgg	R	0	0.09
att	I	0	15.09
atc	I	43	11.30
ata	I	0	16.61
aga	R	0	0.49
agg	R	0	0.28
---	--	------	------
mPD		0.0045	1.2
		nPD:	0.
		N. weight:	2.1
		Sc. PD:	-0.12
		Sc. rank:	-52181.0

,

PB1

Pos.	756	obs:	exp:
tct	S	0	0.16
tcc	S	0	0.15
tca	S	0	0.27
tcg	S	0	0.04
cgt	R	0	771.40
cgc	R	0	1400.00
cga	R	1	3550.00
cgg	R	0	3341.00
aaa	K	414	272.20
aag	K	0	141.80
agt	S	1	0.17
agc	S	0	0.21
aga	R	38904	19082.00
agg	R	0	10761.00
ggt	G	0	0.27
ggc	G	0	0.28
gga	G	2	0.92
ggg	G	0	0.53
---	--	------	------
mPD		0.021	0.90
		nPD:	0.02
		N. weight:	0.78
		Sc. PD:	-0.03
		Sc. rank:	599.9

,

PB1

Pos.	757	obs:	exp:
cgt	R	0	779.70
cgc	R	0	1415.00
cga	R	1	3588.00
cgg	R	39321	3376.00
aga	R	0	19287.00
agg	R	0	10876.00
---	--	------	------
mPD		0.000051	0.89
		nPD:	0.
		N. weight:	2.7
		Sc. PD:	-0.16
		Sc. rank:	-83134.0

,

PB1

Pos.	758	obs:	exp:
tta	L	0	0.12
ttg	L	0	0.21
ctt	L	0	0.21
ctc	L	0	0.15
cta	L	1	0.15
ctg	L	0	0.17
cct	P	0	0.67
ccc	P	0	0.37
cca	P	2	0.68
ccg	P	0	0.28
cat	H	3	28.20
cac	H	47	21.80
caa	Q	39117	23966.00
cag	Q	90	15241.00
cgt	R	0	0.14
cgc	R	0	0.25
cga	R	7	0.64
cgg	R	0	0.60
aaa	K	53	34.84
aag	K	0	18.16
aga	R	0	3.43
agg	R	0	1.94
---	-	2	2.00
---	--	------	------
mPD		0.010	0.48
		nPD:	0.02
		N. weight:	0.52
		Sc. PD:	-0.021
		Sc. rank:	-551.6

,

PB1

Pos.	759	obs:	exp:
gaa	E	2	1.18
gag	E	0	0.82
---	-	39320	39320.00
---	--	------	------
mPD		0	1.3×10^{-9}
		nPD:	0.
		N. weight:	0.
		Sc. PD:	0
		Sc. rank:	0

,

PB1

Pos.	760	obs:	exp:
cgt	R	0	1.51
cgc	R	0	2.73
cga	R	0	6.93
cgg	R	0	6.53
act	T	0	0.62
acc	T	0	0.35
aca	T	2	0.87
acg	T	0	0.16
aat	N	21	12.87
aac	N	2	10.13
aaa	K	38973	25783.00
aag	K	244	13434.00
aga	R	76	37.28
agg	R	0	21.02
gaa	E	2	1.18
gag	E	0	0.82
---	-	2	2.00
---	--	------	------
mPD		0.018	0.46
		nPD:	0.04
		N. weight:	0.42
		Sc. PD:	-0.0099
		Sc. rank:	3980.8

,

PB1

Pos.	761	obs:	exp:
tta	L	3	0.35
ttg	L	0	0.62
tct	S	0	1.45
tcc	S	0	1.34
tca	S	9	2.42
tcg	S	0	0.35
tat	Y	1	1.05
tac	Y	1	0.95
ctt	L	0	0.63
ctc	L	0	0.44
cta	L	0	0.46
ctg	L	0	0.50
caa	Q	816	499.40
cag	Q	1	317.60
aaa	K	1	0.66
aag	K	0	0.34
agt	S	0	1.57
agc	S	0	1.86
---	-	38490	38490.00
---	--	------	------
mPD		0.000032	0.00024
		nPD:	0.13
		N. weight:	0.
		Sc. PD:	0
		Sc. rank:	0

,

PB1

Pos.	762	obs:	exp:
taa	.	37948	22338.00
tag	.	119	4307.00
tga	.	1255	12677.00
---	--	------	------
mPD		0.068	0.63
		nPD:	0.11
		N. weight:	0.47
		Sc. PD:	0.02
		Sc. rank:	10354.0

}

Amino acid level conservation per codon

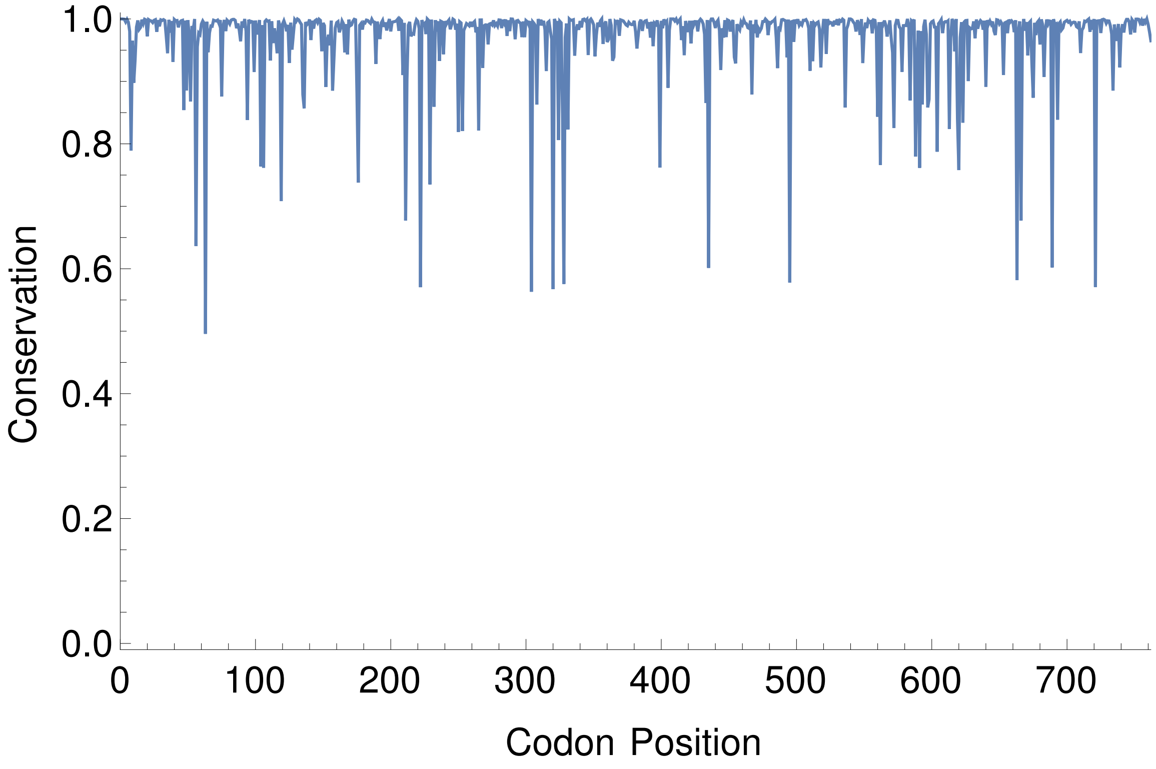

Conservation analysis plots

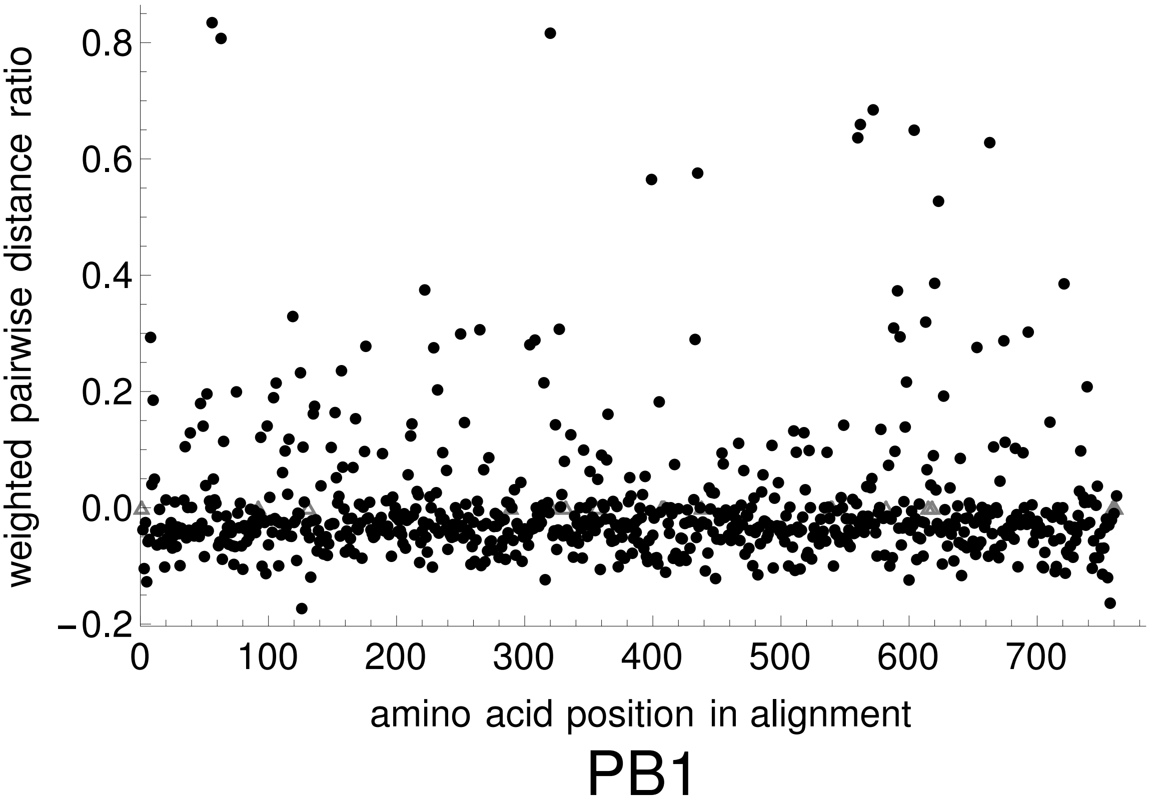

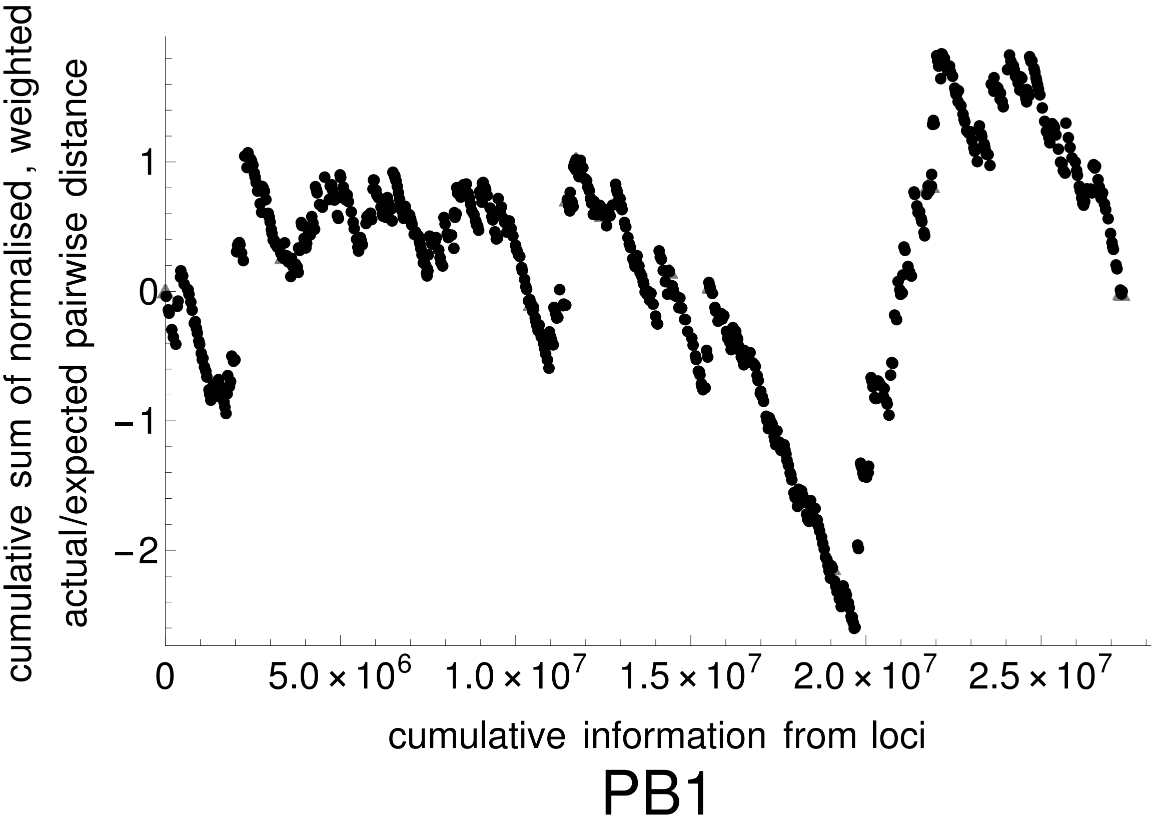

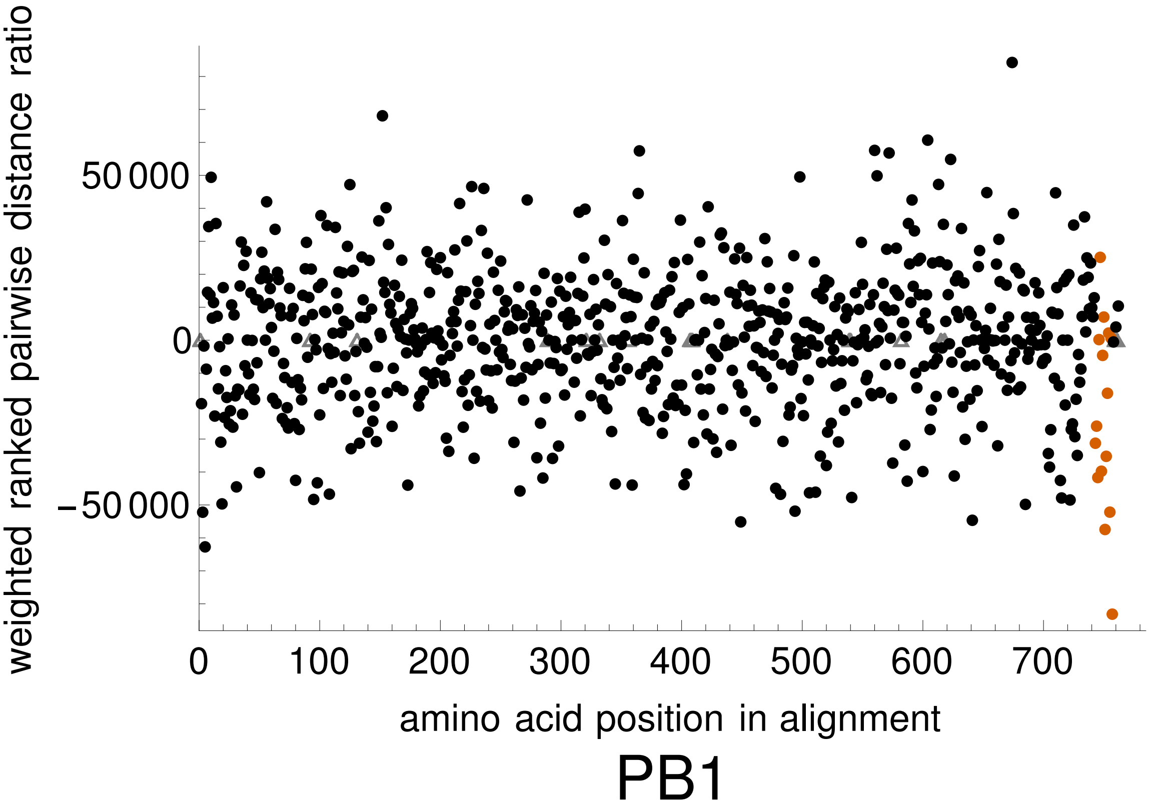
weighted ranked pairwise distance ratio
50 000
0
−50 000
0
100
200
300
400
500
600
700
amino acid position in alignment
PB1

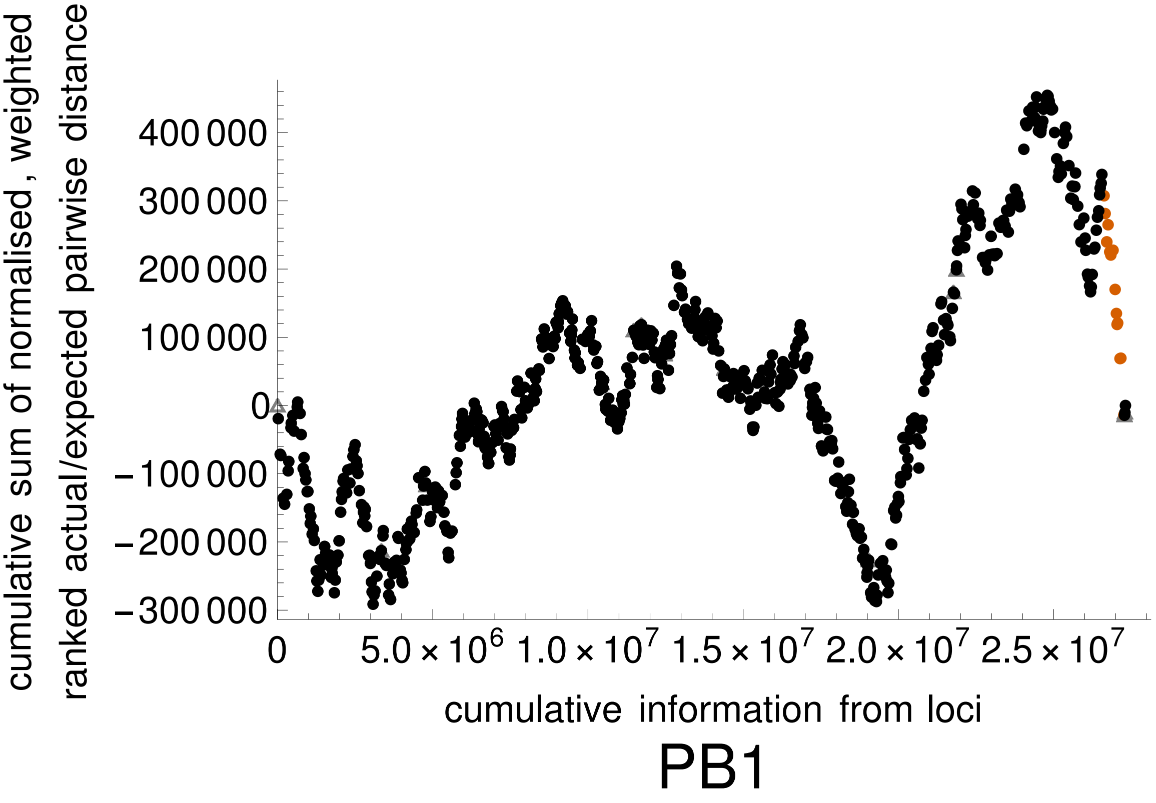
cumulative sum of normalised, weighted
ranked actual/expected pairwise distance
400 000
300 000
200 000
100 000
0
−100 000
−200 000
−300 000
0
5.0 × 10^6
1.0 × 10^7
1.5 × 10^7
2.0 × 10^7
2.5 × 10^7
cumulative information from loci
PB1

PB1-F2

Gene length histogram

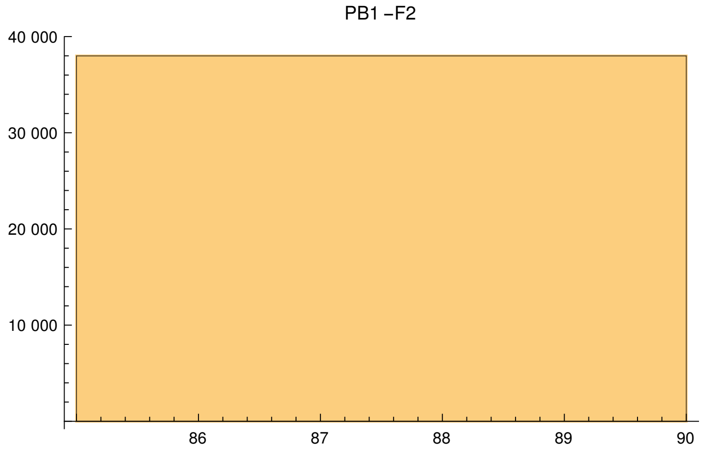

Information vs. nPD

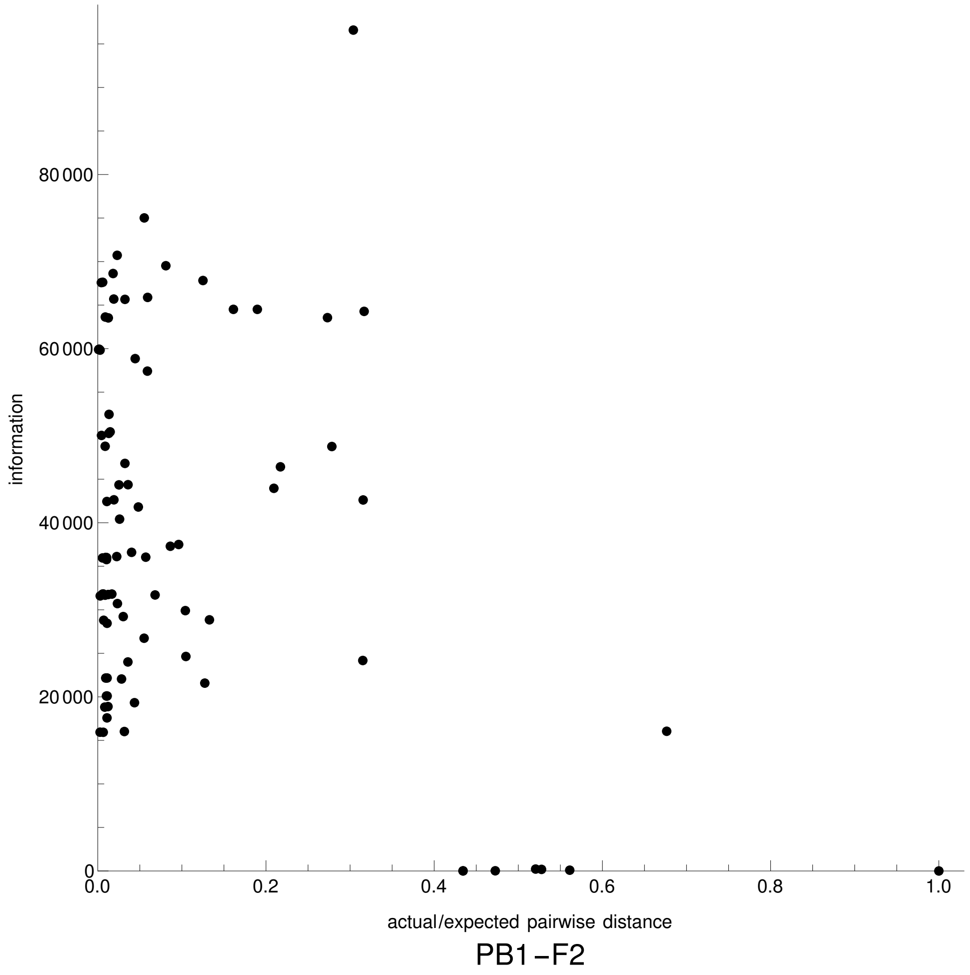

Example sequences highlighted by regions found in analyses to be conserved

```
Interesting points (by weighted raw PD) highlighted for gene PB1-F2:
ATGGAACAGGGACAGGGTACACTATGGACACAGTCAACAGAACACACCAATACTCAGAGAGGGGGAAGTGGACGACAA
    ATACAGAAACTGGGGCTCCCCAGCTCAACCCAATTGATGGACCACTACCTGAGGATAATGAACCAAGTGGATATGCA
    CAAACAGACTGTGTCCTGGAGGCTATGGCCTTCCTTGAAGAATCCCATCCAGGTATCTTTGAAAACTCCTGCC   ----
  ---TTGAAACAATGGAAGCCGTTCAACAGACAAGGG ---------TAG

Interesting points (by weighted ranked PD) highlighted for gene PB1-F2:
ATGGAACAGGGACAGGGTACACTATGGACACAGTCAACAGAACACACCAATACTCAGAGAGGGGGAAGTGGACGACAA
    ATACAGAAACTGGGGCTCCCCAGCTCAACCCAATTGATGGACCACTACCTGAGGATAATGAACCAAGTGGATATGCA
    CAAACAGACTGTGTCCTGGAGGCTATGGCCTTCCTTGAAGAATCCCATCCAGGTATCTTTGAAAACTCCTGCC   ----
  ---TTGAAACAATGGAAGCCGTTCAACAGACAAGGG ---------TAG
```

Per codon conservation report

PB1-F2

Pos.	1	obs:	exp:
atg	M	37993	37993.00
---	--	------	------
mPD		0	0
		nPD:	1.
		N. weight:	0.
		Sc. PD:	0
		Sc. rank:	0

PB1-F2

Pos.	2	obs:	exp:
gtt	V	0	0.25
gtc	V	0	0.20
gta	V	1	0.22
gtg	V	0	0.33
gct	A	0	0.82
gcc	A	0	0.75
gca	A	3	1.21
gcg	A	0	0.22
gat	D	0	0.57
gac	D	1	0.43
gaa	E	37897	22343.00
gag	E	0	15554.00
ggt	G	0	12.25
ggc	G	0	12.70
gga	G	91	42.01
ggg	G	0	24.04
---	--	------	------
mPD		0.0050	0.49
		nPD:	0.01
		N. weight:	0.56
		Sc. PD:	-0.031
		Sc. rank:	-8457.6

PB1-F2

Pos.	3	obs:	exp:
tta	L	0	2.44
ttg	L	0	4.34
ctt	L	0	4.39
ctc	L	0	3.08
cta	L	0	3.22
ctg	L	21	3.52
cct	P	0	0.33
ccc	P	0	0.19
cca	P	0	0.34
ccg	P	1	0.14
caa	Q	1	22565.00
cag	Q	36914	14350.00
cgt	R	0	20.94
cgc	R	0	38.00
cga	R	0	96.35
cgg	R	1056	90.68
aga	R	0	518.00
agg	R	0	292.10
---	--	------	------
mPD		0.055	0.57
		nPD:	0.1
		N. weight:	1.
		Sc. PD:	0.024
		Sc. rank:	17603.0

PB1-F2

Pos.	4	obs:	exp:
gtt	V	0	0.75
gtc	V	0	0.61
gta	V	3	0.65
gtg	V	0	0.99
gaa	E	2063	1216.00
gag	E	0	846.70
ggt	G	0	4837.00
ggc	G	0	5014.00
gga	G	35925	16585.00
ggg	G	2	9492.00
---	--	------	------
mPD		0.10	0.78
		nPD:	0.13
		N. weight:	0.8
		Sc. PD:	0.046
		Sc. rank:	16087.0

PB1-F2

Pos.	5	obs:	exp:
tta	L	0	3.02
ttg	L	0	5.38
ctt	L	0	5.44
ctc	L	0	3.81
cta	L	0	3.99
ctg	L	26	4.36
cct	P	0	2.00
ccc	P	0	1.12
cca	P	0	2.03
ccg	P	6	0.85
caa	Q	0	23192.00
cag	Q	37941	14749.00
cgt	R	0	0.40
cgc	R	0	0.72
cga	R	0	1.82
cgg	R	20	1.72
aga	R	0	9.81
agg	R	0	5.53
---	--	------	------
mPD		0.0027	0.48
		nPD:	0.01
		N. weight:	1.
		Sc. PD:	-0.06
		Sc. rank:	-22870.0

PB1-F2

Pos.	6	obs:	exp:
tct	S	0	0.16
tcc	S	0	0.15
tca	S	0	0.27
tcg	S	0	0.04
tgt	C	1	0.39
tgc	C	0	0.61
agt	S	1	0.17
agc	S	0	0.21
gtt	V	50	12.52
gtc	V	0	10.19
gta	V	0	10.84
gtg	V	0	16.45
gct	A	4	1.09
gcc	A	0	1.00
gca	A	0	1.62
gcg	A	0	0.29
gat	D	706	399.10
gac	D	0	306.90
ggt	G	37217	5012.00
ggc	G	14	5196.00
gga	G	0	17186.00
ggg	G	0	9836.00
---	--	------	------
mPD		0.040	0.73
		nPD:	0.06
		N. weight:	2.1
		Sc. PD:	-0.03
		Sc. rank:	22361.0

PB1-F2

Pos.	7	obs:	exp:
att	I	0	24.92
atc	I	0	18.66
ata	I	71	27.42
act	T	0	11777.00
acc	T	0	6705.00
aca	T	37919	16412.00
acg	T	3	3029.00
---	--	------	------
mPD		0.0039	0.68
		nPD:	0.01
		N. weight:	0.88
		Sc. PD:	-0.053
		Sc. rank:	-20812.0

PB1-F2

Pos.	8	obs:	exp:
tta	L	0	4174.00
ttg	L	0	7437.00
ctt	L	0	7525.00
ctc	L	4	5275.00
cta	L	35965	5524.00
ctg	L	2	6037.00
cct	P	0	666.50
ccc	P	0	374.90
cca	P	2000	675.70
ccg	P	0	282.90
cat	H	0	0.56
cac	H	1	0.44
caa	Q	20	12.23
cag	Q	0	7.77
cgt	R	0	0.02
cgc	R	0	0.04
cga	R	1	0.09
cgg	R	0	0.09
aga	R	0	0.49
agg	R	0	0.28
---	--	------	------
mPD		0.10	1.3
		nPD:	0.08
		N. weight:	1.9
		Sc. PD:	0.018
		Sc. rank:	29977.0

PB1-F2

Pos.	9	obs:	exp:
tta	L	0	0.46
ttg	L	4	0.83
tgg	W	37989	37989.00
ctt	L	0	0.84
ctc	L	0	0.59
cta	L	0	0.61
ctg	L	0	0.67
---	--	------	------
mPD		0.00021	0.00048
		nPD:	0.43
		N. weight:	0.00018
		Sc. PD:	0.000058
		Sc. rank:	5.3

PB1-F2

Pos.	10	obs:	exp:
cgt	R	0	0.02
cgc	R	0	0.04
cga	R	0	0.09
cgg	R	0	0.09
att	I	0	26.68
atc	I	0	19.97
ata	I	76	29.35
act	T	0	11773.00
acc	T	0	6703.00
aca	T	37909	16407.00
acg	T	1	3028.00
aaa	K	5	3.29
aag	K	0	1.71
aga	R	1	0.49
agg	R	0	0.28
gct	A	0	0.27
gcc	A	0	0.25
gca	A	1	0.40
gcg	A	0	0.07
---	--	------	------
mPD		0.0044	0.68
		nPD:	0.01
		N. weight:	0.88
		Sc. PD:	-0.052
		Sc. rank:	-18621.0

PB1-F2

Pos.	11	obs:	exp:
tta	L	0	3.83
ttg	L	0	6.82
ctt	L	0	6.90
ctc	L	0	4.84
cta	L	0	5.07
ctg	L	33	5.54
cct	P	0	0.33
ccc	P	0	0.19
cca	P	0	0.34
ccg	P	1	0.14
caa	Q	1	23168.00
cag	Q	37900	14733.00
cgt	R	0	1.13
cgc	R	0	2.05
cga	R	0	5.20
cgg	R	57	4.89
aaa	K	0	0.66
aag	K	1	0.34
aga	R	0	27.96
agg	R	0	15.77
---	--	------	------
mPD		0.0049	0.48
		nPD:	0.01
		N. weight:	1.
		Sc. PD:	-0.056
		Sc. rank:	-15519.0

PB1-F2			
Pos.	12	obs:	exp:
tta	L	413	47.92
ttg	L	0	85.39
tct	S	0	6064.00
tcc	S	0	5616.00
tca	S	37579	10100.00
tcg	S	0	1479.00
ctt	L	0	86.40
ctc	L	0	60.56
cta	L	0	63.42
ctg	L	0	69.31
cct	P	0	0.33
ccc	P	0	0.19
cca	P	1	0.34
ccg	P	0	0.14
agt	S	0	6567.00
agc	S	0	7752.00
---	--	------	------
mPD		0.022	1.7
		nPD:	0.01
		N. weight:	1.4
		Sc. PD:	-0.074
		Sc. rank:	-11730.0

,

PB1-F2			
Pos.	13	obs:	exp:
tct	S	0	0.16
tcc	S	0	0.15
tca	S	1	0.27
tcg	S	0	0.04
cct	P	0	0.67
ccc	P	0	0.37
cca	P	2	0.68
ccg	P	0	0.28
att	I	0	59.68
atc	I	0	44.67
ata	I	170	65.65
act	T	0	11732.00
acc	T	4	6679.00
aca	T	37773	16349.00
acg	T	0	3017.00
aaa	K	1	0.66
aag	K	0	0.34
agt	S	0	0.17
agc	S	0	0.21
gct	A	0	11.45
gcc	A	0	10.49
gca	A	42	16.99
gcg	A	0	3.07
---	--	------	------
mPD		0.012	0.69
		nPD:	0.02
		N. weight:	0.88
		Sc. PD:	-0.044
		Sc. rank:	-5234.4

,

PB1-F2			
Pos.	14	obs:	exp:
aaa	K	1	0.66
aag	K	0	0.34
gaa	E	37889	22338.00
gag	E	0	15551.00
ggt	G	0	13.87
ggc	G	0	14.38
gga	G	103	47.55
ggg	G	0	27.21
---	--	------	------
mPD		0.0055	0.49
		nPD:	0.01
		N. weight:	0.56
		Sc. PD:	-0.031
		Sc. rank:	-6378.8

,

PB1-F2			
Pos.	15	obs:	exp:
tta	L	0	0.23
ttg	L	0	0.41
tat	Y	0	0.53
tac	Y	1	0.47
ctt	L	0	0.42
ctc	L	2	0.29
cta	L	0	0.31
ctg	L	0	0.34
cct	P	0	13.00
ccc	P	39	7.31
cca	P	0	13.18
ccg	P	0	5.52
cat	H	0	21383.00
cac	H	37910	16527.00
cgt	R	0	0.81
cgc	R	41	1.48
cga	R	0	3.74
cgg	R	0	3.52
aga	R	0	20.11
agg	R	0	11.34
---	--	------	------
mPD		0.0044	0.50
		nPD:	0.01
		N. weight:	0.88
		Sc. PD:	-0.05
		Sc. rank:	-16999.0

,

PB1-F2			
Pos.	16	obs:	exp:
att	I	0	1996.00
atc	I	5684	1494.00
ata	I	1	2196.00
act	T	0	10033.00
acc	T	32307	5712.00
aca	T	1	13982.00
acg	T	0	2580.00
---	--	------	------
mPD		0.25	0.93
		nPD:	0.27
		N. weight:	1.8
		Sc. PD:	0.33
		Sc. rank:	44630.0

,

PB1-F2			
Pos.	17	obs:	exp:
tct	S	0	13.39
tcc	S	0	12.40
tca	S	0	22.31
tcg	S	0	3.27
tat	Y	1	0.53
tac	Y	0	0.47
act	T	2	0.62
acc	T	0	0.35
aca	T	0	0.87
acg	T	0	0.16
aat	N	37903	21206.00
aac	N	0	16697.00
agt	S	83	14.50
agc	S	0	17.12
gat	D	4	2.26
gac	D	0	1.74
---	--	------	------
mPD		0.0047	0.50
		nPD:	0.01
		N. weight:	0.62
		Sc. PD:	-0.035
		Sc. rank:	-9961.0

,

PB1-F2			
Pos.	18	obs:	exp:
att	I	3774	1325.00
atc	I	0	991.70
ata	I	0	1458.00
atg	M	1	1.00
act	T	34211	10625.00
acc	T	0	6049.00
aca	T	1	14806.00
acg	T	0	2732.00
gtt	V	4	1.00
gtc	V	0	0.82
gta	V	0	0.87
gtg	V	0	1.32
gct	A	2	0.55
gcc	A	0	0.50
gca	A	0	0.81
gcg	A	0	0.15
---	--	------	------
mPD		0.18	0.86
		nPD:	0.21
		N. weight:	1.2
		Sc. PD:	0.16
		Sc. rank:	28396.0

,

PB1-F2			
Pos.	19	obs:	exp:
tta	L	0	3.25
ttg	L	0	5.79
ctt	L	0	5.86
ctc	L	0	4.11
cta	L	0	4.30
ctg	L	28	4.70
cct	P	0	0.33
ccc	P	0	0.19
cca	P	0	0.34
ccg	P	1	0.14
caa	Q	25	23181.00
cag	Q	37898	14742.00
cgt	R	0	0.81
cgc	R	0	1.48
cga	R	0	3.74
cgg	R	41	3.52
aga	R	0	20.11
agg	R	0	11.34
---	--	------	------
mPD		0.0050	0.48
		nPD:	0.01
		N. weight:	0.99
		Sc. PD:	-0.055
		Sc. rank:	-14398.0

,

PB1-F2			
Pos.	20	obs:	exp:
tct	S	0	1.61
tcc	S	0	1.49
tca	S	0	2.69
tcg	S	0	0.39
cgt	R	0	733.70
cgc	R	0	1331.00
cga	R	0	3376.00
cgg	R	0	3177.00
att	I	0	2.11
atc	I	0	1.58
ata	I	6	2.32
aaa	K	966	635.10
aag	K	0	330.90
agt	S	7	1.75
agc	S	3	2.06
aga	R	36997	18149.00
agg	R	6	10235.00
ggt	G	0	1.08
ggc	G	0	1.12
gga	G	8	3.69
ggg	G	0	2.11
---	--	------	------
mPD		0.051	0.93
		nPD:	0.06
		N. weight:	0.74
		Sc. PD:	-0.011
		Sc. rank:	6945.1

,

PB1-F2			
Pos.	21	obs:	exp:
tgg	W	10	10.00
cgt	R	0	20.24
cgc	R	0	36.74
cga	R	0	93.16
cgg	R	0	87.67
act	T	0	0.31
acc	T	0	0.18
aca	T	0	0.43
acg	T	1	0.08
aaa	K	0	165.00
aag	K	251	85.98
aga	R	0	500.80
agg	R	1021	282.40
gaa	E	0	2235.00
gag	E	3791	1556.00
ggt	G	0	4432.00
ggc	G	1	4594.00
gga	G	1	15196.00
ggg	G	32916	8697.00
---	-	1	1.00
---	--	------	------
mPD		0.26	0.92
		nPD:	0.28
		N. weight:	1.4
		Sc. PD:	0.26
		Sc. rank:	35450.0

,

PB1-F2			
Pos.	22	obs:	exp:
cct	P	0	0.33
ccc	P	0	0.19
cca	P	1	0.34
ccg	P	0	0.14
gtt	V	0	0.50
gtc	V	0	0.41
gta	V	2	0.43
gtg	V	0	0.66
gaa	E	396	233.50
gag	E	0	162.50
ggt	G	0	5061.00
ggc	G	0	5247.00
gga	G	37588	17354.00
ggg	G	6	9932.00
---	--	------	------
mPD		0.021	0.70
		nPD:	0.03
		N. weight:	0.81
		Sc. PD:	-0.03
		Sc. rank:	1915.9

,

PB1-F2

Pos.	23	obs:	exp:
tct	S	0	6034.00
tcc	S	0	5588.00
tca	S	0	10050.00
tcg	S	0	1472.00
tgt	C	3	1.17
tgc	C	0	1.83
cgt	R	0	0.02
cgc	R	1	0.04
cga	R	0	0.09
cgg	R	0	0.09
att	I	8	2.81
atc	I	0	2.10
ata	I	0	3.09
aat	N	509	284.80
aac	N	0	224.20
aaa	K	0	0.66
aag	K	1	0.34
agt	S	37393	6535.00
agc	S	0	7714.00
aga	R	0	0.49
agg	R	0	0.28
gat	D	1	0.57
gac	D	0	0.43
ggt	G	77	10.37
ggc	G	0	10.75
gga	G	0	35.54
ggg	G	0	20.34
---	--	------	------
mPD		0.031	1.7
		nPD:	0.02
		N. weight:	1.8
		Sc. PD:	-0.087
		Sc. rank:	-6303.6

,

PB1-F2

Pos.	24	obs:	exp:
tgg	W	1	1.00
cgt	R	0	0.02
cgc	R	0	0.04
cga	R	1	0.09
cgg	R	0	0.09
aga	R	0	0.49
agg	R	0	0.28
ggt	G	1	5115.00
ggc	G	0	5302.00
gga	G	37903	17537.00
ggg	G	87	10037.00
---	--	------	------
mPD		0.0048	0.68
		nPD:	0.01
		N. weight:	0.8
		Sc. PD:	-0.047
		Sc. rank:	-16195.0

,

PB1-F2

Pos.	25	obs:	exp:
tta	L	0	4.53
ttg	L	0	8.06
ctt	L	0	8.16
ctc	L	0	5.72
cta	L	39	5.99
ctg	L	0	6.55
cct	P	0	4.67
ccc	P	0	2.62
cca	P	14	4.73
ccg	P	0	1.98
cat	H	0	0.56
cac	H	1	0.44
caa	Q	13868	8480.00
cag	Q	4	5392.00
cgt	R	0	477.00
cgc	R	0	865.70
cga	R	24037	2195.00
cgg	R	2	2066.00
act	T	0	0.31
acc	T	0	0.18
aca	T	0	0.43
acg	T	1	0.08
aaa	K	6	3.94
aag	K	0	2.06
aga	R	20	11801.00
agg	R	0	6655.00
---	-	1	1.00
---	--	------	------
mPD		0.47	1.5
		nPD:	0.32
		N. weight:	1.8
		Sc. PD:	0.4
		Sc. rank:	54213.0

,

PB1-F2

Pos.	26	obs:	exp:
tta	L	0	6.61
ttg	L	0	11.78
ctt	L	0	11.92
ctc	L	0	8.36
cta	L	57	8.75
ctg	L	0	9.57
cct	P	0	0.33
ccc	P	0	0.19
cca	P	1	0.34
ccg	P	0	0.14
caa	Q	36448	22280.00
cag	Q	0	14168.00
cgt	R	0	29.27
cgc	R	0	53.11
cga	R	1475	134.70
cgg	R	0	126.70
aaa	K	10	6.57
aag	K	0	3.43
aga	R	1	724.00
agg	R	0	408.30
gaa	E	1	0.59
gag	E	0	0.41
---	--	------	------
mPD		0.078	0.61
		nPD:	0.13
		N. weight:	0.6
		Sc. PD:	0.031
		Sc. rank:	11715.0

,

PB1-F2

Pos.	27	obs:	exp:
att	I	2	13067.00
atc	I	0	9781.00
ata	I	37222	14376.00
atg	M	12	12.00
act	T	0	227.00
acc	T	0	129.20
aca	T	731	316.40
acg	T	0	58.38
aaa	K	2	1.31
aag	K	0	0.69
gtt	V	0	6.01
gtc	V	0	4.89
gta	V	24	5.20
gtg	V	0	7.90
---	--	------	------
mPD		0.040	0.70
		nPD:	0.06
		N. weight:	1.
		Sc. PD:	-0.013
		Sc. rank:	11408.0

,

PB1-F2

Pos.	28	obs:	exp:
tta	L	0	2.90
ttg	L	0	5.17
ctt	L	0	5.23
ctc	L	0	3.67
cta	L	0	3.84
ctg	L	25	4.20
cct	P	0	2.67
ccc	P	0	1.50
cca	P	0	2.70
ccg	P	8	1.13
cat	H	0	0.56
cac	H	1	0.44
caa	Q	0	22648.00
cag	Q	37050	14402.00
cgt	R	0	18.00
cgc	R	0	32.67
cga	R	0	82.85
cgg	R	908	77.97
aaa	K	0	0.66
aag	K	1	0.34
aga	R	0	445.40
agg	R	0	251.20
---	--	------	------
mPD		0.048	0.56
		nPD:	0.09
		N. weight:	1.
		Sc. PD:	0.015
		Sc. rank:	16794.0

,

PB1-F2

Pos.	29	obs:	exp:
cgt	R	0	5.63
cgc	R	0	10.22
cga	R	0	25.91
cgg	R	0	24.39
aaa	K	37709	24791.00
aag	K	0	12918.00
aga	R	284	139.30
agg	R	0	78.55
---	--	------	------
mPD		0.015	0.47
		nPD:	0.03
		N. weight:	0.44
		Sc. PD:	-0.016
		Sc. rank:	1181.3

,

PB1-F2

Pos.	30	obs:	exp:
tta	L	0	4395.00
ttg	L	6	7831.00
ctt	L	2	7923.00
ctc	L	0	5554.00
cta	L	5	5816.00
ctg	L	37864	6357.00
cct	P	0	10.00
ccc	P	0	5.62
cca	P	0	10.14
ccg	P	30	4.24
caa	Q	0	49.51
cag	Q	81	31.49
cgt	R	0	0.08
cgc	R	0	0.14
cga	R	0	0.36
cgg	R	4	0.34
atg	M	1	1.00
aga	R	0	1.96
agg	R	0	1.11
---	--	------	------
mPD		0.0068	1.2
		nPD:	0.01
		N. weight:	1.9
		Sc. PD:	-0.11
		Sc. rank:	-40673.0

,

PB1-F2

Pos.	31	obs:	exp:
cgt	R	0	0.36
cgc	R	0	0.65
cga	R	0	1.64
cgg	R	0	1.55
aga	R	0	8.83
agg	R	18	4.98
gtt	V	0	1.00
gtc	V	0	0.82
gta	V	0	0.87
gtg	V	4	1.32
gct	A	0	0.27
gcc	A	0	0.25
gca	A	0	0.40
gcg	A	1	0.07
gat	D	1	0.57
gac	D	0	0.43
gaa	E	0	95.51
gag	E	162	66.49
ggt	G	0	5090.00
ggc	G	0	5277.00
gga	G	6	17452.00
ggg	G	37801	9988.00
---	--	------	------
mPD		0.010	0.69
		nPD:	0.01
		N. weight:	1.4
		Sc. PD:	-0.072
		Sc. rank:	-9120.0

,

PB1-F2

Pos.	32	obs:	exp:
ttt	F	0	2.43
ttc	F	5	2.57
tta	L	0	1891.00
ttg	L	0	3368.00
tgt	C	0	1.17
tgc	C	3	1.83
ctt	L	27	3408.00
ctc	L	16266	2389.00
cta	L	0	2502.00
ctg	L	0	2734.00
cct	P	0	16.00
ccc	P	48	9.00
cca	P	0	16.22
ccg	P	0	6.79
cat	H	2	1437.00
cac	H	2545	1110.00
cgt	R	13	378.60
cgc	R	19078	687.10
cga	R	2	1742.00
cgg	R	4	1640.00
aga	R	0	9367.00
agg	R	0	5282.00
---	--	------	------
mPD		0.56	1.8
		nPD:	0.3
		N. weight:	2.7
		Sc. PD:	0.57
		Sc. rank:	74996.0

,

PB1-F2

Pos.	33	obs:	exp:
tta	L	0	11.84
ttg	L	0	21.09
tct	S	0	1.78
tcc	S	11	1.64
tca	S	0	2.96
tcg	S	0	0.43
ctt	L	0	21.34
ctc	L	102	14.96
cta	L	0	15.66
ctg	L	0	17.12
cct	P	0	12619.00
ccc	P	37869	7099.00
cca	P	0	12794.00
ccg	P	0	5356.00
cat	H	0	1.69
cac	H	3	1.31
act	T	0	2.48
acc	T	8	1.41
aca	T	0	3.46
acg	T	0	0.64
agt	S	0	1.92
agc	S	0	2.27
---	--	------	------
mPD		0.0065	0.73
		nPD:	0.01
		N. weight:	1.8
		Sc. PD:	-0.1
		Sc. rank:	-30489.0

,

PB1-F2

Pos.	34	obs:	exp:
tct	S	0	5893.00
tcc	S	0	5458.00
tca	S	0	9816.00
tcg	S	0	1438.00
tgt	C	0	0.39
tgc	C	1	0.61
cgt	R	0	0.08
cgc	R	0	0.14
cga	R	0	0.36
cgg	R	0	0.34
aat	N	0	820.70
aac	N	1467	646.30
agt	S	0	6382.00
agc	S	36521	7534.00
aga	R	4	1.96
agg	R	0	1.11
---	--	------	------
mPD		0.075	1.7
		nPD:	0.04
		N. weight:	1.6
		Sc. PD:	-0.04
		Sc. rank:	13227.0

,

PB1-F2

Pos.	35	obs:	exp:
tta	L	135	15.67
ttg	L	0	27.91
tct	S	0	6109.00
tcc	S	0	5657.00
tca	S	37857	10175.00
tcg	S	0	1490.00
tat	Y	1	0.53
tac	Y	0	0.47
ctt	L	0	28.24
ctc	L	0	19.80
cta	L	0	20.73
ctg	L	0	22.66
agt	S	0	6616.00
agc	S	0	7810.00
---	--	------	------
mPD		0.0072	1.6
		nPD:	0.
		N. weight:	1.4
		Sc. PD:	-0.085
		Sc. rank:	-33552.0

,

PB1-F2

Pos.	36	obs:	exp:
tct	S	0	0.16
tcc	S	1	0.15
tca	S	0	0.27
tcg	S	0	0.04
att	I	0	152.00
atc	I	433	113.80
ata	I	0	167.20
act	T	0	11664.00
acc	T	37558	6640.00
aca	T	0	16254.00
acg	T	0	3000.00
agt	S	0	0.17
agc	S	0	0.21
gct	A	0	0.27
gcc	A	1	0.25
gca	A	0	0.40
gcg	A	0	0.07
---	--	------	------
mPD		0.023	0.70
		nPD:	0.03
		N. weight:	1.8
		Sc. PD:	-0.065
		Sc. rank:	8614.6

,

PB1-F2

Pos.	37	obs:	exp:
tta	L	0	1.62
ttg	L	0	2.89
ctt	L	0	2.93
ctc	L	0	2.05
cta	L	14	2.15
ctg	L	0	2.35
cct	P	0	0.67
ccc	P	0	0.37
cca	P	2	0.68
ccg	P	0	0.28
caa	Q	37563	22972.00
cag	Q	18	14609.00
cgt	R	0	7.85
cgc	R	0	14.25
cga	R	396	36.13
cgg	R	0	34.00
aga	R	0	194.20
agg	R	0	109.50
---	--	------	------
mPD		0.022	0.51
		nPD:	0.04
		N. weight:	0.54
		Sc. PD:	-0.013
		Sc. rank:	3763.3

,

PB1-F2

Pos.	38	obs:	exp:
tta	L	3	4403.00
ttg	L	37933	7846.00
tct	S	0	7.26
tcc	S	0	6.72
tca	S	0	12.10
tcg	S	45	1.77
ctt	L	0	7938.00
ctc	L	0	5565.00
cta	L	0	5827.00
ctg	L	12	6369.00
agt	S	0	7.86
agc	S	0	9.28
---	--	------	------
mPD		0.0032	1.2
		nPD:	0.
		N. weight:	1.7
		Sc. PD:	-0.1
		Sc. rank:	-44684.0

,

PB1-F2

Pos.	39	obs:	exp:
atg	M	37905	37905.00
act	T	0	27.02
acc	T	0	15.38
aca	T	0	37.65
acg	T	87	6.95
---	-	1	1.00
---	--	------	------
mPD		0.0046	0.0088
		nPD:	0.52
		N. weight:	0.0061
		Sc. PD:	0.0025
		Sc. rank:	185.5

,

PB1-F2

Pos.	40	obs:	exp:
gct	A	0	2.73
gcc	A	10	2.50
gca	A	0	4.05
gcg	A	0	0.73
gat	D	2	21411.00
gac	D	37876	16467.00
ggt	G	0	14.14
ggc	G	105	14.65
gga	G	0	48.47
ggg	G	0	27.74
---	--	------	------
mPD		0.0061	0.50
		nPD:	0.01
		N. weight:	0.88
		Sc. PD:	-0.047
		Sc. rank:	-9255.6

,

PB1-F2

Pos.	41	obs:	exp:
cct	P	0	9.33
ccc	P	28	5.25
cca	P	0	9.46
ccg	P	0	3.96
cat	H	140	21384.00
cac	H	37773	16529.00
caa	Q	7	4.28
cag	Q	0	2.72
cgt	R	2	0.89
cgc	R	43	1.62
cga	R	0	4.11
cgg	R	0	3.86
aga	R	0	22.07
agg	R	0	12.45
---	--	------	------
mPD		0.012	0.50
		nPD:	0.02
		N. weight:	0.85
		Sc. PD:	-0.037
		Sc. rank:	-120.3

,

PB1-F2

Pos.	42	obs:	exp:
tct	S	0	2.90
tcc	S	18	2.69
tca	S	0	4.84
tcg	S	0	0.71
tat	Y	0	19971.00
tac	Y	37887	17916.00
tgt	C	0	34.39
tgc	C	88	53.61
agt	S	0	3.15
agc	S	0	3.71
---	--	------	------
mPD		0.0056	0.50
		nPD:	0.01
		N. weight:	0.79
		Sc. PD:	-0.043
		Sc. rank:	-9643.4

,

PB1-F2

Pos.	43	obs:	exp:
tta	L	0	4401.00
ttg	L	1	7841.00
ctt	L	0	7934.00
ctc	L	0	5562.00
cta	L	26	5824.00
ctg	L	37900	6365.00
cct	P	0	14.66
ccc	P	0	8.25
cca	P	0	14.87
ccg	P	44	6.22
caa	Q	0	8.56
cag	Q	14	5.44
cgt	R	0	0.14
cgc	R	0	0.25
cga	R	0	0.64
cgg	R	7	0.60
atg	M	1	1.00
aga	R	0	3.43
agg	R	0	1.94
---	--	------	------
mPD		0.0049	1.2
		nPD:	0.
		N. weight:	1.9
		Sc. PD:	-0.11
		Sc. rank:	-47054.0

,

PB1-F2

Pos.	44	obs:	exp:
tgg	W	3	3.00
cgt	R	0	658.20
cgc	R	0	1194.00
cga	R	0	3029.00
cgg	R	0	2850.00
att	I	0	3.16
atc	I	0	2.36
ata	I	9	3.48
atg	M	205	205.00
aaa	K	4	2999.00
aag	K	4558	1563.00
aga	R	164	16281.00
agg	R	33030	9181.00
gtt	V	0	1.00
gtc	V	0	0.82
gta	V	0	0.87
gtg	V	4	1.32
gaa	E	0	1.18
gag	E	2	0.82
ggt	G	0	1.88
ggc	G	0	1.95
gga	G	0	6.46
ggg	G	14	3.70
---	--	------	------
mPD		0.23	1.1
		nPD:	0.22
		N. weight:	1.3
		Sc. PD:	0.17
		Sc. rank:	31090.0

,

PB1-F2

Pos.	45	obs:	exp:
att	I	0	13235.00
atc	I	0	9907.00
ata	I	37703	14561.00
atg	M	11	11.00
act	T	0	81.68
acc	T	0	46.50
aca	T	263	113.80
acg	T	0	21.01
gtt	V	0	4.01
gtc	V	0	3.26
gta	V	16	3.47
gtg	V	0	5.26
---	--	------	------
mPD		0.015	0.67
		nPD:	0.02
		N. weight:	1.
		Sc. PD:	-0.045
		Sc. rank:	-2012.7

,

PB1-F2

Pos.	46	obs:	exp:
att	I	0	2.11
atc	I	1	1.58
ata	I	5	2.32
atg	M	37953	37953.00
act	T	0	10.56
acc	T	0	6.01
aca	T	0	14.71
acg	T	34	2.72
---	--	------	------
mPD		0.0021	0.0037
		nPD:	0.56
		N. weight:	0.0025
		Sc. PD:	0.0011
		Sc. rank:	76.1

,

PB1-F2

Pos.	47	obs:	exp:
tct	S	0	117.50
tcc	S	0	108.80
tca	S	0	195.70
tcg	S	0	28.66
att	I	0	0.70
atc	I	2	0.53
ata	I	0	0.77
act	T	0	1.86
acc	T	6	1.06
aca	T	0	2.60
acg	T	0	0.48
aat	N	0	20842.00
aac	N	37253	16411.00
agt	S	0	127.20
agc	S	728	150.20
gat	D	0	2.26
gac	D	4	1.74
---	--	------	------
mPD		0.038	0.56
		nPD:	0.07
		N. weight:	0.88
		Sc. PD:	-0.0022
		Sc. rank:	12545.0

,

PB1-F2

Pos.	48	obs:	exp:
tta	L	0	0.35
ttg	L	0	0.62
ctt	L	0	0.63
ctc	L	0	0.44
cta	L	3	0.46
ctg	L	0	0.50
cct	P	0	1.33
ccc	P	0	0.75
cca	P	4	1.35
ccg	P	0	0.57
cat	H	2	1.13
cac	H	0	0.87
caa	Q	37917	23178.00
cag	Q	1	14740.00
cgt	R	0	1.31
cgc	R	0	2.37
cga	R	66	6.02
cgg	R	0	5.67
aga	R	0	32.37
agg	R	0	18.26
---	--	------	------
mPD		0.0040	0.48
		nPD:	0.01
		N. weight:	0.52
		Sc. PD:	-0.03
		Sc. rank:	-10404.0

,

PB1-F2

Pos.	49	obs:	exp:
tta	L	0	0.58
ttg	L	5	1.03
ctt	L	0	1.05
ctc	L	0	0.73
cta	L	0	0.77
ctg	L	0	0.84
atg	M	7	7.00
gtt	V	0	9478.00
gtc	V	0	7710.00
gta	V	0	8201.00
gtg	V	37839	12450.00
gct	A	0	38.70
gcc	A	0	35.48
gca	A	0	57.44
gcg	A	142	10.38
---	--	------	------
mPD		0.0081	0.75
		nPD:	0.01
		N. weight:	1.2
		Sc. PD:	-0.065
		Sc. rank:	-15384.0

,

PB1-F2

Pos.	50	obs:	exp:
gtt	V	4	1.00
gtc	V	0	0.82
gta	V	0	0.87
gtg	V	0	1.32
gct	A	7	1.91
gcc	A	0	1.75
gca	A	0	2.83
gcg	A	0	0.51
gat	D	37717	21321.00
gac	D	1	16397.00
ggt	G	264	35.54
ggc	G	0	36.85
gga	G	0	121.90
ggg	G	0	69.75
---	--	------	------
mPD		0.014	0.51
		nPD:	0.03
		N. weight:	0.61
		Sc. PD:	-0.024
		Sc. rank:	1116.7

,

PB1-F2

Pos.	51	obs:	exp:
att	I	2	0.70
atc	I	0	0.53
ata	I	0	0.77
atg	M	37920	37920.00
act	T	0	22.05
acc	T	0	12.55
aca	T	0	30.73
acg	T	71	5.67
---	--	------	------
mPD		0.0038	0.0073
		nPD:	0.53
		N. weight:	0.005
		Sc. PD:	0.0021
		Sc. rank:	153.1

,

PB1-F2

Pos.	52	obs:	exp:
tat	Y	0	0.53
tac	Y	1	0.47
cct	P	0	1.67
ccc	P	5	0.94
cca	P	0	1.69
ccg	P	0	0.71
cat	H	0	21414.00
cac	H	37966	16552.00
cgt	R	0	0.40
cgc	R	20	0.72
cga	R	0	1.82
cgg	R	0	1.72
aat	N	0	0.56
aac	N	1	0.44
aga	R	0	9.81
agg	R	0	5.53
---	--	------	------
mPD		0.0014	0.49
		nPD:	0.
		N. weight:	0.88
		Sc. PD:	-0.055
		Sc. rank:	-23088.0

,

PB1-F2

Pos.	53	obs:	exp:
cgt	R	0	0.46
cgc	R	0	0.83
cga	R	0	2.10
cgg	R	0	1.97
aaa	K	37970	24963.00
aag	K	0	13007.00
aga	R	23	11.28
agg	R	0	6.36
---	--	------	------
mPD		0.0012	0.45
		nPD:	0.
		N. weight:	0.44
		Sc. PD:	-0.028
		Sc. rank:	-12393.0

,

PB1-F2

Pos.	54	obs:	exp:
tta	L	0	0.46
ttg	L	0	0.83
ctt	L	0	0.84
ctc	L	0	0.59
cta	L	0	0.61
ctg	L	4	0.67
cct	P	0	0.33
ccc	P	0	0.19
cca	P	0	0.34
ccg	P	1	0.14
caa	Q	1	23172.00
cag	Q	37907	14736.00
cgt	R	0	1.59
cgc	R	0	2.88
cga	R	0	7.30
cgg	R	80	6.87
aga	R	0	39.24
agg	R	0	22.13
---	--	------	------
mPD		0.0045	0.48
		nPD:	0.01
		N. weight:	1.
		Sc. PD:	-0.057
		Sc. rank:	-16594.0

,

PB1-F2

Pos.	55	obs:	exp:
tct	S	0	0.32
tcc	S	0	0.30
tca	S	0	0.54
tcg	S	0	0.08
att	I	482	169.20
atc	I	0	126.70
ata	I	0	186.10
act	T	37506	11648.00
acc	T	0	6631.00
aca	T	0	16232.00
acg	T	0	2996.00
aat	N	3	1.68
aac	N	0	1.32
agt	S	2	0.35
agc	S	0	0.41
---	--	------	------
mPD		0.025	0.70
		nPD:	0.04
		N. weight:	1.2
		Sc. PD:	-0.039
		Sc. rank:	7371.0

,

PB1-F2

Pos.	56	obs:	exp:
atg	M	2	2.00
gtt	V	0	9447.00
gtc	V	0	7685.00
gta	V	1	8175.00
gtg	V	37716	12410.00
gct	A	0	74.68
gcc	A	0	68.46
gca	A	0	110.80
gcg	A	274	20.03
---	--	------	------
mPD		0.014	0.76
		nPD:	0.02
		N. weight:	1.2
		Sc. PD:	-0.056
		Sc. rank:	-3162.5

,

PB1-F2

Pos.	57	obs:	exp:
ttt	F	10	259.80
ttc	F	524	274.20
tct	S	1224	6023.00
tcc	S	36103	5578.00
tca	S	0	10033.00
tcg	S	0	1469.00
tat	Y	0	69.58
tac	Y	132	62.42
agt	S	0	6523.00
agc	S	0	7700.00
---	--	------	------
mPD		0.097	1.6
		nPD:	0.06
		N. weight:	1.8
		Sc. PD:	-0.02
		Sc. rank:	25002.0

,

PB1-F2

Pos.	58	obs:	exp:
tta	L	0	0.93
ttg	L	8	1.65
tgg	W	37985	37985.00
ctt	L	0	1.67
ctc	L	0	1.17
cta	L	0	1.23
ctg	L	0	1.34
---	--	------	------
mPD		0.00042	0.00097
		nPD:	0.43
		N. weight:	0.00035
		Sc. PD:	0.00012
		Sc. rank:	10.6

,

PB1-F2

Pos.	59	obs :	exp :
cgt	R	0	750.40
cgc	R	0	1362.00
cga	R	0	3453.00
cgg	R	0	3250.00
aaa	K	0	97.30
aag	K	148	50.70
aga	R	1	18562.00
agg	R	37844	10468.00
---	--	------	------
mPD		0.0078	0.89
		nPD :	0.01
		N. weight :	1.4
		Sc. PD :	-0.077
		Sc. rank :	-24962.0

PB1-F2

Pos.	60	obs :	exp :
tta	L	2	4348.00
ttg	L	0	7747.00
ctt	L	0	7838.00
ctc	L	0	5495.00
cta	L	37468	5754.00
ctg	L	0	6289.00
cct	P	0	149.60
ccc	P	0	84.18
cca	P	449	151.70
ccg	P	0	63.50
caa	Q	69	42.18
cag	Q	0	26.82
cgt	R	0	0.10
cgc	R	0	0.18
cga	R	5	0.46
cgg	R	0	0.43
aga	R	0	2.45
agg	R	0	1.38
---	--	------	------
mPD		0.027	1.2
		nPD :	0.02
		N. weight :	2.
		Sc. PD :	-0.086
		Sc. rank :	-1386.6

PB1-F2

Pos.	61	obs :	exp :
tgt	C	1	0.39
tgc	C	0	0.61
tgg	W	37992	37992.00
---	--	------	------
mPD		0.000053	0.000053
		nPD :	1.
		N. weight :	0.000026
		Sc. PD :	0.000022
		Sc. rank :	0.8

PB1-F2

Pos.	62	obs :	exp :
tta	L	0	67.54
ttg	L	0	120.30
tct	S	2	0.32
tcc	S	0	0.30
tca	S	0	0.54
tcg	S	0	0.08
ctt	L	582	121.70
ctc	L	0	85.34
cta	L	0	89.37
ctg	L	0	97.68
cct	P	37280	12430.00
ccc	P	20	6993.00
cca	P	0	12602.00
ccg	P	0	5276.00
cat	H	109	61.48
cac	H	0	47.52
agt	S	0	0.35
agc	S	0	0.41
---	--	------	------
mPD		0.037	0.77
		nPD :	0.05
		N. weight :	1.2
		Sc. PD :	-0.024
		Sc. rank :	10290.0

PB1-F2

Pos.	63	obs :	exp :
ttt	F	0	3029.00
ttc	F	6227	3198.00
tct	S	0	5120.00
tcc	S	31728	4741.00
tca	S	0	8528.00
tcg	S	0	1249.00
tat	Y	0	5.80
tac	Y	11	5.20
tgt	C	0	0.39
tgc	C	1	0.61
cct	P	0	7.66
ccc	P	23	4.31
cca	P	0	7.77
ccg	P	0	3.25
act	T	0	0.62
acc	T	2	0.35
aca	T	0	0.87
acg	T	0	0.16
agt	S	0	5545.00
agc	S	0	6545.00
gct	A	0	0.27
gcc	A	1	0.25
gca	A	0	0.40
gcg	A	0	0.07
---	--	------	------
mPD		0.28	1.7
		nPD :	0.16
		N. weight :	1.8
		Sc. PD :	0.15
		Sc. rank :	38101.0

PB1-F2

Pos.	64	obs :	exp :
tta	L	0	4405.00
ttg	L	37959	7848.00
tct	S	0	5.16
tcc	S	0	4.78
tca	S	0	8.60
tcg	S	32	1.26
tgg	W	2	2.00
ctt	L	0	7941.00
ctc	L	0	5566.00
cta	L	0	5829.00
ctg	L	0	6371.00
agt	S	0	5.59
agc	S	0	6.60
---	--	------	------
mPD		0.0018	1.2
		nPD :	0.
		N. weight :	1.7
		Sc. PD :	-0.11
		Sc. rank :	-48918.0

PB1-F2

Pos.	65	obs :	exp :
cgt	R	0	3.71
cgc	R	0	6.73
cga	R	0	17.06
cgg	R	0	16.06
act	T	0	0.31
acc	T	0	0.18
aca	T	0	0.43
acg	T	1	0.08
aaa	K	39	24853.00
aag	K	37764	12950.00
aga	R	0	91.72
agg	R	187	51.72
gaa	E	0	1.18
gag	E	2	0.82
---	--	------	------
mPD		0.012	0.46
		nPD :	0.03
		N. weight :	1.1
		Sc. PD :	-0.046
		Sc. rank :	1591.4

PB1-F2

Pos.	66	obs :	exp :
tct	S	0	11.94
tcc	S	0	11.06
tca	S	0	19.89
tcg	S	0	2.91
cat	H	1	0.56
cac	H	0	0.44
att	I	4	1.40
atc	I	0	1.05
ata	I	0	1.54
act	T	22	6.83
acc	T	0	3.89
aca	T	0	9.52
acg	T	0	1.76
aat	N	37887	21197.00
aac	N	0	16690.00
aaa	K	1	1.31
aag	K	1	0.69
agt	S	74	12.93
agc	S	0	15.27
gat	D	3	1.70
gac	D	0	1.30
---	--	------	------
mPD		0.0056	0.50
		nPD :	0.01
		N. weight :	0.62
		Sc. PD :	-0.034
		Sc. rank :	-7262.0

PB1-F2

Pos.	67	obs :	exp :
tta	L	0	8.70
ttg	L	0	15.51
tct	S	0	0.16
tcc	S	1	0.15
tca	S	0	0.27
tcg	S	0	0.04
ctt	L	0	15.69
ctc	L	75	11.00
cta	L	0	11.52
ctg	L	0	12.59
cct	P	0	12603.00
ccc	P	37819	7090.00
cca	P	1	12778.00
ccg	P	0	5349.00
cat	H	0	54.71
cac	H	97	42.29
agt	S	0	0.17
agc	S	0	0.21
---	--	------	------
mPD		0.0091	0.73
		nPD :	0.01
		N. weight :	1.8
		Sc. PD :	-0.095
		Sc. rank :	-16457.0

PB1-F2

Pos.	68	obs :	exp :
att	I	0	1159.00
atc	I	3301	867.40
ata	I	0	1275.00
act	T	1	10773.00
acc	T	34690	6133.00
aca	T	0	15013.00
acg	T	0	2771.00
aat	N	0	0.56
aac	N	1	0.44
---	--	------	------
mPD		0.16	0.84
		nPD :	0.19
		N. weight :	1.8
		Sc. PD :	0.2
		Sc. rank :	40227.0

PB1-F2

Pos.	69	obs :	exp :
tta	L	0	0.46
ttg	L	0	0.83
ctt	L	0	0.84
ctc	L	0	0.59
cta	L	0	0.61
ctg	L	4	0.67
cct	P	0	1.67
ccc	P	0	0.94
cca	P	0	1.69
ccg	P	5	0.71
cat	H	3	1.69
cac	H	0	1.31
caa	Q	4	23167.00
cag	Q	37896	14733.00
cgt	R	0	1.61
cgc	R	0	2.91
cga	R	0	7.39
cgg	R	81	6.96
aga	R	0	39.73
agg	R	0	22.40
---	--	------	------
mPD		0.0051	0.48
		nPD :	0.01
		N. weight :	1.
		Sc. PD :	-0.055
		Sc. rank :	-13824.0

PB1-F2

Pos.	70	obs :	exp :
att	I	0	1.05
atc	I	0	0.79
ata	I	3	1.16
gtt	V	0	9292.00
gtc	V	0	7559.00
gta	V	37094	8040.00
gtg	V	3	12206.00
gct	A	0	70.05
gcc	A	0	64.21
gca	A	257	104.00
gcg	A	0	18.79
gaa	E	13	7.66
gag	E	0	5.34
ggt	G	0	83.87
ggc	G	0	86.95
gga	G	623	287.60
ggg	G	0	164.60
---	--	------	------
mPD		0.046	0.79
		nPD :	0.06
		N. weight :	1.6
		Sc. PD :	-0.017
		Sc. rank :	19856.0

PB1-F2			
Pos.	71	obs:	exp:
ttt	F	131	63.72
ttc	F	0	67.28
tct	S	37420	6038.00
tcc	S	0	5592.00
tca	S	0	10058.00
tcg	S	0	1473.00
tat	Y	441	233.00
tac	Y	1	209.00
agt	S	0	6539.00
agc	S	0	7720.00
---	--	------	------
mPD		0.030	1.6
		nPD:	0.02
		N. weight:	1.9
		Sc. PD:	-0.092
		Sc. rank:	-8888.0

,

PB1-F2			
Pos.	72	obs:	exp:
tta	L	4	4405.00
ttg	L	37953	7847.00
tct	S	0	5.49
tcc	S	0	5.08
tca	S	0	9.14
tcg	S	34	1.34
ctt	L	0	7940.00
ctc	L	0	5566.00
cta	L	0	5829.00
ctg	L	0	6370.00
atg	M	1	1.00
agt	S	0	5.94
agc	S	0	7.01
---	-	1	1.00
---	--	------	------
mPD		0.0021	1.2
		nPD:	0.
		N. weight:	1.7
		Sc. PD:	-0.11
		Sc. rank:	-47058.0

,

PB1-F2			
Pos.	73	obs:	exp:
tct	S	0	0.16
tcc	S	0	0.15
tca	S	0	0.27
tcg	S	0	0.04
cgt	R	0	571.20
cgc	R	0	1037.00
cga	R	0	2629.00
cgg	R	0	2474.00
att	I	0	3.16
atc	I	0	2.36
ata	I	9	3.48
atg	M	1	1.00
aat	N	0	0.56
aac	N	1	0.44
aaa	K	9170	6029.00
aag	K	0	3141.00
agt	S	0	0.17
agc	S	1	0.21
aga	R	28782	14131.00
agg	R	28	7969.00
---	-	1	1.00
---	--	------	------
mPD		0.37	1.2
		nPD:	0.32
		N. weight:	0.67
		Sc. PD:	0.15
		Sc. rank:	19070.0

,

PB1-F2			
Pos.	74	obs:	exp:
cct	P	20	6.66
ccc	P	0	3.75
cca	P	0	6.76
ccg	P	0	2.83
att	I	277	97.24
atc	I	0	72.78
ata	I	0	107.00
act	T	37657	11696.00
acc	T	0	6659.00
aca	T	1	16299.00
acg	T	4	3008.00
aat	N	3	1.68
aac	N	0	1.32
gct	A	30	8.18
gcc	A	0	7.50
gca	A	0	12.14
gcg	A	0	2.19
---	-	1	1.00
---	--	------	------
mPD		0.018	0.70
		nPD:	0.03
		N. weight:	1.2
		Sc. PD:	-0.052
		Sc. rank:	830.9

,

PB1-F2			
Pos.	75	obs:	exp:
tta	L	0	3.95
ttg	L	0	7.03
ctt	L	34	7.11
ctc	L	0	4.99
cta	L	0	5.22
ctg	L	0	5.71
cct	P	29566	9853.00
ccc	P	0	5543.00
cca	P	1	9989.00
ccg	P	0	4182.00
cat	H	6816	3845.00
cac	H	0	2971.00
cgt	R	1576	31.25
cgc	R	0	56.71
cga	R	0	143.80
cgg	R	0	135.30
aga	R	0	773.00
agg	R	0	435.90
---	--	------	------
mPD		0.36	1.1
		nPD:	0.32
		N. weight:	1.2
		Sc. PD:	0.27
		Sc. rank:	34542.0

,

PB1-F2			
Pos.	76	obs:	exp:
gtt	V	0	36.82
gtc	V	146	29.95
gta	V	1	31.86
gtg	V	0	48.37
gct	A	11	10315.00
gcc	A	37815	9455.00
gca	A	19	15308.00
gcg	A	0	2767.00
ggt	G	0	0.13
ggc	G	1	0.14
gga	G	0	0.46
ggg	G	0	0.26
---	--	------	------
mPD		0.0094	0.70
		nPD:	0.01
		N. weight:	1.5
		Sc. PD:	-0.077
		Sc. rank:	-10835.0

,

PB1-F2			
Pos.	77	obs:	exp:
tta	L	1	0.12
ttg	L	0	0.21
ctt	L	0	0.21
ctc	L	0	0.15
cta	L	0	0.15
ctg	L	0	0.17
---	-	37992	37992.00
---	--	------	------
mPD		0	8.0×10^{-10}
		nPD:	0.
		N. weight:	0.
		Sc. PD:	0
		Sc. rank:	0

,

PB1-F2			
Pos.	78	obs:	exp:
atg	M	1	1.00
---	-	37992	37992.00
---	--	------	------
mPD		0	0
		nPD:	1.
		N. weight:	0.
		Sc. PD:	0
		Sc. rank:	0

,

PB1-F2			
Pos.	79	obs:	exp:
aat	N	1	0.56
aac	N	0	0.44
---	-	37992	37992.00
---	--	------	------
mPD		0	3.4×10^{-10}
		nPD:	0.
		N. weight:	0.
		Sc. PD:	0
		Sc. rank:	0

,

PB1-F2			
Pos.	80	obs:	exp:
tta	L	0	4407.00
ttg	L	37973	7851.00
tct	S	0	2.10
tcc	S	0	1.94
tca	S	0	3.49
tcg	S	13	0.51
tgg	W	2	2.00
ctt	L	0	7944.00
ctc	L	0	5569.00
cta	L	0	5831.00
ctg	L	1	6373.00
caa	Q	0	0.61
cag	Q	1	0.39
atg	M	2	2.00
agt	S	0	2.27
agc	S	0	2.68
gtt	V	0	0.25
gtc	V	0	0.20
gta	V	0	0.22
gtg	V	1	0.33
---	--	------	------
mPD		0.0011	1.2
		nPD:	0.
		N. weight:	1.7
		Sc. PD:	-0.11
		Sc. rank:	-50714.0

,

PB1-F2			
Pos.	81	obs:	exp:
cgt	R	0	1.01
cgc	R	0	1.84
cga	R	0	4.65
cgg	R	0	4.38
act	T	0	0.31
acc	T	0	0.18
aca	T	1	0.43
acg	T	0	0.08
aaa	K	37937	24942.00
aag	K	2	12997.00
aga	R	51	25.01
agg	R	0	14.11
gaa	E	2	1.18
gag	E	0	0.82
---	--	------	------
mPD		0.0029	0.45
		nPD:	0.01
		N. weight:	0.44
		Sc. PD:	-0.026
		Sc. rank:	-9193.6

,

PB1-F2			
Pos.	82	obs:	exp:
tta	L	0	3.02
ttg	L	0	5.38
ctt	L	0	5.44
ctc	L	0	3.81
cta	L	26	3.99
ctg	L	0	4.36
cct	P	0	0.33
ccc	P	0	0.19
cca	P	1	0.34
ccg	P	0	0.14
cat	H	2	1.13
cac	H	0	0.87
caa	Q	37884	23158.00
cag	Q	0	14726.00
cgt	R	0	1.57
cgc	R	0	2.84
cga	R	78	7.21
cgg	R	1	6.78
aga	R	0	38.75
agg	R	0	21.85
ggt	G	0	0.13
ggc	G	1	0.14
gga	G	0	0.46
ggg	G	0	0.26
---	--	------	------
mPD		0.0059	0.49
		nPD:	0.01
		N. weight:	0.52
		Sc. PD:	-0.028
		Sc. rank:	-5810.9

,

PB1-F2

Pos.	83	obs:	exp:
tta	L	0	0.93
ttg	L	8	1.65
tct	S	0	0.16
tcc	S	0	0.15
tca	S	0	0.27
tcg	S	1	0.04
tgg	W	35407	35407.00
ctt	L	0	1.67
ctc	L	0	1.17
cta	L	0	1.23
ctg	L	0	1.34
cgt	R	0	0.04
cgc	R	0	0.07
cga	R	0	0.18
cgg	R	1	0.17
agt	S	0	0.17
agc	S	0	0.21
aga	R	0	0.98
agg	R	1	0.55
ggt	G	0	0.13
ggc	G	0	0.14
gga	G	0	0.46
ggg	G	1	0.26
---	-	2574	2574.00
---	--	------	------
mPD		0.00059	0.0012
		nPD:	0.47
		N. weight:	0.00056
		Sc. PD:	0.00021
		Sc. rank:	17.1

,

PB1-F2

Pos.	84	obs:	exp:
cgt	R	0	2.04
cgc	R	0	3.71
cga	R	0	9.40
cgg	R	0	8.84
atg	M	3	3.00
act	T	0	0.31
acc	T	0	0.18
aca	T	0	0.43
acg	T	1	0.08
aat	N	20	11.19
aac	N	0	8.81
aaa	K	197	23201.00
aag	K	35093	12089.00
aga	R	2	50.52
agg	R	101	28.49
gaa	E	0	1.18
gag	E	2	0.82
---	-	2574	2574.00
---	--	------	------
mPD		0.016	0.40
		nPD:	0.04
		N. weight:	1.
		Sc. PD:	-0.029
		Sc. rank:	6764.8

,

PB1-F2

Pos.	85	obs:	exp:
tta	L	0	40.96
ttg	L	253	72.98
tct	S	0	97.30
tcc	S	0	90.11
tca	S	4	162.10
tcg	S	599	23.74
ctt	L	0	73.84
ctc	L	0	51.76
cta	L	0	54.21
ctg	L	100	59.24
cct	P	0	11343.00
ccc	P	0	6381.00
cca	P	205	11500.00
ccg	P	33833	4814.00
caa	Q	3	256.10
cag	Q	416	162.90
cgt	R	0	0.04
cgc	R	0	0.07
cga	R	0	0.18
cgg	R	2	0.17
act	T	0	0.31
acc	T	0	0.18
aca	T	0	0.43
acg	T	1	0.08
agt	S	0	105.40
agc	S	0	124.40
aga	R	0	0.98
agg	R	0	0.55
---	-	2577	2577.00
---	--	------	------
mPD		0.089	0.71
		nPD:	0.13
		N. weight:	1.9
		Sc. PD:	0.094
		Sc. rank:	36075.0

,

PB1-F2

Pos.	86	obs:	exp:
ttt	F	0	17063.00
ttc	F	35078	18015.00
tta	L	0	1.04
ttg	L	0	1.86
tct	S	0	52.12
tcc	S	323	48.27
tca	S	0	86.82
tcg	S	0	12.71
tat	Y	0	1.58
tac	Y	3	1.42
tgt	C	0	1.17
tgc	C	3	1.83
ctt	L	0	1.88
ctc	L	9	1.32
cta	L	0	1.38
ctg	L	0	1.51
agt	S	0	56.45
agc	S	0	66.63
---	-	2577	2577.00
---	--	------	------
mPD		0.016	0.46
		nPD:	0.04
		N. weight:	0.67
		Sc. PD:	-0.021
		Sc. rank:	3444.5

,

PB1-F2

Pos.	87	obs:	exp:
tct	S	0	185.90
tcc	S	0	172.20
tca	S	0	309.60
tcg	S	0	45.35
aat	N	0	19168.00
aac	N	34261	15093.00
aaa	K	2	1.97
aag	K	1	1.03
agt	S	0	201.30
agc	S	1152	237.70
---	-	2577	2577.00
---	--	------	------
mPD		0.055	0.53
		nPD:	0.1
		N. weight:	0.83
		Sc. PD:	0.025
		Sc. rank:	14494.0

,

PB1-F2

Pos.	88	obs:	exp:
cgt	R	0	664.60
cgc	R	0	1206.00
cga	R	0	3058.00
cgg	R	0	2878.00
act	T	0	0.31
acc	T	0	0.18
aca	T	1	0.43
acg	T	0	0.08
aaa	K	1893	1245.00
aag	K	1	648.80
aga	R	33516	16441.00
agg	R	3	9271.00
ggt	G	0	0.13
ggc	G	0	0.14
gga	G	1	0.46
ggg	G	0	0.26
---	-	2578	2578.00
---	--	------	------
mPD		0.088	0.84
		nPD:	0.1
		N. weight:	0.68
		Sc. PD:	0.021
		Sc. rank:	12252.0

,

PB1-F2

Pos.	89	obs:	exp:
tta	L	0	3.60
ttg	L	0	6.41
ctt	L	0	6.48
ctc	L	0	4.55
cta	L	31	4.76
ctg	L	0	5.20
cct	P	0	1.00
ccc	P	0	0.56
cca	P	3	1.01
ccg	P	0	0.42
cat	H	0	2.26
cac	H	4	1.74
caa	Q	35321	21591.00
cag	Q	0	13730.00
cgt	R	0	1.11
cgc	R	0	2.01
cga	R	56	5.11
cgg	R	0	4.81
aga	R	0	27.47
agg	R	0	15.49
---	-	2578	2578.00
---	--	------	------
mPD		0.0046	0.42
		nPD:	0.01
		N. weight:	0.49
		Sc. PD:	-0.027
		Sc. rank:	-6215.5

,

PB1-F2

Pos.	90	obs:	exp:
cgt	R	0	0.08
cgc	R	0	0.14
cga	R	0	0.36
cgg	R	0	0.34
aga	R	0	1.96
agg	R	4	1.11
gaa	E	0	225.80
gag	E	383	157.20
ggt	G	0	4716.00
ggc	G	0	4889.00
gga	G	17	16169.00
ggg	G	35010	9254.00
---	-	2579	2579.00
---	--	------	------
mPD		0.020	0.61
		nPD:	0.03
		N. weight:	1.3
		Sc. PD:	-0.046
		Sc. rank:	4572.2

,

PB1-F2

Pos.	91	obs:	exp:
tta	L	0	6.61
ttg	L	57	11.78
tgg	W	26608	26608.00
ctt	L	0	11.92
ctc	L	0	8.36
cta	L	0	8.75
ctg	L	0	9.57
---	-	11328	11328.00
---	--	------	------
mPD		0.0021	0.0048
		nPD:	0.43
		N. weight:	0.
		Sc. PD:	0
		Sc. rank:	0

,

PB1-F2

Pos.	92	obs:	exp:
cgt	R	0	0.02
cgc	R	0	0.04
cga	R	0	0.09
cgg	R	0	0.09
att	I	0	91.97
atc	I	0	68.84
ata	I	262	101.20
act	T	0	8199.00
acc	T	0	4668.00
aca	T	26402	11426.00
acg	T	0	2109.00
aga	R	1	0.49
agg	R	0	0.28
---	-	11328	11328.00
---	--	------	------
mPD		0.0096	0.34
		nPD:	0.03
		N. weight:	0.
		Sc. PD:	0
		Sc. rank:	0

,

PB1-F2

Pos.	93	obs:	exp:
tct	S	0	3.07
tcc	S	0	2.84
tca	S	0	5.11
tcg	S	0	0.75
act	T	0	0.31
acc	T	1	0.18
aca	T	0	0.43
acg	T	0	0.08
aat	N	50	14871.00
aac	N	26531	11710.00
aaa	K	2	1.31
aag	K	0	0.69
agt	S	0	3.32
agc	S	19	3.92
gat	D	0	35.05
gac	D	62	26.95
ggt	G	0	0.13
ggc	G	0	0.14
gga	G	0	0.46
ggg	G	1	0.26
---	-	11327	11327.00
---	--	------	------
mPD		0.0051	0.25
		nPD:	0.02
		N. weight:	0.
		Sc. PD:	0
		Sc. rank:	0

,

PB1-F2

Pos.	94	obs:	exp:
taa	.	26496	21583.00
tag	.	11325	4162.00
tga	.	172	12249.00
---	--	------	------
mPD		0.43	0.63
		nPD:	0.68
		N. weight:	0.45
		Sc. PD:	0.25
		Sc. rank:	13670.0

}

Amino acid level conservation per codon

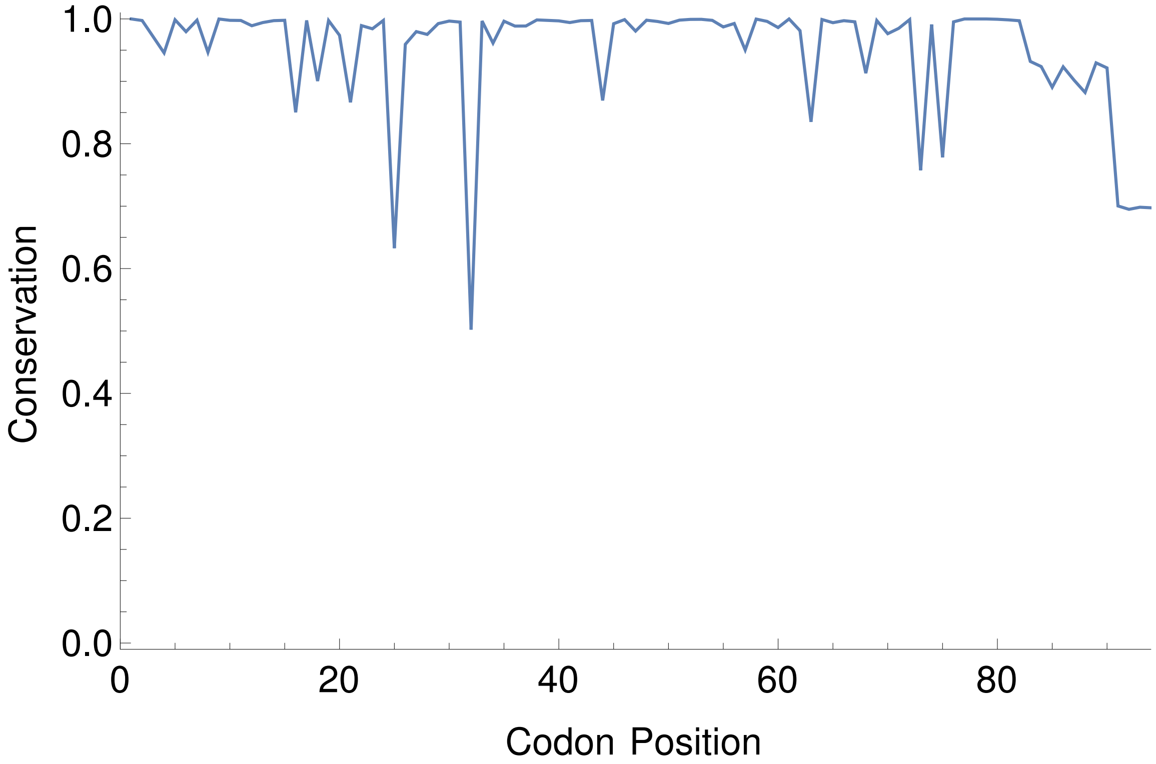

Conservation analysis plots

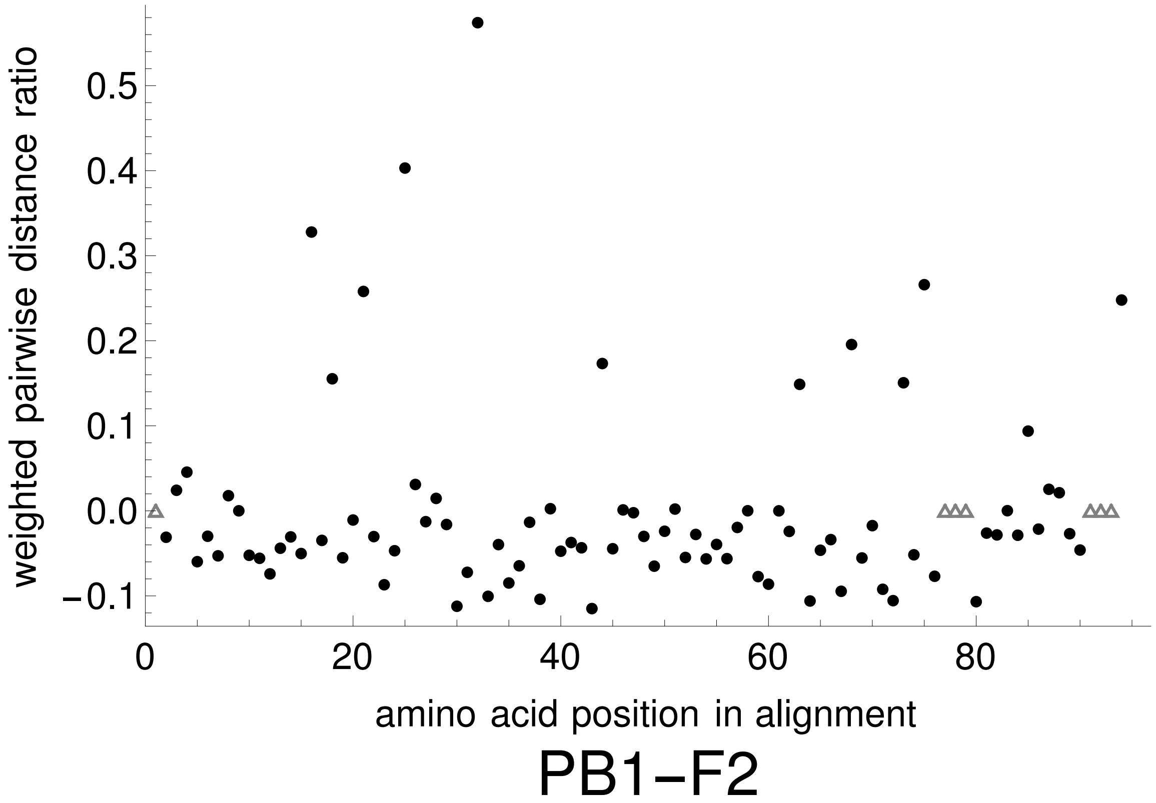

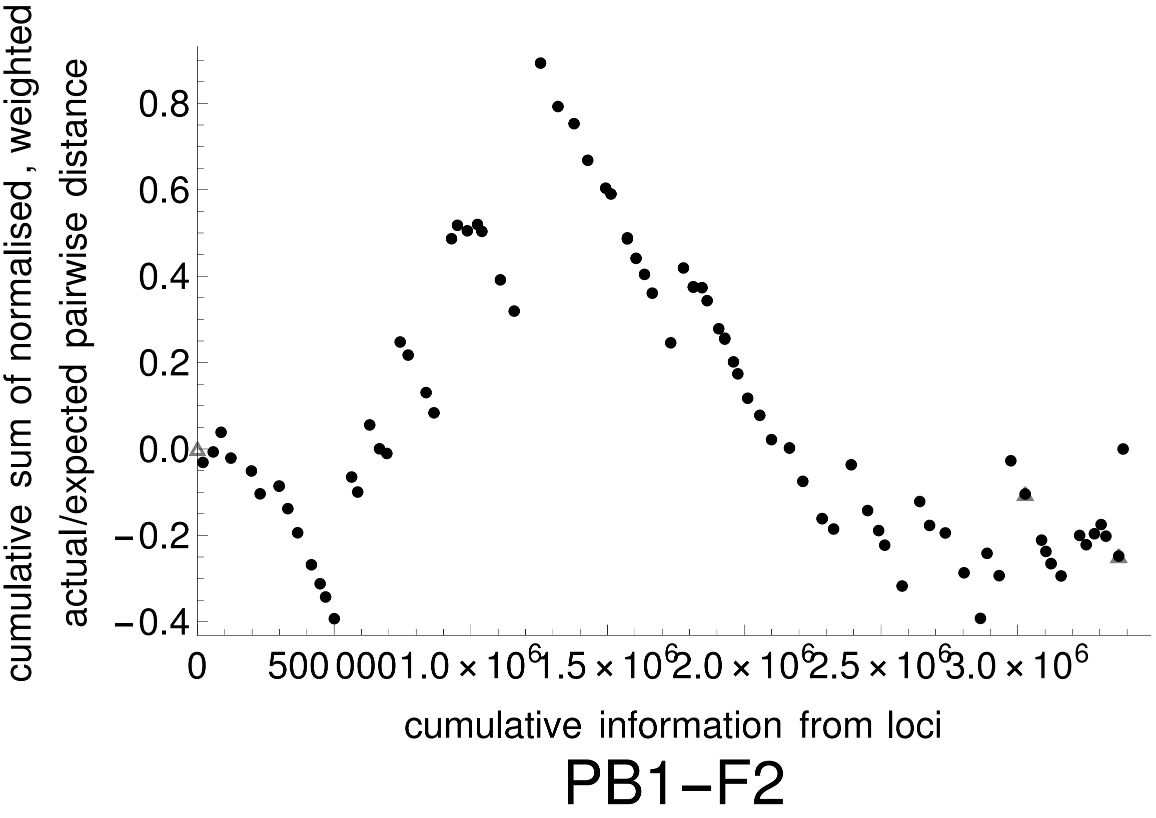

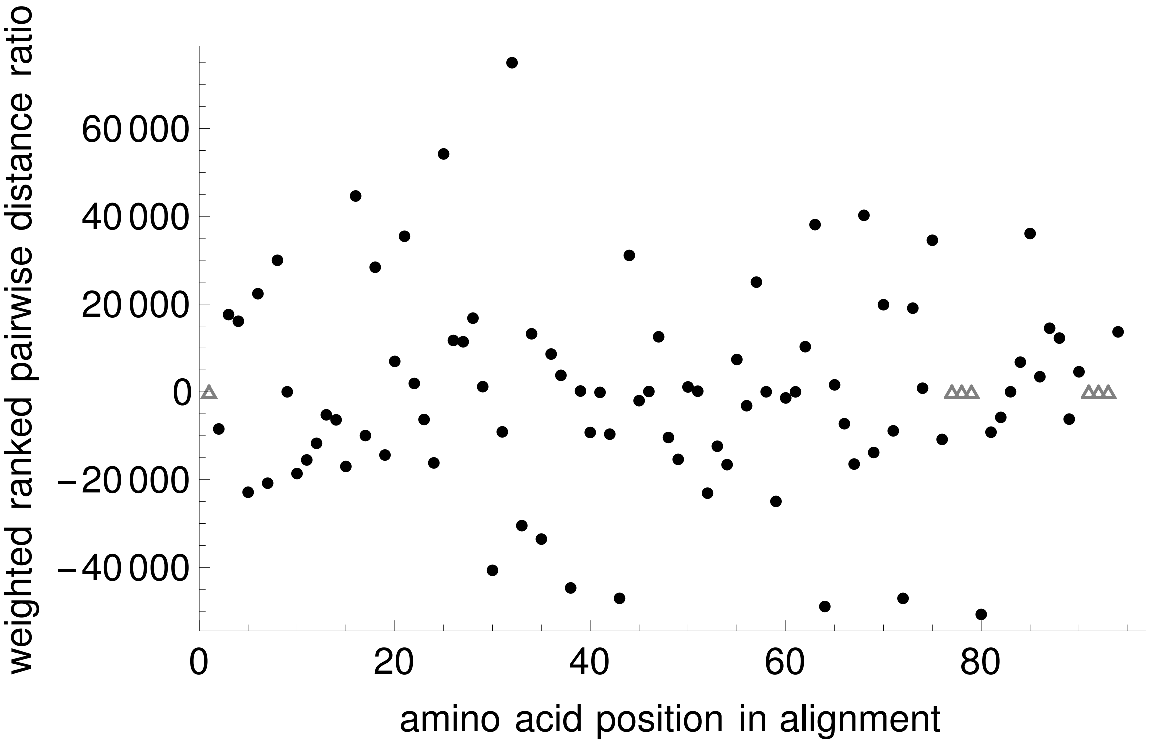
weighted ranked pairwise distance ratio
60 000
40 000
20 000
0
−20 000
−40 000
0
20
40
60
80
amino acid position in alignment

PB1−F2

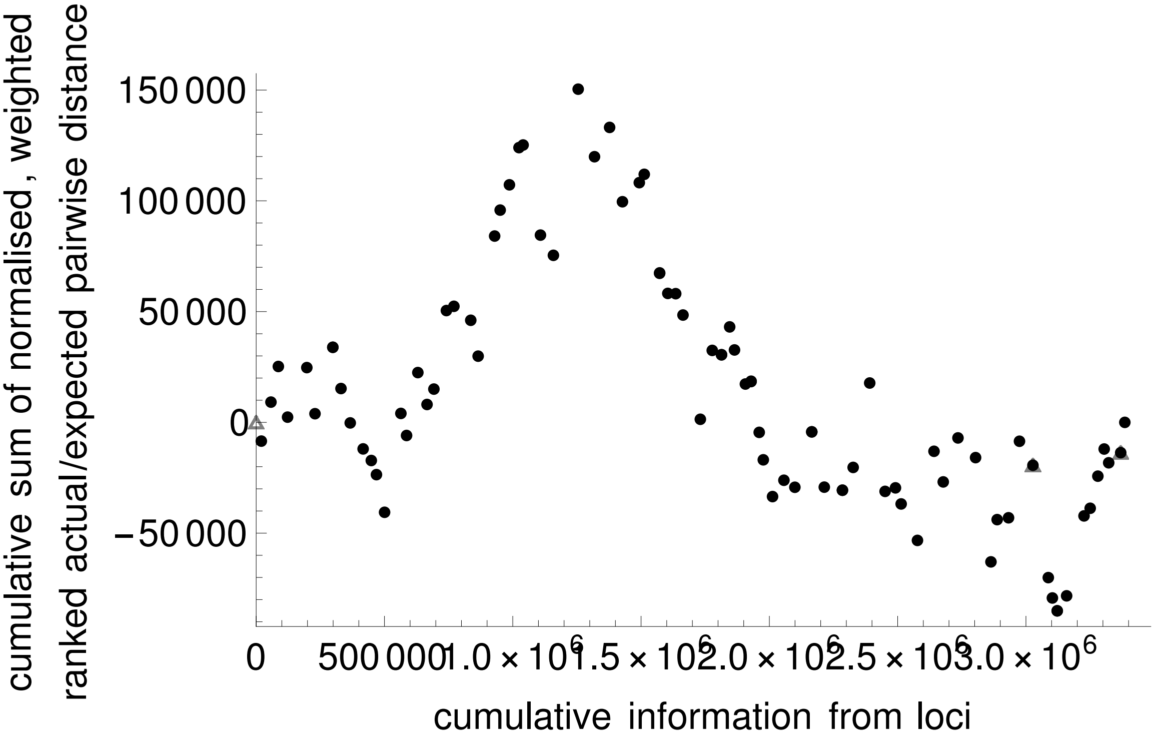
cumulative sum of normalised, weighted
ranked actual/expected pairwise distance
150 000
100 000
50 000
0
−50 000
0
500 000
1.0 × 10^6
1.5 × 10^6
2.0 × 10^6
2.5 × 10^6
3.0 × 10^6
cumulative information from loci

PB1−F2

PA

Gene length histogram

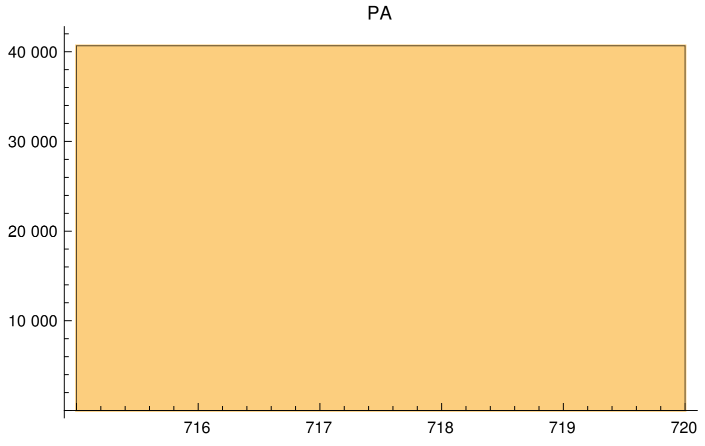

Information vs. nPD

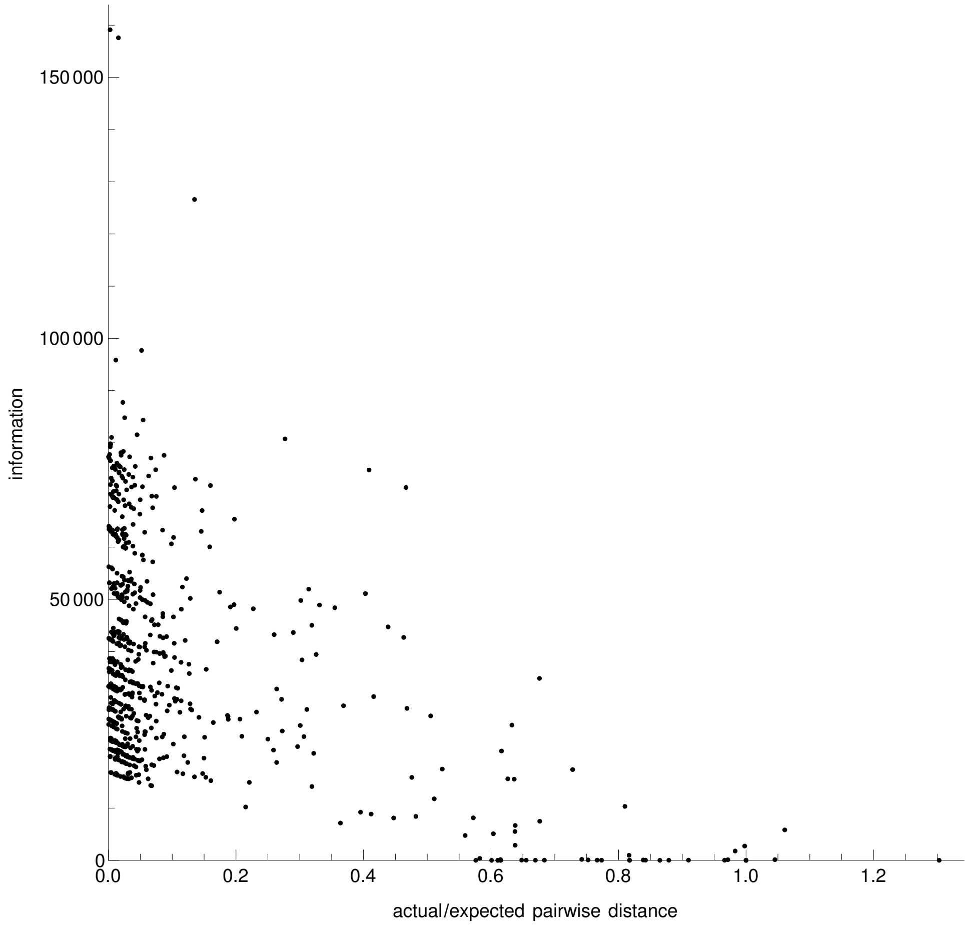

PA

Example sequences highlighted by regions found in analyses to be conserved

Interesting points (by weighted raw PD) highlighted for gene PA:

ATGGAAGATTTTGTGCGACAATGCTTCAACCCGATGATTGTCGAACTTGCAGAAAAAGCAATGAAAGAGTATGGGG⋱
AGGATCTGAAAATTGAAACCAACAAATTTGCAGCAATATGCACTCACTTGGAGGTGTGTTTCATGTATTCAGATT⋱
TCCATTTCATCAATGAACAAGGCGAATCAATAGTAGTAGAACTTGACGATCCAAATGCACTGTTAAAACACAGAT⋱
TTGAAATAATCGAGGGGAGAGACAGAACAATGGCCTGGACAGTAGTAAACAGTATCTGCAACACTACTGGAGCTG⋱
GAAAACCGAAGTTCTTACCGGATTTGTATGATTACAAAGAAAACAGATTCATCGAAATTGGAGTGACAAGGAGAG⋱
AAGTCCACATATATTACCTTGAAAAGGCCAATAAGATTAAATCTGAGAACACACACATTCACATTTTTTCATTCA⋱
CTGGGGAGGAAATGGCCACAAGGGCAGACTACACTCTCGACGAGGAAAGCAGG -------------------------
-------GCTAGGATCAAAACCAGGCTGTTT
ACCATAAGACAAGAAATGGCCAACAAAGGCCTCTGGGATTCCTTTCGTCAGTCCG
AAAGAGGCGAAGAAACAATTGAAGAAAAATTTGAAATCACAGGAACTATGCG
CAGGCTTGCCGACCAAAGTCTCCCACCGAACTTCTCCTGCCTTGAGAATTTT
AGAGCCTATGTGGATGGATTCGAACCGAACGGCTGCATTGAGGGCAAGCTTT
CTCAAATGTCCAAAGAAGTGAATGCCCAAATTGAACCTTTTCTGAAGACAACACCA⋱
AGACCAATCAAACTTCCTAGTGGACCTCCTTGTTATCAGCGATCCAAATTCCTCCTGATGGATGCTTTGA⋱
AATTGAGCATTGAAGACCCAAGTCACGAAGGAGAAGGGATCCCATTATATGATGCAATCAAGTGCATAAA⋱
AACATTCTTTGGATGGAAAGAACCTTATATAGTCAAACCACACGAAAAGGGAATAAATTCAAATTACCTG⋱
CTGTCATGGAAGCAAGTACTGTCAGAATTGCAAGACATTGAGAATGAGGAGAAGATCCCAAGAACTAAAA⋱
ACATGAAGAAAACGAGTCAACTGAAGTGGGCTCTTGGTGAAAACATGGCACCAGAGAAGGTAGACTTTGA⋱
AAACTGCAGAGACATAAGCGATTTGAAGCAATATGATAGTGAAGAACCTGAATTAAGGTCACTTTCAAGC⋱
TGGATACAAAGTGAGTTCAACAAGGCCTGTGAGCTAACTGATTCAGTCTGGATAGAGCTCGATGAGATTG⋱
GAGAGGACGTAGCCCCAATTGAACACATTGCAAGCATGAGAAGGAATTATTTCACAGCAGAGGTGTCCCA⋱
TTGTAGAGCTACTGAATACATAATGAAAGGGGTATACATTAATACTGCCCTGCTCAATGCATCCTGTGCA⋱
GCAATGGACGATTTTCAACTAATTCCCATGATAAGCAAGTGCAGAACTAAAGAGGGAAGGCGAAAAACCA⋱
ATTTATATGGATTCATCATAAAGGGAAGATCTCATCTGAGGAATGACACAGACGTGGTAAACTTTGTGAG⋱
CATGGAGTTTTCTCTCACAGATCCGAGACTCGAACCACATAAATGGGAGAAATATTGTGTCCTTGAGATA⋱
GGAGATATGTTACTAAGGAGTGCTATAGGCCAAATTTCAAGGCCGATGTTCTTGTATGTGAGGACAAACG⋱
GAACATCAAAGGTCAAAATGAAATGGGGAATGGAGATGAGACGTTGCCTCCTTCAGTCACTCCAGCAGAT⋱
CGAGAGCATGATTGAAGCCGAGTCCTCAGTTAAAGAGAAAGACATGACCAAAGAGTTTTTTGAGAATAAA⋱
TCAGAAGCATGGCCCATTGGGGAGTCCCCCAAAGGAGTGGAAGAAGGTTCCATTGGGAAAGTCTGTAGGA⋱
CTCTATTGGCTAAGTCAGTATTCAATAGCCTGTATGCATCACCACAATTGGAAGGATTTTCAGCAGAGTC⋱
AAGAAAACTGCTCCTTATTGTTCAGGCTCTTAGGGACAAACTCGAA ---CCTGGGACC
TTTGATCTTGGGGGGCTATATGAAGCAATTGAGGAGTGCCTGATTAATGATCCCT
GGGTTTTGCTCAATGCGTCTTGGTTCAACTCCTTCCTGACACATGCATTAAAA ------TAG

Interesting points (by weighted ranked PD) highlighted for gene PA:

ATGGAAGATTTTGTGCGACAATGCTTCAACCCGATGATTGTCGAACTTGCAGAAAAAGCAATGAAAGAGTATGGGGAG⋱
GATCTGAAAATTGAAACCAACAAATTTGCAGCAATATGCACTCACTTGGAGGTGTGTTTCATGTATTCAGATTTCCA⋱
TTTCATCAATGAACAAGGCGAATCAATAGTAGTAGAACTTGACGATCCAAATGCACTGTTAAAACACAGATTTGAAA⋱
TAATCGAGGGGAGAGACAGAACAATGGCCTGGACAGTAGTAAACAGTATCTGCAACACTACTGGAGCTGGAAAACCG⋱
AAGTTCTTACCGGATTTGTATGATTACAAAGAAAACAGATTCATCGAAATTGGAGTGACAAGGAGAGAAGTCCACAT⋱
ATATTACCTTGAAAAGGCCAATAAGATTAAATCTGAGAACACACACATTCACATTTTTTCATTCACTGGGGAGGAAA⋱
TGGCCACAAGGGCAGACTACACTCTCGACGAGGAAAGCAGG --------------------------------
GCTAGGATCAAAACCAGGCTGTTTACCATAAGACAAGAAATGGCCAACAAAGGCCTCTGG

GATTCCTTTCGTCAGTCCGAAAGAGGCGAAGAAACAATTGAAGAAAAATTTGAAA
TCACAGGAACTATGCGCAGGCTTGCCGACCAAAGTCTCCCACCGAACTTCTC
CTGCCTTGAGAATTTTAGAGCCTATGTGGATGGATTCGAACCGAACGGCTGC
ATTGAGGGCAAGCTTTCTCAAATGTCCAAAGAAGTGAATGCCCAAATTGAACCTTTTCTGAAG∵
ACAACACCAAGACCAATCAAACTTCCTAGTGGACCTCCTTGTTATCAGCGATCCAAATTCCTCCTGATGG∵
ATGCTTTGAAATTGAGCATTGAAGACCCAAGTCACGAAGGAGAAGGGATCCCATTATATGATGCAATCAA∵
GTGCATAAAAACATTCTTTGGATGGAAAGAACCTTATATAGTCAAACCACACGAAAAGGGAATAAATTCA∵
AATTACCTGCTGTCATGGAAGCAAGTACTGTCAGAATTGCAAGACATTGAGAATGAGGAGAAGATCCCAA∵
GAACTAAAAACATGAAGAAAACGAGTCAACTGAAGTGGGCTCTTGGTGAAAACATGGCACCAGAGAAGGT∵
AGACTTTGAAAACTGCAGAGACATAAGCGATTTGAAGCAATATGATAGTGAAGAACCTGAATTAAGGTCA∵
CTTTCAAGCTGGATACAAAGTGAGTTCAACAAGGCCTGTGAGCTAACTGATTCAGTCTGGATAGAGCTCG∵
ATGAGATTGGAGAGGACGTAGCCCCAATTGAACACATTGCAAGCATGAGAAGGAATTATTTCACAGCAGA∵
GGTGTCCCATTGTAGAGCTACTGAATACATAATGAAAGGGGTATACATTAATACTGCCCTGCTCAATGCA∵
TCCTGTGCAGCAATGGACGATTTTCAACTAATTCCCATGATAAGCAAGTGCAGAACTAAAGAGGGAAGGC∵
GAAAAACCAATTTATATGGATTCATCATAAAGGGAAGATCTCATCTGAGGAATGACACAGACGTGGTAAA∵
CTTTGTGAGCATGGAGTTTTCTCTCACAGATCCGAGACTCGAACCACATAAATGGGAGAAATATTGTGTC∵
CTTGAGATAGGAGATATGTTACTAAGGAGTGCTATAGGCCAAATTTCAAGGCCGATGTTCTTGTATGTGA∵
GGACAAACGGAACATCAAAGGTCAAAATGAAATGGGGAATGGAGATGAGACGTTGCCTCCTTCAGTCACT∵
CCAGCAGATCGAGAGCATGATTGAAGCCGAGTCCTCAGTTAAAGAGAAAGACATGACCAAAGAGTTTTTT∵
GAGAATAAATCAGAAGCATGGCCCATTGGGGAGTCCCCCAAAGGAGTGGAAGAAGGTTCCATTGGGAAAG∵
TCTGTAGGACTCTATTGGCTAAGTCAGTATTCAATAGCCTGTATGCATCACCACAATTGGAAGGATTTTC∵
AGCAGAGTCAAGAAAACTGCTCCTTATTGTTCAGGCTCTTAGGGACAAACTCGAA ---CCTGGGACC
TTTGATCTTGGGGGGCTATATGAAGCAATTGAGGAGTGCCTGATTAATGATCCCT
GGGTTTTGCTCAATGCGTCTTGGTTCAACTCCTTCCTGACACATGCATTAAAA ------
TAG

Per codon conservation report

PA

Pos.	1	obs:	exp:
atg	M	40665	40665.00
---	--	------	------
mPD		0	0
		nPD:	1.
		N. weight:	0.
		Sc. PD:	0
		Sc. rank:	0

PA

Pos.	2	obs:	exp:
gaa	E	40595	23973.00
gag	E	68	16690.00
ggt	G	0	0.27
ggc	G	0	0.28
gga	G	2	0.92
ggg	G	0	0.53
---	--	------	------
mPD		0.0034	0.48
		nPD:	0.01
		N. weight:	0.57
		Sc. PD:	-0.027
		Sc. rank:	-11261.0

PA

Pos.	3	obs:	exp:
tat	Y	2	1.05
tac	Y	0	0.95
aat	N	16	8.95
aac	N	0	7.05
gct	A	0	0.27
gcc	A	1	0.25
gca	A	0	0.40
gcg	A	0	0.07
gat	D	40430	22971.00
gac	D	208	17667.00
gaa	E	2	1.18
gag	E	0	0.82
ggt	G	6	0.81
ggc	G	0	0.84
gga	G	0	2.77
ggg	G	0	1.59
---	--	------	------
mPD		0.012	0.49
		nPD:	0.02
		N. weight:	0.6
		Sc. PD:	-0.019
		Sc. rank:	-357.9

PA

Pos.	4	obs:	exp:
ttt	F	40632	19779.00
ttc	F	29	20882.00
tta	L	1	0.46
ttg	L	0	0.83
ctt	L	3	0.84
ctc	L	0	0.59
cta	L	0	0.61
ctg	L	0	0.67
---	--	------	------
mPD		0.0016	0.50
		nPD:	0.
		N. weight:	0.79
		Sc. PD:	-0.041
		Sc. rank:	-20626.0

PA

Pos.	5	obs:	exp:
tta	L	0	0.23
ttg	L	2	0.41
ctt	L	0	0.42
ctc	L	0	0.29
cta	L	0	0.31
ctg	L	0	0.34
atg	M	2	2.00
gtt	V	0	10184.00
gtc	V	0	8285.00
gta	V	110	8813.00
gtg	V	40549	13378.00
gct	A	0	0.55
gcc	A	0	0.50
gca	A	0	0.81
gcg	A	2	0.15
---	--	------	------
mPD		0.0057	0.74
		nPD:	0.01
		N. weight:	1.2
		Sc. PD:	-0.057
		Sc. rank:	-22872.0

PA

Pos.	6	obs:	exp:
cgt	R	2	806.30
cgc	R	3	1463.00
cga	R	34861	3710.00
cgg	R	5763	3492.00
aga	R	34	19946.00
agg	R	2	11248.00
---	--	------	------
mPD		0.25	0.89
		nPD:	0.28
		N. weight:	2.2
		Sc. PD:	0.47
		Sc. rank:	63617.0

PA

Pos.	7	obs:	exp:
tta	L	0	0.12
ttg	L	0	0.21
ctt	L	0	0.21
ctc	L	0	0.15
cta	L	1	0.15
ctg	L	0	0.17
caa	Q	40244	24855.00
cag	Q	416	15805.00
cgt	R	0	0.06
cgc	R	0	0.11
cga	R	3	0.27
cgg	R	0	0.26
aaa	K	1	0.66
aag	K	0	0.34
aga	R	0	1.47
agg	R	0	0.83
---	--	------	------
mPD		0.020	0.48
		nPD:	0.04
		N. weight:	0.49
		Sc. PD:	-0.0061
		Sc. rank:	5890.9

PA

Pos.	8	obs:	exp:
tct	S	0	0.81
tcc	S	1	0.75
tca	S	0	1.34
tcg	S	0	0.20
tat	Y	0	3.69
tac	Y	7	3.31
tgt	C	118	15886.00
tgc	C	40534	24766.00
cat	H	0	0.56
cac	H	1	0.44
agt	S	0	0.87
agc	S	4	1.03
---	--	------	------
mPD		0.0065	0.48
		nPD:	0.01
		N. weight:	0.53
		Sc. PD:	-0.022
		Sc. rank:	-6194.6

PA

Pos.	9	obs:	exp:
ttt	F	61	19780.00
ttc	F	40603	20884.00
tat	Y	0	0.53
tac	Y	1	0.47
---	--	------	------
mPD		0.0030	0.50
		nPD:	0.01
		N. weight:	0.73
		Sc. PD:	-0.035
		Sc. rank:	-15806.0

PA

Pos.	10	obs:	exp:
tct	S	0	0.32
tcc	S	0	0.30
tca	S	0	0.54
tcg	S	0	0.08
att	I	0	0.35
atc	I	1	0.26
ata	I	0	0.39
aat	N	3532	22749.00
aac	N	37130	17913.00
agt	S	0	0.35
agc	S	2	0.41
---	--	------	------
mPD		0.16	0.49
		nPD:	0.32
		N. weight:	0.56
		Sc. PD:	0.14
		Sc. rank:	16630.0

PA

Pos.	11	obs:	exp:
tta	L	0	0.12
ttg	L	0	0.21
ctt	L	0	0.21
ctc	L	0	0.15
cta	L	0	0.15
ctg	L	1	0.17
cct	P	141	13550.00
ccc	P	0	7623.00
cca	P	479	13738.00
ccg	P	40043	5751.00
ggt	G	0	0.13
ggc	G	0	0.14
gga	G	0	0.46
ggg	G	1	0.26
---	--	------	------
mPD		0.030	0.72
		nPD:	0.04
		N. weight:	2.1
		Sc. PD:	-0.028
		Sc. rank:	24405.0

PA

Pos.	12	obs:	exp:
tta	L	0	0.23
ttg	L	2	0.41
ctt	L	0	0.42
ctc	L	0	0.29
cta	L	0	0.31
ctg	L	0	0.34
att	I	6	11.94
atc	I	0	8.93
ata	I	28	13.13
atg	M	40629	40629.00
---	--	------	------
mPD		0.0018	0.0018
		nPD:	0.97
		N. weight:	0.0006
		Sc. PD:	0.00053
		Sc. rank:	19.0

PA

Pos.	13	obs:	exp:
att	I	40330	14259.00
atc	I	288	10673.00
ata	I	2	15687.00
gtt	V	30	11.27
gtc	V	15	9.17
gta	V	0	9.75
gtg	V	0	14.81
---	--	------	------
mPD		0.017	0.66
		nPD:	0.03
		N. weight:	1.1
		Sc. PD:	-0.033
		Sc. rank:	1664.2

PA

Pos.	14	obs:	exp:
att	I	0	1.76
atc	I	5	1.31
ata	I	0	1.93
gtt	V	189	10184.00
gtc	V	40459	8285.00
gta	V	11	8813.00
gtg	V	0	13378.00
gct	A	0	0.27
gcc	A	1	0.25
gca	A	0	0.40
gcg	A	0	0.07
---	--	------	------
mPD		0.010	0.74
		nPD:	0.01
		N. weight:	1.7
		Sc. PD:	-0.071
		Sc. rank:	-19882.0

PA

Pos.	15	obs:	exp:
gat	D	11	6.78
gac	D	1	5.22
gaa	E	40187	23967.00
gag	E	466	16686.00
---	--	------	------
mPD		0.023	0.48
		nPD:	0.05
		N. weight:	0.52
		Sc. PD:	-0.0041
		Sc. rank:	7046.0

PA

Pos.	16	obs:	exp:
ttt	F	2	0.97
ttc	F	0	1.03
tta	L	0	4717.00
ttg	L	0	8404.00
ctt	L	40574	8504.00
ctc	L	49	5961.00
cta	L	28	6242.00
ctg	L	0	6822.00
cat	H	1	0.56
cac	H	0	0.44
cgt	R	1	0.02
cgc	R	0	0.04
cga	R	0	0.09
cgg	R	0	0.09
att	I	10	3.51
atc	I	0	2.63
ata	I	0	3.86
aga	R	0	0.49
agg	R	0	0.28
---	--	------	------
mPD		0.0045	1.2
		nPD:	0.
		N. weight:	1.7
		Sc. PD:	-0.087
		Sc. rank:	-42690.0

PA

Pos.	17	obs:	exp:
tct	S	0	0.16
tcc	S	0	0.15
tca	S	1	0.27
tcg	S	0	0.04
act	T	0	0.62
acc	T	0	0.35
aca	T	2	0.87
acg	T	0	0.16
aaa	K	1	0.66
aag	K	0	0.34
agt	S	0	0.17
agc	S	0	0.21
gtt	V	0	0.25
gtc	V	0	0.20
gta	V	1	0.22
gtg	V	0	0.33
gct	A	3	11082.00
gcc	A	16	10159.00
gca	A	40367	16447.00
gcg	A	274	2972.00
---	--	------	------
mPD		0.015	0.69
		nPD:	0.02
		N. weight:	0.97
		Sc. PD:	-0.033
		Sc. rank:	-3301.7

PA

Pos.	18	obs:	exp:
tct	S	0	0.16
tcc	S	0	0.15
tca	S	1	0.27
tcg	S	0	0.04
agt	S	0	0.17
agc	S	0	0.21
gaa	E	40615	23973.00
gag	E	48	16690.00
ggt	G	0	0.13
ggc	G	0	0.14
gga	G	1	0.46
ggg	G	0	0.26
---	--	------	------
mPD		0.0025	0.48
		nPD:	0.01
		N. weight:	0.58
		Sc. PD:	-0.028
		Sc. rank:	-13133.0

PA

Pos.	19	obs:	exp:
cct	P	0	0.33
ccc	P	0	0.19
cca	P	1	0.34
ccg	P	0	0.14
cgt	R	0	0.32
cgc	R	0	0.58
cga	R	0	1.46
cgg	R	0	1.37
aaa	K	40037	26723.00
aag	K	610	13924.00
aga	R	13	7.85
agg	R	3	4.43
gaa	E	1	0.59
gag	E	0	0.41
---	--	------	------
mPD		0.031	0.45
		nPD:	0.07
		N. weight:	0.39
		Sc. PD:	0.0045
		Sc. rank:	7338.9

PA

Pos.	20	obs:	exp:
tct	S	0	1.29
tcc	S	0	1.20
tca	S	8	2.15
tcg	S	0	0.31
act	T	0	138.50
acc	T	0	78.85
aca	T	446	193.00
acg	T	0	35.62
agt	S	0	1.40
agc	S	0	1.65
gtt	V	0	2.00
gtc	V	0	1.63
gta	V	8	1.73
gtg	V	0	2.63
gct	A	28	10957.00
gcc	A	0	10044.00
gca	A	40151	16261.00
gcg	A	21	2939.00
gat	D	0	0.57
gac	D	1	0.43
gaa	E	2	1.18
gag	E	0	0.82
---	--	------	------
mPD		0.025	0.72
		nPD:	0.03
		N. weight:	0.99
		Sc. PD:	-0.02
		Sc. rank:	8183.7

PA

Pos.	21	obs:	exp:
att	I	0	1.05
atc	I	0	0.79
ata	I	3	1.16
atg	M	40660	40660.00
---	-	2	2.00
---	--	------	------
mPD		0.00015	0.00015
		nPD:	1.
		N. weight:	0.000078
		Sc. PD:	0.000071
		Sc. rank:	2.5

PA

Pos.	22	obs:	exp:
cgt	R	0	0.44
cgc	R	0	0.79
cga	R	0	2.01
cgg	R	0	1.89
aaa	K	40048	26720.00
aag	K	595	13923.00
aga	R	22	10.79
agg	R	0	6.09
---	--	------	------
mPD		0.030	0.45
		nPD:	0.07
		N. weight:	0.39
		Sc. PD:	0.0039
		Sc. rank:	7188.5

PA

Pos.	23	obs:	exp:
gat	D	1	0.57
gac	D	0	0.43
gaa	E	508	23973.00
gag	E	40154	16689.00
ggt	G	0	0.27
ggc	G	0	0.28
gga	G	0	0.92
ggg	G	2	0.53
---	--	------	------
mPD		0.025	0.48
		nPD:	0.05
		N. weight:	0.91
		Sc. PD:	-0.0042
		Sc. rank:	13357.0

PA

Pos.	24	obs:	exp:
ttt	F	5	2.43
ttc	F	0	2.57
tat	Y	40611	21428.00
tac	Y	40	19223.00
cat	H	8	4.51
cac	H	0	3.49
gat	D	1	0.57
gac	D	0	0.43
---	--	------	------
mPD		0.0027	0.50
		nPD:	0.01
		N. weight:	0.7
		Sc. PD:	-0.034
		Sc. rank:	-15780.0

PA

Pos.	25	obs:	exp:
cgt	R	0	0.02
cgc	R	0	0.04
cga	R	0	0.09
cgg	R	0	0.09
aga	R	0	0.49
agg	R	1	0.28
gct	A	0	0.27
gcc	A	0	0.25
gca	A	1	0.40
gcg	A	0	0.07
ggt	G	7	5474.00
ggc	G	0	5675.00
gga	G	8940	18771.00
ggg	G	31716	10743.00
---	--	------	------
mPD		0.34	0.68
		nPD:	0.51
		N. weight:	0.75
		Sc. PD:	0.33
		Sc. rank:	23515.0

PA

Pos.	26	obs:	exp:
aaa	K	0	0.66
aag	K	1	0.34
gat	D	1	0.57
gac	D	0	0.43
gaa	E	511	23971.00
gag	E	40148	16688.00
ggt	G	0	0.54
ggc	G	0	0.56
gga	G	0	1.85
ggg	G	4	1.06
---	--	------	------
mPD		0.025	0.48
		nPD:	0.05
		N. weight:	0.91
		Sc. PD:	-0.0037
		Sc. rank:	13423.0

PA

Pos.	27	obs:	exp:
cat	H	1	0.56
cac	H	0	0.44
aat	N	162	91.75
aac	N	2	72.25
gat	D	40361	22891.00
gac	D	136	17606.00
ggt	G	3	0.40
ggc	G	0	0.42
gga	G	0	1.38
ggg	G	0	0.79
---	--	------	------
mPD		0.015	0.50
		nPD:	0.03
		N. weight:	0.61
		Sc. PD:	-0.015
		Sc. rank:	3056.6

PA

Pos.	28	obs:	exp:
tta	L	0	4691.00
ttg	L	83	8358.00
tct	S	0	28.08
tcc	S	0	26.00
tca	S	0	46.77
tcg	S	174	6.85
ctt	L	29	8457.00
ctc	L	1	5929.00
cta	L	147	6208.00
ctg	L	40169	6785.00
cct	P	0	16.33
ccc	P	12	9.19
cca	P	2	16.55
ccg	P	35	6.93
caa	Q	0	4.89
cag	Q	8	3.11
atg	M	5	5.00
agt	S	0	30.41
agc	S	0	35.90
---	--	------	------
mPD		0.033	1.2
		nPD:	0.03
		N. weight:	1.9
		Sc. PD:	-0.052
		Sc. rank:	7555.8

PA

Pos.	29	obs:	exp:
cgt	R	0	0.12
cgc	R	0	0.22
cga	R	0	0.55
cgg	R	0	0.52
aaa	K	40626	26731.00
aag	K	33	13928.00
aga	R	6	2.94
agg	R	0	1.66
---	--	------	------
mPD		0.0019	0.45
		nPD:	0.
		N. weight:	0.46
		Sc. PD:	-0.023
		Sc. rank:	-11074.0

PA

Pos.	30	obs:	exp:
tct	S	0	0.16
tcc	S	0	0.15
tca	S	0	0.27
tcg	S	0	0.04
att	I	40021	14271.00
atc	I	628	10682.00
ata	I	5	15700.00
act	T	1	0.93
acc	T	2	0.53
aca	T	0	1.30
acg	T	0	0.24
aat	N	2	1.12
aac	N	0	0.88
agt	S	0	0.17
agc	S	1	0.21
gtt	V	4	1.25
gtc	V	1	1.02
gta	V	0	1.08
gtg	V	0	1.65
---	--	------	------
mPD		0.031	0.66
		nPD:	0.05
		N. weight:	1.1
		Sc. PD:	-0.0088
		Sc. rank:	14274.0

PA

Pos.	31	obs:	exp:
aaa	K	3	1.97
aag	K	0	1.03
gtt	V	0	0.25
gtc	V	0	0.20
gta	V	1	0.22
gtg	V	0	0.33
gat	D	0	0.57
gac	D	1	0.43
gaa	E	40543	23972.00
gag	E	117	16688.00
---	--	------	------
mPD		0.0060	0.48
		nPD:	0.01
		N. weight:	0.56
		Sc. PD:	-0.024
		Sc. rank:	-7516.4

,

PA

Pos.	32	obs:	exp:
tct	S	0	0.16
tcc	S	0	0.15
tca	S	0	0.27
tcg	S	0	0.04
cct	P	0	0.33
ccc	P	1	0.19
cca	P	0	0.34
ccg	P	0	0.14
att	I	2	2.11
atc	I	4	1.58
ata	I	0	2.32
act	T	3687	12625.00
acc	T	35591	7188.00
aca	T	1375	17594.00
acg	T	1	3247.00
aat	N	0	1.68
aac	N	3	1.32
agt	S	0	0.17
agc	S	1	0.21
---	--	------	------
mPD		0.22	0.68
		nPD:	0.33
		N. weight:	1.3
		Sc. PD:	0.36
		Sc. rank:	39962.0

,

PA

Pos.	33	obs:	exp:
tat	Y	0	0.53
tac	Y	1	0.47
aat	N	133	22750.00
aac	N	40530	17913.00
aaa	K	1	0.66
aag	K	0	0.34
---	--	------	------
mPD		0.0066	0.49
		nPD:	0.01
		N. weight:	0.88
		Sc. PD:	-0.036
		Sc. rank:	-10863.0

,

PA

Pos.	34	obs:	exp:
cgt	R	0	0.04
cgc	R	0	0.07
cga	R	0	0.18
cgg	R	0	0.17
aaa	K	40399	26733.00
aag	K	264	13930.00
aga	R	2	0.98
agg	R	0	0.55
---	--	------	------
mPD		0.013	0.45
		nPD:	0.03
		N. weight:	0.43
		Sc. PD:	-0.011
		Sc. rank:	1826.7

,

PA

Pos.	35	obs:	exp:
ttt	F	40314	19778.00
ttc	F	346	20882.00
tta	L	0	0.58
ttg	L	0	1.03
ctt	L	4	1.05
ctc	L	1	0.73
cta	L	0	0.77
ctg	L	0	0.84
---	--	------	------
mPD		0.017	0.50
		nPD:	0.03
		N. weight:	0.74
		Sc. PD:	-0.016
		Sc. rank:	6031.8

,

PA

Pos.	36	obs:	exp:
act	T	0	0.31
acc	T	0	0.18
aca	T	1	0.43
acg	T	0	0.08
gtt	V	0	0.25
gtc	V	0	0.20
gta	V	1	0.22
gtg	V	0	0.33
gct	A	215	11082.00
gcc	A	1374	10159.00
gca	A	38778	16448.00
gcg	A	294	2972.00
gaa	E	2	1.18
gag	E	0	0.82
---	--	------	------
mPD		0.089	0.69
		nPD:	0.13
		N. weight:	0.79
		Sc. PD:	0.056
		Sc. rank:	20048.0

,

PA

Pos.	37	obs:	exp:
tct	S	0	0.32
tcc	S	0	0.30
tca	S	2	0.54
tcg	S	0	0.08
att	I	1	0.35
atc	I	0	0.26
ata	I	0	0.39
act	T	0	1.55
acc	T	0	0.88
aca	T	5	2.16
acg	T	0	0.40
agt	S	0	0.35
agc	S	0	0.41
gct	A	5	11081.00
gcc	A	4	10158.00
gca	A	40584	16446.00
gcg	A	64	2972.00
---	--	------	------
mPD		0.0041	0.69
		nPD:	0.01
		N. weight:	0.99
		Sc. PD:	-0.048
		Sc. rank:	-21895.0

,

PA

Pos.	38	obs:	exp:
tta	L	0	0.23
ttg	L	0	0.41
ctt	L	0	0.42
ctc	L	0	0.29
cta	L	2	0.31
ctg	L	0	0.34
att	I	13	14261.00
atc	I	2	10675.00
ata	I	40610	15689.00
atg	M	11	11.00
act	T	0	5.90
acc	T	0	3.36
aca	T	19	8.22
acg	T	0	1.52
aat	N	1	0.56
aac	N	0	0.44
gtt	V	0	1.25
gtc	V	0	1.02
gta	V	5	1.08
gtg	V	0	1.65
---	-	2	2.00
---	--	------	------
mPD		0.0027	0.66
		nPD:	0.
		N. weight:	1.
		Sc. PD:	-0.053
		Sc. rank:	-25950.0

,

PA

Pos.	39	obs:	exp:
tta	L	0	0.12
ttg	L	1	0.21
tgt	C	128	15891.00
tgc	C	40535	24772.00
ctt	L	0	0.21
ctc	L	0	0.15
cta	L	0	0.15
ctg	L	0	0.17
cgt	R	0	0.02
cgc	R	0	0.04
cga	R	0	0.09
cgg	R	0	0.09
aga	R	0	0.49
agg	R	1	0.28
---	--	------	------
mPD		0.0065	0.48
		nPD:	0.01
		N. weight:	0.53
		Sc. PD:	-0.022
		Sc. rank:	-6151.9

,

PA

Pos.	40	obs:	exp:
att	I	3	1.05
atc	I	0	0.79
ata	I	0	1.16
act	T	39743	12626.00
acc	T	685	7188.00
aca	T	52	17595.00
acg	T	176	3247.00
aat	N	1	0.56
aac	N	0	0.44
gct	A	5	1.36
gcc	A	0	1.25
gca	A	0	2.02
gcg	A	0	0.37
---	--	------	------
mPD		0.045	0.68
		nPD:	0.07
		N. weight:	1.2
		Sc. PD:	0.011
		Sc. rank:	21356.0

,

PA

Pos.	41	obs:	exp:
cat	H	2672	22935.00
cac	H	37990	17727.00
caa	Q	2	1.22
cag	Q	0	0.78
aaa	K	0	0.66
aag	K	1	0.34
---	--	------	------
mPD		0.12	0.49
		nPD:	0.25
		N. weight:	0.63
		Sc. PD:	0.12
		Sc. rank:	17919.0

,

PA

Pos.	42	obs:	exp:
tta	L	98	4719.00
ttg	L	40405	8407.00
tct	S	0	0.16
tcc	S	0	0.15
tca	S	1	0.27
tcg	S	0	0.04
ctt	L	0	8506.00
ctc	L	0	5963.00
cta	L	0	6244.00
ctg	L	161	6825.00
agt	S	0	0.17
agc	S	0	0.21
---	--	------	------
mPD		0.013	1.2
		nPD:	0.01
		N. weight:	1.7
		Sc. PD:	-0.074
		Sc. rank:	-25774.0

,

PA

Pos.	43	obs:	exp:
gtt	V	0	0.25
gtc	V	0	0.20
gta	V	1	0.22
gtg	V	0	0.33
gaa	E	475	23974.00
gag	E	40189	16690.00
---	--	------	------
mPD		0.023	0.48
		nPD:	0.05
		N. weight:	0.91
		Sc. PD:	-0.0072
		Sc. rank:	12263.0

,

PA

Pos.	44	obs:	exp:
ttt	F	0	0.49
ttc	F	1	0.51
att	I	1	2.11
atc	I	1	1.58
ata	I	4	2.32
atg	M	1	1.00
gtt	V	43	10182.00
gtc	V	285	8283.00
gta	V	749	8810.00
gtg	V	39572	13374.00
gct	A	0	2.18
gcc	A	0	2.00
gca	A	1	3.24
gcg	A	7	0.58
---	--	------	------
mPD		0.053	0.74
		nPD:	0.07
		N. weight:	1.1
		Sc. PD:	0.016
		Sc. rank:	21543.0

,

PA

Pos.	45	obs:	exp:
ttt	F	1	0.49
ttc	F	0	0.51
tat	Y	1	0.53
tac	Y	0	0.47
tgt	C	40472	15890.00
tgc	C	189	24771.00
aat	N	1	0.56
aac	N	0	0.44
---	-	1	1.00
---	--	------	------
mPD		0.0094	0.48
		nPD:	0.02
		N. weight:	1.
		Sc. PD:	-0.035
		Sc. rank:	-4524.1

,

PA			
Pos.	46	obs:	exp:
ttt	F	28	19779.00
ttc	F	40633	20882.00
tta	L	2	0.23
ttg	L	0	0.41
tct	S	0	0.16
tcc	S	0	0.15
tca	S	0	0.27
tcg	S	0	0.04
ctt	L	0	0.42
ctc	L	0	0.29
cta	L	0	0.31
ctg	L	0	0.34
agt	S	0	0.17
agc	S	1	0.21
---	-	1	1.00
---	--	------	------
mPD		0.0016	0.50
		nPD:	0.
		N. weight:	0.73
		Sc. PD:	-0.038
		Sc. rank:	-19251.0

,

PA			
Pos.	47	obs:	exp:
tta	L	0	0.12
ttg	L	0	0.21
ctt	L	0	0.21
ctc	L	0	0.15
cta	L	0	0.15
ctg	L	1	0.17
att	I	0	1.05
atc	I	0	0.79
ata	I	3	1.16
atg	M	40660	40660.00
---	-	1	1.00
---	--	------	------
mPD		0.00020	0.00023
		nPD:	0.86
		N. weight:	0.00013
		Sc. PD:	0.000099
		Sc. rank:	4.0

,

PA			
Pos.	48	obs:	exp:
ttt	F	12	5.84
ttc	F	0	6.16
tat	Y	39084	21429.00
tac	Y	1569	19224.00
---	--	------	------
mPD		0.075	0.50
		nPD:	0.15
		N. weight:	0.53
		Sc. PD:	0.048
		Sc. rank:	14083.0

,

PA			
Pos.	49	obs:	exp:
tta	L	1	0.12
ttg	L	0	0.21
tct	S	5	6561.00
tcc	S	4	6076.00
tca	S	40407	10928.00
tcg	S	243	1600.00
ctt	L	0	0.21
ctc	L	0	0.15
cta	L	0	0.15
ctg	L	0	0.17
cct	P	0	0.33
ccc	P	0	0.19
cca	P	1	0.34
ccg	P	0	0.14
agt	S	0	7105.00
agc	S	0	8388.00
gct	A	0	1.09
gcc	A	0	1.00
gca	A	4	1.62
gcg	A	0	0.29
---	--	------	------
mPD		0.013	1.6
		nPD:	0.01
		N. weight:	1.4
		Sc. PD:	-0.067
		Sc. rank:	-26484.0

,

PA			
Pos.	50	obs:	exp:
gat	D	40440	22986.00
gac	D	225	17679.00
---	--	------	------
mPD		0.011	0.49
		nPD:	0.02
		N. weight:	0.6
		Sc. PD:	-0.019
		Sc. rank:	-1028.2

,

PA			
Pos.	51	obs:	exp:
ttt	F	1054	19778.00
ttc	F	39605	20881.00
tta	L	0	0.70
ttg	L	0	1.24
ctt	L	1	1.26
ctc	L	5	0.88
cta	L	0	0.92
ctg	L	0	1.01
---	--	------	------
mPD		0.051	0.50
		nPD:	0.1
		N. weight:	0.61
		Sc. PD:	0.027
		Sc. rank:	13978.0

,

PA			
Pos.	52	obs:	exp:
cat	H	40451	22937.00
cac	H	214	17728.00
---	--	------	------
mPD		0.010	0.49
		nPD:	0.02
		N. weight:	0.6
		Sc. PD:	-0.02
		Sc. rank:	-1939.9

,

PA			
Pos.	53	obs:	exp:
ttt	F	87	19780.00
ttc	F	40577	20884.00
tta	L	0	0.12
ttg	L	0	0.21
ctt	L	0	0.21
ctc	L	1	0.15
cta	L	0	0.15
ctg	L	0	0.17
---	--	------	------
mPD		0.0043	0.50
		nPD:	0.01
		N. weight:	0.72
		Sc. PD:	-0.033
		Sc. rank:	-12814.0

,

PA			
Pos.	54	obs:	exp:
tta	L	0	0.12
ttg	L	0	0.21
ctt	L	0	0.21
ctc	L	1	0.15
cta	L	0	0.15
ctg	L	0	0.17
att	I	246	14273.00
atc	I	40401	10683.00
ata	I	11	15702.00
gtt	V	0	1.25
gtc	V	5	1.02
gta	V	0	1.08
gtg	V	0	1.65
---	-	1	1.00
---	--	------	------
mPD		0.013	0.66
		nPD:	0.02
		N. weight:	1.4
		Sc. PD:	-0.051
		Sc. rank:	-6966.2

,

PA			
Pos.	55	obs:	exp:
tct	S	0	0.97
tcc	S	0	0.90
tca	S	0	1.61
tcg	S	0	0.24
att	I	1	0.35
atc	I	0	0.26
ata	I	0	0.39
act	T	1	0.31
acc	T	0	0.18
aca	T	0	0.43
acg	T	0	0.08
aat	N	40354	22633.00
aac	N	101	17822.00
agt	S	6	1.05
agc	S	0	1.24
gat	D	15	114.20
gac	D	187	87.82
---	--	------	------
mPD		0.024	0.50
		nPD:	0.05
		N. weight:	0.62
		Sc. PD:	-0.0046
		Sc. rank:	8492.1

,

PA			
Pos.	56	obs:	exp:
aaa	K	1	0.66
aag	K	0	0.34
gtt	V	0	0.50
gtc	V	0	0.41
gta	V	1	0.43
gtg	V	1	0.66
gat	D	2	3.96
gac	D	5	3.04
gaa	E	39578	23967.00
gag	E	1074	16685.00
ggt	G	0	0.40
ggc	G	0	0.42
gga	G	3	1.38
ggg	G	0	0.79
---	--	------	------
mPD		0.052	0.48
		nPD:	0.11
		N. weight:	0.46
		Sc. PD:	0.023
		Sc. rank:	11055.0

,

PA			
Pos.	57	obs:	exp:
tta	L	0	0.12
ttg	L	0	0.21
ctt	L	0	0.21
ctc	L	0	0.15
cta	L	1	0.15
ctg	L	0	0.17
cat	H	4	3.38
cac	H	2	2.62
caa	Q	40113	24762.00
cag	Q	396	15747.00
cgt	R	0	1.21
cgc	R	0	2.19
cga	R	34	5.57
cgg	R	25	5.24
aaa	K	88	57.85
aag	K	0	30.15
aga	R	0	29.92
agg	R	2	16.87
---	--	------	------
mPD		0.028	0.49
		nPD:	0.06
		N. weight:	0.49
		Sc. PD:	0.0011
		Sc. rank:	8485.5

,

PA			
Pos.	58	obs:	exp:
tct	S	0	1.29
tcc	S	0	1.20
tca	S	0	2.15
tcg	S	0	0.31
tgt	C	0	0.78
tgc	C	2	1.22
agt	S	0	1.40
agc	S	8	1.65
gtt	V	0	0.25
gtc	V	1	0.20
gta	V	0	0.22
gtg	V	0	0.33
gat	D	0	0.57
gac	D	1	0.43
ggt	G	377	5473.00
ggc	G	40201	5674.00
gga	G	73	18766.00
ggg	G	2	10740.00
---	--	------	------
mPD		0.023	0.68
		nPD:	0.03
		N. weight:	2.1
		Sc. PD:	-0.047
		Sc. rank:	15701.0

,

PA			
Pos.	59	obs:	exp:
aaa	K	19	12.49
aag	K	0	6.51
gtt	V	0	0.25
gtc	V	0	0.20
gta	V	1	0.22
gtg	V	0	0.33
gat	D	1	2.26
gac	D	3	1.74
gaa	E	40441	23960.00
gag	E	200	16681.00
---	--	------	------
mPD		0.011	0.49
		nPD:	0.02
		N. weight:	0.55
		Sc. PD:	-0.018
		Sc. rank:	-717.3

,

PA			
Pos.	60	obs:	exp:
tct	S	1	6560.00
tcc	S	1	6075.00
tca	S	40614	10927.00
tcg	S	37	1600.00
cct	P	0	2.33
ccc	P	0	1.31
cca	P	7	2.36
ccg	P	0	0.99
act	T	0	0.93
acc	T	0	0.53
aca	T	3	1.30
acg	T	0	0.24
agt	S	0	7104.00
agc	S	0	8387.00
gct	A	0	0.55
gcc	A	0	0.50
gca	A	2	0.81
gcg	A	0	0.15
---	--	------	------
mPD		0.0025	1.6
		nPD:	0.
		N. weight:	1.4
		Sc. PD:	-0.077
		Sc. rank:	-43383.0

,

PA

Pos.	61	obs:	exp:
tta	L	0	1.16
ttg	L	0	2.07
ctt	L	0	2.09
ctc	L	0	1.47
cta	L	10	1.54
ctg	L	0	1.68
att	I	15	14247.00
atc	I	163	10664.00
ata	I	40406	15673.00
atg	M	45	45.00
act	T	0	2.79
acc	T	1	1.59
aca	T	8	3.89
acg	T	0	0.72
gtt	V	0	4.26
gtc	V	0	3.46
gta	V	17	3.68
gtg	V	0	5.59
---	--	------	------
mPD		0.013	0.66
		nPD:	0.02
		N. weight:	1.
		Sc. PD:	-0.036
		Sc. rank:	-5392.0

,

PA

Pos.	62	obs:	exp:
tta	L	10	1.51
ttg	L	3	2.69
ctt	L	0	2.72
ctc	L	0	1.91
cta	L	0	2.00
ctg	L	0	2.18
att	I	226	255.60
atc	I	6	191.30
ata	I	496	281.20
atg	M	38	38.00
gtt	V	19	9990.00
gtc	V	2	8127.00
gta	V	35323	8645.00
gtg	V	4541	13123.00
---	-	1	1.00
---	--	------	------
mPD		0.25	0.78
		nPD:	0.32
		N. weight:	1.2
		Sc. PD:	0.31
		Sc. rank:	36441.0

,

PA

Pos.	63	obs:	exp:
tta	L	4	0.70
ttg	L	1	1.24
ctt	L	0	1.26
ctc	L	1	0.88
cta	L	0	0.92
ctg	L	0	1.01
att	I	0	36.86
atc	I	0	27.59
ata	I	105	40.55
atg	M	3	3.00
aaa	K	0	0.66
aag	K	1	0.34
gtt	V	10	10155.00
gtc	V	0	8261.00
gta	V	39597	8788.00
gtg	V	937	13340.00
gct	A	0	1.64
gcc	A	0	1.50
gca	A	5	2.43
gcg	A	1	0.44
---	--	------	------
mPD		0.052	0.75
		nPD:	0.07
		N. weight:	1.6
		Sc. PD:	0.02
		Sc. rank:	30315.0

,

PA

Pos.	64	obs:	exp:
aaa	K	1	0.66
aag	K	0	0.34
gtt	V	0	0.25
gtc	V	0	0.20
gta	V	1	0.22
gtg	V	0	0.33
gct	A	1	0.55
gcc	A	0	0.50
gca	A	0	0.81
gcg	A	1	0.15
gat	D	1	1.13
gac	D	1	0.87
gaa	E	40259	23970.00
gag	E	399	16688.00
ggt	G	0	0.13
ggc	G	0	0.14
gga	G	1	0.46
ggg	G	0	0.26
---	--	------	------
mPD		0.020	0.48
		nPD:	0.04
		N. weight:	0.53
		Sc. PD:	-0.0077
		Sc. rank:	5950.8

,

PA

Pos.	65	obs:	exp:
ttt	F	46	22.86
ttc	F	1	24.14
tta	L	0	4685.00
ttg	L	0	8347.00
tct	S	200	32.27
tcc	S	0	29.89
tca	S	0	53.76
tcg	S	0	7.87
ctt	L	40342	8446.00
ctc	L	28	5920.00
cta	L	3	6200.00
ctg	L	1	6776.00
cct	P	22	7.33
ccc	P	0	4.12
cca	P	0	7.43
ccg	P	0	3.11
att	I	21	7.37
atc	I	0	5.52
ata	I	0	8.11
aaa	K	0	0.66
aag	K	1	0.34
agt	S	0	34.95
agc	S	0	41.26
---	--	------	------
mPD		0.026	1.2
		nPD:	0.02
		N. weight:	1.7
		Sc. PD:	-0.057
		Sc. rank:	-3929.8

,

PA

Pos.	66	obs:	exp:
tct	S	0	0.16
tcc	S	0	0.15
tca	S	1	0.27
tcg	S	0	0.04
aat	N	1	6.15
aac	N	10	4.85
agt	S	0	0.17
agc	S	0	0.21
gat	D	1576	22856.00
gac	D	38858	17578.00
gaa	E	0	1.18
gag	E	2	0.82
ggt	G	205	29.21
ggc	G	12	30.29
gga	G	0	100.20
ggg	G	0	57.33
---	--	------	------
mPD		0.095	0.51
		nPD:	0.19
		N. weight:	0.74
		Sc. PD:	0.094
		Sc. rank:	20180.0

,

PA

Pos.	67	obs:	exp:
tct	S	0	0.16
tcc	S	0	0.15
tca	S	0	0.27
tcg	S	0	0.04
aat	N	59	33.01
aac	N	0	25.99
agt	S	0	0.17
agc	S	1	0.21
gtt	V	0	0.25
gtc	V	0	0.20
gta	V	1	0.22
gtg	V	0	0.33
gat	D	40448	22951.00
gac	D	155	17652.00
gaa	E	0	0.59
gag	E	1	0.41
---	--	------	------
mPD		0.011	0.49
		nPD:	0.02
		N. weight:	0.6
		Sc. PD:	-0.02
		Sc. rank:	-1469.6

,

PA

Pos.	68	obs:	exp:
tta	L	0	0.12
ttg	L	0	0.21
tct	S	0	1.29
tcc	S	0	1.20
tca	S	8	2.15
tcg	S	0	0.31
ctt	L	0	0.21
ctc	L	0	0.15
cta	L	0	0.15
ctg	L	1	0.17
cct	P	11	13548.00
ccc	P	27	7622.00
cca	P	40080	13735.00
ccg	P	537	5750.00
caa	Q	1	0.61
cag	Q	0	0.39
agt	S	0	1.40
agc	S	0	1.65
---	--	------	------
mPD		0.028	0.72
		nPD:	0.04
		N. weight:	1.1
		Sc. PD:	-0.018
		Sc. rank:	12217.0

,

PA

Pos.	69	obs:	exp:
tat	Y	1	0.53
tac	Y	0	0.47
aat	N	40626	22750.00
aac	N	38	17914.00
---	--	------	------
mPD		0.0019	0.49
		nPD:	0.
		N. weight:	0.63
		Sc. PD:	-0.032
		Sc. rank:	-15762.0

,

PA

Pos.	70	obs:	exp:
tct	S	0	0.16
tcc	S	0	0.15
tca	S	1	0.27
tcg	S	0	0.04
act	T	0	2.79
acc	T	0	1.59
aca	T	9	3.89
acg	T	0	0.72
agt	S	0	0.17
agc	S	0	0.21
gtt	V	0	9.77
gtc	V	0	7.95
gta	V	38	8.45
gtg	V	1	12.83
gct	A	4	11070.00
gcc	A	4	10148.00
gca	A	40457	16429.00
gcg	A	151	2969.00
---	--	------	------
mPD		0.010	0.70
		nPD:	0.01
		N. weight:	0.98
		Sc. PD:	-0.039
		Sc. rank:	-10527.0

,

PA

Pos.	71	obs:	exp:
tta	L	1	4718.00
ttg	L	311	8406.00
tgt	C	1	0.39
tgc	C	0	0.61
ctt	L	53	8506.00
ctc	L	0	5963.00
cta	L	192	6244.00
ctg	L	40104	6824.00
atg	M	2	2.00
gtt	V	0	0.25
gtc	V	0	0.20
gta	V	1	0.22
gtg	V	0	0.33
---	--	------	------
mPD		0.028	1.2
		nPD:	0.02
		N. weight:	1.9
		Sc. PD:	-0.059
		Sc. rank:	-412.7

,

PA

Pos.	72	obs:	exp:
tta	L	38710	4719.00
ttg	L	1620	8407.00
ctt	L	0	8506.00
ctc	L	0	5963.00
cta	L	153	6244.00
ctg	L	181	6825.00
atg	M	1	1.00
---	--	------	------
mPD		0.10	1.2
		nPD:	0.09
		N. weight:	2.1
		Sc. PD:	0.064
		Sc. rank:	46927.0

,

PA

Pos.	73	obs:	exp:
cgt	R	0	0.14
cgc	R	0	0.25
cga	R	0	0.64
cgg	R	0	0.60
atg	M	1	1.00
aat	N	2	1.12
aac	N	0	0.88
aaa	K	16052	26727.00
aag	K	24602	13927.00
aga	R	3	3.43
agg	R	4	1.94
---	-	1	1.00
---	--	------	------
mPD		0.48	0.45
		nPD:	1.06
		N. weight:	0.16
		Sc. PD:	0.15
		Sc. rank:	5034.9

,

PA

Pos.	74	obs:	exp:
cat	H	40	22934.00
cac	H	40621	17727.00
caa	Q	0	0.61
cag	Q	1	0.39
aat	N	0	1.12
aac	N	2	0.88
gat	D	0	0.57
gac	D	1	0.43
---	--	------	------
mPD		0.0022	0.49
		nPD:	0.
		N. weight:	0.91
		Sc. PD:	-0.046
		Sc. rank:	-21900.0

,

PA

Pos.	75	obs:	exp:
cgt	R	0	806.30
cgc	R	0	1463.00
cga	R	207	3710.00
cgg	R	0	3492.00
act	T	0	0.31
acc	T	0	0.18
aca	T	1	0.43
acg	T	0	0.08
aga	R	40377	19945.00
agg	R	80	11248.00
---	--	------	------
mPD		0.014	0.89
		nPD:	0.02
		N. weight:	0.75
		Sc. PD:	-0.029
		Sc. rank:	-6871.9

,

PA			
Pos.	76	obs:	exp:
ttt	F	40209	19780.00
ttc	F	455	20884.00
att	I	1	0.35
atc	I	0	0.26
ata	I	0	0.39
---	--	------	------
mPD		0.022	0.50
		nPD:	0.04
		N. weight:	0.73
		Sc. PD:	-0.0082
		Sc. rank:	9099.6

,

PA			
Pos.	77	obs:	exp:
caa	Q	1	0.61
cag	Q	0	0.39
aaa	K	2	1.31
aag	K	0	0.69
gat	D	0	0.57
gac	D	1	0.43
gaa	E	40375	23971.00
gag	E	284	16688.00
ggt	G	0	0.27
ggc	G	0	0.28
gga	G	2	0.92
ggg	G	0	0.53
---	--	------	------
mPD		0.014	0.48
		nPD:	0.03
		N. weight:	0.54
		Sc. PD:	-0.014
		Sc. rank:	2515.8

,

PA			
Pos.	78	obs:	exp:
tta	L	2	1.04
ttg	L	0	1.86
ctt	L	0	1.88
ctc	L	0	1.32
cta	L	7	1.38
ctg	L	0	1.51
cgt	R	0	0.04
cgc	R	0	0.07
cga	R	0	0.18
cgg	R	0	0.17
att	I	20	14270.00
atc	I	2	10681.00
ata	I	40628	15699.00
act	T	0	0.93
acc	T	0	0.53
aca	T	3	1.30
acg	T	0	0.24
aga	R	1	0.98
agg	R	1	0.55
gtt	V	0	0.25
gtc	V	0	0.20
gta	V	1	0.22
gtg	V	0	0.33
---	--	------	------
mPD		0.0019	0.66
		nPD:	0.
		N. weight:	1.
		Sc. PD:	-0.054
		Sc. rank:	-28722.0

,

PA			
Pos.	79	obs:	exp:
tta	L	0	0.12
ttg	L	0	0.21
ctt	L	0	0.21
ctc	L	0	0.15
cta	L	0	0.15
ctg	L	1	0.17
cgt	R	0	0.02
cgc	R	0	0.04
cga	R	0	0.09
cgg	R	0	0.09
att	I	275	14270.00
atc	I	39995	10682.00
ata	I	381	15699.00
aga	R	0	0.49
agg	R	1	0.28
gtt	V	0	3.01
gtc	V	11	2.45
gta	V	1	2.60
gtg	V	0	3.95
---	--	------	------
mPD		0.033	0.66
		nPD:	0.05
		N. weight:	1.4
		Sc. PD:	-0.0085
		Sc. rank:	19414.0

,

PA			
Pos.	80	obs:	exp:
ttt	F	0	0.49
ttc	F	1	0.51
gat	D	1	0.57
gac	D	0	0.43
gaa	E	1041	23973.00
gag	E	39622	16690.00
---	--	------	------
mPD		0.050	0.48
		nPD:	0.1
		N. weight:	0.84
		Sc. PD:	0.039
		Sc. rank:	19813.0

,

PA			
Pos.	81	obs:	exp:
tct	S	0	0.16
tcc	S	0	0.15
tca	S	0	0.27
tcg	S	0	0.04
agt	S	0	0.17
agc	S	1	0.21
ggt	G	3	5474.00
ggc	G	1	5675.00
gga	G	528	18771.00
ggg	G	40132	10743.00
---	--	------	------
mPD		0.026	0.68
		nPD:	0.04
		N. weight:	1.4
		Sc. PD:	-0.024
		Sc. rank:	13797.0

,

PA			
Pos.	82	obs:	exp:
cgt	R	0	806.30
cgc	R	0	1463.00
cga	R	0	3710.00
cgg	R	0	3492.00
att	I	0	0.35
atc	I	0	0.26
ata	I	1	0.39
aga	R	40387	19945.00
agg	R	277	11248.00
---	--	------	------
mPD		0.014	0.89
		nPD:	0.02
		N. weight:	0.75
		Sc. PD:	-0.03
		Sc. rank:	-7572.1

,

PA			
Pos.	83	obs:	exp:
att	I	0	0.35
atc	I	1	0.26
ata	I	0	0.39
gtt	V	0	0.25
gtc	V	1	0.20
gta	V	0	0.22
gtg	V	0	0.33
gat	D	61	22982.00
gac	D	40596	17675.00
gaa	E	0	1.18
gag	E	2	0.82
ggt	G	0	0.54
ggc	G	4	0.56
gga	G	0	1.85
ggg	G	0	1.06
---	--	------	------
mPD		0.0034	0.49
		nPD:	0.01
		N. weight:	0.91
		Sc. PD:	-0.043
		Sc. rank:	-18327.0

,

PA			
Pos.	84	obs:	exp:
cgt	R	0	805.70
cgc	R	60	1462.00
cga	R	36	3708.00
cgg	R	0	3489.00
act	T	0	0.31
acc	T	0	0.18
aca	T	1	0.43
acg	T	0	0.08
aaa	K	25	17.09
aag	K	1	8.91
aga	R	33865	19932.00
agg	R	6676	11240.00
ggt	G	0	0.13
ggc	G	0	0.14
gga	G	1	0.46
ggg	G	0	0.26
---	--	------	------
mPD		0.28	0.89
		nPD:	0.32
		N. weight:	0.38
		Sc. PD:	0.098
		Sc. rank:	11451.0

,

PA			
Pos.	85	obs:	exp:
tct	S	0	0.16
tcc	S	0	0.15
tca	S	0	0.27
tcg	S	0	0.04
cct	P	0	2.33
ccc	P	0	1.31
cca	P	7	2.36
ccg	P	0	0.99
att	I	0	12.99
atc	I	24	9.72
ata	I	13	14.29
act	T	173	12613.00
acc	T	5	7181.00
aca	T	40408	17577.00
acg	T	28	3244.00
aaa	K	1	0.66
aag	K	0	0.34
agt	S	0	0.17
agc	S	1	0.21
gct	A	0	1.36
gcc	A	0	1.25
gca	A	5	2.02
gcg	A	0	0.37
---	--	------	------
mPD		0.014	0.68
		nPD:	0.02
		N. weight:	0.89
		Sc. PD:	-0.031
		Sc. rank:	-3676.7

,

PA			
Pos.	86	obs:	exp:
tta	L	0	0.35
ttg	L	1	0.62
ctt	L	0	0.63
ctc	L	0	0.44
cta	L	0	0.46
ctg	L	2	0.50
att	I	15	16.15
atc	I	0	12.09
ata	I	31	17.77
atg	M	40571	40571.00
act	T	1	0.31
acc	T	0	0.18
aca	T	0	0.43
acg	T	0	0.08
gtt	V	0	11.02
gtc	V	0	8.97
gta	V	0	9.54
gtg	V	44	14.48
---	--	------	------
mPD		0.0047	0.0062
		nPD:	0.75
		N. weight:	0.002
		Sc. PD:	0.0013
		Sc. rank:	62.4

,

PA			
Pos.	87	obs:	exp:
gct	A	77	11083.00
gcc	A	40474	10160.00
gca	A	113	16449.00
gcg	A	0	2973.00
ggt	G	0	0.13
ggc	G	1	0.14
gga	G	0	0.46
ggg	G	0	0.26
---	--	------	------
mPD		0.0094	0.69
		nPD:	0.01
		N. weight:	1.5
		Sc. PD:	-0.062
		Sc. rank:	-17871.0

,

PA			
Pos.	88	obs:	exp:
tgg	W	40665	40665.00
---	--	------	------
mPD		0	0
		nPD:	1.
		N. weight:	0.
		Sc. PD:	0
		Sc. rank:	0

,

PA			
Pos.	89	obs:	exp:
act	T	71	12628.00
acc	T	3	7189.00
aca	T	40513	17598.00
acg	T	76	3248.00
aaa	K	1	0.66
aag	K	0	0.34
gct	A	0	0.27
gcc	A	0	0.25
gca	A	1	0.40
gcg	A	0	0.07
---	--	------	------
mPD		0.0075	0.68
		nPD:	0.01
		N. weight:	0.9
		Sc. PD:	-0.039
		Sc. rank:	-13912.0

,

PA			
Pos.	90	obs:	exp:
tta	L	5	0.58
ttg	L	0	1.03
ctt	L	0	1.05
ctc	L	0	0.73
cta	L	0	0.77
ctg	L	0	0.84
att	I	0	17.90
atc	I	0	13.40
ata	I	51	19.70
gtt	V	8	10168.00
gtc	V	44	8272.00
gta	V	40247	8799.00
gtg	V	297	13357.00
gct	A	0	3.27
gcc	A	0	3.00
gca	A	12	4.85
gcg	A	0	0.88
---	-	1	1.00
---	--	------	------
mPD		0.020	0.74
		nPD:	0.03
		N. weight:	1.6
		Sc. PD:	-0.045
		Sc. rank:	4980.1

,

PA

Pos.	91	obs:	exp:
tta	L	2	0.23
ttg	L	0	0.41
ctt	L	0	0.42
ctc	L	0	0.29
cta	L	0	0.31
ctg	L	0	0.34
att	I	0	15.45
atc	I	0	11.56
ata	I	44	16.99
gtt	V	3	10174.00
gtc	V	1	8276.00
gta	V	40033	8804.00
gtg	V	582	13365.00
---	--	------	------
mPD		0.031	0.74
		nPD:	0.04
		N. weight:	1.6
		Sc. PD:	-0.023
		Sc. rank:	18738.0

,

PA

Pos.	92	obs:	exp:
cat	H	0	1.69
cac	H	3	1.31
act	T	0	0.62
acc	T	2	0.35
aca	T	0	0.87
acg	T	0	0.16
aat	N	20	22748.00
aac	N	40640	17912.00
---	--	------	------
mPD		0.0012	0.49
		nPD:	0.
		N. weight:	0.9
		Sc. PD:	-0.047
		Sc. rank:	-25335.0

,

PA

Pos.	93	obs:	exp:
tct	S	0	6562.00
tcc	S	0	6076.00
tca	S	0	10929.00
tcg	S	0	1601.00
tgt	C	1	0.39
tgc	C	0	0.61
agt	S	40555	7106.00
agc	S	107	8388.00
ggt	G	2	0.27
ggc	G	0	0.28
gga	G	0	0.92
ggg	G	0	0.53
---	--	------	------
mPD		0.0054	1.6
		nPD:	0.
		N. weight:	1.9
		Sc. PD:	-0.098
		Sc. rank:	-49475.0

,

PA

Pos.	94	obs:	exp:
tta	L	0	0.23
ttg	L	0	0.41
ctt	L	0	0.42
ctc	L	2	0.29
cta	L	0	0.31
ctg	L	0	0.34
att	I	294	14271.00
atc	I	40267	10682.00
ata	I	92	15700.00
gtt	V	0	2.25
gtc	V	9	1.83
gta	V	0	1.95
gtg	V	0	2.96
ggt	G	0	0.13
ggc	G	1	0.14
gga	G	0	0.46
ggg	G	0	0.26
---	--	------	------
mPD		0.019	0.66
		nPD:	0.03
		N. weight:	1.4
		Sc. PD:	-0.036
		Sc. rank:	6942.9

,

PA

Pos.	95	obs:	exp:
tct	S	0	0.16
tcc	S	0	0.15
tca	S	0	0.27
tcg	S	0	0.04
tgt	C	208	15891.00
tgc	C	40456	24773.00
agt	S	0	0.17
agc	S	1	0.21
---	--	------	------
mPD		0.010	0.48
		nPD:	0.02
		N. weight:	0.52
		Sc. PD:	-0.017
		Sc. rank:	-1497.2

,

PA

Pos.	96	obs:	exp:
tct	S	0	0.16
tcc	S	0	0.15
tca	S	0	0.27
tcg	S	0	0.04
att	I	0	0.35
atc	I	1	0.26
ata	I	0	0.39
act	T	0	0.31
acc	T	1	0.18
aca	T	0	0.43
acg	T	0	0.08
aat	N	14	22749.00
aac	N	40648	17913.00
agt	S	0	0.17
agc	S	1	0.21
---	--	------	------
mPD		0.00084	0.49
		nPD:	0.
		N. weight:	0.9
		Sc. PD:	-0.048
		Sc. rank:	-26938.0

,

PA

Pos.	97	obs:	exp:
tct	S	4	0.65
tcc	S	0	0.60
tca	S	0	1.08
tcg	S	0	0.16
att	I	3	2.11
atc	I	3	1.58
ata	I	0	2.32
atg	M	1	1.00
act	T	40284	12618.00
acc	T	228	7183.00
aca	T	114	17584.00
acg	T	4	3245.00
aat	N	19	10.63
aac	N	0	8.37
agt	S	0	0.70
agc	S	0	0.83
gct	A	3	1.09
gcc	A	1	1.00
gca	A	0	1.62
gcg	A	0	0.29
---	-	1	1.00
---	--	------	------
mPD		0.019	0.68
		nPD:	0.03
		N. weight:	1.2
		Sc. PD:	-0.034
		Sc. rank:	4175.6

,

PA

Pos.	98	obs:	exp:
att	I	2	0.70
atc	I	0	0.53
ata	I	0	0.77
act	T	40344	12625.00
acc	T	53	7188.00
aca	T	246	17595.00
acg	T	12	3247.00
aat	N	4	2.24
aac	N	0	1.76
gct	A	3	1.09
gcc	A	0	1.00
gca	A	1	1.62
gcg	A	0	0.29
---	--	------	------
mPD		0.016	0.68
		nPD:	0.02
		N. weight:	1.2
		Sc. PD:	-0.039
		Sc. rank:	-1036.0

,

PA

Pos.	99	obs:	exp:
cgt	R	0	1.17
cgc	R	0	2.12
cga	R	0	5.38
cgg	R	0	5.07
aga	R	56	28.94
agg	R	3	16.32
gaa	E	14	8.25
gag	E	0	5.75
ggt	G	13	5465.00
ggc	G	1	5665.00
gga	G	40229	18738.00
ggg	G	349	10724.00
---	--	------	------
mPD		0.021	0.68
		nPD:	0.03
		N. weight:	0.8
		Sc. PD:	-0.019
		Sc. rank:	4509.1

,

PA

Pos.	100	obs:	exp:
tta	L	0	1.28
ttg	L	0	2.27
tct	S	3	0.48
tcc	S	0	0.45
tca	S	0	0.81
tcg	S	0	0.12
ctt	L	0	2.30
ctc	L	0	1.61
cta	L	0	1.69
ctg	L	11	1.85
att	I	0	4.56
atc	I	0	3.42
ata	I	13	5.02
act	T	91	28.26
acc	T	0	16.09
aca	T	0	39.38
acg	T	0	7.27
agt	S	0	0.52
agc	S	0	0.62
gtt	V	28	53.85
gtc	V	0	43.81
gta	V	187	46.60
gtg	V	0	70.74
gct	A	40177	10992.00
gcc	A	109	10076.00
gca	A	36	16314.00
gcg	A	9	2948.00
gat	D	1	0.57
gac	D	0	0.43
---	--	------	------
mPD		0.035	0.71
		nPD:	0.05
		N. weight:	1.4
		Sc. PD:	-0.0088
		Sc. rank:	20221.0

,

PA

Pos.	101	obs:	exp:
cgt	R	0	1.72
cgc	R	0	3.13
cga	R	0	7.94
cgg	R	0	7.47
aaa	K	0	1.97
aag	K	3	1.03
aga	R	87	42.67
agg	R	0	24.06
gtt	V	0	1.75
gtc	V	0	1.43
gta	V	7	1.52
gtg	V	0	2.30
gaa	E	1089	1022.00
gag	E	644	711.30
ggt	G	10	5228.00
ggc	G	1	5420.00
gga	G	38307	17927.00
ggg	G	517	10260.00
---	--	------	------
mPD		0.14	0.76
		nPD:	0.19
		N. weight:	0.75
		Sc. PD:	0.096
		Sc. rank:	20543.0

,

PA

Pos.	102	obs:	exp:
cgt	R	0	0.08
cgc	R	0	0.14
cga	R	0	0.36
cgg	R	0	0.34
aaa	K	40221	26732.00
aag	K	440	13929.00
aga	R	4	1.96
agg	R	0	1.11
---	--	------	------
mPD		0.022	0.45
		nPD:	0.05
		N. weight:	0.41
		Sc. PD:	-0.0032
		Sc. rank:	5481.6

,

PA

Pos.	103	obs:	exp:
cct	P	140	13551.00
ccc	P	102	7624.00
cca	P	697	13739.00
ccg	P	39726	5751.00
---	--	------	------
mPD		0.045	0.72
		nPD:	0.06
		N. weight:	2.
		Sc. PD:	0.014
		Sc. rank:	36088.0

,

PA

Pos.	104	obs:	exp:
tct	S	0	0.16
tcc	S	0	0.15
tca	S	0	0.27
tcg	S	0	0.04
caa	Q	1	0.61
cag	Q	0	0.39
cgt	R	0	0.77
cgc	R	0	1.40
cga	R	0	3.56
cgg	R	0	3.35
act	T	0	1.86
acc	T	0	1.06
aca	T	0	2.60
acg	T	6	0.48
aat	N	13	7.27
aac	N	0	5.73
aaa	K	636	26693.00
aag	K	39966	13909.00
agt	S	1	0.17
agc	S	0	0.21
aga	R	0	19.13
agg	R	39	10.79
gaa	E	0	1.77
gag	E	3	1.23
---	--	------	------
mPD		0.034	0.45
		nPD:	0.07
		N. weight:	1.1
		Sc. PD:	0.02
		Sc. rank:	22097.0

,

PA

Pos.	105	obs:	exp:
ttt	F	40276	19730.00
ttc	F	285	20831.00
tta	L	2	5.45
ttg	L	2	9.72
tct	S	24	3.87
tcc	S	0	3.59
tca	S	0	6.45
tcg	S	0	0.94
tat	Y	27	14.23
tac	Y	0	12.77
tgt	C	3	1.17
tgc	C	0	1.83
ctt	L	43	9.83
ctc	L	0	6.89
cta	L	0	7.22
ctg	L	0	7.89
agt	S	0	4.19
agc	S	0	4.95
gtt	V	3	0.75
gtc	V	0	0.61
gta	V	0	0.65
gtg	V	0	0.99
---	--	------	------
mPD		0.019	0.51
		nPD:	0.04
		N. weight:	0.75
		Sc. PD:	-0.013
		Sc. rank:	7353.1

,

PA

Pos.	106	obs:	exp:
tta	L	13364	4719.00
ttg	L	14	8407.00
ctt	L	201	8506.00
ctc	L	2	5963.00
cta	L	26775	6244.00
ctg	L	308	6825.00
att	I	0	0.35
atc	I	0	0.26
ata	I	1	0.39
---	--	------	------
mPD		0.47	1.2
		nPD:	0.4
		N. weight:	1.4
		Sc. PD:	0.47
		Sc. rank:	42232.0

,

PA

Pos.	107	obs:	exp:
cct	P	98	13551.00
ccc	P	3	7624.00
cca	P	3023	13739.00
ccg	P	37541	5751.00
---	--	------	------
mPD		0.14	0.72
		nPD:	0.2
		N. weight:	1.8
		Sc. PD:	0.24
		Sc. rank:	49507.0

,

PA

Pos.	108	obs:	exp:
aat	N	1	0.56
aac	N	0	0.44
gat	D	40494	22986.00
gac	D	170	17678.00
---	--	------	------
mPD		0.0084	0.49
		nPD:	0.02
		N. weight:	0.6
		Sc. PD:	-0.023
		Sc. rank:	-4162.8

,

PA

Pos.	109	obs:	exp:
tta	L	359	4719.00
ttg	L	32255	8407.00
ctt	L	10	8507.00
ctc	L	1	5963.00
cta	L	7	6244.00
ctg	L	8033	6825.00
---	--	------	------
mPD		0.34	1.2
		nPD:	0.29
		N. weight:	1.2
		Sc. PD:	0.27
		Sc. rank:	34514.0

,

PA

Pos.	110	obs:	exp:
tat	Y	40398	21435.00
tac	Y	267	19230.00
---	--	------	------
mPD		0.013	0.50
		nPD:	0.03
		N. weight:	0.67
		Sc. PD:	-0.019
		Sc. rank:	1165.6

,

PA

Pos.	111	obs:	exp:
act	T	1	0.31
acc	T	0	0.18
aca	T	0	0.43
acg	T	0	0.08
gat	D	40419	22985.00
gac	D	244	17678.00
ggt	G	1	0.13
ggc	G	0	0.14
gga	G	0	0.46
ggg	G	0	0.26
---	--	------	------
mPD		0.012	0.49
		nPD:	0.02
		N. weight:	0.59
		Sc. PD:	-0.018
		Sc. rank:	218.6

,

PA

Pos.	112	obs:	exp:
tat	Y	39	21433.00
tac	Y	40622	19228.00
tgt	C	0	0.39
tgc	C	1	0.61
cat	H	0	1.13
cac	H	2	0.87
gat	D	0	0.57
gac	D	1	0.43
---	--	------	------
mPD		0.0021	0.50
		nPD:	0.
		N. weight:	0.82
		Sc. PD:	-0.041
		Sc. rank:	-19886.0

,

PA

Pos.	113	obs:	exp:
cgt	R	0	0.14
cgc	R	0	0.25
cga	R	0	0.64
cgg	R	0	0.60
aat	N	1	0.56
aac	N	0	0.44
aaa	K	33591	26729.00
aag	K	7066	13928.00
aga	R	5	3.43
agg	R	2	1.94
---	--	------	------
mPD		0.29	0.45
		nPD:	0.64
		N. weight:	0.079
		Sc. PD:	0.044
		Sc. rank:	2480.8

,

PA

Pos.	114	obs:	exp:
aaa	K	3	5.92
aag	K	6	3.08
gat	D	0	0.57
gac	D	1	0.43
gaa	E	16570	23966.00
gag	E	24081	16685.00
ggt	G	0	0.54
ggc	G	0	0.56
gga	G	2	1.85
ggg	G	2	1.06
---	--	------	------
mPD		0.48	0.48
		nPD:	1.
		N. weight:	0.074
		Sc. PD:	0.068
		Sc. rank:	2361.0

,

PA

Pos.	115	obs:	exp:
tct	S	0	1.13
tcc	S	0	1.05
tca	S	0	1.88
tcg	S	0	0.28
act	T	0	0.31
acc	T	1	0.18
aca	T	0	0.43
acg	T	0	0.08
aat	N	345	22735.00
aac	N	40291	17901.00
aaa	K	4	3.94
aag	K	2	2.06
agt	S	0	1.22
agc	S	7	1.44
gat	D	0	8.48
gac	D	15	6.52
---	--	------	------
mPD		0.018	0.49
		nPD:	0.04
		N. weight:	0.85
		Sc. PD:	-0.016
		Sc. rank:	7881.1

,

PA

Pos.	116	obs:	exp:
cgt	R	0	806.00
cgc	R	0	1463.00
cga	R	178	3709.00
cgg	R	24	3491.00
aaa	K	12	7.89
aag	K	0	4.11
aga	R	39883	19940.00
agg	R	568	11245.00
---	--	------	------
mPD		0.039	0.89
		nPD:	0.04
		N. weight:	0.69
		Sc. PD:	-0.0079
		Sc. rank:	8552.7

,

PA

Pos.	117	obs:	exp:
ttt	F	31	19780.00
ttc	F	40633	20884.00
tgt	C	0	0.39
tgc	C	1	0.61
---	--	------	------
mPD		0.0016	0.50
		nPD:	0.
		N. weight:	0.73
		Sc. PD:	-0.037
		Sc. rank:	-19140.0

,

PA

Pos.	118	obs:	exp:
tta	L	0	0.81
ttg	L	0	1.45
ctt	L	0	1.46
ctc	L	7	1.03
cta	L	0	1.07
ctg	L	0	1.17
att	I	241	14199.00
atc	I	40148	10628.00
ata	I	59	15621.00
gtt	V	175	52.60
gtc	V	35	42.79
gta	V	0	45.52
gtg	V	0	69.09
---	--	------	------
mPD		0.034	0.67
		nPD:	0.05
		N. weight:	1.4
		Sc. PD:	-0.0079
		Sc. rank:	20853.0

,

PA

Pos.	119	obs:	exp:
aaa	K	1	0.66
aag	K	0	0.34
gct	A	0	0.27
gcc	A	0	0.25
gca	A	1	0.40
gcg	A	0	0.07
gat	D	1	0.57
gac	D	0	0.43
gaa	E	40337	23973.00
gag	E	325	16689.00
---	--	------	------
mPD		0.016	0.48
		nPD:	0.03
		N. weight:	0.54
		Sc. PD:	-0.012
		Sc. rank:	3798.7

,

PA			
Pos.	120	obs:	exp:
tta	L	0	0.23
ttg	L	0	0.41
ctt	L	2	0.42
ctc	L	0	0.29
cta	L	0	0.31
ctg	L	0	0.34
att	I	40448	14264.00
atc	I	161	10677.00
ata	I	25	15693.00
gtt	V	29	7.26
gtc	V	0	5.91
gta	V	0	6.29
gtg	V	0	9.54
mPD		0.011	0.66
		nPD:	0.02
		N. weight:	1.1
		Sc. PD:	-0.044
		Sc. rank:	-9955.9

,

PA			
Pos.	121	obs:	exp:
ggt	G	7	5475.00
ggc	G	1	5676.00
gga	G	39831	18772.00
ggg	G	826	10743.00
mPD		0.040	0.68
		nPD:	0.06
		N. weight:	0.76
		Sc. PD:	0.0023
		Sc. rank:	13129.0

,

PA			
Pos.	122	obs:	exp:
att	I	0	0.70
atc	I	0	0.53
ata	I	2	0.77
gtt	V	15	10185.00
gtc	V	0	8285.00
gta	V	1318	8813.00
gtg	V	39329	13379.00
gct	A	0	0.27
gcc	A	0	0.25
gca	A	0	0.40
gcg	A	1	0.07
mPD		0.064	0.74
		nPD:	0.09
		N. weight:	1.1
		Sc. PD:	0.031
		Sc. rank:	23771.0

,

PA			
Pos.	123	obs:	exp:
tct	S	0	0.16
tcc	S	0	0.15
tca	S	1	0.27
tcg	S	0	0.04
act	T	5	12628.00
acc	T	4	7189.00
aca	T	40139	17598.00
acg	T	516	3248.00
agt	S	0	0.17
agc	S	0	0.21
mPD		0.026	0.68
		nPD:	0.04
		N. weight:	0.87
		Sc. PD:	-0.016
		Sc. rank:	8400.5

,

PA			
Pos.	124	obs:	exp:
cgt	R	0	806.20
cgc	R	0	1463.00
cga	R	114	3710.00
cgg	R	27	3492.00
aaa	K	0	1.31
aag	K	2	0.69
aga	R	1008	19945.00
agg	R	39514	11247.00
mPD		0.061	0.89
		nPD:	0.07
		N. weight:	1.3
		Sc. PD:	0.015
		Sc. rank:	24086.0

,

PA			
Pos.	125	obs:	exp:
cgt	R	0	806.20
cgc	R	0	1463.00
cga	R	2	3710.00
cgg	R	0	3491.00
aaa	K	2	1.31
aag	K	0	0.69
aga	R	40364	19944.00
agg	R	295	11247.00
ggt	G	0	0.27
ggc	G	0	0.28
gga	G	2	0.92
ggg	G	0	0.53
mPD		0.015	0.89
		nPD:	0.02
		N. weight:	0.75
		Sc. PD:	-0.029
		Sc. rank:	-5931.9

,

PA			
Pos.	126	obs:	exp:
gat	D	607	397.40
gac	D	96	305.60
gaa	E	39715	23558.00
gag	E	244	16401.00
ggt	G	0	0.40
ggc	G	0	0.42
gga	G	3	1.38
ggg	G	0	0.79
mPD		0.046	0.50
		nPD:	0.09
		N. weight:	0.54
		Sc. PD:	0.019
		Sc. rank:	12198.0

,

PA			
Pos.	127	obs:	exp:
tta	L	0	0.35
ttg	L	0	0.62
ctt	L	0	0.63
ctc	L	3	0.44
cta	L	0	0.46
ctg	L	0	0.50
att	I	0	9.13
atc	I	26	6.83
ata	I	0	10.04
act	T	0	0.31
acc	T	1	0.18
aca	T	0	0.43
acg	T	0	0.08
gtt	V	141	10173.00
gtc	V	40450	8275.00
gta	V	22	8803.00
gtg	V	0	13363.00
gct	A	0	6.00
gcc	A	22	5.50
gca	A	0	8.90
gcg	A	0	1.61
mPD		0.011	0.74
		nPD:	0.01
		N. weight:	1.7
		Sc. PD:	-0.07
		Sc. rank:	-18717.0

,

PA			
Pos.	128	obs:	exp:
tat	Y	0	2.11
tac	Y	4	1.89
cct	P	0	0.33
ccc	P	1	0.19
cca	P	0	0.34
ccg	P	0	0.14
cat	H	62	22915.00
cac	H	40564	17711.00
caa	Q	27	17.12
cag	Q	1	10.88
aat	N	0	3.36
aac	N	6	2.64
mPD		0.0050	0.49
		nPD:	0.01
		N. weight:	0.91
		Sc. PD:	-0.04
		Sc. rank:	-14881.0

,

PA			
Pos.	129	obs:	exp:
tta	L	72	8.70
ttg	L	0	15.51
ctt	L	0	15.69
ctc	L	0	11.00
cta	L	3	11.52
ctg	L	0	12.59
cgt	R	0	0.02
cgc	R	0	0.04
cga	R	0	0.09
cgg	R	0	0.09
att	I	8	14133.00
atc	I	1	10579.00
ata	I	40251	15548.00
atg	M	292	292.00
act	T	0	10.56
acc	T	0	6.01
aca	T	34	14.71
acg	T	0	2.72
aga	R	1	0.49
agg	R	0	0.28
gtt	V	0	0.75
gtc	V	0	0.61
gta	V	3	0.65
gtg	V	0	0.99
mPD		0.020	0.67
		nPD:	0.03
		N. weight:	1.
		Sc. PD:	-0.026
		Sc. rank:	5402.4

,

PA			
Pos.	130	obs:	exp:
tat	Y	40223	21434.00
tac	Y	440	19229.00
cat	H	2	1.13
cac	H	0	0.87
mPD		0.022	0.50
		nPD:	0.04
		N. weight:	0.64
		Sc. PD:	-0.0081
		Sc. rank:	7900.6

,

PA			
Pos.	131	obs:	exp:
tat	Y	61	21434.00
tac	Y	40603	19230.00
tgt	C	0	0.39
tgc	C	1	0.61
mPD		0.0030	0.50
		nPD:	0.01
		N. weight:	0.82
		Sc. PD:	-0.04
		Sc. rank:	-17733.0

,

PA

Pos.	132	obs:	exp:
ttt	F	12	5.84
ttc	F	0	6.16
tta	L	0	4715.00
ttg	L	0	8401.00
ctt	L	40287	8500.00
ctc	L	83	5959.00
cta	L	213	6240.00
ctg	L	51	6820.00
cgt	R	1	0.02
cgc	R	0	0.04
cga	R	0	0.09
cgg	R	0	0.09
att	I	17	5.97
atc	I	0	4.47
ata	I	0	6.57
aga	R	0	0.49
agg	R	0	0.28
gtt	V	0	0.25
gtc	V	0	0.20
gta	V	1	0.22
gtg	V	0	0.33
---	--	------	------
mPD		0.019	1.2
		nPD:	0.02
		N. weight:	1.7
		Sc. PD:	-0.065
		Sc. rank:	-15105.0

,

PA

Pos.	133	obs:	exp:
caa	Q	1	0.61
cag	Q	0	0.39
aaa	K	4	2.63
aag	K	0	1.37
gat	D	1	0.57
gac	D	0	0.43
gaa	E	40210	23971.00
gag	E	449	16688.00
---	--	------	------
mPD		0.022	0.48
		nPD:	0.05
		N. weight:	0.52
		Sc. PD:	-0.0052
		Sc. rank:	6666.4

,

PA

Pos.	134	obs:	exp:
act	T	0	0.31
acc	T	0	0.18
aca	T	0	0.43
acg	T	1	0.08
aaa	K	302	26734.00
aag	K	40362	13930.00
---	--	------	------
mPD		0.015	0.45
		nPD:	0.03
		N. weight:	1.1
		Sc. PD:	-0.025
		Sc. rank:	7960.9

,

PA

Pos.	135	obs:	exp:
tct	S	0	0.16
tcc	S	1	0.15
tca	S	0	0.27
tcg	S	0	0.04
act	T	0	1.55
acc	T	5	0.88
aca	T	0	2.16
acg	T	0	0.40
agt	S	0	0.17
agc	S	0	0.21
gtt	V	0	2.50
gtc	V	10	2.04
gta	V	0	2.17
gtg	V	0	3.29
gct	A	49	11079.00
gcc	A	40573	10156.00
gca	A	26	16442.00
gcg	A	0	2971.00
gat	D	0	0.57
gac	D	1	0.43
---	--	------	------
mPD		0.0045	0.70
		nPD:	0.01
		N. weight:	1.5
		Sc. PD:	-0.073
		Sc. rank:	-32199.0

,

PA

Pos.	136	obs:	exp:
tct	S	0	1.61
tcc	S	0	1.49
tca	S	0	2.69
tcg	S	0	0.39
cat	H	1	0.56
cac	H	0	0.44
aat	N	39781	22741.00
aac	N	867	17907.00
aaa	K	0	0.66
aag	K	1	0.34
agt	S	10	1.75
agc	S	0	2.06
gat	D	5	2.83
gac	D	0	2.17
---	--	------	------
mPD		0.043	0.49
		nPD:	0.09
		N. weight:	0.53
		Sc. PD:	0.016
		Sc. rank:	11754.0

,

PA

Pos.	137	obs:	exp:
cgt	R	0	0.16
cgc	R	0	0.29
cga	R	0	0.73
cgg	R	0	0.69
aat	N	2	1.12
aac	N	0	0.88
aaa	K	8400	26728.00
aag	K	32255	13927.00
aga	R	1	3.92
agg	R	7	2.21
---	--	------	------
mPD		0.33	0.45
		nPD:	0.73
		N. weight:	0.47
		Sc. PD:	0.31
		Sc. rank:	14997.0

,

PA

Pos.	138	obs:	exp:
ttt	F	1	0.49
ttc	F	0	0.51
att	I	39306	14273.00
atc	I	1141	10684.00
ata	I	213	15703.00
gtt	V	3	1.00
gtc	V	1	0.82
gta	V	0	0.87
gtg	V	0	1.32
---	--	------	------
mPD		0.065	0.66
		nPD:	0.1
		N. weight:	0.99
		Sc. PD:	0.041
		Sc. rank:	22623.0

,

PA

Pos.	139	obs:	exp:
cgt	R	0	0.46
cgc	R	0	0.83
cga	R	0	2.10
cgg	R	0	1.97
aat	N	2	2.80
aac	N	3	2.20
aaa	K	40564	26716.00
aag	K	73	13921.00
aga	R	23	11.28
agg	R	0	6.36
---	--	------	------
mPD		0.0050	0.45
		nPD:	0.01
		N. weight:	0.45
		Sc. PD:	-0.02
		Sc. rank:	-6999.8

,

PA

Pos.	140	obs:	exp:
ttt	F	15	7.30
ttc	F	0	7.70
tta	L	1	0.12
ttg	L	0	0.21
tct	S	32588	6557.00
tcc	S	208	6072.00
tca	S	7834	10921.00
tcg	S	2	1599.00
ctt	L	0	0.21
ctc	L	0	0.15
cta	L	0	0.15
ctg	L	0	0.17
cct	P	5	1.67
ccc	P	0	0.94
cca	P	0	1.69
ccg	P	0	0.71
act	T	10	3.11
acc	T	0	1.77
aca	T	0	4.33
acg	T	0	0.80
aat	N	0	0.56
aac	N	1	0.44
agt	S	0	7101.00
agc	S	0	8382.00
gct	A	1	0.27
gcc	A	0	0.25
gca	A	0	0.40
gcg	A	0	0.07
---	--	------	------
mPD		0.32	1.6
		nPD:	0.2
		N. weight:	1.3
		Sc. PD:	0.18
		Sc. rank:	36896.0

,

PA

Pos.	141	obs:	exp:
aat	N	0	1.12
aac	N	2	0.88
aaa	K	0	2.63
aag	K	4	1.37
gat	D	27	19.22
gac	D	7	14.78
gaa	E	335	23949.00
gag	E	40287	16673.00
ggt	G	0	0.40
ggc	G	0	0.42
gga	G	0	1.38
ggg	G	3	0.79
---	--	------	------
mPD		0.019	0.49
		nPD:	0.04
		N. weight:	0.93
		Sc. PD:	-0.016
		Sc. rank:	9311.9

,

PA

Pos.	142	obs:	exp:
tct	S	0	3.39
tcc	S	0	3.14
tca	S	0	5.64
tcg	S	0	0.83
cat	H	0	0.56
cac	H	1	0.44
act	T	3	4.35
acc	T	11	2.48
aca	T	0	6.06
acg	T	0	1.12
aat	N	681	22582.00
aac	N	39682	17781.00
aaa	K	16	143.30
aag	K	202	74.68
agt	S	0	3.67
agc	S	21	4.33
gat	D	2	23.74
gac	D	40	18.26
ggt	G	0	0.81
ggc	G	6	0.84
gga	G	0	2.77
ggg	G	0	1.59
---	--	------	------
mPD		0.048	0.50
		nPD:	0.1
		N. weight:	0.81
		Sc. PD:	0.031
		Sc. rank:	18429.0

,

PA

Pos.	143	obs:	exp:
tct	S	0	0.16
tcc	S	0	0.15
tca	S	1	0.27
tcg	S	0	0.04
cct	P	0	3.00
ccc	P	0	1.69
cca	P	9	3.04
ccg	P	0	1.27
att	I	0	1.76
atc	I	0	1.31
ata	I	5	1.93
act	T	7	12621.00
acc	T	9	7185.00
aca	T	40612	17588.00
acg	T	13	3246.00
aaa	K	2	1.31
aag	K	0	0.69
agt	S	0	0.17
agc	S	0	0.21
gct	A	0	1.91
gcc	A	0	1.75
gca	A	7	2.83
gcg	A	0	0.51
---	--	------	------
mPD		0.0026	0.68
		nPD:	0.
		N. weight:	0.92
		Sc. PD:	-0.047
		Sc. rank:	-23048.0

,

PA

Pos.	144	obs:	exp:
tat	Y	0	2.11
tac	Y	4	1.89
cat	H	89	22927.00
cac	H	40558	17720.00
caa	Q	3	1.83
cag	Q	0	1.17
gtt	V	0	2.50
gtc	V	10	2.04
gta	V	0	2.17
gtg	V	0	3.29
gat	D	0	0.57
gac	D	1	0.43
---	--	------	------
mPD		0.0057	0.49
		nPD:	0.01
		N. weight:	0.9
		Sc. PD:	-0.039
		Sc. rank:	-12960.0

,

PA

Pos.	145	obs:	exp:
tta	L	0	0.23
ttg	L	0	0.41
ctt	L	2	0.42
ctc	L	0	0.29
cta	L	0	0.31
ctg	L	0	0.34
att	I	40182	14271.00
atc	I	461	10682.00
ata	I	10	15700.00
act	T	2	0.62
acc	T	0	0.35
aca	T	0	0.87
acg	T	0	0.16
gtt	V	8	2.00
gtc	V	0	1.63
gta	V	0	1.73
gtg	V	0	2.63
---	--	------	------
mPD		0.023	0.66
		nPD:	0.04
		N. weight:	1.1
		Sc. PD:	-0.022
		Sc. rank:	9409.6

,

PA

Pos.	146	obs:	exp:
cat	H	337	22937.00
cac	H	40328	17728.00
---	--	------	------
mPD		0.016	0.49
		nPD:	0.03
		N. weight:	0.86
		Sc. PD:	-0.019
		Sc. rank:	6545.7

,

PA

Pos.	147	obs:	exp:
att	I	40489	14274.00
atc	I	169	10684.00
ata	I	4	15704.00
gtt	V	3	0.75
gtc	V	0	0.61
gta	V	0	0.65
gtg	V	0	0.99
---	--	------	------
mPD		0.0086	0.66
		nPD:	0.01
		N. weight:	1.1
		Sc. PD:	-0.047
		Sc. rank:	-14361.0

,

PA

Pos.	148	obs:	exp:
ttt	F	37781	19780.00
ttc	F	2883	20884.00
tta	L	0	0.12
ttg	L	0	0.21
ctt	L	1	0.21
ctc	L	0	0.15
cta	L	0	0.15
ctg	L	0	0.17
---	--	------	------
mPD		0.13	0.50
		nPD:	0.26
		N. weight:	0.51
		Sc. PD:	0.1
		Sc. rank:	14568.0

,

PA

Pos.	149	obs:	exp:
tct	S	5	6562.00
tcc	S	1	6077.00
tca	S	40616	10930.00
tcg	S	43	1601.00
agt	S	0	7106.00
agc	S	0	8389.00
---	--	------	------
mPD		0.0024	1.6
		nPD:	0.
		N. weight:	1.4
		Sc. PD:	-0.077
		Sc. rank:	-43515.0

,

PA

Pos.	150	obs:	exp:
ttt	F	69	19779.00
ttc	F	40593	20883.00
tat	Y	0	1.58
tac	Y	3	1.42
---	--	------	------
mPD		0.0035	0.50
		nPD:	0.01
		N. weight:	0.72
		Sc. PD:	-0.034
		Sc. rank:	-14346.0

,

PA

Pos.	151	obs:	exp:
att	I	10	3.51
atc	I	0	2.63
ata	I	0	3.86
act	T	40434	12621.00
acc	T	144	7185.00
aca	T	17	17588.00
acg	T	44	3246.00
aat	N	11	6.15
aac	N	0	4.85
gct	A	5	1.36
gcc	A	0	1.25
gca	A	0	2.02
gcg	A	0	0.37
---	--	------	------
mPD		0.011	0.68
		nPD:	0.02
		N. weight:	1.3
		Sc. PD:	-0.048
		Sc. rank:	-9195.2

,

PA

Pos.	152	obs:	exp:
ggt	G	5	5475.00
ggc	G	0	5676.00
gga	G	925	18772.00
ggg	G	39735	10743.00
---	--	------	------
mPD		0.045	0.68
		nPD:	0.07
		N. weight:	1.3
		Sc. PD:	0.013
		Sc. rank:	24580.0

,

PA

Pos.	153	obs:	exp:
gat	D	4	2.83
gac	D	1	2.17
gaa	E	478	23972.00
gag	E	40182	16688.00
---	--	------	------
mPD		0.023	0.48
		nPD:	0.05
		N. weight:	0.91
		Sc. PD:	-0.0066
		Sc. rank:	12520.0

,

PA

Pos.	154	obs:	exp:
gaa	E	40294	23975.00
gag	E	371	16690.00
---	--	------	------
mPD		0.018	0.48
		nPD:	0.04
		N. weight:	0.53
		Sc. PD:	-0.0096
		Sc. rank:	5068.2

,

PA

Pos.	155	obs:	exp:
tta	L	0	8.59
ttg	L	1	15.30
ctt	L	0	15.48
ctc	L	0	10.85
cta	L	0	11.36
ctg	L	73	12.42
att	I	5	22.12
atc	I	0	16.55
ata	I	58	24.33
atg	M	40524	40524.00
gtt	V	0	1.00
gtc	V	0	0.82
gta	V	0	0.87
gtg	V	4	1.32
---	--	------	------
mPD		0.0069	0.0093
		nPD:	0.74
		N. weight:	0.0048
		Sc. PD:	0.0032
		Sc. rank:	152.4

,

PA			
Pos.	156	obs:	exp:
cgt	R	0	0.02
cgc	R	0	0.04
cga	R	0	0.09
cgg	R	0	0.09
aga	R	0	0.49
agg	R	1	0.28
gtt	V	0	0.75
gtc	V	3	0.61
gta	V	0	0.65
gtg	V	0	0.99
gct	A	122	11082.00
gcc	A	39657	10159.00
gca	A	882	16448.00
gcg	A	0	2972.00
---	--	------	------
mPD		0.049	0.69
		nPD:	0.07
		N. weight:	1.4
		Sc. PD:	0.019
		Sc. rank:	27152.0

,

PA			
Pos.	157	obs:	exp:
tct	S	0	0.97
tcc	S	0	0.90
tca	S	5	1.61
tcg	S	0	0.24
cct	P	0	0.67
ccc	P	0	0.37
cca	P	2	0.68
ccg	P	0	0.28
atg	M	1	1.00
act	T	7	12625.00
acc	T	203	7188.00
aca	T	38786	17594.00
acg	T	1658	3247.00
aaa	K	2	1.31
aag	K	0	0.69
agt	S	1	1.05
agc	S	0	1.24
---	--	------	------
mPD		0.089	0.68
		nPD:	0.13
		N. weight:	0.78
		Sc. PD:	0.057
		Sc. rank:	19957.0

,

PA			
Pos.	158	obs:	exp:
tct	S	0	0.16
tcc	S	0	0.15
tca	S	0	0.27
tcg	S	0	0.04
cgt	R	0	303.90
cgc	R	0	551.50
cga	R	0	1398.00
cgg	R	0	1316.00
atg	M	2	2.00
act	T	0	0.31
acc	T	0	0.18
aca	T	0	0.43
acg	T	1	0.08
aat	N	5	2.80
aac	N	0	2.20
aaa	K	306	16633.00
aag	K	24994	8667.00
agt	S	1	0.17
agc	S	0	0.21
aga	R	36	7518.00
agg	R	15291	4239.00
gaa	E	0	2.36
gag	E	4	1.64
ggt	G	0	3.23
ggc	G	0	3.35
gga	G	0	11.08
ggg	G	24	6.34
---	-	1	1.00
---	--	------	------
mPD		0.49	1.1
		nPD:	0.44
		N. weight:	1.2
		Sc. PD:	0.45
		Sc. rank:	37424.0

,

PA			
Pos.	159	obs:	exp:
tct	S	0	0.32
tcc	S	0	0.30
tca	S	2	0.54
tcg	S	0	0.08
act	T	0	2.17
acc	T	0	1.24
aca	T	7	3.03
acg	T	0	0.56
agt	S	0	0.35
agc	S	0	0.41
gtt	V	0	0.50
gtc	V	0	0.41
gta	V	2	0.43
gtg	V	0	0.66
gct	A	25	11080.00
gcc	A	62	10157.00
gca	A	40342	16444.00
gcg	A	223	2972.00
gaa	E	1	0.59
gag	E	0	0.41
---	-	1	1.00
---	--	------	------
mPD		0.016	0.69
		nPD:	0.02
		N. weight:	0.96
		Sc. PD:	-0.031
		Sc. rank:	-1163.4

,

PA			
Pos.	160	obs:	exp:
tat	Y	0	0.53
tac	Y	1	0.47
aat	N	0	2.24
aac	N	4	1.76
gat	D	155	22981.00
gac	D	40500	17674.00
gaa	E	3	2.36
gag	E	1	1.64
---	-	1	1.00
---	--	------	------
mPD		0.0080	0.49
		nPD:	0.02
		N. weight:	0.89
		Sc. PD:	-0.034
		Sc. rank:	-7496.4

,

PA			
Pos.	161	obs:	exp:
tat	Y	64	21433.00
tac	Y	40598	19229.00
cat	H	0	1.13
cac	H	2	0.87
---	-	1	1.00
---	--	------	------
mPD		0.0032	0.50
		nPD:	0.01
		N. weight:	0.82
		Sc. PD:	-0.039
		Sc. rank:	-17404.0

,

PA			
Pos.	162	obs:	exp:
tct	S	1	0.16
tcc	S	0	0.15
tca	S	0	0.27
tcg	S	0	0.04
cct	P	1	0.33
ccc	P	0	0.19
cca	P	0	0.34
ccg	P	0	0.14
att	I	10	3.51
atc	I	0	2.63
ata	I	0	3.86
act	T	39521	12623.00
acc	T	1105	7187.00
aca	T	21	17592.00
acg	T	1	3247.00
aat	N	2	1.12
aac	N	0	0.88
agt	S	0	0.17
agc	S	0	0.21
gct	A	2	0.82
gcc	A	1	0.75
gca	A	0	1.21
gcg	A	0	0.22
---	--	------	------
mPD		0.055	0.68
		nPD:	0.08
		N. weight:	1.2
		Sc. PD:	0.028
		Sc. rank:	24692.0

,

PA			
Pos.	163	obs:	exp:
tta	L	1	4716.00
ttg	L	0	8402.00
ctt	L	947	8501.00
ctc	L	39016	5959.00
cta	L	674	6240.00
ctg	L	0	6820.00
cgt	R	1	0.02
cgc	R	0	0.04
cga	R	0	0.09
cgg	R	0	0.09
att	I	0	9.13
atc	I	25	6.83
ata	I	1	10.04
aga	R	0	0.49
agg	R	0	0.28
---	--	------	------
mPD		0.079	1.2
		nPD:	0.07
		N. weight:	1.9
		Sc. PD:	0.022
		Sc. rank:	36226.0

,

PA			
Pos.	164	obs:	exp:
gat	D	757	22985.00
gac	D	39906	17678.00
ggt	G	0	0.27
ggc	G	2	0.28
gga	G	0	0.92
ggg	G	0	0.53
---	--	------	------
mPD		0.037	0.49
		nPD:	0.07
		N. weight:	0.81
		Sc. PD:	0.015
		Sc. rank:	16495.0

,

PA			
Pos.	165	obs:	exp:
caa	Q	1	1.22
cag	Q	1	0.78
gat	D	6	3.39
gac	D	0	2.61
gaa	E	1076	23970.00
gag	E	39581	16687.00
---	--	------	------
mPD		0.052	0.48
		nPD:	0.11
		N. weight:	0.84
		Sc. PD:	0.042
		Sc. rank:	20130.0

,

PA			
Pos.	166	obs:	exp:
caa	Q	1	0.61
cag	Q	0	0.39
att	I	1	0.35
atc	I	0	0.26
ata	I	0	0.39
gaa	E	39293	23973.00
gag	E	1370	16690.00
---	--	------	------
mPD		0.065	0.48
		nPD:	0.13
		N. weight:	0.44
		Sc. PD:	0.033
		Sc. rank:	11105.0

,

PA			
Pos.	167	obs:	exp:
tct	S	0	6562.00
tcc	S	0	6077.00
tca	S	1	10929.00
tcg	S	0	1601.00
aat	N	0	0.56
aac	N	1	0.44
agt	S	70	7106.00
agc	S	40592	8389.00
ggt	G	0	0.13
ggc	G	1	0.14
gga	G	0	0.46
ggg	G	0	0.26
---	--	------	------
mPD		0.0037	1.6
		nPD:	0.
		N. weight:	1.7
		Sc. PD:	-0.09
		Sc. rank:	-49841.0

,

PA

Pos.	168	obs:	exp:
cgt	R	0	806.30
cgc	R	0	1463.00
cga	R	0	3710.00
cgg	R	5	3492.00
aga	R	360	19946.00
agg	R	40300	11248.00
---	--	------	------
mPD		0.018	0.89
		nPD:	0.02
		N. weight:	1.4
		Sc. PD:	-0.047
		Sc. rank:	-5710.5

,

PA

Pos.	169	obs:	exp:
cct	P	0	0.33
ccc	P	0	0.19
cca	P	0	0.34
ccg	P	1	0.14
---	-	40664	40664.00
---	--	------	------
mPD		0	4.4×10^{-10}
		nPD:	0.
		N. weight:	0.
		Sc. PD:	0
		Sc. rank:	0

,

PA

Pos.	170	obs:	exp:
atg	M	1	1.00
---	-	40664	40664.00
---	--	------	------
mPD		0	0
		nPD:	1.
		N. weight:	0.
		Sc. PD:	0
		Sc. rank:	0

,

PA

Pos.	171	obs:	exp:
ttt	F	0	0.49
ttc	F	1	0.51
---	-	40664	40664.00
---	--	------	------
mPD		0	3.0×10^{-10}
		nPD:	0.
		N. weight:	0.
		Sc. PD:	0
		Sc. rank:	0

,

PA

Pos.	172	obs:	exp:
tta	L	0	0.12
ttg	L	1	0.21
ctt	L	0	0.21
ctc	L	0	0.15
cta	L	0	0.15
ctg	L	0	0.17
---	-	40664	40664.00
---	--	------	------
mPD		0	7.0×10^{-10}
		nPD:	0.
		N. weight:	0.
		Sc. PD:	0
		Sc. rank:	0

,

PA

Pos.	173	obs:	exp:
tat	Y	1	0.53
tac	Y	0	0.47
---	-	40664	40664.00
---	--	------	------
mPD		0	3.0×10^{-10}
		nPD:	0.
		N. weight:	0.
		Sc. PD:	0
		Sc. rank:	0

,

PA

Pos.	174	obs:	exp:
gtt	V	0	0.25
gtc	V	0	0.20
gta	V	0	0.22
gtg	V	1	0.33
---	-	40664	40664.00
---	--	------	------
mPD		0	4.5×10^{-10}
		nPD:	0.
		N. weight:	0.
		Sc. PD:	0
		Sc. rank:	0

,

PA

Pos.	175	obs:	exp:
cgt	R	0	0.02
cgc	R	0	0.04
cga	R	0	0.09
cgg	R	0	0.09
aga	R	0	0.49
agg	R	1	0.28
---	-	40664	40664.00
---	--	------	------
mPD		0	5.4×10^{-10}
		nPD:	0.
		N. weight:	0.
		Sc. PD:	0
		Sc. rank:	0

,

PA

Pos.	176	obs:	exp:
act	T	0	0.31
acc	T	0	0.18
aca	T	1	0.43
acg	T	0	0.08
---	-	40664	40664.00
---	--	------	------
mPD		0	4.1×10^{-10}
		nPD:	0.
		N. weight:	0.
		Sc. PD:	0
		Sc. rank:	0

,

PA

Pos.	177	obs:	exp:
aat	N	0	0.56
aac	N	1	0.44
---	-	40664	40664.00
---	--	------	------
mPD		0	3.0×10^{-10}
		nPD:	0.
		N. weight:	0.
		Sc. PD:	0
		Sc. rank:	0

,

PA

Pos.	178	obs:	exp:
ggt	G	0	0.13
ggc	G	0	0.14
gga	G	1	0.46
ggg	G	0	0.26
---	-	40664	40664.00
---	--	------	------
mPD		0	4.1×10^{-10}
		nPD:	0.
		N. weight:	0.
		Sc. PD:	0
		Sc. rank:	0

,

PA

Pos.	179	obs:	exp:
act	T	0	0.31
acc	T	0	0.18
aca	T	1	0.43
acg	T	0	0.08
---	-	40664	40664.00
---	--	------	------
mPD		0	4.1×10^{-10}
		nPD:	0.
		N. weight:	0.
		Sc. PD:	0
		Sc. rank:	0

,

PA

Pos.	180	obs:	exp:
tct	S	0	0.16
tcc	S	0	0.15
tca	S	1	0.27
tcg	S	0	0.04
agt	S	0	0.17
agc	S	0	0.21
gct	A	40371	11083.00
gcc	A	87	10160.00
gca	A	202	16449.00
gcg	A	4	2973.00
---	--	------	------
mPD		0.014	0.69
		nPD:	0.02
		N. weight:	1.4
		Sc. PD:	-0.047
		Sc. rank:	-5153.7

,

PA

Pos.	181	obs:	exp:
cgt	R	0	806.20
cgc	R	0	1463.00
cga	R	0	3710.00
cgg	R	1	3492.00
aaa	K	0	0.66
aag	K	1	0.34
aga	R	447	19944.00
agg	R	40214	11247.00
ggt	G	0	0.27
ggc	G	0	0.28
gga	G	0	0.92
ggg	G	2	0.53
---	--	------	------
mPD		0.022	0.89
		nPD:	0.02
		N. weight:	1.3
		Sc. PD:	-0.041
		Sc. rank:	1086.8

,

PA

Pos.	182	obs:	exp:
att	I	11943	14272.00
atc	I	28667	10683.00
ata	I	47	15702.00
aaa	K	0	0.66
aag	K	1	0.34
gtt	V	3	1.75
gtc	V	4	1.43
gta	V	0	1.52
gtg	V	0	2.30
---	--	------	------
mPD		0.42	0.66
		nPD:	0.63
		N. weight:	0.71
		Sc. PD:	0.39
		Sc. rank:	22207.0

,

PA

Pos.	183	obs:	exp:
att	I	0	0.35
atc	I	0	0.26
ata	I	1	0.39
aaa	K	38588	26734.00
aag	K	2076	13930.00
---	--	------	------
mPD		0.097	0.45
		nPD:	0.22
		N. weight:	0.28
		Sc. PD:	0.043
		Sc. rank:	7808.3

,

PA

Pos.	184	obs:	exp:
tct	S	0	0.32
tcc	S	0	0.30
tca	S	0	0.54
tcg	S	0	0.08
atg	M	1	1.00
act	T	131	12628.00
acc	T	40498	7189.00
aca	T	28	17598.00
acg	T	5	3248.00
agt	S	0	0.35
agc	S	2	0.41
---	--	------	------
mPD		0.0082	0.68
		nPD:	0.01
		N. weight:	1.9
		Sc. PD:	-0.08
		Sc. rank:	-25737.0

,

PA

Pos.	185	obs:	exp:
cgt	R	0	806.30
cgc	R	0	1463.00
cga	R	0	3710.00
cgg	R	2	3492.00
aaa	K	1	0.66
aag	K	0	0.34
aga	R	269	19945.00
agg	R	40393	11248.00
---	--	------	------
mPD		0.013	0.89
		nPD:	0.02
		N. weight:	1.4
		Sc. PD:	-0.055
		Sc. rank:	-14267.0

,

PA

Pos.	186	obs:	exp:
tta	L	27	4719.00
ttg	L	139	8407.00
tgg	W	1	1.00
ctt	L	68	8506.00
ctc	L	174	5963.00
cta	L	8522	6244.00
ctg	L	31734	6825.00
---	--	------	------
mPD		0.35	1.2
		nPD:	0.3
		N. weight:	1.4
		Sc. PD:	0.32
		Sc. rank:	39684.0

,

PA

Pos.	187	obs:	exp:
ttt	F	40184	19780.00
ttc	F	479	20883.00
gtt	V	1	0.25
gtc	V	0	0.20
gta	V	0	0.22
gtg	V	0	0.33
---	-	1	1.00
---	--	------	------
mPD		0.023	0.50
		nPD:	0.05
		N. weight:	0.73
		Sc. PD:	-0.0066
		Sc. rank:	9418.3

,

PA

Pos.	188	obs:	exp:
att	I	0	2.81
atc	I	8	2.10
ata	I	0	3.09
act	T	98	12625.00
acc	T	40476	7188.00
aca	T	81	17595.00
acg	T	0	3247.00
gct	A	0	0.27
gcc	A	1	0.25
gca	A	0	0.40
gcg	A	0	0.07
---	-	1	1.00
---	--	------	------
mPD		0.0092	0.68
		nPD:	0.01
		N. weight:	1.9
		Sc. PD:	-0.077
		Sc. rank:	-22129.0

,

PA

Pos.	189	obs:	exp:
tta	L	0	8.70
ttg	L	0	15.51
ctt	L	0	15.69
ctc	L	0	11.00
cta	L	75	11.52
ctg	L	0	12.59
att	I	11	14248.00
atc	I	2	10665.00
ata	I	40575	15675.00
gtt	V	0	0.25
gtc	V	0	0.20
gta	V	1	0.22
gtg	V	0	0.33
---	-	1	1.00
---	--	------	------
mPD		0.0044	0.66
		nPD:	0.01
		N. weight:	1.1
		Sc. PD:	-0.05
		Sc. rank:	-22093.0

,

PA

Pos.	190	obs:	exp:
cgt	R	0	806.30
cgc	R	0	1463.00
cga	R	135	3710.00
cgg	R	0	3492.00
aga	R	39272	19945.00
agg	R	1257	11248.00
ggt	G	0	0.13
ggc	G	0	0.14
gga	G	1	0.46
ggg	G	0	0.26
---	--	------	------
mPD		0.067	0.89
		nPD:	0.08
		N. weight:	0.64
		Sc. PD:	0.012
		Sc. rank:	13152.0

,

PA

Pos.	191	obs:	exp:
caa	Q	40562	24857.00
cag	Q	102	15807.00
atg	M	1	1.00
---	--	------	------
mPD		0.0052	0.48
		nPD:	0.01
		N. weight:	0.53
		Sc. PD:	-0.023
		Sc. rank:	-8253.5

,

PA

Pos.	192	obs:	exp:
gat	D	0	2.83
gac	D	5	2.17
gaa	E	39986	23972.00
gag	E	674	16688.00
---	--	------	------
mPD		0.033	0.48
		nPD:	0.07
		N. weight:	0.5
		Sc. PD:	0.0057
		Sc. rank:	9421.6

,

PA

Pos.	193	obs:	exp:
tta	L	0	10.44
ttg	L	6	18.61
ctt	L	0	18.83
ctc	L	0	13.20
cta	L	0	13.82
ctg	L	84	15.10
atg	M	40575	40575.00
---	--	------	------
mPD		0.0044	0.0072
		nPD:	0.61
		N. weight:	0.0038
		Sc. PD:	0.0021
		Sc. rank:	118.7

,

PA

Pos.	194	obs:	exp:
gct	A	455	11083.00
gcc	A	40157	10160.00
gca	A	52	16449.00
gcg	A	0	2973.00
---	-	1	1.00
---	--	------	------
mPD		0.025	0.69
		nPD:	0.04
		N. weight:	1.5
		Sc. PD:	-0.029
		Sc. rank:	12310.0

,

PA

Pos.	195	obs:	exp:
tct	S	0	36.31
tcc	S	0	33.62
tca	S	0	60.48
tcg	S	0	8.86
cat	H	0	1.13
cac	H	2	0.87
cgt	R	0	0.02
cgc	R	0	0.04
cga	R	0	0.09
cgg	R	0	0.09
act	T	0	0.31
acc	T	1	0.18
aca	T	0	0.43
acg	T	0	0.08
aat	N	332	22622.00
aac	N	40103	17813.00
aaa	K	1	0.66
aag	K	0	0.34
agt	S	203	39.32
agc	S	22	46.42
aga	R	1	0.49
agg	R	0	0.28
---	--	------	------
mPD		0.037	0.51
		nPD:	0.07
		N. weight:	0.86
		Sc. PD:	0.014
		Sc. rank:	17140.0

,

PA

Pos.	196	obs:	exp:
cgt	R	1	806.00
cgc	R	0	1463.00
cga	R	4	3709.00
cgg	R	0	3491.00
aaa	K	11	7.23
aag	K	0	3.77
aga	R	40346	19940.00
agg	R	302	11245.00
ggt	G	0	0.13
ggc	G	0	0.14
gga	G	1	0.46
ggg	G	0	0.26
---	--	------	------
mPD		0.016	0.89
		nPD:	0.02
		N. weight:	0.74
		Sc. PD:	-0.028
		Sc. rank:	-4981.8

,

PA

Pos.	197	obs:	exp:
tct	S	0	5.00
tcc	S	0	4.63
tca	S	0	8.33
tcg	S	0	1.22
tgt	C	0	0.39
tgc	C	1	0.61
agt	S	23	5.42
agc	S	8	6.40
ggt	G	252	5470.00
ggc	G	40361	5671.00
gga	G	20	18757.00
ggg	G	0	10735.00
---	--	------	------
mPD		0.016	0.68
		nPD:	0.02
		N. weight:	2.1
		Sc. PD:	-0.067
		Sc. rank:	-1387.7

,

PA

Pos.	198	obs:	exp:
ttt	F	0	0.49
ttc	F	1	0.51
tta	L	3	4716.00
ttg	L	0	8402.00
ctt	L	40	8502.00
ctc	L	40077	5960.00
cta	L	514	6241.00
ctg	L	7	6821.00
att	I	0	8.07
atc	I	23	6.04
ata	I	0	8.88
---	--	------	------
mPD		0.029	1.2
		nPD:	0.02
		N. weight:	2.
		Sc. PD:	-0.062
		Sc. rank:	1403.1

,

PA

Pos.	199	obs:	exp:
tta	L	0	0.12
ttg	L	0	0.21
tgg	W	40664	40664.00
ctt	L	1	0.21
ctc	L	0	0.15
cta	L	0	0.15
ctg	L	0	0.17
---	--	------	------
mPD		0.00015	0.00011
		nPD:	1.3
		N. weight:	0.000043
		Sc. PD:	0.000052
		Sc. rank:	1.4

,

PA			
Pos.	200	obs:	exp:
caa	Q	0	0.61
cag	Q	1	0.39
gat	D	40597	22986.00
gac	D	67	17678.00
---	--	------	------
mPD		0.0034	0.49
		nPD:	0.01
		N. weight:	0.62
		Sc. PD:	-0.029
		Sc. rank:	-12513.0

,

PA			
Pos.	201	obs:	exp:
ttt	F	0	2.43
ttc	F	5	2.57
tct	S	25	6558.00
tcc	S	40554	6074.00
tca	S	64	10924.00
tcg	S	0	1600.00
cct	P	0	5.33
ccc	P	16	3.00
cca	P	0	5.41
ccg	P	0	2.26
act	T	0	0.31
acc	T	1	0.18
aca	T	0	0.43
acg	T	0	0.08
agt	S	0	7102.00
agc	S	0	8385.00
---	--	------	------
mPD		0.0055	1.6
		nPD:	0.
		N. weight:	2.1
		Sc. PD:	-0.11
		Sc. rank:	-53602.0

,

PA			
Pos.	202	obs:	exp:
ttt	F	40656	19780.00
ttc	F	7	20883.00
tta	L	0	0.23
ttg	L	0	0.41
ctt	L	1	0.42
ctc	L	1	0.29
cta	L	0	0.31
ctg	L	0	0.34
---	--	------	------
mPD		0.00049	0.50
		nPD:	0.
		N. weight:	0.8
		Sc. PD:	-0.043
		Sc. rank:	-24122.0

,

PA			
Pos.	203	obs:	exp:
tta	L	0	0.23
ttg	L	0	0.41
tct	S	0	0.48
tcc	S	0	0.45
tca	S	0	0.81
tcg	S	0	0.12
tgt	C	2	0.78
tgc	C	0	1.22
ctt	L	2	0.42
ctc	L	0	0.29
cta	L	0	0.31
ctg	L	0	0.34
cat	H	11	6.20
cac	H	0	4.80
caa	Q	0	0.61
cag	Q	1	0.39
cgt	R	40619	805.90
cgc	R	21	1462.00
cga	R	2	3709.00
cgg	R	4	3490.00
agt	S	3	0.52
agc	S	0	0.62
aga	R	0	19936.00
agg	R	0	11243.00
---	--	------	------
mPD		0.0023	0.89
		nPD:	0.
		N. weight:	4.3
		Sc. PD:	-0.22
		Sc. rank:	-119920.0

,

PA			
Pos.	204	obs:	exp:
tta	L	0	4.06
ttg	L	0	7.24
ctt	L	0	7.32
ctc	L	0	5.13
cta	L	0	5.37
ctg	L	35	5.87
cat	H	6	4.51
cac	H	2	3.49
caa	Q	7541	24829.00
cag	Q	33077	15789.00
aaa	K	1	2.63
aag	K	3	1.37
---	--	------	------
mPD		0.30	0.48
		nPD:	0.64
		N. weight:	0.42
		Sc. PD:	0.24
		Sc. rank:	13339.0

,

PA			
Pos.	205	obs:	exp:
tct	S	62	6562.00
tcc	S	40571	6077.00
tca	S	31	10930.00
tcg	S	0	1601.00
att	I	0	0.35
atc	I	1	0.26
ata	I	0	0.39
agt	S	0	7106.00
agc	S	0	8389.00
---	--	------	------
mPD		0.0047	1.6
		nPD:	0.
		N. weight:	2.1
		Sc. PD:	-0.11
		Sc. rank:	-56730.0

,

PA			
Pos.	206	obs:	exp:
gaa	E	39952	23975.00
gag	E	713	16690.00
---	--	------	------
mPD		0.034	0.48
		nPD:	0.07
		N. weight:	0.49
		Sc. PD:	0.0073
		Sc. rank:	9753.5

,

PA			
Pos.	207	obs:	exp:
tct	S	0	0.48
tcc	S	0	0.45
tca	S	0	0.81
tcg	S	0	0.12
cgt	R	0	806.10
cgc	R	0	1463.00
cga	R	3	3710.00
cgg	R	0	3491.00
att	I	0	0.35
atc	I	0	0.26
ata	I	1	0.39
aaa	K	3	1.97
aag	K	0	1.03
agt	S	3	0.52
agc	S	0	0.62
aga	R	40649	19942.00
agg	R	5	11246.00
---	-	1	1.00
---	--	------	------
mPD		0.00074	0.89
		nPD:	0.
		N. weight:	0.79
		Sc. PD:	-0.042
		Sc. rank:	-24236.0

,

PA			
Pos.	208	obs:	exp:
tct	S	0	1.61
tcc	S	0	1.49
tca	S	0	2.69
tcg	S	0	0.39
agt	S	0	1.75
agc	S	10	2.06
ggt	G	15	5473.00
ggc	G	40616	5674.00
gga	G	23	18767.00
ggg	G	0	10740.00
---	-	1	1.00
---	--	------	------
mPD		0.0024	0.68
		nPD:	0.
		N. weight:	2.2
		Sc. PD:	-0.11
		Sc. rank:	-55399.0

,

PA			
Pos.	209	obs:	exp:
aaa	K	1	0.66
aag	K	0	0.34
gaa	E	40615	23973.00
gag	E	48	16690.00
---	-	1	1.00
---	--	------	------
mPD		0.0024	0.48
		nPD:	0.
		N. weight:	0.58
		Sc. PD:	-0.028
		Sc. rank:	-13190.0

,

PA			
Pos.	210	obs:	exp:
tct	S	0	0.16
tcc	S	0	0.15
tca	S	0	0.27
tcg	S	0	0.04
aaa	K	2	1.31
aag	K	0	0.69
agt	S	0	0.17
agc	S	1	0.21
gat	D	0	2.83
gac	D	5	2.17
gaa	E	40380	23966.00
gag	E	270	16684.00
ggt	G	0	0.94
ggc	G	0	0.98
gga	G	6	3.23
ggg	G	1	1.85
---	--	------	------
mPD		0.014	0.48
		nPD:	0.03
		N. weight:	0.54
		Sc. PD:	-0.014
		Sc. rank:	2386.3

,

PA			
Pos.	211	obs:	exp:
att	I	0	0.35
atc	I	0	0.26
ata	I	1	0.39
act	T	0	12628.00
acc	T	1	7189.00
aca	T	40614	17598.00
acg	T	49	3248.00
---	--	------	------
mPD		0.0025	0.68
		nPD:	0.
		N. weight:	0.92
		Sc. PD:	-0.047
		Sc. rank:	-23200.0

,

PA

Pos.	212	obs:	exp:
tta	L	0	0.35
ttg	L	0	0.62
ctt	L	3	0.63
ctc	L	0	0.44
cta	L	0	0.46
ctg	L	0	0.50
att	I	40449	14251.00
atc	I	147	10667.00
ata	I	0	15678.00
atg	M	2	2.00
act	T	14	4.66
acc	T	1	2.65
aca	T	0	6.49
acg	T	0	1.20
gtt	V	49	12.27
gtc	V	0	9.98
gta	V	0	10.62
gtg	V	0	16.12
---	--	------	------
mPD		0.011	0.66
		nPD:	0.02
		N. weight:	1.1
		Sc. PD:	-0.044
		Sc. rank:	-10136.0

PA

Pos.	213	obs:	exp:
aaa	K	1	0.66
aag	K	0	0.34
gaa	E	40642	23974.00
gag	E	22	16690.00
---	--	------	------
mPD		0.0011	0.48
		nPD:	0.
		N. weight:	0.58
		Sc. PD:	-0.03
		Sc. rank:	-16597.0

PA

Pos.	214	obs:	exp:
gat	D	1	0.57
gac	D	0	0.43
gaa	E	40600	23974.00
gag	E	64	16690.00
---	--	------	------
mPD		0.0032	0.48
		nPD:	0.01
		N. weight:	0.57
		Sc. PD:	-0.027
		Sc. rank:	-11999.0

PA

Pos.	215	obs:	exp:
cgt	R	0	5.45
cgc	R	0	9.89
cga	R	0	25.09
cgg	R	0	23.61
aat	N	1	0.56
aac	N	0	0.44
aaa	K	40349	26553.00
aag	K	40	13836.00
aga	R	269	134.90
agg	R	6	76.07
---	--	------	------
mPD		0.016	0.47
		nPD:	0.03
		N. weight:	0.46
		Sc. PD:	-0.0099
		Sc. rank:	3579.9

PA

Pos.	216	obs:	exp:
ttt	F	40617	19780.00
ttc	F	48	20885.00
---	--	------	------
mPD		0.0024	0.50
		nPD:	0.
		N. weight:	0.79
		Sc. PD:	-0.039
		Sc. rank:	-18353.0

PA

Pos.	217	obs:	exp:
aaa	K	20	13.15
aag	K	0	6.85
gct	A	0	0.55
gcc	A	0	0.50
gca	A	2	0.81
gcg	A	0	0.15
gat	D	1	0.57
gac	D	0	0.43
gaa	E	40575	23958.00
gag	E	62	16679.00
ggt	G	0	0.67
ggc	G	0	0.70
gga	G	5	2.31
ggg	G	0	1.32
---	--	------	------
mPD		0.0044	0.49
		nPD:	0.01
		N. weight:	0.57
		Sc. PD:	-0.026
		Sc. rank:	-9838.9

PA

Pos.	218	obs:	exp:
tta	L	0	0.12
ttg	L	0	0.21
ctt	L	0	0.21
ctc	L	1	0.15
cta	L	0	0.15
ctg	L	0	0.17
att	I	246	14274.00
atc	I	40410	10684.00
ata	I	6	15704.00
gtt	V	1	0.50
gtc	V	1	0.41
gta	V	0	0.43
gtg	V	0	0.66
---	--	------	------
mPD		0.013	0.66
		nPD:	0.02
		N. weight:	1.4
		Sc. PD:	-0.052
		Sc. rank:	-7941.6

PA

Pos.	219	obs:	exp:
tct	S	0	86.01
tcc	S	0	79.65
tca	S	533	143.30
tcg	S	0	20.98
att	I	0	3.16
atc	I	0	2.36
ata	I	9	3.48
act	T	54	12456.00
acc	T	0	7091.00
aca	T	40045	17358.00
acg	T	9	3203.00
aaa	K	11	7.23
aag	K	0	3.77
agt	S	0	93.14
agc	S	0	110.00
gct	A	0	1.09
gcc	A	0	1.00
gca	A	4	1.62
gcg	A	0	0.29
---	--	------	------
mPD		0.030	0.71
		nPD:	0.04
		N. weight:	0.92
		Sc. PD:	-0.012
		Sc. rank:	11105.0

PA

Pos.	220	obs:	exp:
cgt	R	0	0.61
cgc	R	0	1.12
cga	R	0	2.83
cgg	R	0	2.66
aga	R	31	15.20
agg	R	0	8.57
gaa	E	2	1.18
gag	E	0	0.82
ggt	G	4	5470.00
ggc	G	2	5671.00
gga	G	39746	18756.00
ggg	G	880	10735.00
---	--	------	------
mPD		0.044	0.68
		nPD:	0.06
		N. weight:	0.75
		Sc. PD:	0.0065
		Sc. rank:	13593.0

PA

Pos.	221	obs:	exp:
att	I	15	5.27
atc	I	0	3.94
ata	I	0	5.79
act	T	39449	12616.00
acc	T	1124	7183.00
aca	T	48	17582.00
acg	T	5	3245.00
aat	N	2	1.12
aac	N	0	0.88
gct	A	21	6.00
gcc	A	1	5.50
gca	A	0	8.90
gcg	A	0	1.61
---	--	------	------
mPD		0.058	0.68
		nPD:	0.09
		N. weight:	1.2
		Sc. PD:	0.033
		Sc. rank:	25228.0

PA

Pos.	222	obs:	exp:
tta	L	0	0.81
ttg	L	2	1.45
ctt	L	0	1.46
ctc	L	0	1.03
cta	L	0	1.07
ctg	L	5	1.17
att	I	10	23.87
atc	I	3	17.87
ata	I	55	26.26
atg	M	40569	40569.00
gtt	V	0	5.26
gtc	V	0	4.28
gta	V	0	4.55
gtg	V	21	6.91
---	--	------	------
mPD		0.0047	0.0056
		nPD:	0.84
		N. weight:	0.0017
		Sc. PD:	0.0013
		Sc. rank:	53.9

PA

Pos.	223	obs:	exp:
tta	L	0	1.86
ttg	L	0	3.31
tgt	C	0	0.39
tgc	C	1	0.61
ctt	L	0	3.35
ctc	L	16	2.35
cta	L	0	2.46
ctg	L	0	2.69
cat	H	1	3.95
cac	H	6	3.05
cgt	R	2484	805.80
cgc	R	38072	1462.00
cga	R	85	3708.00
cgg	R	0	3490.00
aga	R	0	19934.00
agg	R	0	11241.00
---	--	------	------
mPD		0.12	0.89
		nPD:	0.13
		N. weight:	3.4
		Sc. PD:	0.26
		Sc. rank:	88937.0

PA

Pos.	224	obs:	exp:
tct	S	0	0.16
tcc	S	0	0.15
tca	S	0	0.27
tcg	S	0	0.04
tgg	W	1	1.00
cgt	R	0	805.10
cgc	R	0	1461.00
cga	R	0	3705.00
cgg	R	7	3487.00
atg	M	8	8.00
aaa	K	4	33.53
aag	K	47	17.47
agt	S	1	0.17
agc	S	0	0.21
aga	R	10	19916.00
agg	R	40587	11231.00
---	--	------	------
mPD		0.0040	0.89
		nPD:	0.
		N. weight:	1.4
		Sc. PD:	-0.071
		Sc. rank:	-33606.0

,

PA

Pos.	225	obs:	exp:
ttt	F	6	2.92
ttc	F	0	3.08
tta	L	0	4718.00
ttg	L	0	8405.00
ctt	L	40618	8505.00
ctc	L	31	5962.00
cta	L	2	6243.00
ctg	L	5	6823.00
att	I	3	1.05
atc	I	0	0.79
ata	I	0	1.16
---	--	------	------
mPD		0.0023	1.2
		nPD:	0.
		N. weight:	1.7
		Sc. PD:	-0.09
		Sc. rank:	-49986.0

,

PA

Pos.	226	obs:	exp:
act	T	0	0.93
acc	T	3	0.53
aca	T	0	1.30
acg	T	0	0.24
gct	A	267	11082.00
gcc	A	40336	10159.00
gca	A	57	16448.00
gcg	A	1	2972.00
ggt	G	0	0.13
ggc	G	1	0.14
gga	G	0	0.46
ggg	G	0	0.26
---	--	------	------
mPD		0.016	0.69
		nPD:	0.02
		N. weight:	1.5
		Sc. PD:	-0.047
		Sc. rank:	-1395.7

,

PA

Pos.	227	obs:	exp:
tat	Y	0	1.58
tac	Y	3	1.42
cat	H	0	1.13
cac	H	2	0.87
aat	N	0	530.40
aac	N	948	417.60
gtt	V	0	1.25
gtc	V	5	1.02
gta	V	0	1.08
gtg	V	0	1.65
gat	D	65	22436.00
gac	D	39626	17255.00
gaa	E	13	7.66
gag	E	0	5.34
ggt	G	0	0.40
ggc	G	3	0.42
gga	G	0	1.38
ggg	G	0	0.79
---	--	------	------
mPD		0.050	0.54
		nPD:	0.09
		N. weight:	0.91
		Sc. PD:	0.033
		Sc. rank:	20619.0

,

PA

Pos.	228	obs:	exp:
tta	L	0	1.16
ttg	L	0	2.07
ctt	L	0	2.09
ctc	L	0	1.47
cta	L	10	1.54
ctg	L	0	1.68
cat	H	1	0.56
cac	H	0	0.44
caa	Q	40639	24849.00
cag	Q	12	15802.00
aaa	K	3	1.97
aag	K	0	1.03
---	--	------	------
mPD		0.0013	0.48
		nPD:	0.
		N. weight:	0.54
		Sc. PD:	-0.028
		Sc. rank:	-14939.0

,

PA

Pos.	229	obs:	exp:
tct	S	0	6562.00
tcc	S	0	6077.00
tca	S	0	10929.00
tcg	S	0	1601.00
agt	S	40439	7106.00
agc	S	224	8389.00
ggt	G	2	0.27
ggc	G	0	0.28
gga	G	0	0.92
ggg	G	0	0.53
---	--	------	------
mPD		0.011	1.6
		nPD:	0.01
		N. weight:	1.9
		Sc. PD:	-0.09
		Sc. rank:	-38707.0

,

PA

Pos.	230	obs:	exp:
tta	L	0	4718.00
ttg	L	0	8406.00
ctt	L	32	8505.00
ctc	L	40621	5962.00
cta	L	5	6243.00
ctg	L	0	6824.00
att	I	0	2.11
atc	I	6	1.58
ata	I	0	2.32
gtt	V	0	0.25
gtc	V	1	0.20
gta	V	0	0.22
gtg	V	0	0.33
---	--	------	------
mPD		0.0022	1.2
		nPD:	0.
		N. weight:	2.1
		Sc. PD:	-0.11
		Sc. rank:	-62671.0

,

PA

Pos.	231	obs:	exp:
cct	P	11	13551.00
ccc	P	0	7624.00
cca	P	40169	13739.00
ccg	P	485	5751.00
---	--	------	------
mPD		0.024	0.72
		nPD:	0.03
		N. weight:	1.1
		Sc. PD:	-0.025
		Sc. rank:	8741.8

,

PA

Pos.	232	obs:	exp:
tta	L	0	0.70
ttg	L	0	1.24
tct	S	0	0.32
tcc	S	0	0.30
tca	S	0	0.54
tcg	S	2	0.08
ctt	L	0	1.26
ctc	L	0	0.88
cta	L	0	0.92
ctg	L	6	1.01
cct	P	9	13548.00
ccc	P	0	7622.00
cca	P	23	13735.00
ccg	P	40623	5750.00
act	T	0	0.62
acc	T	0	0.35
aca	T	0	0.87
acg	T	2	0.16
agt	S	0	0.35
agc	S	0	0.41
---	--	------	------
mPD		0.0021	0.72
		nPD:	0.
		N. weight:	2.2
		Sc. PD:	-0.11
		Sc. rank:	-58306.0

,

PA

Pos.	233	obs:	exp:
tct	S	0	1.61
tcc	S	0	1.49
tca	S	0	2.69
tcg	S	0	0.39
tat	Y	0	0.53
tac	Y	1	0.47
cat	H	0	2.26
cac	H	4	1.74
aat	N	24	22741.00
aac	N	40623	17906.00
aaa	K	3	1.97
aag	K	0	1.03
agt	S	0	1.75
agc	S	10	2.06
---	--	------	------
mPD		0.0021	0.49
		nPD:	0.
		N. weight:	0.9
		Sc. PD:	-0.045
		Sc. rank:	-22181.0

,

PA

Pos.	234	obs:	exp:
ttt	F	5	19780.00
ttc	F	40660	20885.00
---	--	------	------
mPD		0.00025	0.50
		nPD:	0.
		N. weight:	0.74
		Sc. PD:	-0.04
		Sc. rank:	-23082.0

,

PA

Pos.	235	obs:	exp:
ttt	F	0	0.97
ttc	F	2	1.03
tct	S	12	6561.00
tcc	S	40633	6076.00
tca	S	11	10928.00
tcg	S	0	1600.00
cct	P	0	2.33
ccc	P	7	1.31
cca	P	0	2.36
ccg	P	0	0.99
agt	S	0	7105.00
agc	S	0	8387.00
---	--	------	------
mPD		0.0016	1.6
		nPD:	0.
		N. weight:	2.1
		Sc. PD:	-0.11
		Sc. rank:	-63943.0

,

PA

Pos.	236	obs:	exp:
tct	S	0	35.82
tcc	S	0	33.18
tca	S	0	59.67
tcg	S	0	8.74
tgt	C	10	15803.00
tgc	C	40428	24635.00
cgt	R	0	0.04
cgc	R	2	0.07
cga	R	0	0.18
cgg	R	0	0.17
aat	N	0	1.12
aac	N	2	0.88
agt	S	4	38.80
agc	S	218	45.80
aga	R	0	0.98
agg	R	0	0.55
ggt	G	0	0.13
ggc	G	1	0.14
gga	G	0	0.46
ggg	G	0	0.26
---	--	------	------
mPD		0.012	0.49
		nPD:	0.02
		N. weight:	0.55
		Sc. PD:	-0.017
		Sc. rank:	-45.3

,

PA

Pos.	237	obs:	exp:
ttt	F	9	4.38
ttc	F	0	4.62
tta	L	0	4715.00
ttg	L	0	8400.00
ctt	L	40619	8499.00
ctc	L	7	5958.00
cta	L	0	6239.00
ctg	L	3	6819.00
att	I	27	9.48
atc	I	0	7.09
ata	I	0	10.43
---	--	------	------
mPD		0.0023	1.2
		nPD:	0.
		N. weight:	1.7
		Sc. PD:	-0.091
		Sc. rank:	-50783.0

,

PA

Pos.	238	obs:	exp:
aaa	K	0	9.20
aag	K	14	4.80
gct	A	0	0.82
gcc	A	0	0.75
gca	A	0	1.21
gcg	A	3	0.22
gat	D	10	5.65
gac	D	0	4.35
gaa	E	321	23958.00
gag	E	40316	16679.00
ggt	G	0	0.13
ggc	G	0	0.14
gga	G	0	0.46
ggg	G	1	0.26
---	--	------	------
mPD		0.017	0.49
		nPD:	0.04
		N. weight:	0.93
		Sc. PD:	-0.019
		Sc. rank:	7766.9

,

PA

Pos.	239	obs:	exp:
tct	S	0	4.36
tcc	S	0	4.03
tca	S	0	7.26
tcg	S	0	1.06
tat	Y	2	1.05
tac	Y	0	0.95
att	I	6	2.11
atc	I	0	1.58
ata	I	0	2.32
act	T	9	2.79
acc	T	0	1.59
aca	T	0	3.89
acg	T	0	0.72
aat	N	40279	22716.00
aac	N	323	17886.00
aaa	K	16	10.52
aag	K	0	5.48
agt	S	27	4.72
agc	S	0	5.57
gat	D	3	1.70
gac	D	0	1.30
---	--	------	------
mPD		0.019	0.50
		nPD:	0.04
		N. weight:	0.59
		Sc. PD:	-0.01
		Sc. rank:	5841.6

,

PA

Pos.	240	obs:	exp:
ttt	F	40640	19780.00
ttc	F	25	20885.00
---	--	------	------
mPD		0.0012	0.50
		nPD:	0.
		N. weight:	0.79
		Sc. PD:	-0.041
		Sc. rank:	-22410.0

,

PA

Pos.	241	obs:	exp:
cgt	R	0	806.30
cgc	R	0	1463.00
cga	R	0	3710.00
cgg	R	0	3492.00
aga	R	40651	19946.00
agg	R	14	11248.00
---	--	------	------
mPD		0.00069	0.89
		nPD:	0.
		N. weight:	0.79
		Sc. PD:	-0.042
		Sc. rank:	-24250.0

,

PA

Pos.	242	obs:	exp:
tct	S	0	0.16
tcc	S	1	0.15
tca	S	0	0.27
tcg	S	0	0.04
act	T	0	4.66
acc	T	15	2.65
aca	T	0	6.49
acg	T	0	1.20
agt	S	0	0.17
agc	S	0	0.21
gtt	V	0	3.01
gtc	V	12	2.45
gta	V	0	2.60
gtg	V	0	3.95
gct	A	8	11076.00
gcc	A	40595	10153.00
gca	A	34	16438.00
gcg	A	0	2971.00
---	--	------	------
mPD		0.0034	0.70
		nPD:	0.
		N. weight:	1.5
		Sc. PD:	-0.076
		Sc. rank:	-35052.0

,

PA

Pos.	243	obs:	exp:
ttt	F	2	0.97
ttc	F	0	1.03
tat	Y	40662	21434.00
tac	Y	1	19229.00
---	--	------	------
mPD		0.00015	0.50
		nPD:	0.
		N. weight:	0.71
		Sc. PD:	-0.038
		Sc. rank:	-22351.0

,

PA

Pos.	244	obs:	exp:
gtt	V	4	10186.00
gtc	V	1	8286.00
gta	V	241	8814.00
gtg	V	40419	13380.00
---	--	------	------
mPD		0.012	0.74
		nPD:	0.02
		N. weight:	1.2
		Sc. PD:	-0.046
		Sc. rank:	-10293.0

,

PA

Pos.	245	obs:	exp:
aat	N	2	1.12
aac	N	0	0.88
gat	D	40627	22985.00
gac	D	36	17678.00
---	--	------	------
mPD		0.0019	0.49
		nPD:	0.
		N. weight:	0.62
		Sc. PD:	-0.032
		Sc. rank:	-15720.0

,

PA

Pos.	246	obs:	exp:
cgt	R	0	0.40
cgc	R	0	0.72
cga	R	0	1.82
cgg	R	0	1.72
aaa	K	1	0.66
aag	K	0	0.34
aga	R	20	9.81
agg	R	0	5.53
ggt	G	1	5472.00
ggc	G	2	5673.00
gga	G	40616	18762.00
ggg	G	25	10738.00
---	--	------	------
mPD		0.0025	0.68
		nPD:	0.
		N. weight:	0.85
		Sc. PD:	-0.043
		Sc. rank:	-21520.0

,

PA

Pos.	247	obs:	exp:
ttt	F	131	19780.00
ttc	F	40534	20885.00
---	--	------	------
mPD		0.0064	0.50
		nPD:	0.01
		N. weight:	0.71
		Sc. PD:	-0.03
		Sc. rank:	-9348.4

,

PA			
Pos.	248	obs:	exp:
aaa	K	88	57.85
aag	K	0	30.15
gtt	V	0	1.75
gtc	V	0	1.43
gta	V	7	1.52
gtg	V	0	2.30
gaa	E	40477	23917.00
gag	E	91	16651.00
ggt	G	0	0.27
ggc	G	0	0.28
gga	G	2	0.92
ggg	G	0	0.53
---	--	------	------
mPD		0.0092	0.49
		nPD:	0.02
		N. weight:	0.57
		Sc. PD:	-0.02
		Sc. rank:	-3237.0

PA			
Pos.	249	obs:	exp:
tta	L	0	0.35
ttg	L	0	0.62
tct	S	0	0.32
tcc	S	0	0.30
tca	S	0	0.54
tcg	S	2	0.08
ctt	L	0	0.63
ctc	L	0	0.44
cta	L	0	0.46
ctg	L	3	0.50
cct	P	3	13547.00
ccc	P	0	7621.00
cca	P	278	13735.00
ccg	P	40372	5750.00
caa	Q	0	0.61
cag	Q	1	0.39
act	T	0	1.86
acc	T	0	1.06
aca	T	0	2.60
acg	T	6	0.48
agt	S	0	0.35
agc	S	0	0.41
---	--	------	------
mPD		0.014	0.72
		nPD:	0.02
		N. weight:	2.1
		Sc. PD:	-0.074
		Sc. rank:	-9121.1

PA			
Pos.	250	obs:	exp:
tct	S	0	0.48
tcc	S	0	0.45
tca	S	0	0.81
tcg	S	0	0.12
aat	N	72	22749.00
aac	N	40589	17912.00
agt	S	0	0.52
agc	S	3	0.62
gat	D	0	0.57
gac	D	1	0.43
---	--	------	------
mPD		0.0037	0.49
		nPD:	0.01
		N. weight:	0.89
		Sc. PD:	-0.042
		Sc. rank:	-16953.0

PA			
Pos.	251	obs:	exp:
tct	S	0	0.65
tcc	S	0	0.60
tca	S	0	1.08
tcg	S	0	0.16
agt	S	0	0.70
agc	S	4	0.83
gtt	V	0	0.25
gtc	V	1	0.20
gta	V	0	0.22
gtg	V	0	0.33
ggt	G	40	5474.00
ggc	G	40395	5675.00
gga	G	221	18769.00
ggg	G	4	10742.00
---	--	------	------
mPD		0.013	0.68
		nPD:	0.02
		N. weight:	2.1
		Sc. PD:	-0.075
		Sc. rank:	-10592.0

PA			
Pos.	252	obs:	exp:
ttt	F	1	6.32
ttc	F	12	6.68
tct	S	0	0.48
tcc	S	1	0.45
tca	S	0	0.81
tcg	S	0	0.12
tat	Y	0	36.90
tac	Y	70	33.10
tgt	C	41	15854.00
tgc	C	40529	24716.00
cgt	R	0	0.18
cgc	R	9	0.32
cga	R	0	0.82
cgg	R	0	0.77
agt	S	0	0.52
agc	S	2	0.62
aga	R	0	4.41
agg	R	0	2.49
---	--	------	------
mPD		0.0067	0.48
		nPD:	0.01
		N. weight:	0.54
		Sc. PD:	-0.022
		Sc. rank:	-6010.4

PA			
Pos.	253	obs:	exp:
tta	L	0	0.23
ttg	L	0	0.41
ctt	L	2	0.42
ctc	L	0	0.29
cta	L	0	0.31
ctg	L	0	0.34
att	I	40658	14274.00
atc	I	4	10685.00
ata	I	1	15704.00
---	--	------	------
mPD		0.00034	0.66
		nPD:	0.
		N. weight:	1.2
		Sc. PD:	-0.062
		Sc. rank:	-36068.0

PA			
Pos.	254	obs:	exp:
aaa	K	0	0.66
aag	K	1	0.34
gtt	V	0	1.00
gtc	V	0	0.82
gta	V	0	0.87
gtg	V	4	1.32
gaa	E	66	23970.00
gag	E	40592	16688.00
ggt	G	0	0.27
ggc	G	0	0.28
gga	G	0	0.92
ggg	G	2	0.53
---	--	------	------
mPD		0.0036	0.48
		nPD:	0.01
		N. weight:	0.97
		Sc. PD:	-0.046
		Sc. rank:	-18692.0

PA			
Pos.	255	obs:	exp:
tct	S	0	1.78
tcc	S	0	1.64
tca	S	0	2.96
tcg	S	0	0.43
agt	S	0	1.92
agc	S	11	2.27
ggt	G	21	5473.00
ggc	G	40624	5674.00
gga	G	9	18767.00
ggg	G	0	10740.00
---	--	------	------
mPD		0.0020	0.68
		nPD:	0.
		N. weight:	2.2
		Sc. PD:	-0.11
		Sc. rank:	-58313.0

PA			
Pos.	256	obs:	exp:
aat	N	2	1.12
aac	N	0	0.88
aaa	K	74	26733.00
aag	K	40589	13930.00
---	--	------	------
mPD		0.0037	0.45
		nPD:	0.01
		N. weight:	1.2
		Sc. PD:	-0.054
		Sc. rank:	-21134.0

PA			
Pos.	257	obs:	exp:
tta	L	0	4719.00
ttg	L	0	8407.00
ctt	L	40645	8507.00
ctc	L	11	5963.00
cta	L	2	6244.00
ctg	L	7	6825.00
---	--	------	------
mPD		0.00098	1.2
		nPD:	0.
		N. weight:	1.7
		Sc. PD:	-0.092
		Sc. rank:	-52943.0

PA			
Pos.	258	obs:	exp:
ttt	F	0	0.49
ttc	F	1	0.51
tct	S	40459	6561.00
tcc	S	65	6076.00
tca	S	134	10929.00
tcg	S	2	1601.00
agt	S	0	7105.00
agc	S	0	8388.00
gct	A	4	1.09
gcc	A	0	1.00
gca	A	0	1.62
gcg	A	0	0.29
---	--	------	------
mPD		0.010	1.6
		nPD:	0.01
		N. weight:	2.
		Sc. PD:	-0.096
		Sc. rank:	-42545.0

PA			
Pos.	259	obs:	exp:
tta	L	0	0.23
ttg	L	0	0.41
tct	S	0	0.16
tcc	S	0	0.15
tca	S	1	0.27
tcg	S	0	0.04
ctt	L	0	0.42
ctc	L	0	0.29
cta	L	2	0.31
ctg	L	0	0.34
cat	H	1	0.56
cac	H	0	0.44
caa	Q	40639	24855.00
cag	Q	22	15806.00
agt	S	0	0.17
agc	S	0	0.21
---	--	------	------
mPD		0.0013	0.48
		nPD:	0.
		N. weight:	0.54
		Sc. PD:	-0.028
		Sc. rank:	-14851.0

PA			
Pos.	260	obs:	exp:
att	I	0	1.40
atc	I	0	1.05
ata	I	4	1.54
atg	M	40659	40659.00
gtt	V	0	0.50
gtc	V	0	0.41
gta	V	0	0.43
gtg	V	2	0.66
---	--	------	------
mPD		0.00030	0.00036
		nPD:	0.82
		N. weight:	0.00016
		Sc. PD:	0.00012
		Sc. rank:	5.2

PA			
Pos.	261	obs:	exp:
ttt	F	0	1.95
ttc	F	4	2.05
tct	S	44	6558.00
tcc	S	38948	6073.00
tca	S	1647	10923.00
tcg	S	1	1600.00
tat	Y	0	0.53
tac	Y	1	0.47
cct	P	0	6.00
ccc	P	16	3.37
cca	P	2	6.08
ccg	P	0	2.55
act	T	0	0.62
acc	T	2	0.35
aca	T	0	0.87
acg	T	0	0.16
agt	S	0	7102.00
agc	S	0	8384.00
---	--	------	------
mPD		0.081	1.6
		nPD:	0.05
		N. weight:	1.9
		Sc. PD:	-0.011
		Sc. rank:	27323.0

PA			
Pos.	262	obs:	exp:
cgt	R	0	0.44
cgc	R	0	0.79
cga	R	0	2.01
cgg	R	0	1.89
aaa	K	40536	26720.00
aag	K	107	13923.00
aga	R	22	10.79
agg	R	0	6.09
---	--	------	------
mPD		0.0063	0.45
		nPD:	0.01
		N. weight:	0.45
		Sc. PD:	-0.018
		Sc. rank:	-4934.6

PA			
Pos.	263	obs:	exp:
aaa	K	84	56.54
aag	K	2	29.46
gtt	V	0	0.25
gtc	V	0	0.20
gta	V	1	0.22
gtg	V	0	0.33
gat	D	2	1.13
gac	D	0	0.87
gaa	E	40510	23918.00
gag	E	59	16651.00
ggt	G	0	0.94
ggc	G	0	0.98
gga	G	7	3.23
ggg	G	0	1.85
---	--	------	------
mPD		0.0077	0.49
		nPD:	0.02
		N. weight:	0.57
		Sc. PD:	-0.022
		Sc. rank:	-5358.9

PA			
Pos.	264	obs:	exp:
tta	L	0	0.12
ttg	L	1	0.21
ctt	L	0	0.21
ctc	L	0	0.15
cta	L	0	0.15
ctg	L	0	0.17
gtt	V	17	10185.00
gtc	V	0	8286.00
gta	V	206	8814.00
gtg	V	40441	13379.00
---	--	------	------
mPD		0.011	0.74
		nPD:	0.01
		N. weight:	1.2
		Sc. PD:	-0.048
		Sc. rank:	-12603.0

PA			
Pos.	265	obs:	exp:
tct	S	0	27.76
tcc	S	0	25.70
tca	S	0	46.23
tcg	S	0	6.77
cat	H	1	0.56
cac	H	0	0.44
act	T	1	0.31
acc	T	0	0.18
aca	T	0	0.43
acg	T	0	0.08
aat	N	40394	22638.00
aac	N	70	17826.00
aaa	K	3	1.97
aag	K	0	1.03
agt	S	170	30.06
agc	S	2	35.48
gat	D	24	13.57
gac	D	0	10.43
---	--	------	------
mPD		0.013	0.51
		nPD:	0.03
		N. weight:	0.63
		Sc. PD:	-0.018
		Sc. rank:	1223.0

PA			
Pos.	266	obs:	exp:
gct	A	279	11083.00
gcc	A	40216	10160.00
gca	A	170	16449.00
gcg	A	0	2973.00
---	--	------	------
mPD		0.022	0.69
		nPD:	0.03
		N. weight:	1.5
		Sc. PD:	-0.035
		Sc. rank:	8649.8

PA			
Pos.	267	obs:	exp:
tta	L	0	1.04
ttg	L	0	1.86
ctt	L	0	1.88
ctc	L	0	1.32
cta	L	9	1.38
ctg	L	0	1.51
cct	P	0	0.33
ccc	P	0	0.19
cca	P	1	0.34
ccg	P	0	0.14
cat	H	0	3.95
cac	H	7	3.05
caa	Q	39361	24078.00
cag	Q	28	15311.00
cgt	R	0	4.84
cgc	R	0	8.78
cga	R	214	22.26
cgg	R	0	20.95
aaa	K	1009	667.30
aag	K	6	347.70
aga	R	29	119.70
agg	R	1	67.49
---	--	------	------
mPD		0.065	0.55
		nPD:	0.12
		N. weight:	0.55
		Sc. PD:	0.033
		Sc. rank:	13436.0

PA			
Pos.	268	obs:	exp:
tgt	C	1	0.39
tgc	C	0	0.61
att	I	40631	14273.00
atc	I	19	10683.00
ata	I	8	15702.00
gtt	V	6	1.50
gtc	V	0	1.22
gta	V	0	1.30
gtg	V	0	1.97
---	--	------	------
mPD		0.0017	0.66
		nPD:	0.
		N. weight:	1.2
		Sc. PD:	-0.06
		Sc. rank:	-31795.0

PA			
Pos.	269	obs:	exp:
aaa	K	18	11.83
aag	K	0	6.17
gtt	V	0	0.25
gtc	V	0	0.20
gta	V	1	0.22
gtg	V	0	0.33
gat	D	1	15.83
gac	D	27	12.17
gaa	E	40554	23946.00
gag	E	63	16671.00
ggt	G	0	0.13
ggc	G	0	0.14
gga	G	1	0.46
ggg	G	0	0.26
---	--	------	------
mPD		0.0055	0.49
		nPD:	0.01
		N. weight:	0.57
		Sc. PD:	-0.025
		Sc. rank:	-8487.6

PA			
Pos.	270	obs:	exp:
tta	L	0	1.86
ttg	L	0	3.31
tct	S	6	0.97
tcc	S	0	0.90
tca	S	0	1.61
tcg	S	0	0.24
ctt	L	16	3.35
ctc	L	0	2.35
cta	L	0	2.46
ctg	L	0	2.69
cct	P	40367	13542.00
ccc	P	56	7619.00
cca	P	206	13730.00
ccg	P	9	5748.00
cat	H	0	1.13
cac	H	2	0.87
act	T	2	0.62
acc	T	0	0.35
aca	T	0	0.87
acg	T	0	0.16
agt	S	0	1.05
agc	S	0	1.24
gct	A	1	0.27
gcc	A	0	0.25
gca	A	0	0.40
gcg	A	0	0.07
---	--	------	------
mPD		0.015	0.72
		nPD:	0.02
		N. weight:	1.2
		Sc. PD:	-0.04
		Sc. rank:	-4698.8

PA			
Pos.	271	obs:	exp:
ttt	F	40380	19780.00
ttc	F	283	20883.00
tta	L	0	0.12
ttg	L	0	0.21
ctt	L	1	0.21
ctc	L	0	0.15
cta	L	0	0.15
ctg	L	0	0.17
gtt	V	1	0.25
gtc	V	0	0.20
gta	V	0	0.22
gtg	V	0	0.33
---	--	------	------
mPD		0.014	0.50
		nPD:	0.03
		N. weight:	0.75
		Sc. PD:	-0.021
		Sc. rank:	2698.7

PA			
Pos.	272	obs:	exp:
ttt	F	17	8.27
ttc	F	0	8.73
tta	L	0	4710.00
ttg	L	1767	8392.00
ctt	L	114	8491.00
ctc	L	10	5952.00
cta	L	101	6233.00
ctg	L	38600	6812.00
caa	Q	0	1.83
cag	Q	3	1.17
att	I	0	0.70
atc	I	0	0.53
ata	I	2	0.77
atg	M	50	50.00
gtt	V	0	0.25
gtc	V	0	0.20
gta	V	0	0.22
gtg	V	1	0.33
---	--	------	------
mPD		0.098	1.2
		nPD:	0.08
		N. weight:	1.7
		Sc. PD:	0.048
		Sc. rank:	36885.0

,

PA			
Pos.	273	obs:	exp:
tct	S	0	0.16
tcc	S	0	0.15
tca	S	0	0.27
tcg	S	0	0.04
cgt	R	0	4.90
cgc	R	0	8.89
cga	R	0	22.54
cgg	R	0	21.21
atg	M	1	1.00
act	T	0	4.35
acc	T	0	2.48
aca	T	2	6.06
acg	T	12	1.12
aat	N	1167	654.00
aac	N	2	515.00
aaa	K	157	25656.00
aag	K	38868	13369.00
agt	S	1	0.17
agc	S	0	0.21
aga	R	2	121.10
agg	R	245	68.32
gaa	E	0	122.60
gag	E	208	85.37
---	--	------	------
mPD		0.087	0.51
		nPD:	0.17
		N. weight:	1.1
		Sc. PD:	0.13
		Sc. rank:	30919.0

,

PA			
Pos.	274	obs:	exp:
tct	S	0	0.16
tcc	S	0	0.15
tca	S	1	0.27
tcg	S	0	0.04
cct	P	0	0.33
ccc	P	0	0.19
cca	P	1	0.34
ccg	P	0	0.14
cgt	R	0	0.10
cgc	R	0	0.18
cga	R	0	0.46
cgg	R	0	0.43
att	I	0	2.11
atc	I	0	1.58
ata	I	6	2.32
act	T	8	12585.00
acc	T	4	7165.00
aca	T	40352	17538.00
acg	T	160	3237.00
aaa	K	101	66.40
aag	K	0	34.60
agt	S	0	0.17
agc	S	0	0.21
aga	R	5	2.45
agg	R	0	1.38
gct	A	0	7.36
gcc	A	0	6.75
gca	A	27	10.92
gcg	A	0	1.97
---	--	------	------
mPD		0.015	0.69
		nPD:	0.02
		N. weight:	0.9
		Sc. PD:	-0.029
		Sc. rank:	-1605.0

,

PA			
Pos.	275	obs:	exp:
act	T	1	12629.00
acc	T	101	7190.00
aca	T	40499	17599.00
acg	T	64	3248.00
---	--	------	------
mPD		0.0081	0.68
		nPD:	0.01
		N. weight:	0.9
		Sc. PD:	-0.038
		Sc. rank:	-12499.0

,

PA			
Pos.	276	obs:	exp:
tct	S	0	0.32
tcc	S	0	0.30
tca	S	2	0.54
tcg	S	0	0.08
cct	P	8	13550.00
ccc	P	2	7623.00
cca	P	40543	13738.00
ccg	P	110	5751.00
agt	S	0	0.35
agc	S	0	0.41
---	--	------	------
mPD		0.0060	0.72
		nPD:	0.01
		N. weight:	1.2
		Sc. PD:	-0.055
		Sc. rank:	-21207.0

,

PA			
Pos.	277	obs:	exp:
cat	H	0	0.56
cac	H	1	0.44
cgt	R	1	805.10
cgc	R	193	1461.00
cga	R	47	3705.00
cgg	R	2	3487.00
act	T	0	0.31
acc	T	0	0.18
aca	T	1	0.43
acg	T	0	0.08
aaa	K	50	33.53
aag	K	1	17.47
aga	R	39305	19917.00
agg	R	1058	11232.00
ggt	G	0	0.81
ggc	G	0	0.84
gga	G	6	2.77
ggg	G	0	1.59
---	--	------	------
mPD		0.075	0.89
		nPD:	0.08
		N. weight:	0.64
		Sc. PD:	0.018
		Sc. rank:	13688.0

,

PA			
Pos.	278	obs:	exp:
tct	S	0	4.84
tcc	S	0	4.48
tca	S	30	8.06
tcg	S	0	1.18
cct	P	181	13539.00
ccc	P	29	7617.00
cca	P	40383	13726.00
ccg	P	35	5746.00
caa	Q	7	4.28
cag	Q	0	2.72
agt	S	0	5.24
agc	S	0	6.19
---	--	------	------
mPD		0.014	0.72
		nPD:	0.02
		N. weight:	1.2
		Sc. PD:	-0.041
		Sc. rank:	-6286.7

,

PA			
Pos.	279	obs:	exp:
ttt	F	0	0.97
ttc	F	2	1.03
tta	L	0	24.72
ttg	L	0	44.04
ctt	L	6	44.56
ctc	L	207	31.23
cta	L	0	32.71
ctg	L	0	35.75
att	I	88	14193.00
atc	I	40328	10623.00
ata	I	14	15614.00
gtt	V	0	5.01
gtc	V	20	4.08
gta	V	0	4.33
gtg	V	0	6.58
---	--	------	------
mPD		0.017	0.67
		nPD:	0.03
		N. weight:	1.5
		Sc. PD:	-0.044
		Sc. rank:	1638.1

,

PA			
Pos.	280	obs:	exp:
tct	S	0	0.16
tcc	S	0	0.15
tca	S	0	0.27
tcg	S	0	0.04
cgt	R	0	30.02
cgc	R	0	54.48
cga	R	0	138.10
cgg	R	0	130.00
act	T	0	0.93
acc	T	0	0.53
aca	T	3	1.30
acg	T	0	0.24
aat	N	3	8.95
aac	N	13	7.05
aaa	K	38988	25722.00
aag	K	137	13403.00
agt	S	0	0.17
agc	S	1	0.21
aga	R	1503	742.60
agg	R	11	418.80
gaa	E	6	3.54
gag	E	0	2.46
---	--	------	------
mPD		0.080	0.54
		nPD:	0.15
		N. weight:	0.45
		Sc. PD:	0.04
		Sc. rank:	11943.0

,

PA			
Pos.	281	obs:	exp:
ttt	F	13	6.32
ttc	F	0	6.68
tta	L	15	4716.00
ttg	L	186	8402.00
ctt	L	40211	8502.00
ctc	L	175	5960.00
cta	L	19	6241.00
ctg	L	35	6821.00
att	I	11	3.86
atc	I	0	2.89
ata	I	0	4.25
---	--	------	------
mPD		0.032	1.2
		nPD:	0.03
		N. weight:	1.7
		Sc. PD:	-0.046
		Sc. rank:	5413.7

,

PA			
Pos.	282	obs:	exp:
tct	S	4	0.65
tcc	S	0	0.60
tca	S	0	1.08
tcg	S	0	0.16
cct	P	36348	13549.00
ccc	P	2348	7623.00
cca	P	29	13737.00
ccg	P	1935	5751.00
act	T	1	0.31
acc	T	0	0.18
aca	T	0	0.43
acg	T	0	0.08
agt	S	0	0.70
agc	S	0	0.83
---	--	------	------
mPD		0.20	0.72
		nPD:	0.27
		N. weight:	0.84
		Sc. PD:	0.18
		Sc. rank:	24090.0

,

PA			
Pos.	283	obs:	exp:
tct	S	0	5729.00
tcc	S	0	5306.00
tca	S	0	9543.00
tcg	S	0	1398.00
cat	H	2	1.13
cac	H	0	0.87
cgt	R	1	0.12
cgc	R	0	0.22
cga	R	0	0.55
cgg	R	0	0.52
att	I	46	16.15
atc	I	0	12.09
ata	I	0	17.77
act	T	3	0.93
acc	T	0	0.53
aca	T	0	1.30
acg	T	0	0.24
aat	N	4640	2641.00
aac	N	81	2080.00
aaa	K	47	35.50
aag	K	7	18.50
agt	S	35252	6204.00
agc	S	252	7324.00
aga	R	4	2.94
agg	R	1	1.66
gat	D	312	176.90
gac	D	1	136.10
ggt	G	16	2.15
ggc	G	0	2.23
gga	G	0	7.39
ggg	G	0	4.23
---	--	------	------
mPD		0.26	1.8
		nPD:	0.15
		N. weight:	1.7
		Sc. PD:	0.15
		Sc. rank:	44933.0

,

PA			
Pos.	284	obs:	exp:
ggt	G	10	5475.00
ggc	G	4	5676.00
gga	G	40161	18772.00
ggg	G	490	10743.00
---	--	------	------
mPD		0.024	0.68
		nPD:	0.04
		N. weight:	0.79
		Sc. PD:	-0.015
		Sc. rank:	7073.9

,

PA			
Pos.	285	obs:	exp:
tta	L	0	1.62
ttg	L	0	2.89
tct	S	28	4.52
tcc	S	0	4.18
tca	S	0	7.53
tcg	S	0	1.10
ctt	L	12	2.93
ctc	L	1	2.05
cta	L	1	2.15
ctg	L	0	2.35
cct	P	40242	13535.00
ccc	P	361	7614.00
cca	P	4	13722.00
ccg	P	9	5745.00
cat	H	1	1.13
cac	H	1	0.87
act	T	5	1.55
acc	T	0	0.88
aca	T	0	2.16
acg	T	0	0.40
agt	S	0	4.89
agc	S	0	5.78
---	--	------	------
mPD		0.021	0.72
		nPD:	0.03
		N. weight:	1.2
		Sc. PD:	-0.031
		Sc. rank:	4949.2

,

PA			
Pos.	286	obs:	exp:
tta	L	0	4.87
ttg	L	0	8.68
tct	S	6	0.97
tcc	S	0	0.90
tca	S	0	1.61
tcg	S	0	0.24
ctt	L	41	8.79
ctc	L	1	6.16
cta	L	0	6.45
ctg	L	0	7.05
cct	P	40432	13535.00
ccc	P	174	7615.00
cca	P	6	13723.00
ccg	P	5	5745.00
agt	S	0	1.05
agc	S	0	1.24
---	--	------	------
mPD		0.011	0.72
		nPD:	0.02
		N. weight:	1.2
		Sc. PD:	-0.046
		Sc. rank:	-10972.0

,

PA			
Pos.	287	obs:	exp:
tgt	C	40404	15892.00
tgc	C	261	24773.00
---	--	------	------
mPD		0.013	0.48
		nPD:	0.03
		N. weight:	0.99
		Sc. PD:	-0.028
		Sc. rank:	2662.3

,

PA			
Pos.	288	obs:	exp:
ttt	F	118	57.88
ttc	F	1	61.12
tct	S	164	34.21
tcc	S	48	31.68
tca	S	0	56.98
tcg	S	0	8.35
tat	Y	39986	21145.00
tac	Y	129	18970.00
tgt	C	120	46.90
tgc	C	0	73.10
cat	H	95	54.71
cac	H	2	42.29
aat	N	2	1.12
aac	N	0	0.88
agt	S	0	37.05
agc	S	0	43.73
---	--	------	------
mPD		0.036	0.53
		nPD:	0.07
		N. weight:	0.69
		Sc. PD:	0.0075
		Sc. rank:	12870.0

,

PA			
Pos.	289	obs:	exp:
cat	H	1	0.56
cac	H	0	0.44
caa	Q	208	24856.00
cag	Q	40455	15807.00
aat	N	1	0.56
aac	N	0	0.44
---	--	------	------
mPD		0.010	0.48
		nPD:	0.02
		N. weight:	1.
		Sc. PD:	-0.034
		Sc. rank:	-2680.6

,

PA			
Pos.	290	obs:	exp:
caa	Q	1	0.61
cag	Q	0	0.39
cgt	R	19	806.30
cgc	R	2	1463.00
cga	R	31190	3710.00
cgg	R	9322	3492.00
aga	R	78	19945.00
agg	R	53	11248.00
---	--	------	------
mPD		0.36	0.89
		nPD:	0.41
		N. weight:	2.
		Sc. PD:	0.7
		Sc. rank:	62162.0

,

PA			
Pos.	291	obs:	exp:
tct	S	38	6561.00
tcc	S	40400	6076.00
tca	S	223	10929.00
tcg	S	0	1601.00
act	T	0	1.24
acc	T	4	0.71
aca	T	0	1.73
acg	T	0	0.32
agt	S	0	7106.00
agc	S	0	8388.00
---	--	------	------
mPD		0.013	1.6
		nPD:	0.01
		N. weight:	2.1
		Sc. PD:	-0.096
		Sc. rank:	-37581.0

,

PA

Pos.	292	obs:	exp:
caa	Q	1	0.61
cag	Q	0	0.39
aaa	K	37003	26734.00
aag	K	3661	13930.00
---	--	------	------
mPD		0.16	0.45
		nPD:	0.36
		N. weight :	0.19
		Sc. PD:	0.058
		Sc. rank :	5861.2

,

PA

Pos.	293	obs:	exp:
ttt	F	131	19780.00
ttc	F	40534	20885.00
---	--	------	------
mPD		0.0064	0.50
		nPD:	0.01
		N. weight :	0.71
		Sc. PD:	-0.03
		Sc. rank :	-9348.4

,

PA

Pos.	294	obs:	exp:
tta	L	0	4719.00
ttg	L	176	8407.00
ctt	L	1409	8506.00
ctc	L	39012	5963.00
cta	L	33	6244.00
ctg	L	34	6825.00
cct	P	0	0.33
ccc	P	1	0.19
cca	P	0	0.34
ccg	P	0	0.14
---	--	------	------
mPD		0.087	1.2
		nPD:	0.08
		N. weight :	1.9
		Sc. PD:	0.035
		Sc. rank :	39057.0

,

PA

Pos.	295	obs:	exp:
tta	L	1	4717.00
ttg	L	287	8404.00
ctt	L	18	8503.00
ctc	L	0	5961.00
cta	L	62	6242.00
ctg	L	40279	6822.00
atg	M	18	18.00
---	--	------	------
mPD		0.019	1.2
		nPD:	0.02
		N. weight :	1.9
		Sc. PD:	-0.074
		Sc. rank :	-16176.0

,

PA

Pos.	296	obs:	exp:
att	I	3	1.76
atc	I	0	1.31
ata	I	2	1.93
atg	M	40652	40652.00
act	T	0	2.17
acc	T	0	1.24
aca	T	0	3.03
acg	T	7	0.56
gct	A	0	0.27
gcc	A	0	0.25
gca	A	0	0.40
gcg	A	1	0.07
---	--	------	------
mPD		0.00069	0.0011
		nPD:	0.66
		N. weight :	0.00063
		Sc. PD:	0.00036
		Sc. rank :	19.8

,

PA

Pos.	297	obs:	exp:
gat	D	40623	22986.00
gac	D	42	17679.00
---	--	------	------
mPD		0.0021	0.49
		nPD:	0.
		N. weight :	0.62
		Sc. PD:	-0.031
		Sc. rank :	-15140.0

,

PA

Pos.	298	obs:	exp:
tct	S	8	1.29
tcc	S	0	1.20
tca	S	0	2.15
tcg	S	0	0.31
agt	S	0	1.40
agc	S	0	1.65
gct	A	39425	11081.00
gcc	A	1173	10158.00
gca	A	3	16446.00
gcg	A	56	2972.00
---	--	------	------
mPD		0.059	0.69
		nPD:	0.09
		N. weight :	1.3
		Sc. PD:	0.036
		Sc. rank :	27712.0

,

PA

Pos.	299	obs:	exp:
tta	L	2802	4719.00
ttg	L	37601	8407.00
tct	S	0	0.16
tcc	S	0	0.15
tca	S	1	0.27
tcg	S	0	0.04
ctt	L	0	8506.00
ctc	L	0	5963.00
cta	L	16	6244.00
ctg	L	244	6824.00
atg	M	1	1.00
agt	S	0	0.17
agc	S	0	0.21
---	--	------	------
mPD		0.14	1.2
		nPD:	0.12
		N. weight :	1.5
		Sc. PD:	0.095
		Sc. rank :	36613.0

,

PA

Pos.	300	obs:	exp:
aaa	K	40537	26735.00
aag	K	128	13930.00
---	--	------	------
mPD		0.0063	0.45
		nPD:	0.01
		N. weight :	0.44
		Sc. PD:	-0.018
		Sc. rank :	-4960.1

,

PA

Pos.	301	obs:	exp:
ttt	F	2	0.97
ttc	F	0	1.03
tta	L	5355	4718.00
ttg	L	34730	8407.00
ctt	L	0	8506.00
ctc	L	0	5963.00
cta	L	178	6244.00
ctg	L	399	6824.00
att	I	0	0.35
atc	I	0	0.26
ata	I	1	0.39
---	--	------	------
mPD		0.26	1.2
		nPD:	0.23
		N. weight :	1.3
		Sc. PD:	0.22
		Sc. rank :	37032.0

,

PA

Pos.	302	obs:	exp:
tct	S	0	6561.00
tcc	S	0	6076.00
tca	S	0	10928.00
tcg	S	0	1600.00
tgt	C	0	0.78
tgc	C	2	1.22
act	T	0	0.31
acc	T	1	0.18
aca	T	0	0.43
acg	T	0	0.08
aat	N	0	1.12
aac	N	2	0.88
agt	S	455	7105.00
agc	S	40201	8387.00
ggt	G	0	0.54
ggc	G	4	0.56
gga	G	0	1.85
ggg	G	0	1.06
---	--	------	------
mPD		0.023	1.6
		nPD:	0.01
		N. weight :	1.7
		Sc. PD:	-0.069
		Sc. rank :	-18888.0

,

PA

Pos.	303	obs:	exp:
att	I	40589	14272.00
atc	I	56	10683.00
ata	I	10	15701.00
gtt	V	10	2.50
gtc	V	0	2.04
gta	V	0	2.17
gtg	V	0	3.29
---	--	------	------
mPD		0.0037	0.66
		nPD:	0.01
		N. weight :	1.1
		Sc. PD:	-0.056
		Sc. rank :	-25437.0

,

PA			
Pos.	304	obs:	exp:
gat	D	0	0.57
gac	D	1	0.43
gaa	E	40514	23973.00
gag	E	149	16690.00
ggt	G	0	0.13
ggc	G	0	0.14
gga	G	1	0.46
ggg	G	0	0.26
---	--	------	------
mPD		0.0074	0.48
		nPD:	0.02
		N. weight:	0.56
		Sc. PD:	-0.022
		Sc. rank:	-5703.1

,

PA			
Pos.	305	obs:	exp:
aat	N	1	10.07
aac	N	17	7.93
gtt	V	0	0.25
gtc	V	1	0.20
gta	V	0	0.22
gtg	V	0	0.33
gat	D	1694	22974.00
gac	D	38949	17669.00
gaa	E	3	1.77
gag	E	0	1.23
---	--	------	------
mPD		0.081	0.49
		nPD:	0.16
		N. weight:	0.72
		Sc. PD:	0.076
		Sc. rank:	19415.0

,

PA			
Pos.	306	obs:	exp:
cct	P	5	13551.00
ccc	P	2	7624.00
cca	P	40375	13739.00
ccg	P	283	5751.00
---	--	------	------
mPD		0.014	0.72
		nPD:	0.02
		N. weight:	1.2
		Sc. PD:	-0.041
		Sc. rank:	-5406.2

,

PA			
Pos.	307	obs:	exp:
tct	S	0	6556.00
tcc	S	0	6071.00
tca	S	0	10919.00
tcg	S	0	1599.00
cgt	R	0	0.04
cgc	R	0	0.07
cga	R	0	0.18
cgg	R	0	0.17
att	I	2	0.70
atc	I	0	0.53
ata	I	0	0.77
act	T	5	1.55
acc	T	0	0.88
aca	T	0	2.16
acg	T	0	0.40
aat	N	28	15.67
aac	N	0	12.33
agt	S	40417	7099.00
agc	S	208	8381.00
aga	R	2	0.98
agg	R	0	0.55
ggt	G	3	0.40
ggc	G	0	0.42
gga	G	0	1.38
ggg	G	0	0.79
---	--	------	------
mPD		0.012	1.6
		nPD:	0.01
		N. weight:	1.9
		Sc. PD:	-0.089
		Sc. rank:	-36104.0

,

PA			
Pos.	308	obs:	exp:
tat	Y	0	11.07
tac	Y	21	9.93
cat	H	549	22925.00
cac	H	40095	17719.00
---	--	------	------
mPD		0.028	0.49
		nPD:	0.06
		N. weight:	0.84
		Sc. PD:	0.000098
		Sc. rank:	13627.0

,

PA			
Pos.	309	obs:	exp:
aaa	K	2	1.31
aag	K	0	0.69
gaa	E	40557	23973.00
gag	E	106	16690.00
---	--	------	------
mPD		0.0053	0.48
		nPD:	0.01
		N. weight:	0.57
		Sc. PD:	-0.025
		Sc. rank:	-8801.3

,

PA			
Pos.	310	obs:	exp:
cgt	R	0	0.04
cgc	R	0	0.07
cga	R	0	0.18
cgg	R	0	0.17
aga	R	2	0.98
agg	R	0	0.55
ggt	G	6	5474.00
ggc	G	11	5675.00
gga	G	40282	18771.00
ggg	G	364	10743.00
---	--	------	------
mPD		0.019	0.68
		nPD:	0.03
		N. weight:	0.8
		Sc. PD:	-0.022
		Sc. rank:	2504.4

,

PA			
Pos.	311	obs:	exp:
gaa	E	40395	23974.00
gag	E	269	16690.00
ggt	G	0	0.13
ggc	G	0	0.14
gga	G	1	0.46
ggg	G	0	0.26
---	--	------	------
mPD		0.013	0.48
		nPD:	0.03
		N. weight:	0.54
		Sc. PD:	-0.015
		Sc. rank:	1584.5

,

PA			
Pos.	312	obs:	exp:
ggt	G	5	5475.00
ggc	G	1	5676.00
gga	G	740	18772.00
ggg	G	39919	10743.00
---	--	------	------
mPD		0.036	0.68
		nPD:	0.05
		N. weight:	1.4
		Sc. PD:	-0.004
		Sc. rank:	20685.0

,

PA			
Pos.	313	obs:	exp:
tct	S	0	0.16
tcc	S	0	0.15
tca	S	0	0.27
tcg	S	0	0.04
att	I	263	14271.00
atc	I	39884	10682.00
ata	I	506	15700.00
act	T	0	0.31
acc	T	1	0.18
aca	T	0	0.43
acg	T	0	0.08
agt	S	1	0.17
agc	S	0	0.21
gtt	V	0	2.50
gtc	V	10	2.04
gta	V	0	2.17
gtg	V	0	3.29
---	--	------	------
mPD		0.038	0.66
		nPD:	0.06
		N. weight:	1.4
		Sc. PD:	0.0019
		Sc. rank:	23152.0

,

PA			
Pos.	314	obs:	exp:
cct	P	38	13551.00
ccc	P	71	7624.00
cca	P	40532	13739.00
ccg	P	24	5751.00
---	--	------	------
mPD		0.0065	0.72
		nPD:	0.01
		N. weight:	1.2
		Sc. PD:	-0.054
		Sc. rank:	-20240.0

,

PA			
Pos.	315	obs:	exp:
tta	L	39591	4719.00
ttg	L	365	8407.00
ctt	L	0	8507.00
ctc	L	0	5963.00
cta	L	709	6244.00
ctg	L	0	6825.00
---	--	------	------
mPD		0.052	1.2
		nPD:	0.04
		N. weight:	2.2
		Sc. PD:	-0.024
		Sc. rank:	28123.0

,

PA

Pos.	316	obs:	exp:
tat	Y	40596	21411.00
tac	Y	23	19208.00
tgt	C	25	9.77
tgc	C	0	15.23
cat	H	20	11.28
cac	H	0	8.72
aat	N	1	0.56
aac	N	0	0.44
---	--	------	------
mPD		0.0034	0.50
		nPD:	0.01
		N. weight:	0.7
		Sc. PD:	-0.034
		Sc. rank:	-14513.0

PA

Pos.	317	obs:	exp:
gat	D	40609	22986.00
gac	D	56	17679.00
---	--	------	------
mPD		0.0028	0.49
		nPD:	0.01
		N. weight:	0.62
		Sc. PD:	-0.03
		Sc. rank:	-13840.0

PA

Pos.	318	obs:	exp:
tct	S	0	0.81
tcc	S	0	0.75
tca	S	1	1.34
tcg	S	4	0.20
agt	S	0	0.87
agc	S	0	1.03
gct	A	134	11082.00
gcc	A	3	10159.00
gca	A	25568	16447.00
gcg	A	14955	2972.00
---	--	------	------
mPD		0.47	0.69
		nPD:	0.68
		N. weight:	0.95
		Sc. PD:	0.57
		Sc. rank:	30015.0

PA

Pos.	319	obs:	exp:
tta	L	0	0.46
ttg	L	0	0.83
tct	S	0	0.16
tcc	S	0	0.15
tca	S	0	0.27
tcg	S	0	0.04
ctt	L	0	0.84
ctc	L	4	0.59
cta	L	0	0.61
ctg	L	0	0.67
att	I	40	14122.00
atc	I	40164	10571.00
ata	I	26	15537.00
act	T	0	2.17
acc	T	7	1.24
aca	T	0	3.03
acg	T	0	0.56
agt	S	0	0.17
agc	S	1	0.21
gtt	V	0	106.00
gtc	V	423	86.19
gta	V	0	91.68
gtg	V	0	139.20
---	--	------	------
mPD		0.024	0.68
		nPD:	0.04
		N. weight:	1.5
		Sc. PD:	-0.029
		Sc. rank:	13074.0

PA

Pos.	320	obs:	exp:
cgt	R	0	0.44
cgc	R	0	0.79
cga	R	0	2.01
cgg	R	0	1.89
act	T	0	0.31
acc	T	0	0.18
aca	T	0	0.43
acg	T	1	0.08
aat	N	50	27.97
aac	N	0	22.03
aaa	K	1388	26687.00
aag	K	39204	13905.00
aga	R	1	10.79
agg	R	21	6.09
---	--	------	------
mPD		0.069	0.45
		nPD:	0.15
		N. weight:	1.
		Sc. PD:	0.094
		Sc. rank:	26512.0

PA

Pos.	321	obs:	exp:
tgt	C	584	15892.00
tgc	C	40081	24773.00
---	--	------	------
mPD		0.028	0.48
		nPD:	0.06
		N. weight:	0.47
		Sc. PD:	0.0016
		Sc. rank:	8217.9

PA

Pos.	322	obs:	exp:
tta	L	25	4.64
ttg	L	0	8.27
ctt	L	0	8.37
ctc	L	0	5.87
cta	L	14	6.14
ctg	L	1	6.71
att	I	1	13931.00
atc	I	4	10428.00
ata	I	39681	15327.00
atg	M	921	921.00
act	T	0	2.79
acc	T	0	1.59
aca	T	9	3.89
acg	T	0	0.72
gtt	V	0	2.25
gtc	V	0	1.83
gta	V	9	1.95
gtg	V	0	2.96
---	--	------	------
mPD		0.047	0.68
		nPD:	0.07
		N. weight:	1.
		Sc. PD:	0.014
		Sc. rank:	20251.0

PA

Pos.	323	obs:	exp:
caa	Q	1	0.61
cag	Q	0	0.39
cgt	R	0	5.93
cgc	R	0	10.76
cga	R	0	27.28
cgg	R	0	25.67
act	T	0	0.62
acc	T	0	0.35
aca	T	2	0.87
acg	T	0	0.16
aat	N	1	0.56
aac	N	0	0.44
aaa	K	40081	26535.00
aag	K	280	13826.00
aga	R	285	146.70
agg	R	14	82.70
gaa	E	1	0.59
gag	E	0	0.41
---	--	------	------
mPD		0.029	0.47
		nPD:	0.06
		N. weight:	0.42
		Sc. PD:	0.0026
		Sc. rank:	7551.2

PA

Pos.	324	obs:	exp:
cct	P	0	0.67
ccc	P	0	0.37
cca	P	2	0.68
ccg	P	0	0.28
act	T	26	12627.00
acc	T	2	7189.00
aca	T	40604	17596.00
acg	T	27	3247.00
aaa	K	2	1.31
aag	K	0	0.69
gct	A	0	0.55
gcc	A	0	0.50
gca	A	2	0.81
gcg	A	0	0.15
---	--	------	------
mPD		0.0030	0.68
		nPD:	0.
		N. weight:	0.92
		Sc. PD:	-0.046
		Sc. rank:	-21835.0

PA

Pos.	325	obs:	exp:
ttt	F	219	19780.00
ttc	F	40446	20885.00
---	--	------	------
mPD		0.011	0.50
		nPD:	0.02
		N. weight:	0.7
		Sc. PD:	-0.024
		Sc. rank:	-2066.4

PA

Pos.	326	obs:	exp:
ttt	F	40595	19773.00
ttc	F	54	20876.00
tta	L	0	0.58
ttg	L	0	1.03
tct	S	4	0.65
tcc	S	0	0.60
tca	S	0	1.08
tcg	S	0	0.16
tat	Y	3	1.58
tac	Y	0	1.42
tgt	C	1	0.39
tgc	C	0	0.61
ctt	L	5	1.05
ctc	L	0	0.73
cta	L	0	0.77
ctg	L	0	0.84
agt	S	0	0.70
agc	S	0	0.83
gtt	V	3	0.75
gtc	V	0	0.61
gta	V	0	0.65
gtg	V	0	0.99
---	--	------	------
mPD		0.0034	0.50
		nPD:	0.01
		N. weight:	0.79
		Sc. PD:	-0.038
		Sc. rank:	-16041.0

PA

Pos.	327	obs:	exp:
gat	D	0	0.57
gac	D	1	0.43
gaa	E	1	0.59
gag	E	0	0.41
ggt	G	7	5474.00
ggc	G	215	5675.00
gga	G	40205	18771.00
ggg	G	236	10743.00
---	--	------	------
mPD		0.022	0.68
		nPD:	0.03
		N. weight:	0.79
		Sc. PD:	-0.018
		Sc. rank:	5638.5

PA			
Pos.	328	obs:	exp:
tgg	W	40665	40665.00
---	--	------	------
mPD		0	0
		nPD:	1.
		N. weight:	0.
		Sc. PD:	0
		Sc. rank:	0

PA			
Pos.	329	obs:	exp:
cgt	R	0	0.06
cgc	R	0	0.11
cga	R	0	0.27
cgg	R	0	0.26
aaa	K	40549	26733.00
aag	K	113	13929.00
aga	R	3	1.47
agg	R	0	0.83
---	--	------	------
mPD		0.0057	0.45
		nPD:	0.01
		N. weight:	0.45
		Sc. PD:	-0.019
		Sc. rank:	-5895.1

PA			
Pos.	330	obs:	exp:
aaa	K	2	1.31
aag	K	0	0.69
gat	D	1	2.26
gac	D	3	1.74
gaa	E	39482	23971.00
gag	E	1177	16688.00
---	--	------	------
mPD		0.057	0.48
		nPD:	0.12
		N. weight:	0.45
		Sc. PD:	0.027
		Sc. rank:	11103.0

PA			
Pos.	331	obs:	exp:
cct	P	40231	13550.00
ccc	P	408	7623.00
cca	P	16	13738.00
ccg	P	8	5751.00
act	T	1	0.31
acc	T	0	0.18
aca	T	0	0.43
acg	T	0	0.08
gct	A	1	0.27
gcc	A	0	0.25
gca	A	0	0.40
gcg	A	0	0.07
---	--	------	------
mPD		0.021	0.72
		nPD:	0.03
		N. weight:	1.2
		Sc. PD:	-0.03
		Sc. rank:	5540.9

PA			
Pos.	332	obs:	exp:
ttt	F	6	2.92
ttc	F	0	3.08
tct	S	6	1.13
tcc	S	0	1.05
tca	S	0	1.88
tcg	S	0	0.28
tat	Y	36238	19209.00
tac	Y	204	17233.00
tgt	C	3834	1498.00
tgc	C	0	2336.00
cat	H	157	91.37
cac	H	5	70.63
aat	N	58	109.70
aac	N	138	86.34
aaa	K	17	11.18
aag	K	0	5.82
agt	S	1	1.22
agc	S	0	1.44
gat	D	1	0.57
gac	D	0	0.43
---	--	------	------
mPD		0.21	0.69
		nPD:	0.3
		N. weight:	0.7
		Sc. PD:	0.17
		Sc. rank:	20505.0

PA			
Pos.	333	obs:	exp:
tta	L	0	0.46
ttg	L	0	0.83
ctt	L	0	0.84
ctc	L	0	0.59
cta	L	4	0.61
ctg	L	0	0.67
att	I	31	14244.00
atc	I	58	10662.00
ata	I	40488	15671.00
atg	M	1	1.00
act	T	0	0.31
acc	T	0	0.18
aca	T	1	0.43
acg	T	0	0.08
gtt	V	6	20.54
gtc	V	1	16.71
gta	V	74	17.77
gtg	V	1	26.98
---	--	------	------
mPD		0.0091	0.66
		nPD:	0.01
		N. weight:	1.
		Sc. PD:	-0.043
		Sc. rank:	-11848.0

PA			
Pos.	334	obs:	exp:
att	I	0	80.04
atc	I	228	59.91
ata	I	0	88.05
gtt	V	58	10125.00
gtc	V	40283	8236.00
gta	V	80	8761.00
gtg	V	0	13299.00
gct	A	0	4.09
gcc	A	15	3.75
gca	A	0	6.07
gcg	A	0	1.10
gat	D	0	0.57
gac	D	1	0.43
---	--	------	------
mPD		0.019	0.75
		nPD:	0.02
		N. weight:	1.7
		Sc. PD:	-0.052
		Sc. rank:	1721.1

PA			
Pos.	335	obs:	exp:
caa	Q	1	0.61
cag	Q	0	0.39
aaa	K	40633	26734.00
aag	K	31	13930.00
---	--	------	------
mPD		0.0016	0.45
		nPD:	0.
		N. weight:	0.46
		Sc. PD:	-0.023
		Sc. rank:	-11673.0

PA			
Pos.	336	obs:	exp:
tta	L	0	0.12
ttg	L	0	0.21
tct	S	0	1.78
tcc	S	0	1.64
tca	S	11	2.96
tcg	S	0	0.43
ctt	L	0	0.21
ctc	L	0	0.15
cta	L	1	0.15
ctg	L	0	0.17
cct	P	175	13543.00
ccc	P	12	7619.00
cca	P	40426	13731.00
ccg	P	29	5748.00
caa	Q	4	2.45
cag	Q	0	1.55
act	T	0	0.62
acc	T	0	0.35
aca	T	2	0.87
acg	T	0	0.16
agt	S	0	1.92
agc	S	0	2.27
gct	A	0	1.36
gcc	A	0	1.25
gca	A	5	2.02
gcg	A	0	0.37
---	--	------	------
mPD		0.012	0.72
		nPD:	0.02
		N. weight:	1.2
		Sc. PD:	-0.045
		Sc. rank:	-10157.0

PA			
Pos.	337	obs:	exp:
cat	H	573	22937.00
cac	H	40092	17728.00
---	--	------	------
mPD		0.028	0.49
		nPD:	0.06
		N. weight:	0.83
		Sc. PD:	0.00038
		Sc. rank:	13640.0

PA			
Pos.	338	obs:	exp:
aaa	K	20	13.15
aag	K	0	6.85
gct	A	0	0.27
gcc	A	0	0.25
gca	A	1	0.40
gcg	A	0	0.07
gat	D	3	3.39
gac	D	3	2.61
gaa	E	40376	23946.00
gag	E	240	16670.00
ggt	G	0	2.96
ggc	G	0	3.07
gga	G	22	10.16
ggg	G	0	5.81
---	--	------	------
mPD		0.014	0.49
		nPD:	0.03
		N. weight:	0.55
		Sc. PD:	-0.014
		Sc. rank:	2429.1

PA			
Pos.	339	obs:	exp:
cgt	R	0	1.76
cgc	R	0	3.20
cga	R	0	8.12
cgg	R	0	7.64
atg	M	1	1.00
aat	N	1	0.56
aac	N	0	0.44
aaa	K	433	26675.00
aag	K	40141	13899.00
aga	R	7	43.65
agg	R	82	24.62
---	--	------	------
mPD		0.026	0.46
		nPD:	0.06
		N. weight:	1.1
		Sc. PD:	0.00076
		Sc. rank:	18471.0

PA

Pos.	340	obs :	exp :
ggt	G	5	5475.00
ggc	G	206	5676.00
gga	G	40298	18772.00
ggg	G	156	10743.00
---	--	------	------
mPD		0.018	0.68
		nPD :	0.03
		N. weight :	0.8
		Sc. PD :	-0.023
		Sc. rank :	1599.0

,

PA

Pos.	341	obs :	exp :
att	I	6	14263.00
atc	I	0	10676.00
ata	I	40625	15692.00
atg	M	1	1.00
act	T	0	1.55
acc	T	0	0.88
aca	T	5	2.16
acg	T	0	0.40
gtt	V	0	7.01
gtc	V	0	5.71
gta	V	28	6.07
gtg	V	0	9.21
---	--	------	------
mPD		0.0020	0.66
		nPD :	0.
		N. weight :	1.1
		Sc. PD :	-0.054
		Sc. rank :	-28143.0

,

PA

Pos.	342	obs :	exp :
tct	S	0	0.65
tcc	S	0	0.60
tca	S	0	1.08
tcg	S	0	0.16
aat	N	40369	22749.00
aac	N	292	17912.00
agt	S	4	0.70
agc	S	0	0.83
---	--	------	------
mPD		0.014	0.49
		nPD :	0.03
		N. weight :	0.6
		Sc. PD :	-0.015
		Sc. rank :	2807.6

,

PA

Pos.	343	obs :	exp :
tta	L	4	0.81
ttg	L	0	1.45
tct	S	2	6482.00
tcc	S	5	6003.00
tca	S	40124	10797.00
tcg	S	41	1581.00
ctt	L	0	1.46
ctc	L	3	1.03
cta	L	0	1.07
ctg	L	0	1.17
cct	P	1	98.31
ccc	P	188	55.30
cca	P	106	99.67
ccg	P	0	41.72
act	T	0	56.83
acc	T	0	32.35
aca	T	183	79.20
acg	T	0	14.62
agt	S	0	7020.00
agc	S	0	8287.00
gct	A	0	2.18
gcc	A	0	2.00
gca	A	8	3.24
gcg	A	0	0.58
---	--	------	------
mPD		0.036	1.6
		nPD :	0.02
		N. weight :	1.4
		Sc. PD :	-0.048
		Sc. rank :	-3100.2

,

PA

Pos.	344	obs :	exp :
cat	H	1	0.56
cac	H	0	0.44
att	I	1	0.35
atc	I	0	0.26
ata	I	0	0.39
aat	N	40543	22750.00
aac	N	120	17913.00
---	--	------	------
mPD		0.0060	0.49
		nPD :	0.01
		N. weight :	0.62
		Sc. PD :	-0.026
		Sc. rank :	-8456.7

,

PA

Pos.	345	obs :	exp :
ttt	F	0	0.49
ttc	F	1	0.51
tat	Y	1590	21434.00
tac	Y	39074	19230.00
---	--	------	------
mPD		0.075	0.50
		nPD :	0.15
		N. weight :	0.64
		Sc. PD :	0.059
		Sc. rank :	17000.0

,

PA

Pos.	346	obs :	exp :
tta	L	21	4718.00
ttg	L	75	8406.00
ctt	L	16	8505.00
ctc	L	27	5962.00
cta	L	11437	6243.00
ctg	L	29082	6824.00
att	I	0	1.40
atc	I	0	1.05
ata	I	4	1.54
atg	M	2	2.00
gtt	V	0	0.25
gtc	V	1	0.20
gta	V	0	0.22
gtg	V	0	0.33
---	--	------	------
mPD		0.41	1.2
		nPD :	0.35
		N. weight :	1.3
		Sc. PD :	0.38
		Sc. rank :	39694.0

,

PA

Pos.	347	obs :	exp :
tta	L	0	4714.00
ttg	L	55	8399.00
ctt	L	22	8499.00
ctc	L	0	5957.00
cta	L	174	6238.00
ctg	L	40375	6818.00
caa	Q	0	3.67
cag	Q	6	2.33
atg	M	32	32.00
gtt	V	0	0.25
gtc	V	0	0.20
gta	V	0	0.22
gtg	V	1	0.33
---	--	------	------
mPD		0.014	1.2
		nPD :	0.01
		N. weight :	1.9
		Sc. PD :	-0.082
		Sc. rank :	-25901.0

,

PA

Pos.	348	obs :	exp :
tta	L	2	0.23
ttg	L	0	0.41
tct	S	5	6528.00
tcc	S	3	6045.00
tca	S	40421	10873.00
tcg	S	24	1592.00
ctt	L	0	0.42
ctc	L	0	0.29
cta	L	0	0.31
ctg	L	0	0.34
act	T	1	1.24
acc	T	0	0.71
aca	T	3	1.73
acg	T	0	0.32
agt	S	0	7069.00
agc	S	0	8345.00
gct	A	204	56.15
gcc	A	1	51.47
gca	A	1	83.33
gcg	A	0	15.06
---	--	------	------
mPD		0.022	1.6
		nPD :	0.01
		N. weight :	1.4
		Sc. PD :	-0.06
		Sc. rank :	-17643.0

,

PA

Pos.	349	obs :	exp :
tgg	W	40664	40664.00
ggt	G	0	0.13
ggc	G	0	0.14
gga	G	0	0.46
ggg	G	1	0.26
---	--	------	------
mPD		0.000049	0.000085
		nPD :	0.58
		N. weight :	0.000036
		Sc. PD :	0.000018
		Sc. rank :	1.1

,

PA

Pos.	350	obs :	exp :
caa	Q	0	0.61
cag	Q	1	0.39
cgt	R	0	0.71
cgc	R	0	1.30
cga	R	0	3.28
cgg	R	0	3.09
aat	N	0	0.56
aac	N	1	0.44
aaa	K	797	26708.00
aag	K	39827	13916.00
aga	R	0	17.66
agg	R	36	9.96
gaa	E	0	1.77
gag	E	3	1.23
---	--	------	------
mPD		0.040	0.45
		nPD :	0.09
		N. weight :	1.1
		Sc. PD :	0.034
		Sc. rank :	23866.0

,

PA

Pos.	351	obs :	exp :
caa	Q	40563	24858.00
cag	Q	102	15807.00
---	--	------	------
mPD		0.0050	0.48
		nPD :	0.01
		N. weight :	0.53
		Sc. PD :	-0.023
		Sc. rank :	-8411.9

,

PA			
Pos.	352	obs:	exp:
tta	L	26	3.02
ttg	L	0	5.38
ctt	L	0	5.44
ctc	L	0	3.81
cta	L	0	3.99
ctg	L	0	4.36
att	I	0	14.04
atc	I	0	10.51
ata	I	40	15.45
gtt	V	7	10169.00
gtc	V	6	8272.00
gta	V	40291	8799.00
gtg	V	293	13357.00
gct	A	0	0.55
gcc	A	0	0.50
gca	A	2	0.81
gcg	A	0	0.15
---	--	------	------
mPD		0.018	0.74
		nPD:	0.02
		N. weight:	1.6
		Sc. PD:	-0.05
		Sc. rank:	837.8

,

PA			
Pos.	353	obs:	exp:
ttt	F	1	0.49
ttc	F	0	0.51
tta	L	7	4716.00
ttg	L	613	8403.00
ctt	L	21	8502.00
ctc	L	2	5960.00
cta	L	272	6241.00
ctg	L	39728	6821.00
caa	Q	0	3.06
cag	Q	5	1.94
atg	M	16	16.00
---	--	------	------
mPD		0.046	1.2
		nPD:	0.04
		N. weight:	1.8
		Sc. PD:	-0.029
		Sc. rank:	20156.0

,

PA			
Pos.	354	obs:	exp:
tta	L	17	1.97
ttg	L	0	3.51
tct	S	140	6480.00
tcc	S	2	6001.00
tca	S	39784	10794.00
tcg	S	232	1581.00
ctt	L	0	3.56
ctc	L	0	2.49
cta	L	0	2.61
ctg	L	0	2.85
act	T	0	1.24
acc	T	0	0.71
aca	T	4	1.73
acg	T	0	0.32
agt	S	0	7018.00
agc	S	0	8284.00
gct	A	0	132.50
gcc	A	0	121.40
gca	A	483	196.60
gcg	A	3	35.53
---	--	------	------
mPD		0.043	1.6
		nPD:	0.03
		N. weight:	1.4
		Sc. PD:	-0.04
		Sc. rank:	2618.1

,

PA			
Pos.	355	obs:	exp:
gaa	E	40367	23975.00
gag	E	298	16690.00
---	--	------	------
mPD		0.015	0.48
		nPD:	0.03
		N. weight:	0.54
		Sc. PD:	-0.014
		Sc. rank:	2741.4

,

PA			
Pos.	356	obs:	exp:
ttt	F	1	0.97
ttc	F	1	1.03
tta	L	142	4718.00
ttg	L	40158	8406.00
ctt	L	0	8506.00
ctc	L	176	5963.00
cta	L	76	6244.00
ctg	L	109	6824.00
att	I	0	0.35
atc	I	1	0.26
ata	I	0	0.39
atg	M	1	1.00
---	--	------	------
mPD		0.037	1.2
		nPD:	0.03
		N. weight:	1.7
		Sc. PD:	-0.039
		Sc. rank:	10778.0

,

PA			
Pos.	357	obs:	exp:
tta	L	0	1.04
ttg	L	0	1.86
ctt	L	0	1.88
ctc	L	0	1.32
cta	L	0	1.38
ctg	L	9	1.51
cat	H	22	12.41
cac	H	0	9.59
caa	Q	2349	24833.00
cag	Q	38276	15792.00
cgt	R	0	0.10
cgc	R	0	0.18
cga	R	0	0.46
cgg	R	5	0.43
aaa	K	0	2.63
aag	K	4	1.37
aga	R	0	2.45
agg	R	0	1.38
---	--	------	------
mPD		0.11	0.48
		nPD:	0.23
		N. weight:	0.77
		Sc. PD:	0.13
		Sc. rank:	21873.0

,

PA			
Pos.	358	obs:	exp:
tat	Y	0	1.58
tac	Y	3	1.42
aat	N	2	12.31
aac	N	20	9.69
gct	A	0	0.27
gcc	A	1	0.25
gca	A	0	0.40
gcg	A	0	0.07
gat	D	1403	22947.00
gac	D	39192	17648.00
gaa	E	38	22.40
gag	E	0	15.60
ggt	G	1	0.81
ggc	G	5	0.84
gga	G	0	2.77
ggg	G	0	1.59
---	--	------	------
mPD		0.070	0.49
		nPD:	0.14
		N. weight:	0.75
		Sc. PD:	0.062
		Sc. rank:	19423.0

,

PA			
Pos.	359	obs:	exp:
ttt	F	12	5.84
ttc	F	0	6.16
tta	L	0	108.30
ttg	L	0	192.90
tct	S	0	0.16
tcc	S	0	0.15
tca	S	0	0.27
tcg	S	0	0.04
ctt	L	933	195.20
ctc	L	0	136.80
cta	L	0	143.30
ctg	L	0	156.60
att	I	39277	13801.00
atc	I	23	10330.00
ata	I	14	15183.00
atg	M	5	5.00
act	T	7	2.17
acc	T	0	1.24
aca	T	0	3.03
acg	T	0	0.56
agt	S	1	0.17
agc	S	0	0.21
gtt	V	391	98.19
gtc	V	0	79.87
gta	V	1	84.96
gtg	V	0	129.00
gct	A	1	0.27
gcc	A	0	0.25
gca	A	0	0.40
gcg	A	0	0.07
---	--	------	------
mPD		0.067	0.73
		nPD:	0.09
		N. weight:	1.2
		Sc. PD:	0.04
		Sc. rank:	26259.0

,

PA			
Pos.	360	obs:	exp:
aaa	K	5	3.29
aag	K	0	1.71
gct	A	0	0.27
gcc	A	0	0.25
gca	A	0	0.40
gcg	A	1	0.07
gat	D	0	2.83
gac	D	5	2.17
gaa	E	39018	23962.00
gag	E	1625	16681.00
ggt	G	0	1.48
ggc	G	0	1.54
gga	G	10	5.08
ggg	G	1	2.91
---	--	------	------
mPD		0.078	0.49
		nPD:	0.16
		N. weight:	0.42
		Sc. PD:	0.042
		Sc. rank:	11201.0

,

PA			
Pos.	361	obs:	exp:
tct	S	0	4.68
tcc	S	0	4.33
tca	S	0	7.79
tcg	S	0	1.14
cat	H	15	8.46
cac	H	0	6.54
att	I	6	2.11
atc	I	0	1.58
ata	I	0	2.32
act	T	162	61.80
acc	T	37	35.18
aca	T	0	86.12
acg	T	0	15.89
aat	N	40349	22603.00
aac	N	52	17798.00
aaa	K	5	7.89
aag	K	7	4.11
agt	S	29	5.07
agc	S	0	5.98
gat	D	3	1.70
gac	D	0	1.30
---	--	------	------
mPD		0.017	0.51
		nPD:	0.03
		N. weight:	0.63
		Sc. PD:	-0.014
		Sc. rank:	5109.5

,

PA			
Pos.	362	obs:	exp:
aaa	K	0	1.97
aag	K	3	1.03
gtt	V	0	0.50
gtc	V	0	0.41
gta	V	0	0.43
gtg	V	2	0.66
gat	D	28	15.83
gac	D	0	12.17
gaa	E	395	23937.00
gag	E	40206	16664.00
ggt	G	0	4.17
ggc	G	0	4.33
gga	G	0	14.31
ggg	G	31	8.19
---	--	------	------
mPD		0.022	0.49
		nPD:	0.05
		N. weight:	0.92
		Sc. PD:	-0.009
		Sc. rank:	11893.0

,

PA			
Pos.	363	obs:	exp:
aaa	K	0	2.63
aag	K	4	1.37
gtt	V	0	0.50
gtc	V	0	0.41
gta	V	0	0.43
gtg	V	2	0.66
gat	D	22	13.00
gac	D	1	10.00
gaa	E	87	23951.00
gag	E	40538	16674.00
ggt	G	0	1.48
ggc	G	0	1.54
gga	G	0	5.08
ggg	G	11	2.91
---	--	------	------
mPD		0.0062	0.49
		nPD:	0.01
		N. weight:	0.97
		Sc. PD:	-0.041
		Sc. rank:	-12736.0

,

PA

Pos.	364	obs:	exp:
caa	Q	0	4.28
cag	Q	7	2.72
cgt	R	0	0.65
cgc	R	0	1.19
cga	R	0	3.01
cgg	R	0	2.83
atg	M	1	1.00
act	T	0	0.62
acc	T	0	0.35
aca	T	0	0.87
acg	T	2	0.16
aat	N	10	5.59
aac	N	0	4.41
aaa	K	254	26696.00
aag	K	40352	13910.00
aga	R	0	16.19
agg	R	33	9.13
gaa	E	0	3.54
gag	E	6	2.46
---	--	------	------
mPD		0.015	0.45
		nPD:	0.03
		N. weight:	1.1
		Sc. PD:	-0.025
		Sc. rank:	9020.4

,

PA

Pos.	365	obs:	exp:
ttt	F	1	0.49
ttc	F	0	0.51
tct	S	0	0.16
tcc	S	0	0.15
tca	S	0	0.27
tcg	S	0	0.04
att	I	38859	14072.00
atc	I	1223	10533.00
ata	I	4	15481.00
atg	M	1	1.00
act	T	50	17.08
acc	T	5	9.72
aca	T	0	23.80
acg	T	0	4.39
aat	N	1	0.56
aac	N	0	0.44
agt	S	1	0.17
agc	S	0	0.21
gtt	V	518	130.20
gtc	V	2	106.00
gta	V	0	112.70
gtg	V	0	171.10
---	--	------	------
mPD		0.087	0.69
		nPD:	0.13
		N. weight:	1.
		Sc. PD:	0.07
		Sc. rank:	25627.0

,

PA

Pos.	366	obs:	exp:
tta	L	0	0.12
ttg	L	0	0.21
tct	S	0	1.29
tcc	S	0	1.20
tca	S	8	2.15
tcg	S	0	0.31
ctt	L	0	0.21
ctc	L	0	0.15
cta	L	1	0.15
ctg	L	0	0.17
cct	P	1	13548.00
ccc	P	4	7622.00
cca	P	40569	13735.00
ccg	P	81	5750.00
caa	Q	1	0.61
cag	Q	0	0.39
agt	S	0	1.40
agc	S	0	1.65
---	--	------	------
mPD		0.0047	0.72
		nPD:	0.01
		N. weight:	1.2
		Sc. PD:	-0.057
		Sc. rank:	-25021.0

,

PA

Pos.	367	obs:	exp:
cgt	R	0	801.00
cgc	R	0	1454.00
cga	R	8	3686.00
cgg	R	0	3469.00
aaa	K	82	175.50
aag	K	185	91.46
aga	R	39219	19815.00
agg	R	1171	11174.00
---	--	------	------
mPD		0.078	0.90
		nPD:	0.09
		N. weight:	0.66
		Sc. PD:	0.02
		Sc. rank:	14494.0

,

PA

Pos.	368	obs:	exp:
tct	S	2	0.32
tcc	S	0	0.30
tca	S	0	0.54
tcg	S	0	0.08
att	I	14	5.27
atc	I	0	3.94
ata	I	1	5.79
act	T	36632	12611.00
acc	T	101	7179.00
aca	T	3871	17574.00
acg	T	3	3243.00
aat	N	15	8.39
aac	N	0	6.61
agt	S	0	0.35
agc	S	0	0.41
gct	A	25	7.09
gcc	A	0	6.50
gca	A	1	10.52
gcg	A	0	1.90
---	--	------	------
mPD		0.18	0.68
		nPD:	0.26
		N. weight:	0.89
		Sc. PD:	0.18
		Sc. rank:	25574.0

,

PA

Pos.	369	obs:	exp:
cgt	R	0	0.14
cgc	R	0	0.25
cga	R	0	0.64
cgg	R	0	0.60
att	I	0	0.35
atc	I	0	0.26
ata	I	1	0.39
aat	N	1	1.12
aac	N	1	0.88
aaa	K	40396	26728.00
aag	K	259	13927.00
aga	R	7	3.43
agg	R	0	1.94
---	--	------	------
mPD		0.013	0.45
		nPD:	0.03
		N. weight:	0.43
		Sc. PD:	-0.011
		Sc. rank:	1907.9

,

PA

Pos.	370	obs:	exp:
tct	S	0	0.32
tcc	S	0	0.30
tca	S	0	0.54
tcg	S	0	0.08
cat	H	0	0.56
cac	H	1	0.44
act	T	0	15.84
acc	T	51	9.02
aca	T	0	22.07
acg	T	0	4.07
aat	N	93	22720.00
aac	N	40516	17889.00
aaa	K	1	0.66
aag	K	0	0.34
agt	S	0	0.35
agc	S	2	0.41
gat	D	0	0.57
gac	D	1	0.43
---	--	------	------
mPD		0.0073	0.50
		nPD:	0.01
		N. weight:	0.89
		Sc. PD:	-0.036
		Sc. rank:	-9490.9

,

PA

Pos.	371	obs:	exp:
tta	L	0	3.83
ttg	L	24	6.82
ctt	L	0	6.90
ctc	L	0	4.84
cta	L	0	5.07
ctg	L	9	5.54
att	I	1	1.76
atc	I	0	1.31
ata	I	4	1.93
atg	M	40627	40627.00
---	--	------	------
mPD		0.0019	0.0029
		nPD:	0.65
		N. weight:	0.0011
		Sc. PD:	0.00062
		Sc. rank:	34.1

,

PA

Pos.	372	obs:	exp:
cgt	R	0	0.73
cgc	R	0	1.33
cga	R	0	3.38
cgg	R	0	3.18
act	T	0	1.24
acc	T	0	0.71
aca	T	0	1.73
acg	T	4	0.32
aat	N	3	1.68
aac	N	0	1.32
aaa	K	180	26704.00
aag	K	40439	13915.00
aga	R	0	18.15
agg	R	37	10.23
gaa	E	0	1.18
gag	E	2	0.82
---	--	------	------
mPD		0.011	0.45
		nPD:	0.02
		N. weight:	1.2
		Sc. PD:	-0.035
		Sc. rank:	72.4

,

PA

Pos.	373	obs:	exp:
cgt	R	0	4.58
cgc	R	0	8.31
cga	R	0	21.08
cgg	R	0	19.84
act	T	0	0.62
acc	T	0	0.35
aca	T	2	0.87
acg	T	0	0.16
aaa	K	40371	26580.00
aag	K	59	13850.00
aga	R	231	113.30
agg	R	0	63.89
gaa	E	2	1.18
gag	E	0	0.82
---	--	------	------
mPD		0.014	0.47
		nPD:	0.03
		N. weight:	0.46
		Sc. PD:	-0.011
		Sc. rank:	2523.9

,

PA

Pos.	374	obs:	exp:
tct	S	0	0.16
tcc	S	0	0.15
tca	S	0	0.27
tcg	S	1	0.04
act	T	63	12628.00
acc	T	7	7189.00
aca	T	657	17598.00
acg	T	39937	3248.00
agt	S	0	0.17
agc	S	0	0.21
---	--	------	------
mPD		0.035	0.68
		nPD:	0.05
		N. weight:	2.7
		Sc. PD:	-0.011
		Sc. rank:	40003.0

,

PA

Pos.	375	obs:	exp:
tct	S	0	6540.00
tcc	S	0	6056.00
tca	S	0	10893.00
tcg	S	0	1595.00
tgt	C	2	0.78
tgc	C	0	1.22
act	T	2	0.62
acc	T	0	0.35
aca	T	0	0.87
acg	T	0	0.16
aat	N	37	24.06
aac	N	6	18.94
agt	S	40154	7082.00
agc	S	374	8361.00
ggt	G	90	12.12
ggc	G	0	12.56
gga	G	0	41.55
ggg	G	0	23.78
---	--	------	------
mPD		0.025	1.6
		nPD:	0.02
		N. weight:	1.9
		Sc. PD:	-0.074
		Sc. rank:	-18475.0

,

PA

Pos.	376	obs:	exp:
tta	L	0	0.12
ttg	L	0	0.21
ctt	L	0	0.21
ctc	L	0	0.15
cta	L	1	0.15
ctg	L	0	0.17
cat	H	3	1.69
cac	H	0	1.31
caa	Q	40388	24855.00
cag	Q	273	15806.00
---	--	------	------
mPD		0.014	0.48
		nPD:	0.03
		N. weight:	0.5
		Sc. PD:	-0.013
		Sc. rank:	1885.7

,

PA

Pos.	377	obs:	exp:
tta	L	4	4718.00
ttg	L	311	8406.00
ctt	L	106	8506.00
ctc	L	0	5962.00
cta	L	2092	6244.00
ctg	L	38147	6824.00
atg	M	5	5.00
---	--	------	------
mPD		0.12	1.2
		nPD:	0.1
		N. weight:	1.7
		Sc. PD:	0.075
		Sc. rank:	39250.0

,

PA

Pos.	378	obs:	exp:
cgt	R	0	0.26
cgc	R	0	0.47
cga	R	0	1.19
cgg	R	0	1.12
atg	M	1	1.00
aat	N	1	1.12
aac	N	1	0.88
aaa	K	437	26723.00
aag	K	40210	13924.00
aga	R	0	6.38
agg	R	13	3.60
gaa	E	0	1.18
gag	E	2	0.82
---	--	------	------
mPD		0.022	0.45
		nPD:	0.05
		N. weight:	1.1
		Sc. PD:	-0.0075
		Sc. rank:	15408.0

,

PA

Pos.	379	obs:	exp:
tgt	C	1	0.39
tgc	C	0	0.61
tgg	W	40664	40664.00
---	--	------	------
mPD		0.000049	0.000049
		nPD:	1.
		N. weight:	0.000026
		Sc. PD:	0.000023
		Sc. rank:	0.8

,

PA

Pos.	380	obs:	exp:
tct	S	1	0.16
tcc	S	0	0.15
tca	S	0	0.27
tcg	S	0	0.04
act	T	1	0.31
acc	T	0	0.18
aca	T	0	0.43
acg	T	0	0.08
agt	S	0	0.17
agc	S	0	0.21
gtt	V	7	1.75
gtc	V	0	1.43
gta	V	0	1.52
gtg	V	0	2.30
gct	A	40049	11081.00
gcc	A	310	10158.00
gca	A	291	16446.00
gcg	A	6	2972.00
---	--	------	------
mPD		0.030	0.69
		nPD:	0.04
		N. weight:	1.3
		Sc. PD:	-0.017
		Sc. rank:	16348.0

,

PA

Pos.	381	obs:	exp:
ttt	F	1	0.49
ttc	F	0	0.51
tta	L	0	4719.00
ttg	L	0	8407.00
ctt	L	40499	8506.00
ctc	L	94	5963.00
cta	L	49	6244.00
ctg	L	22	6825.00
---	--	------	------
mPD		0.0081	1.2
		nPD:	0.01
		N. weight:	1.7
		Sc. PD:	-0.081
		Sc. rank:	-34006.0

,

PA

Pos.	382	obs:	exp:
ggt	G	40598	5475.00
ggc	G	62	5676.00
gga	G	2	18772.00
ggg	G	3	10743.00
---	--	------	------
mPD		0.0033	0.68
		nPD:	0.
		N. weight:	2.2
		Sc. PD:	-0.11
		Sc. rank:	-50970.0

,

PA

Pos.	383	obs:	exp:
gaa	E	40195	23974.00
gag	E	469	16690.00
ggt	G	0	0.13
ggc	G	0	0.14
gga	G	0	0.46
ggg	G	1	0.26
---	--	------	------
mPD		0.023	0.48
		nPD:	0.05
		N. weight:	0.52
		Sc. PD:	-0.0044
		Sc. rank:	6840.1

,

PA

Pos.	384	obs:	exp:
tct	S	0	0.65
tcc	S	0	0.60
tca	S	0	1.08
tcg	S	0	0.16
act	T	0	0.31
acc	T	1	0.18
aca	T	0	0.43
acg	T	0	0.08
aat	N	247	22744.00
aac	N	40406	17909.00
aaa	K	5	3.29
aag	K	0	1.71
agt	S	0	0.70
agc	S	4	0.83
gat	D	0	1.13
gac	D	2	0.87
---	--	------	------
mPD		0.013	0.49
		nPD:	0.03
		N. weight:	0.86
		Sc. PD:	-0.025
		Sc. rank:	1208.6

,

PA

Pos.	385	obs:	exp:
tta	L	0	0.35
ttg	L	1	0.62
ctt	L	0	0.63
ctc	L	0	0.44
cta	L	0	0.46
ctg	L	2	0.50
cgt	R	0	0.06
cgc	R	0	0.11
cga	R	0	0.27
cgg	R	0	0.26
att	I	3	7.02
atc	I	0	5.26
ata	I	17	7.72
atg	M	40633	40633.00
act	T	0	1.55
acc	T	0	0.88
aca	T	0	2.16
acg	T	5	0.40
aga	R	0	1.47
agg	R	3	0.83
gtt	V	0	0.25
gtc	V	0	0.20
gta	V	0	0.22
gtg	V	1	0.33
---	--	------	------
mPD		0.0016	0.0021
		nPD:	0.77
		N. weight:	0.00092
		Sc. PD:	0.00064
		Sc. rank:	29.3

,

PA

Pos.	386	obs:	exp:
gct	A	6	11083.00
gcc	A	15	10160.00
gca	A	40615	16449.00
gcg	A	29	2973.00
---	--	------	------
mPD		0.0025	0.69
		nPD:	0.
		N. weight:	0.99
		Sc. PD:	-0.051
		Sc. rank:	-25197.0

,

PA

Pos.	387	obs:	exp:
cct	P	13	13551.00
ccc	P	45	7624.00
cca	P	40564	13739.00
ccg	P	43	5751.00
---	--	------	------
mPD		0.0050	0.72
		nPD:	0.01
		N. weight:	1.2
		Sc. PD:	-0.056
		Sc. rank:	-23960.0

,

PA

Pos.	388	obs:	exp:
gat	D	0	0.57
gac	D	1	0.43
gaa	E	1926	23973.00
gag	E	38737	16690.00
ggt	G	0	0.13
ggc	G	0	0.14
gga	G	0	0.46
ggg	G	1	0.26
---	--	------	------
mPD		0.090	0.48
		nPD:	0.19
		N. weight:	0.76
		Sc. PD:	0.096
		Sc. rank:	20645.0

,

PA

Pos.	389	obs:	exp:
cgt	R	0	0.06
cgc	R	0	0.11
cga	R	0	0.27
cgg	R	0	0.26
aaa	K	1067	26733.00
aag	K	39595	13929.00
aga	R	1	1.47
agg	R	2	0.83
---	--	------	------
mPD		0.051	0.45
		nPD:	0.11
		N. weight:	1.
		Sc. PD:	0.058
		Sc. rank:	25032.0

,

PA

Pos.	390	obs:	exp:
tta	L	1	0.35
ttg	L	0	0.62
ctt	L	0	0.63
ctc	L	0	0.44
cta	L	2	0.46
ctg	L	0	0.50
att	I	0	25.27
atc	I	0	18.92
ata	I	72	27.81
atg	M	1	1.00
gtt	V	7	10166.00
gtc	V	4	8270.00
gta	V	40437	8797.00
gtg	V	138	13354.00
gct	A	0	0.82
gcc	A	0	0.75
gca	A	3	1.21
gcg	A	0	0.22
---	--	------	------
mPD		0.011	0.74
		nPD:	0.02
		N. weight:	1.7
		Sc. PD:	-0.066
		Sc. rank:	-17016.0

,

PA

Pos.	391	obs:	exp:
tat	Y	0	1.05
tac	Y	2	0.95
cat	H	0	0.56
cac	H	1	0.44
aat	N	0	1.12
aac	N	2	0.88
gat	D	561	22978.00
gac	D	40090	17673.00
gaa	E	7	4.13
gag	E	0	2.87
ggt	G	0	0.27
ggc	G	2	0.28
gga	G	0	0.92
ggg	G	0	0.53
---	--	------	------
mPD		0.028	0.49
		nPD:	0.06
		N. weight:	0.84
		Sc. PD:	0.00054
		Sc. rank:	13823.0

,

PA

Pos.	392	obs:	exp:
ttt	F	40610	19777.00
ttc	F	48	20881.00
tta	L	0	0.35
ttg	L	0	0.62
tgt	C	1	0.39
tgc	C	0	0.61
ctt	L	3	0.63
ctc	L	0	0.44
cta	L	0	0.46
ctg	L	0	0.50
att	I	2	0.70
atc	I	0	0.53
ata	I	0	0.77
gtt	V	1	0.25
gtc	V	0	0.20
gta	V	0	0.22
gtg	V	0	0.33
---	--	------	------
mPD		0.0027	0.50
		nPD:	0.01
		N. weight:	0.79
		Sc. PD:	-0.039
		Sc. rank:	-17616.0

,

PA

Pos.	393	obs:	exp:
caa	Q	1	0.61
cag	Q	0	0.39
aaa	K	3	1.97
aag	K	0	1.03
gtt	V	0	0.25
gtc	V	0	0.20
gta	V	1	0.22
gtg	V	0	0.33
gat	D	209	452.20
gac	D	591	347.80
gaa	E	39361	23496.00
gag	E	493	16358.00
ggt	G	0	0.81
ggc	G	0	0.84
gga	G	6	2.77
ggg	G	0	1.59
---	--	------	------
mPD		0.063	0.50
		nPD:	0.12
		N. weight:	0.51
		Sc. PD:	0.034
		Sc. rank:	12742.0

,

PA

Pos.	394	obs:	exp:
tct	S	0	4.84
tcc	S	0	4.48
tca	S	0	8.06
tcg	S	0	1.18
cat	H	0	1.69
cac	H	3	1.31
att	I	0	0.35
atc	I	1	0.26
ata	I	0	0.39
act	T	0	0.93
acc	T	3	0.53
aca	T	0	1.30
acg	T	0	0.24
aat	N	196	22581.00
aac	N	40165	17780.00
aaa	K	2	1.31
aag	K	0	0.69
agt	S	0	5.24
agc	S	30	6.19
gat	D	4	149.80
gac	D	261	115.20
---	--	------	------
mPD		0.025	0.51
		nPD:	0.05
		N. weight:	0.87
		Sc. PD:	-0.0064
		Sc. rank:	11934.0

,

PA

Pos.	395	obs:	exp:
ttt	F	0	0.49
ttc	F	1	0.51
tct	S	0	0.16
tcc	S	0	0.15
tca	S	0	0.27
tcg	S	0	0.04
tat	Y	0	1.05
tac	Y	2	0.95
tgt	C	154	15890.00
tgc	C	40506	24770.00
agt	S	0	0.17
agc	S	1	0.21
ggt	G	0	0.13
ggc	G	1	0.14
gga	G	0	0.46
ggg	G	0	0.26
---	--	------	------
mPD		0.0078	0.48
		nPD:	0.02
		N. weight:	0.52
		Sc. PD:	-0.02
		Sc. rank:	-4365.9

,

PA

Pos.	396	obs:	exp:
tct	S	0	0.16
tcc	S	0	0.15
tca	S	0	0.27
tcg	S	0	0.04
cgt	R	0	800.60
cgc	R	0	1453.00
cga	R	2	3684.00
cgg	R	0	3467.00
att	I	0	0.70
atc	I	0	0.53
ata	I	2	0.77
aaa	K	281	184.70
aag	K	0	96.26
agt	S	0	0.17
agc	S	1	0.21
aga	R	40313	19806.00
agg	R	65	11169.00
ggt	G	0	0.13
ggc	G	0	0.14
gga	G	1	0.46
ggg	G	0	0.26
---	--	------	------
mPD		0.017	0.90
		nPD:	0.02
		N. weight:	0.77
		Sc. PD:	-0.028
		Sc. rank:	-4152.2

,

PA

Pos.	397	obs:	exp:
aat	N	0	2.80
aac	N	5	2.20
gat	D	336	22963.00
gac	D	40288	17661.00
gaa	E	27	15.92
gag	E	0	11.08
ggt	G	0	1.21
ggc	G	9	1.26
gga	G	0	4.15
ggg	G	0	2.38
---	--	------	------
mPD		0.018	0.49
		nPD:	0.04
		N. weight:	0.87
		Sc. PD:	-0.016
		Sc. rank:	8231.6

,

PA

Pos.	398	obs:	exp:
tta	L	3	0.46
ttg	L	0	0.83
ctt	L	0	0.84
ctc	L	0	0.59
cta	L	1	0.61
ctg	L	0	0.67
att	I	1	14123.00
atc	I	10	10571.00
ata	I	40220	15537.00
atg	M	23	23.00
act	T	0	47.83
acc	T	0	27.23
aca	T	154	66.65
acg	T	0	12.30
gtt	V	43	63.37
gtc	V	163	51.55
gta	V	47	54.84
gtg	V	0	83.24
---	--	------	------
mPD		0.032	0.68
		nPD:	0.05
		N. weight:	1.
		Sc. PD:	-0.0094
		Sc. rank:	13680.0

,

PA

Pos.	399	obs:	exp:
tct	S	0	6492.00
tcc	S	0	6012.00
tca	S	0	10813.00
tcg	S	0	1584.00
cgt	R	1	0.06
cgc	R	0	0.11
cga	R	0	0.27
cgg	R	0	0.26
att	I	0	6.32
atc	I	18	4.73
ata	I	0	6.95
aat	N	1	44.76
aac	N	79	35.24
agt	S	247	7030.00
agc	S	39982	8299.00
aga	R	2	1.47
agg	R	0	0.83
gat	D	1	0.57
gac	D	0	0.43
ggt	G	180	44.96
ggc	G	126	46.62
gga	G	26	154.20
ggg	G	2	88.24
---	--	------	------
mPD		0.044	1.6
		nPD:	0.03
		N. weight:	1.7
		Sc. PD:	-0.049
		Sc. rank:	3785.9

,

PA			
Pos.	400	obs:	exp:
aat	N	1	0.56
aac	N	0	0.44
gat	D	40422	22985.00
gac	D	241	17678.00
ggt	G	1	0.13
ggc	G	0	0.14
gga	G	0	0.46
ggg	G	0	0.26
---	--	------	------
mPD		0.012	0.49
		nPD:	0.02
		N. weight:	0.59
		Sc. PD:	-0.018
		Sc. rank:	-77.1

,

PA			
Pos.	401	obs:	exp:
ttt	F	1	0.49
ttc	F	0	0.51
tta	L	69	4718.00
ttg	L	40289	8406.00
tct	S	0	0.16
tcc	S	0	0.15
tca	S	0	0.27
tcg	S	1	0.04
tgg	W	2	2.00
ctt	L	31	8506.00
ctc	L	174	5963.00
cta	L	0	6244.00
ctg	L	98	6824.00
agt	S	0	0.17
agc	S	0	0.21
---	--	------	------
mPD		0.028	1.2
		nPD:	0.02
		N. weight:	1.7
		Sc. PD:	-0.051
		Sc. rank:	350.6

,

PA			
Pos.	402	obs:	exp:
caa	Q	1	0.61
cag	Q	0	0.39
cgt	R	0	1.92
cgc	R	0	3.49
cga	R	0	8.85
cgg	R	0	8.33
aat	N	1	0.56
aac	N	0	0.44
aaa	K	6730	26666.00
aag	K	33830	13894.00
aga	R	7	47.58
agg	R	90	26.83
gaa	E	0	3.54
gag	E	6	2.46
---	--	------	------
mPD		0.28	0.46
		nPD:	0.62
		N. weight:	0.57
		Sc. PD:	0.31
		Sc. rank:	17896.0

,

PA			
Pos.	403	obs:	exp:
cat	H	1	0.56
cac	H	0	0.44
caa	Q	40402	24857.00
cag	Q	262	15807.00
---	--	------	------
mPD		0.013	0.48
		nPD:	0.03
		N. weight:	0.5
		Sc. PD:	-0.014
		Sc. rank:	1374.9

,

PA			
Pos.	404	obs:	exp:
ttt	F	16	7.78
ttc	F	0	8.22
tat	Y	40583	21427.00
tac	Y	66	19222.00
---	--	------	------
mPD		0.0040	0.50
		nPD:	0.01
		N. weight:	0.7
		Sc. PD:	-0.032
		Sc. rank:	-12640.0

,

PA			
Pos.	405	obs:	exp:
aat	N	183	155.50
aac	N	95	122.50
gtt	V	2	0.50
gtc	V	0	0.41
gta	V	0	0.43
gtg	V	0	0.66
gct	A	1	0.27
gcc	A	0	0.25
gca	A	0	0.40
gcg	A	0	0.07
gat	D	32716	22802.00
gac	D	7623	17537.00
gaa	E	3	16.51
gag	E	25	11.49
ggt	G	14	2.29
ggc	G	3	2.37
gga	G	0	7.85
ggg	G	0	4.49
---	--	------	------
mPD		0.32	0.51
		nPD:	0.64
		N. weight:	0.15
		Sc. PD:	0.085
		Sc. rank:	4739.2

,

PA			
Pos.	406	obs:	exp:
tct	S	0	6559.00
tcc	S	0	6074.00
tca	S	0	10925.00
tcg	S	0	1600.00
tgt	C	2	0.78
tgc	C	0	1.22
aat	N	12	6.71
aac	N	0	5.29
agt	S	40492	7103.00
agc	S	154	8385.00
ggt	G	5	0.67
ggc	G	0	0.70
gga	G	0	2.31
ggg	G	0	1.32
---	--	------	------
mPD		0.0085	1.6
		nPD:	0.01
		N. weight:	1.9
		Sc. PD:	-0.094
		Sc. rank:	-43109.0

,

PA			
Pos.	407	obs:	exp:
aat	N	0	1.12
aac	N	2	0.88
aaa	K	9	5.92
aag	K	0	3.08
gtt	V	0	0.50
gtc	V	1	0.41
gta	V	1	0.43
gtg	V	0	0.66
gct	A	0	0.55
gcc	A	0	0.50
gca	A	2	0.81
gcg	A	0	0.15
gat	D	242	1727.00
gac	D	2814	1329.00
gaa	E	37515	22151.00
gag	E	57	15421.00
ggt	G	0	2.96
ggc	G	0	3.07
gga	G	22	10.16
ggg	G	0	5.81
---	--	------	------
mPD		0.14	0.56
		nPD:	0.26
		N. weight:	0.57
		Sc. PD:	0.11
		Sc. rank:	16324.0

,

PA			
Pos.	408	obs:	exp:
aaa	K	12	8.55
aag	K	1	4.45
gct	A	0	0.55
gcc	A	0	0.50
gca	A	2	0.81
gcg	A	0	0.15
gat	D	0	0.57
gac	D	1	0.43
gaa	E	40283	23940.00
gag	E	324	16667.00
ggt	G	0	5.65
ggc	G	0	5.86
gga	G	42	19.39
ggg	G	0	11.10
---	--	------	------
mPD		0.019	0.49
		nPD:	0.04
		N. weight:	0.54
		Sc. PD:	-0.0092
		Sc. rank:	5577.1

,

PA			
Pos.	409	obs:	exp:
cct	P	39774	13551.00
ccc	P	36	7624.00
cca	P	849	13739.00
ccg	P	6	5751.00
---	--	------	------
mPD		0.043	0.72
		nPD:	0.06
		N. weight:	1.1
		Sc. PD:	0.0038
		Sc. rank:	19153.0

,

PA			
Pos.	410	obs:	exp:
cgt	R	0	0.02
cgc	R	0	0.04
cga	R	0	0.09
cgg	R	0	0.09
aaa	K	30	19.72
aag	K	0	10.28
aga	R	1	0.49
agg	R	0	0.28
gtt	V	0	0.50
gtc	V	0	0.41
gta	V	2	0.43
gtg	V	0	0.66
gct	A	0	0.27
gcc	A	0	0.25
gca	A	1	0.40
gcg	A	0	0.07
gat	D	0	2.83
gac	D	5	2.17
gaa	E	40140	23919.00
gag	E	431	16652.00
ggt	G	0	7.40
ggc	G	0	7.68
gga	G	54	25.39
ggg	G	1	14.53
---	--	------	------
mPD		0.026	0.49
		nPD:	0.05
		N. weight:	0.52
		Sc. PD:	-0.0018
		Sc. rank:	7915.1

,

PA			
Pos.	411	obs:	exp:
ttt	F	26	12.65
ttc	F	0	13.35
tta	L	39768	4662.00
ttg	L	349	8306.00
tct	S	3	38.57
tcc	S	0	35.72
tca	S	236	64.24
tcg	S	0	9.41
ctt	L	0	8404.00
ctc	L	0	5891.00
cta	L	48	6169.00
ctg	L	8	6742.00
cct	P	175	69.65
ccc	P	31	39.18
cca	P	3	70.61
ccg	P	0	29.56
att	I	0	3.51
atc	I	0	2.63
ata	I	10	3.86
atg	M	1	1.00
agt	S	0	41.77
agc	S	0	49.30
gtt	V	0	1.75
gtc	V	0	1.43
gta	V	7	1.52
gtg	V	0	2.30
---	--	------	------
mPD		0.064	1.2
		nPD:	0.05
		N. weight:	2.3
		Sc. PD:	-0.0037
		Sc. rank:	36947.0

,

PA			
Pos.	412	obs:	exp:
cgt	R	0	806.20
cgc	R	0	1463.00
cga	R	0	3710.00
cgg	R	2	3491.00
aaa	K	3	3.29
aag	K	2	1.71
aga	R	2286	19943.00
agg	R	38372	11247.00
---	--	------	------
mPD		0.11	0.89
		nPD:	0.12
		N. weight:	1.1
		Sc. PD:	0.072
		Sc. rank:	28437.0

,

PA			
Pos.	413	obs:	exp:
tct	S	205	5386.00
tcc	S	8	4988.00
tca	S	33138	8971.00
tcg	S	24	1314.00
act	T	0	1935.00
acc	T	1	1101.00
aca	T	6205	2696.00
acg	T	24	497.60
agt	S	0	5832.00
agc	S	0	6885.00
gct	A	0	288.90
gcc	A	0	264.80
gca	A	1060	428.80
gcg	A	0	77.49
---	--	------	------
mPD		0.32	1.6
		nPD:	0.19
		N. weight:	1.3
		Sc. PD:	0.17
		Sc. rank:	36419.0

,

PA			
Pos.	414	obs:	exp:
ttt	F	44	21.40
ttc	F	0	22.60
tta	L	0	4693.00
ttg	L	0	8362.00
ctt	L	40296	8461.00
ctc	L	91	5931.00
cta	L	51	6211.00
ctg	L	9	6788.00
cct	P	7	2.33
ccc	P	0	1.31
cca	P	0	2.36
ccg	P	0	0.99
att	I	9	58.62
atc	I	0	43.88
ata	I	158	64.49
---	--	------	------
mPD		0.026	1.2
		nPD:	0.02
		N. weight:	1.7
		Sc. PD:	-0.056
		Sc. rank:	-3154.6

,

PA			
Pos.	415	obs:	exp:
tct	S	0	6525.00
tcc	S	2	6043.00
tca	S	40384	10868.00
tcg	S	50	1592.00
cct	P	0	4.33
ccc	P	0	2.44
cca	P	13	4.39
ccg	P	0	1.84
act	T	0	1.24
acc	T	0	0.71
aca	T	4	1.73
acg	T	0	0.32
agt	S	0	7066.00
agc	S	0	8342.00
gct	A	0	57.78
gcc	A	0	52.97
gca	A	211	85.76
gcg	A	1	15.50
---	--	------	------
mPD		0.014	1.6
		nPD:	0.01
		N. weight:	1.4
		Sc. PD:	-0.067
		Sc. rank:	-25746.0

,

PA			
Pos.	416	obs:	exp:
tct	S	0	6560.00
tcc	S	0	6075.00
tca	S	0	10927.00
tcg	S	0	1600.00
att	I	0	1.40
atc	I	4	1.05
ata	I	0	1.54
aat	N	0	2.80
aac	N	5	2.20
agt	S	71	7104.00
agc	S	40583	8387.00
ggt	G	0	0.27
ggc	G	2	0.28
gga	G	0	0.92
ggg	G	0	0.53
---	--	------	------
mPD		0.0040	1.6
		nPD:	0.
		N. weight:	1.7
		Sc. PD:	-0.09
		Sc. rank:	-48780.0

,

PA			
Pos.	417	obs:	exp:
tgg	W	40664	40664.00
cgt	R	0	0.02
cgc	R	0	0.04
cga	R	0	0.09
cgg	R	1	0.09
aga	R	0	0.49
agg	R	0	0.28
---	--	------	------
mPD		0.000049	0.000081
		nPD:	0.61
		N. weight:	0.000067
		Sc. PD:	0.000036
		Sc. rank:	2.1

,

PA			
Pos.	418	obs:	exp:
att	I	8	11898.00
atc	I	65	8906.00
ata	I	33821	13090.00
atg	M	1141	1141.00
act	T	0	1.86
acc	T	0	1.06
aca	T	6	2.60
acg	T	0	0.48
gtt	V	0	1409.00
gtc	V	202	1146.00
gta	V	5416	1219.00
gtg	V	6	1850.00
---	--	------	------
mPD		0.31	0.94
		nPD:	0.33
		N. weight:	1.1
		Sc. PD:	0.28
		Sc. rank:	32099.0

,

PA			
Pos.	419	obs:	exp:
caa	Q	10579	24858.00
cag	Q	30086	15807.00
---	--	------	------
mPD		0.38	0.48
		nPD:	0.81
		N. weight:	0.28
		Sc. PD:	0.21
		Sc. rank:	8921.0

,

PA			
Pos.	420	obs:	exp:
tct	S	0	6177.00
tcc	S	0	5720.00
tca	S	0	10288.00
tcg	S	0	1507.00
tat	Y	1	0.53
tac	Y	0	0.47
tgt	C	1	0.39
tgc	C	0	0.61
att	I	2	0.70
atc	I	0	0.53
ata	I	0	0.77
aat	N	2256	1332.00
aac	N	124	1048.00
agt	S	38257	6689.00
agc	S	20	7896.00
ggt	G	4	0.54
ggc	G	0	0.56
gga	G	0	1.85
ggg	G	0	1.06
---	--	------	------
mPD		0.12	1.7
		nPD:	0.07
		N. weight:	1.8
		Sc. PD:	0.024
		Sc. rank:	35561.0

,

PA			
Pos.	421	obs:	exp:
gat	D	1	0.57
gac	D	0	0.43
gaa	E	1300	23974.00
gag	E	39364	16690.00
---	--	------	------
mPD		0.062	0.48
		nPD:	0.13
		N. weight:	0.82
		Sc. PD:	0.057
		Sc. rank:	20583.0

,

PA			
Pos.	422	obs:	exp:
ttt	F	30	19780.00
ttc	F	40633	20883.00
tta	L	0	0.23
ttg	L	1	0.41
ctt	L	0	0.42
ctc	L	1	0.29
cta	L	0	0.31
ctg	L	0	0.34
---	--	------	------
mPD		0.0016	0.50
		nPD:	0.
		N. weight:	0.73
		Sc. PD:	-0.038
		Sc. rank:	-19193.0

,

PA			
Pos.	423	obs:	exp:
aat	N	107	22751.00
aac	N	40558	17914.00
---	--	------	------
mPD		0.0052	0.49
		nPD:	0.01
		N. weight:	0.89
		Sc. PD:	-0.039
		Sc. rank:	-14090.0

,

PA

Pos.	424	obs:	exp:
cgt	R	0	0.08
cgc	R	0	0.14
cga	R	0	0.36
cgg	R	0	0.34
aaa	K	286	26732.00
aag	K	40375	13929.00
aga	R	0	1.96
agg	R	4	1.11
---	--	------	------
mPD		0.014	0.45
		nPD:	0.03
		N. weight:	1.1
		Sc. PD:	-0.027
		Sc. rank:	6590.7

,

PA

Pos.	425	obs:	exp:
tct	S	0	0.32
tcc	S	2	0.30
tca	S	0	0.54
tcg	S	0	0.08
act	T	0	0.62
acc	T	2	0.35
aca	T	0	0.87
acg	T	0	0.16
agt	S	0	0.35
agc	S	0	0.41
gct	A	63	11082.00
gcc	A	40084	10158.00
gca	A	512	16447.00
gcg	A	0	2972.00
ggt	G	0	0.27
ggc	G	2	0.28
gga	G	0	0.92
ggg	G	0	0.53
---	--	------	------
mPD		0.028	0.69
		nPD:	0.04
		N. weight:	1.4
		Sc. PD:	-0.022
		Sc. rank:	16176.0

,

PA

Pos.	426	obs:	exp:
tct	S	0	0.32
tcc	S	0	0.30
tca	S	0	0.54
tcg	S	0	0.08
tgt	C	39879	15890.00
tgc	C	783	24772.00
tgg	W	1	1.00
agt	S	2	0.35
agc	S	0	0.41
---	--	------	------
mPD		0.038	0.48
		nPD:	0.08
		N. weight:	0.93
		Sc. PD:	0.021
		Sc. rank:	19339.0

,

PA

Pos.	427	obs:	exp:
gat	D	7	3.96
gac	D	0	3.04
gaa	E	376	23970.00
gag	E	40282	16688.00
---	--	------	------
mPD		0.019	0.48
		nPD:	0.04
		N. weight:	0.92
		Sc. PD:	-0.016
		Sc. rank:	9670.3

,

PA

Pos.	428	obs:	exp:
tta	L	174	4718.00
ttg	L	42	8405.00
ctt	L	9	8505.00
ctc	L	2	5962.00
cta	L	40071	6243.00
ctg	L	358	6823.00
att	I	0	3.16
atc	I	0	2.36
ata	I	9	3.48
---	--	------	------
mPD		0.031	1.2
		nPD:	0.03
		N. weight:	2.
		Sc. PD:	-0.056
		Sc. rank:	5121.3

,

PA

Pos.	429	obs:	exp:
tta	L	0	0.23
ttg	L	0	0.41
tct	S	0	0.16
tcc	S	0	0.15
tca	S	0	0.27
tcg	S	0	0.04
ctt	L	0	0.42
ctc	L	0	0.29
cta	L	0	0.31
ctg	L	2	0.34
act	T	40373	12627.00
acc	T	253	7189.00
aca	T	32	17597.00
acg	T	3	3248.00
agt	S	1	0.17
agc	S	0	0.21
ggt	G	1	0.13
ggc	G	0	0.14
gga	G	0	0.46
ggg	G	0	0.26
---	--	------	------
mPD		0.015	0.68
		nPD:	0.02
		N. weight:	1.2
		Sc. PD:	-0.042
		Sc. rank:	-3757.1

,

PA

Pos.	430	obs:	exp:
aat	N	12	7.83
aac	N	2	6.17
gat	D	40517	22971.00
gac	D	120	17666.00
gaa	E	5	5.90
gag	E	5	4.10
ggt	G	4	0.54
ggc	G	0	0.56
gga	G	0	1.85
ggg	G	0	1.06
---	--	------	------
mPD		0.0074	0.49
		nPD:	0.01
		N. weight:	0.61
		Sc. PD:	-0.024
		Sc. rank:	-6407.7

,

PA

Pos.	431	obs:	exp:
tta	L	3	0.35
ttg	L	0	0.62
tct	S	1395	6561.00
tcc	S	4	6076.00
tca	S	38160	10928.00
tcg	S	1099	1600.00
ctt	L	0	0.63
ctc	L	0	0.44
cta	L	0	0.46
ctg	L	0	0.50
cct	P	0	0.33
ccc	P	0	0.19
cca	P	1	0.34
ccg	P	0	0.14
agt	S	0	7105.00
agc	S	0	8388.00
gct	A	0	0.82
gcc	A	0	0.75
gca	A	3	1.21
gcg	A	0	0.22
---	--	------	------
mPD		0.12	1.6
		nPD:	0.07
		N. weight:	1.2
		Sc. PD:	0.019
		Sc. rank:	24491.0

,

PA

Pos.	432	obs:	exp:
tct	S	0	33.73
tcc	S	0	31.23
tca	S	0	56.18
tcg	S	0	8.23
att	I	24	371.80
atc	I	1029	278.30
ata	I	6	409.00
act	T	24	8.70
acc	T	4	4.95
aca	T	0	12.12
acg	T	0	2.24
agt	S	1	36.52
agc	S	208	43.12
gtt	V	7238	9843.00
gtc	V	31857	8007.00
gta	V	201	8517.00
gtg	V	1	12930.00
gct	A	5	19.08
gcc	A	65	17.49
gca	A	0	28.32
gcg	A	0	5.12
ggt	G	0	0.27
ggc	G	2	0.28
gga	G	0	0.92
ggg	G	0	0.53
---	--	------	------
mPD		0.38	0.82
		nPD:	0.46
		N. weight:	1.2
		Sc. PD:	0.46
		Sc. rank:	35878.0

,

PA

Pos.	433	obs:	exp:
tgg	W	40665	40665.00
---	--	------	------
mPD		0	0
		nPD:	1.
		N. weight:	0.
		Sc. PD:	0
		Sc. rank:	0

,

PA

Pos.	434	obs:	exp:
tta	L	3	0.70
ttg	L	0	1.24
ctt	L	0	1.26
ctc	L	0	0.88
cta	L	3	0.92
ctg	L	0	1.01
att	I	57	14253.00
atc	I	0	10669.00
ata	I	40546	15681.00
atg	M	34	34.00
act	T	0	0.31
acc	T	0	0.18
aca	T	1	0.43
acg	T	0	0.08
gtt	V	0	5.26
gtc	V	0	4.28
gta	V	21	4.55
gtg	V	0	6.91
---	--	------	------
mPD		0.0058	0.66
		nPD:	0.01
		N. weight:	1.
		Sc. PD:	-0.048
		Sc. rank:	-18114.0

,

PA

Pos.	435	obs:	exp:
gat	D	2	1.13
gac	D	0	0.87
gaa	E	1152	23973.00
gag	E	39511	16690.00
---	--	------	------
mPD		0.055	0.48
		nPD:	0.11
		N. weight:	0.83
		Sc. PD:	0.047
		Sc. rank:	20225.0

,

PA

Pos.	436	obs:	exp:
tta	L	0	4719.00
ttg	L	0	8407.00
ctt	L	405	8506.00
ctc	L	40223	5963.00
cta	L	35	6244.00
ctg	L	0	6824.00
cat	H	0	0.56
cac	H	1	0.44
att	I	0	0.35
atc	I	1	0.26
ata	I	0	0.39
---	--	------	------
mPD		0.022	1.2
		nPD:	0.02
		N. weight:	2.1
		Sc. PD:	-0.075
		Sc. rank:	-11948.0

,

PA

Pos.	437	obs:	exp:
gat	D	39849	22984.00
gac	D	812	17677.00
gaa	E	3	2.36
gag	E	1	1.64
---	--	------	------
mPD		0.039	0.49
		nPD:	0.08
		N. weight:	0.53
		Sc. PD:	0.012
		Sc. rank:	11079.0

,

PA

Pos.	438	obs:	exp:
gaa	E	36098	23975.00
gag	E	4567	16690.00
---	--	------	------
mPD		0.20	0.48
		nPD:	0.41
		N. weight:	0.24
		Sc. PD:	0.083
		Sc. rank:	7370.1

,

PA

Pos.	439	obs:	exp:
tta	L	1	0.35
ttg	L	0	0.62
ctt	L	2	0.63
ctc	L	0	0.44
cta	L	0	0.46
ctg	L	0	0.50
att	I	40420	14269.00
atc	I	21	10681.00
ata	I	207	15698.00
gtt	V	13	3.51
gtc	V	0	2.85
gta	V	1	3.03
gtg	V	0	4.61
---	--	------	------
mPD		0.012	0.66
		nPD:	0.02
		N. weight:	1.1
		Sc. PD:	-0.041
		Sc. rank:	-6712.7

,

PA

Pos.	440	obs:	exp:
ggt	G	19	5475.00
ggc	G	14	5676.00
gga	G	40495	18772.00
ggg	G	137	10743.00
---	--	------	------
mPD		0.0083	0.68
		nPD:	0.01
		N. weight:	0.83
		Sc. PD:	-0.035
		Sc. rank:	-11045.0

,

PA

Pos.	441	obs:	exp:
aaa	K	0	0.66
aag	K	1	0.34
gat	D	10	5.65
gac	D	0	4.35
gaa	E	527	23968.00
gag	E	40127	16686.00
---	--	------	------
mPD		0.026	0.48
		nPD:	0.05
		N. weight:	0.9
		Sc. PD:	-0.0018
		Sc. rank:	14294.0

,

PA

Pos.	442	obs:	exp:
aat	N	0	3.36
aac	N	6	2.64
gat	D	1145	22982.00
gac	D	39512	17675.00
gaa	E	0	1.18
gag	E	2	0.82
---	--	------	------
mPD		0.055	0.49
		nPD:	0.11
		N. weight:	0.77
		Sc. PD:	0.042
		Sc. rank:	18646.0

,

PA

Pos.	443	obs:	exp:
tta	L	14	1.74
ttg	L	0	3.10
ctt	L	0	3.14
ctc	L	1	2.20
cta	L	0	2.30
ctg	L	0	2.52
att	I	2	81.44
atc	I	0	60.96
ata	I	230	89.60
gtt	V	203	10122.00
gtc	V	3	8234.00
gta	V	39847	8759.00
gtg	V	358	13296.00
gct	A	0	1.91
gcc	A	0	1.75
gca	A	7	2.83
gcg	A	0	0.51
---	--	------	------
mPD		0.040	0.75
		nPD:	0.05
		N. weight:	1.6
		Sc. PD:	-0.0045
		Sc. rank:	24664.0

,

PA

Pos.	444	obs:	exp:
cct	P	0	0.33
ccc	P	1	0.19
cca	P	0	0.34
ccg	P	0	0.14
gct	A	243	11083.00
gcc	A	40363	10160.00
gca	A	58	16449.00
gcg	A	0	2973.00
---	--	------	------
mPD		0.015	0.69
		nPD:	0.02
		N. weight:	1.5
		Sc. PD:	-0.05
		Sc. rank:	-4875.1

,

PA

Pos.	445	obs:	exp:
cct	P	6	13551.00
ccc	P	14	7624.00
cca	P	39456	13739.00
ccg	P	1189	5751.00
---	--	------	------
mPD		0.058	0.72
		nPD:	0.08
		N. weight:	1.1
		Sc. PD:	0.025
		Sc. rank:	22691.0

,

PA

Pos.	446	obs:	exp:
att	I	40637	14274.00
atc	I	18	10685.00
ata	I	8	15704.00
gtt	V	2	0.50
gtc	V	0	0.41
gta	V	0	0.43
gtg	V	0	0.66
---	--	------	------
mPD		0.0014	0.66
		nPD:	0.
		N. weight:	1.2
		Sc. PD:	-0.06
		Sc. rank:	-33320.0

,

PA

Pos.	447	obs:	exp:
gaa	E	36308	23975.00
gag	E	4357	16690.00
---	--	------	------
mPD		0.19	0.48
		nPD:	0.4
		N. weight:	0.25
		Sc. PD:	0.083
		Sc. rank:	7592.4

,

PA

Pos.	448	obs:	exp:
tta	L	0	0.12
ttg	L	0	0.21
tat	Y	7	334.70
tac	Y	628	300.30
tgt	C	0	0.39
tgc	C	1	0.61
ctt	L	0	0.21
ctc	L	1	0.15
cta	L	0	0.15
ctg	L	0	0.17
cat	H	59	22576.00
cac	H	39966	17449.00
att	I	1	0.35
atc	I	0	0.26
ata	I	0	0.39
aat	N	0	1.12
aac	N	2	0.88
---	--	------	------
mPD		0.034	0.52
		nPD:	0.07
		N. weight:	0.9
		Sc. PD:	0.0085
		Sc. rank:	16504.0

,

PA

Pos.	449	obs:	exp:
att	I	40423	14266.00
atc	I	199	10678.00
ata	I	17	15695.00
act	T	5	1.55
acc	T	0	0.88
aca	T	0	2.16
acg	T	0	0.40
gtt	V	20	5.26
gtc	V	1	4.28
gta	V	0	4.55
gtg	V	0	6.91
---	--	------	------
mPD		0.012	0.66
		nPD:	0.02
		N. weight:	1.1
		Sc. PD:	-0.041
		Sc. rank:	-7205.2

,

PA

Pos.	450	obs:	exp:
gct	A	7	11083.00
gcc	A	21	10160.00
gca	A	40611	16449.00
gcg	A	26	2973.00
---	--	------	------
mPD		0.0027	0.69
		nPD:	0.
		N. weight:	0.99
		Sc. PD:	-0.05
		Sc. rank:	-24882.0

,

PA

Pos.	451	obs:	exp:
tct	S	0	6562.00
tcc	S	0	6077.00
tca	S	0	10930.00
tcg	S	0	1601.00
agt	S	4827	7106.00
agc	S	35838	8389.00
---	--	------	------
mPD		0.21	1.6
		nPD:	0.13
		N. weight:	1.4
		Sc. PD:	0.096
		Sc. rank:	34598.0

,

PA

Pos.	452	obs:	exp:
tta	L	0	1.51
ttg	L	12	2.69
ctt	L	0	2.72
ctc	L	0	1.91
cta	L	0	2.00
ctg	L	1	2.18
cgt	R	0	0.06
cgc	R	0	0.11
cga	R	0	0.27
cgg	R	0	0.26
att	I	1	11.23
atc	I	0	8.41
ata	I	31	12.36
atg	M	40485	40485.00
act	T	0	37.89
acc	T	0	21.57
aca	T	0	52.80
acg	T	122	9.74
aaa	K	0	2.63
aag	K	4	1.37
aga	R	0	1.47
agg	R	3	0.83
gtt	V	0	1.25
gtc	V	0	1.02
gta	V	0	1.08
gtg	V	5	1.65
gct	A	0	0.27
gcc	A	0	0.25
gca	A	0	0.40
gcg	A	1	0.07
---	--	------	------
mPD		0.0089	0.015
		nPD:	0.58
		N. weight:	0.01
		Sc. PD:	0.0051
		Sc. rank:	315.4

,

PA

Pos.	453	obs:	exp:
cgt	R	0	806.30
cgc	R	0	1463.00
cga	R	4	3710.00
cgg	R	0	3492.00
aga	R	39595	19946.00
agg	R	1066	11248.00
---	--	------	------
mPD		0.051	0.89
		nPD:	0.06
		N. weight:	0.67
		Sc. PD:	0.0012
		Sc. rank:	11554.0

,

PA

Pos.	454	obs:	exp:
cgt	R	0	806.30
cgc	R	0	1463.00
cga	R	0	3710.00
cgg	R	23	3492.00
aga	R	1227	19946.00
agg	R	39415	11248.00
---	--	------	------
mPD		0.060	0.89
		nPD:	0.07
		N. weight:	1.2
		Sc. PD:	0.014
		Sc. rank:	23430.0

,

PA

Pos.	455	obs:	exp:
tct	S	0	1.29
tcc	S	0	1.20
tca	S	0	2.15
tcg	S	0	0.31
aat	N	40186	22738.00
aac	N	456	17904.00
agt	S	8	1.40
agc	S	0	1.65
gat	D	15	8.48
gac	D	0	6.52
---	--	------	------
mPD		0.023	0.49
		nPD:	0.05
		N. weight:	0.58
		Sc. PD:	-0.005
		Sc. rank:	7578.9

,

PA

Pos.	456	obs:	exp:
ttt	F	12	5.84
ttc	F	0	6.16
tat	Y	33652	21428.00
tac	Y	6999	19223.00
cat	H	2	1.13
cac	H	0	0.87
---	--	------	------
mPD		0.29	0.50
		nPD:	0.57
		N. weight:	0.22
		Sc. PD:	0.11
		Sc. rank:	6935.2

,

PA

Pos.	457	obs:	exp:
ttt	F	469	19780.00
ttc	F	40195	20884.00
gtt	V	0	0.25
gtc	V	1	0.20
gta	V	0	0.22
gtg	V	0	0.33
---	--	------	------
mPD		0.023	0.50
		nPD:	0.05
		N. weight:	0.67
		Sc. PD:	-0.0067
		Sc. rank:	8577.0

,

PA

Pos.	458	obs:	exp:
tct	S	0	0.16
tcc	S	0	0.15
tca	S	1	0.27
tcg	S	0	0.04
att	I	0	0.35
atc	I	0	0.26
ata	I	1	0.39
act	T	16	12625.00
acc	T	31	7188.00
aca	T	40504	17595.00
acg	T	104	3247.00
agt	S	0	0.17
agc	S	0	0.21
gct	A	0	2.18
gcc	A	0	2.00
gca	A	8	3.24
gcg	A	0	0.58
---	--	------	------
mPD		0.0079	0.68
		nPD:	0.01
		N. weight:	0.9
		Sc. PD:	-0.039
		Sc. rank:	-13048.0

,

PA

Pos.	459	obs:	exp:
tct	S	0	2.26
tcc	S	0	2.09
tca	S	14	3.76
tcg	S	0	0.55
act	T	0	21.43
acc	T	0	12.20
aca	T	69	29.86
acg	T	0	5.51
agt	S	0	2.45
agc	S	0	2.89
gtt	V	0	1.25
gtc	V	0	1.02
gta	V	5	1.08
gtg	V	0	1.65
gct	A	81	11059.00
gcc	A	2	10137.00
gca	A	40464	16412.00
gcg	A	27	2966.00
gaa	E	2	1.18
gag	E	0	0.82
ggt	G	0	0.13
ggc	G	0	0.14
gga	G	1	0.46
ggg	G	0	0.26
---	--	------	------
mPD		0.0099	0.70
		nPD:	0.01
		N. weight:	0.98
		Sc. PD:	-0.04
		Sc. rank:	-10773.0

,

PA

Pos.	460	obs:	exp:
gaa	E	843	23975.00
gag	E	39822	16690.00
---	--	------	------
mPD		0.041	0.48
		nPD:	0.08
		N. weight:	0.87
		Sc. PD:	0.023
		Sc. rank:	18370.0

,

PA

Pos.	461	obs:	exp:
gtt	V	23	10185.00
gtc	V	4	8285.00
gta	V	205	8813.00
gtg	V	40430	13379.00
gct	A	0	0.82
gcc	A	0	0.75
gca	A	0	1.21
gcg	A	3	0.22
---	--	------	------
mPD		0.011	0.74
		nPD:	0.02
		N. weight:	1.2
		Sc. PD:	-0.047
		Sc. rank:	-11650.0

,

PA

Pos.	462	obs:	exp:
tct	S	300	6560.00
tcc	S	40347	6075.00
tca	S	8	10927.00
tcg	S	0	1600.00
act	T	0	0.31
acc	T	1	0.18
aca	T	0	0.43
acg	T	0	0.08
agt	S	0	7105.00
agc	S	0	8387.00
gct	A	0	2.45
gcc	A	9	2.25
gca	A	0	3.64
gcg	A	0	0.66
---	--	------	------
mPD		0.016	1.6
		nPD:	0.01
		N. weight:	2.1
		Sc. PD:	-0.093
		Sc. rank:	-34623.0

,

PA

Pos.	463	obs:	exp:
tta	L	0	0.46
ttg	L	0	0.83
tat	Y	12	6.33
tac	Y	0	5.67
ctt	L	4	0.84
ctc	L	0	0.59
cta	L	0	0.61
ctg	L	0	0.67
cat	H	40315	22919.00
cac	H	319	17715.00
caa	Q	1	3.67
cag	Q	5	2.33
cgt	R	9	0.18
cgc	R	0	0.32
cga	R	0	0.82
cgg	R	0	0.77
aga	R	0	4.41
agg	R	0	2.49
---	--	------	------
mPD		0.017	0.49
		nPD:	0.03
		N. weight:	0.59
		Sc. PD:	-0.012
		Sc. rank:	4789.7

,

PA			
Pos.	464	obs:	exp:
tgt	C	33249	15891.00
tgc	C	7415	24773.00
ggt	G	1	0.13
ggc	G	0	0.14
gga	G	0	0.46
ggg	G	0	0.26
---	--	------	------
mPD		0.30	0.48
		nPD:	0.63
		N. weight:	0.42
		Sc. PD:	0.24
		Sc. rank:	13352.0

,

PA			
Pos.	465	obs:	exp:
cgt	R	0	806.30
cgc	R	0	1463.00
cga	R	15	3710.00
cgg	R	0	3492.00
aaa	K	1	0.66
aag	K	0	0.34
aga	R	40349	19945.00
agg	R	300	11248.00
---	--	------	------
mPD		0.015	0.89
		nPD:	0.02
		N. weight:	0.74
		Sc. PD:	-0.028
		Sc. rank:	-5019.9

,

PA			
Pos.	466	obs:	exp:
tct	S	22	3.55
tcc	S	0	3.29
tca	S	0	5.91
tcg	S	0	0.87
agt	S	0	3.84
agc	S	0	4.54
gtt	V	1	0.25
gtc	V	0	0.20
gta	V	0	0.22
gtg	V	0	0.33
gct	A	39171	11077.00
gcc	A	1462	10154.00
gca	A	3	16439.00
gcg	A	5	2971.00
ggt	G	1	0.13
ggc	G	0	0.14
gga	G	0	0.46
ggg	G	0	0.26
---	--	------	------
mPD		0.071	0.70
		nPD:	0.1
		N. weight:	1.3
		Sc. PD:	0.056
		Sc. rank:	29405.0

,

PA			
Pos.	467	obs:	exp:
act	T	39192	12629.00
acc	T	1368	7190.00
aca	T	101	17599.00
acg	T	4	3248.00
---	--	------	------
mPD		0.070	0.68
		nPD:	0.1
		N. weight:	1.1
		Sc. PD:	0.052
		Sc. rank:	26492.0

,

PA			
Pos.	468	obs:	exp:
gaa	E	35644	23975.00
gag	E	5021	16690.00
---	--	------	------
mPD		0.22	0.48
		nPD:	0.45
		N. weight:	0.22
		Sc. PD:	0.084
		Sc. rank:	6790.7

,

PA			
Pos.	469	obs:	exp:
tat	Y	404	21434.00
tac	Y	40260	19230.00
ggt	G	0	0.13
ggc	G	0	0.14
gga	G	0	0.46
ggg	G	1	0.26
---	--	------	------
mPD		0.020	0.50
		nPD:	0.04
		N. weight:	0.77
		Sc. PD:	-0.012
		Sc. rank:	8472.3

,

PA			
Pos.	470	obs:	exp:
tta	L	1	0.12
ttg	L	0	0.21
ctt	L	0	0.21
ctc	L	0	0.15
cta	L	0	0.15
ctg	L	0	0.17
att	I	9	14265.00
atc	I	2	10678.00
ata	I	40625	15694.00
atg	M	11	11.00
act	T	0	0.31
acc	T	0	0.18
aca	T	1	0.43
acg	T	0	0.08
gtt	V	0	3.76
gtc	V	0	3.06
gta	V	15	3.25
gtg	V	0	4.94
ggt	G	0	0.13
ggc	G	0	0.14
gga	G	1	0.46
ggg	G	0	0.26
---	--	------	------
mPD		0.0020	0.66
		nPD:	0.
		N. weight:	1.1
		Sc. PD:	-0.054
		Sc. rank:	-28003.0

,

PA			
Pos.	471	obs:	exp:
tta	L	0	0.12
ttg	L	0	0.21
ctt	L	0	0.21
ctc	L	0	0.15
cta	L	0	0.15
ctg	L	1	0.17
att	I	6	6.67
atc	I	0	4.99
ata	I	13	7.34
atg	M	40641	40641.00
gtt	V	0	1.00
gtc	V	0	0.82
gta	V	0	0.87
gtg	V	4	1.32
---	--	------	------
mPD		0.0012	0.0013
		nPD:	0.88
		N. weight:	0.0004
		Sc. PD:	0.00032
		Sc. rank:	12.7

,

PA			
Pos.	472	obs:	exp:
cgt	R	0	0.02
cgc	R	0	0.04
cga	R	0	0.09
cgg	R	0	0.09
aat	N	1	0.56
aac	N	0	0.44
aaa	K	30787	26733.00
aag	K	9876	13930.00
aga	R	0	0.49
agg	R	1	0.28
---	--	------	------
mPD		0.37	0.45
		nPD:	0.82
		N. weight:	0.026
		Sc. PD:	0.019
		Sc. rank:	826.7

,

PA			
Pos.	473	obs:	exp:
ggt	G	1	5475.00
ggc	G	0	5676.00
gga	G	397	18772.00
ggg	G	40267	10743.00
---	--	------	------
mPD		0.019	0.68
		nPD:	0.03
		N. weight:	1.4
		Sc. PD:	-0.038
		Sc. rank:	5673.1

,

PA			
Pos.	474	obs:	exp:
att	I	0	5.97
atc	I	0	4.47
ata	I	17	6.57
gtt	V	8	10181.00
gtc	V	5	8282.00
gta	V	40260	8810.00
gtg	V	374	13374.00
gct	A	0	0.27
gcc	A	0	0.25
gca	A	1	0.40
gcg	A	0	0.07
---	--	------	------
mPD		0.020	0.74
		nPD:	0.03
		N. weight:	1.6
		Sc. PD:	-0.046
		Sc. rank:	3936.8

,

PA			
Pos.	475	obs:	exp:
tat	Y	238	21433.00
tac	Y	40423	19228.00
cat	H	0	2.26
cac	H	4	1.74
---	--	------	------
mPD		0.012	0.50
		nPD:	0.02
		N. weight:	0.79
		Sc. PD:	-0.025
		Sc. rank:	-302.1

,

PA

Pos.	476	obs:	exp:
ttt	F	2	0.97
ttc	F	0	1.03
tct	S	0	0.16
tcc	S	0	0.15
tca	S	0	0.27
tcg	S	0	0.04
att	I	40240	14251.00
atc	I	149	10667.00
ata	I	208	15678.00
atg	M	29	29.00
act	T	3	0.93
acc	T	0	0.53
aca	T	0	1.30
acg	T	0	0.24
agt	S	1	0.17
agc	S	0	0.21
gtt	V	33	8.27
gtc	V	0	6.72
gta	V	0	7.15
gtg	V	0	10.86
---	--	------	------
mPD		0.021	0.66
		nPD:	0.03
		N. weight:	1.1
		Sc. PD:	-0.026
		Sc. rank:	6263.3

,

PA

Pos.	477	obs:	exp:
tct	S	0	0.97
tcc	S	0	0.90
tca	S	0	1.61
tcg	S	0	0.24
aat	N	16773	22747.00
aac	N	23886	17912.00
agt	S	5	1.05
agc	S	1	1.24
---	--	------	------
mPD		0.49	0.49
		nPD:	0.98
		N. weight:	0.048
		Sc. PD:	0.044
		Sc. rank:	1536.7

,

PA

Pos.	478	obs:	exp:
act	T	40396	12628.00
acc	T	45	7189.00
aca	T	23	17598.00
acg	T	199	3248.00
aat	N	2	1.12
aac	N	0	0.88
---	--	------	------
mPD		0.013	0.68
		nPD:	0.02
		N. weight:	1.3
		Sc. PD:	-0.045
		Sc. rank:	-6477.6

,

PA

Pos.	479	obs:	exp:
gct	A	289	11083.00
gcc	A	40231	10160.00
gca	A	144	16449.00
gcg	A	1	2973.00
---	--	------	------
mPD		0.021	0.69
		nPD:	0.03
		N. weight:	1.5
		Sc. PD:	-0.036
		Sc. rank:	7729.9

,

PA

Pos.	480	obs:	exp:
tta	L	1	4718.00
ttg	L	588	8406.00
ctt	L	25	8505.00
ctc	L	170	5962.00
cta	L	62	6243.00
ctg	L	39813	6824.00
atg	M	6	6.00
---	--	------	------
mPD		0.041	1.2
		nPD:	0.04
		N. weight:	1.8
		Sc. PD:	-0.036
		Sc. rank:	16165.0

,

PA

Pos.	481	obs:	exp:
tta	L	0	4719.00
ttg	L	0	8407.00
ctt	L	299	8506.00
ctc	L	40351	5963.00
cta	L	13	6244.00
ctg	L	0	6824.00
att	I	0	0.70
atc	I	2	0.53
ata	I	0	0.77
---	--	------	------
mPD		0.015	1.2
		nPD:	0.01
		N. weight:	2.1
		Sc. PD:	-0.086
		Sc. rank:	-25961.0

,

PA

Pos.	482	obs:	exp:
aat	N	40623	22751.00
aac	N	42	17914.00
---	--	------	------
mPD		0.0021	0.49
		nPD:	0.
		N. weight:	0.63
		Sc. PD:	-0.032
		Sc. rank:	-15450.0

,

PA

Pos.	483	obs:	exp:
tct	S	0	2.90
tcc	S	0	2.69
tca	S	18	4.84
tcg	S	0	0.71
agt	S	0	3.15
agc	S	0	3.71
gct	A	7	11078.00
gcc	A	11	10155.00
gca	A	40599	16442.00
gcg	A	30	2971.00
---	--	------	------
mPD		0.0032	0.70
		nPD:	0.
		N. weight:	0.99
		Sc. PD:	-0.049
		Sc. rank:	-23196.0

,

PA

Pos.	484	obs:	exp:
tct	S	528	6561.00
tcc	S	40106	6076.00
tca	S	27	10929.00
tcg	S	0	1601.00
agt	S	0	7106.00
agc	S	0	8388.00
gct	A	0	1.09
gcc	A	4	1.00
gca	A	0	1.62
gcg	A	0	0.29
---	--	------	------
mPD		0.027	1.6
		nPD:	0.02
		N. weight:	2.
		Sc. PD:	-0.077
		Sc. rank:	-14977.0

,

PA

Pos.	485	obs:	exp:
tct	S	0	0.16
tcc	S	0	0.15
tca	S	0	0.27
tcg	S	0	0.04
tgt	C	40642	15891.00
tgc	C	21	24772.00
cgt	R	1	0.02
cgc	R	0	0.04
cga	R	0	0.09
cgg	R	0	0.09
agt	S	1	0.17
agc	S	0	0.21
aga	R	0	0.49
agg	R	0	0.28
---	--	------	------
mPD		0.0011	0.48
		nPD:	0.
		N. weight:	1.
		Sc. PD:	-0.054
		Sc. rank:	-29523.0

,

PA

Pos.	486	obs:	exp:
tct	S	0	0.32
tcc	S	0	0.30
tca	S	2	0.54
tcg	S	0	0.08
act	T	0	2.17
acc	T	0	1.24
aca	T	5	3.03
acg	T	2	0.56
agt	S	0	0.35
agc	S	0	0.41
gtt	V	0	0.25
gtc	V	0	0.20
gta	V	1	0.22
gtg	V	0	0.33
gct	A	201	11081.00
gcc	A	11	10157.00
gca	A	32431	16445.00
gcg	A	8012	2972.00
---	--	------	------
mPD		0.33	0.69
		nPD:	0.47
		N. weight:	0.79
		Sc. PD:	0.32
		Sc. rank:	24643.0

,

PA

Pos.	487	obs:	exp:
tct	S	0	0.32
tcc	S	1	0.30
tca	S	1	0.54
tcg	S	0	0.08
act	T	0	1.24
acc	T	0	0.71
aca	T	4	1.73
acg	T	0	0.32
agt	S	0	0.35
agc	S	0	0.41
gct	A	10	11082.00
gcc	A	203	10158.00
gca	A	40413	16447.00
gcg	A	33	2972.00
---	--	------	------
mPD		0.012	0.69
		nPD:	0.02
		N. weight:	0.96
		Sc. PD:	-0.036
		Sc. rank:	-6362.4

,

PA

Pos.	488	obs:	exp:
atg	M	40663	40663.00
aaa	K	0	0.66
aag	K	1	0.34
gtt	V	0	0.25
gtc	V	0	0.20
gta	V	0	0.22
gtg	V	1	0.33
---	--	------	------
mPD		0.000098	0.00016
		nPD:	0.6
		N. weight:	0.000059
		Sc. PD:	0.000031
		Sc. rank:	1.9

,

PA

Pos.	489	obs:	exp:
aat	N	1	1.68
aac	N	2	1.32
gat	D	494	22982.00
gac	D	40164	17676.00
gaa	E	4	2.36
gag	E	0	1.64
---	--	------	------
mPD		0.024	0.49
		nPD:	0.05
		N. weight:	0.85
		Sc. PD:	-0.0053
		Sc. rank:	12050.0

,

PA

Pos.	490	obs:	exp:
aat	N	5	2.80
aac	N	0	2.20
gat	D	40397	22978.00
gac	D	254	17673.00
gaa	E	6	5.31
gag	E	3	3.69
---	--	------	------
mPD		0.013	0.49
		nPD:	0.03
		N. weight:	0.59
		Sc. PD:	-0.017
		Sc. rank:	1347.1

,

PA

Pos.	491	obs:	exp:
ttt	F	34556	19779.00
ttc	F	6106	20883.00
tat	Y	3	1.58
tac	Y	0	1.42
---	--	------	------
mPD		0.26	0.50
		nPD:	0.51
		N. weight:	0.32
		Sc. PD:	0.14
		Sc. rank:	10021.0

,

PA

Pos.	492	obs:	exp:
caa	Q	40341	24858.00
cag	Q	324	15807.00
---	--	------	------
mPD		0.016	0.48
		nPD:	0.03
		N. weight:	0.5
		Sc. PD:	-0.011
		Sc. rank:	3733.5

,

PA

Pos.	493	obs:	exp:
tta	L	521	4719.00
ttg	L	2	8407.00
ctt	L	0	8507.00
ctc	L	7	5963.00
cta	L	39804	6244.00
ctg	L	331	6825.00
---	--	------	------
mPD		0.042	1.2
		nPD:	0.04
		N. weight:	1.9
		Sc. PD:	-0.037
		Sc. rank:	17882.0

,

PA

Pos.	494	obs:	exp:
tta	L	0	0.12
ttg	L	0	0.21
ctt	L	1	0.21
ctc	L	0	0.15
cta	L	0	0.15
ctg	L	0	0.17
att	I	38890	14275.00
atc	I	1772	10685.00
ata	I	2	15704.00
---	--	------	------
mPD		0.083	0.66
		nPD:	0.13
		N. weight:	0.97
		Sc. PD:	0.067
		Sc. rank:	24488.0

,

PA

Pos.	495	obs:	exp:
cct	P	253	13551.00
ccc	P	40094	7624.00
cca	P	318	13739.00
ccg	P	0	5751.00
---	--	------	------
mPD		0.028	0.72
		nPD:	0.04
		N. weight:	1.8
		Sc. PD:	-0.03
		Sc. rank:	18602.0

,

PA

Pos.	496	obs:	exp:
att	I	0	0.70
atc	I	0	0.53
ata	I	2	0.77
atg	M	40662	40662.00
gtt	V	0	0.25
gtc	V	0	0.20
gta	V	0	0.22
gtg	V	1	0.33
---	--	------	------
mPD		0.00015	0.00018
		nPD:	0.82
		N. weight:	0.000082
		Sc. PD:	0.000061
		Sc. rank:	2.6

,

PA

Pos.	497	obs:	exp:
tta	L	5	0.58
ttg	L	0	1.03
ctt	L	0	1.05
ctc	L	0	0.73
cta	L	0	0.77
ctg	L	0	0.84
att	I	1	14269.00
atc	I	4	10681.00
ata	I	40644	15699.00
atg	M	6	6.00
gtt	V	0	1.25
gtc	V	0	1.02
gta	V	5	1.08
gtg	V	0	1.65
---	--	------	------
mPD		0.0010	0.66
		nPD:	0.
		N. weight:	1.1
		Sc. PD:	-0.056
		Sc. rank:	-31427.0

,

PA

Pos.	498	obs:	exp:
tct	S	0	6560.00
tcc	S	0	6075.00
tca	S	0	10926.00
tcg	S	0	1600.00
act	T	0	0.62
acc	T	2	0.35
aca	T	0	0.87
acg	T	0	0.16
aat	N	0	6.71
aac	N	12	5.29
agt	S	138	7104.00
agc	S	40512	8386.00
ggt	G	0	0.13
ggc	G	1	0.14
gga	G	0	0.46
ggg	G	0	0.26
---	--	------	------
mPD		0.0075	1.6
		nPD:	0.
		N. weight:	1.7
		Sc. PD:	-0.086
		Sc. rank:	-40406.0

,

PA

Pos.	499	obs:	exp:
cgt	R	0	0.02
cgc	R	0	0.04
cga	R	0	0.09
cgg	R	0	0.09
aat	N	16	8.95
aac	N	0	7.05
aaa	K	426	26723.00
aag	K	40222	13925.00
aga	R	0	0.49
agg	R	1	0.28
---	--	------	------
mPD		0.022	0.45
		nPD:	0.05
		N. weight:	1.1
		Sc. PD:	-0.0089
		Sc. rank:	14907.0

,

PA

Pos.	500	obs:	exp:
tct	S	0	0.32
tcc	S	0	0.30
tca	S	0	0.54
tcg	S	0	0.08
tgt	C	390	15891.00
tgc	C	40273	24772.00
agt	S	0	0.35
agc	S	2	0.41
---	--	------	------
mPD		0.019	0.48
		nPD:	0.04
		N. weight:	0.49
		Sc. PD:	-0.0076
		Sc. rank:	5478.5

,

PA

Pos.	501	obs:	exp:
cgt	R	0	806.20
cgc	R	0	1463.00
cga	R	6	3710.00
cgg	R	0	3492.00
aaa	K	1	1.31
aag	K	1	0.69
aga	R	39610	19945.00
agg	R	1047	11247.00
---	--	------	------
mPD		0.051	0.89
		nPD:	0.06
		N. weight:	0.67
		Sc. PD:	0.00072
		Sc. rank:	11282.0

,

PA

Pos.	502	obs:	exp:
att	I	1	0.70
atc	I	1	0.53
ata	I	0	0.77
act	T	40325	12624.00
acc	T	290	7187.00
aca	T	16	17592.00
acg	T	18	3247.00
aat	N	12	6.71
aac	N	0	5.29
gct	A	2	0.55
gcc	A	0	0.50
gca	A	0	0.81
gcg	A	0	0.15
---	--	------	------
mPD		0.017	0.68
		nPD:	0.02
		N. weight:	1.2
		Sc. PD:	-0.038
		Sc. rank:	394.7

,

PA

Pos.	503	obs:	exp:
cgt	R	0	6.58
cgc	R	0	11.95
cga	R	0	30.29
cgg	R	0	28.51
aat	N	1	0.56
aac	N	0	0.44
aaa	K	40206	26513.00
aag	K	122	13815.00
aga	R	332	162.80
agg	R	0	91.83
gaa	E	4	2.36
gag	E	0	1.64
---	--	------	------
mPD		0.022	0.47
		nPD:	0.05
		N. weight:	0.45
		Sc. PD:	-0.0037
		Sc. rank:	5892.3

,

PA

Pos.	504	obs:	exp:
gct	A	0	1.64
gcc	A	0	1.50
gca	A	0	2.43
gcg	A	6	0.44
gaa	E	521	23969.00
gag	E	40135	16687.00
ggt	G	0	0.40
ggc	G	0	0.42
gga	G	0	1.38
ggg	G	3	0.79
---	--	------	------
mPD		0.026	0.48
		nPD:	0.05
		N. weight:	0.9
		Sc. PD:	-0.0025
		Sc. rank:	14088.0

,

PA

Pos.	505	obs:	exp:
gtt	V	0	0.25
gtc	V	0	0.20
gta	V	1	0.22
gtg	V	0	0.33
gct	A	0	0.27
gcc	A	0	0.25
gca	A	1	0.40
gcg	A	0	0.07
gaa	E	1	0.59
gag	E	0	0.41
ggt	G	6	5474.00
ggc	G	3	5675.00
gga	G	40541	18770.00
ggg	G	112	10743.00
---	--	------	------
mPD		0.0061	0.68
		nPD:	0.01
		N. weight:	0.83
		Sc. PD:	-0.038
		Sc. rank:	-14456.0

,

PA

Pos.	506	obs:	exp:
cgt	R	0	806.10
cgc	R	0	1463.00
cga	R	0	3709.00
cgg	R	89	3491.00
atg	M	5	5.00
aaa	K	0	1.31
aag	K	2	0.69
aga	R	959	19941.00
agg	R	39607	11245.00
ggt	G	0	0.40
ggc	G	0	0.42
gga	G	0	1.38
ggg	G	3	0.79
---	--	------	------
mPD		0.051	0.89
		nPD:	0.06
		N. weight:	1.3
		Sc. PD:	0.0018
		Sc. rank:	21543.0

,

PA

Pos.	507	obs:	exp:
caa	Q	1	0.61
cag	Q	0	0.39
cgt	R	2	806.30
cgc	R	1	1463.00
cga	R	40456	3710.00
cgg	R	147	3492.00
aga	R	58	19945.00
agg	R	0	11248.00
---	--	------	------
mPD		0.010	0.89
		nPD:	0.01
		N. weight:	2.6
		Sc. PD:	-0.11
		Sc. rank:	-37920.0

,

PA

Pos.	508	obs:	exp:
caa	Q	1	0.61
cag	Q	0	0.39
cgt	R	0	3.79
cgc	R	0	6.87
cga	R	0	17.43
cgg	R	0	16.40
aaa	K	40386	26607.00
aag	K	85	13864.00
aga	R	180	93.68
agg	R	11	52.83
gaa	E	2	1.18
gag	E	0	0.82
---	--	------	------
mPD		0.014	0.46
		nPD:	0.03
		N. weight:	0.45
		Sc. PD:	-0.011
		Sc. rank:	2455.7

PA

Pos.	509	obs:	exp:
att	I	0	1.05
atc	I	3	0.79
ata	I	0	1.16
act	T	63	12628.00
acc	T	40303	7189.00
aca	T	296	17598.00
acg	T	0	3248.00
---	--	------	------
mPD		0.018	0.68
		nPD:	0.03
		N. weight:	1.8
		Sc. PD:	-0.054
		Sc. rank:	3061.2

PA

Pos.	510	obs:	exp:
tct	S	0	0.48
tcc	S	0	0.45
tca	S	0	0.81
tcg	S	0	0.12
aat	N	40563	22749.00
aac	N	99	17913.00
agt	S	3	0.52
agc	S	0	0.62
---	--	------	------
mPD		0.0050	0.49
		nPD:	0.01
		N. weight:	0.62
		Sc. PD:	-0.028
		Sc. rank:	-10232.0

PA

Pos.	511	obs:	exp:
tta	L	40263	4719.00
ttg	L	164	8407.00
ctt	L	0	8506.00
ctc	L	0	5963.00
cta	L	37	6244.00
ctg	L	200	6825.00
aaa	K	1	0.66
aag	K	0	0.34
---	--	------	------
mPD		0.029	1.2
		nPD:	0.03
		N. weight:	2.3
		Sc. PD:	-0.069
		Sc. rank:	3051.3

PA

Pos.	512	obs:	exp:
tat	Y	40443	21435.00
tac	Y	222	19230.00
---	--	------	------
mPD		0.011	0.50
		nPD:	0.02
		N. weight:	0.67
		Sc. PD:	-0.022
		Sc. rank:	-1667.8

PA

Pos.	513	obs:	exp:
ggt	G	15	5475.00
ggc	G	215	5676.00
gga	G	39607	18772.00
ggg	G	828	10743.00
---	--	------	------
mPD		0.051	0.68
		nPD:	0.07
		N. weight:	0.73
		Sc. PD:	0.013
		Sc. rank:	14819.0

PA

Pos.	514	obs:	exp:
ttt	F	81	19780.00
ttc	F	40584	20885.00
---	--	------	------
mPD		0.0040	0.50
		nPD:	0.01
		N. weight:	0.72
		Sc. PD:	-0.034
		Sc. rank:	-13158.0

PA

Pos.	515	obs:	exp:
tta	L	0	0.70
ttg	L	0	1.24
ctt	L	0	1.26
ctc	L	6	0.88
cta	L	0	0.92
ctg	L	0	1.01
att	I	359	14252.00
atc	I	40105	10668.00
ata	I	134	15679.00
gtt	V	0	15.28
gtc	V	61	12.43
gta	V	0	13.22
gtg	V	0	20.07
---	--	------	------
mPD		0.027	0.66
		nPD:	0.04
		N. weight:	1.4
		Sc. PD:	-0.02
		Sc. rank:	15898.0

PA

Pos.	516	obs:	exp:
att	I	5	14266.00
atc	I	1	10679.00
ata	I	40634	15695.00
gtt	V	0	6.26
gtc	V	0	5.09
gta	V	25	5.42
gtg	V	0	8.23
---	--	------	------
mPD		0.0015	0.66
		nPD:	0.
		N. weight:	1.1
		Sc. PD:	-0.055
		Sc. rank:	-30153.0

PA

Pos.	517	obs:	exp:
aat	N	1	0.56
aac	N	0	0.44
aaa	K	822	26734.00
aag	K	39842	13930.00
---	--	------	------
mPD		0.040	0.45
		nPD:	0.09
		N. weight:	1.1
		Sc. PD:	0.033
		Sc. rank:	23693.0

PA

Pos.	518	obs:	exp:
ggt	G	3	5475.00
ggc	G	20	5676.00
gga	G	40391	18772.00
ggg	G	251	10743.00
---	--	------	------
mPD		0.013	0.68
		nPD:	0.02
		N. weight:	0.81
		Sc. PD:	-0.029
		Sc. rank:	-3732.3

PA

Pos.	519	obs:	exp:
cgt	R	0	806.10
cgc	R	0	1463.00
cga	R	11	3710.00
cgg	R	0	3491.00
aaa	K	8	5.26
aag	K	0	2.74
aga	R	40336	19942.00
agg	R	310	11246.00
---	--	------	------
mPD		0.016	0.89
		nPD:	0.02
		N. weight:	0.74
		Sc. PD:	-0.027
		Sc. rank:	-4581.0

PA

Pos.	520	obs:	exp:
tct	S	40267	6562.00
tcc	S	375	6077.00
tca	S	22	10930.00
tcg	S	1	1601.00
agt	S	0	7106.00
agc	S	0	8389.00
---	--	------	------
mPD		0.019	1.6
		nPD:	0.01
		N. weight:	2.
		Sc. PD:	-0.084
		Sc. rank:	-27396.0

PA

Pos.	521	obs:	exp:
cat	H	38321	22937.00
cac	H	2344	17728.00
---	--	------	------
mPD		0.11	0.49
		nPD:	0.22
		N. weight:	0.41
		Sc. PD:	0.065
		Sc. rank:	11426.0

PA

Pos.	522	obs:	exp:
tta	L	2820	4719.00
ttg	L	2068	8407.00
ctt	L	40	8507.00
ctc	L	0	5963.00
cta	L	518	6244.00
ctg	L	35219	6825.00
---	--	------	------
mPD		0.36	1.2
		nPD:	0.31
		N. weight:	1.4
		Sc. PD:	0.35
		Sc. rank:	41892.0

PA

Pos.	523	obs:	exp:
cgt	R	0	806.30
cgc	R	0	1463.00
cga	R	1	3710.00
cgg	R	119	3492.00
aga	R	467	19946.00
agg	R	40078	11248.00
---	--	------	------
mPD		0.029	0.89
		nPD:	0.03
		N. weight:	1.3
		Sc. PD:	-0.031
		Sc. rank:	8989.3

PA

Pos.	524	obs:	exp:
tct	S	0	0.81
tcc	S	0	0.75
tca	S	0	1.34
tcg	S	0	0.20
aat	N	40634	22747.00
aac	N	25	17912.00
agt	S	5	0.87
agc	S	0	1.03
gat	D	1	0.57
gac	D	0	0.43
---	--	------	------
mPD		0.0015	0.49
		nPD:	0.
		N. weight:	0.64
		Sc. PD:	-0.033
		Sc. rank:	-16962.0

PA

Pos.	525	obs:	exp:
aat	N	0	1.12
aac	N	2	0.88
gat	D	316	22984.00
gac	D	40345	17677.00
gaa	E	2	1.18
gag	E	0	0.82
---	--	------	------
mPD		0.016	0.49
		nPD:	0.03
		N. weight:	0.87
		Sc. PD:	-0.02
		Sc. rank:	5302.1

PA

Pos.	526	obs:	exp:
act	T	217	12629.00
acc	T	97	7190.00
aca	T	40287	17599.00
acg	T	64	3248.00
---	--	------	------
mPD		0.018	0.68
		nPD:	0.03
		N. weight:	0.87
		Sc. PD:	-0.024
		Sc. rank:	2484.2

PA

Pos.	527	obs:	exp:
aat	N	0	1.12
aac	N	2	0.88
gat	D	3217	22985.00
gac	D	37446	17678.00
---	--	------	------
mPD		0.15	0.49
		nPD:	0.3
		N. weight:	0.59
		Sc. PD:	0.14
		Sc. rank:	17260.0

PA			
Pos.	528	obs:	exp:
gtt	V	46	10186.00
gtc	V	0	8286.00
gta	V	1577	8814.00
gtg	V	39042	13380.00
---	--	------	------
mPD		0.077	0.74
		nPD:	0.1
		N. weight:	1.1
		Sc. PD:	0.049
		Sc. rank:	25112.0

,

PA			
Pos.	529	obs:	exp:
att	I	0	1.05
atc	I	0	0.79
ata	I	3	1.16
gtt	V	6	10184.00
gtc	V	2	8285.00
gta	V	40317	8813.00
gtg	V	335	13378.00
gct	A	0	0.55
gcc	A	0	0.50
gca	A	2	0.81
gcg	A	0	0.15
---	--	------	------
mPD		0.017	0.74
		nPD:	0.02
		N. weight:	1.6
		Sc. PD:	-0.052
		Sc. rank:	-1752.6

,

PA			
Pos.	530	obs:	exp:
tct	S	0	0.65
tcc	S	0	0.60
tca	S	0	1.08
tcg	S	0	0.16
act	T	0	0.31
acc	T	1	0.18
aca	T	0	0.43
acg	T	0	0.08
aat	N	5513	22748.00
aac	N	35147	17912.00
agt	S	2	0.70
agc	S	2	0.83
---	--	------	------
mPD		0.23	0.49
		nPD:	0.48
		N. weight:	0.43
		Sc. PD:	0.18
		Sc. rank:	13468.0

,

PA			
Pos.	531	obs:	exp:
ttt	F	40612	19775.00
ttc	F	41	20878.00
tta	L	0	0.12
ttg	L	1	0.21
tat	Y	10	5.27
tac	Y	0	4.73
ctt	L	0	0.21
ctc	L	0	0.15
cta	L	0	0.15
ctg	L	0	0.17
att	I	1	0.35
atc	I	0	0.26
ata	I	0	0.39
---	--	------	------
mPD		0.0026	0.50
		nPD:	0.01
		N. weight:	0.79
		Sc. PD:	-0.039
		Sc. rank:	-17824.0

,

PA			
Pos.	532	obs:	exp:
tta	L	0	0.93
ttg	L	8	1.65
ctt	L	0	1.67
ctc	L	0	1.17
cta	L	0	1.23
ctg	L	0	1.34
atg	M	7	7.00
gtt	V	25	10182.00
gtc	V	0	8283.00
gta	V	319	8811.00
gtg	V	40306	13375.00
---	--	------	------
mPD		0.018	0.74
		nPD:	0.02
		N. weight:	1.2
		Sc. PD:	-0.037
		Sc. rank:	-532.1

,

PA			
Pos.	533	obs:	exp:
tct	S	0	6562.00
tcc	S	0	6076.00
tca	S	0	10929.00
tcg	S	0	1601.00
act	T	0	0.62
acc	T	2	0.35
aca	T	0	0.87
acg	T	0	0.16
agt	S	63	7106.00
agc	S	40599	8388.00
ggt	G	0	0.13
ggc	G	1	0.14
gga	G	0	0.46
ggg	G	0	0.26
---	--	------	------
mPD		0.0032	1.6
		nPD:	0.
		N. weight:	1.7
		Sc. PD:	-0.091
		Sc. rank:	-50580.0

,

PA			
Pos.	534	obs:	exp:
tta	L	0	1.16
ttg	L	4	2.07
ctt	L	0	2.09
ctc	L	0	1.47
cta	L	0	1.54
ctg	L	6	1.68
atg	M	40655	40655.00
---	--	------	------
mPD		0.00049	0.00080
		nPD:	0.62
		N. weight:	0.00032
		Sc. PD:	0.00017
		Sc. rank:	10.0

,

PA			
Pos.	535	obs:	exp:
gaa	E	2142	23975.00
gag	E	38523	16690.00
---	--	------	------
mPD		0.10	0.48
		nPD:	0.21
		N. weight:	0.74
		Sc. PD:	0.11
		Sc. rank:	20618.0

,

PA			
Pos.	536	obs:	exp:
ttt	F	39418	19780.00
ttc	F	1247	20885.00
---	--	------	------
mPD		0.059	0.50
		nPD:	0.12
		N. weight:	0.64
		Sc. PD:	0.039
		Sc. rank:	15911.0

,

PA			
Pos.	537	obs:	exp:
tct	S	40227	6562.00
tcc	S	406	6077.00
tca	S	31	10930.00
tcg	S	1	1601.00
agt	S	0	7106.00
agc	S	0	8389.00
---	--	------	------
mPD		0.021	1.6
		nPD:	0.01
		N. weight:	2.
		Sc. PD:	-0.081
		Sc. rank:	-24996.0

,

PA			
Pos.	538	obs:	exp:
tta	L	2	4719.00
ttg	L	0	8407.00
ctt	L	241	8507.00
ctc	L	39376	5963.00
cta	L	1044	6244.00
ctg	L	2	6825.00
---	--	------	------
mPD		0.062	1.2
		nPD:	0.05
		N. weight:	1.9
		Sc. PD:	-0.0051
		Sc. rank:	30655.0

,

PA			
Pos.	539	obs:	exp:
act	T	3028	12628.00
acc	T	17	7189.00
aca	T	37546	17598.00
acg	T	73	3248.00
aat	N	1	0.56
aac	N	0	0.44
---	--	------	------
mPD		0.14	0.68
		nPD:	0.21
		N. weight:	0.65
		Sc. PD:	0.096
		Sc. rank:	18143.0

,

PA			
Pos.	540	obs:	exp:
aat	N	8	5.59
aac	N	2	4.41
gat	D	35078	22981.00
gac	D	5577	17674.00
---	--	------	------
mPD		0.24	0.49
		nPD:	0.48
		N. weight:	0.23
		Sc. PD:	0.095
		Sc. rank:	7132.8

,

PA			
Pos.	541	obs:	exp:
cct	P	17	13551.00
ccc	P	0	7624.00
cca	P	1559	13739.00
ccg	P	39089	5751.00
---	--	------	------
mPD		0.075	0.72
		nPD:	0.1
		N. weight:	1.9
		Sc. PD:	0.09
		Sc. rank:	45973.0

,

PA			
Pos.	542	obs:	exp:
cgt	R	0	805.80
cgc	R	0	1462.00
cga	R	0	3708.00
cgg	R	0	3490.00
att	I	0	0.70
atc	I	0	0.53
ata	I	2	0.77
aaa	K	22	14.46
aag	K	0	7.54
aga	R	40294	19934.00
agg	R	347	11241.00
---	--	------	------
mPD		0.018	0.89
		nPD:	0.02
		N. weight:	0.74
		Sc. PD:	-0.026
		Sc. rank:	-2925.2

,

PA			
Pos.	543	obs:	exp:
ttt	F	6	3.40
ttc	F	1	3.60
tta	L	0	4716.00
ttg	L	0	8403.00
ctt	L	37768	8502.00
ctc	L	2203	5960.00
cta	L	86	6241.00
ctg	L	586	6821.00
cgt	R	1	0.02
cgc	R	0	0.04
cga	R	0	0.09
cgg	R	0	0.09
att	I	11	4.56
atc	I	2	3.42
ata	I	0	5.02
aga	R	0	0.49
agg	R	0	0.28
gtt	V	1	0.25
gtc	V	0	0.20
gta	V	0	0.22
gtg	V	0	0.33
---	--	------	------
mPD		0.13	1.2
		nPD:	0.12
		N. weight:	1.4
		Sc. PD:	0.083
		Sc. rank:	34986.0

,

PA

Pos.	544	obs:	exp:
gaa	E	33441	23973.00
gag	E	7222	16690.00
ggt	G	0	0.27
ggc	G	0	0.28
gga	G	2	0.92
ggg	G	0	0.53
---	--	------	------
mPD		0.29	0.48
		nPD:	0.6
		N. weight:	0.14
		Sc. PD:	0.074
		Sc. rank:	4339.8

PA

Pos.	545	obs:	exp:
tta	L	0	1.39
ttg	L	0	2.48
tct	S	0	1.94
tcc	S	0	1.79
tca	S	12	3.23
tcg	S	0	0.47
ctt	L	0	2.51
ctc	L	0	1.76
cta	L	12	1.84
ctg	L	0	2.01
cct	P	3	13537.00
ccc	P	4	7616.00
cca	P	39364	13725.00
ccg	P	1252	5746.00
caa	Q	4	2.45
cag	Q	0	1.55
cgt	R	0	0.02
cgc	R	0	0.04
cga	R	1	0.09
cgg	R	0	0.09
act	T	0	4.04
acc	T	0	2.30
aca	T	13	5.63
acg	T	0	1.04
agt	S	0	2.10
agc	S	0	2.48
aga	R	0	0.49
agg	R	0	0.28
---	--	------	------
mPD		0.062	0.72
		nPD:	0.09
		N. weight:	1.1
		Sc. PD:	0.031
		Sc. rank:	23524.0

PA

Pos.	546	obs:	exp:
tta	L	0	0.12
ttg	L	0	0.21
tat	Y	57	31.10
tac	Y	2	27.90
ctt	L	1	0.21
ctc	L	0	0.15
cta	L	0	0.15
ctg	L	0	0.17
cat	H	40223	22896.00
cac	H	370	17697.00
caa	Q	1	3.06
cag	Q	4	1.94
aat	N	6	3.92
aac	N	1	3.08
---	--	------	------
mPD		0.022	0.50
		nPD:	0.04
		N. weight:	0.58
		Sc. PD:	-0.0068
		Sc. rank:	7155.0

PA

Pos.	547	obs:	exp:
aaa	K	40576	26735.00
aag	K	89	13930.00
---	--	------	------
mPD		0.0044	0.45
		nPD:	0.01
		N. weight:	0.45
		Sc. PD:	-0.02
		Sc. rank:	-7548.6

PA

Pos.	548	obs:	exp:
tgt	C	1	0.39
tgc	C	0	0.61
tgg	W	40664	40664.00
---	--	------	------
mPD		0.000049	0.000049
		nPD:	1.
		N. weight:	0.000026
		Sc. PD:	0.000023
		Sc. rank:	0.8

PA

Pos.	549	obs:	exp:
aaa	K	1	9.20
aag	K	13	4.80
gtt	V	0	1.25
gtc	V	0	1.02
gta	V	0	1.08
gtg	V	5	1.65
gct	A	0	0.27
gcc	A	0	0.25
gca	A	0	0.40
gcg	A	1	0.07
gat	D	8	5.09
gac	D	1	3.91
gaa	E	334	23956.00
gag	E	40300	16678.00
ggt	G	0	0.27
ggc	G	0	0.28
gga	G	2	0.92
ggg	G	0	0.53
---	--	------	------
mPD		0.018	0.49
		nPD:	0.04
		N. weight:	0.93
		Sc. PD:	-0.017
		Sc. rank:	8683.6

PA

Pos.	550	obs:	exp:
tgg	W	1	1.00
cat	H	1	0.56
cac	H	0	0.44
cgt	R	0	0.38
cgc	R	0	0.68
cga	R	0	1.73
cgg	R	0	1.63
aaa	K	40373	26721.00
aag	K	271	13923.00
aga	R	19	9.32
agg	R	0	5.26
---	--	------	------
mPD		0.014	0.45
		nPD:	0.03
		N. weight:	0.42
		Sc. PD:	-0.0099
		Sc. rank:	2681.5

PA

Pos.	551	obs:	exp:
tct	S	0	0.16
tcc	S	0	0.15
tca	S	0	0.27
tcg	S	0	0.04
tat	Y	32603	21432.00
tac	Y	8057	19228.00
tgt	C	0	0.39
tgc	C	1	0.61
cat	H	0	1.69
cac	H	3	1.31
agt	S	0	0.17
agc	S	1	0.21
---	--	------	------
mPD		0.32	0.50
		nPD:	0.64
		N. weight:	0.18
		Sc. PD:	0.1
		Sc. rank:	5737.2

PA

Pos.	552	obs:	exp:
tct	S	0	0.16
tcc	S	0	0.15
tca	S	0	0.27
tcg	S	0	0.04
tgt	C	40565	15891.00
tgc	C	98	24772.00
tgg	W	1	1.00
agt	S	1	0.17
agc	S	0	0.21
---	--	------	------
mPD		0.0049	0.48
		nPD:	0.01
		N. weight:	1.
		Sc. PD:	-0.045
		Sc. rank:	-16569.0

PA

Pos.	553	obs:	exp:
att	I	0	3.51
atc	I	9	2.63
ata	I	1	3.86
aaa	K	1	0.66
aag	K	0	0.34
gtt	V	401	10182.00
gtc	V	40245	8283.00
gta	V	5	8811.00
gtg	V	1	13375.00
gct	A	0	0.55
gcc	A	2	0.50
gca	A	0	0.81
gcg	A	0	0.15
---	--	------	------
mPD		0.021	0.74
		nPD:	0.03
		N. weight:	1.7
		Sc. PD:	-0.046
		Sc. rank:	5978.3

PA

Pos.	554	obs:	exp:
tta	L	0	4706.00
ttg	L	0	8384.00
ctt	L	40510	8483.00
ctc	L	33	5946.00
cta	L	4	6227.00
ctg	L	4	6806.00
att	I	113	39.67
atc	I	0	29.69
ata	I	0	43.64
act	T	0	0.31
acc	T	1	0.18
aca	T	0	0.43
acg	T	0	0.08
---	--	------	------
mPD		0.0077	1.2
		nPD:	0.01
		N. weight:	1.7
		Sc. PD:	-0.082
		Sc. rank:	-35811.0

PA

Pos.	555	obs:	exp:
aaa	K	0	1.31
aag	K	2	0.69
gat	D	1	0.57
gac	D	0	0.43
gaa	E	670	23970.00
gag	E	39988	16688.00
ggt	G	0	0.54
ggc	G	0	0.56
gga	G	0	1.85
ggg	G	4	1.06
---	--	------	------
mPD		0.033	0.48
		nPD:	0.07
		N. weight:	0.89
		Sc. PD:	0.01
		Sc. rank:	16692.0

PA

Pos.	556	obs:	exp:
cgt	R	0	0.02
cgc	R	0	0.04
cga	R	0	0.09
cgg	R	0	0.09
att	I	16	14251.00
atc	I	0	10667.00
ata	I	40580	15678.00
act	T	0	0.31
acc	T	0	0.18
aca	T	1	0.43
acg	T	0	0.08
aga	R	1	0.49
agg	R	0	0.28
gtt	V	1	16.78
gtc	V	0	13.65
gta	V	66	14.52
gtg	V	0	22.04
---	--	------	------
mPD		0.0042	0.66
		nPD:	0.01
		N. weight:	1.1
		Sc. PD:	-0.051
		Sc. rank:	-22480.0

PA

Pos.	557	obs:	exp:
tct	S	1	0.16
tcc	S	0	0.15
tca	S	0	0.27
tcg	S	0	0.04
agt	S	0	0.17
agc	S	0	0.21
ggt	G	8	5474.00
ggc	G	7	5675.00
gga	G	40530	18771.00
ggg	G	119	10743.00
---	--	------	------
mPD		0.0067	0.68
		nPD:	0.01
		N. weight:	0.83
		Sc. PD:	-0.037
		Sc. rank:	-13808.0

PA

Pos.	558	obs:	exp:
act	T	0	0.31
acc	T	1	0.18
aca	T	0	0.43
acg	T	0	0.08
aat	N	7	4.48
aac	N	1	3.52
gtt	V	1	0.25
gtc	V	0	0.20
gta	V	0	0.22
gtg	V	0	0.33
gat	D	40208	22973.00
gac	D	433	17668.00
gaa	E	6	8.25
gag	E	8	5.75
---	--	------	------
mPD		0.022	0.49
		nPD:	0.05
		N. weight:	0.57
		Sc. PD:	-0.0058
		Sc. rank:	7241.7

PA

Pos.	559	obs:	exp:
tta	L	0	0.12
ttg	L	0	0.21
ctt	L	0	0.21
ctc	L	0	0.15
cta	L	0	0.15
ctg	L	1	0.17
att	I	1	10.88
atc	I	1	8.15
ata	I	29	11.97
atg	M	40627	40627.00
act	T	0	0.31
acc	T	0	0.18
aca	T	0	0.43
acg	T	1	0.08
gtt	V	0	1.00
gtc	V	0	0.82
gta	V	0	0.87
gtg	V	4	1.32
ggt	G	0	0.13
ggc	G	0	0.14
gga	G	1	0.46
ggg	G	0	0.26
---	--	------	------
mPD		0.0020	0.0022
		nPD:	0.91
		N. weight:	0.00089
		Sc. PD:	0.00074
		Sc. rank:	28.2

PA

Pos.	560	obs:	exp:
ttt	F	1	0.49
ttc	F	0	0.51
tta	L	37452	4717.00
ttg	L	2765	8404.00
tct	S	0	1.45
tcc	S	0	1.34
tca	S	9	2.42
tcg	S	0	0.35
ctt	L	7	8503.00
ctc	L	188	5961.00
cta	L	222	6242.00
ctg	L	15	6822.00
cct	P	0	0.33
ccc	P	0	0.19
cca	P	1	0.34
ccg	P	0	0.14
att	I	0	1.76
atc	I	0	1.31
ata	I	5	1.93
agt	S	0	1.57
agc	S	0	1.86
---	--	------	------
mPD		0.16	1.2
		nPD:	0.14
		N. weight:	2.
		Sc. PD:	0.15
		Sc. rank:	51639.0

PA

Pos.	561	obs:	exp:
tta	L	114	4709.00
ttg	L	26	8390.00
ctt	L	5	8490.00
ctc	L	1	5951.00
cta	L	40194	6232.00
ctg	L	243	6811.00
cgt	R	0	0.02
cgc	R	0	0.04
cga	R	0	0.09
cgg	R	0	0.09
att	I	0	27.03
atc	I	0	20.23
ata	I	77	29.74
atg	M	1	1.00
aga	R	1	0.49
agg	R	0	0.28
gtt	V	0	0.75
gtc	V	0	0.61
gta	V	3	0.65
gtg	V	0	0.99
---	--	------	------
mPD		0.024	1.2
		nPD:	0.02
		N. weight:	2.
		Sc. PD:	-0.068
		Sc. rank:	-7016.8

PA

Pos.	562	obs:	exp:
cgt	R	0	802.10
cgc	R	0	1456.00
cga	R	6	3691.00
cgg	R	1	3474.00
atg	M	1	1.00
aaa	K	45	136.70
aag	K	163	71.25
aga	R	14366	19843.00
agg	R	26082	11190.00
ggt	G	0	0.13
ggc	G	0	0.14
gga	G	1	0.46
ggg	G	0	0.26
---	--	------	------
mPD		0.47	0.89
		nPD:	0.52
		N. weight:	0.48
		Sc. PD:	0.22
		Sc. rank:	14891.0

PA

Pos.	563	obs:	exp:
tct	S	0	6523.00
tcc	S	0	6041.00
tca	S	0	10866.00
tcg	S	0	1591.00
tgt	C	2	0.78
tgc	C	0	1.22
cgt	R	2	0.04
cgc	R	0	0.07
cga	R	0	0.18
cgg	R	0	0.17
act	T	203	63.04
acc	T	0	35.89
aca	T	0	87.85
acg	T	0	16.21
aat	N	16	8.95
aac	N	0	7.05
agt	S	40409	7065.00
agc	S	17	8340.00
aga	R	0	0.98
agg	R	0	0.55
gct	A	1	0.27
gcc	A	0	0.25
gca	A	0	0.40
gcg	A	0	0.07
ggt	G	15	2.02
ggc	G	0	2.09
gga	G	0	6.92
ggg	G	0	3.96
---	--	------	------
mPD		0.013	1.6
		nPD:	0.01
		N. weight:	1.9
		Sc. PD:	-0.09
		Sc. rank:	-35998.0

PA

Pos.	564	obs:	exp:
tct	S	1	1.29
tcc	S	7	1.20
tca	S	0	2.15
tcg	S	0	0.31
act	T	1	3.42
acc	T	10	1.94
aca	T	0	4.76
acg	T	0	0.88
agt	S	0	1.40
agc	S	0	1.65
gtt	V	0	0.50
gtc	V	2	0.41
gta	V	0	0.43
gtg	V	0	0.66
gct	A	2776	11078.00
gcc	A	37626	10155.00
gca	A	215	16441.00
gcg	A	27	2971.00
---	--	------	------
mPD		0.14	0.70
		nPD:	0.2
		N. weight:	1.2
		Sc. PD:	0.17
		Sc. rank:	33780.0

PA

Pos.	565	obs:	exp:
tta	L	9	3.48
ttg	L	0	6.20
ctt	L	0	6.28
ctc	L	0	4.40
cta	L	21	4.61
ctg	L	0	5.03
att	I	9	14109.00
atc	I	114	10561.00
ata	I	40068	15522.00
atg	M	11	11.00
aaa	K	1	0.66
aag	K	0	0.34
gtt	V	0	108.20
gtc	V	1	88.02
gta	V	431	93.63
gtg	V	0	142.10
---	--	------	------
mPD		0.029	0.68
		nPD:	0.04
		N. weight:	1.
		Sc. PD:	-0.013
		Sc. rank:	12421.0

PA

Pos.	566	obs:	exp:
tct	S	0	0.65
tcc	S	0	0.60
tca	S	0	1.08
tcg	S	0	0.16
agt	S	1	0.70
agc	S	3	0.83
gat	D	0	0.57
gac	D	1	0.43
ggt	G	947	5474.00
ggc	G	39618	5675.00
gga	G	94	18769.00
ggg	G	1	10742.00
---	--	------	------
mPD		0.050	0.68
		nPD:	0.07
		N. weight:	2.
		Sc. PD:	0.036
		Sc. rank:	41117.0

PA

Pos.	567	obs:	exp:
tta	L	0	0.70
ttg	L	0	1.24
ctt	L	0	1.26
ctc	L	0	0.88
cta	L	6	0.92
ctg	L	0	1.01
cct	P	0	0.67
ccc	P	0	0.37
cca	P	2	0.68
ccg	P	0	0.28
cat	H	1	1.13
cac	H	1	0.87
caa	Q	40437	24849.00
cag	Q	214	15802.00
cgt	R	0	0.06
cgc	R	0	0.11
cga	R	2	0.27
cgg	R	1	0.26
aaa	K	1	0.66
aag	K	0	0.34
aga	R	0	1.47
agg	R	0	0.83
---	--	------	------
mPD		0.011	0.48
		nPD:	0.02
		N. weight:	0.51
		Sc. PD:	-0.016
		Sc. rank:	-283.5

PA			
Pos.	568	obs:	exp:
tta	L	0	0.23
ttg	L	0	0.41
tct	S	0	0.16
tcc	S	0	0.15
tca	S	0	0.27
tcg	S	0	0.04
ctt	L	1	0.42
ctc	L	1	0.29
cta	L	0	0.31
ctg	L	0	0.34
att	I	40069	14101.00
atc	I	97	10555.00
ata	I	2	15513.00
atg	M	249	249.00
act	T	8	2.48
acc	T	0	1.41
aca	T	0	3.46
acg	T	0	0.64
agt	S	1	0.17
agc	S	0	0.21
gtt	V	32	59.36
gtc	V	0	48.29
gta	V	1	51.37
gtg	V	204	77.98
---	--	------	------
mPD		0.039	0.68
		nPD:	0.06
		N. weight:	1.1
		Sc. PD:	0.0019
		Sc. rank:	19444.0

,

PA			
Pos.	569	obs:	exp:
tta	L	3	0.46
ttg	L	1	0.83
tct	S	23	6560.00
tcc	S	0	6075.00
tca	S	40421	10927.00
tcg	S	210	1600.00
ctt	L	0	0.84
ctc	L	0	0.59
cta	L	0	0.61
ctg	L	0	0.67
act	T	0	1.86
acc	T	0	1.06
aca	T	6	2.60
acg	T	0	0.48
agt	S	0	7104.00
agc	S	0	8387.00
gct	A	0	0.27
gcc	A	0	0.25
gca	A	1	0.40
gcg	A	0	0.07
---	--	------	------
mPD		0.012	1.6
		nPD:	0.01
		N. weight:	1.4
		Sc. PD:	-0.067
		Sc. rank:	-27519.0

,

PA			
Pos.	570	obs:	exp:
cgt	R	0	806.10
cgc	R	0	1463.00
cga	R	0	3709.00
cgg	R	9	3491.00
aaa	K	0	5.92
aag	K	9	3.08
aga	R	283	19941.00
agg	R	40364	11245.00
---	--	------	------
mPD		0.015	0.89
		nPD:	0.02
		N. weight:	1.4
		Sc. PD:	-0.052
		Sc. rank:	-10745.0

,

PA			
Pos.	571	obs:	exp:
tct	S	1	0.81
tcc	S	0	0.75
tca	S	0	1.34
tcg	S	4	0.20
cct	P	917	13549.00
ccc	P	220	7622.00
cca	P	1092	13736.00
ccg	P	38429	5751.00
caa	Q	0	1.22
cag	Q	2	0.78
agt	S	0	0.87
agc	S	0	1.03
---	--	------	------
mPD		0.11	0.72
		nPD:	0.15
		N. weight:	1.8
		Sc. PD:	0.16
		Sc. rank:	48065.0

,

PA			
Pos.	572	obs:	exp:
tta	L	0	0.35
ttg	L	3	0.62
ctt	L	0	0.63
ctc	L	0	0.44
cta	L	0	0.46
ctg	L	0	0.50
att	I	23	13.34
atc	I	0	9.98
ata	I	15	14.68
atg	M	40612	40612.00
gtt	V	0	3.01
gtc	V	0	2.45
gta	V	0	2.60
gtg	V	12	3.95
---	--	------	------
mPD		0.0026	0.0031
		nPD:	0.84
		N. weight:	0.0009
		Sc. PD:	0.00068
		Sc. rank:	28.5

,

PA			
Pos.	573	obs:	exp:
ttt	F	89	19777.00
ttc	F	40569	20881.00
tat	Y	0	3.69
tac	Y	7	3.31
---	--	------	------
mPD		0.0047	0.50
		nPD:	0.01
		N. weight:	0.72
		Sc. PD:	-0.033
		Sc. rank:	-12329.0

,

PA			
Pos.	574	obs:	exp:
tta	L	401	4719.00
ttg	L	39599	8407.00
ctt	L	1	8507.00
ctc	L	0	5963.00
cta	L	203	6244.00
ctg	L	461	6825.00
---	--	------	------
mPD		0.061	1.2
		nPD:	0.05
		N. weight:	1.6
		Sc. PD:	-0.0046
		Sc. rank:	24415.0

,

PA			
Pos.	575	obs:	exp:
ttt	F	1	0.49
ttc	F	0	0.51
tat	Y	40542	21434.00
tac	Y	122	19230.00
---	--	------	------
mPD		0.0060	0.50
		nPD:	0.01
		N. weight:	0.69
		Sc. PD:	-0.029
		Sc. rank:	-9493.6

,

PA			
Pos.	576	obs:	exp:
tta	L	0	0.12
ttg	L	1	0.21
tct	S	0	0.16
tcc	S	0	0.15
tca	S	0	0.27
tcg	S	1	0.04
ctt	L	0	0.21
ctc	L	0	0.15
cta	L	0	0.15
ctg	L	0	0.17
atg	M	16	16.00
agt	S	0	0.17
agc	S	0	0.21
gtt	V	15	10181.00
gtc	V	0	8282.00
gta	V	223	8810.00
gtg	V	40409	13374.00
---	--	------	------
mPD		0.013	0.74
		nPD:	0.02
		N. weight:	1.2
		Sc. PD:	-0.045
		Sc. rank:	-8291.2

,

PA			
Pos.	577	obs:	exp:
cgt	R	0	806.30
cgc	R	0	1463.00
cga	R	0	3710.00
cgg	R	4	3492.00
aaa	K	0	0.66
aag	K	1	0.34
aga	R	1452	19945.00
agg	R	39208	11248.00
---	--	------	------
mPD		0.069	0.89
		nPD:	0.08
		N. weight:	1.2
		Sc. PD:	0.026
		Sc. rank:	25577.0

,

PA			
Pos.	578	obs:	exp:
att	I	0	0.35
atc	I	0	0.26
ata	I	1	0.39
act	T	16	12628.00
acc	T	216	7189.00
aca	T	40406	17598.00
acg	T	26	3248.00
---	--	------	------
mPD		0.013	0.68
		nPD:	0.02
		N. weight:	0.89
		Sc. PD:	-0.032
		Sc. rank:	-5099.8

,

PA			
Pos.	579	obs:	exp:
cat	H	0	0.56
cac	H	1	0.44
aat	N	944	22750.00
aac	N	39720	17914.00
---	--	------	------
mPD		0.045	0.49
		nPD:	0.09
		N. weight:	0.78
		Sc. PD:	0.027
		Sc. rank:	17629.0

,

PA

Pos.	580	obs:	exp:
ggt	G	10	5475.00
ggc	G	1	5676.00
gga	G	40434	18772.00
ggg	G	220	10743.00
---	--	------	------
mPD		0.011	0.68
		nPD:	0.02
		N. weight:	0.82
		Sc. PD:	-0.031
		Sc. rank:	-6220.4

,

PA

Pos.	581	obs:	exp:
tct	S	0	0.16
tcc	S	0	0.15
tca	S	1	0.27
tcg	S	0	0.04
att	I	0	1.76
atc	I	0	1.31
ata	I	5	1.93
act	T	13	12626.00
acc	T	211	7188.00
aca	T	40413	17595.00
acg	T	19	3247.00
agt	S	0	0.17
agc	S	0	0.21
gct	A	0	0.82
gcc	A	0	0.75
gca	A	3	1.21
gcg	A	0	0.22
---	--	------	------
mPD		0.012	0.68
		nPD:	0.02
		N. weight:	0.89
		Sc. PD:	-0.033
		Sc. rank:	-5427.5

,

PA

Pos.	582	obs:	exp:
tct	S	5	6562.00
tcc	S	203	6077.00
tca	S	40444	10930.00
tcg	S	13	1601.00
agt	S	0	7106.00
agc	S	0	8389.00
---	--	------	------
mPD		0.011	1.6
		nPD:	0.01
		N. weight:	1.4
		Sc. PD:	-0.068
		Sc. rank:	-29352.0

,

PA

Pos.	583	obs:	exp:
cgt	R	0	0.02
cgc	R	0	0.04
cga	R	0	0.09
cgg	R	0	0.09
aaa	K	25243	26734.00
aag	K	15421	13930.00
aga	R	1	0.49
agg	R	0	0.28
---	--	------	------
mPD		0.47	0.45
		nPD:	1.05
		N. weight:	0.0034
		Sc. PD:	0.0033
		Sc. rank:	108.6

,

PA

Pos.	584	obs:	exp:
ttt	F	0	0.49
ttc	F	1	0.51
att	I	66	220.80
atc	I	563	165.30
ata	I	0	242.90
gtt	V	140	10028.00
gtc	V	39828	8157.00
gta	V	67	8677.00
gtg	V	0	13172.00
---	--	------	------
mPD		0.044	0.77
		nPD:	0.06
		N. weight:	1.7
		Sc. PD:	0.0013
		Sc. rank:	28653.0

,

PA

Pos.	585	obs:	exp:
caa	Q	1	0.61
cag	Q	0	0.39
cgt	R	0	0.02
cgc	R	0	0.04
cga	R	0	0.09
cgg	R	0	0.09
aaa	K	40424	26733.00
aag	K	239	13930.00
aga	R	1	0.49
agg	R	0	0.28
---	--	------	------
mPD		0.012	0.45
		nPD:	0.03
		N. weight:	0.43
		Sc. PD:	-0.012
		Sc. rank:	725.6

,

PA

Pos.	586	obs:	exp:
tta	L	0	0.23
ttg	L	2	0.41
ctt	L	0	0.42
ctc	L	0	0.29
cta	L	0	0.31
ctg	L	0	0.34
atg	M	40663	40663.00
---	--	------	------
mPD		0.000098	0.00016
		nPD:	0.62
		N. weight:	0.000086
		Sc. PD:	0.000047
		Sc. rank:	2.7

,

PA

Pos.	587	obs:	exp:
cgt	R	0	0.14
cgc	R	0	0.25
cga	R	0	0.64
cgg	R	0	0.60
aaa	K	40551	26730.00
aag	K	107	13928.00
aga	R	7	3.43
agg	R	0	1.94
---	--	------	------
mPD		0.0056	0.45
		nPD:	0.01
		N. weight:	0.45
		Sc. PD:	-0.019
		Sc. rank:	-5924.2

,

PA

Pos.	588	obs:	exp:
tgg	W	40665	40665.00
---	--	------	------
mPD		0	0
		nPD:	1.
		N. weight:	0.
		Sc. PD:	0
		Sc. rank:	0

,

PA

Pos.	589	obs:	exp:
tct	S	0	0.97
tcc	S	0	0.90
tca	S	0	1.61
tcg	S	0	0.24
agt	S	0	1.05
agc	S	6	1.24
ggt	G	4	5474.00
ggc	G	150	5675.00
gga	G	40224	18769.00
ggg	G	281	10742.00
---	--	------	------
mPD		0.022	0.68
		nPD:	0.03
		N. weight:	0.79
		Sc. PD:	-0.018
		Sc. rank:	5200.6

,

PA

Pos.	590	obs:	exp:
cgt	R	0	0.02
cgc	R	0	0.04
cga	R	0	0.09
cgg	R	0	0.09
att	I	2	3.86
atc	I	0	2.89
ata	I	9	4.25
atg	M	40646	40646.00
aga	R	0	0.49
agg	R	1	0.28
gtt	V	0	1.75
gtc	V	0	1.43
gta	V	0	1.52
gtg	V	7	2.30
---	--	------	------
mPD		0.00093	0.0012
		nPD:	0.77
		N. weight:	0.00043
		Sc. PD:	0.0003
		Sc. rank:	13.6

,

PA

Pos.	591	obs:	exp:
gaa	E	333	23973.00
gag	E	40330	16690.00
ggt	G	0	0.27
ggc	G	0	0.28
gga	G	0	0.92
ggg	G	2	0.53
---	--	------	------
mPD		0.016	0.48
		nPD:	0.03
		N. weight:	0.93
		Sc. PD:	-0.02
		Sc. rank:	7437.2

,

PA

Pos.	592	obs:	exp:
att	I	0	0.35
atc	I	0	0.26
ata	I	1	0.39
atg	M	40657	40657.00
act	T	0	1.24
acc	T	0	0.71
aca	T	0	1.73
acg	T	4	0.32
aaa	K	0	0.66
aag	K	1	0.34
gtt	V	0	0.25
gtc	V	0	0.20
gta	V	0	0.22
gtg	V	1	0.33
gct	A	1	0.27
gcc	A	0	0.25
gca	A	0	0.40
gcg	A	0	0.07
---	--	------	------
mPD		0.00049	0.00073
		nPD:	0.67
		N. weight:	0.0004
		Sc. PD:	0.00024
		Sc. rank:	12.5

,

PA

Pos.	593	obs:	exp:
cgt	R	0	806.30
cgc	R	0	1463.00
cga	R	8	3710.00
cgg	R	0	3492.00
aga	R	40288	19946.00
agg	R	369	11248.00
---	--	------	------
mPD		0.018	0.89
		nPD:	0.02
		N. weight:	0.74
		Sc. PD:	-0.025
		Sc. rank:	-2826.2

,

PA

Pos.	594	obs:	exp:
tgt	C	1	0.39
tgc	C	0	0.61
cat	H	23	12.97
cac	H	0	10.03
cgt	R	40383	805.80
cgc	R	248	1462.00
cga	R	4	3708.00
cgg	R	5	3490.00
aga	R	1	19934.00
agg	R	0	11241.00
---	--	------	------
mPD		0.014	0.89
		nPD:	0.02
		N. weight:	4.3
		Sc. PD:	-0.17
		Sc. rank:	-40326.0

,

PA

Pos.	595	obs:	exp:
ttt	F	0	0.49
ttc	F	1	0.51
tgt	C	235	15890.00
tgc	C	40427	24772.00
cgt	R	0	0.04
cgc	R	2	0.07
cga	R	0	0.18
cgg	R	0	0.17
aga	R	0	0.98
agg	R	0	0.55
---	--	------	------
mPD		0.012	0.48
		nPD:	0.02
		N. weight:	0.51
		Sc. PD:	-0.016
		Sc. rank:	-19.1

,

PA

Pos.	596	obs:	exp:
ttt	F	0	0.49
ttc	F	1	0.51
tta	L	0	4719.00
ttg	L	0	8407.00
ctt	L	740	8506.00
ctc	L	39902	5963.00
cta	L	20	6244.00
ctg	L	1	6824.00
gtt	V	0	0.25
gtc	V	1	0.20
gta	V	0	0.22
gtg	V	0	0.33
---	--	------	------
mPD		0.037	1.2
		nPD:	0.03
		N. weight:	2.
		Sc. PD:	-0.047
		Sc. rank:	12623.0

,

PA

Pos.	597	obs:	exp:
tta	L	0	4718.00
ttg	L	0	8405.00
ctt	L	40276	8505.00
ctc	L	251	5962.00
cta	L	125	6243.00
ctg	L	3	6823.00
caa	Q	1	0.61
cag	Q	0	0.39
att	I	8	2.81
atc	I	0	2.10
ata	I	0	3.09
ggt	G	1	0.13
ggc	G	0	0.14
gga	G	0	0.46
ggg	G	0	0.26
---	--	------	------
mPD		0.019	1.2
		nPD:	0.02
		N. weight:	1.7
		Sc. PD:	-0.064
		Sc. rank:	-13401.0

,

PA

Pos.	598	obs:	exp:
caa	Q	1079	24858.00
cag	Q	39586	15807.00
---	--	------	------
mPD		0.052	0.48
		nPD:	0.11
		N. weight:	0.9
		Sc. PD:	0.046
		Sc. rank:	21608.0

,

PA

Pos.	599	obs:	exp:
tct	S	159	6560.00
tcc	S	51	6075.00
tca	S	40215	10926.00
tcg	S	225	1600.00
cct	P	0	4.67
ccc	P	0	2.62
cca	P	14	4.73
ccg	P	0	1.98
agt	S	0	7104.00
agc	S	0	8386.00
gct	A	0	0.27
gcc	A	0	0.25
gca	A	1	0.40
gcg	A	0	0.07
---	--	------	------
mPD		0.022	1.6
		nPD:	0.01
		N. weight:	1.4
		Sc. PD:	-0.057
		Sc. rank:	-16800.0

,

PA

Pos.	600	obs:	exp:
tta	L	14	4717.00
ttg	L	0	8405.00
ctt	L	263	8504.00
ctc	L	40251	5961.00
cta	L	125	6243.00
ctg	L	0	6823.00
att	I	0	3.86
atc	I	11	2.89
ata	I	0	4.25
atg	M	1	1.00
---	--	------	------
mPD		0.021	1.2
		nPD:	0.02
		N. weight:	2.1
		Sc. PD:	-0.076
		Sc. rank:	-12812.0

,

PA

Pos.	601	obs:	exp:
cat	H	4	2.26
cac	H	0	1.74
caa	Q	1051	24855.00
cag	Q	39610	15806.00
---	--	------	------
mPD		0.051	0.48
		nPD:	0.11
		N. weight:	0.9
		Sc. PD:	0.044
		Sc. rank:	21510.0

,

PA

Pos.	602	obs:	exp:
caa	Q	367	24858.00
cag	Q	40298	15807.00
---	--	------	------
mPD		0.018	0.48
		nPD:	0.04
		N. weight:	0.98
		Sc. PD:	-0.018
		Sc. rank:	9553.5

,

PA

Pos.	603	obs:	exp:
att	I	4495	14272.00
atc	I	36094	10683.00
ata	I	68	15702.00
atg	M	1	1.00
act	T	0	0.31
acc	T	1	0.18
aca	T	0	0.43
acg	T	0	0.08
gtt	V	2	1.50
gtc	V	4	1.22
gta	V	0	1.30
gtg	V	0	1.97
---	--	------	------
mPD		0.20	0.66
		nPD:	0.3
		N. weight:	1.
		Sc. PD:	0.25
		Sc. rank:	30700.0

,

PA

Pos.	604	obs:	exp:
gaa	E	115	23975.00
gag	E	40550	16690.00
---	--	------	------
mPD		0.0056	0.48
		nPD:	0.01
		N. weight:	0.96
		Sc. PD:	-0.041
		Sc. rank:	-13652.0

,

PA

Pos.	605	obs:	exp:
tct	S	0	6559.00
tcc	S	0	6074.00
tca	S	0	10924.00
tcg	S	0	1600.00
cgt	R	0	0.02
cgc	R	0	0.04
cga	R	0	0.09
cgg	R	0	0.09
aat	N	0	6.71
aac	N	12	5.29
agt	S	424	7103.00
agc	S	40220	8385.00
aga	R	1	0.49
agg	R	0	0.28
ggt	G	0	1.08
ggc	G	8	1.12
gga	G	0	3.69
ggg	G	0	2.11
---	--	------	------
mPD		0.022	1.6
		nPD:	0.01
		N. weight:	1.7
		Sc. PD:	-0.07
		Sc. rank:	-20875.0

,

PA

Pos.	606	obs:	exp:
tta	L	0	0.58
ttg	L	4	1.03
ctt	L	0	1.05
ctc	L	0	0.73
cta	L	0	0.77
ctg	L	1	0.84
att	I	3	43.18
atc	I	0	32.32
ata	I	120	47.50
atg	M	40534	40534.00
aat	N	2	1.12
aac	N	0	0.88
gtt	V	0	0.25
gtc	V	0	0.20
gta	V	0	0.22
gtg	V	1	0.33
---	--	------	------
mPD		0.0065	0.0067
		nPD:	0.97
		N. weight:	0.0031
		Sc. PD:	0.0027
		Sc. rank:	98.0

,

PA

Pos.	607	obs:	exp:
att	I	40593	14267.00
atc	I	46	10679.00
ata	I	3	15696.00
gtt	V	23	5.76
gtc	V	0	4.69
gta	V	0	4.99
gtg	V	0	7.57
---	--	------	------
mPD		0.0035	0.66
		nPD:	0.01
		N. weight:	1.1
		Sc. PD:	-0.056
		Sc. rank:	-25771.0

,

PA

Pos.	608	obs:	exp:
gat	D	2	1.13
gac	D	0	0.87
gaa	E	40360	23973.00
gag	E	303	16690.00
---	--	------	------
mPD		0.015	0.48
		nPD:	0.03
		N. weight:	0.54
		Sc. PD:	-0.013
		Sc. rank:	2969.3

,

PA

Pos.	609	obs:	exp:
tct	S	0	4.20
tcc	S	26	3.89
tca	S	0	6.99
tcg	S	0	1.02
act	T	0	6.52
acc	T	21	3.71
aca	T	0	9.09
acg	T	0	1.68
agt	S	0	4.54
agc	S	0	5.36
gtt	V	1	2.50
gtc	V	9	2.04
gta	V	0	2.17
gtg	V	0	3.29
gct	A	393	11068.00
gcc	A	38993	10146.00
gca	A	1221	16426.00
gcg	A	1	2969.00
---	--	------	------
mPD		0.080	0.70
		nPD:	0.11
		N. weight:	1.3
		Sc. PD:	0.074
		Sc. rank:	31993.0

,

PA

Pos.	610	obs:	exp:
caa	Q	0	0.61
cag	Q	1	0.39
aaa	K	0	0.66
aag	K	1	0.34
gat	D	4	2.26
gac	D	0	1.74
gaa	E	254	23970.00
gag	E	40403	16687.00
ggt	G	0	0.27
ggc	G	0	0.28
gga	G	0	0.92
ggg	G	2	0.53
---	--	------	------
mPD		0.013	0.48
		nPD:	0.03
		N. weight:	0.94
		Sc. PD:	-0.027
		Sc. rank:	1957.0

,

PA

Pos.	611	obs:	exp:
tct	S	684	6561.00
tcc	S	39905	6076.00
tca	S	71	10929.00
tcg	S	0	1601.00
agt	S	0	7105.00
agc	S	0	8388.00
gct	A	0	1.36
gcc	A	5	1.25
gca	A	0	2.02
gcg	A	0	0.37
---	--	------	------
mPD		0.037	1.6
		nPD:	0.02
		N. weight:	2.
		Sc. PD:	-0.065
		Sc. rank:	-3097.2

,

PA			
Pos.	612	obs:	exp:
tct	S	409	6561.00
tcc	S	6	6076.00
tca	S	37644	10929.00
tcg	S	2602	1601.00
agt	S	0	7106.00
agc	S	0	8388.00
gct	A	2	1.09
gcc	A	0	1.00
gca	A	2	1.62
gcg	A	0	0.29
---	--	------	------
mPD		0.14	1.6
		nPD:	0.09
		N. weight:	1.3
		Sc. PD:	0.036
		Sc. rank:	27488.0

,

PA			
Pos.	613	obs:	exp:
ttt	F	3	1.46
ttc	F	0	1.54
tta	L	0	0.23
ttg	L	0	0.41
ctt	L	2	0.42
ctc	L	0	0.29
cta	L	0	0.31
ctg	L	0	0.34
att	I	437	154.80
atc	I	1	115.90
ata	I	3	170.30
act	T	1	0.31
acc	T	0	0.18
aca	T	0	0.43
acg	T	0	0.08
gtt	V	39721	10073.00
gtc	V	475	8195.00
gta	V	7	8717.00
gtg	V	14	13232.00
gct	A	1	0.27
gcc	A	0	0.25
gca	A	0	0.40
gcg	A	0	0.07
---	--	------	------
mPD		0.046	0.76
		nPD:	0.06
		N. weight:	1.5
		Sc. PD:	0.0063
		Sc. rank:	25628.0

,

PA			
Pos.	614	obs:	exp:
cgt	R	0	0.59
cgc	R	0	1.08
cga	R	0	2.74
cgg	R	0	2.58
act	T	0	0.93
acc	T	0	0.53
aca	T	3	1.30
acg	T	0	0.24
aaa	K	40444	26709.00
aag	K	182	13917.00
aga	R	30	14.71
agg	R	0	8.30
gaa	E	6	3.54
gag	E	0	2.46
---	--	------	------
mPD		0.011	0.45
		nPD:	0.02
		N. weight:	0.44
		Sc. PD:	-0.014
		Sc. rank:	-79.2

,

PA			
Pos.	615	obs:	exp:
atg	M	1	1.00
gaa	E	2881	23970.00
gag	E	37777	16688.00
ggt	G	0	0.81
ggc	G	0	0.84
gga	G	0	2.77
ggg	G	6	1.59
---	--	------	------
mPD		0.13	0.48
		nPD:	0.27
		N. weight:	0.67
		Sc. PD:	0.14
		Sc. rank:	19393.0

,

PA			
Pos.	616	obs:	exp:
caa	Q	1	0.61
cag	Q	0	0.39
cgt	R	0	27.98
cgc	R	0	50.77
cga	R	0	128.70
cgg	R	0	121.20
act	T	0	0.62
acc	T	0	0.35
aca	T	2	0.87
acg	T	0	0.16
aaa	K	38956	25802.00
aag	K	291	13445.00
aga	R	1403	692.10
agg	R	8	390.30
gaa	E	3	2.36
gag	E	1	1.64
---	--	------	------
mPD		0.082	0.54
		nPD:	0.15
		N. weight:	0.43
		Sc. PD:	0.041
		Sc. rank:	11484.0

,

PA			
Pos.	617	obs:	exp:
tat	Y	0	0.53
tac	Y	1	0.47
aat	N	0	2.24
aac	N	4	1.76
gat	D	64	22982.00
gac	D	40594	17676.00
gaa	E	1	0.59
gag	E	0	0.41
ggt	G	0	0.13
ggc	G	1	0.14
gga	G	0	0.46
ggg	G	0	0.26
---	--	------	------
mPD		0.0035	0.49
		nPD:	0.01
		N. weight:	0.91
		Sc. PD:	-0.043
		Sc. rank:	-17937.0

,

PA			
Pos.	618	obs:	exp:
tta	L	0	0.58
ttg	L	3	1.03
ctt	L	0	1.05
ctc	L	0	0.73
cta	L	0	0.77
ctg	L	2	0.84
cgt	R	0	0.02
cgc	R	0	0.04
cga	R	0	0.09
cgg	R	0	0.09
att	I	1	3.16
atc	I	1	2.36
ata	I	7	3.48
atg	M	40637	40637.00
act	T	0	0.31
acc	T	0	0.18
aca	T	0	0.43
acg	T	1	0.08
aaa	K	0	0.66
aag	K	1	0.34
aga	R	0	0.49
agg	R	1	0.28
gtt	V	0	2.76
gtc	V	0	2.24
gta	V	0	2.38
gtg	V	11	3.62
---	--	------	------
mPD		0.0014	0.0020
		nPD:	0.68
		N. weight:	0.00076
		Sc. PD:	0.00046
		Sc. rank:	24.0

,

PA			
Pos.	619	obs:	exp:
act	T	64	12628.00
acc	T	40543	7189.00
aca	T	56	17598.00
acg	T	0	3248.00
gct	A	0	0.55
gcc	A	2	0.50
gca	A	0	0.81
gcg	A	0	0.15
---	--	------	------
mPD		0.0060	0.68
		nPD:	0.01
		N. weight:	1.9
		Sc. PD:	-0.087
		Sc. rank:	-33075.0

,

PA			
Pos.	620	obs:	exp:
cgt	R	0	1.13
cgc	R	0	2.05
cga	R	0	5.20
cgg	R	0	4.89
aat	N	1	0.56
aac	N	0	0.44
aaa	K	40377	26696.00
aag	K	230	13911.00
aga	R	10	27.96
agg	R	47	15.77
---	--	------	------
mPD		0.016	0.45
		nPD:	0.04
		N. weight:	0.43
		Sc. PD:	-0.0083
		Sc. rank:	3879.4

,

PA			
Pos.	621	obs:	exp:
aaa	K	0	3.29
aag	K	5	1.71
gct	A	0	0.27
gcc	A	0	0.25
gca	A	0	0.40
gcg	A	1	0.07
gat	D	24	13.57
gac	D	0	10.43
gaa	E	332	23952.00
gag	E	40295	16675.00
ggt	G	0	1.08
ggc	G	0	1.12
gga	G	0	3.69
ggg	G	8	2.11
---	--	------	------
mPD		0.018	0.49
		nPD:	0.04
		N. weight:	0.93
		Sc. PD:	-0.017
		Sc. rank:	8761.8

,

PA			
Pos.	622	obs:	exp:
ttt	F	40300	19780.00
ttc	F	363	20883.00
tta	L	0	0.12
ttg	L	0	0.21
tgt	C	1	0.39
tgc	C	0	0.61
ctt	L	1	0.21
ctc	L	0	0.15
cta	L	0	0.15
ctg	L	0	0.17
---	--	------	------
mPD		0.018	0.50
		nPD:	0.04
		N. weight:	0.74
		Sc. PD:	-0.015
		Sc. rank:	6316.8

,

PA			
Pos.	623	obs:	exp:
ttt	F	40645	19780.00
ttc	F	18	20883.00
tat	Y	2	1.05
tac	Y	0	0.95
---	--	------	------
mPD		0.00098	0.50
		nPD:	0.
		N. weight:	0.79
		Sc. PD:	-0.042
		Sc. rank:	-23255.0

,

PA

Pos.	624	obs:	exp:
aaa	K	0	5.92
aag	K	9	3.08
gat	D	21	11.87
gac	D	0	9.13
gaa	E	500	23950.00
gag	E	40124	16674.00
ggt	G	0	1.35
ggc	G	0	1.40
gga	G	0	4.62
ggg	G	10	2.64
---	-	1	1.00
---	--	------	------
mPD		0.026	0.49
		nPD:	0.05
		N. weight:	0.91
		Sc. PD:	-0.0017
		Sc. rank:	14421.0

,

PA

Pos.	625	obs:	exp:
tct	S	0	9.04
tcc	S	0	8.37
tca	S	0	15.05
tcg	S	0	2.20
act	T	11	3.42
acc	T	0	1.94
aca	T	0	4.76
acg	T	0	0.88
aat	N	40077	22682.00
aac	N	465	17860.00
aaa	K	1	1.31
aag	K	1	0.69
agt	S	56	9.79
agc	S	0	11.55
gat	D	52	29.96
gac	D	1	23.04
---	-	1	1.00
---	--	------	------
mPD		0.029	0.50
		nPD:	0.06
		N. weight:	0.58
		Sc. PD:	0.00064
		Sc. rank:	9754.5

,

PA

Pos.	626	obs:	exp:
caa	Q	1	0.61
cag	Q	0	0.39
cgt	R	0	0.87
cgc	R	0	1.58
cga	R	0	4.01
cgg	R	0	3.78
aat	N	3	2.80
aac	N	2	2.20
aaa	K	40555	26700.00
aag	K	58	13913.00
aga	R	44	21.58
agg	R	0	12.17
gaa	E	2	1.18
gag	E	0	0.82
---	--	------	------
mPD		0.0054	0.45
		nPD:	0.01
		N. weight:	0.45
		Sc. PD:	-0.019
		Sc. rank:	-6336.4

,

PA

Pos.	627	obs:	exp:
tta	L	23	2.67
ttg	L	0	4.76
tct	S	38	6549.00
tcc	S	9	6065.00
tca	S	40317	10908.00
tcg	S	221	1598.00
ctt	L	0	4.81
ctc	L	0	3.37
cta	L	0	3.53
ctg	L	0	3.86
cct	P	0	2.33
ccc	P	0	1.31
cca	P	7	2.36
ccg	P	0	0.99
act	T	0	12.11
acc	T	0	6.90
aca	T	39	16.88
acg	T	0	3.11
agt	S	0	7092.00
agc	S	0	8373.00
gct	A	0	3.00
gcc	A	0	2.75
gca	A	11	4.45
gcg	A	0	0.80
---	--	------	------
mPD		0.017	1.6
		nPD:	0.01
		N. weight:	1.4
		Sc. PD:	-0.063
		Sc. rank:	-22721.0

,

PA

Pos.	628	obs:	exp:
aaa	K	3	1.97
aag	K	0	1.03
gtt	V	0	0.25
gtc	V	0	0.20
gta	V	1	0.22
gtg	V	0	0.33
gat	D	1	6.22
gac	D	10	4.78
gaa	E	40538	23964.00
gag	E	109	16683.00
ggt	G	0	0.40
ggc	G	0	0.42
gga	G	3	1.38
ggg	G	0	0.79
---	--	------	------
mPD		0.0062	0.48
		nPD:	0.01
		N. weight:	0.57
		Sc. PD:	-0.024
		Sc. rank:	-7343.2

,

PA

Pos.	629	obs:	exp:
tct	S	0	4.84
tcc	S	0	4.48
tca	S	30	8.06
tcg	S	0	1.18
caa	Q	1	0.61
cag	Q	0	0.39
att	I	0	0.35
atc	I	0	0.26
ata	I	1	0.39
act	T	1	105.60
acc	T	0	60.11
aca	T	339	147.10
acg	T	0	27.16
agt	S	0	5.24
agc	S	0	6.19
gtt	V	0	11.27
gtc	V	0	9.17
gta	V	45	9.75
gtg	V	0	14.81
gct	A	4	10969.00
gcc	A	2	10055.00
gca	A	40196	16279.00
gcg	A	43	2942.00
gaa	E	3	1.77
gag	E	0	1.23
---	--	------	------
mPD		0.023	0.72
		nPD:	0.03
		N. weight:	0.99
		Sc. PD:	-0.023
		Sc. rank:	6612.6

,

PA

Pos.	630	obs:	exp:
tgg	W	40665	40665.00
---	--	------	------
mPD		0	0
		nPD:	1.
		N. weight:	0.
		Sc. PD:	0
		Sc. rank:	0

,

PA

Pos.	631	obs:	exp:
cct	P	92	13551.00
ccc	P	40346	7624.00
cca	P	227	13739.00
ccg	P	0	5751.00
---	--	------	------
mPD		0.016	0.72
		nPD:	0.02
		N. weight:	1.8
		Sc. PD:	-0.06
		Sc. rank:	-4923.7

,

PA

Pos.	632	obs:	exp:
tta	L	0	1.39
ttg	L	0	2.48
ctt	L	12	2.51
ctc	L	0	1.76
cta	L	0	1.84
ctg	L	0	2.01
att	I	40401	14265.00
atc	I	233	10677.00
ata	I	1	15693.00
act	T	2	0.62
acc	T	0	0.35
aca	T	0	0.87
acg	T	0	0.16
gtt	V	16	4.01
gtc	V	0	3.26
gta	V	0	3.47
gtg	V	0	5.26
---	--	------	------
mPD		0.013	0.66
		nPD:	0.02
		N. weight:	1.1
		Sc. PD:	-0.04
		Sc. rank:	-5340.0

,

PA

Pos.	633	obs:	exp:
cgt	R	0	0.02
cgc	R	0	0.04
cga	R	0	0.09
cgg	R	0	0.09
aga	R	0	0.49
agg	R	1	0.28
ggt	G	85	5474.00
ggc	G	0	5675.00
gga	G	321	18771.00
ggg	G	40258	10743.00
---	--	------	------
mPD		0.020	0.68
		nPD:	0.03
		N. weight:	1.4
		Sc. PD:	-0.036
		Sc. rank:	6448.4

,

PA

Pos.	634	obs:	exp:
gtt	V	0	0.25
gtc	V	0	0.20
gta	V	0	0.22
gtg	V	1	0.33
gat	D	28	15.83
gac	D	0	12.17
gaa	E	3251	23954.00
gag	E	37379	16676.00
ggt	G	0	0.81
ggc	G	0	0.84
gga	G	0	2.77
ggg	G	6	1.59
---	--	------	------
mPD		0.15	0.49
		nPD:	0.31
		N. weight:	0.65
		Sc. PD:	0.16
		Sc. rank:	18992.0

,

PA

Pos.	635	obs:	exp:
tct	S	145	6562.00
tcc	S	40309	6077.00
tca	S	209	10929.00
tcg	S	0	1601.00
act	T	0	0.62
acc	T	2	0.35
aca	T	0	0.87
acg	T	0	0.16
agt	S	0	7106.00
agc	S	0	8389.00
---	--	------	------
mPD		0.017	1.6
		nPD:	0.01
		N. weight:	2.
		Sc. PD:	-0.09
		Sc. rank:	-32038.0

,

PA

Pos.	636	obs:	exp:
cct	P	139	13551.00
ccc	P	40522	7624.00
cca	P	4	13739.00
ccg	P	0	5751.00
---	--	------	------
mPD		0.0070	0.72
		nPD:	0.01
		N. weight:	1.8
		Sc. PD:	-0.082
		Sc. rank:	-30410.0

,

PA

Pos.	637	obs:	exp:
cgt	R	0	19.19
cgc	R	0	34.83
cga	R	0	88.32
cgg	R	0	83.12
att	I	0	0.35
atc	I	0	0.26
ata	I	1	0.39
act	T	0	1.55
acc	T	0	0.88
aca	T	5	2.16
acg	T	0	0.40
aat	N	1	2.80
aac	N	4	2.20
aaa	K	34078	26066.00
aag	K	5570	13582.00
aga	R	936	474.80
agg	R	32	267.70
gaa	E	32	22.40
gag	E	6	15.60
---	--	------	------
mPD		0.29	0.51
		nPD:	0.56
		N. weight:	0.13
		Sc. PD:	0.063
		Sc. rank:	4055.0

,

PA

Pos.	638	obs:	exp:
cgt	R	0	0.59
cgc	R	0	1.08
cga	R	1	2.74
cgg	R	0	2.58
aga	R	29	14.71
agg	R	0	8.30
gtt	V	0	0.50
gtc	V	0	0.41
gta	V	2	0.43
gtg	V	0	0.66
gaa	E	2	1.18
gag	E	0	0.82
ggt	G	7	5470.00
ggc	G	2	5671.00
gga	G	40287	18756.00
ggg	G	335	10734.00
---	--	------	------
mPD		0.018	0.68
		nPD:	0.03
		N. weight:	0.81
		Sc. PD:	-0.023
		Sc. rank:	2250.4

,

PA

Pos.	639	obs:	exp:
tta	L	0	0.46
ttg	L	4	0.83
ctt	L	0	0.84
ctc	L	0	0.59
cta	L	0	0.61
ctg	L	0	0.67
cgt	R	0	0.02
cgc	R	0	0.04
cga	R	0	0.09
cgg	R	0	0.09
att	I	0	5.62
atc	I	0	4.20
ata	I	16	6.18
atg	M	7	7.00
aga	R	0	0.49
agg	R	1	0.28
gtt	V	60	10179.00
gtc	V	0	8280.00
gta	V	6514	8808.00
gtg	V	34063	13371.00
---	--	------	------
mPD		0.27	0.74
		nPD:	0.37
		N. weight:	0.81
		Sc. PD:	0.24
		Sc. rank:	24360.0

,

PA

Pos.	640	obs:	exp:
gaa	E	40176	23974.00
gag	E	488	16690.00
ggt	G	0	0.13
ggc	G	0	0.14
gga	G	1	0.46
ggg	G	0	0.26
---	--	------	------
mPD		0.024	0.48
		nPD:	0.05
		N. weight:	0.52
		Sc. PD:	-0.0035
		Sc. rank:	7195.6

,

PA

Pos.	641	obs:	exp:
aaa	K	30	19.72
aag	K	0	10.28
gtt	V	0	0.25
gtc	V	0	0.20
gta	V	1	0.22
gtg	V	0	0.33
gct	A	0	0.27
gcc	A	0	0.25
gca	A	1	0.40
gcg	A	0	0.07
gat	D	27	18.09
gac	D	5	13.91
gaa	E	40461	23921.00
gag	E	113	16653.00
ggt	G	0	3.63
ggc	G	0	3.77
gga	G	27	12.46
ggg	G	0	7.13
---	--	------	------
mPD		0.010	0.49
		nPD:	0.02
		N. weight:	0.56
		Sc. PD:	-0.019
		Sc. rank:	-2206.3

,

PA

Pos.	642	obs:	exp:
tct	S	0	4.84
tcc	S	0	4.48
tca	S	0	8.06
tcg	S	0	1.18
tgt	C	4	1.56
tgc	C	0	2.44
agt	S	30	5.24
agc	S	0	6.19
gtt	V	4	1.25
gtc	V	1	1.02
gta	V	0	1.08
gtg	V	0	1.65
gct	A	2	0.55
gcc	A	0	0.50
gca	A	0	0.81
gcg	A	0	0.15
ggt	G	39722	5469.00
ggc	G	885	5670.00
gga	G	5	18753.00
ggg	G	12	10732.00
---	--	------	------
mPD		0.045	0.68
		nPD:	0.07
		N. weight:	2.1
		Sc. PD:	0.022
		Sc. rank:	38990.0

,

PA

Pos.	643	obs:	exp:
tct	S	502	6557.00
tcc	S	40114	6072.00
tca	S	17	10921.00
tcg	S	0	1599.00
cct	P	0	8.00
ccc	P	24	4.50
cca	P	0	8.11
ccg	P	0	3.39
act	T	0	2.48
acc	T	8	1.41
aca	T	0	3.46
acg	T	0	0.64
agt	S	0	7101.00
agc	S	0	8382.00
---	--	------	------
mPD		0.027	1.6
		nPD:	0.02
		N. weight:	2.
		Sc. PD:	-0.078
		Sc. rank:	-16546.0

,

PA

Pos.	644	obs:	exp:
tta	L	0	0.23
ttg	L	0	0.41
ctt	L	2	0.42
ctc	L	0	0.29
cta	L	0	0.31
ctg	L	0	0.34
att	I	40569	14273.00
atc	I	87	10684.00
ata	I	3	15702.00
atg	M	2	2.00
gtt	V	2	0.50
gtc	V	0	0.41
gta	V	0	0.43
gtg	V	0	0.66
---	--	------	------
mPD		0.0047	0.66
		nPD:	0.01
		N. weight:	1.1
		Sc. PD:	-0.054
		Sc. rank:	-22366.0

,

PA

Pos.	645	obs:	exp:
ggt	G	25	5475.00
ggc	G	2840	5676.00
gga	G	3672	18772.00
ggg	G	34128	10743.00
---	--	------	------
mPD		0.28	0.68
		nPD:	0.42
		N. weight:	0.85
		Sc. PD:	0.3
		Sc. rank:	26157.0

,

PA

Pos.	646	obs:	exp:
cgt	R	0	0.04
cgc	R	0	0.07
cga	R	0	0.18
cgg	R	0	0.17
aaa	K	40491	26733.00
aag	K	172	13930.00
aga	R	2	0.98
agg	R	0	0.55
---	--	------	------
mPD		0.0085	0.45
		nPD:	0.02
		N. weight:	0.44
		Sc. PD:	-0.016
		Sc. rank:	-2473.8

,

PA

Pos.	647	obs:	exp:
att	I	0	12.64
atc	I	36	9.46
ata	I	0	13.90
gtt	V	39	10176.00
gtc	V	40330	8278.00
gta	V	59	8805.00
gtg	V	198	13367.00
gct	A	0	0.82
gcc	A	3	0.75
gca	A	0	1.21
gcg	A	0	0.22
---	--	------	------
mPD		0.016	0.74
		nPD:	0.02
		N. weight:	1.7
		Sc. PD:	-0.056
		Sc. rank:	-3416.2

,

PA			
Pos.	648	obs:	exp:
tct	S	0	0.16
tcc	S	0	0.15
tca	S	0	0.27
tcg	S	0	0.04
tgt	C	40357	15891.00
tgc	C	306	24772.00
agt	S	1	0.17
agc	S	0	0.21
ggt	G	1	0.13
ggc	G	0	0.14
gga	G	0	0.46
ggg	G	0	0.26
---	--	------	------
mPD		0.015	0.48
		nPD:	0.03
		N. weight:	0.99
		Sc. PD:	-0.023
		Sc. rank:	5946.4

,

PA			
Pos.	649	obs:	exp:
tct	S	0	0.16
tcc	S	0	0.15
tca	S	0	0.27
tcg	S	0	0.04
cgt	R	0	806.20
cgc	R	0	1463.00
cga	R	0	3710.00
cgg	R	4	3492.00
act	T	0	0.31
acc	T	0	0.18
aca	T	0	0.43
acg	T	1	0.08
agt	S	1	0.17
agc	S	0	0.21
aga	R	152	19945.00
agg	R	40507	11247.00
---	--	------	------
mPD		0.0077	0.89
		nPD:	0.01
		N. weight:	1.4
		Sc. PD:	-0.064
		Sc. rank:	-24576.0

,

PA			
Pos.	650	obs:	exp:
att	I	6	2.11
atc	I	0	1.58
ata	I	0	2.32
act	T	40282	12608.00
acc	T	294	7178.00
aca	T	8	17570.00
acg	T	15	3243.00
aat	N	1	0.56
aac	N	0	0.44
gct	A	46	15.81
gcc	A	12	14.49
gca	A	0	23.46
gcg	A	0	4.24
---	-	1	1.00
---	--	------	------
mPD		0.019	0.68
		nPD:	0.03
		N. weight:	1.2
		Sc. PD:	-0.034
		Sc. rank:	4609.2

,

PA			
Pos.	651	obs:	exp:
tta	L	456	4718.00
ttg	L	234	8406.00
ctt	L	4	8506.00
ctc	L	1	5962.00
cta	L	39912	6244.00
ctg	L	53	6824.00
att	I	0	1.40
atc	I	0	1.05
ata	I	4	1.54
---	-	1	1.00
---	--	------	------
mPD		0.048	1.2
		nPD:	0.04
		N. weight:	2.
		Sc. PD:	-0.028
		Sc. rank:	22617.0

,

PA			
Pos.	652	obs:	exp:
tta	L	4307	4719.00
ttg	L	36104	8407.00
ctt	L	0	8506.00
ctc	L	0	5963.00
cta	L	7	6244.00
ctg	L	246	6825.00
---	-	1	1.00
---	--	------	------
mPD		0.20	1.2
		nPD:	0.17
		N. weight:	1.4
		Sc. PD:	0.16
		Sc. rank:	38101.0

,

PA			
Pos.	653	obs:	exp:
tct	S	1	0.16
tcc	S	0	0.15
tca	S	0	0.27
tcg	S	0	0.04
act	T	9	3.11
acc	T	0	1.77
aca	T	1	4.33
acg	T	0	0.80
agt	S	0	0.17
agc	S	0	0.21
gct	A	40257	11080.00
gcc	A	171	10157.00
gca	A	216	16445.00
gcg	A	10	2972.00
---	--	------	------
mPD		0.020	0.69
		nPD:	0.03
		N. weight:	1.4
		Sc. PD:	-0.036
		Sc. rank:	5681.9

,

PA			
Pos.	654	obs:	exp:
cgt	R	0	0.18
cgc	R	0	0.32
cga	R	0	0.82
cgg	R	0	0.77
aat	N	1	0.56
aac	N	0	0.44
aaa	K	300	26728.00
aag	K	40355	13927.00
aga	R	0	4.41
agg	R	9	2.49
---	--	------	------
mPD		0.015	0.45
		nPD:	0.03
		N. weight:	1.1
		Sc. PD:	-0.025
		Sc. rank:	8806.9

,

PA			
Pos.	655	obs:	exp:
tta	L	1	0.12
ttg	L	0	0.21
tct	S	210	6561.00
tcc	S	9	6076.00
tca	S	39933	10929.00
tcg	S	508	1601.00
ctt	L	0	0.21
ctc	L	0	0.15
cta	L	0	0.15
ctg	L	0	0.17
act	T	0	0.31
acc	T	0	0.18
aca	T	1	0.43
acg	T	0	0.08
agt	S	0	7105.00
agc	S	0	8388.00
gct	A	0	0.82
gcc	A	0	0.75
gca	A	3	1.21
gcg	A	0	0.22
---	--	------	------
mPD		0.035	1.6
		nPD:	0.02
		N. weight:	1.4
		Sc. PD:	-0.046
		Sc. rank:	-3484.6

,

PA			
Pos.	656	obs:	exp:
att	I	0	1.40
atc	I	0	1.05
ata	I	4	1.54
gtt	V	85	10182.00
gtc	V	0	8283.00
gta	V	39824	8810.00
gtg	V	740	13374.00
gct	A	0	3.27
gcc	A	0	3.00
gca	A	12	4.85
gcg	A	0	0.88
---	--	------	------
mPD		0.041	0.74
		nPD:	0.05
		N. weight:	1.6
		Sc. PD:	-0.0019
		Sc. rank:	25420.0

,

PA			
Pos.	657	obs:	exp:
ttt	F	584	19780.00
ttc	F	40081	20885.00
---	--	------	------
mPD		0.028	0.50
		nPD:	0.06
		N. weight:	0.66
		Sc. PD:	0.00041
		Sc. rank:	10767.0

,

PA			
Pos.	658	obs:	exp:
tct	S	0	0.48
tcc	S	0	0.45
tca	S	0	0.81
tcg	S	0	0.12
aat	N	40372	22749.00
aac	N	289	17912.00
aaa	K	1	0.66
aag	K	0	0.34
agt	S	3	0.52
agc	S	0	0.62
---	--	------	------
mPD		0.014	0.49
		nPD:	0.03
		N. weight:	0.6
		Sc. PD:	-0.016
		Sc. rank:	2597.9

,

PA			
Pos.	659	obs:	exp:
tct	S	0	6555.00
tcc	S	0	6070.00
tca	S	0	10918.00
tcg	S	0	1599.00
tgt	C	0	0.78
tgc	C	2	1.22
aat	N	0	19.58
aac	N	35	15.42
agt	S	330	7099.00
agc	S	40292	8380.00
ggt	G	0	0.81
ggc	G	6	0.84
gga	G	0	2.77
ggg	G	0	1.59
---	--	------	------
mPD		0.018	1.6
		nPD:	0.01
		N. weight:	1.7
		Sc. PD:	-0.074
		Sc. rank:	-25460.0

,

PA

Pos.	660	obs:	exp:
tta	L	4	4717.00
ttg	L	103	8404.00
ctt	L	258	8504.00
ctc	L	0	5961.00
cta	L	800	6242.00
ctg	L	39486	6822.00
cct	P	0	0.33
ccc	P	0	0.19
cca	P	0	0.34
ccg	P	1	0.14
att	I	1	0.70
atc	I	0	0.53
ata	I	1	0.77
atg	M	11	11.00
---	--	------	------
mPD		0.057	1.2
		nPD:	0.05
		N. weight:	1.8
		Sc. PD:	-0.012
		Sc. rank:	25367.0

,

PA

Pos.	661	obs:	exp:
ttt	F	2	0.97
ttc	F	0	1.03
tat	Y	40364	21433.00
tac	Y	297	19228.00
tgt	C	0	0.39
tgc	C	1	0.61
cat	H	1	0.56
cac	H	0	0.44
---	--	------	------
mPD		0.015	0.50
		nPD:	0.03
		N. weight:	0.66
		Sc. PD:	-0.017
		Sc. rank:	3294.7

,

PA

Pos.	662	obs:	exp:
tct	S	0	0.48
tcc	S	0	0.45
tca	S	3	0.81
tcg	S	0	0.12
agt	S	0	0.52
agc	S	0	0.62
gct	A	15	11083.00
gcc	A	0	10159.00
gca	A	40447	16448.00
gcg	A	200	2972.00
---	--	------	------
mPD		0.011	0.69
		nPD:	0.02
		N. weight:	0.97
		Sc. PD:	-0.038
		Sc. rank:	-9775.1

,

PA

Pos.	663	obs:	exp:
tct	S	573	6561.00
tcc	S	68	6076.00
tca	S	39339	10928.00
tcg	S	679	1600.00
act	T	0	1.24
acc	T	0	0.71
aca	T	4	1.73
acg	T	0	0.32
agt	S	0	7105.00
agc	S	0	8388.00
gct	A	0	0.27
gcc	A	0	0.25
gca	A	1	0.40
gcg	A	0	0.07
---	-	1	1.00
---	--	------	------
mPD		0.064	1.6
		nPD:	0.04
		N. weight:	1.3
		Sc. PD:	-0.022
		Sc. rank:	14058.0

,

PA

Pos.	664	obs:	exp:
tct	S	0	1.61
tcc	S	0	1.49
tca	S	10	2.69
tcg	S	0	0.39
cct	P	0	13546.00
ccc	P	6	7621.00
cca	P	40597	13734.00
ccg	P	48	5750.00
caa	Q	1	0.61
cag	Q	0	0.39
act	T	0	0.62
acc	T	0	0.35
aca	T	2	0.87
acg	T	0	0.16
agt	S	0	1.75
agc	S	0	2.06
---	-	1	1.00
---	--	------	------
mPD		0.0033	0.72
		nPD:	0.
		N. weight:	1.2
		Sc. PD:	-0.059
		Sc. rank:	-28111.0

,

PA

Pos.	665	obs:	exp:
caa	Q	40462	24857.00
cag	Q	202	15807.00
---	-	1	1.00
---	--	------	------
mPD		0.0099	0.48
		nPD:	0.02
		N. weight:	0.51
		Sc. PD:	-0.018
		Sc. rank:	-1885.6

,

PA

Pos.	666	obs:	exp:
ttt	F	8	3.89
ttc	F	0	4.11
tta	L	422	4718.00
ttg	L	39960	8405.00
ctt	L	200	8505.00
ctc	L	0	5962.00
cta	L	6	6243.00
ctg	L	67	6823.00
att	I	1	0.35
atc	I	0	0.26
ata	I	0	0.39
atg	M	1	1.00
---	--	------	------
mPD		0.044	1.2
		nPD:	0.04
		N. weight:	1.6
		Sc. PD:	-0.028
		Sc. rank:	16942.0

,

PA

Pos.	667	obs:	exp:
gaa	E	40348	23975.00
gag	E	317	16690.00
---	--	------	------
mPD		0.015	0.48
		nPD:	0.03
		N. weight:	0.54
		Sc. PD:	-0.013
		Sc. rank:	3417.3

,

PA

Pos.	668	obs:	exp:
gtt	V	0	0.25
gtc	V	0	0.20
gta	V	1	0.22
gtg	V	0	0.33
ggt	G	87	5474.00
ggc	G	47	5675.00
gga	G	39787	18771.00
ggg	G	743	10743.00
---	--	------	------
mPD		0.042	0.68
		nPD:	0.06
		N. weight:	0.74
		Sc. PD:	0.0046
		Sc. rank:	13266.0

,

PA

Pos.	669	obs:	exp:
ttt	F	40568	19780.00
ttc	F	95	20883.00
tat	Y	2	1.05
tac	Y	0	0.95
---	--	------	------
mPD		0.0048	0.50
		nPD:	0.01
		N. weight:	0.78
		Sc. PD:	-0.035
		Sc. rank:	-13283.0

,

PA

Pos.	670	obs:	exp:
tta	L	8	1.04
ttg	L	1	1.86
tct	S	10	6561.00
tcc	S	8	6076.00
tca	S	40405	10928.00
tcg	S	233	1600.00
ctt	L	0	1.88
ctc	L	0	1.32
cta	L	0	1.38
ctg	L	0	1.51
agt	S	0	7105.00
agc	S	0	8387.00
---	--	------	------
mPD		0.013	1.6
		nPD:	0.01
		N. weight:	1.4
		Sc. PD:	-0.067
		Sc. rank:	-26290.0

,

PA

Pos.	671	obs:	exp:
tct	S	1	1.61
tcc	S	0	1.49
tca	S	7	2.69
tcg	S	2	0.39
act	T	0	0.93
acc	T	0	0.53
aca	T	3	1.30
acg	T	0	0.24
agt	S	0	1.75
agc	S	0	2.06
gct	A	241	11080.00
gcc	A	4	10157.00
gca	A	35694	16444.00
gcg	A	4713	2972.00
---	--	------	------
mPD		0.22	0.70
		nPD:	0.31
		N. weight:	0.79
		Sc. PD:	0.19
		Sc. rank:	23212.0

,

PA

Pos.	672	obs:	exp:
gaa	E	769	23974.00
gag	E	39895	16690.00
ggt	G	0	0.13
ggc	G	0	0.14
gga	G	0	0.46
ggg	G	1	0.26
---	--	------	------
mPD		0.037	0.48
		nPD:	0.08
		N. weight:	0.87
		Sc. PD:	0.018
		Sc. rank:	18111.0

,

PA

Pos.	673	obs:	exp:
tta	L	2	0.23
ttg	L	0	0.41
tct	S	5	6561.00
tcc	S	1	6076.00
tca	S	40430	10929.00
tcg	S	224	1601.00
ctt	L	0	0.42
ctc	L	0	0.29
cta	L	0	0.31
ctg	L	0	0.34
act	T	0	0.31
acc	T	0	0.18
aca	T	1	0.43
acg	T	0	0.08
agt	S	0	7105.00
agc	S	0	8388.00
gct	A	0	0.55
gcc	A	0	0.50
gca	A	2	0.81
gcg	A	0	0.15
---	--	------	------
mPD		0.011	1.6
		nPD:	0.01
		N. weight:	1.4
		Sc. PD:	-0.068
		Sc. rank:	-28366.0

,

PA

Pos.	674	obs:	exp:
cgt	R	0	806.30
cgc	R	0	1463.00
cga	R	30	3710.00
cgg	R	0	3492.00
aga	R	40359	19945.00
agg	R	275	11248.00
---	-	1	1.00
---	--	------	------
mPD		0.015	0.89
		nPD:	0.02
		N. weight:	0.74
		Sc. PD:	-0.028
		Sc. rank:	-5245.4

,

PA

Pos.	675	obs:	exp:
cgt	R	0	0.16
cgc	R	0	0.29
cga	R	0	0.73
cgg	R	0	0.69
att	I	0	0.35
atc	I	0	0.26
ata	I	1	0.39
aaa	K	40433	26727.00
aag	K	220	13926.00
aga	R	8	3.92
agg	R	0	2.21
---	-	3	3.00
---	--	------	------
mPD		0.011	0.45
		nPD:	0.02
		N. weight:	0.43
		Sc. PD:	-0.013
		Sc. rank:	363.6

,

PA

Pos.	676	obs:	exp:
tta	L	1	4713.00
ttg	L	504	8397.00
ctt	L	5	8496.00
ctc	L	0	5956.00
cta	L	216	6237.00
ctg	L	39889	6816.00
atg	M	48	48.00
---	-	2	2.00
---	--	------	------
mPD		0.038	1.2
		nPD:	0.03
		N. weight:	1.9
		Sc. PD:	-0.042
		Sc. rank:	12889.0

,

PA

Pos.	677	obs:	exp:
ttt	F	0	0.49
ttc	F	1	0.51
tta	L	1	4718.00
ttg	L	0	8406.00
ctt	L	346	8506.00
ctc	L	40271	5962.00
cta	L	42	6244.00
ctg	L	0	6824.00
att	I	0	1.40
atc	I	4	1.05
ata	I	0	1.54
---	--	------	------
mPD		0.019	1.2
		nPD:	0.02
		N. weight:	2.1
		Sc. PD:	-0.079
		Sc. rank:	-15770.0

,

PA

Pos.	678	obs:	exp:
tta	L	0	4719.00
ttg	L	0	8407.00
ctt	L	40400	8507.00
ctc	L	264	5963.00
cta	L	1	6244.00
ctg	L	0	6825.00
---	--	------	------
mPD		0.013	1.2
		nPD:	0.01
		N. weight:	1.7
		Sc. PD:	-0.074
		Sc. rank:	-25135.0

,

PA

Pos.	679	obs:	exp:
att	I	35322	12405.00
atc	I	16	9286.00
ata	I	1	13648.00
act	T	1	0.31
acc	T	0	0.18
aca	T	0	0.43
acg	T	0	0.08
gtt	V	5140	1334.00
gtc	V	61	1085.00
gta	V	35	1154.00
gtg	V	89	1752.00
---	--	------	------
mPD		0.24	0.91
		nPD:	0.26
		N. weight:	1.2
		Sc. PD:	0.23
		Sc. rank:	33543.0

,

PA

Pos.	680	obs:	exp:
att	I	364	127.80
atc	I	0	95.65
ata	I	0	140.60
gtt	V	40150	10094.00
gtc	V	146	8211.00
gta	V	3	8734.00
gtg	V	0	13259.00
gct	A	2	0.55
gcc	A	0	0.50
gca	A	0	0.81
gcg	A	0	0.15
---	--	------	------
mPD		0.025	0.76
		nPD:	0.03
		N. weight:	1.5
		Sc. PD:	-0.033
		Sc. rank:	10938.0

,

PA

Pos.	681	obs:	exp:
caa	Q	57	24858.00
cag	Q	40608	15807.00
---	--	------	------
mPD		0.0028	0.48
		nPD:	0.01
		N. weight:	1.
		Sc. PD:	-0.05
		Sc. rank:	-22772.0

,

PA

Pos.	682	obs:	exp:
tta	L	0	0.12
ttg	L	0	0.21
ctt	L	0	0.21
ctc	L	1	0.15
cta	L	0	0.15
ctg	L	0	0.17
gtt	V	1	0.25
gtc	V	0	0.20
gta	V	0	0.22
gtg	V	0	0.33
gct	A	40364	11083.00
gcc	A	98	10159.00
gca	A	200	16448.00
gcg	A	1	2973.00
---	--	------	------
mPD		0.015	0.69
		nPD:	0.02
		N. weight:	1.4
		Sc. PD:	-0.047
		Sc. rank:	-4314.4

,

PA

Pos.	683	obs:	exp:
ttt	F	3	1.95
ttc	F	1	2.05
tta	L	0	4718.00
ttg	L	0	8406.00
ctt	L	40563	8506.00
ctc	L	91	5962.00
cta	L	1	6244.00
ctg	L	5	6824.00
cct	P	0	0.33
ccc	P	0	0.19
cca	P	0	0.34
ccg	P	1	0.14
---	--	------	------
mPD		0.0051	1.2
		nPD:	0.
		N. weight:	1.7
		Sc. PD:	-0.086
		Sc. rank:	-40974.0

,

PA			
Pos.	684	obs:	exp:
tct	S	0	0.16
tcc	S	0	0.15
tca	S	0	0.27
tcg	S	0	0.04
cgt	R	0	805.70
cgc	R	0	1462.00
cga	R	0	3707.00
cgg	R	0	3489.00
aaa	K	0	17.75
aag	K	27	9.25
agt	S	1	0.17
agc	S	0	0.21
aga	R	181	19930.00
agg	R	40453	11239.00
ggt	G	0	0.27
ggc	G	0	0.28
gga	G	0	0.92
ggg	G	2	0.53
---	-	1	1.00
---	--	------	------
mPD		0.010	0.89
		nPD:	0.01
		N. weight:	1.4
		Sc. PD:	-0.06
		Sc. rank:	-19791.0

,

PA			
Pos.	685	obs:	exp:
gtt	V	0	0.25
gtc	V	1	0.20
gta	V	0	0.22
gtg	V	0	0.33
gat	D	172	22985.00
gac	D	40491	17678.00
---	-	1	1.00
---	--	------	------
mPD		0.0085	0.49
		nPD:	0.02
		N. weight:	0.89
		Sc. PD:	-0.034
		Sc. rank:	-6081.7

,

PA			
Pos.	686	obs:	exp:
tct	S	0	0.32
tcc	S	0	0.30
tca	S	0	0.54
tcg	S	0	0.08
tat	Y	0	0.53
tac	Y	1	0.47
cat	H	0	0.56
cac	H	1	0.44
cgt	R	0	0.75
cgc	R	0	1.37
cga	R	0	3.47
cgg	R	0	3.26
aat	N	156	2643.00
aac	N	4568	2081.00
aaa	K	31297	23599.00
aag	K	4599	12297.00
agt	S	0	0.35
agc	S	2	0.41
aga	R	27	18.64
agg	R	11	10.51
gat	D	0	0.57
gac	D	1	0.43
gaa	E	1	0.59
gag	E	0	0.41
ggt	G	0	0.13
ggc	G	0	0.14
gga	G	0	0.46
ggg	G	1	0.26
---	--	------	------
mPD		0.38	0.57
		nPD:	0.68
		N. weight:	0.2
		Sc. PD:	0.12
		Sc. rank:	6454.9

,

PA			
Pos.	687	obs:	exp:
tta	L	0	4719.00
ttg	L	175	8407.00
ctt	L	457	8507.00
ctc	L	39928	5963.00
cta	L	74	6244.00
ctg	L	31	6825.00
---	--	------	------
mPD		0.044	1.2
		nPD:	0.04
		N. weight:	2.
		Sc. PD:	-0.034
		Sc. rank:	20380.0

,

PA			
Pos.	688	obs:	exp:
aaa	K	9	5.92
aag	K	0	3.08
gct	A	0	0.27
gcc	A	0	0.25
gca	A	1	0.40
gcg	A	0	0.07
gat	D	0	1.70
gac	D	3	1.30
gaa	E	40614	23967.00
gag	E	38	16685.00
---	--	------	------
mPD		0.0025	0.48
		nPD:	0.01
		N. weight:	0.58
		Sc. PD:	-0.028
		Sc. rank:	-13202.0

,

PA			
Pos.	689	obs:	exp:
aat	N	0	0.56
aac	N	1	0.44
---	-	40664	40664.00
---	--	------	------
mPD		0	3.0×10^{-10}
		nPD:	0.
		N. weight:	0.
		Sc. PD:	0
		Sc. rank:	0

,

PA			
Pos.	690	obs:	exp:
cct	P	40512	13551.00
ccc	P	136	7624.00
cca	P	8	13739.00
ccg	P	9	5751.00
---	--	------	------
mPD		0.0075	0.72
		nPD:	0.01
		N. weight:	1.2
		Sc. PD:	-0.053
		Sc. rank:	-19200.0

,

PA			
Pos.	691	obs:	exp:
ggt	G	15	5475.00
ggc	G	1	5676.00
gga	G	845	18772.00
ggg	G	39804	10743.00
---	--	------	------
mPD		0.041	0.68
		nPD:	0.06
		N. weight:	1.3
		Sc. PD:	0.0065
		Sc. rank:	23852.0

,

PA			
Pos.	692	obs:	exp:
act	T	2284	12628.00
acc	T	38343	7189.00
aca	T	37	17598.00
acg	T	0	3248.00
---	-	1	1.00
---	--	------	------
mPD		0.11	0.68
		nPD:	0.16
		N. weight:	1.6
		Sc. PD:	0.16
		Sc. rank:	43746.0

,

PA			
Pos.	693	obs:	exp:
ttt	F	40553	19780.00
ttc	F	111	20884.00
---	-	1	1.00
---	--	------	------
mPD		0.0054	0.50
		nPD:	0.01
		N. weight:	0.78
		Sc. PD:	-0.034
		Sc. rank:	-12118.0

,

PA			
Pos.	694	obs:	exp:
tat	Y	6	3.16
tac	Y	0	2.84
aat	N	13	7.27
aac	N	0	5.73
gct	A	1	0.27
gcc	A	0	0.25
gca	A	0	0.40
gcg	A	0	0.07
gat	D	40550	22974.00
gac	D	94	17670.00
ggt	G	1	0.13
ggc	G	0	0.14
gga	G	0	0.46
ggg	G	0	0.26
---	--	------	------
mPD		0.0056	0.49
		nPD:	0.01
		N. weight:	0.61
		Sc. PD:	-0.027
		Sc. rank:	-9067.1

,

PA			
Pos.	695	obs:	exp:
ttt	F	1	0.49
ttc	F	0	0.51
tta	L	0	4718.00
ttg	L	0	8406.00
ctt	L	40494	8506.00
ctc	L	166	5963.00
cta	L	1	6244.00
ctg	L	0	6824.00
att	I	3	1.05
atc	I	0	0.79
ata	I	0	1.16
---	--	------	------
mPD		0.0084	1.2
		nPD:	0.01
		N. weight:	1.7
		Sc. PD:	-0.081
		Sc. rank:	-33122.0

,

PA

Pos.	696	obs:	exp:
cgt	R	0	1.25
cgc	R	0	2.27
cga	R	0	5.75
cgg	R	0	5.41
aaa	K	0	0.66
aag	K	1	0.34
aga	R	1	30.90
agg	R	62	17.43
gtt	V	0	7.51
gtc	V	0	6.11
gta	V	0	6.50
gtg	V	30	9.87
gct	A	0	0.27
gcc	A	0	0.25
gca	A	0	0.40
gcg	A	1	0.07
gaa	E	0	66.03
gag	E	112	45.97
ggt	G	30	5447.00
ggc	G	3	5647.00
gga	G	187	18676.00
ggg	G	40238	10689.00
---	--	------	------
mPD		0.021	0.69
		nPD:	0.03
		N. weight:	1.4
		Sc. PD:	-0.035
		Sc. rank:	7748.0

,

PA

Pos.	697	obs:	exp:
cgt	R	0	0.04
cgc	R	0	0.07
cga	R	0	0.18
cgg	R	0	0.17
aga	R	0	0.98
agg	R	2	0.55
ggt	G	5	5474.00
ggc	G	1	5675.00
gga	G	145	18771.00
ggg	G	40512	10743.00
---	--	------	------
mPD		0.0075	0.68
		nPD:	0.01
		N. weight:	1.4
		Sc. PD:	-0.063
		Sc. rank:	-22099.0

,

PA

Pos.	698	obs:	exp:
tta	L	75	4719.00
ttg	L	0	8407.00
ctt	L	0	8506.00
ctc	L	0	5963.00
cta	L	40524	6244.00
ctg	L	64	6824.00
caa	Q	1	0.61
cag	Q	0	0.39
gtt	V	0	0.25
gtc	V	0	0.20
gta	V	1	0.22
gtg	V	0	0.33
---	--	------	------
mPD		0.0069	1.2
		nPD:	0.01
		N. weight:	2.
		Sc. PD:	-0.099
		Sc. rank:	-44731.0

,

PA

Pos.	699	obs:	exp:
tat	Y	40525	21435.00
tac	Y	140	19230.00
---	--	------	------
mPD		0.0069	0.50
		nPD:	0.01
		N. weight:	0.68
		Sc. PD:	-0.028
		Sc. rank:	-7782.7

,

PA

Pos.	700	obs:	exp:
aaa	K	12	7.89
aag	K	0	4.11
gtt	V	0	0.25
gtc	V	0	0.20
gta	V	1	0.22
gtg	V	0	0.33
gct	A	0	0.27
gcc	A	0	0.25
gca	A	1	0.40
gcg	A	0	0.07
gat	D	4	3.39
gac	D	2	2.61
gaa	E	40526	23920.00
gag	E	47	16653.00
ggt	G	0	9.69
ggc	G	0	10.05
gga	G	72	33.24
ggg	G	0	19.02
---	--	------	------
mPD		0.0068	0.49
		nPD:	0.01
		N. weight:	0.58
		Sc. PD:	-0.023
		Sc. rank:	-6418.5

,

PA

Pos.	701	obs:	exp:
tct	S	0	0.48
tcc	S	0	0.45
tca	S	3	0.81
tcg	S	0	0.12
act	T	0	0.31
acc	T	0	0.18
aca	T	1	0.43
acg	T	0	0.08
agt	S	0	0.52
agc	S	0	0.62
gct	A	2	11082.00
gcc	A	0	10159.00
gca	A	40658	16448.00
gcg	A	1	2972.00
---	--	------	------
mPD		0.00034	0.69
		nPD:	0.
		N. weight:	1.
		Sc. PD:	-0.054
		Sc. rank:	-31296.0

,

PA

Pos.	702	obs:	exp:
att	I	40424	14271.00
atc	I	227	10682.00
ata	I	3	15700.00
atg	M	1	1.00
act	T	0	0.31
acc	T	1	0.18
aca	T	0	0.43
acg	T	0	0.08
gtt	V	9	2.25
gtc	V	0	1.83
gta	V	0	1.95
gtg	V	0	2.96
---	--	------	------
mPD		0.012	0.66
		nPD:	0.02
		N. weight:	1.1
		Sc. PD:	-0.041
		Sc. rank:	-7310.8

,

PA

Pos.	703	obs:	exp:
gaa	E	12	23975.00
gag	E	40653	16690.00
---	--	------	------
mPD		0.00059	0.48
		nPD:	0.
		N. weight:	0.98
		Sc. PD:	-0.052
		Sc. rank:	-29697.0

,

PA

Pos.	704	obs:	exp:
gaa	E	98	23975.00
gag	E	40567	16690.00
---	--	------	------
mPD		0.0048	0.48
		nPD:	0.01
		N. weight:	0.97
		Sc. PD:	-0.043
		Sc. rank:	-15950.0

,

PA

Pos.	705	obs:	exp:
tgt	C	99	15891.00
tgc	C	40565	24773.00
cgt	R	0	0.02
cgc	R	1	0.04
cga	R	0	0.09
cgg	R	0	0.09
aga	R	0	0.49
agg	R	0	0.28
---	--	------	------
mPD		0.0049	0.48
		nPD:	0.01
		N. weight:	0.53
		Sc. PD:	-0.024
		Sc. rank:	-8672.0

,

PA

Pos.	706	obs:	exp:
tta	L	0	4719.00
ttg	L	24	8407.00
ctt	L	4	8506.00
ctc	L	0	5963.00
cta	L	45	6244.00
ctg	L	40591	6825.00
atg	M	1	1.00
---	--	------	------
mPD		0.0036	1.2
		nPD:	0.
		N. weight:	2.
		Sc. PD:	-0.1
		Sc. rank:	-51694.0

,

PA

Pos.	707	obs:	exp:
att	I	40652	14273.00
atc	I	8	10684.00
ata	I	0	15703.00
gtt	V	5	1.25
gtc	V	0	1.02
gta	V	0	1.08
gtg	V	0	1.65
---	--	------	------
mPD		0.00064	0.66
		nPD:	0.
		N. weight:	1.2
		Sc. PD:	-0.062
		Sc. rank:	-35140.0

,

PA

Pos.	708	obs:	exp:
tct	S	0	0.48
tcc	S	0	0.45
tca	S	0	0.81
tcg	S	0	0.12
aat	N	40621	22749.00
aac	N	40	17912.00
agt	S	3	0.52
agc	S	0	0.62
---	-	1	1.00
---	--	------	------
mPD		0.0021	0.49
		nPD:	0.
		N. weight:	0.63
		Sc. PD:	-0.032
		Sc. rank:	-15321.0

,

PA

Pos.	709	obs:	exp:
aat	N	5	2.80
aac	N	0	2.20
gat	D	40634	22983.00
gac	D	25	17676.00
gaa	E	1	0.59
gag	E	0	0.41
---	--	------	------
mPD		0.0015	0.49
		nPD:	0.
		N. weight:	0.63
		Sc. PD:	-0.032
		Sc. rank:	-16621.0

,

PA

Pos.	710	obs:	exp:
tct	S	0	0.16
tcc	S	1	0.15
tca	S	0	0.27
tcg	S	0	0.04
cct	P	30	13551.00
ccc	P	40629	7623.00
cca	P	5	13738.00
ccg	P	0	5751.00
agt	S	0	0.17
agc	S	0	0.21
---	--	------	------
mPD		0.0018	0.72
		nPD:	0.
		N. weight:	1.8
		Sc. PD:	-0.096
		Sc. rank:	-52319.0

,

PA

Pos.	711	obs:	exp:
tgg	W	40665	40665.00
---	--	------	------
mPD		0	0
		nPD:	1.
		N. weight:	0.
		Sc. PD:	0
		Sc. rank:	0

,

PA

Pos. 712		obs:	exp:
gtt	V	40658	10185.00
gtc	V	6	8286.00
gta	V	0	8814.00
gtg	V	0	13379.00
gct	A	1	0.27
gcc	A	0	0.25
gca	A	0	0.40
gcg	A	0	0.07
---	--	------	------
mPD		0.00034	0.74
		nPD:	0.
		N. weight:	1.5
		Sc. PD:	-0.083
		Sc. rank:	-48089.0

,

PA

Pos. 713		obs:	exp:
ttt	F	1	0.49
ttc	F	0	0.51
tta	L	8	4719.00
ttg	L	40651	8407.00
ctt	L	0	8506.00
ctc	L	0	5963.00
cta	L	0	6244.00
ctg	L	5	6825.00
---	--	------	------
mPD		0.00069	1.2
		nPD:	0.
		N. weight:	1.7
		Sc. PD:	-0.094
		Sc. rank:	-53894.0

,

PA

Pos. 714		obs:	exp:
tta	L	0	4719.00
ttg	L	0	8407.00
ctt	L	331	8506.00
ctc	L	40267	5963.00
cta	L	66	6244.00
ctg	L	0	6825.00
att	I	0	0.35
atc	I	1	0.26
ata	I	0	0.39
---	--	------	------
mPD		0.019	1.2
		nPD:	0.02
		N. weight:	2.1
		Sc. PD:	-0.078
		Sc. rank:	-14654.0

,

PA

Pos. 715		obs:	exp:
tct	S	0	0.32
tcc	S	0	0.30
tca	S	0	0.54
tcg	S	0	0.08
cat	H	1	0.56
cac	H	0	0.44
act	T	1	0.31
acc	T	0	0.18
aca	T	0	0.43
acg	T	0	0.08
aat	N	40633	22749.00
aac	N	28	17912.00
agt	S	2	0.35
agc	S	0	0.41
---	--	------	------
mPD		0.0016	0.49
		nPD:	0.
		N. weight:	0.64
		Sc. PD:	-0.033
		Sc. rank:	-16644.0

,

PA

Pos. 716		obs:	exp:
tct	S	0	0.16
tcc	S	0	0.15
tca	S	1	0.27
tcg	S	0	0.04
agt	S	0	0.17
agc	S	0	0.21
gtt	V	0	0.50
gtc	V	0	0.41
gta	V	0	0.43
gtg	V	2	0.66
gct	A	64	11082.00
gcc	A	0	10159.00
gca	A	8179	16448.00
gcg	A	32418	2972.00
ggt	G	0	0.13
ggc	G	0	0.14
gga	G	0	0.46
ggg	G	1	0.26
---	--	------	------
mPD		0.32	0.69
		nPD:	0.47
		N. weight:	1.9
		Sc. PD:	0.77
		Sc. rank:	60334.0

,

PA

Pos. 717		obs:	exp:
ttt	F	3	1.46
ttc	F	0	1.54
tct	S	40526	6561.00
tcc	S	88	6076.00
tca	S	3	10929.00
tcg	S	44	1601.00
cct	P	1	0.33
ccc	P	0	0.19
cca	P	0	0.34
ccg	P	0	0.14
agt	S	0	7106.00
agc	S	0	8388.00
---	--	------	------
mPD		0.0068	1.6
		nPD:	0.
		N. weight:	2.
		Sc. PD:	-0.1
		Sc. rank:	-48680.0

,

PA

Pos. 718		obs:	exp:
tgg	W	40664	40664.00
cgt	R	0	0.02
cgc	R	0	0.04
cga	R	0	0.09
cgg	R	1	0.09
aga	R	0	0.49
agg	R	0	0.28
---	--	------	------
mPD		0.000049	0.000081
		nPD:	0.61
		N. weight:	0.000067
		Sc. PD:	0.000036
		Sc. rank:	2.1

,

PA

Pos. 719		obs:	exp:
ttt	F	34	19777.00
ttc	F	40624	20881.00
tta	L	3	0.70
ttg	L	0	1.24
ctt	L	0	1.26
ctc	L	3	0.88
cta	L	0	0.92
ctg	L	0	1.01
gtt	V	0	0.25
gtc	V	1	0.20
gta	V	0	0.22
gtg	V	0	0.33
---	--	------	------
mPD		0.0020	0.50
		nPD:	0.
		N. weight:	0.73
		Sc. PD:	-0.037
		Sc. rank:	-18019.0

,

PA

Pos. 720		obs:	exp:
aat	N	1	22751.00
aac	N	40664	17914.00
---	--	------	------
mPD		0.000049	0.49
		nPD:	0.
		N. weight:	0.91
		Sc. PD:	-0.049
		Sc. rank:	-28838.0

,

PA

Pos. 721		obs:	exp:
ttt	F	0	0.97
ttc	F	2	1.03
tct	S	0	6562.00
tcc	S	40661	6076.00
tca	S	1	10929.00
tcg	S	0	1601.00
agt	S	0	7106.00
agc	S	0	8388.00
gct	A	0	0.27
gcc	A	1	0.25
gca	A	0	0.40
gcg	A	0	0.07
---	--	------	------
mPD		0.00020	1.6
		nPD:	0.
		N. weight:	2.1
		Sc. PD:	-0.11
		Sc. rank:	-66493.0

,

PA

Pos. 722		obs:	exp:
ttt	F	3	19779.00
ttc	F	40659	20883.00
tta	L	0	0.35
ttg	L	0	0.62
ctt	L	0	0.63
ctc	L	3	0.44
cta	L	0	0.46
ctg	L	0	0.50
---	--	------	------
mPD		0.00030	0.50
		nPD:	0.
		N. weight:	0.74
		Sc. PD:	-0.04
		Sc. rank:	-22915.0

,

PA

Pos. 723		obs:	exp:
tta	L	0	4717.00
ttg	L	444	8405.00
ctt	L	1532	8504.00
ctc	L	203	5961.00
cta	L	217	6243.00
ctg	L	38257	6823.00
atg	M	12	12.00
---	--	------	------
mPD		0.11	1.2
		nPD:	0.1
		N. weight:	1.6
		Sc. PD:	0.068
		Sc. rank:	37957.0

,

PA			
Pos.	724	obs:	exp:
att	I	0	22.12
atc	I	0	16.55
ata	I	63	24.33
atg	M	2	2.00
act	T	7	12558.00
acc	T	1	7150.00
aca	T	39159	17501.00
acg	T	1271	3230.00
aaa	K	42	28.27
aag	K	1	14.73
gct	A	0	32.43
gcc	A	0	29.73
gca	A	118	48.14
gcg	A	1	8.70
---	--	------	------
mPD		0.072	0.69
		nPD:	0.1
		N. weight:	0.83
		Sc. PD:	0.039
		Sc. rank:	19758.0

,

PA			
Pos.	725	obs:	exp:
tta	L	0	0.12
ttg	L	0	0.21
tat	Y	6	3.16
tac	Y	0	2.84
ctt	L	1	0.21
ctc	L	0	0.15
cta	L	0	0.15
ctg	L	0	0.17
cat	H	40603	22930.00
cac	H	50	17723.00
caa	Q	1	1.22
cag	Q	1	0.78
cgt	R	3	0.06
cgc	R	0	0.11
cga	R	0	0.27
cgg	R	0	0.26
aga	R	0	1.47
agg	R	0	0.83
---	--	------	------
mPD		0.0030	0.49
		nPD:	0.01
		N. weight:	0.62
		Sc. PD:	-0.03
		Sc. rank:	-13522.0

,

PA			
Pos.	726	obs:	exp:
tct	S	0	0.48
tcc	S	0	0.45
tca	S	3	0.81
tcg	S	0	0.12
act	T	0	2.79
acc	T	0	1.59
aca	T	9	3.89
acg	T	0	0.72
agt	S	0	0.52
agc	S	0	0.62
gtt	V	0	0.25
gtc	V	0	0.20
gta	V	1	0.22
gtg	V	0	0.33
gct	A	7	11079.00
gcc	A	0	10156.00
gca	A	40638	16443.00
gcg	A	5	2972.00
gaa	E	2	1.18
gag	E	0	0.82
---	--	------	------
mPD		0.0013	0.70
		nPD:	0.
		N. weight:	1.
		Sc. PD:	-0.052
		Sc. rank:	-29500.0

,

PA			
Pos.	727	obs:	exp:
ttt	F	2	0.97
ttc	F	0	1.03
tta	L	36754	4718.00
ttg	L	275	8406.00
tct	S	0	0.81
tcc	S	0	0.75
tca	S	5	1.34
tcg	S	0	0.20
ctt	L	0	8505.00
ctc	L	0	5962.00
cta	L	3440	6243.00
ctg	L	189	6824.00
agt	S	0	0.87
agc	S	0	1.03
---	--	------	------
mPD		0.19	1.2
		nPD:	0.16
		N. weight:	2.
		Sc. PD:	0.2
		Sc. rank:	52633.0

,

PA			
Pos.	728	obs:	exp:
tct	S	0	0.97
tcc	S	0	0.90
tca	S	0	1.61
tcg	S	0	0.24
caa	Q	1	0.61
cag	Q	0	0.39
cgt	R	0	2.78
cgc	R	0	5.04
cga	R	0	12.77
cgg	R	0	12.02
att	I	0	3.16
atc	I	0	2.36
ata	I	9	3.48
aat	N	20	16.78
aac	N	10	13.22
aaa	K	40267	26561.00
aag	K	134	13840.00
agt	S	6	1.05
agc	S	0	1.24
aga	R	140	68.67
agg	R	0	38.72
gat	D	2	1.13
gac	D	0	0.87
gaa	E	69	41.27
gag	E	1	28.73
ggt	G	0	0.81
ggc	G	0	0.84
gga	G	6	2.77
ggg	G	0	1.59
---	--	------	------
mPD		0.020	0.47
		nPD:	0.04
		N. weight:	0.44
		Sc. PD:	-0.0054
		Sc. rank:	5458.7

,

PA			
Pos.	729	obs:	exp:
tat	Y	1	0.53
tac	Y	0	0.47
tgg	W	44	44.00
caa	Q	0	0.61
cag	Q	1	0.39
---	-	40619	40619.00
---	--	------	------
mPD		2.2×10^{-7}	2.5×10^{-7}
		nPD:	0.87
		N. weight:	0.
		Sc. PD:	0
		Sc. rank:	0

,

PA			
Pos.	730	obs:	exp:
tta	L	47	5.45
ttg	L	0	9.72
ctt	L	0	9.83
ctc	L	0	6.89
cta	L	0	7.22
ctg	L	0	7.89
---	-	40618	40618.00
---	--	------	------
mPD		0	1.5×10^{-6}
		nPD:	0.
		N. weight:	0.
		Sc. PD:	0
		Sc. rank:	0

,

PA			
Pos.	731	obs:	exp:
taa	.	292	23101.00
tag	.	40373	4454.00
tga	.	0	13110.00
---	--	------	------
mPD		0.014	0.63
		nPD:	0.02
		N. weight:	2.4
		Sc. PD:	-0.078
		Sc. rank:	-3214.7

}

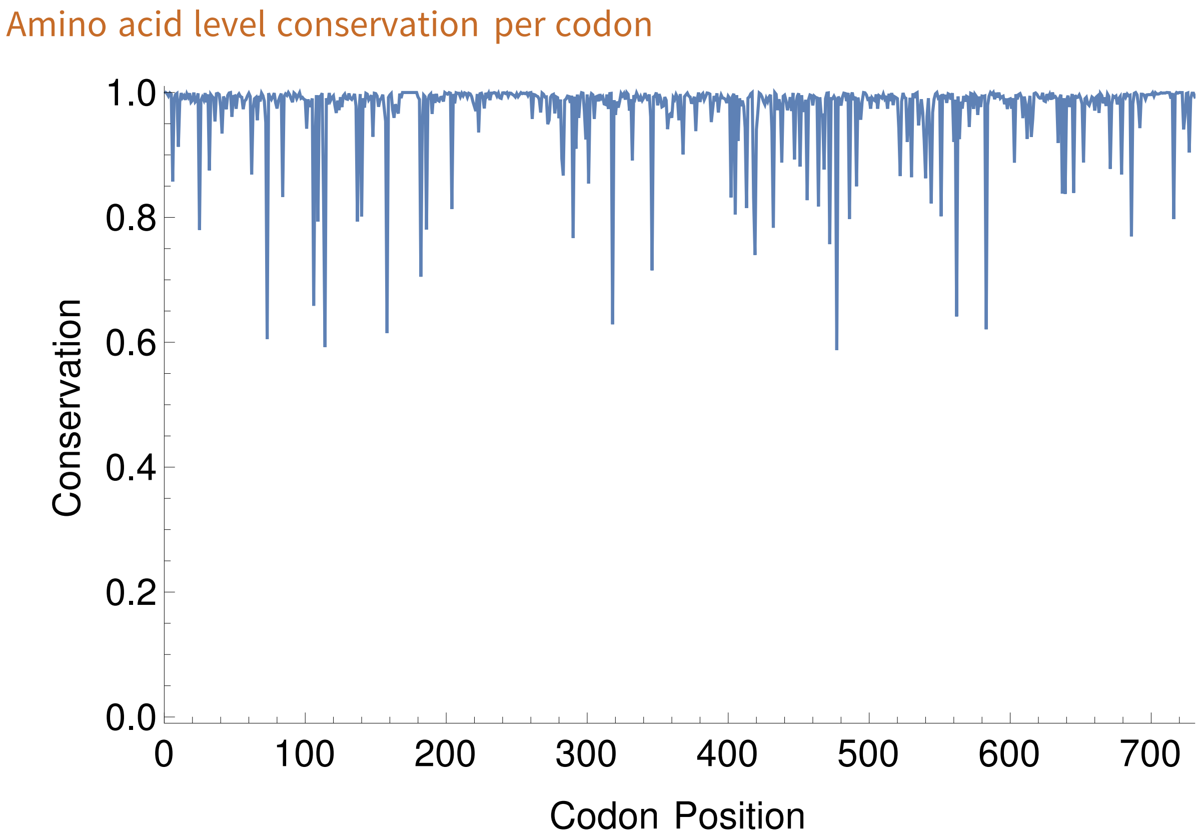
Amino acid level conservation per codon
Conservation
1.0
0.8
0.6
0.4
0.2
0.0
0
100
200
300
400
500
600
700
Codon Position

Conservation analysis plots

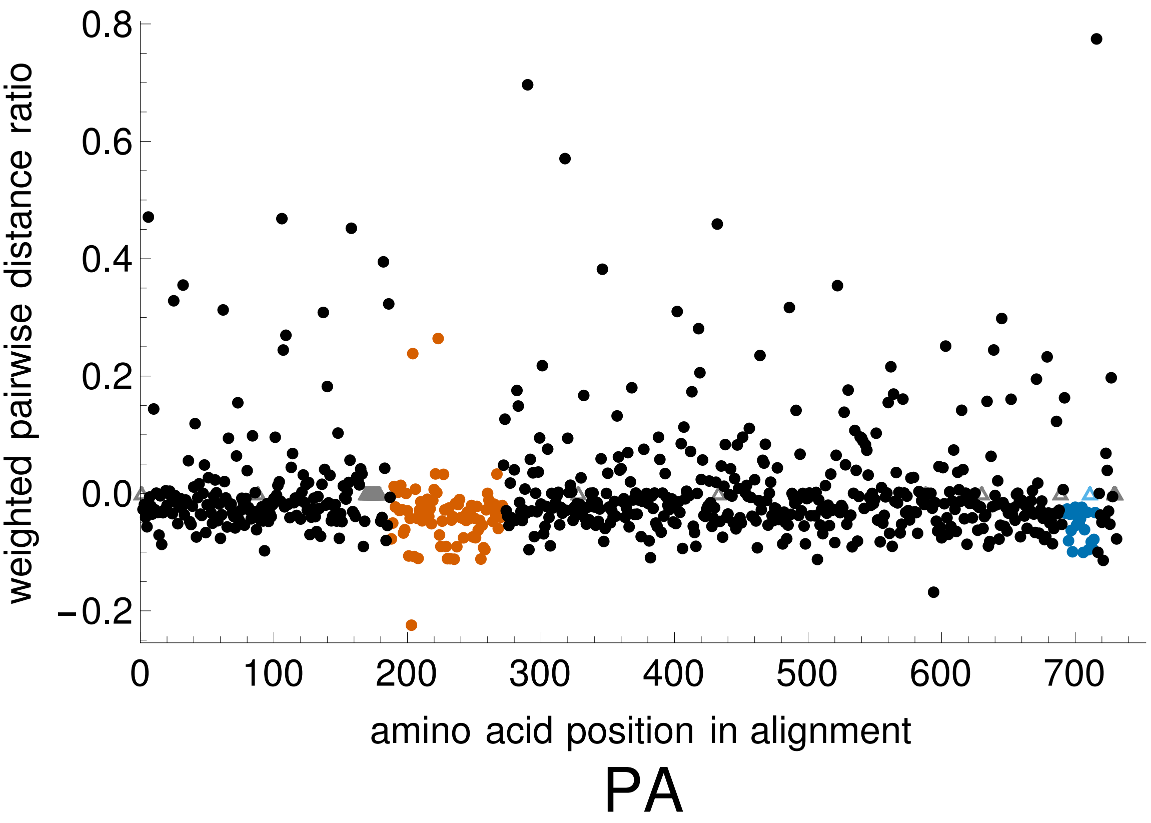

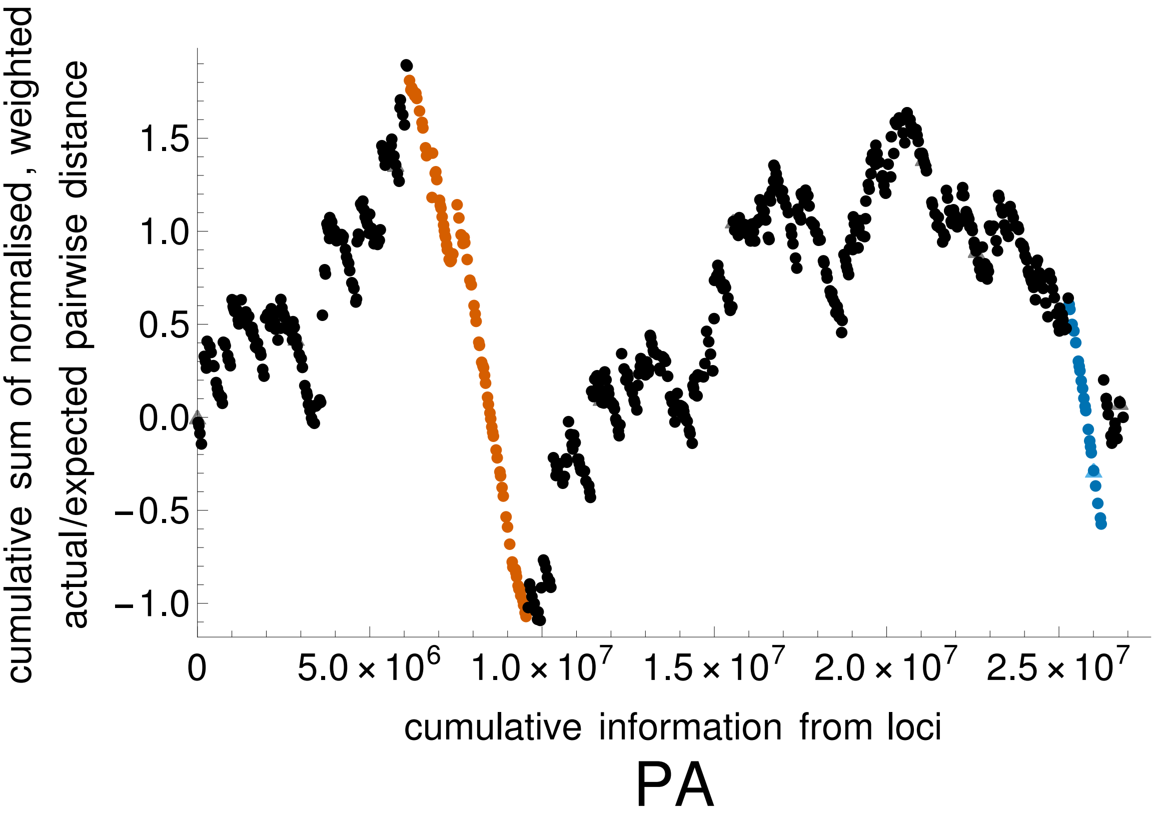

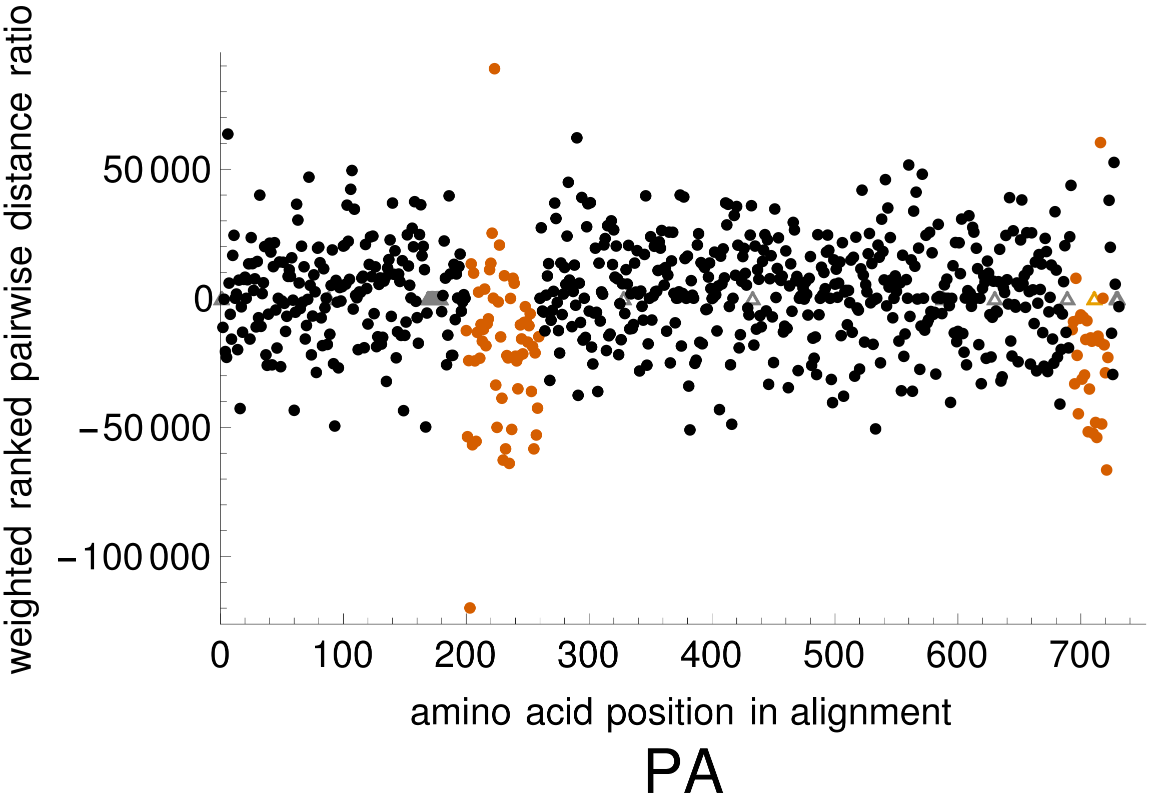
weighted ranked pairwise distance ratio
50 000
0
−50 000
−100 000
0
100
200
300
400
500
600
700
amino acid position in alignment
PA

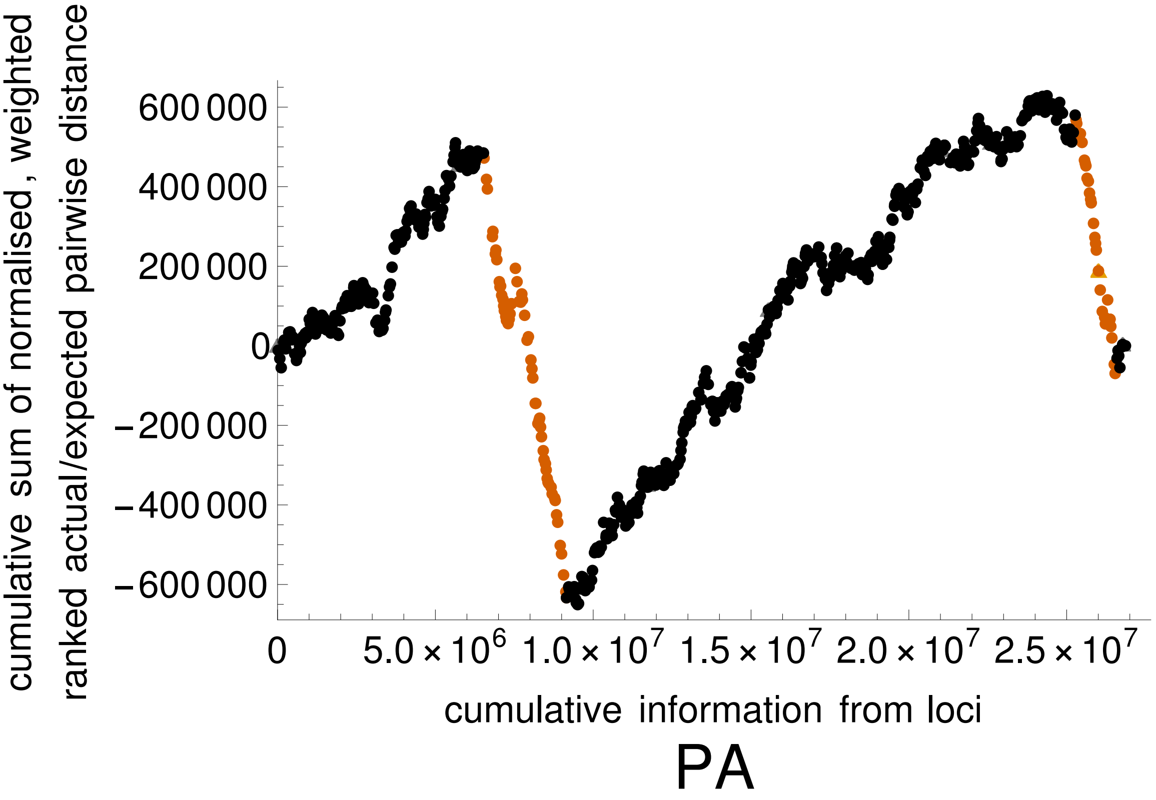
cumulative sum of normalised, weighted ranked actual/expected pairwise distance
600 000
400 000
200 000
0
−200 000
−400 000
−600 000
0
5.0 × 10^6
1.0 × 10^7
1.5 × 10^7
2.0 × 10^7
2.5 × 10^7
cumulative information from loci
PA

PA-X

Gene length histogram

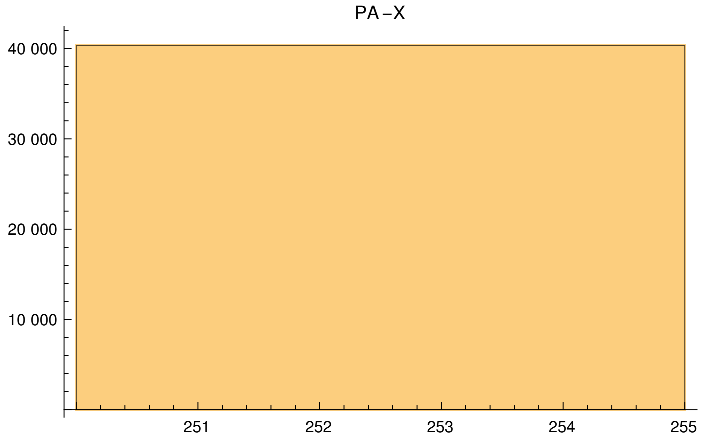

Information vs. nPD

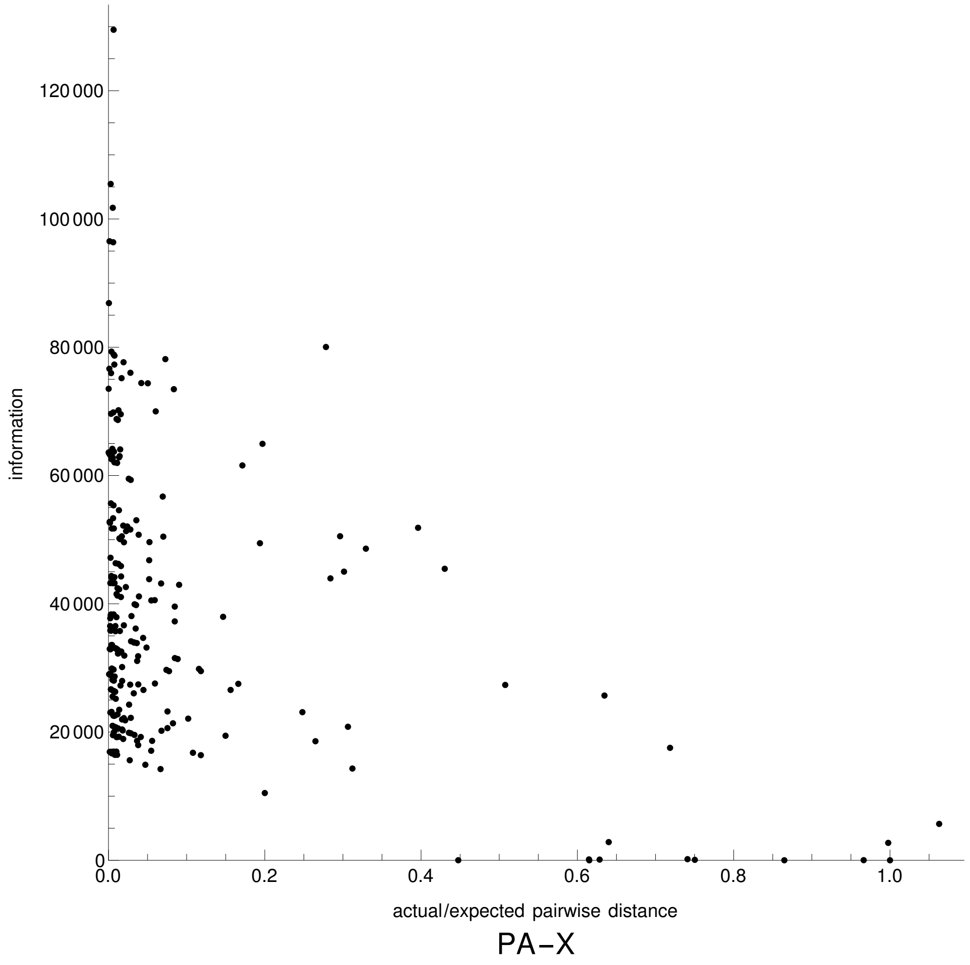

Example sequences highlighted by regions found in analyses to be conserved

Interesting points (by weighted raw PD) highlighted for gene PA-X:

ATGGAAGATTTTGTGCGACAATGCTTCAACCCGATGATTGTCGAACTTGCAGAAAAAGCAATGAAAGAGTATGGGGAG
GATCTGAAAATTGAAACCAACAAATTTGCAGCAATATGCACTCACTTGGAGGTGTGTTTCATGTATTCAGATTTCCA
TTTCATCAATGAACAAGGCGAATCAATAGTAGTAGAACTTGACGATCCAAATGCACTGTTAAAACACAGATTTGAAA
TAATCGAGGGGAGAGACAGAACAATGGCCTGGACAGTAGTAAACAGTATCTGCAACACTACTGGAGCTGGAAAA ---
--CCGAAGTTCTTACCGGATTTGTATGATTACAAAGAAAACAGATTCATCGAAATTGGAGTGACAAGGAGAGAAGT
CCACATATATTACCTTGAAAAGGCCAATAAGATTAAATCTGAGAACACACACATTCACATTTTTTCATTCACTGG
GGAGGAAATGGCCACAAGGGCAGACTACACTCTCGACGAGGAAAGCAGGGCTAGGATCAAAACCAGGCTGTTTAC
CATAAGACAAGAAATGGCCAACAAAGGCCTCTGGGATTCCTTTGTCAGT
**CCGAAAGAGGCGAAGAAACAATTGAAGAAAAATTTGAAATCACAGGAACTATGCG
CAGGCTTGCCGACCAAAGTCTCCCACCGAACTTCTCCTGCCTTGAGAATTTT
AGAGCCTATGTGGATGGATTCGAACCGAACGGCTGCATTGAGGGCAAGCTTT
CTCAAATGT**CCAAAGAAG ------------------------TGA

Interesting points (by weighted ranked PD) highlighted for gene PA-X:

ATGGAAGATTTTGTGCGACAATGCTTCAACCCGATGATTGTCGAACTTGCAGAAAAAGCAATGAAAGAGTATGGGGAG
GATCTGAAAATTGAAACCAACAAATTTGCAGCAATATGCACTCACTTGGAGGTGTGTTTCATGTATTCAGATTTCCA
TTTCATCAATGAACAAGGCGAATCAATAGTAGTAGAACTTGACGATCCAAATGCACTGTTAAAACACAGATTTGAAA
TAATCGAGGGGAGAGACAGAACAATGGCCTGGACAGTAGTAAACAGTATCTGCAACACTACTGGAGCTGGAAAA ---
--CCGAAGTTCTTACCGGATTTGTATGATTACAAAGAAAACAGATTCATCGAAATTGGAGTGACAAGGAGAGAAGT
CCACATATATTACCTTGAAAAGGCCAATAAGATTAAATCTGAGAACACACACATTCACATTTTTTCATTCACTGG
GGAGGAAATGGCCACAAGGGCAGACTACACTCTCGACGAGGAAAGCAGGGCTAGGATCAAAACCAGGCTGTTTAC
CATAAGACAAGAAATGGCCAACAAAGGCCTCTGG
GATTCCTTTGTCAGTCCGAAAGAGGCGAAGAAACAATTGAAGAAAAATTTGAAA
TCACAGGAACTATGCGCAGGCTTGCCGACCAAAGTC
**TCCCACCGAACTTCTCCTGCCTTGAGAATTTTAGAGCCTATGTGGATGGATTCGA
ACCGAACGGCTGCATTGAGGGCAAGCTTTCTCAAATGT**CCAAAGAAG ------------
------------TGA

Per codon conservation report

PA-X

Pos.	1	obs:	exp:
atg	M	40345	40345.00
---	--	------	------
mPD		0	0
		nPD:	1.
		N. weight:	0.
		Sc. PD:	0
		Sc. rank:	0

PA-X

Pos.	2	obs:	exp:
gaa	E	40275	23784.00
gag	E	67	16558.00
ggt	G	0	0.27
ggc	G	0	0.28
gga	G	2	0.92
ggg	G	0	0.53
---	-	1	1.00
---	--	------	------
mPD		0.0034	0.48
		nPD:	0.01
		N. weight:	0.57
		Sc. PD:	-0.02
		Sc. rank:	-4791.8

PA-X

Pos.	3	obs:	exp:
tat	Y	2	1.05
tac	Y	0	0.95
aat	N	16	8.95
aac	N	0	7.05
gat	D	40258	22790.00
gac	D	60	17528.00
gaa	E	2	1.18
gag	E	0	0.82
ggt	G	6	0.81
ggc	G	0	0.84
gga	G	0	2.77
ggg	G	0	1.59
---	-	1	1.00
---	--	------	------
mPD		0.0043	0.49
		nPD:	0.01
		N. weight:	0.61
		Sc. PD:	-0.021
		Sc. rank:	-3130.9

PA-X

Pos.	4	obs:	exp:
ttt	F	40311	19622.00
ttc	F	29	20718.00
tta	L	1	0.46
ttg	L	0	0.83
ctt	L	3	0.84
ctc	L	0	0.59
cta	L	0	0.61
ctg	L	0	0.67
---	-	1	1.00
---	--	------	------
mPD		0.0016	0.50
		nPD:	0.
		N. weight:	0.78
		Sc. PD:	-0.03
		Sc. rank:	-16809.0

PA-X

Pos.	5	obs:	exp:
tta	L	0	0.23
ttg	L	2	0.41
ctt	L	0	0.42
ctc	L	0	0.29
cta	L	0	0.31
ctg	L	0	0.34
atg	M	2	2.00
gtt	V	0	10104.00
gtc	V	0	8219.00
gta	V	109	8743.00
gtg	V	40229	13272.00
gct	A	0	0.55
gcc	A	0	0.50
gca	A	0	0.81
gcg	A	2	0.15
---	-	1	1.00
---	--	------	------
mPD		0.0057	0.74
		nPD:	0.01
		N. weight:	1.2
		Sc. PD:	-0.041
		Sc. rank:	-8080.9

PA-X

Pos.	6	obs:	exp:
cgt	R	1	799.90
cgc	R	3	1452.00
cga	R	34552	3681.00
cgg	R	5752	3464.00
aga	R	34	19788.00
agg	R	2	11159.00
---	-	1	1.00
---	--	------	------
mPD		0.25	0.89
		nPD:	0.28
		N. weight:	2.2
		Sc. PD:	0.48
		Sc. rank:	63179.0

PA-X

Pos.	7	obs:	exp:
tta	L	0	0.12
ttg	L	0	0.21
ctt	L	0	0.21
ctc	L	0	0.15
cta	L	1	0.15
ctg	L	0	0.17
caa	Q	39976	24658.00
cag	Q	363	15681.00
cgt	R	0	0.06
cgc	R	0	0.11
cga	R	3	0.27
cgg	R	0	0.26
aaa	K	1	0.66
aag	K	0	0.34
aga	R	0	1.47
agg	R	0	0.83
---	-	1	1.00
---	--	------	------
mPD		0.018	0.48
		nPD:	0.04
		N. weight:	0.49
		Sc. PD:	-0.0028
		Sc. rank:	7989.7

PA-X

Pos.	8	obs:	exp:
tct	S	0	0.81
tcc	S	1	0.75
tca	S	0	1.34
tcg	S	0	0.20
tat	Y	0	3.69
tac	Y	7	3.31
tgt	C	115	15761.00
tgc	C	40216	24570.00
cat	H	0	0.56
cac	H	1	0.44
agt	S	0	0.87
agc	S	4	1.03
---	-	1	1.00
---	--	------	------
mPD		0.0064	0.48
		nPD:	0.01
		N. weight:	0.52
		Sc. PD:	-0.015
		Sc. rank:	645.1

PA-X

Pos.	9	obs:	exp:
ttt	F	60	19624.00
ttc	F	40283	20719.00
tat	Y	0	0.53
tac	Y	1	0.47
---	-	1	1.00
---	--	------	------
mPD		0.0030	0.50
		nPD:	0.01
		N. weight:	0.72
		Sc. PD:	-0.026
		Sc. rank:	-9530.2

PA-X

Pos.	10	obs:	exp:
tct	S	0	0.32
tcc	S	0	0.30
tca	S	0	0.54
tcg	S	0	0.08
att	I	0	0.35
atc	I	1	0.26
ata	I	0	0.39
aat	N	3318	22570.00
aac	N	37023	17771.00
agt	S	0	0.35
agc	S	2	0.41
---	-	1	1.00
---	--	------	------
mPD		0.15	0.49
		nPD:	0.31
		N. weight:	0.56
		Sc. PD:	0.14
		Sc. rank:	17031.0

PA-X

Pos.	11	obs:	exp:
tta	L	0	0.12
ttg	L	0	0.21
ctt	L	0	0.21
ctc	L	0	0.15
cta	L	0	0.15
ctg	L	1	0.17
cct	P	141	13443.00
ccc	P	0	7563.00
cca	P	267	13630.00
ccg	P	39934	5706.00
ggt	G	0	0.13
ggc	G	0	0.14
gga	G	0	0.46
ggg	G	1	0.26
---	-	1	1.00
---	--	------	------
mPD		0.020	0.72
		nPD:	0.03
		N. weight:	2.1
		Sc. PD:	-0.031
		Sc. rank:	25814.0

PA-X

Pos.	12	obs:	exp:
tta	L	0	0.23
ttg	L	2	0.41
ctt	L	0	0.42
ctc	L	0	0.29
cta	L	0	0.31
ctg	L	0	0.34
att	I	6	11.94
atc	I	0	8.93
ata	I	28	13.13
atg	M	40308	40308.00
---	-	1	1.00
---	--	------	------
mPD		0.0018	0.0018
		nPD:	0.97
		N. weight:	0.0006
		Sc. PD:	0.00052
		Sc. rank:	18.9

PA-X

Pos.	13	obs:	exp:
att	I	40207	14153.00
atc	I	107	10593.00
ata	I	2	15570.00
gtt	V	29	7.26
gtc	V	0	5.91
gta	V	0	6.29
gtg	V	0	9.54
---	--	------	------
mPD		0.0068	0.66
		nPD:	0.01
		N. weight:	1.1
		Sc. PD:	-0.036
		Sc. rank:	-3574.6

PA-X

Pos.	14	obs:	exp:
att	I	0	1.05
atc	I	3	0.79
ata	I	0	1.16
gtt	V	189	10105.00
gtc	V	40141	8220.00
gta	V	11	8744.00
gtg	V	0	13273.00
gct	A	0	0.27
gcc	A	1	0.25
gca	A	0	0.40
gcg	A	0	0.07
---	--	------	------
mPD		0.010	0.74
		nPD:	0.01
		N. weight:	1.7
		Sc. PD:	-0.049
		Sc. rank:	2809.7

PA-X

Pos.	15	obs:	exp:
gat	D	11	6.78
gac	D	1	5.22
gaa	E	40064	23779.00
gag	E	269	16554.00
---	--	------	------
mPD		0.014	0.48
		nPD:	0.03
		N. weight:	0.54
		Sc. PD:	-0.0079
		Sc. rank:	7082.1

PA-X

Pos.	16	obs:	exp:
ttt	F	2	0.97
ttc	F	0	1.03
tta	L	0	4680.00
ttg	L	0	8338.00
ctt	L	40254	8437.00
ctc	L	49	5914.00
cta	L	28	6193.00
ctg	L	0	6769.00
cat	H	1	0.56
cac	H	0	0.44
cgt	R	1	0.02
cgc	R	0	0.04
cga	R	0	0.09
cgg	R	0	0.09
att	I	10	3.51
atc	I	0	2.63
ata	I	0	3.86
aga	R	0	0.49
agg	R	0	0.28
---	--	------	------
mPD		0.0045	1.2
		nPD:	0.
		N. weight:	1.7
		Sc. PD:	-0.065
		Sc. rank:	-31877.0

,

PA-X

Pos.	17	obs:	exp:
tct	S	0	0.16
tcc	S	0	0.15
tca	S	1	0.27
tcg	S	0	0.04
act	T	0	0.62
acc	T	0	0.35
aca	T	2	0.87
acg	T	0	0.16
aaa	K	1	0.66
aag	K	0	0.34
agt	S	0	0.17
agc	S	0	0.21
gtt	V	0	0.25
gtc	V	0	0.20
gta	V	1	0.22
gtg	V	0	0.33
gct	A	3	10995.00
gcc	A	16	10079.00
gca	A	40240	16318.00
gcg	A	81	2949.00
---	--	------	------
mPD		0.0052	0.69
		nPD:	0.01
		N. weight:	0.97
		Sc. PD:	-0.034
		Sc. rank:	-7255.4

,

PA-X

Pos.	18	obs:	exp:
tct	S	0	0.16
tcc	S	0	0.15
tca	S	1	0.27
tcg	S	0	0.04
agt	S	0	0.17
agc	S	0	0.21
gaa	E	40295	23785.00
gag	E	48	16558.00
ggt	G	0	0.13
ggc	G	0	0.14
gga	G	1	0.46
ggg	G	0	0.26
---	--	------	------
mPD		0.0025	0.48
		nPD:	0.01
		N. weight:	0.57
		Sc. PD:	-0.021
		Sc. rank:	-9495.3

,

PA-X

Pos.	19	obs:	exp:
cct	P	0	0.33
ccc	P	0	0.19
cca	P	1	0.34
ccg	P	0	0.14
cgt	R	0	0.32
cgc	R	0	0.58
cga	R	0	1.46
cgg	R	0	1.37
aaa	K	39915	26512.00
aag	K	412	13815.00
aga	R	13	7.85
agg	R	3	4.43
gaa	E	1	0.59
gag	E	0	0.41
---	--	------	------
mPD		0.021	0.45
		nPD:	0.05
		N. weight:	0.4
		Sc. PD:	0.0012
		Sc. rank:	7395.3

,

PA-X

Pos.	20	obs:	exp:
tct	S	0	1.29
tcc	S	0	1.20
tca	S	8	2.15
tcg	S	0	0.31
act	T	0	136.30
acc	T	0	77.62
aca	T	439	190.00
acg	T	0	35.06
agt	S	0	1.40
agc	S	0	1.65
gtt	V	0	2.00
gtc	V	0	1.63
gta	V	8	1.73
gtg	V	0	2.63
gct	A	28	10871.00
gcc	A	0	9965.00
gca	A	39838	16134.00
gcg	A	21	2916.00
gat	D	0	0.57
gac	D	1	0.43
gaa	E	2	1.18
gag	E	0	0.82
---	--	------	------
mPD		0.025	0.72
		nPD:	0.03
		N. weight:	0.98
		Sc. PD:	-0.0087
		Sc. rank:	14614.0

,

PA-X

Pos.	21	obs:	exp:
att	I	0	1.05
atc	I	0	0.79
ata	I	3	1.16
atg	M	40340	40340.00
---	-	2	2.00
---	--	------	------
mPD		0.00015	0.00015
		nPD:	1.
		N. weight:	0.000077
		Sc. PD:	0.00007
		Sc. rank:	2.5

,

PA-X

Pos.	22	obs:	exp:
cgt	R	0	0.44
cgc	R	0	0.79
cga	R	0	2.01
cgg	R	0	1.89
aaa	K	39729	26510.00
aag	K	594	13813.00
aga	R	22	10.79
agg	R	0	6.09
---	--	------	------
mPD		0.030	0.45
		nPD:	0.07
		N. weight:	0.39
		Sc. PD:	0.0082
		Sc. rank:	8271.8

,

PA-X

Pos.	23	obs:	exp:
gat	D	1	0.57
gac	D	0	0.43
gaa	E	320	23784.00
gag	E	40022	16558.00
ggt	G	0	0.27
ggc	G	0	0.28
gga	G	0	0.92
ggg	G	2	0.53
---	--	------	------
mPD		0.016	0.48
		nPD:	0.03
		N. weight:	0.92
		Sc. PD:	-0.0099
		Sc. rank:	13151.0

,

PA-X

Pos.	24	obs:	exp:
ttt	F	5	2.43
ttc	F	0	2.57
tat	Y	40292	21259.00
tac	Y	39	19072.00
cat	H	8	4.51
cac	H	0	3.49
gat	D	1	0.57
gac	D	0	0.43
---	--	------	------
mPD		0.0026	0.50
		nPD:	0.01
		N. weight:	0.69
		Sc. PD:	-0.026
		Sc. rank:	-11446.0

,

PA-X

Pos.	25	obs:	exp:
cgt	R	0	0.02
cgc	R	0	0.04
cga	R	0	0.09
cgg	R	0	0.09
aga	R	0	0.49
agg	R	1	0.28
gct	A	0	0.27
gcc	A	0	0.25
gca	A	1	0.40
gcg	A	0	0.07
ggt	G	7	5431.00
ggc	G	0	5631.00
gga	G	8927	18623.00
ggg	G	31409	10658.00
---	--	------	------
mPD		0.35	0.68
		nPD:	0.51
		N. weight:	0.74
		Sc. PD:	0.33
		Sc. rank:	23283.0

,

PA-X

Pos.	26	obs:	exp:
aaa	K	0	0.66
aag	K	1	0.34
gat	D	1	0.57
gac	D	0	0.43
gaa	E	313	23782.00
gag	E	40026	16557.00
ggt	G	0	0.54
ggc	G	0	0.56
gga	G	0	1.85
ggg	G	4	1.06
---	--	------	------
mPD		0.016	0.48
		nPD:	0.03
		N. weight:	0.92
		Sc. PD:	-0.01
		Sc. rank:	12959.0

,

PA-X

Pos.	27	obs:	exp:
cat	H	1	0.56
cac	H	0	0.44
aat	N	158	89.52
aac	N	2	70.48
gat	D	40058	22713.00
gac	D	123	17468.00
ggt	G	3	0.40
ggc	G	0	0.42
gga	G	0	1.38
ggg	G	0	0.79
---	--	------	------
mPD		0.014	0.50
		nPD:	0.03
		N. weight:	0.6
		Sc. PD:	-0.0089
		Sc. rank:	7866.4

,

PA-X

Pos.	28	obs:	exp:
tta	L	0	4677.00
ttg	L	83	8333.00
tct	S	0	0.16
tcc	S	0	0.15
tca	S	0	0.27
tcg	S	1	0.04
ctt	L	29	8432.00
ctc	L	1	5911.00
cta	L	146	6189.00
ctg	L	40047	6764.00
cct	P	0	8.33
ccc	P	1	4.69
cca	P	1	8.45
ccg	P	23	3.54
caa	Q	0	4.89
cag	Q	8	3.11
atg	M	5	5.00
agt	S	0	0.17
agc	S	0	0.21
---	--	------	------
mPD		0.015	1.2
		nPD:	0.01
		N. weight:	1.9
		Sc. PD:	-0.056
		Sc. rank:	1424.3

,

PA-X

Pos.	29	obs:	exp:
cgt	R	0	0.12
cgc	R	0	0.22
cga	R	0	0.55
cgg	R	0	0.52
aaa	K	40306	26520.00
aag	K	33	13819.00
aga	R	6	2.94
agg	R	0	1.66
---	--	------	------
mPD		0.0019	0.45
		nPD:	0.
		N. weight:	0.45
		Sc. PD:	-0.017
		Sc. rank:	-8294.2

,

PA-X

Pos.	30	obs:	exp:
tct	S	0	0.16
tcc	S	0	0.15
tca	S	0	0.27
tcg	S	0	0.04
att	I	39897	14160.00
atc	I	436	10599.00
ata	I	4	15578.00
act	T	1	0.31
acc	T	0	0.18
aca	T	0	0.43
acg	T	0	0.08
aat	N	2	1.12
aac	N	0	0.88
agt	S	0	0.17
agc	S	1	0.21
gtt	V	4	1.00
gtc	V	0	0.82
gta	V	0	0.87
gtg	V	0	1.32
---	--	------	------
mPD		0.022	0.66
		nPD:	0.03
		N. weight:	1.1
		Sc. PD:	-0.011
		Sc. rank:	15887.0

,

PA-X			
Pos.	31	obs:	exp:
aaa	K	3	1.97
aag	K	0	1.03
gtt	V	0	0.25
gtc	V	0	0.20
gta	V	1	0.22
gtg	V	0	0.33
gat	D	0	0.57
gac	D	1	0.43
gaa	E	40223	23783.00
gag	E	117	16557.00
---	--	------	------
mPD		0.0060	0.48
		nPD:	0.01
		N. weight:	0.56
		Sc. PD:	-0.017
		Sc. rank:	-80.9

,

PA-X			
Pos.	32	obs:	exp:
tct	S	0	0.16
tcc	S	0	0.15
tca	S	0	0.27
tcg	S	0	0.04
cct	P	0	0.33
ccc	P	1	0.19
cca	P	0	0.34
ccg	P	0	0.14
att	I	2	2.11
atc	I	4	1.58
ata	I	0	2.32
act	T	3624	12526.00
acc	T	35339	7131.00
aca	T	1370	17456.00
acg	T	1	3221.00
aat	N	0	1.68
aac	N	3	1.32
agt	S	0	0.17
agc	S	1	0.21
---	--	------	------
mPD		0.22	0.68
		nPD:	0.33
		N. weight:	1.3
		Sc. PD:	0.36
		Sc. rank:	40293.0

,

PA-X			
Pos.	33	obs:	exp:
tat	Y	0	0.53
tac	Y	1	0.47
aat	N	119	22571.00
aac	N	40224	17772.00
aaa	K	1	0.66
aag	K	0	0.34
---	--	------	------
mPD		0.0060	0.49
		nPD:	0.01
		N. weight:	0.87
		Sc. PD:	-0.027
		Sc. rank:	-245.1

,

PA-X			
Pos.	34	obs:	exp:
cgt	R	0	0.04
cgc	R	0	0.07
cga	R	0	0.18
cgg	R	0	0.17
aaa	K	40271	26523.00
aag	K	72	13820.00
aga	R	2	0.98
agg	R	0	0.55
---	--	------	------
mPD		0.0037	0.45
		nPD:	0.01
		N. weight:	0.45
		Sc. PD:	-0.015
		Sc. rank:	-2519.6

,

PA-X			
Pos.	35	obs:	exp:
ttt	F	40169	19622.00
ttc	F	171	20718.00
tta	L	0	0.58
ttg	L	0	1.03
ctt	L	4	1.05
ctc	L	1	0.73
cta	L	0	0.77
ctg	L	0	0.84
---	--	------	------
mPD		0.0087	0.50
		nPD:	0.02
		N. weight:	0.76
		Sc. PD:	-0.019
		Sc. rank:	5384.3

,

PA-X			
Pos.	36	obs:	exp:
act	T	0	0.31
acc	T	0	0.18
aca	T	1	0.43
acg	T	0	0.08
gtt	V	0	0.25
gtc	V	0	0.20
gta	V	1	0.22
gtg	V	0	0.33
gct	A	18	10995.00
gcc	A	1366	10079.00
gca	A	38664	16318.00
gcg	A	293	2949.00
gaa	E	2	1.18
gag	E	0	0.82
---	--	------	------
mPD		0.080	0.69
		nPD:	0.12
		N. weight:	0.81
		Sc. PD:	0.055
		Sc. rank:	21076.0

,

PA-X			
Pos.	37	obs:	exp:
tct	S	0	0.32
tcc	S	0	0.30
tca	S	2	0.54
tcg	S	0	0.08
att	I	1	0.35
atc	I	0	0.26
ata	I	0	0.39
act	T	0	1.55
acc	T	0	0.88
aca	T	5	2.16
acg	T	0	0.40
agt	S	0	0.35
agc	S	0	0.41
gct	A	5	10994.00
gcc	A	4	10078.00
gca	A	40265	16317.00
gcg	A	63	2949.00
---	--	------	------
mPD		0.0041	0.69
		nPD:	0.01
		N. weight:	0.98
		Sc. PD:	-0.035
		Sc. rank:	-14475.0

,

PA-X			
Pos.	38	obs:	exp:
tta	L	0	0.23
ttg	L	0	0.41
ctt	L	0	0.42
ctc	L	0	0.29
cta	L	2	0.31
ctg	L	0	0.34
att	I	2	14149.00
atc	I	2	10591.00
ata	I	40301	15566.00
atg	M	11	11.00
act	T	0	5.90
acc	T	0	3.36
aca	T	19	8.22
acg	T	0	1.52
aat	N	1	0.56
aac	N	0	0.44
gtt	V	0	1.25
gtc	V	0	1.02
gta	V	5	1.08
gtg	V	0	1.65
---	-	2	2.00
---	--	------	------
mPD		0.0021	0.66
		nPD:	0.
		N. weight:	1.
		Sc. PD:	-0.04
		Sc. rank:	-22541.0

,

PA-X			
Pos.	39	obs:	exp:
tta	L	0	0.12
ttg	L	1	0.21
tgt	C	118	15766.00
tgc	C	40225	24577.00
ctt	L	0	0.21
ctc	L	0	0.15
cta	L	0	0.15
ctg	L	0	0.17
cgt	R	0	0.02
cgc	R	0	0.04
cga	R	0	0.09
cgg	R	0	0.09
aga	R	0	0.49
agg	R	1	0.28
---	--	------	------
mPD		0.0060	0.48
		nPD:	0.01
		N. weight:	0.52
		Sc. PD:	-0.016
		Sc. rank:	149.4

,

PA-X			
Pos.	40	obs:	exp:
att	I	3	1.05
atc	I	0	0.79
ata	I	0	1.16
act	T	39620	12526.00
acc	T	685	7131.00
aca	T	27	17456.00
acg	T	4	3222.00
aat	N	1	0.56
aac	N	0	0.44
gct	A	5	1.36
gcc	A	0	1.25
gca	A	0	2.02
gcg	A	0	0.37
---	--	------	------
mPD		0.035	0.68
		nPD:	0.05
		N. weight:	1.2
		Sc. PD:	0.009
		Sc. rank:	22947.0

,

PA-X			
Pos.	41	obs:	exp:
cat	H	2630	22755.00
cac	H	37712	17587.00
caa	Q	2	1.22
cag	Q	0	0.78
aaa	K	0	0.66
aag	K	1	0.34
---	--	------	------
mPD		0.12	0.49
		nPD:	0.25
		N. weight:	0.63
		Sc. PD:	0.12
		Sc. rank:	17826.0

,

PA-X			
Pos.	42	obs:	exp:
tta	L	98	4682.00
ttg	L	40087	8341.00
tct	S	0	0.16
tcc	S	0	0.15
tca	S	1	0.27
tcg	S	0	0.04
ctt	L	0	8440.00
ctc	L	0	5916.00
cta	L	0	6195.00
ctg	L	159	6771.00
agt	S	0	0.17
agc	S	0	0.21
---	--	------	------
mPD		0.013	1.2
		nPD:	0.01
		N. weight:	1.7
		Sc. PD:	-0.053
		Sc. rank:	-3675.3

,

PA-X			
Pos.	43	obs:	exp:
gtt	V	0	0.25
gtc	V	0	0.20
gta	V	1	0.22
gtg	V	0	0.33
gaa	E	282	23785.00
gag	E	40062	16559.00
---	--	------	------
mPD		0.014	0.48
		nPD:	0.03
		N. weight:	0.93
		Sc. PD:	-0.013
		Sc. rank:	12423.0

,

PA-X			
Pos.	44	obs:	exp:
ttt	F	0	0.49
ttc	F	1	0.51
att	I	1	1.76
atc	I	0	1.31
ata	I	4	1.93
atg	M	1	1.00
gtt	V	21	10102.00
gtc	V	112	8218.00
gta	V	747	8741.00
gtg	V	39450	13269.00
gct	A	0	2.18
gcc	A	0	2.00
gca	A	1	3.24
gcg	A	7	0.58
---	--	------	------
mPD		0.044	0.74
		nPD:	0.06
		N. weight:	1.1
		Sc. PD:	0.016
		Sc. rank:	22781.0

,

PA-X			
Pos.	45	obs:	exp:
ttt	F	1	0.49
ttc	F	0	0.51
tat	Y	1	0.53
tac	Y	0	0.47
tgt	C	40155	15765.00
tgc	C	186	24576.00
aat	N	1	0.56
aac	N	0	0.44
---	-	1	1.00
---	--	------	------
mPD		0.0094	0.48
		nPD:	0.02
		N. weight:	0.99
		Sc. PD:	-0.023
		Sc. rank:	8533.2

,

PA-X

Pos. 46		obs:	exp:
ttt	F	28	19623.00
ttc	F	40313	20718.00
tta	L	2	0.23
ttg	L	0	0.41
tct	S	0	0.16
tcc	S	0	0.15
tca	S	0	0.27
tcg	S	0	0.04
ctt	L	0	0.42
ctc	L	0	0.29
cta	L	0	0.31
ctg	L	0	0.34
agt	S	0	0.17
agc	S	1	0.21
---	-	1	1.00
---	--	------	------
mPD		0.0016	0.50
		nPD:	0.
		N. weight:	0.72
		Sc. PD:	-0.028
		Sc. rank:	-15986.0

,

PA-X

Pos. 47		obs:	exp:
tta	L	0	0.12
ttg	L	0	0.21
ctt	L	0	0.21
ctc	L	0	0.15
cta	L	0	0.15
ctg	L	1	0.17
att	I	0	1.05
atc	I	0	0.79
ata	I	3	1.16
atg	M	40340	40340.00
---	-	1	1.00
---	--	------	------
mPD		0.00020	0.00023
		nPD:	0.86
		N. weight:	0.00013
		Sc. PD:	0.000098
		Sc. rank:	4.0

,

PA-X

Pos. 48		obs:	exp:
ttt	F	12	5.84
ttc	F	0	6.16
tat	Y	38777	21260.00
tac	Y	1556	19073.00
---	--	------	------
mPD		0.075	0.50
		nPD:	0.15
		N. weight:	0.53
		Sc. PD:	0.053
		Sc. rank:	14072.0

,

PA-X

Pos. 49		obs:	exp:
tta	L	1	0.12
ttg	L	0	0.21
tct	S	5	6509.00
tcc	S	4	6028.00
tca	S	40280	10842.00
tcg	S	50	1588.00
ctt	L	0	0.21
ctc	L	0	0.15
cta	L	0	0.15
ctg	L	0	0.17
cct	P	0	0.33
ccc	P	0	0.19
cca	P	1	0.34
ccg	P	0	0.14
agt	S	0	7049.00
agc	S	0	8322.00
gct	A	0	1.09
gcc	A	0	1.00
gca	A	4	1.62
gcg	A	0	0.29
---	--	------	------
mPD		0.0032	1.6
		nPD:	0.
		N. weight:	1.4
		Sc. PD:	-0.057
		Sc. rank:	-38059.0

,

PA-X

Pos. 50		obs:	exp:
gat	D	40285	22805.00
gac	D	60	17540.00
---	--	------	------
mPD		0.0030	0.49
		nPD:	0.01
		N. weight:	0.61
		Sc. PD:	-0.022
		Sc. rank:	-8247.0

,

PA-X

Pos. 51		obs:	exp:
ttt	F	1049	19622.00
ttc	F	39290	20717.00
tta	L	0	0.70
ttg	L	0	1.24
ctt	L	1	1.26
ctc	L	5	0.88
cta	L	0	0.92
ctg	L	0	1.01
---	--	------	------
mPD		0.051	0.50
		nPD:	0.1
		N. weight:	0.6
		Sc. PD:	0.033
		Sc. rank:	15414.0

,

PA-X

Pos. 52		obs:	exp:
cat	H	40131	22756.00
cac	H	214	17589.00
---	--	------	------
mPD		0.011	0.49
		nPD:	0.02
		N. weight:	0.59
		Sc. PD:	-0.013
		Sc. rank:	5572.5

,

PA-X

Pos. 53		obs:	exp:
ttt	F	86	19624.00
ttc	F	40258	20720.00
tta	L	0	0.12
ttg	L	0	0.21
ctt	L	0	0.21
ctc	L	1	0.15
cta	L	0	0.15
ctg	L	0	0.17
---	--	------	------
mPD		0.0043	0.50
		nPD:	0.01
		N. weight:	0.71
		Sc. PD:	-0.024
		Sc. rank:	-3747.8

,

PA-X

Pos. 54		obs:	exp:
tta	L	0	0.12
ttg	L	0	0.21
ctt	L	0	0.21
ctc	L	1	0.15
cta	L	0	0.15
ctg	L	0	0.17
att	I	62	14160.00
atc	I	40265	10599.00
ata	I	11	15578.00
gtt	V	0	1.25
gtc	V	5	1.02
gta	V	0	1.08
gtg	V	0	1.65
---	-	1	1.00
---	--	------	------
mPD		0.0039	0.66
		nPD:	0.01
		N. weight:	1.4
		Sc. PD:	-0.052
		Sc. rank:	-20895.0

,

PA-X

Pos. 55		obs:	exp:
tct	S	0	0.97
tcc	S	0	0.90
tca	S	0	1.61
tcg	S	0	0.24
att	I	1	0.35
atc	I	0	0.26
ata	I	0	0.39
act	T	1	0.31
acc	T	0	0.18
aca	T	0	0.43
acg	T	0	0.08
aat	N	40231	22565.00
aac	N	101	17767.00
agt	S	6	1.05
agc	S	0	1.24
gat	D	3	2.83
gac	D	2	2.17
---	--	------	------
mPD		0.0057	0.49
		nPD:	0.01
		N. weight:	0.62
		Sc. PD:	-0.019
		Sc. rank:	-716.7

,

PA-X

Pos. 56		obs:	exp:
aaa	K	1	0.66
aag	K	0	0.34
gtt	V	0	0.50
gtc	V	0	0.41
gta	V	1	0.43
gtg	V	1	0.66
gat	D	2	3.96
gac	D	5	3.04
gaa	E	39261	23778.00
gag	E	1071	16554.00
ggt	G	0	0.40
ggc	G	0	0.42
gga	G	3	1.38
ggg	G	0	0.79
---	--	------	------
mPD		0.052	0.48
		nPD:	0.11
		N. weight:	0.45
		Sc. PD:	0.028
		Sc. rank:	11751.0

,

PA-X

Pos. 57		obs:	exp:
tta	L	0	0.12
ttg	L	0	0.21
ctt	L	0	0.21
ctc	L	0	0.15
cta	L	1	0.15
ctg	L	0	0.17
cat	H	4	3.38
cac	H	2	2.62
caa	Q	39990	24582.00
cag	Q	224	15632.00
cgt	R	0	0.71
cgc	R	0	1.30
cga	R	33	3.28
cgg	R	3	3.09
aaa	K	88	57.85
aag	K	0	30.15
aga	R	0	17.66
agg	R	0	9.96
---	--	------	------
mPD		0.018	0.48
		nPD:	0.04
		N. weight:	0.51
		Sc. PD:	-0.0036
		Sc. rank:	8018.1

,

PA-X

Pos. 58		obs:	exp:
tct	S	0	1.29
tcc	S	0	1.20
tca	S	0	2.15
tcg	S	0	0.31
tgt	C	0	0.78
tgc	C	2	1.22
agt	S	0	1.40
agc	S	8	1.65
gtt	V	0	0.25
gtc	V	1	0.20
gta	V	0	0.22
gtg	V	0	0.33
gat	D	0	0.57
gac	D	1	0.43
ggt	G	179	5430.00
ggc	G	40079	5629.00
gga	G	73	18618.00
ggg	G	2	10656.00
---	--	------	------
mPD		0.013	0.68
		nPD:	0.02
		N. weight:	2.1
		Sc. PD:	-0.05
		Sc. rank:	17579.0

,

PA-X

Pos. 59		obs:	exp:
aaa	K	19	12.49
aag	K	0	6.51
gtt	V	0	0.25
gtc	V	0	0.20
gta	V	1	0.22
gtg	V	0	0.33
gat	D	1	2.26
gac	D	3	1.74
gaa	E	40173	23772.00
gag	E	148	16549.00
---	--	------	------
mPD		0.0085	0.49
		nPD:	0.02
		N. weight:	0.55
		Sc. PD:	-0.014
		Sc. rank:	4000.7

,

PA-X

Pos. 60		obs:	exp:
tct	S	1	6508.00
tcc	S	1	6027.00
tca	S	40295	10841.00
tcg	S	36	1588.00
cct	P	0	2.33
ccc	P	0	1.31
cca	P	7	2.36
ccg	P	0	0.99
act	T	0	0.93
acc	T	0	0.53
aca	T	3	1.30
acg	T	0	0.24
agt	S	0	7048.00
agc	S	0	8321.00
gct	A	0	0.55
gcc	A	0	0.50
gca	A	2	0.81
gcg	A	0	0.15
---	--	------	------
mPD		0.0025	1.6
		nPD:	0.
		N. weight:	1.4
		Sc. PD:	-0.058
		Sc. rank:	-39118.0

,

PA-X

Pos. 61		obs:	exp:
tta	L	0	1.16
ttg	L	0	2.07
ctt	L	0	2.09
ctc	L	0	1.47
cta	L	10	1.54
ctg	L	0	1.68
att	I	4	14136.00
atc	I	2	10581.00
ata	I	40264	15552.00
atg	M	44	44.00
act	T	0	2.48
acc	T	0	1.41
aca	T	8	3.46
acg	T	0	0.64
gtt	V	0	3.26
gtc	V	0	2.65
gta	V	13	2.82
gtg	V	0	4.28
---	--	------	------
mPD		0.0040	0.66
		nPD:	0.01
		N. weight:	1.
		Sc. PD:	-0.037
		Sc. rank:	-13543.0

,

PA-X

Pos. 62		obs:	exp:
tta	L	10	1.51
ttg	L	3	2.69
ctt	L	0	2.72
ctc	L	0	1.91
cta	L	0	2.00
ctg	L	0	2.18
att	I	29	186.40
atc	I	6	139.50
ata	I	496	205.10
atg	M	38	38.00
gtt	V	19	9959.00
gtc	V	2	8102.00
gta	V	35207	8618.00
gtg	V	4534	13083.00
---	-	1	1.00
---	--	------	------
mPD		0.23	0.77
		nPD:	0.3
		N. weight:	1.2
		Sc. PD:	0.3
		Sc. rank:	36648.0

,

PA-X

Pos. 63		obs:	exp:
tta	L	3	0.58
ttg	L	1	1.03
ctt	L	0	1.05
ctc	L	1	0.73
cta	L	0	0.77
ctg	L	0	0.84
att	I	0	36.51
atc	I	0	27.33
ata	I	104	40.16
atg	M	3	3.00
aaa	K	0	0.66
aag	K	1	0.34
gtt	V	10	10076.00
gtc	V	0	8196.00
gta	V	39285	8719.00
gtg	V	931	13235.00
gct	A	0	1.64
gcc	A	0	1.50
gca	A	5	2.43
gcg	A	1	0.44
---	--	------	------
mPD		0.052	0.75
		nPD:	0.07
		N. weight:	1.5
		Sc. PD:	0.037
		Sc. rank:	34200.0

,

PA-X

Pos. 64		obs:	exp:
aaa	K	1	0.66
aag	K	0	0.34
gtt	V	0	0.25
gtc	V	0	0.20
gta	V	1	0.22
gtg	V	0	0.33
gct	A	1	0.55
gcc	A	0	0.50
gca	A	0	0.81
gcg	A	1	0.15
gat	D	1	1.13
gac	D	1	0.87
gaa	E	39939	23782.00
gag	E	399	16556.00
ggt	G	0	0.13
ggc	G	0	0.14
gga	G	1	0.46
ggg	G	0	0.26
---	--	------	------
mPD		0.020	0.48
		nPD:	0.04
		N. weight:	0.52
		Sc. PD:	-0.0014
		Sc. rank:	9029.8

,

PA-X

Pos. 65		obs:	exp:
ttt	F	46	22.86
ttc	F	1	24.14
tta	L	0	4671.00
ttg	L	0	8322.00
tct	S	3	0.48
tcc	S	0	0.45
tca	S	0	0.81
tcg	S	0	0.12
ctt	L	40219	8420.00
ctc	L	28	5902.00
cta	L	3	6181.00
ctg	L	1	6755.00
cct	P	22	7.33
ccc	P	0	4.12
cca	P	0	7.43
ccg	P	0	3.11
att	I	21	7.37
atc	I	0	5.52
ata	I	0	8.11
aaa	K	0	0.66
aag	K	1	0.34
agt	S	0	0.52
agc	S	0	0.62
---	--	------	------
mPD		0.0065	1.2
		nPD:	0.01
		N. weight:	1.7
		Sc. PD:	-0.062
		Sc. rank:	-26307.0

,

PA-X

Pos. 66		obs:	exp:
tct	S	0	0.16
tcc	S	0	0.15
tca	S	1	0.27
tcg	S	0	0.04
aat	N	1	6.15
aac	N	10	4.85
agt	S	0	0.17
agc	S	0	0.21
gat	D	1573	22786.00
gac	D	38738	17525.00
gaa	E	0	1.18
gag	E	2	0.82
ggt	G	8	2.69
ggc	G	12	2.79
gga	G	0	9.23
ggg	G	0	5.28
---	--	------	------
mPD		0.077	0.49
		nPD:	0.16
		N. weight:	0.72
		Sc. PD:	0.077
		Sc. rank:	19376.0

,

PA-X

Pos. 67		obs:	exp:
tct	S	0	0.16
tcc	S	0	0.15
tca	S	0	0.27
tcg	S	0	0.04
aat	N	59	33.01
aac	N	0	25.99
agt	S	0	0.17
agc	S	1	0.21
gtt	V	0	0.25
gtc	V	0	0.20
gta	V	1	0.22
gtg	V	0	0.33
gat	D	40151	22770.00
gac	D	132	17513.00
gaa	E	0	0.59
gag	E	1	0.41
---	--	------	------
mPD		0.0097	0.49
		nPD:	0.02
		N. weight:	0.6
		Sc. PD:	-0.014
		Sc. rank:	5250.8

,

PA-X

Pos. 68		obs:	exp:
tta	L	0	0.12
ttg	L	0	0.21
tct	S	0	1.29
tcc	S	0	1.20
tca	S	8	2.15
tcg	S	0	0.31
ctt	L	0	0.21
ctc	L	0	0.15
cta	L	0	0.15
ctg	L	1	0.17
cct	P	10	13441.00
ccc	P	27	7562.00
cca	P	39772	13627.00
ccg	P	526	5705.00
caa	Q	1	0.61
cag	Q	0	0.39
agt	S	0	1.40
agc	S	0	1.65
---	--	------	------
mPD		0.028	0.72
		nPD:	0.04
		N. weight:	1.1
		Sc. PD:	-0.0054
		Sc. rank:	19178.0

,

PA-X

Pos. 69		obs:	exp:
tat	Y	1	0.53
tac	Y	0	0.47
aat	N	40306	22571.00
aac	N	38	17773.00
---	--	------	------
mPD		0.0019	0.49
		nPD:	0.
		N. weight:	0.63
		Sc. PD:	-0.024
		Sc. rank:	-11700.0

,

PA-X

Pos. 70		obs:	exp:
tct	S	0	0.16
tcc	S	0	0.15
tca	S	1	0.27
tcg	S	0	0.04
act	T	0	2.79
acc	T	0	1.59
aca	T	9	3.89
acg	T	0	0.72
agt	S	0	0.17
agc	S	0	0.21
gtt	V	0	9.77
gtc	V	0	7.95
gta	V	38	8.45
gtg	V	1	12.83
gct	A	3	10983.00
gcc	A	3	10068.00
gca	A	40140	16300.00
gcg	A	150	2946.00
---	--	------	------
mPD		0.010	0.70
		nPD:	0.01
		N. weight:	0.97
		Sc. PD:	-0.027
		Sc. rank:	2969.2

,

PA-X

Pos. 71		obs:	exp:
tta	L	0	4681.00
ttg	L	138	8340.00
tgt	C	1	0.39
tgc	C	0	0.61
ctt	L	53	8439.00
ctc	L	0	5916.00
cta	L	171	6195.00
ctg	L	39979	6770.00
atg	M	2	2.00
gtt	V	0	0.25
gtc	V	0	0.20
gta	V	1	0.22
gtg	V	0	0.33
---	--	------	------
mPD		0.018	1.2
		nPD:	0.02
		N. weight:	1.9
		Sc. PD:	-0.051
		Sc. rank:	8397.9

,

PA-X

Pos. 72		obs:	exp:
tta	L	38587	4682.00
ttg	L	1598	8341.00
ctt	L	0	8440.00
ctc	L	0	5916.00
cta	L	152	6195.00
ctg	L	7	6771.00
atg	M	1	1.00
---	--	------	------
mPD		0.084	1.2
		nPD:	0.07
		N. weight:	2.1
		Sc. PD:	0.058
		Sc. rank:	48918.0

,

PA-X

Pos. 73		obs:	exp:
cgt	R	0	0.14
cgc	R	0	0.25
cga	R	0	0.64
cgg	R	0	0.60
atg	M	1	1.00
aat	N	2	1.12
aac	N	0	0.88
aaa	K	16024	26517.00
aag	K	24310	13817.00
aga	R	3	3.43
agg	R	4	1.94
---	-	1	1.00
---	--	------	------
mPD		0.48	0.45
		nPD:	1.06
		N. weight:	0.15
		Sc. PD:	0.15
		Sc. rank:	4887.0

,

PA-X

Pos. 74		obs:	exp:
cat	H	40	22754.00
cac	H	40301	17587.00
caa	Q	0	0.61
cag	Q	1	0.39
aat	N	0	1.12
aac	N	2	0.88
gat	D	0	0.57
gac	D	1	0.43
---	--	------	------
mPD		0.0022	0.49
		nPD:	0.
		N. weight:	0.9
		Sc. PD:	-0.034
		Sc. rank:	-15992.0

,

PA-X

Pos. 75		obs:	exp:
cgt	R	0	799.90
cgc	R	0	1452.00
cga	R	10	3681.00
cgg	R	0	3464.00
act	T	0	0.31
acc	T	0	0.18
aca	T	1	0.43
acg	T	0	0.08
aga	R	40255	19788.00
agg	R	79	11159.00
---	--	------	------
mPD		0.0045	0.89
		nPD:	0.01
		N. weight:	0.76
		Sc. PD:	-0.028
		Sc. rank:	-12935.0

,

PA-X			
Pos.	76	obs:	exp:
ttt	F	39889	19624.00
ttc	F	455	20720.00
att	I	1	0.35
atc	I	0	0.26
ata	I	0	0.39
mPD		0.022	0.50
		nPD:	0.04
		N. weight:	0.72
		Sc. PD:	0.00043
		Sc. rank:	13116.0

PA-X			
Pos.	77	obs:	exp:
caa	Q	1	0.61
cag	Q	0	0.39
aaa	K	2	1.31
aag	K	0	0.69
gat	D	0	0.57
gac	D	1	0.43
gaa	E	40251	23782.00
gag	E	88	16557.00
ggt	G	0	0.27
ggc	G	0	0.28
gga	G	2	0.92
ggg	G	0	0.53
mPD		0.0047	0.48
		nPD:	0.01
		N. weight:	0.56
		Sc. PD:	-0.018
		Sc. rank:	-2263.4

PA-X			
Pos.	78	obs:	exp:
tta	L	2	1.04
ttg	L	0	1.86
ctt	L	0	1.88
ctc	L	0	1.32
cta	L	7	1.38
ctg	L	0	1.51
cgt	R	0	0.04
cgc	R	0	0.07
cga	R	0	0.18
cgg	R	0	0.17
att	I	20	14157.00
atc	I	2	10597.00
ata	I	40308	15575.00
act	T	0	0.93
acc	T	0	0.53
aca	T	3	1.30
acg	T	0	0.24
aga	R	1	0.98
agg	R	1	0.55
gtt	V	0	0.25
gtc	V	0	0.20
gta	V	1	0.22
gtg	V	0	0.33
mPD		0.0019	0.66
		nPD:	0.
		N. weight:	1.
		Sc. PD:	-0.041
		Sc. rank:	-23712.0

PA-X			
Pos.	79	obs:	exp:
tta	L	0	0.12
ttg	L	0	0.21
ctt	L	0	0.21
ctc	L	0	0.15
cta	L	0	0.15
ctg	L	1	0.17
cgt	R	0	0.02
cgc	R	0	0.04
cga	R	0	0.09
cgg	R	0	0.09
att	I	124	14158.00
atc	I	39829	10597.00
ata	I	378	15576.00
aga	R	0	0.49
agg	R	1	0.28
gtt	V	0	3.01
gtc	V	11	2.45
gta	V	1	2.60
gtg	V	0	3.95
mPD		0.025	0.66
		nPD:	0.04
		N. weight:	1.4
		Sc. PD:	-0.0071
		Sc. rank:	23291.0

PA-X			
Pos.	80	obs:	exp:
ttt	F	0	0.49
ttc	F	1	0.51
gat	D	1	0.57
gac	D	0	0.43
gaa	E	843	23785.00
gag	E	39500	16558.00
mPD		0.041	0.48
		nPD:	0.08
		N. weight:	0.86
		Sc. PD:	0.033
		Sc. rank:	21462.0

PA-X			
Pos.	81	obs:	exp:
tct	S	0	0.16
tcc	S	0	0.15
tca	S	0	0.27
tcg	S	0	0.04
agt	S	0	0.17
agc	S	1	0.21
ggt	G	3	5431.00
ggc	G	1	5631.00
gga	G	332	18623.00
ggg	G	40008	10658.00
mPD		0.017	0.68
		nPD:	0.02
		N. weight:	1.4
		Sc. PD:	-0.026
		Sc. rank:	14983.0

PA-X			
Pos.	82	obs:	exp:
cgt	R	0	799.90
cgc	R	0	1452.00
cga	R	0	3681.00
cgg	R	0	3464.00
att	I	0	0.35
atc	I	0	0.26
ata	I	1	0.39
aga	R	40068	19788.00
agg	R	276	11159.00
mPD		0.014	0.89
		nPD:	0.02
		N. weight:	0.74
		Sc. PD:	-0.02
		Sc. rank:	2951.5

PA-X			
Pos.	83	obs:	exp:
att	I	0	0.35
atc	I	1	0.26
ata	I	0	0.39
gtt	V	0	0.25
gtc	V	1	0.20
gta	V	0	0.22
gtg	V	0	0.33
gat	D	61	22801.00
gac	D	40276	17536.00
gaa	E	0	1.18
gag	E	2	0.82
ggt	G	0	0.54
ggc	G	4	0.56
gga	G	0	1.85
ggg	G	0	1.06
mPD		0.0035	0.49
		nPD:	0.01
		N. weight:	0.9
		Sc. PD:	-0.032
		Sc. rank:	-7741.1

PA-X			
Pos.	84	obs:	exp:
cgt	R	0	799.40
cgc	R	60	1451.00
cga	R	13	3679.00
cgg	R	0	3462.00
act	T	0	0.31
acc	T	0	0.18
aca	T	1	0.43
acg	T	0	0.08
aaa	K	24	16.44
aag	K	1	8.56
aga	R	33773	19775.00
agg	R	6472	11152.00
ggt	G	0	0.13
ggc	G	0	0.14
gga	G	1	0.46
ggg	G	0	0.26
mPD		0.28	0.89
		nPD:	0.31
		N. weight:	0.39
		Sc. PD:	0.099
		Sc. rank:	11742.0

PA-X			
Pos.	85	obs:	exp:
tct	S	0	0.16
tcc	S	0	0.15
tca	S	0	0.27
tcg	S	0	0.04
cct	P	0	2.33
ccc	P	0	1.31
cca	P	7	2.36
ccg	P	0	0.99
att	I	0	4.91
atc	I	2	3.68
ata	I	12	5.41
act	T	1	12521.00
acc	T	3	7128.00
aca	T	40286	17448.00
acg	T	27	3220.00
aaa	K	1	0.66
aag	K	0	0.34
agt	S	0	0.17
agc	S	1	0.21
gct	A	0	1.36
gcc	A	0	1.25
gca	A	5	2.02
gcg	A	0	0.37
mPD		0.0031	0.68
		nPD:	0.
		N. weight:	0.91
		Sc. PD:	-0.034
		Sc. rank:	-15986.0

PA-X			
Pos.	86	obs:	exp:
tta	L	0	0.35
ttg	L	1	0.62
ctt	L	0	0.63
ctc	L	0	0.44
cta	L	0	0.46
ctg	L	2	0.50
att	I	15	15.80
atc	I	0	11.82
ata	I	30	17.38
atg	M	40252	40252.00
act	T	1	0.31
acc	T	0	0.18
aca	T	0	0.43
acg	T	0	0.08
gtt	V	0	11.02
gtc	V	0	8.97
gta	V	0	9.54
gtg	V	44	14.48
mPD		0.0047	0.0062
		nPD:	0.75
		N. weight:	0.0019
		Sc. PD:	0.0013
		Sc. rank:	61.7

PA-X			
Pos.	87	obs:	exp:
gct	A	74	10996.00
gcc	A	40157	10080.00
gca	A	113	16319.00
gcg	A	0	2949.00
ggt	G	0	0.13
ggc	G	1	0.14
gga	G	0	0.46
ggg	G	0	0.26
mPD		0.0093	0.69
		nPD:	0.01
		N. weight:	1.5
		Sc. PD:	-0.043
		Sc. rank:	1640.0

PA-X			
Pos.	88	obs:	exp:
tgg	W	40345	40345.00
mPD		0	0
		nPD:	1.
		N. weight:	0.
		Sc. PD:	0
		Sc. rank:	0

PA-X			
Pos.	89	obs:	exp:
act	T	71	12529.00
acc	T	3	7133.00
aca	T	40193	17460.00
acg	T	76	3222.00
aaa	K	1	0.66
aag	K	0	0.34
gct	A	0	0.27
gcc	A	0	0.25
gca	A	1	0.40
gcg	A	0	0.07
mPD		0.0075	0.68
		nPD:	0.01
		N. weight:	0.89
		Sc. PD:	-0.028
		Sc. rank:	-1659.1

PA-X			
Pos.	90	obs:	exp:
tta	L	5	0.58
ttg	L	0	1.03
ctt	L	0	1.05
ctc	L	0	0.73
cta	L	0	0.77
ctg	L	0	0.84
att	I	0	17.55
atc	I	0	13.14
ata	I	50	19.31
gtt	V	8	10088.00
gtc	V	44	8207.00
gta	V	39952	8730.00
gtg	V	273	13252.00
gct	A	0	3.27
gcc	A	0	3.00
gca	A	12	4.85
gcg	A	0	0.88
---	-	1	1.00
mPD		0.019	0.74
		nPD:	0.03
		N. weight:	1.6
		Sc. PD:	-0.028
		Sc. rank:	17921.0

PA-X

Pos.	91	obs:	exp:
tta	L	2	0.23
ttg	L	0	0.41
ctt	L	0	0.42
ctc	L	0	0.29
cta	L	0	0.31
ctg	L	0	0.34
att	I	0	15.45
atc	I	0	11.56
ata	I	44	16.99
gtt	V	3	10094.00
gtc	V	1	8211.00
gta	V	39914	8734.00
gtg	V	381	13259.00
---	--	------	------
mPD		0.021	0.74
		nPD:	0.03
		N. weight:	1.6
		Sc. PD:	-0.024
		Sc. rank:	20770.0

,

PA-X

Pos.	92	obs:	exp:
cat	H	0	1.69
cac	H	3	1.31
act	T	0	0.62
acc	T	2	0.35
aca	T	0	0.87
acg	T	0	0.16
aat	N	20	22569.00
aac	N	40320	17771.00
---	--	------	------
mPD		0.0012	0.49
		nPD:	0.
		N. weight:	0.89
		Sc. PD:	-0.035
		Sc. rank:	-21643.0

,

PA-X

Pos.	93	obs:	exp:
tct	S	0	6510.00
tcc	S	0	6029.00
tca	S	0	10843.00
tcg	S	0	1588.00
tgt	C	1	0.39
tgc	C	0	0.61
agt	S	40235	7050.00
agc	S	107	8322.00
ggt	G	2	0.27
ggc	G	0	0.28
gga	G	0	0.92
ggg	G	0	0.53
---	--	------	------
mPD		0.0054	1.6
		nPD:	0.
		N. weight:	1.9
		Sc. PD:	-0.073
		Sc. rank:	-39713.0

,

PA-X

Pos.	94	obs:	exp:
tta	L	0	0.23
ttg	L	0	0.41
ctt	L	0	0.42
ctc	L	2	0.29
cta	L	0	0.31
ctg	L	0	0.34
att	I	293	14159.00
atc	I	39971	10598.00
ata	I	69	15576.00
gtt	V	0	2.25
gtc	V	9	1.83
gta	V	0	1.95
gtg	V	0	2.96
ggt	G	0	0.13
ggc	G	1	0.14
gga	G	0	0.46
ggg	G	0	0.26
---	--	------	------
mPD		0.018	0.66
		nPD:	0.03
		N. weight:	1.4
		Sc. PD:	-0.021
		Sc. rank:	16814.0

,

PA-X

Pos.	95	obs:	exp:
tct	S	0	0.16
tcc	S	0	0.15
tca	S	0	0.27
tcg	S	0	0.04
tgt	C	181	15766.00
tgc	C	40163	24578.00
agt	S	0	0.17
agc	S	1	0.21
---	--	------	------
mPD		0.0090	0.48
		nPD:	0.02
		N. weight:	0.51
		Sc. PD:	-0.012
		Sc. rank:	3843.8

,

PA-X

Pos.	96	obs:	exp:
tct	S	0	0.16
tcc	S	0	0.15
tca	S	0	0.27
tcg	S	0	0.04
att	I	0	0.35
atc	I	1	0.26
ata	I	0	0.39
act	T	0	0.31
acc	T	1	0.18
aca	T	0	0.43
acg	T	0	0.08
aat	N	13	22570.00
aac	N	40329	17772.00
agt	S	0	0.17
agc	S	1	0.21
---	--	------	------
mPD		0.00079	0.49
		nPD:	0.
		N. weight:	0.89
		Sc. PD:	-0.036
		Sc. rank:	-24239.0

,

PA-X

Pos.	97	obs:	exp:
tct	S	4	0.65
tcc	S	0	0.60
tca	S	0	1.08
tcg	S	0	0.16
att	I	3	2.11
atc	I	3	1.58
ata	I	0	2.32
act	T	40174	12519.00
acc	T	106	7127.00
aca	T	28	17446.00
acg	T	4	3220.00
aat	N	19	10.63
aac	N	0	8.37
agt	S	0	0.70
agc	S	0	0.83
gct	A	3	0.82
gcc	A	0	0.75
gca	A	0	1.21
gcg	A	0	0.22
---	-	1	1.00
---	--	------	------
mPD		0.0086	0.68
		nPD:	0.01
		N. weight:	1.3
		Sc. PD:	-0.037
		Sc. rank:	199.9

,

PA-X

Pos.	98	obs:	exp:
att	I	2	0.70
atc	I	0	0.53
ata	I	0	0.77
act	T	40220	12526.00
acc	T	53	7131.00
aca	T	49	17456.00
acg	T	12	3221.00
aat	N	5	2.80
aac	N	0	2.20
gct	A	3	1.09
gcc	A	0	1.00
gca	A	1	1.62
gcg	A	0	0.29
---	--	------	------
mPD		0.0062	0.68
		nPD:	0.01
		N. weight:	1.3
		Sc. PD:	-0.042
		Sc. rank:	-5438.5

,

PA-X

Pos.	99	obs:	exp:
cgt	R	0	1.11
cgc	R	0	2.01
cga	R	0	5.11
cgg	R	0	4.81
aga	R	56	27.47
agg	R	0	15.49
gaa	E	14	8.25
gag	E	0	5.75
ggt	G	13	5422.00
ggc	G	1	5621.00
gga	G	40104	18592.00
ggg	G	157	10640.00
---	--	------	------
mPD		0.012	0.68
		nPD:	0.02
		N. weight:	0.82
		Sc. PD:	-0.021
		Sc. rank:	5651.1

,

PA-X

Pos.	100	obs:	exp:
tta	L	0	1.28
ttg	L	0	2.27
tct	S	3	0.48
tcc	S	0	0.45
tca	S	0	0.81
tcg	S	0	0.12
ctt	L	0	2.30
ctc	L	0	1.61
cta	L	0	1.69
ctg	L	11	1.85
att	I	0	0.70
atc	I	0	0.53
ata	I	2	0.77
act	T	91	28.26
acc	T	0	16.09
aca	T	0	39.38
acg	T	0	7.27
agt	S	0	0.52
agc	S	0	0.62
gtt	V	28	7.51
gtc	V	0	6.11
gta	V	2	6.50
gtg	V	0	9.87
gct	A	40053	10958.00
gcc	A	109	10045.00
gca	A	35	16264.00
gcg	A	9	2939.00
gat	D	2	1.13
gac	D	0	0.87
---	--	------	------
mPD		0.016	0.70
		nPD:	0.02
		N. weight:	1.4
		Sc. PD:	-0.028
		Sc. rank:	13966.0

,

PA-X

Pos.	101	obs:	exp:
cgt	R	0	1.72
cgc	R	0	3.13
cga	R	0	7.94
cgg	R	0	7.47
aaa	K	0	1.97
aag	K	3	1.03
aga	R	87	42.67
agg	R	0	24.06
gtt	V	0	1.75
gtc	V	0	1.43
gta	V	7	1.52
gtg	V	0	2.30
gaa	E	1089	902.60
gag	E	442	628.40
ggt	G	10	5212.00
ggc	G	1	5404.00
gga	G	38189	17872.00
ggg	G	517	10229.00
---	--	------	------
mPD		0.13	0.75
		nPD:	0.17
		N. weight:	0.75
		Sc. PD:	0.086
		Sc. rank:	20206.0

,

PA-X

Pos.	102	obs:	exp:
cgt	R	0	0.08
cgc	R	0	0.14
cga	R	0	0.36
cgg	R	0	0.34
act	T	0	0.31
acc	T	0	0.18
aca	T	0	0.43
acg	T	1	0.08
aaa	K	40097	26521.00
aag	K	243	13819.00
aga	R	4	1.96
agg	R	0	1.11
---	--	------	------
mPD		0.012	0.45
		nPD:	0.03
		N. weight:	0.42
		Sc. PD:	-0.0068
		Sc. rank:	4866.1

,

PA-X

Pos.	103	obs:	exp:
tta	L	0	0.12
ttg	L	1	0.21
ctt	L	0	0.21
ctc	L	0	0.15
cta	L	0	0.15
ctg	L	0	0.17
---	-	40344	40344.00
---	--	------	------
mPD		0	7.1×10^{-10}
		nPD:	0.
		N. weight:	0.
		Sc. PD:	0
		Sc. rank:	0

,

PA-X

Pos.	104	obs:	exp:
cct	P	1	0.33
ccc	P	0	0.19
cca	P	0	0.34
ccg	P	0	0.14
---	-	40344	40344.00
---	--	------	------
mPD		0	4.4×10^{-10}
		nPD:	0.
		N. weight:	0.
		Sc. PD:	0
		Sc. rank:	0

,

PA-X

Pos.	105	obs:	exp:
cct	P	42	13444.00
ccc	P	4	7564.00
cca	P	698	13631.00
ccg	P	39601	5706.00
---	--	------	------
mPD		0.036	0.72
		nPD:	0.05
		N. weight:	2.
		Sc. PD:	0.012
		Sc. rank:	38356.0

,

PA-X

Pos.	106	obs:	exp:
tct	S	0	0.32
tcc	S	0	0.30
tca	S	1	0.54
tcg	S	0	0.08
cgt	R	0	0.77
cgc	R	0	1.40
cga	R	0	3.56
cgg	R	0	3.35
act	T	0	1.86
acc	T	0	1.06
aca	T	0	2.60
acg	T	6	0.48
aat	N	13	7.27
aac	N	0	5.73
aaa	K	441	26483.00
aag	K	39841	13799.00
agt	S	1	0.35
agc	S	0	0.41
aga	R	0	19.13
agg	R	39	10.79
gaa	E	0	1.77
gag	E	3	1.23
---	--	------	------
mPD		0.025	0.45
		nPD:	0.05
		N. weight:	1.1
		Sc. PD:	0.011
		Sc. rank:	22326.0

,

PA-X

Pos.	107	obs:	exp:
ttt	F	39955	19574.00
ttc	F	285	20666.00
tta	L	2	5.45
ttg	L	2	9.72
tct	S	24	3.87
tcc	S	0	3.59
tca	S	0	6.45
tcg	S	0	0.94
tat	Y	27	14.23
tac	Y	0	12.77
tgt	C	3	1.17
tgc	C	0	1.83
ctt	L	43	9.83
ctc	L	0	6.89
cta	L	0	7.22
ctg	L	0	7.89
agt	S	0	4.19
agc	S	0	4.95
gtt	V	3	1.00
gtc	V	1	0.82
gta	V	0	0.87
gtg	V	0	1.32
---	--	------	------
mPD		0.019	0.51
		nPD:	0.04
		N. weight:	0.74
		Sc. PD:	-0.0042
		Sc. rank:	12333.0

,

PA-X

Pos.	108	obs:	exp:
tta	L	13341	4681.00
ttg	L	14	8341.00
ctt	L	5	8439.00
ctc	L	1	5916.00
cta	L	26675	6195.00
ctg	L	307	6771.00
att	I	0	0.35
atc	I	0	0.26
ata	I	1	0.39
act	T	1	0.31
acc	T	0	0.18
aca	T	0	0.43
acg	T	0	0.08
---	--	------	------
mPD		0.46	1.2
		nPD:	0.4
		N. weight:	1.4
		Sc. PD:	0.47
		Sc. rank:	43473.0

,

PA-X

Pos.	109	obs:	exp:
cct	P	75	13444.00
ccc	P	3	7564.00
cca	P	3014	13631.00
ccg	P	37253	5706.00
---	--	------	------
mPD		0.14	0.72
		nPD:	0.2
		N. weight:	1.8
		Sc. PD:	0.26
		Sc. rank:	49727.0

,

PA-X

Pos.	110	obs:	exp:
aat	N	1	0.56
aac	N	0	0.44
gct	A	0	0.27
gcc	A	0	0.25
gca	A	1	0.40
gcg	A	0	0.07
gat	D	40173	22804.00
gac	D	170	17539.00
---	--	------	------
mPD		0.0085	0.49
		nPD:	0.02
		N. weight:	0.6
		Sc. PD:	-0.015
		Sc. rank:	4000.6

,

PA-X

Pos.	111	obs:	exp:
tta	L	185	4682.00
ttg	L	32119	8341.00
ctt	L	10	8440.00
ctc	L	1	5916.00
cta	L	7	6195.00
ctg	L	8022	6771.00
---	-	1	1.00
---	--	------	------
mPD		0.33	1.2
		nPD:	0.28
		N. weight:	1.2
		Sc. PD:	0.27
		Sc. rank:	35043.0

,

PA-X

Pos.	112	obs:	exp:
tat	Y	40250	21266.00
tac	Y	94	19078.00
---	-	1	1.00
---	--	------	------
mPD		0.0046	0.50
		nPD:	0.01
		N. weight:	0.68
		Sc. PD:	-0.023
		Sc. rank:	-2843.5

,

PA-X

Pos.	113	obs:	exp:
act	T	1	0.31
acc	T	0	0.18
aca	T	0	0.43
acg	T	0	0.08
gat	D	40273	22804.00
gac	D	70	17539.00
ggt	G	1	0.13
ggc	G	0	0.14
gga	G	0	0.46
ggg	G	0	0.26
---	--	------	------
mPD		0.0036	0.49
		nPD:	0.01
		N. weight:	0.61
		Sc. PD:	-0.021
		Sc. rank:	-5005.8

,

PA-X

Pos.	114	obs:	exp:
tat	Y	39	21264.00
tac	Y	40302	19077.00
tgt	C	0	0.39
tgc	C	1	0.61
cat	H	0	1.13
cac	H	2	0.87
gat	D	0	0.57
gac	D	1	0.43
---	--	------	------
mPD		0.0021	0.50
		nPD:	0.
		N. weight:	0.81
		Sc. PD:	-0.031
		Sc. rank:	-14959.0

,

PA-X

Pos.	115	obs:	exp:
cgt	R	0	0.12
cgc	R	0	0.22
cga	R	0	0.55
cgg	R	0	0.52
aat	N	1	0.56
aac	N	0	0.44
aaa	K	33296	26520.00
aag	K	7042	13818.00
aga	R	4	2.94
agg	R	2	1.66
---	--	------	------
mPD		0.29	0.45
		nPD:	0.64
		N. weight:	0.077
		Sc. PD:	0.044
		Sc. rank:	2431.1

,

PA-X

Pos.	116	obs:	exp:
aaa	K	3	5.92
aag	K	6	3.08
gat	D	0	0.57
gac	D	1	0.43
gaa	E	16433	23778.00
gag	E	23899	16554.00
ggt	G	0	0.40
ggc	G	0	0.42
gga	G	1	1.38
ggg	G	2	0.79
---	--	------	------
mPD		0.48	0.48
		nPD:	1.
		N. weight:	0.074
		Sc. PD:	0.067
		Sc. rank:	2337.0

,

PA-X

Pos.	117	obs:	exp:
tct	S	0	1.13
tcc	S	0	1.05
tca	S	0	1.88
tcg	S	0	0.28
act	T	0	0.31
acc	T	1	0.18
aca	T	0	0.43
acg	T	0	0.08
aat	N	174	22556.00
aac	N	40142	17760.00
aaa	K	4	3.94
aag	K	2	2.06
agt	S	0	1.22
agc	S	7	1.44
gat	D	0	8.48
gac	D	15	6.52
---	--	------	------
mPD		0.010	0.49
		nPD:	0.02
		N. weight:	0.87
		Sc. PD:	-0.02
		Sc. rank:	8024.3

,

PA-X

Pos.	118	obs:	exp:
cgt	R	0	799.70
cgc	R	0	1451.00
cga	R	4	3680.00
cgg	R	2	3463.00
aaa	K	12	7.89
aag	K	0	4.11
aga	R	39760	19783.00
agg	R	567	11156.00
---	--	------	------
mPD		0.029	0.89
		nPD:	0.03
		N. weight:	0.71
		Sc. PD:	-0.0078
		Sc. rank:	9768.9

,

PA-X

Pos.	119	obs:	exp:
ttt	F	31	19624.00
ttc	F	40313	20720.00
tgt	C	0	0.39
tgc	C	1	0.61
---	--	------	------
mPD		0.0016	0.50
		nPD:	0.
		N. weight:	0.72
		Sc. PD:	-0.028
		Sc. rank:	-15844.0

,

PA-X			
Pos.	120	obs:	exp:
tta	L	0	0.81
ttg	L	0	1.45
ctt	L	0	1.46
ctc	L	7	1.03
cta	L	0	1.07
ctg	L	0	1.17
att	I	217	14147.00
atc	I	40025	10590.00
ata	I	59	15564.00
gtt	V	2	9.27
gtc	V	35	7.54
gta	V	0	8.02
gtg	V	0	12.17
---	--	------	------
mPD		0.016	0.66
		nPD:	0.02
		N. weight:	1.4
		Sc. PD:	-0.027
		Sc. rank:	14647.0

,

PA-X			
Pos.	121	obs:	exp:
aaa	K	1	0.66
aag	K	0	0.34
gct	A	0	0.27
gcc	A	0	0.25
gca	A	1	0.40
gcg	A	0	0.07
gat	D	1	0.57
gac	D	0	0.43
gaa	E	40018	23784.00
gag	E	324	16558.00
---	--	------	------
mPD		0.016	0.48
		nPD:	0.03
		N. weight:	0.53
		Sc. PD:	-0.0055
		Sc. rank:	7642.2

,

PA-X			
Pos.	122	obs:	exp:
tta	L	0	0.23
ttg	L	0	0.41
ctt	L	2	0.42
ctc	L	0	0.29
cta	L	0	0.31
ctg	L	0	0.34
att	I	40132	14152.00
atc	I	158	10593.00
ata	I	25	15570.00
gtt	V	28	7.01
gtc	V	0	5.71
gta	V	0	6.07
gtg	V	0	9.21
---	--	------	------
mPD		0.011	0.66
		nPD:	0.02
		N. weight:	1.1
		Sc. PD:	-0.03
		Sc. rank:	5257.3

,

PA-X			
Pos.	123	obs:	exp:
ggt	G	7	5431.00
ggc	G	1	5631.00
gga	G	39513	18624.00
ggg	G	824	10659.00
---	--	------	------
mPD		0.040	0.68
		nPD:	0.06
		N. weight:	0.75
		Sc. PD:	0.011
		Sc. rank:	15615.0

,

PA-X			
Pos.	124	obs:	exp:
att	I	0	0.70
atc	I	0	0.53
ata	I	2	0.77
gtt	V	15	10105.00
gtc	V	0	8220.00
gta	V	1289	8744.00
gtg	V	39038	13273.00
gct	A	0	0.27
gcc	A	0	0.25
gca	A	0	0.40
gcg	A	1	0.07
---	--	------	------
mPD		0.063	0.74
		nPD:	0.08
		N. weight:	1.1
		Sc. PD:	0.041
		Sc. rank:	26440.0

,

PA-X			
Pos.	125	obs:	exp:
tct	S	0	0.16
tcc	S	0	0.15
tca	S	1	0.27
tcg	S	0	0.04
act	T	5	12529.00
acc	T	4	7133.00
aca	T	39819	17460.00
acg	T	516	3222.00
agt	S	0	0.17
agc	S	0	0.21
---	--	------	------
mPD		0.026	0.68
		nPD:	0.04
		N. weight:	0.86
		Sc. PD:	-0.005
		Sc. rank:	14053.0

,

PA-X			
Pos.	126	obs:	exp:
cgt	R	0	799.90
cgc	R	0	1452.00
cga	R	1	3681.00
cgg	R	4	3464.00
aaa	K	0	1.31
aag	K	2	0.69
aga	R	945	19788.00
agg	R	39393	11159.00
---	--	------	------
mPD		0.046	0.89
		nPD:	0.05
		N. weight:	1.3
		Sc. PD:	0.0096
		Sc. rank:	24885.0

,

PA-X			
Pos.	127	obs:	exp:
cgt	R	0	799.90
cgc	R	0	1452.00
cga	R	2	3681.00
cgg	R	0	3464.00
aaa	K	2	1.31
aag	K	0	0.69
aga	R	40229	19787.00
agg	R	110	11158.00
ggt	G	0	0.27
ggc	G	0	0.28
gga	G	2	0.92
ggg	G	0	0.53
---	--	------	------
mPD		0.0057	0.89
		nPD:	0.01
		N. weight:	0.76
		Sc. PD:	-0.027
		Sc. rank:	-9421.7

,

PA-X			
Pos.	128	obs:	exp:
gat	D	607	397.40
gac	D	96	305.60
gaa	E	39569	23370.00
gag	E	70	16269.00
ggt	G	0	0.40
ggc	G	0	0.42
gga	G	3	1.38
ggg	G	0	0.79
---	--	------	------
mPD		0.038	0.50
		nPD:	0.08
		N. weight:	0.56
		Sc. PD:	0.017
		Sc. rank:	13087.0

,

PA-X			
Pos.	129	obs:	exp:
tta	L	0	0.35
ttg	L	0	0.62
ctt	L	0	0.63
ctc	L	3	0.44
cta	L	0	0.46
ctg	L	0	0.50
att	I	0	8.78
atc	I	25	6.57
ata	I	0	9.65
act	T	0	0.31
acc	T	1	0.18
aca	T	0	0.43
acg	T	0	0.08
gtt	V	141	10093.00
gtc	V	40131	8210.00
gta	V	22	8733.00
gtg	V	0	13258.00
gct	A	0	6.00
gcc	A	22	5.50
gca	A	0	8.90
gcg	A	0	1.61
---	--	------	------
mPD		0.011	0.74
		nPD:	0.01
		N. weight:	1.7
		Sc. PD:	-0.048
		Sc. rank:	4831.9

,

PA-X			
Pos.	130	obs:	exp:
tat	Y	0	2.11
tac	Y	4	1.89
cct	P	0	0.33
ccc	P	1	0.19
cca	P	0	0.34
ccg	P	0	0.14
cat	H	61	22734.00
cac	H	40245	17572.00
caa	Q	27	17.12
cag	Q	1	10.88
aat	N	0	3.36
aac	N	6	2.64
---	--	------	------
mPD		0.0049	0.49
		nPD:	0.01
		N. weight:	0.9
		Sc. PD:	-0.029
		Sc. rank:	-3410.7

,

PA-X			
Pos.	131	obs:	exp:
tta	L	57	6.96
ttg	L	0	12.40
ctt	L	0	12.55
ctc	L	0	8.80
cta	L	3	9.21
ctg	L	0	10.07
cgt	R	0	0.02
cgc	R	0	0.04
cga	R	0	0.09
cgg	R	0	0.09
att	I	8	14026.00
atc	I	1	10499.00
ata	I	39946	15431.00
atg	M	292	292.00
act	T	0	10.56
acc	T	0	6.01
aca	T	34	14.71
acg	T	0	2.72
aga	R	1	0.49
agg	R	0	0.28
gtt	V	0	0.75
gtc	V	0	0.61
gta	V	3	0.65
gtg	V	0	0.99
---	--	------	------
mPD		0.020	0.67
		nPD:	0.03
		N. weight:	1.
		Sc. PD:	-0.014
		Sc. rank:	14112.0

,

PA-X

Pos.	132	obs:	exp:
tat	Y	40078	21265.00
tac	Y	265	19078.00
cat	H	2	1.13
cac	H	0	0.87
---	--	------	------
mPD		0.013	0.50
		nPD:	0.03
		N. weight:	0.66
		Sc. PD:	-0.011
		Sc. rank:	7502.3

,

PA-X

Pos.	133	obs:	exp:
tat	Y	59	21266.00
tac	Y	40285	19078.00
tgt	C	0	0.39
tgc	C	1	0.61
---	--	------	------
mPD		0.0030	0.50
		nPD:	0.01
		N. weight:	0.81
		Sc. PD:	-0.029
		Sc. rank:	-11502.0

,

PA-X

Pos.	134	obs:	exp:
ttt	F	12	5.84
ttc	F	0	6.16
tta	L	0	4678.00
ttg	L	0	8335.00
ctt	L	40167	8433.00
ctc	L	81	5912.00
cta	L	15	6190.00
ctg	L	51	6766.00
cgt	R	1	0.02
cgc	R	0	0.04
cga	R	0	0.09
cgg	R	0	0.09
att	I	17	5.97
atc	I	0	4.47
ata	I	0	6.57
aga	R	0	0.49
agg	R	0	0.28
gtt	V	0	0.25
gtc	V	0	0.20
gta	V	1	0.22
gtg	V	0	0.33
---	--	------	------
mPD		0.0089	1.2
		nPD:	0.01
		N. weight:	1.7
		Sc. PD:	-0.058
		Sc. rank:	-11847.0

,

PA-X

Pos.	135	obs:	exp:
caa	Q	1	0.61
cag	Q	0	0.39
aaa	K	4	2.63
aag	K	0	1.37
gat	D	1	0.57
gac	D	0	0.43
gaa	E	40088	23782.00
gag	E	251	16557.00
---	--	------	------
mPD		0.013	0.48
		nPD:	0.03
		N. weight:	0.54
		Sc. PD:	-0.0092
		Sc. rank:	6060.7

,

PA-X

Pos.	136	obs:	exp:
act	T	0	0.31
acc	T	0	0.18
aca	T	0	0.43
acg	T	1	0.08
aaa	K	104	26524.00
aag	K	40240	13820.00
---	--	------	------
mPD		0.0052	0.45
		nPD:	0.01
		N. weight:	1.2
		Sc. PD:	-0.036
		Sc. rank:	-1508.9

,

PA-X

Pos.	137	obs:	exp:
tct	S	0	0.16
tcc	S	1	0.15
tca	S	0	0.27
tcg	S	0	0.04
act	T	0	1.24
acc	T	4	0.71
aca	T	0	1.73
acg	T	0	0.32
agt	S	0	0.17
agc	S	0	0.21
gtt	V	0	2.50
gtc	V	10	2.04
gta	V	0	2.17
gtg	V	0	3.29
gct	A	49	10992.00
gcc	A	40254	10076.00
gca	A	26	16313.00
gcg	A	0	2948.00
gat	D	0	0.57
gac	D	1	0.43
---	--	------	------
mPD		0.0045	0.70
		nPD:	0.01
		N. weight:	1.5
		Sc. PD:	-0.054
		Sc. rank:	-18059.0

,

PA-X

Pos.	138	obs:	exp:
tct	S	0	1.61
tcc	S	0	1.49
tca	S	0	2.69
tcg	S	0	0.39
cat	H	1	0.56
cac	H	0	0.44
aat	N	39658	22562.00
aac	N	670	17766.00
aaa	K	0	0.66
aag	K	1	0.34
agt	S	10	1.75
agc	S	0	2.06
gat	D	5	2.83
gac	D	0	2.17
---	--	------	------
mPD		0.034	0.49
		nPD:	0.07
		N. weight:	0.55
		Sc. PD:	0.012
		Sc. rank:	11974.0

,

PA-X

Pos.	139	obs:	exp:
cgt	R	0	0.16
cgc	R	0	0.29
cga	R	0	0.73
cgg	R	0	0.69
aat	N	2	1.12
aac	N	0	0.88
aaa	K	8189	26518.00
aag	K	32146	13817.00
aga	R	1	3.92
agg	R	7	2.21
---	--	------	------
mPD		0.32	0.45
		nPD:	0.72
		N. weight:	0.48
		Sc. PD:	0.3
		Sc. rank:	15075.0

,

PA-X

Pos.	140	obs:	exp:
ttt	F	1	0.49
ttc	F	0	0.51
att	I	39186	14161.00
atc	I	1139	10600.00
ata	I	15	15579.00
gtt	V	3	1.00
gtc	V	1	0.82
gta	V	0	0.87
gtg	V	0	1.32
---	--	------	------
mPD		0.056	0.66
		nPD:	0.08
		N. weight:	1.
		Sc. PD:	0.039
		Sc. rank:	25145.0

,

PA-X

Pos.	141	obs:	exp:
cgt	R	0	0.46
cgc	R	0	0.83
cga	R	0	2.10
cgg	R	0	1.97
aat	N	2	2.80
aac	N	3	2.20
aaa	K	40244	26506.00
aag	K	73	13811.00
aga	R	23	11.28
agg	R	0	6.36
---	--	------	------
mPD		0.0050	0.45
		nPD:	0.01
		N. weight:	0.45
		Sc. PD:	-0.014
		Sc. rank:	-927.0

,

PA-X

Pos.	142	obs:	exp:
ttt	F	15	7.30
ttc	F	0	7.70
tta	L	1	0.12
ttg	L	0	0.21
tct	S	32447	6505.00
tcc	S	36	6024.00
tca	S	7827	10835.00
tcg	S	2	1587.00
ctt	L	0	0.21
ctc	L	0	0.15
cta	L	0	0.15
ctg	L	0	0.17
cct	P	5	1.67
ccc	P	0	0.94
cca	P	0	1.69
ccg	P	0	0.71
act	T	10	3.11
acc	T	0	1.77
aca	T	0	4.33
acg	T	0	0.80
aat	N	0	0.56
aac	N	1	0.44
agt	S	0	7045.00
agc	S	0	8316.00
gct	A	1	0.27
gcc	A	0	0.25
gca	A	0	0.40
gcg	A	0	0.07
---	--	------	------
mPD		0.32	1.6
		nPD:	0.19
		N. weight:	1.3
		Sc. PD:	0.19
		Sc. rank:	37285.0

,

PA-X

Pos.	143	obs:	exp:
aat	N	0	1.12
aac	N	2	0.88
aaa	K	0	2.63
aag	K	4	1.37
gat	D	6	6.78
gac	D	6	5.22
gaa	E	334	23774.00
gag	E	39990	16550.00
ggt	G	0	0.40
ggc	G	0	0.42
gga	G	0	1.38
ggg	G	3	0.79
---	--	------	------
mPD		0.018	0.48
		nPD:	0.04
		N. weight:	0.92
		Sc. PD:	-0.0069
		Sc. rank:	14465.0

,

PA-X

Pos.	144	obs:	exp:
tct	S	0	3.39
tcc	S	0	3.14
tca	S	0	5.64
tcg	S	0	0.83
cat	H	0	0.56
cac	H	1	0.44
act	T	3	4.35
acc	T	11	2.48
aca	T	0	6.06
acg	T	0	1.12
aat	N	675	22513.00
aac	N	39564	17726.00
aaa	K	15	14.46
aag	K	7	7.54
agt	S	0	3.67
agc	S	21	4.33
gat	D	2	23.74
gac	D	40	18.26
ggt	G	0	0.81
ggc	G	6	0.84
gga	G	0	2.77
ggg	G	0	1.59
---	--	------	------
mPD		0.039	0.50
		nPD:	0.08
		N. weight:	0.8
		Sc. PD:	0.025
		Sc. rank:	19004.0

PA-X

Pos.	145	obs:	exp:
tct	S	0	0.16
tcc	S	0	0.15
tca	S	1	0.27
tcg	S	0	0.04
cct	P	0	3.00
ccc	P	0	1.69
cca	P	9	3.04
ccg	P	0	1.27
att	I	0	1.76
atc	I	0	1.31
ata	I	5	1.93
act	T	6	12522.00
acc	T	9	7129.00
aca	T	40293	17450.00
acg	T	13	3220.00
aaa	K	2	1.31
aag	K	0	0.69
agt	S	0	0.17
agc	S	0	0.21
gct	A	0	1.91
gcc	A	0	1.75
gca	A	7	2.83
gcg	A	0	0.51
---	--	------	------
mPD		0.0026	0.68
		nPD:	0.
		N. weight:	0.91
		Sc. PD:	-0.035
		Sc. rank:	-17962.0

PA-X

Pos.	146	obs:	exp:
tat	Y	0	2.11
tac	Y	4	1.89
cat	H	89	22746.00
cac	H	40238	17581.00
caa	Q	3	1.83
cag	Q	0	1.17
gtt	V	0	2.50
gtc	V	10	2.04
gta	V	0	2.17
gtg	V	0	3.29
gat	D	0	0.57
gac	D	1	0.43
---	--	------	------
mPD		0.0058	0.49
		nPD:	0.01
		N. weight:	0.89
		Sc. PD:	-0.027
		Sc. rank:	-841.8

PA-X

Pos.	147	obs:	exp:
tta	L	0	0.23
ttg	L	0	0.41
ctt	L	2	0.42
ctc	L	0	0.29
cta	L	0	0.31
ctg	L	0	0.34
att	I	39870	14159.00
atc	I	453	10598.00
ata	I	10	15576.00
act	T	2	0.62
acc	T	0	0.35
aca	T	0	0.87
acg	T	0	0.16
gtt	V	8	2.00
gtc	V	0	1.63
gta	V	0	1.73
gtg	V	0	2.63
---	--	------	------
mPD		0.023	0.66
		nPD:	0.04
		N. weight:	1.1
		Sc. PD:	-0.009
		Sc. rank:	16379.0

PA-X

Pos.	148	obs:	exp:
cat	H	159	22756.00
cac	H	40186	17589.00
---	--	------	------
mPD		0.0079	0.49
		nPD:	0.02
		N. weight:	0.88
		Sc. PD:	-0.023
		Sc. rank:	4339.3

PA-X

Pos.	149	obs:	exp:
att	I	40192	14162.00
atc	I	147	10600.00
ata	I	3	15580.00
gtt	V	3	0.75
gtc	V	0	0.61
gta	V	0	0.65
gtg	V	0	0.99
---	--	------	------
mPD		0.0076	0.66
		nPD:	0.01
		N. weight:	1.1
		Sc. PD:	-0.035
		Sc. rank:	-1780.7

PA-X

Pos.	150	obs:	exp:
ttt	F	37471	19624.00
ttc	F	2873	20720.00
tta	L	0	0.12
ttg	L	0	0.21
ctt	L	1	0.21
ctc	L	0	0.15
cta	L	0	0.15
ctg	L	0	0.17
---	--	------	------
mPD		0.13	0.50
		nPD:	0.26
		N. weight:	0.5
		Sc. PD:	0.11
		Sc. rank:	14390.0

PA-X

Pos.	151	obs:	exp:
tct	S	5	6510.00
tcc	S	1	6029.00
tca	S	40297	10844.00
tcg	S	42	1588.00
agt	S	0	7050.00
agc	S	0	8323.00
---	--	------	------
mPD		0.0024	1.6
		nPD:	0.
		N. weight:	1.4
		Sc. PD:	-0.058
		Sc. rank:	-39569.0

PA-X

Pos.	152	obs:	exp:
ttt	F	67	19623.00
ttc	F	40275	20719.00
tat	Y	0	1.58
tac	Y	3	1.42
---	--	------	------
mPD		0.0035	0.50
		nPD:	0.01
		N. weight:	0.72
		Sc. PD:	-0.025
		Sc. rank:	-6621.6

PA-X

Pos.	153	obs:	exp:
att	I	10	3.51
atc	I	0	2.63
ata	I	0	3.86
act	T	40125	12524.00
acc	T	144	7130.00
aca	T	16	17453.00
acg	T	44	3221.00
aat	N	1	0.56
aac	N	0	0.44
gct	A	5	1.36
gcc	A	0	1.25
gca	A	0	2.02
gcg	A	0	0.37
---	--	------	------
mPD		0.011	0.68
		nPD:	0.02
		N. weight:	1.2
		Sc. PD:	-0.033
		Sc. rank:	6517.5

PA-X

Pos.	154	obs:	exp:
ggt	G	5	5431.00
ggc	G	0	5631.00
gga	G	727	18624.00
ggg	G	39613	10659.00
---	--	------	------
mPD		0.036	0.68
		nPD:	0.05
		N. weight:	1.3
		Sc. PD:	0.011
		Sc. rank:	26828.0

PA-X

Pos.	155	obs:	exp:
gat	D	4	2.83
gac	D	1	2.17
gaa	E	476	23783.00
gag	E	39864	16557.00
---	--	------	------
mPD		0.024	0.48
		nPD:	0.05
		N. weight:	0.9
		Sc. PD:	0.0039
		Sc. rank:	16665.0

PA-X

Pos.	156	obs:	exp:
gaa	E	40167	23786.00
gag	E	178	16559.00
---	--	------	------
mPD		0.0088	0.48
		nPD:	0.02
		N. weight:	0.55
		Sc. PD:	-0.014
		Sc. rank:	4043.9

PA-X

Pos.	157	obs:	exp:
tta	L	0	8.59
ttg	L	1	15.30
ctt	L	0	15.48
ctc	L	0	10.85
cta	L	0	11.36
ctg	L	73	12.42
att	I	5	21.76
atc	I	0	16.29
ata	I	57	23.94
atg	M	40205	40205.00
gtt	V	0	1.00
gtc	V	0	0.82
gta	V	0	0.87
gtg	V	4	1.32
---	--	------	------
mPD		0.0069	0.0094
		nPD:	0.74
		N. weight:	0.0048
		Sc. PD:	0.0032
		Sc. rank:	151.1

PA-X

Pos.	158	obs:	exp:
gtt	V	0	0.75
gtc	V	3	0.61
gta	V	0	0.65
gtg	V	0	0.99
gct	A	121	10995.00
gcc	A	39340	10079.00
gca	A	881	16319.00
gcg	A	0	2949.00
---	--	------	------
mPD		0.049	0.69
		nPD:	0.07
		N. weight:	1.4
		Sc. PD:	0.034
		Sc. rank:	30886.0

PA-X

Pos.	159	obs:	exp:
tct	S	0	0.81
tcc	S	0	0.75
tca	S	5	1.34
tcg	S	0	0.20
cct	P	0	0.67
ccc	P	0	0.37
cca	P	2	0.68
ccg	P	0	0.28
atg	M	1	1.00
act	T	6	12526.00
acc	T	17	7131.00
aca	T	38657	17456.00
acg	T	1655	3222.00
aaa	K	2	1.31
aag	K	0	0.69
agt	S	0	0.87
agc	S	0	1.03
---	--	------	------
mPD		0.080	0.68
		nPD:	0.12
		N. weight:	0.8
		Sc. PD:	0.056
		Sc. rank:	21076.0

PA-X

Pos.	160	obs:	exp:
tct	S	0	0.16
tcc	S	0	0.15
tca	S	0	0.27
tcg	S	0	0.04
cgt	R	0	303.40
cgc	R	0	550.60
cga	R	0	1396.00
cgg	R	0	1314.00
atg	M	2	2.00
act	T	0	0.31
acc	T	0	0.18
aca	T	0	0.43
acg	T	1	0.08
aat	N	5	2.80
aac	N	0	2.20
aaa	K	109	16440.00
aag	K	24897	8566.00
agt	S	1	0.17
agc	S	0	0.21
aga	R	36	7505.00
agg	R	15266	4233.00
gaa	E	0	2.36
gag	E	4	1.64
ggt	G	0	3.23
ggc	G	0	3.35
gga	G	0	11.08
ggg	G	24	6.34
---	--	------	------
mPD		0.48	1.1
		nPD:	0.43
		N. weight:	1.2
		Sc. PD:	0.45
		Sc. rank:	38483.0

,

PA-X

Pos.	161	obs:	exp:
tct	S	0	0.32
tcc	S	0	0.30
tca	S	2	0.54
tcg	S	0	0.08
act	T	0	2.17
acc	T	0	1.24
aca	T	7	3.03
acg	T	0	0.56
agt	S	0	0.35
agc	S	0	0.41
gtt	V	0	0.50
gtc	V	0	0.41
gta	V	2	0.43
gtg	V	0	0.66
gct	A	25	10993.00
gcc	A	62	10077.00
gca	A	40220	16315.00
gcg	A	26	2948.00
gaa	E	1	0.59
gag	E	0	0.41
---	--	------	------
mPD		0.0062	0.69
		nPD:	0.01
		N. weight:	0.97
		Sc. PD:	-0.032
		Sc. rank:	-4489.3

,

PA-X

Pos.	162	obs:	exp:
tat	Y	0	0.53
tac	Y	1	0.47
aat	N	0	2.24
aac	N	4	1.76
gat	D	154	22800.00
gac	D	40182	17536.00
gaa	E	3	2.36
gag	E	1	1.64
---	--	------	------
mPD		0.0081	0.49
		nPD:	0.02
		N. weight:	0.88
		Sc. PD:	-0.023
		Sc. rank:	5071.4

,

PA-X

Pos.	163	obs:	exp:
tat	Y	64	21265.00
tac	Y	40279	19078.00
cat	H	0	1.13
cac	H	2	0.87
---	--	------	------
mPD		0.0033	0.50
		nPD:	0.01
		N. weight:	0.81
		Sc. PD:	-0.029
		Sc. rank:	-9542.7

,

PA-X

Pos.	164	obs:	exp:
tct	S	1	0.16
tcc	S	0	0.15
tca	S	0	0.27
tcg	S	0	0.04
cct	P	1	0.33
ccc	P	0	0.19
cca	P	0	0.34
ccg	P	0	0.14
att	I	10	3.51
atc	I	0	2.63
ata	I	0	3.86
act	T	39401	12524.00
acc	T	906	7130.00
aca	T	21	17453.00
acg	T	1	3221.00
aat	N	2	1.12
aac	N	0	0.88
agt	S	0	0.17
agc	S	0	0.21
gct	A	2	0.55
gcc	A	0	0.50
gca	A	0	0.81
gcg	A	0	0.15
---	--	------	------
mPD		0.046	0.68
		nPD:	0.07
		N. weight:	1.2
		Sc. PD:	0.026
		Sc. rank:	25435.0

,

PA-X

Pos.	165	obs:	exp:
tta	L	1	4679.00
ttg	L	0	8336.00
ctt	L	749	8434.00
ctc	L	38895	5912.00
cta	L	674	6191.00
ctg	L	0	6767.00
cgt	R	1	0.02
cgc	R	0	0.04
cga	R	0	0.09
cgg	R	0	0.09
att	I	0	8.78
atc	I	25	6.57
ata	I	0	9.65
aga	R	0	0.49
agg	R	0	0.28
---	--	------	------
mPD		0.070	1.2
		nPD:	0.06
		N. weight:	1.9
		Sc. PD:	0.029
		Sc. rank:	40531.0

,

PA-X

Pos.	166	obs:	exp:
gat	D	748	22805.00
gac	D	39596	17539.00
ggt	G	0	0.13
ggc	G	1	0.14
gga	G	0	0.46
ggg	G	0	0.26
---	--	------	------
mPD		0.036	0.49
		nPD:	0.07
		N. weight:	0.81
		Sc. PD:	0.023
		Sc. rank:	18744.0

,

PA-X

Pos.	167	obs:	exp:
caa	Q	0	0.61
cag	Q	1	0.39
gat	D	6	3.39
gac	D	0	2.61
gaa	E	877	23782.00
gag	E	39461	16556.00
---	--	------	------
mPD		0.043	0.48
		nPD:	0.09
		N. weight:	0.85
		Sc. PD:	0.036
		Sc. rank:	21540.0

,

PA-X

Pos.	168	obs:	exp:
caa	Q	1	0.61
cag	Q	0	0.39
gaa	E	39156	23785.00
gag	E	1188	16559.00
---	--	------	------
mPD		0.057	0.48
		nPD:	0.12
		N. weight:	0.44
		Sc. PD:	0.031
		Sc. rank:	11625.0

,

PA-X

Pos.	169	obs:	exp:
tct	S	0	6510.00
tcc	S	0	6029.00
tca	S	0	10843.00
tcg	S	0	1588.00
aat	N	0	0.56
aac	N	1	0.44
agt	S	70	7050.00
agc	S	40273	8323.00
ggt	G	0	0.13
ggc	G	1	0.14
gga	G	0	0.46
ggg	G	0	0.26
---	--	------	------
mPD		0.0036	1.6
		nPD:	0.
		N. weight:	1.7
		Sc. PD:	-0.068
		Sc. rank:	-44557.0

,

PA-X

Pos.	170	obs:	exp:
cgt	R	0	799.90
cgc	R	0	1452.00
cga	R	0	3681.00
cgg	R	5	3464.00
aga	R	352	19789.00
agg	R	39988	11159.00
---	--	------	------
mPD		0.018	0.89
		nPD:	0.02
		N. weight:	1.3
		Sc. PD:	-0.031
		Sc. rank:	12185.0

,

PA-X

Pos.	171	obs:	exp:
gct	A	40248	10996.00
gcc	A	87	10080.00
gca	A	7	16320.00
gcg	A	3	2949.00
---	--	------	------
mPD		0.0048	0.69
		nPD:	0.01
		N. weight:	1.4
		Sc. PD:	-0.049
		Sc. rank:	-13483.0

,

PA-X

Pos.	172	obs:	exp:
cgt	R	0	799.90
cgc	R	0	1452.00
cga	R	0	3681.00
cgg	R	1	3464.00
aga	R	248	19788.00
agg	R	40094	11159.00
ggt	G	0	0.27
ggc	G	0	0.28
gga	G	0	0.92
ggg	G	2	0.53
---	--	------	------
mPD		0.012	0.89
		nPD:	0.01
		N. weight:	1.4
		Sc. PD:	-0.039
		Sc. rank:	3283.3

,

PA-X

Pos.	173	obs:	exp:
att	I	11929	14160.00
atc	I	28373	10599.00
ata	I	36	15578.00
aaa	K	0	0.66
aag	K	1	0.34
gtt	V	3	1.50
gtc	V	3	1.22
gta	V	0	1.30
gtg	V	0	1.97
---	--	------	------
mPD		0.42	0.66
		nPD:	0.63
		N. weight:	0.7
		Sc. PD:	0.39
		Sc. rank:	21992.0

,

PA-X

Pos.	174	obs:	exp:
att	I	0	0.35
atc	I	0	0.26
ata	I	1	0.39
aaa	K	38437	26524.00
aag	K	1907	13820.00
---	--	------	------
mPD		0.090	0.45
		nPD:	0.2
		N. weight:	0.28
		Sc. PD:	0.042
		Sc. rank:	8050.7

,

PA-X

Pos.	175	obs:	exp:
tct	S	0	0.32
tcc	S	0	0.30
tca	S	0	0.54
tcg	S	0	0.08
act	T	107	12529.00
acc	T	40203	7133.00
aca	T	28	17460.00
acg	T	5	3222.00
agt	S	0	0.35
agc	S	2	0.41
---	--	------	------
mPD		0.0070	0.68
		nPD:	0.01
		N. weight:	1.9
		Sc. PD:	-0.06
		Sc. rank:	-5078.8

,

PA-X

Pos.	176	obs:	exp:
cgt	R	0	799.90
cgc	R	0	1452.00
cga	R	0	3681.00
cgg	R	2	3464.00
aga	R	268	19789.00
agg	R	40075	11159.00
---	--	------	------
mPD		0.013	0.89
		nPD:	0.02
		N. weight:	1.4
		Sc. PD:	-0.037
		Sc. rank:	4606.5

,

PA-X

Pos.	177	obs:	exp:
tta	L	27	4682.00
ttg	L	138	8341.00
ctt	L	40	8440.00
ctc	L	2	5916.00
cta	L	8511	6195.00
ctg	L	31627	6771.00
---	--	------	------
mPD		0.34	1.2
		nPD:	0.3
		N. weight:	1.4
		Sc. PD:	0.33
		Sc. rank:	40739.0

,

PA-X

Pos.	178	obs:	exp:
ttt	F	40063	19624.00
ttc	F	281	20720.00
gtt	V	1	0.25
gtc	V	0	0.20
gta	V	0	0.22
gtg	V	0	0.33
---	--	------	------
mPD		0.014	0.50
		nPD:	0.03
		N. weight:	0.74
		Sc. PD:	-0.011
		Sc. rank:	8676.3

,

PA-X

Pos.	179	obs:	exp:
att	I	0	2.81
atc	I	8	2.10
ata	I	0	3.09
act	T	75	12526.00
acc	T	40180	7131.00
aca	T	81	17456.00
acg	T	0	3222.00
gct	A	0	0.27
gcc	A	1	0.25
gca	A	0	0.40
gcg	A	0	0.07
---	--	------	------
mPD		0.0082	0.68
		nPD:	0.01
		N. weight:	1.9
		Sc. PD:	-0.057
		Sc. rank:	-917.5

,

PA-X

Pos.	180	obs:	exp:
tta	L	0	8.70
ttg	L	0	15.51
ctt	L	0	15.69
ctc	L	0	11.00
cta	L	75	11.52
ctg	L	0	12.59
att	I	11	14136.00
atc	I	2	10581.00
ata	I	40256	15552.00
gtt	V	0	0.25
gtc	V	0	0.20
gta	V	1	0.22
gtg	V	0	0.33
---	--	------	------
mPD		0.0044	0.66
		nPD:	0.01
		N. weight:	1.
		Sc. PD:	-0.037
		Sc. rank:	-10614.0

,

PA-X

Pos.	181	obs:	exp:
cgt	R	0	799.90
cgc	R	0	1452.00
cga	R	134	3681.00
cgg	R	0	3464.00
aga	R	38956	19789.00
agg	R	1255	11159.00
---	--	------	------
mPD		0.067	0.89
		nPD:	0.08
		N. weight:	0.63
		Sc. PD:	0.019
		Sc. rank:	14832.0

,

PA-X

Pos.	182	obs:	exp:
caa	Q	40245	24662.00
cag	Q	100	15683.00
---	--	------	------
mPD		0.0049	0.48
		nPD:	0.01
		N. weight:	0.52
		Sc. PD:	-0.017
		Sc. rank:	-1351.8

,

PA-X

Pos.	183	obs:	exp:
gat	D	0	2.83
gac	D	5	2.17
gaa	E	39792	23783.00
gag	E	548	16557.00
---	--	------	------
mPD		0.027	0.48
		nPD:	0.06
		N. weight:	0.51
		Sc. PD:	0.0056
		Sc. rank:	10322.0

,

PA-X

Pos.	184	obs:	exp:
tta	L	0	10.44
ttg	L	6	18.61
ctt	L	0	18.83
ctc	L	0	13.20
cta	L	0	13.82
ctg	L	84	15.10
atg	M	40255	40255.00
---	--	------	------
mPD		0.0045	0.0072
		nPD:	0.61
		N. weight:	0.0038
		Sc. PD:	0.002
		Sc. rank:	118.5

,

PA-X

Pos.	185	obs:	exp:
gct	A	455	10996.00
gcc	A	39840	10080.00
gca	A	50	16320.00
gcg	A	0	2949.00
---	--	------	------
mPD		0.025	0.69
		nPD:	0.04
		N. weight:	1.4
		Sc. PD:	-0.012
		Sc. rank:	22323.0

,

PA-X

Pos.	186	obs:	exp:
tct	S	0	4.52
tcc	S	0	4.18
tca	S	0	7.53
tcg	S	0	1.10
cat	H	0	1.13
cac	H	2	0.87
act	T	0	0.31
acc	T	1	0.18
aca	T	0	0.43
acg	T	0	0.08
aat	N	332	22554.00
aac	N	39981	17759.00
aaa	K	1	0.66
aag	K	0	0.34
agt	S	6	4.89
agc	S	22	5.78
---	--	------	------
mPD		0.018	0.50
		nPD:	0.04
		N. weight:	0.84
		Sc. PD:	-0.0059
		Sc. rank:	13551.0

,

PA-X

Pos.	187	obs:	exp:
cgt	R	0	799.70
cgc	R	0	1451.00
cga	R	4	3680.00
cgg	R	0	3463.00
aaa	K	11	7.23
aag	K	0	3.77
aga	R	40221	19783.00
agg	R	108	11156.00
ggt	G	0	0.13
ggc	G	0	0.14
gga	G	1	0.46
ggg	G	0	0.26
---	--	------	------
mPD		0.0061	0.89
		nPD:	0.01
		N. weight:	0.76
		Sc. PD:	-0.027
		Sc. rank:	-7161.8

,

PA-X

Pos.	188	obs:	exp:
tct	S	0	1.29
tcc	S	0	1.20
tca	S	0	2.15
tcg	S	0	0.31
agt	S	1	1.40
agc	S	7	1.65
ggt	G	77	5430.00
ggc	G	40240	5630.00
gga	G	20	18620.00
ggg	G	0	10657.00
---	--	------	------
mPD		0.0052	0.68
		nPD:	0.01
		N. weight:	2.1
		Sc. PD:	-0.074
		Sc. rank:	-13300.0

,

PA-X

Pos.	189	obs:	exp:
ttt	F	0	0.49
ttc	F	1	0.51
tta	L	3	4679.00
ttg	L	0	8336.00
ctt	L	40	8435.00
ctc	L	39953	5913.00
cta	L	325	6192.00
ctg	L	0	6767.00
att	I	0	8.07
atc	I	23	6.04
ata	I	0	8.88
---	--	------	------
mPD		0.019	1.2
		nPD:	0.02
		N. weight:	2.
		Sc. PD:	-0.053
		Sc. rank:	12720.0

,

PA-X

Pos.	190	obs:	exp:
tgg	W	40345	40345.00
---	--	------	------
mPD		0	0
		nPD:	1.
		N. weight:	0.
		Sc. PD:	0
		Sc. rank:	0

,

PA-X

Pos.	191	obs:	exp:
gat	D	40278	22805.00
gac	D	67	17540.00
---	--	------	------
mPD		0.0033	0.49
		nPD:	0.01
		N. weight:	0.61
		Sc. PD:	-0.022
		Sc. rank:	-6150.1

,

PA-X

Pos.	192	obs:	exp:
ttt	F	0	2.43
ttc	F	5	2.57
tct	S	25	6507.00
tcc	S	40235	6026.00
tca	S	63	10838.00
tcg	S	0	1587.00
cct	P	0	5.33
ccc	P	16	3.00
cca	P	0	5.41
ccg	P	0	2.26
act	T	0	0.31
acc	T	1	0.18
aca	T	0	0.43
acg	T	0	0.08
agt	S	0	7047.00
agc	S	0	8319.00
---	--	------	------
mPD		0.0054	1.6
		nPD:	0.
		N. weight:	2.1
		Sc. PD:	-0.08
		Sc. rank:	-42303.0

,

PA-X

Pos.	193	obs:	exp:
ttt	F	40337	19624.00
ttc	F	7	20720.00
tta	L	0	0.12
ttg	L	0	0.21
ctt	L	1	0.21
ctc	L	0	0.15
cta	L	0	0.15
ctg	L	0	0.17
---	--	------	------
mPD		0.00040	0.50
		nPD:	0.
		N. weight:	0.79
		Sc. PD:	-0.032
		Sc. rank:	-23937.0

,

PA-X

Pos.	194	obs:	exp:
ttt	F	0	0.49
ttc	F	1	0.51
cgt	R	11	0.22
cgc	R	0	0.40
cga	R	0	1.00
cgg	R	0	0.94
att	I	0	3.16
atc	I	9	2.36
ata	I	0	3.48
aga	R	0	5.40
agg	R	0	3.04
gtt	V	0	10093.00
gtc	V	40293	8211.00
gta	V	4	8734.00
gtg	V	0	13259.00
gct	A	0	5.72
gcc	A	21	5.25
gca	A	0	8.49
gcg	A	0	1.54
gat	D	0	1.13
gac	D	2	0.87
ggt	G	0	0.54
ggc	G	4	0.56
gga	G	0	1.85
ggg	G	0	1.06
---	--	------	------
mPD		0.0037	0.74
		nPD:	0.
		N. weight:	1.7
		Sc. PD:	-0.065
		Sc. rank:	-29812.0

,

PA-X

Pos.	195	obs:	exp:
tct	S	0	5287.00
tcc	S	0	4896.00
tca	S	0	8807.00
tcg	S	0	1290.00
tgt	C	35	13.68
tgc	C	0	21.32
caa	Q	1	1.22
cag	Q	1	0.78
att	I	6	2.11
atc	I	0	1.58
ata	I	0	2.32
act	T	2	0.62
acc	T	0	0.35
aca	T	0	0.87
acg	T	0	0.16
aat	N	7535	4216.00
aac	N	0	3319.00
agt	S	32765	5726.00
agc	S	0	6759.00
---	--	------	------
mPD		0.31	1.8
		nPD:	0.17
		N. weight:	1.7
		Sc. PD:	0.2
		Sc. rank:	45890.0

,

PA-X

Pos.	196	obs:	exp:
tta	L	0	7.19
ttg	L	0	12.82
tct	S	0	0.32
tcc	S	2	0.30
tca	S	0	0.54
tcg	S	0	0.08
ctt	L	0	12.97
ctc	L	0	9.09
cta	L	0	9.52
ctg	L	62	10.41
cct	P	0	13413.00
ccc	P	0	7546.00
cca	P	0	13599.00
ccg	P	40250	5693.00
caa	Q	0	18.95
cag	Q	31	12.05
agt	S	0	0.35
agc	S	0	0.41
---	--	------	------
mPD		0.0048	0.73
		nPD:	0.01
		N. weight:	2.1
		Sc. PD:	-0.076
		Sc. rank:	-22377.0

,

PA-X

Pos.	197	obs:	exp:
cgt	R	0	10.65
cgc	R	0	19.32
cga	R	0	49.00
cgg	R	0	46.11
aat	N	0	1.68
aac	N	3	1.32
aaa	K	39804	26169.00
aag	K	0	13635.00
aga	R	537	263.40
agg	R	0	148.50
gaa	E	1	0.59
gag	E	0	0.41
---	--	------	------
mPD		0.026	0.48
		nPD:	0.05
		N. weight:	0.46
		Sc. PD:	0.0046
		Sc. rank:	9287.4

,

PA-X

Pos.	198	obs:	exp:
cgt	R	0	0.02
cgc	R	0	0.04
cga	R	0	0.09
cgg	R	0	0.09
aaa	K	0	1.31
aag	K	2	0.69
aga	R	1	0.49
agg	R	0	0.28
gtt	V	0	0.75
gtc	V	0	0.61
gta	V	0	0.65
gtg	V	3	0.99
gaa	E	10	23779.00
gag	E	40324	16555.00
ggt	G	0	0.67
ggc	G	0	0.70
gga	G	0	2.31
ggg	G	5	1.32
---	--	------	------
mPD		0.0011	0.48
		nPD:	0.
		N. weight:	0.97
		Sc. PD:	-0.038
		Sc. rank:	-24395.0

,

PA-X

Pos.	199	obs:	exp:
gtt	V	0	3.76
gtc	V	0	3.06
gta	V	0	3.25
gtg	V	15	4.94
gct	A	0	10986.00
gcc	A	0	10070.00
gca	A	1	16304.00
gcg	A	40306	2947.00
gaa	E	0	12.97
gag	E	22	9.03
ggt	G	0	0.13
ggc	G	1	0.14
gga	G	0	0.46
ggg	G	0	0.26
---	--	------	------
mPD		0.0020	0.70
		nPD:	0.
		N. weight:	2.9
		Sc. PD:	-0.11
		Sc. rank:	-66501.0

,

PA-X

Pos.	200	obs:	exp:
cgt	R	0	0.95
cgc	R	0	1.73
cga	R	0	4.38
cgg	R	0	4.12
aaa	K	2	26492.00
aag	K	40294	13804.00
aga	R	0	23.54
agg	R	48	13.28
gaa	E	1	0.59
gag	E	0	0.41
---	--	------	------
mPD		0.0026	0.45
		nPD:	0.01
		N. weight:	1.2
		Sc. PD:	-0.043
		Sc. rank:	-17767.0

,

PA-X

Pos.	201	obs:	exp:
cgt	R	0	1.61
cgc	R	0	2.91
cga	R	0	7.39
cgg	R	0	6.96
act	T	0	1.55
acc	T	0	0.88
aca	T	5	2.16
acg	T	0	0.40
aaa	K	40252	26463.00
aag	K	0	13789.00
aga	R	81	39.73
agg	R	0	22.40
gaa	E	7	4.13
gag	E	0	2.87
---	--	------	------
mPD		0.0046	0.46
		nPD:	0.01
		N. weight:	0.46
		Sc. PD:	-0.015
		Sc. rank:	-1699.6

,

PA-X

Pos.	202	obs:	exp:
cct	P	0	0.33
ccc	P	0	0.19
cca	P	1	0.34
ccg	P	0	0.14
cat	H	0	1.69
cac	H	3	1.31
caa	Q	40291	24658.00
cag	Q	48	15681.00
cgt	R	0	0.02
cgc	R	0	0.04
cga	R	1	0.09
cgg	R	0	0.09
act	T	0	0.31
acc	T	0	0.18
aca	T	1	0.43
acg	T	0	0.08
aga	R	0	0.49
agg	R	0	0.28
---	--	------	------
mPD		0.0027	0.48
		nPD:	0.01
		N. weight:	0.53
		Sc. PD:	-0.019
		Sc. rank:	-7956.5

,

PA-X

Pos.	203	obs:	exp:
tta	L	1	4664.00
ttg	L	40181	8310.00
tct	S	0	23.72
tcc	S	0	21.97
tca	S	0	39.51
tcg	S	147	5.79
tgg	W	2	2.00
ctt	L	0	8408.00
ctc	L	0	5894.00
cta	L	0	6172.00
ctg	L	12	6746.00
cct	P	0	0.33
ccc	P	0	0.19
cca	P	0	0.34
ccg	P	1	0.14
att	I	1	0.35
atc	I	0	0.26
ata	I	0	0.39
agt	S	0	25.69
agc	S	0	30.33
---	--	------	------
mPD		0.0082	1.2
		nPD:	0.01
		N. weight:	1.7
		Sc. PD:	-0.061
		Sc. rank:	-15260.0

,

PA-X

Pos.	204	obs:	exp:
cgt	R	0	0.40
cgc	R	0	0.72
cga	R	0	1.82
cgg	R	0	1.72
aaa	K	0	26510.00
aag	K	40324	13814.00
aga	R	0	9.81
agg	R	20	5.53
gaa	E	1	0.59
gag	E	0	0.41
---	--	------	------
mPD		0.0011	0.45
		nPD:	0.
		N. weight:	1.2
		Sc. PD:	-0.046
		Sc. rank:	-28688.0

,

PA-X

Pos.	205	obs:	exp:
cgt	R	0	1.23
cgc	R	0	2.23
cga	R	0	5.66
cgg	R	0	5.32
att	I	0	0.35
atc	I	0	0.26
ata	I	1	0.39
aaa	K	40282	26483.00
aag	K	0	13799.00
aga	R	62	30.41
agg	R	0	17.15
---	--	------	------
mPD		0.0031	0.45
		nPD:	0.01
		N. weight:	0.46
		Sc. PD:	-0.016
		Sc. rank:	-4570.7

,

PA-X

Pos.	206	obs:	exp:
tct	S	0	4.68
tcc	S	0	4.33
tca	S	0	7.79
tcg	S	0	1.14
att	I	1	0.35
atc	I	0	0.26
ata	I	0	0.39
aat	N	40212	22497.00
aac	N	0	17715.00
agt	S	29	5.07
agc	S	0	5.98
gat	D	97	54.83
gac	D	0	42.17
ggt	G	6	0.81
ggc	G	0	0.84
gga	G	0	2.77
ggg	G	0	1.59
---	--	------	------
mPD		0.0069	0.50
		nPD:	0.01
		N. weight:	0.64
		Sc. PD:	-0.018
		Sc. rank:	1325.0

,

PA-X

Pos.	207	obs:	exp:
tta	L	20	4682.00
ttg	L	40325	8341.00
ctt	L	0	8440.00
ctc	L	0	5916.00
cta	L	0	6195.00
ctg	L	0	6771.00
---	--	------	------
mPD		0.00099	1.2
		nPD:	0.
		N. weight:	1.7
		Sc. PD:	-0.071
		Sc. rank:	-51625.0

,

PA-X

Pos.	208	obs:	exp:
caa	Q	2	1.22
cag	Q	0	0.78
cgt	R	0	0.71
cgc	R	0	1.30
cga	R	0	3.28
cgg	R	0	3.09
att	I	0	0.35
atc	I	0	0.26
ata	I	1	0.39
aat	N	0	0.56
aac	N	1	0.44
aaa	K	40299	26495.00
aag	K	1	13805.00
aga	R	36	17.66
agg	R	0	9.96
gaa	E	5	2.95
gag	E	0	2.05
---	--	------	------
mPD		0.0023	0.45
		nPD:	0.01
		N. weight:	0.46
		Sc. PD:	-0.017
		Sc. rank:	-7727.2

,

PA-X

Pos.	209	obs:	exp:
tta	L	48	5.57
ttg	L	0	9.92
tct	S	520	6503.00
tcc	S	0	6022.00
tca	S	39774	10831.00
tcg	S	3	1586.00
ctt	L	0	10.04
ctc	L	0	7.04
cta	L	0	7.37
ctg	L	0	8.06
agt	S	0	7042.00
agc	S	0	8313.00
---	--	------	------
mPD		0.028	1.6
		nPD:	0.02
		N. weight:	1.4
		Sc. PD:	-0.035
		Sc. rank:	9002.5

,

PA-X

Pos.	210	obs:	exp:
tta	L	0	6.27
ttg	L	0	11.16
ctt	L	0	11.30
ctc	L	0	7.92
cta	L	0	8.29
ctg	L	54	9.06
caa	Q	31	24620.00
cag	Q	40246	15657.00
cgt	R	0	0.10
cgc	R	0	0.18
cga	R	0	0.46
cgg	R	5	0.43
aaa	K	0	5.92
aag	K	9	3.08
aga	R	0	2.45
agg	R	0	1.38
---	--	------	------
mPD		0.0049	0.48
		nPD:	0.01
		N. weight:	1.
		Sc. PD:	-0.033
		Sc. rank:	-3545.2

,

PA-X

Pos.	211	obs:	exp:
aaa	K	2	1.31
aag	K	0	0.69
gtt	V	0	1.00
gtc	V	0	0.82
gta	V	4	0.87
gtg	V	0	1.32
gct	A	0	0.55
gcc	A	0	0.50
gca	A	2	0.81
gcg	A	0	0.15
gaa	E	39441	23265.00
gag	E	20	16196.00
ggt	G	0	117.90
ggc	G	0	122.30
gga	G	874	404.40
ggg	G	2	231.40
---	--	------	------
mPD		0.044	0.53
		nPD:	0.08
		N. weight:	0.58
		Sc. PD:	0.021
		Sc. rank:	13847.0

,

PA-X			
Pos.	212	obs:	exp:
tta	L	15	4565.00
ttg	L	0	8134.00
ctt	L	2	8230.00
ctc	L	5	5769.00
cta	L	39302	6042.00
ctg	L	20	6603.00
cct	P	0	315.60
ccc	P	0	177.50
cca	P	947	319.90
ccg	P	0	133.90
caa	Q	47	28.73
cag	Q	0	18.27
cgt	R	0	0.10
cgc	R	0	0.18
cga	R	5	0.46
cgg	R	0	0.43
att	I	0	0.70
atc	I	0	0.53
ata	I	2	0.77
aga	R	0	2.45
agg	R	0	1.38
---	--	------	------
mPD		0.051	1.2
		nPD:	0.04
		N. weight:	2.
		Sc. PD:	-0.004
		Sc. rank:	35933.0

,

PA-X			
Pos.	213	obs:	exp:
ttt	F	0	4.86
ttc	F	10	5.14
tct	S	0	0.48
tcc	S	3	0.45
tca	S	0	0.81
tcg	S	0	0.12
tat	Y	0	28.99
tac	Y	55	26.01
tgt	C	1	15740.00
tgc	C	40276	24537.00
agt	S	0	0.52
agc	S	0	0.62
---	--	------	------
mPD		0.0034	0.48
		nPD:	0.01
		N. weight:	0.54
		Sc. PD:	-0.019
		Sc. rank:	-4526.3

,

PA-X			
Pos.	214	obs:	exp:
tct	S	0	2.42
tcc	S	0	2.24
tca	S	15	4.03
tcg	S	0	0.59
att	I	0	0.35
atc	I	0	0.26
ata	I	1	0.39
act	T	0	1.86
acc	T	0	1.06
aca	T	6	2.60
acg	T	0	0.48
agt	S	0	2.62
agc	S	0	3.09
gtt	V	0	621.20
gtc	V	0	505.30
gta	V	2480	537.50
gtg	V	0	816.00
gct	A	1	10291.00
gcc	A	7	9433.00
gca	A	37750	15273.00
gcg	A	0	2760.00
gaa	E	85	50.11
gag	E	0	34.89
---	--	------	------
mPD		0.12	0.82
		nPD:	0.15
		N. weight:	1.
		Sc. PD:	0.1
		Sc. rank:	27379.0

,

PA-X			
Pos.	215	obs:	exp:
tct	S	0	4.03
tcc	S	0	3.74
tca	S	0	6.72
tcg	S	0	0.98
tgt	C	0	3.13
tgc	C	8	4.87
agt	S	0	4.37
agc	S	25	5.16
gtt	V	0	0.25
gtc	V	1	0.20
gta	V	0	0.22
gtg	V	0	0.33
gat	D	0	5.65
gac	D	10	4.35
ggt	G	6	5426.00
ggc	G	40292	5625.00
gga	G	3	18604.00
ggg	G	0	10647.00
---	--	------	------
mPD		0.0026	0.68
		nPD:	0.
		N. weight:	2.2
		Sc. PD:	-0.082
		Sc. rank:	-41320.0

,

PA-X			
Pos.	216	obs:	exp:
tta	L	3	4679.00
ttg	L	40315	8336.00
tct	S	0	3.55
tcc	S	0	3.29
tca	S	0	5.91
tcg	S	22	0.87
tgg	W	5	5.00
ctt	L	0	8434.00
ctc	L	0	5912.00
cta	L	0	6191.00
ctg	L	0	6766.00
agt	S	0	3.84
agc	S	0	4.54
---	--	------	------
mPD		0.0015	1.2
		nPD:	0.
		N. weight:	1.7
		Sc. PD:	-0.07
		Sc. rank:	-49440.0

,

PA-X			
Pos.	217	obs:	exp:
tta	L	0	30.87
ttg	L	0	54.99
ctt	L	0	55.64
ctc	L	0	39.01
cta	L	2	40.85
ctg	L	264	44.64
cct	P	3	13337.00
ccc	P	2	7503.00
cca	P	944	13522.00
ccg	P	39073	5661.00
caa	Q	0	33.62
cag	Q	55	21.38
cgt	R	0	0.02
cgc	R	0	0.04
cga	R	0	0.09
cgg	R	1	0.09
aga	R	0	0.49
agg	R	0	0.28
gct	A	0	0.27
gcc	A	0	0.25
gca	A	0	0.40
gcg	A	1	0.07
---	--	------	------
mPD		0.062	0.74
		nPD:	0.08
		N. weight:	2.
		Sc. PD:	0.075
		Sc. rank:	48579.0

,

PA-X			
Pos.	218	obs:	exp:
tct	S	0	0.81
tcc	S	5	0.75
tca	S	0	1.34
tcg	S	0	0.20
att	I	0	21.76
atc	I	62	16.29
ata	I	0	23.94
act	T	0	12503.00
acc	T	40259	7118.00
aca	T	3	17424.00
acg	T	0	3216.00
aat	N	0	7.27
aac	N	13	5.73
agt	S	0	0.87
agc	S	0	1.03
gct	A	0	0.82
gcc	A	3	0.75
gca	A	0	1.21
gcg	A	0	0.22
---	--	------	------
mPD		0.0043	0.68
		nPD:	0.01
		N. weight:	1.9
		Sc. PD:	-0.068
		Sc. rank:	-23824.0

,

PA-X			
Pos.	219	obs:	exp:
cgt	R	0	0.24
cgc	R	0	0.43
cga	R	0	1.09
cgg	R	0	1.03
att	I	0	0.35
atc	I	0	0.26
ata	I	1	0.39
aaa	K	40330	26516.00
aag	K	2	13816.00
aga	R	12	5.89
agg	R	0	3.32
---	--	------	------
mPD		0.00074	0.45
		nPD:	0.
		N. weight:	0.46
		Sc. PD:	-0.018
		Sc. rank:	-12382.0

,

PA-X			
Pos.	220	obs:	exp:
gtt	V	0	10050.00
gtc	V	40116	8175.00
gta	V	6	8696.00
gtg	V	1	13201.00
gct	A	0	60.51
gcc	A	222	55.46
gca	A	0	89.80
gcg	A	0	16.23
---	--	------	------
mPD		0.011	0.75
		nPD:	0.02
		N. weight:	1.7
		Sc. PD:	-0.048
		Sc. rank:	6627.2

,

PA-X			
Pos.	221	obs:	exp:
ttt	F	0	15.57
ttc	F	32	16.43
tct	S	0	6504.00
tcc	S	40308	6024.00
tca	S	0	10834.00
tcg	S	0	1587.00
tat	Y	0	2.64
tac	Y	5	2.36
agt	S	0	7044.00
agc	S	0	8315.00
---	--	------	------
mPD		0.0018	1.6
		nPD:	0.
		N. weight:	2.1
		Sc. PD:	-0.085
		Sc. rank:	-60456.0

,

PA-X			
Pos.	222	obs:	exp:
tta	L	0	1.28
ttg	L	0	2.27
ctt	L	0	2.30
ctc	L	11	1.61
cta	L	0	1.69
ctg	L	0	1.85
cat	H	2	22479.00
cac	H	39851	17374.00
caa	Q	2	1.22
cag	Q	0	0.78
cgt	R	0	9.50
cgc	R	479	17.23
cga	R	0	43.70
cgg	R	0	41.13
aga	R	0	234.90
agg	R	0	132.50
---	--	------	------
mPD		0.024	0.55
		nPD:	0.04
		N. weight:	0.94
		Sc. PD:	0.00023
		Sc. rank:	16961.0

,

PA-X			
Pos.	223	obs:	exp:
tta	L	0	1.04
ttg	L	0	1.86
ctt	L	0	1.88
ctc	L	0	1.32
cta	L	9	1.38
ctg	L	0	1.51
caa	Q	10	6.11
cag	Q	0	3.89
cgt	R	1	799.60
cgc	R	4	1451.00
cga	R	40321	3679.00
cgg	R	0	3463.00
aga	R	0	19779.00
agg	R	0	11154.00
---	--	------	------
mPD		0.0012	0.89
		nPD:	0.
		N. weight:	2.6
		Sc. PD:	-0.11
		Sc. rank:	-73379.0

,

PA-X

Pos.	224	obs:	exp:
att	I	23	8.07
atc	I	0	6.04
ata	I	0	8.88
act	T	40309	12518.00
acc	T	0	7127.00
aca	T	0	17445.00
acg	T	0	3219.00
aat	N	3	1.68
aac	N	0	1.32
gct	A	10	2.73
gcc	A	0	2.50
gca	A	0	4.05
gcg	A	0	0.73
---	--	------	------
mPD		0.0018	0.68
		nPD:	0.
		N. weight:	1.3
		Sc. PD:	-0.05
		Sc. rank:	-30636.0

PA-X

Pos.	225	obs:	exp:
tct	S	40338	6510.00
tcc	S	7	6029.00
tca	S	0	10844.00
tcg	S	0	1588.00
agt	S	0	7050.00
agc	S	0	8323.00
---	--	------	------
mPD		0.00035	1.6
		nPD:	0.
		N. weight:	2.
		Sc. PD:	-0.083
		Sc. rank:	-63027.0

PA-X

Pos.	226	obs:	exp:
tta	L	0	1.39
ttg	L	0	2.48
tct	S	2	0.32
tcc	S	0	0.30
tca	S	0	0.54
tcg	S	0	0.08
ctt	L	12	2.51
ctc	L	0	1.76
cta	L	0	1.84
ctg	L	0	2.01
cct	P	40290	13436.00
ccc	P	2	7559.00
cca	P	28	13622.00
ccg	P	0	5703.00
cat	H	11	6.20
cac	H	0	4.80
agt	S	0	0.35
agc	S	0	0.41
---	--	------	------
mPD		0.0027	0.72
		nPD:	0.
		N. weight:	1.2
		Sc. PD:	-0.046
		Sc. rank:	-23854.0

PA-X

Pos.	227	obs:	exp:
gtt	V	0	2.50
gtc	V	10	2.04
gta	V	0	2.17
gtg	V	0	3.29
gct	A	8	10993.00
gcc	A	40301	10077.00
gca	A	26	16316.00
gcg	A	0	2949.00
---	--	------	------
mPD		0.0022	0.69
		nPD:	0.
		N. weight:	1.5
		Sc. PD:	-0.059
		Sc. rank:	-33637.0

PA-X

Pos.	228	obs:	exp:
tta	L	14	4680.00
ttg	L	40321	8339.00
tct	S	0	1.13
tcc	S	0	1.05
tca	S	0	1.88
tcg	S	7	0.28
tgg	W	3	3.00
ctt	L	0	8438.00
ctc	L	0	5915.00
cta	L	0	6194.00
ctg	L	0	6769.00
agt	S	0	1.22
agc	S	0	1.44
---	--	------	------
mPD		0.0012	1.2
		nPD:	0.
		N. weight:	1.7
		Sc. PD:	-0.07
		Sc. rank:	-50946.0

PA-X

Pos.	229	obs:	exp:
tct	S	0	0.32
tcc	S	0	0.30
tca	S	0	0.54
tcg	S	0	0.08
cgt	R	0	797.20
cgc	R	0	1447.00
cga	R	3	3669.00
cgg	R	0	3453.00
att	I	0	3.51
atc	I	0	2.63
ata	I	10	3.86
aaa	K	124	81.52
aag	K	0	42.48
agt	S	2	0.35
agc	S	0	0.41
aga	R	40202	19721.00
agg	R	3	11122.00
ggt	G	0	0.13
ggc	G	0	0.14
gga	G	1	0.46
ggg	G	0	0.26
---	--	------	------
mPD		0.0071	0.89
		nPD:	0.01
		N. weight:	0.78
		Sc. PD:	-0.027
		Sc. rank:	-4474.8

PA-X

Pos.	230	obs:	exp:
ttt	F	6	2.92
ttc	F	0	3.08
tta	L	0	1.04
ttg	L	0	1.86
ctt	L	9	1.88
ctc	L	0	1.32
cta	L	0	1.38
ctg	L	0	1.51
att	I	40160	14098.00
atc	I	0	10553.00
ata	I	0	15510.00
act	T	127	39.44
acc	T	0	22.45
aca	T	0	54.96
acg	T	0	10.14
aat	N	16	8.95
aac	N	0	7.05
gtt	V	27	6.76
gtc	V	0	5.50
gta	V	0	5.85
gtg	V	0	8.88
---	--	------	------
mPD		0.0091	0.67
		nPD:	0.01
		N. weight:	1.1
		Sc. PD:	-0.033
		Sc. rank:	2208.1

PA-X

Pos.	231	obs:	exp:
tta	L	40332	4680.00
ttg	L	0	8338.00
tct	S	0	2.10
tcc	S	0	1.94
tca	S	13	3.49
tcg	S	0	0.51
ctt	L	0	8437.00
ctc	L	0	5914.00
cta	L	0	6193.00
ctg	L	0	6769.00
agt	S	0	2.27
agc	S	0	2.68
---	--	------	------
mPD		0.00064	1.2
		nPD:	0.
		N. weight:	2.4
		Sc. PD:	-0.097
		Sc. rank:	-72142.0

PA-X

Pos.	232	obs:	exp:
gat	D	1	0.57
gac	D	0	0.43
gaa	E	15	23777.00
gag	E	40315	16553.00
ggt	G	0	1.88
ggc	G	0	1.95
gga	G	0	6.46
ggg	G	14	3.70
---	--	------	------
mPD		0.0015	0.48
		nPD:	0.
		N. weight:	0.97
		Sc. PD:	-0.038
		Sc. rank:	-21991.0

PA-X

Pos.	233	obs:	exp:
tta	L	0	0.93
ttg	L	0	1.65
tct	S	12	1.94
tcc	S	0	1.79
tca	S	0	3.23
tcg	S	0	0.47
ctt	L	8	1.67
ctc	L	0	1.17
cta	L	0	1.23
ctg	L	0	1.34
cct	P	40292	13427.00
ccc	P	0	7554.00
cca	P	0	13613.00
ccg	P	0	5699.00
cat	H	33	18.61
cac	H	0	14.39
agt	S	0	2.10
agc	S	0	2.48
---	--	------	------
mPD		0.0026	0.72
		nPD:	0.
		N. weight:	1.2
		Sc. PD:	-0.046
		Sc. rank:	-24334.0

PA-X

Pos.	234	obs:	exp:
tta	L	0	0.23
ttg	L	2	0.41
ctt	L	0	0.42
ctc	L	0	0.29
cta	L	0	0.31
ctg	L	0	0.34
atg	M	40343	40343.00
---	--	------	------
mPD		0.000099	0.00016
		nPD:	0.62
		N. weight:	0.000086
		Sc. PD:	0.000046
		Sc. rank:	2.7

PA-X

Pos.	235	obs:	exp:
tta	L	0	0.46
ttg	L	4	0.83
tct	S	0	0.16
tcc	S	0	0.15
tca	S	0	0.27
tcg	S	1	0.04
tgg	W	40340	40340.00
ctt	L	0	0.84
ctc	L	0	0.59
cta	L	0	0.61
ctg	L	0	0.67
agt	S	0	0.17
agc	S	0	0.21
---	--	------	------
mPD		0.00025	0.00055
		nPD:	0.45
		N. weight:	0.00026
		Sc. PD:	0.000099
		Sc. rank:	8.1

PA-X			
Pos.	236	obs:	exp:
att	I	0	7.02
atc	I	0	5.26
ata	I	20	7.72
atg	M	40289	40289.00
act	T	0	11.18
acc	T	0	6.36
aca	T	0	15.58
acg	T	36	2.88
---	--	------	------
mPD		0.0028	0.0044
		nPD:	0.63
		N. weight:	0.003
		Sc. PD:	0.0017
		Sc. rank:	93.7

PA-X			
Pos.	237	obs:	exp:
aat	N	1	0.56
aac	N	0	0.44
gtt	V	1	0.25
gtc	V	0	0.20
gta	V	0	0.22
gtg	V	0	0.33
gct	A	2	0.55
gcc	A	0	0.50
gca	A	0	0.81
gcg	A	0	0.15
gat	D	40321	22792.00
gac	D	0	17529.00
ggt	G	20	2.69
ggc	G	0	2.79
gga	G	0	9.23
ggg	G	0	5.28
---	--	------	------
mPD		0.0012	0.49
		nPD:	0.
		N. weight:	0.63
		Sc. PD:	-0.025
		Sc. rank:	-15474.0

PA-X			
Pos.	238	obs:	exp:
tta	L	0	14.97
ttg	L	129	26.67
tct	S	0	6490.00
tcc	S	0	6010.00
tca	S	88	10809.00
tcg	S	40128	1583.00
ctt	L	0	26.99
ctc	L	0	18.92
cta	L	0	19.81
ctg	L	0	21.65
agt	S	0	7028.00
agc	S	0	8296.00
---	--	------	------
mPD		0.011	1.6
		nPD:	0.01
		N. weight:	3.5
		Sc. PD:	-0.13
		Sc. rank:	-38568.0

PA-X			
Pos.	239	obs:	exp:
tct	S	0	10.49
tcc	S	0	9.71
tca	S	0	17.47
tcg	S	0	2.56
tat	Y	0	3.69
tac	Y	7	3.31
aat	N	2	22527.00
aac	N	40263	17738.00
aaa	K	6	3.94
aag	K	0	2.06
agt	S	0	11.36
agc	S	65	13.41
gat	D	0	1.13
gac	D	2	0.87
---	--	------	------
mPD		0.0041	0.50
		nPD:	0.01
		N. weight:	0.9
		Sc. PD:	-0.031
		Sc. rank:	-4876.0

PA-X			
Pos.	240	obs:	exp:
tta	L	0	0.35
ttg	L	0	0.62
ctt	L	0	0.63
ctc	L	0	0.44
cta	L	3	0.46
ctg	L	0	0.50
caa	Q	104	63.57
cag	Q	0	40.43
cgt	R	0	797.80
cgc	R	0	1448.00
cga	R	40236	3671.00
cgg	R	1	3455.00
aga	R	1	19736.00
agg	R	0	11130.00
---	--	------	------
mPD		0.0054	0.89
		nPD:	0.01
		N. weight:	2.6
		Sc. PD:	-0.094
		Sc. rank:	-35582.0

PA-X			
Pos.	241	obs:	exp:
atg	M	69	69.00
act	T	0	12507.00
acc	T	0	7120.00
aca	T	4	17429.00
acg	T	40269	3217.00
gct	A	0	0.82
gcc	A	0	0.75
gca	A	0	1.21
gcg	A	3	0.22
---	--	------	------
mPD		0.0038	0.68
		nPD:	0.01
		N. weight:	2.8
		Sc. PD:	-0.1
		Sc. rank:	-43764.0

PA-X			
Pos.	242	obs:	exp:
tct	S	1	0.16
tcc	S	0	0.15
tca	S	0	0.27
tcg	S	0	0.04
agt	S	0	0.17
agc	S	0	0.21
gtt	V	36	9.02
gtc	V	0	7.34
gta	V	0	7.80
gtg	V	0	11.84
gct	A	40074	10925.00
gcc	A	9	10015.00
gca	A	2	16215.00
gcg	A	0	2930.00
gat	D	219	123.80
gac	D	0	95.21
ggt	G	4	0.54
ggc	G	0	0.56
gga	G	0	1.85
ggg	G	0	1.06
---	--	------	------
mPD		0.013	0.71
		nPD:	0.02
		N. weight:	1.4
		Sc. PD:	-0.034
		Sc. rank:	11089.0

PA-X			
Pos.	243	obs:	exp:
tta	L	1	0.12
ttg	L	0	0.21
tct	S	0	1.94
tcc	S	0	1.79
tca	S	12	3.23
tcg	S	0	0.47
ctt	L	0	0.21
ctc	L	0	0.15
cta	L	0	0.15
ctg	L	0	0.17
cct	P	0	0.33
ccc	P	0	0.19
cca	P	1	0.34
ccg	P	0	0.14
act	T	0	20.81
acc	T	0	11.85
aca	T	67	29.00
acg	T	0	5.35
agt	S	0	2.10
agc	S	0	2.48
gtt	V	0	10.27
gtc	V	0	8.35
gta	V	41	8.89
gtg	V	0	13.49
gct	A	0	10963.00
gcc	A	2	10049.00
gca	A	40221	16270.00
gcg	A	0	2940.00
---	--	------	------
mPD		0.0062	0.70
		nPD:	0.01
		N. weight:	0.99
		Sc. PD:	-0.033
		Sc. rank:	-4823.4

PA-X			
Pos.	244	obs:	exp:
tta	L	1	4681.00
ttg	L	40340	8340.00
tct	S	0	0.65
tcc	S	0	0.60
tca	S	0	1.08
tcg	S	4	0.16
ctt	L	0	8439.00
ctc	L	0	5916.00
cta	L	0	6195.00
ctg	L	0	6770.00
agt	S	0	0.70
agc	S	0	0.83
---	--	------	------
mPD		0.00025	1.2
		nPD:	0.
		N. weight:	1.7
		Sc. PD:	-0.072
		Sc. rank:	-53788.0

PA-X			
Pos.	245	obs:	exp:
tgg	W	4	4.00
cgt	R	0	798.60
cgc	R	0	1449.00
cga	R	0	3675.00
cgg	R	0	3459.00
aaa	K	0	40.10
aag	K	61	20.90
aga	R	10	19756.00
agg	R	40268	11141.00
ggt	G	0	0.27
ggc	G	0	0.28
gga	G	0	0.92
ggg	G	2	0.53
---	--	------	------
mPD		0.0038	0.89
		nPD:	0.
		N. weight:	1.4
		Sc. PD:	-0.053
		Sc. rank:	-25249.0

PA-X			
Pos.	246	obs:	exp:
gtt	V	0	5.26
gtc	V	0	4.28
gta	V	21	4.55
gtg	V	0	6.91
gct	A	0	10988.00
gcc	A	0	10072.00
gca	A	40315	16308.00
gcg	A	0	2947.00
gaa	E	9	5.31
gag	E	0	3.69
---	--	------	------
mPD		0.0015	0.70
		nPD:	0.
		N. weight:	0.99
		Sc. PD:	-0.039
		Sc. rank:	-26171.0

PA-X			
Pos.	247	obs:	exp:
tct	S	0	6498.00
tcc	S	0	6018.00
tca	S	0	10824.00
tcg	S	0	1585.00
att	I	0	0.70
atc	I	2	0.53
ata	I	0	0.77
aat	N	0	40.84
aac	N	73	32.16
agt	S	0	7037.00
agc	S	40270	8308.00
---	--	------	------
mPD		0.0037	1.6
		nPD:	0.
		N. weight:	1.7
		Sc. PD:	-0.068
		Sc. rank:	-44148.0

PA-X			
Pos.	248	obs:	exp:
ttt	F	40321	19613.00
ttc	F	0	20708.00
tta	L	0	0.46
ttg	L	4	0.83
tct	S	11	1.78
tcc	S	0	1.64
tca	S	0	2.96
tcg	S	0	0.43
tat	Y	2	1.05
tac	Y	0	0.95
tgt	C	7	2.74
tgc	C	0	4.26
ctt	L	0	0.84
ctc	L	0	0.59
cta	L	0	0.61
ctg	L	0	0.67
agt	S	0	1.92
agc	S	0	2.27
---	--	------	------
mPD		0.0012	0.50
		nPD:	0.
		N. weight:	0.79
		Sc. PD:	-0.031
		Sc. rank:	-19653.0

PA-X			
Pos.	249	obs:	exp:
tta	L	0	4661.00
ttg	L	0	8305.00
tct	S	1	0.16
tcc	S	0	0.15
tca	S	0	0.27
tcg	S	0	0.04
ctt	L	0	8403.00
ctc	L	40170	5891.00
cta	L	0	6168.00
ctg	L	0	6742.00
cct	P	0	12.66
ccc	P	38	7.12
cca	P	0	12.84
ccg	P	0	5.37
cat	H	0	75.58
cac	H	134	58.42
cgt	R	0	0.04
cgc	R	2	0.07
cga	R	0	0.18
cgg	R	0	0.17
agt	S	0	0.17
agc	S	0	0.21
aga	R	0	0.98
agg	R	0	0.55
---	--	------	------
mPD		0.0087	1.2
		nPD:	0.01
		N. weight:	2.1
		Sc. PD:	-0.073
		Sc. rank:	-16112.0

PA-X			
Pos.	250	obs:	exp:
caa	Q	1	0.61
cag	Q	0	0.39
cgt	R	0	0.44
cgc	R	0	0.79
cga	R	0	2.01
cgg	R	0	1.89
att	I	0	0.35
atc	I	0	0.26
ata	I	1	0.39
aaa	K	40319	26508.00
aag	K	2	13813.00
aga	R	22	10.79
agg	R	0	6.09
---	--	------	------
mPD		0.0013	0.45
		nPD:	0.
		N. weight:	0.46
		Sc. PD:	-0.018
		Sc. rank:	-10611.0

PA-X			
Pos.	251	obs:	exp:
tat	Y	4	2.11
tac	Y	0	1.89
tgt	C	40323	15765.00
tgc	C	18	24576.00
---	--	------	------
mPD		0.0011	0.48
		nPD:	0.
		N. weight:	1.
		Sc. PD:	-0.041
		Sc. rank:	-25934.0

PA-X			
Pos.	252	obs:	exp:
tta	L	0	5.11
ttg	L	0	9.10
tct	S	0	0.65
tcc	S	0	0.60
tca	S	4	1.08
tcg	S	0	0.16
ctt	L	0	9.20
ctc	L	0	6.45
cta	L	44	6.76
ctg	L	0	7.38
cct	P	0	12945.00
ccc	P	0	7282.00
cca	P	38845	13124.00
ccg	P	0	5494.00
caa	Q	1451	887.00
cag	Q	0	564.00
act	T	0	0.31
acc	T	0	0.18
aca	T	1	0.43
acg	T	0	0.08
agt	S	0	0.70
agc	S	0	0.83
---	--	------	------
mPD		0.072	0.79
		nPD:	0.09
		N. weight:	1.2
		Sc. PD:	0.051
		Sc. rank:	29808.0

PA-X			
Pos.	253	obs:	exp:
cgt	R	0	2.06
cgc	R	0	3.74
cga	R	0	9.49
cgg	R	0	8.93
aaa	K	81	26442.00
aag	K	40139	13778.00
aga	R	0	51.01
agg	R	104	28.77
gaa	E	0	12.38
gag	E	21	8.62
---	--	------	------
mPD		0.010	0.46
		nPD:	0.02
		N. weight:	1.2
		Sc. PD:	-0.024
		Sc. rank:	11202.0

PA-X			
Pos.	254	obs:	exp:
cgt	R	0	1.21
cgc	R	0	2.19
cga	R	0	5.57
cgg	R	0	5.24
atg	M	2	2.00
aat	N	1	0.56
aac	N	0	0.44
aaa	K	0	26477.00
aag	K	40273	13796.00
aga	R	0	29.92
agg	R	61	16.87
gaa	E	0	4.13
gag	E	7	2.87
---	-	1	1.00
---	--	------	------
mPD		0.0035	0.45
		nPD:	0.01
		N. weight:	1.2
		Sc. PD:	-0.04
		Sc. rank:	-6972.0

PA-X			
Pos.	255	obs:	exp:
tta	L	1	0.12
ttg	L	0	0.21
ctt	L	0	0.21
ctc	L	0	0.15
cta	L	0	0.15
ctg	L	0	0.17
---	-	40344	40344.00
---	--	------	------
mPD		0	7.1×10^{-10}
		nPD:	0.
		N. weight:	0.
		Sc. PD:	0
		Sc. rank:	0

PA-X			
Pos.	256	obs:	exp:
atg	M	1	1.00
---	-	40344	40344.00
---	--	------	------
mPD		0	0
		nPD:	1.
		N. weight:	0.
		Sc. PD:	0
		Sc. rank:	0

PA-X			
Pos.	257	obs:	exp:
cct	P	0	0.33
ccc	P	1	0.19
cca	P	0	0.34
ccg	P	0	0.14
---	-	40344	40344.00
---	--	------	------
mPD		0	4.4×10^{-10}
		nPD:	0.
		N. weight:	0.
		Sc. PD:	0
		Sc. rank:	0

PA-X			
Pos.	258	obs:	exp:
aaa	K	1	0.66
aag	K	0	0.34
---	-	40344	40344.00
---	--	------	------
mPD		0	2.8×10^{-10}
		nPD:	0.
		N. weight:	0.
		Sc. PD:	0
		Sc. rank:	0

PA-X			
Pos.	259	obs:	exp:
tta	L	0	0.12
ttg	L	1	0.21
ctt	L	0	0.21
ctc	L	0	0.15
cta	L	0	0.15
ctg	L	0	0.17
---	-	40344	40344.00
---	--	------	------
mPD		0	7.1×10^{-10}
		nPD:	0.
		N. weight:	0.
		Sc. PD:	0
		Sc. rank:	0

PA-X			
Pos.	260	obs:	exp:
aat	N	0	0.56
aac	N	1	0.44
---	-	40344	40344.00
---	--	------	------
mPD		0	3.0×10^{-10}
		nPD:	0.
		N. weight:	0.
		Sc. PD:	0
		Sc. rank:	0

PA-X			
Pos.	261	obs:	exp:
tta	L	0	0.12
ttg	L	0	0.21
ctt	L	1	0.21
ctc	L	0	0.15
cta	L	0	0.15
ctg	L	0	0.17
---	-	40344	40344.00
---	--	------	------
mPD		0	7.1×10^{-10}
		nPD:	0.
		N. weight:	0.
		Sc. PD:	0
		Sc. rank:	0

PA-X			
Pos.	262	obs:	exp:
ttt	F	1	0.49
ttc	F	0	0.51
---	-	40344	40344.00
---	--	------	------
mPD		0	3.1×10^{-10}
		nPD:	0.
		N. weight:	0.
		Sc. PD:	0
		Sc. rank:	0

PA-X			
Pos.	263	obs:	exp:
taa	.	205	22919.00
tag	.	1	4419.00
tga	.	40139	13007.00
---	--	------	------
mPD		0.010	0.63
		nPD:	0.02
		N. weight:	1.2
		Sc. PD:	-0.032
		Sc. rank:	6636.9

Amino acid level conservation per codon

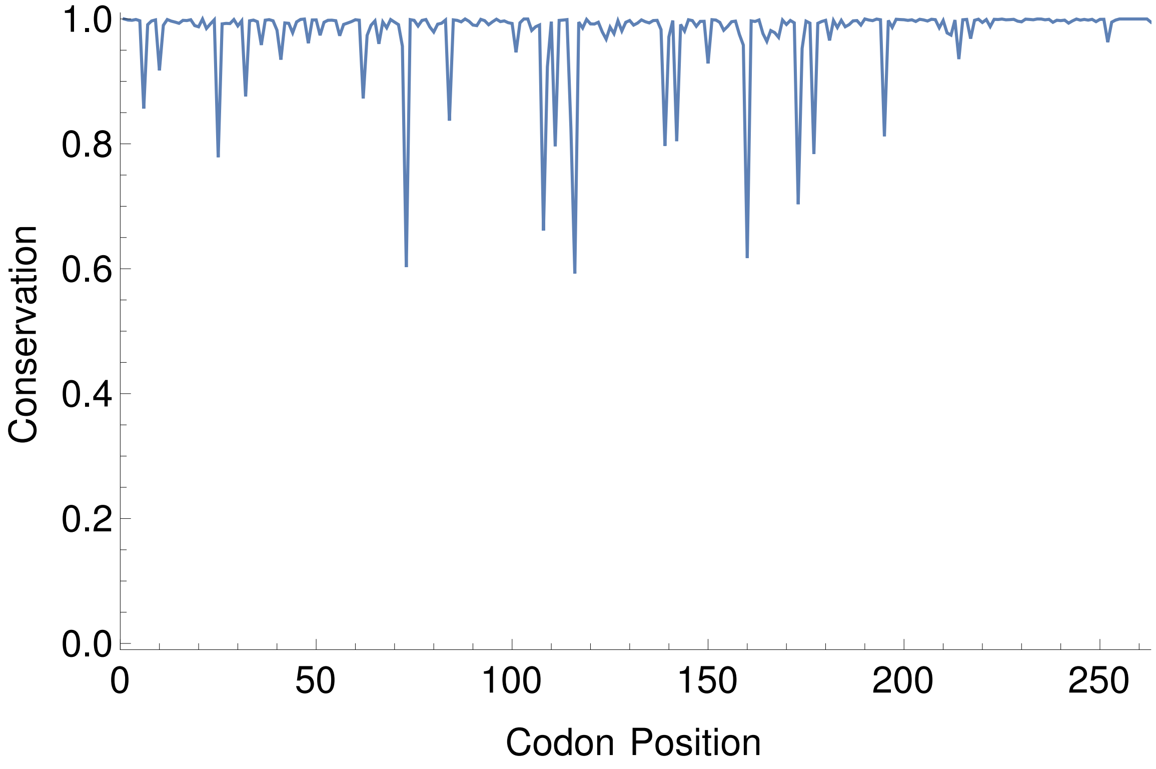

Conservation analysis plots

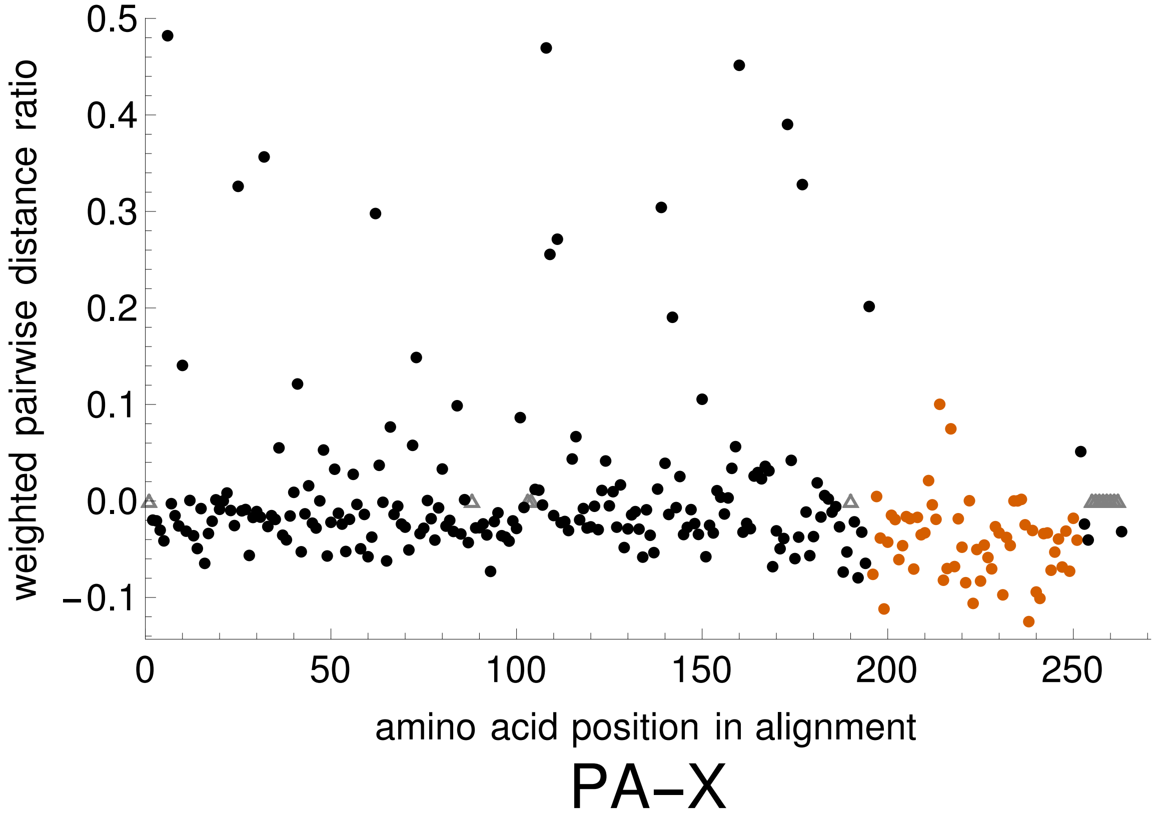

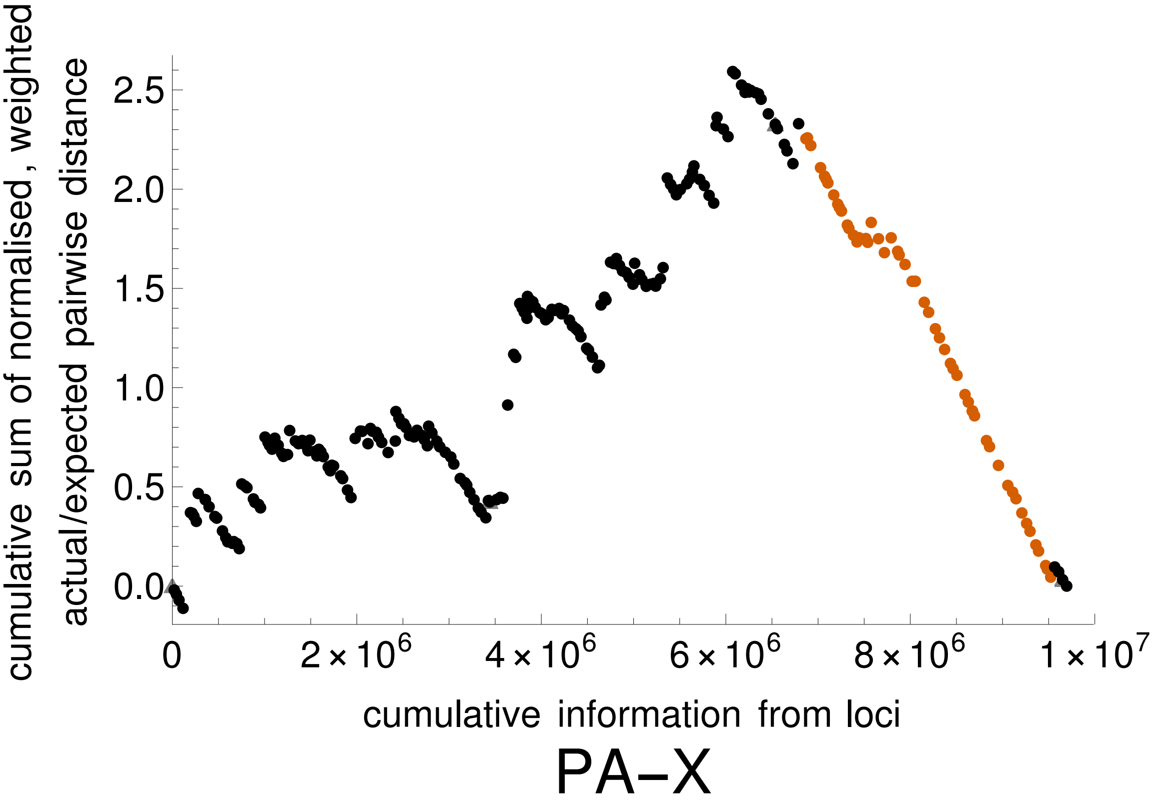

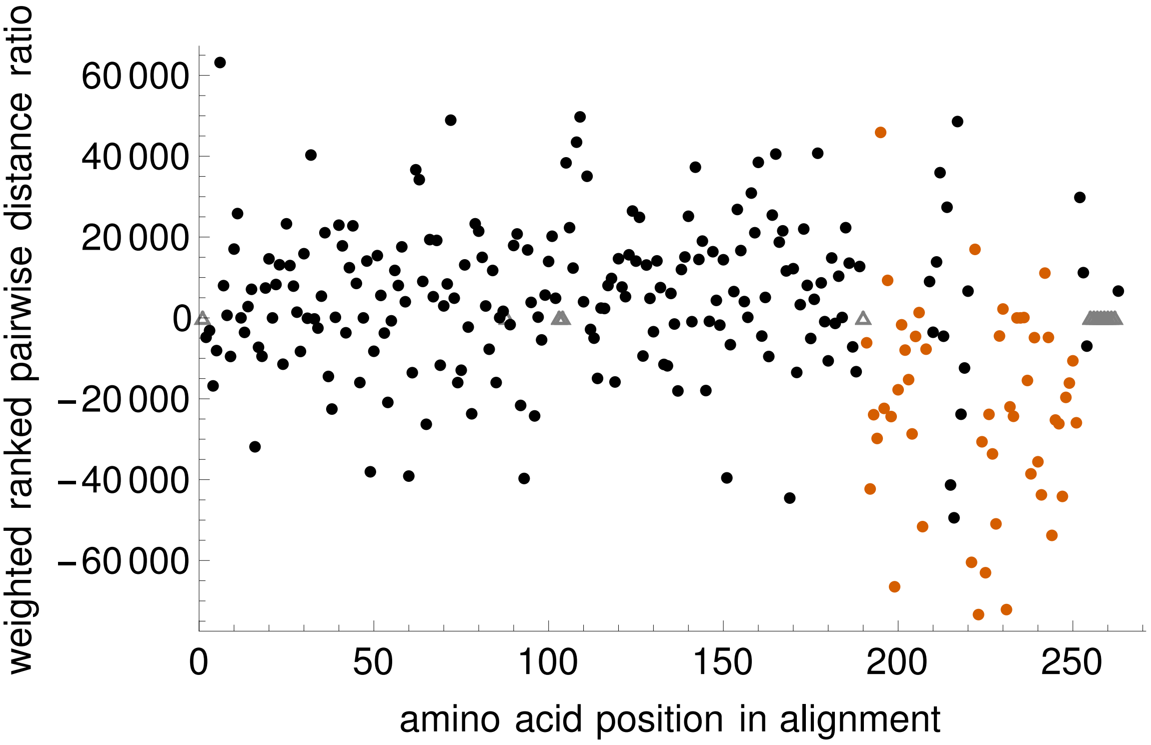
weighted ranked pairwise distance ratio
60 000
40 000
20 000
0
−20 000
−40 000
−60 000
0
50
100
150
200
250
amino acid position in alignment

PA–X

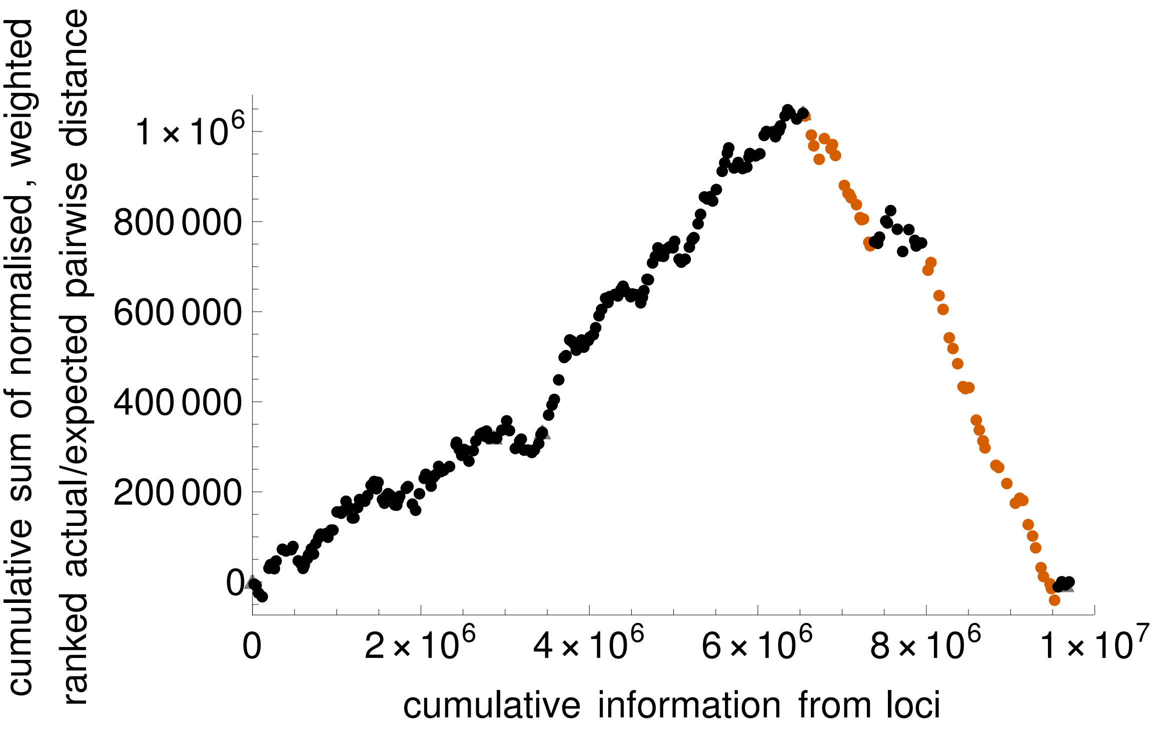
cumulative sum of normalised, weighted ranked actual/expected pairwise distance
1 × 10^6
800 000
600 000
400 000
200 000
0
2 × 10^6
4 × 10^6
6 × 10^6
8 × 10^6
1 × 10^7
cumulative information from loci

PA–X

HA

Gene length histogram

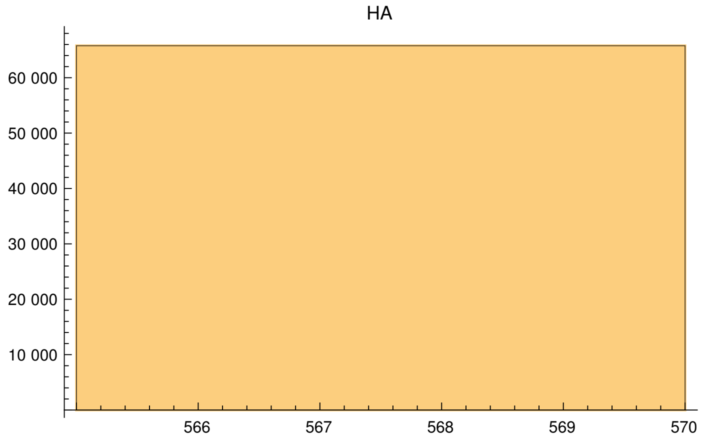

Information vs. nPD

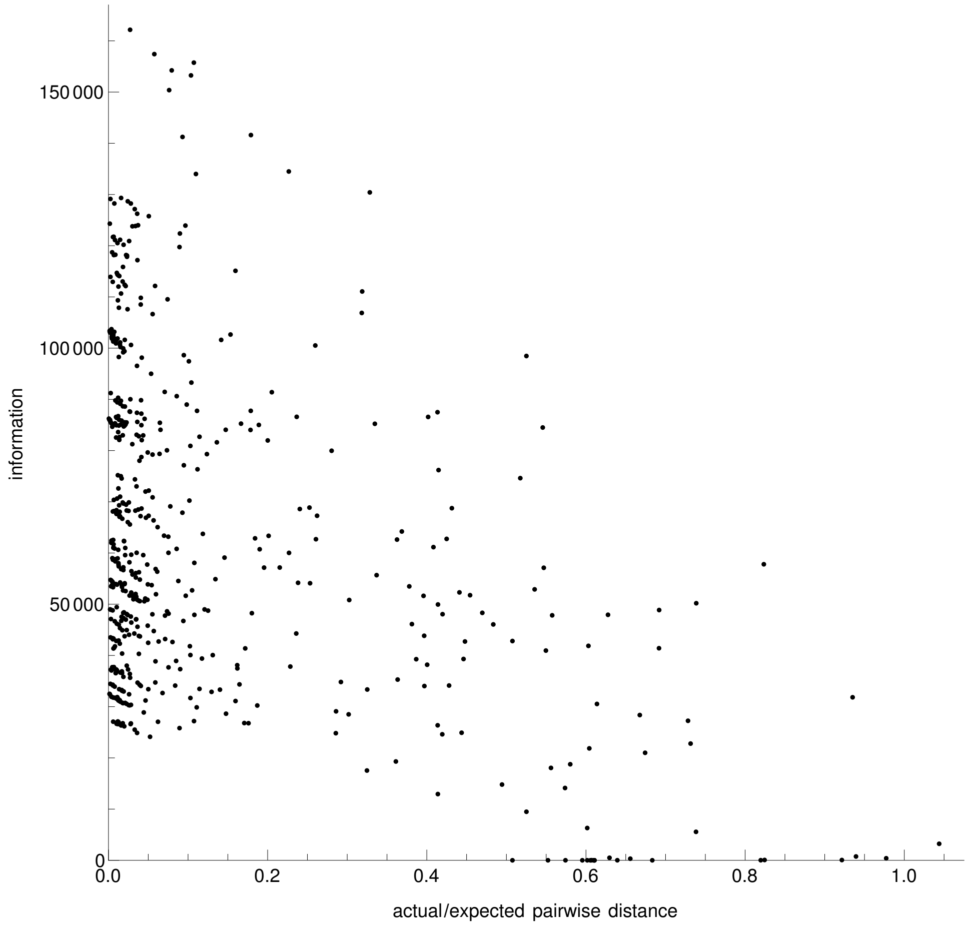

HA

Example sequences highlighted by regions found in analyses to be conserved

```
Interesting points (by weighted raw PD) highlighted for gene HA:
ATGAAGACTATCATTGCTTTGAGCAACATTCTATGTCTTGTTTTCGCT ---CAAAAAATACCTGGAAATGAC ---
  AATAGCACAGCA ---ACGCTGTGCCTTGGGCACCATGCAGTACCAAACGGAACGATAGTGAAAACAATCACAAATG
   ACCGAATTGAAGTTACT ---AATGCTACTGAGTTGGTTCAGAATTCATCAATAGGTGAAATATGCAAC ----------
  --AGTCCTCATCAGATCCTTGATGGAGGGAACTGCACACTAATAGATGCTCTATTGGGGGACCCTCAGTGTGACGG
   CTTTCAAAAT ---AAGGAATGGGACCTTTTTGTTGAACGAAGCAGAGCC ---AACAGCAGCTGTTACCCTTATGAT
   GTGCCGGATTATGCCTCCCTTAGGTCACTAGTTGCCTCATCCGGCACACTGGAG ------------
  TTTAAAAATGAAAGCTTCAATTGGACCGGAGTCAAACAAAAC ---GGAACAAGTTCTGCGTGCATAAGGGGATCTA
   GTAGTAGTTTTTTTAGTAGATTAAATTGGTTGACCAGCTTAAACAACATATATCCAGCACAGAACGTGACTATGC
   CAAACAAGGAACAATTTGACAAATTGTACATTTGGGGGGTTCACCACCCGGATACGGACAAGAACCAATTCTCCC
   TGTTTGCTCAATCATCAGGAAGAATCACAGTATCTACCAAAAGAAGCCAACAAGCTGTAATCCCAAATATCGGAT
   CTAGACCCAGAATAAGGGATATCCCTAGCAGAATAAGCATCTATTGGACAATAGTAAAACCGGGAGACATACTTT
   TGATTAACAGCACAGGGAATCTAATTGCTCCTAGGGGTTACTTCAAAATA ---CGAAGTGGGAAAAGCTCAATAAT
   GAGATCAGACGCACCCATTGGCAAATGTAAGTCTGAATGCATCACTCCAAATGGAAGCATTCCCAATGACAAACC
   GTTCCAAAATGTAAACAGGATCACATACGGGGCCTGTCCCAGATACGTTAAGCAAAGCACCCTGAAATTGGCAAC
   AGGAATGCGAAATGTACCAGAGAAACAAACCAGAGGCATATTTGGCGCAATAGCGGGTTTCATAGAAAATGGATG
   GGAGGGAATGGTGGATGGTTGGTACGGTTTCAGGCATCAAAATTCTGAGGGAAGAGGACAAGCAGCAGATCTCAA
   AAGCACTCAAGCAGCAATCGATCAAATCAGTGGGAAGCTGAATCGATTGATCGGAAAAACCAACGAGAAATTCCA
   TCAGATTGAAAAAGAATTCTCAGAAGTAGAGGGAAGAGTTCAAGACCTTGAGAAATATGTTGAGGACACTAAAAT
   AGATCTCTGGTCATACAACGCGGAGCTTCTTGTTGCCCTGGAGAACCAACATACGATTGACCTAACTGACTCAGA
   AATGAACAAACTGTTTGAAAAAACAAAGAAGCAACTGAGGGAAAATGCTGAAGATATGGGAAATGGTTGTTTCAA
   AATATACCACAAATGTGACAATGCCTGCATAGGATCAATAAGAAATGAAACTTATGACCACAATGTGTACAGGGA
   TGAAGCATTAAACAACCGGTTCCAGATCAAGGGAGTTGAGCTGAAGTCAGGGTACAAAGATTGGATCCTATGGAT
   TTCCTTTGCCATGTCATGTTTTTTGCTTTGTATTGCTTTGTTGGGGTTCATC ---ATGTGG------GCCTGC----
  --CAAAAGGGCAACATTAGATGCAACATT ---------TGCATT---------TGA
```

```
Interesting points (by weighted ranked PD) highlighted for gene HA:
ATGAAGACTATCATTGCTTTGAGCAACATTCTATGTCTTGTTTTCGCT ---CAAAAAATACCTGGAAATGAC ---
  AATAGCACAGCA ---ACGCTGTGCCTTGGGCACCATGCAGTACCAAACGGAACGATAGTGAAAACAATCACAAATG
   ACCGAATTGAAGTTACT ---AATGCTACTGAGTTGGTTCAGAATTCATCAATAGGTGAAATATGCAAC ----------
  --AGTCCTCATCAGATCCTTGATGGAGGGAACTGCACACTAATAGATGCTCTATTGGGGGACCCTCAGTGTGACGG
   CTTTCAAAAT ---AAGGAATGGGACCTTTTTGTTGAACGAAGCAGAGCC ---AACAGCAGCTGTTACCCTTATGAT
   GTGCCGGATTATGCCTCCCTTAGGTCACTAGTTGCCTCATCCGGCACACTGGAG ------------
  TTTAAAAATGAAAGCTTCAATTGGACCGGAGTCAAACAAAAC ---GGAACAAGTTCTGCGTGCATAAGGGGATCTA
   GTAGTAGTTTTTTTAGTAGATTAAATTGGTTGACCAGCTTAAACAACATATATCCAGCACAGAACGTGACTATGC
   CAAACAAGGAACAATTTGACAAATTGTACATTTGGGGGGTTCACCACCCGGATACGGACAAGAACCAATTCTCCC
   TGTTTGCTCAATCATCAGGAAGAATCACAGTATCTACCAAAAGAAGCCAACAAGCTGTAATCCCAAATATCGGAT
   CTAGACCCAGAATAAGGGATATCCCTAGCAGAATAAGCATCTATTGGACAATAGTAAAACCGGGAGACATACTTT
   TGATTAACAGCACAGGGAATCTAATTGCTCCTAGGGGTTACTTCAAAATA ---CGAAGTGGGAAAAGCTCAATAAT
   GAGATCAGACGCACCCATTGGCAAATGTAAGTCTGAATGCATCACTCCAAATGGAAGCATTCCCAATGACAAACC
   GTTCCAAAATGTAAACAGGATCACATACGGGGCCTGTCCCAGATACGTTAAGCAAAGCACCCTGAAATTGGCAAC
   AGGAATGCGAAATGTACCAGAGAAACAAACCAGAGGCATATTTGGCGCAATAGCGGGTTTCATAGAAAATGGATG
   GGAGGGAATGGTGGATGGTTGGTACGGTTTCAGGCATCAAAATTCTGAGGGAAGAGGACAAGCAGCAGATCTCAA
   AAGCACTCAAGCAGCAATCGATCAAATCAGTGGGAAGCTGAATCGATTGATCGGAAAAACCAACGAGAAATTCCA
```

TCAGATTGAAAAAGAATTCTCAGAAGTAGAGGGAAGAGTTCAAGACCTTGAGAAATATGTTGAGGACACTAAAAT∵.
AGATCTCTGGTCATACAACGCGGAGCTTCTTGTTGCCCTGGAGAACCAACATACGATTGACCTAACTGACTCAGA∵.
AATGAACAAACTGTTTGAAAAAACAAAGAAGCAACTGAGGGAAAATGCTGAAGATATGGGAAATGGTTGTTTCAA∵.
AATATACCACAAATGTGACAATGCCTGCATAGGATCAATAAGAAATGAAACTTATGACCACAATGTGTACAGGGA∵.
TGAAGCATTAAACAACCGGTTCCAGATCAAGGGAGTTGAGCTGAAGTCAGGGTACAAAGATTGGATC
CTATGGATTTCCTTTGCCATGTCATGTTTTTTGCTTTGTATTGCTTTGTTGGGGTTCATC --
–ATGTGG------GCCTGC------CAAAAGGGCAACATTAGATGCAACATT ---------TGCATT---------TGA

Per codon conservation report

HA

Pos.	1	obs:	exp:
atg	M	65774	65774.00
---	--	------	------
mPD		0	0
		nPD:	1.
		N. weight:	0.
		Sc. PD:	0
		Sc. rank:	0

HA

Pos.	2	obs:	exp:
caa	Q	0	2.45
cag	Q	4	1.55
cgt	R	0	1.33
cgc	R	0	2.41
cga	R	0	6.11
cgg	R	0	5.75
atg	M	2	2.00
aat	N	102	57.63
aac	N	1	45.37
aaa	K	92	43117.00
aag	K	65492	22467.00
aga	R	1	32.86
agg	R	66	18.53
gaa	E	0	8.25
gag	E	14	5.75
---	--	------	------
mPD		0.0086	0.46
		nPD:	0.02
		N. weight:	1.2
		Sc. PD:	-0.072
		Sc. rank:	-11472.0

HA

Pos.	3	obs:	exp:
tct	S	2	0.48
tcc	S	0	0.45
tca	S	0	0.81
tcg	S	0	0.12
cct	P	2	0.67
ccc	P	0	0.37
cca	P	0	0.68
ccg	P	0	0.28
att	I	75	26.33
atc	I	0	19.71
ata	I	0	28.96
atg	M	1	1.00
act	T	64625	20354.00
acc	T	821	11588.00
aca	T	80	28365.00
acg	T	15	5235.00
aat	N	35	20.14
aac	N	1	15.86
agt	S	1	0.52
agc	S	0	0.62
gct	A	114	31.62
gcc	A	0	28.98
gca	A	2	46.92
gcg	A	0	8.48
---	--	------	------
mPD		0.035	0.69
		nPD:	0.05
		N. weight:	1.2
		Sc. PD:	-0.038
		Sc. rank:	20542.0

HA

Pos.	4	obs:	exp:
ttt	F	0	3.89
ttc	F	8	4.11
tta	L	0	0.35
ttg	L	0	0.62
ctt	L	0	0.63
ctc	L	3	0.44
cta	L	0	0.46
ctg	L	0	0.50
att	I	324	22933.00
atc	I	64971	17166.00
ata	I	33	25230.00
atg	M	1	1.00
act	T	1	68.94
acc	T	221	39.25
aca	T	0	96.08
acg	T	0	17.73
aat	N	0	4.48
aac	N	8	3.52
gtt	V	1	51.10
gtc	V	203	41.57
gta	V	0	44.22
gtg	V	0	67.12
---	--	------	------
mPD		0.024	0.67
		nPD:	0.04
		N. weight:	1.4
		Sc. PD:	-0.064
		Sc. rank:	12342.0

HA

Pos.	5	obs:	exp:
tta	L	0	0.46
ttg	L	0	0.83
tct	S	0	0.16
tcc	S	1	0.15
tca	S	0	0.27
tcg	S	0	0.04
ctt	L	2	0.84
ctc	L	0	0.59
cta	L	2	0.61
ctg	L	0	0.67
cat	H	1	0.56
cac	H	0	0.44
att	I	65562	23070.00
atc	I	43	17269.00
ata	I	115	25381.00
atg	M	3	3.00
act	T	24	7.45
acc	T	0	4.24
aca	T	0	10.39
acg	T	0	1.92
agt	S	0	0.17
agc	S	0	0.21
gtt	V	21	5.26
gtc	V	0	4.28
gta	V	0	4.55
gtg	V	0	6.91
---	--	------	------
mPD		0.0066	0.66
		nPD:	0.01
		N. weight:	1.1
		Sc. PD:	-0.079
		Sc. rank:	-33733.0

HA

Pos.	6	obs:	exp:
tgt	C	0	0.39
tgc	C	1	0.61
cct	P	2	0.67
ccc	P	0	0.37
cca	P	0	0.68
ccg	P	0	0.28
act	T	3	0.93
acc	T	0	0.53
aca	T	0	1.30
acg	T	0	0.24
gtt	V	1	0.75
gtc	V	0	0.61
gta	V	2	0.65
gtg	V	0	0.99
gct	A	64636	17924.00
gcc	A	1063	16430.00
gca	A	41	26601.00
gcg	A	22	4807.00
ggt	G	0	0.40
ggc	G	2	0.42
gga	G	1	1.38
ggg	G	0	0.79
---	--	------	------
mPD		0.034	0.69
		nPD:	0.05
		N. weight:	1.3
		Sc. PD:	-0.043
		Sc. rank:	21611.0

HA

Pos.	7	obs:	exp:
ttt	F	161	78.80
ttc	F	1	83.20
tta	L	145	7609.00
ttg	L	65338	13557.00
tct	S	0	0.97
tcc	S	1	0.90
tca	S	0	1.61
tcg	S	5	0.24
tgg	W	14	14.00
ctt	L	0	13718.00
ctc	L	0	9616.00
cta	L	1	10069.00
ctg	L	91	11005.00
atg	M	4	4.00
agt	S	0	1.05
agc	S	0	1.24
gtt	V	0	2.76
gtc	V	0	2.24
gta	V	1	2.38
gtg	V	10	3.62
---	-	2	2.00
---	--	------	------
mPD		0.013	1.2
		nPD:	0.01
		N. weight:	1.7
		Sc. PD:	-0.12
		Sc. rank:	-44981.0

HA

Pos.	8	obs:	exp:
tct	S	0	10583.00
tcc	S	0	9801.00
tca	S	0	17628.00
tcg	S	0	2582.00
tgt	C	0	9.38
tgc	C	24	14.62
cgt	R	0	0.10
cgc	R	0	0.18
cga	R	0	0.46
cgg	R	0	0.43
att	I	0	1.76
atc	I	5	1.31
ata	I	0	1.93
aat	N	0	5.04
aac	N	9	3.96
agt	S	14	11461.00
agc	S	65572	13530.00
aga	R	1	2.45
agg	R	4	1.38
gat	D	0	0.57
gac	D	1	0.43
gaa	E	1	0.59
gag	E	0	0.41
ggt	G	0	18.98
ggc	G	141	19.68
gga	G	0	65.09
ggg	G	0	37.25
---	-	2	2.00
---	--	------	------
mPD		0.0062	1.6
		nPD:	0.
		N. weight:	1.7
		Sc. PD:	-0.13
		Sc. rank:	-78887.0

HA

Pos.	9	obs:	exp:
ttt	F	0	2.43
ttc	F	5	2.57
tta	L	1	0.12
ttg	L	0	0.21
tct	S	0	18.56
tcc	S	3	17.19
tca	S	0	30.91
tcg	S	1	4.53
tat	Y	10	25939.00
tac	Y	49200	23271.00
tgt	C	7	2420.00
tgc	C	6186	3773.00
ctt	L	0	0.21
ctc	L	0	0.15
cta	L	0	0.15
ctg	L	0	0.17
cat	H	0	148.30
cac	H	263	114.70
caa	Q	1	0.61
cag	Q	0	0.39
att	I	0	0.35
atc	I	1	0.26
ata	I	0	0.39
aat	N	4	5583.00
aac	N	9975	4396.00
aaa	K	1	1.31
aag	K	1	0.69
agt	S	0	20.10
agc	S	111	23.72
gat	D	0	1.13
gac	D	2	0.87
---	-	2	2.00
---	--	------	------
mPD		0.44	0.94
		nPD:	0.47
		N. weight:	0.81
		Sc. PD:	0.3
		Sc. rank:	39123.0

HA

Pos.	10	obs:	exp:
ttt	F	168	81.72
ttc	F	0	86.28
tta	L	0	1.39
ttg	L	1	2.48
tct	S	0	1.13
tcc	S	0	1.05
tca	S	0	1.88
tcg	S	0	0.28
tat	Y	1	0.53
tac	Y	0	0.47
ctt	L	11	2.51
ctc	L	0	1.76
cta	L	0	1.84
ctg	L	0	2.01
cat	H	1	0.56
cac	H	0	0.44
att	I	65272	22938.00
atc	I	70	17170.00
ata	I	1	25235.00
atg	M	10	10.00
act	T	132	41.30
acc	T	1	23.51
aca	T	0	57.56
acg	T	0	10.62
agt	S	7	1.22
agc	S	0	1.44
gtt	V	98	24.80
gtc	V	0	20.17
gta	V	0	21.46
gtg	V	1	32.57
---	--	------	------
mPD		0.015	0.67
		nPD:	0.02
		N. weight:	1.1
		Sc. PD:	-0.066
		Sc. rank:	-4621.9

HA			
Pos.	11	obs:	exp:
ttt	F	0	54.48
ttc	F	112	57.52
tta	L	376	7614.00
ttg	L	0	13566.00
tct	S	1	0.65
tcc	S	1	0.60
tca	S	2	1.08
tcg	S	0	0.16
ctt	L	14	13727.00
ctc	L	14	9622.00
cta	L	65165	10076.00
ctg	L	50	11013.00
cct	P	0	3.33
ccc	P	0	1.87
cca	P	10	3.38
ccg	P	0	1.41
caa	Q	8	4.89
cag	Q	0	3.11
cgt	R	0	0.04
cgc	R	0	0.07
cga	R	2	0.18
cgg	R	0	0.17
att	I	0	5.97
atc	I	0	4.47
ata	I	17	6.57
act	T	0	0.31
acc	T	0	0.18
aca	T	0	0.43
acg	T	1	0.08
agt	S	0	0.70
agc	S	0	0.83
aga	R	0	0.98
agg	R	0	0.55
gtt	V	0	0.25
gtc	V	0	0.20
gta	V	1	0.22
gtg	V	0	0.33
---	--	------	------
mPD		0.022	1.2
		nPD:	0.02
		N. weight :	2.
		Sc. PD:	-0.12
		Sc. rank :	-19086.0

,

HA			
Pos.	12	obs:	exp:
ttt	F	5	2.43
ttc	F	0	2.57
tta	L	0	0.23
ttg	L	0	0.41
tct	S	1	4.03
tcc	S	0	3.74
tca	S	0	6.72
tcg	S	0	0.98
tat	Y	137	73.80
tac	Y	3	66.20
tgt	C	65082	25582.00
tgc	C	381	39881.00
tgg	W	1	1.00
ctt	L	0	0.42
ctc	L	0	0.29
cta	L	2	0.31
ctg	L	0	0.34
cat	H	1	0.56
cac	H	0	0.44
cgt	R	105	2.12
cgc	R	1	3.85
cga	R	0	9.76
cgg	R	0	9.19
atg	M	1	1.00
agt	S	24	4.37
agc	S	0	5.16
aga	R	1	52.48
agg	R	0	29.60
ggt	G	29	3.90
ggc	G	0	4.05
gga	G	0	13.39
ggg	G	0	7.66
---	--	------	------
mPD		0.021	0.49
		nPD:	0.04
		N. weight :	1.
		Sc. PD:	-0.038
		Sc. rank :	14361.0

,

HA			
Pos.	13	obs:	exp:
ttt	F	8	3.89
ttc	F	0	4.11
tta	L	1	7544.00
ttg	L	149	13441.00
tct	S	1	0.16
tcc	S	0	0.15
tca	S	0	0.27
tcg	S	0	0.04
tat	Y	2	1.05
tac	Y	0	0.95
ctt	L	11642	13600.00
ctc	L	4	9533.00
cta	L	350	9983.00
ctg	L	52866	10911.00
cct	P	3	7.33
ccc	P	0	4.12
cca	P	0	7.43
ccg	P	19	3.11
caa	Q	1	50.74
cag	Q	82	32.26
cgt	R	0	0.57
cgc	R	0	1.04
cga	R	0	2.65
cgg	R	29	2.49
att	I	26	9.13
atc	I	0	6.83
ata	I	0	10.04
atg	M	585	585.00
aaa	K	0	0.66
aag	K	1	0.34
agt	S	0	0.17
agc	S	0	0.21
aga	R	0	14.22
agg	R	0	8.02
gtt	V	0	1.25
gtc	V	0	1.02
gta	V	0	1.08
gtg	V	5	1.65
---	--	------	------
mPD		0.33	1.2
		nPD:	0.28
		N. weight :	1.3
		Sc. PD:	0.25
		Sc. rank :	56545.0

,

HA			
Pos.	14	obs:	exp:
ttt	F	4	1.95
ttc	F	0	2.05
tgt	C	1	0.39
tgc	C	0	0.61
att	I	553	194.80
atc	I	2	145.80
ata	I	0	214.30
act	T	1	0.93
acc	T	0	0.53
aca	T	2	1.30
acg	T	0	0.24
gtt	V	64758	16254.00
gtc	V	106	13222.00
gta	V	6	14065.00
gtg	V	22	21351.00
gct	A	189	55.87
gcc	A	16	51.22
gca	A	0	82.92
gcg	A	0	14.99
gat	D	1	0.57
gac	D	0	0.43
ggt	G	113	15.21
ggc	G	0	15.77
gga	G	0	52.16
ggg	G	0	29.85
---	--	------	------
mPD		0.031	0.77
		nPD:	0.04
		N. weight :	1.5
		Sc. PD:	-0.061
		Sc. rank :	18822.0

,

HA			
Pos.	15	obs:	exp:
ttt	F	293	31727.00
ttc	F	64931	33497.00
tta	L	24	14.85
ttg	L	2	26.46
tct	S	17	57.77
tcc	S	341	53.50
tca	S	0	96.22
tcg	S	0	14.09
tat	Y	0	13.18
tac	Y	25	11.82
tgt	C	0	1.56
tgc	C	4	2.44
ctt	L	0	26.78
ctc	L	102	18.77
cta	L	0	19.66
ctg	L	0	21.48
att	I	0	6.67
atc	I	19	4.99
ata	I	0	7.34
agt	S	0	62.56
agc	S	0	73.85
gtt	V	1	4.01
gtc	V	15	3.26
gta	V	0	3.47
gtg	V	0	5.26
---	--	------	------
mPD		0.026	0.52
		nPD:	0.05
		N. weight :	0.71
		Sc. PD:	-0.023
		Sc. rank :	11726.0

,

HA			
Pos.	16	obs:	exp:
ttt	F	1	0.49
ttc	F	0	0.51
tta	L	0	0.12
ttg	L	0	0.21
tct	S	85	14.04
tcc	S	1	13.00
tca	S	0	23.38
tcg	S	0	3.42
ctt	L	1	0.21
ctc	L	0	0.15
cta	L	0	0.15
ctg	L	0	0.17
cct	P	4	1.33
ccc	P	0	0.75
cca	P	0	1.35
ccg	P	0	0.57
cgt	R	1	0.06
cgc	R	1	0.11
cga	R	1	0.27
cgg	R	0	0.26
act	T	2370	739.40
acc	T	9	421.00
aca	T	1	1030.00
acg	T	1	190.20
agt	S	0	15.20
agc	S	1	17.95
aga	R	0	1.47
agg	R	0	0.83
gtt	V	1	0.25
gtc	V	0	0.20
gta	V	0	0.22
gtg	V	0	0.33
gct	A	62859	17238.00
gcc	A	333	15801.00
gca	A	47	25583.00
gcg	A	6	4623.00
ggt	G	0	6.87
ggc	G	51	7.12
gga	G	0	23.54
ggg	G	0	13.47
---	--	------	------
mPD		0.088	0.77
		nPD:	0.11
		N. weight :	1.4
		Sc. PD:	0.041
		Sc. rank :	45494.0

,

HA			
Pos.	17	obs:	exp:
ttt	F	0	0.49
ttc	F	1	0.51
tta	L	0	0.23
ttg	L	0	0.41
tat	Y	0	0.53
tac	Y	1	0.47
tgt	C	0	0.39
tgc	C	1	0.61
ctt	L	0	0.42
ctc	L	2	0.29
cta	L	0	0.31
ctg	L	0	0.34
cat	H	4	2.82
cac	H	1	2.18
---	-	65764	65764.00
---	--	------	------
mPD		3.3×10^{-8}	3.7×10^{-8}
		nPD:	0.89
		N. weight :	0.
		Sc. PD:	0
		Sc. rank :	0

,

HA			
Pos.	18	obs:	exp:
tta	L	0	0.35
ttg	L	0	0.62
tct	S	0	0.16
tcc	S	0	0.15
tca	S	1	0.27
tcg	S	0	0.04
ctt	L	0	0.63
ctc	L	0	0.44
cta	L	2	0.46
ctg	L	1	0.50
cct	P	0	0.33
ccc	P	1	0.19
cca	P	0	0.34
ccg	P	0	0.14
cat	H	1	1.69
cac	H	2	1.31
caa	Q	65581	40168.00
cag	Q	130	25543.00
cgt	R	0	0.14
cgc	R	0	0.25
cga	R	7	0.64
cgg	R	0	0.60
aaa	K	44	28.93
aag	K	0	15.07
agt	S	0	0.17
agc	S	0	0.21
aga	R	0	3.43
agg	R	0	1.94
gaa	E	1	0.59
gag	E	0	0.41
---	-	3	3.00
---	--	------	------
mPD		0.0059	0.48
		nPD:	0.01
		N. weight :	0.53
		Sc. PD:	-0.036
		Sc. rank :	-12868.0

,

HA			
Pos.	19	obs:	exp:
caa	Q	52	32.40
cag	Q	1	20.60
cgt	R	0	2.60
cgc	R	0	4.71
cga	R	0	11.95
cgg	R	0	11.25
act	T	1	0.93
acc	T	0	0.53
aca	T	2	1.30
acg	T	0	0.24
aat	N	34	30.21
aac	N	20	23.79
aaa	K	64417	42566.00
aag	K	329	22180.00
aga	R	127	64.25
agg	R	4	36.23
gat	D	5	37.31
gac	D	61	28.69
gaa	E	714	422.70
gag	E	3	294.30
ggt	G	0	0.27
ggc	G	0	0.28
gga	G	2	0.92
ggg	G	0	0.53
---	-	2	2.00
---	--	------	------
mPD		0.043	0.48
		nPD:	0.09
		N. weight :	0.43
		Sc. PD:	0.0025
		Sc. rank :	11357.0

,

HA			
Pos.	20	obs:	exp:
ttt	F	138	67.13
ttc	F	0	70.87
tta	L	14	1490.00
ttg	L	0	2655.00
tct	S	0	1.45
tcc	S	0	1.34
tca	S	0	2.42
tcg	S	0	0.35
ctt	L	12680	2686.00
ctc	L	140	1883.00
cta	L	2	1972.00
ctg	L	4	2155.00
cct	P	6	2.00
ccc	P	0	1.12
cca	P	0	2.03
ccg	P	0	0.85
cat	H	2	1.13
cac	H	0	0.87
att	I	40865	18483.00
atc	I	1288	13835.00
ata	I	10498	20334.00
atg	M	1	1.00
act	T	31	15.84
acc	T	1	9.02
aca	T	19	22.07
acg	T	0	4.07
aat	N	11	6.15
aac	N	0	4.85
agt	S	9	1.57
agc	S	0	1.86
gtt	V	56	15.78
gtc	V	0	12.84
gta	V	7	13.65
gtg	V	0	20.73
---	-	2	2.00
---	--	------	------
mPD		0.63	1.0
		nPD:	0.6
		N. weight :	0.7
		Sc. PD:	0.35
		Sc. rank :	35273.0

,

HA			
Pos.	21	obs:	exp:
tta	L	0	2.09
ttg	L	0	3.72
tct	S	54	8.88
tcc	S	1	8.22
tca	S	0	14.78
tcg	S	0	2.17
ctt	L	18	3.77
ctc	L	0	2.64
cta	L	0	2.76
ctg	L	0	3.02
cct	P	64406	21879.00
ccc	P	1136	12309.00
cca	P	96	22182.00
ccg	P	17	9286.00
cat	H	18	10.15
cac	H	0	7.85
caa	Q	0	1.22
cag	Q	2	0.78
act	T	22	7.45
acc	T	0	4.24
aca	T	1	10.39
acg	T	1	1.92
agt	S	0	9.61
agc	S	0	11.35
---	-	2	2.00
---	--	------	------
mPD		0.041	0.72
		nPD:	0.06
		N. weight:	1.1
		Sc. PD:	-0.028
		Sc. rank:	21064.0

,

HA			
Pos.	22	obs:	exp:
cgt	R	0	3.97
cgc	R	0	7.20
cga	R	0	18.25
cgg	R	0	17.17
aga	R	200	98.10
agg	R	0	55.32
gtt	V	0	8.77
gtc	V	0	7.13
gta	V	34	7.59
gtg	V	1	11.52
gct	A	0	1.91
gcc	A	0	1.75
gca	A	7	2.83
gcg	A	0	0.51
gaa	E	564	334.30
gag	E	3	232.70
ggt	G	58	8746.00
ggc	G	1	9067.00
gga	G	64492	29988.00
ggg	G	412	17163.00
---	-	2	2.00
---	--	------	------
mPD		0.039	0.70
		nPD:	0.06
		N. weight:	0.81
		Sc. PD:	-0.022
		Sc. rank:	14485.0

,

HA			
Pos.	23	obs:	exp:
tct	S	0	28.08
tcc	S	0	26.00
tca	S	0	46.77
tcg	S	0	6.85
tat	Y	1	0.53
tac	Y	0	0.47
cat	H	3	1.69
cac	H	0	1.31
att	I	59	20.71
atc	I	0	15.50
ata	I	0	22.79
act	T	6	1.86
acc	T	0	1.06
aca	T	0	2.60
acg	T	0	0.48
aat	N	65388	36613.00
aac	N	54	28829.00
aaa	K	7	10.52
aag	K	9	5.48
agt	S	174	30.41
agc	S	0	35.90
gat	D	71	40.13
gac	D	0	30.87
---	-	2	2.00
---	--	------	------
mPD		0.012	0.51
		nPD:	0.02
		N. weight:	0.64
		Sc. PD:	-0.037
		Sc. rank:	-2327.8

,

HA			
Pos.	24	obs:	exp:
tat	Y	0	6.85
tac	Y	13	6.15
cat	H	0	1.69
cac	H	3	1.31
aat	N	2	48.11
aac	N	84	37.89
gat	D	69	37083.00
gac	D	65534	28520.00
gaa	E	27	18.28
gag	E	4	12.72
ggt	G	0	4.85
ggc	G	36	5.02
gga	G	0	16.62
ggg	G	0	9.51
---	-	2	2.00
---	--	------	------
mPD		0.0073	0.50
		nPD:	0.01
		N. weight:	0.91
		Sc. PD:	-0.06
		Sc. rank:	-16219.0

,

HA			
Pos.	25	obs:	exp:
aat	N	0	1.68
aac	N	3	1.32
---	-	65771	65771.00
---	--	------	------
mPD		0	1.0×10^{-9}
		nPD:	0.
		N. weight:	0.
		Sc. PD:	0
		Sc. rank:	0

,

HA			
Pos.	26	obs:	exp:
ttt	F	0	0.49
ttc	F	1	0.51
tct	S	0	2.42
tcc	S	0	2.24
tca	S	0	4.03
tcg	S	0	0.59
tat	Y	4	2.64
tac	Y	1	2.36
cat	H	4	2.82
cac	H	1	2.18
act	T	5	2.17
acc	T	2	1.24
aca	T	0	3.03
acg	T	0	0.56
aat	N	59383	36632.00
aac	N	6093	28844.00
aaa	K	19	28.27
aag	K	24	14.73
agt	S	13	2.62
agc	S	2	3.09
gat	D	61	125.50
gac	D	161	96.51
---	--	------	------
mPD		0.18	0.50
		nPD:	0.36
		N. weight:	0.32
		Sc. PD:	0.086
		Sc. rank:	14333.0

,

HA			
Pos.	27	obs:	exp:
tct	S	0	10560.00
tcc	S	0	9780.00
tca	S	0	17590.00
tcg	S	0	2576.00
tgt	C	0	0.78
tgc	C	2	1.22
cgt	R	0	0.40
cgc	R	3	0.72
cga	R	0	1.82
cgg	R	0	1.72
att	I	0	4.21
atc	I	12	3.15
ata	I	0	4.63
act	T	0	0.31
acc	T	1	0.18
aca	T	0	0.43
acg	T	0	0.08
aat	N	0	134.80
aac	N	241	106.20
agt	S	57	11436.00
agc	S	65385	13500.00
aga	R	14	9.81
agg	R	3	5.53
gct	A	0	0.55
gcc	A	0	0.50
gca	A	2	0.81
gcg	A	0	0.15
gat	D	0	0.57
gac	D	1	0.43
ggt	G	0	7.14
ggc	G	53	7.40
gga	G	0	24.47
ggg	G	0	14.00
---	--	------	------
mPD		0.012	1.6
		nPD:	0.01
		N. weight:	1.7
		Sc. PD:	-0.13
		Sc. rank:	-60201.0

,

HA			
Pos.	28	obs:	exp:
tct	S	0	0.65
tcc	S	0	0.60
tca	S	0	1.08
tcg	S	4	0.16
cct	P	0	1.00
ccc	P	0	0.56
cca	P	0	1.01
ccg	P	3	0.42
cgt	R	0	0.18
cgc	R	0	0.32
cga	R	0	0.82
cgg	R	0	0.77
atg	M	598	598.00
act	T	98	20214.00
acc	T	0	11508.00
aca	T	1665	28169.00
acg	T	63327	5199.00
aat	N	2	1.12
aac	N	0	0.88
aaa	K	1	17.75
aag	K	26	9.25
agt	S	0	0.70
agc	S	0	0.83
aga	R	0	4.41
agg	R	9	2.49
gct	A	0	11.17
gcc	A	0	10.24
gca	A	0	16.58
gcg	A	41	3.00
---	--	------	------
mPD		0.073	0.70
		nPD:	0.1
		N. weight:	2.6
		Sc. PD:	0.05
		Sc. rank:	77711.0

,

HA			
Pos.	29	obs:	exp:
act	T	0	1.86
acc	T	0	1.06
aca	T	6	2.60
acg	T	0	0.48
gtt	V	0	0.25
gtc	V	0	0.20
gta	V	1	0.22
gtg	V	0	0.33
gct	A	21	17925.00
gcc	A	1	16431.00
gca	A	64888	26603.00
gcg	A	856	4808.00
ggt	G	0	0.13
ggc	G	0	0.14
gga	G	1	0.46
ggg	G	0	0.26
---	--	------	------
mPD		0.027	0.69
		nPD:	0.04
		N. weight:	0.94
		Sc. PD:	-0.04
		Sc. rank:	9937.4

,

HA			
Pos.	30	obs:	exp:
gat	D	0	1.13
gac	D	2	0.87
---	-	65772	65772.00
---	--	------	------
mPD		0	4.5×10^{-10}
		nPD:	0.
		N. weight:	0.
		Sc. PD:	0
		Sc. rank:	0

,

HA

Pos.	31	obs:	exp:
atg	M	104	104.00
act	T	39	20385.00
acc	T	3	11605.00
aca	T	437	28407.00
acg	T	65161	5243.00
aaa	K	0	16.44
aag	K	25	8.56
gtt	V	0	0.25
gtc	V	0	0.20
gta	V	0	0.22
gtg	V	1	0.33
gct	A	0	1.09
gcc	A	0	1.00
gca	A	0	1.62
gcg	A	4	0.29
---	--	------	------
mPD		0.019	0.68
		nPD:	0.03
		N. weight:	2.7
		Sc. PD:	-0.15
		Sc. rank:	2522.3

,

HA

Pos.	32	obs:	exp:
ttt	F	1	0.49
ttc	F	0	0.51
tta	L	3	7632.00
ttg	L	138	13598.00
ctt	L	66	13758.00
ctc	L	1	9645.00
cta	L	215	10099.00
ctg	L	65347	11038.00
att	I	1	0.35
atc	I	0	0.26
ata	I	0	0.39
atg	M	1	1.00
gtt	V	0	0.25
gtc	V	0	0.20
gta	V	0	0.22
gtg	V	1	0.33
---	--	------	------
mPD		0.013	1.2
		nPD:	0.01
		N. weight:	1.9
		Sc. PD:	-0.13
		Sc. rank:	-51745.0

,

HA

Pos.	33	obs:	exp:
tta	L	0	0.12
ttg	L	1	0.21
tgt	C	216	25702.00
tgc	C	65553	40067.00
ctt	L	0	0.21
ctc	L	0	0.15
cta	L	0	0.15
ctg	L	0	0.17
cgt	R	0	0.02
cgc	R	0	0.04
cga	R	0	0.09
cgg	R	1	0.09
aga	R	0	0.49
agg	R	0	0.28
ggt	G	0	0.40
ggc	G	3	0.42
gga	G	0	1.38
ggg	G	0	0.79
---	--	------	------
mPD		0.0068	0.48
		nPD:	0.01
		N. weight:	0.52
		Sc. PD:	-0.035
		Sc. rank:	-10113.0

,

HA

Pos.	34	obs:	exp:
ttt	F	11	5.35
ttc	F	0	5.65
tta	L	0	7624.00
ttg	L	1	13583.00
ctt	L	65065	13744.00
ctc	L	43	9634.00
cta	L	17	10089.00
ctg	L	575	11026.00
cct	P	1	0.33
ccc	P	0	0.19
cca	P	0	0.34
ccg	P	0	0.14
att	I	58	21.06
atc	I	0	15.77
ata	I	2	23.17
gtt	V	1	0.25
gtc	V	0	0.20
gta	V	0	0.22
gtg	V	0	0.33
---	--	------	------
mPD		0.021	1.2
		nPD:	0.02
		N. weight:	1.7
		Sc. PD:	-0.1
		Sc. rank:	-17865.0

,

HA

Pos.	35	obs:	exp:
tgg	W	2	2.00
cgt	R	0	0.02
cgc	R	0	0.04
cga	R	0	0.09
cgg	R	0	0.09
aga	R	0	0.49
agg	R	1	0.28
gtt	V	0	0.50
gtc	V	0	0.41
gta	V	0	0.43
gtg	V	2	0.66
ggt	G	186	8854.00
ggc	G	15	9179.00
gga	G	1257	30360.00
ggg	G	64311	17376.00
---	--	------	------
mPD		0.044	0.68
		nPD:	0.06
		N. weight:	1.3
		Sc. PD:	-0.024
		Sc. rank:	28166.0

,

HA

Pos.	36	obs:	exp:
tat	Y	2	1.05
tac	Y	0	0.95
cct	P	0	0.67
ccc	P	2	0.37
cca	P	0	0.68
ccg	P	0	0.28
cat	H	493	37096.00
cac	H	65275	28672.00
cgt	R	0	0.02
cgc	R	1	0.04
cga	R	0	0.09
cgg	R	0	0.09
aga	R	0	0.49
agg	R	0	0.28
gct	A	0	0.27
gcc	A	1	0.25
gca	A	0	0.40
gcg	A	0	0.07
---	--	------	------
mPD		0.015	0.49
		nPD:	0.03
		N. weight:	0.87
		Sc. PD:	-0.043
		Sc. rank:	3491.5

,

HA

Pos.	37	obs:	exp:
tta	L	0	0.12
ttg	L	0	0.21
tat	Y	24	12.65
tac	Y	0	11.35
ctt	L	1	0.21
ctc	L	0	0.15
cta	L	0	0.15
ctg	L	0	0.17
cct	P	0	0.33
ccc	P	0	0.19
cca	P	1	0.34
ccg	P	0	0.14
cat	H	65702	37083.00
cac	H	43	28662.00
caa	Q	1	1.22
cag	Q	1	0.78
aat	N	1	0.56
aac	N	0	0.44
---	--	------	------
mPD		0.0022	0.49
		nPD:	0.
		N. weight:	0.63
		Sc. PD:	-0.047
		Sc. rank:	-27306.0

,

HA

Pos.	38	obs:	exp:
tct	S	0	2.90
tcc	S	0	2.69
tca	S	18	4.84
tcg	S	0	0.71
act	T	0	1.55
acc	T	0	0.88
aca	T	5	2.16
acg	T	0	0.40
agt	S	0	3.15
agc	S	0	3.71
gtt	V	0	0.25
gtc	V	0	0.20
gta	V	1	0.22
gtg	V	0	0.33
gct	A	5	17920.00
gcc	A	1	16427.00
gca	A	65662	26596.00
gcg	A	82	4806.00
---	--	------	------
mPD		0.0034	0.70
		nPD:	0.
		N. weight:	0.99
		Sc. PD:	-0.074
		Sc. rank:	-41891.0

,

HA

Pos.	39	obs:	exp:
tta	L	1	0.12
ttg	L	0	0.21
ctt	L	0	0.21
ctc	L	0	0.15
cta	L	0	0.15
ctg	L	0	0.17
att	I	0	8.07
atc	I	0	6.04
ata	I	23	8.88
act	T	0	0.31
acc	T	0	0.18
aca	T	1	0.43
acg	T	0	0.08
aat	N	0	1.12
aac	N	2	0.88
gtt	V	5	16466.00
gtc	V	9	13395.00
gta	V	63290	14249.00
gtg	V	2436	21630.00
gct	A	0	0.55
gcc	A	0	0.50
gca	A	2	0.81
gcg	A	0	0.15
ggt	G	0	0.67
ggc	G	0	0.70
gga	G	5	2.31
ggg	G	0	1.32
---	--	------	------
mPD		0.073	0.74
		nPD:	0.1
		N. weight:	1.5
		Sc. PD:	0.021
		Sc. rank:	42877.0

,

HA

Pos.	40	obs:	exp:
tta	L	0	1.16
ttg	L	0	2.07
tct	S	0	66.48
tcc	S	0	61.57
tca	S	412	110.70
tcg	S	0	16.22
ctt	L	0	2.09
ctc	L	0	1.47
cta	L	10	1.54
ctg	L	0	1.68
cct	P	3	21766.00
ccc	P	7	12245.00
cca	P	64763	22068.00
ccg	P	545	9238.00
caa	Q	29	17.73
cag	Q	0	11.27
act	T	0	0.93
acc	T	0	0.53
aca	T	3	1.30
acg	T	0	0.24
aat	N	2	1.12
aac	N	0	0.88
agt	S	0	72.00
agc	S	0	84.99
---	--	------	------
mPD		0.031	0.74
		nPD:	0.04
		N. weight:	1.2
		Sc. PD:	-0.046
		Sc. rank:	15467.0

,

HA

Pos.	41	obs:	exp:
tct	S	0	1.61
tcc	S	0	1.49
tca	S	2	2.69
tcg	S	0	0.39
cat	H	0	0.56
cac	H	1	0.44
act	T	0	1.24
acc	T	4	0.71
aca	T	0	1.73
acg	T	0	0.32
aat	N	2891	36786.00
aac	N	62860	28965.00
aaa	K	7	4.60
aag	K	0	2.40
agt	S	0	1.75
agc	S	8	2.06
gat	D	0	0.57
gac	D	1	0.43
---	--	------	------
mPD		0.085	0.49
		nPD:	0.17
		N. weight:	0.69
		Sc. PD:	0.059
		Sc. rank:	25415.0

,

HA

Pos.	42	obs:	exp:
act	T	0	0.62
acc	T	0	0.35
aca	T	2	0.87
acg	T	0	0.16
gtt	V	0	0.25
gtc	V	0	0.20
gta	V	1	0.22
gtg	V	0	0.33
ggt	G	7	8854.00
ggc	G	3	9179.00
gga	G	65544	30361.00
ggg	G	217	17376.00
---	--	------	------
mPD		0.0070	0.68
		nPD:	0.01
		N. weight:	0.83
		Sc. PD:	-0.058
		Sc. rank:	-23840.0

,

HA

Pos.	43	obs:	exp:
tct	S	0	0.32
tcc	S	0	0.30
tca	S	0	0.54
tcg	S	2	0.08
cct	P	0	0.33
ccc	P	0	0.19
cca	P	0	0.34
ccg	P	1	0.14
atg	M	14	14.00
act	T	354	20416.00
acc	T	1	11623.00
aca	T	1425	28451.00
acg	T	63960	5251.00
aat	N	0	0.56
aac	N	1	0.44
aaa	K	0	3.94
aag	K	6	2.06
agt	S	0	0.35
agc	S	0	0.41
gct	A	0	2.45
gcc	A	0	2.25
gca	A	0	3.64
gcg	A	9	0.66
gat	D	0	0.57
gac	D	1	0.43
---	--	------	------
mPD		0.054	0.68
		nPD:	0.08
		N. weight:	2.6
		Sc. PD:	-0.0095
		Sc. rank:	64325.0

,

HA

Pos.	44	obs:	exp:
tta	L	152	68.00
ttg	L	0	121.20
ctt	L	0	122.60
ctc	L	0	85.93
cta	L	434	89.98
ctg	L	0	98.35
cgt	R	0	0.02
cgc	R	0	0.04
cga	R	0	0.09
cgg	R	0	0.09
att	I	4	22463.00
atc	I	1	16814.00
ata	I	63984	24712.00
atg	M	156	156.00
act	T	2	7.45
acc	T	0	4.24
aca	T	22	10.39
acg	T	0	1.92
aga	R	1	0.49
agg	R	0	0.28
gtt	V	0	255.00
gtc	V	0	207.40
gta	V	1014	220.60
gtg	V	4	334.90
---	--	------	------
mPD		0.054	0.71
		nPD:	0.08
		N. weight:	1.1
		Sc. PD:	-0.0082
		Sc. rank:	24744.0

,

HA			
Pos.	45	obs:	exp:
gtt	V	8	16475.00
gtc	V	1	13402.00
gta	V	349	14256.00
gtg	V	65416	21641.00
---	--	------	------
mPD		0.011	0.74
		nPD:	0.01
		N. weight:	1.2
		Sc. PD:	-0.078
		Sc. rank:	-21652.0

,

HA			
Pos.	46	obs:	exp:
caa	Q	4	2.45
cag	Q	0	1.55
cgt	R	0	1.33
cgc	R	0	2.41
cga	R	0	6.11
cgg	R	0	5.75
act	T	0	0.93
acc	T	0	0.53
aca	T	3	1.30
acg	T	0	0.24
aat	N	0	1.12
aac	N	2	0.88
aaa	K	65594	43188.00
aag	K	98	22504.00
aga	R	67	32.86
agg	R	0	18.53
gat	D	0	1.13
gac	D	2	0.87
gaa	E	4	2.36
gag	E	0	1.64
---	--	------	------
mPD		0.0055	0.45
		nPD:	0.01
		N. weight:	0.45
		Sc. PD:	-0.031
		Sc. rank:	-11238.0

,

HA			
Pos.	47	obs:	exp:
cct	P	0	0.33
ccc	P	0	0.19
cca	P	1	0.34
ccg	P	0	0.14
act	T	7	20422.00
acc	T	3	11626.00
aca	T	65694	28459.00
acg	T	56	5252.00
gct	A	0	3.54
gcc	A	0	3.25
gca	A	13	5.26
gcg	A	0	0.95
---	--	------	------
mPD		0.0024	0.68
		nPD:	0.
		N. weight:	0.92
		Sc. PD:	-0.07
		Sc. rank:	-42358.0

,

HA			
Pos.	48	obs:	exp:
ttt	F	0	0.97
ttc	F	2	1.03
tta	L	0	0.81
ttg	L	0	1.45
ctt	L	0	1.46
ctc	L	7	1.03
cta	L	0	1.07
ctg	L	0	1.17
att	I	150	23076.00
atc	I	65486	17273.00
ata	I	100	25387.00
act	T	0	0.93
acc	T	3	0.53
aca	T	0	1.30
acg	T	0	0.24
gtt	V	0	6.51
gtc	V	24	5.30
gta	V	2	5.64
gtg	V	0	8.55
---	--	------	------
mPD		0.0088	0.66
		nPD:	0.01
		N. weight:	1.4
		Sc. PD:	-0.096
		Sc. rank:	-30406.0

,

HA			
Pos.	49	obs:	exp:
tta	L	0	0.23
ttg	L	0	0.41
tct	S	0	0.65
tcc	S	0	0.60
tca	S	3	1.08
tcg	S	1	0.16
ctt	L	0	0.42
ctc	L	0	0.29
cta	L	2	0.31
ctg	L	0	0.34
att	I	0	1.05
atc	I	0	0.79
ata	I	3	1.16
atg	M	15	15.00
act	T	64	20379.00
acc	T	7	11602.00
aca	T	45631	28400.00
acg	T	19920	5241.00
aat	N	0	0.56
aac	N	1	0.44
agt	S	0	0.70
agc	S	0	0.83
gct	A	0	34.61
gcc	A	0	31.73
gca	A	99	51.37
gcg	A	28	9.28
---	--	------	------
mPD		0.43	0.68
		nPD:	0.63
		N. weight:	0.81
		Sc. PD:	0.42
		Sc. rank:	40618.0

,

HA			
Pos.	50	obs:	exp:
tct	S	0	194.60
tcc	S	0	180.20
tca	S	0	324.10
tcg	S	0	47.47
tat	Y	2	1.05
tac	Y	0	0.95
cat	H	9	5.08
cac	H	0	3.92
act	T	10	3.11
acc	T	0	1.77
aca	T	0	4.33
acg	T	0	0.80
aat	N	64170	35953.00
aac	N	92	28309.00
aaa	K	2	1.31
aag	K	0	0.69
agt	S	1206	210.80
agc	S	0	248.80
gat	D	279	158.30
gac	D	1	121.70
gaa	E	2	1.18
gag	E	0	0.82
ggt	G	1	0.13
ggc	G	0	0.14
gga	G	0	0.46
ggg	G	0	0.26
---	--	------	------
mPD		0.048	0.57
		nPD:	0.09
		N. weight:	0.65
		Sc. PD:	0.0011
		Sc. rank:	16458.0

,

HA			
Pos.	51	obs:	exp:
aat	N	0	5.04
aac	N	9	3.96
aaa	K	0	1.31
aag	K	2	0.69
gtt	V	0	0.25
gtc	V	1	0.20
gta	V	0	0.22
gtg	V	0	0.33
gat	D	398	37142.00
gac	D	65309	28565.00
gaa	E	28	17.69
gag	E	2	12.31
ggt	G	0	3.37
ggc	G	25	3.49
gga	G	0	11.54
ggg	G	0	6.60
---	--	------	------
mPD		0.014	0.49
		nPD:	0.03
		N. weight:	0.88
		Sc. PD:	-0.046
		Sc. rank:	2343.2

,

HA			
Pos.	52	obs:	exp:
tta	L	0	0.70
ttg	L	0	1.24
ctt	L	0	1.26
ctc	L	0	0.88
cta	L	6	0.92
ctg	L	0	1.01
cat	H	0	7.33
cac	H	13	5.67
caa	Q	6955	4332.00
cag	Q	131	2754.00
cgt	R	3	1163.00
cgc	R	0	2110.00
cga	R	58413	5351.00
cgg	R	186	5036.00
aat	N	2	6.71
aac	N	10	5.29
aaa	K	5	3.29
aag	K	0	1.71
aga	R	50	28768.00
agg	R	0	16223.00
---	--	------	------
mPD		0.21	1.1
		nPD:	0.18
		N. weight:	2.4
		Sc. PD:	0.22
		Sc. rank:	89331.0

,

HA			
Pos.	53	obs:	exp:
tta	L	0	0.70
ttg	L	0	1.24
tct	S	0	0.16
tcc	S	0	0.15
tca	S	0	0.27
tcg	S	0	0.04
ctt	L	6	1.26
ctc	L	0	0.88
cta	L	0	0.92
ctg	L	0	1.01
att	I	65466	23020.00
atc	I	81	17231.00
ata	I	30	25326.00
atg	M	3	3.00
act	T	24	7.45
acc	T	0	4.24
aca	T	0	10.39
acg	T	0	1.92
aat	N	1	0.56
aac	N	0	0.44
agt	S	1	0.17
agc	S	0	0.21
gtt	V	160	40.58
gtc	V	0	33.01
gta	V	2	35.11
gtg	V	0	53.30
---	--	------	------
mPD		0.0094	0.67
		nPD:	0.01
		N. weight:	1.1
		Sc. PD:	-0.075
		Sc. rank:	-22122.0

,

HA			
Pos.	54	obs:	exp:
act	T	0	0.62
acc	T	0	0.35
aca	T	2	0.87
acg	T	0	0.16
gct	A	0	0.55
gcc	A	0	0.50
gca	A	2	0.81
gcg	A	0	0.15
gaa	E	65508	38774.00
gag	E	259	26993.00
ggt	G	0	0.40
ggc	G	0	0.42
gga	G	3	1.38
ggg	G	0	0.79
---	--	------	------
mPD		0.0081	0.48
		nPD:	0.02
		N. weight:	0.56
		Sc. PD:	-0.035
		Sc. rank:	-7794.6

,

HA			
Pos.	55	obs:	exp:
gtt	V	64755	16475.00
gtc	V	411	13402.00
gta	V	20	14256.00
gtg	V	588	21641.00
---	--	------	------
mPD		0.031	0.74
		nPD:	0.04
		N. weight:	1.4
		Sc. PD:	-0.057
		Sc. rank:	18846.0

,

HA			
Pos.	56	obs:	exp:
att	I	6	2.11
atc	I	0	1.58
ata	I	0	2.32
act	T	64978	20424.00
acc	T	776	11628.00
aca	T	6	28463.00
acg	T	8	5253.00
---	--	------	------
mPD		0.024	0.68
		nPD:	0.04
		N. weight:	1.2
		Sc. PD:	-0.056
		Sc. rank:	8329.5

,

HA			
Pos.	57	obs:	exp:
tta	L	1	0.12
ttg	L	0	0.21
ctt	L	0	0.21
ctc	L	0	0.15
cta	L	0	0.15
ctg	L	0	0.17
---	-	65773	65773.00
---	--	------	------
mPD		0	2.7×10^{-10}
		nPD:	0.
		N. weight:	0.
		Sc. PD:	0
		Sc. rank:	0

,

HA			
Pos.	58	obs:	exp:
tct	S	0	0.65
tcc	S	0	0.60
tca	S	0	1.08
tcg	S	0	0.16
tat	Y	1	0.53
tac	Y	0	0.47
cat	H	0	1.13
cac	H	2	0.87
act	T	2	0.62
acc	T	0	0.35
aca	T	0	0.87
acg	T	0	0.16
aat	N	65672	36792.00
aac	N	90	28970.00
aaa	K	3	1.97
aag	K	0	1.03
agt	S	4	0.70
agc	S	0	0.83
---	--	------	------
mPD		0.0032	0.49
		nPD:	0.01
		N. weight:	0.63
		Sc. PD:	-0.046
		Sc. rank:	-23498.0

,

HA			
Pos.	59	obs:	exp:
tct	S	7	1.13
tcc	S	0	1.05
tca	S	0	1.88
tcg	S	0	0.28
act	T	1	0.31
acc	T	0	0.18
aca	T	0	0.43
acg	T	0	0.08
agt	S	0	1.22
agc	S	0	1.44
gct	A	65495	17924.00
gcc	A	215	16431.00
gca	A	44	26602.00
gcg	A	11	4808.00
ggt	G	1	0.13
ggc	G	0	0.14
gga	G	0	0.46
ggg	G	0	0.26
---	--	------	------
mPD		0.0085	0.69
		nPD:	0.01
		N. weight:	1.4
		Sc. PD:	-0.095
		Sc. rank:	-34987.0

,

HA			
Pos.	60	obs:	exp:
att	I	3	1.05
atc	I	0	0.79
ata	I	0	1.16
act	T	65512	20424.00
acc	T	187	11628.00
aca	T	49	28462.00
acg	T	18	5253.00
aat	N	1	0.56
aac	N	0	0.44
gtt	V	2	0.50
gtc	V	0	0.41
gta	V	0	0.43
gtg	V	0	0.66
gct	A	2	0.55
gcc	A	0	0.50
gca	A	0	0.81
gcg	A	0	0.15
---	--	------	------
mPD		0.0080	0.68
		nPD:	0.01
		N. weight:	1.3
		Sc. PD:	-0.086
		Sc. rank:	-32504.0

,

HA			
Pos.	61	obs:	exp:
aat	N	1	1.12
aac	N	1	0.88
gat	D	2	1.13
gac	D	0	0.87
gaa	E	1976	38776.00
gag	E	63794	26994.00
---	--	------	------
mPD		0.058	0.48
		nPD:	0.12
		N. weight:	0.82
		Sc. PD:	0.029
		Sc. rank:	27315.0

,

HA			
Pos.	62	obs:	exp:
tta	L	97	7631.00
ttg	L	39020	13595.00
tct	S	0	0.32
tcc	S	0	0.30
tca	S	0	0.54
tcg	S	2	0.08
ctt	L	29	13756.00
ctc	L	0	9643.00
cta	L	215	10098.00
ctg	L	26398	11036.00
atg	M	10	10.00
agt	S	0	0.35
agc	S	0	0.41
gtt	V	0	0.75
gtc	V	0	0.61
gta	V	0	0.65
gtg	V	3	0.99
---	--	------	------
mPD		0.49	1.2
		nPD:	0.42
		N. weight:	1.1
		Sc. PD:	0.34
		Sc. rank:	49700.0

,

HA			
Pos.	63	obs:	exp:
tta	L	0	0.23
ttg	L	0	0.41
tct	S	1	0.16
tcc	S	0	0.15
tca	S	0	0.27
tcg	S	0	0.04
ctt	L	0	0.42
ctc	L	0	0.29
cta	L	1	0.31
ctg	L	1	0.34
att	I	57	20.01
atc	I	0	14.98
ata	I	0	22.01
agt	S	0	0.17
agc	S	0	0.21
gtt	V	65122	16460.00
gtc	V	555	13390.00
gta	V	27	14243.00
gtg	V	9	21621.00
gat	D	1	0.57
gac	D	0	0.43
---	--	------	------
mPD		0.020	0.74
		nPD:	0.03
		N. weight:	1.5
		Sc. PD:	-0.079
		Sc. rank:	-146.1

,

HA			
Pos.	64	obs:	exp:
tta	L	0	0.46
ttg	L	0	0.83
ctt	L	0	0.84
ctc	L	0	0.59
cta	L	0	0.61
ctg	L	4	0.67
cat	H	13	7.33
cac	H	0	5.67
caa	Q	744	40191.00
cag	Q	65005	25558.00
cgt	R	0	0.10
cgc	R	0	0.18
cga	R	0	0.46
cgg	R	5	0.43
aga	R	0	2.45
agg	R	0	1.38
gaa	E	2	1.77
gag	E	1	1.23
---	--	------	------
mPD		0.023	0.48
		nPD:	0.05
		N. weight:	0.97
		Sc. PD:	-0.032
		Sc. rank:	15173.0

,

HA			
Pos.	65	obs:	exp:
tct	S	0	878.60
tcc	S	1	813.70
tca	S	0	1464.00
tcg	S	0	214.30
cat	H	3	1.69
cac	H	0	1.31
cgt	R	0	0.02
cgc	R	0	0.04
cga	R	0	0.09
cgg	R	0	0.09
att	I	64	22.47
atc	I	0	16.82
ata	I	0	24.72
act	T	21	6.52
acc	T	0	3.71
aca	T	0	9.09
acg	T	0	1.68
aat	N	54298	33672.00
aac	N	5888	26514.00
aaa	K	4	7.89
aag	K	8	4.11
agt	S	5383	951.50
agc	S	61	1123.00
aga	R	0	0.49
agg	R	1	0.28
gat	D	26	18.09
gac	D	6	13.91
ggt	G	10	1.35
ggc	G	0	1.40
gga	G	0	4.62
ggg	G	0	2.64
---	--	------	------
mPD		0.32	0.78
		nPD:	0.41
		N. weight:	0.44
		Sc. PD:	0.14
		Sc. rank:	20523.0

,

HA			
Pos.	66	obs:	exp:
ttt	F	0	51.56
ttc	F	106	54.44
tct	S	293	10539.00
tcc	S	54434	9760.00
tca	S	10573	17553.00
tcg	S	8	2571.00
tat	Y	0	4.74
tac	Y	9	4.26
cct	P	0	2.00
ccc	P	5	1.12
cca	P	1	2.03
ccg	P	0	0.85
cat	H	0	0.56
cac	H	1	0.44
att	I	0	0.35
atc	I	1	0.26
ata	I	0	0.39
act	T	0	105.90
acc	T	335	60.29
aca	T	6	147.60
acg	T	0	27.24
aaa	K	0	1.31
aag	K	2	0.69
agt	S	0	11413.00
agc	S	0	13473.00
---	--	------	------
mPD		0.29	1.6
		nPD:	0.18
		N. weight:	1.5
		Sc. PD:	0.13
		Sc. rank:	54755.0

,

HA			
Pos.	67	obs:	exp:
tta	L	6	0.70
ttg	L	0	1.24
tct	S	72	10566.00
tcc	S	12	9784.00
tca	S	65333	17598.00
tcg	S	58	2577.00
ctt	L	0	1.26
ctc	L	0	0.88
cta	L	0	0.92
ctg	L	0	1.01
cct	P	0	12.66
ccc	P	0	7.12
cca	P	38	12.84
ccg	P	0	5.37
cat	H	2	1.13
cac	H	0	0.87
act	T	0	74.84
acc	T	0	42.61
aca	T	241	104.30
acg	T	0	19.25
agt	S	0	11442.00
agc	S	0	13507.00
gct	A	0	3.27
gcc	A	0	3.00
gca	A	12	4.85
gcg	A	0	0.88
---	--	------	------
mPD		0.013	1.6
		nPD:	0.01
		N. weight:	1.4
		Sc. PD:	-0.1
		Sc. rank:	-47254.0

,

HA			
Pos.	68	obs:	exp:
tta	L	2	1.51
ttg	L	0	2.69
ctt	L	0	2.72
ctc	L	0	1.91
cta	L	11	2.00
ctg	L	0	2.18
cct	P	0	0.67
ccc	P	0	0.37
cca	P	2	0.68
ccg	P	0	0.28
cgt	R	0	1.71
cgc	R	0	3.09
cga	R	0	7.85
cgg	R	0	7.38
att	I	19	20861.00
atc	I	182	15615.00
ata	I	59224	22950.00
atg	M	266	266.00
act	T	2	1785.00
acc	T	1	1016.00
aca	T	5672	2487.00
acg	T	72	459.00
aat	N	0	1.12
aac	N	2	0.88
aaa	K	23	15.12
aag	K	0	7.88
aga	R	86	42.18
agg	R	0	23.79
gtt	V	0	7.51
gtc	V	1	6.11
gta	V	28	6.50
gtg	V	1	9.87
gct	A	0	49.06
gcc	A	0	44.97
gca	A	179	72.81
gcg	A	1	13.16
---	--	------	------
mPD		0.19	0.84
		nPD:	0.23
		N. weight:	1.
		Sc. PD:	0.14
		Sc. rank:	40317.0

,

HA			
Pos.	69	obs:	exp:
tct	S	0	14.20
tcc	S	0	13.15
tca	S	0	23.65
tcg	S	0	3.46
tgt	C	4	1.56
tgc	C	0	2.44
agt	S	88	15.38
agc	S	0	18.15
gtt	V	4	1.00
gtc	V	0	0.82
gta	V	0	0.87
gtg	V	0	1.32
gat	D	57	32.22
gac	D	0	24.78
ggt	G	65225	8834.00
ggc	G	72	9159.00
gga	G	272	30292.00
ggg	G	52	17336.00
---	--	------	------
mPD		0.017	0.69
		nPD:	0.02
		N. weight:	2.2
		Sc. PD:	-0.12
		Sc. rank:	-4604.7

,

HA			
Pos.	70	obs:	exp:
caa	Q	2	1.22
cag	Q	0	0.78
cgt	R	0	9.22
cgc	R	0	16.73
cga	R	8	42.43
cgg	R	0	39.93
att	I	0	0.35
atc	I	0	0.26
ata	I	1	0.39
aaa	K	1787	1175.00
aag	K	0	612.20
aga	R	454	228.10
agg	R	3	128.60
gtt	V	0	4.26
gtc	V	0	3.46
gta	V	17	3.68
gtg	V	0	5.59
gct	A	0	0.55
gcc	A	0	0.50
gca	A	2	0.81
gcg	A	0	0.15
gat	D	0	2.26
gac	D	4	1.74
gaa	E	63021	37182.00
gag	E	46	25885.00
ggt	G	0	57.75
ggc	G	0	59.87
gga	G	425	198.00
ggg	G	4	113.30
---	--	------	------
mPD		0.095	0.58
		nPD:	0.16
		N. weight:	0.58
		Sc. PD:	0.045
		Sc. rank:	20840.0

,

HA			
Pos.	71	obs:	exp:
tta	L	1	0.81
ttg	L	0	1.45
ctt	L	0	1.46
ctc	L	0	1.03
cta	L	6	1.07
ctg	L	0	1.17
att	I	38	23079.00
atc	I	18	17275.00
ata	I	65688	25390.00
atg	M	17	17.00
gtt	V	0	1.50
gtc	V	0	1.22
gta	V	6	1.30
gtg	V	0	1.97
---	--	------	------
mPD		0.0026	0.66
		nPD:	0.
		N. weight:	1.
		Sc. PD:	-0.079
		Sc. rank:	-46886.0

,

HA			
Pos.	72	obs:	exp:
tct	S	0	0.32
tcc	S	0	0.30
tca	S	0	0.54
tcg	S	0	0.08
tgt	C	354	25703.00
tgc	C	65417	40068.00
tgg	W	1	1.00
agt	S	0	0.35
agc	S	2	0.41
---	--	------	------
mPD		0.011	0.48
		nPD:	0.02
		N. weight:	0.51
		Sc. PD:	-0.03
		Sc. rank:	-2297.9

,

HA			
Pos.	73	obs:	exp:
tct	S	0	18.56
tcc	S	0	17.19
tca	S	0	30.91
tcg	S	0	4.53
tat	Y	1	9.49
tac	Y	17	8.51
cat	H	0	1.13
cac	H	2	0.87
aat	N	42	3919.00
aac	N	6962	3085.00
aaa	K	3	1.97
aag	K	0	1.03
agt	S	2	20.10
agc	S	113	23.72
gtt	V	0	0.25
gtc	V	1	0.20
gta	V	0	0.22
gtg	V	0	0.33
gct	A	1	0.55
gcc	A	1	0.50
gca	A	0	0.81
gcg	A	0	0.15
gat	D	56	29429.00
gac	D	52006	22633.00
gaa	E	226	133.80
gag	E	1	93.17
ggt	G	3	853.50
ggc	G	6336	884.90
gga	G	1	2927.00
ggg	G	0	1675.00
---	--	------	------
mPD		0.38	0.93
		nPD:	0.41
		N. weight:	1.
		Sc. PD:	0.32
		Sc. rank:	47264.0

,

HA			
Pos.	74	obs:	exp:
tta	L	0	0.23
ttg	L	0	0.41
ctt	L	0	0.42
ctc	L	0	0.29
cta	L	2	0.31
ctg	L	0	0.34
---	-	65772	65772.00
---	--	------	------
mPD		0	1.1×10^{-9}
		nPD:	0.
		N. weight:	0.
		Sc. PD:	0
		Sc. rank:	0

,

HA			
Pos.	75	obs:	exp:
cgt	R	0	0.04
cgc	R	0	0.07
cga	R	0	0.18
cgg	R	0	0.17
aga	R	2	0.98
agg	R	0	0.55
---	-	65772	65772.00
---	--	------	------
mPD		0	8.2×10^{-10}
		nPD:	0.
		N. weight:	0.
		Sc. PD:	0
		Sc. rank:	0

,

HA			
Pos.	76	obs:	exp:
ggt	G	0	0.27
ggc	G	0	0.28
gga	G	0	0.92
ggg	G	2	0.53
---	-	65772	65772.00
---	--	------	------
mPD		0	6.3×10^{-10}
		nPD:	0.
		N. weight:	0.
		Sc. PD:	0
		Sc. rank:	0

,

HA			
Pos.	77	obs:	exp:
gtt	V	0	0.50
gtc	V	0	0.41
gta	V	2	0.43
gtg	V	0	0.66
---	-	65772	65772.00
---	--	------	------
mPD		0	6.8×10^{-10}
		nPD:	0.
		N. weight:	0.
		Sc. PD:	0
		Sc. rank:	0

,

HA			
Pos.	78	obs:	exp:
tct	S	0	10570.00
tcc	S	0	9789.00
tca	S	0	17606.00
tcg	S	0	2578.00
cgt	R	23	1.47
cgc	R	0	2.66
cga	R	0	6.75
cgg	R	0	6.35
att	I	7	2.46
atc	I	0	1.84
ata	I	0	2.70
atg	M	1	1.00
aat	N	153	85.60
aac	N	0	67.40
agt	S	65101	11447.00
agc	S	401	13513.00
aga	R	4	36.30
agg	R	47	20.47
gct	A	0	0.55
gcc	A	2	0.50
gca	A	0	0.81
gcg	A	0	0.15
ggt	G	35	4.71
ggc	G	0	4.88
gga	G	0	16.16
ggg	G	0	9.25
---	--	------	------
mPD		0.021	1.6
		nPD:	0.01
		N. weight:	1.9
		Sc. PD:	-0.13
		Sc. rank:	-43617.0

,

HA

Pos.	79	obs:	exp:
tta	L	0	0.23
ttg	L	0	0.41
tct	S	3	0.48
tcc	S	0	0.45
tca	S	0	0.81
tcg	S	0	0.12
ctt	L	2	0.42
ctc	L	0	0.29
cta	L	0	0.31
ctg	L	0	0.34
cct	P	65245	21915.00
ccc	P	495	12329.00
cca	P	19	22218.00
ccg	P	4	9301.00
cat	H	4	2.26
cac	H	0	1.74
act	T	2	0.62
acc	T	0	0.35
aca	T	0	0.87
acg	T	0	0.16
agt	S	0	0.52
agc	S	0	0.62
---	--	------	------
mPD		0.016	0.72
		nPD:	0.02
		N. weight:	1.2
		Sc. PD:	-0.068
		Sc. rank:	-5728.0

,

HA

Pos.	80	obs:	exp:
tta	L	0	0.93
ttg	L	2	1.65
tat	Y	724	383.70
tac	Y	4	344.30
ctt	L	6	1.67
ctc	L	0	1.17
cta	L	0	1.23
ctg	L	0	1.34
cat	H	64348	36678.00
cac	H	679	28349.00
caa	Q	0	1.22
cag	Q	2	0.78
cgt	R	3	0.06
cgc	R	0	0.11
cga	R	0	0.27
cgg	R	0	0.26
aat	N	6	3.36
aac	N	0	2.64
aga	R	0	1.47
agg	R	0	0.83
---	--	------	------
mPD		0.043	0.51
		nPD:	0.08
		N. weight:	0.57
		Sc. PD:	0.00029
		Sc. rank:	14355.0

,

HA

Pos.	81	obs:	exp:
tta	L	0	5.34
ttg	L	0	9.51
ctt	L	0	9.62
ctc	L	0	6.75
cta	L	0	7.06
ctg	L	46	7.72
cat	H	163	93.07
cac	H	2	71.93
caa	Q	750	39748.00
cag	Q	64274	25276.00
cgt	R	0	7.46
cgc	R	0	13.53
cga	R	313	34.31
cgg	R	62	32.29
aaa	K	2	107.20
aag	K	161	55.84
aga	R	0	184.40
agg	R	1	104.00
---	--	------	------
mPD		0.055	0.51
		nPD:	0.11
		N. weight:	0.98
		Sc. PD:	0.023
		Sc. rank:	30550.0

,

HA

Pos.	82	obs:	exp:
tta	L	0	0.23
ttg	L	2	0.41
ctt	L	0	0.42
ctc	L	0	0.29
cta	L	0	0.31
ctg	L	0	0.34
att	I	400	22714.00
atc	I	64288	17002.00
ata	I	18	24989.00
act	T	1	2.48
acc	T	7	1.41
aca	T	0	3.46
acg	T	0	0.64
gtt	V	5	265.00
gtc	V	1053	215.60
gta	V	0	229.30
gtg	V	0	348.10
---	--	------	------
mPD		0.045	0.69
		nPD:	0.06
		N. weight:	1.4
		Sc. PD:	-0.026
		Sc. rank:	30679.0

,

HA

Pos.	83	obs:	exp:
ttt	F	3	1.46
ttc	F	0	1.54
tta	L	0	7622.00
ttg	L	0	13580.00
ctt	L	65506	13741.00
ctc	L	161	9632.00
cta	L	12	10086.00
ctg	L	6	11024.00
att	I	84	29.49
atc	I	0	22.07
ata	I	0	32.44
---	-	2	2.00
---	--	------	------
mPD		0.0081	1.2
		nPD:	0.01
		N. weight:	1.7
		Sc. PD:	-0.12
		Sc. rank:	-60515.0

,

HA

Pos.	84	obs:	exp:
aat	N	1	0.56
aac	N	0	0.44
gat	D	65712	37177.00
gac	D	58	28593.00
gaa	E	1	0.59
gag	E	0	0.41
---	-	2	2.00
---	--	------	------
mPD		0.0018	0.49
		nPD:	0.
		N. weight:	0.62
		Sc. PD:	-0.047
		Sc. rank:	-28502.0

,

HA

Pos.	85	obs:	exp:
gaa	E	0	0.59
gag	E	1	0.41
ggt	G	17	8854.00
ggc	G	13	9179.00
gga	G	65230	30361.00
ggg	G	511	17376.00
---	-	2	2.00
---	--	------	------
mPD		0.016	0.68
		nPD:	0.02
		N. weight:	0.8
		Sc. PD:	-0.045
		Sc. rank:	-2016.0

,

HA

Pos.	86	obs:	exp:
cgt	R	0	1.27
cgc	R	0	2.30
cga	R	0	5.84
cgg	R	0	5.50
att	I	0	25.27
atc	I	0	18.92
ata	I	72	27.81
atg	M	6	6.00
act	T	0	0.62
acc	T	0	0.35
aca	T	2	0.87
acg	T	0	0.16
aat	N	0	0.56
aac	N	1	0.44
aaa	K	2124	1409.00
aag	K	19	734.10
aga	R	0	31.39
agg	R	64	17.70
gtt	V	0	17.03
gtc	V	0	13.86
gta	V	68	14.74
gtg	V	0	22.37
gct	A	0	0.27
gcc	A	0	0.25
gca	A	1	0.40
gcg	A	0	0.07
gat	D	3	3.96
gac	D	4	3.04
gaa	E	33406	24593.00
gag	E	8308	17121.00
ggt	G	3	2921.00
ggc	G	0	3028.00
gga	G	423	10015.00
ggg	G	21270	5732.00
---	--	------	------
mPD		1.0	1.1
		nPD:	0.94
		N. weight:	0.53
		Sc. PD:	0.43
		Sc. rank:	27494.0

,

HA

Pos.	87	obs:	exp:
tct	S	0	2.10
tcc	S	0	1.94
tca	S	0	3.49
tcg	S	0	0.51
aat	N	552	36745.00
aac	N	65127	28934.00
aaa	K	24	16.44
aag	K	1	8.56
agt	S	0	2.27
agc	S	13	2.68
gat	D	0	32.22
gac	D	57	24.78
---	--	------	------
mPD		0.020	0.50
		nPD:	0.04
		N. weight:	0.85
		Sc. PD:	-0.036
		Sc. rank:	9344.3

,

HA

Pos.	88	obs:	exp:
tgt	C	417	25703.00
tgc	C	65355	40069.00
tgg	W	1	1.00
cgt	R	0	0.02
cgc	R	1	0.04
cga	R	0	0.09
cgg	R	0	0.09
aga	R	0	0.49
agg	R	0	0.28
---	--	------	------
mPD		0.013	0.48
		nPD:	0.03
		N. weight:	0.51
		Sc. PD:	-0.027
		Sc. rank:	-180.3

,

HA			
Pos.	89	obs:	exp:
tct	S	0	1.29
tcc	S	0	1.20
tca	S	8	2.15
tcg	S	0	0.31
att	I	0	0.35
atc	I	0	0.26
ata	I	1	0.39
act	T	266	20420.00
acc	T	24	11625.00
aca	T	65365	28456.00
acg	T	98	5252.00
aat	N	0	1.12
aac	N	2	0.88
aaa	K	8	5.26
aag	K	0	2.74
agt	S	0	1.40
agc	S	0	1.65
gct	A	0	0.55
gcc	A	0	0.50
gca	A	2	0.81
gcg	A	0	0.15
---	--	------	------
mPD		0.012	0.68
		nPD:	0.02
		N. weight:	0.88
		Sc. PD:	-0.055
		Sc. rank:	-10004.0

,

HA			
Pos.	90	obs:	exp:
tta	L	212	7629.00
ttg	L	1	13592.00
ctt	L	2	13753.00
ctc	L	46	9641.00
cta	L	64925	10095.00
ctg	L	558	11034.00
cgt	R	0	0.02
cgc	R	0	0.04
cga	R	1	0.09
cgg	R	0	0.09
att	I	2	9.83
atc	I	0	7.36
ata	I	26	10.81
aga	R	0	0.49
agg	R	0	0.28
gtt	V	0	0.25
gtc	V	0	0.20
gta	V	1	0.22
gtg	V	0	0.33
---	--	------	------
mPD		0.026	1.2
		nPD:	0.02
		N. weight:	2.
		Sc. PD:	-0.12
		Sc. rank:	-9063.4

,

HA			
Pos.	91	obs:	exp:
tta	L	1	0.35
ttg	L	0	0.62
ctt	L	0	0.63
ctc	L	0	0.44
cta	L	2	0.46
ctg	L	0	0.50
att	I	482	23042.00
atc	I	5	17247.00
ata	I	65152	25350.00
atg	M	91	91.00
act	T	0	0.31
acc	T	0	0.18
aca	T	1	0.43
acg	T	0	0.08
gtt	V	0	9.52
gtc	V	0	7.74
gta	V	38	8.24
gtg	V	0	12.50
gct	A	1	0.55
gcc	A	0	0.50
gca	A	0	0.81
gcg	A	1	0.15
---	--	------	------
mPD		0.019	0.66
		nPD:	0.03
		N. weight:	1.
		Sc. PD:	-0.052
		Sc. rank:	2521.8

,

HA			
Pos.	92	obs:	exp:
tat	Y	1	0.53
tac	Y	0	0.47
aat	N	5	2.80
aac	N	0	2.20
gat	D	65729	37175.00
gac	D	37	28591.00
ggt	G	0	0.27
ggc	G	2	0.28
gga	G	0	0.92
ggg	G	0	0.53
---	--	------	------
mPD		0.0014	0.49
		nPD:	0.
		N. weight:	0.63
		Sc. PD:	-0.048
		Sc. rank:	-29295.0

,

HA			
Pos.	93	obs:	exp:
tct	S	9	1.45
tcc	S	0	1.34
tca	S	0	2.42
tcg	S	0	0.35
tgg	W	2	2.00
act	T	5	1.55
acc	T	0	0.88
aca	T	0	2.16
acg	T	0	0.40
agt	S	0	1.57
agc	S	0	1.86
gtt	V	3	0.75
gtc	V	0	0.61
gta	V	0	0.65
gtg	V	0	0.99
gct	A	64488	17922.00
gcc	A	1241	16428.00
gca	A	20	26598.00
gcg	A	6	4807.00
---	--	------	------
mPD		0.038	0.70
		nPD:	0.06
		N. weight:	1.3
		Sc. PD:	-0.035
		Sc. rank:	24195.0

,

HA			
Pos.	94	obs:	exp:
tta	L	199	7632.00
ttg	L	0	13597.00
ctt	L	2	13758.00
ctc	L	10	9644.00
cta	L	65463	10099.00
ctg	L	94	11038.00
att	I	0	2.11
atc	I	2	1.58
ata	I	4	2.32
---	--	------	------
mPD		0.0095	1.2
		nPD:	0.01
		N. weight:	2.
		Sc. PD:	-0.15
		Sc. rank:	-67773.0

,

HA			
Pos.	95	obs:	exp:
tta	L	215	7632.00
ttg	L	65217	13598.00
ctt	L	0	13759.00
ctc	L	0	9645.00
cta	L	23	10100.00
ctg	L	318	11039.00
atg	M	1	1.00
---	--	------	------
mPD		0.018	1.2
		nPD:	0.02
		N. weight:	1.7
		Sc. PD:	-0.11
		Sc. rank:	-28209.0

,

HA			
Pos.	96	obs:	exp:
cgt	R	0	0.02
cgc	R	0	0.04
cga	R	1	0.09
cgg	R	0	0.09
aga	R	0	0.49
agg	R	0	0.28
ggt	G	11	8855.00
ggc	G	2	9180.00
gga	G	42412	30362.00
ggg	G	23348	17377.00
---	--	------	------
mPD		0.46	0.68
		nPD:	0.67
		N. weight:	0.35
		Sc. PD:	0.2
		Sc. rank:	17840.0

,

HA			
Pos.	97	obs:	exp:
aat	N	2	1.12
aac	N	0	0.88
gat	D	341	37176.00
gac	D	65427	28592.00
gaa	E	2	1.18
gag	E	0	0.82
ggt	G	0	0.27
ggc	G	2	0.28
gga	G	0	0.92
ggg	G	0	0.53
---	--	------	------
mPD		0.011	0.49
		nPD:	0.02
		N. weight:	0.88
		Sc. PD:	-0.052
		Sc. rank:	-5143.6

,

HA			
Pos.	98	obs:	exp:
tta	L	0	0.23
ttg	L	0	0.41
ctt	L	2	0.42
ctc	L	0	0.29
cta	L	0	0.31
ctg	L	0	0.34
cct	P	64964	21917.00
ccc	P	783	12330.00
cca	P	21	22221.00
ccg	P	3	9302.00
act	T	1	0.31
acc	T	0	0.18
aca	T	0	0.43
acg	T	0	0.08
---	--	------	------
mPD		0.024	0.72
		nPD:	0.03
		N. weight:	1.1
		Sc. PD:	-0.054
		Sc. rank:	6098.0

,

HA			
Pos.	99	obs:	exp:
tta	L	0	1.16
ttg	L	0	2.07
ctt	L	0	2.09
ctc	L	0	1.47
cta	L	0	1.54
ctg	L	10	1.68
cat	H	957	541.50
cac	H	3	418.50
caa	Q	250	39605.00
cag	Q	64540	25185.00
cgt	R	0	0.12
cgc	R	0	0.22
cga	R	0	0.55
cgg	R	6	0.52
aat	N	3	1.68
aac	N	0	1.32
aaa	K	0	1.97
aag	K	3	1.03
aga	R	0	2.94
agg	R	0	1.66
gaa	E	0	1.18
gag	E	2	0.82
---	--	------	------
mPD		0.037	0.49
		nPD:	0.08
		N. weight:	1.
		Sc. PD:	-0.0076
		Sc. rank:	23690.0

,

HA			
Pos.	100	obs:	exp:
tgt	C	65653	25703.00
tgc	C	118	40068.00
tgg	W	2	2.00
ggt	G	1	0.13
ggc	G	0	0.14
gga	G	0	0.46
ggg	G	0	0.26
---	--	------	------
mPD		0.0037	0.48
		nPD:	0.01
		N. weight:	1.
		Sc. PD:	-0.074
		Sc. rank:	-34799.0

,

HA			
Pos.	101	obs:	exp:
tat	Y	1	1.58
tac	Y	2	1.42
aat	N	6	5.04
aac	N	3	3.96
gtt	V	0	0.25
gtc	V	1	0.20
gta	V	0	0.22
gtg	V	0	0.33
gat	D	41959	37133.00
gac	D	23732	28558.00
gaa	E	34	40.68
gag	E	35	28.32
ggt	G	1	0.13
ggc	G	0	0.14
gga	G	0	0.46
ggg	G	0	0.26
---	--	------	------
mPD		0.46	0.49
		nPD:	0.94
		N. weight:	0.013
		Sc. PD:	0.01
		Sc. rank:	647.3

,

HA			
Pos.	102	obs:	exp:
tct	S	0	59.71
tcc	S	0	55.29
tca	S	2	99.45
tcg	S	0	14.56
cat	H	0	0.56
cac	H	1	0.44
aat	N	0	0.56
aac	N	1	0.44
agt	S	1	64.66
agc	S	367	76.33
gtt	V	30	8.52
gtc	V	4	6.93
gta	V	0	7.37
gtg	V	0	11.19
gct	A	0	0.27
gcc	A	1	0.25
gca	A	0	0.40
gcg	A	0	0.07
gat	D	0	469.70
gac	D	831	361.30
gaa	E	1	0.59
gag	E	0	0.41
ggt	G	57	8688.00
ggc	G	63634	9007.00
gga	G	835	29790.00
ggg	G	9	17050.00
---	--	------	------
mPD		0.065	0.73
		nPD:	0.09
		N. weight:	2.1
		Sc. PD:	0.013
		Sc. rank:	54574.0

,

HA			
Pos.	103	obs:	exp:
ttt	F	57692	31609.00
ttc	F	7290	33373.00
tta	L	36	28.66
ttg	L	2	51.07
tct	S	2	0.48
tcc	S	1	0.45
tca	S	0	0.81
tcg	S	0	0.12
tat	Y	1	0.53
tac	Y	0	0.47
ctt	L	186	51.67
ctc	L	23	36.22
cta	L	0	37.93
ctg	L	0	41.45
att	I	10	3.86
atc	I	1	2.89
ata	I	0	4.25
agt	S	0	0.52
agc	S	0	0.62
gtt	V	529	132.80
gtc	V	0	108.00
gta	V	1	114.90
gtg	V	0	174.40
---	--	------	------
mPD		0.22	0.53
		nPD:	0.42
		N. weight:	0.41
		Sc. PD:	0.13
		Sc. rank:	19352.0

,

HA			
Pos.	104	obs:	exp:
tct	S	0	0.32
tcc	S	2	0.30
tca	S	0	0.54
tcg	S	0	0.08
cat	H	0	0.56
cac	H	1	0.44
caa	Q	65634	40163.00
cag	Q	70	25541.00
cgt	R	0	0.24
cgc	R	0	0.43
cga	R	10	1.09
cgg	R	0	1.03
aaa	K	27	36.16
aag	K	28	18.84
agt	S	0	0.35
agc	S	0	0.41
aga	R	2	5.89
agg	R	0	3.32
---	--	------	------
mPD		0.0053	0.48
		nPD:	0.01
		N. weight:	0.53
		Sc. PD:	-0.037
		Sc. rank:	-14730.0

,

HA			
Pos.	105	obs:	exp:
tct	S	0	0.81
tcc	S	0	0.75
tca	S	0	1.34
tcg	S	0	0.20
cat	H	3	1.69
cac	H	0	1.31
att	I	1	0.35
atc	I	0	0.26
ata	I	0	0.39
act	T	0	0.62
acc	T	0	0.35
aca	T	2	0.87
acg	T	0	0.16
aat	N	63280	36716.00
aac	N	2347	28911.00
aaa	K	7	4.60
aag	K	0	2.40
agt	S	5	0.87
agc	S	0	1.03
gat	D	124	72.92
gac	D	5	56.08
---	--	------	------
mPD		0.073	0.50
		nPD:	0.15
		N. weight:	0.48
		Sc. PD:	0.029
		Sc. rank:	16866.0

,

HA			
Pos.	106	obs:	exp:
gct	A	0	0.55
gcc	A	0	0.50
gca	A	2	0.81
gcg	A	0	0.15
---	-	65772	65772.00
---	--	------	------
mPD		0	6.4×10^{-10}
		nPD:	0.
		N. weight:	0.
		Sc. PD:	0
		Sc. rank:	0

,

HA			
Pos.	107	obs:	exp:
tct	S	0	0.32
tcc	S	0	0.30
tca	S	0	0.54
tcg	S	0	0.08
tgg	W	1	1.00
caa	Q	0	0.61
cag	Q	1	0.39
cgt	R	0	17.01
cgc	R	0	30.87
cga	R	0	78.28
cgg	R	0	73.67
act	T	0	2.17
acc	T	0	1.24
aca	T	0	3.03
acg	T	7	0.56
aat	N	72	40.84
aac	N	1	32.16
aaa	K	197	42548.00
aag	K	64521	22170.00
agt	S	1	0.35
agc	S	1	0.41
aga	R	4	420.80
agg	R	854	237.30
gaa	E	0	66.62
gag	E	113	46.38
ggt	G	0	0.13
ggc	G	0	0.14
gga	G	0	0.46
ggg	G	1	0.26
---	--	------	------
mPD		0.038	0.49
		nPD:	0.08
		N. weight:	1.2
		Sc. PD:	-0.0062
		Sc. rank:	28300.0

,

HA			
Pos.	108	obs:	exp:
tct	S	0	0.32
tcc	S	0	0.30
tca	S	2	0.54
tcg	S	0	0.08
caa	Q	1	0.61
cag	Q	0	0.39
cgt	R	0	11.40
cgc	R	0	20.69
cga	R	0	52.46
cgg	R	0	49.37
act	T	0	16.15
acc	T	0	9.19
aca	T	52	22.50
acg	T	0	4.15
aat	N	48	36.93
aac	N	18	29.07
aaa	K	52250	34953.00
aag	K	915	18212.00
agt	S	0	0.35
agc	S	0	0.41
aga	R	572	282.00
agg	R	3	159.00
gat	D	0	0.57
gac	D	1	0.43
gaa	E	11893	7021.00
gag	E	15	4887.00
ggt	G	0	0.54
ggc	G	0	0.56
gga	G	4	1.85
ggg	G	0	1.06
---	--	------	------
mPD		0.35	0.78
		nPD:	0.44
		N. weight:	0.42
		Sc. PD:	0.14
		Sc. rank:	19947.0

,

HA			
Pos.	109	obs:	exp:
tgg	W	65773	65773.00
cgt	R	0	0.02
cgc	R	0	0.04
cga	R	0	0.09
cgg	R	1	0.09
aga	R	0	0.49
agg	R	0	0.28
---	--	------	------
mPD		0.000030	0.000050
		nPD:	0.61
		N. weight:	0.000041
		Sc. PD:	0.000021
		Sc. rank:	2.1

,

HA			
Pos.	110	obs:	exp:
tct	S	0	0.32
tcc	S	2	0.30
tca	S	0	0.54
tcg	S	0	0.08
aat	N	0	9.51
aac	N	17	7.49
agt	S	0	0.35
agc	S	0	0.41
gat	D	1173	37158.00
gac	D	64563	28578.00
gaa	E	6	3.54
gag	E	0	2.46
ggt	G	0	1.75
ggc	G	12	1.81
gga	G	1	6.00
ggg	G	0	3.43
---	--	------	------
mPD		0.036	0.49
		nPD:	0.07
		N. weight:	0.82
		Sc. PD:	-0.0075
		Sc. rank:	18627.0

,

HA			
Pos.	111	obs:	exp:
ttt	F	1	0.49
ttc	F	0	0.51
tta	L	0	7631.00
ttg	L	0	13596.00
tat	Y	0	1.05
tac	Y	2	0.95
ctt	L	65610	13756.00
ctc	L	142	9643.00
cta	L	2	10098.00
ctg	L	6	11036.00
cct	P	1	0.33
ccc	P	0	0.19
cca	P	0	0.34
ccg	P	0	0.14
att	I	10	3.51
atc	I	0	2.63
ata	I	0	3.86
---	--	------	------
mPD		0.0051	1.2
		nPD:	0.
		N. weight:	1.7
		Sc. PD:	-0.13
		Sc. rank:	-74707.0

,

HA			
Pos.	112	obs:	exp:
ttt	F	60366	31980.00
ttc	F	5379	33765.00
tta	L	1	0.58
ttg	L	0	1.03
tct	S	1	0.16
tcc	S	0	0.15
tca	S	0	0.27
tcg	S	0	0.04
tat	Y	19	10.02
tac	Y	0	8.98
ctt	L	4	1.05
ctc	L	0	0.73
cta	L	0	0.77
ctg	L	0	0.84
att	I	4	1.40
atc	I	0	1.05
ata	I	0	1.54
agt	S	0	0.17
agc	S	0	0.21
---	--	------	------
mPD		0.15	0.50
		nPD:	0.3
		N. weight:	0.48
		Sc. PD:	0.1
		Sc. rank:	20317.0

,

HA			
Pos.	113	obs:	exp:
tta	L	0	0.35
ttg	L	0	0.62
ctt	L	3	0.63
ctc	L	0	0.44
cta	L	0	0.46
ctg	L	0	0.50
att	I	200	70.21
atc	I	0	52.55
ata	I	0	77.24
gtt	V	65388	16424.00
gtc	V	171	13361.00
gta	V	5	14212.00
gtg	V	7	21574.00
---	--	------	------
mPD		0.012	0.75
		nPD:	0.02
		N. weight:	1.5
		Sc. PD:	-0.097
		Sc. rank:	-24156.0

,

HA			
Pos.	114	obs:	exp:
gtt	V	1	0.25
gtc	V	0	0.20
gta	V	0	0.22
gtg	V	0	0.33
gat	D	1	0.57
gac	D	0	0.43
gaa	E	65685	38776.00
gag	E	86	26995.00
ggt	G	0	0.13
ggc	G	0	0.14
gga	G	1	0.46
ggg	G	0	0.26
---	--	------	------
mPD		0.0027	0.48
		nPD:	0.01
		N. weight:	0.57
		Sc. PD:	-0.042
		Sc. rank:	-22676.0

,

HA

Pos.	115	obs:	exp:
caa	Q	1	0.61
cag	Q	0	0.39
cgt	R	7	1304.00
cgc	R	2145	2367.00
cga	R	47325	6001.00
cgg	R	4744	5648.00
act	T	0	0.62
acc	T	0	0.35
aca	T	2	0.87
acg	T	0	0.16
aga	R	11496	32260.00
agg	R	54	18192.00
---	--	------	------
mPD		0.48	0.89
		nPD:	0.55
		N. weight:	1.4
		Sc. PD:	0.62
		Sc. rank:	70173.0

,

HA

Pos.	116	obs:	exp:
tct	S	1	9654.00
tcc	S	0	8940.00
tca	S	0	16080.00
tcg	S	0	2355.00
tat	Y	0	0.53
tac	Y	1	0.47
tgt	C	0	0.78
tgc	C	2	1.22
cct	P	1	0.33
ccc	P	0	0.19
cca	P	0	0.34
ccg	P	0	0.14
cgt	R	0	0.56
cgc	R	13	1.01
cga	R	0	2.55
cgg	R	0	2.40
att	I	0	1.05
atc	I	3	0.79
ata	I	0	1.16
act	T	0	0.31
acc	T	1	0.18
aca	T	0	0.43
acg	T	0	0.08
aat	N	8	3291.00
aac	N	5875	2592.00
aaa	K	1	0.66
aag	K	0	0.34
agt	S	302	10455.00
agc	S	59523	12342.00
aga	R	10	13.73
agg	R	5	7.74
gat	D	0	1.13
gac	D	2	0.87
ggt	G	0	3.50
ggc	G	26	3.63
gga	G	0	12.00
ggg	G	0	6.87
---	--	------	------
mPD		0.17	1.7
		nPD:	0.1
		N. weight:	1.6
		Sc. PD:	0.028
		Sc. rank:	47412.0

,

HA

Pos.	117	obs:	exp:
tct	S	0	0.65
tcc	S	0	0.60
tca	S	0	1.08
tcg	S	0	0.16
caa	Q	7	4.28
cag	Q	0	2.72
cgt	R	0	488.20
cgc	R	0	886.00
cga	R	0	2247.00
cgg	R	0	2114.00
att	I	0	2.46
atc	I	0	1.84
ata	I	7	2.70
act	T	0	67.39
acc	T	0	38.37
aca	T	217	93.91
acg	T	0	17.33
aat	N	7	11.19
aac	N	13	8.81
aaa	K	40844	26879.00
aag	K	41	14006.00
agt	S	3	0.70
agc	S	1	0.83
aga	R	24546	12077.00
agg	R	77	6811.00
gat	D	1	0.57
gac	D	0	0.43
gaa	E	6	3.54
gag	E	0	2.46
ggt	G	0	0.54
ggc	G	0	0.56
gga	G	4	1.85
ggg	G	0	1.06
---	--	------	------
mPD		0.48	1.1
		nPD:	0.43
		N. weight:	0.57
		Sc. PD:	0.19
		Sc. rank:	27083.0

,

HA

Pos.	118	obs:	exp:
tct	S	0	2.42
tcc	S	13	2.24
tca	S	2	4.03
tcg	S	0	0.59
act	T	0	9.94
acc	T	32	5.66
aca	T	0	13.85
acg	T	0	2.56
agt	S	0	2.62
agc	S	0	3.09
gtt	V	0	1.75
gtc	V	7	1.43
gta	V	0	1.52
gtg	V	0	2.30
gct	A	449	17912.00
gcc	A	65151	16420.00
gca	A	118	26584.00
gcg	A	2	4804.00
---	--	------	------
mPD		0.019	0.70
		nPD:	0.03
		N. weight:	1.5
		Sc. PD:	-0.079
		Sc. rank:	666.9

,

HA

Pos.	119	obs:	exp:
gat	D	0	1.13
gac	D	2	0.87
---	-	65772	65772.00
---	--	------	------
mPD		0	4.5×10^{-10}
		nPD:	0.
		N. weight:	0.
		Sc. PD:	0
		Sc. rank:	0

,

HA

Pos.	120	obs:	exp:
ttt	F	0	47.18
ttc	F	97	49.82
tct	S	0	9.20
tcc	S	5	8.52
tca	S	0	15.32
tcg	S	0	2.24
tat	Y	27	27414.00
tac	Y	51981	24594.00
tgt	C	0	8.99
tgc	C	23	14.01
cat	H	1	1457.00
cac	H	2582	1126.00
caa	Q	3	1.83
cag	Q	0	1.17
cgt	R	0	0.02
cgc	R	1	0.04
cga	R	0	0.09
cgg	R	0	0.09
aat	N	7	6126.00
aac	N	10942	4823.00
aaa	K	52	34.19
aag	K	0	17.81
agt	S	0	9.96
agc	S	52	11.76
aga	R	0	0.49
agg	R	0	0.28
gat	D	0	0.57
gac	D	1	0.43
---	--	------	------
mPD		0.35	0.85
		nPD:	0.41
		N. weight:	0.84
		Sc. PD:	0.26
		Sc. rank:	39040.0

,

HA

Pos.	121	obs:	exp:
tct	S	0	10609.00
tcc	S	0	9825.00
tca	S	0	17671.00
tcg	S	0	2588.00
cgt	R	0	0.08
cgc	R	0	0.14
cga	R	0	0.36
cgg	R	0	0.34
att	I	0	0.35
atc	I	1	0.26
ata	I	0	0.39
aat	N	0	1.68
aac	N	3	1.32
agt	S	53	11489.00
agc	S	65693	13563.00
aga	R	4	1.96
agg	R	0	1.11
ggt	G	0	2.69
ggc	G	18	2.79
gga	G	2	9.23
ggg	G	0	5.28
---	--	------	------
mPD		0.0025	1.6
		nPD:	0.
		N. weight:	1.7
		Sc. PD:	-0.14
		Sc. rank:	-88174.0

,

HA

Pos.	122	obs:	exp:
tct	S	0	439.70
tcc	S	0	407.20
tca	S	0	732.40
tcg	S	0	107.30
tat	Y	0	0.53
tac	Y	1	0.47
tgt	C	0	0.39
tgc	C	1	0.61
caa	Q	1	0.61
cag	Q	0	0.39
att	I	0	0.35
atc	I	1	0.26
ata	I	0	0.39
act	T	0	1.24
acc	T	2	0.71
aca	T	0	1.73
acg	T	2	0.32
aat	N	140	35244.00
aac	N	62855	27751.00
aaa	K	29	21.70
aag	K	4	11.30
agt	S	8	476.20
agc	S	2717	562.20
gat	D	0	6.78
gac	D	12	5.22
ggt	G	0	0.13
ggc	G	1	0.14
gga	G	0	0.46
ggg	G	0	0.26
---	--	------	------
mPD		0.086	0.64
		nPD:	0.13
		N. weight:	0.92
		Sc. PD:	0.045
		Sc. rank:	31295.0

,

HA

Pos.	123	obs:	exp:
tgt	C	65677	25704.00
tgc	C	96	40069.00
tgg	W	1	1.00
---	--	------	------
mPD		0.0029	0.48
		nPD:	0.01
		N. weight:	1.
		Sc. PD:	-0.075
		Sc. rank:	-39236.0

,

HA

Pos.	124	obs:	exp:
tat	Y	358	34670.00
tac	Y	65415	31103.00
aat	N	0	0.56
aac	N	1	0.44
---	--	------	------
mPD		0.011	0.50
		nPD:	0.02
		N. weight:	0.79
		Sc. PD:	-0.046
		Sc. rank:	-4036.4

,

HA

Pos.	125	obs:	exp:
tct	S	1	0.16
tcc	S	0	0.15
tca	S	0	0.27
tcg	S	0	0.04
cct	P	65348	21918.00
ccc	P	404	12331.00
cca	P	13	22222.00
ccg	P	8	9303.00
agt	S	0	0.17
agc	S	0	0.21
---	--	------	------
mPD		0.013	0.72
		nPD:	0.02
		N. weight:	1.2
		Sc. PD:	-0.073
		Sc. rank:	-14624.0

,

HA

Pos.	126	obs:	exp:
ttt	F	5	2.43
ttc	F	0	2.57
tat	Y	60701	34667.00
tac	Y	5066	31100.00
ggt	G	0	0.27
ggc	G	0	0.28
gga	G	2	0.92
ggg	G	0	0.53
---	--	------	------
mPD		0.14	0.50
		nPD:	0.29
		N. weight:	0.42
		Sc. PD:	0.08
		Sc. rank:	17581.0

,

HA

Pos.	127	obs:	exp:
tat	Y	153	81.18
tac	Y	1	72.82
aat	N	17	9.51
aac	N	0	7.49
gat	D	65499	37073.00
gac	D	86	28512.00
gaa	E	12	10.61
gag	E	6	7.39
---	--	------	------
mPD		0.0084	0.50
		nPD:	0.02
		N. weight:	0.62
		Sc. PD:	-0.039
		Sc. rank:	-8604.3

,

HA

Pos.	128	obs:	exp:
ttt	F	0	0.97
ttc	F	2	1.03
tta	L	0	0.23
ttg	L	2	0.41
ctt	L	0	0.42
ctc	L	0	0.29
cta	L	0	0.31
ctg	L	0	0.34
att	I	1	0.35
atc	I	0	0.26
ata	I	0	0.39
atg	M	15	15.00
gtt	V	52	16470.00
gtc	V	5	13398.00
gta	V	844	14252.00
gtg	V	64853	21635.00
---	--	------	------
mPD		0.028	0.74
		nPD:	0.04
		N. weight:	1.2
		Sc. PD:	-0.05
		Sc. rank:	11182.0

,

HA

Pos.	129	obs:	exp:
tta	L	0	0.81
ttg	L	0	1.45
tct	S	0	7.58
tcc	S	0	7.02
tca	S	0	12.63
tcg	S	47	1.85
ctt	L	0	1.46
ctc	L	0	1.03
cta	L	0	1.07
ctg	L	7	1.17
cct	P	60	21890.00
ccc	P	5	12315.00
cca	P	659	22193.00
ccg	P	64965	9291.00
caa	Q	1	14.06
cag	Q	22	8.94
att	I	0	0.70
atc	I	2	0.53
ata	I	0	0.77
act	T	0	1.55
acc	T	0	0.88
aca	T	0	2.16
acg	T	5	0.40
agt	S	0	8.21
agc	S	0	9.70
gct	A	0	0.27
gcc	A	0	0.25
gca	A	0	0.40
gcg	A	1	0.07
---	--	------	------
mPD		0.025	0.72
		nPD:	0.03
		N. weight:	2.1
		Sc. PD:	-0.098
		Sc. rank:	11813.0

,

HA

Pos.	130	obs:	exp:
tct	S	0	0.16
tcc	S	0	0.15
tca	S	0	0.27
tcg	S	0	0.04
tat	Y	1	0.53
tac	Y	0	0.47
aat	N	106	59.30
aac	N	0	46.70
agt	S	1	0.17
agc	S	0	0.21
gct	A	2	0.55
gcc	A	0	0.50
gca	A	0	0.81
gcg	A	0	0.15
gat	D	59760	33899.00
gac	D	211	26072.00
gaa	E	2	17.69
gag	E	28	12.31
ggt	G	5660	762.40
ggc	G	2	790.40
gga	G	0	2614.00
ggg	G	1	1496.00
---	--	------	------
mPD		0.17	0.71
		nPD:	0.24
		N. weight:	0.74
		Sc. PD:	0.11
		Sc. rank:	29920.0

,

HA

Pos.	131	obs:	exp:
ttt	F	7	3.40
ttc	F	0	3.60
tct	S	1	0.16
tcc	S	0	0.15
tca	S	0	0.27
tcg	S	0	0.04
tat	Y	65640	34621.00
tac	Y	41	31060.00
tgt	C	1	0.39
tgc	C	0	0.61
cat	H	84	47.38
cac	H	0	36.62
agt	S	0	0.17
agc	S	0	0.21
---	--	------	------
mPD		0.0041	0.50
		nPD:	0.01
		N. weight:	0.7
		Sc. PD:	-0.05
		Sc. rank:	-23627.0

,

HA

Pos.	132	obs:	exp:
tct	S	0	2.10
tcc	S	12	1.94
tca	S	1	3.49
tcg	S	0	0.51
act	T	0	46.27
acc	T	121	26.34
aca	T	28	64.48
acg	T	0	11.90
agt	S	0	2.27
agc	S	0	2.68
gtt	V	0	62.62
gtc	V	249	50.94
gta	V	1	54.19
gtg	V	0	82.26
gct	A	202	17813.00
gcc	A	59742	16328.00
gca	A	5410	26436.00
gcg	A	1	4778.00
gat	D	0	0.57
gac	D	1	0.43
gaa	E	1	1.77
gag	E	2	1.23
ggt	G	0	0.40
ggc	G	3	0.42
gga	G	0	1.38
ggg	G	0	0.79
---	--	------	------
mPD		0.17	0.71
		nPD:	0.24
		N. weight:	1.2
		Sc. PD:	0.17
		Sc. rank:	47047.0

,

HA

Pos.	133	obs:	exp:
ttt	F	0	1.95
ttc	F	4	2.05
tct	S	565	10589.00
tcc	S	64990	9806.00
tca	S	64	17637.00
tcg	S	0	2583.00
cct	P	0	1.67
ccc	P	5	0.94
cca	P	0	1.69
ccg	P	0	0.71
act	T	1	44.72
acc	T	143	25.46
aca	T	0	62.32
acg	T	0	11.50
agt	S	0	11467.00
agc	S	0	13537.00
gaa	E	0	1.18
gag	E	2	0.82
---	--	------	------
mPD		0.024	1.6
		nPD:	0.01
		N. weight:	2.
		Sc. PD:	-0.13
		Sc. rank:	-37368.0

,

HA

Pos.	134	obs:	exp:
ttt	F	2	0.97
ttc	F	0	1.03
tta	L	0	7632.00
ttg	L	0	13598.00
ctt	L	65547	13758.00
ctc	L	137	9645.00
cta	L	13	10099.00
ctg	L	73	11038.00
att	I	2	0.70
atc	I	0	0.53
ata	I	0	0.77
---	--	------	------
mPD		0.0069	1.2
		nPD:	0.01
		N. weight:	1.7
		Sc. PD:	-0.13
		Sc. rank:	-66400.0

,

HA

Pos.	135	obs:	exp:
cgt	R	0	1304.00
cgc	R	0	2366.00
cga	R	0	6000.00
cgg	R	8	5646.00
atg	M	1	1.00
aaa	K	2	11.18
aag	K	15	5.82
aga	R	6071	32252.00
agg	R	59677	18188.00
---	--	------	------
mPD		0.17	0.89
		nPD:	0.19
		N. weight:	1.
		Sc. PD:	0.1
		Sc. rank:	39168.0

,

HA

Pos.	136	obs:	exp:
tta	L	3	0.35
ttg	L	0	0.62
tct	S	7	10613.00
tcc	S	23	9828.00
tca	S	65712	17677.00
tcg	S	26	2589.00
ctt	L	0	0.63
ctc	L	0	0.44
cta	L	0	0.46
ctg	L	0	0.50
agt	S	0	11493.00
agc	S	0	13568.00
gct	A	0	0.27
gcc	A	0	0.25
gca	A	1	0.40
gcg	A	0	0.07
gaa	E	0	1.18
gag	E	2	0.82
---	--	------	------
mPD		0.0020	1.6
		nPD:	0.
		N. weight:	1.4
		Sc. PD:	-0.11
		Sc. rank:	-73899.0

,

HA

Pos.	137	obs:	exp:
tta	L	184	7628.00
ttg	L	0	13591.00
ctt	L	4	13752.00
ctc	L	1	9640.00
cta	L	65341	10095.00
ctg	L	209	11033.00
caa	Q	2	1.22
cag	Q	0	0.78
att	I	0	11.58
atc	I	0	8.67
ata	I	33	12.74
---	--	------	------
mPD		0.013	1.2
		nPD:	0.01
		N. weight:	2.
		Sc. PD:	-0.14
		Sc. rank:	-53811.0

,

HA

Pos.	138	obs:	exp:
ttt	F	5	2.43
ttc	F	0	2.57
tta	L	0	0.23
ttg	L	2	0.41
ctt	L	0	0.42
ctc	L	0	0.29
cta	L	0	0.31
ctg	L	0	0.34
att	I	202	70.91
atc	I	0	53.08
ata	I	0	78.01
gtt	V	65477	16421.00
gtc	V	39	13358.00
gta	V	33	14209.00
gtg	V	8	21570.00
gct	A	8	2.18
gcc	A	0	2.00
gca	A	0	3.24
gcg	A	0	0.58
---	--	------	------
mPD		0.0091	0.75
		nPD:	0.01
		N. weight:	1.5
		Sc. PD:	-0.1
		Sc. rank:	-38159.0

,

HA

Pos.	139	obs:	exp:
tct	S	0	1.78
tcc	S	9	1.64
tca	S	0	2.96
tcg	S	0	0.43
agt	S	0	1.92
agc	S	2	2.27
gct	A	102	17924.00
gcc	A	65577	16430.00
gca	A	83	26601.00
gcg	A	0	4807.00
ggt	G	0	0.13
ggc	G	1	0.14
gga	G	0	0.46
ggg	G	0	0.26
---	--	------	------
mPD		0.0060	0.69
		nPD:	0.01
		N. weight:	1.5
		Sc. PD:	-0.11
		Sc. rank:	-48559.0

,

HA

Pos.	140	obs:	exp:
tta	L	11	1.28
ttg	L	0	2.27
tct	S	36	10533.00
tcc	S	56	9755.00
tca	S	65070	17545.00
tcg	S	113	2569.00
ctt	L	0	2.30
ctc	L	0	1.61
cta	L	0	1.69
ctg	L	0	1.85
act	T	0	135.40
acc	T	0	77.09
aca	T	436	188.70
acg	T	0	34.82
agt	S	0	11407.00
agc	S	0	13466.00
gct	A	0	14.17
gcc	A	0	12.99
gca	A	52	21.03
gcg	A	0	3.80
---	--	------	------
mPD		0.021	1.6
		nPD:	0.01
		N. weight:	1.4
		Sc. PD:	-0.095
		Sc. rank:	-31090.0

,

HA

Pos.	141	obs:	exp:
ttt	F	0	4.86
ttc	F	10	5.14
tct	S	872	10609.00
tcc	S	64513	9825.00
tca	S	337	17671.00
tcg	S	22	2588.00
tat	Y	0	0.53
tac	Y	1	0.47
cct	P	0	0.33
ccc	P	1	0.19
cca	P	0	0.34
ccg	P	0	0.14
act	T	0	1.55
acc	T	5	0.88
aca	T	0	2.16
acg	T	0	0.40
agt	S	0	11489.00
agc	S	0	13563.00
gtt	V	0	0.50
gtc	V	0	0.41
gta	V	0	0.43
gtg	V	2	0.66
gct	A	0	3.00
gcc	A	11	2.75
gca	A	0	4.45
gcg	A	0	0.80
---	--	------	------
mPD		0.038	1.6
		nPD:	0.02
		N. weight:	2.
		Sc. PD:	-0.11
		Sc. rank:	-6538.0

,

HA

Pos.	142	obs:	exp:
tct	S	0	0.32
tcc	S	0	0.30
tca	S	2	0.54
tcg	S	0	0.08
agt	S	0	0.35
agc	S	0	0.41
ggt	G	419	8855.00
ggc	G	64939	9180.00
gga	G	413	30361.00
ggg	G	1	17376.00
---	--	------	------
mPD		0.025	0.68
		nPD:	0.04
		N. weight:	2.1
		Sc. PD:	-0.091
		Sc. rank:	19830.0

,

HA

Pos.	143	obs:	exp:
tct	S	0	2.42
tcc	S	0	2.24
tca	S	15	4.03
tcg	S	0	0.59
att	I	0	1.05
atc	I	0	0.79
ata	I	3	1.16
atg	M	1	1.00
act	T	133	20376.00
acc	T	264	11600.00
aca	T	64805	28395.00
acg	T	410	5240.00
aat	N	14	78.33
aac	N	126	61.67
aaa	K	1	0.66
aag	K	0	0.34
agt	S	0	2.62
agc	S	0	3.09
gct	A	0	0.55
gcc	A	0	0.50
gca	A	1	0.81
gcg	A	1	0.15
---	--	------	------
mPD		0.033	0.68
		nPD:	0.05
		N. weight:	0.85
		Sc. PD:	-0.028
		Sc. rank:	13492.0

,

HA

Pos.	144	obs:	exp:
ttt	F	2	0.97
ttc	F	0	1.03
tta	L	0	7631.00
ttg	L	111	13595.00
ctt	L	28	13756.00
ctc	L	0	9643.00
cta	L	245	10098.00
ctg	L	65375	11036.00
atg	M	13	13.00
---	--	------	------
mPD		0.012	1.2
		nPD:	0.01
		N. weight:	1.9
		Sc. PD:	-0.13
		Sc. rank:	-54527.0

,

HA

Pos.	145	obs:	exp:
ttt	F	1	0.49
ttc	F	0	0.51
att	I	0	0.35
atc	I	0	0.26
ata	I	1	0.39
gat	D	16	9.04
gac	D	0	6.96
gaa	E	849	38765.00
gag	E	64903	26987.00
ggt	G	0	0.54
ggc	G	0	0.56
gga	G	1	1.85
ggg	G	3	1.06
---	--	------	------
mPD		0.026	0.48
		nPD:	0.05
		N. weight:	0.9
		Sc. PD:	-0.025
		Sc. rank:	16075.0

,

HA

Pos.	146	obs:	exp:
cgt	R	0	0.04
cgc	R	0	0.07
cga	R	0	0.18
cgg	R	0	0.17
aga	R	0	0.98
agg	R	2	0.55
---	-	65772	65772.00
---	--	------	------
mPD		0	8.2×10^{-10}
		nPD:	0.
		N. weight:	0.
		Sc. PD:	0
		Sc. rank:	0

,

HA			
Pos.	147	obs:	exp:
ttt	F	2	0.97
ttc	F	0	1.03
---	-	65772	65772.00
---	--	------	------
mPD		0	4.6×10^{-10}
		nPD:	0.
		N. weight:	0.
		Sc. PD:	0
		Sc. rank:	0

,

HA			
Pos.	148	obs:	exp:
gaa	E	2	1.18
gag	E	0	0.82
---	-	65772	65772.00
---	--	------	------
mPD		0	4.5×10^{-10}
		nPD:	0.
		N. weight:	0.
		Sc. PD:	0
		Sc. rank:	0

,

HA			
Pos.	149	obs:	exp:
att	I	0	0.70
atc	I	0	0.53
ata	I	2	0.77
---	-	65772	65772.00
---	--	------	------
mPD		0	6.1×10^{-10}
		nPD:	0.
		N. weight:	0.
		Sc. PD:	0
		Sc. rank:	0

,

HA			
Pos.	150	obs:	exp:
ttt	F	65601	31994.00
ttc	F	173	33780.00
---	--	------	------
mPD		0.0052	0.50
		nPD:	0.01
		N. weight:	0.78
		Sc. PD:	-0.054
		Sc. rank:	-21857.0

,

HA			
Pos.	151	obs:	exp:
tct	S	0	7.58
tcc	S	0	7.02
tca	S	0	12.63
tcg	S	0	1.85
tat	Y	0	2.11
tac	Y	4	1.89
cct	P	0	0.67
ccc	P	2	0.37
cca	P	0	0.68
ccg	P	0	0.28
cat	H	0	2.26
cac	H	4	1.74
caa	Q	3	1.83
cag	Q	0	1.17
cgt	R	0	0.12
cgc	R	0	0.22
cga	R	0	0.55
cgg	R	0	0.52
att	I	1	71.26
atc	I	201	53.34
ata	I	1	78.40
act	T	2	78.57
acc	T	244	44.73
aca	T	7	109.50
acg	T	0	20.21
aat	N	51	19147.00
aac	N	34173	15077.00
aaa	K	30334	19967.00
aag	K	37	10404.00
agt	S	0	8.21
agc	S	47	9.70
aga	R	6	2.94
agg	R	0	1.66
gat	D	0	334.60
gac	D	592	257.40
gaa	E	65	38.32
gag	E	0	26.68
---	--	------	------
mPD		0.53	0.77
		nPD:	0.69
		N. weight:	0.7
		Sc. PD:	0.4
		Sc. rank:	35274.0

,

HA			
Pos.	152	obs:	exp:
tct	S	0	20.82
tcc	S	0	19.28
tca	S	0	34.67
tcg	S	0	5.08
tat	Y	39	20.56
tac	Y	0	18.44
cat	H	14	7.90
cac	H	0	6.10
caa	Q	145	88.64
cag	Q	0	56.36
att	I	13	4.56
atc	I	0	3.42
ata	I	0	5.02
act	T	127	61.49
acc	T	71	35.01
aca	T	0	85.69
acg	T	0	15.81
aat	N	62800	35204.00
aac	N	124	27720.00
aaa	K	27	27.61
aag	K	15	14.39
agt	S	129	22.54
agc	S	0	26.61
gat	D	2265	1280.00
gac	D	0	984.70
gaa	E	0	1.77
gag	E	3	1.23
ggt	G	2	0.27
ggc	G	0	0.28
gga	G	0	0.92
ggg	G	0	0.53
---	--	------	------
mPD		0.094	0.58
		nPD:	0.16
		N. weight:	0.63
		Sc. PD:	0.047
		Sc. rank:	22669.0

,

HA			
Pos.	153	obs:	exp:
act	T	0	0.62
acc	T	0	0.35
aca	T	2	0.87
acg	T	0	0.16
gaa	E	65652	38777.00
gag	E	120	26995.00
---	--	------	------
mPD		0.0038	0.48
		nPD:	0.01
		N. weight:	0.57
		Sc. PD:	-0.041
		Sc. rank:	-19332.0

,

HA			
Pos.	154	obs:	exp:
tct	S	0	10469.00
tcc	S	0	9695.00
tca	S	0	17438.00
tcg	S	0	2554.00
tgt	C	0	0.39
tgc	C	1	0.61
cgt	R	1	2.34
cgc	R	34	4.25
cga	R	0	10.77
cgg	R	0	10.13
att	I	0	7.37
atc	I	21	5.52
ata	I	0	8.11
act	T	1	0.31
acc	T	0	0.18
aca	T	0	0.43
acg	T	0	0.08
aat	N	2	229.90
aac	N	409	181.10
agt	S	460	11338.00
agc	S	64418	13384.00
aga	R	83	57.88
agg	R	0	32.64
gat	D	1	83.09
gac	D	146	63.91
gaa	E	2	1.18
gag	E	0	0.82
ggt	G	35	26.25
ggc	G	160	27.22
gga	G	0	90.02
ggg	G	0	51.52
---	--	------	------
mPD		0.047	1.6
		nPD:	0.03
		N. weight:	1.7
		Sc. PD:	-0.088
		Sc. rank:	3958.7

,

HA			
Pos.	155	obs:	exp:
ttt	F	37	31991.00
ttc	F	65730	33776.00
tta	L	1	0.46
ttg	L	0	0.83
tat	Y	0	0.53
tac	Y	1	0.47
ctt	L	0	0.84
ctc	L	3	0.59
cta	L	0	0.61
ctg	L	0	0.67
---	-	2	2.00
---	--	------	------
mPD		0.0013	0.50
		nPD:	0.
		N. weight:	0.73
		Sc. PD:	-0.056
		Sc. rank:	-34930.0

,

HA			
Pos.	156	obs:	exp:
tct	S	0	4.20
tcc	S	0	3.89
tca	S	2	6.99
tcg	S	0	1.02
tat	Y	1	0.53
tac	Y	0	0.47
att	I	7	2.46
atc	I	0	1.84
ata	I	0	2.70
act	T	58	18.01
acc	T	0	10.25
aca	T	0	25.10
acg	T	0	4.63
aat	N	65373	36656.00
aac	N	146	28863.00
aaa	K	4	34.84
aag	K	49	18.16
agt	S	24	4.54
agc	S	0	5.36
gat	D	109	62.18
gac	D	1	47.82
---	--	------	------
mPD		0.012	0.50
		nPD:	0.02
		N. weight:	0.63
		Sc. PD:	-0.035
		Sc. rank:	-1145.2

,

HA			
Pos.	157	obs:	exp:
tta	L	0	0.12
ttg	L	1	0.21
tgt	C	1	0.39
tgc	C	0	0.61
tgg	W	65771	65771.00
ctt	L	0	0.21
ctc	L	0	0.15
cta	L	0	0.15
ctg	L	0	0.17
ggt	G	0	0.13
ggc	G	0	0.14
gga	G	0	0.46
ggg	G	1	0.26
---	--	------	------
mPD		0.000091	0.00015
		nPD:	0.6
		N. weight:	0.000065
		Sc. PD:	0.000031
		Sc. rank:	3.2

,

HA			
Pos.	158	obs:	exp:
tct	S	20	3.23
tcc	S	0	2.99
tca	S	0	5.38
tcg	S	0	0.79
cct	P	11	4.33
ccc	P	2	2.44
cca	P	0	4.39
ccg	P	0	1.84
att	I	38	13.34
atc	I	0	9.98
ata	I	0	14.68
act	T	47029	15483.00
acc	T	2759	8815.00
aca	T	32	21577.00
acg	T	36	3982.00
aat	N	76	42.52
aac	N	0	33.48
agt	S	0	3.50
agc	S	0	4.13
gtt	V	22	5.51
gtc	V	0	4.48
gta	V	0	4.77
gtg	V	0	7.24
gct	A	15644	4287.00
gcc	A	77	3930.00
gca	A	7	6362.00
gcg	A	1	1150.00
gat	D	20	11.31
gac	D	0	8.69
---	--	------	------
mPD		0.45	1.1
		nPD:	0.43
		N. weight:	1.2
		Sc. PD:	0.38
		Sc. rank:	54793.0

,

HA			
Pos.	159	obs:	exp:
cgt	R	0	0.20
cgc	R	0	0.36
cga	R	0	0.91
cgg	R	0	0.86
aat	N	2	1.12
aac	N	0	0.88
aga	R	10	4.90
agg	R	0	2.77
gaa	E	11	7.07
gag	E	1	4.93
ggt	G	16	8852.00
ggc	G	2	9177.00
gga	G	64658	30351.00
ggg	G	1074	17371.00
---	--	------	------
mPD		0.034	0.68
		nPD:	0.05
		N. weight:	0.77
		Sc. PD:	-0.025
		Sc. rank:	12254.0

,

HA			
Pos.	160	obs:	exp:
tta	L	0	0.12
ttg	L	0	0.21
ctt	L	0	0.21
ctc	L	1	0.15
cta	L	0	0.15
ctg	L	0	0.17
cat	H	2	1.13
cac	H	0	0.87
att	I	0	12.64
atc	I	36	9.46
ata	I	0	13.90
act	T	0	0.31
acc	T	1	0.18
aca	T	0	0.43
acg	T	0	0.08
gtt	V	227	16461.00
gtc	V	65277	13390.00
gta	V	212	14244.00
gtg	V	1	21622.00
gct	A	0	4.63
gcc	A	17	4.25
gca	A	0	6.88
gcg	A	0	1.24
---	--	------	------
mPD		0.015	0.74
		nPD:	0.02
		N. weight:	1.7
		Sc. PD:	-0.1
		Sc. rank:	-11823.0

,

HA			
Pos.	161	obs:	exp:
tct	S	3	0.81
tcc	S	0	0.75
tca	S	0	1.34
tcg	S	0	0.20
cgt	R	0	0.20
cgc	R	0	0.36
cga	R	0	0.91
cgg	R	0	0.86
att	I	78	27.38
atc	I	0	20.50
ata	I	0	30.12
act	T	42200	13172.00
acc	T	198	7499.00
aca	T	15	18357.00
acg	T	3	3388.00
aat	N	89	50.35
aac	N	1	39.65
aaa	K	22572	14929.00
aag	K	136	7779.00
agt	S	2	0.87
agc	S	0	1.03
aga	R	10	4.90
agg	R	0	2.77
gct	A	453	124.00
gcc	A	2	113.70
gca	A	0	184.00
gcg	A	0	33.26
gat	D	10	6.78
gac	D	2	5.22
---	--	------	------
mPD		0.93	1.1
		nPD:	0.82
		N. weight:	0.97
		Sc. PD:	0.68
		Sc. rank:	49840.0

,

HA			
Pos.	162	obs:	exp:
tct	S	0	0.32
tcc	S	0	0.30
tca	S	0	0.54
tcg	S	2	0.08
cct	P	0	2.00
ccc	P	0	1.12
cca	P	6	2.03
ccg	P	0	0.85
caa	Q	65077	40201.00
cag	Q	689	25565.00
agt	S	0	0.35
agc	S	0	0.41
---	--	------	------
mPD		0.021	0.48
		nPD:	0.04
		N. weight:	0.48
		Sc. PD:	-0.018
		Sc. rank:	6998.7

,

HA			
Pos.	163	obs:	exp:
tct	S	0	19.04
tcc	S	0	17.63
tca	S	0	31.72
tcg	S	0	4.64
tat	Y	0	0.53
tac	Y	1	0.47
cat	H	0	0.56
cac	H	1	0.44
att	I	0	0.35
atc	I	1	0.26
ata	I	0	0.39
act	T	0	0.93
acc	T	3	0.53
aca	T	0	1.30
acg	T	0	0.24
aat	N	1096	36503.00
aac	N	64149	28742.00
agt	S	110	20.62
agc	S	8	24.34
gat	D	110	228.40
gac	D	294	175.60
ggt	G	0	0.13
ggc	G	1	0.14
gga	G	0	0.46
ggg	G	0	0.26
---	--	------	------
mPD		0.055	0.51
		nPD:	0.11
		N. weight:	0.8
		Sc. PD:	0.019
		Sc. rank:	25322.0

,

HA			
Pos.	164	obs:	exp:
aaa	K	2	1.31
aag	K	0	0.69
---	-	65772	65772.00
---	--	------	------
mPD		0	4.2×10^{-10}
		nPD:	0.
		N. weight:	0.
		Sc. PD:	0
		Sc. rank:	0

,

HA			
Pos.	165	obs:	exp:
gaa	E	2	1.18
gag	E	0	0.82
ggt	G	5	8855.00
ggc	G	3	9180.00
gga	G	65141	30361.00
ggg	G	623	17376.00
---	--	------	------
mPD		0.019	0.68
		nPD:	0.03
		N. weight:	0.8
		Sc. PD:	-0.042
		Sc. rank:	1206.2

,

HA			
Pos.	166	obs:	exp:
tct	S	0	23.08
tcc	S	0	21.37
tca	S	143	38.44
tcg	S	0	5.63
caa	Q	1	0.61
cag	Q	0	0.39
cgt	R	0	0.32
cgc	R	0	0.58
cga	R	0	1.46
cgg	R	0	1.37
att	I	0	1.76
atc	I	0	1.31
ata	I	5	1.93
act	T	3	17413.00
acc	T	35	9913.00
aca	T	56006	24266.00
acg	T	27	4478.00
aat	N	684	384.90
aac	N	4	303.10
aaa	K	8487	5588.00
aag	K	13	2912.00
agt	S	0	24.99
agc	S	0	29.50
aga	R	16	7.85
agg	R	0	4.43
gtt	V	0	0.50
gtc	V	0	0.41
gta	V	2	0.43
gtg	V	0	0.66
gct	A	0	38.70
gcc	A	0	35.48
gca	A	142	57.44
gcg	A	0	10.38
gat	D	0	7.91
gac	D	14	6.09
gaa	E	53	31.25
gag	E	0	21.75
ggt	G	0	18.71
ggc	G	0	19.40
gga	G	139	64.16
ggg	G	0	36.72
---	--	------	------
mPD		0.28	0.94
		nPD:	0.3
		N. weight:	0.85
		Sc. PD:	0.18
		Sc. rank:	36358.0

,

HA

Pos.	167	obs:	exp:
tct	S	0	10613.00
tcc	S	0	9829.00
tca	S	0	17678.00
tcg	S	0	2589.00
cgt	R	0	0.02
cgc	R	0	0.04
cga	R	0	0.09
cgg	R	0	0.09
act	T	0	0.62
acc	T	0	0.35
aca	T	0	0.87
acg	T	2	0.16
agt	S	59615	11494.00
agc	S	6156	13568.00
aga	R	0	0.49
agg	R	1	0.28
---	--	------	------
mPD		0.17	1.6
		nPD:	0.1
		N. weight:	1.6
		Sc. PD:	0.031
		Sc. rank:	47726.0

,

HA

Pos.	168	obs:	exp:
ttt	F	1386	674.20
ttc	F	0	711.80
tta	L	0	0.23
ttg	L	0	0.41
tct	S	63828	10313.00
tcc	S	29	9550.00
tca	S	31	17177.00
tcg	S	10	2516.00
tat	Y	396	208.70
tac	Y	0	187.30
tgt	C	2	0.78
tgc	C	0	1.22
ctt	L	2	0.42
ctc	L	0	0.29
cta	L	0	0.31
ctg	L	0	0.34
cct	P	2	0.67
ccc	P	0	0.37
cca	P	0	0.68
ccg	P	0	0.28
cat	H	1	0.56
cac	H	0	0.44
cgt	R	2	0.04
cgc	R	0	0.07
cga	R	0	0.18
cgg	R	0	0.17
att	I	1	0.35
atc	I	0	0.26
ata	I	0	0.39
act	T	1	0.31
acc	T	0	0.18
aca	T	0	0.43
acg	T	0	0.08
aat	N	54	30.21
aac	N	0	23.79
agt	S	11	11168.00
agc	S	0	13184.00
aga	R	0	0.98
agg	R	0	0.55
gct	A	15	4.63
gcc	A	0	4.25
gca	A	2	6.88
gcg	A	0	1.24
ggt	G	1	0.13
ggc	G	0	0.14
gga	G	0	0.46
ggg	G	0	0.26
---	--	------	------
mPD		0.060	1.7
		nPD:	0.04
		N. weight:	2.
		Sc. PD:	-0.088
		Sc. rank:	17567.0

,

HA

Pos.	169	obs:	exp:
tct	S	10036	1623.00
tcc	S	12	1503.00
tca	S	4	2703.00
tcg	S	6	395.90
act	T	19	6.21
acc	T	1	3.54
aca	T	0	8.66
acg	T	0	1.60
agt	S	0	1758.00
agc	S	0	2075.00
gtt	V	1	0.25
gtc	V	0	0.20
gta	V	0	0.22
gtg	V	0	0.33
gct	A	43845	15180.00
gcc	A	199	13915.00
gca	A	227	22529.00
gcg	A	11424	4071.00
---	--	------	------
mPD		0.56	1.1
		nPD:	0.52
		N. weight:	1.3
		Sc. PD:	0.52
		Sc. rank:	61068.0

,

HA

Pos.	170	obs:	exp:
tgt	C	7289	25704.00
tgc	C	58485	40070.00
---	--	------	------
mPD		0.20	0.48
		nPD:	0.41
		N. weight:	0.22
		Sc. PD:	0.068
		Sc. rank:	10078.0

,

HA

Pos.	171	obs:	exp:
tta	L	9	5.80
ttg	L	1	10.34
ctt	L	0	10.46
ctc	L	0	7.33
cta	L	40	7.68
ctg	L	0	8.39
cct	P	2	0.67
ccc	P	0	0.37
cca	P	0	0.68
ccg	P	0	0.28
caa	Q	2	1.22
cag	Q	0	0.78
cgt	R	0	4.32
cgc	R	0	7.84
cga	R	0	19.89
cgg	R	0	18.72
att	I	186	21882.00
atc	I	9	16379.00
ata	I	62139	24073.00
atg	M	1244	1244.00
act	T	0	30.12
acc	T	0	17.15
aca	T	97	41.98
acg	T	0	7.75
aaa	K	1776	1168.00
aag	K	0	608.40
aga	R	218	106.90
agg	R	0	60.30
gtt	V	0	11.27
gtc	V	0	9.17
gta	V	45	9.75
gtg	V	0	14.81
gaa	E	6	3.54
gag	E	0	2.46
---	--	------	------
mPD		0.11	0.74
		nPD:	0.15
		N. weight:	0.99
		Sc. PD:	0.059
		Sc. rank:	34492.0

,

HA

Pos.	172	obs:	exp:
tct	S	0	0.16
tcc	S	0	0.15
tca	S	0	0.27
tcg	S	0	0.04
cat	H	0	0.56
cac	H	1	0.44
cgt	R	1	1303.00
cgc	R	0	2365.00
cga	R	0	5998.00
cgg	R	7	5645.00
act	T	0	0.31
acc	T	0	0.18
aca	T	0	0.43
acg	T	1	0.08
aaa	K	0	21.70
aag	K	33	11.30
agt	S	1	0.17
agc	S	0	0.21
aga	R	349	32242.00
agg	R	65379	18183.00
ggt	G	0	0.27
ggc	G	0	0.28
gga	G	0	0.92
ggg	G	2	0.53
---	--	------	------
mPD		0.012	0.89
		nPD:	0.01
		N. weight:	1.4
		Sc. PD:	-0.091
		Sc. rank:	-28270.0

,

HA

Pos.	173	obs:	exp:
tct	S	0	4.20
tcc	S	0	3.89
tca	S	0	6.99
tcg	S	0	1.02
cgt	R	0	426.80
cgc	R	0	774.60
cga	R	1	1964.00
cgg	R	0	1849.00
aat	N	4	2.24
aac	N	0	1.76
aaa	K	8222	5410.00
aag	K	7	2819.00
agt	S	20	4.54
agc	S	6	5.36
aga	R	21445	10559.00
agg	R	82	5955.00
gct	A	2	0.55
gcc	A	0	0.50
gca	A	0	0.81
gcg	A	0	0.15
gat	D	2	1.13
gac	D	0	0.87
gaa	E	73	43.04
gag	E	0	29.96
ggt	G	1267	4834.00
ggc	G	6	5012.00
gga	G	34427	16577.00
ggg	G	210	9487.00
---	--	------	------
mPD		0.76	1.4
		nPD:	0.55
		N. weight:	0.69
		Sc. PD:	0.31
		Sc. rank:	34202.0

,

HA

Pos.	174	obs:	exp:
ttt	F	12	5.84
ttc	F	0	6.16
tct	S	65415	10596.00
tcc	S	220	9812.00
tca	S	24	17649.00
tcg	S	3	2585.00
tat	Y	14	7.38
tac	Y	0	6.62
cct	P	77	25.66
ccc	P	0	14.44
cca	P	0	26.01
ccg	P	0	10.89
act	T	6	1.86
acc	T	0	1.06
aca	T	0	2.60
acg	T	0	0.48
agt	S	0	11475.00
agc	S	0	13546.00
gct	A	1	0.27
gcc	A	0	0.25
gca	A	0	0.40
gcg	A	0	0.07
ggt	G	0	0.27
ggc	G	0	0.28
gga	G	2	0.92
ggg	G	0	0.53
---	--	------	------
mPD		0.011	1.6
		nPD:	0.01
		N. weight:	2.
		Sc. PD:	-0.14
		Sc. rank:	-72168.0

,

HA

Pos.	175	obs:	exp:
tct	S	0	6852.00
tcc	S	0	6345.00
tca	S	0	11413.00
tcg	S	0	1671.00
cat	H	5	2.82
cac	H	0	2.18
cgt	R	605	20.80
cgc	R	0	37.74
cga	R	0	95.71
cgg	R	0	90.08
att	I	55	19.66
atc	I	1	14.71
ata	I	0	21.63
atg	M	1	1.00
act	T	27	8.38
acc	T	0	4.77
aca	T	0	11.68
acg	T	0	2.16
aat	N	11982	6720.00
aac	N	29	5291.00
aaa	K	9221	6090.00
aag	K	43	3174.00
agt	S	42351	7420.00
agc	S	110	8760.00
aga	R	301	514.50
agg	R	143	290.20
gtt	V	360	90.17
gtc	V	0	73.35
gta	V	0	78.03
gtg	V	0	118.40
gct	A	0	0.55
gcc	A	0	0.50
gca	A	2	0.81
gcg	A	0	0.15
gat	D	388	222.10
gac	D	5	170.90
gaa	E	4	2.36
gag	E	0	1.64
ggt	G	141	18.98
ggc	G	0	19.68
gga	G	0	65.09
ggg	G	0	37.25
---	--	------	------
mPD		0.75	1.9
		nPD:	0.4
		N. weight:	1.5
		Sc. PD:	0.44
		Sc. rank:	66652.0

,

HA

Pos.	176	obs:	exp:
tct	S	0	9703.00
tcc	S	0	8986.00
tca	S	0	16162.00
tcg	S	0	2367.00
tgt	C	0	0.39
tgc	C	1	0.61
caa	Q	1	0.61
cag	Q	0	0.39
cgt	R	6	0.48
cgc	R	0	0.86
cga	R	0	2.19
cgg	R	0	2.06
att	I	2	3.16
atc	I	7	2.36
ata	I	0	3.48
aat	N	3225	2846.00
aac	N	1862	2241.00
aaa	K	464	306.40
aag	K	2	159.60
agt	S	59487	10508.00
agc	S	644	12405.00
aga	R	14	11.77
agg	R	4	6.64
gat	D	0	0.57
gac	D	1	0.43
ggt	G	54	7.27
ggc	G	0	7.54
gga	G	0	24.93
ggg	G	0	14.27
---	--	------	------
mPD		0.24	1.7
		nPD:	0.14
		N. weight:	1.7
		Sc. PD:	0.095
		Sc. rank:	59006.0

,

HA

Pos.	177	obs:	exp:
ttt	F	1	0.49
ttc	F	0	0.51
tct	S	0	10592.00
tcc	S	0	9809.00
tca	S	0	17643.00
tcg	S	0	2584.00
tgt	C	1	0.39
tgc	C	0	0.61
cgt	R	1	0.02
cgc	R	0	0.04
cga	R	0	0.09
cgg	R	0	0.09
act	T	1	0.31
acc	T	0	0.18
aca	T	0	0.43
acg	T	0	0.08
aat	N	10	5.59
aac	N	0	4.41
agt	S	64918	11471.00
agc	S	722	13541.00
aga	R	0	0.49
agg	R	0	0.28
ggt	G	120	16.15
ggc	G	0	16.75
gga	G	0	55.39
ggg	G	0	31.70
---	--	------	------
mPD		0.026	1.6
		nPD:	0.02
		N. weight:	1.9
		Sc. PD:	-0.12
		Sc. rank:	-29195.0

,

HA

Pos.	178	obs:	exp:
ttt	F	11403	31994.00
ttc	F	54370	33779.00
tta	L	0	0.12
ttg	L	0	0.21
ctt	L	0	0.21
ctc	L	1	0.15
cta	L	0	0.15
ctg	L	0	0.17
---	--	------	------
mPD		0.29	0.50
		nPD:	0.57
		N. weight:	0.24
		Sc. PD:	0.11
		Sc. rank:	11850.0

,

HA

Pos.	179	obs:	exp:
ttt	F	65238	31993.00
ttc	F	533	33778.00
tta	L	1	0.12
ttg	L	0	0.21
tat	Y	0	1.05
tac	Y	2	0.95
ctt	L	0	0.21
ctc	L	0	0.15
cta	L	0	0.15
ctg	L	0	0.17
---	--	------	------
mPD		0.016	0.50
		nPD:	0.03
		N. weight:	0.74
		Sc. PD:	-0.036
		Sc. rank:	3203.7

,

HA

Pos.	180	obs:	exp:
tct	S	0	10610.00
tcc	S	0	9826.00
tca	S	0	17672.00
tcg	S	0	2588.00
cgt	R	1	0.04
cgc	R	0	0.07
cga	R	0	0.18
cgg	R	0	0.17
att	I	1	0.35
atc	I	0	0.26
ata	I	0	0.39
aat	N	11	6.15
aac	N	0	4.85
aaa	K	2	1.31
aag	K	0	0.69
agt	S	65643	11490.00
agc	S	107	13564.00
aga	R	1	0.98
agg	R	0	0.55
ggt	G	8	1.08
ggc	G	0	1.12
gga	G	0	3.69
ggg	G	0	2.11
---	--	------	------
mPD		0.0040	1.6
		nPD:	0.
		N. weight:	1.9
		Sc. PD:	-0.15
		Sc. rank:	-91666.0

,

HA

Pos.	181	obs:	exp:
tct	S	0	0.32
tcc	S	0	0.30
tca	S	0	0.54
tcg	S	0	0.08
cgt	R	0	1293.00
cgc	R	0	2346.00
cga	R	6	5948.00
cgg	R	0	5598.00
att	I	0	2.11
atc	I	0	1.58
ata	I	6	2.32
act	T	0	0.31
acc	T	0	0.18
aca	T	1	0.43
acg	T	0	0.08
aat	N	4	2.80
aac	N	1	2.20
aaa	K	570	374.70
aag	K	0	195.30
agt	S	2	0.35
agc	S	0	0.41
aga	R	64639	31974.00
agg	R	544	18031.00
ggt	G	0	0.13
ggc	G	0	0.14
gga	G	1	0.46
ggg	G	0	0.26
---	--	------	------
mPD		0.034	0.90
		nPD:	0.04
		N. weight:	0.74
		Sc. PD:	-0.032
		Sc. rank:	7533.4

,

HA

Pos.	182	obs:	exp:
tta	L	59190	7632.00
ttg	L	6268	13598.00
tct	S	1	0.32
tcc	S	0	0.30
tca	S	1	0.54
tcg	S	0	0.08
ctt	L	0	13759.00
ctc	L	0	9645.00
cta	L	193	10100.00
ctg	L	120	11038.00
att	I	0	0.35
atc	I	0	0.26
ata	I	1	0.39
agt	S	0	0.35
agc	S	0	0.41
---	--	------	------
mPD		0.18	1.2
		nPD:	0.16
		N. weight:	1.9
		Sc. PD:	0.14
		Sc. rank:	69034.0

,

HA			
Pos.	183	obs:	exp:
tat	Y	1	0.53
tac	Y	0	0.47
att	I	0	0.70
atc	I	0	0.53
ata	I	2	0.77
aat	N	65615	36794.00
aac	N	151	28972.00
aaa	K	5	3.29
aag	K	0	1.71
---	--	------	------
mPD		0.0049	0.49
		nPD:	0.01
		N. weight:	0.62
		Sc. PD:	-0.044
		Sc. rank:	-18653.0

,

HA			
Pos.	184	obs:	exp:
tct	S	0	0.16
tcc	S	0	0.15
tca	S	0	0.27
tcg	S	1	0.04
tgg	W	65771	65771.00
agt	S	0	0.17
agc	S	0	0.21
ggt	G	0	0.27
ggc	G	0	0.28
gga	G	0	0.92
ggg	G	2	0.53
---	--	------	------
mPD		0.000091	0.00017
		nPD:	0.55
		N. weight:	0.000099
		Sc. PD:	0.000044
		Sc. rank:	4.9

,

HA			
Pos.	185	obs:	exp:
tta	L	273	7632.00
ttg	L	65420	13598.00
ctt	L	0	13759.00
ctc	L	0	9645.00
cta	L	2	10100.00
ctg	L	78	11039.00
gtt	V	0	0.25
gtc	V	0	0.20
gta	V	0	0.22
gtg	V	1	0.33
---	--	------	------
mPD		0.011	1.2
		nPD:	0.01
		N. weight:	1.7
		Sc. PD:	-0.12
		Sc. rank:	-53263.0

,

HA			
Pos.	186	obs:	exp:
tat	Y	145	104.40
tac	Y	53	93.63
cat	H	2	192.30
cac	H	339	148.70
att	I	0	0.35
atc	I	1	0.26
ata	I	0	0.39
act	T	134	20258.00
acc	T	65083	11533.00
aca	T	14	28230.00
acg	T	0	5210.00
gtt	V	2	0.50
gtc	V	0	0.41
gta	V	0	0.43
gtg	V	0	0.66
gct	A	0	0.27
gcc	A	1	0.25
gca	A	0	0.40
gcg	A	0	0.07
---	--	------	------
mPD		0.042	0.71
		nPD:	0.06
		N. weight:	1.9
		Sc. PD:	-0.044
		Sc. rank:	37317.0

,

HA			
Pos.	187	obs:	exp:
tct	S	0	1515.00
tcc	S	0	1403.00
tca	S	0	2524.00
tcg	S	0	369.60
tat	Y	0	0.53
tac	Y	1	0.47
cat	H	1807	31416.00
cac	H	53891	24282.00
caa	Q	234	144.30
cag	Q	2	91.74
cgt	R	0	0.18
cgc	R	8	0.32
cga	R	0	0.82
cgg	R	0	0.77
aat	N	6	78.33
aac	N	134	61.67
aaa	K	194	127.50
aag	K	0	66.46
agt	S	11	1641.00
agc	S	9378	1937.00
aga	R	1	4.41
agg	R	0	2.49
gaa	E	106	63.08
gag	E	1	43.92
---	--	------	------
mPD		0.57	1.1
		nPD:	0.54
		N. weight:	0.89
		Sc. PD:	0.38
		Sc. rank:	43712.0

,

HA			
Pos.	188	obs:	exp:
ttt	F	1	1.46
ttc	F	2	1.54
tta	L	61070	7140.00
ttg	L	383	12722.00
tct	S	0	302.90
tcc	S	0	280.50
tca	S	1876	504.50
tcg	S	1	73.89
ctt	L	0	12872.00
ctc	L	0	9023.00
cta	L	79	9449.00
ctg	L	2	10327.00
cct	P	0	1.00
ccc	P	0	0.56
cca	P	3	1.01
ccg	P	0	0.42
att	I	0	806.00
atc	I	0	603.30
ata	I	2296	886.70
atg	M	33	33.00
act	T	0	0.62
acc	T	0	0.35
aca	T	2	0.87
acg	T	0	0.16
aaa	K	26	17.09
aag	K	0	8.91
agt	S	0	328.00
agc	S	0	387.20
---	--	------	------
mPD		0.14	1.3
		nPD:	0.11
		N. weight:	2.3
		Sc. PD:	0.057
		Sc. rank:	71684.0

,

HA			
Pos.	189	obs:	exp:
tct	S	0	4.36
tcc	S	0	4.03
tca	S	0	7.26
tcg	S	0	1.06
tat	Y	0	1.58
tac	Y	3	1.42
cat	H	1	13.54
cac	H	23	10.46
caa	Q	1	0.61
cag	Q	0	0.39
cgt	R	0	1.25
cgc	R	0	2.27
cga	R	0	5.75
cgg	R	0	5.41
act	T	2	0.93
acc	T	1	0.53
aca	T	0	1.30
acg	T	0	0.24
aat	N	904	35715.00
aac	N	62933	28122.00
aaa	K	1491	981.60
aag	K	2	511.40
agt	S	0	4.72
agc	S	27	5.57
aga	R	63	30.90
agg	R	0	17.43
gat	D	36	36.18
gac	D	28	27.82
gaa	E	192	113.20
gag	E	0	78.80
ggt	G	0	9.02
ggc	G	0	9.35
gga	G	67	30.93
ggg	G	0	17.70
---	--	------	------
mPD		0.097	0.54
		nPD:	0.18
		N. weight:	0.81
		Sc. PD:	0.075
		Sc. rank:	30554.0

,

HA			
Pos.	190	obs:	exp:
ttt	F	5	5265.00
ttc	F	10819	5559.00
tct	S	1	1249.00
tcc	S	7659	1157.00
tca	S	0	2080.00
tcg	S	0	304.70
tat	Y	45	19334.00
tac	Y	36635	17346.00
cat	H	0	2.26
cac	H	4	1.74
caa	Q	1	0.61
cag	Q	0	0.39
cgt	R	0	0.02
cgc	R	0	0.04
cga	R	0	0.09
cgg	R	0	0.09
aat	N	5	5887.00
aac	N	10518	4636.00
agt	S	0	1353.00
agc	S	80	1597.00
aga	R	1	0.49
agg	R	0	0.28
gat	D	0	0.57
gac	D	1	0.43
---	--	------	------
mPD		0.72	1.3
		nPD:	0.55
		N. weight:	0.96
		Sc. PD:	0.42
		Sc. rank:	47590.0

,

HA

Pos.	191	obs:	exp:
tta	L	3	0.35
ttg	L	0	0.62
tct	S	0	0.81
tcc	S	0	0.75
tca	S	5	1.34
tcg	S	0	0.20
ctt	L	0	0.63
ctc	L	0	0.44
cta	L	0	0.46
ctg	L	0	0.50
caa	Q	4	2.45
cag	Q	0	1.55
cgt	R	0	0.75
cgc	R	0	1.37
cga	R	0	3.47
cgg	R	0	3.26
att	I	0	3727.00
atc	I	1	2790.00
ata	I	10617	4101.00
atg	M	14	14.00
act	T	6	10934.00
acc	T	6	6225.00
aca	T	35163	15237.00
acg	T	32	2812.00
aat	N	1	1.12
aac	N	1	0.88
aaa	K	19403	13010.00
aag	K	386	6779.00
agt	S	0	0.87
agc	S	0	1.03
aga	R	38	18.64
agg	R	0	10.51
gtt	V	0	2.50
gtc	V	0	2.04
gta	V	10	2.17
gtg	V	0	3.29
gct	A	0	22.62
gcc	A	0	20.74
gca	A	82	33.57
gcg	A	1	6.07
gaa	E	1	0.59
gag	E	0	0.41
---	--	------	------
mPD		0.61	1.3
		nPD:	0.48
		N. weight:	0.77
		Sc. PD:	0.29
		Sc. rank:	37397.0

,

HA

Pos.	192	obs:	exp:
ttt	F	0	2.43
ttc	F	5	2.57
tct	S	1	0.16
tcc	S	0	0.15
tca	S	0	0.27
tcg	S	0	0.04
tat	Y	38105	34640.00
tac	Y	27612	31077.00
cat	H	48	28.77
cac	H	3	22.23
agt	S	0	0.17
agc	S	0	0.21
---	--	------	------
mPD		0.49	0.50
		nPD:	0.98
		N. weight:	0.0067
		Sc. PD:	0.0057
		Sc. rank:	345.1

,

HA

Pos.	193	obs:	exp:
tta	L	0	0.46
ttg	L	0	0.83
tct	S	0	18.56
tcc	S	0	17.19
tca	S	115	30.91
tcg	S	0	4.53
ctt	L	0	0.84
ctc	L	0	0.59
cta	L	4	0.61
ctg	L	0	0.67
cct	P	28	21863.00
ccc	P	30	12300.00
cca	P	65515	22166.00
ccg	P	35	9279.00
caa	Q	35	21.39
cag	Q	0	13.61
act	T	0	3.73
acc	T	0	2.12
aca	T	12	5.19
acg	T	0	0.96
agt	S	0	20.10
agc	S	0	23.72
---	--	------	------
mPD		0.0079	0.73
		nPD:	0.01
		N. weight:	1.2
		Sc. PD:	-0.082
		Sc. rank:	-32861.0

,

HA

Pos.	194	obs:	exp:
tct	S	0	6.78
tcc	S	0	6.28
tca	S	42	11.29
tcg	S	0	1.65
act	T	0	4.35
acc	T	0	2.48
aca	T	14	6.06
acg	T	0	1.12
aaa	K	0	1.31
aag	K	2	0.69
agt	S	0	7.34
agc	S	0	8.66
gtt	V	0	20.04
gtc	V	0	16.30
gta	V	5	17.34
gtg	V	75	26.32
gct	A	19	17851.00
gcc	A	3	16363.00
gca	A	65256	26493.00
gcg	A	216	4788.00
gaa	E	128	83.13
gag	E	13	57.87
ggt	G	0	0.13
ggc	G	1	0.14
gga	G	0	0.46
ggg	G	0	0.26
---	--	------	------
mPD		0.019	0.70
		nPD:	0.03
		N. weight:	0.98
		Sc. PD:	-0.053
		Sc. rank:	-580.2

,

HA

Pos.	195	obs:	exp:
ttt	F	1	0.49
ttc	F	0	0.51
tta	L	10625	6392.00
ttg	L	30717	11389.00
ctt	L	8	11523.00
ctc	L	2	8078.00
cta	L	98	8459.00
ctg	L	13635	9245.00
caa	Q	95	6531.00
cag	Q	10589	4153.00
atg	M	3	3.00
gtt	V	0	0.25
gtc	V	0	0.20
gta	V	0	0.22
gtg	V	1	0.33
---	--	------	------
mPD		1.0	1.4
		nPD:	0.74
		N. weight:	0.84
		Sc. PD:	0.53
		Sc. rank:	43136.0

,

HA

Pos.	196	obs:	exp:
tct	S	0	1.29
tcc	S	0	1.20
tca	S	0	2.15
tcg	S	0	0.31
tat	Y	0	0.53
tac	Y	1	0.47
cat	H	0	0.56
cac	H	1	0.44
act	T	0	0.93
acc	T	3	0.53
aca	T	0	1.30
acg	T	0	0.24
aat	N	1417	36746.00
aac	N	64263	28934.00
aaa	K	17	11.18
aag	K	0	5.82
agt	S	0	1.40
agc	S	8	1.65
gat	D	0	3.39
gac	D	6	2.61
gaa	E	58	34.19
gag	E	0	23.81
---	--	------	------
mPD		0.047	0.50
		nPD:	0.09
		N. weight:	0.78
		Sc. PD:	0.0081
		Sc. rank:	21511.0

,

HA

Pos.	197	obs:	exp:
tta	L	0	0.23
ttg	L	1	0.41
ctt	L	0	0.42
ctc	L	0	0.29
cta	L	0	0.31
ctg	L	1	0.34
caa	Q	1	0.61
cag	Q	0	0.39
att	I	0	0.35
atc	I	0	0.26
ata	I	1	0.39
atg	M	29	29.00
aaa	K	1	1.31
aag	K	1	0.69
gtt	V	42	16463.00
gtc	V	1	13392.00
gta	V	532	14245.00
gtg	V	65150	21625.00
gct	A	0	2.18
gcc	A	0	2.00
gca	A	0	3.24
gcg	A	8	0.58
gaa	E	0	3.54
gag	E	6	2.46
---	--	------	------
mPD		0.019	0.74
		nPD:	0.03
		N. weight:	1.2
		Sc. PD:	-0.064
		Sc. rank:	-1571.1

,

HA

Pos.	198	obs:	exp:
ttt	F	1	0.49
ttc	F	0	0.51
tct	S	8	40.99
tcc	S	2	37.96
tca	S	0	68.27
tcg	S	0	10.00
att	I	8	2.81
atc	I	0	2.10
ata	I	0	3.09
act	T	65023	20340.00
acc	T	449	11580.00
aca	T	6	28345.00
acg	T	18	5231.00
aat	N	1	0.56
aac	N	0	0.44
agt	S	243	44.39
agc	S	1	52.40
gct	A	13	3.82
gcc	A	1	3.50
gca	A	0	5.66
gcg	A	0	1.02
---	--	------	------
mPD		0.023	0.69
		nPD:	0.03
		N. weight:	1.2
		Sc. PD:	-0.06
		Sc. rank:	6208.5

,

HA

Pos.	199	obs:	exp:
tta	L	0	0.35
ttg	L	3	0.62
tat	Y	0	1.05
tac	Y	2	0.95
ctt	L	0	0.63
ctc	L	0	0.44
cta	L	0	0.46
ctg	L	0	0.50
cgt	R	0	0.02
cgc	R	0	0.04
cga	R	0	0.09
cgg	R	0	0.09
att	I	3	22.47
atc	I	5	16.82
ata	I	56	24.72
atg	M	65458	65458.00
act	T	0	0.31
acc	T	0	0.18
aca	T	0	0.43
acg	T	1	0.08
aaa	K	0	1.31
aag	K	2	0.69
aga	R	0	0.49
agg	R	1	0.28
gtt	V	0	60.87
gtc	V	0	49.51
gta	V	0	52.67
gtg	V	243	79.95
mPD		0.0097	0.015
		nPD:	0.66
		N. weight:	0.0054
		Sc. PD:	0.0029
		Sc. rank:	270.5

,

HA

Pos.	200	obs:	exp:
tta	L	0	3.95
ttg	L	0	7.03
tct	S	0	7.91
tcc	S	0	7.32
tca	S	49	13.17
tcg	S	0	1.93
ctt	L	1	7.11
ctc	L	0	4.99
cta	L	33	5.22
ctg	L	0	5.71
cct	P	112	21886.00
ccc	P	195	12312.00
cca	P	65176	22189.00
ccg	P	193	9289.00
caa	Q	10	6.11
cag	Q	0	3.89
att	I	2	0.70
atc	I	0	0.53
ata	I	0	0.77
act	T	0	0.93
acc	T	0	0.53
aca	T	3	1.30
acg	T	0	0.24
agt	S	0	8.56
agc	S	0	10.11
mPD		0.018	0.72
		nPD:	0.03
		N. weight:	1.1
		Sc. PD:	-0.063
		Sc. rank:	-1868.6

,

HA

Pos.	201	obs:	exp:
act	T	0	0.62
acc	T	2	0.35
aca	T	0	0.87
acg	T	0	0.16
aat	N	51	36797.00
aac	N	65721	28975.00
mPD		0.0016	0.49
		nPD:	0.
		N. weight:	0.9
		Sc. PD:	-0.068
		Sc. rank:	-41839.0

,

HA

Pos.	202	obs:	exp:
tct	S	0	2.58
tcc	S	0	2.39
tca	S	0	4.30
tcg	S	0	0.63
cat	H	1	0.56
cac	H	0	0.44
caa	Q	0	0.61
cag	Q	1	0.39
cgt	R	0	1.33
cgc	R	0	2.41
cga	R	0	6.11
cgg	R	0	5.75
att	I	1	0.35
atc	I	0	0.26
ata	I	0	0.39
atg	M	2	2.00
act	T	3	2.17
acc	T	0	1.24
aca	T	0	3.03
acg	T	4	0.56
aat	N	35218	19722.00
aac	N	33	15529.00
aaa	K	759	19953.00
aag	K	29590	10396.00
agt	S	16	2.80
agc	S	0	3.30
aga	R	0	32.86
agg	R	67	18.53
gat	D	77	43.53
gac	D	0	33.47
gaa	E	0	1.18
gag	E	2	0.82
mPD		0.51	0.74
		nPD:	0.69
		N. weight:	0.82
		Sc. PD:	0.48
		Sc. rank:	41741.0

,

HA

Pos.	203	obs:	exp:
aaa	K	14	9.20
aag	K	0	4.80
gct	A	0	0.27
gcc	A	0	0.25
gca	A	1	0.40
gcg	A	0	0.07
gat	D	1	184.80
gac	D	326	142.20
gaa	E	64803	38439.00
gag	E	396	26760.00
ggt	G	0	31.37
ggc	G	150	32.52
gga	G	83	107.60
ggg	G	0	61.56
mPD		0.034	0.50
		nPD:	0.07
		N. weight:	0.55
		Sc. PD:	-0.008
		Sc. rank:	11917.0

,

HA

Pos.	204	obs:	exp:
tta	L	0	0.81
ttg	L	0	1.45
ctt	L	0	1.46
ctc	L	0	1.03
cta	L	7	1.07
ctg	L	0	1.17
cct	P	0	2.33
ccc	P	0	1.31
cca	P	6	2.36
ccg	P	1	0.99
cat	H	51	32.15
cac	H	6	24.85
caa	Q	64159	39270.00
cag	Q	83	24972.00
cgt	R	0	0.54
cgc	R	0	0.97
cga	R	24	2.46
cgg	R	0	2.32
act	T	0	0.31
acc	T	0	0.18
aca	T	1	0.43
acg	T	0	0.08
aat	N	158	94.55
aac	N	11	74.45
aaa	K	1098	726.50
aag	K	7	378.50
aga	R	3	13.24
agg	R	0	7.47
gaa	E	156	91.97
gag	E	0	64.03
ggt	G	1	0.40
ggc	G	0	0.42
gga	G	0	1.38
ggg	G	2	0.79
mPD		0.054	0.52
		nPD:	0.1
		N. weight:	0.53
		Sc. PD:	0.01
		Sc. rank:	15832.0

,

HA

Pos.	205	obs:	exp:
ttt	F	65648	31981.00
ttc	F	100	33767.00
tta	L	2	0.35
ttg	L	0	0.62
tct	S	16	2.58
tcc	S	0	2.39
tca	S	0	4.30
tcg	S	0	0.63
tat	Y	2	1.05
tac	Y	0	0.95
ctt	L	1	0.63
ctc	L	0	0.44
cta	L	0	0.46
ctg	L	0	0.50
aaa	K	2	1.31
aag	K	0	0.69
agt	S	0	2.80
agc	S	0	3.30
gtt	V	3	0.75
gtc	V	0	0.61
gta	V	0	0.65
gtg	V	0	0.99
mPD		0.0039	0.50
		nPD:	0.01
		N. weight:	0.78
		Sc. PD:	-0.056
		Sc. rank:	-26491.0

,

HA

Pos.	206	obs:	exp:
tat	Y	0	0.53
tac	Y	1	0.47
aat	N	0	10.63
aac	N	19	8.37
gat	D	111	37159.00
gac	D	65626	28578.00
gaa	E	14	8.84
gag	E	1	6.16
ggt	G	0	0.27
ggc	G	2	0.28
gga	G	0	0.92
ggg	G	0	0.53
mPD		0.0045	0.49
		nPD:	0.01
		N. weight:	0.91
		Sc. PD:	-0.064
		Sc. rank:	-28777.0

,

HA

Pos.	207	obs:	exp:
cgt	R	0	0.04
cgc	R	0	0.07
cga	R	0	0.18
cgg	R	0	0.17
aaa	K	65627	43240.00
aag	K	143	22530.00
aga	R	2	0.98
agg	R	0	0.55
gtt	V	0	0.50
gtc	V	2	0.41
gta	V	0	0.43
gtg	V	0	0.66
---	--	------	------
mPD		0.0046	0.45
		nPD:	0.01
		N. weight:	0.45
		Sc. PD:	-0.031
		Sc. rank:	-13128.0

,

HA

Pos.	208	obs:	exp:
ttt	F	1	0.49
ttc	F	0	0.51
tta	L	294	7632.00
ttg	L	65287	13597.00
ctt	L	0	13758.00
ctc	L	2	9644.00
cta	L	66	10099.00
ctg	L	117	11037.00
atg	M	7	7.00
---	--	------	------
mPD		0.017	1.2
		nPD:	0.01
		N. weight:	1.7
		Sc. PD:	-0.11
		Sc. rank:	-31626.0

,

HA

Pos.	209	obs:	exp:
tat	Y	39	34668.00
tac	Y	65731	31102.00
cat	H	0	1.13
cac	H	2	0.87
gtt	V	0	0.50
gtc	V	0	0.41
gta	V	0	0.43
gtg	V	2	0.66
---	--	------	------
mPD		0.0014	0.50
		nPD:	0.
		N. weight:	0.82
		Sc. PD:	-0.063
		Sc. rank:	-38597.0

,

HA

Pos.	210	obs:	exp:
tta	L	0	0.70
ttg	L	0	1.24
ctt	L	4	1.26
ctc	L	0	0.88
cta	L	0	0.92
ctg	L	2	1.01
att	I	65488	23066.00
atc	I	212	17265.00
ata	I	6	25375.00
gtt	V	62	15.53
gtc	V	0	12.63
gta	V	0	13.44
gtg	V	0	20.40
---	--	------	------
mPD		0.0087	0.66
		nPD:	0.01
		N. weight:	1.1
		Sc. PD:	-0.076
		Sc. rank:	-24126.0

,

HA

Pos.	211	obs:	exp:
tta	L	0	0.12
ttg	L	1	0.21
tgt	C	0	0.39
tgc	C	1	0.61
tgg	W	65772	65772.00
ctt	L	0	0.21
ctc	L	0	0.15
cta	L	0	0.15
ctg	L	0	0.17
---	--	------	------
mPD		0.000061	0.00010
		nPD:	0.61
		N. weight:	0.000035
		Sc. PD:	0.000017
		Sc. rank:	1.7

,

HA

Pos.	212	obs:	exp:
ggt	G	5684	8855.00
ggc	G	3	9180.00
gga	G	495	30362.00
ggg	G	59592	17377.00
---	--	------	------
mPD		0.17	0.68
		nPD:	0.25
		N. weight:	1.2
		Sc. PD:	0.19
		Sc. rank:	47469.0

,

HA

Pos.	213	obs:	exp:
ttt	F	2	0.97
ttc	F	0	1.03
att	I	146	54.06
atc	I	8	40.47
ata	I	0	59.47
gtt	V	64147	16434.00
gtc	V	1422	13368.00
gta	V	19	14220.00
gtg	V	21	21587.00
gct	A	8	2.18
gcc	A	0	2.00
gca	A	0	3.24
gcg	A	0	0.58
ggt	G	1	0.13
ggc	G	0	0.14
gga	G	0	0.46
ggg	G	0	0.26
---	--	------	------
mPD		0.049	0.75
		nPD:	0.07
		N. weight:	1.4
		Sc. PD:	-0.024
		Sc. rank:	30532.0

,

HA

Pos.	214	obs:	exp:
ttt	F	0	0.49
ttc	F	1	0.51
tta	L	0	3.48
ttg	L	0	6.20
tat	Y	0	0.53
tac	Y	1	0.47
ctt	L	0	6.28
ctc	L	30	4.40
cta	L	0	4.61
ctg	L	0	5.03
cat	H	703	37079.00
cac	H	65036	28660.00
caa	Q	1	0.61
cag	Q	0	0.39
cgt	R	0	0.04
cgc	R	2	0.07
cga	R	0	0.18
cgg	R	0	0.17
aga	R	0	0.98
agg	R	0	0.55
---	--	------	------
mPD		0.022	0.49
		nPD:	0.05
		N. weight:	0.85
		Sc. PD:	-0.031
		Sc. rank:	12385.0

,

HA

Pos.	215	obs:	exp:
tta	L	0	0.12
ttg	L	0	0.21
tat	Y	0	0.53
tac	Y	1	0.47
ctt	L	0	0.21
ctc	L	1	0.15
cta	L	0	0.15
ctg	L	0	0.17
cct	P	0	0.33
ccc	P	1	0.19
cca	P	0	0.34
ccg	P	0	0.14
cat	H	481	37094.00
cac	H	65284	28671.00
caa	Q	1	0.61
cag	Q	0	0.39
aat	N	0	2.80
aac	N	5	2.20
---	--	------	------
mPD		0.015	0.49
		nPD:	0.03
		N. weight:	0.87
		Sc. PD:	-0.044
		Sc. rank:	2911.0

,

HA

Pos.	216	obs:	exp:
tta	L	0	0.23
ttg	L	0	0.41
ctt	L	0	0.42
ctc	L	0	0.29
cta	L	0	0.31
ctg	L	2	0.34
cct	P	93	21917.00
ccc	P	3	12330.00
cca	P	620	22221.00
ccg	P	65055	9302.00
cgt	R	0	0.02
cgc	R	0	0.04
cga	R	0	0.09
cgg	R	1	0.09
aga	R	0	0.49
agg	R	0	0.28
---	--	------	------
mPD		0.022	0.72
		nPD:	0.03
		N. weight:	2.1
		Sc. PD:	-0.11
		Sc. rank:	7728.2

,

HA

Pos.	217	obs:	exp:
tct	S	1	103.00
tcc	S	0	95.34
tca	S	0	171.50
tcg	S	0	25.11
cct	P	1	0.33
ccc	P	0	0.19
cca	P	0	0.34
ccg	P	0	0.14
cgt	R	6	0.20
cgc	R	0	0.36
cga	R	0	0.91
cgg	R	0	0.86
att	I	5	2.46
atc	I	2	1.84
ata	I	0	2.70
aat	N	12	6.71
aac	N	0	5.29
agt	S	389	111.50
agc	S	248	131.60
aga	R	2	4.90
agg	R	2	2.77
gtt	V	69	17.53
gtc	V	1	14.26
gta	V	0	15.17
gtg	V	0	23.03
gct	A	61	17.72
gcc	A	4	16.24
gca	A	0	26.29
gcg	A	0	4.75
gat	D	11170	6349.00
gac	D	62	4883.00
gaa	E	0	0.59
gag	E	1	0.41
ggt	G	53208	7234.00
ggc	G	346	7500.00
gga	G	176	24806.00
ggg	G	8	14197.00
---	--	------	------
mPD		0.33	1.0
		nPD:	0.32
		N. weight:	1.9
		Sc. PD:	0.42
		Sc. rank:	80636.0

,

HA

Pos.	218	obs:	exp:
tct	S	0	1.45
tcc	S	0	1.34
tca	S	0	2.42
tcg	S	9	0.35
att	I	0	0.35
atc	I	1	0.26
ata	I	0	0.39
atg	M	8	8.00
act	T	193	20410.00
acc	T	0	11620.00
aca	T	1064	28443.00
acg	T	64465	5249.00
aaa	K	0	1.31
aag	K	2	0.69
agt	S	0	1.57
agc	S	0	1.86
gct	A	0	8.72
gcc	A	0	7.99
gca	A	0	12.94
gcg	A	32	2.34
---	--	------	------
mPD		0.039	0.68
		nPD:	0.06
		N. weight:	2.6
		Sc. PD:	-0.065
		Sc. rank:	51514.0

,

HA			
Pos.	219	obs:	exp:
tat	Y	0	13.70
tac	Y	26	12.30
act	T	2	0.62
acc	T	0	0.35
aca	T	0	0.87
acg	T	0	0.16
aat	N	0	69.93
aac	N	125	55.07
gct	A	0	1.36
gcc	A	5	1.25
gca	A	0	2.02
gcg	A	0	0.37
gat	D	91	37044.00
gac	D	65443	28490.00
gaa	E	50	30.07
gag	E	1	20.93
ggt	G	0	4.17
ggc	G	31	4.33
gga	G	0	14.31
ggg	G	0	8.19
---	--	------	------
mPD		0.010	0.50
		nPD:	0.02
		N. weight:	0.91
		Sc. PD:	-0.054
		Sc. rank:	-6563.1

,

HA			
Pos.	220	obs:	exp:
tct	S	0	56.32
tcc	S	0	52.15
tca	S	0	93.80
tcg	S	0	13.74
caa	Q	53	32.40
cag	Q	0	20.60
cgt	R	1	2.97
cgc	R	0	5.40
cga	R	0	13.69
cgg	R	0	12.88
atg	M	1	1.00
act	T	0	0.62
acc	T	0	0.35
aca	T	1	0.87
acg	T	1	0.16
aat	N	1147	642.30
aac	N	1	505.70
aaa	K	1175	42121.00
aag	K	62894	21948.00
agt	S	349	60.99
agc	S	0	72.00
aga	R	100	73.57
agg	R	49	41.49
gct	A	2	0.55
gcc	A	0	0.50
gca	A	0	0.81
gcg	A	0	0.15
---	--	------	------
mPD		0.10	0.50
		nPD:	0.2
		N. weight:	1.1
		Sc. PD:	0.12
		Sc. rank:	41486.0

,

HA			
Pos.	221	obs:	exp:
tct	S	0	1.45
tcc	S	0	1.34
tca	S	0	2.42
tcg	S	0	0.35
att	I	0	0.35
atc	I	1	0.26
ata	I	0	0.39
act	T	0	2.17
acc	T	7	1.24
aca	T	0	3.03
acg	T	0	0.56
aat	N	15	6278.00
aac	N	11207	4944.00
agt	S	0	1.57
agc	S	9	1.86
gtt	V	0	1.00
gtc	V	4	0.82
gta	V	0	0.87
gtg	V	0	1.32
gct	A	0	0.55
gcc	A	2	0.50
gca	A	0	0.81
gcg	A	0	0.15
gat	D	139	30717.00
gac	D	54202	23624.00
gaa	E	184	108.50
gag	E	0	75.52
ggt	G	0	0.54
ggc	G	3	0.56
gga	G	1	1.85
ggg	G	0	1.06
---	--	------	------
mPD		0.29	0.78
		nPD:	0.38
		N. weight:	0.9
		Sc. PD:	0.25
		Sc. rank:	40292.0

,

HA			
Pos.	222	obs:	exp:
cct	P	0	0.33
ccc	P	0	0.19
cca	P	1	0.34
ccg	P	0	0.14
caa	Q	65717	40206.00
cag	Q	56	25567.00
---	--	------	------
mPD		0.0017	0.48
		nPD:	0.
		N. weight:	0.54
		Sc. PD:	-0.041
		Sc. rank:	-24623.0

,

HA			
Pos.	223	obs:	exp:
ttt	F	0	1361.00
ttc	F	2798	1437.00
tta	L	2	1.86
ttg	L	0	3.31
tct	S	0	1.13
tcc	S	0	1.05
tca	S	0	1.88
tcg	S	0	0.28
ctt	L	0	3.35
ctc	L	14	2.35
cta	L	0	2.46
ctg	L	0	2.69
caa	Q	2	1.22
cag	Q	0	0.78
att	I	148	21582.00
atc	I	61309	16154.00
ata	I	22	23743.00
act	T	0	391.30
acc	T	1260	222.80
aca	T	0	545.30
acg	T	0	100.60
aat	N	1	32.45
aac	N	57	25.55
aaa	K	1	0.66
aag	K	0	0.34
agt	S	0	1.22
agc	S	7	1.44
gtt	V	0	38.32
gtc	V	153	31.17
gta	V	0	33.16
gtg	V	0	50.34
---	--	------	------
mPD		0.13	0.79
		nPD:	0.17
		N. weight:	1.4
		Sc. PD:	0.11
		Sc. rank:	52081.0

,

HA			
Pos.	224	obs:	exp:
ttt	F	110	22163.00
ttc	F	45452	23399.00
tta	L	2	1.39
ttg	L	0	2.48
tct	S	43	3218.00
tcc	S	19241	2980.00
tca	S	21	5361.00
tcg	S	0	785.10
tat	Y	0	10.02
tac	Y	19	8.98
tgt	C	0	0.39
tgc	C	1	0.61
ctt	L	1	2.51
ctc	L	9	1.76
cta	L	0	1.84
ctg	L	0	2.01
cgt	R	0	0.08
cgc	R	0	0.14
cga	R	0	0.36
cgg	R	0	0.34
att	I	0	1.76
atc	I	5	1.31
ata	I	0	1.93
act	T	0	0.93
acc	T	3	0.53
aca	T	0	1.30
acg	T	0	0.24
aat	N	1	115.30
aac	N	205	90.75
aaa	K	2	1.31
aag	K	0	0.69
agt	S	4	3485.00
agc	S	635	4114.00
aga	R	3	1.96
agg	R	1	1.11
gct	A	0	0.82
gcc	A	3	0.75
gca	A	0	1.21
gcg	A	0	0.22
gat	D	0	5.65
gac	D	10	4.35
ggt	G	0	0.40
ggc	G	3	0.42
gga	G	0	1.38
ggg	G	0	0.79
---	--	------	------
mPD		0.47	1.3
		nPD:	0.37
		N. weight:	1.1
		Sc. PD:	0.29
		Sc. rank:	48197.0

,

HA			
Pos.	225	obs:	exp:
tta	L	3	7616.00
ttg	L	296	13570.00
ctt	L	12	13730.00
ctc	L	2	9625.00
cta	L	1089	10079.00
ctg	L	64233	11015.00
cct	P	1	34.99
ccc	P	0	19.68
cca	P	0	35.47
ccg	P	104	14.85
cgt	R	0	0.04
cgc	R	0	0.07
cga	R	0	0.18
cgg	R	2	0.17
att	I	0	10.18
atc	I	0	7.62
ata	I	29	11.20
atg	M	2	2.00
aga	R	0	0.98
agg	R	0	0.55
gtt	V	0	0.25
gtc	V	0	0.20
gta	V	1	0.22
gtg	V	0	0.33
---	--	------	------
mPD		0.047	1.2
		nPD:	0.04
		N. weight:	1.8
		Sc. PD:	-0.075
		Sc. rank:	22345.0

,

HA			
Pos.	226	obs:	exp:
ttt	F	10041	5213.00
ttc	F	676	5504.00
tat	Y	54871	29015.00
tac	Y	174	26030.00
cat	H	9	5.08
cac	H	0	3.92
aat	N	3	1.68
aac	N	0	1.32
---	--	------	------
mPD		0.30	0.77
		nPD:	0.39
		N. weight:	0.66
		Sc. PD:	0.19
		Sc. rank:	29746.0

,

HA			
Pos.	227	obs:	exp:
tct	S	12	2.42
tcc	S	0	2.24
tca	S	3	4.03
tcg	S	0	0.59
caa	Q	0	1.22
cag	Q	2	0.78
att	I	28	9.83
atc	I	0	7.36
ata	I	0	10.81
act	T	9	3.73
acc	T	1	2.12
aca	T	2	5.19
acg	T	0	0.96
agt	S	0	2.62
agc	S	0	3.09
gtt	V	266	104.20
gtc	V	146	84.76
gta	V	4	90.16
gtg	V	0	136.90
gct	A	57098	17798.00
gcc	A	491	16315.00
gca	A	7701	26414.00
gcg	A	10	4774.00
gat	D	1	0.57
gac	D	0	0.43
---	--	------	------
mPD		0.24	0.71
		nPD:	0.34
		N. weight:	0.94
		Sc. PD:	0.23
		Sc. rank:	41300.0

,

HA			
Pos.	228	obs:	exp:
tta	L	0	0.70
ttg	L	0	1.24
ctt	L	0	1.26
ctc	L	0	0.88
cta	L	6	0.92
ctg	L	0	1.01
cat	H	605	465.30
cac	H	220	359.70
caa	Q	60329	36946.00
cag	Q	111	23494.00
cgt	R	9	72.03
cgc	R	0	130.70
cga	R	3592	331.50
cgg	R	9	312.00
aat	N	2	1.12
aac	N	0	0.88
aaa	K	867	570.70
aag	K	1	297.30
aga	R	23	1782.00
agg	R	0	1005.00
---	--	------	------
mPD		0.16	0.70
		nPD:	0.23
		N. weight:	0.64
		Sc. PD:	0.088
		Sc. rank:	25479.0

,

HA			
Pos.	229	obs:	exp:
tta	L	13	1.51
ttg	L	0	2.69
tct	S	115	9309.00
tcc	S	8	8621.00
tca	S	57338	15505.00
tcg	S	225	2271.00
ctt	L	0	2.72
ctc	L	0	1.91
cta	L	0	2.00
ctg	L	0	2.18
cct	P	0	1006.00
ccc	P	0	566.20
cca	P	3016	1020.00
ccg	P	4	427.10
caa	Q	13	7.95
cag	Q	0	5.05
act	T	0	4.97
acc	T	0	2.83
aca	T	16	6.92
acg	T	0	1.28
agt	S	0	10081.00
agc	S	0	11900.00
gct	A	7	1369.00
gcc	A	0	1255.00
gca	A	4994	2031.00
gcg	A	21	367.10
gaa	E	2	1.18
gag	E	0	0.82
---	-	2	2.00
---	--	------	------
mPD		0.23	1.7
		nPD:	0.14
		N. weight:	1.4
		Sc. PD:	0.069
		Sc. rank:	46855.0

,

HA			
Pos.	230	obs:	exp:
tta	L	34	3.95
ttg	L	0	7.03
tct	S	14	10447.00
tcc	S	6	9675.00
tca	S	64657	17401.00
tcg	S	65	2548.00
ctt	L	0	7.11
ctc	L	0	4.99
cta	L	0	5.22
ctg	L	0	5.71
cct	P	0	74.65
ccc	P	0	41.99
cca	P	224	75.68
ccg	P	0	31.68
att	I	0	0.35
atc	I	0	0.26
ata	I	1	0.39
act	T	0	3.42
acc	T	0	1.94
aca	T	11	4.76
acg	T	0	0.88
agt	S	0	11314.00
agc	S	0	13356.00
gct	A	0	207.10
gcc	A	0	189.90
gca	A	760	307.40
gcg	A	0	55.56
gat	D	2	1.13
gac	D	0	0.87
---	--	------	------
mPD		0.034	1.6
		nPD:	0.02
		N. weight:	1.4
		Sc. PD:	-0.086
		Sc. rank:	-9591.2

,

HA			
Pos.	231	obs:	exp:
cgt	R	0	0.08
cgc	R	0	0.14
cga	R	0	0.36
cgg	R	0	0.34
aga	R	4	1.96
agg	R	0	1.11
gtt	V	0	0.25
gtc	V	0	0.20
gta	V	1	0.22
gtg	V	0	0.33
gct	A	0	0.82
gcc	A	0	0.75
gca	A	3	1.21
gcg	A	0	0.22
gaa	E	1	0.59
gag	E	0	0.41
ggt	G	6	8854.00
ggc	G	2	9179.00
gga	G	64991	30358.00
ggg	G	766	17374.00
---	--	------	------
mPD		0.024	0.68
		nPD:	0.03
		N. weight:	0.79
		Sc. PD:	-0.037
		Sc. rank:	4970.7

,

HA			
Pos.	232	obs:	exp:
tct	S	0	0.32
tcc	S	0	0.30
tca	S	0	0.54
tcg	S	0	0.08
tat	Y	2	1.05
tac	Y	0	0.95
cgt	R	0	1285.00
cgc	R	0	2333.00
cga	R	33	5915.00
cgg	R	0	5567.00
att	I	0	30.54
atc	I	0	22.86
ata	I	87	33.60
act	T	0	0.93
acc	T	0	0.53
aca	T	3	1.30
acg	T	0	0.24
aaa	K	838	550.90
aag	K	0	287.10
agt	S	1	0.35
agc	S	1	0.41
aga	R	64675	31798.00
agg	R	121	17932.00
gtt	V	0	0.25
gtc	V	0	0.20
gta	V	1	0.22
gtg	V	0	0.33
ggt	G	0	1.62
ggc	G	0	1.67
gga	G	12	5.54
ggg	G	0	3.17
---	--	------	------
mPD		0.033	0.91
		nPD:	0.04
		N. weight:	0.77
		Sc. PD:	-0.034
		Sc. rank:	6938.0

,

HA			
Pos.	233	obs:	exp:
att	I	58	22849.00
atc	I	64895	17103.00
ata	I	136	25137.00
act	T	0	4.04
acc	T	13	2.30
aca	T	0	5.63
acg	T	0	1.04
gtt	V	147	168.30
gtc	V	525	136.90
gta	V	0	145.70
gtg	V	0	221.10
---	--	------	------
mPD		0.031	0.68
		nPD:	0.05
		N. weight:	1.4
		Sc. PD:	-0.052
		Sc. rank:	21472.0

,

HA			
Pos.	234	obs:	exp:
ttt	F	2	0.97
ttc	F	0	1.03
tct	S	0	0.32
tcc	S	0	0.30
tca	S	2	0.54
tcg	S	0	0.08
cgt	R	0	0.02
cgc	R	0	0.04
cga	R	0	0.09
cgg	R	0	0.09
att	I	0	89.87
atc	I	0	67.27
ata	I	256	98.87
act	T	22	20343.00
acc	T	37	11582.00
aca	T	65319	28349.00
acg	T	128	5232.00
agt	S	0	0.35
agc	S	0	0.41
aga	R	1	0.49
agg	R	0	0.28
gct	A	1	1.91
gcc	A	0	1.75
gca	A	6	2.83
gcg	A	0	0.51
---	--	------	------
mPD		0.014	0.69
		nPD:	0.02
		N. weight:	0.91
		Sc. PD:	-0.054
		Sc. rank:	-7208.6

,

HA			
Pos.	235	obs:	exp:
att	I	0	23.52
atc	I	4	17.61
ata	I	63	25.88
gtt	V	32	16458.00
gtc	V	2300	13388.00
gta	V	62961	14241.00
gtg	V	414	21619.00
---	--	------	------
mPD		0.083	0.74
		nPD:	0.11
		N. weight:	1.5
		Sc. PD:	0.039
		Sc. rank:	47450.0

,

HA			
Pos.	236	obs:	exp:
ttt	F	359	174.60
ttc	F	0	184.40
tct	S	64790	10554.00
tcc	S	540	9774.00
tca	S	69	17580.00
tcg	S	7	2575.00
tat	Y	4	2.11
tac	Y	0	1.89
cct	P	1	0.33
ccc	P	0	0.19
cca	P	0	0.34
ccg	P	0	0.14
agt	S	0	11430.00
agc	S	0	13493.00
gct	A	2	0.55
gcc	A	0	0.50
gca	A	0	0.81
gcg	A	0	0.15
ggt	G	0	0.27
ggc	G	0	0.28
gga	G	0	0.92
ggg	G	2	0.53
---	--	------	------
mPD		0.030	1.6
		nPD:	0.02
		N. weight:	1.9
		Sc. PD:	-0.12
		Sc. rank:	-22469.0

,

HA			
Pos.	237	obs:	exp:
att	I	0	0.35
atc	I	1	0.26
ata	I	0	0.39
act	T	69	20426.00
acc	T	65654	11629.00
aca	T	49	28465.00
acg	T	0	5253.00
aat	N	0	0.56
aac	N	1	0.44
---	--	------	------
mPD		0.0036	0.68
		nPD:	0.01
		N. weight:	1.9
		Sc. PD:	-0.14
		Sc. rank:	-77517.0

,

HA			
Pos.	238	obs:	exp:
tct	S	0	0.32
tcc	S	0	0.30
tca	S	2	0.54
tcg	S	0	0.08
caa	Q	58	35.45
cag	Q	0	22.55
cgt	R	0	32.44
cgc	R	0	58.87
cga	R	0	149.30
cgg	R	0	140.50
att	I	0	1.05
atc	I	0	0.79
ata	I	3	1.16
act	T	0	0.62
acc	T	0	0.35
aca	T	2	0.87
acg	T	0	0.16
aat	N	1	2.80
aac	N	4	2.20
aaa	K	63937	42113.00
aag	K	120	21944.00
agt	S	0	0.35
agc	S	0	0.41
aga	R	1614	802.40
agg	R	22	452.50
gaa	E	10	5.90
gag	E	0	4.10
ggt	G	0	0.13
ggc	G	0	0.14
gga	G	1	0.46
ggg	G	0	0.26
---	--	------	------
mPD		0.055	0.52
		nPD:	0.11
		N. weight:	0.46
		Sc. PD:	0.011
		Sc. rank:	14221.0

,

HA			
Pos.	239	obs:	exp:
tct	S	0	0.65
tcc	S	0	0.60
tca	S	0	1.08
tcg	S	0	0.16
tgg	W	2	2.00
cgt	R	0	1297.00
cgc	R	0	2354.00
cga	R	7	5970.00
cgg	R	0	5618.00
att	I	0	16.15
atc	I	0	12.09
ata	I	46	17.77
act	T	0	0.31
acc	T	0	0.18
aca	T	1	0.43
acg	T	0	0.08
aat	N	2	1.12
aac	N	0	0.88
aaa	K	83	54.57
aag	K	0	28.43
agt	S	3	0.70
agc	S	1	0.83
aga	R	65101	32093.00
agg	R	323	18098.00
ggt	G	0	27.60
ggc	G	0	28.61
gga	G	205	94.63
ggg	G	0	54.16
---	--	------	------
mPD		0.021	0.89
		nPD:	0.02
		N. weight:	0.75
		Sc. PD:	-0.043
		Sc. rank:	-2836.9

,

HA			
Pos.	240	obs:	exp:
tct	S	0	10589.00
tcc	S	0	9806.00
tca	S	0	17638.00
tcg	S	0	2583.00
tat	Y	0	1.05
tac	Y	2	0.95
cgt	R	0	0.08
cgc	R	2	0.14
cga	R	0	0.36
cgg	R	0	0.34
att	I	0	1.05
atc	I	3	0.79
ata	I	0	1.16
act	T	0	0.62
acc	T	2	0.35
aca	T	0	0.87
acg	T	0	0.16
aat	N	0	57.63
aac	N	103	45.37
agt	S	173	11467.00
agc	S	65448	13537.00
aga	R	2	1.96
agg	R	0	1.11
ggt	G	0	5.25
ggc	G	39	5.44
gga	G	0	18.00
ggg	G	0	10.30
---	--	------	------
mPD		0.010	1.6
		nPD:	0.01
		N. weight:	1.7
		Sc. PD:	-0.13
		Sc. rank:	-66412.0

,

HA			
Pos.	241	obs:	exp:
tct	S	0	0.32
tcc	S	0	0.30
tca	S	0	0.54
tcg	S	0	0.08
cat	H	0	0.56
cac	H	1	0.44
caa	Q	65542	40204.00
cag	Q	228	25566.00
cgt	R	0	0.02
cgc	R	0	0.04
cga	R	0	0.09
cgg	R	1	0.09
agt	S	0	0.35
agc	S	2	0.41
aga	R	0	0.49
agg	R	0	0.28
---	--	------	------
mPD		0.0072	0.48
		nPD:	0.02
		N. weight:	0.52
		Sc. PD:	-0.034
		Sc. rank:	-8868.0

,

HA			
Pos.	242	obs:	exp:
cat	H	0	1.13
cac	H	2	0.87
caa	Q	65559	40200.00
cag	Q	205	25564.00
cgt	R	0	0.06
cgc	R	0	0.11
cga	R	3	0.27
cgg	R	0	0.26
aaa	K	3	3.29
aag	K	2	1.71
aga	R	0	1.47
agg	R	0	0.83
---	--	------	------
mPD		0.0066	0.48
		nPD:	0.01
		N. weight:	0.52
		Sc. PD:	-0.035
		Sc. rank:	-10360.0

,

HA			
Pos.	243	obs:	exp:
tct	S	220	35.66
tcc	S	0	33.03
tca	S	1	59.40
tcg	S	0	8.70
att	I	1	0.35
atc	I	0	0.26
ata	I	0	0.39
act	T	4972	1551.00
acc	T	20	882.80
aca	T	1	2161.00
acg	T	0	398.80
aaa	K	0	1.31
aag	K	2	0.69
agt	S	0	38.62
agc	S	0	45.59
gtt	V	1	0.25
gtc	V	0	0.20
gta	V	0	0.22
gtg	V	0	0.33
gct	A	60457	16505.00
gcc	A	44	15129.00
gca	A	28	24495.00
gcg	A	27	4427.00
---	--	------	------
mPD		0.15	0.84
		nPD:	0.18
		N. weight:	1.4
		Sc. PD:	0.13
		Sc. rank:	52066.0

,

HA			
Pos.	244	obs:	exp:
ttt	F	0	0.97
ttc	F	2	1.03
att	I	0	37.56
atc	I	0	28.12
ata	I	107	41.32
gtt	V	28	16366.00
gtc	V	7	13314.00
gta	V	64434	14162.00
gtg	V	872	21499.00
gct	A	0	87.76
gcc	A	0	80.45
gca	A	322	130.30
gcg	A	0	23.54
gaa	E	2	1.18
gag	E	0	0.82
---	--	------	------
mPD		0.040	0.75
		nPD:	0.05
		N. weight:	1.6
		Sc. PD:	-0.045
		Sc. rank:	28173.0

,

HA			
Pos.	245	obs:	exp:
ttt	F	0	1.46
ttc	F	3	1.54
tta	L	0	1.28
ttg	L	0	2.27
tct	S	0	3.07
tcc	S	0	2.84
tca	S	0	5.11
tcg	S	0	0.75
ctt	L	4	2.30
ctc	L	7	1.61
cta	L	0	1.69
ctg	L	0	1.85
att	I	1762	22718.00
atc	I	62913	17005.00
ata	I	41	24993.00
act	T	1	284.50
acc	T	915	162.00
aca	T	0	396.40
acg	T	0	73.16
aat	N	0	3.36
aac	N	6	2.64
aaa	K	0	1.31
aag	K	2	0.69
agt	S	0	3.32
agc	S	19	3.92
gtt	V	8	25.30
gtc	V	93	20.58
gta	V	0	21.89
gtg	V	0	33.23
---	--	------	------
mPD		0.086	0.69
		nPD:	0.12
		N. weight:	1.3
		Sc. PD:	0.051
		Sc. rank:	44530.0

,

HA			
Pos.	246	obs:	exp:
tta	L	0	0.12
ttg	L	0	0.21
tct	S	0	2.42
tcc	S	0	2.24
tca	S	10	4.03
tcg	S	5	0.59
ctt	L	0	0.21
ctc	L	0	0.15
cta	L	1	0.15
ctg	L	0	0.17
cct	P	69	21913.00
ccc	P	12	12328.00
cca	P	47552	22216.00
ccg	P	18124	9300.00
caa	Q	0	0.61
cag	Q	1	0.39
agt	S	0	2.62
agc	S	0	3.09
---	--	------	------
mPD		0.40	0.72
		nPD:	0.56
		N. weight:	0.8
		Sc. PD:	0.36
		Sc. rank:	40105.0

,

HA			
Pos.	247	obs:	exp:
tct	S	0	13.23
tcc	S	0	12.25
tca	S	0	22.04
tcg	S	0	3.23
tat	Y	3	1.58
tac	Y	0	1.42
cat	H	11	6.20
cac	H	0	4.80
att	I	2	0.70
atc	I	0	0.53
ata	I	0	0.77
act	T	9	3.73
acc	T	3	2.12
aca	T	0	5.19
acg	T	0	0.96
aat	N	65337	36729.00
aac	N	313	28921.00
agt	S	82	14.33
agc	S	0	16.92
gat	D	12	6.78
gac	D	0	5.22
gaa	E	2	1.18
gag	E	0	0.82
---	--	------	------
mPD		0.013	0.50
		nPD:	0.03
		N. weight:	0.61
		Sc. PD:	-0.033
		Sc. rank:	4.2

,

HA			
Pos.	248	obs:	exp:
ttt	F	0	0.49
ttc	F	1	0.51
tta	L	0	0.12
ttg	L	0	0.21
ctt	L	0	0.21
ctc	L	1	0.15
cta	L	0	0.15
ctg	L	0	0.17
att	I	656	23036.00
atc	I	64818	17243.00
ata	I	149	25343.00
act	T	0	5.28
acc	T	17	3.01
aca	T	0	7.36
acg	T	0	1.36
gtt	V	4	32.81
gtc	V	127	26.69
gta	V	0	28.39
gtg	V	0	43.10
gct	A	0	0.27
gcc	A	1	0.25
gca	A	0	0.40
gcg	A	0	0.07
---	--	------	------
mPD		0.029	0.66
		nPD:	0.04
		N. weight:	1.4
		Sc. PD:	-0.053
		Sc. rank:	19750.0

,

HA			
Pos.	249	obs:	exp:
tgg	W	4	4.00
cgt	R	0	0.30
cgc	R	0	0.54
cga	R	0	1.37
cgg	R	0	1.29
aga	R	15	7.36
agg	R	0	4.15
gct	A	0	0.55
gcc	A	0	0.50
gca	A	2	0.81
gcg	A	0	0.15
gaa	E	5	7.66
gag	E	8	5.34
ggt	G	24	8850.00
ggc	G	4	9175.00
gga	G	64997	30347.00
ggg	G	715	17368.00
---	--	------	------
mPD		0.024	0.68
		nPD:	0.03
		N. weight:	0.79
		Sc. PD:	-0.036
		Sc. rank:	5083.6

,

HA			
Pos.	250	obs:	exp:
ttt	F	3281	1668.00
ttc	F	149	1762.00
tct	S	53422	9840.00
tcc	S	7528	9112.00
tca	S	22	16389.00
tcg	S	5	2400.00
tat	Y	1327	711.10
tac	Y	22	637.90
tgt	C	1	0.39
tgc	C	0	0.61
cct	P	9	3.00
ccc	P	0	1.69
cca	P	0	3.04
ccg	P	0	1.27
att	I	1	0.70
atc	I	0	0.53
ata	I	1	0.77
act	T	2	1.86
acc	T	3	1.06
aca	T	1	2.60
acg	T	0	0.48
agt	S	0	10656.00
agc	S	0	12579.00
---	--	------	------
mPD		0.35	1.7
		nPD:	0.21
		N. weight:	1.5
		Sc. PD:	0.18
		Sc. rank:	60261.0

,

HA			
Pos.	251	obs:	exp:
tct	S	0	1.45
tcc	S	0	1.34
tca	S	0	2.42
tcg	S	0	0.35
cgt	R	0	1301.00
cgc	R	0	2362.00
cga	R	8	5989.00
cgg	R	0	5636.00
aaa	K	122	80.21
aag	K	0	41.79
agt	S	1	1.57
agc	S	8	1.86
aga	R	63035	32194.00
agg	R	2595	18155.00
ggt	G	0	0.67
ggc	G	0	0.70
gga	G	5	2.31
ggg	G	0	1.32
---	--	------	------
mPD		0.080	0.89
		nPD:	0.09
		N. weight:	0.63
		Sc. PD:	0.0041
		Sc. rank:	16713.0

,

HA			
Pos.	252	obs:	exp:
tta	L	0	13.81
ttg	L	0	24.60
tct	S	0	2.10
tcc	S	13	1.94
tca	S	0	3.49
tcg	S	0	0.51
ctt	L	0	24.89
ctc	L	119	17.45
cta	L	0	18.27
ctg	L	0	19.97
cct	P	63	21854.00
ccc	P	65491	12295.00
cca	P	26	22156.00
ccg	P	0	9275.00
cat	H	0	20.87
cac	H	37	16.13
act	T	0	6.21
acc	T	20	3.54
aca	T	0	8.66
acg	T	0	1.60
agt	S	0	2.27
agc	S	0	2.68
gct	A	0	1.36
gcc	A	5	1.25
gca	A	0	2.02
gcg	A	0	0.37
---	--	------	------
mPD		0.0086	0.73
		nPD:	0.01
		N. weight:	1.8
		Sc. PD:	-0.12
		Sc. rank:	-46682.0

,

HA			
Pos.	253	obs:	exp:
tgg	W	551	551.00
cgt	R	0	1292.00
cgc	R	0	2344.00
cga	R	151	5944.00
cgg	R	0	5594.00
att	I	0	0.70
atc	I	0	0.53
ata	I	2	0.77
aaa	K	72	47.99
aag	K	1	25.01
aga	R	64435	31954.00
agg	R	562	18020.00
---	--	------	------
mPD		0.057	0.90
		nPD:	0.06
		N. weight:	0.72
		Sc. PD:	-0.014
		Sc. rank:	14998.0

,

HA			
Pos.	254	obs:	exp:
tta	L	1	0.23
ttg	L	0	0.41
ctt	L	0	0.42
ctc	L	0	0.29
cta	L	1	0.31
ctg	L	0	0.34
att	I	2	21393.00
atc	I	408	16013.00
ata	I	60532	23536.00
atg	M	7	7.00
act	T	0	0.62
acc	T	0	0.35
aca	T	2	0.87
acg	T	0	0.16
gtt	V	3	1208.00
gtc	V	2	982.30
gta	V	4795	1045.00
gtg	V	21	1586.00
---	--	------	------
mPD		0.15	0.81
		nPD:	0.18
		N. weight:	1.1
		Sc. PD:	0.1
		Sc. rank:	40001.0

,

HA			
Pos.	255	obs:	exp:
cgt	R	0	1304.00
cgc	R	0	2366.00
cga	R	0	6001.00
cgg	R	19	5648.00
atg	M	1	1.00
aaa	K	0	0.66
aag	K	1	0.34
aga	R	258	32259.00
agg	R	65493	18192.00
ggt	G	0	0.27
ggc	G	0	0.28
gga	G	0	0.92
ggg	G	2	0.53
---	--	------	------
mPD		0.0085	0.89
		nPD:	0.01
		N. weight:	1.4
		Sc. PD:	-0.097
		Sc. rank:	-42243.0

,

HA			
Pos.	256	obs:	exp:
tct	S	0	0.16
tcc	S	0	0.15
tca	S	0	0.27
tcg	S	0	0.04
act	T	1	0.31
acc	T	0	0.18
aca	T	0	0.43
acg	T	0	0.08
aat	N	10305	5793.00
aac	N	50	4562.00
agt	S	1	0.17
agc	S	0	0.21
gat	D	53988	30984.00
gac	D	825	23829.00
gaa	E	0	1.18
gag	E	2	0.82
ggt	G	582	81.04
ggc	G	12	84.02
gga	G	1	277.90
ggg	G	7	159.00
---	--	------	------
mPD		0.31	0.78
		nPD:	0.4
		N. weight:	0.57
		Sc. PD:	0.17
		Sc. rank:	25991.0

,

HA			
Pos.	257	obs:	exp:
ttt	F	0	0.49
ttc	F	1	0.51
tta	L	1	25.41
ttg	L	0	45.28
tct	S	0	0.32
tcc	S	0	0.30
tca	S	0	0.54
tcg	S	0	0.08
ctt	L	0	45.81
ctc	L	1	32.11
cta	L	2	33.63
ctg	L	215	36.75
cct	P	0	0.33
ccc	P	0	0.19
cca	P	0	0.34
ccg	P	1	0.14
caa	Q	2	11.00
cag	Q	16	7.00
att	I	1180	22841.00
atc	I	63805	17097.00
ata	I	82	25129.00
atg	M	1	1.00
act	T	0	1.24
acc	T	4	0.71
aca	T	0	1.73
acg	T	0	0.32
aat	N	0	1.12
aac	N	2	0.88
agt	S	0	0.35
agc	S	2	0.41
gtt	V	3	114.70
gtc	V	335	93.32
gta	V	120	99.27
gtg	V	0	150.70
gct	A	0	0.27
gcc	A	1	0.25
gca	A	0	0.40
gcg	A	0	0.07
---	--	------	------
mPD		0.070	0.68
		nPD:	0.1
		N. weight:	1.4
		Sc. PD:	0.025
		Sc. rank:	40136.0

,

HA			
Pos.	258	obs:	exp:
tta	L	0	0.12
ttg	L	0	0.21
tct	S	101	115.20
tcc	S	613	106.70
tca	S	0	191.90
tcg	S	0	28.11
ctt	L	1	0.21
ctc	L	0	0.15
cta	L	0	0.15
ctg	L	0	0.17
cct	P	64052	21678.00
ccc	P	984	12196.00
cca	P	13	21978.00
ccg	P	3	9201.00
cat	H	5	2.82
cac	H	0	2.18
agt	S	0	124.80
agc	S	0	147.30
gct	A	0	0.27
gcc	A	0	0.25
gca	A	1	0.40
gcg	A	0	0.07
gaa	E	1	0.59
gag	E	0	0.41
---	--	------	------
mPD		0.070	0.75
		nPD:	0.09
		N. weight:	1.1
		Sc. PD:	0.01
		Sc. rank:	30616.0

,

HA

Pos.	259	obs:	exp:
tct	S	0	10613.00
tcc	S	0	9829.00
tca	S	0	17678.00
tcg	S	0	2589.00
act	T	0	0.31
acc	T	1	0.18
aca	T	0	0.43
acg	T	0	0.08
aat	N	0	0.56
aac	N	1	0.44
agt	S	328	11494.00
agc	S	65442	13568.00
ggt	G	0	0.27
ggc	G	0	0.28
gga	G	0	0.92
ggg	G	2	0.53
---	--	------	------
mPD		0.010	1.6
		nPD:	0.01
		N. weight:	1.7
		Sc. PD:	-0.13
		Sc. rank:	-64636.0

,

HA

Pos.	260	obs:	exp:
caa	Q	4	2.45
cag	Q	0	1.55
cgt	R	0	1301.00
cgc	R	0	2360.00
cga	R	79	5985.00
cgg	R	1	5633.00
att	I	0	55.82
atc	I	0	41.78
ata	I	159	61.41
aaa	K	1	0.66
aag	K	0	0.34
aga	R	65354	32176.00
agg	R	167	18145.00
ggt	G	0	1.21
ggc	G	0	1.26
gga	G	9	4.15
ggg	G	0	2.38
---	--	------	------
mPD		0.013	0.89
		nPD:	0.01
		N. weight:	0.76
		Sc. PD:	-0.05
		Sc. rank:	-14477.0

,

HA

Pos.	261	obs:	exp:
tta	L	0	1.16
ttg	L	0	2.07
ctt	L	1	2.09
ctc	L	0	1.47
cta	L	9	1.54
ctg	L	0	1.68
att	I	5	22506.00
atc	I	2	16846.00
ata	I	64105	24760.00
atg	M	18	18.00
aaa	K	1	0.66
aag	K	0	0.34
gtt	V	0	409.00
gtc	V	0	332.70
gta	V	1624	353.90
gtg	V	9	537.30
---	--	------	------
mPD		0.050	0.71
		nPD:	0.07
		N. weight:	1.1
		Sc. PD:	-0.014
		Sc. rank:	23320.0

,

HA

Pos.	262	obs:	exp:
tct	S	0	10612.00
tcc	S	0	9828.00
tca	S	0	17676.00
tcg	S	0	2589.00
cgt	R	0	0.04
cgc	R	1	0.07
cga	R	0	0.18
cgg	R	0	0.17
act	T	0	0.31
acc	T	1	0.18
aca	T	0	0.43
acg	T	0	0.08
aat	N	0	1.68
aac	N	3	1.32
agt	S	78	11492.00
agc	S	65685	13567.00
aga	R	0	0.98
agg	R	1	0.55
ggt	G	0	0.67
ggc	G	5	0.70
gga	G	0	2.31
ggg	G	0	1.32
---	--	------	------
mPD		0.0027	1.6
		nPD:	0.
		N. weight:	1.7
		Sc. PD:	-0.13
		Sc. rank:	-87513.0

,

HA

Pos.	263	obs:	exp:
ttt	F	0	2.92
ttc	F	6	3.08
tta	L	0	0.12
ttg	L	0	0.21
tat	Y	2	1.05
tac	Y	0	0.95
ctt	L	0	0.21
ctc	L	1	0.15
cta	L	0	0.15
ctg	L	0	0.17
att	I	120	23081.00
atc	I	65555	17277.00
ata	I	75	25392.00
atg	M	1	1.00
act	T	0	0.31
acc	T	1	0.18
aca	T	0	0.43
acg	T	0	0.08
aaa	K	1	0.66
aag	K	0	0.34
gtt	V	0	3.01
gtc	V	12	2.45
gta	V	0	2.60
gtg	V	0	3.95
---	--	------	------
mPD		0.0068	0.66
		nPD:	0.01
		N. weight:	1.5
		Sc. PD:	-0.1
		Sc. rank:	-41878.0

,

HA

Pos.	264	obs:	exp:
ttt	F	2	1.46
ttc	F	1	1.54
tat	Y	65656	34649.00
tac	Y	78	31085.00
cat	H	37	20.87
cac	H	0	16.13
---	--	------	------
mPD		0.0036	0.50
		nPD:	0.01
		N. weight:	0.7
		Sc. PD:	-0.05
		Sc. rank:	-24643.0

,

HA

Pos.	265	obs:	exp:
tgg	W	65773	65773.00
cgt	R	0	0.02
cgc	R	0	0.04
cga	R	0	0.09
cgg	R	1	0.09
aga	R	0	0.49
agg	R	0	0.28
---	--	------	------
mPD		0.000030	0.000050
		nPD:	0.61
		N. weight:	0.000041
		Sc. PD:	0.000021
		Sc. rank:	2.1

,

HA

Pos.	266	obs:	exp:
cct	P	0	0.33
ccc	P	0	0.19
cca	P	1	0.34
ccg	P	0	0.14
att	I	0	0.35
atc	I	0	0.26
ata	I	1	0.39
act	T	5	20424.00
acc	T	27	11628.00
aca	T	65539	28462.00
acg	T	196	5253.00
gct	A	0	0.55
gcc	A	0	0.50
gca	A	2	0.81
gcg	A	0	0.15
ggt	G	3	0.40
ggc	G	0	0.42
gga	G	0	1.38
ggg	G	0	0.79
---	--	------	------
mPD		0.0073	0.68
		nPD:	0.01
		N. weight:	0.9
		Sc. PD:	-0.062
		Sc. rank:	-25231.0

,

HA

Pos.	267	obs:	exp:
tta	L	2	0.93
ttg	L	0	1.65
ctt	L	1	1.67
ctc	L	0	1.17
cta	L	5	1.23
ctg	L	0	1.34
cat	H	1	0.56
cac	H	0	0.44
att	I	11	23057.00
atc	I	3	17259.00
ata	I	65669	25367.00
atg	M	5	5.00
act	T	0	1.24
acc	T	0	0.71
aca	T	4	1.73
acg	T	0	0.32
gtt	V	0	18.28
gtc	V	0	14.87
gta	V	73	15.82
gtg	V	0	24.02
---	--	------	------
mPD		0.0033	0.66
		nPD:	0.
		N. weight:	1.
		Sc. PD:	-0.078
		Sc. rank:	-44178.0

,

HA

Pos.	268	obs:	exp:
att	I	0	1.40
atc	I	0	1.05
ata	I	4	1.54
gtt	V	78	16474.00
gtc	V	7	13401.00
gta	V	65457	14255.00
gtg	V	228	21640.00
---	--	------	------
mPD		0.0096	0.74
		nPD:	0.01
		N. weight:	1.7
		Sc. PD:	-0.11
		Sc. rank:	-36624.0

,

HA

Pos.	269	obs:	exp:
caa	Q	4	2.45
cag	Q	0	1.55
cgt	R	0	1.94
cgc	R	0	3.53
cga	R	0	8.94
cgg	R	0	8.42
att	I	0	0.35
atc	I	0	0.26
ata	I	1	0.39
act	T	0	0.31
acc	T	0	0.18
aca	T	1	0.43
acg	T	0	0.08
aat	N	3	11.19
aac	N	17	8.81
aaa	K	65508	43153.00
aag	K	130	22485.00
aga	R	97	48.07
agg	R	1	27.11
gaa	E	10	7.07
gag	E	2	4.93
---	--	------	------
mPD		0.0082	0.46
		nPD:	0.02
		N. weight:	0.45
		Sc. PD:	-0.028
		Sc. rank:	-5566.3

,

HA

Pos.	270	obs:	exp:
tct	S	0	8.39
tcc	S	0	7.77
tca	S	5	13.98
tcg	S	47	2.05
cct	P	66	21897.00
ccc	P	3	12319.00
cca	P	6732	22200.00
ccg	P	58909	9294.00
caa	Q	0	3.67
cag	Q	6	2.33
act	T	0	0.93
acc	T	0	0.53
aca	T	0	1.30
acg	T	3	0.24
agt	S	0	9.09
agc	S	0	10.73
gct	A	0	0.82
gcc	A	0	0.75
gca	A	0	1.21
gcg	A	3	0.22
---	--	------	------
mPD		0.19	0.72
		nPD:	0.26
		N. weight:	1.7
		Sc. PD:	0.28
		Sc. rank:	70045.0

,

HA

Pos.	271	obs:	exp:
cgt	R	0	0.63
cgc	R	0	1.15
cga	R	0	2.92
cgg	R	0	2.75
aga	R	32	15.70
agg	R	0	8.85
gct	A	0	0.27
gcc	A	0	0.25
gca	A	1	0.40
gcg	A	0	0.07
gaa	E	1	0.59
gag	E	0	0.41
ggt	G	229	8850.00
ggc	G	2	9175.00
gga	G	65259	30347.00
ggg	G	250	17368.00
---	--	------	------
mPD		0.016	0.68
		nPD:	0.02
		N. weight:	0.81
		Sc. PD:	-0.046
		Sc. rank:	-3140.7

,

HA

Pos.	272	obs:	exp:
gct	A	0	0.27
gcc	A	1	0.25
gca	A	0	0.40
gcg	A	0	0.07
gat	D	118	37177.00
gac	D	65652	28593.00
gaa	E	1	1.18
gag	E	1	0.82
ggt	G	0	0.13
ggc	G	0	0.14
gga	G	1	0.46
ggg	G	0	0.26
---	--	------	------
mPD		0.0037	0.49
		nPD:	0.01
		N. weight:	0.91
		Sc. PD:	-0.065
		Sc. rank:	-30966.0

,

HA

Pos.	273	obs:	exp:
tta	L	14	5.92
ttg	L	0	10.54
ctt	L	0	10.67
ctc	L	0	7.48
cta	L	37	7.83
ctg	L	0	8.56
att	I	7	22946.00
atc	I	14	17176.00
ata	I	65345	25244.00
atg	M	142	142.00
act	T	0	14.91
acc	T	0	8.49
aca	T	48	20.77
acg	T	0	3.83
aaa	K	4	2.63
aag	K	0	1.37
gtt	V	0	40.83
gtc	V	0	33.21
gta	V	163	35.33
gtg	V	0	53.63
---	--	------	------
mPD		0.013	0.67
		nPD:	0.02
		N. weight:	1.
		Sc. PD:	-0.064
		Sc. rank:	-9205.0

,

HA

Pos.	274	obs:	exp:
ttt	F	26	12.65
ttc	F	0	13.35
tta	L	0	7623.00
ttg	L	0	13582.00
ctt	L	65417	13743.00
ctc	L	139	9634.00
cta	L	3	10088.00
ctg	L	136	11025.00
att	I	51	18.61
atc	I	0	13.93
ata	I	2	20.47
---	--	------	------
mPD		0.011	1.2
		nPD:	0.01
		N. weight:	1.7
		Sc. PD:	-0.12
		Sc. rank:	-52485.0

,

HA

Pos.	275	obs:	exp:
ttt	F	5	2.43
ttc	F	0	2.57
tta	L	330	7624.00
ttg	L	65278	13583.00
tct	S	0	0.65
tcc	S	0	0.60
tca	S	0	1.08
tcg	S	4	0.16
ctt	L	0	13743.00
ctc	L	0	9634.00
cta	L	2	10088.00
ctg	L	88	11026.00
att	I	0	1.05
atc	I	0	0.79
ata	I	3	1.16
atg	M	2	2.00
act	T	0	0.62
acc	T	0	0.35
aca	T	2	0.87
acg	T	0	0.16
agt	S	0	0.70
agc	S	0	0.83
gtt	V	0	15.03
gtc	V	0	12.23
gta	V	59	13.00
gtg	V	1	19.74
---	--	------	------
mPD		0.017	1.2
		nPD:	0.01
		N. weight:	1.7
		Sc. PD:	-0.11
		Sc. rank:	-29733.0

,

HA

Pos.	276	obs:	exp:
ttt	F	3	2.43
ttc	F	2	2.57
tct	S	0	0.16
tcc	S	0	0.15
tca	S	0	0.27
tcg	S	0	0.04
att	I	65554	23076.00
atc	I	172	17273.00
ata	I	9	25387.00
atg	M	1	1.00
agt	S	1	0.17
agc	S	0	0.21
gtt	V	32	8.02
gtc	V	0	6.52
gta	V	0	6.94
gtg	V	0	10.53
---	--	------	------
mPD		0.0067	0.66
		nPD:	0.01
		N. weight:	1.1
		Sc. PD:	-0.079
		Sc. rank:	-33073.0

,

HA

Pos.	277	obs:	exp:
tct	S	0	5.97
tcc	S	0	5.53
tca	S	0	9.94
tcg	S	0	1.46
tat	Y	0	1.05
tac	Y	2	0.95
cat	H	0	9.59
cac	H	17	7.41
act	T	0	2.17
acc	T	7	1.24
aca	T	0	3.03
acg	T	0	0.56
aat	N	536	36752.00
aac	N	65155	28939.00
aaa	K	15	9.86
aag	K	0	5.14
agt	S	4	6.47
agc	S	33	7.63
gat	D	0	1.70
gac	D	3	1.30
gaa	E	2	1.18
gag	E	0	0.82
---	--	------	------
mPD		0.019	0.50
		nPD:	0.04
		N. weight:	0.85
		Sc. PD:	-0.037
		Sc. rank:	8404.2

,

HA

Pos.	278	obs:	exp:
tct	S	0	10609.00
tcc	S	0	9825.00
tca	S	0	17671.00
tcg	S	0	2588.00
tgt	C	0	5.47
tgc	C	14	8.53
aat	N	0	5.04
aac	N	9	3.96
agt	S	201	11489.00
agc	S	65543	13563.00
gct	A	0	0.55
gcc	A	0	0.50
gca	A	2	0.81
gcg	A	0	0.15
ggt	G	0	0.67
ggc	G	5	0.70
gga	G	0	2.31
ggg	G	0	1.32
---	--	------	------
mPD		0.0071	1.6
		nPD:	0.
		N. weight:	1.7
		Sc. PD:	-0.13
		Sc. rank:	-75669.0

,

HA

Pos.	279	obs:	exp:
tct	S	0	2.58
tcc	S	0	2.39
tca	S	15	4.30
tcg	S	0	0.63
cct	P	0	0.33
ccc	P	0	0.19
cca	P	1	0.34
ccg	P	0	0.14
cgt	R	0	0.02
cgc	R	0	0.04
cga	R	0	0.09
cgg	R	0	0.09
att	I	0	4.21
atc	I	1	3.15
ata	I	11	4.63
act	T	72	20391.00
acc	T	151	11609.00
aca	T	64804	28416.00
acg	T	633	5244.00
aat	N	73	41.40
aac	N	1	32.60
aaa	K	7	4.60
aag	K	0	2.40
agt	S	1	2.80
agc	S	0	3.30
aga	R	0	0.49
agg	R	1	0.28
gct	A	0	0.82
gcc	A	0	0.75
gca	A	3	1.21
gcg	A	0	0.22
---	--	------	------
mPD		0.032	0.68
		nPD:	0.05
		N. weight:	0.86
		Sc. PD:	-0.03
		Sc. rank:	12859.0

,

HA

Pos.	280	obs:	exp:
cgt	R	0	0.02
cgc	R	0	0.04
cga	R	0	0.09
cgg	R	0	0.09
aga	R	0	0.49
agg	R	1	0.28
ggt	G	6	8855.00
ggc	G	0	9180.00
gga	G	941	30362.00
ggg	G	64826	17377.00
---	--	------	------
mPD		0.028	0.68
		nPD:	0.04
		N. weight:	1.4
		Sc. PD:	-0.054
		Sc. rank:	18804.0

,

HA

Pos.	281	obs:	exp:
aat	N	65330	36798.00
aac	N	443	28975.00
gat	D	1	0.57
gac	D	0	0.43
---	--	------	------
mPD		0.013	0.49
		nPD:	0.03
		N. weight:	0.6
		Sc. PD:	-0.032
		Sc. rank:	616.8

,

HA

Pos.	282	obs:	exp:
tta	L	134	7632.00
ttg	L	0	13597.00
ctt	L	1	13758.00
ctc	L	12	9644.00
cta	L	65559	10099.00
ctg	L	61	11038.00
att	I	0	2.46
atc	I	0	1.84
ata	I	7	2.70
---	--	------	------
mPD		0.0065	1.2
		nPD:	0.01
		N. weight:	2.
		Sc. PD:	-0.15
		Sc. rank:	-81054.0

,

HA

Pos.	283	obs:	exp:
tta	L	0	0.35
ttg	L	1	0.62
ctt	L	2	0.63
ctc	L	0	0.44
cta	L	0	0.46
ctg	L	0	0.50
att	I	65458	23063.00
atc	I	73	17263.00
ata	I	168	25373.00
act	T	1	0.31
acc	T	0	0.18
aca	T	0	0.43
acg	T	0	0.08
aat	N	1	1.12
aac	N	1	0.88
gtt	V	68	17.28
gtc	V	0	14.06
gta	V	0	14.96
gtg	V	1	22.70
---	--	------	------
mPD		0.0097	0.66
		nPD:	0.01
		N. weight:	1.1
		Sc. PD:	-0.074
		Sc. rank:	-20255.0

,

HA

Pos.	284	obs:	exp:
gtt	V	1	0.75
gtc	V	0	0.61
gta	V	2	0.65
gtg	V	0	0.99
gct	A	65363	17925.00
gcc	A	382	16431.00
gca	A	19	26603.00
gcg	A	3	4808.00
ggt	G	4	0.54
ggc	G	0	0.56
gga	G	0	1.85
ggg	G	0	1.06
---	--	------	------
mPD		0.012	0.69
		nPD:	0.02
		N. weight:	1.4
		Sc. PD:	-0.087
		Sc. rank:	-16925.0

,

HA

Pos.	285	obs:	exp:
tta	L	0	0.23
ttg	L	0	0.41
tct	S	1	0.16
tcc	S	0	0.15
tca	S	0	0.27
tcg	S	0	0.04
tgt	C	1	0.39
tgc	C	0	0.61
ctt	L	2	0.42
ctc	L	0	0.29
cta	L	0	0.31
ctg	L	0	0.34
cct	P	64642	21917.00
ccc	P	1050	12330.00
cca	P	60	22220.00
ccg	P	17	9302.00
agt	S	0	0.17
agc	S	0	0.21
gct	A	1	0.27
gcc	A	0	0.25
gca	A	0	0.40
gcg	A	0	0.07
---	--	------	------
mPD		0.034	0.72
		nPD:	0.05
		N. weight:	1.1
		Sc. PD:	-0.039
		Sc. rank:	17385.0

,

HA

Pos.	286	obs:	exp:
tct	S	0	0.65
tcc	S	0	0.60
tca	S	0	1.08
tcg	S	0	0.16
tgg	W	1	1.00
cgt	R	0	1304.00
cgc	R	0	2366.00
cga	R	2	5999.00
cgg	R	888	5646.00
atg	M	3	3.00
aaa	K	0	9.20
aag	K	14	4.80
agt	S	4	0.70
agc	S	0	0.83
aga	R	305	32250.00
agg	R	64557	18187.00
---	--	------	------
mPD		0.037	0.89
		nPD:	0.04
		N. weight:	1.3
		Sc. PD:	-0.053
		Sc. rank:	17137.0

,

HA			
Pos.	287	obs:	exp:
tat	Y	2	1.05
tac	Y	0	0.95
cgt	R	0	0.02
cgc	R	0	0.04
cga	R	0	0.09
cgg	R	0	0.09
aga	R	1	0.49
agg	R	0	0.28
gaa	E	0	0.59
gag	E	1	0.41
ggt	G	65422	8854.00
ggc	G	121	9179.00
gga	G	3	30361.00
ggg	G	224	17376.00
---	--	------	------
mPD		0.011	0.68
		nPD:	0.02
		N. weight:	2.2
		Sc. PD:	-0.14
		Sc. rank:	-33300.0

,

HA			
Pos.	288	obs:	exp:
ttt	F	0	1.46
ttc	F	3	1.54
tat	Y	259	34667.00
tac	Y	65508	31100.00
cat	H	0	0.56
cac	H	1	0.44
aaa	K	1	0.66
aag	K	0	0.34
gct	A	0	0.55
gcc	A	0	0.50
gca	A	2	0.81
gcg	A	0	0.15
---	--	------	------
mPD		0.0082	0.50
		nPD:	0.02
		N. weight:	0.8
		Sc. PD:	-0.051
		Sc. rank:	-11828.0

,

HA			
Pos.	289	obs:	exp:
ttt	F	232	31993.00
ttc	F	65539	33778.00
tct	S	0	0.16
tcc	S	1	0.15
tca	S	0	0.27
tcg	S	0	0.04
tat	Y	0	1.05
tac	Y	2	0.95
agt	S	0	0.17
agc	S	0	0.21
---	--	------	------
mPD		0.0071	0.50
		nPD:	0.01
		N. weight:	0.71
		Sc. PD:	-0.047
		Sc. rank:	-13601.0

,

HA			
Pos.	290	obs:	exp:
cgt	R	0	2.02
cgc	R	0	3.67
cga	R	0	9.31
cgg	R	0	8.76
att	I	0	0.35
atc	I	0	0.26
ata	I	1	0.39
act	T	0	0.62
acc	T	0	0.35
aca	T	2	0.87
acg	T	0	0.16
aaa	K	65280	43171.00
aag	K	386	22495.00
aga	R	102	50.03
agg	R	0	28.21
gct	A	0	0.27
gcc	A	0	0.25
gca	A	1	0.40
gcg	A	0	0.07
gaa	E	2	1.18
gag	E	0	0.82
---	--	------	------
mPD		0.015	0.45
		nPD:	0.03
		N. weight:	0.43
		Sc. PD:	-0.021
		Sc. rank:	1877.2

,

HA			
Pos.	291	obs:	exp:
tta	L	35	6.50
ttg	L	0	11.58
ctt	L	0	11.71
ctc	L	0	8.21
cta	L	21	8.60
ctg	L	0	9.40
att	I	5	22942.00
atc	I	5	17173.00
ata	I	65345	25240.00
atg	M	269	269.00
act	T	0	0.93
acc	T	0	0.53
aca	T	3	1.30
acg	T	0	0.24
aat	N	1	0.56
aac	N	0	0.44
gtt	V	0	22.54
gtc	V	0	18.34
gta	V	89	19.51
gtg	V	1	29.61
---	--	------	------
mPD		0.013	0.67
		nPD:	0.02
		N. weight:	1.
		Sc. PD:	-0.063
		Sc. rank:	-9005.7

,

HA			
Pos.	292	obs:	exp:
gaa	E	2	1.18
gag	E	0	0.82
---	-	65772	65772.00
---	--	------	------
mPD		0	4.5×10^{-10}
		nPD:	0.
		N. weight:	0.
		Sc. PD:	0
		Sc. rank:	0

,

HA			
Pos.	293	obs:	exp:
tta	L	0	34.93
ttg	L	0	62.23
ctt	L	0	62.97
ctc	L	1	44.14
cta	L	300	46.22
ctg	L	0	50.52
cct	P	0	1.00
ccc	P	0	0.56
cca	P	3	1.01
ccg	P	0	0.42
cat	H	1	2.26
cac	H	3	1.74
caa	Q	9634	5897.00
cag	Q	13	3750.00
cgt	R	0	1107.00
cgc	R	95	2008.00
cga	R	55114	5093.00
cgg	R	506	4793.00
aaa	K	3	1.97
aag	K	0	1.03
aga	R	100	27377.00
agg	R	1	15439.00
---	--	------	------
mPD		0.28	1.2
		nPD:	0.23
		N. weight:	2.3
		Sc. PD:	0.31
		Sc. rank:	89934.0

,

HA			
Pos.	294	obs:	exp:
tct	S	0	9906.00
tcc	S	0	9174.00
tca	S	0	16500.00
tcg	S	0	2416.00
cat	H	1	0.56
cac	H	0	0.44
cgt	R	5	0.67
cgc	R	0	1.22
cga	R	0	3.10
cgg	R	0	2.92
att	I	18	6.32
atc	I	0	4.73
ata	I	0	6.95
act	T	182	56.52
acc	T	0	32.18
aca	T	0	78.77
acg	T	0	14.54
aat	N	4080	2283.00
aac	N	1	1798.00
aaa	K	0	1.31
aag	K	2	0.69
agt	S	61353	10728.00
agc	S	36	12664.00
aga	R	6	16.68
agg	R	23	9.40
gat	D	2	1.13
gac	D	0	0.87
ggt	G	65	8.75
ggc	G	0	9.07
gga	G	0	30.01
ggg	G	0	17.17
---	--	------	------
mPD		0.13	1.7
		nPD:	0.07
		N. weight:	1.8
		Sc. PD:	-0.016
		Sc. rank:	42557.0

,

HA			
Pos .	295	obs :	exp :
tgg	W	1	1.00
cgt	R	0	1.13
cgc	R	0	2.05
cga	R	0	5.20
cgg	R	0	4.89
aga	R	0	27.96
agg	R	57	15.77
gtt	V	0	0.50
gtc	V	0	0.41
gta	V	0	0.43
gtg	V	2	0.66
gct	A	2	0.55
gcc	A	0	0.50
gca	A	0	0.81
gcg	A	0	0.15
gaa	E	1	12.38
gag	E	20	8.62
ggt	G	27	8844.00
ggc	G	0	9168.00
gga	G	751	30324.00
ggg	G	64913	17355.00
---	--	------	------
mPD		0.026	0.68
		nPD :	0.04
		N. weight :	1.4
		Sc. PD :	-0.06
		Sc. rank :	14060.0

,

HA			
Pos .	296	obs :	exp :
cgt	R	0	2.66
cgc	R	0	4.82
cga	R	0	12.23
cgg	R	0	11.51
act	T	0	2.17
acc	T	0	1.24
aca	T	7	3.03
acg	T	0	0.56
aat	N	1	22.94
aac	N	40	18.06
aaa	K	65349	43057.00
aag	K	143	22435.00
aga	R	134	65.72
agg	R	0	37.06
gaa	E	98	57.78
gag	E	0	40.22
ggt	G	0	0.27
ggc	G	0	0.28
gga	G	2	0.92
ggg	G	0	0.53
---	--	------	------
mPD		0.013	0.46
		nPD :	0.03
		N. weight :	0.45
		Sc. PD :	-0.024
		Sc. rank :	880.2

,

HA			
Pos .	297	obs :	exp :
tct	S	2	10607.00
tcc	S	0	9822.00
tca	S	0	17667.00
tcg	S	0	2587.00
aat	N	0	3.92
aac	N	7	3.08
agt	S	60	11486.00
agc	S	65667	13560.00
gat	D	0	0.57
gac	D	1	0.43
ggt	G	0	4.98
ggc	G	37	5.16
gga	G	0	17.08
ggg	G	0	9.78
---	--	------	------
mPD		0.0034	1.6
		nPD :	0.
		N. weight :	1.7
		Sc. PD :	-0.13
		Sc. rank :	-85084.0

,

HA			
Pos .	298	obs :	exp :
tct	S	30	10613.00
tcc	S	9	9828.00
tca	S	65590	17677.00
tcg	S	140	2589.00
cct	P	0	0.33
ccc	P	0	0.19
cca	P	1	0.34
ccg	P	0	0.14
act	T	0	0.31
acc	T	0	0.18
aca	T	1	0.43
acg	T	0	0.08
agt	S	0	11493.00
agc	S	0	13568.00
gct	A	0	0.27
gcc	A	0	0.25
gca	A	1	0.40
gcg	A	0	0.07
ggt	G	2	0.27
ggc	G	0	0.28
gga	G	0	0.92
ggg	G	0	0.53
---	--	------	------
mPD		0.0057	1.6
		nPD :	0.
		N. weight :	1.4
		Sc. PD :	-0.11
		Sc. rank :	-66406.0

,

HA			
Pos .	299	obs :	exp :
tta	L	0	0.12
ttg	L	0	0.21
ctt	L	0	0.21
ctc	L	0	0.15
cta	L	1	0.15
ctg	L	0	0.17
att	I	8	23047.00
atc	I	0	17251.00
ata	I	65644	25355.00
atg	M	10	10.00
act	T	0	2.17
acc	T	0	1.24
aca	T	7	3.03
acg	T	0	0.56
aaa	K	1	0.66
aag	K	0	0.34
gtt	V	0	25.80
gtc	V	0	20.99
gta	V	103	22.32
gtg	V	0	33.89
---	--	------	------
mPD		0.0039	0.66
		nPD :	0.01
		N. weight :	1.1
		Sc. PD :	-0.077
		Sc. rank :	-40807.0

,

HA			
Pos .	300	obs :	exp :
tta	L	0	0.58
ttg	L	0	1.03
ctt	L	0	1.05
ctc	L	0	0.73
cta	L	0	0.77
ctg	L	5	0.84
att	I	7	25.63
atc	I	3	19.18
ata	I	63	28.19
atg	M	65690	65690.00
aaa	K	0	0.66
aag	K	1	0.34
gtt	V	0	1.25
gtc	V	0	1.02
gta	V	0	1.08
gtg	V	5	1.65
---	--	------	------
mPD		0.0026	0.0028
		nPD :	0.92
		N. weight :	0.00092
		Sc. PD :	0.00074
		Sc. rank :	47.3

,

HA			
Pos .	301	obs :	exp :
tct	S	0	0.32
tcc	S	0	0.30
tca	S	0	0.54
tcg	S	0	0.08
cgt	R	0	1299.00
cgc	R	0	2358.00
cga	R	13	5979.00
cgg	R	0	5627.00
att	I	2	3.51
atc	I	0	2.63
ata	I	8	3.86
aaa	K	226	150.60
aag	K	3	78.45
agt	S	0	0.35
agc	S	2	0.41
aga	R	65016	32140.00
agg	R	498	18125.00
ggt	G	0	0.81
ggc	G	0	0.84
gga	G	6	2.77
ggg	G	0	1.59
---	--	------	------
mPD		0.023	0.89
		nPD :	0.03
		N. weight :	0.74
		Sc. PD :	-0.04
		Sc. rank :	-895.2

,

HA			
Pos .	302	obs :	exp :
tct	S	16	10606.00
tcc	S	2	9822.00
tca	S	65668	17666.00
tcg	S	40	2587.00
act	T	0	14.60
acc	T	0	8.31
aca	T	47	20.34
acg	T	0	3.75
agt	S	0	11486.00
agc	S	0	13559.00
gct	A	0	0.27
gcc	A	0	0.25
gca	A	1	0.40
gcg	A	0	0.07
---	--	------	------
mPD		0.0032	1.6
		nPD :	0.
		N. weight :	1.4
		Sc. PD :	-0.11
		Sc. rank :	-71865.0

,

HA			
Pos .	303	obs :	exp :
tat	Y	1	0.53
tac	Y	0	0.47
aat	N	126	70.49
aac	N	0	55.51
gat	D	62876	37104.00
gac	D	2764	28536.00
gaa	E	2	1.18
gag	E	0	0.82
ggt	G	5	0.67
ggc	G	0	0.70
gga	G	0	2.31
ggg	G	0	1.32
---	--	------	------
mPD		0.085	0.50
		nPD :	0.17
		N. weight :	0.45
		Sc. PD :	0.037
		Sc. rank :	16407.0

,

HA			
Pos .	304	obs :	exp :
tct	S	0	1.78
tcc	S	0	1.64
tca	S	11	2.96
tcg	S	0	0.43
act	T	0	19.88
acc	T	0	11.32
aca	T	64	27.70
acg	T	0	5.11
agt	S	0	1.92
agc	S	0	2.27
gtt	V	0	5.01
gtc	V	0	4.08
gta	V	20	4.33
gtg	V	0	6.58
gct	A	14	17900.00
gcc	A	52	16408.00
gca	A	65575	26566.00
gcg	A	34	4801.00
gat	D	0	0.57
gac	D	1	0.43
gaa	E	3	1.77
gag	E	0	1.23
---	--	------	------
mPD		0.0061	0.70
		nPD :	0.01
		N. weight :	0.99
		Sc. PD :	-0.07
		Sc. rank :	-31628.0

,

HA			
Pos .	305	obs :	exp :
tta	L	0	3.83
ttg	L	0	6.82
tct	S	1	7.10
tcc	S	43	6.58
tca	S	0	11.83
tcg	S	0	1.73
ctt	L	0	6.90
ctc	L	33	4.84
cta	L	0	5.07
ctg	L	0	5.54
cct	P	232	21834.00
ccc	P	65199	12284.00
cca	P	91	22137.00
ccg	P	0	9267.00
cat	H	0	82.91
cac	H	147	64.09
cgt	R	0	0.02
cgc	R	1	0.04
cga	R	0	0.09
cgg	R	0	0.09
act	T	0	8.38
acc	T	27	4.77
aca	T	0	11.68
acg	T	0	2.16
agt	S	0	7.69
agc	S	0	9.08
aga	R	0	0.49
agg	R	0	0.28
---	--	------	------
mPD		0.017	0.73
		nPD :	0.02
		N. weight :	1.8
		Sc. PD :	-0.1
		Sc. rank :	-4780.8

,

HA			
Pos .	306	obs :	exp :
tta	L	0	0.35
ttg	L	0	0.62
ctt	L	3	0.63
ctc	L	0	0.44
cta	L	0	0.46
ctg	L	0	0.50
cat	H	1	0.56
cac	H	0	0.44
att	I	65399	23069.00
atc	I	221	17268.00
ata	I	96	25379.00
atg	M	22	22.00
act	T	3	0.93
acc	T	0	0.53
aca	T	0	1.30
acg	T	0	0.24
gtt	V	27	7.26
gtc	V	2	5.91
gta	V	0	6.29
gtg	V	0	9.54
---	--	------	------
mPD		0.011	0.66
		nPD :	0.02
		N. weight :	1.1
		Sc. PD :	-0.07
		Sc. rank :	-14806.0

,

HA			
Pos.	307	obs:	exp:
tct	S	0	7.26
tcc	S	0	6.72
tca	S	0	12.10
tcg	S	0	1.77
tat	Y	0	1.05
tac	Y	2	0.95
tgt	C	0	0.39
tgc	C	1	0.61
cat	H	0	1.13
cac	H	2	0.87
agt	S	0	7.86
agc	S	45	9.28
gtt	V	0	1.75
gtc	V	7	1.43
gta	V	0	1.52
gtg	V	0	2.30
gat	D	172	117.60
gac	D	36	90.43
gaa	E	2	1.18
gag	E	0	0.82
ggt	G	82	8819.00
ggc	G	65129	9143.00
gga	G	295	30239.00
ggg	G	1	17306.00
---	--	------	------
mPD		0.025	0.69
		nPD:	0.04
		N. weight:	2.1
		Sc. PD:	-0.095
		Sc. rank:	17359.0

,

HA			
Pos.	308	obs:	exp:
tct	S	0	0.16
tcc	S	0	0.15
tca	S	0	0.27
tcg	S	0	0.04
caa	Q	6	3.67
cag	Q	0	2.33
cgt	R	0	114.00
cgc	R	0	206.80
cga	R	0	524.40
cgg	R	0	493.60
att	I	0	0.35
atc	I	1	0.26
ata	I	0	0.39
atg	M	1	1.00
act	T	1	72.36
acc	T	188	41.19
aca	T	43	100.80
acg	T	1	18.61
aat	N	14	115.80
aac	N	193	91.19
aaa	K	54025	39054.00
aag	K	5379	20350.00
agt	S	1	0.17
agc	S	0	0.21
aga	R	5725	2819.00
agg	R	23	1590.00
gct	A	0	0.27
gcc	A	1	0.25
gca	A	0	0.40
gcg	A	0	0.07
gat	D	2	1.13
gac	D	0	0.87
gaa	E	167	99.64
gag	E	2	69.36
ggt	G	0	0.13
ggc	G	0	0.14
gga	G	1	0.46
ggg	G	0	0.26
---	--	------	------
mPD		0.33	0.67
		nPD:	0.49
		N. weight:	0.25
		Sc. PD:	0.097
		Sc. rank:	12021.0

,

HA			
Pos.	309	obs:	exp:
tct	S	0	0.16
tcc	S	0	0.15
tca	S	0	0.27
tcg	S	0	0.04
tat	Y	0	0.53
tac	Y	1	0.47
tgt	C	9634	25703.00
tgc	C	56138	40069.00
agt	S	0	0.17
agc	S	1	0.21
---	--	------	------
mPD		0.25	0.48
		nPD:	0.53
		N. weight:	0.16
		Sc. PD:	0.067
		Sc. rank:	7812.1

,

HA			
Pos.	310	obs:	exp:
tct	S	0	32.43
tcc	S	0	30.04
tca	S	0	54.02
tcg	S	0	7.91
tat	Y	2	1.05
tac	Y	0	0.95
cat	H	2	1.13
cac	H	0	0.87
cgt	R	0	1.15
cgc	R	0	2.09
cga	R	0	5.29
cgg	R	0	4.98
att	I	52	18.25
atc	I	0	13.66
ata	I	0	20.08
atg	M	4	4.00
act	T	0	0.31
acc	T	0	0.18
aca	T	0	0.43
acg	T	1	0.08
aat	N	5815	3280.00
aac	N	48	2583.00
aaa	K	804	39142.00
aag	K	58733	20395.00
agt	S	188	35.13
agc	S	13	41.47
aga	R	0	28.45
agg	R	58	16.04
gtt	V	1	0.25
gtc	V	0	0.20
gta	V	0	0.22
gtg	V	0	0.33
gat	D	3	1.70
gac	D	0	1.30
gaa	E	1	29.48
gag	E	49	20.52
---	--	------	------
mPD		0.20	0.56
		nPD:	0.36
		N. weight:	1.1
		Sc. PD:	0.28
		Sc. rank:	46704.0

,

HA			
Pos.	311	obs:	exp:
ttt	F	96	46.70
ttc	F	0	49.30
tta	L	0	0.12
ttg	L	0	0.21
tct	S	65314	10568.00
tcc	S	69	9787.00
tca	S	91	17603.00
tcg	S	17	2578.00
tat	Y	176	94.35
tac	Y	3	84.65
tgt	C	1	0.39
tgc	C	0	0.61
ctt	L	1	0.21
ctc	L	0	0.15
cta	L	0	0.15
ctg	L	0	0.17
act	T	0	0.62
acc	T	0	0.35
aca	T	2	0.87
acg	T	0	0.16
agt	S	0	11445.00
agc	S	0	13511.00
gct	A	4	1.09
gcc	A	0	1.00
gca	A	0	1.62
gcg	A	0	0.29
---	--	------	------
mPD		0.014	1.6
		nPD:	0.01
		N. weight:	2.
		Sc. PD:	-0.14
		Sc. rank:	-64470.0

,

HA			
Pos.	312	obs:	exp:
tct	S	0	0.65
tcc	S	0	0.60
tca	S	4	1.08
tcg	S	0	0.16
act	T	2	15.84
acc	T	0	9.02
aca	T	49	22.07
acg	T	0	4.07
aaa	K	29	19.07
aag	K	0	9.93
agt	S	0	0.70
agc	S	0	0.83
gtt	V	0	0.50
gtc	V	0	0.41
gta	V	2	0.43
gtg	V	0	0.66
gct	A	2	429.30
gcc	A	3	393.50
gca	A	1570	637.10
gcg	A	0	115.10
gat	D	5	8.48
gac	D	10	6.52
gaa	E	63727	37756.00
gag	E	314	26285.00
ggt	G	0	7.67
ggc	G	0	7.96
gga	G	57	26.31
ggg	G	0	15.06
---	--	------	------
mPD		0.063	0.55
		nPD:	0.11
		N. weight:	0.56
		Sc. PD:	0.017
		Sc. rank:	18277.0

,

HA			
Pos.	313	obs:	exp:
tgt	C	158	25704.00
tgc	C	65615	40069.00
cgt	R	0	0.02
cgc	R	1	0.04
cga	R	0	0.09
cgg	R	0	0.09
aga	R	0	0.49
agg	R	0	0.28
---	--	------	------
mPD		0.0048	0.48
		nPD:	0.01
		N. weight:	0.53
		Sc. PD:	-0.037
		Sc. rank:	-15580.0

,

HA			
Pos.	314	obs:	exp:
caa	Q	0	1.22
cag	Q	2	0.78
att	I	2413	23076.00
atc	I	63259	17273.00
ata	I	65	25387.00
act	T	0	3.73
acc	T	12	2.12
aca	T	0	5.19
acg	T	0	0.96
gtt	V	0	5.76
gtc	V	23	4.69
gta	V	0	4.99
gtg	V	0	7.57
---	--	------	------
mPD		0.074	0.66
		nPD:	0.11
		N. weight:	1.3
		Sc. PD:	0.035
		Sc. rank:	41565.0

,

HA

Pos.	315	obs:	exp:
tct	S	1	0.81
tcc	S	0	0.75
tca	S	0	1.34
tcg	S	0	0.20
act	T	64719	20422.00
acc	T	1013	11627.00
aca	T	28	28460.00
acg	T	1	5252.00
agt	S	4	0.87
agc	S	0	1.03
gct	A	8	2.18
gcc	A	0	2.00
gca	A	0	3.24
gcg	A	0	0.58
---	--	------	------
mPD		0.032	0.68
		nPD:	0.05
		N. weight:	1.2
		Sc. PD:	-0.042
		Sc. rank:	18369.0

,

HA

Pos.	316	obs:	exp:
tct	S	0	0.16
tcc	S	0	0.15
tca	S	1	0.27
tcg	S	0	0.04
cct	P	3	21916.00
ccc	P	11	12330.00
cca	P	65614	22220.00
ccg	P	139	9302.00
caa	Q	3	1.83
cag	Q	0	1.17
act	T	0	0.93
acc	T	0	0.53
aca	T	3	1.30
acg	T	0	0.24
agt	S	0	0.17
agc	S	0	0.21
---	--	------	------
mPD		0.0049	0.72
		nPD:	0.01
		N. weight:	1.2
		Sc. PD:	-0.086
		Sc. rank:	-43380.0

,

HA

Pos.	317	obs:	exp:
tct	S	0	0.32
tcc	S	0	0.30
tca	S	0	0.54
tcg	S	0	0.08
tat	Y	2	1.05
tac	Y	0	0.95
cat	H	2	1.13
cac	H	0	0.87
att	I	1	0.35
atc	I	0	0.26
ata	I	0	0.39
act	T	1	0.31
acc	T	0	0.18
aca	T	0	0.43
acg	T	0	0.08
aat	N	65687	36791.00
aac	N	73	28969.00
aaa	K	0	0.66
aag	K	1	0.34
agt	S	2	0.35
agc	S	0	0.41
gat	D	4	2.26
gac	D	0	1.74
gaa	E	0	0.59
gag	E	1	0.41
---	--	------	------
mPD		0.0027	0.49
		nPD:	0.01
		N. weight:	0.63
		Sc. PD:	-0.047
		Sc. rank:	-25773.0

,

HA

Pos.	318	obs:	exp:
ggt	G	15	8855.00
ggc	G	0	9180.00
gga	G	65337	30362.00
ggg	G	422	17377.00
---	--	------	------
mPD		0.013	0.68
		nPD:	0.02
		N. weight:	0.81
		Sc. PD:	-0.049
		Sc. rank:	-7294.2

,

HA

Pos.	319	obs:	exp:
tct	S	0	10613.00
tcc	S	0	9828.00
tca	S	0	17677.00
tcg	S	0	2589.00
aat	N	0	1.68
aac	N	3	1.32
agt	S	108	11493.00
agc	S	65660	13568.00
gct	A	2	0.55
gcc	A	0	0.50
gca	A	0	0.81
gcg	A	0	0.15
ggt	G	0	0.13
ggc	G	1	0.14
gga	G	0	0.46
ggg	G	0	0.26
---	--	------	------
mPD		0.0036	1.6
		nPD:	0.
		N. weight:	1.7
		Sc. PD:	-0.13
		Sc. rank:	-84315.0

,

HA

Pos.	320	obs:	exp:
tta	L	0	0.70
ttg	L	0	1.24
ctt	L	6	1.26
ctc	L	0	0.88
cta	L	0	0.92
ctg	L	0	1.01
att	I	65472	23082.00
atc	I	266	17277.00
ata	I	15	25394.00
act	T	2	0.62
acc	T	0	0.35
aca	T	0	0.87
acg	T	0	0.16
gtt	V	13	3.26
gtc	V	0	2.65
gta	V	0	2.82
gtg	V	0	4.28
---	--	------	------
mPD		0.0091	0.66
		nPD:	0.01
		N. weight:	1.1
		Sc. PD:	-0.074
		Sc. rank:	-22132.0

,

HA

Pos.	321	obs:	exp:
ttt	F	0	0.49
ttc	F	1	0.51
tta	L	0	2.32
ttg	L	0	4.13
tct	S	1	52.93
tcc	S	325	49.02
tca	S	2	88.16
tcg	S	0	12.91
ctt	L	0	4.18
ctc	L	19	2.93
cta	L	1	3.07
ctg	L	0	3.36
cct	P	65	21797.00
ccc	P	59546	12263.00
cca	P	5793	22099.00
ccg	P	6	9251.00
cat	H	0	1.13
cac	H	2	0.87
act	T	0	2.79
acc	T	9	1.59
aca	T	0	3.89
acg	T	0	0.72
aat	N	0	1.12
aac	N	2	0.88
agt	S	0	57.32
agc	S	0	67.67
gct	A	0	0.55
gcc	A	2	0.50
gca	A	0	0.81
gcg	A	0	0.15
---	--	------	------
mPD		0.17	0.73
		nPD:	0.24
		N. weight:	1.5
		Sc. PD:	0.21
		Sc. rank:	58870.0

,

HA

Pos.	322	obs:	exp:
tct	S	0	3.39
tcc	S	0	3.14
tca	S	0	5.64
tcg	S	0	0.83
tat	Y	1	0.53
tac	Y	0	0.47
cct	P	2	0.67
ccc	P	0	0.37
cca	P	0	0.68
ccg	P	0	0.28
cat	H	2	1.13
cac	H	0	0.87
att	I	2	0.70
atc	I	0	0.53
ata	I	0	0.77
act	T	7	2.79
acc	T	2	1.59
aca	T	0	3.89
acg	T	0	0.72
aat	N	65667	36776.00
aac	N	66	28957.00
aaa	K	1	0.66
aag	K	0	0.34
agt	S	21	3.67
agc	S	0	4.33
gat	D	3	1.70
gac	D	0	1.30
---	--	------	------
mPD		0.0034	0.49
		nPD:	0.01
		N. weight:	0.63
		Sc. PD:	-0.046
		Sc. rank:	-22780.0

,

HA

Pos.	323	obs:	exp:
tct	S	0	0.32
tcc	S	0	0.30
tca	S	0	0.54
tcg	S	0	0.08
tat	Y	0	3.16
tac	Y	6	2.84
act	T	0	0.31
acc	T	0	0.18
aca	T	1	0.43
acg	T	0	0.08
aat	N	0	106.90
aac	N	191	84.14
agt	S	0	0.35
agc	S	2	0.41
gat	D	454	36875.00
gac	D	64781	28360.00
gaa	E	252	152.10
gag	E	6	105.90
ggt	G	1	10.90
ggc	G	80	11.30
gga	G	0	37.39
ggg	G	0	21.40
---	--	------	------
mPD		0.030	0.51
		nPD:	0.06
		N. weight:	0.87
		Sc. PD:	-0.02
		Sc. rank:	17751.0

,

HA

Pos.	324	obs:	exp:
tta	L	0	0.23
ttg	L	0	0.41
ctt	L	0	0.42
ctc	L	2	0.29
cta	L	0	0.31
ctg	L	0	0.34
cgt	R	0	1.15
cgc	R	0	2.09
cga	R	0	5.29
cgg	R	0	4.98
att	I	0	0.70
atc	I	0	0.53
ata	I	2	0.77
aat	N	0	0.56
aac	N	1	0.44
aaa	K	64993	43201.00
aag	K	718	22510.00
aga	R	57	28.45
agg	R	1	16.04
---	--	------	------
mPD		0.024	0.45
		nPD:	0.05
		N. weight:	0.41
		Sc. PD:	-0.012
		Sc. rank:	7040.6

,

HA

Pos.	325	obs:	exp:
tct	S	0	0.32
tcc	S	0	0.30
tca	S	2	0.54
tcg	S	0	0.08
cct	P	329	21917.00
ccc	P	119	12330.00
cca	P	53702	22221.00
ccg	P	11621	9302.00
act	T	0	0.31
acc	T	0	0.18
aca	T	1	0.43
acg	T	0	0.08
agt	S	0	0.35
agc	S	0	0.41
---	--	------	------
mPD		0.30	0.72
		nPD:	0.42
		N. weight:	0.81
		Sc. PD:	0.26
		Sc. rank:	37909.0

,

HA

Pos.	326	obs:	exp:
ttt	F	1260	31992.00
ttc	F	64510	33778.00
tta	L	0	0.46
ttg	L	0	0.83
ctt	L	0	0.84
ctc	L	4	0.59
cta	L	0	0.61
ctg	L	0	0.67
---	--	------	------
mPD		0.038	0.50
		nPD:	0.08
		N. weight:	0.63
		Sc. PD:	-0.0047
		Sc. rank:	14935.0

,

HA

Pos.	327	obs:	exp:
cat	H	1	0.56
cac	H	0	0.44
caa	Q	65710	40206.00
cag	Q	63	25567.00
---	--	------	------
mPD		0.0019	0.48
		nPD:	0.
		N. weight:	0.54
		Sc. PD:	-0.04
		Sc. rank:	-23879.0

,

HA

Pos.	328	obs:	exp:
tct	S	0	1.45
tcc	S	0	1.34
tca	S	0	2.42
tcg	S	0	0.35
cat	H	5	2.82
cac	H	0	2.18
aat	N	65496	36790.00
aac	N	263	28969.00
aaa	K	0	0.66
aag	K	1	0.34
agt	S	9	1.57
agc	S	0	1.86
---	--	------	------
mPD		0.0084	0.49
		nPD:	0.02
		N. weight:	0.61
		Sc. PD:	-0.039
		Sc. rank:	-8367.2

,

HA

Pos.	329	obs:	exp:
tta	L	3	0.35
ttg	L	0	0.62
ctt	L	0	0.63
ctc	L	0	0.44
cta	L	0	0.46
ctg	L	0	0.50
att	I	0	17.20
atc	I	0	12.88
ata	I	49	18.92
gtt	V	23	16462.00
gtc	V	101	13391.00
gta	V	64009	14245.00
gtg	V	1588	21624.00
gct	A	0	0.27
gcc	A	1	0.25
gca	A	0	0.40
gcg	A	0	0.07
---	--	------	------
mPD		0.052	0.74
		nPD:	0.07
		N. weight:	1.5
		Sc. PD:	-0.019
		Sc. rank:	34059.0

,

HA

Pos.	330	obs:	exp:
tct	S	0	0.81
tcc	S	0	0.75
tca	S	0	1.34
tcg	S	0	0.20
cat	H	2	2.82
cac	H	3	2.18
aat	N	75	36790.00
aac	N	65683	28968.00
agt	S	0	0.87
agc	S	5	1.03
gat	D	0	3.39
gac	D	6	2.61
---	--	------	------
mPD		0.0028	0.49
		nPD:	0.01
		N. weight:	0.9
		Sc. PD:	-0.066
		Sc. rank:	-35286.0

,

HA

Pos.	331	obs:	exp:
tct	S	0	0.16
tcc	S	0	0.15
tca	S	0	0.27
tcg	S	0	0.04
tgg	W	3	3.00
cct	P	0	0.67
ccc	P	0	0.37
cca	P	0	0.68
ccg	P	2	0.28
cgt	R	0	1296.00
cgc	R	0	2352.00
cga	R	0	5965.00
cgg	R	6	5614.00
atg	M	4	4.00
aaa	K	2	251.10
aag	K	380	130.90
agt	S	1	0.17
agc	S	0	0.21
aga	R	483	32067.00
agg	R	64890	18084.00
ggt	G	0	0.40
ggc	G	0	0.42
gga	G	0	1.38
ggg	G	3	0.79
---	--	------	------
mPD		0.027	0.90
		nPD:	0.03
		N. weight:	1.4
		Sc. PD:	-0.069
		Sc. rank:	4374.6

,

HA

Pos.	332	obs:	exp:
ttt	F	0	0.49
ttc	F	1	0.51
tta	L	0	0.12
ttg	L	0	0.21
ctt	L	0	0.21
ctc	L	1	0.15
cta	L	0	0.15
ctg	L	0	0.17
att	I	81	23049.00
atc	I	65510	17253.00
ata	I	68	25357.00
atg	M	1	1.00
act	T	0	1.55
acc	T	5	0.88
aca	T	0	2.16
acg	T	0	0.40
aat	N	0	1.12
aac	N	2	0.88
gtt	V	2	26.30
gtc	V	103	21.39
gta	V	0	22.76
gtg	V	0	34.55
---	--	------	------
mPD		0.0081	0.66
		nPD:	0.01
		N. weight:	1.5
		Sc. PD:	-0.099
		Sc. rank:	-35805.0

,

HA

Pos.	333	obs:	exp:
tct	S	0	0.16
tcc	S	0	0.15
tca	S	1	0.27
tcg	S	0	0.04
att	I	0	1.05
atc	I	0	0.79
ata	I	3	1.16
act	T	24	20420.00
acc	T	33	11625.00
aca	T	65666	28457.00
acg	T	31	5252.00
aaa	K	1	0.66
aag	K	0	0.34
agt	S	0	0.17
agc	S	0	0.21
gct	A	0	4.09
gcc	A	0	3.75
gca	A	15	6.07
gcg	A	0	1.10
---	--	------	------
mPD		0.0033	0.68
		nPD:	0.
		N. weight:	0.91
		Sc. PD:	-0.068
		Sc. rank:	-38801.0

,

HA

Pos.	334	obs:	exp:
ttt	F	0	0.97
ttc	F	2	1.03
tct	S	0	0.32
tcc	S	1	0.30
tca	S	0	0.54
tcg	S	1	0.08
tat	Y	1327	34662.00
tac	Y	64432	31097.00
tgt	C	0	0.39
tgc	C	1	0.61
cat	H	0	3.38
cac	H	6	2.62
att	I	2	0.70
atc	I	0	0.53
ata	I	0	0.77
aat	N	0	1.12
aac	N	2	0.88
agt	S	0	0.35
agc	S	0	0.41
---	--	------	------
mPD		0.040	0.50
		nPD:	0.08
		N. weight:	0.72
		Sc. PD:	-0.002
		Sc. rank:	17868.0

,

HA			
Pos.	335	obs:	exp:
ggt	G	68	8855.00
ggc	G	1	9180.00
gga	G	280	30362.00
ggg	G	65425	17377.00
---	--	------	------
mPD		0.011	0.68
		nPD:	0.02
		N. weight:	1.4
		Sc. PD:	-0.092
		Sc. rank:	-23284.0

,

HA			
Pos.	336	obs:	exp:
tct	S	0	0.16
tcc	S	1	0.15
tca	S	0	0.27
tcg	S	0	0.04
cct	P	0	9.66
ccc	P	29	5.44
cca	P	0	9.80
ccg	P	0	4.10
act	T	2	115.80
acc	T	365	65.95
aca	T	6	161.40
acg	T	0	29.79
aat	N	0	0.56
aac	N	1	0.44
aaa	K	2	1.31
aag	K	0	0.69
agt	S	0	0.17
agc	S	0	0.21
gtt	V	0	10.27
gtc	V	40	8.35
gta	V	1	8.89
gtg	V	0	13.49
gct	A	118	17799.00
gcc	A	59101	16316.00
gca	A	6086	26417.00
gcg	A	1	4774.00
gat	D	0	11.31
gac	D	20	8.69
gaa	E	1	0.59
gag	E	0	0.41
---	--	------	------
mPD		0.19	0.71
		nPD:	0.26
		N. weight:	1.1
		Sc. PD:	0.19
		Sc. rank:	47307.0

,

HA			
Pos.	337	obs:	exp:
tgt	C	65444	25704.00
tgc	C	329	40069.00
cgt	R	1	0.02
cgc	R	0	0.04
cga	R	0	0.09
cgg	R	0	0.09
aga	R	0	0.49
agg	R	0	0.28
---	--	------	------
mPD		0.010	0.48
		nPD:	0.02
		N. weight:	1.
		Sc. PD:	-0.059
		Sc. rank:	-6161.6

,

HA			
Pos.	338	obs:	exp:
tct	S	0	1.29
tcc	S	6	1.20
tca	S	2	2.15
tcg	S	0	0.31
cct	P	245	21915.00
ccc	P	65465	12329.00
cca	P	54	22219.00
ccg	P	0	9301.00
agt	S	0	1.40
agc	S	0	1.65
gct	A	0	0.55
gcc	A	2	0.50
gca	A	0	0.81
gcg	A	0	0.15
---	--	------	------
mPD		0.0094	0.72
		nPD:	0.01
		N. weight:	1.8
		Sc. PD:	-0.12
		Sc. rank:	-38945.0

,

HA			
Pos.	339	obs:	exp:
cgt	R	0	1302.00
cgc	R	0	2363.00
cga	R	16	5991.00
cgg	R	0	5638.00
att	I	0	4.21
atc	I	0	3.15
ata	I	12	4.63
aaa	K	23	64.43
aag	K	75	33.57
aga	R	65338	32207.00
agg	R	309	18162.00
ggt	G	0	0.13
ggc	G	0	0.14
gga	G	1	0.46
ggg	G	0	0.26
---	--	------	------
mPD		0.015	0.89
		nPD:	0.02
		N. weight:	0.75
		Sc. PD:	-0.047
		Sc. rank:	-9873.6

,

HA			
Pos.	340	obs:	exp:
ttt	F	12	5.84
ttc	F	0	6.16
tat	Y	63042	34662.00
tac	Y	2717	31097.00
cat	H	3	1.69
cac	H	0	1.31
---	--	------	------
mPD		0.080	0.50
		nPD:	0.16
		N. weight:	0.52
		Sc. PD:	0.038
		Sc. rank:	18701.0

,

HA			
Pos.	341	obs:	exp:
ttt	F	1	0.49
ttc	F	0	0.51
att	I	363	129.20
atc	I	5	96.70
ata	I	0	142.10
gtt	V	64749	16382.00
gtc	V	620	13327.00
gta	V	23	14176.00
gtg	V	13	21520.00
---	--	------	------
mPD		0.031	0.75
		nPD:	0.04
		N. weight:	1.5
		Sc. PD:	-0.059
		Sc. rank:	18646.0

,

HA			
Pos.	342	obs:	exp:
cgt	R	0	1.86
cgc	R	0	3.38
cga	R	0	8.58
cgg	R	0	8.07
act	T	0	1.24
acc	T	0	0.71
aca	T	0	1.73
acg	T	4	0.32
aat	N	2	1.12
aac	N	0	0.88
aaa	K	505	43176.00
aag	K	65168	22497.00
aga	R	0	46.11
agg	R	94	26.00
gaa	E	0	0.59
gag	E	1	0.41
---	--	------	------
mPD		0.018	0.45
		nPD:	0.04
		N. weight:	1.1
		Sc. PD:	-0.046
		Sc. rank:	12955.0

,

HA

Pos.	343	obs:	exp:
tta	L	0	1.86
ttg	L	0	3.31
tct	S	0	0.32
tcc	S	0	0.30
tca	S	0	0.54
tcg	S	0	0.08
tat	Y	4	2.11
tac	Y	0	1.89
ctt	L	10	3.35
ctc	L	0	2.35
cta	L	2	2.46
ctg	L	4	2.69
cat	H	22784	12873.00
cac	H	38	9949.00
caa	Q	39102	26240.00
cag	Q	3824	16686.00
aaa	K	4	2.63
aag	K	0	1.37
agt	S	0	0.35
agc	S	2	0.41
mPD		0.52	0.72
		nPD:	0.73
		N. weight:	0.38
		Sc. PD:	0.24
		Sc. rank:	19524.0

HA

Pos.	344	obs:	exp:
tct	S	0	9697.00
tcc	S	0	8980.00
tca	S	0	16152.00
tcg	S	0	2365.00
tgt	C	0	0.78
tgc	C	2	1.22
cat	H	0	2.82
cac	H	5	2.18
cgt	R	0	1.76
cgc	R	7	3.20
cga	R	0	8.12
cgg	R	0	7.64
att	I	0	0.70
atc	I	2	0.53
ata	I	0	0.77
act	T	0	1.86
acc	T	4	1.06
aca	T	2	2.60
acg	T	0	0.48
aat	N	7	3081.00
aac	N	5500	2426.00
aaa	K	7	4.60
aag	K	0	2.40
agt	S	124	10501.00
agc	S	59968	12397.00
aga	R	80	43.65
agg	R	2	24.62
gat	D	0	2.83
gac	D	5	2.17
ggt	G	0	7.94
ggc	G	58	8.23
gga	G	1	27.24
ggg	G	0	15.59
mPD		0.16	1.7
		nPD:	0.09
		N. weight:	1.7
		Sc. PD:	0.018
		Sc. rank:	45874.0

HA

Pos.	345	obs:	exp:
tct	S	8	1.61
tcc	S	0	1.49
tca	S	0	2.69
tcg	S	0	0.39
cct	P	2	0.67
ccc	P	0	0.37
cca	P	0	0.68
ccg	P	0	0.28
act	T	53257	20419.00
acc	T	12465	11625.00
aca	T	27	28455.00
acg	T	1	5251.00
aat	N	8	4.48
aac	N	0	3.52
aaa	K	2	1.31
aag	K	0	0.69
agt	S	2	1.75
agc	S	0	2.06
gct	A	1	0.55
gcc	A	1	0.50
gca	A	0	0.81
gcg	A	0	0.15
mPD		0.31	0.68
		nPD:	0.45
		N. weight:	0.87
		Sc. PD:	0.31
		Sc. rank:	41788.0

HA

Pos.	346	obs:	exp:
tta	L	1	7632.00
ttg	L	139	13598.00
ctt	L	24	13759.00
ctc	L	1	9645.00
cta	L	306	10100.00
ctg	L	65301	11038.00
atg	M	1	1.00
gtt	V	0	0.25
gtc	V	0	0.20
gta	V	0	0.22
gtg	V	1	0.33
mPD		0.014	1.2
		nPD:	0.01
		N. weight:	1.9
		Sc. PD:	-0.13
		Sc. rank:	-45854.0

HA

Pos.	347	obs:	exp:
cgt	R	0	0.54
cgc	R	0	0.97
cga	R	0	2.46
cgg	R	0	2.32
aaa	K	65591	43224.00
aag	K	156	22523.00
aga	R	27	13.24
agg	R	0	7.47
mPD		0.0056	0.45
		nPD:	0.01
		N. weight:	0.45
		Sc. PD:	-0.03
		Sc. rank:	-10828.0

HA

Pos.	348	obs:	exp:
ttt	F	1	0.49
ttc	F	0	0.51
tta	L	122	7631.00
ttg	L	65002	13596.00
tct	S	0	0.32
tcc	S	0	0.30
tca	S	0	0.54
tcg	S	2	0.08
ctt	L	0	13757.00
ctc	L	0	9644.00
cta	L	0	10098.00
ctg	L	639	11037.00
atg	M	6	6.00
agt	S	0	0.35
agc	S	0	0.41
gtt	V	0	0.50
gtc	V	0	0.41
gta	V	0	0.43
gtg	V	2	0.66
mPD		0.023	1.2
		nPD:	0.02
		N. weight:	1.7
		Sc. PD:	-0.1
		Sc. rank:	-13758.0

HA

Pos.	349	obs:	exp:
cgt	R	0	0.02
cgc	R	0	0.04
cga	R	0	0.09
cgg	R	0	0.09
aga	R	0	0.49
agg	R	1	0.28
gct	A	224	17927.00
gcc	A	8	16433.00
gca	A	65486	26605.00
gcg	A	55	4808.00
mPD		0.0088	0.69
		nPD:	0.01
		N. weight:	0.97
		Sc. PD:	-0.065
		Sc. rank:	-22142.0

HA

Pos.	350	obs:	exp:
cct	P	0	0.33
ccc	P	0	0.19
cca	P	1	0.34
ccg	P	0	0.14
att	I	0	1.05
atc	I	0	0.79
ata	I	3	1.16
act	T	46	20425.00
acc	T	7	11628.00
aca	T	65657	28463.00
acg	T	59	5253.00
gct	A	0	0.27
gcc	A	0	0.25
gca	A	1	0.40
gcg	A	0	0.07
mPD		0.0036	0.68
		nPD:	0.01
		N. weight:	0.91
		Sc. PD:	-0.068
		Sc. rank:	-37898.0

HA

Pos.	351	obs:	exp:
gaa	E	2	1.18
gag	E	0	0.82
ggt	G	3	8855.00
ggc	G	33	9180.00
gga	G	63404	30361.00
ggg	G	2332	17376.00
mPD		0.070	0.68
		nPD:	0.1
		N. weight:	0.7
		Sc. PD:	0.013
		Sc. rank:	20572.0

HA

Pos.	352	obs:	exp:
tta	L	0	0.23
ttg	L	2	0.41
ctt	L	0	0.42
ctc	L	0	0.29
cta	L	0	0.31
ctg	L	0	0.34
atg	M	65771	65771.00
gtt	V	0	0.25
gtc	V	0	0.20
gta	V	0	0.22
gtg	V	1	0.33
mPD		0.000091	0.00015
		nPD:	0.61
		N. weight:	0.000072
		Sc. PD:	0.000036
		Sc. rank:	3.6

HA

Pos.	353	obs:	exp:
cct	P	0	0.33
ccc	P	0	0.19
cca	P	1	0.34
ccg	P	0	0.14
cgt	R	13	1304.00
cgc	R	2	2367.00
cga	R	63004	6001.00
cgg	R	1015	5648.00
aga	R	1736	32261.00
agg	R	3	18193.00
mPD		0.082	0.89
		nPD:	0.09
		N. weight:	2.4
		Sc. PD:	0.022
		Sc. rank:	64687.0

HA

Pos.	354	obs:	exp:
aat	N	65532	36798.00
aac	N	241	28975.00
gat	D	1	0.57
gac	D	0	0.43
mPD		0.0073	0.49
		nPD:	0.01
		N. weight:	0.62
		Sc. PD:	-0.04
		Sc. rank:	-10556.0

HA			
Pos.	355	obs:	exp:
tta	L	5	0.70
ttg	L	0	1.24
ctt	L	0	1.26
ctc	L	0	0.88
cta	L	1	0.92
ctg	L	0	1.01
att	I	0	58.62
atc	I	0	43.88
ata	I	167	64.49
gtt	V	16	16429.00
gtc	V	11	13364.00
gta	V	63610	14216.00
gtg	V	1952	21580.00
gct	A	0	3.00
gcc	A	0	2.75
gca	A	11	4.45
gcg	A	0	0.80
gaa	E	1	0.59
gag	E	0	0.41
---	--	------	------
mPD		0.064	0.75
		nPD:	0.09
		N. weight:	1.5
		Sc. PD:	0.0036
		Sc. rank:	38981.0

,

HA			
Pos.	356	obs:	exp:
tta	L	0	0.35
ttg	L	0	0.62
ctt	L	0	0.63
ctc	L	0	0.44
cta	L	3	0.46
ctg	L	0	0.50
cct	P	207	21914.00
ccc	P	6	12328.00
cca	P	65457	22218.00
ccg	P	91	9301.00
caa	Q	8	5.50
cag	Q	1	3.50
act	T	0	0.31
acc	T	0	0.18
aca	T	1	0.43
acg	T	0	0.08
---	--	------	------
mPD		0.0096	0.72
		nPD:	0.01
		N. weight:	1.2
		Sc. PD:	-0.077
		Sc. rank:	-24263.0

,

HA			
Pos.	357	obs:	exp:
tct	S	2	0.32
tcc	S	0	0.30
tca	S	0	0.54
tcg	S	0	0.08
caa	Q	0	0.61
cag	Q	1	0.39
aaa	K	3	4.60
aag	K	4	2.40
agt	S	0	0.35
agc	S	0	0.41
gct	A	0	0.27
gcc	A	0	0.25
gca	A	0	0.40
gcg	A	1	0.07
gat	D	18	10.17
gac	D	0	7.83
gaa	E	436	38753.00
gag	E	65296	26979.00
ggt	G	0	1.75
ggc	G	0	1.81
gga	G	0	6.00
ggg	G	13	3.43
---	--	------	------
mPD		0.015	0.49
		nPD:	0.03
		N. weight:	0.94
		Sc. PD:	-0.047
		Sc. rank:	3635.8

,

HA			
Pos.	358	obs:	exp:
cgt	R	0	147.00
cgc	R	0	266.80
cga	R	0	676.50
cgg	R	0	636.70
att	I	2	1.05
atc	I	0	0.79
ata	I	1	1.16
act	T	0	1.86
acc	T	0	1.06
aca	T	6	2.60
acg	T	0	0.48
aaa	K	58255	38359.00
aag	K	92	19988.00
aga	R	7408	3637.00
agg	R	7	2051.00
ggt	G	0	0.40
ggc	G	0	0.42
gga	G	3	1.38
ggg	G	0	0.79
---	--	------	------
mPD		0.20	0.71
		nPD:	0.29
		N. weight:	0.49
		Sc. PD:	0.094
		Sc. rank:	20647.0

,

HA			
Pos.	359	obs:	exp:
cat	H	1	2.26
cac	H	3	1.74
caa	Q	65577	40189.00
cag	Q	169	25557.00
cgt	R	0	0.18
cgc	R	0	0.32
cga	R	9	0.82
cgg	R	0	0.77
aaa	K	12	7.89
aag	K	0	4.11
aga	R	0	4.41
agg	R	0	2.49
gaa	E	1	0.59
gag	E	0	0.41
---	-	2	2.00
---	--	------	------
mPD		0.0059	0.48
		nPD:	0.01
		N. weight:	0.53
		Sc. PD:	-0.035
		Sc. rank:	-12515.0

,

HA			
Pos.	360	obs:	exp:
tct	S	2	1.78
tcc	S	0	1.64
tca	S	0	2.96
tcg	S	0	0.43
cct	P	1	3.67
ccc	P	10	2.06
cca	P	0	3.72
ccg	P	0	1.56
att	I	13	4.91
atc	I	1	3.68
ata	I	0	5.41
act	T	53755	20389.00
acc	T	11873	11608.00
aca	T	18	28413.00
acg	T	7	5244.00
aat	N	12	6.71
aac	N	0	5.29
agt	S	9	1.92
agc	S	0	2.27
gct	A	44	19.90
gcc	A	29	18.24
gca	A	0	29.53
gcg	A	0	5.34
---	--	------	------
mPD		0.30	0.68
		nPD:	0.44
		N. weight:	0.88
		Sc. PD:	0.3
		Sc. rank:	41826.0

,

HA			
Pos.	361	obs:	exp:
cgt	R	0	1304.00
cgc	R	0	2366.00
cga	R	6	5999.00
cgg	R	0	5645.00
aaa	K	27	18.41
aag	K	1	9.59
aga	R	59783	32247.00
agg	R	5957	18185.00
---	--	------	------
mPD		0.17	0.89
		nPD:	0.19
		N. weight:	0.51
		Sc. PD:	0.05
		Sc. rank:	19280.0

,

HA			
Pos.	362	obs:	exp:
gtt	V	0	0.25
gtc	V	1	0.20
gta	V	0	0.22
gtg	V	0	0.33
ggt	G	124	8855.00
ggc	G	65609	9180.00
gga	G	40	30362.00
ggg	G	0	17377.00
---	--	------	------
mPD		0.0050	0.68
		nPD:	0.01
		N. weight:	2.2
		Sc. PD:	-0.16
		Sc. rank:	-74019.0

,

HA			
Pos.	363	obs:	exp:
tta	L	1	5.34
ttg	L	0	9.51
ctt	L	0	9.62
ctc	L	0	6.75
cta	L	45	7.06
ctg	L	0	7.72
att	I	8	23069.00
atc	I	14	17268.00
ata	I	65694	25379.00
atg	M	6	6.00
gtt	V	0	1.50
gtc	V	0	1.22
gta	V	5	1.30
gtg	V	1	1.97
---	--	------	------
mPD		0.0025	0.66
		nPD:	0.
		N. weight:	1.
		Sc. PD:	-0.079
		Sc. rank:	-47791.0

,

HA			
Pos.	364	obs:	exp:
ttt	F	65118	31992.00
ttc	F	651	33777.00
tta	L	2	0.58
ttg	L	2	1.03
ctt	L	1	1.05
ctc	L	0	0.73
cta	L	0	0.77
ctg	L	0	0.84
---	--	------	------
mPD		0.020	0.50
		nPD:	0.04
		N. weight:	0.73
		Sc. PD:	-0.031
		Sc. rank:	8284.3

,

HA			
Pos.	365	obs:	exp:
tct	S	0	1.13
tcc	S	0	1.05
tca	S	0	1.88
tcg	S	0	0.28
agt	S	0	1.22
agc	S	7	1.44
gat	D	0	0.57
gac	D	1	0.43
ggt	G	1893	8854.00
ggc	G	63719	9179.00
gga	G	153	30359.00
ggg	G	1	17375.00
---	--	------	------
mPD		0.061	0.68
		nPD:	0.09
		N. weight:	2.
		Sc. PD:	0.011
		Sc. rank:	52528.0

,

HA			
Pos.	366	obs:	exp:
cct	P	0	0.33
ccc	P	0	0.19
cca	P	1	0.34
ccg	P	0	0.14
act	T	0	0.31
acc	T	0	0.18
aca	T	1	0.43
acg	T	0	0.08
gtt	V	0	0.25
gtc	V	0	0.20
gta	V	1	0.22
gtg	V	0	0.33
gct	A	17	17926.00
gcc	A	23	16432.00
gca	A	65578	26605.00
gcg	A	153	4808.00
---	--	------	------
mPD		0.0059	0.69
		nPD:	0.01
		N. weight:	0.98
		Sc. PD:	-0.07
		Sc. rank:	-32167.0

,

HA			
Pos.	367	obs:	exp:
att	I	179	23083.00
atc	I	2158	17278.00
ata	I	63419	25395.00
aaa	K	8	5.26
aag	K	0	2.74
gtt	V	0	2.50
gtc	V	0	2.04
gta	V	10	2.17
gtg	V	0	3.29
---	--	------	------
mPD		0.069	0.66
		nPD:	0.11
		N. weight:	0.89
		Sc. PD:	0.018
		Sc. rank:	27105.0

,

HA			
Pos.	368	obs:	exp:
gct	A	1072	17927.00
gcc	A	2	16433.00
gca	A	1448	26605.00
gcg	A	63251	4808.00
gaa	E	0	0.59
gag	E	1	0.41
---	--	------	------
mPD		0.074	0.69
		nPD:	0.11
		N. weight:	2.6
		Sc. PD:	0.06
		Sc. rank:	81276.0

,

HA			
Pos.	369	obs:	exp:
cgt	R	1	0.02
cgc	R	0	0.04
cga	R	0	0.09
cgg	R	0	0.09
aga	R	0	0.49
agg	R	0	0.28
gtt	V	4	1.00
gtc	V	0	0.82
gta	V	0	0.87
gtg	V	0	1.32
gat	D	2	1.13
gac	D	0	0.87
ggt	G	65030	8854.00
ggc	G	471	9179.00
gga	G	6	30359.00
ggg	G	260	17375.00
---	--	------	------
mPD		0.022	0.68
		nPD:	0.03
		N. weight:	2.1
		Sc. PD:	-0.1
		Sc. rank:	10149.0

,

HA			
Pos.	370	obs:	exp:
ttt	F	89	31994.00
ttc	F	65684	33779.00
tta	L	0	0.12
ttg	L	0	0.21
ctt	L	0	0.21
ctc	L	1	0.15
cta	L	0	0.15
ctg	L	0	0.17
---	--	------	------
mPD		0.0027	0.50
		nPD:	0.01
		N. weight:	0.72
		Sc. PD:	-0.054
		Sc. rank:	-29272.0

,

HA			
Pos.	371	obs:	exp:
tta	L	2	0.23
ttg	L	0	0.41
ctt	L	0	0.42
ctc	L	0	0.29
cta	L	0	0.31
ctg	L	0	0.34
att	I	40	23087.00
atc	I	32	17281.00
ata	I	65696	25399.00
atg	M	2	2.00
gtt	V	0	0.50
gtc	V	0	0.41
gta	V	2	0.43
gtg	V	0	0.66
---	--	------	------
mPD		0.0024	0.66
		nPD:	0.
		N. weight:	1.
		Sc. PD:	-0.079
		Sc. rank:	-47868.0

,

HA			
Pos.	372	obs:	exp:
gaa	E	65736	38778.00
gag	E	38	26996.00
---	--	------	------
mPD		0.0012	0.48
		nPD:	0.
		N. weight:	0.58
		Sc. PD:	-0.045
		Sc. rank:	-28131.0

,

HA			
Pos.	373	obs:	exp:
tct	S	0	0.16
tcc	S	0	0.15
tca	S	0	0.27
tcg	S	0	0.04
tat	Y	2	1.05
tac	Y	0	0.95
cat	H	1	0.56
cac	H	0	0.44
aat	N	65701	36795.00
aac	N	67	28973.00
agt	S	1	0.17
agc	S	0	0.21
ggt	G	0	0.27
ggc	G	0	0.28
gga	G	0	0.92
ggg	G	2	0.53
---	--	------	------
mPD		0.0023	0.49
		nPD:	0.
		N. weight:	0.63
		Sc. PD:	-0.047
		Sc. rank:	-27131.0

,

HA			
Pos.	374	obs:	exp:
gtt	V	4	1.00
gtc	V	0	0.82
gta	V	0	0.87
gtg	V	0	1.32
ggt	G	54728	8854.00
ggc	G	298	9179.00
gga	G	10741	30361.00
ggg	G	3	17376.00
---	--	------	------
mPD		0.28	0.68
		nPD:	0.41
		N. weight:	1.5
		Sc. PD:	0.46
		Sc. rank:	68000.0

,

HA			
Pos.	375	obs:	exp:
tta	L	0	0.12
ttg	L	1	0.21
tgg	W	65772	65772.00
ctt	L	0	0.21
ctc	L	0	0.15
cta	L	0	0.15
ctg	L	0	0.17
cgt	R	0	0.02
cgc	R	0	0.04
cga	R	0	0.09
cgg	R	1	0.09
aga	R	0	0.49
agg	R	0	0.28
---	--	------	------
mPD		0.000061	0.00012
		nPD:	0.51
		N. weight:	0.000068
		Sc. PD:	0.000027
		Sc. rank:	3.3

,

HA			
Pos.	376	obs:	exp:
act	T	0	0.62
acc	T	0	0.35
aca	T	2	0.87
acg	T	0	0.16
aaa	K	0	0.66
aag	K	1	0.34
gtt	V	0	1.00
gtc	V	0	0.82
gta	V	0	0.87
gtg	V	4	1.32
gct	A	0	0.27
gcc	A	0	0.25
gca	A	0	0.40
gcg	A	1	0.07
gat	D	4	2.26
gac	D	0	1.74
gaa	E	2040	38770.00
gag	E	63721	26991.00
ggt	G	0	0.13
ggc	G	0	0.14
gga	G	0	0.46
ggg	G	1	0.26
---	--	------	------
mPD		0.061	0.48
		nPD:	0.13
		N. weight:	0.82
		Sc. PD:	0.033
		Sc. rank:	27486.0

,

HA			
Pos.	377	obs:	exp:
cgt	R	0	0.02
cgc	R	0	0.04
cga	R	0	0.09
cgg	R	0	0.09
aga	R	1	0.49
agg	R	0	0.28
gaa	E	2	1.18
gag	E	0	0.82
ggt	G	61	8854.00
ggc	G	72	9179.00
gga	G	65357	30361.00
ggg	G	281	17376.00
---	--	------	------
mPD		0.013	0.68
		nPD:	0.02
		N. weight:	0.81
		Sc. PD:	-0.05
		Sc. rank:	-8586.5

,

HA			
Pos.	378	obs:	exp:
tta	L	2	477.30
ttg	L	4082	850.30
ctt	L	0	860.40
ctc	L	0	603.10
cta	L	0	631.60
ctg	L	29	690.30
att	I	0	0.35
atc	I	0	0.26
ata	I	1	0.39
atg	M	61660	61660.00
---	--	------	------
mPD		0.12	0.20
		nPD:	0.6
		N. weight:	0.11
		Sc. PD:	0.052
		Sc. rank:	5298.8

,

HA			
Pos.	379	obs:	exp:
tta	L	1	1.97
ttg	L	12	3.51
ctt	L	0	3.56
ctc	L	0	2.49
cta	L	0	2.61
ctg	L	4	2.85
att	I	0	27.73
atc	I	0	20.76
ata	I	79	30.51
atg	M	5876	5876.00
aaa	K	6	689.70
aag	K	1043	359.30
gtt	V	52	14716.00
gtc	V	1	11971.00
gta	V	671	12734.00
gtg	V	58028	19331.00
ggt	G	0	0.13
ggc	G	0	0.14
gga	G	0	0.46
ggg	G	1	0.26
---	--	------	------
mPD		0.25	0.95
		nPD:	0.26
		N. weight:	1.1
		Sc. PD:	0.18
		Sc. rank:	43881.0

,

HA			
Pos.	380	obs:	exp:
aat	N	19	10.63
aac	N	0	8.37
gat	D	64954	37169.00
gac	D	801	28586.00
---	--	------	------
mPD		0.025	0.49
		nPD:	0.05
		N. weight:	0.56
		Sc. PD:	-0.018
		Sc. rank:	9279.2

,

HA			
Pos.	381	obs:	exp:
tct	S	0	0.16
tcc	S	0	0.15
tca	S	0	0.27
tcg	S	0	0.04
agt	S	1	0.17
agc	S	0	0.21
ggt	G	64622	8855.00
ggc	G	1097	9180.00
gga	G	50	30362.00
ggg	G	4	17377.00
---	--	------	------
mPD		0.034	0.68
		nPD:	0.05
		N. weight:	2.1
		Sc. PD:	-0.066
		Sc. rank:	36564.0

,

HA			
Pos.	382	obs:	exp:
tct	S	0	0.16
tcc	S	0	0.15
tca	S	0	0.27
tcg	S	1	0.04
tgg	W	65769	65769.00
cgt	R	0	0.04
cgc	R	0	0.07
cga	R	0	0.18
cgg	R	1	0.17
agt	S	0	0.17
agc	S	0	0.21
aga	R	0	0.98
agg	R	1	0.55
ggt	G	0	0.27
ggc	G	0	0.28
gga	G	0	0.92
ggg	G	2	0.53
---	--	------	------
mPD		0.00015	0.00026
		nPD:	0.57
		N. weight:	0.00015
		Sc. PD:	0.00007
		Sc. rank:	7.5

,

HA			
Pos.	383	obs:	exp:
ttt	F	0	0.97
ttc	F	2	1.03
tct	S	0	0.16
tcc	S	0	0.15
tca	S	0	0.27
tcg	S	0	0.04
tat	Y	469	34668.00
tac	Y	65301	31102.00
aat	N	0	0.56
aac	N	1	0.44
agt	S	0	0.17
agc	S	1	0.21
---	--	------	------
mPD		0.014	0.50
		nPD:	0.03
		N. weight:	0.78
		Sc. PD:	-0.041
		Sc. rank:	2186.5

,

HA			
Pos.	384	obs:	exp:
tgt	C	1	0.39
tgc	C	0	0.61
aaa	K	3	1.97
aag	K	0	1.03
gat	D	1	0.57
gac	D	0	0.43
ggt	G	57670	8854.00
ggc	G	8077	9179.00
gga	G	15	30360.00
ggg	G	7	17376.00
---	--	------	------
mPD		0.22	0.68
		nPD:	0.32
		N. weight:	1.8
		Sc. PD:	0.4
		Sc. rank:	77012.0

,

HA			
Pos.	385	obs:	exp:
ttt	F	57	31984.00
ttc	F	65697	33770.00
tta	L	1	0.23
ttg	L	0	0.41
tct	S	0	0.48
tcc	S	0	0.45
tca	S	0	0.81
tcg	S	0	0.12
tat	Y	2	7.91
tac	Y	13	7.09
ctt	L	0	0.42
ctc	L	1	0.29
cta	L	0	0.31
ctg	L	0	0.34
agt	S	0	0.52
agc	S	3	0.62
---	--	------	------
mPD		0.0025	0.50
		nPD:	0.
		N. weight:	0.73
		Sc. PD:	-0.054
		Sc. rank:	-30577.0

,

HA			
Pos.	386	obs:	exp:
tgt	C	0	0.39
tgc	C	1	0.61
cat	H	0	1.13
cac	H	2	0.87
cgt	R	0	1304.00
cgc	R	0	2366.00
cga	R	0	6000.00
cgg	R	16	5647.00
act	T	0	0.31
acc	T	0	0.18
aca	T	0	0.43
acg	T	1	0.08
aaa	K	1	7.23
aag	K	10	3.77
aga	R	3071	32254.00
agg	R	62672	18189.00
---	--	------	------
mPD		0.090	0.89
		nPD:	0.1
		N. weight:	1.2
		Sc. PD:	0.021
		Sc. rank:	34420.0

,

HA			
Pos.	387	obs:	exp:
tta	L	0	0.12
ttg	L	0	0.21
tat	Y	2	1.05
tac	Y	0	0.95
ctt	L	1	0.21
ctc	L	0	0.15
cta	L	0	0.15
ctg	L	0	0.17
cat	H	65469	37097.00
cac	H	301	28673.00
caa	Q	1	0.61
cag	Q	0	0.39
---	--	------	------
mPD		0.0092	0.49
		nPD:	0.02
		N. weight:	0.6
		Sc. PD:	-0.037
		Sc. rank:	-6104.9

,

HA			
Pos.	388	obs:	exp:
tta	L	0	6.50
ttg	L	0	11.58
ctt	L	0	11.71
ctc	L	0	8.21
cta	L	56	8.60
ctg	L	0	9.40
cct	P	0	0.33
ccc	P	0	0.19
cca	P	1	0.34
ccg	P	0	0.14
cat	H	10	5.64
cac	H	0	4.36
caa	Q	65328	40158.00
cag	Q	368	25538.00
cgt	R	0	0.06
cgc	R	0	0.11
cga	R	2	0.27
cgg	R	1	0.26
aaa	K	8	5.26
aag	K	0	2.74
aga	R	0	1.47
agg	R	0	0.83
---	--	------	------
mPD		0.014	0.48
		nPD:	0.03
		N. weight:	0.51
		Sc. PD:	-0.027
		Sc. rank:	1044.6

,

HA			
Pos.	389	obs:	exp:
tat	Y	1	0.53
tac	Y	0	0.47
att	I	3	1.05
atc	I	0	0.79
ata	I	0	1.16
aat	N	62785	36796.00
aac	N	2985	28974.00
---	--	------	------
mPD		0.087	0.49
		nPD:	0.18
		N. weight:	0.45
		Sc. PD:	0.04
		Sc. rank:	16478.0

,

HA			
Pos.	390	obs:	exp:
ttt	F	44	21.40
ttc	F	0	22.60
tct	S	65530	10598.00
tcc	S	91	9815.00
tca	S	45	17653.00
tcg	S	11	2585.00
tat	Y	32	16.87
tac	Y	0	15.13
tgt	C	10	3.91
tgc	C	0	6.09
cct	P	3	1.00
ccc	P	0	0.56
cca	P	0	1.01
ccg	P	0	0.42
agt	S	0	11477.00
agc	S	0	13549.00
gct	A	6	1.64
gcc	A	0	1.50
gca	A	0	2.43
gcg	A	0	0.44
gaa	E	0	1.18
gag	E	2	0.82
---	--	------	------
mPD		0.0075	1.6
		nPD:	0.
		N. weight:	2.
		Sc. PD:	-0.15
		Sc. rank:	-85668.0

,

HA			
Pos.	391	obs:	exp:
caa	Q	0	1.22
cag	Q	2	0.78
aaa	K	0	1.97
aag	K	3	1.03
gtt	V	0	0.25
gtc	V	0	0.20
gta	V	0	0.22
gtg	V	1	0.33
gat	D	12	6.78
gac	D	0	5.22
gaa	E	6392	38767.00
gag	E	59364	26989.00
---	--	------	------
mPD		0.18	0.48
		nPD:	0.36
		N. weight:	0.59
		Sc. PD:	0.16
		Sc. rank:	26381.0

,

HA			
Pos.	392	obs:	exp:
ggt	G	3	8855.00
ggc	G	701	9180.00
gga	G	64483	30362.00
ggg	G	587	17377.00
---	--	------	------
mPD		0.039	0.68
		nPD:	0.06
		N. weight:	0.75
		Sc. PD:	-0.019
		Sc. rank:	14304.0

,

HA			
Pos.	393	obs:	exp:
tct	S	0	0.81
tcc	S	0	0.75
tca	S	2	1.34
tcg	S	0	0.20
cgt	R	0	1274.00
cgc	R	0	2313.00
cga	R	25	5865.00
cgg	R	2	5519.00
att	I	0	227.10
atc	I	0	170.00
ata	I	647	249.90
atg	M	15	15.00
act	T	0	247.80
acc	T	0	141.10
aca	T	798	345.40
acg	T	0	63.74
aaa	K	24	16.44
aag	K	1	8.56
agt	S	2	0.87
agc	S	1	1.03
aga	R	62567	31526.00
agg	R	1682	17779.00
gtt	V	0	1.00
gtc	V	0	0.82
gta	V	4	0.87
gtg	V	0	1.32
ggt	G	0	0.54
ggc	G	0	0.56
gga	G	4	1.85
ggg	G	0	1.06
---	--	------	------
mPD		0.096	0.93
		nPD:	0.1
		N. weight:	0.67
		Sc. PD:	0.013
		Sc. rank:	19961.0

,

HA			
Pos.	394	obs:	exp:
gaa	E	3	2.95
gag	E	2	2.05
ggt	G	5	8854.00
ggc	G	13	9179.00
gga	G	62705	30360.00
ggg	G	3046	17376.00
---	--	------	------
mPD		0.089	0.68
		nPD:	0.13
		N. weight:	0.67
		Sc. PD:	0.031
		Sc. rank:	22733.0

,

HA			
Pos.	395	obs:	exp:
tta	L	0	0.23
ttg	L	0	0.41
tat	Y	2	1.05
tac	Y	0	0.95
ctt	L	0	0.42
ctc	L	0	0.29
cta	L	2	0.31
ctg	L	0	0.34
caa	Q	65500	40204.00
cag	Q	270	25566.00
---	--	------	------
mPD		0.0084	0.48
		nPD:	0.02
		N. weight:	0.52
		Sc. PD:	-0.032
		Sc. rank:	-6712.1

,

HA			
Pos.	396	obs:	exp:
tct	S	0	0.97
tcc	S	0	0.90
tca	S	6	1.61
tcg	S	0	0.24
act	T	0	0.62
acc	T	0	0.35
aca	T	2	0.87
acg	T	0	0.16
agt	S	0	1.05
agc	S	0	1.24
gtt	V	0	2.25
gtc	V	0	1.83
gta	V	9	1.95
gtg	V	0	2.96
gct	A	62	17922.00
gcc	A	4	16429.00
gca	A	65487	26599.00
gcg	A	203	4807.00
ggt	G	1	0.13
ggc	G	0	0.14
gga	G	0	0.46
ggg	G	0	0.26
---	--	------	------
mPD		0.0087	0.69
		nPD:	0.01
		N. weight:	0.97
		Sc. PD:	-0.066
		Sc. rank:	-22422.0

,

HA			
Pos.	397	obs:	exp:
tct	S	0	0.16
tcc	S	0	0.15
tca	S	1	0.27
tcg	S	0	0.04
cct	P	0	0.33
ccc	P	0	0.19
cca	P	1	0.34
ccg	P	0	0.14
act	T	0	0.31
acc	T	0	0.18
aca	T	1	0.43
acg	T	0	0.08
agt	S	0	0.17
agc	S	0	0.21
gct	A	35	17926.00
gcc	A	9	16432.00
gca	A	65646	26605.00
gcg	A	81	4808.00
---	--	------	------
mPD		0.0039	0.69
		nPD:	0.01
		N. weight:	0.99
		Sc. PD:	-0.073
		Sc. rank:	-39436.0

,

HA			
Pos.	398	obs:	exp:
cat	H	1	0.56
cac	H	0	0.44
aat	N	3	1.68
aac	N	0	1.32
gct	A	2	0.55
gcc	A	0	0.50
gca	A	0	0.81
gcg	A	0	0.15
gat	D	65626	37174.00
gac	D	139	28591.00
gaa	E	1	0.59
gag	E	0	0.41
ggt	G	2	0.27
ggc	G	0	0.28
gga	G	0	0.92
ggg	G	0	0.53
---	--	------	------
mPD		0.0045	0.49
		nPD:	0.01
		N. weight:	0.61
		Sc. PD:	-0.043
		Sc. rank:	-19445.0

,

HA			
Pos.	399	obs:	exp:
ttt	F	9	12.16
ttc	F	16	12.84
tta	L	0	7628.00
ttg	L	0	13591.00
ctt	L	6383	13752.00
ctc	L	59313	9640.00
cta	L	40	10094.00
ctg	L	2	11033.00
cat	H	0	0.56
cac	H	1	0.44
cgt	R	2	0.04
cgc	R	0	0.07
cga	R	0	0.18
cgg	R	0	0.17
att	I	1	2.81
atc	I	7	2.10
ata	I	0	3.09
aga	R	0	0.98
agg	R	0	0.55
---	--	------	------
mPD		0.18	1.2
		nPD:	0.15
		N. weight:	1.7
		Sc. PD:	0.11
		Sc. rank:	60994.0

,

HA			
Pos.	400	obs:	exp:
caa	Q	5	3.06
cag	Q	0	1.94
cgt	R	0	0.79
cgc	R	0	1.44
cga	R	0	3.65
cgg	R	0	3.43
att	I	0	0.35
atc	I	0	0.26
ata	I	1	0.39
act	T	0	1.86
acc	T	0	1.06
aca	T	6	2.60
acg	T	0	0.48
aat	N	0	0.56
aac	N	1	0.44
aaa	K	65618	43204.00
aag	K	98	22512.00
aga	R	40	19.62
agg	R	0	11.06
gaa	E	5	2.95
gag	E	0	2.05
---	--	------	------
mPD		0.0047	0.45
		nPD:	0.01
		N. weight:	0.45
		Sc. PD:	-0.031
		Sc. rank:	-12956.0

,

HA			
Pos.	401	obs:	exp:
tct	S	0	10613.00
tcc	S	0	9829.00
tca	S	0	17678.00
tcg	S	0	2589.00
tgt	C	0	0.39
tgc	C	1	0.61
act	T	0	0.31
acc	T	1	0.18
aca	T	0	0.43
acg	T	0	0.08
agt	S	107	11494.00
agc	S	65664	13568.00
ggt	G	0	0.13
ggc	G	1	0.14
gga	G	0	0.46
ggg	G	0	0.26
---	--	------	------
mPD		0.0033	1.6
		nPD:	0.
		N. weight:	1.7
		Sc. PD:	-0.13
		Sc. rank:	-85611.0

,

HA			
Pos.	402	obs:	exp:
cgt	R	0	0.02
cgc	R	0	0.04
cga	R	0	0.09
cgg	R	0	0.09
act	T	64520	20426.00
acc	T	681	11629.00
aca	T	27	28465.00
acg	T	544	5253.00
aga	R	0	0.49
agg	R	1	0.28
gct	A	1	0.27
gcc	A	0	0.25
gca	A	0	0.40
gcg	A	0	0.07
---	--	------	------
mPD		0.038	0.68
		nPD:	0.06
		N. weight:	1.2
		Sc. PD:	-0.032
		Sc. rank:	21891.0

,

HA			
Pos.	403	obs:	exp:
caa	Q	65487	40204.00
cag	Q	283	25566.00
cgt	R	0	0.08
cgc	R	0	0.14
cga	R	4	0.36
cgg	R	0	0.34
aga	R	0	1.96
agg	R	0	1.11
---	--	------	------
mPD		0.0087	0.48
		nPD:	0.02
		N. weight:	0.52
		Sc. PD:	-0.032
		Sc. rank:	-5905.8

,

HA			
Pos.	404	obs:	exp:
tct	S	0	19.53
tcc	S	0	18.08
tca	S	121	32.52
tcg	S	0	4.76
act	T	0	10.56
acc	T	0	6.01
aca	T	34	14.71
acg	T	0	2.72
aat	N	2	1.12
aac	N	0	0.88
agt	S	0	21.15
agc	S	0	24.96
gtt	V	0	2.50
gtc	V	0	2.04
gta	V	10	2.17
gtg	V	0	3.29
gct	A	34	17879.00
gcc	A	3	16389.00
gca	A	65512	26535.00
gcg	A	50	4795.00
gaa	E	8	4.72
gag	E	0	3.28
---	--	------	------
mPD		0.0081	0.70
		nPD:	0.01
		N. weight:	0.99
		Sc. PD:	-0.068
		Sc. rank:	-25859.0

,

HA			
Pos.	405	obs:	exp:
tct	S	0	0.16
tcc	S	0	0.15
tca	S	1	0.27
tcg	S	0	0.04
act	T	0	0.93
acc	T	1	0.53
aca	T	2	1.30
acg	T	0	0.24
agt	S	0	0.17
agc	S	0	0.21
gtt	V	0	3.26
gtc	V	0	2.65
gta	V	10	2.82
gtg	V	3	4.28
gct	A	77	17922.00
gcc	A	72	16429.00
gca	A	65349	26599.00
gcg	A	258	4807.00
gaa	E	1	0.59
gag	E	0	0.41
---	--	------	------
mPD		0.013	0.69
		nPD:	0.02
		N. weight:	0.96
		Sc. PD:	-0.059
		Sc. rank:	-9848.3

,

HA			
Pos.	406	obs:	exp:
ttt	F	0	0.97
ttc	F	2	1.03
tta	L	0	1.62
ttg	L	0	2.89
ctt	L	0	2.93
ctc	L	14	2.05
cta	L	0	2.15
ctg	L	0	2.35
att	I	1421	23042.00
atc	I	64140	17247.00
ata	I	78	25350.00
atg	M	3	3.00
act	T	0	7.14
acc	T	23	4.07
aca	T	0	9.95
acg	T	0	1.84
gtt	V	0	23.04
gtc	V	92	18.75
gta	V	0	19.94
gtg	V	0	30.27
gct	A	0	0.27
gcc	A	1	0.25
gca	A	0	0.40
gcg	A	0	0.07
---	--	------	------
mPD		0.049	0.66
		nPD:	0.07
		N. weight:	1.3
		Sc. PD:	-0.013
		Sc. rank:	30484.0

,

HA			
Pos.	407	obs:	exp:
tat	Y	1	0.53
tac	Y	0	0.47
act	T	0	0.31
acc	T	1	0.18
aca	T	0	0.43
acg	T	0	0.08
aat	N	341	491.20
aac	N	537	386.80
gtt	V	2	0.50
gtc	V	0	0.41
gta	V	0	0.43
gtg	V	0	0.66
gct	A	1	0.27
gcc	A	0	0.25
gca	A	0	0.40
gcg	A	0	0.07
gat	D	64066	36679.00
gac	D	823	28210.00
gaa	E	1	0.59
gag	E	0	0.41
ggt	G	1	0.13
ggc	G	0	0.14
gga	G	0	0.46
ggg	G	0	0.26
---	--	------	------
mPD		0.067	0.52
		nPD:	0.13
		N. weight:	0.55
		Sc. PD:	0.024
		Sc. rank:	18597.0

,

HA			
Pos.	408	obs:	exp:
cct	P	0	0.33
ccc	P	0	0.19
cca	P	1	0.34
ccg	P	0	0.14
cat	H	2	1.13
cac	H	0	0.87
caa	Q	65680	40200.00
cag	Q	84	25564.00
aaa	K	5	4.60
aag	K	2	2.40
---	--	------	------
mPD		0.0029	0.48
		nPD:	0.01
		N. weight:	0.53
		Sc. PD:	-0.039
		Sc. rank:	-20518.0

,

HA			
Pos.	409	obs:	exp:
tct	S	0	0.16
tcc	S	0	0.15
tca	S	0	0.27
tcg	S	0	0.04
att	I	262	23085.00
atc	I	65330	17279.00
ata	I	169	25397.00
agt	S	0	0.17
agc	S	1	0.21
gtt	V	0	3.01
gtc	V	12	2.45
gta	V	0	2.60
gtg	V	0	3.95
---	--	------	------
mPD		0.013	0.66
		nPD:	0.02
		N. weight:	1.4
		Sc. PD:	-0.085
		Sc. rank:	-10524.0

,

HA			
Pos.	410	obs:	exp:
tct	S	0	418.60
tcc	S	0	387.60
tca	S	0	697.20
tcg	S	0	102.10
tat	Y	1	0.53
tac	Y	0	0.47
att	I	2	0.70
atc	I	0	0.53
ata	I	0	0.77
act	T	4	45.65
acc	T	143	25.99
aca	T	0	63.62
acg	T	0	11.74
aat	N	62421	35262.00
aac	N	606	27765.00
agt	S	2594	453.30
agc	S	0	535.10
gat	D	2	1.13
gac	D	0	0.87
ggt	G	1	0.13
ggc	G	0	0.14
gga	G	0	0.46
ggg	G	0	0.26
---	--	------	------
mPD		0.10	0.64
		nPD:	0.16
		N. weight:	0.64
		Sc. PD:	0.048
		Sc. rank:	22982.0

,

HA			
Pos.	411	obs:	exp:
cgt	R	0	0.75
cgc	R	0	1.37
cga	R	0	3.47
cgg	R	0	3.26
aat	N	0	1.12
aac	N	2	0.88
aga	R	0	18.64
agg	R	38	10.51
gaa	E	0	17.10
gag	E	29	11.90
ggt	G	11	8846.00
ggc	G	1	9170.00
gga	G	715	30331.00
ggg	G	64978	17359.00
---	--	------	------
mPD		0.024	0.68
		nPD:	0.04
		N. weight:	1.4
		Sc. PD:	-0.064
		Sc. rank:	9817.3

,

HA			
Pos.	412	obs:	exp:
cgt	R	0	0.06
cgc	R	0	0.11
cga	R	0	0.27
cgg	R	0	0.26
aat	N	11	6.15
aac	N	0	4.85
aaa	K	914	43233.00
aag	K	64846	22527.00
aga	R	0	1.47
agg	R	3	0.83
---	--	------	------
mPD		0.028	0.45
		nPD:	0.06
		N. weight:	1.1
		Sc. PD:	-0.022
		Sc. rank:	22614.0

,

HA			
Pos.	413	obs:	exp:
tta	L	1	7629.00
ttg	L	277	13593.00
ctt	L	82	13754.00
ctc	L	1	9641.00
cta	L	289	10096.00
ctg	L	65097	11034.00
att	I	0	0.35
atc	I	0	0.26
ata	I	1	0.39
atg	M	23	23.00
gtt	V	1	0.75
gtc	V	0	0.61
gta	V	2	0.65
gtg	V	0	0.99
---	--	------	------
mPD		0.021	1.2
		nPD:	0.02
		N. weight:	1.9
		Sc. PD:	-0.12
		Sc. rank:	-24043.0

,

HA			
Pos.	414	obs:	exp:
tct	S	0	0.48
tcc	S	0	0.45
tca	S	0	0.81
tcg	S	0	0.12
att	I	1	0.35
atc	I	0	0.26
ata	I	0	0.39
aat	N	65601	36792.00
aac	N	162	28971.00
agt	S	3	0.52
agc	S	0	0.62
gat	D	7	3.96
gac	D	0	3.04
---	--	------	------
mPD		0.0052	0.49
		nPD:	0.01
		N. weight:	0.62
		Sc. PD:	-0.043
		Sc. rank:	-17492.0

,

HA

Pos.	415	obs:	exp:
tta	L	0	0.23
ttg	L	0	0.41
tct	S	2	0.32
tcc	S	0	0.30
tca	S	0	0.54
tcg	S	0	0.08
ctt	L	0	0.42
ctc	L	0	0.29
cta	L	2	0.31
ctg	L	0	0.34
caa	Q	57	39.12
cag	Q	7	24.88
cgt	R	6	1302.00
cgc	R	131	2364.00
cga	R	48758	5993.00
cgg	R	11561	5641.00
aaa	K	5	10.52
aag	K	11	5.48
agt	S	0	0.35
agc	S	0	0.41
aga	R	3981	32219.00
agg	R	1252	18170.00
ggt	G	0	0.13
ggc	G	0	0.14
gga	G	1	0.46
ggg	G	0	0.26
---	--	------	------
mPD		0.47	0.89
		nPD:	0.53
		N. weight:	1.7
		Sc. PD:	0.7
		Sc. rank:	81060.0

,

HA

Pos.	416	obs:	exp:
ttt	F	2	0.97
ttc	F	0	1.03
tta	L	1090	7605.00
ttg	L	64372	13549.00
tgg	W	1	1.00
ctt	L	0	13710.00
ctc	L	0	9610.00
cta	L	0	10064.00
ctg	L	75	10999.00
att	I	0	1.05
atc	I	0	0.79
ata	I	3	1.16
atg	M	1	1.00
gtt	V	2	57.61
gtc	V	0	46.86
gta	V	221	49.85
gtg	V	7	75.68
---	--	------	------
mPD		0.049	1.2
		nPD:	0.04
		N. weight:	1.6
		Sc. PD:	-0.065
		Sc. rank:	22966.0

,

HA

Pos.	417	obs:	exp:
ttt	F	0	0.49
ttc	F	1	0.51
att	I	318	23067.00
atc	I	65305	17266.00
ata	I	88	25377.00
atg	M	4	4.00
act	T	0	1.55
acc	T	5	0.88
aca	T	0	2.16
acg	T	0	0.40
gtt	V	0	13.28
gtc	V	53	10.80
gta	V	0	11.49
gtg	V	0	17.44
---	--	------	------
mPD		0.014	0.66
		nPD:	0.02
		N. weight:	1.4
		Sc. PD:	-0.084
		Sc. rank:	-7973.0

,

HA

Pos.	418	obs:	exp:
tgg	W	1	1.00
cgt	R	0	0.57
cgc	R	0	1.04
cga	R	0	2.65
cgg	R	0	2.49
act	T	0	0.31
acc	T	0	0.18
aca	T	0	0.43
acg	T	1	0.08
aaa	K	3	96.64
aag	K	144	50.36
aga	R	6	14.22
agg	R	23	8.02
gtt	V	0	0.50
gtc	V	0	0.41
gta	V	0	0.43
gtg	V	2	0.66
gat	D	2	1.13
gac	D	0	0.87
gaa	E	9	243.50
gag	E	404	169.50
ggt	G	10	8775.00
ggc	G	3	9097.00
gga	G	27701	30088.00
ggg	G	37465	17220.00
---	--	------	------
mPD		0.51	0.70
		nPD:	0.73
		N. weight:	0.46
		Sc. PD:	0.28
		Sc. rank:	23308.0

,

HA

Pos.	419	obs:	exp:
cgt	R	0	1.01
cgc	R	0	1.84
cga	R	0	4.65
cgg	R	0	4.38
aat	N	0	0.56
aac	N	1	0.44
aaa	K	65478	43207.00
aag	K	242	22513.00
aga	R	51	25.01
agg	R	0	14.11
gaa	E	2	1.18
gag	E	0	0.82
---	--	------	------
mPD		0.0090	0.45
		nPD:	0.02
		N. weight:	0.44
		Sc. PD:	-0.027
		Sc. rank:	-3749.0

,

HA

Pos.	420	obs:	exp:
tct	S	0	0.48
tcc	S	1	0.45
tca	S	0	0.81
tcg	S	0	0.12
cct	P	0	1.00
ccc	P	3	0.56
cca	P	0	1.01
ccg	P	0	0.42
atg	M	2	2.00
act	T	103	20421.00
acc	T	64862	11626.00
aca	T	272	28458.00
acg	T	520	5252.00
agt	S	0	0.52
agc	S	2	0.62
gct	A	0	2.45
gcc	A	9	2.25
gca	A	0	3.64
gcg	A	0	0.66
---	--	------	------
mPD		0.028	0.68
		nPD:	0.04
		N. weight:	1.8
		Sc. PD:	-0.074
		Sc. rank:	21496.0

,

HA

Pos.	421	obs:	exp:
tct	S	0	0.81
tcc	S	0	0.75
tca	S	0	1.34
tcg	S	0	0.20
tat	Y	0	0.53
tac	Y	1	0.47
att	I	1	7.37
atc	I	20	5.52
ata	I	0	8.11
act	T	0	0.31
acc	T	1	0.18
aca	T	0	0.43
acg	T	0	0.08
aat	N	483	36782.00
aac	N	65262	28963.00
agt	S	0	0.87
agc	S	5	1.03
gat	D	0	0.57
gac	D	1	0.43
---	--	------	------
mPD		0.015	0.49
		nPD:	0.03
		N. weight:	0.86
		Sc. PD:	-0.042
		Sc. rank:	3576.9

,

HA

Pos.	422	obs:	exp:
caa	Q	0	0.61
cag	Q	1	0.39
act	T	0	0.62
acc	T	0	0.35
aca	T	2	0.87
acg	T	0	0.16
aaa	K	0	15.12
aag	K	23	7.88
gtt	V	0	10.52
gtc	V	0	8.56
gta	V	0	9.10
gtg	V	42	13.82
gct	A	0	0.27
gcc	A	0	0.25
gca	A	0	0.40
gcg	A	1	0.07
gat	D	7	3.96
gac	D	0	3.04
gaa	E	324	38697.00
gag	E	65313	26940.00
ggt	G	0	8.21
ggc	G	0	8.51
gga	G	0	28.16
ggg	G	61	16.12
---	--	------	------
mPD		0.014	0.49
		nPD:	0.03
		N. weight:	0.95
		Sc. PD:	-0.049
		Sc. rank:	2814.9

,

HA

Pos.	423	obs:	exp:
caa	Q	0	1.22
cag	Q	2	0.78
cgt	R	0	0.10
cgc	R	0	0.18
cga	R	0	0.46
cgg	R	0	0.43
aat	N	2	1.12
aac	N	0	0.88
aaa	K	65614	43232.00
aag	K	145	22527.00
aga	R	5	2.45
agg	R	0	1.38
gaa	E	6	3.54
gag	E	0	2.46
---	--	------	------
mPD		0.0049	0.45
		nPD:	0.01
		N. weight:	0.45
		Sc. PD:	-0.031
		Sc. rank:	-12367.0

,

HA

Pos.	424	obs:	exp:
ttt	F	142	31985.00
ttc	F	65613	33770.00
tta	L	0	0.46
ttg	L	0	0.83
tct	S	0	0.48
tcc	S	3	0.45
tca	S	0	0.81
tcg	S	0	0.12
tat	Y	0	6.33
tac	Y	12	5.67
ctt	L	0	0.84
ctc	L	4	0.59
cta	L	0	0.61
ctg	L	0	0.67
agt	S	0	0.52
agc	S	0	0.62
---	--	------	------
mPD		0.0049	0.50
		nPD:	0.01
		N. weight:	0.72
		Sc. PD:	-0.05
		Sc. rank:	-21753.0

,

HA

Pos.	425	obs:	exp:
tta	L	0	0.12
ttg	L	0	0.21
tat	Y	1	0.53
tac	Y	0	0.47
ctt	L	1	0.21
ctc	L	0	0.15
cta	L	0	0.15
ctg	L	0	0.17
cat	H	65140	37096.00
cac	H	628	28672.00
caa	Q	1	0.61
cag	Q	0	0.39
cgt	R	1	0.02
cgc	R	0	0.04
cga	R	0	0.09
cgg	R	0	0.09
act	T	0	0.62
acc	T	0	0.35
aca	T	2	0.87
acg	T	0	0.16
aga	R	0	0.49
agg	R	0	0.28
---	--	------	------
mPD		0.019	0.49
		nPD:	0.04
		N. weight:	0.58
		Sc. PD:	-0.024
		Sc. rank:	6246.8

,

HA

Pos.	426	obs:	exp:
tta	L	0	0.23
ttg	L	0	0.41
ctt	L	0	0.42
ctc	L	0	0.29
cta	L	1	0.31
ctg	L	1	0.34
cat	H	3	1.69
cac	H	0	1.31
caa	Q	925	40200.00
cag	Q	64839	25564.00
cgt	R	0	0.02
cgc	R	0	0.04
cga	R	0	0.09
cgg	R	1	0.09
aaa	K	0	1.31
aag	K	2	0.69
aga	R	0	0.49
agg	R	0	0.28
gct	A	0	0.55
gcc	A	0	0.50
gca	A	2	0.81
gcg	A	0	0.15
---	--	------	------
mPD		0.028	0.48
		nPD:	0.06
		N. weight:	0.96
		Sc. PD:	-0.022
		Sc. rank:	19288.0

,

HA			
Pos.	427	obs:	exp:
tct	S	0	0.16
tcc	S	0	0.15
tca	S	0	0.27
tcg	S	0	0.04
att	I	65188	23085.00
atc	I	532	17279.00
ata	I	41	25397.00
act	T	7	2.17
acc	T	0	1.24
aca	T	0	3.03
acg	T	0	0.56
agt	S	1	0.17
agc	S	0	0.21
gtt	V	3	1.25
gtc	V	0	1.02
gta	V	1	1.08
gtg	V	1	1.65
---	--	------	------
mPD		0.018	0.66
		nPD:	0.03
		N. weight:	1.1
		Sc. PD:	-0.059
		Sc. rank:	218.1

,

HA			
Pos.	428	obs:	exp:
gtt	V	0	0.25
gtc	V	0	0.20
gta	V	1	0.22
gtg	V	0	0.33
gaa	E	65361	38776.00
gag	E	410	26995.00
ggt	G	2	0.27
ggc	G	0	0.28
gga	G	0	0.92
ggg	G	0	0.53
---	--	------	------
mPD		0.013	0.48
		nPD:	0.03
		N. weight:	0.54
		Sc. PD:	-0.03
		Sc. rank:	-607.5

,

HA			
Pos.	429	obs:	exp:
cgt	R	0	0.06
cgc	R	0	0.11
cga	R	0	0.27
cgg	R	0	0.26
aat	N	7	5.04
aac	N	2	3.96
aaa	K	65237	43234.00
aag	K	524	22527.00
aga	R	3	1.47
agg	R	0	0.83
gaa	E	1	0.59
gag	E	0	0.41
---	--	------	------
mPD		0.016	0.45
		nPD:	0.04
		N. weight:	0.42
		Sc. PD:	-0.019
		Sc. rank:	3266.8

,

HA			
Pos.	430	obs:	exp:
aaa	K	1	0.66
aag	K	0	0.34
gat	D	0	1.13
gac	D	2	0.87
gaa	E	60118	38776.00
gag	E	5653	26995.00
---	--	------	------
mPD		0.16	0.48
		nPD:	0.32
		N. weight:	0.29
		Sc. PD:	0.068
		Sc. rank:	12743.0

,

HA			
Pos.	431	obs:	exp:
ttt	F	631	31993.00
ttc	F	65140	33778.00
tta	L	0	0.12
ttg	L	0	0.21
tct	S	0	0.32
tcc	S	2	0.30
tca	S	0	0.54
tcg	S	0	0.08
ctt	L	0	0.21
ctc	L	1	0.15
cta	L	0	0.15
ctg	L	0	0.17
agt	S	0	0.35
agc	S	0	0.41
---	--	------	------
mPD		0.019	0.50
		nPD:	0.04
		N. weight:	0.68
		Sc. PD:	-0.029
		Sc. rank:	7011.3

,

HA			
Pos.	432	obs:	exp:
tta	L	33	3.95
ttg	L	0	7.03
tct	S	61	10606.00
tcc	S	26	9822.00
tca	S	65537	17665.00
tcg	S	100	2587.00
ctt	L	0	7.11
ctc	L	0	4.99
cta	L	1	5.22
ctg	L	0	5.71
cct	P	0	0.33
ccc	P	0	0.19
cca	P	1	0.34
ccg	P	0	0.14
act	T	0	4.04
acc	T	0	2.30
aca	T	13	5.63
acg	T	0	1.04
aat	N	0	1.12
aac	N	2	0.88
agt	S	0	11485.00
agc	S	0	13559.00
---	--	------	------
mPD		0.0073	1.6
		nPD:	0.
		N. weight:	1.4
		Sc. PD:	-0.11
		Sc. rank:	-61992.0

,

HA			
Pos.	433	obs:	exp:
cat	H	0	1.13
cac	H	2	0.87
aaa	K	5	3.29
aag	K	0	1.71
gct	A	0	0.55
gcc	A	0	0.50
gca	A	2	0.81
gcg	A	0	0.15
gat	D	1	27.70
gac	D	48	21.30
gaa	E	65025	38741.00
gag	E	686	26970.00
ggt	G	0	0.67
ggc	G	0	0.70
gga	G	5	2.31
ggg	G	0	1.32
---	--	------	------
mPD		0.023	0.49
		nPD:	0.05
		N. weight:	0.52
		Sc. PD:	-0.018
		Sc. rank:	8009.2

,

HA			
Pos.	434	obs:	exp:
tta	L	4	0.81
ttg	L	0	1.45
ctt	L	0	1.46
ctc	L	0	1.03
cta	L	1	1.07
ctg	L	2	1.17
att	I	1	39.32
atc	I	5	29.43
ata	I	106	43.25
gtt	V	22	16440.00
gtc	V	5008	13374.00
gta	V	60486	14226.00
gtg	V	120	21596.00
gct	A	0	4.63
gcc	A	0	4.25
gca	A	17	6.88
gcg	A	0	1.24
ggt	G	0	0.27
ggc	G	0	0.28
gga	G	2	0.92
ggg	G	0	0.53
---	--	------	------
mPD		0.15	0.74
		nPD:	0.2
		N. weight:	1.4
		Sc. PD:	0.15
		Sc. rank:	53427.0

,

HA			
Pos.	435	obs:	exp:
caa	Q	1	0.61
cag	Q	0	0.39
gaa	E	65559	38776.00
gag	E	211	26994.00
ggt	G	0	0.40
ggc	G	0	0.42
gga	G	3	1.38
ggg	G	0	0.79
---	--	------	------
mPD		0.0065	0.48
		nPD:	0.01
		N. weight:	0.56
		Sc. PD:	-0.037
		Sc. rank:	-11641.0

,

HA			
Pos.	436	obs:	exp:
tgg	W	1	1.00
cgt	R	0	0.18
cgc	R	0	0.32
cga	R	0	0.82
cgg	R	0	0.77
aaa	K	2	1.31
aag	K	0	0.69
aga	R	6	4.41
agg	R	3	2.49
gaa	E	6	4.72
gag	E	2	3.28
ggt	G	5	8852.00
ggc	G	4	9177.00
gga	G	46811	30353.00
ggg	G	18934	17372.00
---	--	------	------
mPD		0.41	0.68
		nPD:	0.6
		N. weight:	0.37
		Sc. PD:	0.18
		Sc. rank:	18436.0

,

HA			
Pos.	437	obs:	exp:
tct	S	0	0.16
tcc	S	0	0.15
tca	S	0	0.27
tcg	S	0	0.04
cgt	R	0	1304.00
cgc	R	0	2366.00
cga	R	106	5999.00
cgg	R	0	5646.00
att	I	0	2.11
atc	I	0	1.58
ata	I	6	2.32
aaa	K	6	4.60
aag	K	1	2.40
agt	S	1	0.17
agc	S	0	0.21
aga	R	65181	32251.00
agg	R	466	18187.00
ggt	G	0	0.94
ggc	G	0	0.98
gga	G	7	3.23
ggg	G	0	1.85
---	--	------	------
mPD		0.018	0.89
		nPD:	0.02
		N. weight:	0.73
		Sc. PD:	-0.044
		Sc. rank:	-5961.9

,

HA			
Pos.	438	obs:	exp:
ttt	F	30	14.59
ttc	F	0	15.41
tta	L	0	0.93
ttg	L	0	1.65
ctt	L	8	1.67
ctc	L	0	1.17
cta	L	0	1.23
ctg	L	0	1.34
att	I	35879	12660.00
atc	I	172	9476.00
ata	I	14	13928.00
atg	M	3	3.00
act	T	15	4.66
acc	T	0	2.65
aca	T	0	6.49
acg	T	0	1.20
aat	N	1	0.56
aac	N	0	0.44
gtt	V	29393	7427.00
gtc	V	231	6041.00
gta	V	26	6426.00
gtg	V	0	9756.00
gct	A	2	0.55
gcc	A	0	0.50
gca	A	0	0.81
gcg	A	0	0.15
---	--	------	------
mPD		0.51	1.2
		nPD:	0.41
		N. weight:	1.3
		Sc. PD:	0.4
		Sc. rank:	59835.0

,

HA			
Pos.	439	obs:	exp:
tta	L	0	0.70
ttg	L	0	1.24
ctt	L	0	1.26
ctc	L	0	0.88
cta	L	0	0.92
ctg	L	6	1.01
cct	P	0	0.33
ccc	P	0	0.19
cca	P	1	0.34
ccg	P	0	0.14
cat	H	3	3.38
cac	H	3	2.62
caa	Q	30038	40195.00
cag	Q	35717	25560.00
cgt	R	0	0.06
cgc	R	0	0.11
cga	R	3	0.27
cgg	R	0	0.26
aaa	K	0	0.66
aag	K	1	0.34
aga	R	0	1.47
agg	R	0	0.83
gaa	E	0	1.18
gag	E	2	0.82
---	--	------	------
mPD		0.50	0.48
		nPD:	1.04
		N. weight:	0.054
		Sc. PD:	0.05
		Sc. rank:	2790.6

,

HA			
Pos.	440	obs:	exp:
tat	Y	0	1.58
tac	Y	3	1.42
aat	N	3	36.37
aac	N	62	28.63
gtt	V	0	1.00
gtc	V	4	0.82
gta	V	0	0.87
gtg	V	0	1.32
gct	A	0	0.82
gcc	A	3	0.75
gca	A	0	1.21
gcg	A	0	0.22
gat	D	449	37120.00
gac	D	65219	28548.00
gaa	E	6	4.72
gag	E	2	3.28
ggt	G	0	3.10
ggc	G	23	3.21
gga	G	0	10.62
ggg	G	0	6.08
---	--	------	------
mPD		0.017	0.49
		nPD:	0.03
		N. weight:	0.87
		Sc. PD:	-0.041
		Sc. rank:	5092.8

,

HA			
Pos.	441	obs:	exp:
ttt	F	4	1.95
ttc	F	0	2.05
tta	L	2	7613.00
ttg	L	0	13565.00
ctt	L	64392	13725.00
ctc	L	985	9621.00
cta	L	224	10075.00
ctg	L	7	11011.00
cct	P	2	0.67
ccc	P	0	0.37
cca	P	0	0.68
ccg	P	0	0.28
cgt	R	2	0.04
cgc	R	0	0.07
cga	R	0	0.18
cgg	R	0	0.17
att	I	151	54.76
atc	I	5	40.99
ata	I	0	60.25
aga	R	0	0.98
agg	R	0	0.55
---	--	------	------
mPD		0.042	1.2
		nPD:	0.04
		N. weight:	1.6
		Sc. PD:	-0.073
		Sc. rank:	12560.0

,

HA			
Pos.	442	obs:	exp:
aat	N	2	1.12
aac	N	0	0.88
aaa	K	0	1.31
aag	K	2	0.69
gct	A	0	0.27
gcc	A	0	0.25
gca	A	0	0.40
gcg	A	1	0.07
gat	D	3	1.70
gac	D	0	1.30
gaa	E	280	38768.00
gag	E	65477	26989.00
ggt	G	0	1.21
ggc	G	0	1.26
gga	G	0	4.15
ggg	G	9	2.38
---	--	------	------
mPD		0.0091	0.48
		nPD:	0.02
		N. weight:	0.95
		Sc. PD:	-0.059
		Sc. rank:	-9915.8

,

HA			
Pos.	443	obs:	exp:
cgt	R	0	1.69
cgc	R	0	3.06
cga	R	0	7.76
cgg	R	0	7.30
act	T	0	0.93
acc	T	0	0.53
aca	T	3	1.30
acg	T	0	0.24
aat	N	1	0.56
aac	N	0	0.44
aaa	K	65585	43178.00
aag	K	92	22499.00
aga	R	84	41.69
agg	R	1	23.51
gaa	E	8	4.72
gag	E	0	3.28
---	--	------	------
mPD		0.0058	0.45
		nPD:	0.01
		N. weight:	0.45
		Sc. PD:	-0.03
		Sc. rank:	-10315.0

,

HA			
Pos.	444	obs:	exp:
ttt	F	2	0.97
ttc	F	0	1.03
tat	Y	65494	34668.00
tac	Y	275	31101.00
aat	N	1	0.56
aac	N	0	0.44
aaa	K	2	1.31
aag	K	0	0.69
---	--	------	------
mPD		0.0085	0.50
		nPD:	0.02
		N. weight:	0.68
		Sc. PD:	-0.043
		Sc. rank:	-9158.9

,

HA			
Pos.	445	obs:	exp:
tta	L	0	0.23
ttg	L	0	0.41
ctt	L	2	0.42
ctc	L	0	0.29
cta	L	0	0.31
ctg	L	0	0.34
att	I	73	25.63
atc	I	0	19.18
ata	I	0	28.19
gtt	V	65330	16455.00
gtc	V	313	13386.00
gta	V	24	14239.00
gtg	V	29	21615.00
gct	A	3	0.82
gcc	A	0	0.75
gca	A	0	1.21
gcg	A	0	0.22
---	--	------	------
mPD		0.013	0.74
		nPD:	0.02
		N. weight:	1.5
		Sc. PD:	-0.092
		Sc. rank:	-17695.0

,

HA			
Pos.	446	obs:	exp:
gtt	V	0	0.50
gtc	V	0	0.41
gta	V	0	0.43
gtg	V	2	0.66
gat	D	3	1.70
gac	D	0	1.30
gaa	E	627	38774.00
gag	E	65140	26993.00
ggt	G	0	0.27
ggc	G	0	0.28
gga	G	0	0.92
ggg	G	2	0.53
---	--	------	------
mPD		0.019	0.48
		nPD:	0.04
		N. weight:	0.92
		Sc. PD:	-0.038
		Sc. rank:	10268.0

,

HA			
Pos.	447	obs:	exp:
aat	N	0	2.24
aac	N	4	1.76
gct	A	0	0.55
gcc	A	2	0.50
gca	A	0	0.81
gcg	A	0	0.15
gat	D	80	37176.00
gac	D	65688	28592.00
---	--	------	------
mPD		0.0026	0.49
		nPD:	0.01
		N. weight:	0.91
		Sc. PD:	-0.068
		Sc. rank:	-37559.0

,

HA			
Pos.	448	obs:	exp:
tct	S	21	3.71
tcc	S	0	3.44
tca	S	1	6.18
tcg	S	0	0.91
att	I	18	6.32
atc	I	0	4.73
ata	I	0	6.95
act	T	53777	20405.00
acc	T	846	11617.00
aca	T	11033	28436.00
acg	T	49	5248.00
aat	N	18	10.07
aac	N	0	7.93
agt	S	1	4.02
agc	S	0	4.74
gct	A	5	2.18
gcc	A	0	2.00
gca	A	3	3.24
gcg	A	0	0.58
ggt	G	1	0.27
ggc	G	0	0.28
gga	G	1	0.92
ggg	G	0	0.53
---	--	------	------
mPD		0.30	0.68
		nPD:	0.45
		N. weight:	0.66
		Sc. PD:	0.23
		Sc. rank:	31551.0

,

HA			
Pos.	449	obs:	exp:
ttt	F	0	0.97
ttc	F	2	1.03
tta	L	1	0.12
ttg	L	0	0.21
ctt	L	0	0.21
ctc	L	0	0.15
cta	L	0	0.15
ctg	L	0	0.17
act	T	0	0.62
acc	T	0	0.35
aca	T	2	0.87
acg	T	0	0.16
aat	N	1	2.24
aac	N	3	1.76
aaa	K	65691	43236.00
aag	K	73	22528.00
gaa	E	1	0.59
gag	E	0	0.41
---	--	------	------
mPD		0.0027	0.45
		nPD:	0.01
		N. weight:	0.45
		Sc. PD:	-0.033
		Sc. rank:	-17878.0

,

HA			
Pos.	450	obs:	exp:
ttt	F	0	0.49
ttc	F	1	0.51
tta	L	4	0.70
ttg	L	0	1.24
ctt	L	0	1.26
ctc	L	0	0.88
cta	L	0	0.92
ctg	L	2	1.01
att	I	137	23011.00
atc	I	94	17224.00
ata	I	65320	25316.00
atg	M	6	6.00
act	T	2	21.12
acc	T	0	12.02
aca	T	66	29.43
acg	T	0	5.43
gtt	V	1	35.57
gtc	V	0	28.93
gta	V	138	30.78
gtg	V	3	46.72
---	--	------	------
mPD		0.014	0.67
		nPD:	0.02
		N. weight:	1.
		Sc. PD:	-0.061
		Sc. rank:	-6124.1

,

HA			
Pos.	451	obs:	exp:
aat	N	2	1.12
aac	N	0	0.88
gat	D	65626	37176.00
gac	D	142	28592.00
gaa	E	3	1.77
gag	E	0	1.23
ggt	G	1	0.13
ggc	G	0	0.14
gga	G	0	0.46
ggg	G	0	0.26
---	--	------	------
mPD		0.0045	0.49
		nPD:	0.01
		N. weight:	0.61
		Sc. PD:	-0.043
		Sc. rank:	-19369.0

,

HA			
Pos.	452	obs:	exp:
ttt	F	0	4.86
ttc	F	10	5.14
tta	L	0	7620.00
ttg	L	0	13576.00
ctt	L	812	13736.00
ctc	L	64773	9629.00
cta	L	75	10083.00
ctg	L	4	11020.00
att	I	2	35.10
atc	I	98	26.28
ata	I	0	38.62
---	--	------	------
mPD		0.030	1.2
		nPD:	0.03
		N. weight:	2.
		Sc. PD:	-0.11
		Sc. rank:	-1550.0

,

HA			
Pos.	453	obs:	exp:
tgg	W	65770	65770.00
cgt	R	0	0.06
cgc	R	0	0.11
cga	R	0	0.27
cgg	R	1	0.26
aga	R	0	1.47
agg	R	2	0.83
ggt	G	0	0.13
ggc	G	0	0.14
gga	G	0	0.46
ggg	G	1	0.26
---	--	------	------
mPD		0.00012	0.00020
		nPD:	0.6
		N. weight:	0.000088
		Sc. PD:	0.000044
		Sc. rank:	4.4

,

HA			
Pos.	454	obs:	exp:
tct	S	477	10612.00
tcc	S	4	9828.00
tca	S	65033	17676.00
tcg	S	250	2589.00
tgt	C	0	0.39
tgc	C	1	0.61
act	T	2	2.17
acc	T	0	1.24
aca	T	5	3.03
acg	T	0	0.56
agt	S	0	11492.00
agc	S	0	13567.00
gtt	V	0	0.25
gtc	V	0	0.20
gta	V	1	0.22
gtg	V	0	0.33
ggt	G	1	0.13
ggc	G	0	0.14
gga	G	0	0.46
ggg	G	0	0.26
---	--	------	------
mPD		0.023	1.6
		nPD:	0.01
		N. weight:	1.4
		Sc. PD:	-0.092
		Sc. rank:	-27665.0

,

HA			
Pos.	455	obs:	exp:
ttt	F	0	0.49
ttc	F	1	0.51
tct	S	0	0.16
tcc	S	1	0.15
tca	S	0	0.27
tcg	S	0	0.04
tat	Y	1184	34668.00
tac	Y	64586	31102.00
cat	H	1	0.56
cac	H	0	0.44
aat	N	0	0.56
aac	N	1	0.44
agt	S	0	0.17
agc	S	0	0.21
---	--	------	------
mPD		0.036	0.50
		nPD:	0.07
		N. weight:	0.73
		Sc. PD:	-0.0084
		Sc. rank:	16282.0

,

HA			
Pos.	456	obs:	exp:
tct	S	0	2.10
tcc	S	0	1.94
tca	S	0	3.49
tcg	S	0	0.51
tgt	C	1	0.39
tgc	C	0	0.61
tgg	W	1	1.00
cgt	R	0	0.04
cgc	R	0	0.07
cga	R	0	0.18
cgg	R	0	0.17
att	I	0	1.76
atc	I	5	1.31
ata	I	0	1.93
aat	N	5090	36771.00
aac	N	60634	28953.00
aaa	K	0	18.41
aag	K	28	9.59
agt	S	0	2.27
agc	S	13	2.68
aga	R	0	0.98
agg	R	2	0.55
---	--	------	------
mPD		0.14	0.49
		nPD:	0.29
		N. weight:	0.58
		Sc. PD:	0.12
		Sc. rank:	24773.0

,

HA			
Pos.	457	obs:	exp:
ttt	F	1	0.49
ttc	F	0	0.51
tct	S	1	6.45
tcc	S	0	5.98
tca	S	1	10.75
tcg	S	38	1.57
tgt	C	1	0.39
tgc	C	0	0.61
act	T	0	0.62
acc	T	0	0.35
aca	T	0	0.87
acg	T	2	0.16
agt	S	0	6.99
agc	S	0	8.25
gtt	V	0	0.25
gtc	V	0	0.20
gta	V	0	0.22
gtg	V	1	0.33
gct	A	6039	17912.00
gcc	A	6	16420.00
gca	A	2258	26584.00
gcg	A	57417	4804.00
ggt	G	0	1.21
ggc	G	0	1.26
gga	G	0	4.15
ggg	G	9	2.38
---	--	------	------
mPD		0.23	0.70
		nPD:	0.33
		N. weight:	2.2
		Sc. PD:	0.51
		Sc. rank:	95838.0

,

HA			
Pos.	458	obs:	exp:
caa	Q	0	2.45
cag	Q	4	1.55
aaa	K	0	0.66
aag	K	1	0.34
gat	D	20	11.31
gac	D	0	8.69
gaa	E	161	38751.00
gag	E	65568	26978.00
ggt	G	0	2.69
ggc	G	0	2.79
gga	G	0	9.23
ggg	G	20	5.28
---	--	------	------
mPD		0.0063	0.49
		nPD:	0.01
		N. weight:	0.96
		Sc. PD:	-0.065
		Sc. rank:	-21664.0

,

HA

Pos.	459	obs:	exp:
ttt	F	52	25.29
ttc	F	0	26.71
tta	L	0	7619.00
ttg	L	0	13574.00
tct	S	1	0.16
tcc	S	0	0.15
tca	S	0	0.27
tcg	S	0	0.04
ctt	L	65517	13735.00
ctc	L	92	9628.00
cta	L	41	10082.00
ctg	L	6	11019.00
cct	P	2	0.67
ccc	P	0	0.37
cca	P	0	0.68
ccg	P	0	0.28
cat	H	2	1.13
cac	H	0	0.87
att	I	61	21.41
atc	I	0	16.03
ata	I	0	23.56
agt	S	0	0.17
agc	S	0	0.21
---	--	------	------
mPD		0.0078	1.2
		nPD:	0.01
		N. weight:	1.7
		Sc. PD:	-0.12
		Sc. rank:	-61645.0

,

HA

Pos.	460	obs:	exp:
ttt	F	9	4.38
ttc	F	0	4.62
tta	L	0	7627.00
ttg	L	2	13589.00
ctt	L	65616	13750.00
ctc	L	76	9639.00
cta	L	5	10093.00
ctg	L	30	11031.00
cct	P	2	0.67
ccc	P	0	0.37
cca	P	0	0.68
ccg	P	0	0.28
att	I	29	10.18
atc	I	0	7.62
ata	I	0	11.20
gtt	V	5	1.25
gtc	V	0	1.02
gta	V	0	1.08
gtg	V	0	1.65
---	--	------	------
mPD		0.0049	1.2
		nPD:	0.
		N. weight:	1.7
		Sc. PD:	-0.13
		Sc. rank:	-75831.0

,

HA

Pos.	461	obs:	exp:
att	I	38	13.34
atc	I	0	9.98
ata	I	0	14.68
act	T	1	0.31
acc	T	0	0.18
aca	T	0	0.43
acg	T	0	0.08
gtt	V	65503	16464.00
gtc	V	182	13393.00
gta	V	35	14247.00
gtg	V	12	21627.00
gat	D	2	1.13
gac	D	0	0.87
ggt	G	1	0.13
ggc	G	0	0.14
gga	G	0	0.46
ggg	G	0	0.26
---	--	------	------
mPD		0.0083	0.74
		nPD:	0.01
		N. weight:	1.5
		Sc. PD:	-0.1
		Sc. rank:	-40956.0

,

HA

Pos.	462	obs:	exp:
tta	L	0	0.23
ttg	L	0	0.41
tct	S	0	5.65
tcc	S	35	5.23
tca	S	0	9.41
tcg	S	0	1.38
ctt	L	0	0.42
ctc	L	0	0.29
cta	L	2	0.31
ctg	L	0	0.34
act	T	0	9.94
acc	T	32	5.66
aca	T	0	13.85
acg	T	0	2.56
aat	N	0	0.56
aac	N	1	0.44
agt	S	0	6.12
agc	S	0	7.22
gtt	V	0	2.00
gtc	V	8	1.63
gta	V	0	1.73
gtg	V	0	2.63
gct	A	298	17905.00
gcc	A	65305	16413.00
gca	A	90	26573.00
gcg	A	0	4802.00
gat	D	0	0.57
gac	D	1	0.43
gaa	E	1	0.59
gag	E	0	0.41
ggt	G	0	0.13
ggc	G	1	0.14
gga	G	0	0.46
ggg	G	0	0.26
---	--	------	------
mPD		0.014	0.70
		nPD:	0.02
		N. weight:	1.5
		Sc. PD:	-0.089
		Sc. rank:	-9553.5

,

HA

Pos.	463	obs:	exp:
tta	L	2	7625.00
ttg	L	250	13586.00
ctt	L	50	13746.00
ctc	L	0	9636.00
cta	L	155	10091.00
ctg	L	65256	11028.00
att	I	0	0.35
atc	I	0	0.26
ata	I	1	0.39
atg	M	60	60.00
---	--	------	------
mPD		0.016	1.2
		nPD:	0.01
		N. weight:	1.9
		Sc. PD:	-0.13
		Sc. rank:	-38653.0

,

HA

Pos.	464	obs:	exp:
caa	Q	0	0.61
cag	Q	1	0.39
aat	N	0	0.56
aac	N	1	0.44
aaa	K	0	0.66
aag	K	1	0.34
gtt	V	0	0.25
gtc	V	0	0.20
gta	V	0	0.22
gtg	V	1	0.33
gat	D	0	0.57
gac	D	1	0.43
gaa	E	546	38775.00
gag	E	65223	26994.00
---	--	------	------
mPD		0.017	0.48
		nPD:	0.03
		N. weight:	0.93
		Sc. PD:	-0.043
		Sc. rank:	5708.8

,

HA

Pos.	465	obs:	exp:
tct	S	0	0.32
tcc	S	0	0.30
tca	S	0	0.54
tcg	S	0	0.08
tat	Y	0	0.53
tac	Y	1	0.47
aat	N	1169	36796.00
aac	N	64601	28974.00
agt	S	0	0.35
agc	S	2	0.41
gat	D	0	0.57
gac	D	1	0.43
---	--	------	------
mPD		0.035	0.49
		nPD:	0.07
		N. weight:	0.8
		Sc. PD:	-0.0094
		Sc. rank:	17904.0

,

HA

Pos.	466	obs:	exp:
tat	Y	0	1.05
tac	Y	2	0.95
tgt	C	1	0.39
tgc	C	0	0.61
cat	H	0	3.38
cac	H	6	2.62
caa	Q	65583	40199.00
cag	Q	180	25564.00
gaa	E	2	1.18
gag	E	0	0.82
---	--	------	------
mPD		0.0059	0.48
		nPD:	0.01
		N. weight:	0.52
		Sc. PD:	-0.035
		Sc. rank:	-12438.0

,

HA

Pos.	467	obs:	exp:
tta	L	0	0.23
ttg	L	0	0.41
tat	Y	6	3.16
tac	Y	0	2.84
tgt	C	1	0.39
tgc	C	0	0.61
ctt	L	2	0.42
ctc	L	0	0.29
cta	L	0	0.31
ctg	L	0	0.34
cat	H	63932	37077.00
cac	H	1802	28657.00
caa	Q	10	15.28
cag	Q	15	9.72
cgt	R	0	0.04
cgc	R	0	0.07
cga	R	0	0.18
cgg	R	0	0.17
aat	N	4	2.24
aac	N	0	1.76
aga	R	2	0.98
agg	R	0	0.55
---	--	------	------
mPD		0.055	0.49
		nPD:	0.11
		N. weight:	0.5
		Sc. PD:	0.013
		Sc. rank:	16021.0

,

HA

Pos.	468	obs:	exp:
tct	S	0	0.32
tcc	S	0	0.30
tca	S	2	0.54
tcg	S	0	0.08
cct	P	0	0.33
ccc	P	0	0.19
cca	P	1	0.34
ccg	P	0	0.14
att	I	0	1.05
atc	I	0	0.79
ata	I	3	1.16
act	T	10	20423.00
acc	T	3	11627.00
aca	T	55232	28461.00
acg	T	10519	5253.00
agt	S	0	0.35
agc	S	0	0.41
gct	A	0	1.09
gcc	A	0	1.00
gca	A	4	1.62
gcg	A	0	0.29
---	--	------	------
mPD		0.27	0.68
		nPD:	0.4
		N. weight:	0.74
		Sc. PD:	0.22
		Sc. rank:	33414.0

,

HA

Pos.	469	obs:	exp:
tta	L	0	2.44
ttg	L	2	4.34
tgg	W	2	2.00
ctt	L	19	4.39
ctc	L	0	3.08
cta	L	0	3.22
ctg	L	0	3.52
att	I	65561	23038.00
atc	I	61	17245.00
ata	I	7	25346.00
atg	M	1	1.00
aat	N	1	0.56
aac	N	0	0.44
gtt	V	119	29.81
gtc	V	0	24.25
gta	V	0	25.79
gtg	V	0	39.15
gct	A	1	0.27
gcc	A	0	0.25
gca	A	0	0.40
gcg	A	0	0.07
---	--	------	------
mPD		0.0067	0.66
		nPD:	0.01
		N. weight:	1.1
		Sc. PD:	-0.08
		Sc. rank:	-33850.0

,

HA

Pos.	470	obs:	exp:
tct	S	0	0.16
tcc	S	0	0.15
tca	S	0	0.27
tcg	S	0	0.04
tat	Y	1	0.53
tac	Y	0	0.47
cat	H	17	9.59
cac	H	0	7.41
agt	S	1	0.17
agc	S	0	0.21
gtt	V	1	0.25
gtc	V	0	0.20
gta	V	0	0.22
gtg	V	0	0.33
gat	D	50096	37163.00
gac	D	15649	28582.00
gaa	E	1	2.95
gag	E	4	2.05
ggt	G	4	0.54
ggc	G	0	0.56
gga	G	0	1.85
ggg	G	0	1.06
---	--	------	------
mPD		0.36	0.49
		nPD:	0.74
		N. weight:	0.093
		Sc. PD:	0.058
		Sc. rank:	4767.1

,

HA

Pos.	471	obs:	exp:
ttt	F	1	0.49
ttc	F	0	0.51
tta	L	214	7630.00
ttg	L	1	13593.00
tat	Y	1	1.05
tac	Y	1	0.95
ctt	L	7	13754.00
ctc	L	6	9642.00
cta	L	64889	10096.00
ctg	L	633	11035.00
caa	Q	2	1.22
cag	Q	0	0.78
att	I	0	5.27
atc	I	0	3.94
ata	I	15	5.79
atg	M	1	1.00
gtt	V	0	0.25
gtc	V	0	0.20
gta	V	1	0.22
gtg	V	0	0.33
gaa	E	2	1.18
gag	E	0	0.82
---	--	------	------
mPD		0.027	1.2
		nPD:	0.02
		N. weight:	2.
		Sc. PD:	-0.11
		Sc. rank:	-5868.5

,

HA

Pos.	472	obs:	exp:
cct	P	1	0.33
ccc	P	0	0.19
cca	P	0	0.34
ccg	P	0	0.14
cat	H	0	1.13
cac	H	2	0.87
att	I	0	1.05
atc	I	3	0.79
ata	I	0	1.16
act	T	65410	20422.00
acc	T	199	11626.00
aca	T	102	28459.00
acg	T	48	5252.00
gct	A	9	2.45
gcc	A	0	2.25
gca	A	0	3.64
gcg	A	0	0.66
---	--	------	------
mPD		0.011	0.68
		nPD:	0.02
		N. weight:	1.3
		Sc. PD:	-0.08
		Sc. rank:	-17965.0

,

HA

Pos.	473	obs:	exp:
aat	N	0	0.56
aac	N	1	0.44
gct	A	0	1.09
gcc	A	1	1.00
gca	A	3	1.62
gcg	A	0	0.29
gat	D	549	37170.00
gac	D	65208	28587.00
gaa	E	5	4.72
gag	E	3	3.28
ggt	G	1	0.54
ggc	G	3	0.56
gga	G	0	1.85
ggg	G	0	1.06
---	--	------	------
mPD		0.017	0.49
		nPD:	0.03
		N. weight:	0.86
		Sc. PD:	-0.04
		Sc. rank:	5688.3

,

HA

Pos.	474	obs:	exp:
tta	L	6	0.93
ttg	L	0	1.65
tct	S	32	10567.00
tcc	S	22	9786.00
tca	S	65246	17601.00
tcg	S	185	2578.00
ctt	L	0	1.67
ctc	L	0	1.17
cta	L	0	1.23
ctg	L	2	1.34
act	T	0	0.31
acc	T	0	0.18
aca	T	1	0.43
acg	T	0	0.08
agt	S	0	11444.00
agc	S	0	13509.00
gct	A	0	76.31
gcc	A	0	69.96
gca	A	279	113.30
gcg	A	1	20.47
---	--	------	------
mPD		0.016	1.6
		nPD:	0.01
		N. weight:	1.4
		Sc. PD:	-0.1
		Sc. rank:	-42785.0

,

HA

Pos.	475	obs:	exp:
caa	Q	1	0.61
cag	Q	0	0.39
aat	N	2	1.12
aac	N	0	0.88
aaa	K	8	5.26
aag	K	0	2.74
gtt	V	0	0.50
gtc	V	2	0.41
gta	V	0	0.43
gtg	V	0	0.66
gct	A	0	0.55
gcc	A	2	0.50
gca	A	0	0.81
gcg	A	0	0.15
gaa	E	65514	38768.00
gag	E	243	26989.00
ggt	G	0	0.27
ggc	G	0	0.28
gga	G	2	0.92
ggg	G	0	0.53
---	--	------	------
mPD		0.0081	0.48
		nPD:	0.02
		N. weight:	0.56
		Sc. PD:	-0.035
		Sc. rank:	-7956.0

,

HA

Pos.	476	obs:	exp:
tta	L	0	0.58
ttg	L	5	1.03
ctt	L	0	1.05
ctc	L	0	0.73
cta	L	0	0.77
ctg	L	0	0.84
att	I	0	2.11
atc	I	0	1.58
ata	I	6	2.32
atg	M	65757	65757.00
act	T	0	0.31
acc	T	0	0.18
aca	T	0	0.43
acg	T	1	0.08
gtt	V	0	1.25
gtc	V	3	1.02
gta	V	0	1.08
gtg	V	2	1.65
---	--	------	------
mPD		0.00061	0.00074
		nPD:	0.82
		N. weight:	0.00035
		Sc. PD:	0.00024
		Sc. rank:	17.9

,

HA

Pos.	477	obs:	exp:
ttt	F	1	0.97
ttc	F	1	1.03
tct	S	0	31.63
tcc	S	0	29.29
tca	S	0	52.68
tcg	S	0	7.72
tat	Y	0	2.11
tac	Y	4	1.89
cat	H	0	1.69
cac	H	3	1.31
att	I	0	0.35
atc	I	1	0.26
ata	I	0	0.39
act	T	0	3.73
acc	T	12	2.12
aca	T	0	5.19
acg	T	0	0.96
aat	N	36	36668.00
aac	N	65504	28872.00
aaa	K	5	4.60
aag	K	2	2.40
agt	S	0	34.25
agc	S	196	40.43
gtt	V	2	0.50
gtc	V	0	0.41
gta	V	0	0.43
gtg	V	0	0.66
gat	D	0	3.96
gac	D	7	3.04
---	--	------	------
mPD		0.0084	0.50
		nPD:	0.02
		N. weight:	0.9
		Sc. PD:	-0.057
		Sc. rank:	-12725.0

,

HA

Pos.	478	obs:	exp:
tta	L	2	0.23
ttg	L	0	0.41
tgt	C	2	0.78
tgc	C	0	1.22
ctt	L	0	0.42
ctc	L	0	0.29
cta	L	0	0.31
ctg	L	0	0.34
cgt	R	0	0.12
cgc	R	0	0.22
cga	R	0	0.55
cgg	R	0	0.52
att	I	0	0.35
atc	I	0	0.26
ata	I	1	0.39
act	T	0	0.31
acc	T	0	0.18
aca	T	1	0.43
acg	T	0	0.08
aat	N	0	9.51
aac	N	17	7.49
aaa	K	65607	43223.00
aag	K	138	22522.00
aga	R	6	2.94
agg	R	0	1.66
---	--	------	------
mPD		0.0053	0.45
		nPD:	0.01
		N. weight:	0.45
		Sc. PD:	-0.031
		Sc. rank:	-11678.0

,

HA

Pos.	479	obs:	exp:
ttt	F	0	0.97
ttc	F	2	1.03
tta	L	2	7631.00
ttg	L	428	13596.00
ctt	L	98	13757.00
ctc	L	4	9644.00
cta	L	233	10098.00
ctg	L	64999	11037.00
cat	H	0	0.56
cac	H	1	0.44
atg	M	5	5.00
gtt	V	0	0.50
gtc	V	0	0.41
gta	V	0	0.43
gtg	V	2	0.66
---	--	------	------
mPD		0.024	1.2
		nPD:	0.02
		N. weight:	1.9
		Sc. PD:	-0.11
		Sc. rank:	-14399.0

,

HA

Pos.	480	obs:	exp:
ttt	F	65561	31991.00
ttc	F	206	33776.00
tta	L	0	0.23
ttg	L	0	0.41
tct	S	1	0.16
tcc	S	0	0.15
tca	S	0	0.27
tcg	S	0	0.04
tat	Y	3	1.58
tac	Y	0	1.42
ctt	L	1	0.42
ctc	L	0	0.29
cta	L	0	0.31
ctg	L	1	0.34
agt	S	0	0.17
agc	S	0	0.21
gtt	V	1	0.25
gtc	V	0	0.20
gta	V	0	0.22
gtg	V	0	0.33
---	--	------	------
mPD		0.0065	0.50
		nPD:	0.01
		N. weight:	0.77
		Sc. PD:	-0.052
		Sc. rank:	-17040.0

,

HA

Pos.	481	obs:	exp:
tct	S	0	0.16
tcc	S	0	0.15
tca	S	0	0.27
tcg	S	0	0.04
aaa	K	2	1.31
aag	K	0	0.69
agt	S	1	0.17
agc	S	0	0.21
gtt	V	0	1.25
gtc	V	0	1.02
gta	V	5	1.08
gtg	V	0	1.65
gat	D	6	3.39
gac	D	0	2.61
gaa	E	65692	38767.00
gag	E	64	26989.00
ggt	G	1	0.54
ggc	G	0	0.56
gga	G	3	1.85
ggg	G	0	1.06
---	--	------	------
mPD		0.0026	0.48
		nPD:	0.01
		N. weight:	0.58
		Sc. PD:	-0.043
		Sc. rank:	-23716.0

,

HA

Pos.	482	obs:	exp:
cat	H	1	0.56
cac	H	0	0.44
caa	Q	2	1.22
cag	Q	0	0.78
cgt	R	0	17.07
cgc	R	0	30.98
cga	R	0	78.56
cgg	R	0	73.93
att	I	0	0.70
atc	I	0	0.53
ata	I	2	0.77
act	T	0	0.62
acc	T	0	0.35
aca	T	2	0.87
acg	T	0	0.16
aat	N	0	1.12
aac	N	2	0.88
aaa	K	64782	42664.00
aag	K	113	22231.00
aga	R	853	422.30
agg	R	8	238.20
gat	D	1	0.57
gac	D	0	0.43
gaa	E	8	4.72
gag	E	0	3.28
---	--	------	------
mPD		0.030	0.48
		nPD:	0.06
		N. weight:	0.45
		Sc. PD:	-0.0091
		Sc. rank:	9439.0

,

HA

Pos.	483	obs:	exp:
tta	L	0	0.12
ttg	L	0	0.21
tct	S	0	0.16
tcc	S	0	0.15
tca	S	1	0.27
tcg	S	0	0.04
ctt	L	0	0.21
ctc	L	0	0.15
cta	L	0	0.15
ctg	L	1	0.17
att	I	0	2.81
atc	I	0	2.10
ata	I	8	3.09
act	T	19	20423.00
acc	T	3	11627.00
aca	T	65719	28460.00
acg	T	21	5252.00
agt	S	0	0.17
agc	S	0	0.21
gtt	V	0	0.50
gtc	V	0	0.41
gta	V	2	0.43
gtg	V	0	0.66
---	--	------	------
mPD		0.0018	0.68
		nPD:	0.
		N. weight:	0.92
		Sc. PD:	-0.071
		Sc. rank:	-43561.0

,

HA

Pos.	484	obs:	exp:
caa	Q	0	1.22
cag	Q	2	0.78
cgt	R	0	11.42
cgc	R	0	20.73
cga	R	0	52.55
cgg	R	0	49.46
act	T	0	0.31
acc	T	0	0.18
aca	T	0	0.43
acg	T	1	0.08
aat	N	2	1.12
aac	N	0	0.88
aaa	K	1127	42855.00
aag	K	64058	22330.00
aga	R	159	282.50
agg	R	417	159.30
gaa	E	0	4.72
gag	E	8	3.28
---	--	------	------
mPD		0.056	0.47
		nPD:	0.12
		N. weight:	1.1
		Sc. PD:	0.036
		Sc. rank:	35385.0

,

HA

Pos.	485	obs:	exp:
tct	S	0	0.32
tcc	S	0	0.30
tca	S	2	0.54
tcg	S	0	0.08
tgt	C	1	0.39
tgc	C	0	0.61
caa	Q	0	0.61
cag	Q	1	0.39
cgt	R	0	4.90
cgc	R	0	8.89
cga	R	0	22.54
cgg	R	0	21.21
atg	M	1	1.00
act	T	0	0.93
acc	T	0	0.53
aca	T	0	1.30
acg	T	3	0.24
aat	N	5	4.48
aac	N	3	3.52
aaa	K	494	43067.00
aag	K	65014	22441.00
agt	S	0	0.35
agc	S	0	0.41
aga	R	3	121.10
agg	R	244	68.32
gaa	E	0	1.77
gag	E	3	1.23
---	--	------	------
mPD		0.023	0.46
		nPD:	0.05
		N. weight:	1.1
		Sc. PD:	-0.035
		Sc. rank:	18905.0

,

HA

Pos.	486	obs:	exp:
tta	L	0	0.81
ttg	L	0	1.45
ctt	L	0	1.46
ctc	L	0	1.03
cta	L	7	1.07
ctg	L	0	1.17
cat	H	1	1.69
cac	H	2	1.31
caa	Q	65374	40187.00
cag	Q	368	25555.00
cgt	R	0	0.28
cgc	R	0	0.50
cga	R	14	1.28
cgg	R	0	1.20
aaa	K	8	5.26
aag	K	0	2.74
aga	R	0	6.87
agg	R	0	3.87
---	--	------	------
mPD		0.012	0.48
		nPD:	0.03
		N. weight:	0.51
		Sc. PD:	-0.028
		Sc. rank:	-785.0

,

HA

Pos.	487	obs:	exp:
tta	L	6	7630.00
ttg	L	1843	13594.00
ctt	L	10	13754.00
ctc	L	0	9642.00
cta	L	279	10096.00
ctg	L	63613	11035.00
atg	M	22	22.00
act	T	1	0.31
acc	T	0	0.18
aca	T	0	0.43
acg	T	0	0.08
---	--	------	------
mPD		0.064	1.2
		nPD:	0.06
		N. weight:	1.8
		Sc. PD:	-0.047
		Sc. rank:	33500.0

,

HA

Pos.	488	obs:	exp:
cgt	R	0	1304.00
cgc	R	0	2366.00
cga	R	1	5999.00
cgg	R	148	5646.00
atg	M	3	3.00
aaa	K	2	8.55
aag	K	11	4.45
aga	R	973	32250.00
agg	R	64630	18187.00
gct	A	0	0.27
gcc	A	1	0.25
gca	A	0	0.40
gcg	A	0	0.07
gaa	E	0	0.59
gag	E	1	0.41
ggt	G	0	0.54
ggc	G	0	0.56
gga	G	0	1.85
ggg	G	4	1.06
---	--	------	------
mPD		0.035	0.89
		nPD:	0.04
		N. weight:	1.3
		Sc. PD:	-0.055
		Sc. rank:	14083.0

,

HA

Pos.	489	obs:	exp:
aat	N	0	1.12
aac	N	2	0.88
gtt	V	0	0.25
gtc	V	1	0.20
gta	V	0	0.22
gtg	V	0	0.33
gct	A	0	0.27
gcc	A	0	0.25
gca	A	1	0.40
gcg	A	0	0.07
gaa	E	65472	38774.00
gag	E	296	26994.00
ggt	G	0	0.27
ggc	G	0	0.28
gga	G	2	0.92
ggg	G	0	0.53
---	--	------	------
mPD		0.0092	0.48
		nPD:	0.02
		N. weight:	0.55
		Sc. PD:	-0.034
		Sc. rank:	-5182.7

,

HA

Pos.	490	obs:	exp:
aat	N	65631	36797.00
aac	N	141	28975.00
gat	D	1	0.57
gac	D	0	0.43
ggt	G	1	0.13
ggc	G	0	0.14
gga	G	0	0.46
ggg	G	0	0.26
---	--	------	------
mPD		0.0044	0.49
		nPD:	0.01
		N. weight:	0.63
		Sc. PD:	-0.044
		Sc. rank:	-19961.0

,

HA			
Pos.	491	obs:	exp:
tat	Y	0	0.53
tac	Y	1	0.47
act	T	1	0.31
acc	T	0	0.18
aca	T	0	0.43
acg	T	0	0.08
gtt	V	1	0.25
gtc	V	0	0.20
gta	V	0	0.22
gtg	V	0	0.33
gct	A	65666	17926.00
gcc	A	66	16432.00
gca	A	20	26605.00
gcg	A	19	4808.00
---	--	------	------
mPD		0.0033	0.69
		nPD:	0.
		N. weight:	1.4
		Sc. PD:	-0.11
		Sc. rank:	-60582.0

,

HA			
Pos.	492	obs:	exp:
atg	M	1	1.00
aaa	K	0	1.31
aag	K	2	0.69
gat	D	2	1.13
gac	D	0	0.87
gaa	E	249	38774.00
gag	E	65519	26994.00
ggt	G	0	0.13
ggc	G	0	0.14
gga	G	0	0.46
ggg	G	1	0.26
---	--	------	------
mPD		0.0078	0.48
		nPD:	0.02
		N. weight:	0.95
		Sc. PD:	-0.061
		Sc. rank:	-14406.0

,

HA			
Pos.	493	obs:	exp:
tat	Y	1	0.53
tac	Y	0	0.47
aat	N	7	3.92
aac	N	0	3.08
gat	D	65110	37172.00
gac	D	650	28588.00
gaa	E	3	2.95
gag	E	2	2.05
ggt	G	1	0.13
ggc	G	0	0.14
gga	G	0	0.46
ggg	G	0	0.26
---	--	------	------
mPD		0.020	0.49
		nPD:	0.04
		N. weight:	0.57
		Sc. PD:	-0.023
		Sc. rank:	6984.5

,

HA			
Pos.	494	obs:	exp:
tta	L	0	3.71
ttg	L	2	6.62
ctt	L	0	6.69
ctc	L	0	4.69
cta	L	0	4.91
ctg	L	30	5.37
att	I	4	27.73
atc	I	1	20.76
ata	I	74	30.51
atg	M	65468	65468.00
act	T	0	38.82
acc	T	0	22.10
aca	T	1	54.10
acg	T	124	9.98
aaa	K	0	0.66
aag	K	1	0.34
gtt	V	0	17.28
gtc	V	0	14.06
gta	V	0	14.96
gtg	V	69	22.70
---	--	------	------
mPD		0.0093	0.015
		nPD:	0.63
		N. weight:	0.0083
		Sc. PD:	0.0043
		Sc. rank:	419.5

,

HA			
Pos.	495	obs:	exp:
tct	S	0	0.32
tcc	S	0	0.30
tca	S	0	0.54
tcg	S	0	0.08
agt	S	0	0.35
agc	S	2	0.41
gct	A	0	1.36
gcc	A	5	1.25
gca	A	0	2.02
gcg	A	0	0.37
ggt	G	43	8854.00
ggc	G	19926	9179.00
gga	G	44320	30359.00
ggg	G	1478	17375.00
---	--	------	------
mPD		0.45	0.68
		nPD:	0.67
		N. weight:	0.48
		Sc. PD:	0.26
		Sc. rank:	24074.0

,

HA			
Pos.	496	obs:	exp:
tct	S	0	0.32
tcc	S	0	0.30
tca	S	0	0.54
tcg	S	0	0.08
aat	N	65688	36795.00
aac	N	79	28972.00
aaa	K	0	0.66
aag	K	1	0.34
agt	S	2	0.35
agc	S	0	0.41
gat	D	4	2.26
gac	D	0	1.74
---	--	------	------
mPD		0.0026	0.49
		nPD:	0.01
		N. weight:	0.63
		Sc. PD:	-0.047
		Sc. rank:	-26186.0

,

HA			
Pos.	497	obs:	exp:
tct	S	0	0.16
tcc	S	0	0.15
tca	S	0	0.27
tcg	S	0	0.04
tgt	C	1	0.39
tgc	C	0	0.61
tgg	W	1	1.00
agt	S	1	0.17
agc	S	0	0.21
ggt	G	65141	8854.00
ggc	G	618	9179.00
gga	G	7	30361.00
ggg	G	5	17376.00
---	--	------	------
mPD		0.019	0.68
		nPD:	0.03
		N. weight:	2.2
		Sc. PD:	-0.11
		Sc. rank:	4055.0

,

HA			
Pos.	498	obs:	exp:
ttt	F	11	5.35
ttc	F	0	5.65
tta	L	0	0.12
ttg	L	1	0.21
tgt	C	64801	25699.00
tgc	C	961	40063.00
ctt	L	0	0.21
ctc	L	0	0.15
cta	L	0	0.15
ctg	L	0	0.17
---	--	------	------
mPD		0.029	0.48
		nPD:	0.06
		N. weight:	0.95
		Sc. PD:	-0.02
		Sc. rank:	19413.0

,

HA			
Pos.	499	obs:	exp:
ttt	F	72	31979.00
ttc	F	65671	33764.00
tta	L	6	3.48
ttg	L	0	6.20
tct	S	0	0.16
tcc	S	1	0.15
tca	S	0	0.27
tcg	S	0	0.04
ctt	L	0	6.28
ctc	L	24	4.40
cta	L	0	4.61
ctg	L	0	5.03
agt	S	0	0.17
agc	S	0	0.21
---	--	------	------
mPD		0.0031	0.50
		nPD:	0.01
		N. weight:	0.73
		Sc. PD:	-0.053
		Sc. rank:	-27398.0

,

HA			
Pos.	500	obs:	exp:
caa	Q	12	7.34
cag	Q	0	4.66
cgt	R	0	1.82
cgc	R	0	3.31
cga	R	0	8.39
cgg	R	0	7.90
att	I	0	0.35
atc	I	0	0.26
ata	I	1	0.39
act	T	0	41.92
acc	T	0	23.87
aca	T	135	58.42
acg	T	0	10.78
aat	N	0	0.56
aac	N	1	0.44
aaa	K	65353	43078.00
aag	K	171	22446.00
aga	R	92	45.12
agg	R	0	25.45
gaa	E	9	5.31
gag	E	0	3.69
---	--	------	------
mPD		0.013	0.46
		nPD:	0.03
		N. weight:	0.45
		Sc. PD:	-0.024
		Sc. rank:	612.1

,

HA			
Pos.	501	obs:	exp:
ttt	F	2	0.97
ttc	F	0	1.03
tta	L	1	0.23
ttg	L	0	0.41
ctt	L	0	0.42
ctc	L	0	0.29
cta	L	1	0.31
ctg	L	0	0.34
att	I	29	23083.00
atc	I	89	17278.00
ata	I	65638	25395.00
atg	M	1	1.00
gtt	V	0	3.26
gtc	V	0	2.65
gta	V	13	2.82
gtg	V	0	4.28
---	--	------	------
mPD		0.0042	0.66
		nPD:	0.01
		N. weight:	1.
		Sc. PD:	-0.076
		Sc. rank:	-38727.0

,

HA			
Pos.	502	obs:	exp:
ttt	F	0	2.92
ttc	F	6	3.08
tat	Y	302	34666.00
tac	Y	65464	31100.00
cat	H	0	1.13
cac	H	2	0.87
---	--	------	------
mPD		0.0094	0.50
		nPD:	0.02
		N. weight:	0.79
		Sc. PD:	-0.049
		Sc. rank:	-7951.1

,

HA			
Pos.	503	obs:	exp:
tta	L	0	0.35
ttg	L	0	0.62
ctt	L	0	0.63
ctc	L	3	0.44
cta	L	0	0.46
ctg	L	0	0.50
cat	H	547	37098.00
cac	H	65224	28673.00
---	--	------	------
mPD		0.017	0.49
		nPD:	0.03
		N. weight:	0.86
		Sc. PD:	-0.041
		Sc. rank:	4411.4

,

HA			
Pos.	504	obs:	exp:
caa	Q	42	25.67
cag	Q	0	16.33
cgt	R	0	0.87
cgc	R	0	1.58
cga	R	0	4.01
cgg	R	0	3.78
act	T	0	0.62
acc	T	0	0.35
aca	T	2	0.87
acg	T	0	0.16
aat	N	0	1.68
aac	N	3	1.32
aaa	K	65598	43172.00
aag	K	69	22495.00
aga	R	44	21.58
agg	R	0	12.17
gaa	E	16	9.43
gag	E	0	6.57
---	--	------	------
mPD		0.0053	0.45
		nPD:	0.01
		N. weight:	0.46
		Sc. PD:	-0.031
		Sc. rank:	-11820.0

,

HA			
Pos.	505	obs:	exp:
tgt	C	65677	25704.00
tgc	C	97	40070.00
---	--	------	------
mPD		0.0029	0.48
		nPD:	0.01
		N. weight:	1.
		Sc. PD:	-0.075
		Sc. rank:	-39050.0

,

HA			
Pos.	506	obs:	exp:
aat	N	0	38.60
aac	N	69	30.40
gtt	V	0	0.25
gtc	V	1	0.20
gta	V	0	0.22
gtg	V	0	0.33
gat	D	202	37137.00
gac	D	65497	28562.00
ggt	G	0	0.67
ggc	G	5	0.70
gga	G	0	2.31
ggg	G	0	1.32
---	--	------	------
mPD		0.0084	0.49
		nPD:	0.02
		N. weight:	0.9
		Sc. PD:	-0.057
		Sc. rank:	-12311.0

,

HA			
Pos.	507	obs:	exp:
tct	S	0	0.48
tcc	S	0	0.45
tca	S	0	0.81
tcg	S	0	0.12
att	I	1	0.35
atc	I	0	0.26
ata	I	0	0.39
aat	N	65693	36791.00
aac	N	67	28969.00
aaa	K	1	0.66
aag	K	0	0.34
agt	S	3	0.52
agc	S	0	0.62
gat	D	9	5.09
gac	D	0	3.91
---	--	------	------
mPD		0.0025	0.49
		nPD:	0.
		N. weight:	0.63
		Sc. PD:	-0.047
		Sc. rank:	-26501.0

,

HA			
Pos.	508	obs:	exp:
tct	S	0	0.48
tcc	S	3	0.45
tca	S	0	0.81
tcg	S	0	0.12
att	I	0	0.35
atc	I	1	0.26
ata	I	0	0.39
act	T	0	40.37
acc	T	128	22.98
aca	T	0	56.26
acg	T	2	10.38
agt	S	0	0.52
agc	S	0	0.62
gtt	V	0	20.29
gtc	V	81	16.50
gta	V	0	17.56
gtg	V	0	26.65
gct	A	354	17861.00
gcc	A	64947	16373.00
gca	A	233	26509.00
gcg	A	0	4791.00
gat	D	0	14.13
gac	D	25	10.87
---	--	------	------
mPD		0.025	0.70
		nPD:	0.04
		N. weight:	1.5
		Sc. PD:	-0.066
		Sc. rank:	10955.0

,

HA			
Pos.	509	obs:	exp:
tgt	C	129	25704.00
tgc	C	65645	40070.00
---	--	------	------
mPD		0.0039	0.48
		nPD:	0.01
		N. weight:	0.53
		Sc. PD:	-0.038
		Sc. rank:	-17704.0

,

HA			
Pos.	510	obs:	exp:
tta	L	1	0.12
ttg	L	0	0.21
ctt	L	0	0.21
ctc	L	0	0.15
cta	L	0	0.15
ctg	L	0	0.17
att	I	15	21190.00
atc	I	16	15861.00
ata	I	60333	23312.00
atg	M	5400	5400.00
act	T	0	1.24
acc	T	0	0.71
aca	T	4	1.73
acg	T	0	0.32
gtt	V	0	1.25
gtc	V	0	1.02
gta	V	4	1.08
gtg	V	1	1.65
---	--	------	------
mPD		0.15	0.71
		nPD:	0.22
		N. weight:	0.96
		Sc. PD:	0.12
		Sc. rank:	37850.0

,

HA			
Pos.	511	obs:	exp:
tct	S	0	4.52
tcc	S	0	4.18
tca	S	0	7.53
tcg	S	0	1.10
tgt	C	1	0.39
tgc	C	0	0.61
cgt	R	0	0.24
cgc	R	0	0.43
cga	R	0	1.09
cgg	R	0	1.03
aaa	K	1	0.66
aag	K	0	0.34
agt	S	26	4.89
agc	S	2	5.78
aga	R	11	5.89
agg	R	1	3.32
gtt	V	0	3.51
gtc	V	0	2.85
gta	V	13	3.03
gtg	V	1	4.61
gct	A	0	1.91
gcc	A	0	1.75
gca	A	7	2.83
gcg	A	0	0.51
gat	D	19	10.74
gac	D	0	8.26
gaa	E	2582	1555.00
gag	E	56	1083.00
ggt	G	7181	8489.00
ggc	G	269	8800.00
gga	G	49163	29107.00
ggg	G	6441	16658.00
---	--	------	------
mPD		0.44	0.75
		nPD:	0.58
		N. weight:	0.31
		Sc. PD:	0.15
		Sc. rank:	15756.0

,

HA			
Pos.	512	obs:	exp:
ttt	F	0	0.49
ttc	F	1	0.51
tta	L	5	0.58
ttg	L	0	1.03
tct	S	5	10611.00
tcc	S	45	9826.00
tca	S	65639	17674.00
tcg	S	64	2588.00
ctt	L	0	1.05
ctc	L	0	0.73
cta	L	0	0.77
ctg	L	0	0.84
cct	P	0	0.67
ccc	P	0	0.37
cca	P	2	0.68
ccg	P	0	0.28
act	T	0	2.48
acc	T	0	1.41
aca	T	8	3.46
acg	T	0	0.64
agt	S	2	11491.00
agc	S	0	13565.00
gct	A	0	0.82
gcc	A	0	0.75
gca	A	3	1.21
gcg	A	0	0.22
---	--	------	------
mPD		0.0043	1.6
		nPD:	0.
		N. weight:	1.4
		Sc. PD:	-0.11
		Sc. rank:	-68359.0

,

HA			
Pos.	513	obs:	exp:
tta	L	4	1.28
ttg	L	0	2.27
ctt	L	0	2.30
ctc	L	0	1.61
cta	L	7	1.69
ctg	L	0	1.85
att	I	16	23082.00
atc	I	18452	17278.00
ata	I	47286	25394.00
gtt	V	0	2.25
gtc	V	2	1.83
gta	V	7	1.95
gtg	V	0	2.96
---	--	------	------
mPD		0.40	0.66
		nPD:	0.61
		N. weight:	0.51
		Sc. PD:	0.26
		Sc. rank:	25796.0

,

HA			
Pos.	514	obs:	exp:
cgt	R	0	1304.00
cgc	R	0	2366.00
cga	R	4	5999.00
cgg	R	0	5646.00
att	I	0	3.51
atc	I	0	2.63
ata	I	10	3.86
aaa	K	16	11.18
aag	K	1	5.82
aga	R	61404	32248.00
agg	R	4339	18186.00
---	--	------	------
mPD		0.12	0.89
		nPD:	0.14
		N. weight:	0.56
		Sc. PD:	0.03
		Sc. rank:	19183.0

,

HA			
Pos.	515	obs:	exp:
tct	S	0	1.29
tcc	S	0	1.20
tca	S	0	2.15
tcg	S	0	0.31
cat	H	2	1.13
cac	H	0	0.87
atg	M	1	1.00
act	T	3	0.93
acc	T	0	0.53
aca	T	0	1.30
acg	T	0	0.24
aat	N	65618	36787.00
aac	N	135	28966.00
aaa	K	0	0.66
aag	K	1	0.34
agt	S	8	1.40
agc	S	0	1.65
gat	D	6	3.39
gac	D	0	2.61
---	--	------	------
mPD		0.0048	0.49
		nPD:	0.01
		N. weight:	0.63
		Sc. PD:	-0.044
		Sc. rank:	-19007.0

,

HA			
Pos.	516	obs:	exp:
cgt	R	0	0.02
cgc	R	0	0.04
cga	R	0	0.09
cgg	R	0	0.09
aaa	K	2	1.31
aag	K	0	0.69
aga	R	1	0.49
agg	R	0	0.28
gat	D	2	1.70
gac	D	1	1.30
gaa	E	27622	16305.00
gag	E	34	11351.00
ggt	G	7	5131.00
ggc	G	7	5319.00
gga	G	37928	17593.00
ggg	G	170	10069.00
---	--	------	------
mPD		0.49	1.1
		nPD:	0.45
		N. weight:	0.72
		Sc. PD:	0.25
		Sc. rank:	34383.0

,

HA			
Pos.	517	obs:	exp:
att	I	20	7.02
atc	I	0	5.26
ata	I	0	7.72
act	T	65426	20417.00
acc	T	294	11624.00
aca	T	6	28452.00
acg	T	18	5251.00
aat	N	6	3.36
aac	N	0	2.64
gct	A	4	1.09
gcc	A	0	1.00
gca	A	0	1.62
gcg	A	0	0.29
---	--	------	------
mPD		0.011	0.68
		nPD:	0.02
		N. weight:	1.3
		Sc. PD:	-0.081
		Sc. rank:	-20834.0

,

HA			
Pos.	518	obs:	exp:
tat	Y	65671	34670.00
tac	Y	103	31104.00
---	--	------	------
mPD		0.0031	0.50
		nPD:	0.01
		N. weight:	0.69
		Sc. PD:	-0.051
		Sc. rank:	-26089.0

,

HA			
Pos.	519	obs:	exp:
tct	S	0	0.16
tcc	S	0	0.15
tca	S	0	0.27
tcg	S	0	0.04
cat	H	0	1.13
cac	H	2	0.87
aat	N	4	661.90
aac	N	1179	521.10
aaa	K	1	0.66
aag	K	0	0.34
agt	S	0	0.17
agc	S	1	0.21
gtt	V	0	0.25
gtc	V	1	0.20
gta	V	0	0.22
gtg	V	0	0.33
gct	A	0	0.27
gcc	A	1	0.25
gca	A	0	0.40
gcg	A	0	0.07
gat	D	508	36504.00
gac	D	64071	28075.00
gaa	E	5	2.95
gag	E	0	2.05
ggt	G	0	0.13
ggc	G	1	0.14
gga	G	0	0.46
ggg	G	0	0.26
---	--	------	------
mPD		0.051	0.53
		nPD:	0.1
		N. weight:	0.87
		Sc. PD:	0.011
		Sc. rank:	24664.0

,

HA			
Pos.	520	obs:	exp:
tta	L	0	0.12
ttg	L	0	0.21
tat	Y	0	1.05
tac	Y	2	0.95
ctt	L	0	0.21
ctc	L	1	0.15
cta	L	0	0.15
ctg	L	0	0.17
cct	P	0	1.33
ccc	P	4	0.75
cca	P	0	1.35
ccg	P	0	0.57
cat	H	1236	37094.00
cac	H	64529	28671.00
cgt	R	1	0.04
cgc	R	1	0.07
cga	R	0	0.18
cgg	R	0	0.17
aga	R	0	0.98
agg	R	0	0.55
---	--	------	------
mPD		0.037	0.49
		nPD:	0.08
		N. weight:	0.81
		Sc. PD:	-0.006
		Sc. rank:	19206.0

,

HA			
Pos.	521	obs:	exp:
tct	S	0	7.75
tcc	S	0	7.17
tca	S	0	12.90
tcg	S	0	1.89
tat	Y	55	31.10
tac	Y	4	27.90
cct	P	0	0.67
ccc	P	0	0.37
cca	P	2	0.68
ccg	P	0	0.28
cat	H	32	18.05
cac	H	0	13.95
act	T	1009	313.30
acc	T	0	178.40
aca	T	0	436.70
acg	T	0	80.59
aat	N	52696	29511.00
aac	N	52	23237.00
aaa	K	2	15.12
aag	K	21	7.88
agt	S	48	8.39
agc	S	0	9.90
gtt	V	1	0.25
gtc	V	0	0.20
gta	V	0	0.22
gtg	V	0	0.33
gct	A	4	1.09
gcc	A	0	1.00
gca	A	0	1.62
gcg	A	0	0.29
gat	D	11816	6691.00
gac	D	21	5146.00
gaa	E	2	4.13
gag	E	5	2.87
ggt	G	4	0.54
ggc	G	0	0.56
gga	G	0	1.85
ggg	G	0	1.06
---	--	------	------
mPD		0.33	0.83
		nPD:	0.4
		N. weight:	0.64
		Sc. PD:	0.19
		Sc. rank:	29240.0

,

HA			
Pos.	522	obs:	exp:
tta	L	6	1.51
ttg	L	7	2.69
ctt	L	0	2.72
ctc	L	0	1.91
cta	L	0	2.00
ctg	L	0	2.18
att	I	0	58.62
atc	I	0	43.88
ata	I	167	64.49
atg	M	81	81.00
aaa	K	2	1.31
aag	K	0	0.69
gtt	V	19	16406.00
gtc	V	43	13346.00
gta	V	15955	14196.00
gtg	V	49481	21550.00
gct	A	0	3.00
gcc	A	0	2.75
gca	A	8	4.45
gcg	A	3	0.80
gaa	E	1	0.59
gag	E	0	0.41
ggt	G	0	0.13
ggc	G	0	0.14
gga	G	0	0.46
ggg	G	1	0.26
---	--	------	------
mPD		0.38	0.75
		nPD:	0.51
		N. weight:	0.72
		Sc. PD:	0.29
		Sc. rank:	34884.0

,

HA

Pos.	523	obs:	exp:
tct	S	0	0.16
tcc	S	1	0.15
tca	S	0	0.27
tcg	S	0	0.04
tat	Y	30	34668.00
tac	Y	65740	31102.00
tgt	C	0	0.39
tgc	C	1	0.61
cat	H	0	0.56
cac	H	1	0.44
aat	N	0	0.56
aac	N	1	0.44
agt	S	0	0.17
agc	S	0	0.21
---	--	------	------
mPD		0.0010	0.50
		nPD:	0.
		N. weight:	0.82
		Sc. PD:	-0.064
		Sc. rank:	-40589.0

,

HA

Pos.	524	obs:	exp:
tct	S	0	0.32
tcc	S	0	0.30
tca	S	2	0.54
tcg	S	0	0.08
tgg	W	1	1.00
cgt	R	0	1304.00
cgc	R	0	2366.00
cga	R	1	6001.00
cgg	R	138	5647.00
act	T	0	0.31
acc	T	0	0.18
aca	T	0	0.43
acg	T	1	0.08
aaa	K	1	0.66
aag	K	0	0.34
agt	S	0	0.35
agc	S	0	0.41
aga	R	8273	32259.00
agg	R	57357	18192.00
---	--	------	------
mPD		0.22	0.89
		nPD:	0.25
		N. weight:	0.91
		Sc. PD:	0.15
		Sc. rank:	37433.0

,

HA

Pos.	525	obs:	exp:
tct	S	0	0.16
tcc	S	0	0.15
tca	S	0	0.27
tcg	S	0	0.04
tat	Y	1	0.53
tac	Y	0	0.47
aat	N	200	190.80
aac	N	141	150.20
agt	S	0	0.17
agc	S	1	0.21
gtt	V	1	0.25
gtc	V	0	0.20
gta	V	0	0.22
gtg	V	0	0.33
gct	A	1	0.55
gcc	A	1	0.50
gca	A	0	0.81
gcg	A	0	0.15
gat	D	64937	36968.00
gac	D	462	28431.00
gaa	E	7	7.66
gag	E	6	5.34
ggt	G	16	2.15
ggc	G	0	2.23
gga	G	0	7.39
ggg	G	0	4.23
---	--	------	------
mPD		0.030	0.50
		nPD:	0.06
		N. weight:	0.58
		Sc. PD:	-0.014
		Sc. rank:	11615.0

,

HA

Pos.	526	obs:	exp:
gtt	V	0	0.75
gtc	V	0	0.61
gta	V	3	0.65
gtg	V	0	0.99
gct	A	0	0.27
gcc	A	0	0.25
gca	A	1	0.40
gcg	A	0	0.07
gaa	E	65498	38776.00
gag	E	272	26994.00
---	--	------	------
mPD		0.0084	0.48
		nPD:	0.02
		N. weight:	0.56
		Sc. PD:	-0.035
		Sc. rank:	-7454.6

,

HA

Pos.	527	obs:	exp:
tct	S	0	4.36
tcc	S	0	4.03
tca	S	27	7.26
tcg	S	0	1.06
act	T	0	0.62
acc	T	0	0.35
aca	T	2	0.87
acg	T	0	0.16
agt	S	0	4.72
agc	S	0	5.57
gct	A	956	17919.00
gcc	A	70	16426.00
gca	A	64626	26594.00
gcg	A	93	4806.00
---	--	------	------
mPD		0.034	0.70
		nPD:	0.05
		N. weight:	0.9
		Sc. PD:	-0.029
		Sc. rank:	14764.0

,

HA

Pos.	528	obs:	exp:
ttt	F	1	0.97
ttc	F	1	1.03
tta	L	61899	7627.00
ttg	L	3585	13590.00
tct	S	0	0.16
tcc	S	0	0.15
tca	S	1	0.27
tcg	S	0	0.04
ctt	L	0	13750.00
ctc	L	0	9639.00
cta	L	240	10093.00
ctg	L	7	11032.00
att	I	0	8.42
atc	I	0	6.31
ata	I	24	9.27
aaa	K	2	1.31
aag	K	0	0.69
agt	S	0	0.17
agc	S	0	0.21
gtt	V	0	3.51
gtc	V	0	2.85
gta	V	13	3.03
gtg	V	1	4.61
---	--	------	------
mPD		0.11	1.2
		nPD:	0.1
		N. weight:	2.1
		Sc. PD:	0.026
		Sc. rank:	58861.0

,

HA

Pos.	529	obs:	exp:
tta	L	2	0.23
ttg	L	0	0.41
tct	S	0	9.68
tcc	S	0	8.97
tca	S	0	16.13
tcg	S	0	2.36
ctt	L	0	0.42
ctc	L	0	0.29
cta	L	0	0.31
ctg	L	0	0.34
cat	H	0	0.56
cac	H	1	0.44
att	I	1	0.35
atc	I	0	0.26
ata	I	0	0.39
act	T	0	1.86
acc	T	5	1.06
aca	T	1	2.60
acg	T	0	0.48
aat	N	353	36756.00
aac	N	65345	28942.00
aaa	K	2	1.31
aag	K	0	0.69
agt	S	0	10.49
agc	S	60	12.38
gat	D	0	2.26
gac	D	4	1.74
---	--	------	------
mPD		0.013	0.50
		nPD:	0.03
		N. weight:	0.87
		Sc. PD:	-0.047
		Sc. rank:	-384.6

,

HA

Pos.	530	obs:	exp:
tct	S	0	2.42
tcc	S	0	2.24
tca	S	0	4.03
tcg	S	0	0.59
cat	H	0	0.56
cac	H	1	0.44
act	T	0	1.55
acc	T	5	0.88
aca	T	0	2.16
acg	T	0	0.40
aat	N	492	36742.00
aac	N	65181	28931.00
aaa	K	78	52.59
aag	K	2	27.41
agt	S	0	2.62
agc	S	15	3.09
---	--	------	------
mPD		0.018	0.50
		nPD:	0.04
		N. weight:	0.85
		Sc. PD:	-0.038
		Sc. rank:	7123.5

,

HA			
Pos.	531	obs:	exp:
cgt	R	105	1304.00
cgc	R	1	2367.00
cga	R	2011	6001.00
cgg	R	63493	5648.00
aga	R	6	32261.00
agg	R	157	18193.00
ggt	G	0	0.13
ggc	G	0	0.14
gga	G	0	0.46
ggg	G	1	0.26
---	--	------	------
mPD		0.068	0.89
		nPD:	0.08
		N. weight:	2.5
		Sc. PD:	-0.017
		Sc. rank:	61073.0

,

HA			
Pos.	532	obs:	exp:
ttt	F	968	31987.00
ttc	F	64792	33773.00
tta	L	2	1.16
ttg	L	0	2.07
tct	S	0	0.16
tcc	S	1	0.15
tca	S	0	0.27
tcg	S	0	0.04
tat	Y	0	0.53
tac	Y	1	0.47
ctt	L	0	2.09
ctc	L	8	1.47
cta	L	0	1.54
ctg	L	0	1.68
agt	S	0	0.17
agc	S	0	0.21
gaa	E	2	1.18
gag	E	0	0.82
---	--	------	------
mPD		0.030	0.50
		nPD:	0.06
		N. weight:	0.65
		Sc. PD:	-0.015
		Sc. rank:	13068.0

,

HA			
Pos.	533	obs:	exp:
tta	L	0	0.35
ttg	L	0	0.62
ctt	L	0	0.63
ctc	L	0	0.44
cta	L	0	0.46
ctg	L	3	0.50
cct	P	0	0.67
ccc	P	0	0.37
cca	P	0	0.68
ccg	P	2	0.28
cat	H	170	97.02
cac	H	2	74.98
caa	Q	305	40050.00
cag	Q	65214	25469.00
cgt	R	0	0.63
cgc	R	0	1.15
cga	R	0	2.92
cgg	R	32	2.75
aaa	K	2	29.58
aag	K	43	15.42
aga	R	0	15.70
agg	R	0	8.85
gaa	E	1	0.59
gag	E	0	0.41
---	--	------	------
mPD		0.017	0.48
		nPD:	0.04
		N. weight:	1.
		Sc. PD:	-0.046
		Sc. rank:	7271.6

,

HA			
Pos.	534	obs:	exp:
att	I	135	23078.00
atc	I	65567	17274.00
ata	I	40	25389.00
gtt	V	0	8.02
gtc	V	32	6.52
gta	V	0	6.94
gtg	V	0	10.53
---	--	------	------
mPD		0.0063	0.66
		nPD:	0.01
		N. weight:	1.5
		Sc. PD:	-0.1
		Sc. rank:	-44645.0

,

HA			
Pos.	535	obs:	exp:
tct	S	0	0.32
tcc	S	0	0.30
tca	S	0	0.54
tcg	S	2	0.08
caa	Q	24	14.67
cag	Q	0	9.33
cgt	R	0	1.69
cgc	R	0	3.06
cga	R	0	7.76
cgg	R	0	7.30
act	T	0	0.62
acc	T	0	0.35
aca	T	0	0.87
acg	T	2	0.16
aat	N	27	15.67
aac	N	1	12.33
aaa	K	3593	43146.00
aag	K	62035	22482.00
agt	S	0	0.35
agc	S	0	0.41
aga	R	1	41.69
agg	R	84	23.51
gat	D	2	1.13
gac	D	0	0.87
gaa	E	0	1.77
gag	E	3	1.23
---	--	------	------
mPD		0.11	0.46
		nPD:	0.24
		N. weight:	0.91
		Sc. PD:	0.13
		Sc. rank:	36982.0

,

HA			
Pos.	536	obs:	exp:
tct	S	0	0.48
tcc	S	0	0.45
tca	S	0	0.81
tcg	S	0	0.12
cgt	R	0	0.20
cgc	R	0	0.36
cga	R	0	0.91
cgg	R	0	0.86
agt	S	2	0.52
agc	S	1	0.62
aga	R	10	4.90
agg	R	0	2.77
gat	D	1	0.57
gac	D	0	0.43
gaa	E	40	23.58
gag	E	0	16.42
ggt	G	846	8848.00
ggc	G	505	9172.00
gga	G	63073	30337.00
ggg	G	1296	17363.00
---	--	------	------
mPD		0.080	0.68
		nPD:	0.12
		N. weight:	0.66
		Sc. PD:	0.022
		Sc. rank:	21749.0

,

HA			
Pos.	537	obs:	exp:
ttt	F	1	0.49
ttc	F	0	0.51
tta	L	0	0.12
ttg	L	0	0.21
ctt	L	1	0.21
ctc	L	0	0.15
cta	L	0	0.15
ctg	L	0	0.17
att	I	580	204.30
atc	I	0	152.90
ata	I	2	224.80
gtt	V	65084	16328.00
gtc	V	54	13282.00
gta	V	47	14129.00
gtg	V	2	21448.00
gct	A	2	0.55
gcc	A	0	0.50
gca	A	0	0.81
gcg	A	0	0.15
gat	D	1	0.57
gac	D	0	0.43
---	--	------	------
mPD		0.021	0.76
		nPD:	0.03
		N. weight:	1.5
		Sc. PD:	-0.08
		Sc. rank:	1954.2

,

HA			
Pos.	538	obs:	exp:
caa	Q	0	88.64
cag	Q	145	56.36
aaa	K	0	5.92
aag	K	9	3.08
gtt	V	0	1.00
gtc	V	0	0.82
gta	V	0	0.87
gtg	V	4	1.32
gat	D	9	5.09
gac	D	0	3.91
gaa	E	386	38678.00
gag	E	65219	26927.00
ggt	G	0	0.27
ggc	G	0	0.28
gga	G	0	0.92
ggg	G	2	0.53
---	--	------	------
mPD		0.017	0.49
		nPD:	0.03
		N. weight:	0.94
		Sc. PD:	-0.044
		Sc. rank:	5645.9

,

HA			
Pos.	539	obs:	exp:
tta	L	1	7632.00
ttg	L	163	13597.00
ctt	L	193	13758.00
ctc	L	0	9644.00
cta	L	465	10099.00
ctg	L	64945	11038.00
atg	M	3	3.00
gtt	V	0	1.00
gtc	V	0	0.82
gta	V	0	0.87
gtg	V	4	1.32
---	--	------	------
mPD		0.025	1.2
		nPD:	0.02
		N. weight:	1.9
		Sc. PD:	-0.11
		Sc. rank:	-9887.9

,

HA			
Pos.	540	obs:	exp:
caa	Q	1	1.22
cag	Q	1	0.78
cgt	R	0	3.15
cgc	R	0	5.72
cga	R	0	14.51
cgg	R	0	13.65
atg	M	19	19.00
act	T	0	2.79
acc	T	0	1.59
aca	T	0	3.89
acg	T	9	0.72
aat	N	90	50.91
aac	N	1	40.09
aaa	K	2768	43044.00
aag	K	62704	22428.00
aga	R	3	77.99
agg	R	156	43.98
gat	D	2	1.13
gac	D	0	0.87
gaa	E	3	11.79
gag	E	17	8.21
---	--	------	------
mPD		0.090	0.46
		nPD:	0.2
		N. weight:	0.96
		Sc. PD:	0.1
		Sc. rank:	37015.0

,

HA			
Pos.	541	obs:	exp:
tta	L	38	4.41
ttg	L	0	7.86
tct	S	95	10602.00
tcc	S	1	9818.00
tca	S	65480	17659.00
tcg	S	123	2586.00
ctt	L	0	7.95
ctc	L	0	5.57
cta	L	0	5.84
ctg	L	0	6.38
cct	P	0	3.33
ccc	P	0	1.87
cca	P	10	3.38
ccg	P	0	1.41
act	T	0	4.35
acc	T	0	2.48
aca	T	14	6.06
acg	T	0	1.12
agt	S	0	11481.00
agc	S	0	13553.00
gct	A	0	3.54
gcc	A	0	3.25
gca	A	13	5.26
gcg	A	0	0.95
---	--	------	------
mPD		0.0089	1.6
		nPD:	0.01
		N. weight:	1.4
		Sc. PD:	-0.11
		Sc. rank:	-57898.0

,

HA			
Pos.	542	obs:	exp:
tgg	W	6	6.00
cgt	R	0	0.06
cgc	R	0	0.11
cga	R	0	0.27
cgg	R	1	0.26
act	T	0	0.62
acc	T	0	0.35
aca	T	2	0.87
acg	T	0	0.16
aga	R	1	1.47
agg	R	1	0.83
gtt	V	0	0.25
gtc	V	0	0.20
gta	V	0	0.22
gtg	V	1	0.33
gaa	E	0	94.92
gag	E	161	66.08
ggt	G	32	8832.00
ggc	G	23	9156.00
gga	G	1960	30283.00
ggg	G	63586	17331.00
---	--	------	------
mPD		0.065	0.68
		nPD:	0.09
		N. weight:	1.3
		Sc. PD:	0.014
		Sc. rank:	36165.0

,

HA			
Pos.	543	obs:	exp:
ttt	F	0	0.49
ttc	F	1	0.51
tat	Y	56	34657.00
tac	Y	65692	31091.00
tgt	C	0	1.56
tgc	C	4	2.44
cat	H	0	10.15
cac	H	18	7.85
cgt	R	0	0.04
cgc	R	0	0.07
cga	R	0	0.18
cgg	R	0	0.17
aat	N	0	0.56
aac	N	1	0.44
aga	R	0	0.98
agg	R	2	0.55
---	--	------	------
mPD		0.0026	0.50
		nPD:	0.01
		N. weight:	0.82
		Sc. PD:	-0.061
		Sc. rank:	-34196.0

,

HA			
Pos.	544	obs:	exp:
caa	Q	7	4.28
cag	Q	0	2.72
cgt	R	0	0.30
cgc	R	0	0.54
cga	R	0	1.37
cgg	R	0	1.29
att	I	1	0.70
atc	I	1	0.53
ata	I	0	0.77
act	T	0	0.93
acc	T	0	0.53
aca	T	3	1.30
acg	T	0	0.24
aat	N	1	0.56
aac	N	0	0.44
aaa	K	65539	43223.00
aag	K	206	22522.00
aga	R	15	7.36
agg	R	0	4.15
gaa	E	1	0.59
gag	E	0	0.41
---	--	------	------
mPD		0.0072	0.45
		nPD:	0.02
		N. weight:	0.44
		Sc. PD:	-0.028
		Sc. rank:	-6747.7

,

HA			
Pos.	545	obs:	exp:
tat	Y	2	2.11
tac	Y	2	1.89
aat	N	75	41.96
aac	N	0	33.04
aaa	K	1	0.66
aag	K	0	0.34
gat	D	65179	37130.00
gac	D	507	28556.00
gaa	E	3	4.13
gag	E	4	2.87
ggt	G	1	0.13
ggc	G	0	0.14
gga	G	0	0.46
ggg	G	0	0.26
---	--	------	------
mPD		0.018	0.49
		nPD:	0.04
		N. weight:	0.58
		Sc. PD:	-0.026
		Sc. rank:	5336.5

,

HA			
Pos.	546	obs:	exp:
tta	L	0	0.35
ttg	L	3	0.62
tgt	C	1	0.39
tgc	C	0	0.61
tgg	W	65763	65763.00
ctt	L	0	0.63
ctc	L	0	0.44
cta	L	0	0.46
ctg	L	0	0.50
caa	Q	0	1.22
cag	Q	2	0.78
cgt	R	0	0.08
cgc	R	0	0.14
cga	R	0	0.36
cgg	R	4	0.34
aga	R	0	1.96
agg	R	0	1.11
ggt	G	0	0.13
ggc	G	0	0.14
gga	G	0	0.46
ggg	G	1	0.26
---	--	------	------
mPD		0.00040	0.00065
		nPD:	0.61
		N. weight:	0.00031
		Sc. PD:	0.00016
		Sc. rank:	15.8

,

HA			
Pos.	547	obs:	exp:
ttt	F	0	10.70
ttc	F	22	11.30
tta	L	0	1.39
ttg	L	0	2.48
ctt	L	0	2.51
ctc	L	12	1.76
cta	L	0	1.84
ctg	L	0	2.01
att	I	312	23040.00
atc	I	65277	17246.00
ata	I	43	25347.00
gtt	V	0	27.05
gtc	V	108	22.01
gta	V	0	23.41
gtg	V	0	35.53
---	--	------	------
mPD		0.015	0.66
		nPD:	0.02
		N. weight:	1.4
		Sc. PD:	-0.083
		Sc. rank:	-6070.8

,

HA			
Pos.	548	obs:	exp:
tta	L	83	7626.00
ttg	L	2	13587.00
ctt	L	2	13748.00
ctc	L	0	9637.00
cta	L	65527	10091.00
ctg	L	104	11029.00
cct	P	0	0.67
ccc	P	0	0.37
cca	P	2	0.68
ccg	P	0	0.28
caa	Q	7	4.28
cag	Q	0	2.72
att	I	0	16.15
atc	I	0	12.09
ata	I	46	17.77
gtt	V	0	0.25
gtc	V	0	0.20
gta	V	1	0.22
gtg	V	0	0.33
---	--	------	------
mPD		0.0076	1.2
		nPD:	0.01
		N. weight:	2.
		Sc. PD:	-0.15
		Sc. rank:	-75472.0

,

HA			
Pos.	549	obs:	exp:
tgg	W	65772	65772.00
gct	A	0	0.55
gcc	A	0	0.50
gca	A	0	0.81
gcg	A	2	0.15
---	--	------	------
mPD		0.00012	0.00018
		nPD:	0.68
		N. weight:	0.000088
		Sc. PD:	0.00005
		Sc. rank:	4.4

,

HA			
Pos.	550	obs:	exp:
ttt	F	1	0.49
ttc	F	0	0.51
tct	S	0	0.16
tcc	S	0	0.15
tca	S	0	0.27
tcg	S	0	0.04
att	I	65655	23083.00
atc	I	96	17278.00
ata	I	4	25394.00
atg	M	14	14.00
agt	S	1	0.17
agc	S	0	0.21
gtt	V	3	0.75
gtc	V	0	0.61
gta	V	0	0.65
gtg	V	0	0.99
---	--	------	------
mPD		0.0036	0.66
		nPD:	0.01
		N. weight:	1.1
		Sc. PD:	-0.085
		Sc. rank:	-45964.0

,

HA			
Pos.	551	obs:	exp:
tct	S	57	10613.00
tcc	S	65685	9829.00
tca	S	28	17678.00
tcg	S	0	2589.00
tat	Y	1	1.05
tac	Y	1	0.95
cct	P	1	0.33
ccc	P	0	0.19
cca	P	0	0.34
ccg	P	0	0.14
act	T	0	0.31
acc	T	1	0.18
aca	T	0	0.43
acg	T	0	0.08
agt	S	0	11494.00
agc	S	0	13568.00
---	--	------	------
mPD		0.0028	1.6
		nPD:	0.
		N. weight:	2.1
		Sc. PD:	-0.16
		Sc. rank:	-104620.0

,

HA			
Pos.	552	obs:	exp:
ttt	F	65725	31987.00
ttc	F	35	33773.00
tta	L	0	1.16
ttg	L	1	2.07
tct	S	1	0.48
tcc	S	0	0.45
tca	S	2	0.81
tcg	S	0	0.12
tat	Y	1	0.53
tac	Y	0	0.47
ctt	L	9	2.09
ctc	L	0	1.47
cta	L	0	1.54
ctg	L	0	1.68
agt	S	0	0.52
agc	S	0	0.62
---	--	------	------
mPD		0.0016	0.50
		nPD:	0.
		N. weight:	0.79
		Sc. PD:	-0.06
		Sc. rank:	-36968.0

,

HA			
Pos.	553	obs:	exp:
tct	S	0	2.58
tcc	S	16	2.39
tca	S	0	4.30
tcg	S	0	0.63
cct	P	0	0.33
ccc	P	1	0.19
cca	P	0	0.34
ccg	P	0	0.14
act	T	2	13.66
acc	T	42	7.78
aca	T	0	19.04
acg	T	0	3.51
agt	S	0	2.80
agc	S	0	3.30
gct	A	0	17910.00
gcc	A	65712	16418.00
gca	A	1	26581.00
gcg	A	0	4804.00
---	--	------	------
mPD		0.0019	0.70
		nPD:	0.
		N. weight:	1.5
		Sc. PD:	-0.12
		Sc. rank:	-72183.0

,

HA			
Pos.	554	obs:	exp:
att	I	3	18997.00
atc	I	1	14220.00
ata	I	54113	20900.00
atg	M	11407	11407.00
act	T	0	75.15
acc	T	0	42.79
aca	T	235	104.70
acg	T	7	19.33
aaa	K	2	1.31
aag	K	0	0.69
gtt	V	0	0.75
gtc	V	2	0.61
gta	V	0	0.65
gtg	V	1	0.99
gaa	E	3	1.77
gag	E	0	1.23
---	--	------	------
mPD		0.29	0.74
		nPD:	0.4
		N. weight:	0.87
		Sc. PD:	0.26
		Sc. rank:	39244.0

,

HA			
Pos.	555	obs:	exp:
tct	S	4	10613.00
tcc	S	2	9829.00
tca	S	65748	17678.00
tcg	S	17	2589.00
act	T	0	0.31
acc	T	0	0.18
aca	T	1	0.43
acg	T	0	0.08
agt	S	0	11494.00
agc	S	0	13568.00
gct	A	0	0.55
gcc	A	2	0.50
gca	A	0	0.81
gcg	A	0	0.15
---	--	------	------
mPD		0.00085	1.6
		nPD:	0.
		N. weight:	1.4
		Sc. PD:	-0.11
		Sc. rank:	-74477.0

,

HA			
Pos.	556	obs:	exp:
ttt	F	1	0.49
ttc	F	0	0.51
tct	S	0	2.74
tcc	S	0	2.54
tca	S	0	4.57
tcg	S	0	0.67
tgt	C	64368	25692.00
tgc	C	1376	40052.00
agt	S	17	2.97
agc	S	0	3.51
gtt	V	1	0.25
gtc	V	0	0.20
gta	V	0	0.22
gtg	V	0	0.33
ggt	G	11	1.48
ggc	G	0	1.54
gga	G	0	5.08
ggg	G	0	2.91
---	--	------	------
mPD		0.042	0.48
		nPD:	0.09
		N. weight:	0.92
		Sc. PD:	0.0039
		Sc. rank:	23604.0

,

HA			
Pos.	557	obs:	exp:
ttt	F	65508	31914.00
ttc	F	102	33696.00
tta	L	1	16.83
ttg	L	3	29.98
tct	S	11	2.10
tcc	S	0	1.94
tca	S	2	3.49
tcg	S	0	0.51
tat	Y	1	0.53
tac	Y	0	0.47
tgt	C	2	0.78
tgc	C	0	1.22
ctt	L	141	30.33
ctc	L	0	21.26
cta	L	0	22.27
ctg	L	0	24.34
att	I	1	0.35
atc	I	0	0.26
ata	I	0	0.39
agt	S	0	2.27
agc	S	0	2.68
gtt	V	2	0.50
gtc	V	0	0.41
gta	V	0	0.43
gtg	V	0	0.66
---	--	------	------
mPD		0.0081	0.50
		nPD:	0.02
		N. weight:	0.79
		Sc. PD:	-0.05
		Sc. rank:	-11741.0

,

HA			
Pos.	558	obs:	exp:
ttt	F	3	1.46
ttc	F	0	1.54
tta	L	101	7629.00
ttg	L	65498	13592.00
tgg	W	1	1.00
ctt	L	1	13753.00
ctc	L	0	9641.00
cta	L	1	10096.00
ctg	L	144	11034.00
atg	M	20	20.00
gtt	V	0	1.00
gtc	V	0	0.82
gta	V	0	0.87
gtg	V	4	1.32
---	-	1	1.00
---	--	------	------
mPD		0.0084	1.2
		nPD:	0.01
		N. weight:	1.7
		Sc. PD:	-0.12
		Sc. rank:	-59985.0

,

HA			
Pos.	559	obs:	exp:
ttt	F	37	18.00
ttc	F	0	19.00
tta	L	0	7624.00
ttg	L	0	13584.00
ctt	L	65619	13745.00
ctc	L	82	9635.00
cta	L	0	10089.00
ctg	L	4	11027.00
att	I	25	8.78
atc	I	0	6.57
ata	I	0	9.65
gtt	V	4	1.50
gtc	V	0	1.22
gta	V	2	1.30
gtg	V	0	1.97
---	-	1	1.00
---	--	------	------
mPD		0.0047	1.2
		nPD:	0.
		N. weight:	1.7
		Sc. PD:	-0.13
		Sc. rank:	-76664.0

,

HA			
Pos.	560	obs:	exp:
tta	L	0	0.23
ttg	L	0	0.41
tct	S	2	2.74
tcc	S	0	2.54
tca	S	0	4.57
tcg	S	0	0.67
tat	Y	1	0.53
tac	Y	0	0.47
tgt	C	65585	25693.00
tgc	C	160	40052.00
tgg	W	2	2.00
ctt	L	0	0.42
ctc	L	0	0.29
cta	L	0	0.31
ctg	L	2	0.34
cgt	R	1	0.02
cgc	R	0	0.04
cga	R	0	0.09
cgg	R	0	0.09
agt	S	15	2.97
agc	S	0	3.51
aga	R	0	0.49
agg	R	0	0.28
ggt	G	6	0.81
ggc	G	0	0.84
gga	G	0	2.77
ggg	G	0	1.59
---	--	------	------
mPD		0.0059	0.48
		nPD:	0.01
		N. weight:	1.
		Sc. PD:	-0.069
		Sc. rank:	-24851.0

,

HA			
Pos.	561	obs:	exp:
ttt	F	71	34.54
ttc	F	0	36.46
tta	L	0	2.44
ttg	L	0	4.34
tct	S	0	0.16
tcc	S	0	0.15
tca	S	0	0.27
tcg	S	0	0.04
ctt	L	21	4.39
ctc	L	0	3.08
cta	L	0	3.22
ctg	L	0	3.52
att	I	16111	5657.00
atc	I	3	4234.00
ata	I	1	6224.00
act	T	17	5.28
acc	T	0	3.01
aca	T	0	7.36
acg	T	0	1.36
aat	N	1	0.56
aac	N	0	0.44
agt	S	1	0.17
agc	S	0	0.21
gtt	V	49455	12396.00
gtc	V	31	10084.00
gta	V	5	10727.00
gtg	V	0	16284.00
gct	A	54	14.72
gcc	A	0	13.49
gca	A	0	21.84
gcg	A	0	3.95
ggt	G	3	0.40
ggc	G	0	0.42
gga	G	0	1.38
ggg	G	0	0.79
---	--	------	------
mPD		0.38	1.1
		nPD:	0.33
		N. weight:	1.4
		Sc. PD:	0.34
		Sc. rank:	62992.0

,

HA			
Pos.	562	obs:	exp:
ttt	F	2	0.97
ttc	F	0	1.03
tct	S	5	0.81
tcc	S	0	0.75
tca	S	0	1.34
tcg	S	0	0.20
att	I	3	1.05
atc	I	0	0.79
ata	I	0	1.16
act	T	34	10.56
acc	T	0	6.01
aca	T	0	14.71
acg	T	0	2.72
agt	S	0	0.87
agc	S	0	1.03
gtt	V	5669	1427.00
gtc	V	26	1161.00
gta	V	0	1235.00
gtg	V	1	1874.00
gct	A	59928	16362.00
gcc	A	84	14998.00
gca	A	13	24283.00
gcg	A	6	4388.00
gat	D	1	0.57
gac	D	0	0.43
ggt	G	2	0.27
ggc	G	0	0.28
gga	G	0	0.92
ggg	G	0	0.53
---	--	------	------
mPD		0.16	0.87
		nPD:	0.19
		N. weight:	1.4
		Sc. PD:	0.14
		Sc. rank:	54570.0

,

HA			
Pos.	563	obs:	exp:
ttt	F	16	7.78
ttc	F	0	8.22
tta	L	108	7625.00
ttg	L	59619	13585.00
tct	S	0	4.36
tcc	S	2	4.03
tca	S	0	7.26
tcg	S	25	1.06
tgg	W	1	1.00
ctt	L	7	13746.00
ctc	L	0	9636.00
cta	L	6	10090.00
ctg	L	5971	11028.00
atg	M	16	16.00
agt	S	0	4.72
agc	S	0	5.57
gtt	V	0	0.75
gtc	V	0	0.61
gta	V	0	0.65
gtg	V	3	0.99
---	--	------	------
mPD		0.17	1.2
		nPD:	0.15
		N. weight:	1.4
		Sc. PD:	0.086
		Sc. rank:	49403.0

,

HA			
Pos.	564	obs:	exp:
ttt	F	1	0.49
ttc	F	0	0.51
tta	L	12	7629.00
ttg	L	65072	13592.00
tct	S	0	0.16
tcc	S	0	0.15
tca	S	0	0.27
tcg	S	1	0.04
tgg	W	4	4.00
ctt	L	6	13753.00
ctc	L	0	9641.00
cta	L	4	10095.00
ctg	L	649	11034.00
atg	M	1	1.00
agt	S	0	0.17
agc	S	0	0.21
gtt	V	0	6.01
gtc	V	0	4.89
gta	V	0	5.20
gtg	V	24	7.90
---	--	------	------
mPD		0.021	1.2
		nPD:	0.02
		N. weight:	1.7
		Sc. PD:	-0.1
		Sc. rank:	-18487.0

,

HA			
Pos.	565	obs:	exp:
gtt	V	0	3.01
gtc	V	0	2.45
gta	V	0	2.60
gtg	V	12	3.95
ggt	G	27	8853.00
ggc	G	0	9178.00
gga	G	392	30356.00
ggg	G	65342	17373.00
---	-	1	1.00
---	--	------	------
mPD		0.013	0.68
		nPD:	0.02
		N. weight:	1.4
		Sc. PD:	-0.087
		Sc. rank:	-12965.0

,

HA			
Pos.	566	obs:	exp:
ttt	F	137	31961.00
ttc	F	65569	33745.00
tta	L	11	2.67
ttg	L	0	4.76
tct	S	2	4.84
tcc	S	28	4.48
tca	S	0	8.06
tcg	S	0	1.18
tat	Y	0	1.05
tac	Y	2	0.95
ctt	L	0	4.81
ctc	L	12	3.37
cta	L	0	3.53
ctg	L	0	3.86
att	I	0	0.70
atc	I	2	0.53
ata	I	0	0.77
agt	S	0	5.24
agc	S	0	6.19
gtt	V	1	2.00
gtc	V	7	1.63
gta	V	0	1.73
gtg	V	0	2.63
gct	A	0	0.55
gcc	A	0	0.50
gca	A	2	0.81
gcg	A	0	0.15
---	-	1	1.00
---	--	------	------
mPD		0.0064	0.50
		nPD:	0.01
		N. weight:	0.72
		Sc. PD:	-0.048
		Sc. rank:	-16318.0

,

HA

Pos.	567	obs:	exp:
ttt	F	0	1.46
ttc	F	3	1.54
tta	L	0	1.28
ttg	L	0	2.27
ctt	L	0	2.30
ctc	L	11	1.61
cta	L	0	1.69
ctg	L	0	1.85
cat	H	1	1.13
cac	H	1	0.87
att	I	191	23063.00
atc	I	65417	17263.00
ata	I	91	25373.00
act	T	1	10.87
acc	T	34	6.19
aca	T	0	15.15
acg	T	0	2.80
aat	N	0	0.56
aac	N	1	0.44
gtt	V	0	5.51
gtc	V	22	4.48
gta	V	0	4.77
gtg	V	0	7.24
---	-	1	1.00
---	--	------	------
mPD		0.011	0.66
		nPD:	0.02
		N. weight:	1.4
		Sc. PD:	-0.092
		Sc. rank:	-20997.0

,

HA

Pos.	568	obs:	exp:
tct	S	0	0.32
tcc	S	0	0.30
tca	S	0	0.54
tcg	S	0	0.08
agt	S	1	0.35
agc	S	1	0.41
---	-	65772	65772.00
---	--	------	------
mPD		4.6×10^{-10}	1.5×10^{-9}
		nPD:	0.31
		N. weight:	0.
		Sc. PD:	0
		Sc. rank:	0

,

HA

Pos.	569	obs:	exp:
ttt	F	0	0.97
ttc	F	2	1.03
tta	L	0	0.46
ttg	L	2	0.83
tgt	C	1	0.39
tgc	C	0	0.61
ctt	L	0	0.84
ctc	L	0	0.59
cta	L	0	0.61
ctg	L	2	0.67
cat	H	1	0.56
cac	H	0	0.44
att	I	1	13.34
atc	I	0	9.98
ata	I	37	14.68
atg	M	65712	65712.00
act	T	0	1.55
acc	T	0	0.88
aca	T	0	2.16
acg	T	5	0.40
gtt	V	0	2.76
gtc	V	0	2.24
gta	V	0	2.38
gtg	V	11	3.62
---	--	------	------
mPD		0.0021	0.0025
		nPD:	0.82
		N. weight:	0.0011
		Sc. PD:	0.00077
		Sc. rank:	56.3

,

HA

Pos.	570	obs:	exp:
tta	L	0	0.35
ttg	L	3	0.62
tgt	C	2	0.78
tgc	C	0	1.22
tgg	W	65762	65762.00
ctt	L	0	0.63
ctc	L	0	0.44
cta	L	0	0.46
ctg	L	0	0.50
cgt	R	0	0.02
cgc	R	0	0.04
cga	R	0	0.09
cgg	R	1	0.09
aga	R	0	0.49
agg	R	0	0.28
gtt	V	0	0.25
gtc	V	0	0.20
gta	V	0	0.22
gtg	V	1	0.33
ggt	G	0	0.67
ggc	G	0	0.70
gga	G	1	2.31
ggg	G	4	1.32
---	--	------	------
mPD		0.00043	0.00067
		nPD:	0.64
		N. weight:	0.00025
		Sc. PD:	0.00013
		Sc. rank:	12.4

,

HA

Pos.	571	obs:	exp:
ggt	G	1	0.13
ggc	G	0	0.14
gga	G	0	0.46
ggg	G	0	0.26
---	-	65773	65773.00
---	--	------	------
mPD		0	1.6×10^{-10}
		nPD:	0.
		N. weight:	0.
		Sc. PD:	0
		Sc. rank:	0

,

HA

Pos.	572	obs:	exp:
tct	S	0	0.16
tcc	S	0	0.15
tca	S	1	0.27
tcg	S	0	0.04
agt	S	0	0.17
agc	S	0	0.21
---	-	65773	65773.00
---	--	------	------
mPD		0	3.8×10^{-10}
		nPD:	0.
		N. weight:	0.
		Sc. PD:	0
		Sc. rank:	0

,

HA

Pos.	573	obs:	exp:
tct	S	0	2.90
tcc	S	17	2.69
tca	S	1	4.84
tcg	S	0	0.71
cct	P	0	0.33
ccc	P	1	0.19
cca	P	0	0.34
ccg	P	0	0.14
atg	M	2	2.00
act	T	0	7.45
acc	T	24	4.24
aca	T	0	10.39
acg	T	0	1.92
agt	S	0	3.15
agc	S	0	3.71
gtt	V	0	0.75
gtc	V	3	0.61
gta	V	0	0.65
gtg	V	0	0.99
gct	A	150	17913.00
gcc	A	65438	16421.00
gca	A	136	26586.00
gcg	A	0	4805.00
gat	D	0	0.57
gac	D	1	0.43
ggt	G	0	0.13
ggc	G	1	0.14
gga	G	0	0.46
ggg	G	0	0.26
---	--	------	------
mPD		0.010	0.70
		nPD:	0.01
		N. weight:	1.5
		Sc. PD:	-0.098
		Sc. rank:	-25819.0

,

HA

Pos.	574	obs:	exp:
tta	L	0	0.12
ttg	L	0	0.21
tct	S	0	0.32
tcc	S	0	0.30
tca	S	0	0.54
tcg	S	0	0.08
tat	Y	0	1.05
tac	Y	2	0.95
tgt	C	12	25701.00
tgc	C	65755	40066.00
tgg	W	1	1.00
ctt	L	0	0.21
ctc	L	0	0.15
cta	L	0	0.15
ctg	L	1	0.17
cgt	R	0	0.02
cgc	R	1	0.04
cga	R	0	0.09
cgg	R	0	0.09
agt	S	0	0.35
agc	S	2	0.41
aga	R	0	0.49
agg	R	0	0.28
---	--	------	------
mPD		0.00064	0.48
		nPD:	0.
		N. weight:	0.55
		Sc. PD:	-0.043
		Sc. rank:	-27876.0

,

HA			
Pos.	575	obs:	exp:
ggt	G	0	0.13
ggc	G	0	0.14
gga	G	0	0.46
ggg	G	1	0.26
---	-	65773	65773.00
---	--	------	------
mPD		0	1.6×10^{-10}
		nPD:	0.
		N. weight:	0.
		Sc. PD:	0
		Sc. rank:	0

,

HA			
Pos.	576	obs:	exp:
cct	P	1	0.33
ccc	P	0	0.19
cca	P	0	0.34
ccg	P	0	0.14
---	-	65773	65773.00
---	--	------	------
mPD		0	1.7×10^{-10}
		nPD:	0.
		N. weight:	0.
		Sc. PD:	0
		Sc. rank:	0

,

HA			
Pos.	577	obs:	exp:
tta	L	1	0.81
ttg	L	0	1.45
tct	S	2	0.32
tcc	S	0	0.30
tca	S	0	0.54
tcg	S	0	0.08
ctt	L	1	1.46
ctc	L	0	1.03
cta	L	5	1.07
ctg	L	0	1.17
cct	P	0	1.67
ccc	P	0	0.94
cca	P	5	1.69
ccg	P	0	0.71
cat	H	3	2.26
cac	H	1	1.74
caa	Q	65560	40190.00
cag	Q	187	25557.00
cgt	R	0	0.02
cgc	R	0	0.04
cga	R	1	0.09
cgg	R	0	0.09
aaa	K	7	4.60
aag	K	0	2.40
agt	S	0	0.35
agc	S	0	0.41
aga	R	0	0.49
agg	R	0	0.28
gct	A	0	0.27
gcc	A	1	0.25
gca	A	0	0.40
gcg	A	0	0.07
---	--	------	------
mPD		0.0067	0.48
		nPD:	0.01
		N. weight:	0.52
		Sc. PD:	-0.034
		Sc. rank:	-10245.0

,

HA			
Pos.	578	obs:	exp:
tta	L	2	0.23
ttg	L	0	0.41
ctt	L	0	0.42
ctc	L	0	0.29
cta	L	0	0.31
ctg	L	0	0.34
caa	Q	0	1.83
cag	Q	3	1.17
cgt	R	0	1.57
cgc	R	0	2.84
cga	R	0	7.21
cgg	R	0	6.78
atg	M	2	2.00
act	T	0	0.62
acc	T	0	0.35
aca	T	0	0.87
acg	T	2	0.16
aat	N	47	26.85
aac	N	1	21.15
aaa	K	6098	43145.00
aag	K	59528	22481.00
aga	R	32	38.75
agg	R	47	21.85
gaa	E	0	7.07
gag	E	12	4.93
---	--	------	------
mPD		0.17	0.46
		nPD:	0.38
		N. weight:	0.77
		Sc. PD:	0.22
		Sc. rank:	34846.0

,

HA			
Pos.	579	obs:	exp:
tct	S	0	0.65
tcc	S	0	0.60
tca	S	0	1.08
tcg	S	0	0.16
tgt	C	0	0.39
tgc	C	1	0.61
cat	H	0	0.56
cac	H	1	0.44
agt	S	0	0.70
agc	S	4	0.83
gtt	V	1	1.00
gtc	V	2	0.82
gta	V	1	0.87
gtg	V	0	1.32
gct	A	0	0.55
gcc	A	2	0.50
gca	A	0	0.81
gcg	A	0	0.15
gat	D	0	1.13
gac	D	2	0.87
ggt	G	6	8853.00
ggc	G	65722	9178.00
gga	G	28	30356.00
ggg	G	4	17373.00
---	--	------	------
mPD		0.0017	0.68
		nPD:	0.
		N. weight:	2.2
		Sc. PD:	-0.17
		Sc. rank:	-104630.0

,

HA			
Pos.	580	obs:	exp:
tct	S	2	1.94
tcc	S	0	1.79
tca	S	0	3.23
tcg	S	0	0.47
cat	H	0	4.51
cac	H	8	3.49
caa	Q	1	0.61
cag	Q	0	0.39
aat	N	34	36785.00
aac	N	65715	28964.00
aaa	K	1	0.66
aag	K	0	0.34
agt	S	0	2.10
agc	S	10	2.48
gat	D	0	1.70
gac	D	3	1.30
---	--	------	------
mPD		0.0019	0.49
		nPD:	0.
		N. weight:	0.9
		Sc. PD:	-0.068
		Sc. rank:	-40653.0

,

HA			
Pos.	581	obs:	exp:
tta	L	0	0.70
ttg	L	0	1.24
ctt	L	4	1.26
ctc	L	0	0.88
cta	L	2	0.92
ctg	L	0	1.01
cct	P	1	0.67
ccc	P	1	0.37
cca	P	0	0.68
ccg	P	0	0.28
att	I	65252	23082.00
atc	I	496	17278.00
ata	I	6	25394.00
act	T	0	0.93
acc	T	1	0.53
aca	T	2	1.30
acg	T	0	0.24
gtt	V	9	2.25
gtc	V	0	1.83
gta	V	0	1.95
gtg	V	0	2.96
---	--	------	------
mPD		0.016	0.66
		nPD:	0.02
		N. weight:	1.1
		Sc. PD:	-0.062
		Sc. rank:	-2149.5

,

HA			
Pos.	582	obs:	exp:
caa	Q	0	1.22
cag	Q	2	0.78
cgt	R	0	1301.00
cgc	R	0	2361.00
cga	R	4	5988.00
cgg	R	2	5635.00
atg	M	4	4.00
act	T	0	0.62
acc	T	0	0.35
aca	T	1	0.87
acg	T	1	0.16
aaa	K	41	89.41
aag	K	95	46.59
aga	R	37498	32189.00
agg	R	28123	18152.00
ggt	G	0	0.40
ggc	G	0	0.42
gga	G	2	1.38
ggg	G	1	0.79
---	--	------	------
mPD		0.49	0.89
		nPD:	0.56
		N. weight:	0.3
		Sc. PD:	0.14
		Sc. rank:	15087.0

,

HA			
Pos.	583	obs:	exp:
ttt	F	0	1.46
ttc	F	3	1.54
tct	S	0	0.16
tcc	S	0	0.15
tca	S	0	0.27
tcg	S	0	0.04
tgt	C	22	25702.00
tgc	C	65746	40066.00
cgt	R	0	0.04
cgc	R	2	0.07
cga	R	0	0.18
cgg	R	0	0.17
agt	S	0	0.17
agc	S	1	0.21
aga	R	0	0.98
agg	R	0	0.55
---	--	------	------
mPD		0.00085	0.48
		nPD:	0.
		N. weight:	0.54
		Sc. PD:	-0.042
		Sc. rank:	-27233.0

HA			
Pos.	584	obs:	exp:
tct	S	0	9.52
tcc	S	0	8.82
tca	S	0	15.86
tcg	S	0	2.32
cat	H	0	0.56
cac	H	1	0.44
cgt	R	0	0.04
cgc	R	0	0.07
cga	R	0	0.18
cgg	R	0	0.17
att	I	0	0.35
atc	I	1	0.26
ata	I	0	0.39
act	T	2	3.11
acc	T	1	1.77
aca	T	7	4.33
acg	T	0	0.80
aat	N	5649	36705.00
aac	N	59958	28902.00
aaa	K	1	0.66
aag	K	0	0.34
agt	S	1	10.31
agc	S	58	12.17
aga	R	2	0.98
agg	R	0	0.55
gat	D	2	51.44
gac	D	89	39.56
---	-	2	2.00
---	--	------	------
mPD		0.16	0.50
		nPD:	0.33
		N. weight:	0.56
		Sc. PD:	0.13
		Sc. rank:	24297.0

HA			
Pos.	585	obs:	exp:
ttt	F	10	4.86
ttc	F	0	5.14
tta	L	0	0.12
ttg	L	0	0.21
ctt	L	1	0.21
ctc	L	0	0.15
cta	L	0	0.15
ctg	L	0	0.17
cat	H	2	1.13
cac	H	0	0.87
att	I	63868	23080.00
atc	I	1870	17276.00
ata	I	9	25391.00
act	T	1	0.31
acc	T	0	0.18
aca	T	0	0.43
acg	T	0	0.08
aat	N	1	0.56
aac	N	0	0.44
aaa	K	1	1.31
aag	K	1	0.69
gtt	V	9	2.50
gtc	V	1	2.04
gta	V	0	2.17
gtg	V	0	3.29
---	--	------	------
mPD		0.056	0.66
		nPD:	0.09
		N. weight:	1.
		Sc. PD:	0.0023
		Sc. rank:	25887.0

HA			
Pos.	586	obs:	exp:
tgt	C	0	1.17
tgc	C	3	1.83
gct	A	0	0.27
gcc	A	0	0.25
gca	A	1	0.40
gcg	A	0	0.07
---	-	65770	65770.00
---	--	------	------
mPD		4.2×10^{-9}	5.0×10^{-9}
		nPD:	0.84
		N. weight:	0.
		Sc. PD:	0
		Sc. rank:	0

HA			
Pos.	587	obs:	exp:
ttt	F	1	0.49
ttc	F	0	0.51
aaa	K	3	1.97
aag	K	0	1.03
---	-	65770	65770.00
---	--	------	------
mPD		4.2×10^{-9}	5.2×10^{-9}
		nPD:	0.8
		N. weight:	0.
		Sc. PD:	0
		Sc. rank:	0

HA			
Pos.	588	obs:	exp:
tgt	C	3	1.17
tgc	C	0	1.83
gaa	E	0	0.59
gag	E	1	0.41
---	-	65770	65770.00
---	--	------	------
mPD		4.2×10^{-9}	5.3×10^{-9}
		nPD:	0.79
		N. weight:	0.
		Sc. PD:	0
		Sc. rank:	0

HA			
Pos.	589	obs:	exp:
tta	L	1	0.12
ttg	L	0	0.21
tct	S	0	0.65
tcc	S	1	0.60
tca	S	1	1.08
tcg	S	0	0.16
tgt	C	319	25700.00
tgc	C	65446	40065.00
ctt	L	0	0.21
ctc	L	0	0.15
cta	L	0	0.15
ctg	L	0	0.17
agt	S	0	0.70
agc	S	2	0.83
ggt	G	1	0.40
ggc	G	2	0.42
gga	G	0	1.38
ggg	G	0	0.79
---	-	1	1.00
---	--	------	------
mPD		0.010	0.48
		nPD:	0.02
		N. weight:	0.52
		Sc. PD:	-0.031
		Sc. rank:	-3267.3

HA			
Pos.	590	obs:	exp:
ttt	F	1	0.49
ttc	F	0	0.51
tta	L	0	0.23
ttg	L	1	0.41
tat	Y	3	1.58
tac	Y	0	1.42
ctt	L	1	0.42
ctc	L	0	0.29
cta	L	0	0.31
ctg	L	0	0.34
cgt	R	0	0.06
cgc	R	0	0.11
cga	R	0	0.27
cgg	R	0	0.26
att	I	65646	23075.00
atc	I	80	17272.00
ata	I	7	25386.00
atg	M	1	1.00
aat	N	3	1.68
aac	N	0	1.32
aaa	K	16	10.52
aag	K	0	5.48
aga	R	3	1.47
agg	R	0	0.83
gtt	V	11	2.76
gtc	V	0	2.24
gta	V	0	2.38
gtg	V	0	3.62
---	-	1	1.00
---	--	------	------
mPD		0.0046	0.66
		nPD:	0.01
		N. weight:	1.1
		Sc. PD:	-0.083
		Sc. rank:	-40943.0

HA			
Pos.	591	obs:	exp:
ttt	F	0	0.49
ttc	F	1	0.51
tgt	C	21	8.21
tgc	C	0	12.79
tgg	W	1	1.00
cgt	R	0	0.02
cgc	R	0	0.04
cga	R	0	0.09
cgg	R	1	0.09
aga	R	0	0.49
agg	R	0	0.28
---	-	65750	65750.00
---	--	------	------
mPD		5.1×10^{-8}	9.5×10^{-8}
		nPD:	0.54
		N. weight:	0.
		Sc. PD:	0
		Sc. rank:	0

HA			
Pos.	592	obs:	exp:
ttt	F	0	0.49
ttc	F	1	0.51
tgt	C	0	8.21
tgc	C	21	12.79
gtt	V	1	0.50
gtc	V	0	0.41
gta	V	0	0.43
gtg	V	1	0.66
---	-	65750	65750.00
---	--	------	------
mPD		7.0×10^{-8}	1.2×10^{-7}
		nPD:	0.59
		N. weight:	0.
		Sc. PD:	0
		Sc. rank:	0

HA			
Pos.	593	obs:	exp:
tat	Y	2	1.05
tac	Y	0	0.95
att	I	1	6.32
atc	I	17	4.73
ata	I	0	6.95
act	T	0	1.24
acc	T	4	0.71
aca	T	0	1.73
acg	T	0	0.32
aat	N	2	1.12
aac	N	0	0.88
---	-	65748	65748.00
---	--	------	------
mPD		1.4×10^{-7}	2.0×10^{-7}
		nPD:	0.72
		N. weight:	0.
		Sc. PD:	0
		Sc. rank:	0

HA			
Pos.	594	obs:	exp:
taa	.	260	37364.00
tag	.	0	7205.00
tga	.	65514	21205.00
---	--	------	------
mPD		0.0079	0.63
		nPD:	0.01
		N. weight:	1.2
		Sc. PD:	-0.082
		Sc. rank:	-28671.0

Amino acid level conservation per codon

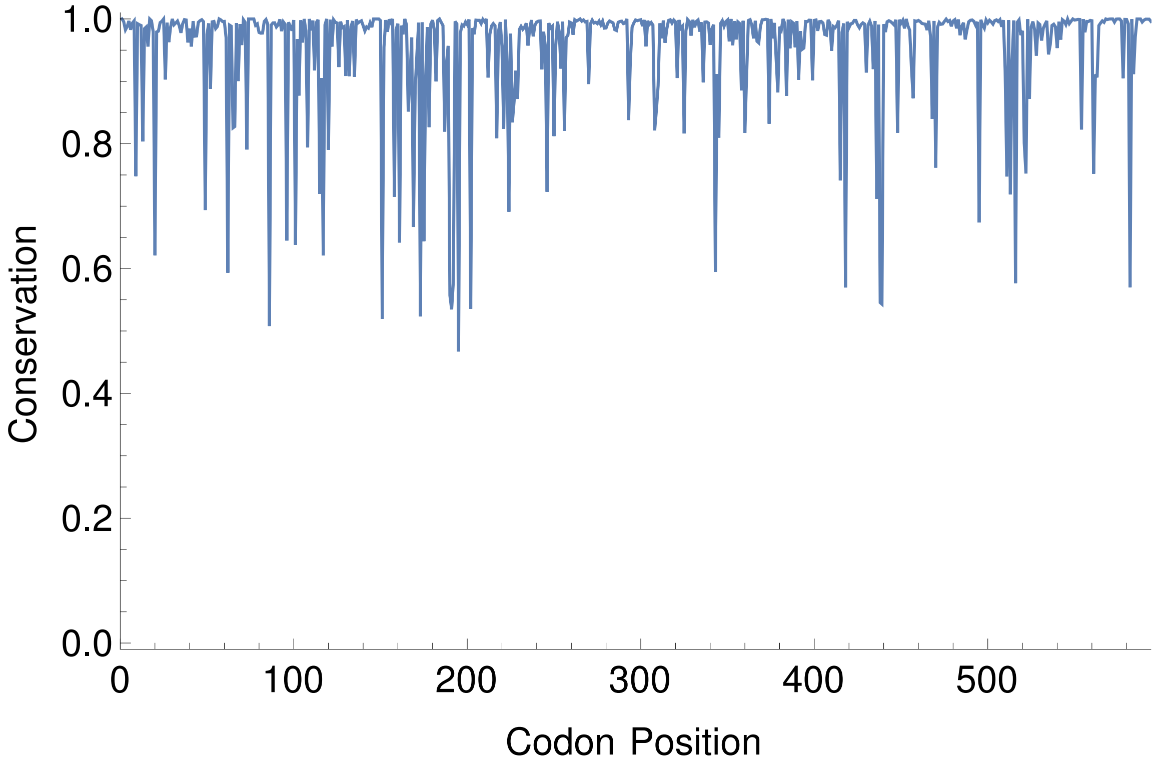

Conservation analysis plots

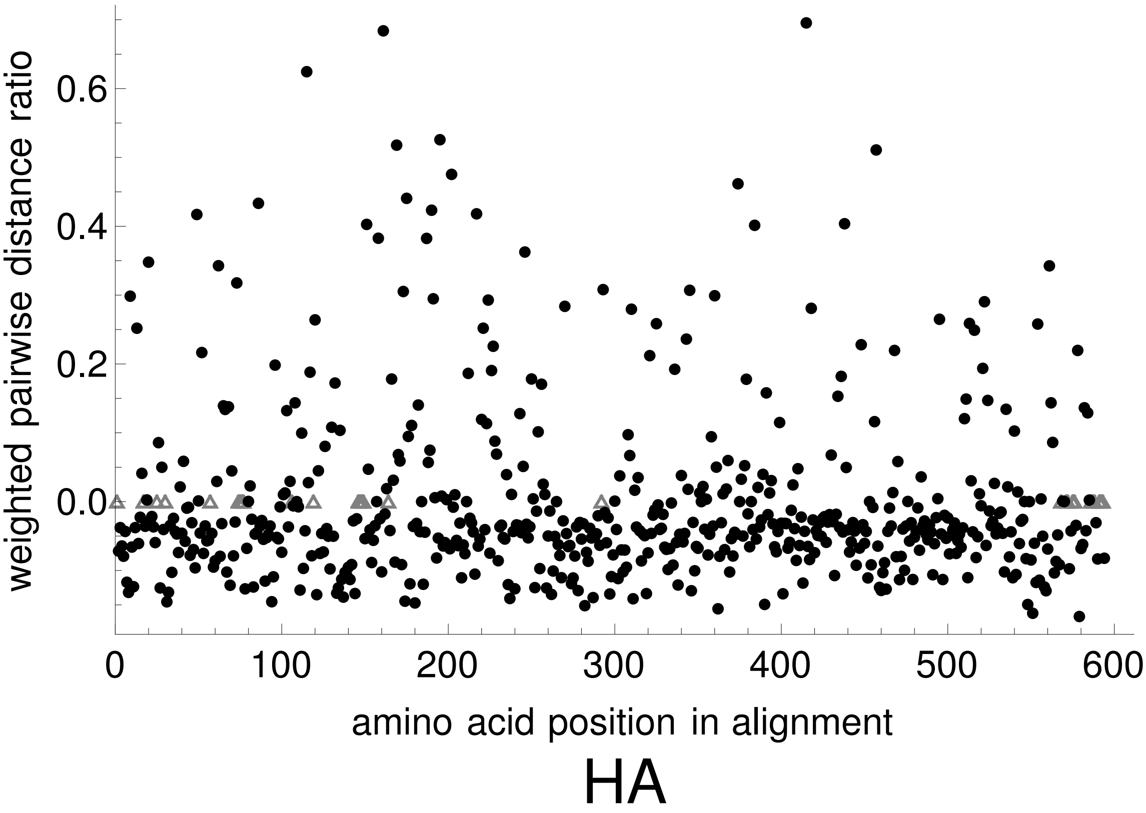

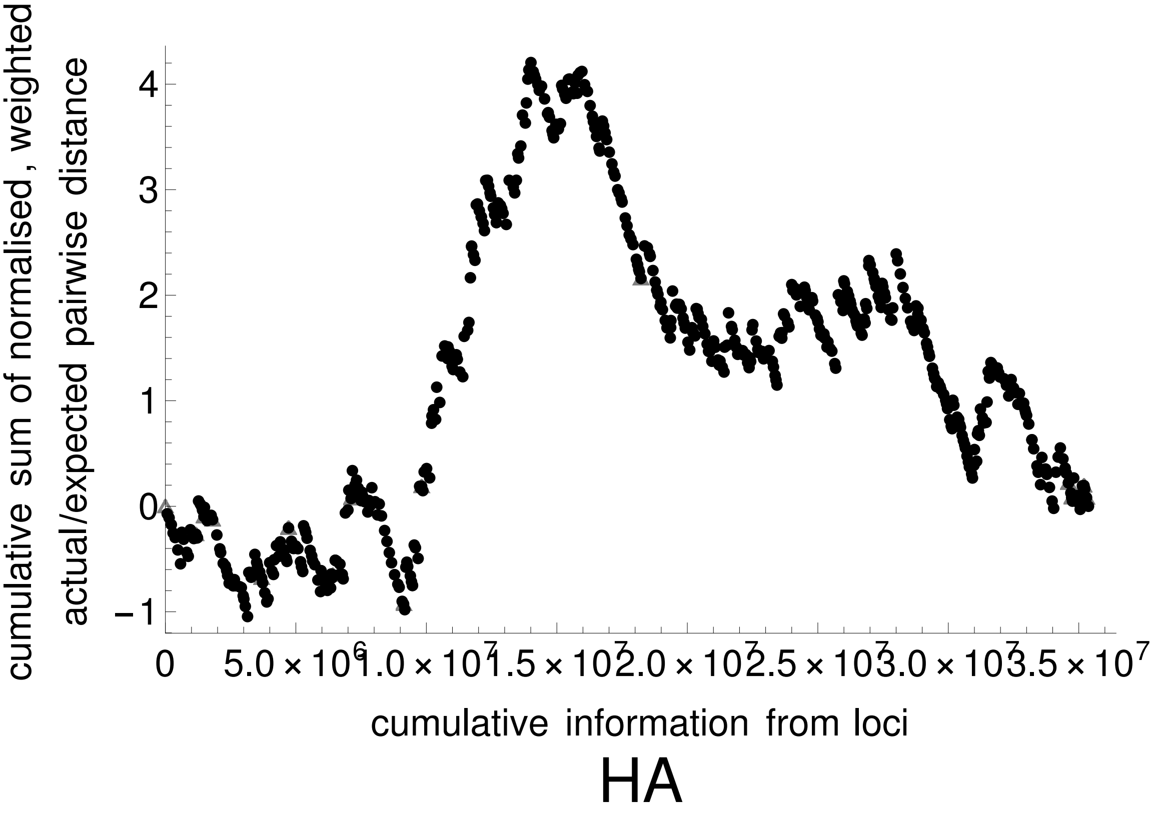

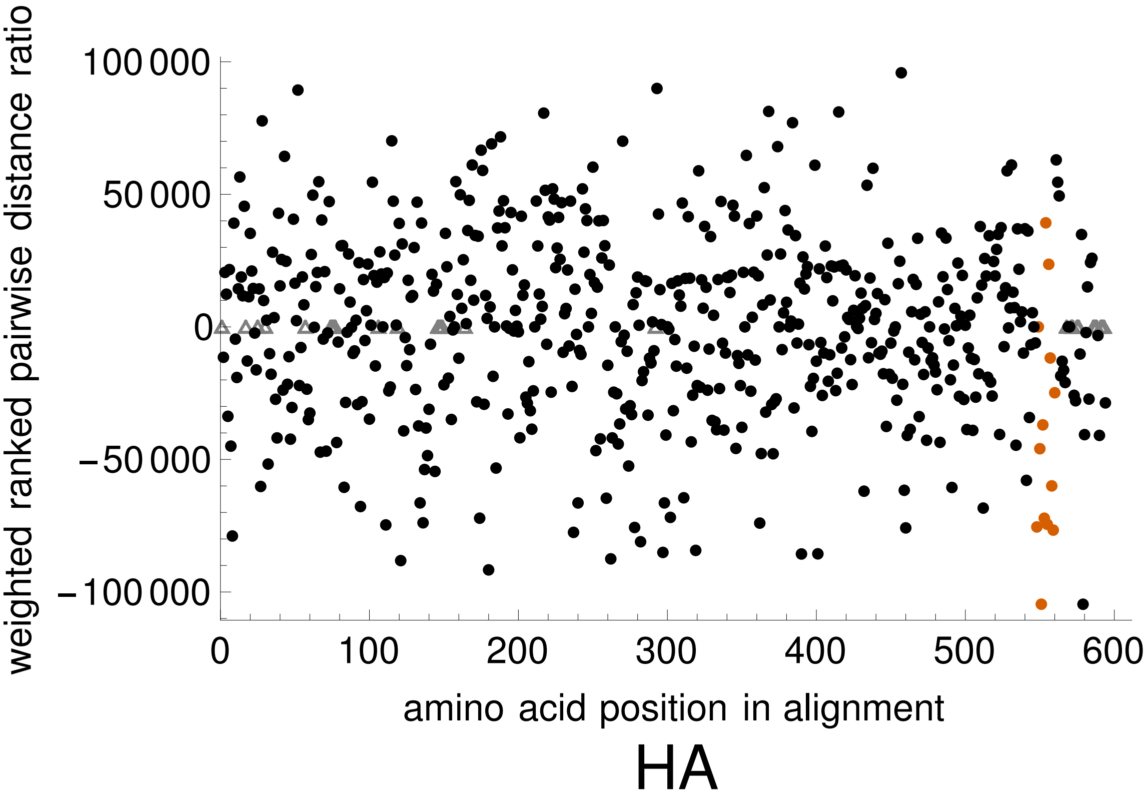
weighted ranked pairwise distance ratio
100 000
50 000
0
−50 000
−100 000
0
100
200
300
400
500
600
amino acid position in alignment
HA

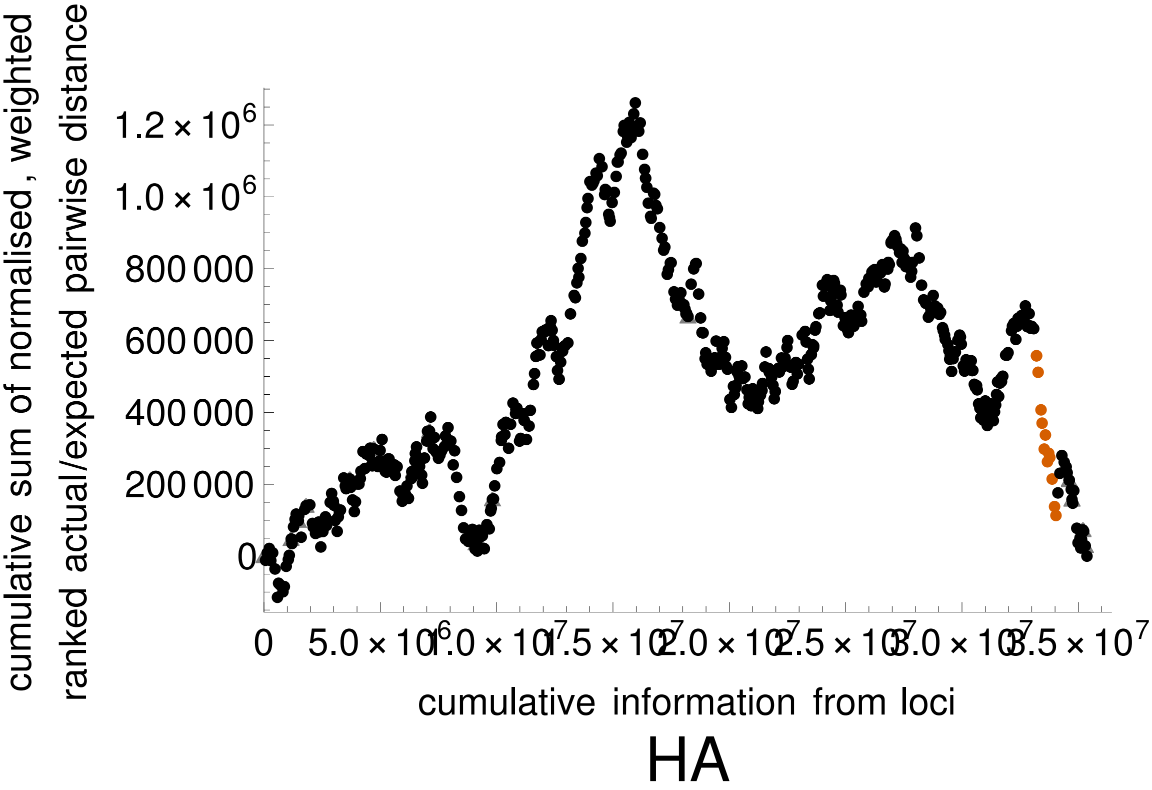
cumulative sum of normalised, weighted ranked actual/expected pairwise distance
1.2 × 10^6
1.0 × 10^6
800 000
600 000
400 000
200 000
0
0
5.0 × 10^6
1.0 × 10^7
1.5 × 10^7
2.0 × 10^7
2.5 × 10^7
3.0 × 10^7
3.5 × 10^7
cumulative information from loci
HA

NP

Gene length histogram

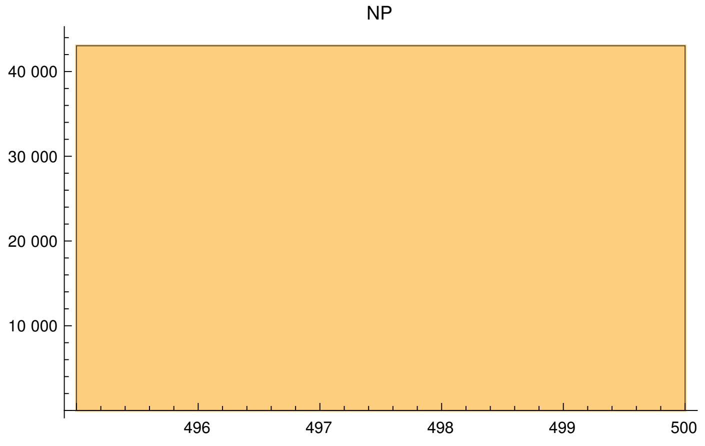

Information vs. nPD

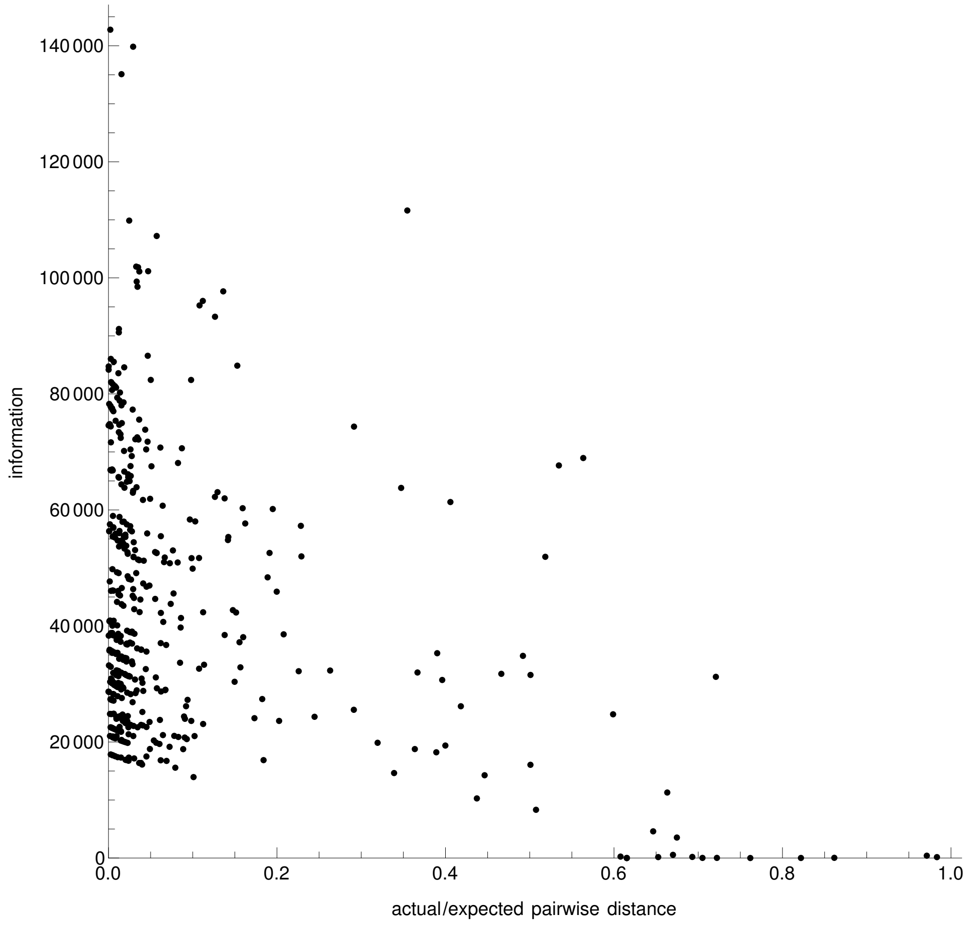

NP

Example sequences highlighted by regions found in analyses to be conserved

```
Interesting points (by weighted raw PD) highlighted for gene NP:
---ATGGCGTCCCAAGGCACCAAACGGTCTTATGAACAGATGGAAACTGATGGAGATCGCCAGAATGCAACTGAGA⋱
    TTAGGGCATCCGTCGGGAAGATGATTGATGGAATTGGGAGATTCTACATCCAAATGTGCACTGAACTTAAACTCA⋱
    GTGATCATGAAGGACGGTTGATCCAAAACAGCTTGACAATAGAGAAAATGGTACTCTCTGCTTTTGATGAAAGAA⋱
    GGAATAAATACCTGGAAGAACACCCCAGCGCGGGGAAAGATCCCAAGAAAACTGGGGGGCCCATATACAGGAGAG⋱
    TCGATGGGAAATGGATGAGGGAACTCGTCCTTTATGACAAAGAAGAAATAAGGCGAATCTGGCGCCAAGCCAACA⋱
    ATGGTGAGGATGCTACATCTGGTCTAACCCACATAATGATTTGGCATTCCAATTTGAATGATGCAACATACCAGA⋱
    GGACAAGAGCTCTTGTTCGGACTGGAATGGATCCCAGAATGTGCTCTCTGATGCAGGGCTCGACTCTCCCTAGAA⋱
    GGTCCGGAGCTGCAGGTGCTGCAGTCAAAGGAATCGGAACAATGGTGATGGAACTGATCAGAATGATCAAACGGG⋱
    GGATCAACGATCGAAATTTTTGGAGGGGTGAGAATGGGCGGAAAACAAGAAGTGCTTATGAGAGAATGTGCAACA⋱
    TTCTTAAAGGAAAATTTCAAACAGCTGCACAAAGAGCAATGGTGGATCAAGTTAGAGAAAGTCGGAACCCAGGAA⋱
    ATGCTGAGATCGAAGATCTCATATTTTTAGCAAGATCTGCACTGATATTGAGAGGATCAGTTGCTCACAAATCTT⋱
    GCCTACCTGCCTGTGCATATGGACCTGCAGTATCCAGTGGTTACGATTTTGAAAAAGAGGGATATTCCTTGGTGG⋱
    GAATAGACCCTTTCAAACTACTTCAAAATAGCCAAATATACAGCTTAATCAGACCTAATGAGAATCCAGCACACA⋱
    AGAGTCAGCTGGTATGGATGGCATGCCATTCTGCTGCATTTGAAGATTTAAGATTGTTAAGCTTCATCAGAGGGA⋱
    CAAAAGTATCTCCTCGGGGGAAACTGTCAACTAGAGGAATACAAATTGCTTCAAATGAGAACATGGATAATATGG⋱
    GATCAAGCACTCTTGAACTGAGAAGCGGGTACTGGGCCATAAGGACCAGGAGTGGAGGAAACACTAATCAACAGA⋱
    GGGCCTCCGCAGGCCAAACCAGTGTGCAACCTACGTTTTCTGTACAAAGAAACATCCCATTTGAAAAGTCAACCA⋱
    TCATGGCAGCATTCACTGGAAATACGGAGGGAAGAACTTCAGACATGAGGGCAGAAATCATAAGGATGATGGAAG⋱
    GTGCAAAACCAGAAGAAGTGTCATTCCGGGGGAGGGGAGTTTTCGAGCTCTCAGACGAGAAGGCAGCGAACCCGA⋱
    TCGTGCCCTCTTTTGATATGAGTAACGAAGGATCTTATTTCTTCGGAGACAATGCAGAAGAGTACGACAATTAA

Interesting points (by weighted ranked PD) highlighted for gene NP:
---ATGGCGTCCCAAGGCACCAAACGGTCTTATGAACAGATGGAAACTGATGGAGATCGCCAGAATGCAACTGAGATT⋱
    AGGGCATCCGTCGGGAAGATGATTGATGGAATTGGGAGATTCTACATCCAAATGTGCACTGAACTTAAACTCAGT⋱
    GATCATGAAGGACGGTTGATCCAAAACAGCTTGACAATAGAGAAAATGGTACTCTCTGCTTTTGATGAAAGAAGG⋱
    AATAAATACCTGGAAGAACACCCCAGCGCGGGGAAAGATCCCAAGAAAACTGGGGGGCCCATATACAGGAGAGTC⋱
    GATGGGAAATGGATGAGGGAACTCGTCCTTTATGACAAAGAAGAAATAAGGCGAATCTGGCGCCAAGCCAACAAT⋱
    GGTGAGGATGCTACATCTGGTCTAACCCACATAATGATTTGGCATTCCAATTTGAATGATGCAACATACCAGAGG⋱
    ACAAGAGCTCTTGTTCGGACTGGAATGGATCCCAGAATGTGCTCTCTGATGCAGGGCTCGACTCTCCCTAGAAGG⋱
    TCCGGAGCTGCAGGTGCTGCAGTCAAAGGAATCGGAACAATGGTGATGGAACTGATCAGAATGATCAAACGGGGG⋱
    ATCAACGATCGAAATTTTTGGAGGGGTGAGAATGGGCGGAAAACAAGAAGTGCTTATGAGAGAATGTGCAACATT⋱
    CTTAAAGGAAAATTTCAAACAGCTGCACAAAGAGCAATGGTGGATCAAGTTAGAGAAAGTCGGAACCCAGGAAAT⋱
    GCTGAGATCGAAGATCTCATATTTTTAGCAAGATCTGCACTGATATTGAGAGGATCAGTTGCTCACAAATCTTGC⋱
    CTACCTGCCTGTGCATATGGACCTGCAGTATCCAGTGGTTACGATTTTGAAAAAGAGGGATATTCCTTGGTGGGA⋱
    ATAGACCCTTTCAAACTACTTCAAAATAGCCAAATATACAGCTTAATCAGACCTAATGAGAATCCAGCACACAAG⋱
    AGTCAGCTGGTATGGATGGCATGCCATTCTGCTGCATTTGAAGATTTAAGATTGTTAAGCTTCATCAGAGGGACA⋱
    AAAGTATCTCCTCGGGGGAAACTGTCAACTAGAGGAATACAAATTGCTTCAAATGAGAACATGGATAATATGGGA⋱
    TCAAGCACTCTTGAACTGAGAAGCGGGTACTGGGCCATAAGGACCAGGAGTGGAGGAAACACTAATCAACAGAGG⋱
    GCCTCCGCAGGCCAAACCAGTGTGCAACCTACGTTTTCTGTACAAAGAAACATCCCATTTGAAAAGTCAACCATC⋱
    ATGGCAGCATTCACTGGAAATACGGAGGGAAGAACTTCAGACATGAGGGCAGAAATCATAAGGATGATGGAAGGT⋱
    GCAAAACCAGAAGAAGTGTCATTCCGGGGGAGGGGAGTTTTCGAGCTCTCAGACGAGAAGGCAGCG
```

AACCCGATCGTGCCCTCTTTTGATATGAGTAACGAAGGATCTTATTTCTTCGGAG
ACAATGCAGAAGAGTACGACAATTAA

Per codon conservation report

NP			
Pos.	1	obs:	exp:
atg	M	1	1.00
---	-	43039	43039.00
---	--	------	------
mPD		0	0
		nPD:	1.
		N. weight:	0.
		Sc. PD:	0
		Sc. rank:	0

NP			
Pos.	2	obs:	exp:
atg	M	43040	43040.00
---	--	------	------
mPD		0	0
		nPD:	1.
		N. weight:	0.
		Sc. PD:	0
		Sc. rank:	0

NP			
Pos.	3	obs:	exp:
gtt	V	0	4.76
gtc	V	0	3.87
gta	V	0	4.12
gtg	V	19	6.25
gct	A	13	11726.00
gcc	A	0	10748.00
gca	A	339	17402.00
gcg	A	42669	3145.00
---	--	------	------
mPD		0.017	0.70
		nPD:	0.02
		N. weight:	2.6
		Sc. PD:	-0.078
		Sc. rank:	9075.5

NP			
Pos.	4	obs:	exp:
ttt	F	0	4.86
ttc	F	10	5.14
tct	S	595	6940.00
tcc	S	42403	6427.00
tca	S	8	11559.00
tcg	S	0	1693.00
cct	P	0	1.00
ccc	P	3	0.56
cca	P	0	1.01
ccg	P	0	0.42
act	T	0	6.52
acc	T	21	3.71
aca	T	0	9.09
acg	T	0	1.68
agt	S	0	7515.00
agc	S	0	8872.00
---	--	------	------
mPD		0.029	1.6
		nPD:	0.02
		N. weight:	1.9
		Sc. PD:	-0.068
		Sc. rank:	-7166.3

NP			
Pos.	5	obs:	exp:
cat	H	65	41.74
cac	H	9	32.26
caa	Q	42941	26260.00
cag	Q	19	16700.00
aaa	K	6	3.94
aag	K	0	2.06
---	--	------	------
mPD		0.0046	0.48
		nPD:	0.01
		N. weight:	0.51
		Sc. PD:	-0.022
		Sc. rank:	-8494.0

NP			
Pos.	6	obs:	exp:
tct	S	0	0.16
tcc	S	0	0.15
tca	S	0	0.27
tcg	S	0	0.04
agt	S	0	0.17
agc	S	1	0.21
ggt	G	2	5794.00
ggc	G	43037	6007.00
gga	G	0	19868.00
ggg	G	0	11370.00
---	--	------	------
mPD		0.00014	0.68
		nPD:	0.
		N. weight:	2.
		Sc. PD:	-0.11
		Sc. rank:	-71636.0

NP			
Pos.	7	obs:	exp:
tct	S	0	0.16
tcc	S	1	0.15
tca	S	0	0.27
tcg	S	0	0.04
act	T	1	13366.00
acc	T	43038	7609.00
aca	T	0	18626.00
acg	T	0	3437.00
agt	S	0	0.17
agc	S	0	0.21
---	--	------	------
mPD		0.000093	0.68
		nPD:	0.
		N. weight:	1.8
		Sc. PD:	-0.096
		Sc. rank:	-64091.0

NP			
Pos.	8	obs:	exp:
aaa	K	43014	28296.00
aag	K	26	14744.00
---	--	------	------
mPD		0.0012	0.45
		nPD:	0.
		N. weight:	0.43
		Sc. PD:	-0.022
		Sc. rank:	-13019.0

NP			
Pos.	9	obs:	exp:
cgt	R	0	853.40
cgc	R	1	1549.00
cga	R	913	3927.00
cgg	R	42123	3696.00
aga	R	0	21110.00
agg	R	3	11905.00
---	--	------	------
mPD		0.042	0.89
		nPD:	0.05
		N. weight:	2.4
		Sc. PD:	-0.019
		Sc. rank:	42894.0

NP			
Pos.	10	obs:	exp:
ttt	F	1	0.49
ttc	F	0	0.51
tct	S	42834	6945.00
tcc	S	77	6432.00
tca	S	121	11568.00
tcg	S	6	1694.00
act	T	1	0.31
acc	T	0	0.18
aca	T	0	0.43
acg	T	0	0.08
agt	S	0	7521.00
agc	S	0	8879.00
---	--	------	------
mPD		0.0095	1.6
		nPD:	0.01
		N. weight:	1.9
		Sc. PD:	-0.088
		Sc. rank:	-39282.0

NP			
Pos.	11	obs:	exp:
ttt	F	2	0.97
ttc	F	0	1.03
tat	Y	43015	22685.00
tac	Y	22	20352.00
cat	H	1	0.56
cac	H	0	0.44
---	--	------	------
mPD		0.0012	0.50
		nPD:	0.
		N. weight:	0.66
		Sc. PD:	-0.034
		Sc. rank:	-20750.0

NP			
Pos.	12	obs:	exp:
gaa	E	43014	25375.00
gag	E	26	17665.00
---	--	------	------
mPD		0.0012	0.48
		nPD:	0.
		N. weight:	0.54
		Sc. PD:	-0.028
		Sc. rank:	-16891.0

NP			
Pos.	13	obs:	exp:
caa	Q	712	26309.00
cag	Q	42328	16731.00
---	--	------	------
mPD		0.033	0.48
		nPD:	0.07
		N. weight:	0.88
		Sc. PD:	0.011
		Sc. rank:	19637.0

NP			
Pos.	14	obs:	exp:
atg	M	43040	43040.00
---	--	------	------
mPD		0	0
		nPD:	1.
		N. weight:	0.
		Sc. PD:	0
		Sc. rank:	0

NP			
Pos.	15	obs:	exp:
gat	D	1	0.57
gac	D	0	0.43
gaa	E	42731	25374.00
gag	E	308	17665.00
---	--	------	------
mPD		0.014	0.48
		nPD:	0.03
		N. weight:	0.51
		Sc. PD:	-0.013
		Sc. rank:	4217.1

NP			
Pos.	16	obs:	exp:
att	I	1	0.35
atc	I	0	0.26
ata	I	0	0.39
act	T	42971	13366.00
acc	T	34	7609.00
aca	T	30	18626.00
acg	T	4	3437.00
---	--	------	------
mPD		0.0032	0.68
		nPD:	0.
		N. weight:	1.2
		Sc. PD:	-0.059
		Sc. rank:	-29579.0

NP			
Pos.	17	obs:	exp:
tct	S	0	0.16
tcc	S	0	0.15
tca	S	0	0.27
tcg	S	0	0.04
agt	S	1	0.17
agc	S	0	0.21
gtt	V	2	0.50
gtc	V	0	0.41
gta	V	0	0.43
gtg	V	0	0.66
gat	D	42891	24262.00
gac	D	31	18660.00
ggt	G	115	15.48
ggc	G	0	16.05
gga	G	0	53.09
ggg	G	0	30.38
---	--	------	------
mPD		0.0070	0.50
		nPD:	0.01
		N. weight:	0.59
		Sc. PD:	-0.024
		Sc. rank:	-5580.7

NP			
Pos.	18	obs:	exp:
gtt	V	0	0.25
gtc	V	0	0.20
gta	V	0	0.22
gtg	V	1	0.33
ggt	G	5	5794.00
ggc	G	1	6007.00
gga	G	39842	19868.00
ggg	G	3191	11370.00
---	--	------	------
mPD		0.14	0.68
		nPD:	0.2
		N. weight:	0.57
		Sc. PD:	0.081
		Sc. rank:	18038.0

NP			
Pos.	19	obs:	exp:
aat	N	1	0.56
aac	N	0	0.44
gat	D	42510	24108.00
gac	D	140	18542.00
gaa	E	277	228.20
gag	E	110	158.80
ggt	G	2	0.27
ggc	G	0	0.28
gga	G	0	0.92
ggg	G	0	0.53
---	--	------	------
mPD		0.024	0.50
		nPD:	0.05
		N. weight:	0.57
		Sc. PD:	-0.0035
		Sc. rank:	10083.0

NP			
Pos.	20	obs:	exp:
tta	L	0	0.12
ttg	L	0	0.21
tgt	C	0	0.39
tgc	C	1	0.61
ctt	L	0	0.21
ctc	L	1	0.15
cta	L	0	0.15
ctg	L	0	0.17
cat	H	0	1.13
cac	H	2	0.87
cgt	R	28	853.30
cgc	R	42998	1548.00
cga	R	10	3927.00
cgg	R	0	3695.00
aga	R	0	21108.00
agg	R	0	11904.00
---	--	------	------
mPD		0.0020	0.89
		nPD:	0.
		N. weight:	3.4
		Sc. PD:	-0.18
		Sc. rank:	-109760.0

,

NP			
Pos.	21	obs:	exp:
cct	P	0	1.00
ccc	P	0	0.56
cca	P	0	1.01
ccg	P	3	0.42
cat	H	1	0.56
cac	H	0	0.44
caa	Q	281	26307.00
cag	Q	42755	16729.00
---	--	------	------
mPD		0.013	0.48
		nPD:	0.03
		N. weight:	0.94
		Sc. PD:	-0.025
		Sc. rank:	5579.3

,

NP			
Pos.	22	obs:	exp:
tct	S	0	2.42
tcc	S	0	2.24
tca	S	0	4.03
tcg	S	0	0.59
cat	H	1	0.56
cac	H	0	0.44
act	T	11	3.42
acc	T	0	1.94
aca	T	0	4.76
acg	T	0	0.88
aat	N	42860	24000.00
aac	N	37	18897.00
agt	S	15	2.62
agc	S	0	3.09
gat	D	116	65.57
gac	D	0	50.43
---	--	------	------
mPD		0.0083	0.50
		nPD:	0.02
		N. weight:	0.6
		Sc. PD:	-0.022
		Sc. rank:	-3014.2

,

NP			
Pos.	23	obs:	exp:
act	T	0	4.97
acc	T	4	2.83
aca	T	12	6.92
acg	T	0	1.28
gtt	V	0	0.50
gtc	V	0	0.41
gta	V	2	0.43
gtg	V	0	0.66
gct	A	5	11726.00
gcc	A	119	10749.00
gca	A	42849	17403.00
gcg	A	49	3145.00
---	--	------	------
mPD		0.0090	0.70
		nPD:	0.01
		N. weight:	0.91
		Sc. PD:	-0.037
		Sc. rank:	-9950.0

,

NP			
Pos.	24	obs:	exp:
tct	S	2	0.32
tcc	S	0	0.30
tca	S	0	0.54
tcg	S	0	0.08
act	T	42646	13357.00
acc	T	171	7605.00
aca	T	194	18615.00
acg	T	1	3435.00
aat	N	25	13.99
aac	N	0	11.01
agt	S	0	0.35
agc	S	0	0.41
gct	A	1	0.27
gcc	A	0	0.25
gca	A	0	0.40
gcg	A	0	0.07
---	--	------	------
mPD		0.018	0.68
		nPD:	0.03
		N. weight:	1.2
		Sc. PD:	-0.032
		Sc. rank:	6323.4

,

NP			
Pos.	25	obs:	exp:
gat	D	2	1.13
gac	D	0	0.87
gaa	E	406	25374.00
gag	E	42632	17664.00
---	--	------	------
mPD		0.019	0.48
		nPD:	0.04
		N. weight:	0.86
		Sc. PD:	-0.014
		Sc. rank:	12329.0

,

NP			
Pos.	26	obs:	exp:
att	I	42787	15108.00
atc	I	221	11309.00
ata	I	31	16622.00
gtt	V	1	0.25
gtc	V	0	0.20
gta	V	0	0.22
gtg	V	0	0.33
---	--	------	------
mPD		0.012	0.66
		nPD:	0.02
		N. weight:	1.
		Sc. PD:	-0.038
		Sc. rank:	-4449.3

,

NP			
Pos.	27	obs:	exp:
tct	S	0	0.16
tcc	S	0	0.15
tca	S	0	0.27
tcg	S	0	0.04
cgt	R	0	853.20
cgc	R	0	1548.00
cga	R	0	3926.00
cgg	R	5	3695.00
aaa	K	0	5.92
aag	K	9	3.08
agt	S	1	0.17
agc	S	0	0.21
aga	R	692	21106.00
agg	R	42333	11902.00
---	--	------	------
mPD		0.032	0.89
		nPD:	0.04
		N. weight:	1.2
		Sc. PD:	-0.022
		Sc. rank:	16508.0

,

NP			
Pos.	28	obs:	exp:
tct	S	0	20.33
tcc	S	0	18.83
tca	S	126	33.87
tcg	S	0	4.96
act	T	0	0.62
acc	T	0	0.35
aca	T	2	0.87
acg	T	0	0.16
agt	S	0	22.02
agc	S	0	25.99
gct	A	11	11696.00
gcc	A	13	10721.00
gca	A	42865	17358.00
gcg	A	23	3137.00
---	--	------	------
mPD		0.0081	0.70
		nPD:	0.01
		N. weight:	0.93
		Sc. PD:	-0.039
		Sc. rank:	-13660.0

,

NP			
Pos.	29	obs:	exp:
tct	S	320	6945.00
tcc	S	42572	6432.00
tca	S	148	11568.00
tcg	S	0	1694.00
agt	S	0	7521.00
agc	S	0	8879.00
---	--	------	------
mPD		0.022	1.6
		nPD:	0.01
		N. weight:	1.9
		Sc. PD:	-0.077
		Sc. rank:	-19299.0

,

NP			
Pos.	30	obs:	exp:
att	I	0	3.51
atc	I	10	2.63
ata	I	0	3.86
gtt	V	334	10778.00
gtc	V	42615	8768.00
gta	V	81	9326.00
gtg	V	0	14158.00
---	--	------	------
mPD		0.020	0.74
		nPD:	0.03
		N. weight:	1.6
		Sc. PD:	-0.044
		Sc. rank:	8413.5

,

NP			
Pos.	31	obs:	exp:
ggt	G	14	5794.00
ggc	G	1	6007.00
gga	G	838	19868.00
ggg	G	42187	11371.00
---	--	------	------
mPD		0.039	0.68
		nPD:	0.06
		N. weight:	1.3
		Sc. PD:	0.0025
		Sc. rank:	24817.0

,

NP			
Pos.	32	obs:	exp:
cgt	R	0	5.06
cgc	R	0	9.18
cga	R	0	23.27
cgg	R	0	21.90
aaa	K	176	28128.00
aag	K	42609	14657.00
aga	R	128	125.10
agg	R	127	70.53
---	--	------	------
mPD		0.026	0.47
		nPD:	0.06
		N. weight:	1.1
		Sc. PD:	0.00028
		Sc. rank:	20501.0

,

NP			
Pos.	33	obs:	exp:
atg	M	43040	43040.00
---	--	------	------
mPD		0	0
		nPD:	1.
		N. weight:	0.
		Sc. PD:	0
		Sc. rank:	0

,

NP			
Pos.	34	obs:	exp:
att	I	42602	15092.00
atc	I	374	11297.00
ata	I	17	16604.00
gtt	V	47	11.77
gtc	V	0	9.58
gta	V	0	10.19
gtg	V	0	15.46
---	--	------	------
mPD		0.020	0.66
		nPD:	0.03
		N. weight:	1.
		Sc. PD:	-0.025
		Sc. rank:	9624.2

,

NP			
Pos.	35	obs:	exp:
aat	N	27	15.11
aac	N	0	11.89
gtt	V	1	0.25
gtc	V	0	0.20
gta	V	0	0.22
gtg	V	0	0.33
gct	A	4	1.09
gcc	A	0	1.00
gca	A	0	1.62
gcg	A	0	0.29
gat	D	42843	24240.00
gac	D	39	18642.00
ggt	G	126	16.96
ggc	G	0	17.59
gga	G	0	58.16
ggg	G	0	33.29
---	--	------	------
mPD		0.0091	0.50
		nPD:	0.02
		N. weight:	0.59
		Sc. PD:	-0.021
		Sc. rank:	-1948.6

,

NP			
Pos.	36	obs:	exp:
ggt	G	1	5794.00
ggc	G	1	6007.00
gga	G	42867	19868.00
ggg	G	171	11371.00
---	--	------	------
mPD		0.0080	0.68
		nPD:	0.01
		N. weight:	0.78
		Sc. PD:	-0.033
		Sc. rank:	-11021.0

,

NP			
Pos.	37	obs:	exp:
tta	L	0	0.12
ttg	L	1	0.21
ctt	L	0	0.21
ctc	L	0	0.15
cta	L	0	0.15
ctg	L	0	0.17
att	I	42897	15108.00
atc	I	139	11309.00
ata	I	2	16621.00
gtt	V	1	0.25
gtc	V	0	0.20
gta	V	0	0.22
gtg	V	0	0.33
---	--	------	------
mPD		0.0067	0.66
		nPD:	0.01
		N. weight:	1.1
		Sc. PD:	-0.046
		Sc. rank:	-17281.0

,

NP			
Pos.	38	obs:	exp:
ggt	G	4	5794.00
ggc	G	2	6007.00
gga	G	261	19868.00
ggg	G	42773	11371.00
---	--	------	------
mPD		0.012	0.68
		nPD:	0.02
		N. weight:	1.3
		Sc. PD:	-0.048
		Sc. rank:	-4807.0

,

NP			
Pos.	39	obs:	exp:
cgt	R	0	851.10
cgc	R	0	1544.00
cga	R	434	3916.00
cgg	R	1	3686.00
aaa	K	115	75.61
aag	K	0	39.39
aga	R	41109	21054.00
agg	R	1381	11873.00
---	--	------	------
mPD		0.087	0.89
		nPD:	0.1
		N. weight:	0.57
		Sc. PD:	0.024
		Sc. rank:	14445.0

,

NP			
Pos.	40	obs:	exp:
ttt	F	54	20933.00
ttc	F	42980	22101.00
tat	Y	0	3.16
tac	Y	6	2.84
---	--	------	------
mPD		0.0028	0.50
		nPD:	0.01
		N. weight:	0.68
		Sc. PD:	-0.033
		Sc. rank:	-15139.0

,

NP			
Pos.	41	obs:	exp:
tat	Y	1030	22686.00
tac	Y	42008	20352.00
cat	H	0	1.13
cac	H	2	0.87
---	--	------	------
mPD		0.047	0.50
		nPD:	0.09
		N. weight:	0.66
		Sc. PD:	0.025
		Sc. rank:	16291.0

,

NP			
Pos.	42	obs:	exp:
tta	L	0	0.12
ttg	L	0	0.21
ctt	L	0	0.21
ctc	L	1	0.15
cta	L	0	0.15
ctg	L	0	0.17
att	I	49	15102.00
atc	I	42960	11305.00
ata	I	13	16615.00
act	T	0	1.55
acc	T	5	0.88
aca	T	0	2.16
acg	T	0	0.40
gtt	V	0	3.01
gtc	V	12	2.45
gta	V	0	2.60
gtg	V	0	3.95
---	--	------	------
mPD		0.0037	0.66
		nPD:	0.01
		N. weight:	1.4
		Sc. PD:	-0.066
		Sc. rank:	-29990.0

,

NP			
Pos.	43	obs:	exp:
caa	Q	43022	26309.00
cag	Q	18	16731.00
---	--	------	------
mPD		0.00084	0.48
		nPD:	0.
		N. weight:	0.51
		Sc. PD:	-0.026
		Sc. rank:	-16521.0

,

NP			
Pos.	44	obs:	exp:
atg	M	43040	43040.00
---	--	------	------
mPD		0	0
		nPD:	1.
		N. weight:	0.
		Sc. PD:	0
		Sc. rank:	0

,

NP			
Pos.	45	obs:	exp:
tct	S	0	0.16
tcc	S	0	0.15
tca	S	0	0.27
tcg	S	0	0.04
tgt	C	52	16819.00
tgc	C	42987	26220.00
agt	S	0	0.17
agc	S	1	0.21
---	--	------	------
mPD		0.0025	0.48
		nPD:	0.01
		N. weight:	0.5
		Sc. PD:	-0.024
		Sc. rank:	-11643.0

,

NP			
Pos.	46	obs:	exp:
act	T	42430	13366.00
acc	T	572	7610.00
aca	T	11	18627.00
acg	T	27	3438.00
---	--	------	------
mPD		0.028	0.68
		nPD:	0.04
		N. weight:	1.1
		Sc. PD:	-0.015
		Sc. rank:	17327.0

,

NP			
Pos.	47	obs:	exp:
gat	D	1	0.57
gac	D	0	0.43
gaa	E	43002	25374.00
gag	E	37	17665.00
---	--	------	------
mPD		0.0018	0.48
		nPD:	0.
		N. weight:	0.54
		Sc. PD:	-0.027
		Sc. rank:	-15021.0

,

NP			
Pos.	48	obs:	exp:
ttt	F	3	1.46
ttc	F	0	1.54
tta	L	1	4994.00
ttg	L	0	8897.00
ctt	L	42728	9002.00
ctc	L	290	6311.00
cta	L	4	6608.00
ctg	L	11	7222.00
att	I	3	1.05
atc	I	0	0.79
ata	I	0	1.16
---	--	------	------
mPD		0.014	1.2
		nPD:	0.01
		N. weight:	1.6
		Sc. PD:	-0.065
		Sc. rank:	-18636.0

,

NP			
Pos.	49	obs:	exp:
cgt	R	0	0.02
cgc	R	0	0.04
cga	R	0	0.09
cgg	R	0	0.09
aaa	K	42964	28295.00
aag	K	75	14744.00
aga	R	1	0.49
agg	R	0	0.28
---	--	------	------
mPD		0.0035	0.45
		nPD:	0.01
		N. weight:	0.42
		Sc. PD:	-0.019
		Sc. rank:	-7996.5

,

NP			
Pos.	50	obs:	exp:
ttt	F	0	1.95
ttc	F	4	2.05
tta	L	0	4994.00
ttg	L	0	8897.00
ctt	L	21	9003.00
ctc	L	42963	6311.00
cta	L	52	6608.00
ctg	L	0	7223.00
---	--	------	------
mPD		0.0036	1.2
		nPD:	0.
		N. weight:	2.
		Sc. PD:	-0.1
		Sc. rank:	-56657.0

,

NP			
Pos.	51	obs:	exp:
tct	S	0	6943.00
tcc	S	0	6429.00
tca	S	0	11564.00
tcg	S	0	1694.00
att	I	1	0.35
atc	I	0	0.26
ata	I	0	0.39
act	T	5	1.55
acc	T	0	0.88
aca	T	0	2.16
acg	T	0	0.40
aat	N	5	2.80
aac	N	0	2.20
agt	S	42989	7519.00
agc	S	35	8876.00
ggt	G	5	0.67
ggc	G	0	0.70
gga	G	0	2.31
ggg	G	0	1.32
---	--	------	------
mPD		0.0024	1.6
		nPD:	0.
		N. weight:	1.8
		Sc. PD:	-0.094
		Sc. rank:	-59772.0

,

NP			
Pos.	52	obs:	exp:
gat	D	42346	24328.00
gac	D	693	18711.00
ggt	G	0	0.13
ggc	G	1	0.14
gga	G	0	0.46
ggg	G	0	0.26
---	--	------	------
mPD		0.032	0.49
		nPD:	0.06
		N. weight:	0.51
		Sc. PD:	0.0047
		Sc. rank:	10911.0

,

NP			
Pos.	53	obs:	exp:
tta	L	0	0.12
ttg	L	0	0.21
tat	Y	988	527.10
tac	Y	12	472.90
tgt	C	2	0.78
tgc	C	0	1.22
ctt	L	1	0.21
ctc	L	0	0.15
cta	L	0	0.15
ctg	L	0	0.17
cat	H	41992	23704.00
cac	H	33	18321.00
caa	Q	3	3.67
cag	Q	3	2.33
aat	N	6	3.36
aac	N	0	2.64
---	--	------	------
mPD		0.048	0.54
		nPD:	0.09
		N. weight:	0.59
		Sc. PD:	0.02
		Sc. rank:	14335.0

,

NP			
Pos.	54	obs:	exp:
gat	D	6	3.39
gac	D	0	2.61
gaa	E	42790	25371.00
gag	E	243	17662.00
ggt	G	0	0.13
ggc	G	0	0.14
gga	G	1	0.46
ggg	G	0	0.26
---	--	------	------
mPD		0.012	0.48
		nPD:	0.02
		N. weight:	0.51
		Sc. PD:	-0.016
		Sc. rank:	1394.3

,

NP			
Pos.	55	obs:	exp:
cgt	R	0	0.16
cgc	R	0	0.29
cga	R	0	0.73
cgg	R	0	0.69
aga	R	8	3.92
agg	R	0	2.21
gct	A	0	0.27
gcc	A	0	0.25
gca	A	0	0.40
gcg	A	1	0.07
ggt	G	38	5793.00
ggc	G	2	6006.00
gga	G	36834	19864.00
ggg	G	6157	11368.00
---	--	------	------
mPD		0.25	0.68
		nPD:	0.36
		N. weight:	0.45
		Sc. PD:	0.13
		Sc. rank:	15220.0

,

NP			
Pos.	56	obs:	exp:
cgt	R	810	853.40
cgc	R	281	1549.00
cga	R	475	3927.00
cgg	R	40938	3696.00
aga	R	5	21110.00
agg	R	531	11905.00
---	--	------	------
mPD		0.096	0.89
		nPD:	0.11
		N. weight:	2.3
		Sc. PD:	0.12
		Sc. rank:	61198.0

,

NP			
Pos.	57	obs:	exp:
tta	L	508	4994.00
ttg	L	42312	8898.00
ctt	L	0	9004.00
ctc	L	0	6311.00
cta	L	113	6609.00
ctg	L	107	7223.00
---	--	------	------
mPD		0.039	1.2
		nPD:	0.03
		N. weight:	1.5
		Sc. PD:	-0.033
		Sc. rank:	16678.0

,

NP			
Pos.	58	obs:	exp:
tta	L	0	0.46
ttg	L	0	0.83
ctt	L	0	0.84
ctc	L	4	0.59
cta	L	0	0.61
ctg	L	0	0.67
att	I	50	15105.00
atc	I	42959	11307.00
ata	I	21	16618.00
gtt	V	0	1.50
gtc	V	6	1.22
gta	V	0	1.30
gtg	V	0	1.97
---	--	------	------
mPD		0.0038	0.66
		nPD:	0.01
		N. weight:	1.4
		Sc. PD:	-0.066
		Sc. rank:	-29410.0

,

NP

Pos.	59	obs:	exp:
caa	Q	34410	26309.00
cag	Q	8630	16731.00
---	--	------	------
mPD		0.32	0.48
		nPD:	0.67
		N. weight:	0.085
		Sc. PD:	0.051
		Sc. rank:	3037.5

,

NP

Pos.	60	obs:	exp:
aat	N	8859	24080.00
aac	N	34181	18960.00
---	--	------	------
mPD		0.33	0.49
		nPD:	0.66
		N. weight:	0.27
		Sc. PD:	0.16
		Sc. rank:	9715.0

,

NP

Pos.	61	obs:	exp:
tct	S	0	6945.00
tcc	S	0	6432.00
tca	S	0	11568.00
tcg	S	0	1694.00
agt	S	179	7521.00
agc	S	42861	8879.00
---	--	------	------
mPD		0.0083	1.6
		nPD:	0.01
		N. weight:	1.6
		Sc. PD:	-0.078
		Sc. rank:	-38100.0

,

NP

Pos.	62	obs:	exp:
tta	L	208	4980.00
ttg	L	42566	8873.00
ctt	L	0	8978.00
ctc	L	0	6294.00
cta	L	0	6590.00
ctg	L	144	7203.00
att	I	0	40.72
atc	I	0	30.48
ata	I	116	44.80
atg	M	6	6.00
---	--	------	------
mPD		0.027	1.2
		nPD:	0.02
		N. weight:	1.6
		Sc. PD:	-0.049
		Sc. rank:	3957.7

,

NP

Pos.	63	obs:	exp:
act	T	30	13365.00
acc	T	3	7609.00
aca	T	42977	18625.00
acg	T	26	3437.00
gct	A	0	1.09
gcc	A	0	1.00
gca	A	4	1.62
gcg	A	0	0.29
---	--	------	------
mPD		0.0029	0.68
		nPD:	0.
		N. weight:	0.86
		Sc. PD:	-0.042
		Sc. rank:	-22391.0

,

NP

Pos.	64	obs:	exp:
tta	L	0	0.12
ttg	L	0	0.21
ctt	L	0	0.21
ctc	L	0	0.15
cta	L	1	0.15
ctg	L	0	0.17
att	I	4	15098.00
atc	I	3	11301.00
ata	I	43003	16610.00
atg	M	4	4.00
act	T	0	0.31
acc	T	0	0.18
aca	T	1	0.43
acg	T	0	0.08
gtt	V	0	6.01
gtc	V	0	4.89
gta	V	24	5.20
gtg	V	0	7.90
---	--	------	------
mPD		0.0017	0.66
		nPD:	0.
		N. weight:	0.99
		Sc. PD:	-0.05
		Sc. rank:	-30418.0

,

NP

Pos.	65	obs:	exp:
caa	Q	0	0.61
cag	Q	1	0.39
aaa	K	0	0.66
aag	K	1	0.34
gaa	E	99	25374.00
gag	E	42939	17664.00
---	--	------	------
mPD		0.0047	0.48
		nPD:	0.01
		N. weight:	0.91
		Sc. PD:	-0.04
		Sc. rank:	-15080.0

,

NP

Pos.	66	obs:	exp:
cgt	R	0	7.02
cgc	R	0	12.74
cga	R	0	32.30
cgg	R	0	30.40
aaa	K	42590	28063.00
aag	K	96	14623.00
aga	R	344	173.60
agg	R	10	97.92
---	--	------	------
mPD		0.021	0.47
		nPD:	0.05
		N. weight:	0.42
		Sc. PD:	-0.0041
		Sc. rank:	6808.3

,

NP

Pos.	67	obs:	exp:
atg	M	43040	43040.00
---	--	------	------
mPD		0	0
		nPD:	1.
		N. weight:	0.
		Sc. PD:	0
		Sc. rank:	0

,

NP

Pos.	68	obs:	exp:
tta	L	1	0.12
ttg	L	0	0.21
ctt	L	0	0.21
ctc	L	0	0.15
cta	L	0	0.15
ctg	L	0	0.17
att	I	0	1.76
atc	I	0	1.31
ata	I	5	1.93
gtt	V	2	10775.00
gtc	V	4	8765.00
gta	V	41441	9324.00
gtg	V	1570	14154.00
gct	A	0	4.63
gcc	A	0	4.25
gca	A	17	6.88
gcg	A	0	1.24
---	--	------	------
mPD		0.072	0.74
		nPD:	0.1
		N. weight:	1.4
		Sc. PD:	0.056
		Sc. rank:	35150.0

,

NP

Pos.	69	obs:	exp:
tta	L	0	4994.00
ttg	L	0	8898.00
ctt	L	123	9003.00
ctc	L	42832	6311.00
cta	L	82	6609.00
ctg	L	1	7223.00
att	I	0	0.70
atc	I	2	0.53
ata	I	0	0.77
---	--	------	------
mPD		0.0096	1.2
		nPD:	0.01
		N. weight:	2.
		Sc. PD:	-0.089
		Sc. rank:	-36134.0

,

NP

Pos.	70	obs:	exp:
tct	S	42955	6943.00
tcc	S	43	6429.00
tca	S	20	11564.00
tcg	S	6	1694.00
cct	P	16	5.33
ccc	P	0	3.00
cca	P	0	5.41
ccg	P	0	2.26
agt	S	0	7519.00
agc	S	0	8876.00
---	--	------	------
mPD		0.0039	1.6
		nPD:	0.
		N. weight:	1.9
		Sc. PD:	-0.096
		Sc. rank:	-58562.0

,

NP

Pos.	71	obs:	exp:
tct	S	37	5.97
tcc	S	0	5.53
tca	S	0	9.94
tcg	S	0	1.46
act	T	1	0.31
acc	T	0	0.18
aca	T	0	0.43
acg	T	0	0.08
agt	S	0	6.47
agc	S	0	7.63
gct	A	42813	11720.00
gcc	A	167	10744.00
gca	A	8	17395.00
gcg	A	14	3144.00
---	--	------	------
mPD		0.011	0.70
		nPD:	0.02
		N. weight:	1.3
		Sc. PD:	-0.051
		Sc. rank:	-9838.6

,

NP

Pos.	72	obs:	exp:
ttt	F	42914	20935.00
ttc	F	124	22103.00
tat	Y	2	1.05
tac	Y	0	0.95
---	--	------	------
mPD		0.0058	0.50
		nPD:	0.01
		N. weight:	0.73
		Sc. PD:	-0.031
		Sc. rank:	-10470.0

,

NP

Pos.	73	obs:	exp:
gct	A	18	4.91
gcc	A	0	4.50
gca	A	0	7.28
gcg	A	0	1.32
gat	D	42940	24319.00
gac	D	82	18703.00
---	--	------	------
mPD		0.0046	0.49
		nPD:	0.01
		N. weight:	0.58
		Sc. PD:	-0.026
		Sc. rank:	-9806.7

,

NP

Pos.	74	obs:	exp:
gtt	V	0	1.25
gtc	V	0	1.02
gta	V	5	1.08
gtg	V	0	1.65
gct	A	0	0.27
gcc	A	0	0.25
gca	A	1	0.40
gcg	A	0	0.07
gat	D	0	1.70
gac	D	3	1.30
gaa	E	42441	25367.00
gag	E	585	17659.00
ggt	G	0	0.67
ggc	G	0	0.70
gga	G	5	2.31
ggg	G	0	1.32
---	--	------	------
mPD		0.027	0.48
		nPD:	0.06
		N. weight:	0.48
		Sc. PD:	0.00069
		Sc. rank:	9205.1

,

NP

Pos.	75	obs:	exp:
cgt	R	0	853.40
cgc	R	0	1549.00
cga	R	17	3927.00
cgg	R	0	3696.00
aga	R	42752	21110.00
agg	R	271	11905.00
---	--	------	------
mPD		0.013	0.89
		nPD:	0.02
		N. weight:	0.7
		Sc. PD:	-0.027
		Sc. rank:	-5410.8

,

NP

Pos.	76	obs:	exp:
cgt	R	0	853.40
cgc	R	0	1549.00
cga	R	0	3927.00
cgg	R	10	3696.00
aga	R	3101	21110.00
agg	R	39929	11905.00
---	--	------	------
mPD		0.13	0.89
		nPD:	0.15
		N. weight:	1.
		Sc. PD:	0.095
		Sc. rank:	30147.0

,

NP

Pos.	77	obs:	exp:
cat	H	1	0.56
cac	H	0	0.44
act	T	1	0.31
acc	T	0	0.18
aca	T	0	0.43
acg	T	0	0.08
aat	N	42195	24078.00
aac	N	843	18960.00
---	--	------	------
mPD		0.038	0.49
		nPD:	0.08
		N. weight:	0.51
		Sc. PD:	0.011
		Sc. rank:	11697.0

,

NP

Pos.	78	obs:	exp:
cgt	R	0	8.72
cgc	R	0	15.83
cga	R	0	40.15
cgg	R	0	37.78
act	T	0	0.62
acc	T	0	0.35
aca	T	2	0.87
acg	T	0	0.16
aat	N	0	2.24
aac	N	4	1.76
aaa	K	42328	28003.00
aag	K	266	14591.00
aga	R	439	215.80
agg	R	1	121.70
---	--	------	------
mPD		0.033	0.48
		nPD:	0.07
		N. weight:	0.4
		Sc. PD:	0.0054
		Sc. rank:	8974.1

,

NP

Pos.	79	obs:	exp:
ttt	F	0	0.49
ttc	F	1	0.51
tat	Y	672	22683.00
tac	Y	42361	20350.00
cat	H	1	3.38
cac	H	5	2.62
---	--	------	------
mPD		0.031	0.50
		nPD:	0.06
		N. weight:	0.69
		Sc. PD:	0.0048
		Sc. rank:	14580.0

NP

Pos.	80	obs:	exp:
tta	L	0	4994.00
ttg	L	61	8898.00
ctt	L	31	9004.00
ctc	L	0	6311.00
cta	L	813	6609.00
ctg	L	42135	7223.00
---	--	------	------
mPD		0.041	1.2
		nPD:	0.04
		N. weight:	1.7
		Sc. PD:	-0.033
		Sc. rank:	22335.0

NP

Pos.	81	obs:	exp:
gat	D	2	1.13
gac	D	0	0.87
gaa	E	43000	25373.00
gag	E	37	17664.00
ggt	G	0	0.13
ggc	G	0	0.14
gga	G	1	0.46
ggg	G	0	0.26
---	--	------	------
mPD		0.0019	0.48
		nPD:	0.
		N. weight:	0.54
		Sc. PD:	-0.027
		Sc. rank:	-14855.0

NP

Pos.	82	obs:	exp:
aaa	K	4	2.63
aag	K	0	1.37
gtt	V	0	0.25
gtc	V	0	0.20
gta	V	1	0.22
gtg	V	0	0.33
gat	D	0	39.00
gac	D	69	30.00
gaa	E	42470	25328.00
gag	E	491	17633.00
ggt	G	0	0.67
ggc	G	0	0.70
gga	G	5	2.31
ggg	G	0	1.32
---	--	------	------
mPD		0.026	0.49
		nPD:	0.05
		N. weight:	0.49
		Sc. PD:	-0.00062
		Sc. rank:	9136.1

NP

Pos.	83	obs:	exp:
tat	Y	0	0.53
tac	Y	1	0.47
cat	H	295	24276.00
cac	H	42744	18763.00
---	--	------	------
mPD		0.014	0.49
		nPD:	0.03
		N. weight:	0.82
		Sc. PD:	-0.022
		Sc. rank:	4963.3

NP

Pos.	84	obs:	exp:
cct	P	14	14343.00
ccc	P	42995	8069.00
cca	P	31	14541.00
ccg	P	0	6087.00
---	--	------	------
mPD		0.0021	0.72
		nPD:	0.
		N. weight:	1.7
		Sc. PD:	-0.087
		Sc. rank:	-50436.0

NP

Pos.	85	obs:	exp:
tct	S	0	6939.00
tcc	S	0	6426.00
tca	S	0	11558.00
tcg	S	0	1693.00
act	T	0	0.31
acc	T	1	0.18
aca	T	0	0.43
acg	T	0	0.08
aat	N	2	20.14
aac	N	34	15.86
agt	S	1011	7514.00
agc	S	41989	8871.00
ggt	G	0	0.40
ggc	G	3	0.42
gga	G	0	1.38
ggg	G	0	0.79
---	--	------	------
mPD		0.048	1.6
		nPD:	0.03
		N. weight:	1.5
		Sc. PD:	-0.038
		Sc. rank:	12336.0

NP

Pos.	86	obs:	exp:
tct	S	0	4.52
tcc	S	0	4.18
tca	S	1	7.53
tcg	S	27	1.10
act	T	0	21.43
acc	T	0	12.20
aca	T	0	29.86
acg	T	69	5.51
agt	S	0	4.89
agc	S	0	5.78
gtt	V	0	5.26
gtc	V	0	4.28
gta	V	0	4.55
gtg	V	21	6.91
gct	A	267	11692.00
gcc	A	0	10717.00
gca	A	463	17352.00
gcg	A	42167	3136.00
ggt	G	0	3.37
ggc	G	0	3.49
gga	G	0	11.54
ggg	G	25	6.60
---	--	------	------
mPD		0.040	0.70
		nPD:	0.06
		N. weight:	2.6
		Sc. PD:	0.0052
		Sc. rank:	51572.0

NP

Pos.	87	obs:	exp:
ggt	G	2	5794.00
ggc	G	0	6007.00
gga	G	183	19868.00
ggg	G	42855	11371.00
---	--	------	------
mPD		0.0086	0.68
		nPD:	0.01
		N. weight:	1.3
		Sc. PD:	-0.056
		Sc. rank:	-15404.0

NP

Pos.	88	obs:	exp:
cgt	R	0	0.02
cgc	R	0	0.04
cga	R	0	0.09
cgg	R	0	0.09
aaa	K	42808	28295.00
aag	K	231	14744.00
aga	R	1	0.49
agg	R	0	0.28
---	--	------	------
mPD		0.011	0.45
		nPD:	0.02
		N. weight:	0.4
		Sc. PD:	-0.012
		Sc. rank:	1066.0

NP

Pos.	89	obs:	exp:
aat	N	1	0.56
aac	N	0	0.44
gat	D	40996	24328.00
gac	D	2043	18711.00
---	--	------	------
mPD		0.090	0.49
		nPD:	0.18
		N. weight:	0.41
		Sc. PD:	0.051
		Sc. rank:	12554.0

NP

Pos.	90	obs:	exp:
tta	L	0	0.12
ttg	L	0	0.21
tct	S	0	0.32
tcc	S	2	0.30
tca	S	0	0.54
tcg	S	0	0.08
ctt	L	0	0.21
ctc	L	1	0.15
cta	L	0	0.15
ctg	L	0	0.17
cct	P	779	14341.00
ccc	P	42235	8068.00
cca	P	21	14539.00
ccg	P	0	6087.00
cat	H	0	0.56
cac	H	1	0.44
act	T	0	0.31
acc	T	1	0.18
aca	T	0	0.43
acg	T	0	0.08
agt	S	0	0.35
agc	S	0	0.41
---	--	------	------
mPD		0.037	0.72
		nPD:	0.05
		N. weight:	1.6
		Sc. PD:	-0.0065
		Sc. rank:	30331.0

NP

Pos.	91	obs:	exp:
cgt	R	0	0.44
cgc	R	0	0.79
cga	R	0	2.01
cgg	R	0	1.89
atg	M	1	1.00
aaa	K	6	28281.00
aag	K	43011	14736.00
aga	R	0	10.79
agg	R	22	6.09
---	--	------	------
mPD		0.0013	0.45
		nPD:	0.
		N. weight:	1.1
		Sc. PD:	-0.056
		Sc. rank:	-32239.0

NP

Pos.	92	obs:	exp:
cgt	R	0	0.02
cgc	R	0	0.04
cga	R	0	0.09
cgg	R	0	0.09
aaa	K	42991	28295.00
aag	K	47	14743.00
aga	R	1	0.49
agg	R	0	0.28
gaa	E	1	0.59
gag	E	0	0.41
---	--	------	------
mPD		0.0023	0.45
		nPD:	0.01
		N. weight:	0.43
		Sc. PD:	-0.021
		Sc. rank:	-10285.0

NP			
Pos.	93	obs:	exp:
act	T	42863	13366.00
acc	T	75	7610.00
aca	T	40	18627.00
acg	T	62	3438.00
---	--	------	------
mPD		0.0082	0.68
		nPD:	0.01
		N. weight:	1.2
		Sc. PD:	-0.049
		Sc. rank:	-15565.0

,

NP			
Pos.	94	obs:	exp:
ggt	G	10	5794.00
ggc	G	0	6007.00
gga	G	841	19868.00
ggg	G	42189	11371.00
---	--	------	------
mPD		0.039	0.68
		nPD:	0.06
		N. weight:	1.3
		Sc. PD:	0.0023
		Sc. rank:	24598.0

,

NP			
Pos.	95	obs:	exp:
ggt	G	18	5794.00
ggc	G	63	6007.00
gga	G	361	19868.00
ggg	G	42598	11371.00
---	--	------	------
mPD		0.020	0.68
		nPD:	0.03
		N. weight:	1.3
		Sc. PD:	-0.032
		Sc. rank:	11738.0

,

NP			
Pos.	96	obs:	exp:
tct	S	0	0.65
tcc	S	3	0.60
tca	S	1	1.08
tcg	S	0	0.16
cct	P	76	14341.00
ccc	P	40419	8068.00
cca	P	2538	14540.00
ccg	P	3	6087.00
agt	S	0	0.70
agc	S	0	0.83
---	--	------	------
mPD		0.11	0.72
		nPD:	0.16
		N. weight:	1.5
		Sc. PD:	0.15
		Sc. rank:	44033.0

,

NP			
Pos.	97	obs:	exp:
tta	L	30	6.15
ttg	L	0	10.96
ctt	L	0	11.09
ctc	L	0	7.77
cta	L	23	8.14
ctg	L	0	8.89
att	I	2	15081.00
atc	I	7	11288.00
ata	I	42952	16591.00
act	T	0	0.31
acc	T	0	0.18
aca	T	1	0.43
acg	T	0	0.08
gtt	V	0	6.26
gtc	V	0	5.09
gta	V	25	5.42
gtg	V	0	8.23
---	--	------	------
mPD		0.0041	0.66
		nPD:	0.01
		N. weight:	0.99
		Sc. PD:	-0.047
		Sc. rank:	-20208.0

,

NP			
Pos.	98	obs:	exp:
tat	Y	127	22687.00
tac	Y	42913	20353.00
---	--	------	------
mPD		0.0059	0.50
		nPD:	0.01
		N. weight:	0.76
		Sc. PD:	-0.032
		Sc. rank:	-10419.0

,

NP			
Pos.	99	obs:	exp:
cgt	R	0	844.10
cgc	R	0	1532.00
cga	R	2	3884.00
cgg	R	0	3656.00
aaa	K	132	305.70
aag	K	333	159.30
aga	R	9857	20882.00
agg	R	32715	11776.00
ggt	G	0	0.13
ggc	G	0	0.14
gga	G	0	0.46
ggg	G	1	0.26
---	--	------	------
mPD		0.38	0.90
		nPD:	0.42
		N. weight:	0.63
		Sc. PD:	0.22
		Sc. rank:	21686.0

,

NP			
Pos.	100	obs:	exp:
cgt	R	0	853.10
cgc	R	0	1548.00
cga	R	4	3926.00
cgg	R	0	3695.00
aaa	K	14	9.20
aag	K	0	4.80
aga	R	42960	21104.00
agg	R	62	11901.00
---	--	------	------
mPD		0.0037	0.89
		nPD:	0.
		N. weight:	0.73
		Sc. PD:	-0.036
		Sc. rank:	-19473.0

,

NP			
Pos.	101	obs:	exp:
ttt	F	0	1.46
ttc	F	3	1.54
tta	L	2	0.35
ttg	L	0	0.62
ctt	L	0	0.63
ctc	L	1	0.44
cta	L	0	0.46
ctg	L	0	0.50
att	I	5	646.30
atc	I	1804	483.70
ata	I	32	711.00
atg	M	2	2.00
act	T	0	0.62
acc	T	2	0.35
aca	T	0	0.87
acg	T	0	0.16
gtt	V	686	10301.00
gtc	V	38832	8380.00
gta	V	1602	8914.00
gtg	V	6	13531.00
gct	A	0	17.17
gcc	A	63	15.74
gca	A	0	25.48
gcg	A	0	4.61
---	--	------	------
mPD		0.19	0.83
		nPD:	0.23
		N. weight:	1.4
		Sc. PD:	0.23
		Sc. rank:	44312.0

,

NP			
Pos.	102	obs:	exp:
aat	N	14	10.07
aac	N	4	7.93
gat	D	42863	24310.00
gac	D	143	18696.00
gaa	E	4	4.13
gag	E	3	2.87
ggt	G	9	1.21
ggc	G	0	1.26
gga	G	0	4.15
ggg	G	0	2.38
---	--	------	------
mPD		0.0084	0.49
		nPD:	0.02
		N. weight:	0.57
		Sc. PD:	-0.021
		Sc. rank:	-2687.6

,

NP			
Pos.	103	obs:	exp:
cgt	R	0	0.24
cgc	R	0	0.43
cga	R	0	1.09
cgg	R	0	1.03
aga	R	11	5.89
agg	R	1	3.32
ggt	G	3	5793.00
ggc	G	1	6005.00
gga	G	12208	19862.00
ggg	G	30816	11368.00
---	--	------	------
mPD		0.41	0.68
		nPD:	0.6
		N. weight:	0.6
		Sc. PD:	0.31
		Sc. rank:	21282.0

,

NP			
Pos.	104	obs:	exp:
cgt	R	0	7.85
cgc	R	0	14.25
cga	R	0	36.13
cgg	R	0	34.00
aat	N	0	0.56
aac	N	1	0.44
aaa	K	42407	28035.00
aag	K	236	14608.00
aga	R	392	194.20
agg	R	4	109.50
---	--	------	------
mPD		0.029	0.47
		nPD:	0.06
		N. weight:	0.41
		Sc. PD:	0.0027
		Sc. rank:	8327.8

,

NP			
Pos.	105	obs:	exp:
tgg	W	43040	43040.00
---	--	------	------
mPD		0	0
		nPD:	1.
		N. weight:	0.
		Sc. PD:	0
		Sc. rank:	0

NP			
Pos.	106	obs:	exp:
cgt	R	0	0.08
cgc	R	0	0.14
cga	R	0	0.36
cgg	R	0	0.34
att	I	49	57.22
atc	I	0	42.83
ata	I	114	62.95
atg	M	42647	42647.00
act	T	1	52.79
acc	T	0	30.06
aca	T	1	73.57
acg	T	168	13.58
aaa	K	0	1.31
aag	K	2	0.69
aga	R	0	1.96
agg	R	4	1.11
gtt	V	0	13.53
gtc	V	0	11.00
gta	V	0	11.70
gtg	V	54	17.77
---	--	------	------
mPD		0.018	0.027
		nPD:	0.67
		N. weight:	0.013
		Sc. PD:	0.0078
		Sc. rank:	470.2

NP			
Pos.	107	obs:	exp:
cgt	R	0	853.40
cgc	R	0	1549.00
cga	R	1	3927.00
cgg	R	1	3696.00
aga	R	573	21110.00
agg	R	42465	11905.00
---	--	------	------
mPD		0.026	0.89
		nPD:	0.03
		N. weight:	1.3
		Sc. PD:	-0.031
		Sc. rank:	10962.0

NP			
Pos.	108	obs:	exp:
gaa	E	42271	25374.00
gag	E	768	17665.00
ggt	G	0	0.13
ggc	G	0	0.14
gga	G	1	0.46
ggg	G	0	0.26
---	--	------	------
mPD		0.035	0.48
		nPD:	0.07
		N. weight:	0.46
		Sc. PD:	0.0077
		Sc. rank:	10309.0

NP			
Pos.	109	obs:	exp:
tta	L	0	4992.00
ttg	L	0	8895.00
ctt	L	130	9000.00
ctc	L	42849	6309.00
cta	L	44	6606.00
ctg	L	0	7220.00
att	I	0	5.97
atc	I	17	4.47
ata	I	0	6.57
---	--	------	------
mPD		0.0088	1.2
		nPD:	0.01
		N. weight:	2.
		Sc. PD:	-0.09
		Sc. rank:	-37793.0

NP			
Pos.	110	obs:	exp:
ttt	F	0	0.97
ttc	F	2	1.03
att	I	105	69.86
atc	I	94	52.29
ata	I	0	76.85
act	T	0	1.24
acc	T	4	0.71
aca	T	0	1.73
acg	T	0	0.32
gtt	V	93	10725.00
gtc	V	42724	8724.00
gta	V	1	9280.00
gtg	V	0	14088.00
gct	A	0	4.63
gcc	A	17	4.25
gca	A	0	6.88
gcg	A	0	1.24
---	--	------	------
mPD		0.020	0.75
		nPD:	0.03
		N. weight:	1.6
		Sc. PD:	-0.046
		Sc. rank:	7971.1

NP			
Pos.	111	obs:	exp:
tta	L	0	4994.00
ttg	L	0	8898.00
ctt	L	42982	9003.00
ctc	L	43	6311.00
cta	L	7	6609.00
ctg	L	6	7223.00
cct	P	1	0.33
ccc	P	0	0.19
cca	P	0	0.34
ccg	P	0	0.14
att	I	1	0.35
atc	I	0	0.26
ata	I	0	0.39
---	--	------	------
mPD		0.0027	1.2
		nPD:	0.
		N. weight:	1.6
		Sc. PD:	-0.082
		Sc. rank:	-51055.0

NP			
Pos.	112	obs:	exp:
tat	Y	42993	22686.00
tac	Y	45	20352.00
tgt	C	2	0.78
tgc	C	0	1.22
---	--	------	------
mPD		0.0022	0.50
		nPD:	0.
		N. weight:	0.66
		Sc. PD:	-0.032
		Sc. rank:	-16897.0

NP			
Pos.	113	obs:	exp:
aat	N	0	1.68
aac	N	3	1.32
gat	D	217	24324.00
gac	D	42814	18707.00
gaa	E	5	3.54
gag	E	1	2.46
---	--	------	------
mPD		0.010	0.49
		nPD:	0.02
		N. weight:	0.83
		Sc. PD:	-0.027
		Sc. rank:	-18.9

NP			
Pos.	114	obs:	exp:
aaa	K	42843	28295.00
aag	K	196	14744.00
gaa	E	1	0.59
gag	E	0	0.41
---	--	------	------
mPD		0.0091	0.45
		nPD:	0.02
		N. weight:	0.41
		Sc. PD:	-0.014
		Sc. rank:	-315.7

NP			
Pos.	115	obs:	exp:
aaa	K	2	1.31
aag	K	0	0.69
gat	D	3	3.39
gac	D	3	2.61
gaa	E	34681	25368.00
gag	E	8348	17661.00
ggt	G	0	0.40
ggc	G	0	0.42
gga	G	3	1.38
ggg	G	0	0.79
---	--	------	------
mPD		0.31	0.48
		nPD:	0.65
		N. weight:	0.11
		Sc. PD:	0.063
		Sc. rank:	3952.7

NP			
Pos.	116	obs:	exp:
aaa	K	1	0.66
aag	K	0	0.34
gat	D	0	0.57
gac	D	1	0.43
gaa	E	42989	25374.00
gag	E	49	17664.00
---	--	------	------
mPD		0.0024	0.48
		nPD:	0.
		N. weight:	0.54
		Sc. PD:	-0.026
		Sc. rank:	-13111.0

NP			
Pos.	117	obs:	exp:
tta	L	0	0.23
ttg	L	0	0.41
ctt	L	0	0.42
ctc	L	0	0.29
cta	L	2	0.31
ctg	L	0	0.34
att	I	1	15101.00
atc	I	1	11303.00
ata	I	43016	16613.00
atg	M	11	11.00
gtt	V	0	2.25
gtc	V	0	1.83
gta	V	9	1.95
gtg	V	0	2.96
---	--	------	------
mPD		0.0011	0.66
		nPD:	0.
		N. weight:	0.99
		Sc. PD:	-0.051
		Sc. rank:	-32211.0

,

NP			
Pos.	118	obs:	exp:
cgt	R	0	853.40
cgc	R	0	1549.00
cga	R	0	3927.00
cgg	R	1	3696.00
aga	R	239	21110.00
agg	R	42800	11905.00
---	--	------	------
mPD		0.011	0.89
		nPD:	0.01
		N. weight:	1.3
		Sc. PD:	-0.053
		Sc. rank:	-15023.0

,

NP			
Pos.	119	obs:	exp:
tta	L	0	0.12
ttg	L	0	0.21
ctt	L	0	0.21
ctc	L	0	0.15
cta	L	1	0.15
ctg	L	0	0.17
caa	Q	4	2.45
cag	Q	0	1.55
cgt	R	4	853.30
cgc	R	1	1548.00
cga	R	42379	3926.00
cgg	R	193	3695.00
aga	R	456	21108.00
agg	R	2	11903.00
---	--	------	------
mPD		0.031	0.89
		nPD:	0.03
		N. weight:	2.4
		Sc. PD:	-0.047
		Sc. rank:	28451.0

,

NP			
Pos.	120	obs:	exp:
tta	L	0	0.12
ttg	L	0	0.21
ctt	L	0	0.21
ctc	L	1	0.15
cta	L	0	0.15
ctg	L	0	0.17
att	I	93	15062.00
atc	I	42783	11275.00
ata	I	32	16571.00
act	T	0	3.73
acc	T	12	2.12
aca	T	0	5.19
acg	T	0	0.96
gtt	V	113	29.81
gtc	V	6	24.25
gta	V	0	25.79
gtg	V	0	39.15
---	--	------	------
mPD		0.017	0.67
		nPD:	0.03
		N. weight:	1.4
		Sc. PD:	-0.039
		Sc. rank:	5751.6

,

NP			
Pos.	121	obs:	exp:
tgg	W	43040	43040.00
---	--	------	------
mPD		0	0
		nPD:	1.
		N. weight:	0.
		Sc. PD:	0
		Sc. rank:	0

,

NP			
Pos.	122	obs:	exp:
tct	S	0	0.32
tcc	S	0	0.30
tca	S	0	0.54
tcg	S	0	0.08
tgt	C	0	0.39
tgc	C	1	0.61
cat	H	0	3.38
cac	H	6	2.62
cgt	R	381	853.20
cgc	R	42477	1548.00
cga	R	4	3926.00
cgg	R	168	3695.00
act	T	0	0.31
acc	T	1	0.18
aca	T	0	0.43
acg	T	0	0.08
agt	S	0	0.35
agc	S	2	0.41
aga	R	0	21106.00
agg	R	0	11902.00
---	--	------	------
mPD		0.026	0.89
		nPD:	0.03
		N. weight:	3.4
		Sc. PD:	-0.084
		Sc. rank:	26520.0

,

NP			
Pos.	123	obs:	exp:
tta	L	0	0.12
ttg	L	0	0.21
ctt	L	0	0.21
ctc	L	0	0.15
cta	L	1	0.15
ctg	L	0	0.17
cat	H	1	0.56
cac	H	0	0.44
caa	Q	42957	26308.00
cag	Q	81	16730.00
---	--	------	------
mPD		0.0038	0.48
		nPD:	0.01
		N. weight:	0.5
		Sc. PD:	-0.023
		Sc. rank:	-9330.4

,

NP			
Pos.	124	obs:	exp:
tct	S	0	0.65
tcc	S	4	0.60
tca	S	0	1.08
tcg	S	0	0.16
agt	S	0	0.70
agc	S	0	0.83
gct	A	186	11730.00
gcc	A	42714	10752.00
gca	A	136	17408.00
gcg	A	0	3146.00
---	--	------	------
mPD		0.015	0.69
		nPD:	0.02
		N. weight:	1.4
		Sc. PD:	-0.045
		Sc. rank:	518.2

,

NP			
Pos.	125	obs:	exp:
cat	H	0	1.13
cac	H	2	0.87
aat	N	303	24078.00
aac	N	42735	18960.00
---	--	------	------
mPD		0.014	0.49
		nPD:	0.03
		N. weight:	0.81
		Sc. PD:	-0.021
		Sc. rank:	5354.3

,

NP			
Pos.	126	obs:	exp:
tct	S	0	1.61
tcc	S	0	1.49
tca	S	0	2.69
tcg	S	0	0.39
att	I	1	0.35
atc	I	0	0.26
ata	I	0	0.39
aat	N	43003	24072.00
aac	N	24	18955.00
aaa	K	1	0.66
aag	K	0	0.34
agt	S	10	1.75
agc	S	0	2.06
gat	D	1	0.57
gac	D	0	0.43
---	--	------	------
mPD		0.0017	0.49
		nPD:	0.
		N. weight:	0.6
		Sc. PD:	-0.03
		Sc. rank:	-16641.0

,

NP			
Pos.	127	obs:	exp:
tct	S	0	1.61
tcc	S	0	1.49
tca	S	0	2.69
tcg	S	0	0.39
agt	S	10	1.75
agc	S	0	2.06
ggt	G	42999	5793.00
ggc	G	26	6006.00
gga	G	4	19863.00
ggg	G	1	11368.00
---	--	------	------
mPD		0.0019	0.68
		nPD:	0.
		N. weight:	2.1
		Sc. PD:	-0.1
		Sc. rank:	-61352.0

,

NP			
Pos.	128	obs:	exp:
aat	N	1	0.56
aac	N	0	0.44
aaa	K	0	0.66
aag	K	1	0.34
gtt	V	0	1.50
gtc	V	0	1.22
gta	V	0	1.30
gtg	V	6	1.97
gat	D	251	145.30
gac	D	6	111.70
gaa	E	953	25214.00
gag	E	41815	17554.00
ggt	G	0	0.94
ggc	G	0	0.98
gga	G	1	3.23
ggg	G	6	1.85
---	--	------	------
mPD		0.056	0.49
		nPD:	0.11
		N. weight:	0.8
		Sc. PD:	0.045
		Sc. rank:	21946.0

,

NP			
Pos.	129	obs:	exp:
tat	Y	1	0.53
tac	Y	0	0.47
aat	N	9	5.04
aac	N	0	3.96
gat	D	42941	24288.00
gac	D	27	18680.00
gaa	E	34	32.43
gag	E	21	22.57
ggt	G	7	0.94
ggc	G	0	0.98
gga	G	0	3.23
ggg	G	0	1.85
---	--	------	------
mPD		0.0046	0.49
		nPD:	0.01
		N. weight:	0.59
		Sc. PD:	-0.026
		Sc. rank:	-9987.2

,

NP			
Pos.	130	obs:	exp:
tct	S	38	6.45
tcc	S	2	5.98
tca	S	0	10.75
tcg	S	0	1.57
act	T	1	0.31
acc	T	0	0.18
aca	T	0	0.43
acg	T	0	0.08
agt	S	0	6.99
agc	S	0	8.25
gct	A	41930	11720.00
gcc	A	205	10743.00
gca	A	193	17393.00
gcg	A	671	3143.00
---	--	------	------
mPD		0.051	0.70
		nPD:	0.07
		N. weight:	1.2
		Sc. PD:	0.021
		Sc. rank:	27539.0

,

NP			
Pos.	131	obs:	exp:
act	T	16	13362.00
acc	T	16	7607.00
aca	T	42929	18622.00
acg	T	67	3437.00
aaa	K	2	1.97
aag	K	1	1.03
gct	A	0	2.45
gcc	A	0	2.25
gca	A	9	3.64
gcg	A	0	0.66
---	--	------	------
mPD		0.0052	0.68
		nPD:	0.01
		N. weight:	0.85
		Sc. PD:	-0.039
		Sc. rank:	-16344.0

,

NP			
Pos.	132	obs:	exp:
tct	S	42011	6792.00
tcc	S	40	6290.00
tca	S	35	11313.00
tcg	S	4	1657.00
tat	Y	5	2.64
tac	Y	0	2.36
cct	P	20	6.66
ccc	P	0	3.75
cca	P	0	6.76
ccg	P	0	2.83
act	T	4	1.24
acc	T	0	0.71
aca	T	0	1.73
acg	T	0	0.32
agt	S	0	7355.00
agc	S	0	8683.00
gct	A	910	251.00
gcc	A	1	230.10
gca	A	10	372.50
gcg	A	0	67.33
---	--	------	------
mPD		0.047	1.6
		nPD:	0.03
		N. weight:	1.9
		Sc. PD:	-0.048
		Sc. rank:	13350.0

,

NP			
Pos.	133	obs:	exp:
tct	S	0	0.16
tcc	S	0	0.15
tca	S	0	0.27
tcg	S	0	0.04
agt	S	1	0.17
agc	S	0	0.21
ggt	G	42765	5794.00
ggc	G	262	6007.00
gga	G	5	19868.00
ggg	G	7	11370.00
---	--	------	------
mPD		0.013	0.68
		nPD:	0.02
		N. weight:	2.
		Sc. PD:	-0.072
		Sc. rank:	-5510.3

,

NP			
Pos.	134	obs:	exp:
tta	L	169	4994.00
ttg	L	1	8898.00
ctt	L	124	9003.00
ctc	L	11	6311.00
cta	L	42655	6609.00
ctg	L	78	7223.00
att	I	0	0.70
atc	I	0	0.53
ata	I	2	0.77
---	--	------	------
mPD		0.018	1.2
		nPD:	0.02
		N. weight:	1.9
		Sc. PD:	-0.072
		Sc. rank:	-12896.0

,

NP			
Pos.	135	obs:	exp:
att	I	1	0.70
atc	I	1	0.53
ata	I	0	0.77
act	T	24708	13364.00
acc	T	18317	7608.00
aca	T	6	18624.00
acg	T	2	3437.00
gct	A	5	1.36
gcc	A	0	1.25
gca	A	0	2.02
gcg	A	0	0.37
---	--	------	------
mPD		0.49	0.68
		nPD:	0.72
		N. weight:	0.75
		Sc. PD:	0.48
		Sc. rank:	26966.0

,

NP			
Pos.	136	obs:	exp:
cat	H	204	24276.00
cac	H	42836	18764.00
---	--	------	------
mPD		0.0094	0.49
		nPD:	0.02
		N. weight:	0.83
		Sc. PD:	-0.029
		Sc. rank:	-1614.4

,

NP			
Pos.	137	obs:	exp:
tta	L	21	57.09
ttg	L	1	101.70
ctt	L	0	102.90
ctc	L	0	72.15
cta	L	465	75.55
ctg	L	5	82.57
att	I	49	11810.00
atc	I	114	8840.00
ata	I	33481	12993.00
atg	M	8901	8901.00
act	T	0	0.31
acc	T	0	0.18
aca	T	0	0.43
acg	T	1	0.08
gtt	V	0	0.50
gtc	V	0	0.41
gta	V	1	0.43
gtg	V	1	0.66
---	--	------	------
mPD		0.36	0.77
		nPD:	0.47
		N. weight:	0.76
		Sc. PD:	0.3
		Sc. rank:	26469.0

,

NP			
Pos.	138	obs:	exp:
atg	M	43040	43040.00
---	--	------	------
mPD		0	0
		nPD:	1.
		N. weight:	0.
		Sc. PD:	0
		Sc. rank:	0

,

NP			
Pos.	139	obs:	exp:
att	I	41790	15108.00
atc	I	1242	11309.00
ata	I	7	16622.00
gtt	V	1	0.25
gtc	V	0	0.20
gta	V	0	0.22
gtg	V	0	0.33
---	--	------	------
mPD		0.056	0.66
		nPD:	0.09
		N. weight:	0.96
		Sc. PD:	0.028
		Sc. rank:	22831.0

,

NP			
Pos.	140	obs:	exp:
tgg	W	43040	43040.00
---	--	------	------
mPD		0	0
		nPD:	1.
		N. weight:	0.
		Sc. PD:	0
		Sc. rank:	0

,

NP			
Pos.	141	obs:	exp:
cat	H	42823	24276.00
cac	H	217	18764.00
---	--	------	------
mPD		0.010	0.49
		nPD:	0.02
		N. weight:	0.56
		Sc. PD:	-0.019
		Sc. rank:	-389.7

,

NP			
Pos.	142	obs:	exp:
tct	S	37	6945.00
tcc	S	42891	6432.00
tca	S	112	11568.00
tcg	S	0	1694.00
agt	S	0	7521.00
agc	S	0	8879.00
---	--	------	------
mPD		0.0069	1.6
		nPD:	0.
		N. weight:	1.9
		Sc. PD:	-0.096
		Sc. rank:	-51485.0

,

NP			
Pos.	143	obs:	exp:
aat	N	42913	24080.00
aac	N	127	18960.00
---	--	------	------
mPD		0.0059	0.49
		nPD:	0.01
		N. weight:	0.58
		Sc. PD:	-0.024
		Sc. rank:	-7848.2

,

NP			
Pos.	144	obs:	exp:
tta	L	144	4994.00
ttg	L	41385	8898.00
ctt	L	0	9004.00
ctc	L	0	6311.00
cta	L	3	6609.00
ctg	L	1508	7223.00
---	--	------	------
mPD		0.075	1.2
		nPD:	0.06
		N. weight:	1.5
		Sc. PD:	0.013
		Sc. rank:	31157.0

,

NP

Pos.	145	obs:	exp:
aat	N	43021	24080.00
aac	N	19	18960.00
---	--	------	------
mPD		0.00088	0.49
		nPD:	0.
		N. weight:	0.6
		Sc. PD:	-0.031
		Sc. rank:	-19454.0

,

NP

Pos.	146	obs:	exp:
gat	D	42828	24328.00
gac	D	211	18711.00
ggt	G	1	0.13
ggc	G	0	0.14
gga	G	0	0.46
ggg	G	0	0.26
---	--	------	------
mPD		0.0098	0.49
		nPD:	0.02
		N. weight:	0.56
		Sc. PD:	-0.019
		Sc. rank:	-682.7

,

NP

Pos.	147	obs:	exp:
tct	S	0	1.13
tcc	S	0	1.05
tca	S	7	1.88
tcg	S	0	0.28
act	T	0	110.20
acc	T	0	62.76
aca	T	354	153.60
acg	T	1	28.35
agt	S	0	1.22
agc	S	0	1.44
gtt	V	0	5.26
gtc	V	0	4.28
gta	V	18	4.55
gtg	V	3	6.91
gct	A	12	11626.00
gcc	A	123	10657.00
gca	A	33401	17255.00
gcg	A	9121	3118.00
---	--	------	------
mPD		0.36	0.71
		nPD:	0.5
		N. weight:	0.76
		Sc. PD:	0.33
		Sc. rank:	26526.0

,

NP

Pos.	148	obs:	exp:
att	I	0	0.70
atc	I	0	0.53
ata	I	2	0.77
act	T	230	13366.00
acc	T	44	7609.00
aca	T	42634	18626.00
acg	T	130	3437.00
---	--	------	------
mPD		0.019	0.68
		nPD:	0.03
		N. weight:	0.81
		Sc. PD:	-0.022
		Sc. rank:	4539.3

,

NP

Pos.	149	obs:	exp:
tat	Y	98	22687.00
tac	Y	42942	20353.00
---	--	------	------
mPD		0.0045	0.50
		nPD:	0.01
		N. weight:	0.76
		Sc. PD:	-0.034
		Sc. rank:	-13014.0

,

NP

Pos.	150	obs:	exp:
cat	H	0	0.56
cac	H	1	0.44
caa	Q	643	26309.00
cag	Q	42396	16730.00
---	--	------	------
mPD		0.029	0.48
		nPD:	0.06
		N. weight:	0.89
		Sc. PD:	0.0059
		Sc. rank:	18426.0

,

NP

Pos.	151	obs:	exp:
cgt	R	0	853.40
cgc	R	0	1549.00
cga	R	2	3927.00
cgg	R	80	3696.00
aga	R	489	21110.00
agg	R	42469	11905.00
---	--	------	------
mPD		0.026	0.89
		nPD:	0.03
		N. weight:	1.2
		Sc. PD:	-0.031
		Sc. rank:	10723.0

,

NP

Pos.	152	obs:	exp:
act	T	3	13366.00
acc	T	0	7610.00
aca	T	43021	18627.00
acg	T	16	3438.00
---	--	------	------
mPD		0.00088	0.68
		nPD:	0.
		N. weight:	0.87
		Sc. PD:	-0.045
		Sc. rank:	-28922.0

,

NP

Pos.	153	obs:	exp:
cgt	R	0	853.40
cgc	R	0	1549.00
cga	R	0	3927.00
cgg	R	0	3696.00
aaa	K	1	0.66
aag	K	0	0.34
aga	R	42978	21110.00
agg	R	61	11905.00
---	--	------	------
mPD		0.0029	0.89
		nPD:	0.
		N. weight:	0.73
		Sc. PD:	-0.037
		Sc. rank:	-20717.0

,

NP

Pos.	154	obs:	exp:
tct	S	1	0.16
tcc	S	0	0.15
tca	S	0	0.27
tcg	S	0	0.04
agt	S	0	0.17
agc	S	0	0.21
gct	A	42827	11730.00
gcc	A	88	10753.00
gca	A	14	17409.00
gcg	A	110	3146.00
---	--	------	------
mPD		0.0099	0.69
		nPD:	0.01
		N. weight:	1.3
		Sc. PD:	-0.052
		Sc. rank:	-12196.0

,

NP

Pos.	155	obs:	exp:
tta	L	0	4994.00
ttg	L	0	8897.00
ctt	L	42958	9002.00
ctc	L	66	6310.00
cta	L	9	6608.00
ctg	L	0	7222.00
att	I	7	2.46
atc	I	0	1.84
ata	I	0	2.70
---	--	------	------
mPD		0.0038	1.2
		nPD:	0.
		N. weight:	1.6
		Sc. PD:	-0.081
		Sc. rank:	-45386.0

,

NP

Pos.	156	obs:	exp:
gtt	V	42955	10781.00
gtc	V	77	8770.00
gta	V	8	9329.00
gtg	V	0	14161.00
---	--	------	------
mPD		0.0039	0.74
		nPD:	0.01
		N. weight:	1.4
		Sc. PD:	-0.068
		Sc. rank:	-32369.0

,

NP

Pos.	157	obs:	exp:
cgt	R	4	853.40
cgc	R	418	1549.00
cga	R	12871	3927.00
cgg	R	28298	3696.00
aga	R	74	21110.00
agg	R	1375	11905.00
---	--	------	------
mPD		0.50	0.89
		nPD:	0.56
		N. weight:	1.7
		Sc. PD:	0.82
		Sc. rank:	59090.0

,

NP

Pos.	158	obs:	exp:
tct	S	0	0.16
tcc	S	0	0.15
tca	S	0	0.27
tcg	S	0	0.04
att	I	1	0.35
atc	I	0	0.26
ata	I	0	0.39
act	T	42318	13357.00
acc	T	645	7605.00
aca	T	27	18615.00
acg	T	22	3435.00
agt	S	1	0.17
agc	S	0	0.21
gct	A	26	7.09
gcc	A	0	6.50
gca	A	0	10.52
gcg	A	0	1.90
---	--	------	------
mPD		0.033	0.68
		nPD:	0.05
		N. weight:	1.1
		Sc. PD:	-0.0072
		Sc. rank:	20101.0

,

NP

Pos.	159	obs:	exp:
ggt	G	1	5794.00
ggc	G	30	6007.00
gga	G	42730	19868.00
ggg	G	279	11371.00
---	--	------	------
mPD		0.014	0.68
		nPD:	0.02
		N. weight:	0.76
		Sc. PD:	-0.025
		Sc. rank:	-346.5

,

NP

Pos.	160	obs:	exp:
atg	M	43040	43040.00
---	--	------	------
mPD		0	0
		nPD:	1.
		N. weight:	0.
		Sc. PD:	0
		Sc. rank:	0

,

NP			
Pos.	161	obs:	exp:
gat	D	42814	24329.00
gac	D	226	18711.00
---	--	------	------
mPD		0.010	0.49
		nPD:	0.02
		N. weight:	0.56
		Sc. PD:	-0.018
		Sc. rank:	32.2

,

NP			
Pos.	162	obs:	exp:
cct	P	402	14343.00
ccc	P	42608	8069.00
cca	P	30	14541.00
ccg	P	0	6087.00
---	--	------	------
mPD		0.020	0.72
		nPD:	0.03
		N. weight:	1.7
		Sc. PD:	-0.044
		Sc. rank:	9724.7

,

NP			
Pos.	163	obs:	exp:
cgt	R	0	853.30
cgc	R	0	1549.00
cga	R	4	3927.00
cgg	R	1	3695.00
aaa	K	3	1.97
aag	K	0	1.03
aga	R	42613	21109.00
agg	R	419	11904.00
---	--	------	------
mPD		0.020	0.89
		nPD:	0.02
		N. weight:	0.69
		Sc. PD:	-0.022
		Sc. rank:	716.0

,

NP			
Pos.	164	obs:	exp:
tta	L	0	0.23
ttg	L	0	0.41
ctt	L	0	0.42
ctc	L	0	0.29
cta	L	0	0.31
ctg	L	2	0.34
atg	M	43038	43038.00
---	--	------	------
mPD		0.000093	0.00015
		nPD:	0.62
		N. weight:	0.000086
		Sc. PD:	0.000047
		Sc. rank:	3.1

,

NP			
Pos.	165	obs:	exp:
tgt	C	83	16820.00
tgc	C	42957	26220.00
---	--	------	------
mPD		0.0038	0.48
		nPD:	0.01
		N. weight:	0.5
		Sc. PD:	-0.023
		Sc. rank:	-9425.9

,

NP			
Pos.	166	obs:	exp:
tct	S	42897	6945.00
tcc	S	111	6432.00
tca	S	14	11568.00
tcg	S	18	1694.00
agt	S	0	7521.00
agc	S	0	8879.00
---	--	------	------
mPD		0.0066	1.6
		nPD:	0.
		N. weight:	1.9
		Sc. PD:	-0.092
		Sc. rank:	-50365.0

,

NP			
Pos.	167	obs:	exp:
tta	L	0	4994.00
ttg	L	27	8898.00
ctt	L	12	9003.00
ctc	L	0	6311.00
cta	L	173	6609.00
ctg	L	42827	7223.00
atg	M	1	1.00
---	--	------	------
mPD		0.0099	1.2
		nPD:	0.01
		N. weight:	1.8
		Sc. PD:	-0.082
		Sc. rank:	-32741.0

,

NP			
Pos.	168	obs:	exp:
tta	L	0	0.12
ttg	L	0	0.21
ctt	L	0	0.21
ctc	L	0	0.15
cta	L	0	0.15
ctg	L	1	0.17
atg	M	43039	43039.00
---	--	------	------
mPD		0.000046	0.000076
		nPD:	0.62
		N. weight:	0.000043
		Sc. PD:	0.000023
		Sc. rank:	1.5

,

NP			
Pos.	169	obs:	exp:
tta	L	0	0.12
ttg	L	0	0.21
ctt	L	0	0.21
ctc	L	0	0.15
cta	L	0	0.15
ctg	L	1	0.17
caa	Q	317	26309.00
cag	Q	42722	16730.00
---	--	------	------
mPD		0.015	0.48
		nPD:	0.03
		N. weight:	0.93
		Sc. PD:	-0.022
		Sc. rank:	8799.3

,

NP			
Pos.	170	obs:	exp:
ggt	G	433	5794.00
ggc	G	33612	6007.00
gga	G	664	19868.00
ggg	G	8331	11371.00
---	--	------	------
mPD		0.35	0.68
		nPD:	0.52
		N. weight:	1.3
		Sc. PD:	0.56
		Sc. rank:	43906.0

,

NP			
Pos.	171	obs:	exp:
tct	S	49	6945.00
tcc	S	0	6432.00
tca	S	499	11568.00
tcg	S	42492	1694.00
agt	S	0	7521.00
agc	S	0	8879.00
---	--	------	------
mPD		0.025	1.6
		nPD:	0.02
		N. weight:	3.3
		Sc. PD:	-0.13
		Sc. rank:	-20808.0

,

NP			
Pos.	172	obs:	exp:
cct	P	1	0.33
ccc	P	0	0.19
cca	P	0	0.34
ccg	P	0	0.14
act	T	42685	13366.00
acc	T	214	7609.00
aca	T	129	18626.00
acg	T	11	3437.00
---	--	------	------
mPD		0.016	0.68
		nPD:	0.02
		N. weight:	1.2
		Sc. PD:	-0.035
		Sc. rank:	3406.5

,

NP			
Pos.	173	obs:	exp:
tta	L	0	4994.00
ttg	L	0	8898.00
ctt	L	202	9003.00
ctc	L	42813	6311.00
cta	L	23	6609.00
ctg	L	0	7223.00
att	I	0	0.70
atc	I	2	0.53
ata	I	0	0.77
---	--	------	------
mPD		0.010	1.2
		nPD:	0.01
		N. weight:	2.
		Sc. PD:	-0.087
		Sc. rank:	-33676.0

,

NP			
Pos.	174	obs:	exp:
tct	S	1	0.16
tcc	S	0	0.15
tca	S	0	0.27
tcg	S	0	0.04
cct	P	39324	14342.00
ccc	P	797	8069.00
cca	P	1755	14541.00
ccg	P	1163	6087.00
agt	S	0	0.17
agc	S	0	0.21
---	--	------	------
mPD		0.16	0.72
		nPD:	0.23
		N. weight:	0.78
		Sc. PD:	0.13
		Sc. rank:	24765.0

,

NP			
Pos.	175	obs:	exp:
cgt	R	0	853.40
cgc	R	0	1549.00
cga	R	3	3927.00
cgg	R	0	3696.00
aga	R	42904	21110.00
agg	R	133	11905.00
---	--	------	------
mPD		0.0063	0.89
		nPD:	0.01
		N. weight:	0.72
		Sc. PD:	-0.033
		Sc. rank:	-14054.0

,

NP			
Pos.	176	obs:	exp:
cgt	R	0	853.10
cgc	R	0	1548.00
cga	R	0	3926.00
cgg	R	3	3695.00
aaa	K	0	8.55
aag	K	13	4.45
aga	R	418	21104.00
agg	R	42606	11901.00
---	--	------	------
mPD		0.020	0.89
		nPD:	0.02
		N. weight:	1.3
		Sc. PD:	-0.04
		Sc. rank:	1821.0

,

NP			
Pos.	177	obs:	exp:
tct	S	620	6941.00
tcc	S	36425	6428.00
tca	S	5941	11561.00
tcg	S	28	1693.00
agt	S	0	7517.00
agc	S	0	8874.00
gct	A	0	7.09
gcc	A	26	6.50
gca	A	0	10.52
gcg	A	0	1.90
---	--	------	------
mPD		0.26	1.6
		nPD:	0.16
		N. weight:	1.4
		Sc. PD:	0.14
		Sc. rank:	42572.0

,

NP			
Pos.	178	obs:	exp:
ggt	G	22	5794.00
ggc	G	0	6007.00
gga	G	36297	19868.00
ggg	G	6721	11371.00
---	--	------	------
mPD		0.26	0.68
		nPD:	0.39
		N. weight:	0.44
		Sc. PD:	0.14
		Sc. rank:	14846.0

,

NP			
Pos.	179	obs:	exp:
tct	S	3	0.48
tcc	S	0	0.45
tca	S	0	0.81
tcg	S	0	0.12
act	T	3	0.93
acc	T	0	0.53
aca	T	0	1.30
acg	T	0	0.24
agt	S	0	0.52
agc	S	0	0.62
gct	A	42773	11729.00
gcc	A	177	10752.00
gca	A	56	17407.00
gcg	A	28	3146.00
---	--	------	------
mPD		0.012	0.69
		nPD:	0.02
		N. weight:	1.3
		Sc. PD:	-0.047
		Sc. rank:	-5298.7

,

NP			
Pos.	180	obs:	exp:
tct	S	0	0.32
tcc	S	0	0.30
tca	S	2	0.54
tcg	S	0	0.08
agt	S	0	0.35
agc	S	0	0.41
gct	A	133	11730.00
gcc	A	2211	10753.00
gca	A	40681	17409.00
gcg	A	13	3146.00
---	--	------	------
mPD		0.10	0.69
		nPD:	0.15
		N. weight:	0.73
		Sc. PD:	0.067
		Sc. rank:	21532.0

,

NP

Pos.	181	obs:	exp:
cgt	R	1	0.02
cgc	R	0	0.04
cga	R	0	0.09
cgg	R	0	0.09
aga	R	0	0.49
agg	R	0	0.28
ggt	G	42293	5794.00
ggc	G	727	6007.00
gga	G	5	19868.00
ggg	G	14	11370.00
---	--	------	------
mPD		0.034	0.68
		nPD:	0.05
		N. weight:	2.
		Sc. PD:	-0.0095
		Sc. rank:	36555.0

,

NP

Pos.	182	obs:	exp:
tct	S	16	2.58
tcc	S	0	2.39
tca	S	0	4.30
tcg	S	0	0.63
act	T	8	2.48
acc	T	0	1.41
aca	T	0	3.46
acg	T	0	0.64
agt	S	0	2.80
agc	S	0	3.30
gct	A	42923	11724.00
gcc	A	54	10747.00
gca	A	25	17400.00
gcg	A	14	3145.00
---	--	------	------
mPD		0.0054	0.70
		nPD:	0.01
		N. weight:	1.3
		Sc. PD:	-0.061
		Sc. rank:	-25240.0

,

NP

Pos.	183	obs:	exp:
tct	S	0	2.26
tcc	S	0	2.09
tca	S	14	3.76
tcg	S	0	0.55
agt	S	0	2.45
agc	S	0	2.89
gct	A	11	11727.00
gcc	A	10	10750.00
gca	A	42982	17404.00
gcg	A	23	3145.00
---	--	------	------
mPD		0.0027	0.70
		nPD:	0.
		N. weight:	0.93
		Sc. PD:	-0.046
		Sc. rank:	-25423.0

,

NP

Pos.	184	obs:	exp:
ttt	F	0	0.49
ttc	F	1	0.51
att	I	0	10.53
atc	I	30	7.88
ata	I	0	11.59
gtt	V	87	10773.00
gtc	V	42739	8763.00
gta	V	177	9322.00
gtg	V	5	14151.00
gct	A	0	0.27
gcc	A	1	0.25
gca	A	0	0.40
gcg	A	0	0.07
---	--	------	------
mPD		0.014	0.74
		nPD:	0.02
		N. weight:	1.6
		Sc. PD:	-0.056
		Sc. rank:	-3969.6

,

NP

Pos.	185	obs:	exp:
aat	N	1	0.56
aac	N	0	0.44
aaa	K	42990	28295.00
aag	K	49	14744.00
---	--	------	------
mPD		0.0023	0.45
		nPD:	0.01
		N. weight:	0.43
		Sc. PD:	-0.021
		Sc. rank:	-9988.8

,

NP

Pos.	186	obs:	exp:
ggt	G	27	5794.00
ggc	G	5	6007.00
gga	G	42900	19868.00
ggg	G	108	11371.00
---	--	------	------
mPD		0.0065	0.68
		nPD:	0.01
		N. weight:	0.78
		Sc. PD:	-0.034
		Sc. rank:	-13133.0

,

NP

Pos.	187	obs:	exp:
att	I	293	14941.00
atc	I	41583	11184.00
ata	I	687	16438.00
gtt	V	151	119.50
gtc	V	326	97.19
gta	V	0	103.40
gtg	V	0	156.90
---	--	------	------
mPD		0.073	0.68
		nPD:	0.11
		N. weight:	1.2
		Sc. PD:	0.063
		Sc. rank:	32675.0

,

NP

Pos.	188	obs:	exp:
ggt	G	9	5794.00
ggc	G	43	6007.00
gga	G	37717	19868.00
ggg	G	5271	11371.00
---	--	------	------
mPD		0.22	0.68
		nPD:	0.32
		N. weight:	0.48
		Sc. PD:	0.12
		Sc. rank:	15751.0

,

NP

Pos.	189	obs:	exp:
act	T	81	13366.00
acc	T	4	7610.00
aca	T	42910	18627.00
acg	T	45	3438.00
---	--	------	------
mPD		0.0060	0.68
		nPD:	0.01
		N. weight:	0.85
		Sc. PD:	-0.038
		Sc. rank:	-15048.0

,

NP

Pos.	190	obs:	exp:
tta	L	0	0.12
ttg	L	1	0.21
ctt	L	0	0.21
ctc	L	0	0.15
cta	L	0	0.15
ctg	L	0	0.17
att	I	1	57.22
atc	I	0	42.83
ata	I	162	62.95
atg	M	42873	42873.00
gtt	V	0	0.75
gtc	V	0	0.61
gta	V	0	0.65
gtg	V	3	0.99
---	--	------	------
mPD		0.0077	0.0079
		nPD:	0.98
		N. weight:	0.0037
		Sc. PD:	0.0034
		Sc. rank:	133.8

,

NP

Pos.	191	obs:	exp:
att	I	0	0.35
atc	I	0	0.26
ata	I	1	0.39
gtt	V	17	10751.00
gtc	V	0	8746.00
gta	V	223	9303.00
gtg	V	42682	14122.00
gct	A	5	31.89
gcc	A	0	29.23
gca	A	110	47.33
gcg	A	2	8.55
---	--	------	------
mPD		0.022	0.75
		nPD:	0.03
		N. weight:	1.1
		Sc. PD:	-0.028
		Sc. rank:	9215.2

,

NP

Pos.	192	obs:	exp:
tta	L	0	0.12
ttg	L	0	0.21
ctt	L	0	0.21
ctc	L	0	0.15
cta	L	0	0.15
ctg	L	1	0.17
cgt	R	0	0.02
cgc	R	0	0.04
cga	R	0	0.09
cgg	R	0	0.09
att	I	0	2.46
atc	I	0	1.84
ata	I	7	2.70
atg	M	43030	43030.00
aga	R	0	0.49
agg	R	1	0.28
gtt	V	0	0.25
gtc	V	0	0.20
gta	V	0	0.22
gtg	V	1	0.33
---	--	------	------
mPD		0.00046	0.00057
		nPD:	0.82
		N. weight:	0.00026
		Sc. PD:	0.00019
		Sc. rank:	9.4

,

NP

Pos.	193	obs:	exp:
caa	Q	2	1.22
cag	Q	0	0.78
gat	D	0	0.57
gac	D	1	0.43
gaa	E	42400	25373.00
gag	E	637	17664.00
---	--	------	------
mPD		0.029	0.48
		nPD:	0.06
		N. weight:	0.47
		Sc. PD:	0.0025
		Sc. rank:	9536.9

,

NP

Pos.	194	obs:	exp:
tta	L	10	4994.00
ttg	L	210	8898.00
ctt	L	2565	9003.00
ctc	L	0	6311.00
cta	L	548	6609.00
ctg	L	39705	7223.00
att	I	1	0.35
atc	I	0	0.26
ata	I	0	0.39
gtt	V	0	0.25
gtc	V	0	0.20
gta	V	0	0.22
gtg	V	1	0.33
---	--	------	------
mPD		0.15	1.2
		nPD:	0.13
		N. weight:	1.5
		Sc. PD:	0.1
		Sc. rank:	41329.0

,

NP

Pos.	195	obs:	exp:
att	I	13	15104.00
atc	I	42959	11305.00
ata	I	53	16616.00
act	T	0	0.31
acc	T	1	0.18
aca	T	0	0.43
acg	T	0	0.08
gtt	V	0	3.51
gtc	V	14	2.85
gta	V	0	3.03
gtg	V	0	4.61
---	--	------	------
mPD		0.0038	0.66
		nPD:	0.01
		N. weight:	1.4
		Sc. PD:	-0.066
		Sc. rank:	-29702.0

,

NP

Pos.	196	obs:	exp:
cgt	R	0	853.40
cgc	R	0	1549.00
cga	R	1	3927.00
cgg	R	0	3696.00
aaa	K	1	0.66
aag	K	0	0.34
aga	R	35121	21110.00
agg	R	7917	11905.00
---	--	------	------
mPD		0.30	0.89
		nPD:	0.34
		N. weight:	0.35
		Sc. PD:	0.097
		Sc. rank:	11633.0

,

NP			
Pos.	197	obs:	exp:
tta	L	0	0.12
ttg	L	0	0.21
ctt	L	0	0.21
ctc	L	0	0.15
cta	L	0	0.15
ctg	L	1	0.17
att	I	1	0.35
atc	I	0	0.26
ata	I	0	0.39
atg	M	43038	43038.00
---	--	------	------
mPD		0.000093	0.00012
		nPD:	0.76
		N. weight:	0.000068
		Sc. PD:	0.000047
		Sc. rank:	2.4

,

NP			
Pos.	198	obs:	exp:
att	I	22	8616.00
atc	I	24456	6449.00
ata	I	67	9479.00
act	T	0	0.62
acc	T	2	0.35
aca	T	0	0.87
acg	T	0	0.16
gtt	V	9	4632.00
gtc	V	18468	3768.00
gta	V	14	4008.00
gtg	V	1	6084.00
gct	A	0	0.27
gcc	A	1	0.25
gca	A	0	0.40
gcg	A	0	0.07
---	--	------	------
mPD		0.50	1.2
		nPD:	0.41
		N. weight:	1.5
		Sc. PD:	0.5
		Sc. rank:	50736.0

,

NP			
Pos.	199	obs:	exp:
cgt	R	0	0.12
cgc	R	0	0.22
cga	R	0	0.55
cgg	R	0	0.52
aaa	K	42649	28291.00
aag	K	384	14742.00
aga	R	6	2.94
agg	R	0	1.66
gaa	E	1	0.59
gag	E	0	0.41
---	--	------	------
mPD		0.018	0.45
		nPD:	0.04
		N. weight:	0.39
		Sc. PD:	-0.0057
		Sc. rank:	5647.3

,

NP			
Pos.	200	obs:	exp:
cgt	R	192	853.40
cgc	R	0	1549.00
cga	R	358	3927.00
cgg	R	42409	3696.00
aga	R	2	21110.00
agg	R	79	11905.00
---	--	------	------
mPD		0.029	0.89
		nPD:	0.03
		N. weight:	2.5
		Sc. PD:	-0.053
		Sc. rank:	25640.0

,

NP			
Pos.	201	obs:	exp:
ggt	G	10	5794.00
ggc	G	0	6007.00
gga	G	286	19868.00
ggg	G	42744	11371.00
---	--	------	------
mPD		0.014	0.68
		nPD:	0.02
		N. weight:	1.3
		Sc. PD:	-0.045
		Sc. rank:	-1109.8

,

NP			
Pos.	202	obs:	exp:
att	I	31	15104.00
atc	I	42956	11306.00
ata	I	39	16617.00
act	T	0	0.62
acc	T	2	0.35
aca	T	0	0.87
acg	T	0	0.16
aat	N	0	0.56
aac	N	1	0.44
gtt	V	0	2.76
gtc	V	11	2.24
gta	V	0	2.38
gtg	V	0	3.62
---	--	------	------
mPD		0.0039	0.66
		nPD:	0.01
		N. weight:	1.4
		Sc. PD:	-0.065
		Sc. rank:	-28773.0

,

NP			
Pos.	203	obs:	exp:
att	I	0	0.35
atc	I	1	0.26
ata	I	0	0.39
aat	N	474	24079.00
aac	N	42565	18960.00
---	--	------	------
mPD		0.022	0.49
		nPD:	0.04
		N. weight:	0.78
		Sc. PD:	-0.0083
		Sc. rank:	12429.0

,

NP			
Pos.	204	obs:	exp:
aat	N	1	0.56
aac	N	0	0.44
gat	D	42671	24328.00
gac	D	368	18711.00
---	--	------	------
mPD		0.017	0.49
		nPD:	0.03
		N. weight:	0.54
		Sc. PD:	-0.011
		Sc. rank:	6553.6

,

NP			
Pos.	205	obs:	exp:
cgt	R	46	853.40
cgc	R	11	1549.00
cga	R	42394	3927.00
cgg	R	542	3696.00
aga	R	47	21110.00
agg	R	0	11905.00
---	--	------	------
mPD		0.030	0.89
		nPD:	0.03
		N. weight:	2.4
		Sc. PD:	-0.05
		Sc. rank:	26752.0

,

NP			
Pos.	206	obs:	exp:
tct	S	0	0.65
tcc	S	0	0.60
tca	S	0	1.08
tcg	S	0	0.16
aat	N	42141	24077.00
aac	N	895	18959.00
agt	S	4	0.70
agc	S	0	0.83
---	--	------	------
mPD		0.041	0.49
		nPD:	0.08
		N. weight:	0.5
		Sc. PD:	0.013
		Sc. rank:	11865.0

,

NP			
Pos.	207	obs:	exp:
ttt	F	42026	20936.00
ttc	F	1014	22104.00
---	--	------	------
mPD		0.046	0.50
		nPD:	0.09
		N. weight:	0.63
		Sc. PD:	0.022
		Sc. rank:	15530.0

,

NP			
Pos.	208	obs:	exp:
tgg	W	43040	43040.00
---	--	------	------
mPD		0	0
		nPD:	1.
		N. weight:	0.
		Sc. PD:	0
		Sc. rank:	0

,

NP			
Pos.	209	obs:	exp:
cgt	R	0	853.20
cgc	R	0	1548.00
cga	R	2	3926.00
cgg	R	1	3695.00
aaa	K	10	6.57
aag	K	0	3.43
aga	R	40781	21106.00
agg	R	2246	11902.00
---	--	------	------
mPD		0.10	0.89
		nPD:	0.11
		N. weight:	0.56
		Sc. PD:	0.031
		Sc. rank:	15074.0

,

NP			
Pos.	210	obs:	exp:
ggt	G	42948	5794.00
ggc	G	54	6007.00
gga	G	11	19868.00
ggg	G	27	11371.00
---	--	------	------
mPD		0.0043	0.68
		nPD:	0.01
		N. weight:	2.1
		Sc. PD:	-0.097
		Sc. rank:	-41449.0

,

NP			
Pos.	211	obs:	exp:
gat	D	14	7.91
gac	D	0	6.09
gaa	E	395	25367.00
gag	E	42631	17659.00
---	--	------	------
mPD		0.019	0.48
		nPD:	0.04
		N. weight:	0.87
		Sc. PD:	-0.014
		Sc. rank:	12455.0

,

NP			
Pos.	212	obs:	exp:
tct	S	0	1.61
tcc	S	0	1.49
tca	S	0	2.69
tcg	S	0	0.39
act	T	6	1.86
acc	T	0	1.06
aca	T	0	2.60
acg	T	0	0.48
aat	N	42925	24070.00
aac	N	98	18953.00
agt	S	9	1.75
agc	S	1	2.06
gat	D	1	0.57
gac	D	0	0.43
---	--	------	------
mPD		0.0054	0.49
		nPD:	0.01
		N. weight:	0.59
		Sc. PD:	-0.025
		Sc. rank:	-8782.8

,

NP			
Pos.	213	obs:	exp:
ggt	G	3	5794.00
ggc	G	78	6007.00
gga	G	917	19868.00
ggg	G	42042	11371.00
---	--	------	------
mPD		0.045	0.68
		nPD:	0.07
		N. weight:	1.2
		Sc. PD:	0.014
		Sc. rank:	27291.0

NP			
Pos.	214	obs:	exp:
cgt	R	5	853.40
cgc	R	4	1549.00
cga	R	327	3927.00
cgg	R	42338	3696.00
aga	R	2	21110.00
agg	R	364	11905.00
---	--	------	------
mPD		0.032	0.89
		nPD:	0.04
		N. weight:	2.4
		Sc. PD:	-0.044
		Sc. rank:	33366.0

NP			
Pos.	215	obs:	exp:
cgt	R	0	2.82
cgc	R	0	5.11
cga	R	0	12.96
cgg	R	0	12.19
aaa	K	42748	28203.00
aag	K	150	14695.00
aga	R	132	69.65
agg	R	10	39.28
---	--	------	------
mPD		0.014	0.46
		nPD:	0.03
		N. weight:	0.41
		Sc. PD:	-0.0099
		Sc. rank:	3791.0

NP			
Pos.	216	obs:	exp:
act	T	5	13366.00
acc	T	0	7610.00
aca	T	43006	18627.00
acg	T	29	3438.00
---	--	------	------
mPD		0.0016	0.68
		nPD:	0.
		N. weight:	0.86
		Sc. PD:	-0.044
		Sc. rank:	-27225.0

NP			
Pos.	217	obs:	exp:
cgt	R	0	853.00
cgc	R	0	1548.00
cga	R	4	3925.00
cgg	R	0	3694.00
aaa	K	19	12.49
aag	K	0	6.51
aga	R	42533	21101.00
agg	R	484	11900.00
---	--	------	------
mPD		0.023	0.89
		nPD:	0.03
		N. weight:	0.68
		Sc. PD:	-0.019
		Sc. rank:	3453.9

NP			
Pos.	218	obs:	exp:
tct	S	0	6844.00
tcc	S	0	6338.00
tca	S	0	11399.00
tcg	S	0	1669.00
cgt	R	0	0.02
cgc	R	0	0.04
cga	R	0	0.09
cgg	R	0	0.09
att	I	134	47.04
atc	I	0	35.21
ata	I	0	51.75
aat	N	78	44.76
aac	N	2	35.24
agt	S	41910	7412.00
agc	S	502	8749.00
aga	R	0	0.49
agg	R	1	0.28
gtt	V	10	2.50
gtc	V	0	2.04
gta	V	0	2.17
gtg	V	0	3.29
ggt	G	403	54.25
ggc	G	0	56.25
gga	G	0	186.00
ggg	G	0	106.50
---	--	------	------
mPD		0.053	1.7
		nPD:	0.03
		N. weight:	1.7
		Sc. PD:	-0.039
		Sc. rank:	17370.0

NP			
Pos.	219	obs:	exp:
gct	A	42877	11731.00
gcc	A	50	10753.00
gca	A	109	17410.00
gcg	A	4	3146.00
---	--	------	------
mPD		0.0076	0.69
		nPD:	0.01
		N. weight:	1.3
		Sc. PD:	-0.056
		Sc. rank:	-19860.0

NP			
Pos.	220	obs:	exp:
ttt	F	6	2.92
ttc	F	0	3.08
tat	Y	42975	22684.00
tac	Y	59	20350.00
---	--	------	------
mPD		0.0030	0.50
		nPD:	0.01
		N. weight:	0.65
		Sc. PD:	-0.031
		Sc. rank:	-13540.0

NP			
Pos.	221	obs:	exp:
gat	D	8384	4741.00
gac	D	4	3647.00
gaa	E	108	20430.00
gag	E	34544	14222.00
---	--	------	------
mPD		0.32	0.65
		nPD:	0.49
		N. weight:	0.84
		Sc. PD:	0.35
		Sc. rank:	29152.0

NP			
Pos.	222	obs:	exp:
cgt	R	0	853.40
cgc	R	0	1549.00
cga	R	5	3927.00
cgg	R	0	3696.00
aga	R	42916	21110.00
agg	R	119	11905.00
---	--	------	------
mPD		0.0057	0.89
		nPD:	0.01
		N. weight:	0.72
		Sc. PD:	-0.034
		Sc. rank:	-14339.0

NP			
Pos.	223	obs:	exp:
tta	L	0	0.12
ttg	L	0	0.21
ctt	L	0	0.21
ctc	L	0	0.15
cta	L	0	0.15
ctg	L	1	0.17
att	I	27	11.58
atc	I	0	8.67
ata	I	6	12.74
atg	M	42997	42997.00
act	T	0	0.31
acc	T	0	0.18
aca	T	0	0.43
acg	T	1	0.08
gtt	V	0	2.00
gtc	V	0	1.63
gta	V	0	1.73
gtg	V	8	2.63
---	--	------	------
mPD		0.0020	0.0023
		nPD:	0.86
		N. weight:	0.0008
		Sc. PD:	0.00062
		Sc. rank:	28.7

NP			
Pos.	224	obs:	exp:
tgt	C	67	16820.00
tgc	C	42973	26220.00
---	--	------	------
mPD		0.0031	0.48
		nPD:	0.01
		N. weight:	0.5
		Sc. PD:	-0.024
		Sc. rank:	-9978.1

NP			
Pos.	225	obs:	exp:
aat	N	135	24080.00
aac	N	42905	18960.00
---	--	------	------
mPD		0.0063	0.49
		nPD:	0.01
		N. weight:	0.83
		Sc. PD:	-0.034
		Sc. rank:	-9349.7

NP			
Pos.	226	obs:	exp:
att	I	42819	15106.00
atc	I	211	11307.00
ata	I	3	16619.00
atg	M	2	2.00
act	T	1	0.31
acc	T	0	0.18
aca	T	0	0.43
acg	T	0	0.08
gtt	V	4	1.00
gtc	V	0	0.82
gta	V	0	0.87
gtg	V	0	1.32
---	--	------	------
mPD		0.010	0.66
		nPD:	0.02
		N. weight:	1.1
		Sc. PD:	-0.04
		Sc. rank:	-6378.3

NP			
Pos.	227	obs:	exp:
tta	L	0	4994.00
ttg	L	0	8898.00
ctt	L	40298	9003.00
ctc	L	2725	6311.00
cta	L	11	6609.00
ctg	L	4	7223.00
att	I	2	0.70
atc	I	0	0.53
ata	I	0	0.77
---	--	------	------
mPD		0.12	1.2
		nPD:	0.1
		N. weight:	1.4
		Sc. PD:	0.065
		Sc. rank:	36406.0

NP			
Pos.	228	obs:	exp:
aaa	K	42934	28296.00
aag	K	106	14744.00
---	--	------	------
mPD		0.0049	0.45
		nPD:	0.01
		N. weight:	0.42
		Sc. PD:	-0.018
		Sc. rank:	-6199.4

NP			
Pos.	229	obs:	exp:
gtt	V	0	0.25
gtc	V	0	0.20
gta	V	1	0.22
gtg	V	0	0.33
ggt	G	3	5794.00
ggc	G	136	6007.00
gga	G	42185	19868.00
ggg	G	715	11370.00
---	--	------	------
mPD		0.039	0.68
		nPD:	0.06
		N. weight:	0.7
		Sc. PD:	0.0016
		Sc. rank:	14145.0

,

NP			
Pos.	230	obs:	exp:
aaa	K	42038	28296.00
aag	K	1002	14744.00
---	--	------	------
mPD		0.045	0.45
		nPD:	0.1
		N. weight:	0.34
		Sc. PD:	0.015
		Sc. rank:	8662.3

,

NP			
Pos.	231	obs:	exp:
ttt	F	42884	20932.00
ttc	F	148	22100.00
tta	L	2	0.81
ttg	L	0	1.45
tat	Y	1	0.53
tac	Y	0	0.47
ctt	L	5	1.46
ctc	L	0	1.03
cta	L	0	1.07
ctg	L	0	1.17
---	--	------	------
mPD		0.0072	0.50
		nPD:	0.01
		N. weight:	0.72
		Sc. PD:	-0.028
		Sc. rank:	-6358.5

,

NP			
Pos.	232	obs:	exp:
tta	L	0	0.12
ttg	L	0	0.21
ctt	L	0	0.21
ctc	L	0	0.15
cta	L	1	0.15
ctg	L	0	0.17
caa	Q	42891	26309.00
cag	Q	148	16730.00
---	--	------	------
mPD		0.0069	0.48
		nPD:	0.01
		N. weight:	0.49
		Sc. PD:	-0.019
		Sc. rank:	-4206.7

,

NP			
Pos.	233	obs:	exp:
att	I	0	2.46
atc	I	0	1.84
ata	I	7	2.70
act	T	33	13363.00
acc	T	1	7608.00
aca	T	42973	18623.00
acg	T	24	3437.00
gct	A	0	0.55
gcc	A	0	0.50
gca	A	2	0.81
gcg	A	0	0.15
---	--	------	------
mPD		0.0031	0.68
		nPD:	0.
		N. weight:	0.86
		Sc. PD:	-0.042
		Sc. rank:	-21545.0

,

NP			
Pos.	234	obs:	exp:
cct	P	1	0.33
ccc	P	0	0.19
cca	P	0	0.34
ccg	P	0	0.14
gct	A	42785	11730.00
gcc	A	50	10753.00
gca	A	128	17409.00
gcg	A	76	3146.00
---	--	------	------
mPD		0.012	0.69
		nPD:	0.02
		N. weight:	1.3
		Sc. PD:	-0.048
		Sc. rank:	-6268.6

,

NP			
Pos.	235	obs:	exp:
gtt	V	0	0.25
gtc	V	0	0.20
gta	V	1	0.22
gtg	V	0	0.33
gct	A	1	11730.00
gcc	A	115	10753.00
gca	A	42905	17409.00
gcg	A	18	3146.00
---	--	------	------
mPD		0.0063	0.69
		nPD:	0.01
		N. weight:	0.92
		Sc. PD:	-0.041
		Sc. rank:	-16076.0

,

NP			
Pos.	236	obs:	exp:
caa	Q	42849	26309.00
cag	Q	191	16731.00
---	--	------	------
mPD		0.0088	0.48
		nPD:	0.02
		N. weight:	0.48
		Sc. PD:	-0.017
		Sc. rank:	-1447.7

,

NP			
Pos.	237	obs:	exp:
cgt	R	0	843.60
cgc	R	0	1531.00
cga	R	8	3882.00
cgg	R	0	3653.00
aaa	K	491	325.40
aag	K	4	169.60
aga	R	42232	20868.00
agg	R	305	11768.00
---	--	------	------
mPD		0.037	0.90
		nPD:	0.04
		N. weight:	0.69
		Sc. PD:	-0.0093
		Sc. rank:	10614.0

,

NP			
Pos.	238	obs:	exp:
act	T	0	0.93
acc	T	0	0.53
aca	T	3	1.30
acg	T	0	0.24
gct	A	45	11730.00
gcc	A	1	10752.00
gca	A	42971	17409.00
gcg	A	20	3146.00
---	--	------	------
mPD		0.0032	0.69
		nPD:	0.
		N. weight:	0.93
		Sc. PD:	-0.045
		Sc. rank:	-23147.0

,

NP			
Pos.	239	obs:	exp:
tta	L	0	0.23
ttg	L	2	0.41
ctt	L	0	0.42
ctc	L	0	0.29
cta	L	0	0.31
ctg	L	0	0.34
atg	M	43038	43038.00
---	--	------	------
mPD		0.000093	0.00015
		nPD:	0.62
		N. weight:	0.000076
		Sc. PD:	0.000041
		Sc. rank:	2.7

,

NP			
Pos.	240	obs:	exp:
atg	M	857	857.00
gtt	V	6	10565.00
gtc	V	0	8594.00
gta	V	1053	9142.00
gtg	V	41119	13878.00
gct	A	0	1.36
gcc	A	0	1.25
gca	A	0	2.02
gcg	A	5	0.37
---	--	------	------
mPD		0.087	0.78
		nPD:	0.11
		N. weight:	1.
		Sc. PD:	0.056
		Sc. rank:	27788.0

,

NP			
Pos.	241	obs:	exp:
gat	D	36755	24329.00
gac	D	6285	18711.00
---	--	------	------
mPD		0.25	0.49
		nPD:	0.51
		N. weight:	0.2
		Sc. PD:	0.088
		Sc. rank:	6994.7

,

NP			
Pos.	242	obs:	exp:
caa	Q	42806	26309.00
cag	Q	233	16730.00
cgt	R	0	0.02
cgc	R	0	0.04
cga	R	1	0.09
cgg	R	0	0.09
aga	R	0	0.49
agg	R	0	0.28
---	--	------	------
mPD		0.011	0.48
		nPD:	0.02
		N. weight:	0.48
		Sc. PD:	-0.015
		Sc. rank:	718.7

,

NP			
Pos.	243	obs:	exp:
att	I	19	6.67
atc	I	0	4.99
ata	I	0	7.34
gtt	V	39598	10776.00
gtc	V	192	8766.00
gta	V	29	9324.00
gtg	V	3201	14155.00
gct	A	1	0.27
gcc	A	0	0.25
gca	A	0	0.40
gcg	A	0	0.07
---	--	------	------
mPD		0.15	0.74
		nPD:	0.2
		N. weight:	1.1
		Sc. PD:	0.15
		Sc. rank:	34950.0

,

NP			
Pos.	244	obs:	exp:
cgt	R	0	852.00
cgc	R	0	1546.00
cga	R	6	3921.00
cgg	R	0	3690.00
aaa	K	67	44.05
aag	K	0	22.95
aga	R	42910	21078.00
agg	R	57	11886.00
---	--	------	------
mPD		0.0060	0.89
		nPD:	0.01
		N. weight:	0.73
		Sc. PD:	-0.034
		Sc. rank:	-14309.0

,

NP			
Pos.	245	obs:	exp:
aaa	K	0	0.66
aag	K	1	0.34
gaa	E	42951	25374.00
gag	E	88	17665.00
---	--	------	------
mPD		0.0042	0.48
		nPD:	0.01
		N. weight:	0.53
		Sc. PD:	-0.024
		Sc. rank:	-9578.6

,

NP			
Pos.	246	obs:	exp:
tct	S	0	6945.00
tcc	S	0	6431.00
tca	S	0	11567.00
tcg	S	0	1694.00
agt	S	42103	7521.00
agc	S	934	8878.00
ggt	G	3	0.40
ggc	G	0	0.42
gga	G	0	1.38
ggg	G	0	0.79
---	--	------	------
mPD		0.043	1.6
		nPD:	0.03
		N. weight:	1.7
		Sc. PD:	-0.048
		Sc. rank:	7912.5

,

NP			
Pos.	247	obs:	exp:
cgt	R	234	853.40
cgc	R	0	1549.00
cga	R	384	3927.00
cgg	R	42363	3696.00
aga	R	0	21110.00
agg	R	59	11905.00
---	--	------	------
mPD		0.031	0.89
		nPD:	0.04
		N. weight:	2.5
		Sc. PD:	-0.047
		Sc. rank:	30917.0

,

NP			
Pos.	248	obs:	exp:
tct	S	0	8.39
tcc	S	0	7.77
tca	S	0	13.98
tcg	S	0	2.05
cat	H	1	0.56
cac	H	0	0.44
act	T	0	0.93
acc	T	3	0.53
aca	T	0	1.30
acg	T	0	0.24
aat	N	100	24048.00
aac	N	42884	18936.00
agt	S	0	9.09
agc	S	52	10.73
---	--	------	------
mPD		0.0073	0.50
		nPD:	0.01
		N. weight:	0.83
		Sc. PD:	-0.033
		Sc. rank:	-6698.3

,

NP			
Pos.	249	obs:	exp:
cct	P	21	14342.00
ccc	P	16	8069.00
cca	P	42964	14541.00
ccg	P	38	6087.00
act	T	0	0.31
acc	T	0	0.18
aca	T	1	0.43
acg	T	0	0.08
---	--	------	------
mPD		0.0035	0.72
		nPD:	0.
		N. weight:	1.1
		Sc. PD:	-0.054
		Sc. rank:	-26843.0

,

NP			
Pos.	250	obs:	exp:
ggt	G	2	5794.00
ggc	G	5	6007.00
gga	G	42862	19868.00
ggg	G	171	11371.00
---	--	------	------
mPD		0.0082	0.68
		nPD:	0.01
		N. weight:	0.78
		Sc. PD:	-0.032
		Sc. rank:	-10116.0

,

NP			
Pos.	251	obs:	exp:
tct	S	0	0.32
tcc	S	0	0.30
tca	S	0	0.54
tcg	S	0	0.08
aat	N	2772	24078.00
aac	N	40266	18960.00
agt	S	0	0.35
agc	S	2	0.41
---	--	------	------
mPD		0.12	0.49
		nPD:	0.24
		N. weight:	0.59
		Sc. PD:	0.11
		Sc. rank:	18996.0

,

NP			
Pos.	252	obs:	exp:
tct	S	1	0.16
tcc	S	0	0.15
tca	S	0	0.27
tcg	S	0	0.04
agt	S	0	0.17
agc	S	0	0.21
gtt	V	13	3.26
gtc	V	0	2.65
gta	V	0	2.82
gtg	V	0	4.28
gct	A	42964	11727.00
gcc	A	33	10750.00
gca	A	28	17404.00
gcg	A	1	3145.00
---	--	------	------
mPD		0.0035	0.70
		nPD:	0.01
		N. weight:	1.3
		Sc. PD:	-0.065
		Sc. rank:	-31922.0

,

NP			
Pos.	253	obs:	exp:
gaa	E	151	25375.00
gag	E	42889	17665.00
---	--	------	------
mPD		0.0070	0.48
		nPD:	0.01
		N. weight:	0.9
		Sc. PD:	-0.035
		Sc. rank:	-7985.6

,

NP			
Pos.	254	obs:	exp:
ttt	F	0	2.43
ttc	F	5	2.57
att	I	224	15101.00
atc	I	42755	11303.00
ata	I	39	16613.00
act	T	0	3.73
acc	T	11	2.12
aca	T	1	5.19
acg	T	0	0.96
aat	N	0	0.56
aac	N	1	0.44
gtt	V	0	1.00
gtc	V	4	0.82
gta	V	0	0.87
gtg	V	0	1.32
---	--	------	------
mPD		0.013	0.66
		nPD:	0.02
		N. weight:	1.3
		Sc. PD:	-0.046
		Sc. rank:	-1375.2

,

NP			
Pos.	255	obs:	exp:
gaa	E	42938	25375.00
gag	E	102	17665.00
---	--	------	------
mPD		0.0047	0.48
		nPD:	0.01
		N. weight:	0.53
		Sc. PD:	-0.023
		Sc. rank:	-8809.0

,

NP			
Pos.	256	obs:	exp:
gat	D	42761	24328.00
gac	D	277	18710.00
ggt	G	2	0.27
ggc	G	0	0.28
gga	G	0	0.92
ggg	G	0	0.53
---	--	------	------
mPD		0.013	0.49
		nPD:	0.03
		N. weight:	0.55
		Sc. PD:	-0.015
		Sc. rank:	2752.8

,

NP			
Pos.	257	obs:	exp:
ttt	F	0	0.49
ttc	F	1	0.51
tta	L	0	4994.00
ttg	L	0	8898.00
ctt	L	56	9003.00
ctc	L	42955	6311.00
cta	L	27	6609.00
ctg	L	0	7223.00
att	I	0	0.35
atc	I	1	0.26
ata	I	0	0.39
---	--	------	------
mPD		0.0039	1.2
		nPD:	0.
		N. weight:	2.
		Sc. PD:	-0.099
		Sc. rank:	-55135.0

,

NP			
Pos.	258	obs:	exp:
tta	L	1	0.35
ttg	L	0	0.62
ctt	L	0	0.63
ctc	L	0	0.44
cta	L	2	0.46
ctg	L	0	0.50
att	I	127	15094.00
atc	I	187	11298.00
ata	I	42685	16606.00
atg	M	20	20.00
act	T	4	5.59
acc	T	2	3.18
aca	T	12	7.79
acg	T	0	1.44
---	--	------	------
mPD		0.017	0.66
		nPD:	0.03
		N. weight:	0.94
		Sc. PD:	-0.027
		Sc. rank:	3554.3

,

NP			
Pos.	259	obs:	exp:
ttt	F	42884	20935.00
ttc	F	155	22104.00
tct	S	1	0.16
tcc	S	0	0.15
tca	S	0	0.27
tcg	S	0	0.04
agt	S	0	0.17
agc	S	0	0.21
---	--	------	------
mPD		0.0072	0.50
		nPD:	0.01
		N. weight:	0.72
		Sc. PD:	-0.028
		Sc. rank:	-6274.4

,

NP			
Pos.	260	obs:	exp:
tta	L	40780	4974.00
ttg	L	1817	8863.00
tct	S	0	27.59
tcc	S	0	25.55
tca	S	3	45.96
tcg	S	168	6.73
ctt	L	0	8967.00
ctc	L	0	6286.00
cta	L	124	6582.00
ctg	L	146	7194.00
caa	Q	0	1.22
cag	Q	2	0.78
agt	S	0	29.88
agc	S	0	35.28
---	--	------	------
mPD		0.11	1.2
		nPD:	0.1
		N. weight:	2.
		Sc. PD:	0.082
		Sc. rank:	50208.0

,

NP

Pos.	261	obs:	exp:
tct	S	0	0.65
tcc	S	0	0.60
tca	S	4	1.08
tcg	S	0	0.16
act	T	0	4.04
acc	T	0	2.30
aca	T	13	5.63
acg	T	0	1.04
agt	S	0	0.70
agc	S	0	0.83
gtt	V	0	0.25
gtc	V	0	0.20
gta	V	1	0.22
gtg	V	0	0.33
gct	A	7	11726.00
gcc	A	0	10749.00
gca	A	42998	17403.00
gcg	A	17	3145.00
---	--	------	------
mPD		0.0020	0.70
		nPD:	0.
		N. weight:	0.93
		Sc. PD:	-0.047
		Sc. rank:	-27526.0

,

NP

Pos.	262	obs:	exp:
cgt	R	0	853.40
cgc	R	0	1549.00
cga	R	5	3927.00
cgg	R	190	3696.00
aga	R	42612	21110.00
agg	R	233	11905.00
---	--	------	------
mPD		0.028	0.89
		nPD:	0.03
		N. weight:	0.68
		Sc. PD:	-0.015
		Sc. rank:	6907.7

,

NP

Pos.	263	obs:	exp:
tct	S	41811	6945.00
tcc	S	1094	6432.00
tca	S	129	11568.00
tcg	S	6	1694.00
agt	S	0	7521.00
agc	S	0	8879.00
---	--	------	------
mPD		0.056	1.6
		nPD:	0.03
		N. weight:	1.7
		Sc. PD:	-0.036
		Sc. rank:	20356.0

,

NP

Pos.	264	obs:	exp:
tct	S	0	0.65
tcc	S	0	0.60
tca	S	4	1.08
tcg	S	0	0.16
agt	S	0	0.70
agc	S	0	0.83
gct	A	33	11730.00
gcc	A	5	10752.00
gca	A	42957	17408.00
gcg	A	41	3146.00
---	--	------	------
mPD		0.0039	0.69
		nPD:	0.01
		N. weight:	0.93
		Sc. PD:	-0.044
		Sc. rank:	-20655.0

,

NP

Pos.	265	obs:	exp:
tta	L	281	4994.00
ttg	L	2655	8898.00
ctt	L	114	9003.00
ctc	L	48	6311.00
cta	L	267	6609.00
ctg	L	39673	7223.00
caa	Q	0	0.61
cag	Q	1	0.39
atg	M	1	1.00
---	--	------	------
mPD		0.16	1.2
		nPD:	0.14
		N. weight:	1.5
		Sc. PD:	0.12
		Sc. rank:	42792.0

,

NP

Pos.	266	obs:	exp:
att	I	118	15101.00
atc	I	7	11303.00
ata	I	42892	16613.00
atg	M	2	2.00
gtt	V	0	5.26
gtc	V	0	4.28
gta	V	21	4.55
gtg	V	0	6.91
---	--	------	------
mPD		0.0069	0.66
		nPD:	0.01
		N. weight:	0.97
		Sc. PD:	-0.042
		Sc. rank:	-15308.0

,

NP

Pos.	267	obs:	exp:
ttt	F	1	0.49
ttc	F	0	0.51
tta	L	189	4994.00
ttg	L	39321	8898.00
ctt	L	2	9003.00
ctc	L	0	6311.00
cta	L	107	6609.00
ctg	L	3420	7223.00
---	--	------	------
mPD		0.16	1.2
		nPD:	0.14
		N. weight:	1.3
		Sc. PD:	0.11
		Sc. rank:	38261.0

,

NP

Pos.	268	obs:	exp:
cgt	R	0	853.40
cgc	R	0	1549.00
cga	R	2	3927.00
cgg	R	0	3696.00
aga	R	42870	21110.00
agg	R	168	11905.00
---	--	------	------
mPD		0.0079	0.89
		nPD:	0.01
		N. weight:	0.71
		Sc. PD:	-0.032
		Sc. rank:	-12607.0

,

NP

Pos.	269	obs:	exp:
ggt	G	6	5794.00
ggc	G	1	6007.00
gga	G	42027	19868.00
ggg	G	1006	11371.00
---	--	------	------
mPD		0.046	0.68
		nPD:	0.07
		N. weight:	0.7
		Sc. PD:	0.0084
		Sc. rank:	15421.0

,

NP

Pos.	270	obs:	exp:
tct	S	15	6945.00
tcc	S	3	6432.00
tca	S	43009	11568.00
tcg	S	12	1694.00
agt	S	0	7521.00
agc	S	0	8879.00
gct	A	0	0.27
gcc	A	0	0.25
gca	A	1	0.40
gcg	A	0	0.07
---	--	------	------
mPD		0.0014	1.6
		nPD:	0.
		N. weight:	1.4
		Sc. PD:	-0.071
		Sc. rank:	-46238.0

,

NP

Pos.	271	obs:	exp:
tta	L	0	0.35
ttg	L	0	0.62
ctt	L	3	0.63
ctc	L	0	0.44
cta	L	0	0.46
ctg	L	0	0.50
att	I	93	32.65
atc	I	0	24.44
ata	I	0	35.92
gtt	V	42830	10753.00
gtc	V	70	8747.00
gta	V	23	9305.00
gtg	V	7	14125.00
gct	A	14	3.82
gcc	A	0	3.50
gca	A	0	5.66
gcg	A	0	1.02
---	--	------	------
mPD		0.0097	0.75
		nPD:	0.01
		N. weight:	1.4
		Sc. PD:	-0.058
		Sc. rank:	-15166.0

,

NP

Pos.	272	obs:	exp:
gct	A	42723	11731.00
gcc	A	188	10753.00
gca	A	126	17410.00
gcg	A	3	3146.00
---	--	------	------
mPD		0.015	0.69
		nPD:	0.02
		N. weight:	1.3
		Sc. PD:	-0.043
		Sc. rank:	-349.8

,

NP

Pos.	273	obs:	exp:
cat	H	207	24276.00
cac	H	42833	18764.00
---	--	------	------
mPD		0.0096	0.49
		nPD:	0.02
		N. weight:	0.83
		Sc. PD:	-0.029
		Sc. rank:	-1074.7

,

NP

Pos.	274	obs:	exp:
aaa	K	42836	28296.00
aag	K	204	14744.00
---	--	------	------
mPD		0.0094	0.45
		nPD:	0.02
		N. weight:	0.41
		Sc. PD:	-0.013
		Sc. rank:	-230.1

,

NP

Pos.	275	obs:	exp:
tct	S	42873	6945.00
tcc	S	79	6432.00
tca	S	70	11568.00
tcg	S	18	1694.00
agt	S	0	7521.00
agc	S	0	8879.00
---	--	------	------
mPD		0.0077	1.6
		nPD:	0.
		N. weight:	1.9
		Sc. PD:	-0.091
		Sc. rank:	-45402.0

,

NP

Pos.	276	obs:	exp:
tgt	C	510	16820.00
tgc	C	42530	26220.00
---	--	------	------
mPD		0.023	0.48
		nPD:	0.05
		N. weight:	0.45
		Sc. PD:	-0.0026
		Sc. rank:	8109.2

,

NP			
Pos.	277	obs:	exp:
tta	L	599	4994.00
ttg	L	1	8898.00
ctt	L	48	9004.00
ctc	L	11	6311.00
cta	L	42122	6609.00
ctg	L	259	7223.00
---	--	------	------
mPD		0.042	1.2
		nPD:	0.04
		N. weight:	1.8
		Sc. PD:	-0.033
		Sc. rank:	23978.0

NP			
Pos.	278	obs:	exp:
cct	P	38496	14342.00
ccc	P	82	8069.00
cca	P	4452	14541.00
ccg	P	9	6087.00
caa	Q	1	0.61
cag	Q	0	0.39
---	--	------	------
mPD		0.19	0.72
		nPD:	0.26
		N. weight:	0.78
		Sc. PD:	0.16
		Sc. rank:	25303.0

NP			
Pos.	279	obs:	exp:
tct	S	0	0.97
tcc	S	6	0.90
tca	S	0	1.61
tcg	S	0	0.24
agt	S	0	1.05
agc	S	0	1.24
gtt	V	0	0.25
gtc	V	1	0.20
gta	V	0	0.22
gtg	V	0	0.33
gct	A	250	11729.00
gcc	A	42766	10751.00
gca	A	17	17407.00
gcg	A	0	3146.00
---	--	------	------
mPD		0.013	0.69
		nPD:	0.02
		N. weight:	1.4
		Sc. PD:	-0.05
		Sc. rank:	-4741.9

NP			
Pos.	280	obs:	exp:
tgt	C	42992	16820.00
tgc	C	48	26220.00
---	--	------	------
mPD		0.0022	0.48
		nPD:	0.
		N. weight:	0.96
		Sc. PD:	-0.047
		Sc. rank:	-23959.0

NP			
Pos.	281	obs:	exp:
tct	S	0	0.48
tcc	S	0	0.45
tca	S	3	0.81
tcg	S	0	0.12
act	T	0	1.24
acc	T	0	0.71
aca	T	4	1.73
acg	T	0	0.32
agt	S	0	0.52
agc	S	0	0.62
gtt	V	2	233.40
gtc	V	0	189.90
gta	V	102	202.00
gtg	V	828	306.60
gct	A	14	11475.00
gcc	A	4	10519.00
gca	A	37263	17030.00
gcg	A	4820	3078.00
---	--	------	------
mPD		0.27	0.74
		nPD:	0.37
		N. weight:	0.77
		Sc. PD:	0.23
		Sc. rank:	26001.0

NP			
Pos.	282	obs:	exp:
tat	Y	42999	22687.00
tac	Y	41	20353.00
---	--	------	------
mPD		0.0019	0.50
		nPD:	0.
		N. weight:	0.66
		Sc. PD:	-0.033
		Sc. rank:	-18069.0

NP			
Pos.	283	obs:	exp:
ggt	G	2	5794.00
ggc	G	3	6007.00
gga	G	42683	19868.00
ggg	G	352	11371.00
---	--	------	------
mPD		0.016	0.68
		nPD:	0.02
		N. weight:	0.76
		Sc. PD:	-0.023
		Sc. rank:	2304.6

NP			
Pos.	284	obs:	exp:
tta	L	0	13.81
ttg	L	0	24.60
tct	S	4	0.65
tcc	S	0	0.60
tca	S	0	1.08
tcg	S	0	0.16
tat	Y	1	0.53
tac	Y	0	0.47
ctt	L	119	24.89
ctc	L	0	17.45
cta	L	0	18.27
ctg	L	0	19.97
cct	P	42793	14301.00
ccc	P	106	8045.00
cca	P	9	14499.00
ccg	P	6	6070.00
cat	H	2	1.13
cac	H	0	0.87
agt	S	0	0.70
agc	S	0	0.83
---	--	------	------
mPD		0.011	0.73
		nPD:	0.02
		N. weight:	1.1
		Sc. PD:	-0.043
		Sc. rank:	-6603.1

NP			
Pos.	285	obs:	exp:
act	T	0	0.31
acc	T	0	0.18
aca	T	1	0.43
acg	T	0	0.08
gtt	V	0	0.25
gtc	V	0	0.20
gta	V	1	0.22
gtg	V	0	0.33
gct	A	119	11730.00
gcc	A	92	10753.00
gca	A	42710	17409.00
gcg	A	117	3146.00
---	--	------	------
mPD		0.015	0.69
		nPD:	0.02
		N. weight:	0.89
		Sc. PD:	-0.029
		Sc. rank:	647.3

NP			
Pos.	286	obs:	exp:
att	I	0	16.50
atc	I	0	12.35
ata	I	47	18.15
atg	M	1	1.00
gtt	V	7	10768.00
gtc	V	6	8760.00
gta	V	42795	9318.00
gtg	V	183	14145.00
gct	A	0	0.27
gcc	A	0	0.25
gca	A	1	0.40
gcg	A	0	0.07
---	--	------	------
mPD		0.011	0.74
		nPD:	0.02
		N. weight:	1.6
		Sc. PD:	-0.06
		Sc. rank:	-11236.0

NP			
Pos.	287	obs:	exp:
tct	S	82	6914.00
tcc	S	42673	6403.00
tca	S	90	11516.00
tcg	S	0	1687.00
agt	S	0	7487.00
agc	S	0	8839.00
gct	A	0	53.15
gcc	A	79	48.72
gca	A	116	78.88
gcg	A	0	14.25
---	--	------	------
mPD		0.022	1.6
		nPD:	0.01
		N. weight:	1.9
		Sc. PD:	-0.078
		Sc. rank:	-19104.0

NP			
Pos.	288	obs:	exp:
tct	S	0	6935.00
tcc	S	0	6423.00
tca	S	0	11552.00
tcg	S	0	1692.00
att	I	1	0.35
atc	I	0	0.26
ata	I	0	0.39
aat	N	60	33.57
aac	N	0	26.43
agt	S	42524	7511.00
agc	S	455	8866.00
---	--	------	------
mPD		0.024	1.6
		nPD:	0.01
		N. weight:	1.7
		Sc. PD:	-0.069
		Sc. rank:	-14592.0

NP			
Pos.	289	obs:	exp:
ggt	G	37214	5794.00
ggc	G	71	6007.00
gga	G	257	19868.00
ggg	G	5498	11371.00
---	--	------	------
mPD		0.24	0.68
		nPD:	0.35
		N. weight:	1.5
		Sc. PD:	0.43
		Sc. rank:	51007.0

NP			
Pos.	290	obs:	exp:
tat	Y	196	22625.00
tac	Y	42726	20297.00
cat	H	115	66.56
cac	H	3	51.44
---	--	------	------
mPD		0.020	0.50
		nPD:	0.04
		N. weight:	0.75
		Sc. PD:	-0.011
		Sc. rank:	10799.0

NP			
Pos.	291	obs:	exp:
aat	N	0	15.67
aac	N	28	12.33
gct	A	0	0.27
gcc	A	1	0.25
gca	A	0	0.40
gcg	A	0	0.07
gat	D	1963	24289.00
gac	D	41006	18680.00
gaa	E	6	3.54
gag	E	0	2.46
ggt	G	1	4.85
ggc	G	35	5.02
gga	G	0	16.62
ggg	G	0	9.51
---	--	------	------
mPD		0.090	0.50
		nPD:	0.18
		N. weight:	0.66
		Sc. PD:	0.081
		Sc. rank:	20349.0

NP			
Pos.	292	obs:	exp:
ttt	F	37536	20936.00
ttc	F	5504	22104.00
---	--	------	------
mPD		0.22	0.50
		nPD:	0.45
		N. weight:	0.34
		Sc. PD:	0.13
		Sc. rank:	11858.0

NP			
Pos.	293	obs:	exp:
gaa	E	42948	25372.00
gag	E	88	17664.00
ggt	G	0	0.54
ggc	G	0	0.56
gga	G	4	1.85
ggg	G	0	1.06
---	--	------	------
mPD		0.0043	0.48
		nPD:	0.01
		N. weight:	0.53
		Sc. PD:	-0.024
		Sc. rank:	-9537.8

,

NP			
Pos.	294	obs:	exp:
cgt	R	0	2.70
cgc	R	0	4.89
cga	R	0	12.41
cgg	R	0	11.68
aaa	K	42365	28207.00
aag	K	539	14697.00
aga	R	18	66.71
agg	R	118	37.62
---	--	------	------
mPD		0.036	0.46
		nPD:	0.08
		N. weight:	0.38
		Sc. PD:	0.0088
		Sc. rank:	8670.2

,

NP			
Pos.	295	obs:	exp:
gaa	E	357	25375.00
gag	E	42683	17665.00
---	--	------	------
mPD		0.016	0.48
		nPD:	0.03
		N. weight:	0.87
		Sc. PD:	-0.018
		Sc. rank:	9925.8

,

NP			
Pos.	296	obs:	exp:
ggt	G	91	5794.00
ggc	G	21	6007.00
gga	G	42443	19868.00
ggg	G	485	11371.00
---	--	------	------
mPD		0.027	0.68
		nPD:	0.04
		N. weight:	0.73
		Sc. PD:	-0.01
		Sc. rank:	10724.0

,

NP			
Pos.	297	obs:	exp:
tat	Y	42604	22687.00
tac	Y	436	20353.00
---	--	------	------
mPD		0.020	0.50
		nPD:	0.04
		N. weight:	0.61
		Sc. PD:	-0.0088
		Sc. rank:	8876.5

,

NP			
Pos.	298	obs:	exp:
tct	S	220	6945.00
tcc	S	42672	6432.00
tca	S	148	11568.00
tcg	S	0	1694.00
agt	S	0	7521.00
agc	S	0	8879.00
---	--	------	------
mPD		0.017	1.6
		nPD:	0.01
		N. weight:	1.9
		Sc. PD:	-0.083
		Sc. rank:	-29769.0

,

NP			
Pos.	299	obs:	exp:
tta	L	378	4994.00
ttg	L	42498	8898.00
ctt	L	0	9003.00
ctc	L	0	6311.00
cta	L	5	6609.00
ctg	L	158	7223.00
atg	M	1	1.00
---	--	------	------
mPD		0.025	1.2
		nPD:	0.02
		N. weight:	1.6
		Sc. PD:	-0.05
		Sc. rank:	935.2

,

NP			
Pos.	300	obs:	exp:
gtt	V	11	10781.00
gtc	V	121	8770.00
gta	V	479	9329.00
gtg	V	42429	14161.00
---	--	------	------
mPD		0.028	0.74
		nPD:	0.04
		N. weight:	1.1
		Sc. PD:	-0.018
		Sc. rank:	15026.0

,

NP			
Pos.	301	obs:	exp:
ggt	G	2	5794.00
ggc	G	1	6007.00
gga	G	42754	19868.00
ggg	G	283	11371.00
---	--	------	------
mPD		0.013	0.68
		nPD:	0.02
		N. weight:	0.76
		Sc. PD:	-0.026
		Sc. rank:	-1082.1

,

NP			
Pos.	302	obs:	exp:
tta	L	0	0.23
ttg	L	0	0.41
ctt	L	0	0.42
ctc	L	0	0.29
cta	L	2	0.31
ctg	L	0	0.34
cgt	R	0	0.02
cgc	R	0	0.04
cga	R	0	0.09
cgg	R	0	0.09
att	I	335	15087.00
atc	I	1	11293.00
ata	I	42641	16598.00
atg	M	10	10.00
act	T	0	0.31
acc	T	0	0.18
aca	T	1	0.43
acg	T	0	0.08
aga	R	1	0.49
agg	R	0	0.28
gtt	V	2	12.27
gtc	V	0	9.98
gta	V	47	10.62
gtg	V	0	16.12
---	--	------	------
mPD		0.019	0.66
		nPD:	0.03
		N. weight:	0.94
		Sc. PD:	-0.025
		Sc. rank:	6025.1

,

NP			
Pos.	303	obs:	exp:
gat	D	205	24328.00
gac	D	42834	18711.00
gaa	E	0	0.59
gag	E	1	0.41
---	--	------	------
mPD		0.0095	0.49
		nPD:	0.02
		N. weight:	0.83
		Sc. PD:	-0.029
		Sc. rank:	-1269.0

,

NP			
Pos.	304	obs:	exp:
tct	S	1	0.16
tcc	S	0	0.15
tca	S	0	0.27
tcg	S	0	0.04
cct	P	42860	14342.00
ccc	P	46	8068.00
cca	P	119	14541.00
ccg	P	13	6087.00
cat	H	1	0.56
cac	H	0	0.44
agt	S	0	0.17
agc	S	0	0.21
---	--	------	------
mPD		0.0083	0.72
		nPD:	0.01
		N. weight:	1.1
		Sc. PD:	-0.047
		Sc. rank:	-16097.0

,

NP			
Pos.	305	obs:	exp:
ttt	F	171	20936.00
ttc	F	42869	22104.00
---	--	------	------
mPD		0.0079	0.50
		nPD:	0.02
		N. weight:	0.66
		Sc. PD:	-0.025
		Sc. rank:	-3849.2

,

NP			
Pos.	306	obs:	exp:
cgt	R	0	2.22
cgc	R	0	4.03
cga	R	1	10.22
cgg	R	0	9.62
aaa	K	42804	28222.00
aag	K	124	14706.00
aga	R	110	54.93
agg	R	1	30.98
---	--	------	------
mPD		0.011	0.46
		nPD:	0.02
		N. weight:	0.42
		Sc. PD:	-0.013
		Sc. rank:	1156.5

,

NP			
Pos.	307	obs:	exp:
tta	L	415	4994.00
ttg	L	4	8898.00
ctt	L	3	9003.00
ctc	L	4	6311.00
cta	L	40790	6609.00
ctg	L	1822	7223.00
att	I	0	0.35
atc	I	0	0.26
ata	I	1	0.39
atg	M	1	1.00
---	--	------	------
mPD		0.10	1.2
		nPD:	0.09
		N. weight:	1.7
		Sc. PD:	0.053
		Sc. rank:	41250.0

,

NP			
Pos.	308	obs:	exp:
tta	L	0	4994.00
ttg	L	0	8898.00
ctt	L	39145	9003.00
ctc	L	3835	6311.00
cta	L	3	6609.00
ctg	L	56	7223.00
att	I	1	0.35
atc	I	0	0.26
ata	I	0	0.39
---	--	------	------
mPD		0.16	1.2
		nPD:	0.14
		N. weight:	1.3
		Sc. PD:	0.11
		Sc. rank:	38885.0

,

NP			
Pos.	309	obs:	exp:
caa	Q	42984	26309.00
cag	Q	56	16731.00
---	--	------	------
mPD		0.0026	0.48
		nPD:	0.01
		N. weight:	0.5
		Sc. PD:	-0.024
		Sc. rank:	-11312.0

NP			
Pos.	310	obs:	exp:
tct	S	0	1.13
tcc	S	0	1.05
tca	S	0	1.88
tcg	S	0	0.28
act	T	0	0.62
acc	T	2	0.35
aca	T	0	0.87
acg	T	0	0.16
aat	N	42031	24075.00
aac	N	1000	18956.00
agt	S	7	1.22
agc	S	0	1.44
---	--	------	------
mPD		0.046	0.49
		nPD:	0.09
		N. weight:	0.49
		Sc. PD:	0.018
		Sc. rank:	12203.0

NP			
Pos.	311	obs:	exp:
tct	S	0	6944.00
tcc	S	0	6431.00
tca	S	0	11566.00
tcg	S	0	1694.00
cgt	R	0	0.02
cgc	R	0	0.04
cga	R	0	0.09
cgg	R	0	0.09
act	T	0	0.62
acc	T	2	0.35
aca	T	0	0.87
acg	T	0	0.16
aat	N	0	2.24
aac	N	4	1.76
agt	S	414	7520.00
agc	S	42619	8878.00
aga	R	1	0.49
agg	R	0	0.28
---	--	------	------
mPD		0.019	1.6
		nPD:	0.01
		N. weight:	1.6
		Sc. PD:	-0.066
		Sc. rank:	-21462.0

NP			
Pos.	312	obs:	exp:
cat	H	1	0.56
cac	H	0	0.44
caa	Q	42830	26309.00
cag	Q	209	16730.00
---	--	------	------
mPD		0.0097	0.48
		nPD:	0.02
		N. weight:	0.48
		Sc. PD:	-0.016
		Sc. rank:	-300.0

NP			
Pos.	313	obs:	exp:
tta	L	0	0.12
ttg	L	0	0.21
ctt	L	0	0.21
ctc	L	0	0.15
cta	L	1	0.15
ctg	L	0	0.17
att	I	2	14853.00
atc	I	9	11117.00
ata	I	42299	16340.00
atg	M	8	8.00
gtt	V	0	180.60
gtc	V	0	146.90
gta	V	475	156.30
gtg	V	246	237.20
---	--	------	------
mPD		0.045	0.70
		nPD:	0.07
		N. weight:	0.98
		Sc. PD:	0.0093
		Sc. rank:	21091.0

NP			
Pos.	314	obs:	exp:
ttt	F	0	51.07
ttc	F	105	53.93
tat	Y	35	22623.00
tac	Y	42884	20296.00
tgt	C	0	0.39
tgc	C	1	0.61
cat	H	0	2.26
cac	H	4	1.74
gtt	V	0	2.76
gtc	V	11	2.24
gta	V	0	2.38
gtg	V	0	3.62
---	--	------	------
mPD		0.0077	0.50
		nPD:	0.02
		N. weight:	0.77
		Sc. PD:	-0.03
		Sc. rank:	-5488.6

NP			
Pos.	315	obs:	exp:
tct	S	0	6944.00
tcc	S	0	6431.00
tca	S	0	11567.00
tcg	S	0	1694.00
tgt	C	0	2.34
tgc	C	6	3.66
agt	S	146	7520.00
agc	S	42888	8878.00
---	--	------	------
mPD		0.0070	1.6
		nPD:	0.
		N. weight:	1.6
		Sc. PD:	-0.079
		Sc. rank:	-41739.0

NP			
Pos.	316	obs:	exp:
tta	L	41973	4994.00
ttg	L	154	8898.00
ctt	L	0	9003.00
ctc	L	0	6311.00
cta	L	795	6609.00
ctg	L	117	7223.00
att	I	0	0.35
atc	I	0	0.26
ata	I	1	0.39
---	--	------	------
mPD		0.054	1.2
		nPD:	0.05
		N. weight:	2.1
		Sc. PD:	-0.017
		Sc. rank:	35982.0

NP			
Pos.	317	obs:	exp:
tta	L	0	0.12
ttg	L	0	0.21
ctt	L	0	0.21
ctc	L	1	0.15
cta	L	0	0.15
ctg	L	0	0.17
att	I	32	15097.00
atc	I	42854	11301.00
ata	I	121	16609.00
atg	M	11	11.00
act	T	0	1.55
acc	T	5	0.88
aca	T	0	2.16
acg	T	0	0.40
gtt	V	0	4.01
gtc	V	16	3.26
gta	V	0	3.47
gtg	V	0	5.26
---	--	------	------
mPD		0.0086	0.66
		nPD:	0.01
		N. weight:	1.4
		Sc. PD:	-0.055
		Sc. rank:	-14277.0

NP			
Pos.	318	obs:	exp:
cgt	R	0	853.40
cgc	R	0	1549.00
cga	R	2	3927.00
cgg	R	0	3696.00
aga	R	42833	21110.00
agg	R	205	11905.00
---	--	------	------
mPD		0.0096	0.89
		nPD:	0.01
		N. weight:	0.71
		Sc. PD:	-0.03
		Sc. rank:	-10894.0

NP			
Pos.	319	obs:	exp:
tta	L	0	0.35
ttg	L	0	0.62
tct	S	17	2.74
tcc	S	0	2.54
tca	S	0	4.57
tcg	S	0	0.67
ctt	L	2	0.63
ctc	L	0	0.44
cta	L	0	0.46
ctg	L	1	0.50
cct	P	40809	14327.00
ccc	P	1632	8060.00
cca	P	129	14525.00
ccg	P	423	6081.00
cat	H	21	11.84
cac	H	0	9.16
act	T	4	1.24
acc	T	0	0.71
aca	T	0	1.73
acg	T	0	0.32
agt	S	0	2.97
agc	S	0	3.51
gct	A	2	0.55
gcc	A	0	0.50
gca	A	0	0.81
gcg	A	0	0.15
---	--	------	------
mPD		0.10	0.72
		nPD:	0.14
		N. weight:	0.93
		Sc. PD:	0.074
		Sc. rank:	26659.0

NP			
Pos.	320	obs:	exp:
tct	S	0	2.42
tcc	S	0	2.24
tca	S	0	4.03
tcg	S	0	0.59
act	T	5	2.17
acc	T	2	1.24
aca	T	0	3.03
acg	T	0	0.56
aat	N	37742	24065.00
aac	N	5272	18949.00
aaa	K	3	1.97
aag	K	0	1.03
agt	S	13	2.62
agc	S	2	3.09
gat	D	1	0.57
gac	D	0	0.43
---	--	------	------
mPD		0.22	0.49
		nPD:	0.44
		N. weight:	0.25
		Sc. PD:	0.091
		Sc. rank:	8526.4

NP

Pos.	321	obs:	exp:
gaa	E	233	25374.00
gag	E	42806	17665.00
ggt	G	0	0.13
ggc	G	0	0.14
gga	G	0	0.46
ggg	G	1	0.26
---	--	------	------
mPD		0.011	0.48
		nPD:	0.02
		N. weight:	0.89
		Sc. PD:	-0.028
		Sc. rank:	1109.8

,

NP

Pos.	322	obs:	exp:
tct	S	0	0.16
tcc	S	0	0.15
tca	S	0	0.27
tcg	S	0	0.04
cat	H	2	1.13
cac	H	0	0.87
aat	N	42802	24078.00
aac	N	235	18959.00
agt	S	1	0.17
agc	S	0	0.21
---	--	------	------
mPD		0.011	0.49
		nPD:	0.02
		N. weight:	0.57
		Sc. PD:	-0.018
		Sc. rank:	639.2

,

NP

Pos.	323	obs:	exp:
cct	P	13	14343.00
ccc	P	1	8069.00
cca	P	42948	14541.00
ccg	P	78	6087.00
---	--	------	------
mPD		0.0043	0.72
		nPD:	0.01
		N. weight:	1.1
		Sc. PD:	-0.053
		Sc. rank:	-23108.0

,

NP

Pos.	324	obs:	exp:
tct	S	0	1.61
tcc	S	0	1.49
tca	S	9	2.69
tcg	S	1	0.39
act	T	0	1.86
acc	T	0	1.06
aca	T	6	2.60
acg	T	0	0.48
agt	S	0	1.75
agc	S	0	2.06
gtt	V	0	1.50
gtc	V	0	1.22
gta	V	5	1.30
gtg	V	1	1.97
gct	A	132	11724.00
gcc	A	0	10747.00
gca	A	42620	17401.00
gcg	A	265	3145.00
ggt	G	0	0.13
ggc	G	0	0.14
gga	G	1	0.46
ggg	G	0	0.26
---	--	------	------
mPD		0.019	0.70
		nPD:	0.03
		N. weight:	0.89
		Sc. PD:	-0.023
		Sc. rank:	5820.7

,

NP

Pos.	325	obs:	exp:
cat	H	47	24276.00
cac	H	42993	18764.00
---	--	------	------
mPD		0.0022	0.49
		nPD:	0.
		N. weight:	0.85
		Sc. PD:	-0.042
		Sc. rank:	-21847.0

,

NP

Pos.	326	obs:	exp:
aaa	K	851	28296.00
aag	K	42189	14744.00
---	--	------	------
mPD		0.039	0.45
		nPD:	0.09
		N. weight:	1.
		Sc. PD:	0.03
		Sc. rank:	23922.0

,

NP

Pos.	327	obs:	exp:
tct	S	0	6945.00
tcc	S	0	6432.00
tca	S	0	11568.00
tcg	S	0	1694.00
agt	S	42945	7521.00
agc	S	95	8879.00
---	--	------	------
mPD		0.0044	1.6
		nPD:	0.
		N. weight:	1.8
		Sc. PD:	-0.091
		Sc. rank:	-53788.0

,

NP

Pos.	328	obs:	exp:
caa	Q	1666	26309.00
cag	Q	41374	16731.00
---	--	------	------
mPD		0.074	0.48
		nPD:	0.16
		N. weight:	0.79
		Sc. PD:	0.078
		Sc. rank:	23838.0

,

NP

Pos.	329	obs:	exp:
tta	L	106	4992.00
ttg	L	53	8895.00
ctt	L	6	9000.00
ctc	L	1	6309.00
cta	L	5241	6606.00
ctg	L	37616	7220.00
att	I	0	0.35
atc	I	0	0.26
ata	I	1	0.39
atg	M	16	16.00
---	--	------	------
mPD		0.23	1.2
		nPD:	0.2
		N. weight:	1.4
		Sc. PD:	0.2
		Sc. rank:	45564.0

,

NP

Pos.	330	obs:	exp:
att	I	0	3.86
atc	I	0	2.89
ata	I	11	4.25
gtt	V	26	10778.00
gtc	V	4	8767.00
gta	V	7625	9326.00
gtg	V	35374	14158.00
---	--	------	------
mPD		0.29	0.74
		nPD:	0.4
		N. weight:	0.74
		Sc. PD:	0.24
		Sc. rank:	25169.0

,

NP

Pos.	331	obs:	exp:
tgg	W	43040	43040.00
---	--	------	------
mPD		0	0
		nPD:	1.
		N. weight:	0.
		Sc. PD:	0
		Sc. rank:	0

,

NP

Pos.	332	obs:	exp:
tta	L	0	0.23
ttg	L	2	0.41
ctt	L	0	0.42
ctc	L	0	0.29
cta	L	0	0.31
ctg	L	0	0.34
att	I	1	2.46
atc	I	0	1.84
ata	I	6	2.70
atg	M	43024	43024.00
act	T	0	0.93
acc	T	0	0.53
aca	T	0	1.30
acg	T	3	0.24
gtt	V	0	1.00
gtc	V	0	0.82
gta	V	0	0.87
gtg	V	4	1.32
---	--	------	------
mPD		0.00074	0.0011
		nPD:	0.71
		N. weight:	0.00048
		Sc. PD:	0.0003
		Sc. rank:	17.2

,

NP

Pos.	333	obs:	exp:
gct	A	20	11731.00
gcc	A	9	10753.00
gca	A	42958	17410.00
gcg	A	53	3146.00
---	--	------	------
mPD		0.0038	0.69
		nPD:	0.01
		N. weight:	0.93
		Sc. PD:	-0.044
		Sc. rank:	-20776.0

,

NP

Pos.	334	obs:	exp:
tgt	C	159	16820.00
tgc	C	42881	26220.00
---	--	------	------
mPD		0.0074	0.48
		nPD:	0.02
		N. weight:	0.49
		Sc. PD:	-0.019
		Sc. rank:	-3041.4

,

NP

Pos.	335	obs:	exp:
cat	H	42803	24245.00
cac	H	182	18740.00
aat	N	55	30.77
aac	N	0	24.23
---	--	------	------
mPD		0.011	0.49
		nPD:	0.02
		N. weight:	0.57
		Sc. PD:	-0.018
		Sc. rank:	534.9

,

NP

Pos.	336	obs:	exp:
tct	S	42948	6945.00
tcc	S	56	6432.00
tca	S	23	11568.00
tcg	S	11	1694.00
agt	S	0	7521.00
agc	S	0	8879.00
gct	A	2	0.55
gcc	A	0	0.50
gca	A	0	0.81
gcg	A	0	0.15
---	--	------	------
mPD		0.0043	1.6
		nPD:	0.
		N. weight:	1.9
		Sc. PD:	-0.095
		Sc. rank:	-57102.0

,

NP

Pos.	337	obs:	exp:
act	T	1	0.31
acc	T	0	0.18
aca	T	0	0.43
acg	T	0	0.08
gct	A	42563	11730.00
gcc	A	16	10753.00
gca	A	459	17409.00
gcg	A	1	3146.00
---	--	------	------
mPD		0.022	0.69
		nPD:	0.03
		N. weight:	1.3
		Sc. PD:	-0.029
		Sc. rank:	12454.0

,

NP

Pos.	338	obs:	exp:
cct	P	0	0.67
ccc	P	0	0.37
cca	P	2	0.68
ccg	P	0	0.28
act	T	0	1.55
acc	T	0	0.88
aca	T	5	2.16
acg	T	0	0.40
gct	A	19	11729.00
gcc	A	16	10751.00
gca	A	42887	17407.00
gcg	A	111	3146.00
---	--	------	------
mPD		0.0071	0.69
		nPD:	0.01
		N. weight:	0.92
		Sc. PD:	-0.04
		Sc. rank:	-14789.0

,

NP

Pos.	339	obs:	exp:
ttt	F	42996	20936.00
ttc	F	44	22104.00
---	--	------	------
mPD		0.0020	0.50
		nPD:	0.
		N. weight:	0.74
		Sc. PD:	-0.036
		Sc. rank:	-19857.0

,

NP

Pos.	340	obs:	exp:
gaa	E	42897	25375.00
gag	E	143	17665.00
---	--	------	------
mPD		0.0066	0.48
		nPD:	0.01
		N. weight:	0.53
		Sc. PD:	-0.021
		Sc. rank:	-5159.7

,

NP

Pos.	341	obs:	exp:
gat	D	42723	24329.00
gac	D	317	18711.00
---	--	------	------
mPD		0.015	0.49
		nPD:	0.03
		N. weight:	0.55
		Sc. PD:	-0.013
		Sc. rank:	4609.2

,

NP

Pos.	342	obs:	exp:
tta	L	42733	4994.00
ttg	L	82	8898.00
ctt	L	0	9003.00
ctc	L	0	6311.00
cta	L	223	6609.00
ctg	L	1	7223.00
att	I	0	0.35
atc	I	0	0.26
ata	I	1	0.39
---	--	------	------
mPD		0.014	1.2
		nPD:	0.01
		N. weight:	2.2
		Sc. PD:	-0.09
		Sc. rank:	-26939.0

,

NP

Pos.	343	obs:	exp:
cgt	R	0	852.50
cgc	R	0	1547.00
cga	R	5	3923.00
cgg	R	0	3692.00
aaa	K	43	28.27
aag	K	0	14.73
aga	R	42776	21089.00
agg	R	215	11893.00
ggt	G	0	0.13
ggc	G	0	0.14
gga	G	1	0.46
ggg	G	0	0.26
---	--	------	------
mPD		0.012	0.89
		nPD:	0.01
		N. weight:	0.71
		Sc. PD:	-0.028
		Sc. rank:	-6869.9

,

NP

Pos.	344	obs:	exp:
tta	L	180	4973.00
ttg	L	42603	8861.00
ctt	L	0	8966.00
ctc	L	0	6285.00
cta	L	0	6581.00
ctg	L	76	7193.00
att	I	0	0.35
atc	I	0	0.26
ata	I	1	0.39
gtt	V	0	45.09
gtc	V	0	36.68
gta	V	148	39.01
gtg	V	32	59.22
---	--	------	------
mPD		0.027	1.2
		nPD:	0.02
		N. weight:	1.6
		Sc. PD:	-0.049
		Sc. rank:	3452.8

,

NP

Pos.	345	obs:	exp:
tta	L	42728	4980.00
ttg	L	135	8873.00
tct	S	0	20.01
tcc	S	0	18.53
tca	S	124	33.33
tcg	S	0	4.88
ctt	L	0	8978.00
ctc	L	0	6293.00
cta	L	53	6590.00
ctg	L	0	7203.00
agt	S	0	21.67
agc	S	0	25.58
---	--	------	------
mPD		0.014	1.2
		nPD:	0.01
		N. weight:	2.2
		Sc. PD:	-0.091
		Sc. rank:	-26422.0

,

NP

Pos.	346	obs:	exp:
tct	S	0	6944.00
tcc	S	0	6431.00
tca	S	0	11566.00
tcg	S	0	1694.00
aat	N	0	3.36
aac	N	6	2.64
agt	S	8170	7520.00
agc	S	34862	8877.00
ggt	G	0	0.27
ggc	G	2	0.28
gga	G	0	0.92
ggg	G	0	0.53
---	--	------	------
mPD		0.31	1.6
		nPD:	0.19
		N. weight:	1.2
		Sc. PD:	0.15
		Sc. rank:	36189.0

,

NP

Pos.	347	obs:	exp:
ttt	F	62	20933.00
ttc	F	42973	22102.00
tta	L	3	0.46
ttg	L	0	0.83
tat	Y	0	0.53
tac	Y	1	0.47
ctt	L	0	0.84
ctc	L	1	0.59
cta	L	0	0.61
ctg	L	0	0.67
---	--	------	------
mPD		0.0031	0.50
		nPD:	0.01
		N. weight:	0.68
		Sc. PD:	-0.032
		Sc. rank:	-13878.0

,

NP

Pos.	348	obs:	exp:
tta	L	0	0.12
ttg	L	0	0.21
ctt	L	0	0.21
ctc	L	1	0.15
cta	L	0	0.15
ctg	L	0	0.17
att	I	106	15108.00
atc	I	42766	11308.00
ata	I	165	16621.00
gtt	V	0	0.50
gtc	V	2	0.41
gta	V	0	0.43
gtg	V	0	0.66
---	--	------	------
mPD		0.013	0.66
		nPD:	0.02
		N. weight:	1.3
		Sc. PD:	-0.047
		Sc. rank:	-2352.7

,

NP

Pos.	349	obs:	exp:
cgt	R	0	853.20
cgc	R	0	1548.00
cga	R	0	3926.00
cgg	R	0	3695.00
aaa	K	9	5.92
aag	K	0	3.08
aga	R	42954	21106.00
agg	R	77	11902.00
---	--	------	------
mPD		0.0040	0.89
		nPD:	0.
		N. weight:	0.73
		Sc. PD:	-0.036
		Sc. rank:	-18451.0

,

NP

Pos.	350	obs:	exp:
cgt	R	0	0.02
cgc	R	0	0.04
cga	R	0	0.09
cgg	R	0	0.09
aga	R	0	0.49
agg	R	1	0.28
ggt	G	15	5794.00
ggc	G	0	6007.00
gga	G	1220	19868.00
ggg	G	41804	11370.00
---	--	------	------
mPD		0.056	0.68
		nPD:	0.08
		N. weight:	1.2
		Sc. PD:	0.032
		Sc. rank:	28586.0

,

NP

Pos.	351	obs:	exp:
tct	S	0	1.61
tcc	S	2	1.49
tca	S	8	2.69
tcg	S	0	0.39
att	I	1	10.53
atc	I	0	7.88
ata	I	29	11.59
act	T	60	13119.00
acc	T	675	7469.00
aca	T	41481	18282.00
acg	T	28	3374.00
aat	N	0	0.56
aac	N	1	0.44
aaa	K	2	77.58
aag	K	116	40.42
agt	S	0	1.75
agc	S	0	2.06
gct	A	0	173.60
gcc	A	2	159.10
gca	A	635	257.70
gcg	A	0	46.57
---	--	------	------
mPD		0.077	0.72
		nPD:	0.11
		N. weight:	0.79
		Sc. PD:	0.04
		Sc. rank:	20707.0

,

NP

Pos.	352	obs:	exp:
cgt	R	0	0.50
cgc	R	0	0.90
cga	R	0	2.28
cgg	R	0	2.15
aaa	K	42895	28280.00
aag	K	120	14735.00
aga	R	25	12.26
agg	R	0	6.92
---	--	------	------
mPD		0.0067	0.45
		nPD:	0.01
		N. weight:	0.42
		Sc. PD:	-0.016
		Sc. rank:	-3295.5

,

NP

Pos.	353	obs:	exp:
tta	L	7	0.81
ttg	L	0	1.45
ctt	L	0	1.46
ctc	L	0	1.03
cta	L	0	1.07
ctg	L	0	1.17
att	I	0	7.72
atc	I	0	5.78
ata	I	22	8.50
gtt	V	90	10773.00
gtc	V	8	8763.00
gta	V	42574	9322.00
gtg	V	337	14151.00
gct	A	0	0.55
gcc	A	0	0.50
gca	A	2	0.81
gcg	A	0	0.15
---	--	------	------
mPD		0.021	0.74
		nPD:	0.03
		N. weight:	1.5
		Sc. PD:	-0.038
		Sc. rank:	11206.0

,

NP

Pos.	354	obs:	exp:
ttt	F	22	11.19
ttc	F	1	11.81
tta	L	1	12.76
ttg	L	0	22.74
tct	S	41533	6893.00
tcc	S	205	6383.00
tca	S	967	11481.00
tcg	S	9	1681.00
tat	Y	147	77.49
tac	Y	0	69.51
ctt	L	105	23.01
ctc	L	4	16.13
cta	L	0	16.89
ctg	L	0	18.46
cct	P	1	0.33
ccc	P	0	0.19
cca	P	0	0.34
ccg	P	0	0.14
att	I	6	8.42
atc	I	17	6.31
ata	I	1	9.27
act	T	17	5.28
acc	T	0	3.01
aca	T	0	7.36
acg	T	0	1.36
agt	S	0	7464.00
agc	S	0	8812.00
gtt	V	0	0.50
gtc	V	2	0.41
gta	V	0	0.43
gtg	V	0	0.66
gct	A	2	0.55
gcc	A	0	0.50
gca	A	0	0.81
gcg	A	0	0.15
---	--	------	------
mPD		0.076	1.6
		nPD:	0.05
		N. weight:	1.7
		Sc. PD:	-0.015
		Sc. rank:	29309.0

,

NP

Pos.	355	obs:	exp:
tct	S	2	0.32
tcc	S	0	0.30
tca	S	0	0.54
tcg	S	0	0.08
cct	P	42061	14342.00
ccc	P	230	8068.00
cca	P	215	14541.00
ccg	P	532	6087.00
agt	S	0	0.35
agc	S	0	0.41
---	--	------	------
mPD		0.045	0.72
		nPD:	0.06
		N. weight:	1.
		Sc. PD:	0.0069
		Sc. rank:	21362.0

,

NP

Pos.	356	obs:	exp:
cgt	R	14	853.40
cgc	R	0	1549.00
cga	R	1432	3927.00
cgg	R	40679	3696.00
aga	R	126	21110.00
agg	R	788	11905.00
ggt	G	0	0.13
ggc	G	0	0.14
gga	G	0	0.46
ggg	G	1	0.26
---	--	------	------
mPD		0.11	0.89
		nPD:	0.13
		N. weight:	2.2
		Sc. PD:	0.15
		Sc. rank:	62672.0

,

NP

Pos.	357	obs:	exp:
ggt	G	2	5794.00
ggc	G	1	6007.00
gga	G	1511	19868.00
ggg	G	41526	11371.00
---	--	------	------
mPD		0.068	0.68
		nPD:	0.1
		N. weight:	1.2
		Sc. PD:	0.052
		Sc. rank:	30911.0

,

NP

Pos.	358	obs:	exp:
caa	Q	1	0.61
cag	Q	0	0.39
cgt	R	0	1.11
cgc	R	0	2.01
cga	R	0	5.11
cgg	R	0	4.81
act	T	0	0.31
acc	T	0	0.18
aca	T	1	0.43
acg	T	0	0.08
aaa	K	42662	28258.00
aag	K	320	14724.00
aga	R	54	27.47
agg	R	2	15.49
---	--	------	------
mPD		0.018	0.45
		nPD:	0.04
		N. weight:	0.39
		Sc. PD:	-0.0063
		Sc. rank:	5549.6

,

NP

Pos.	359	obs:	exp:
tta	L	0	4994.00
ttg	L	113	8898.00
ctt	L	749	9003.00
ctc	L	8	6311.00
cta	L	263	6609.00
ctg	L	41904	7223.00
atg	M	3	3.00
---	--	------	------
mPD		0.052	1.2
		nPD:	0.04
		N. weight:	1.7
		Sc. PD:	-0.017
		Sc. rank:	27294.0

,

NP

Pos.	360	obs:	exp:
tta	L	29	6.27
ttg	L	25	11.16
tct	S	17	6935.00
tcc	S	166	6422.00
tca	S	40628	11551.00
tcg	S	2166	1692.00
ctt	L	0	11.30
ctc	L	0	7.92
cta	L	0	8.29
ctg	L	0	9.06
act	T	0	1.86
acc	T	0	1.06
aca	T	4	2.60
acg	T	2	0.48
agt	S	0	7510.00
agc	S	0	8866.00
gct	A	0	0.82
gcc	A	0	0.75
gca	A	3	1.21
gcg	A	0	0.22
---	--	------	------
mPD		0.11	1.6
		nPD:	0.07
		N. weight:	1.2
		Sc. PD:	0.013
		Sc. rank:	26652.0

,

NP

Pos.	361	obs:	exp:
att	I	1	0.35
atc	I	0	0.26
ata	I	0	0.39
act	T	42893	13366.00
acc	T	27	7609.00
aca	T	111	18626.00
acg	T	8	3437.00
---	--	------	------
mPD		0.0068	0.68
		nPD:	0.01
		N. weight:	1.2
		Sc. PD:	-0.052
		Sc. rank:	-19463.0

,

NP

Pos.	362	obs:	exp:
cgt	R	0	853.40
cgc	R	0	1549.00
cga	R	4	3927.00
cgg	R	0	3696.00
aga	R	43002	21110.00
agg	R	34	11905.00
---	--	------	------
mPD		0.0018	0.89
		nPD:	0.
		N. weight:	0.73
		Sc. PD:	-0.038
		Sc. rank:	-23712.0

,

NP

Pos.	363	obs:	exp:
ggt	G	43	5794.00
ggc	G	33	6007.00
gga	G	42575	19868.00
ggg	G	389	11371.00
---	--	------	------
mPD		0.021	0.68
		nPD:	0.03
		N. weight:	0.74
		Sc. PD:	-0.017
		Sc. rank:	7081.4

,

NP

Pos.	364	obs:	exp:
tta	L	6	0.93
ttg	L	0	1.65
ctt	L	0	1.67
ctc	L	0	1.17
cta	L	2	1.23
ctg	L	0	1.34
att	I	0	792.70
atc	I	0	593.30
ata	I	2258	872.00
gtt	V	12	10212.00
gtc	V	113	8307.00
gta	V	40572	8837.00
gtg	V	74	13415.00
gct	A	0	0.27
gcc	A	0	0.25
gca	A	1	0.40
gcg	A	0	0.07
ggt	G	0	0.27
ggc	G	1	0.28
gga	G	1	0.92
ggg	G	0	0.53
---	--	------	------
mPD		0.11	0.84
		nPD:	0.13
		N. weight:	1.5
		Sc. PD:	0.11
		Sc. rank:	42691.0

,

NP

Pos.	365	obs:	exp:
cct	P	0	0.33
ccc	P	0	0.19
cca	P	1	0.34
ccg	P	0	0.14
caa	Q	42887	26308.00
cag	Q	151	16730.00
aaa	K	1	0.66
aag	K	0	0.34
---	--	------	------
mPD		0.0071	0.48
		nPD:	0.01
		N. weight:	0.49
		Sc. PD:	-0.019
		Sc. rank:	-3820.5

,

NP

Pos.	366	obs:	exp:
tta	L	0	0.12
ttg	L	0	0.21
ctt	L	1	0.21
ctc	L	0	0.15
cta	L	0	0.15
ctg	L	0	0.17
att	I	42509	15104.00
atc	I	513	11306.00
ata	I	4	16617.00
act	T	2	0.62
acc	T	0	0.35
aca	T	0	0.87
acg	T	0	0.16
gtt	V	11	2.76
gtc	V	0	2.24
gta	V	0	2.38
gtg	V	0	3.62
---	--	------	------
mPD		0.024	0.66
		nPD:	0.04
		N. weight:	1.
		Sc. PD:	-0.018
		Sc. rank:	14140.0

,

NP

Pos.	367	obs:	exp:
tct	S	1	0.16
tcc	S	0	0.15
tca	S	0	0.27
tcg	S	0	0.04
agt	S	0	0.17
agc	S	0	0.21
gct	A	42795	11730.00
gcc	A	67	10753.00
gca	A	1	17409.00
gcg	A	176	3146.00
---	--	------	------
mPD		0.011	0.69
		nPD:	0.02
		N. weight:	1.3
		Sc. PD:	-0.049
		Sc. rank:	-7023.1

,

NP

Pos.	368	obs:	exp:
tct	S	0	6945.00
tcc	S	5	6432.00
tca	S	43012	11568.00
tcg	S	23	1694.00
agt	S	0	7521.00
agc	S	0	8879.00
---	--	------	------
mPD		0.0013	1.6
		nPD:	0.
		N. weight:	1.4
		Sc. PD:	-0.071
		Sc. rank:	-46907.0

,

NP

Pos.	369	obs:	exp:
tct	S	0	5.16
tcc	S	0	4.78
tca	S	0	8.60
tcg	S	0	1.26
att	I	1	0.35
atc	I	0	0.26
ata	I	0	0.39
aat	N	42980	24058.00
aac	N	22	18944.00
aaa	K	2	3.29
aag	K	3	1.71
agt	S	32	5.59
agc	S	0	6.60
---	--	------	------
mPD		0.0028	0.50
		nPD:	0.01
		N. weight:	0.6
		Sc. PD:	-0.029
		Sc. rank:	-13190.0

,

NP

Pos.	370	obs:	exp:
gaa	E	221	25375.00
gag	E	42819	17665.00
---	--	------	------
mPD		0.010	0.48
		nPD:	0.02
		N. weight:	0.89
		Sc. PD:	-0.029
		Sc. rank:	-126.2

,

NP

Pos.	371	obs:	exp:
tct	S	0	0.81
tcc	S	0	0.75
tca	S	0	1.34
tcg	S	0	0.20
cat	H	0	0.56
cac	H	1	0.44
aat	N	218	24075.00
aac	N	42813	18956.00
aaa	K	3	1.97
aag	K	0	1.03
agt	S	0	0.87
agc	S	5	1.03
---	--	------	------
mPD		0.010	0.49
		nPD:	0.02
		N. weight:	0.82
		Sc. PD:	-0.027
		Sc. rank:	142.7

,

NP

Pos.	372	obs:	exp:
tta	L	0	0.35
ttg	L	1	0.62
ctt	L	0	0.63
ctc	L	0	0.44
cta	L	0	0.46
ctg	L	2	0.50
att	I	13	24.57
atc	I	0	18.39
ata	I	57	27.03
atg	M	42834	42834.00
act	T	0	0.31
acc	T	0	0.18
aca	T	0	0.43
acg	T	1	0.08
gtt	V	0	33.06
gtc	V	0	26.90
gta	V	0	28.61
gtg	V	132	43.43
---	--	------	------
mPD		0.0095	0.014
		nPD:	0.69
		N. weight:	0.0046
		Sc. PD:	0.0028
		Sc. rank:	163.3

,

NP

Pos.	373	obs:	exp:
aat	N	1	0.56
aac	N	0	0.44
gat	D	42625	24249.00
gac	D	273	18649.00
gaa	E	131	81.95
gag	E	8	57.05
ggt	G	2	0.27
ggc	G	0	0.28
gga	G	0	0.92
ggg	G	0	0.53
---	--	------	------
mPD		0.019	0.49
		nPD:	0.04
		N. weight:	0.55
		Sc. PD:	-0.0088
		Sc. rank:	7814.8

,

NP

Pos.	374	obs:	exp:
tct	S	1	3.87
tcc	S	0	3.59
tca	S	0	6.45
tcg	S	0	0.94
cat	H	1	0.56
cac	H	0	0.44
act	T	46	17.70
acc	T	10	10.08
aca	T	1	24.67
acg	T	0	4.55
aat	N	42614	23958.00
aac	N	209	18865.00
aaa	K	1	0.66
aag	K	0	0.34
agt	S	23	4.19
agc	S	0	4.95
gct	A	29	36.52
gcc	A	105	33.48
gca	A	0	54.20
gcg	A	0	9.80
---	--	------	------
mPD		0.031	0.51
		nPD:	0.06
		N. weight:	0.57
		Sc. PD:	0.0033
		Sc. rank:	11593.0

,

NP

Pos.	375	obs:	exp:
tta	L	0	0.46
ttg	L	3	0.83
ctt	L	0	0.84
ctc	L	0	0.59
cta	L	0	0.61
ctg	L	1	0.67
att	I	20	167.80
atc	I	0	125.60
ata	I	458	184.60
atg	M	42542	42542.00
gtt	V	0	4.01
gtc	V	0	3.26
gta	V	0	3.47
gtg	V	16	5.26
---	--	------	------
mPD		0.023	0.024
		nPD:	0.97
		N. weight:	0.0096
		Sc. PD:	0.0085
		Sc. rank:	344.5

,

NP

Pos.	376	obs:	exp:
aaa	K	1	0.66
aag	K	0	0.34
gtt	V	0	0.25
gtc	V	0	0.20
gta	V	1	0.22
gtg	V	0	0.33
gat	D	105	65.57
gac	D	11	50.43
gaa	E	226	133.20
gag	E	0	92.76
ggt	G	2	5748.00
ggc	G	7	5959.00
gga	G	42308	19709.00
ggg	G	379	11280.00
---	--	------	------
mPD		0.039	0.70
		nPD:	0.06
		N. weight:	0.75
		Sc. PD:	0.00075
		Sc. rank:	14371.0

,

NP

Pos.	377	obs:	exp:
tct	S	13	6945.00
tcc	S	118	6432.00
tca	S	32418	11568.00
tcg	S	10489	1694.00
act	T	0	0.31
acc	T	0	0.18
aca	T	1	0.43
acg	T	0	0.08
agt	S	0	7521.00
agc	S	0	8879.00
gct	A	0	0.27
gcc	A	0	0.25
gca	A	1	0.40
gcg	A	0	0.07
---	--	------	------
mPD		0.37	1.6
		nPD:	0.23
		N. weight:	1.3
		Sc. PD:	0.21
		Sc. rank:	40461.0

,

NP

Pos.	378	obs:	exp:
tct	S	0	6909.00
tcc	S	0	6398.00
tca	S	0	11508.00
tcg	S	0	1685.00
cgt	R	0	0.02
cgc	R	0	0.04
cga	R	0	0.09
cgg	R	0	0.09
att	I	0	0.35
atc	I	1	0.26
ata	I	0	0.39
aat	N	115	67.70
aac	N	6	53.30
agt	S	555	7482.00
agc	S	42259	8832.00
aga	R	1	0.49
agg	R	0	0.28
ggt	G	0	13.87
ggc	G	103	14.38
gga	G	0	47.55
ggg	G	0	27.21
---	--	------	------
mPD		0.041	1.6
		nPD:	0.03
		N. weight:	1.6
		Sc. PD:	-0.045
		Sc. rank:	5718.6

,

NP

Pos.	379	obs:	exp:
act	T	41881	13366.00
acc	T	1149	7609.00
aca	T	7	18626.00
acg	T	2	3437.00
gct	A	1	0.27
gcc	A	0	0.25
gca	A	0	0.40
gcg	A	0	0.07
---	--	------	------
mPD		0.052	0.68
		nPD:	0.08
		N. weight:	1.1
		Sc. PD:	0.023
		Sc. rank:	25256.0

,

NP

Pos.	380	obs:	exp:
tta	L	105	4994.00
ttg	L	0	8898.00
ctt	L	41884	9004.00
ctc	L	1013	6311.00
cta	L	23	6609.00
ctg	L	15	7223.00
---	--	------	------
mPD		0.057	1.2
		nPD:	0.05
		N. weight:	1.5
		Sc. PD:	-0.0083
		Sc. rank:	27039.0

,

NP

Pos.	381	obs:	exp:
gaa	E	42887	25374.00
gag	E	152	17665.00
ggt	G	0	0.13
ggc	G	0	0.14
gga	G	1	0.46
ggg	G	0	0.26
---	--	------	------
mPD		0.0071	0.48
		nPD:	0.01
		N. weight:	0.53
		Sc. PD:	-0.021
		Sc. rank:	-4281.5

,

NP

Pos.	382	obs:	exp:
tta	L	1	4994.00
ttg	L	185	8897.00
ctt	L	8	9003.00
ctc	L	0	6311.00
cta	L	121	6608.00
ctg	L	42721	7223.00
atg	M	4	4.00
---	--	------	------
mPD		0.015	1.2
		nPD:	0.01
		N. weight:	1.8
		Sc. PD:	-0.074
		Sc. rank:	-19867.0

,

NP

Pos.	383	obs:	exp:
tct	S	0	0.16
tcc	S	0	0.15
tca	S	0	0.27
tcg	S	0	0.04
cgt	R	0	853.40
cgc	R	0	1549.00
cga	R	1	3927.00
cgg	R	0	3696.00
agt	S	0	0.17
agc	S	1	0.21
aga	R	42954	21110.00
agg	R	84	11905.00
---	--	------	------
mPD		0.0040	0.89
		nPD:	0.
		N. weight:	0.73
		Sc. PD:	-0.036
		Sc. rank:	-18348.0

,

NP

Pos.	384	obs:	exp:
tct	S	0	6945.00
tcc	S	0	6432.00
tca	S	0	11568.00
tcg	S	0	1694.00
agt	S	1487	7521.00
agc	S	41553	8879.00
---	--	------	------
mPD		0.067	1.6
		nPD:	0.04
		N. weight:	1.5
		Sc. PD:	-0.02
		Sc. rank:	22353.0

,

NP			
Pos.	385	obs:	exp:
tct	S	0	0.65
tcc	S	0	0.60
tca	S	0	1.08
tcg	S	0	0.16
cgt	R	0	7.83
cgc	R	0	14.21
cga	R	0	36.04
cgg	R	1	33.92
atg	M	4	4.00
aaa	K	0	5.26
aag	K	8	2.74
agt	S	3	0.70
agc	S	1	0.83
aga	R	126	193.70
agg	R	268	109.30
gct	A	0	0.27
gcc	A	0	0.25
gca	A	0	0.40
gcg	A	1	0.07
ggt	G	112	5739.00
ggc	G	0	5949.00
gga	G	6126	19678.00
ggg	G	36390	11262.00
---	--	------	------
mPD		0.27	0.70
		nPD:	0.39
		N. weight:	0.85
		Sc. PD:	0.28
		Sc. rank:	28862.0

,

NP			
Pos.	386	obs:	exp:
ttt	F	0	0.49
ttc	F	1	0.51
tat	Y	40	22676.00
tac	Y	42980	20344.00
cat	H	0	10.72
cac	H	19	8.28
---	--	------	------
mPD		0.0028	0.50
		nPD:	0.01
		N. weight:	0.77
		Sc. PD:	-0.037
		Sc. rank:	-17004.0

,

NP			
Pos.	387	obs:	exp:
tgg	W	43040	43040.00
---	--	------	------
mPD		0	0
		nPD:	1.
		N. weight:	0.
		Sc. PD:	0
		Sc. rank:	0

,

NP			
Pos.	388	obs:	exp:
gct	A	43	11731.00
gcc	A	42790	10753.00
gca	A	207	17410.00
gcg	A	0	3146.00
---	--	------	------
mPD		0.012	0.69
		nPD:	0.02
		N. weight:	1.4
		Sc. PD:	-0.052
		Sc. rank:	-7181.6

,

NP			
Pos.	389	obs:	exp:
att	I	11	15108.00
atc	I	25	11309.00
ata	I	43003	16622.00
gtt	V	0	0.25
gtc	V	0	0.20
gta	V	1	0.22
gtg	V	0	0.33
---	--	------	------
mPD		0.0017	0.66
		nPD:	0.
		N. weight:	0.98
		Sc. PD:	-0.05
		Sc. rank:	-30101.0

,

NP			
Pos.	390	obs:	exp:
cgt	R	0	853.30
cgc	R	0	1549.00
cga	R	0	3927.00
cgg	R	0	3696.00
aaa	K	0	1.31
aag	K	2	0.69
aga	R	4415	21109.00
agg	R	38623	11904.00
---	--	------	------
mPD		0.18	0.89
		nPD:	0.21
		N. weight:	0.93
		Sc. PD:	0.14
		Sc. rank:	29552.0

,

NP			
Pos.	391	obs:	exp:
act	T	182	13366.00
acc	T	42831	7610.00
aca	T	27	18627.00
acg	T	0	3438.00
---	--	------	------
mPD		0.0097	0.68
		nPD:	0.01
		N. weight:	1.8
		Sc. PD:	-0.07
		Sc. rank:	-15879.0

,

NP			
Pos.	392	obs:	exp:
cgt	R	0	853.20
cgc	R	0	1548.00
cga	R	0	3926.00
cgg	R	14	3695.00
aaa	K	0	6.57
aag	K	10	3.43
aga	R	654	21106.00
agg	R	42362	11902.00
---	--	------	------
mPD		0.031	0.89
		nPD:	0.04
		N. weight:	1.2
		Sc. PD:	-0.024
		Sc. rank:	15183.0

,

NP			
Pos.	393	obs:	exp:
tct	S	0	6945.00
tcc	S	0	6432.00
tca	S	0	11568.00
tcg	S	0	1694.00
agt	S	34729	7521.00
agc	S	8311	8879.00
---	--	------	------
mPD		0.31	1.6
		nPD:	0.19
		N. weight:	1.3
		Sc. PD:	0.17
		Sc. rank:	39566.0

,

NP			
Pos.	394	obs:	exp:
ggt	G	31	5794.00
ggc	G	3	6007.00
gga	G	42034	19868.00
ggg	G	972	11371.00
---	--	------	------
mPD		0.046	0.68
		nPD:	0.07
		N. weight:	0.7
		Sc. PD:	0.0081
		Sc. rank:	15312.0

,

NP			
Pos.	395	obs:	exp:
ggt	G	10	5794.00
ggc	G	0	6007.00
gga	G	42880	19868.00
ggg	G	150	11371.00
---	--	------	------
mPD		0.0074	0.68
		nPD:	0.01
		N. weight:	0.78
		Sc. PD:	-0.033
		Sc. rank:	-11560.0

,

NP			
Pos.	396	obs:	exp:
tct	S	0	0.81
tcc	S	0	0.75
tca	S	0	1.34
tcg	S	0	0.20
cat	H	0	0.56
cac	H	1	0.44
act	T	0	4.04
acc	T	13	2.30
aca	T	0	5.63
acg	T	0	1.04
aat	N	168	24068.00
aac	N	42852	18952.00
agt	S	0	0.87
agc	S	5	1.03
gat	D	0	0.57
gac	D	1	0.43
---	--	------	------
mPD		0.0087	0.49
		nPD:	0.02
		N. weight:	0.82
		Sc. PD:	-0.03
		Sc. rank:	-3703.0

,

NP			
Pos.	397	obs:	exp:
tct	S	2	0.32
tcc	S	0	0.30
tca	S	0	0.54
tcg	S	0	0.08
act	T	42705	13359.00
acc	T	288	7605.00
aca	T	10	18616.00
acg	T	13	3436.00
aat	N	18	10.07
aac	N	0	7.93
agt	S	0	0.35
agc	S	0	0.41
gct	A	4	1.09
gcc	A	0	1.00
gca	A	0	1.62
gcg	A	0	0.29
---	--	------	------
mPD		0.015	0.68
		nPD:	0.02
		N. weight:	1.2
		Sc. PD:	-0.037
		Sc. rank:	1955.5

,

NP			
Pos.	398	obs:	exp:
tct	S	0	4.68
tcc	S	0	4.33
tca	S	0	7.79
tcg	S	0	1.14
att	I	4	1.40
atc	I	0	1.05
ata	I	0	1.54
act	T	3	0.93
acc	T	0	0.53
aca	T	0	1.30
acg	T	0	0.24
aat	N	42602	24059.00
aac	N	402	18945.00
agt	S	29	5.07
agc	S	0	5.98
---	--	------	------
mPD		0.020	0.50
		nPD:	0.04
		N. weight:	0.55
		Sc. PD:	-0.0077
		Sc. rank:	8159.4

,

NP			
Pos.	399	obs:	exp:
cat	H	0	0.56
cac	H	1	0.44
caa	Q	43000	26306.00
cag	Q	34	16728.00
cgt	R	0	0.04
cgc	R	0	0.07
cga	R	2	0.18
cgg	R	0	0.17
aaa	K	3	1.97
aag	K	0	1.03
aga	R	0	0.98
agg	R	0	0.55
---	--	------	------
mPD		0.0019	0.48
		nPD:	0.
		N. weight:	0.5
		Sc. PD:	-0.025
		Sc. rank:	-13739.0

,

NP			
Pos.	400	obs:	exp:
cat	H	5	2.82
cac	H	0	2.18
caa	Q	221	26306.00
cag	Q	42813	16728.00
aaa	K	0	0.66
aag	K	1	0.34
---	--	------	------
mPD		0.010	0.48
		nPD:	0.02
		N. weight:	0.94
		Sc. PD:	-0.03
		Sc. rank:	814.2

,

NP			
Pos.	401	obs:	exp:
cgt	R	0	829.00
cgc	R	0	1504.00
cga	R	0	3815.00
cgg	R	4	3590.00
aaa	K	105	808.00
aag	K	1124	421.00
aga	R	234	20508.00
agg	R	41573	11565.00
---	--	------	------
mPD		0.071	0.93
		nPD:	0.08
		N. weight:	1.3
		Sc. PD:	0.026
		Sc. rank:	29161.0

,

NP			
Pos.	402	obs:	exp:
act	T	0	4.04
acc	T	13	2.30
aca	T	0	5.63
acg	T	0	1.04
gtt	V	0	0.50
gtc	V	2	0.41
gta	V	0	0.43
gtg	V	0	0.66
gct	A	207	11727.00
gcc	A	42651	10749.00
gca	A	167	17404.00
gcg	A	0	3145.00
---	--	------	------
mPD		0.018	0.70
		nPD:	0.03
		N. weight:	1.4
		Sc. PD:	-0.039
		Sc. rank:	6090.6

,

NP			
Pos.	403	obs:	exp:
tct	S	1221	6945.00
tcc	S	40791	6432.00
tca	S	1025	11568.00
tcg	S	1	1694.00
act	T	0	0.31
acc	T	1	0.18
aca	T	0	0.43
acg	T	0	0.08
agt	S	0	7521.00
agc	S	0	8879.00
gct	A	0	0.27
gcc	A	1	0.25
gca	A	0	0.40
gcg	A	0	0.07
---	--	------	------
mPD		0.10	1.6
		nPD:	0.06
		N. weight:	1.7
		Sc. PD:	0.011
		Sc. rank:	34878.0

,

NP			
Pos.	404	obs:	exp:
tct	S	0	0.65
tcc	S	0	0.60
tca	S	4	1.08
tcg	S	0	0.16
act	T	0	5.28
acc	T	0	3.01
aca	T	17	7.36
acg	T	0	1.36
agt	S	0	0.70
agc	S	0	0.83
gct	A	16	11725.00
gcc	A	2	10748.00
gca	A	42856	17401.00
gcg	A	144	3145.00
ggt	G	0	0.13
ggc	G	0	0.14
gga	G	1	0.46
ggg	G	0	0.26
---	--	------	------
mPD		0.0085	0.70
		nPD:	0.01
		N. weight:	0.92
		Sc. PD:	-0.038
		Sc. rank:	-11847.0

,

NP			
Pos.	405	obs:	exp:
tct	S	0	0.48
tcc	S	0	0.45
tca	S	0	0.81
tcg	S	0	0.12
agt	S	0	0.52
agc	S	3	0.62
ggt	G	149	5794.00
ggc	G	42867	6007.00
gga	G	20	19867.00
ggg	G	1	11370.00
---	--	------	------
mPD		0.0080	0.68
		nPD:	0.01
		N. weight:	2.
		Sc. PD:	-0.084
		Sc. rank:	-27986.0

,

NP			
Pos.	406	obs:	exp:
caa	Q	42868	26309.00
cag	Q	172	16731.00
---	--	------	------
mPD		0.0080	0.48
		nPD:	0.02
		N. weight:	0.49
		Sc. PD:	-0.018
		Sc. rank:	-2421.2

,

NP			
Pos.	407	obs:	exp:
att	I	3	214.80
atc	I	608	160.80
ata	I	1	236.40
act	T	40	13175.00
acc	T	42372	7501.00
aca	T	14	18361.00
acg	T	0	3389.00
aat	N	0	0.56
aac	N	1	0.44
gct	A	0	0.27
gcc	A	1	0.25
gca	A	0	0.40
gcg	A	0	0.07
---	--	------	------
mPD		0.031	0.71
		nPD:	0.04
		N. weight:	1.8
		Sc. PD:	-0.02
		Sc. rank:	27979.0

,

NP			
Pos.	408	obs:	exp:
tct	S	0	6945.00
tcc	S	0	6432.00
tca	S	0	11568.00
tcg	S	0	1694.00
agt	S	43001	7521.00
agc	S	39	8879.00
---	--	------	------
mPD		0.0018	1.6
		nPD:	0.
		N. weight:	1.8
		Sc. PD:	-0.094
		Sc. rank:	-60446.0

,

NP			
Pos.	409	obs:	exp:
att	I	0	2.11
atc	I	0	1.58
ata	I	6	2.32
gtt	V	278	10779.00
gtc	V	0	8768.00
gta	V	171	9327.00
gtg	V	42585	14159.00
---	--	------	------
mPD		0.021	0.74
		nPD:	0.03
		N. weight:	1.1
		Sc. PD:	-0.028
		Sc. rank:	7516.1

,

NP			
Pos.	410	obs:	exp:
caa	Q	42977	26309.00
cag	Q	63	16731.00
---	--	------	------
mPD		0.0029	0.48
		nPD:	0.01
		N. weight:	0.5
		Sc. PD:	-0.024
		Sc. rank:	-10319.0

,

NP			
Pos.	411	obs:	exp:
cct	P	40486	14343.00
ccc	P	98	8069.00
cca	P	2451	14541.00
ccg	P	5	6087.00
---	--	------	------
mPD		0.11	0.72
		nPD:	0.16
		N. weight:	0.9
		Sc. PD:	0.087
		Sc. rank:	26872.0

,

NP			
Pos.	412	obs:	exp:
act	T	105	13343.00
acc	T	5	7597.00
aca	T	9958	18595.00
acg	T	32899	3432.00
gct	A	0	19.90
gcc	A	0	18.24
gca	A	66	29.53
gcg	A	7	5.34
---	--	------	------
mPD		0.36	0.68
		nPD:	0.53
		N. weight:	1.6
		Sc. PD:	0.76
		Sc. rank:	57604.0

,

NP			
Pos.	413	obs:	exp:
ttt	F	42676	20936.00
ttc	F	364	22104.00
---	--	------	------
mPD		0.017	0.50
		nPD:	0.03
		N. weight:	0.7
		Sc. PD:	-0.015
		Sc. rank:	7851.5

,

NP			
Pos.	414	obs:	exp:
tct	S	42479	6945.00
tcc	S	256	6432.00
tca	S	250	11568.00
tcg	S	55	1694.00
agt	S	0	7521.00
agc	S	0	8879.00
---	--	------	------
mPD		0.026	1.6
		nPD:	0.02
		N. weight:	1.8
		Sc. PD:	-0.069
		Sc. rank:	-9994.6

,

NP			
Pos.	415	obs:	exp:
att	I	0	0.35
atc	I	0	0.26
ata	I	1	0.39
gtt	V	12	10780.00
gtc	V	3	8770.00
gta	V	42736	9328.00
gtg	V	288	14161.00
---	--	------	------
mPD		0.014	0.74
		nPD:	0.02
		N. weight:	1.5
		Sc. PD:	-0.054
		Sc. rank:	-3463.5

,

NP			
Pos.	416	obs:	exp:
caa	Q	42987	26309.00
cag	Q	53	16731.00
---	--	------	------
mPD		0.0025	0.48
		nPD:	0.01
		N. weight:	0.5
		Sc. PD:	-0.024
		Sc. rank:	-11522.0

,

NP			
Pos.	417	obs:	exp:
cgt	R	0	853.40
cgc	R	0	1549.00
cga	R	35	3927.00
cgg	R	106	3696.00
aga	R	42807	21110.00
agg	R	92	11905.00
---	--	------	------
mPD		0.016	0.89
		nPD:	0.02
		N. weight:	0.71
		Sc. PD:	-0.026
		Sc. rank:	-3102.4

,

NP			
Pos.	418	obs:	exp:
tct	S	0	7.42
tcc	S	0	6.87
tca	S	0	12.36
tcg	S	0	1.81
act	T	1	17.08
acc	T	54	9.72
aca	T	0	23.80
acg	T	0	4.39
aat	N	4703	24021.00
aac	N	38233	18915.00
agt	S	10	8.04
agc	S	36	9.49
gat	D	2	1.70
gac	D	1	1.30
---	--	------	------
mPD		0.20	0.50
		nPD:	0.4
		N. weight:	0.47
		Sc. PD:	0.16
		Sc. rank:	15930.0

,

NP			
Pos.	419	obs:	exp:
tta	L	0	3685.00
ttg	L	0	6565.00
ctt	L	141	6643.00
ctc	L	31537	4656.00
cta	L	76	4876.00
ctg	L	0	5329.00
att	I	21	3960.00
atc	I	11256	2964.00
ata	I	5	4357.00
gtt	V	0	1.00
gtc	V	4	0.82
gta	V	0	0.87
gtg	V	0	1.32
---	--	------	------
mPD		0.40	1.4
		nPD:	0.29
		N. weight:	1.8
		Sc. PD:	0.41
		Sc. rank:	58842.0

,

NP			
Pos.	420	obs:	exp:
cct	P	132	14343.00
ccc	P	15	8069.00
cca	P	42826	14541.00
ccg	P	67	6087.00
---	--	------	------
mPD		0.0099	0.72
		nPD:	0.01
		N. weight:	1.1
		Sc. PD:	-0.044
		Sc. rank:	-10410.0

,

NP			
Pos.	421	obs:	exp:
ttt	F	42914	20935.00
ttc	F	124	22103.00
tgt	C	2	0.78
tgc	C	0	1.22
---	--	------	------
mPD		0.0058	0.50
		nPD:	0.01
		N. weight:	0.73
		Sc. PD:	-0.031
		Sc. rank:	-10394.0

,

NP			
Pos.	422	obs:	exp:
gat	D	256	503.10
gac	D	634	386.90
gaa	E	41909	24850.00
gag	E	241	17300.00
---	--	------	------
mPD		0.052	0.50
		nPD:	0.1
		N. weight:	0.51
		Sc. PD:	0.023
		Sc. rank:	13089.0

,

NP			
Pos.	423	obs:	exp:
caa	Q	0	0.61
cag	Q	1	0.39
cgt	R	0	2.58
cgc	R	0	4.68
cga	R	104	11.86
cgg	R	0	11.16
atg	M	1	1.00
aaa	K	370	28209.00
aag	K	42538	14699.00
aga	R	25	63.76
agg	R	1	35.96
---	--	------	------
mPD		0.034	0.46
		nPD:	0.07
		N. weight:	1.1
		Sc. PD:	0.019
		Sc. rank:	23901.0

,

NP			
Pos.	424	obs:	exp:
tta	L	2	0.23
ttg	L	0	0.41
tct	S	15	6915.00
tcc	S	0	6404.00
tca	S	42747	11517.00
tcg	S	89	1687.00
ctt	L	0	0.42
ctc	L	0	0.29
cta	L	0	0.31
ctg	L	0	0.34
cct	P	0	13.66
ccc	P	0	7.69
cca	P	40	13.85
ccg	P	1	5.80
act	T	0	4.97
acc	T	0	2.83
aca	T	16	6.92
acg	T	0	1.28
agt	S	0	7488.00
agc	S	0	8840.00
gct	A	0	35.43
gcc	A	0	32.48
gca	A	130	52.59
gcg	A	0	9.50
---	--	------	------
mPD		0.014	1.6
		nPD:	0.01
		N. weight:	1.3
		Sc. PD:	-0.061
		Sc. rank:	-24630.0

,

NP			
Pos.	425	obs:	exp:
tct	S	0	0.16
tcc	S	0	0.15
tca	S	0	0.27
tcg	S	0	0.04
att	I	0	13.34
atc	I	38	9.98
ata	I	0	14.68
act	T	1119	13354.00
acc	T	41800	7602.00
aca	T	81	18609.00
acg	T	0	3434.00
agt	S	0	0.17
agc	S	1	0.21
gct	A	0	0.27
gcc	A	1	0.25
gca	A	0	0.40
gcg	A	0	0.07
---	--	------	------
mPD		0.056	0.68
		nPD:	0.08
		N. weight:	1.6
		Sc. PD:	0.043
		Sc. rank:	38597.0

,

NP			
Pos.	426	obs:	exp:
att	I	147	14927.00
atc	I	42261	11173.00
ata	I	114	16422.00
gtt	V	147	129.70
gtc	V	371	105.50
gta	V	0	112.30
gtg	V	0	170.40
---	--	------	------
mPD		0.043	0.69
		nPD:	0.06
		N. weight:	1.3
		Sc. PD:	0.009
		Sc. rank:	27852.0

,

NP			
Pos.	427	obs:	exp:
tta	L	4	12.18
ttg	L	101	21.71
ctt	L	0	21.96
ctc	L	0	15.40
cta	L	0	16.12
ctg	L	0	17.62
att	I	0	2.46
atc	I	0	1.84
ata	I	7	2.70
atg	M	42928	42928.00
---	--	------	------
mPD		0.0054	0.0082
		nPD:	0.65
		N. weight:	0.0038
		Sc. PD:	0.0022
		Sc. rank:	136.7

,

NP			
Pos.	428	obs:	exp:
gtt	V	0	0.50
gtc	V	0	0.41
gta	V	2	0.43
gtg	V	0	0.66
gct	A	23	11730.00
gcc	A	41	10753.00
gca	A	42655	17409.00
gcg	A	319	3146.00
---	--	------	------
mPD		0.018	0.69
		nPD:	0.03
		N. weight:	0.89
		Sc. PD:	-0.026
		Sc. rank:	3601.5

,

NP

Pos.	429	obs:	exp:
act	T	0	1.55
acc	T	0	0.88
aca	T	5	2.16
acg	T	0	0.40
gtt	V	0	0.25
gtc	V	0	0.20
gta	V	1	0.22
gtg	V	0	0.33
gct	A	117	11729.00
gcc	A	1	10752.00
gca	A	42885	17407.00
gcg	A	31	3146.00
---	--	------	------
mPD		0.0072	0.69
		nPD:	0.01
		N. weight:	0.92
		Sc. PD:	-0.04
		Sc. rank:	-14630.0

,

NP

Pos.	430	obs:	exp:
ttt	F	115	20935.00
ttc	F	42923	22103.00
tta	L	0	0.23
ttg	L	0	0.41
ctt	L	0	0.42
ctc	L	2	0.29
cta	L	0	0.31
ctg	L	0	0.34
---	--	------	------
mPD		0.0054	0.50
		nPD:	0.01
		N. weight:	0.67
		Sc. PD:	-0.029
		Sc. rank:	-10249.0

,

NP

Pos.	431	obs:	exp:
tct	S	0	18.56
tcc	S	0	17.19
tca	S	0	30.91
tcg	S	0	4.53
att	I	31	10.88
atc	I	0	8.15
ata	I	0	11.97
act	T	42661	13306.00
acc	T	151	7575.00
aca	T	35	18543.00
acg	T	0	3422.00
aat	N	38	22.94
aac	N	3	18.06
agt	S	7	20.10
agc	S	108	23.72
gct	A	6	1.64
gcc	A	0	1.50
gca	A	0	2.43
gcg	A	0	0.44
---	--	------	------
mPD		0.023	0.69
		nPD:	0.03
		N. weight:	1.2
		Sc. PD:	-0.025
		Sc. rank:	12554.0

,

NP

Pos.	432	obs:	exp:
cgt	R	0	0.02
cgc	R	0	0.04
cga	R	0	0.09
cgg	R	0	0.09
aga	R	1	0.49
agg	R	0	0.28
ggt	G	3	5794.00
ggc	G	0	6007.00
gga	G	42754	19867.00
ggg	G	281	11370.00
---	-	1	1.00
---	--	------	------
mPD		0.013	0.68
		nPD:	0.02
		N. weight:	0.76
		Sc. PD:	-0.026
		Sc. rank:	-1257.6

,

NP

Pos.	433	obs:	exp:
tct	S	0	2.26
tcc	S	0	2.09
tca	S	0	3.76
tcg	S	0	0.55
tat	Y	1	0.53
tac	Y	0	0.47
cat	H	4	2.26
cac	H	0	1.74
act	T	5	1.55
acc	T	0	0.88
aca	T	0	2.16
acg	T	0	0.40
aat	N	42554	24065.00
aac	N	460	18949.00
agt	S	14	2.45
agc	S	0	2.89
gat	D	1	0.57
gac	D	0	0.43
---	-	1	1.00
---	--	------	------
mPD		0.022	0.49
		nPD:	0.05
		N. weight:	0.54
		Sc. PD:	-0.0053
		Sc. rank:	8894.8

,

NP

Pos.	434	obs:	exp:
tct	S	0	0.65
tcc	S	0	0.60
tca	S	0	1.08
tcg	S	4	0.16
att	I	0	0.70
atc	I	0	0.53
ata	I	2	0.77
atg	M	40	40.00
act	T	116	13304.00
acc	T	0	7574.00
aca	T	1659	18540.00
acg	T	41064	3422.00
aat	N	114	63.78
aac	N	0	50.22
aaa	K	0	1.97
aag	K	3	1.03
agt	S	0	0.70
agc	S	0	0.83
gct	A	0	10.36
gcc	A	0	9.49
gca	A	2	15.37
gcg	A	36	2.78
---	--	------	------
mPD		0.094	0.69
		nPD:	0.14
		N. weight:	2.4
		Sc. PD:	0.18
		Sc. rank:	66920.0

,

NP

Pos.	435	obs:	exp:
gat	D	2	1.13
gac	D	0	0.87
gaa	E	3280	25372.00
gag	E	39756	17664.00
ggt	G	0	0.27
ggc	G	0	0.28
gga	G	0	0.92
ggg	G	2	0.53
---	--	------	------
mPD		0.14	0.48
		nPD:	0.29
		N. weight:	0.62
		Sc. PD:	0.14
		Sc. rank:	20060.0

,

NP

Pos.	436	obs:	exp:
ggt	G	13	5794.00
ggc	G	2	6007.00
gga	G	42666	19868.00
ggg	G	359	11371.00
---	--	------	------
mPD		0.017	0.68
		nPD:	0.03
		N. weight:	0.75
		Sc. PD:	-0.022
		Sc. rank:	2936.7

,

NP

Pos.	437	obs:	exp:
cgt	R	0	853.40
cgc	R	0	1549.00
cga	R	4	3927.00
cgg	R	115	3696.00
aga	R	42826	21110.00
agg	R	95	11905.00
---	--	------	------
mPD		0.015	0.89
		nPD:	0.02
		N. weight:	0.71
		Sc. PD:	-0.026
		Sc. rank:	-3272.4

,

NP

Pos.	438	obs:	exp:
tct	S	3	0.48
tcc	S	0	0.45
tca	S	0	0.81
tcg	S	0	0.12
act	T	42373	13364.00
acc	T	497	7608.00
aca	T	156	18624.00
acg	T	7	3437.00
aat	N	3	2.24
aac	N	1	1.76
agt	S	0	0.52
agc	S	0	0.62
---	--	------	------
mPD		0.031	0.68
		nPD:	0.05
		N. weight:	1.1
		Sc. PD:	-0.011
		Sc. rank:	18602.0

,

NP

Pos.	439	obs:	exp:
tct	S	29	6945.00
tcc	S	133	6432.00
tca	S	42861	11568.00
tcg	S	16	1694.00
cct	P	0	0.33
ccc	P	0	0.19
cca	P	1	0.34
ccg	P	0	0.14
agt	S	0	7521.00
agc	S	0	8879.00
---	--	------	------
mPD		0.0083	1.6
		nPD:	0.01
		N. weight:	1.3
		Sc. PD:	-0.065
		Sc. rank:	-31335.0

,

NP

Pos.	440	obs:	exp:
gat	D	164	24329.00
gac	D	42876	18711.00
---	--	------	------
mPD		0.0076	0.49
		nPD:	0.02
		N. weight:	0.84
		Sc. PD:	-0.032
		Sc. rank:	-5249.1

,

NP

Pos.	441	obs:	exp:
atg	M	43040	43040.00
---	--	------	------
mPD		0	0
		nPD:	1.
		N. weight:	0.
		Sc. PD:	0
		Sc. rank:	0

,

NP

Pos.	442	obs:	exp:
cgt	R	0	853.40
cgc	R	0	1549.00
cga	R	116	3927.00
cgg	R	1	3696.00
aga	R	577	21110.00
agg	R	42346	11905.00
---	--	------	------
mPD		0.037	0.89
		nPD:	0.04
		N. weight:	1.2
		Sc. PD:	-0.016
		Sc. rank:	19105.0

,

NP

Pos.	443	obs:	exp:
act	T	0	38.20
acc	T	4	21.75
aca	T	119	53.23
acg	T	0	9.82
gct	A	58	11697.00
gcc	A	5	10722.00
gca	A	42824	17360.00
gcg	A	30	3137.00
---	--	------	------
mPD		0.010	0.70
		nPD:	0.01
		N. weight:	0.92
		Sc. PD:	-0.036
		Sc. rank:	-7591.1

,

NP

Pos.	444	obs:	exp:
gaa	E	42883	25375.00
gag	E	157	17665.00
---	--	------	------
mPD		0.0073	0.48
		nPD:	0.02
		N. weight:	0.52
		Sc. PD:	-0.02
		Sc. rank:	-4013.2

,

NP			
Pos.	445	obs:	exp:
att	I	108	15065.00
atc	I	42755	11276.00
ata	I	51	16573.00
gtt	V	115	31.56
gtc	V	11	25.67
gta	V	0	27.31
gtg	V	0	41.46
---	--	------	------
mPD		0.019	0.67
		nPD:	0.03
		N. weight:	1.4
		Sc. PD:	-0.036
		Sc. rank:	8508.9

,

NP			
Pos.	446	obs:	exp:
att	I	4	15108.00
atc	I	10	11309.00
ata	I	43025	16622.00
act	T	0	0.31
acc	T	0	0.18
aca	T	1	0.43
acg	T	0	0.08
---	--	------	------
mPD		0.00070	0.66
		nPD:	0.
		N. weight:	0.98
		Sc. PD:	-0.051
		Sc. rank:	-33259.0

,

NP			
Pos.	447	obs:	exp:
tct	S	0	0.16
tcc	S	0	0.15
tca	S	0	0.27
tcg	S	0	0.04
cgt	R	0	852.60
cgc	R	0	1547.00
cga	R	0	3923.00
cgg	R	4	3692.00
aaa	K	3	23.67
aag	K	33	12.33
agt	S	1	0.17
agc	S	0	0.21
aga	R	2981	21092.00
agg	R	40017	11894.00
ggt	G	0	0.13
ggc	G	0	0.14
gga	G	1	0.46
ggg	G	0	0.26
---	--	------	------
mPD		0.13	0.89
		nPD:	0.15
		N. weight:	1.
		Sc. PD:	0.092
		Sc. rank:	30177.0

,

NP			
Pos.	448	obs:	exp:
tta	L	0	0.81
ttg	L	0	1.45
ctt	L	0	1.46
ctc	L	0	1.03
cta	L	0	1.07
ctg	L	7	1.17
att	I	3	2.81
atc	I	0	2.10
ata	I	5	3.09
atg	M	43021	43021.00
act	T	0	0.31
acc	T	0	0.18
aca	T	0	0.43
acg	T	1	0.08
gtt	V	0	0.75
gtc	V	0	0.61
gta	V	0	0.65
gtg	V	3	0.99
---	--	------	------
mPD		0.00088	0.0012
		nPD:	0.72
		N. weight:	0.00054
		Sc. PD:	0.00035
		Sc. rank:	19.2

,

NP			
Pos.	449	obs:	exp:
atg	M	43040	43040.00
---	--	------	------
mPD		0	0
		nPD:	1.
		N. weight:	0.
		Sc. PD:	0
		Sc. rank:	0

,

NP			
Pos.	450	obs:	exp:
gaa	E	42971	25375.00
gag	E	69	17665.00
---	--	------	------
mPD		0.0032	0.48
		nPD:	0.01
		N. weight:	0.54
		Sc. PD:	-0.025
		Sc. rank:	-10603.0

,

NP			
Pos.	451	obs:	exp:
tct	S	0	472.20
tcc	S	0	437.30
tca	S	0	786.50
tcg	S	0	115.20
aat	N	47	26.30
aac	N	0	20.70
agt	S	2925	511.30
agc	S	1	603.60
gtt	V	6	1.50
gtc	V	0	1.22
gta	V	0	1.30
gtg	V	0	1.97
gat	D	140	79.14
gac	D	0	60.86
ggt	G	39879	5374.00
ggc	G	10	5572.00
gga	G	26	18428.00
ggg	G	6	10547.00
---	--	------	------
mPD		0.14	0.91
		nPD:	0.15
		N. weight:	2.
		Sc. PD:	0.19
		Sc. rank:	61054.0

,

NP			
Pos.	452	obs:	exp:
tct	S	0	1.29
tcc	S	0	1.20
tca	S	8	2.15
tcg	S	0	0.31
act	T	0	11.18
acc	T	0	6.36
aca	T	36	15.58
acg	T	0	2.88
agt	S	0	1.40
agc	S	0	1.65
gtt	V	0	0.50
gtc	V	0	0.41
gta	V	2	0.43
gtg	V	0	0.66
gct	A	3	11718.00
gcc	A	4	10742.00
gca	A	42973	17391.00
gcg	A	14	3143.00
---	--	------	------
mPD		0.0031	0.70
		nPD:	0.
		N. weight:	0.93
		Sc. PD:	-0.046
		Sc. rank:	-23844.0

,

NP			
Pos.	453	obs:	exp:
caa	Q	3	1.83
cag	Q	0	1.17
cgt	R	0	0.44
cgc	R	0	0.79
cga	R	0	2.01
cgg	R	0	1.89
att	I	0	0.35
atc	I	0	0.26
ata	I	1	0.39
act	T	0	0.62
acc	T	0	0.35
aca	T	2	0.87
acg	T	0	0.16
aat	N	0	3.36
aac	N	6	2.64
aaa	K	42684	28273.00
aag	K	321	14732.00
aga	R	21	10.79
agg	R	1	6.09
gaa	E	1	0.59
gag	E	0	0.41
---	--	------	------
mPD		0.016	0.45
		nPD:	0.04
		N. weight:	0.39
		Sc. PD:	-0.0072
		Sc. rank:	5094.3

,

NP			
Pos.	454	obs:	exp:
tta	L	0	0.70
ttg	L	0	1.24
tct	S	0	9.20
tcc	S	0	8.52
tca	S	48	15.32
tcg	S	9	2.24
ctt	L	0	1.26
ctc	L	0	0.88
cta	L	6	0.92
ctg	L	0	1.01
cct	P	4	14312.00
ccc	P	7	8051.00
cca	P	42573	14510.00
ccg	P	363	6074.00
caa	Q	16	9.78
cag	Q	0	6.22
act	T	0	1.86
acc	T	0	1.06
aca	T	6	2.60
acg	T	0	0.48
agt	S	0	9.96
agc	S	0	11.76
gct	A	0	2.18
gcc	A	0	2.00
gca	A	8	3.24
gcg	A	0	0.58
---	--	------	------
mPD		0.022	0.73
		nPD:	0.03
		N. weight:	1.1
		Sc. PD:	-0.026
		Sc. rank:	9835.0

,

NP			
Pos.	455	obs:	exp:
gat	D	5	6.22
gac	D	6	4.78
gaa	E	42886	25363.00
gag	E	134	17657.00
ggt	G	0	1.21
ggc	G	0	1.26
gga	G	9	4.15
ggg	G	0	2.38
---	--	------	------
mPD		0.0071	0.48
		nPD:	0.01
		N. weight:	0.53
		Sc. PD:	-0.021
		Sc. rank:	-4196.9

,

NP			
Pos.	456	obs:	exp:
gat	D	119	70.66
gac	D	6	54.34
gaa	E	42901	25301.00
gag	E	14	17614.00
---	--	------	------
mPD		0.0064	0.49
		nPD:	0.01
		N. weight:	0.54
		Sc. PD:	-0.022
		Sc. rank:	-5682.7

,

NP			
Pos.	457	obs:	exp:
tta	L	0	14.97
ttg	L	127	26.67
ctt	L	0	26.99
ctc	L	0	18.92
cta	L	0	19.81
ctg	L	2	21.65
att	I	8	3.51
atc	I	0	2.63
ata	I	2	3.86
atg	M	120	120.00
gtt	V	2151	10716.00
gtc	V	228	8717.00
gta	V	66	9272.00
gtg	V	40336	14076.00
---	--	------	------
mPD		0.12	0.75
		nPD:	0.16
		N. weight:	0.92
		Sc. PD:	0.093
		Sc. rank:	27921.0

,

NP			
Pos.	458	obs:	exp:
tct	S	209	6945.00
tcc	S	370	6432.00
tca	S	42227	11568.00
tcg	S	233	1694.00
agt	S	0	7521.00
agc	S	0	8879.00
gct	A	0	0.27
gcc	A	0	0.25
gca	A	1	0.40
gcg	A	0	0.07
---	--	------	------
mPD		0.037	1.6
		nPD:	0.02
		N. weight:	1.3
		Sc. PD:	-0.039
		Sc. rank:	2340.8

,

NP			
Pos.	459	obs:	exp:
ttt	F	310	20936.00
ttc	F	42730	22104.00
---	--	------	------
mPD		0.014	0.50
		nPD:	0.03
		N. weight:	0.65
		Sc. PD:	-0.017
		Sc. rank:	4365.1

,

NP			
Pos.	460	obs:	exp:
tta	L	0	0.12
ttg	L	0	0.21
tgg	W	2	2.00
ctt	L	0	0.21
ctc	L	0	0.15
cta	L	0	0.15
ctg	L	1	0.17
caa	Q	0	97.80
cag	Q	160	62.20
cgt	R	394	850.00
cgc	R	2	1542.00
cga	R	530	3911.00
cgg	R	41284	3681.00
aaa	K	0	4.60
aag	K	7	2.40
aga	R	443	21027.00
agg	R	216	11858.00
ggt	G	0	0.13
ggc	G	0	0.14
gga	G	0	0.46
ggg	G	1	0.26
---	--	------	------
mPD		0.10	0.90
		nPD:	0.11
		N. weight:	2.3
		Sc. PD:	0.13
		Sc. rank:	62463.0

,

NP			
Pos.	461	obs:	exp:
gaa	E	0	1.18
gag	E	2	0.82
ggt	G	5	5794.00
ggc	G	0	6007.00
gga	G	818	19867.00
ggg	G	42215	11370.00
---	--	------	------
mPD		0.038	0.68
		nPD:	0.06
		N. weight:	1.3
		Sc. PD:	0.00021
		Sc. rank:	24012.0

,

NP			
Pos.	462	obs:	exp:
cgt	R	0	826.90
cgc	R	0	1501.00
cga	R	1	3805.00
cgg	R	552	3581.00
aaa	K	0	878.30
aag	K	1336	457.70
aga	R	145	20455.00
agg	R	41006	11535.00
---	--	------	------
mPD		0.092	0.94
		nPD:	0.1
		N. weight:	1.2
		Sc. PD:	0.052
		Sc. rank:	31819.0

,

NP			
Pos.	463	obs:	exp:
ggt	G	8	5794.00
ggc	G	2	6007.00
gga	G	43000	19868.00
ggg	G	30	11371.00
---	--	------	------
mPD		0.0019	0.68
		nPD:	0.
		N. weight:	0.79
		Sc. PD:	-0.04
		Sc. rank:	-23741.0

,

NP			
Pos.	464	obs:	exp:
att	I	2	0.70
atc	I	0	0.53
ata	I	0	0.77
gtt	V	42295	10780.00
gtc	V	736	8769.00
gta	V	0	9328.00
gtg	V	7	14160.00
---	--	------	------
mPD		0.034	0.74
		nPD:	0.05
		N. weight:	1.3
		Sc. PD:	-0.012
		Sc. rank:	22515.0

,

NP			
Pos.	465	obs:	exp:
ttt	F	0	20936.00
ttc	F	43040	22104.00
---	--	------	------
mPD		0	0.50
		nPD:	0.
		N. weight:	0.69
		Sc. PD:	-0.037
		Sc. rank:	-24830.0

,

NP			
Pos.	466	obs:	exp:
gaa	E	0	25373.00
gag	E	43037	17664.00
ggt	G	0	0.40
ggc	G	0	0.42
gga	G	0	1.38
ggg	G	3	0.79
---	--	------	------
mPD		0.00014	0.48
		nPD:	0.
		N. weight:	0.92
		Sc. PD:	-0.049
		Sc. rank:	-32282.0

,

NP			
Pos.	467	obs:	exp:
ttt	F	0	2.43
ttc	F	5	2.57
tta	L	0	4994.00
ttg	L	0	8897.00
ctt	L	60	9002.00
ctc	L	42911	6311.00
cta	L	64	6608.00
ctg	L	0	7222.00
---	--	------	------
mPD		0.0060	1.2
		nPD:	0.01
		N. weight:	2.
		Sc. PD:	-0.095
		Sc. rank:	-45518.0

,

NP			
Pos.	468	obs:	exp:
tta	L	9	1.04
ttg	L	0	1.86
tct	S	462	6943.00
tcc	S	3	6430.00
tca	S	42365	11565.00
tcg	S	197	1694.00
ctt	L	0	1.88
ctc	L	0	1.32
cta	L	0	1.38
ctg	L	0	1.51
act	T	0	0.93
acc	T	0	0.53
aca	T	3	1.30
acg	T	0	0.24
agt	S	0	7519.00
agc	S	0	8876.00
gct	A	0	0.27
gcc	A	0	0.25
gca	A	1	0.40
gcg	A	0	0.07
---	--	------	------
mPD		0.031	1.6
		nPD:	0.02
		N. weight:	1.3
		Sc. PD:	-0.045
		Sc. rank:	-2657.1

,

NP			
Pos.	469	obs:	exp:
gat	D	56	24328.00
gac	D	42983	18711.00
gaa	E	1	0.59
gag	E	0	0.41
---	--	------	------
mPD		0.0026	0.49
		nPD:	0.01
		N. weight:	0.85
		Sc. PD:	-0.041
		Sc. rank:	-19335.0

,

NP			
Pos.	470	obs:	exp:
aaa	K	0	1.31
aag	K	2	0.69
gtt	V	0	0.25
gtc	V	0	0.20
gta	V	0	0.22
gtg	V	1	0.33
gat	D	1	0.57
gac	D	0	0.43
gaa	E	468	25371.00
gag	E	42566	17663.00
ggt	G	0	0.27
ggc	G	0	0.28
gga	G	1	0.92
ggg	G	1	0.53
---	--	------	------
mPD		0.022	0.48
		nPD:	0.05
		N. weight:	0.86
		Sc. PD:	-0.0084
		Sc. rank:	13947.0

,

NP			
Pos.	471	obs:	exp:
cgt	R	0	0.67
cgc	R	0	1.22
cga	R	0	3.10
cgg	R	0	2.92
aaa	K	83	28274.00
aag	K	42923	14732.00
aga	R	0	16.68
agg	R	34	9.40
---	--	------	------
mPD		0.0054	0.45
		nPD:	0.01
		N. weight:	1.1
		Sc. PD:	-0.046
		Sc. rank:	-14594.0

,

NP			
Pos.	472	obs:	exp:
gct	A	3	11731.00
gcc	A	2	10753.00
gca	A	42984	17410.00
gcg	A	51	3146.00
---	--	------	------
mPD		0.0026	0.69
		nPD:	0.
		N. weight:	0.93
		Sc. PD:	-0.046
		Sc. rank:	-25692.0

,

NP

Pos.	473	obs:	exp:
tct	S	0	2.42
tcc	S	0	2.24
tca	S	0	4.03
tcg	S	15	0.59
act	T	0	3405.00
acc	T	0	1939.00
aca	T	0	4745.00
acg	T	10965	875.80
agt	S	0	2.62
agc	S	0	3.09
gtt	V	0	0.25
gtc	V	0	0.20
gta	V	0	0.22
gtg	V	1	0.33
gct	A	0	8738.00
gcc	A	0	8010.00
gca	A	1	12968.00
gcg	A	32058	2344.00
---	--	------	------
mPD		0.38	1.1
		nPD:	0.35
		N. weight:	2.7
		Sc. PD:	0.78
		Sc. rank:	90278.0

,

NP

Pos.	474	obs:	exp:
tct	S	0	18.40
tcc	S	0	17.04
tca	S	0	30.64
tcg	S	0	4.49
cat	H	0	0.56
cac	H	1	0.44
act	T	0	0.62
acc	T	2	0.35
aca	T	0	0.87
acg	T	0	0.16
aat	N	0	24014.00
aac	N	42923	18909.00
agt	S	0	19.92
agc	S	114	23.52
---	--	------	------
mPD		0.0054	0.50
		nPD:	0.01
		N. weight:	0.85
		Sc. PD:	-0.037
		Sc. rank:	-13165.0

,

NP

Pos.	475	obs:	exp:
tct	S	0	0.32
tcc	S	0	0.30
tca	S	0	0.54
tcg	S	2	0.08
cct	P	1	14342.00
ccc	P	0	8068.00
cca	P	0	14541.00
ccg	P	43037	6087.00
agt	S	0	0.35
agc	S	0	0.41
---	--	------	------
mPD		0.00014	0.72
		nPD:	0.
		N. weight:	2.
		Sc. PD:	-0.11
		Sc. rank:	-71757.0

,

NP

Pos.	476	obs:	exp:
tta	L	0	0.12
ttg	L	0	0.21
ctt	L	0	0.21
ctc	L	1	0.15
cta	L	0	0.15
ctg	L	0	0.17
att	I	0	15101.00
atc	I	43016	11303.00
ata	I	1	16613.00
act	T	0	0.93
acc	T	3	0.53
aca	T	0	1.30
acg	T	0	0.24
gtt	V	0	4.76
gtc	V	19	3.87
gta	V	0	4.12
gtg	V	0	6.25
---	--	------	------
mPD		0.0011	0.66
		nPD:	0.
		N. weight:	1.4
		Sc. PD:	-0.072
		Sc. rank:	-45468.0

,

NP

Pos.	477	obs:	exp:
gtt	V	19	10780.00
gtc	V	0	8769.00
gta	V	2	9328.00
gtg	V	43016	14160.00
gct	A	0	0.82
gcc	A	0	0.75
gca	A	0	1.21
gcg	A	3	0.22
---	--	------	------
mPD		0.0011	0.74
		nPD:	0.
		N. weight:	1.1
		Sc. PD:	-0.06
		Sc. rank:	-37912.0

,

NP

Pos.	478	obs:	exp:
cct	P	288	14343.00
ccc	P	42752	8069.00
cca	P	0	14541.00
ccg	P	0	6087.00
---	--	------	------
mPD		0.013	0.72
		nPD:	0.02
		N. weight:	1.7
		Sc. PD:	-0.06
		Sc. rank:	-5183.8

,

NP

Pos.	479	obs:	exp:
tct	S	42890	6944.00
tcc	S	136	6431.00
tca	S	5	11567.00
tcg	S	3	1694.00
cct	P	5	1.67
ccc	P	0	0.94
cca	P	0	1.69
ccg	P	0	0.71
act	T	1	0.31
acc	T	0	0.18
aca	T	0	0.43
acg	T	0	0.08
agt	S	0	7520.00
agc	S	0	8878.00
---	--	------	------
mPD		0.0069	1.6
		nPD:	0.
		N. weight:	1.9
		Sc. PD:	-0.092
		Sc. rank:	-48971.0

,

NP

Pos.	480	obs:	exp:
ttt	F	43007	20925.00
ttc	F	10	22092.00
tta	L	3	1.74
ttg	L	8	3.10
tat	Y	8	4.22
tac	Y	0	3.78
ctt	L	4	3.14
ctc	L	0	2.20
cta	L	0	2.30
ctg	L	0	2.52
---	--	------	------
mPD		0.0015	0.50
		nPD:	0.
		N. weight:	0.75
		Sc. PD:	-0.038
		Sc. rank:	-21572.0

,

NP

Pos.	481	obs:	exp:
tat	Y	2	1.05
tac	Y	0	0.95
aat	N	32	18.46
aac	N	1	14.54
gat	D	42055	24223.00
gac	D	797	18629.00
gaa	E	141	88.43
gag	E	9	61.57
ggt	G	3	0.40
ggc	G	0	0.42
gga	G	0	1.38
ggg	G	0	0.79
---	--	------	------
mPD		0.045	0.50
		nPD:	0.09
		N. weight:	0.5
		Sc. PD:	0.017
		Sc. rank:	12240.0

,

NP

Pos.	482	obs:	exp:
tta	L	0	3.02
ttg	L	26	5.38
ctt	L	0	5.44
ctc	L	0	3.81
cta	L	0	3.99
ctg	L	0	4.36
cgt	R	0	0.02
cgc	R	0	0.04
cga	R	0	0.09
cgg	R	0	0.09
att	I	0	7.72
atc	I	0	5.78
ata	I	22	8.50
atg	M	42888	42888.00
act	T	0	14.91
acc	T	0	8.49
aca	T	0	20.77
acg	T	48	3.83
aaa	K	0	0.66
aag	K	1	0.34
aga	R	0	0.49
agg	R	1	0.28
gtt	V	0	13.53
gtc	V	0	11.00
gta	V	0	11.70
gtg	V	54	17.77
---	--	------	------
mPD		0.0071	0.012
		nPD:	0.61
		N. weight:	0.0059
		Sc. PD:	0.0032
		Sc. rank:	211.0

,

NP

Pos.	483	obs:	exp:
tct	S	0	6911.00
tcc	S	0	6400.00
tca	S	0	11511.00
tcg	S	0	1686.00
tgt	C	1	0.39
tgc	C	0	0.61
att	I	2	0.70
atc	I	0	0.53
ata	I	0	0.77
act	T	5	1.55
acc	T	0	0.88
aca	T	0	2.16
acg	T	0	0.40
aat	N	204	114.10
aac	N	0	89.87
agt	S	42605	7484.00
agc	S	223	8835.00
---	--	------	------
mPD		0.020	1.6
		nPD:	0.01
		N. weight:	1.8
		Sc. PD:	-0.073
		Sc. rank:	-22383.0

,

NP

Pos.	484	obs:	exp:
att	I	0	0.35
atc	I	1	0.26
ata	I	0	0.39
act	T	0	0.31
acc	T	1	0.18
aca	T	0	0.43
acg	T	0	0.08
aat	N	6198	24072.00
aac	N	36829	18955.00
aaa	K	4	2.63
aag	K	0	1.37
gat	D	1	3.96
gac	D	6	3.04
---	--	------	------
mPD		0.25	0.49
		nPD:	0.5
		N. weight:	0.39
		Sc. PD:	0.17
		Sc. rank:	13464.0

,

NP			
Pos.	485	obs:	exp:
gat	D	0	0.57
gac	D	1	0.43
gaa	E	42993	25374.00
gag	E	45	17664.00
ggt	G	0	0.13
ggc	G	0	0.14
gga	G	1	0.46
ggg	G	0	0.26
---	--	------	------
mPD		0.0022	0.48
		nPD:	0.
		N. weight:	0.54
		Sc. PD:	-0.026
		Sc. rank:	-13619.0

NP			
Pos.	486	obs:	exp:
cgt	R	0	0.02
cgc	R	0	0.04
cga	R	0	0.09
cgg	R	0	0.09
aga	R	1	0.49
agg	R	0	0.28
gtt	V	0	0.50
gtc	V	0	0.41
gta	V	2	0.43
gtg	V	0	0.66
gaa	E	6	3.54
gag	E	0	2.46
ggt	G	104	5793.00
ggc	G	0	6006.00
gga	G	42695	19864.00
ggg	G	232	11368.00
---	--	------	------
mPD		0.016	0.68
		nPD:	0.02
		N. weight:	0.76
		Sc. PD:	-0.023
		Sc. rank:	1719.2

NP			
Pos.	487	obs:	exp:
tct	S	43010	6945.00
tcc	S	21	6432.00
tca	S	2	11568.00
tcg	S	5	1694.00
tat	Y	2	1.05
tac	Y	0	0.95
agt	S	0	7521.00
agc	S	0	8879.00
---	--	------	------
mPD		0.0014	1.6
		nPD:	0.
		N. weight:	1.9
		Sc. PD:	-0.099
		Sc. rank:	-64634.0

NP			
Pos.	488	obs:	exp:
tat	Y	43013	22687.00
tac	Y	27	20353.00
---	--	------	------
mPD		0.0013	0.50
		nPD:	0.
		N. weight:	0.66
		Sc. PD:	-0.034
		Sc. rank:	-20431.0

NP			
Pos.	489	obs:	exp:
ttt	F	8	20936.00
ttc	F	43032	22104.00
---	--	------	------
mPD		0.00037	0.50
		nPD:	0.
		N. weight:	0.69
		Sc. PD:	-0.036
		Sc. rank:	-23945.0

NP			
Pos.	490	obs:	exp:
ttt	F	998	20935.00
ttc	F	42041	22104.00
tta	L	0	0.12
ttg	L	0	0.21
ctt	L	0	0.21
ctc	L	1	0.15
cta	L	0	0.15
ctg	L	0	0.17
---	--	------	------
mPD		0.045	0.50
		nPD:	0.09
		N. weight:	0.58
		Sc. PD:	0.02
		Sc. rank:	14191.0

NP			
Pos.	491	obs:	exp:
ggt	G	0	5794.00
ggc	G	0	6007.00
gga	G	43032	19868.00
ggg	G	8	11371.00
---	--	------	------
mPD		0.00037	0.68
		nPD:	0.
		N. weight:	0.8
		Sc. PD:	-0.042
		Sc. rank:	-27870.0

NP			
Pos.	492	obs:	exp:
gat	D	11	24329.00
gac	D	43029	18711.00
---	--	------	------
mPD		0.00051	0.49
		nPD:	0.
		N. weight:	0.86
		Sc. PD:	-0.045
		Sc. rank:	-29249.0

NP			
Pos.	493	obs:	exp:
tct	S	0	6.13
tcc	S	0	5.68
tca	S	0	10.21
tcg	S	0	1.50
att	I	1	0.35
atc	I	0	0.26
ata	I	0	0.39
act	T	6	1.86
acc	T	0	1.06
aca	T	0	2.60
acg	T	0	0.48
aat	N	42972	24053.00
aac	N	21	18940.00
aaa	K	2	1.31
aag	K	0	0.69
agt	S	38	6.64
agc	S	0	7.84
---	--	------	------
mPD		0.0032	0.50
		nPD:	0.01
		N. weight:	0.6
		Sc. PD:	-0.028
		Sc. rank:	-12000.0

NP			
Pos.	494	obs:	exp:
act	T	0	1.24
acc	T	0	0.71
aca	T	4	1.73
acg	T	0	0.32
gtt	V	0	0.25
gtc	V	0	0.20
gta	V	1	0.22
gtg	V	0	0.33
gct	A	17	11729.00
gcc	A	1	10752.00
gca	A	43000	17408.00
gcg	A	17	3146.00
---	--	------	------
mPD		0.0019	0.69
		nPD:	0.
		N. weight:	0.93
		Sc. PD:	-0.047
		Sc. rank:	-28270.0

NP			
Pos.	495	obs:	exp:
aaa	K	4	2.63
aag	K	0	1.37
gat	D	2	32.79
gac	D	56	25.21
gaa	E	42094	25338.00
gag	E	884	17640.00
---	--	------	------
mPD		0.043	0.49
		nPD:	0.09
		N. weight:	0.45
		Sc. PD:	0.015
		Sc. rank:	10990.0

NP			
Pos.	496	obs:	exp:
gat	D	9	5.09
gac	D	0	3.91
gaa	E	894	25369.00
gag	E	42136	17661.00
ggt	G	0	0.13
ggc	G	0	0.14
gga	G	0	0.46
ggg	G	1	0.26
---	--	------	------
mPD		0.041	0.48
		nPD:	0.08
		N. weight:	0.81
		Sc. PD:	0.023
		Sc. rank:	19232.0

NP			
Pos.	497	obs:	exp:
ttt	F	0	0.49
ttc	F	1	0.51
tat	Y	1944	22685.00
tac	Y	41093	20352.00
tgt	C	0	0.39
tgc	C	1	0.61
aat	N	0	0.56
aac	N	1	0.44
---	--	------	------
mPD		0.086	0.50
		nPD:	0.17
		N. weight:	0.58
		Sc. PD:	0.066
		Sc. rank:	17845.0

NP			
Pos.	498	obs:	exp:
tat	Y	0	1.58
tac	Y	3	1.42
aat	N	0	0.56
aac	N	1	0.44
gat	D	21	24322.00
gac	D	43007	18706.00
gaa	E	5	2.95
gag	E	0	2.05
ggt	G	0	0.40
ggc	G	3	0.42
gga	G	0	1.38
ggg	G	0	0.79
---	--	------	------
mPD		0.0015	0.49
		nPD:	0.
		N. weight:	0.86
		Sc. PD:	-0.043
		Sc. rank:	-24529.0

NP			
Pos.	499	obs:	exp:
tct	S	0	21.62
tcc	S	0	20.02
tca	S	0	36.02
tcg	S	0	5.27
cat	H	5	3.38
cac	H	1	2.62
att	I	5	1.76
atc	I	0	1.31
ata	I	0	1.93
aat	N	42791	23997.00
aac	N	101	18895.00
aaa	K	1	1.97
aag	K	2	1.03
agt	S	134	23.42
agc	S	0	27.64
---	--	------	------
mPD		0.012	0.50
		nPD:	0.02
		N. weight:	0.59
		Sc. PD:	-0.018
		Sc. rank:	1142.2

NP			
Pos.	500	obs:	exp:
taa	.	42717	24450.00
tag	.	54	4714.00
tga	.	269	13876.00
---	--	------	------
mPD		0.015	0.63
		nPD:	0.02
		N. weight:	0.54
		Sc. PD:	-0.017
		Sc. rank:	1401.4

Amino acid level conservation per codon

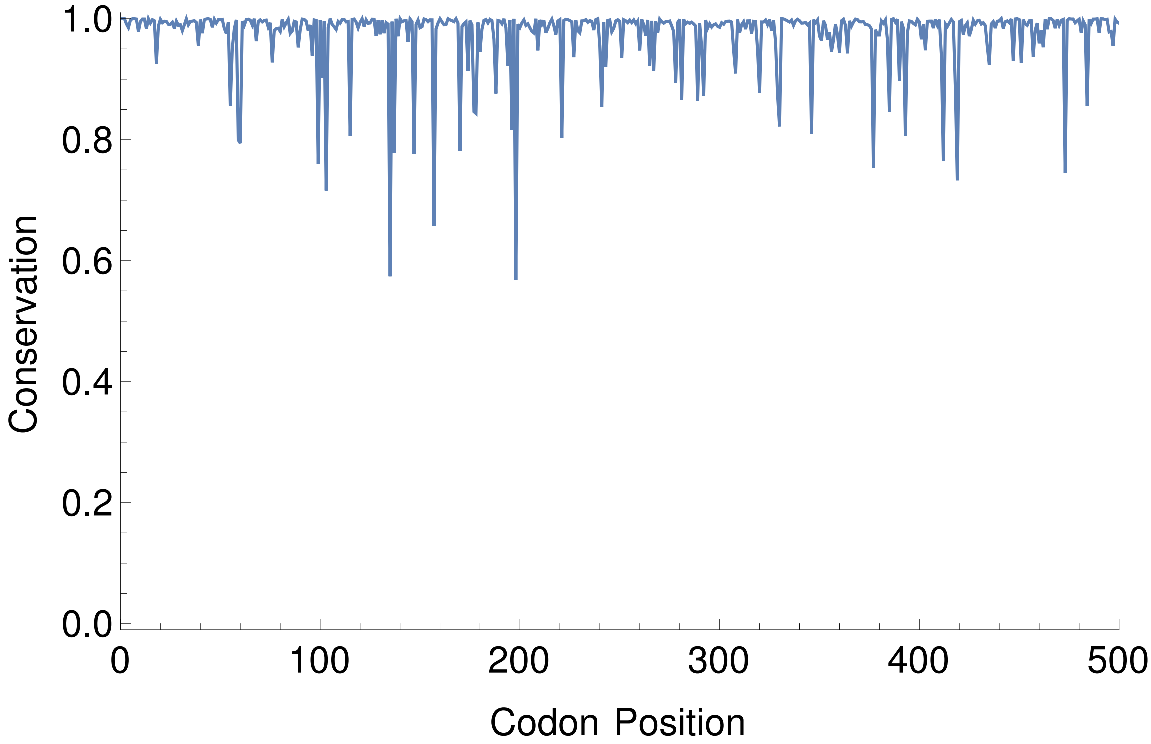

Conservation analysis plots

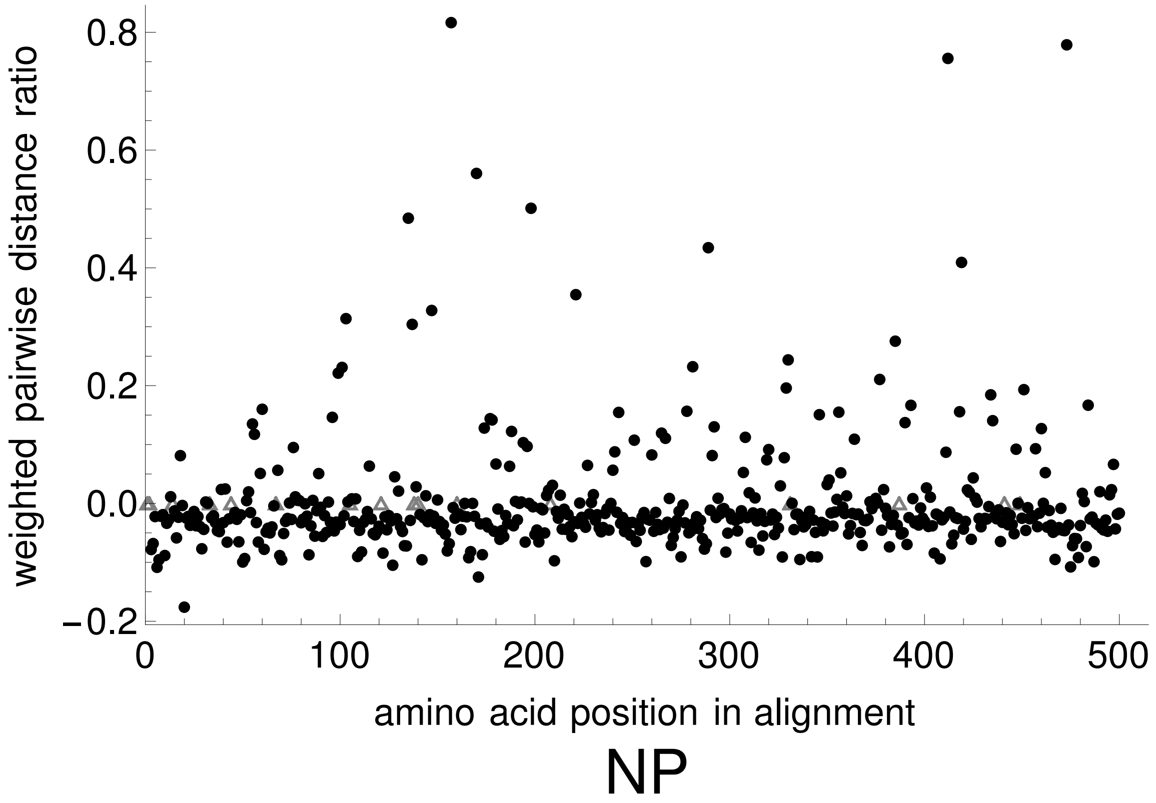

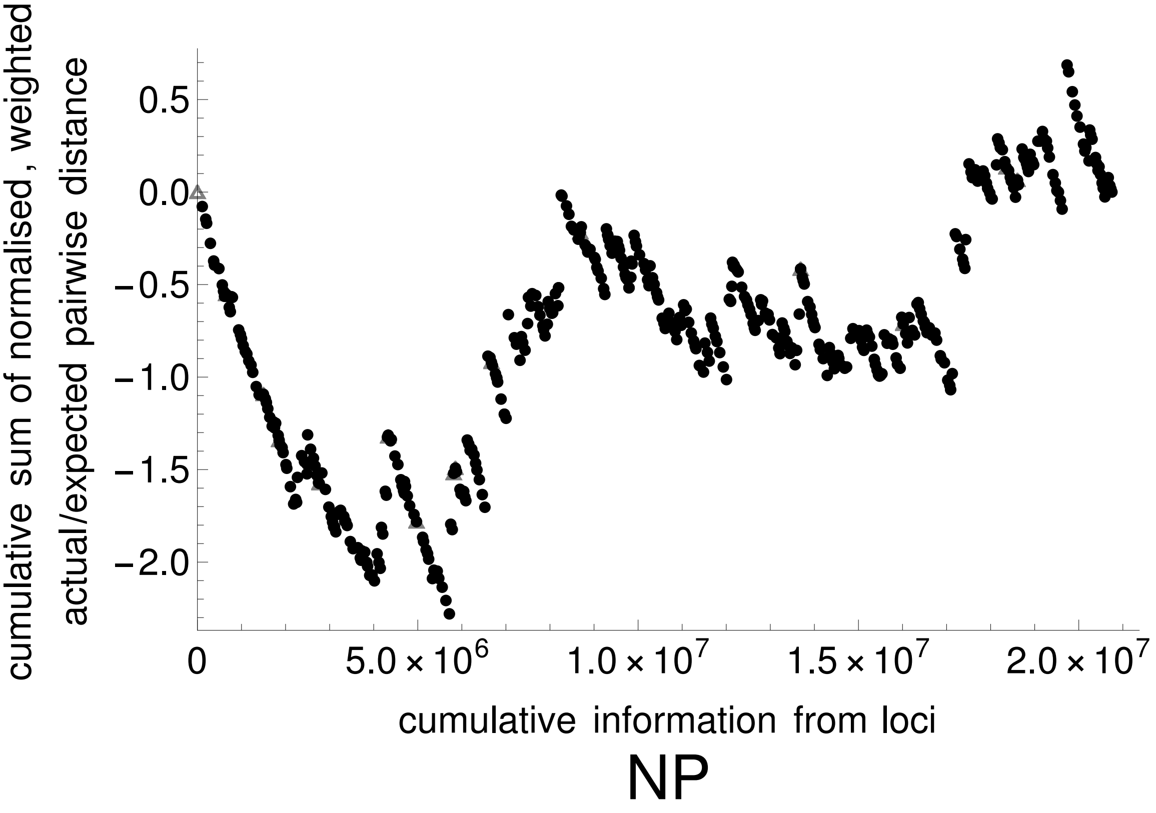

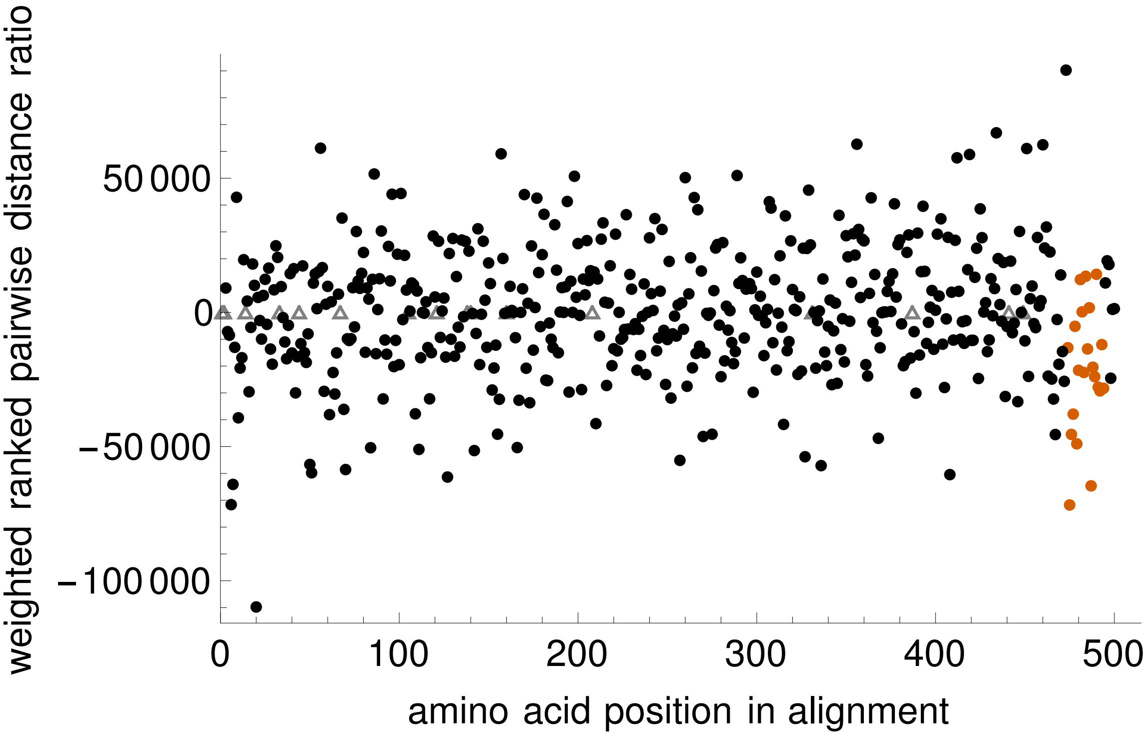
weighted ranked pairwise distance ratio
50 000
0
−50 000
−100 000
0
100
200
300
400
500
amino acid position in alignment

NP

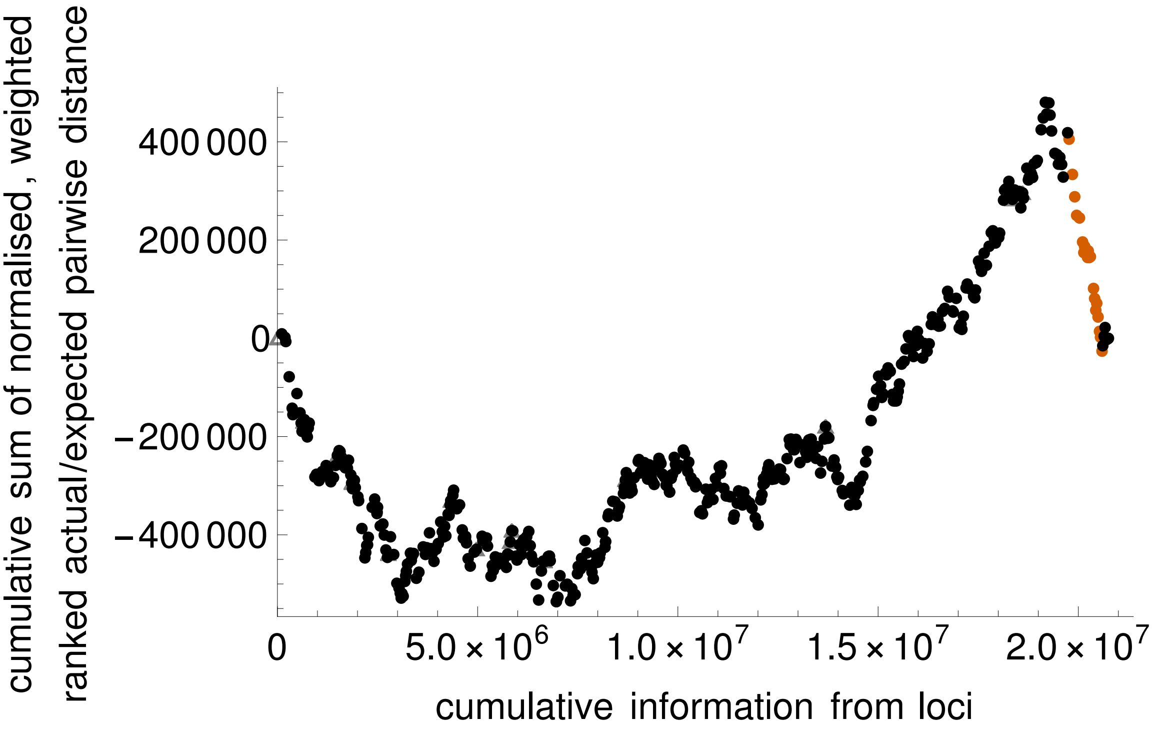
cumulative sum of normalised, weighted
ranked actual/expected pairwise distance
400 000
200 000
0
−200 000
−400 000
0
5.0 × 10^6
1.0 × 10^7
1.5 × 10^7
2.0 × 10^7
cumulative information from loci

NP

NA

Gene length histogram

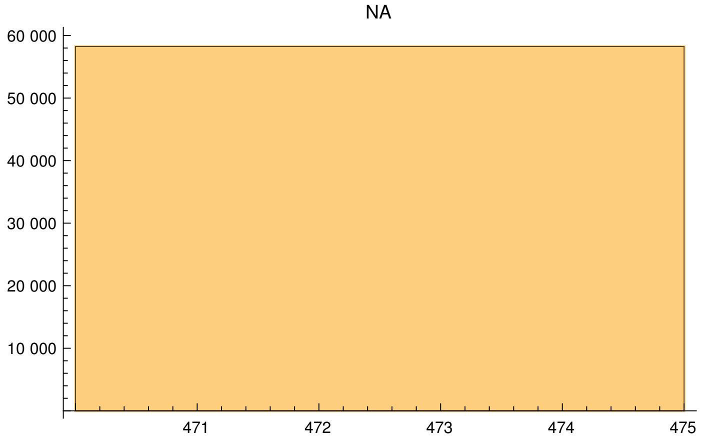

Information vs. nPD

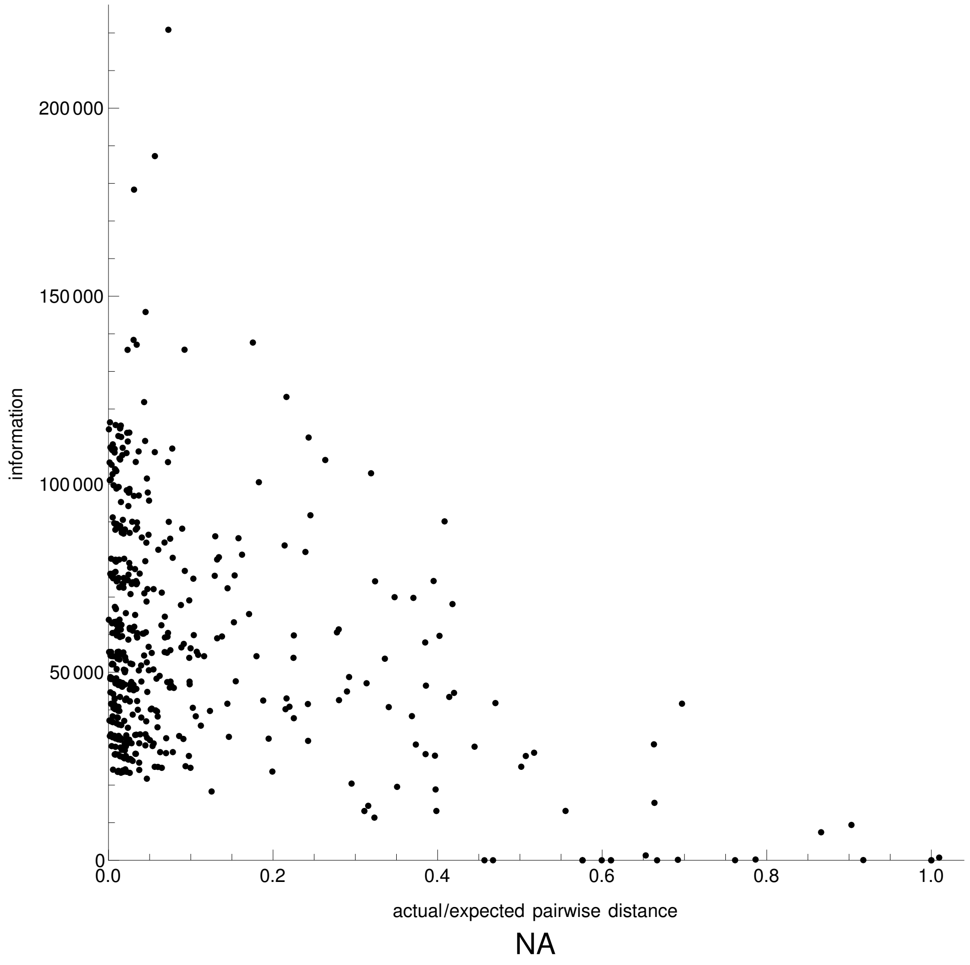

Example sequences highlighted by regions found in analyses to be conserved

Interesting points (by weighted raw PD) highlighted for gene NA:

--------------------------ATGAATCCAAATCAAAAGATAATAACGATTGGCTCTGTTTCTCTCACAATT⋱
TCCATAATATGCTTCTTCATGCAAATTGCCATCCTGATAACTACTGTAACATTGCATTTCAAGCAATATGAATTC⋱
AAC---TCC---CCCCCA------------------------AAT---AACCAAGTGATGCTGTGTGAACCA ---
ACAATA---ATAGAAAGAAACATA ---------ACAGAGATAGTGTATTTGACCAACACCACCATA ------------
----
GAGAAGGAAATATGCCCCAAACCAGCAGAATACAGAAATTGGTCAAAACCGCAATGTGGC ------ATTACAGGAT⋱
TTGCACCTTTCTCTAAGGACAATTCGATTAGGCTTTCCGCTGGTGGGGACATCTGGGTGACAAGAGAACCTTATG⋱
TGTCATGCGATCTTGACAAGTGTTATCAATTTGCCCTTGGACAGGGAACAACACTAAACAATGTGCATTCAAATA⋱
ACACAGTACGTGATAGAACCCCTTATCGGACTCTATTGATGAATGAGTTGGGT ---
GTTCCTTTCCATCTGGGGACCAAGCAAGTGTGCATAGCATGGTCCAGCTCAAGTTGTCACGATGGA ------------
---AAAGCATGGCTGCATGTTTGTATAACGGGGGATGATAAAAATGCAACTGCTAGCTTCATTTACAAT ---
GGGAGGCTTGTAGATAGTGTTGTTTCATGGTCCAACGAT ---ATTCTCAGAACCCAGGAGTCAGAATGCGTTTGTA⋱
TCAATGGAACTTGTACAGTAGTAATGACTGATGGAAATGCTACAGGAAAAGCTGATACTAAAATACTATTCATTG⋱
AGGAGGGGAAAATCGTTCATACTAGCAAATTGTCAGGAAGTGCTCAGCATGTCGAAGAGTGCTCTTGCTATCCTC⋱
GATATCCTGGTGTCAGATGTGTCTGCAGAGACAACTGGAAAGGATCCAACCGGCCCATCATAGATATAAACATAA⋱
AG---GATCATAGCATTGTTTCCAGGTATGTGTGTTCTGGACTTGTTGGAGACACACCCAGAAAAAACGACAGCTC⋱
CAGCAGTAGCCACTGTTTGAACCCTAACAATGAAAAAGGTGATCATGGAGTGAAAGGCTGGGCCTTTGATGATGG⋱
AAATGACGTGTGGATGGGGAGAACAATCAATGAGACGTCACGCTTAGGGTATGAAACCTTCAAAGTCGTTGAAGG⋱
CTGGTCCAACCCTAAGTCCAAATTGCAGATAAATAGGCAAGTCATAGTTGACAGAGGCGATAGGTCCGGTTATTC⋱
TGGTATTTTC ------------TCTGTTGAAGGCAAAAGCTGCATCAATCGGTGCTTTTATGTGGAGTTGATTAGGG⋱
GAAGAAAAGAGGAAACTGAAGTCTTGTGGACTTCAAACAGTATTGTTGTGTTTTGTGGC ---
ACCTCAGGTACATATGGAACAGGCTCATGG ------------------CCTGATGGGGCGAAC ------------
CTCAGT---------CTCATGCATATA ------------------TAA

Interesting points (by weighted ranked PD) highlighted for gene NA:

--------------------------ATGAATCCAAATCAAAAGATAATAACGATTGGCTCTGTTTCTCTCACAATTTCC⋱
ATAATATGCTTCTTCATGCAAATTGCCATCCTGATAACTACTGTAACATTGCATTTCAAGCAATATGAATTCAA⋱
C---
TCC---CCCCCA------------------------AAT---AACCAAGTGATGCTGTGTGAACCA ---ACAATA---
ATAGAAAGAAACATA ---------ACAGAGATAGTGTATTTGACCAACACCACCATA ----------------------
-----------------GAGAAGGAAATATGCCCCAAACCAGCAGAATACAGAAATTGGTCAAAACCGCAATGTGGC --
----ATTACAGGATTTGCACCTTTCTCTAAGGACAATTCGATTAGGCTTTCCGCTGGTGGGGACATCTGGGTGACA⋱
AGAGAACCTTATGTGTCATGCGATCTTGACAAGTGTTATCAATTTGCCCTTGGACAGGGAACAACACTAAACAAT⋱
GTGCATTCAAATAACACAGTACGTGATAGAACCCCTTATCGGACTCTATTGATGAATGAGTTGGGT ---
GTTCCTTTCCATCTGGGGACCAAGCAAGTGTGCATAGCATGG
TCCAGCTCAAGTTGTCACGATGGA----------------AAAGCATGGCTGCA
TGTTTGTATAACGGGGGATGATAAAAATGCAACTGCTAGCTTCATTTACAAT ---
GGGAGGCTTGTAGATAGTGTTGTTTCATGGTCCAACGAT ---ATTCTCAGAACCCAGGAGTCAGAATGCGTTTGTA⋱
TCAATGGAACTTGTACAGTAGTAATGACTGATGGAAATGCTACAGGAAAAGCTGATACTAAAATACTATTCATTG⋱
AGGAGGGGAAAATCGTTCATACTAGCAAATTGTCAGGAAGTGCTCAGCATGTCGAAGAGTGCTCTTGCTATCCTC⋱
GATATCCTGGTGTCAGATGTGTCTGCAGAGACAACTGGAAAGGATCCAACCGGCCCATCATAGATATAAACATAA⋱
AG---GATCATAGCATTGTTTCCAGGTATGTGTGTTCTGGACTTGTTGGAGACACACCCAGAAAAAACGACAGCTC⋱
CAGCAGTAGCCACTGTTTGAACCCTAACAATGAAAAAGGTGATCATGGAGTGAAAGGCTGGGCCTTTGATGATGG⋱

AAATGACGTGTGGATGGGGAGAACAATCAATGAGACGTCACGCTTAGGGTATGAAACCTTCAAAGTCGTTGAAGG˙.
CTGGTCCAACCCTAAGTCCAAATTGCAGATAAATAGGCAAGTCATAGTTGACAGAGGCGATAGGTCCGGTTATTC˙.
TGGTATTTTC ––––––––––––TCTGTTGAAGGCAAAAGCTGCATCAATCGGTGCTTTTATGTGGAGTTGATTAGGG˙.
GAAGAAAAGAGGAAACTGAAGTCTTGTGGACTTCAAACAGTATTGTTGTGTTTTGTGGC ––-ACC
TCAGGTACATATGGAACAGGCTCATGG–––––––––––––––––––CCTGAT
GGGGCGAAC ––––––––––––CTCAGT–––––––––CTCATGCATATA ––––––––––––––––––TAA

Per codon conservation report

NA			
Pos. 1		obs:	exp:
atg	M	10	10.00
---	-	58238	58238.00
---	--	------	------
mPD		0	0
		nPD:	1.
		N. weight:	0.
		Sc. PD:	0
		Sc. rank:	0

NA			
Pos. 2		obs:	exp:
cct	P	0	0.33
ccc	P	0	0.19
cca	P	1	0.34
ccg	P	0	0.14
---	-	58247	58247.00
---	--	------	------
mPD		0	2.1×10^{-10}
		nPD:	0.
		N. weight:	0.
		Sc. PD:	0
		Sc. rank:	0

NA			
Pos. 3		obs:	exp:
gtt	V	0	0.25
gtc	V	0	0.20
gta	V	0	0.22
gtg	V	1	0.33
---	-	58247	58247.00
---	--	------	------
mPD		0	2.2×10^{-10}
		nPD:	0.
		N. weight:	0.
		Sc. PD:	0
		Sc. rank:	0

NA			
Pos. 4		obs:	exp:
tta	L	0	0.12
ttg	L	0	0.21
ctt	L	0	0.21
ctc	L	0	0.15
cta	L	1	0.15
ctg	L	0	0.17
---	-	58247	58247.00
---	--	------	------
mPD		0	3.4×10^{-10}
		nPD:	0.
		N. weight:	0.
		Sc. PD:	0
		Sc. rank:	0

NA			
Pos. 5		obs:	exp:
aaa	K	1	0.66
aag	K	0	0.34
---	-	58247	58247.00
---	--	------	------
mPD		0	1.3×10^{-10}
		nPD:	0.
		N. weight:	0.
		Sc. PD:	0
		Sc. rank:	0

NA			
Pos. 6		obs:	exp:
gct	A	0	0.27
gcc	A	0	0.25
gca	A	1	0.40
gcg	A	0	0.07
---	-	58247	58247.00
---	--	------	------
mPD		0	2.0×10^{-10}
		nPD:	0.
		N. weight:	0.
		Sc. PD:	0
		Sc. rank:	0

NA			
Pos. 7		obs:	exp:
ggt	G	0	0.13
ggc	G	0	0.14
gga	G	1	0.46
ggg	G	0	0.26
---	-	58247	58247.00
---	--	------	------
mPD		0	2.0×10^{-10}
		nPD:	0.
		N. weight:	0.
		Sc. PD:	0
		Sc. rank:	0

NA			
Pos. 8		obs:	exp:
gtt	V	0	0.25
gtc	V	0	0.20
gta	V	0	0.22
gtg	V	1	0.33
---	-	58247	58247.00
---	--	------	------
mPD		0	2.2×10^{-10}
		nPD:	0.
		N. weight:	0.
		Sc. PD:	0
		Sc. rank:	0

NA			
Pos. 9		obs:	exp:
aaa	K	0	0.66
aag	K	1	0.34
---	-	58247	58247.00
---	--	------	------
mPD		0	1.3×10^{-10}
		nPD:	0.
		N. weight:	0.
		Sc. PD:	0
		Sc. rank:	0

NA			
Pos. 10		obs:	exp:
atg	M	58248	58248.00
---	--	------	------
mPD		0	0
		nPD:	1.
		N. weight:	0.
		Sc. PD:	0
		Sc. rank:	0

NA			
Pos. 11		obs:	exp:
tct	S	0	0.16
tcc	S	0	0.15
tca	S	0	0.27
tcg	S	0	0.04
cat	H	4	2.26
cac	H	0	1.74
aat	N	57594	32568.00
aac	N	619	25645.00
aaa	K	9	5.92
aag	K	0	3.08
agt	S	1	0.17
agc	S	0	0.21
gat	D	19	10.74
gac	D	0	8.26
---	-	2	2.00
---	--	------	------
mPD		0.022	0.49
		nPD:	0.04
		N. weight:	0.61
		Sc. PD:	-0.011
		Sc. rank:	8312.3

NA			
Pos. 12		obs:	exp:
tta	L	0	0.12
ttg	L	0	0.21
tct	S	0	2.10
tcc	S	9	1.94
tca	S	4	3.49
tcg	S	0	0.51
ctt	L	0	0.21
ctc	L	0	0.15
cta	L	1	0.15
ctg	L	0	0.17
cct	P	6	19390.00
ccc	P	8	10909.00
cca	P	58168	19659.00
ccg	P	5	8230.00
caa	Q	1	0.61
cag	Q	0	0.39
att	I	0	0.35
atc	I	1	0.26
ata	I	0	0.39
act	T	0	0.62
acc	T	0	0.35
aca	T	2	0.87
acg	T	0	0.16
agt	S	0	2.27
agc	S	0	2.68
gct	A	0	11.17
gcc	A	0	10.24
gca	A	41	16.58
gcg	A	0	3.00
gat	D	1	0.57
gac	D	0	0.43
---	-	1	1.00
---	--	------	------
mPD		0.0032	0.72
		nPD:	0.
		N. weight:	1.3
		Sc. PD:	-0.066
		Sc. rank:	-45703.0

NA			
Pos. 13		obs:	exp:
tct	S	0	0.81
tcc	S	0	0.75
tca	S	0	1.34
tcg	S	0	0.20
tat	Y	2	1.05
tac	Y	0	0.95
cat	H	1	0.56
cac	H	0	0.44
caa	Q	1	0.61
cag	Q	0	0.39
att	I	4	1.76
atc	I	1	1.31
ata	I	0	1.93
act	T	32	9.94
acc	T	0	5.66
aca	T	0	13.85
acg	T	0	2.56
aat	N	57706	32561.00
aac	N	494	25639.00
aaa	K	1	0.66
aag	K	0	0.34
agt	S	5	0.87
agc	S	0	1.03
---	-	1	1.00
---	--	------	------
mPD		0.018	0.49
		nPD:	0.04
		N. weight:	0.62
		Sc. PD:	-0.015
		Sc. rank:	6595.7

NA			
Pos. 14		obs:	exp:
tta	L	0	0.12
ttg	L	0	0.21
tct	S	0	0.48
tcc	S	0	0.45
tca	S	3	0.81
tcg	S	0	0.12
tat	Y	0	2.11
tac	Y	4	1.89
ctt	L	0	0.21
ctc	L	0	0.15
cta	L	1	0.15
ctg	L	0	0.17
cct	P	0	0.67
ccc	P	0	0.37
cca	P	2	0.68
ccg	P	0	0.28
caa	Q	57957	35522.00
cag	Q	154	22589.00
cgt	R	0	0.56
cgc	R	0	1.01
cga	R	28	2.55
cgg	R	0	2.40
aaa	K	98	64.43
aag	K	0	33.57
agt	S	0	0.52
agc	S	0	0.62
aga	R	0	13.73
agg	R	0	7.74
---	-	1	1.00
---	--	------	------
mPD		0.010	0.48
		nPD:	0.02
		N. weight:	0.55
		Sc. PD:	-0.021
		Sc. rank:	-3246.9

NA

Pos.	15	obs:	exp:
caa	Q	0	1.22
cag	Q	2	0.78
cgt	R	0	0.38
cgc	R	0	0.68
cga	R	0	1.73
cgg	R	0	1.63
atg	M	2	2.00
aat	N	1	0.56
aac	N	0	0.44
aaa	K	124	38278.00
aag	K	58099	19945.00
aga	R	0	9.32
agg	R	19	5.26
---	-	1	1.00
---	--	------	------
mPD		0.0051	0.45
		nPD:	0.01
		N. weight:	1.2
		Sc. PD:	-0.057
		Sc. rank:	-24598.0

,

NA

Pos.	16	obs:	exp:
tta	L	2	0.23
ttg	L	0	0.41
ctt	L	0	0.42
ctc	L	0	0.29
cta	L	0	0.31
ctg	L	0	0.34
att	I	3	20443.00
atc	I	0	15302.00
ata	I	58231	22490.00
act	T	0	1.55
acc	T	0	0.88
aca	T	5	2.16
acg	T	0	0.40
aaa	K	3	1.97
aag	K	0	1.03
gtt	V	0	0.50
gtc	V	0	0.41
gta	V	2	0.43
gtg	V	0	0.66
---	-	2	2.00
---	--	------	------
mPD		0.00051	0.66
		nPD:	0.
		N. weight:	1.1
		Sc. PD:	-0.061
		Sc. rank:	-47263.0

,

NA

Pos.	17	obs:	exp:
cgt	R	0	0.02
cgc	R	0	0.04
cga	R	0	0.09
cgg	R	0	0.09
att	I	4	20441.00
atc	I	1	15300.00
ata	I	58224	22488.00
atg	M	8	8.00
act	T	0	1.86
acc	T	0	1.06
aca	T	6	2.60
acg	T	0	0.48
aaa	K	1	0.66
aag	K	0	0.34
aga	R	1	0.49
agg	R	0	0.28
gtt	V	0	0.50
gtc	V	0	0.41
gta	V	2	0.43
gtg	V	0	0.66
---	-	1	1.00
---	--	------	------
mPD		0.00079	0.66
		nPD:	0.
		N. weight:	1.1
		Sc. PD:	-0.061
		Sc. rank:	-46913.0

,

NA

Pos.	18	obs:	exp:
tct	S	0	9.52
tcc	S	0	8.82
tca	S	1	15.86
tcg	S	58	2.32
cct	P	0	0.33
ccc	P	0	0.19
cca	P	0	0.34
ccg	P	1	0.14
atg	M	6	6.00
act	T	85	18066.00
acc	T	1	10285.00
aca	T	1729	25177.00
acg	T	56360	4646.00
agt	S	0	10.31
agc	S	0	12.17
gct	A	0	1.64
gcc	A	0	1.50
gca	A	0	2.43
gcg	A	6	0.44
---	-	1	1.00
---	--	------	------
mPD		0.063	0.68
		nPD:	0.09
		N. weight:	2.7
		Sc. PD:	0.063
		Sc. rank:	74431.0

,

NA

Pos.	19	obs:	exp:
tta	L	0	0.46
ttg	L	0	0.83
ctt	L	3	0.84
ctc	L	1	0.59
cta	L	0	0.61
ctg	L	0	0.67
att	I	58156	20439.00
atc	I	54	15299.00
ata	I	14	22486.00
atg	M	3	3.00
gtt	V	16	4.01
gtc	V	0	3.26
gta	V	0	3.47
gtg	V	0	5.26
---	-	1	1.00
---	--	------	------
mPD		0.0032	0.66
		nPD:	0.
		N. weight:	1.2
		Sc. PD:	-0.063
		Sc. rank:	-42558.0

,

NA

Pos.	20	obs:	exp:
tct	S	0	1.29
tcc	S	0	1.20
tca	S	0	2.15
tcg	S	0	0.31
tgt	C	0	0.39
tgc	C	1	0.61
agt	S	0	1.40
agc	S	8	1.65
gtt	V	1	0.25
gtc	V	0	0.20
gta	V	0	0.22
gtg	V	0	0.33
gct	A	0	0.27
gcc	A	1	0.25
gca	A	0	0.40
gcg	A	0	0.07
ggt	G	220	7840.00
ggc	G	57942	8128.00
gga	G	66	26883.00
ggg	G	9	15386.00
---	--	------	------
mPD		0.010	0.68
		nPD:	0.02
		N. weight:	2.2
		Sc. PD:	-0.097
		Sc. rank:	-28076.0

,

NA

Pos.	21	obs:	exp:
ttt	F	42	20.43
ttc	F	0	21.57
tta	L	0	0.12
ttg	L	1	0.21
tct	S	58068	9391.00
tcc	S	73	8697.00
tca	S	52	15642.00
tcg	S	4	2291.00
tat	Y	2	1.05
tac	Y	0	0.95
tgt	C	1	0.39
tgc	C	0	0.61
ctt	L	0	0.21
ctc	L	0	0.15
cta	L	0	0.15
ctg	L	0	0.17
act	T	3	0.93
acc	T	0	0.53
aca	T	0	1.30
acg	T	0	0.24
agt	S	0	10170.00
agc	S	0	12006.00
gct	A	2	0.55
gcc	A	0	0.50
gca	A	0	0.81
gcg	A	0	0.15
---	--	------	------
mPD		0.0062	1.6
		nPD:	0.
		N. weight:	2.1
		Sc. PD:	-0.11
		Sc. rank:	-78799.0

,

NA

Pos.	22	obs:	exp:
ttt	F	6	2.92
ttc	F	0	3.08
att	I	44	15.80
atc	I	1	11.82
ata	I	0	17.38
gtt	V	57678	14566.00
gtc	V	446	11849.00
gta	V	5	12604.00
gtg	V	24	19134.00
gct	A	35	10.08
gcc	A	2	9.24
gca	A	0	14.97
gcg	A	0	2.70
gat	D	3	1.70
gac	D	0	1.30
ggt	G	4	0.54
ggc	G	0	0.56
gga	G	0	1.85
ggg	G	0	1.06
---	--	------	------
mPD		0.020	0.74
		nPD:	0.03
		N. weight:	1.6
		Sc. PD:	-0.052
		Sc. rank:	1894.3

,

NA

Pos.	23	obs:	exp:
ttt	F	5	2.43
ttc	F	0	2.57
tct	S	57868	9397.00
tcc	S	355	8702.00
tca	S	8	15652.00
tcg	S	1	2292.00
tgt	C	2	0.78
tgc	C	0	1.22
tgg	W	1	1.00
cct	P	3	1.00
ccc	P	0	0.56
cca	P	0	1.01
ccg	P	0	0.42
act	T	3	0.93
acc	T	0	0.53
aca	T	0	1.30
acg	T	0	0.24
agt	S	0	10176.00
agc	S	0	12013.00
gct	A	2	0.55
gcc	A	0	0.50
gca	A	0	0.81
gcg	A	0	0.15
---	--	------	------
mPD		0.013	1.6
		nPD:	0.01
		N. weight:	2.1
		Sc. PD:	-0.1
		Sc. rank:	-60006.0

,

NA

Pos.	24	obs:	exp:
ttt	F	0	8.27
ttc	F	17	8.73
tta	L	0	6756.00
ttg	L	0	12037.00
ctt	L	83	12179.00
ctc	L	58071	8538.00
cta	L	65	8940.00
ctg	L	3	9771.00
cat	H	0	0.56
cac	H	1	0.44
cgt	R	0	0.02
cgc	R	1	0.04
cga	R	0	0.09
cgg	R	0	0.09
att	I	0	2.11
atc	I	6	1.58
ata	I	0	2.32
atg	M	1	1.00
aga	R	0	0.49
agg	R	0	0.28
---	--	------	------
mPD		0.0061	1.2
		nPD:	0.01
		N. weight:	2.2
		Sc. PD:	-0.11
		Sc. rank:	-73904.0

,

NA			
Pos.	25	obs:	exp:
tct	S	0	1.94
tcc	S	5	1.79
tca	S	2	3.23
tcg	S	0	0.47
cct	P	0	0.33
ccc	P	1	0.19
cca	P	0	0.34
ccg	P	0	0.14
att	I	2	78.98
atc	I	214	59.12
ata	I	9	86.89
atg	M	1	1.00
act	T	268	18012.00
acc	T	36420	10254.00
aca	T	21280	25101.00
acg	T	31	4632.00
agt	S	0	2.10
agc	S	5	2.48
gct	A	0	2.73
gcc	A	5	2.50
gca	A	5	4.05
gcg	A	0	0.73
---	--	------	------
mPD		0.48	0.69
		nPD:	0.7
		N. weight:	0.83
		Sc. PD:	0.45
		Sc. rank:	35909.0

,

NA			
Pos.	26	obs:	exp:
ttt	F	21	10.21
ttc	F	0	10.79
tta	L	0	3.25
ttg	L	0	5.79
tct	S	0	0.32
tcc	S	0	0.30
tca	S	0	0.54
tcg	S	0	0.08
ctt	L	28	5.86
ctc	L	0	4.11
cta	L	0	4.30
ctg	L	0	4.70
att	I	57296	20413.00
atc	I	852	15280.00
ata	I	3	22458.00
atg	M	6	6.00
act	T	8	3.11
acc	T	2	1.77
aca	T	0	4.33
acg	T	0	0.80
aat	N	2	1.12
aac	N	0	0.88
agt	S	2	0.35
agc	S	0	0.41
gtt	V	28	7.01
gtc	V	0	5.71
gta	V	0	6.07
gtg	V	0	9.21
---	--	------	------
mPD		0.032	0.66
		nPD:	0.05
		N. weight:	1.1
		Sc. PD:	-0.016
		Sc. rank:	19042.0

,

NA			
Pos.	27	obs:	exp:
ttt	F	0	0.97
ttc	F	2	1.03
tta	L	0	0.12
ttg	L	0	0.21
tct	S	182	9284.00
tcc	S	57293	8597.00
tca	S	55	15463.00
tcg	S	2	2265.00
tat	Y	0	1.05
tac	Y	2	0.95
tgt	C	0	0.78
tgc	C	2	1.22
tgg	W	2	2.00
ctt	L	0	0.21
ctc	L	1	0.15
cta	L	0	0.15
ctg	L	0	0.17
cct	P	0	0.33
ccc	P	1	0.19
cca	P	0	0.34
ccg	P	0	0.14
act	T	0	3.11
acc	T	10	1.77
aca	T	0	4.33
acg	T	0	0.80
agt	S	0	10054.00
agc	S	0	11869.00
gct	A	0	189.40
gcc	A	597	173.60
gca	A	97	281.10
gcg	A	1	50.81
ggt	G	0	0.13
ggc	G	0	0.14
gga	G	1	0.46
ggg	G	0	0.26
---	--	------	------
mPD		0.036	1.6
		nPD:	0.02
		N. weight:	2.2
		Sc. PD:	-0.081
		Sc. rank:	-10762.0

,

NA			
Pos.	28	obs:	exp:
tct	S	0	2.58
tcc	S	0	2.39
tca	S	16	4.30
tcg	S	0	0.63
cgt	R	0	0.02
cgc	R	0	0.04
cga	R	0	0.09
cgg	R	0	0.09
att	I	0	15.80
atc	I	1	11.82
ata	I	44	17.38
atg	M	2	2.00
act	T	18	18048.00
acc	T	11	10275.00
aca	T	58066	25151.00
acg	T	21	4642.00
agt	S	0	2.80
agc	S	0	3.30
aga	R	0	0.49
agg	R	1	0.28
gct	A	0	18.53
gcc	A	0	16.99
gca	A	67	27.51
gcg	A	1	4.97
---	--	------	------
mPD		0.0064	0.68
		nPD:	0.01
		N. weight:	0.97
		Sc. PD:	-0.047
		Sc. rank:	-23922.0

,

NA			
Pos.	29	obs:	exp:
tta	L	1	0.81
ttg	L	0	1.45
ctt	L	0	1.46
ctc	L	0	1.03
cta	L	6	1.07
ctg	L	0	1.17
att	I	2	20376.00
atc	I	3	15252.00
ata	I	58040	22417.00
atg	M	30	30.00
act	T	0	19.25
acc	T	0	10.96
aca	T	62	26.83
acg	T	0	4.95
aat	N	1	0.56
aac	N	0	0.44
gtt	V	0	25.30
gtc	V	0	20.58
gta	V	100	21.89
gtg	V	1	33.23
gct	A	1	0.55
gcc	A	0	0.50
gca	A	1	0.81
gcg	A	0	0.15
---	--	------	------
mPD		0.0073	0.67
		nPD:	0.01
		N. weight:	1.1
		Sc. PD:	-0.052
		Sc. rank:	-23773.0

,

NA			
Pos.	30	obs:	exp:
tct	S	0	1.78
tcc	S	0	1.64
tca	S	0	2.96
tcg	S	0	0.43
tat	Y	0	1.05
tac	Y	2	0.95
tgt	C	91	22754.00
tgc	C	58134	35471.00
aat	N	0	0.56
aac	N	1	0.44
agt	S	0	1.92
agc	S	11	2.27
gct	A	1	0.27
gcc	A	0	0.25
gca	A	0	0.40
gcg	A	0	0.07
ggt	G	0	1.08
ggc	G	8	1.12
gga	G	0	3.69
ggg	G	0	2.11
---	--	------	------
mPD		0.0040	0.48
		nPD:	0.01
		N. weight:	0.56
		Sc. PD:	-0.028
		Sc. rank:	-15878.0

,

NA			
Pos.	31	obs:	exp:
ttt	F	1210	28299.00
ttc	F	56967	29878.00
tta	L	27	7.43
ttg	L	1	13.23
tct	S	1	0.16
tcc	S	0	0.15
tca	S	0	0.27
tcg	S	0	0.04
tgt	C	0	0.39
tgc	C	1	0.61
ctt	L	0	13.39
ctc	L	36	9.39
cta	L	0	9.83
ctg	L	0	10.74
att	I	0	0.70
atc	I	2	0.53
ata	I	0	0.77
agt	S	0	0.17
agc	S	0	0.21
gtt	V	0	0.75
gtc	V	3	0.61
gta	V	0	0.65
gtg	V	0	0.99
---	--	------	------
mPD		0.043	0.50
		nPD:	0.09
		N. weight:	0.66
		Sc. PD:	0.012
		Sc. rank:	17196.0

,

NA			
Pos.	32	obs:	exp:
ttt	F	111	27879.00
ttc	F	57203	29435.00
tta	L	55	103.70
ttg	L	0	184.80
tct	S	0	5.81
tcc	S	35	5.38
tca	S	1	9.68
tcg	S	0	1.42
ctt	L	619	187.00
ctc	L	220	131.10
cta	L	0	137.30
ctg	L	0	150.00
att	I	0	0.35
atc	I	0	0.26
ata	I	1	0.39
agt	S	0	6.29
agc	S	0	7.43
gtt	V	0	0.75
gtc	V	3	0.61
gta	V	0	0.65
gtg	V	0	0.99
---	--	------	------
mPD		0.056	0.53
		nPD:	0.11
		N. weight:	0.76
		Sc. PD:	0.027
		Sc. rank:	22555.0

,

NA			
Pos.	33	obs:	exp:
tta	L	1	2.09
ttg	L	13	3.72
ctt	L	0	3.77
ctc	L	0	2.64
cta	L	0	2.76
ctg	L	4	3.02
att	I	31	50.55
atc	I	0	37.84
ata	I	113	55.61
atg	M	58018	58018.00
act	T	0	8.70
acc	T	0	4.95
aca	T	0	12.12
acg	T	28	2.24
gtt	V	0	10.02
gtc	V	0	8.15
gta	V	0	8.67
gtg	V	40	13.16
---	--	------	------
mPD		0.0079	0.010
		nPD:	0.79
		N. weight:	0.004
		Sc. PD:	0.0025
		Sc. rank:	174.0

,

NA			
Pos.	34	obs:	exp:
cat	H	1	1.13
cac	H	1	0.87
caa	Q	57980	35603.00
cag	Q	263	22640.00
cgt	R	0	0.02
cgc	R	0	0.04
cga	R	1	0.09
cgg	R	0	0.09
aaa	K	1	0.66
aag	K	0	0.34
aga	R	0	0.49
agg	R	0	0.28
gaa	E	1	0.59
gag	E	0	0.41
---	--	------	------
mPD		0.0092	0.48
		nPD:	0.02
		N. weight:	0.54
		Sc. PD:	-0.021
		Sc. rank:	-4145.6

,

NA			
Pos.	35	obs:	exp:
ttt	F	1	0.49
ttc	F	0	0.51
tta	L	0	2.09
ttg	L	0	3.72
tct	S	0	0.65
tcc	S	0	0.60
tca	S	0	1.08
tcg	S	0	0.16
ctt	L	18	3.77
ctc	L	0	2.64
cta	L	0	2.76
ctg	L	0	3.02
att	I	56870	19996.00
atc	I	73	14967.00
ata	I	18	21998.00
act	T	234	110.60
acc	T	118	62.94
aca	T	4	154.10
acg	T	0	28.43
aat	N	1	0.56
aac	N	0	0.44
agt	S	4	0.70
agc	S	0	0.83
gtt	V	901	225.90
gtc	V	0	183.80
gta	V	1	195.50
gtg	V	0	296.80
gct	A	5	1.36
gcc	A	0	1.25
gca	A	0	2.02
gcg	A	0	0.37
---	--	------	------
mPD		0.051	0.71
		nPD:	0.07
		N. weight:	1.2
		Sc. PD:	0.0073
		Sc. rank:	28037.0

,

NA			
Pos.	36	obs:	exp:
tct	S	0	0.97
tcc	S	6	0.90
tca	S	0	1.61
tcg	S	0	0.24
act	T	0	10.56
acc	T	34	6.01
aca	T	0	14.71
acg	T	0	2.72
agt	S	0	1.05
agc	S	0	1.24
gtt	V	0	4.26
gtc	V	17	3.46
gta	V	0	3.68
gtg	V	0	5.59
gct	A	99	15859.00
gcc	A	58074	14537.00
gca	A	14	23537.00
gcg	A	0	4254.00
ggt	G	0	0.54
ggc	G	3	0.56
gga	G	1	1.85
ggg	G	0	1.06
---	--	------	------
mPD		0.0060	0.70
		nPD:	0.01
		N. weight:	1.6
		Sc. PD:	-0.078
		Sc. rank:	-43419.0

,

NA			
Pos.	37	obs:	exp:
ttt	F	0	0.49
ttc	F	1	0.51
tta	L	0	0.70
ttg	L	0	1.24
tct	S	0	0.81
tcc	S	0	0.75
tca	S	0	1.34
tcg	S	0	0.20
ctt	L	1	1.26
ctc	L	5	0.88
cta	L	0	0.92
ctg	L	0	1.01
att	I	1645	20379.00
atc	I	56300	15254.00
ata	I	107	22420.00
atg	M	3	3.00
act	T	1	20.81
acc	T	66	11.85
aca	T	0	29.00
acg	T	0	5.35
aat	N	0	0.56
aac	N	1	0.44
agt	S	1	0.87
agc	S	4	1.03
gtt	V	1	28.05
gtc	V	111	22.82
gta	V	0	24.28
gtg	V	0	36.85
gct	A	0	0.27
gcc	A	0	0.25
gca	A	1	0.40
gcg	A	0	0.07
---	--	------	------
mPD		0.065	0.67
		nPD:	0.1
		N. weight:	1.4
		Sc. PD:	0.039
		Sc. rank:	38766.0

,

NA			
Pos.	38	obs:	exp:
ttt	F	3	2.43
ttc	F	2	2.57
tta	L	811	6758.00
ttg	L	17529	12040.00
ctt	L	7	12183.00
ctc	L	0	8540.00
cta	L	202	8943.00
ctg	L	39688	9774.00
att	I	0	0.35
atc	I	0	0.26
ata	I	1	0.39
atg	M	5	5.00
---	--	------	------
mPD		0.47	1.2
		nPD:	0.4
		N. weight:	1.2
		Sc. PD:	0.35
		Sc. rank:	49558.0

,

NA			
Pos.	39	obs:	exp:
tta	L	69	10.68
ttg	L	0	19.02
ctt	L	0	19.25
ctc	L	0	13.49
cta	L	23	14.13
ctg	L	0	15.44
att	I	3	20012.00
atc	I	10	14979.00
ata	I	56994	22016.00
atg	M	54	54.00
act	T	0	22.67
acc	T	0	12.91
aca	T	73	31.59
acg	T	0	5.83
gtt	V	0	255.50
gtc	V	0	207.80
gta	V	856	221.10
gtg	V	164	335.60
gct	A	0	0.55
gcc	A	0	0.50
gca	A	2	0.81
gcg	A	0	0.15
---	--	------	------
mPD		0.048	0.70
		nPD:	0.07
		N. weight:	1.1
		Sc. PD:	0.0028
		Sc. rank:	23868.0

,

NA			
Pos.	40	obs:	exp:
tct	S	0	0.65
tcc	S	0	0.60
tca	S	1	1.08
tcg	S	0	0.16
att	I	58	20.36
atc	I	0	15.24
ata	I	0	22.40
atg	M	1	1.00
act	T	57601	18055.00
acc	T	516	10279.00
aca	T	10	25162.00
acg	T	13	4644.00
aat	N	16	8.95
aac	N	0	7.05
agt	S	3	0.70
agc	S	0	0.83
gct	A	29	7.90
gcc	A	0	7.25
gca	A	0	11.73
gcg	A	0	2.12
---	--	------	------
mPD		0.022	0.68
		nPD:	0.03
		N. weight:	1.3
		Sc. PD:	-0.037
		Sc. rank:	7706.1

,

NA			
Pos.	41	obs:	exp:
att	I	12	4.56
atc	I	0	3.42
ata	I	1	5.02
act	T	58074	18081.00
acc	T	91	10294.00
aca	T	40	25197.00
acg	T	17	4650.00
aat	N	6	3.36
aac	N	0	2.64
gct	A	7	1.91
gcc	A	0	1.75
gca	A	0	2.83
gcg	A	0	0.51
---	--	------	------
mPD		0.0060	0.68
		nPD:	0.01
		N. weight:	1.3
		Sc. PD:	-0.065
		Sc. rank:	-35621.0

,

NA			
Pos.	42	obs:	exp:
tta	L	4	0.46
ttg	L	0	0.83
tgg	W	1	1.00
ctt	L	0	0.84
ctc	L	0	0.59
cta	L	0	0.61
ctg	L	0	0.67
att	I	0	59.68
atc	I	0	44.67
ata	I	170	65.65
act	T	0	0.31
acc	T	0	0.18
aca	T	1	0.43
acg	T	0	0.08
gtt	V	17	14539.00
gtc	V	17	11828.00
gta	V	57906	12581.00
gtg	V	107	19099.00
gct	A	0	5.45
gcc	A	0	5.00
gca	A	20	8.09
gcg	A	0	1.46
gaa	E	2	1.18
gag	E	0	0.82
ggt	G	0	0.40
ggc	G	0	0.42
gga	G	3	1.38
ggg	G	0	0.79
---	--	------	------
mPD		0.012	0.75
		nPD:	0.02
		N. weight:	1.8
		Sc. PD:	-0.075
		Sc. rank:	-20601.0

,

NA			
Pos.	43	obs:	exp:
tct	S	0	0.16
tcc	S	0	0.15
tca	S	1	0.27
tcg	S	0	0.04
cct	P	0	0.33
ccc	P	0	0.19
cca	P	1	0.34
ccg	P	0	0.14
att	I	1	0.70
atc	I	0	0.53
ata	I	1	0.77
act	T	17	18078.00
acc	T	5	10292.00
aca	T	58172	25193.00
acg	T	18	4649.00
aaa	K	7	4.60
aag	K	0	2.40
agt	S	0	0.17
agc	S	0	0.21
gct	A	0	6.81
gcc	A	0	6.25
gca	A	25	10.11
gcg	A	0	1.83
---	--	------	------
mPD		0.0026	0.68
		nPD:	0.
		N. weight:	0.97
		Sc. PD:	-0.051
		Sc. rank:	-36160.0

,

NA			
Pos.	44	obs:	exp:
tta	L	348	6758.00
ttg	L	57570	12040.00
tct	S	0	0.32
tcc	S	0	0.30
tca	S	0	0.54
tcg	S	1	0.08
ctt	L	0	12182.00
ctc	L	0	8540.00
cta	L	0	8942.00
ctg	L	318	9774.00
atg	M	8	8.00
agt	S	0	0.35
agc	S	1	0.41
gtt	V	0	0.50
gtc	V	0	0.41
gta	V	0	0.43
gtg	V	2	0.66
---	--	------	------
mPD		0.023	1.2
		nPD:	0.02
		N. weight:	1.8
		Sc. PD:	-0.068
		Sc. rank:	-12082.0

,

NA			
Pos.	45	obs:	exp:
tat	Y	9	4.74
tac	Y	0	4.26
cct	P	1	0.33
ccc	P	0	0.19
cca	P	0	0.34
ccg	P	0	0.14
cat	H	58103	32841.00
cac	H	121	25383.00
caa	Q	3	3.06
cag	Q	2	1.94
cgt	R	3	0.06
cgc	R	0	0.11
cga	R	0	0.27
cgg	R	0	0.26
aat	N	6	3.36
aac	N	0	2.64
aga	R	0	1.47
agg	R	0	0.83
---	--	------	------
mPD		0.0050	0.49
		nPD:	0.01
		N. weight:	0.65
		Sc. PD:	-0.031
		Sc. rank:	-14689.0

,

NA			
Pos.	46	obs:	exp:
ttt	F	134	28317.00
ttc	F	58081	29898.00
tta	L	6	1.97
ttg	L	0	3.51
tct	S	0	2.26
tcc	S	14	2.09
tca	S	0	3.76
tcg	S	0	0.55
ctt	L	0	3.56
ctc	L	11	2.49
cta	L	0	2.61
ctg	L	0	2.85
att	I	0	0.35
atc	I	1	0.26
ata	I	0	0.39
agt	S	0	2.45
agc	S	0	2.89
---	-	1	1.00
---	--	------	------
mPD		0.0057	0.50
		nPD:	0.01
		N. weight:	0.76
		Sc. PD:	-0.035
		Sc. rank:	-15086.0

,

NA			
Pos.	47	obs:	exp:
tta	L	0	0.12
ttg	L	0	0.21
ctt	L	0	0.21
ctc	L	0	0.15
cta	L	0	0.15
ctg	L	1	0.17
caa	Q	0	1.22
cag	Q	2	0.78
cgt	R	0	0.91
cgc	R	0	1.66
cga	R	0	4.20
cgg	R	0	3.95
act	T	0	0.62
acc	T	0	0.35
aca	T	0	0.87
acg	T	2	0.16
aat	N	5	2.80
aac	N	0	2.20
aaa	K	140	38256.00
aag	K	58050	19934.00
aga	R	0	22.56
agg	R	46	12.72
gaa	E	0	0.59
gag	E	1	0.41
---	-	1	1.00
---	--	------	------
mPD		0.0068	0.45
		nPD:	0.01
		N. weight:	1.2
		Sc. PD:	-0.053
		Sc. rank:	-16863.0

,

NA			
Pos.	48	obs:	exp:
tta	L	0	0.12
ttg	L	0	0.21
ctt	L	0	0.21
ctc	L	0	0.15
cta	L	1	0.15
ctg	L	0	0.17
cct	P	0	0.33
ccc	P	0	0.19
cca	P	1	0.34
ccg	P	0	0.14
cat	H	20	15.79
cac	H	8	12.21
caa	Q	57876	35564.00
cag	Q	304	22616.00
cgt	R	0	0.42
cgc	R	0	0.76
cga	R	21	1.92
cgg	R	0	1.80
aaa	K	16	10.52
aag	K	0	5.48
aga	R	0	10.30
agg	R	0	5.81
---	-	1	1.00
---	--	------	------
mPD		0.013	0.48
		nPD:	0.03
		N. weight:	0.54
		Sc. PD:	-0.018
		Sc. rank:	701.9

,

NA			
Pos.	49	obs:	exp:
ttt	F	10	4.86
ttc	F	0	5.14
tct	S	1	0.32
tcc	S	0	0.30
tca	S	0	0.54
tcg	S	0	0.08
tat	Y	57551	30362.00
tac	Y	49	27238.00
tgt	C	250	97.70
tgc	C	0	152.30
cat	H	375	212.60
cac	H	2	164.40
cgt	R	1	0.02
cgc	R	0	0.04
cga	R	0	0.09
cgg	R	0	0.09
aat	N	4	2.24
aac	N	0	1.76
agt	S	1	0.35
agc	S	0	0.41
aga	R	0	0.49
agg	R	0	0.28
gat	D	3	1.70
gac	D	0	1.30
---	-	1	1.00
---	--	------	------
mPD		0.024	0.52
		nPD:	0.05
		N. weight:	0.74
		Sc. PD:	-0.012
		Sc. rank:	10706.0

,

NA			
Pos.	50	obs:	exp:
aat	N	50	29.09
aac	N	2	22.91
aaa	K	243	159.80
aag	K	0	83.24
gtt	V	0	0.25
gtc	V	0	0.20
gta	V	1	0.22
gtg	V	0	0.33
gct	A	0	0.55
gcc	A	0	0.50
gca	A	2	0.81
gcg	A	0	0.15
gat	D	125	102.30
gac	D	56	78.69
gaa	E	57378	34023.00
gag	E	331	23686.00
ggt	G	0	7.94
ggc	G	0	8.23
gga	G	59	27.24
ggg	G	0	15.59
---	-	1	1.00
---	--	------	------
mPD		0.031	0.50
		nPD:	0.06
		N. weight:	0.57
		Sc. PD:	-0.0013
		Sc. rank:	11853.0

,

NA			
Pos.	51	obs:	exp:
ttt	F	79	27920.00
ttc	F	57319	29478.00
tta	L	44	10.91
ttg	L	0	19.43
tct	S	0	6.78
tcc	S	41	6.28
tca	S	1	11.29
tcg	S	0	1.65
tat	Y	0	9.49
tac	Y	18	8.51
tgt	C	0	266.10
tgc	C	681	414.90
ctt	L	0	19.66
ctc	L	50	13.78
cta	L	0	14.43
ctg	L	0	15.78
cct	P	0	0.67
ccc	P	2	0.37
cca	P	0	0.68
ccg	P	0	0.28
att	I	0	1.76
atc	I	5	1.31
ata	I	0	1.93
agt	S	0	7.34
agc	S	0	8.66
gtt	V	0	1.75
gtc	V	7	1.43
gta	V	0	1.52
gtg	V	0	2.30
---	-	1	1.00
---	--	------	------
mPD		0.032	0.53
		nPD:	0.06
		N. weight:	0.76
		Sc. PD:	-0.0037
		Sc. rank:	15306.0

,

NA			
Pos.	52	obs:	exp:
tct	S	0	30.50
tcc	S	0	28.24
tca	S	0	50.80
tcg	S	0	7.44
tat	Y	0	82.23
tac	Y	156	73.77
cat	H	0	33.28
cac	H	59	25.72
att	I	0	0.35
atc	I	1	0.26
ata	I	0	0.39
act	T	0	0.93
acc	T	3	0.53
aca	T	0	1.30
acg	T	0	0.24
aat	N	293	32112.00
aac	N	57104	25285.00
aaa	K	97	63.77
aag	K	0	33.23
agt	S	1	33.03
agc	S	188	38.99
gat	D	3	195.00
gac	D	342	150.00
---	-	1	1.00
---	--	------	------
mPD		0.039	0.53
		nPD:	0.07
		N. weight:	0.92
		Sc. PD:	0.0072
		Sc. rank:	22340.0

,

NA			
Pos.	53	obs:	exp:
cct	P	0	1.33
ccc	P	4	0.75
cca	P	0	1.35
ccg	P	0	0.57
act	T	0	0.93
acc	T	3	0.53
aca	T	0	1.30
acg	T	0	0.24
---	-	58241	58241.00
---	--	------	------
mPD		7.1×10^{-9}	1.7×10^{-8}
		nPD:	0.41
		N. weight:	0.
		Sc. PD:	0
		Sc. rank:	0

,

NA			
Pos.	54	obs:	exp:
ttt	F	0	62.26
ttc	F	128	65.74
tta	L	0	0.58
ttg	L	1	1.03
tct	S	189	8975.00
tcc	S	55415	8312.00
tca	S	14	14949.00
tcg	S	1	2189.00
tat	Y	0	17.92
tac	Y	34	16.08
ctt	L	0	1.05
ctc	L	4	0.73
cta	L	0	0.77
ctg	L	0	0.84
cct	P	1	810.40
ccc	P	2431	455.90
cca	P	0	821.70
ccg	P	0	344.00
att	I	0	0.35
atc	I	1	0.26
ata	I	0	0.39
act	T	1	8.70
acc	T	27	4.95
aca	T	0	12.12
acg	T	0	2.24
agt	S	0	9720.00
agc	S	0	11474.00
gct	A	0	0.27
gcc	A	1	0.25
gca	A	0	0.40
gcg	A	0	0.07
---	--	------	------
mPD		0.094	1.7
		nPD:	0.06
		N. weight:	2.2
		Sc. PD:	-0.017
		Sc. rank:	40769.0

,

NA			
Pos.	55	obs:	exp:
cct	P	0	1.67
ccc	P	5	0.94
cca	P	0	1.69
ccg	P	0	0.71
---	-	58243	58243.00
---	--	------	------
mPD		0	5.3×10^{-9}
		nPD:	0.
		N. weight:	0.
		Sc. PD:	0
		Sc. rank:	0

,

NA			
Pos.	56	obs:	exp:
tta	L	0	19.15
ttg	L	0	34.11
tct	S	1	60.03
tcc	S	371	55.59
tca	S	0	99.99
tcg	S	0	14.64
ctt	L	1	34.52
ctc	L	164	24.20
cta	L	0	25.34
ctg	L	0	27.69
cct	P	2157	19199.00
ccc	P	55331	10801.00
cca	P	125	19465.00
ccg	P	0	8149.00
cat	H	0	49.07
cac	H	87	37.93
act	T	0	1.86
acc	T	6	1.06
aca	T	0	2.60
acg	T	0	0.48
agt	S	0	65.01
agc	S	0	76.74
gct	A	0	0.55
gcc	A	2	0.50
gca	A	0	0.81
gcg	A	0	0.15
---	-	3	3.00
---	--	------	------
mPD		0.097	0.75
		nPD:	0.13
		N. weight:	1.7
		Sc. PD:	0.095
		Sc. rank:	53047.0

,

NA			
Pos.	57	obs:	exp:
tta	L	0	1.39
ttg	L	0	2.48
tct	S	0	17.10
tcc	S	0	15.84
tca	S	104	28.49
tcg	S	2	4.17
ctt	L	0	2.51
ctc	L	0	1.76
cta	L	10	1.84
ctg	L	2	2.01
cct	P	2	19248.00
ccc	P	5	10828.00
cca	P	56307	19514.00
ccg	P	1445	8169.00
caa	Q	8	4.89
cag	Q	0	3.11
act	T	0	3.73
acc	T	0	2.12
aca	T	12	5.19
acg	T	0	0.96
aaa	K	1	0.66
aag	K	0	0.34
agt	S	0	18.52
agc	S	0	21.87
gct	A	0	94.85
gcc	A	0	86.94
gca	A	283	140.80
gcg	A	65	25.44
---	-	2	2.00
---	--	------	------
mPD		0.068	0.74
		nPD:	0.09
		N. weight:	1.1
		Sc. PD:	0.026
		Sc. rank:	31049.0

,

NA			
Pos.	58	obs:	exp:
aaa	K	7	4.60
aag	K	0	2.40
---	-	58241	58241.00
---	--	------	------
mPD		0	6.5×10^{-9}
		nPD:	0.
		N. weight:	0.
		Sc. PD:	0
		Sc. rank:	0

,

NA			
Pos.	59	obs:	exp:
caa	Q	1	0.61
cag	Q	0	0.39
---	-	58247	58247.00
---	--	------	------
mPD		0	1.4×10^{-10}
		nPD:	0.
		N. weight:	0.
		Sc. PD:	0
		Sc. rank:	0

,

NA			
Pos.	60	obs:	exp:
cct	P	0	0.33
ccc	P	0	0.19
cca	P	1	0.34
ccg	P	0	0.14
---	-	58247	58247.00
---	--	------	------
mPD		0	2.1×10^{-10}
		nPD:	0.
		N. weight:	0.
		Sc. PD:	0
		Sc. rank:	0

,

NA			
Pos.	61	obs:	exp:
tct	S	0	0.16
tcc	S	0	0.15
tca	S	0	0.27
tcg	S	0	0.04
agt	S	1	0.17
agc	S	0	0.21
---	-	58247	58247.00
---	--	------	------
mPD		0	4.8×10^{-10}
		nPD:	0.
		N. weight:	0.
		Sc. PD:	0
		Sc. rank:	0

,

NA			
Pos.	62	obs:	exp:
att	I	1	0.35
atc	I	0	0.26
ata	I	0	0.39
gat	D	1	0.57
gac	D	0	0.43
---	-	58246	58246.00
---	--	------	------
mPD		1.2×10^{-9}	1.9×10^{-9}
		nPD:	0.61
		N. weight:	0.
		Sc. PD:	0
		Sc. rank:	0

,

NA			
Pos.	63	obs:	exp:
caa	Q	1	0.61
cag	Q	0	0.39
gct	A	1	0.27
gcc	A	0	0.25
gca	A	0	0.40
gcg	A	0	0.07
---	-	58246	58246.00
---	--	------	------
mPD		1.8×10^{-9}	2.0×10^{-9}
		nPD:	0.91
		N. weight:	0.
		Sc. PD:	0
		Sc. rank:	0

,

NA			
Pos.	64	obs:	exp:
att	I	1	0.35
atc	I	0	0.26
ata	I	0	0.39
gtt	V	0	0.25
gtc	V	0	0.20
gta	V	0	0.22
gtg	V	1	0.33
---	-	58246	58246.00
---	--	------	------
mPD		1.2×10^{-9}	1.5×10^{-9}
		nPD:	0.81
		N. weight:	0.
		Sc. PD:	0
		Sc. rank:	0

,

NA			
Pos.	65	obs:	exp:
gtt	V	0	0.25
gtc	V	0	0.20
gta	V	0	0.22
gtg	V	1	0.33
ggt	G	0	0.13
ggc	G	0	0.14
gga	G	0	0.46
ggg	G	1	0.26
---	-	58246	58246.00
---	--	------	------
mPD		5.9×10^{-10}	1.5×10^{-9}
		nPD:	0.41
		N. weight:	0.
		Sc. PD:	0
		Sc. rank:	0

,

NA			
Pos.	66	obs:	exp:
tct	S	0	11.46
tcc	S	0	10.61
tca	S	0	19.08
tcg	S	0	2.79
tat	Y	3	2.64
tac	Y	2	2.36
cat	H	2	1.13
cac	H	0	0.87
act	T	3	3.73
acc	T	9	2.12
aca	T	0	5.19
acg	T	0	0.96
aat	N	28348	32511.00
aac	N	29762	25599.00
aaa	K	38	26.95
aag	K	3	14.05
agt	S	16	12.41
agc	S	55	14.65
gat	D	2	3.96
gac	D	5	3.04
---	--	------	------
mPD		0.50	0.50
		nPD:	1.01
		N. weight:	0.014
		Sc. PD:	0.012
		Sc. rank:	615.6

,

NA			
Pos.	67	obs:	exp:
caa	Q	1	0.61
cag	Q	0	0.39
aat	N	0	1.12
aac	N	2	0.88
---	-	58245	58245.00
---	--	------	------
mPD		2.4×10^{-9}	3.1×10^{-9}
		nPD:	0.77
		N. weight:	0.
		Sc. PD:	0
		Sc. rank:	0

,

NA

Pos.	68	obs:	exp:
tct	S	0	0.81
tcc	S	0	0.75
tca	S	0	1.34
tcg	S	0	0.20
caa	Q	1	0.61
cag	Q	0	0.39
aat	N	204	32577.00
aac	N	58024	25651.00
aaa	K	5	3.29
aag	K	0	1.71
agt	S	0	0.87
agc	S	5	1.03
gat	D	0	5.09
gac	D	9	3.91
---	--	------	------
mPD		0.0077	0.49
		nPD:	0.02
		N. weight:	0.92
		Sc. PD:	-0.04
		Sc. rank:	-11316.0

,

NA

Pos.	69	obs:	exp:
cct	P	0	0.33
ccc	P	0	0.19
cca	P	1	0.34
ccg	P	0	0.14
cat	H	52	31.59
cac	H	4	24.41
caa	Q	58086	35556.00
cag	Q	81	22611.00
cgt	R	0	0.38
cgc	R	0	0.68
cga	R	19	1.73
cgg	R	0	1.63
aaa	K	5	3.29
aag	K	0	1.71
aga	R	0	9.32
agg	R	0	5.26
---	--	------	------
mPD		0.0056	0.48
		nPD:	0.01
		N. weight:	0.56
		Sc. PD:	-0.026
		Sc. rank:	-11142.0

,

NA

Pos.	70	obs:	exp:
tta	L	1	1.39
ttg	L	9	2.48
ctt	L	0	2.51
ctc	L	0	1.76
cta	L	0	1.84
ctg	L	2	2.01
att	I	1	0.70
atc	I	0	0.53
ata	I	1	0.77
atg	M	76	76.00
gtt	V	10	14552.00
gtc	V	0	11838.00
gta	V	820	12592.00
gtg	V	57267	19115.00
gct	A	0	14.72
gcc	A	0	13.49
gca	A	16	21.84
gcg	A	38	3.95
gaa	E	0	1.18
gag	E	2	0.82
ggt	G	0	0.54
ggc	G	0	0.56
gga	G	0	1.85
ggg	G	4	1.06
---	-	1	1.00
---	--	------	------
mPD		0.034	0.75
		nPD:	0.05
		N. weight:	1.2
		Sc. PD:	-0.021
		Sc. rank:	17423.0

,

NA

Pos.	71	obs:	exp:
tta	L	0	2.09
ttg	L	16	3.72
ctt	L	0	3.77
ctc	L	0	2.64
cta	L	0	2.76
ctg	L	2	3.02
att	I	21	68.10
atc	I	3	50.98
ata	I	170	74.92
atg	M	56885	56885.00
act	T	0	11.49
acc	T	0	6.54
aca	T	2	16.01
acg	T	35	2.96
aaa	K	0	3.94
aag	K	6	2.06
gtt	V	0	277.00
gtc	V	0	225.40
gta	V	42	239.70
gtg	V	1064	363.90
gaa	E	1	0.59
gag	E	0	0.41
---	-	1	1.00
---	--	------	------
mPD		0.048	0.073
		nPD:	0.65
		N. weight:	0.026
		Sc. PD:	0.013
		Sc. rank:	1109.9

,

NA

Pos.	72	obs:	exp:
ttt	F	93	45.24
ttc	F	0	47.76
tta	L	6	6710.00
ttg	L	42	11956.00
ctt	L	97	12097.00
ctc	L	1	8480.00
cta	L	963	8880.00
ctg	L	56720	9705.00
cct	P	0	86.31
ccc	P	0	48.56
cca	P	21	87.50
ccg	P	238	36.63
caa	Q	0	23.23
cag	Q	38	14.77
cgt	R	0	0.10
cgc	R	0	0.18
cga	R	1	0.46
cgg	R	4	0.43
att	I	0	0.35
atc	I	0	0.26
ata	I	1	0.39
atg	M	15	15.00
act	T	0	0.31
acc	T	0	0.18
aca	T	1	0.43
acg	T	0	0.08
aga	R	0	2.45
agg	R	0	1.38
gtt	V	0	1.25
gtc	V	0	1.02
gta	V	0	1.08
gtg	V	5	1.65
---	-	2	2.00
---	--	------	------
mPD		0.056	1.2
		nPD:	0.05
		N. weight:	2.
		Sc. PD:	-0.03
		Sc. rank:	31915.0

,

NA

Pos.	73	obs:	exp:
ttt	F	4	1.95
ttc	F	0	2.05
tct	S	0	0.48
tcc	S	0	0.45
tca	S	0	0.81
tcg	S	0	0.12
tat	Y	11	5.80
tac	Y	0	5.20
tgt	C	58167	22754.00
tgc	C	58	35471.00
cgt	R	3	0.08
cgc	R	1	0.14
cga	R	0	0.36
cgg	R	0	0.34
agt	S	3	0.52
agc	S	0	0.62
aga	R	0	1.96
agg	R	0	1.11
---	-	1	1.00
---	--	------	------
mPD		0.0028	0.48
		nPD:	0.01
		N. weight:	1.1
		Sc. PD:	-0.055
		Sc. rank:	-35193.0

,

NA

Pos.	74	obs:	exp:
caa	Q	9	5.50
cag	Q	0	3.50
aat	N	1	0.56
aac	N	0	0.44
aaa	K	36	23.67
aag	K	0	12.33
gct	A	0	0.55
gcc	A	0	0.50
gca	A	2	0.81
gcg	A	0	0.15
gat	D	2	6.22
gac	D	9	4.78
gaa	E	58040	34287.00
gag	E	116	23869.00
ggt	G	0	4.31
ggc	G	0	4.47
gga	G	32	14.77
ggg	G	0	8.45
---	-	1	1.00
---	--	------	------
mPD		0.0071	0.49
		nPD:	0.01
		N. weight:	0.6
		Sc. PD:	-0.026
		Sc. rank:	-8484.5

,

NA

Pos.	75	obs:	exp:
tct	S	0	31.95
tcc	S	0	29.59
tca	S	198	53.22
tcg	S	0	7.79
cct	P	3	19339.00
ccc	P	1	10880.00
cca	P	57693	19607.00
ccg	P	337	8208.00
caa	Q	7	4.89
cag	Q	1	3.11
act	T	0	2.17
acc	T	0	1.24
aca	T	7	3.03
acg	T	0	0.56
agt	S	0	34.60
agc	S	0	40.85
---	-	1	1.00
---	--	------	------
mPD		0.019	0.73
		nPD:	0.03
		N. weight:	1.2
		Sc. PD:	-0.042
		Sc. rank:	843.4

,

NA

Pos.	76	obs:	exp:
tct	S	0	0.16
tcc	S	0	0.15
tca	S	0	0.27
tcg	S	0	0.04
act	T	0	0.62
acc	T	0	0.35
aca	T	2	0.87
acg	T	0	0.16
agt	S	0	0.17
agc	S	1	0.21
---	-	58245	58245.00
---	--	------	------
mPD		2.4×10^{-9}	3.3×10^{-9}
		nPD:	0.71
		N. weight:	0.
		Sc. PD:	0
		Sc. rank:	0

,

NA

Pos.	77	obs:	exp:
tct	S	0	0.32
tcc	S	0	0.30
tca	S	2	0.54
tcg	S	0	0.08
cgt	R	0	0.08
cgc	R	0	0.14
cga	R	0	0.36
cgg	R	0	0.34
att	I	4	44.93
atc	I	1	33.63
ata	I	123	49.43
act	T	2209	18002.00
acc	T	3	10249.00
aca	T	55699	25087.00
acg	T	56	4630.00
aat	N	2	1.12
aac	N	0	0.88
aaa	K	98	64.43
aag	K	0	33.57
agt	S	0	0.35
agc	S	0	0.41
aga	R	4	1.96
agg	R	0	1.11
gtt	V	0	0.25
gtc	V	1	0.20
gta	V	0	0.22
gtg	V	0	0.33
gct	A	3	12.26
gcc	A	0	11.24
gca	A	42	18.20
gcg	A	0	3.29
---	-	1	1.00
---	--	------	------
mPD		0.085	0.69
		nPD:	0.12
		N. weight:	0.79
		Sc. PD:	0.04
		Sc. rank:	23998.0

,

NA			
Pos.	78	obs:	exp:
tta	L	1	0.46
ttg	L	0	0.83
ctt	L	0	0.84
ctc	L	0	0.59
cta	L	3	0.61
ctg	L	0	0.67
cgt	R	0	0.06
cgc	R	0	0.11
cga	R	0	0.27
cgg	R	0	0.26
att	I	3	19741.00
atc	I	4	14777.00
ata	I	56229	21718.00
atg	M	1641	1641.00
act	T	0	66.46
acc	T	0	37.84
aca	T	214	92.61
acg	T	0	17.09
aaa	K	2	1.31
aag	K	0	0.69
aga	R	3	1.47
agg	R	0	0.83
gtt	V	0	37.07
gtc	V	0	30.16
gta	V	146	32.08
gtg	V	2	48.70
---	--	------	------
mPD		0.068	0.69
		nPD:	0.1
		N. weight:	1.1
		Sc. PD:	0.03
		Sc. rank:	30380.0

,

NA			
Pos.	79	obs:	exp:
att	I	0	0.35
atc	I	0	0.26
ata	I	1	0.39
act	T	1	0.31
acc	T	0	0.18
aca	T	0	0.43
acg	T	0	0.08
---	-	58246	58246.00
---	--	------	------
mPD		1.2×10^{-9}	1.4×10^{-9}
		nPD:	0.85
		N. weight:	0.
		Sc. PD:	0
		Sc. rank:	0

,

NA			
Pos.	80	obs:	exp:
tta	L	1	1.51
ttg	L	0	2.69
tat	Y	1	0.53
tac	Y	0	0.47
ctt	L	0	2.72
ctc	L	0	1.91
cta	L	12	2.00
ctg	L	0	2.18
att	I	82	20377.00
atc	I	8	15252.00
ata	I	57956	22417.00
atg	M	38	38.00
act	T	0	4.97
acc	T	0	2.83
aca	T	16	6.92
acg	T	0	1.28
gtt	V	0	33.56
gtc	V	0	27.30
gta	V	134	29.04
gtg	V	0	44.09
---	--	------	------
mPD		0.010	0.67
		nPD:	0.02
		N. weight:	1.1
		Sc. PD:	-0.047
		Sc. rank:	-14468.0

,

NA			
Pos.	81	obs:	exp:
caa	Q	1	0.61
cag	Q	0	0.39
aaa	K	26	17.09
aag	K	0	8.91
gtt	V	0	1.75
gtc	V	0	1.43
gta	V	7	1.52
gtg	V	0	2.30
gat	D	0	2.26
gac	D	4	1.74
gaa	E	56894	34311.00
gag	E	1304	23887.00
ggt	G	0	1.62
ggc	G	0	1.67
gga	G	12	5.54
ggg	G	0	3.17
---	--	------	------
mPD		0.045	0.49
		nPD:	0.09
		N. weight:	0.5
		Sc. PD:	0.012
		Sc. rank:	13878.0

,

NA			
Pos.	82	obs:	exp:
tct	S	0	0.16
tcc	S	0	0.15
tca	S	0	0.27
tcg	S	0	0.04
cgt	R	0	1150.00
cgc	R	0	2087.00
cga	R	8	5291.00
cgg	R	0	4980.00
aat	N	0	0.56
aac	N	1	0.44
aaa	K	248	163.70
aag	K	1	85.30
agt	S	0	0.17
agc	S	1	0.21
aga	R	57479	28446.00
agg	R	508	16041.00
ggt	G	0	0.27
ggc	G	0	0.28
gga	G	2	0.92
ggg	G	0	0.53
---	--	------	------
mPD		0.026	0.89
		nPD:	0.03
		N. weight:	0.77
		Sc. PD:	-0.024
		Sc. rank:	2559.5

,

NA			
Pos.	83	obs:	exp:
tct	S	0	0.32
tcc	S	0	0.30
tca	S	0	0.54
tcg	S	0	0.08
att	I	0	0.35
atc	I	0	0.26
ata	I	1	0.39
act	T	0	0.62
acc	T	2	0.35
aca	T	0	0.87
acg	T	0	0.16
aat	N	160	32582.00
aac	N	58077	25655.00
aaa	K	3	1.97
aag	K	0	1.03
agt	S	0	0.35
agc	S	2	0.41
gat	D	0	1.70
gac	D	3	1.30
---	--	------	------
mPD		0.0059	0.49
		nPD:	0.01
		N. weight:	0.93
		Sc. PD:	-0.043
		Sc. rank:	-18093.0

,

NA			
Pos.	84	obs:	exp:
tta	L	3	0.70
ttg	L	0	1.24
ctt	L	0	1.26
ctc	L	0	0.88
cta	L	3	0.92
ctg	L	0	1.01
cgt	R	0	0.16
cgc	R	0	0.29
cga	R	0	0.73
cgg	R	0	0.69
att	I	12	20173.00
atc	I	61	15100.00
ata	I	57392	22193.00
atg	M	348	348.00
act	T	2	41.92
acc	T	0	23.87
aca	T	133	58.42
acg	T	0	10.78
aaa	K	179	118.30
aag	K	1	61.66
aga	R	8	3.92
agg	R	0	2.21
gtt	V	0	26.55
gtc	V	0	21.60
gta	V	105	22.97
gtg	V	1	34.88
---	--	------	------
mPD		0.029	0.68
		nPD:	0.04
		N. weight:	1.1
		Sc. PD:	-0.021
		Sc. rank:	13382.0

,

NA			
Pos.	85	obs:	exp:
tgg	W	1	1.00
---	-	58247	58247.00
---	--	------	------
mPD		0	0
		nPD:	1.
		N. weight:	0.
		Sc. PD:	0
		Sc. rank:	0

,

NA			
Pos.	86	obs:	exp:
gtt	V	0	0.25
gtc	V	0	0.20
gta	V	1	0.22
gtg	V	0	0.33
---	-	58247	58247.00
---	--	------	------
mPD		0	2.2×10^{-10}
		nPD:	0.
		N. weight:	0.
		Sc. PD:	0
		Sc. rank:	0

,

NA

Pos.	87	obs:	exp:
aat	N	1	0.56
aac	N	0	0.44
---	-	58247	58247.00
---	--	------	------
mPD		0	1.5×10^{-10}
		nPD:	0.
		N. weight:	0.
		Sc. PD:	0
		Sc. rank:	0

,

NA

Pos.	88	obs:	exp:
caa	Q	0	0.61
cag	Q	1	0.39
act	T	10	18088.00
acc	T	15	10298.00
aca	T	57913	25208.00
acg	T	308	4652.00
gct	A	0	0.27
gcc	A	0	0.25
gca	A	1	0.40
gcg	A	0	0.07
---	--	------	------
mPD		0.012	0.68
		nPD:	0.02
		N. weight:	0.94
		Sc. PD:	-0.039
		Sc. rank:	-9631.3

,

NA

Pos.	89	obs:	exp:
act	T	0	0.31
acc	T	0	0.18
aca	T	1	0.43
acg	T	0	0.08
aaa	K	4	201.80
aag	K	303	105.20
gct	A	0	0.27
gcc	A	0	0.25
gca	A	0	0.40
gcg	A	1	0.07
gat	D	6	3.39
gac	D	0	2.61
gaa	E	204	34150.00
gag	E	57720	23774.00
ggt	G	0	1.21
ggc	G	0	1.26
gga	G	0	4.15
ggg	G	9	2.38
---	--	------	------
mPD		0.018	0.49
		nPD:	0.04
		N. weight:	1.
		Sc. PD:	-0.025
		Sc. rank:	10514.0

,

NA

Pos.	90	obs:	exp:
tta	L	1	0.12
ttg	L	0	0.21
tat	Y	1	0.53
tac	Y	0	0.47
ctt	L	0	0.21
ctc	L	0	0.15
cta	L	0	0.15
ctg	L	0	0.17
cgt	R	0	0.04
cgc	R	0	0.07
cga	R	0	0.18
cgg	R	0	0.17
att	I	186	20383.00
atc	I	21	15257.00
ata	I	57858	22425.00
atg	M	34	34.00
act	T	0	11.80
acc	T	0	6.72
aca	T	38	16.45
acg	T	0	3.04
aga	R	2	0.98
agg	R	0	0.55
gtt	V	0	26.80
gtc	V	0	21.80
gta	V	106	23.19
gtg	V	1	35.21
---	--	------	------
mPD		0.013	0.66
		nPD:	0.02
		N. weight:	1.1
		Sc. PD:	-0.042
		Sc. rank:	-7234.0

,

NA

Pos.	91	obs:	exp:
tta	L	0	17.06
ttg	L	143	30.39
ctt	L	0	30.75
ctc	L	0	21.56
cta	L	0	22.57
ctg	L	4	24.67
atg	M	40	40.00
gtt	V	19	14542.00
gtc	V	1	11830.00
gta	V	84	12584.00
gtg	V	57955	19103.00
gct	A	0	0.27
gcc	A	0	0.25
gca	A	0	0.40
gcg	A	1	0.07
ggt	G	0	0.13
ggc	G	0	0.14
gga	G	0	0.46
ggg	G	1	0.26
---	--	------	------
mPD		0.010	0.75
		nPD:	0.01
		N. weight:	1.3
		Sc. PD:	-0.057
		Sc. rank:	-21042.0

,

NA

Pos.	92	obs:	exp:
ttt	F	3	1.46
ttc	F	0	1.54
tta	L	1	0.12
ttg	L	0	0.21
tat	Y	57223	30673.00
tac	Y	968	27518.00
tgt	C	1	0.39
tgc	C	0	0.61
ctt	L	0	0.21
ctc	L	0	0.15
cta	L	0	0.15
ctg	L	0	0.17
cat	H	39	28.77
cac	H	12	22.23
aat	N	0	0.56
aac	N	1	0.44
---	--	------	------
mPD		0.035	0.50
		nPD:	0.07
		N. weight:	0.65
		Sc. PD:	0.0027
		Sc. rank:	14375.0

,

NA

Pos.	93	obs:	exp:
ttt	F	13	6.81
ttc	F	1	7.19
tta	L	1497	6753.00
ttg	L	47597	12032.00
tct	S	0	1.61
tcc	S	0	1.49
tca	S	1	2.69
tcg	S	9	0.39
tgg	W	2	2.00
ctt	L	5	12174.00
ctc	L	0	8534.00
cta	L	270	8937.00
ctg	L	8828	9767.00
cct	P	0	3.00
ccc	P	0	1.69
cca	P	0	3.04
ccg	P	9	1.27
att	I	0	0.35
atc	I	1	0.26
ata	I	0	0.39
atg	M	9	9.00
agt	S	0	1.75
agc	S	0	2.06
gtt	V	0	1.50
gtc	V	0	1.22
gta	V	0	1.30
gtg	V	6	1.97
---	--	------	------
mPD		0.32	1.2
		nPD:	0.28
		N. weight:	1.2
		Sc. PD:	0.23
		Sc. rank:	46813.0

,

NA

Pos.	94	obs:	exp:
tct	S	0	0.65
tcc	S	1	0.60
tca	S	0	1.08
tcg	S	0	0.16
cct	P	0	1.67
ccc	P	5	0.94
cca	P	0	1.69
ccg	P	0	0.71
att	I	0	29.84
atc	I	85	22.33
ata	I	0	32.83
act	T	239	18028.00
acc	T	57774	10263.00
aca	T	37	25123.00
acg	T	0	4636.00
aat	N	35	29.65
aac	N	18	23.35
agt	S	0	0.70
agc	S	3	0.83
gct	A	4	13.90
gcc	A	47	12.74
gca	A	0	20.63
gcg	A	0	3.73
---	--	------	------
mPD		0.018	0.69
		nPD:	0.03
		N. weight:	2.
		Sc. PD:	-0.067
		Sc. rank:	-830.4

,

NA

Pos.	95	obs:	exp:
tct	S	0	1.13
tcc	S	0	1.05
tca	S	0	1.88
tcg	S	0	0.28
act	T	0	0.62
acc	T	2	0.35
aca	T	0	0.87
acg	T	0	0.16
aat	N	411	32570.00
aac	N	57805	25646.00
aaa	K	5	3.29
aag	K	0	1.71
agt	S	0	1.22
agc	S	7	1.44
gat	D	0	10.17
gac	D	18	7.83
---	--	------	------
mPD		0.015	0.49
		nPD:	0.03
		N. weight:	0.9
		Sc. PD:	-0.027
		Sc. rank:	3960.0

,

NA

Pos.	96	obs:	exp:
tct	S	0	2.74
tcc	S	1	2.54
tca	S	1	4.57
tcg	S	0	0.67
att	I	3	50.90
atc	I	142	38.10
ata	I	0	56.00
act	T	356	18031.00
acc	T	57514	10265.00
aca	T	192	25128.00
acg	T	0	4637.00
aat	N	1	8.95
aac	N	15	7.05
agt	S	0	2.97
agc	S	15	3.51
gct	A	1	2.18
gcc	A	7	2.00
gca	A	0	3.24
gcg	A	0	0.58
---	--	------	------
mPD		0.025	0.69
		nPD:	0.04
		N. weight:	1.9
		Sc. PD:	-0.047
		Sc. rank:	19891.0

NA

Pos.	97	obs:	exp:
tct	S	0	0.32
tcc	S	1	0.30
tca	S	0	0.54
tcg	S	0	0.08
att	I	0	16.85
atc	I	48	12.61
ata	I	0	18.54
act	T	381	18070.00
acc	T	57743	10287.00
aca	T	62	25182.00
acg	T	0	4647.00
aat	N	0	4.48
aac	N	8	3.52
agt	S	0	0.35
agc	S	1	0.41
gct	A	0	1.09
gcc	A	4	1.00
gca	A	0	1.62
gcg	A	0	0.29
---	--	------	------
mPD		0.017	0.68
		nPD:	0.03
		N. weight:	2.
		Sc. PD:	-0.067
		Sc. rank:	-2145.3

NA

Pos.	98	obs:	exp:
ttt	F	1	0.49
ttc	F	0	0.51
tta	L	7	1.39
ttg	L	0	2.48
ctt	L	0	2.51
ctc	L	0	1.76
cta	L	5	1.84
ctg	L	0	2.01
cgt	R	0	0.04
cgc	R	0	0.07
cga	R	0	0.18
cgg	R	0	0.17
att	I	6	20334.00
atc	I	22	15220.00
ata	I	57896	22370.00
atg	M	7	7.00
act	T	0	30.74
acc	T	0	17.50
aca	T	99	42.84
acg	T	0	7.91
aga	R	2	0.98
agg	R	0	0.55
gtt	V	0	50.60
gtc	V	0	41.16
gta	V	201	43.78
gtg	V	1	66.46
ggt	G	0	0.13
ggc	G	0	0.14
gga	G	1	0.46
ggg	G	0	0.26
---	--	------	------
mPD		0.012	0.67
		nPD:	0.02
		N. weight:	1.1
		Sc. PD:	-0.045
		Sc. rank:	-9961.6

NA

Pos.	99	obs:	exp:
gct	A	1	0.27
gcc	A	0	0.25
gca	A	0	0.40
gcg	A	0	0.07
---	-	58247	58247.00
---	--	------	------
mPD		0	2.0×10^{-10}
		nPD:	0.
		N. weight:	0.
		Sc. PD:	0
		Sc. rank:	0

NA

Pos.	100	obs:	exp:
gct	A	1	0.27
gcc	A	0	0.25
gca	A	0	0.40
gcg	A	0	0.07
---	-	58247	58247.00
---	--	------	------
mPD		0	2.0×10^{-10}
		nPD:	0.
		N. weight:	0.
		Sc. PD:	0
		Sc. rank:	0

NA

Pos.	101	obs:	exp:
ggt	G	0	0.13
ggc	G	0	0.14
gga	G	1	0.46
ggg	G	0	0.26
---	-	58247	58247.00
---	--	------	------
mPD		0	2.0×10^{-10}
		nPD:	0.
		N. weight:	0.
		Sc. PD:	0
		Sc. rank:	0

NA

Pos.	102	obs:	exp:
caa	Q	0	0.61
cag	Q	1	0.39
---	-	58247	58247.00
---	--	------	------
mPD		0	1.4×10^{-10}
		nPD:	0.
		N. weight:	0.
		Sc. PD:	0
		Sc. rank:	0

NA

Pos.	103	obs:	exp:
tct	S	0	0.16
tcc	S	0	0.15
tca	S	1	0.27
tcg	S	0	0.04
agt	S	0	0.17
agc	S	0	0.21
---	-	58247	58247.00
---	--	------	------
mPD		0	4.8×10^{-10}
		nPD:	0.
		N. weight:	0.
		Sc. PD:	0
		Sc. rank:	0

NA

Pos.	104	obs:	exp:
gtt	V	0	0.25
gtc	V	0	0.20
gta	V	0	0.22
gtg	V	1	0.33
---	-	58247	58247.00
---	--	------	------
mPD		0	2.2×10^{-10}
		nPD:	0.
		N. weight:	0.
		Sc. PD:	0
		Sc. rank:	0

NA

Pos.	105	obs:	exp:
gtt	V	1	0.25
gtc	V	0	0.20
gta	V	0	0.22
gtg	V	0	0.33
---	-	58247	58247.00
---	--	------	------
mPD		0	2.2×10^{-10}
		nPD:	0.
		N. weight:	0.
		Sc. PD:	0
		Sc. rank:	0

NA

Pos.	106	obs:	exp:
tct	S	0	0.16
tcc	S	1	0.15
tca	S	0	0.27
tcg	S	0	0.04
agt	S	0	0.17
agc	S	0	0.21
---	-	58247	58247.00
---	--	------	------
mPD		0	4.8×10^{-10}
		nPD:	0.
		N. weight:	0.
		Sc. PD:	0
		Sc. rank:	0

NA

Pos.	107	obs:	exp:
gtt	V	0	0.25
gtc	V	0	0.20
gta	V	0	0.22
gtg	V	1	0.33
---	-	58247	58247.00
---	--	------	------
mPD		0	2.2×10^{-10}
		nPD:	0.
		N. weight:	0.
		Sc. PD:	0
		Sc. rank:	0

NA

Pos.	108	obs:	exp:
aaa	K	1	0.66
aag	K	0	0.34
---	-	58247	58247.00
---	--	------	------
mPD		0	1.3×10^{-10}
		nPD:	0.
		N. weight:	0.
		Sc. PD:	0
		Sc. rank:	0

NA

Pos.	109	obs:	exp:
tta	L	1	0.12
ttg	L	0	0.21
ctt	L	0	0.21
ctc	L	0	0.15
cta	L	0	0.15
ctg	L	0	0.17
---	-	58247	58247.00
---	--	------	------
mPD		0	3.4×10^{-10}
		nPD:	0.
		N. weight:	0.
		Sc. PD:	0
		Sc. rank:	0

NA

Pos.	110	obs:	exp:
gct	A	0	0.27
gcc	A	0	0.25
gca	A	0	0.40
gcg	A	1	0.07
---	-	58247	58247.00
---	--	------	------
mPD		0	2.0×10^{-10}
		nPD:	0.
		N. weight:	0.
		Sc. PD:	0
		Sc. rank:	0

NA

Pos.	111	obs:	exp:
ggt	G	0	0.13
ggc	G	1	0.14
gga	G	0	0.46
ggg	G	0	0.26
---	-	58247	58247.00
---	--	------	------
mPD		0	2.0×10^{-10}
		nPD:	0.
		N. weight:	0.
		Sc. PD:	0
		Sc. rank:	0

NA			
Pos.	112	obs:	exp:
atg	M	1	1.00
aat	N	1	0.56
aac	N	0	0.44
aaa	K	0	24.33
aag	K	37	12.67
gtt	V	0	12.52
gtc	V	0	10.19
gta	V	0	10.84
gtg	V	50	16.45
gct	A	0	0.82
gcc	A	0	0.75
gca	A	0	1.21
gcg	A	3	0.22
gat	D	1	0.57
gac	D	0	0.43
gaa	E	195	34285.00
gag	E	57959	23869.00
ggt	G	0	0.13
ggc	G	0	0.14
gga	G	0	0.46
ggg	G	1	0.26
---	--	------	------
mPD		0.010	0.49
		nPD:	0.02
		N. weight:	1.
		Sc. PD:	-0.039
		Sc. rank:	-6465.0

,

NA			
Pos.	113	obs:	exp:
tct	S	0	0.16
tcc	S	1	0.15
tca	S	0	0.27
tcg	S	0	0.04
cgt	R	0	108.90
cgc	R	0	197.50
cga	R	0	500.90
cgg	R	6	471.40
atg	M	1	1.00
aaa	K	449	34682.00
aag	K	52304	18071.00
agt	S	0	0.17
agc	S	0	0.21
aga	R	6	2693.00
agg	R	5478	1519.00
gaa	E	0	1.77
gag	E	3	1.23
---	--	------	------
mPD		0.19	0.67
		nPD:	0.28
		N. weight:	1.2
		Sc. PD:	0.22
		Sc. rank:	45975.0

,

NA			
Pos.	114	obs:	exp:
tct	S	1	0.16
tcc	S	0	0.15
tca	S	0	0.27
tcg	S	0	0.04
aaa	K	8	5.26
aag	K	0	2.74
agt	S	0	0.17
agc	S	0	0.21
gtt	V	0	0.25
gtc	V	0	0.20
gta	V	1	0.22
gtg	V	0	0.33
gat	D	2	1.13
gac	D	0	0.87
gaa	E	57785	34331.00
gag	E	447	23901.00
ggt	G	0	0.54
ggc	G	0	0.56
gga	G	4	1.85
ggg	G	0	1.06
---	--	------	------
mPD		0.016	0.48
		nPD:	0.03
		N. weight:	0.56
		Sc. PD:	-0.016
		Sc. rank:	3456.3

,

NA			
Pos.	115	obs:	exp:
tta	L	3	2.32
ttg	L	0	4.13
ctt	L	1	4.18
ctc	L	1	2.93
cta	L	15	3.07
ctg	L	0	3.36
cgt	R	0	0.02
cgc	R	0	0.04
cga	R	0	0.09
cgg	R	0	0.09
att	I	9	20081.00
atc	I	6	15031.00
ata	I	57189	22092.00
atg	M	229	229.00
act	T	0	148.10
acc	T	0	84.33
aca	T	476	206.40
acg	T	1	38.10
aaa	K	35	23.01
aag	K	0	11.99
aga	R	1	0.49
agg	R	0	0.28
gtt	V	0	70.38
gtc	V	0	57.26
gta	V	280	60.90
gtg	V	1	92.46
gct	A	0	0.27
gcc	A	0	0.25
gca	A	1	0.40
gcg	A	0	0.07
---	--	------	------
mPD		0.036	0.69
		nPD:	0.05
		N. weight:	1.1
		Sc. PD:	-0.012
		Sc. rank:	19458.0

,

NA			
Pos.	116	obs:	exp:
tct	S	0	0.16
tcc	S	0	0.15
tca	S	0	0.27
tcg	S	0	0.04
tat	Y	0	3.69
tac	Y	7	3.31
tgt	C	224	22760.00
tgc	C	58016	35480.00
agt	S	0	0.17
agc	S	1	0.21
---	--	------	------
mPD		0.0079	0.48
		nPD:	0.02
		N. weight:	0.55
		Sc. PD:	-0.023
		Sc. rank:	-6095.9

,

NA			
Pos.	117	obs:	exp:
tta	L	0	5.22
ttg	L	0	9.30
tct	S	0	18.23
tcc	S	113	16.89
tca	S	0	30.37
tcg	S	0	4.45
ctt	L	0	9.41
ctc	L	45	6.60
cta	L	0	6.91
ctg	L	0	7.55
cct	P	1322	19339.00
ccc	P	56660	10880.00
cca	P	46	19607.00
ccg	P	5	8208.00
cat	H	0	27.07
cac	H	48	20.93
act	T	0	2.79
acc	T	9	1.59
aca	T	0	3.89
acg	T	0	0.72
agt	S	0	19.75
agc	S	0	23.31
---	--	------	------
mPD		0.053	0.73
		nPD:	0.07
		N. weight:	1.8
		Sc. PD:	0.012
		Sc. rank:	43518.0

,

NA			
Pos.	118	obs:	exp:
caa	Q	3	1.83
cag	Q	0	1.17
cgt	R	0	1.27
cgc	R	0	2.30
cga	R	0	5.84
cgg	R	0	5.50
att	I	0	0.35
atc	I	0	0.26
ata	I	1	0.39
act	T	0	7.45
acc	T	0	4.24
aca	T	24	10.39
acg	T	0	1.92
aat	N	1	20.70
aac	N	36	16.30
aaa	K	58012	38194.00
aag	K	84	19902.00
aga	R	64	31.39
agg	R	0	17.70
gaa	E	22	12.97
gag	E	0	9.03
---	-	1	1.00
---	--	------	------
mPD		0.0081	0.46
		nPD:	0.02
		N. weight:	0.48
		Sc. PD:	-0.02
		Sc. rank:	-4538.4

,

NA			
Pos.	119	obs:	exp:
tta	L	128	508.80
ttg	L	1	906.60
tct	S	0	29.53
tcc	S	0	27.35
tca	S	183	49.19
tcg	S	0	7.20
ctt	L	0	917.30
ctc	L	0	643.00
cta	L	4241	673.30
ctg	L	15	735.90
cct	P	7	17851.00
ccc	P	10	10043.00
cca	P	53492	18098.00
ccg	P	60	7577.00
caa	Q	19	11.61
cag	Q	0	7.39
cgt	R	0	0.02
cgc	R	0	0.04
cga	R	1	0.09
cgg	R	0	0.09
att	I	0	2.46
atc	I	0	1.84
ata	I	7	2.70
atg	M	2	2.00
act	T	0	14.60
acc	T	0	8.31
aca	T	47	20.34
acg	T	0	3.75
agt	S	0	31.98
agc	S	0	37.75
aga	R	0	0.49
agg	R	0	0.28
gtt	V	0	7.76
gtc	V	0	6.32
gta	V	31	6.72
gtg	V	0	10.20
gct	A	0	0.82
gcc	A	0	0.75
gca	A	3	1.21
gcg	A	0	0.22
---	-	1	1.00
---	--	------	------
mPD		0.16	0.92
		nPD:	0.17
		N. weight:	1.3
		Sc. PD:	0.12
		Sc. rank:	43837.0

,

NA			
Pos.	120	obs:	exp:
tct	S	0	22.43
tcc	S	0	20.77
tca	S	138	37.36
tcg	S	1	5.47
att	I	0	0.35
atc	I	0	0.26
ata	I	1	0.39
atg	M	1	1.00
act	T	0	52.17
acc	T	0	29.70
aca	T	168	72.71
acg	T	0	13.42
agt	S	0	24.29
agc	S	0	28.68
gtt	V	0	63.87
gtc	V	0	51.96
gta	V	157	55.27
gtg	V	98	83.90
gct	A	53	15713.00
gcc	A	3	14404.00
gca	A	57518	23321.00
gcg	A	79	4215.00
gat	D	1	0.57
gac	D	0	0.43
gaa	E	29	17.10
gag	E	0	11.90
---	-	1	1.00
---	--	------	------
mPD		0.028	0.72
		nPD:	0.04
		N. weight :	1.
		Sc. PD:	-0.023
		Sc. rank:	11590.0

,

NA			
Pos.	121	obs:	exp:
aaa	K	150	98.62
aag	K	0	51.38
gct	A	0	0.82
gcc	A	0	0.75
gca	A	3	1.21
gcg	A	0	0.22
gat	D	3	9.61
gac	D	14	7.39
gaa	E	57943	34199.00
gag	E	64	23808.00
ggt	G	0	9.42
ggc	G	0	9.77
gga	G	70	32.31
ggg	G	0	18.49
---	-	1	1.00
---	--	------	------
mPD		0.010	0.49
		nPD:	0.02
		N. weight :	0.6
		Sc. PD:	-0.023
		Sc. rank:	-3490.1

,

NA			
Pos.	122	obs:	exp:
ttt	F	0	16.54
ttc	F	34	17.46
tat	Y	70	30683.00
tac	Y	58139	27526.00
cat	H	0	1.69
cac	H	3	1.31
gat	D	1	0.57
gac	D	0	0.43
---	-	1	1.00
---	--	------	------
mPD		0.0037	0.50
		nPD:	0.01
		N. weight :	0.86
		Sc. PD:	-0.043
		Sc. rank:	-25924.0

,

NA			
Pos.	123	obs:	exp:
cgt	R	0	1154.00
cgc	R	0	2094.00
cga	R	12	5311.00
cgg	R	0	4998.00
att	I	0	0.70
atc	I	0	0.53
ata	I	2	0.77
aaa	K	34	22.35
aag	K	0	11.65
aga	R	57903	28550.00
agg	R	293	16100.00
ggt	G	0	0.40
ggc	G	0	0.42
gga	G	3	1.38
ggg	G	0	0.79
---	-	1	1.00
---	--	------	------
mPD		0.012	0.89
		nPD:	0.01
		N. weight :	0.79
		Sc. PD:	-0.036
		Sc. rank:	-13628.0

,

NA			
Pos.	124	obs:	exp:
tct	S	0	10.33
tcc	S	0	9.56
tca	S	0	17.20
tcg	S	0	2.52
tat	Y	1	0.53
tac	Y	0	0.47
cat	H	4	2.26
cac	H	0	1.74
att	I	4	1.40
atc	I	0	1.05
ata	I	0	1.54
act	T	11	3.42
acc	T	0	1.94
aca	T	0	4.76
acg	T	0	0.88
aat	N	57938	32466.00
aac	N	92	25564.00
aaa	K	14	26.30
aag	K	26	13.70
agt	S	62	11.18
agc	S	2	13.20
gat	D	87	49.18
gac	D	0	37.82
gaa	E	0	2.95
gag	E	5	2.05
ggt	G	1	0.13
ggc	G	0	0.14
gga	G	0	0.46
ggg	G	0	0.26
---	-	1	1.00
---	--	------	------
mPD		0.011	0.50
		nPD:	0.02
		N. weight :	0.66
		Sc. PD:	-0.025
		Sc. rank:	-3688.6

,

NA			
Pos.	125	obs:	exp:
tgg	W	58246	58246.00
cgt	R	0	0.02
cgc	R	0	0.04
cga	R	0	0.09
cgg	R	0	0.09
aga	R	0	0.49
agg	R	1	0.28
---	-	1	1.00
---	--	------	------
mPD		0.000034	0.000056
		nPD:	0.61
		N. weight :	0.000026
		Sc. PD:	0.000012
		Sc. rank:	1.1

,

NA			
Pos.	126	obs:	exp:
tta	L	36	4.18
ttg	L	0	7.44
tct	S	2	9391.00
tcc	S	9	8696.00
tca	S	58141	15641.00
tcg	S	42	2291.00
ctt	L	0	7.53
ctc	L	0	5.28
cta	L	0	5.53
ctg	L	0	6.04
cct	P	0	0.33
ccc	P	0	0.19
cca	P	1	0.34
ccg	P	0	0.14
act	T	0	4.04
acc	T	0	2.30
aca	T	13	5.63
acg	T	0	1.04
agt	S	0	10170.00
agc	S	0	12005.00
gct	A	0	0.82
gcc	A	0	0.75
gca	A	3	1.21
gcg	A	0	0.22
---	-	1	1.00
---	--	------	------
mPD		0.0036	1.6
		nPD:	0.
		N. weight :	1.5
		Sc. PD:	-0.083
		Sc. rank:	-61968.0

,

NA			
Pos.	127	obs:	exp:
cgt	R	0	0.02
cgc	R	0	0.04
cga	R	0	0.09
cgg	R	0	0.09
atg	M	1	1.00
aat	N	0	0.56
aac	N	1	0.44
aaa	K	53644	38292.00
aag	K	4600	19952.00
aga	R	1	0.49
agg	R	0	0.28
---	-	1	1.00
---	--	------	------
mPD		0.15	0.45
		nPD:	0.32
		N. weight :	0.23
		Sc. PD:	0.05
		Sc. rank:	8896.9

,

NA			
Pos.	128	obs:	exp:
tta	L	0	0.70
ttg	L	0	1.24
tct	S	0	0.48
tcc	S	0	0.45
tca	S	0	0.81
tcg	S	3	0.12
ctt	L	0	1.26
ctc	L	0	0.88
cta	L	0	0.92
ctg	L	6	1.01
cct	P	423	19403.00
ccc	P	2	10916.00
cca	P	330	19672.00
ccg	P	57471	8235.00
caa	Q	0	6.11
cag	Q	10	3.89
act	T	0	0.62
acc	T	0	0.35
aca	T	0	0.87
acg	T	2	0.16
agt	S	0	0.52
agc	S	0	0.62
---	-	1	1.00
---	--	------	------
mPD		0.026	0.72
		nPD:	0.04
		N. weight:	2.2
		Sc. PD:	-0.054
		Sc. rank:	21622.0

,

NA			
Pos.	129	obs:	exp:
caa	Q	57837	35604.00
cag	Q	408	22641.00
cgt	R	0	0.04
cgc	R	0	0.07
cga	R	2	0.18
cgg	R	0	0.17
aga	R	0	0.98
agg	R	0	0.55
---	-	1	1.00
---	--	------	------
mPD		0.014	0.48
		nPD:	0.03
		N. weight:	0.53
		Sc. PD:	-0.016
		Sc. rank:	1557.1

,

NA			
Pos.	130	obs:	exp:
tgt	C	58198	22762.00
tgc	C	49	35485.00
---	-	1	1.00
---	--	------	------
mPD		0.0017	0.48
		nPD:	0.
		N. weight:	1.1
		Sc. PD:	-0.058
		Sc. rank:	-41058.0

,

NA			
Pos.	131	obs:	exp:
tct	S	0	84.56
tcc	S	0	78.31
tca	S	0	140.80
tcg	S	0	20.63
tat	Y	0	1.58
tac	Y	3	1.42
cat	H	0	0.56
cac	H	1	0.44
caa	Q	79	48.90
cag	Q	1	31.10
cgt	R	0	0.02
cgc	R	0	0.04
cga	R	0	0.09
cgg	R	0	0.09
aat	N	28	294.30
aac	N	498	231.70
aaa	K	311	205.80
aag	K	2	107.20
agt	S	0	91.57
agc	S	524	108.10
aga	R	0	0.49
agg	R	1	0.28
gtt	V	0	4.26
gtc	V	17	3.46
gta	V	0	3.68
gtg	V	0	5.59
gct	A	0	0.55
gcc	A	2	0.50
gca	A	0	0.81
gcg	A	0	0.15
gat	D	12	4686.00
gac	D	8278	3604.00
gaa	E	22	13.56
gag	E	1	9.44
ggt	G	72	6525.00
ggc	G	48380	6764.00
gga	G	15	22373.00
ggg	G	0	12805.00
---	-	1	1.00
---	--	------	------
mPD		0.33	1.0
		nPD:	0.32
		N. weight:	2.1
		Sc. PD:	0.45
		Sc. rank:	80651.0

,

NA			
Pos.	132	obs:	exp:
att	I	1	0.35
atc	I	0	0.26
ata	I	0	0.39
---	-	58247	58247.00
---	--	------	------
mPD		0	1.9×10^{-10}
		nPD:	0.
		N. weight:	0.
		Sc. PD:	0
		Sc. rank:	0

,

NA			
Pos.	133	obs:	exp:
aat	N	0	0.56
aac	N	1	0.44
---	-	58247	58247.00
---	--	------	------
mPD		0	1.5×10^{-10}
		nPD:	0.
		N. weight:	0.
		Sc. PD:	0
		Sc. rank:	0

,
[truncated: 614,399 more chars]
