# Supplementary material for: Locations and structures of influenza A virus packaging-associated signals and other functional elements via an in silico pipeline for predicting constrained features in RNA viruses
Source: PLoS Comput Biol. 2024 Apr 22;20(4):e1012009. doi: 10.1371/journal.pcbi.1012009 (PMC11034665; doi:10.1371/journal.pcbi.1012009)
Supplement: Attachment — Submitted filename: Skittrall_PLoS_Computational_Biology_cover_letter_20240305.pdf [file pcbi.1012009.s130.pdf]

Jordan P. Skittrall Ph.D. MRCP(UK) FRCPath
NIHR Clinical Lecturer in Virology

Professor Shi-Jie Chen and Professor William Noble
PLoS Computational Biology

UNIVERSITY OF CAMBRIDGE

Department of Pathology

5th March 2024

Dear Professors Chen and Noble,

Revised article submission: ***Locations and structures of influenza A virus packaging-associated signals and other functional elements via an* in silico *pipeline for predicting conserved features in RNA viruses***

Thank you very much for your initial response to our submitted article. We are grateful for the time and effort that has clearly gone into both reviews, and for the helpful guidance of the reviewers and for the additional steers you have given. We are also grateful for the additional time allowed for us to optimise manuscript revisions, as this has allowed us to make the most of the reviewer and editorial time and effort.

We have spent some time revising the manuscript in response to these comments, and include a revised version of the manuscript with this letter. Below, we detail how we have responded to each of the reviewer comments:

Reviewer #1: This manuscript reports on the results of a sequence and structural analysis of influenza A virus RNAs. The authors utilize a novel approach for estimating constraints on codon evolution and apply it to various strains of IAV to deduce regions with significant constraint. Using this they extract subregions and alignments for secondary structure prediction via RNAalifold. The authors use codon level constraints to highlight known functional sites, such as packaging signals, and post-transcriptional regulatory elements that include structured RNAs previously described, then go on to discuss potentially novel regions that arise from their analysis. My main concern with this paper is that it revisits a target that has been previously studied using similar approaches (identification of constrained codon usage, secondary structure prediction, and comparative sequence analysis) and was subsequently extensively studied using multiple in vitro and in cellulo chemical structure probing approaches and covariation analysis. To add value to this field, this work has to improve or expand on this deep body of previous work, but the manuscript, as presented, only partially does this.

The major contribution of this current work, in my opinion, is the ability to home in on codon level constraints to better identify local regions of constraint that may be functional (beyond coding for proteins). It is difficult though to determine what exactly we learn that is new, due to extensive digressions away from this main finding. In particular, the attempt to link this to secondary structure was problematic, as RNAalifold is highly sensitive to the sequences aligned and the extent of sequence fragments analyzed. The criteria the authors used - organizing sequences by antigenic subtype and clustered constrained codon sites - almost guarantees the highly diverse (in structure and levels of conservation) structure models presented in each of their seventeen main text figures: e.g. the variable and poorly conserved structures presented in Fig. 4. I don't think there is much value in presenting these models as main text figures, as the predicted differences between strains and RNAdescent hits are arising from biases in the "covariation" score in RNAalifold (arising from sequences used) and the potential alternative

Division of Virology
Department of Pathology
University of Cambridge
Addenbrooke's Hospital
Cambridge CB2 0QQ
UK

Tel: +44 (0)1223 336922
E-mail: jps55@cam.ac.uk

pairing from having more or less sequence to fold - and are not likely to represent actual functionally significant conformational differences between strains.

My recommendation is for a significant revision of the manuscript to emphasize the major novel contributions of their analysis, which are the RNAdescent results. For RNA structural analyses, I think the RNAalifold results could perhaps be added as supplemental data, if presented at all (primarily as a point of comparison to existing models). For the main text it would be valuable to focus on interpreting previous structure analyses through the lens of their novel results: e.g. as they did when revisiting the pseudoknot model presented by Priore et. al. Most IAV sequence fragments have been probed and/or modeled at this point. Please note some notable example publications below:

https://academic.oup.com/nar/article/47/13/7003/5485530
https://doi.org/10.1016/j.csbj.2023.10.036
https://www.nature.com/articles/s41591-022-01908-x
https://doi.org/10.1016/j.celrep.2020.107823
https://www.nature.com/articles/s41598-021-03767-x

If there is a compelling reason to perform additional structural analyses: e.g. if strain specific conformations are strongly suspected or a region was missed in previous work, a more careful approach for modeling should be used. RNAalifold results could serve as a good starting point, but artifacts arising from the length and the choice of sequences need to be considered when determining final models to present. Additionally, validation of RNAalifold predictions using a tool like R-Scape could be especially valuable here, where sites of significant covariation could overlap constrained codons.

Thank you for these comments. There is a lot to unpack here, and our responses to some of the comments are influenced by others.

To summarize our understanding of the comments above:

1. The major novel contribution is the output from applying the constraint analysis to influenza A data.
2. Secondary structure is an area that has previously been studied, both *in vitro* and *in vivo*.
3. Going beyond this into secondary structural considerations represents "digression" and dilutes the novel contribution.
4. The presentation of alternate predicted secondary structures for analogous regions ends up discussing likely computational artefact.
5. Computational structural predictions from a minimum free energy algorithm modified by covariation data should be validated using a different algorithm that uses covariation data.
6. The presented alternate secondary structures would be better placed in supplementary material.
7. Interpretation with regard to results of preceding experimental work [including interpretation that depends on secondary structural prediction] is reasonable in the main text.
8. Consider also structural predictions arising from previous experimental work [references given].

(We have grouped some items, which results in their being in a different order from the original, more expansive and detailed, comments.)

Before considering these point-by-point, we think it may be helpful to address what we think is the overall thrust of these points in terms of suggested outcome: that there is too much discussion of secondary structural predictions in the main text and such predictions would, in most part, be better placed in supplementary material (point 6). This is very helpful feedback, for which we are grateful: one of the challenges we faced in authoring this manuscript is that the study has produced a large amount of data addressing multiple different regions likely to be important in the lifecycles of influenza A viruses. We did not want simply to produce a database paper: maximising the utility of the data involves us using our understanding of it to help relate it to the rest of the literature (and indeed, the mention of relationship to experimental literature in *PLoS Computational Biology*'s statement of scope is one reason we submitted here). However, we are very aware that in consequence we have submitted a long and figure-heavy manuscript. We are particularly grateful for the feedback on what aids understanding and what acts as a hindrance when reading in linear fashion. We are very happy to move to supplementary material as much of the presentation in the results section of differing structural predictions and accompanying figures as we can, to increase the clarity on reading the main text.

To understand our actions in response to the remainder of these comments, we think it is helpful to respond to the comments *en masse* and then explain how we have modified the work in consequence.

We very much agree that the majority of novel utility of our work arises from applying the recently-developed constraint analysis to a large influenza A dataset (point 1). Because this focuses on RNA-level constraint separately from codon-level constraint, one major reason for constraint is the presence of secondary structural elements. This is particularly the case for influenza A, with its large set of packaging signals that are crucial for the segmented virus to remain viable. It is therefore not surprising that despite having previously been heavily studied (point 2), the way that influenza A packaging works is still not understood and is regarded of sufficient importance that multiple additional studies continue to be undertaken (point 8). Relating the results of experimental probes of structure to functional outcomes is challenging, partly because there is not necessarily only a single structure for a given region, but partly because of the many computational challenges in structural prediction algorithms. It is important to recognise firstly that the output of structural probing experiments such as those described in the additional references (point 8) is itself used as input to computational prediction algorithms within those references – that is, it is not inherently the case that the earlier work has already fully solved the problems we seek to address. It is then important to recognise secondly that there is one major advantage in using multiple sequence alignments to predict structure (as we have done), namely that the collection of primary sequences itself is informative about the secondary structures that feasibly form. However, algorithms that predict secondary structure from multiple sequence alignments (such as RNAalifold) cannot easily be run on long alignments with large numbers of sequences (because of the rapid increase in memory requirements), and even if they could, doing so would not necessarily be informative because of the lack of input information about the length scale over which important interactions occur in a particular piece of RNA. By determining regions of significant constraint within RNA, we can specify a length scale of importance for analysis and use this as input to algorithms. We would argue that although it is not as "clean" in terms of data input/output as the first step, this second step does enhance understanding and should be included somewhere (even if in a supplement): it is not simply a "digression" (point 3) but a key step in being able to interpret how our results explain previous experimental work (point 7).

We acknowledge that some presented structures are either computational artefact or represent real structures that exist either as members of an ensemble or at different lifecycle stages (point 4). Our intention in presenting these structures was not primarily to suggest that the *differences* were biologically significant, but to look for (and present) important substructures that remain *consistent* even in the presence of noise. (We acknowledge, in hindsight, that by including detailed descriptions of differences between output in the main text and figure captions, we have given the impression that we regard such differences as important, which was not our primary intention. However, we would point out that for some of our proposed applications, such as changing tendency towards frameshifting or splicing, it is of key importance that different structures can form, since the formation of different structures would be how a lifecycle element is regulated.) We have then returned to our original analysis output to corroborate the importance of these substructures through checking the degree of constraint in the nucleotides that would be critical to their formation. This form of verification differs from the use of a covariation analysis (point 5), because it investigates at codon level the degree of constraint, and inspects whether apparent non-constraint can be explained by covariation or by non-canonical base pairing (univariation). This analysis does not produce additional numerical output directly related to covariation, but instead is using the numerical output of the main codon-by-codon analysis of constraint. In investigating these data at codon level, we go beyond simply confirming using one covariation algorithm that another algorithm designed to use covariation in its predictions has indeed succeeded.

We have undertaken the following steps in response to the above:

- Where the meaning of the text can be maintained with less mention of the differences between RNAalifold secondary structure predictions, we have either moved such mention into the appendix, or removed it entirely.
- Substantially truncated Fig 1 so that it has a single exemplar prediction of secondary structure, and placed the other predictions into a figure in the appendix. (We have also changed the example RNAdescent output in Fig 1 for consistency with the remaining structure, and removed unnecessary detail of discussion of the alternate secondary structure predictions in the main text.)
- Moved (what was) Fig 2 entirely to the appendix, and moved to the appendix detailed discussion of the differing predicted secondary structures in the PB2 5′ vRNA, along with Table 2 (which accompanies this discussion).
- Added a note about the PB2 5′ vRNA structure determined in Hagey *et al*, plus references in appropriate tables.
- Further truncated the displayed secondary structure predictions in Fig 3 (renumbered to Fig 2) so that they only contain the small stem-loops discussed in the text.

- Moved (what was) Fig 4 entirely to the appendix, and moved to the appendix detailed discussion of the differing predicted secondary structures away from the consistently predicted stem-loop.
- Moved (what was) Fig 7 entirely to the appendix, and slightly reworded structural discussion in the main text.
- Moved (what was) Fig 8 entirely to the appendix, and slightly reworded structural discussion in the main text; added mention of Takizawa/Kawaguchi.
- Moved (what was) Fig 9 entirely to the appendix. We have left the structural discussion in the main text unchanged as it is brief and we think it would be misleading to the reader to remove it here.
- Moved the entire subsection discussing the PB1-F2/PB1-N40 initiation regions (which is entirely a structural discussion based around constraint observed in one subtype/host combination), plus the associated (what was) Fig 14, to the appendix.
- Altered the caption for (what was) Fig 15 (now Fig 7) to clarify how we believe the differing secondary structural predictions reflect the presence of an ensemble of structures.
- We have kept the discussion regarding a possible stem-loop structure in the NS intron, plus figure (previously Fig 16, now Fig 8), and indeed have slightly expanded it in response to a comment by Reviewer 2 (see below).
- Added a paragraph to the Discussion section, relating to properties of the structural prediction algorithm used and the potential for artefact ("Some limitations…").
- Reiterated the content of this new paragraph in the Discussion section, in the introduction in the appendix to the figures that have been moved there.
- Added references to Peterson *et al.* where there is overlap between structural predictions.
- Added references to Takizawa/Kawaguchi in tables of conserved regions for NA $3'$ conserved regions.
- Added an entry in the Discussion regarding the findings of RNAdescent in comparison with the predictions of Le Sage *et al.* (see also response to Reviewer 2).
- Where appropriate, cross-referenced findings with those of Simon *et al.* (see also response to Reviewer 2).

Minor Comments:

Rather than use the term "folds" it may be better to refer to "secondary structures", "secondary structure models/predictions", etc. "Fold" seem to imply alternate conformations.

Thank you for this comment. We have particularly considered it in light of the comments above about the utility of secondary structural predictions from multiple subtypes. Although with regard to RNA, many would use the terms "secondary structural prediction" and "fold" interchangeably, we take the point that the choice of register may nuance how the predictions are regarded. We recognise that the prediction algorithm being used predicts a single secondary structure per input alignment. However, we think it is important to get across that each prediction is one of a set of possible predictions, and that in many cases more than one secondary structure may occur *in vivo*. One of the utilities of comparing the secondary structure predictions for analogous regions in multiple subtypes is gaining additional information on which subregions are likely to have consistent secondary structures of biological importance – indeed, this is the main reason for our producing these different predictions. So on balance, if we have correctly understood that this comment suggests that the term "fold" connotes the possibility of alternate folds, then we think this is something that we should in fact aim to retain.

Throughout the manuscript "conserved codons" are discussed but I think "constrained codons" would be a better term to describe what they mean.

Thank you for this comment – probably the most succinct English phrase that conveys the intended meaning with very high technical accuracy would be "candidate naturally selected codons", which would be very clunky! We have changed references to "conserved" to "constrained" when at nucleic acid level.

Little typos like spelling errors and missing articles: e.g.
Line 5: "hospitalizatons" should be "hospitalizations"
Line 88: and then THE analysis WAS performed
Line 96: we performed THE analysis

Thank you for highlighting the typographical error and places where abstract noun usage results in confusion; we have amended the wording in each of these places.

A few rounds of additional review would be good.

Reviewer #2: Beniston et. al. utilized RNAdescent algorithms to analyze the influenza A virus (IAV) sequences from the GISAID database. Their studies identified highly conserved regions, and predicted conserved RNA secondary structures that are related with packaging and other critical functions. IAV poses a significant threat to human public health by causing seasonal outbreaks and potential pandemics, while also inflicting economic losses in poultry industries due to the virus's ability to infect avian species, resulting in widespread poultry losses. The complexity of IAV subtypes across various hosts has made IAV RNA structure-function studies challenging. The authors employed computational tools to assess sequence conservation within and among subtypes, predicting consensus secondary structures for RNAs in these conserved regions. Many identified conserved structural regions are implicated in virus packaging, translation, and splicing. This systematic analysis provides a robust tool for examining previous observed experimental data and guiding the future experiment design. The manuscript presents an extensive analysis of RNA structural motifs in each segment RNA, providing valuable structural information to explain previous experimental observations.

I'd recommend the authors to consult recent in vivo RNA structure probing papers to validate their approaches and models. The in vivo H1N1 mRNA (cRNA) and vRNA structures have been probed by SHAPE and secondary structure models have been presented (E.g. Simon et. al. 2019 PMC6648356); Mirska et. al. 2023 PMC10153785). The authors should validate their structural models with these experimental data.

We are grateful for the pointers to these two structural probing papers. It is important to bear in mind that structural probing techniques such as SHAPE and DMS probing give information on chemical accessibility of some bases under particular experimental conditions. This information is then used as additional input to structural prediction software. Whilst it is certainly reassuring when predictions made by these differing techniques agree, it is also not altogether surprising when differences arise, given the known limitations of the prediction algorithms themselves and of the underlying information that it is possible to gain from the structural probing. Both of these papers probe the RNA structures of a single H1N1 influenza A virus, in the earlier paper's case one isolated in 1934 and likely to be different from current strains. We have borne in mind the spirit of Reviewer 1's comments about minor differences arising from structural prediction algorithms, and do not wish to disappear down a rabbit hole discussing, in cases of disagreement, whether more functionally useful structural predictions arise from these individual virus structure probing experiments or from predictions that consider possible structures from many sequences representing the same, selected conserved region. (We would, however, reiterate our view that combining the output of high numbers of experiments to determine primary structure is a valid way of probing secondary structure!) That said, your (Reviewer 2's) comment does show that one subset of likely readers will want information cross-referencing where others' structural predictions and areas our algorithm deems conserved match.

We have therefore been through both papers and their supplementary material and added cross-references to them where there is overlap between areas we predict to be conserved and their predictions of structured regions. It is difficult to be too detailed comparing predictions in regions where there is not additional corroborative information on function, and we have erred away from this. Moreover, the 2019 paper by Simon *et al.* only considers cRNA, and the form of nucleotide numbering in the paper and supplement is sometimes challenging to unpick, limiting the comparisons that can be made.

In terms of packaging signals, the authors reviewed a few packaging papers and predicted how the mutations affect the RNA secondary structures and packaging based on their models. IAV RNA packaging and reassortment are complicate, and RNA long-range interactions among various vRNA segments have been observed by several groups (Dadonaite et. al. 2019, PMC7191640 Sage. et. al. 2020; PMC7372595.) It would be very helpful to know that whether these residues in the observed inter-segment interactions are conserved. Are they predicted to form local structures by RNAalifold or they are single stranded and thus can readily form inter-segment long-range interactions?

Thank you for this comment. Our conservation prediction algorithm did not find a high level of conservation in both strands for any of the major pairings discussed in these earlier works. Although the algorithm can yield false negatives, the earlier works themselves contain evidence that a high level of conservation of these pairings is not required in a viable virus, so the lack of conservation found is likely unsurprising. We have added a note about this in the Discussion section, including mention of the limitation that there is not a structural prediction algorithm capable of working with multiple sequence alignments and of predicting long-range folds. Although it would be interesting to consider whether a multiple sequence alignment fold predicted structure in the regions identified previously, the dataset we investigated did not include pre-2009 H1N1 viruses.

The secondary structures of RNA sequences in the conserved regions were analyzed using RNAalifold, which take co-variation into consideration when predicting RNA secondary structures. However, only one consensus structures are generated for each set of sequences. This raises the concern that how the software handles RNAs

that exist in equilibrium of multiple conformations or switching between conformations for function regulation. PA-X frameshift stimulator structures shown in Fig 15 varies among different subtypes. I wonder if the RNA structure prediction algorithm allows searching a few alternative structures with comparable free energies. If so, a consensus structure maybe observed among IAV subtypes.

This is an entirely reasonable question but sadly there is not an algorithm that both makes use of multiple sequence alignments and includes sub-optimal predictions in its output. We have added a mention of this as a limitation in the Discussion section.

IAV RNAs are coated with nucleoprotein (NP), and how the NP binding affects vRNA and cRNA secondary structure is not discussed in the manuscript. The current approaches in the manuscript may not be able to address this issue, but it should be included in the discussion.

Thank you for reminding us of this important point. We have extended the appropriate portion of the Discussion to include it.

the Author Summary, the authors stated their work "allows design and evaluation of vaccine candidates to ensure attenuated viruses remain able to stimulate the immune system". I feel this is overstated as there was no such description in the results section. It was not clear to me in the Discussion (lines 669-671) "we highlighted where appropriate mutation may result in attenuated vaccine strains... ".

Thank you for highlighting that the logical flow here seems incomplete. We think that, strategically, there are two possible approaches here: either expand discussion of the vaccine design implications, or reduce what is written so that it makes no claim beyond suggesting that there are likely to be implications for vaccine design. Bearing in mind Reviewer 1's suggesting that we focus on the RNAdescent results and avoid excessive interpretation except in the light of previous work in the literature, we have implemented the first approach. For the Author Summary, we have therefore simply deleted the sentence about vaccines. We have also deleted the mention in the abstract. In the Discussion, we have reworked the section about vaccine design to be clear that we do not go beyond indicating a potential use of our results.

Fig. 16 shows a stem-loop with high number of G-C pairs in the NS intron, by manually inspection of the sequence. Did RNAalifold predict such structure? What is the rationale to switch from RNAalifold in this case?

Thank you for prompting us to go back and think about what we had done here! The presented structure is similar to one presented for H7N7 viruses, although RNAalifold had predicted for those viruses two more base pairs "in" the loop, which may or may not be seen *in vivo*, and which might affect spliceosome inhibition. It was the presence of multiple predicted structures that prompted us to go and check our more detailed conservation data to work out what corroborative evidence there was for each structure. Reflecting further on this takes us to the question asked above about alternative/sub-optimal structures. If an inhibitory structure forms, it will only be useful in modifying splice product abundances if it does not form all the time, and at least one alternative structure therefore forms as well. So predictions of multiple alternative structures may be regarded as supportive of the interpretation that this region can modify spliceosome activity.

We have reworded the section so that this logic is more apparent, and have included the H7N7 predicted structure in the figure.

A stem-loop motif was observed in the HA of H7N9 that has potential to transit to high-pathogenicity avian influenza viruses. Can the authors please comment on their findings on HA of H5 subtypes? H5 HA also has been shown to be able to evolve from low pathogenicity to high pathogenicity viruses.

The region identified is near the identification threshold, so we would be cautious about over-interpreting finding the region in one subtype and not in others. We have added a sentence to emphasise this. (In any case, we cannot give a clear biological explanation as to why the region should be conserved at RNA level in the case where it is deemed conserved!)

Fig. 5 is missing.

Our apologies. The submission system requires us to upload the figures separately – we shall double check this has worked, but if you have any difficulties viewing any figures, please do contact us via the editorial office during the review cycle and we can correct any technical error. Please note that having moved some figures to the supplement, what was Fig 5 is now Fig 3.

Some of the RNA secondary structure figures are difficult to read. It will be helpful to include some labels, such as the 5'-. 3'-, and some residue numbers.

Thank you for this feedback. We suspect both the confusion, and the challenge in labelling, arises because of the use of both cRNA and vRNA, together with per-subtype reference sequences where the same nucleotide numbering may refer to different positions within a segment. We have tried to ensure that the supplementary information contains sufficient detail to understand explicitly the regions being referred to at precise sequence level. We erred on the side of including less information in the figures, hoping that this would minimise confusion, but are grateful for the feedback that in fact the opposite is the case. We have added additional labels in the figures to assist with reading, as well as taking the opportunity to correct a propagated typographical error we have spotted in the labelling related to what was Fig 8 (but has now been moved to the supplement).

We should like to reiterate our overall thanks for the work you and the reviewers have done: we feel that the manuscript is much improved by their comments, and hope you will agree.

Yours sincerely,

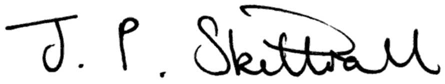

Jordan P. Skittrall (Dr)
NIHR Clinical Lecturer in Virology and
Honorary Specialty Registrar in Infectious Diseases and Medical Virology
